# Predicting Protein Phenotypes Based on Protein-Protein Interaction Network

**DOI:** 10.1371/journal.pone.0017668

**Published:** 2011-03-10

**Authors:** Lele Hu, Tao Huang, Xiao-Jun Liu, Yu-Dong Cai

**Affiliations:** 1 Institute of Systems Biology, Shanghai University, Shanghai, China; 2 Department of Chemistry, College of Sciences, Shanghai University, Shanghai, China; 3 Key Laboratory of Systems Biology, Shanghai Institutes for Biological Sciences, Chinese Academy of Sciences, Shanghai, China; 4 Shanghai Center for Bioinformation Technology, Shanghai, China; 5 College of Animal Science and Technology, Shihezi University, Shihezi City, Xinjiang, China; 6 Centre for Computational Systems Biology, Fudan University, Shanghai, China; Tokyo Medical and Dental University, Japan

## Abstract

**Background:**

Identifying associated phenotypes of proteins is a challenge of the modern genetics since the multifactorial trait often results from contributions of many proteins. Besides the high-through phenotype assays, the computational methods are alternative ways to identify the phenotypes of proteins.

**Methodology/Principal Findings:**

Here, we proposed a new method for predicting protein phenotypes in yeast based on protein-protein interaction network. Instead of only the most likely phenotype, a series of possible phenotypes for the query protein were generated and ranked acording to the tethering potential score. As a result, the first order prediction accuracy of our method achieved 65.4% evaluated by Jackknife test of 1,267 proteins in budding yeast, much higher than the success rate (15.4%) of a random guess. And the likelihood of the first 3 predicted phenotypes including all the real phenotypes of the proteins was 70.6%.

**Conclusions/Significance:**

The candidate phenotypes predicted by our method provided useful clues for the further validation. In addition, the method can be easily applied to the prediction of protein associated phenotypes in other organisms.

## Introduction

Identifying phenotypes of proteins is a central challenge of the modern genetics in post-genome era. The study on phenotypes always involves many major diseases, such as HIV [1], [2], [3], [4], [5], different kinds of cancers [6], [7], [8], [9], chronic liver diseases [10], Gaucher disease [11]. The high-throughput phenotype assays [12], [13] combining with gene perturbation technology [14], [15] provide fast identification for gene active in a response [16]. For example, yeast mutant strain collections has become increasingly used to identify the phenotypes [17]. However, these assays are often trapped in the high false negative rates [18]. On the other hand, the study on phenotypes is highly complex for the multifactorial trait often results from contributions of many proteins. Consequently, using experimental approaches alone is insufficient, and the computational methods should be applied for the identification of protein phenotypes [18].

In principle, there are two kinds of computational methods: the sequence-based methods and network-based methods. A sequence-based method is often designed on a benchmark dataset, sequence features such as amino acid composition [19], pseudo amino acid [20] (PseAAC), are used to represent the data (e.g. protein sequence), then a prediction model can be built according to the machine learning algorithm (e.g. nearest neighbor algorithm). In the past decade, a series of predictors have been designed for phenotype prediction. For example, Resch W *et al.* used a neural network model to identify the phenotype of HIV type 1 from loop 3 sequences [21]. Pillai S *et al.* proposed a classifier based on support vector machine for V3 phenotype prediction [22]. Recently, Onuki R *et al.* also employed a support vector machine method for predicting phenotype from genotype data [23]. With the ever-increasing build-up of high-throughput techniques, biological data acquisition has never increased more rapidly. More and more biological networks, such as gene-regulatory networks and metabolic networks are constructed from multi data sources (e.g. microarrays, literature mining, and protein-protein interaction). Consequently, many network-based methods are proposed to contribute to various aspects of biology, including phenotype prediction. For instance, Keleta C *et al.* implemented the prediction of the 16 different growth phenotypes in E.*coli* based on regulated metabolic networks [24]. McGary KL *et al.* demonstrate that the loss-of function *Saccharomyces cerevisiae* phenotypes are predictable in the functional gene network, and the proposed network-based method succeeded in the identification of yeast orthologs of human disease genes.

In this research, we presented a new network-based method for predicting budding yeast protein phenotypes. Unlike previous methods, our method can rank the possible phenotypes associated with the query protein and shows a more comprehensive view of the protein's biological effects. With the results, we also demonstrated that using protein-protein network is effective for predicting protein phenotypes. Owing to many protein-protein network of other organisms are available, we suggest that this method will be widely applied.

## Materials and Methods

### Data Set

Because of the complexity of phenotype research, we selected the budding yeast *Saccharomyces cerevisiae* (a well studied model organism [25], [26]) as a model system. The protein data used here was taken from CYGD [27] (the MIPS Comprehensive Yeast Genome Database, ftp://ftpmips.gsf.de/yeast/), which dedicated to information on the molecular structure and functional network of the budding yeast. Among the 6,732 proteins of the yeast proteome, only those with both sequence and phenotypic annotations were selected. Thus we obtained 1,460 such proteins belonging to 11 phenotypic categories (see Table S1). The number of proteins in each category was listed in the Table 1, from which we can easily find that the total number of proteins (2,397) in 11 phenotypic categories is much larger than the total number of proteins (1,460). That is because many proteins exhibit more than one phenotype and this is the reason why we developed this method to predict the possible phenotypes with ranked scores, rather than only one predicted phenotype like previous tools.

**Table 1:** Breakdown of 1,460 budding yeast proteins according to their 11 phenotypes.

Number	Phenotype category	Number of proteins
1	Conditional phenotypes	536
2	Cell cycle defects	271
3	Mating and sporulation defects	198
4	Auxotrophies, carbon and nitrogen utilization defects	266
5	Cell morphology and organelle mutants	534
6	Stress response defects	147
7	Carbohydrate and lipid biosynthesis defects	46
8	Nucleic acid metabolism defects	218
9	Sensitivity to amino acid analogs and other drugs	124
10	Sensitivity to antibiotics	43
11	Sensitivity to immunosuppressants	14

See the texts of the paper for further explanation.

The yeast protein-protein interaction (PPI) network used here was retrieved from STRING [28] (http://string.embl.de/), whose primary mission is to provide researchers with both physical (direct) and functional (indirect) interactions. For each species, a PPI network is constructed by integrating huge information derived from numerous sources such as experimental repositories, computational methods, and text-mining methods. In the functional protein association network, the interaction unit consists of two nodes (proteins) and an edge between them. The interaction confidence score is used as the edge weight to represent the likelihood that a predicted association exists between two nodes. Weight confidence limits are as follows: low confidence −15% (or better), medium confidence −40%, high confidence −70%, highest confidence −90%. In this research, we chose the highest confidence limit −90% to obtain reliable yeast PPI network (see Table S2), which contains 32,513 functional linkages among 4,209 yeast proteins.

Among the 1,460 proteins with phenotypic annotations, 1,267 proteins could be mapped to the yeast PPI network downloaded from STRING. Thus, the nodes in the network could be grouped into two kinds: those with phenotypic information, others without phenotypic information. Here, we called the protein with phenotypic annotation in the PPI network “seed protein”, and the dataset consisting of 1,267 seed proteins “seed set”, which were then used to test the network-based method.

### The availability of using the PPI network to predict protein phenotypes

**Figure 1:**
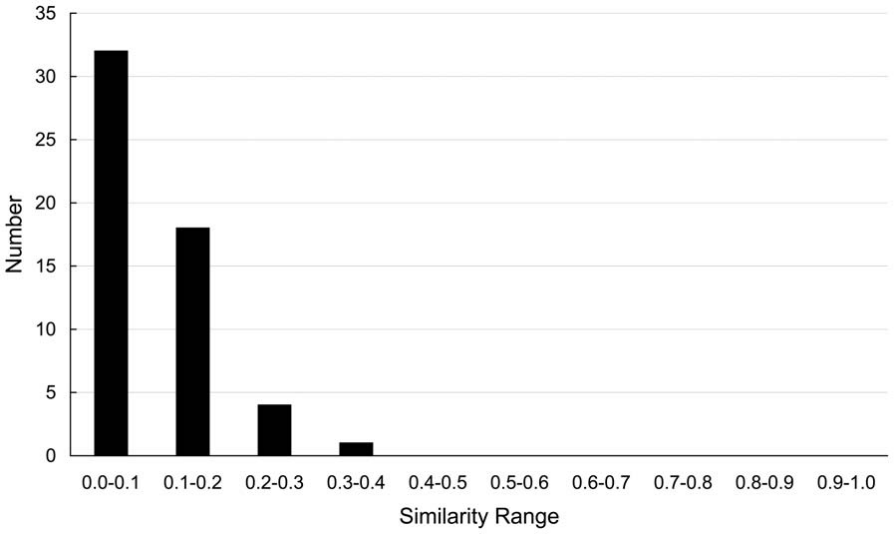
The distribution of the similarities of 11 phenotypes that were represented by protein complex information.

**Figure 2:**
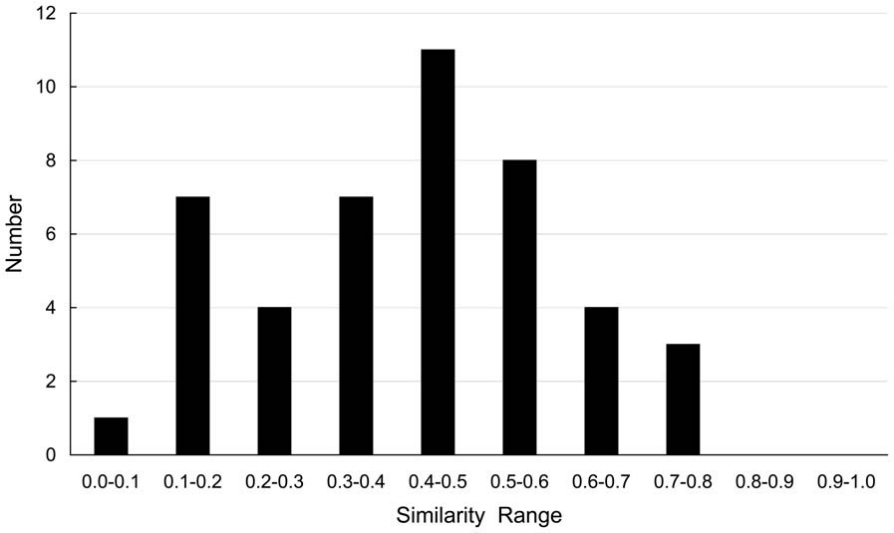
The distribution of the similarities of 10 phenotypes that were represented by protein pathway information.

In the functional network, PPI contains both physical (direct) and functional (indirect) interactions. Physically interacting proteins exist in the same complex, while functional interacting proteins tend to participate in the same pathway or cellular process. Here, we investigated the relationships between complex/pathway and phenotype to explain the availability of using the PPI network to predict protein phenotypes. In order to analyze the relationship conveniently, we selected the proteins with single phenotype. The complex annotation of proteins was also downloaded from CYGD [27], and the pathway annotation of proteins was retrieved from KEGG [29] (Kyoto Encyclopedia of Genes and Genomes) (see Table S3 and Table S4). Totally, these proteins belonged to 733 complexes and 86 pathways. Each protein was coded by the vectors:
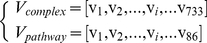
(1)where 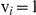 if the protein belonged to the *i-th* complex/pathway, otherwise 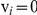. Then *m-th* phenotype can be represented by the protein complex/pathway information as the vector:
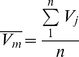
(2)where *n* is the number of proteins that had the *m-th* phenotype. The similarity between any two phenotypes was calculated as:
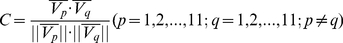
(3)where 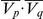 is the vectors' inner product, 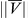 is the module of vector. Generally, two phenotypes are difficult to discriminated from each other using the complex/pathway if the value of the similarity of them is larger than 0.5. Using the protein complex information, the distribution of the similarities of 11 phenotypes was shown in **Figure 1**. Clearly, all the 55 similarities are smaller than 0.5. Because the proteins with the phenotype of sensitivity to immunosuppressants lacked the pathway annotation, the similarities of other 10 phenotypes were calculated using the protein pathway information. The distribution of the similarities of 10 phenotypes was shown in **Figure 2**, where two thirds of the similarities are smaller than 0.5. The results indicate the phenotypes can be classified by using protein complex/pathway information. Therefore, protein phenotypes can be predicted by using the functional PPI network.

### Network-based Method

In the PPI network, when we were to predict the phenotypes of a node (protein), just like the weighted vote, not only the number of its neighbor nodes, but also the strengths of interactions (i.e., the edge weights) were considered by the method. The phenotypic categories of each protein in the network can be predicted as following.

First, let us consider the PPI network consisting of 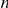 proteins 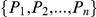, in which seed proteins belonged to 11 phenotypic categories (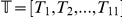), where 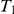 represents the “Conditional phenotypes” category, 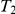 the “Cell cycle defects”, 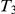 the “Mating and sporulation defects”, and so forth (cf. **Table 1**). And the phenotypes of the 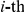 protein in the network can be denoted by 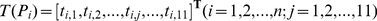
(4)where
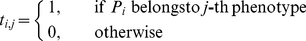
(5)


Towards a query protein 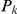, its interaction weights with *m* seed proteins can be defined as follows
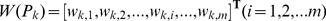
(6)where 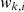 is the interaction weight (confidence score [28]) between 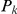 and the 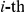 protein in the seed set. If there is no edge between them, 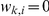. Since we did not consider the self-interaction of protein, 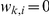 when 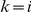. Subsequently, we proposed a new concept called “tethering potential” of protein 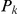 to the 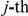 phenotype to reflect the potential of protein 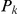 belonging to the 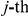 phenotype, which can be calculated as follows
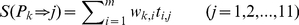
(7)


From this equation, we know that the proteins in seed set without association with the query protein do not contribute to the score of 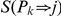. Thus the tethering potential of protein 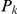 to the 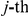 phenotype can be also described as the sum of interaction weights of it with neighbor proteins of the 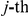 phenotype in seed set. Obviously, the larger the value of 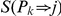 is, the more likely the protein 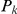 belongs to the 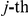 phenotypic category. Therefore, the most likely phenotype of the query protein 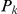 can be predicted to belong to the 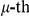 phenotypic category as follows
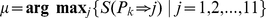
(8)where 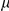 stands for the argument of 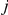 that maximizes the value of 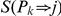. However, many proteins in yeast give rise to more than one phenotype; the prediction result with only the most likely candidate phenotype is insufficient. In view of this, to make the method able to handle the proteins with multiple phenotypes and benefit biologists with more flexible information in prioritizing candidate phenotypes, we introduced a 11-D (dimensional) vector to reflect the likelihood that the query protein may give rise to each of the 11 phenotypes, which can be formulated as follows
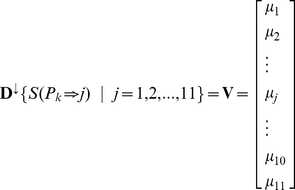
(9)where 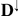 is a descending operator to sort the 11 scores of 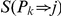 in descending order. Hence, we have 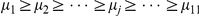. Accordingly, if 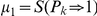, 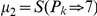, 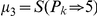, …, then that the query protein 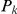 gives rise to the 1^st^ phenotype (Conditional phenotypes) will have the maximum likelihood, that 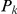 gives rise to the 7^th^ phenotype (Carbohydrate and lipid biosynthesis defects) will have the second maximum likelihood, that 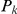 gives rise to the 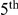 phenotype (Cell morphology and organelle mutants) will have the third maximum likelihood, and so forth (cf. **Table 1**). In rare cases, when more than one element of the vector in Eq.6 has the same value, the order will be randomly sorted. Based on the descending order of Eq.6, the predicted results are respectively called the 1^st^-order predicted result, the 2^nd^-order predicted result, the 3^rd^-order predicted result, and so forth.

### Jackknife Cross-validation and Evaluation

In statistical prediction, three cross-validation methods are often used to examine the prediction quality: subsampling (K-fold) test, independent dataset test and jackknife test [30]. Among the three methods, jackknife test is regarded as the most objective as discussed in Chou's work [31], [32] and has been used more and more frequently to test and evaluate various predictors [33], [34], [35], [36], [37], [38], [39], [40], [41], [42]. In this research, the jackknife cross-validation was also applied to test the network-based method. During the validation, each protein in the seed set is in turn knocked out as a query protein sample, and the remaining proteins of the seed set in the PPI network are used for prediction by the network-based method. Thus, the *i-th* order prediction accuracy 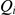 can be calculated as follows
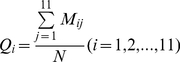
(10)


Where 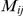 is the number of correctly predicted proteins of the *j-th* phenotypic category in the seed set, and *N* is the total number of proteins in the seed set. Finally, the 11-order prediction accuracies are obtained to evaluate the network-based method. The large 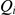 with a small *i* and the small 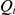 with a large *i* imply a good performance of the method.

The average number of phenotypes that each protein in the network exhibits can be calculated as follows
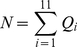
(11)


Therefore, another evaluation for the network-based method was proposed as the likelihood that the first *r-order* predicted results include all the phenotypes of proteins, which can be calculated as follows
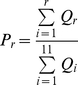
(12)


A large 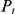 accompanied with a small *r* also implies a good performance of the method for the protein phenotype prediction.

### Prediction

Besides the seed proteins, there are also 2,942 proteins in the PPI network. The tethering potential of such protein to the each phenotype can be calculated according to Eq. (7) and then ranked in descending order. In this manner, the phenotypes of these proteins can be predicted by the network-based method.

## Results and Discussion

### Performance of Network-based method

**Figure 3:**
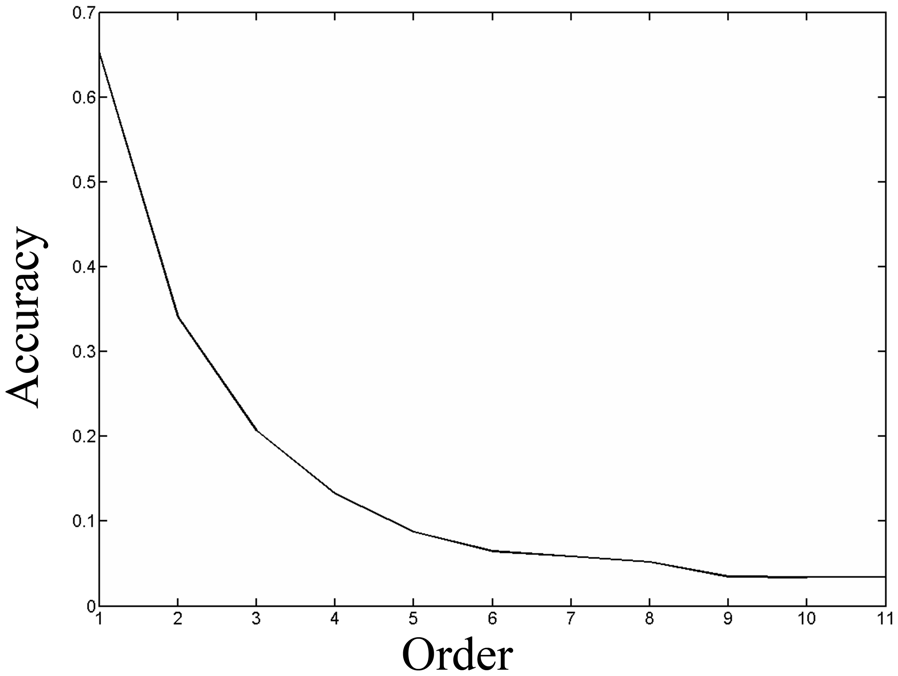
A downward-slope curve to show the relations among the different order prediction accuracies.

Through leave-one-out cross-validation, the overall 11-order success rates by the network-based method on the aforementioned 1,267 seed proteins are listed in **Table 2**. As we can see from the table, the most likely (first-order) prediction accuracy is 65.4%, and the least likely (last-order) one is 3.39%. The former minus the latter equals 61%. Based on the prediction criteria, the bigger the difference value is, the better the method performs. According to **Table 2**, a downward-slope curve is drawn in the **Figure 3**, showing that higher-order phenotype prediction is better than the lower-order one. This is the exact phenomenon that we want to see, and it may imply that the predicted phenotypic categories of proteins are well arranged by the method according to the prediction criteria.

**Table 2:** The leave-one-out cross-validation (Jackknife test) success rates by a random guess and the network-based method.

Most likely category							
	Order	1	2	3	4	5	6
Random Guess	Accuracy (%)	15.5	15.5	15.5	15.5	15.5	15.5
Network-based Method		65.4	34.1	20.7	13.3	8.76	6.47

Least likely category							
	Order	7	8	9	10	11	
Random Guess	Accuracy (%)	15.5	15.5	15.5	15.5	15.5	
Network-based Method		5.84	5.21	3.47	3.39	3.39	

The average number of phenotypes that each seed protein has is 1.7 according to Eq. (11). The chance that a random guess of a protein phenotype will succeed is 1.7/11 = 15.4%, much lower than the first order prediction success rate. As is shown in the **Table 2**, the first 3 prediction accuracies are larger than the success rates of random guess. And the likelihood of the first 3-order predicted results including the phenotypic categories of the proteins in seed set is 70.6% according to the Eq. (12). These results may imply that our method performs well in the prediction of protein phenotypes in budding yeast.

In genetics, mutations that cause the same phenotype are inferred to functionally associated, and vice versa [18]. Phenotype is a multifactorial trait that often results from the contribution of many proteins. Because the interacting proteins are often in the same complex or pathway, it is rational to expect that interacting proteins often share the common phenotypes. For example, the interactions of seed protein YBR039W with the other seed proteins are listed in **Table 3**. The complex information about those proteins is retrieved from CYGD [27]. We can easily see that protein YBR039W and its neighbors YBL099W, YDL004W, YDR298C, YLR295C, YML081C-A, YPL078C, YPL271W are members of the same F0/F1 ATP synthase (complex V) complex. Additionally, proteins YDR298C and YPL078C are also members of complex in [43], and protein YPR024W is component of Yme1 protease complex. And these proteins share the common phenotype auxotrophies, carbon and nitrogen utilization defects. Therefore, when protein YBR039W is predicted as a test sample by the method, the first candidate phenotype will be assigned its real phenotype. For another example, the interactions of seed protein YDL028C with the other seed proteins are listed in **Table 4**. The information of pathways that yeast proteins participate in is retrieved from Kyoto Encyclopedia of Genes and Genomes [44] (KEGG). Except proteins YDR168W, YKL042W, YPL209C with no pathway annotation, proteins YDL028C, YBL084C, YGL116W, YGR113W, YGR188C, YIL106W, YKL022C, YMR055C, YOR026W involve in the same pathway sce04111 (Cell cycle in budding yeast). The loss-of-function of any one of these 9 proteins likely disrupts the mitotic cell cycle progression and lead to cell cycle defects. Based on the interactions listed in the table, we can arrange the first, second candidate phenotype of protein YDL028C as the cell cycle defects, cell morphology and organelle mutants respectively according to the prediction criteria. The correct phenotype predictions of proteins YBR039W and YDL028C support the hypothesis that the functional associated proteins often share the same phenotypes. Therefore, the protein phenotypes can be predicted from the phenotypes of its interacting proteins by the method.

**Table 3:** Interactions of protein YBR039W with its neighbor proteins.

Protein A	Phenotype	Complex	Protein B	Phenotype	Complex	Weight
YBR039W	P1	C1	YBL099W	P1	C1	999
YBR039W	P1	C1	YDL004W	P1	C1	999
YBR039W	P1	C1	YDR298C	P1	C1; C2	999
YBR039W	P1	C1	YLR295C	P1	C1	917
YBR039W	P1	C1	YML081C-A	P1; P2	C1	934
YBR039W	P1	C1	YPL078C	P1	C1; C2	999
YBR039W	P1	C1	YPL271W	P1	C1	997
YBR039W	P1	C1	YPR024W	P1; P2; P3	C3	986

C1 represents F0/F1 ATP synthase (complex V), C2 represents Complex in study [38], C3 represents Yme1 protease complex, P1 represents Auxotrophies, carbon and nitrogen utilization defects, P2 represents Cell morphology and organelle mutants, P3 represents Conditional phenotypes.

**Table 4:** Interactions of protein YDL028C with its neighbor proteins.

Protein A	Phenotype	Pathway	Protein B	Phenotype	Pathway	Weight
YDL028C	P4; P5	sce04111	YBL084C	P4; P6	sce04111; sce04113; sce04120	929
YDL028C	P4; P5	sce04111	YDR168W	P4	no annotation	999
YDL028C	P4; P5	sce04111	YGL116W	P4	sce04111; sce04113; sce04120	956
YDL028C	P4; P5	sce04111	YGR113W	P4; P5	sce04111	999
YDL028C	P4; P5	sce04111	YGR188C	P5	sce04111; sce04113	999
YDL028C	P4; P5	sce04111	YIL106W	P4; P5	sce04111	988
YDL028C	P4; P5	sce04111	YKL022C	P4; P7	sce04111; sce04113; sce04120	929
YDL028C	P4; P5	sce04111	YKL042W	P4	no annotation	990
YDL028C	P4; P5	sce04111	YMR055C	P4	sce04111	984
YDL028C	P4; P5	sce04111	YOR026W	P4; P7	sce04111	978
YDL028C	P4; P5	sce04111	YPL209C	P4; P5; P7	no annotation	984

P4 represents Cell cycle defects, P5 represents Cell morphology and organelle mutants, P6 represents Nucleic acid metabolism defects, P7 represents Conditional phenotypes, Sce04111 represents cell cycle pathway in budding yeast.

### Protein phenotype prediction with inactivating its interacting protein

Here, we discuss the robustness of our method by applying the method to the proteins whose interacting proteins are inactivated. First, we chose a protein and took away one of its interacting proteins from the PPI network. Then the phenotype of the protein was predicted by the method based on the broken PPI network. In this way, the phenotypes of 6 proteins were predicted, as shown in **Table 5**. The phenotypes predicted from the unbroken network and the recent phenotype studies focusing on these proteins are also listed in **Table 5**. We found that the phenotypes predicted from the broken network were different from the phenotypes predicted from the unbroken network, while the proteins were verified to have these new phenotypes predicted from broken network in the recent studies. For example, with protein YOR196C in the network, the 1^st^ order predicted phenotype of protein YER178W by the method is “auxotrophies, carbon and nitrogen utilization defects”, which is the same as the annotation from CYGD [27]. After inactivating protein YOR196C, the phenotype of protein YER178W is predicted as the “conditional phenotypes”. In the study [45], protein YER178W was reported to have the phenotype-“Heat sensitivity: increased”, which is one kind of “conditional phenotypes” according to the phenotype classification in CYGD. In the table, the new phenotypes of other proteins predicted from the broken network can also be supported by the literatures [13], [46], [47], [48], [49], [50]. The examples listed in the table indicate that our method may provide new phenotypes for proteins and serve as a complementary tool for the existing resources.

**Table 5:** Phenotypes of proteins predicted by our method with/without inactivating its interacting protein.

Protein	Phenotype from CYGD [27]	Phenotype predicted by our method without inactivating the interacting protein	Inactivated interacting protein	Phenotype predicted by our method with inactivating the interacting protein	Phenotype from literatures
YER178W	Auxotrophies, carbon and nitrogen utilization defects	Auxotrophies, carbon and nitrogen utilization defects	YOR196C	Conditional phenotypes	Heat sensitivity: increased [45]
YML035C	Conditional phenotypes	Conditional phenotypes	YDR226W	Cell morphology and organelle mutants	Toxin resistance: increased [46]
YMR198W	Cell cycle defects	Cell cycle defects	YPR141C	Cell morphology and organelle mutants	Bud morphology: abnormal [47]
YOR254C	Conditional phenotypes Cell cycle defects Mating and sporulation defects	Conditional phenotypes	YKL073W	Cell morphology and organelle mutants	Mitochondrial morphology: abnormal [48] Telomere length: increased [49]
YDL198C	Conditional phenotypes	Conditional phenotypes	YPL240C	Auxotrophies, carbon and nitrogen utilization defects	Utilization of nitrogen source: absent [50] Utilization of carbon source: decreased [13]
YPR166C	Auxotrophies, carbon and nitrogen utilization defects Cell morphology and organelle mutants	Auxotrophies, carbon and nitrogen utilization defects	YHR147C	Conditional phenotypes	Heat sensitivity: increased [45]

### Application and improvement

As is discussed above, the first 3-order predicted results (approximately double the average number of phenotypes 1.7) can be considered as the candidate phenotypes of the proteins concerned by the biologists. Genetic experiments can focus on these candidate phenotypes of the proteins, which may accelerate the research progress and decrease the cost. At least, the last three predicted phenotypes can be excluded because the last 3-order prediction accuracies are lower than 5% (See **Table 2**).

The effectiveness of the functional network for predicting phenotypes of proteins in yeast suggests the possibility of application to other species. The method is based on the functional protein association network. Besides an abundance of such networks in STRING [28] (Version 8.0 of STRING covered 630 networks of different organisms), the PPI networks can also derived from worm PPI database [51], fly database [52], human PPI database [53], [54], [55], and so on. When in possession a series of proteins with known phenotypes, one can predict the possible phenotypes of other proteins in the networks. Therefore, the method can be easily applied to the prediction of protein phenotypes in other organisms, especially model organisms.

The performance of our method can be improved if the following problems are solved. First, increase the quality of PPI network and exclude the false positive interaction; currently we used high confidence score cutoff to filter the network (See section Data Set). Second, proteins in the same complex or pathway may exert opposite effects on a phenotype, playing as actors or repressors [18]. If the network can discriminate the positive or negative regulation, our method can be modified and the performance will be improved. Third, the performance of the network-based method depends on the number of seed proteins. This problem can be solved in future when the phenotypes of more proteins are investigated. In summary, identification of protein phenotypes is an extremely complicated work and there is a long way to go.

### Conclusion

In this research, we proposed a multi-target model [40] to predict phenotypes of proteins in budding yeast based on the protein-protein network. Because some proteins can give rise to more than one phenotype, rather than the most likely phenotype, a series of candidate phenotypes are predicted for each protein. With the performance of the method, it is anticipated that the promising approach may serve as a useful tool for annotating the phenotypes for uncharacterized protein sequences.

## Supporting Information

Table S1 **The 1,460 proteins with both sequence and phenotype information retrieved from CYGD (the Comprehensive Yeast Genome Database) (Guldener U, Munsterkotter M, Kastenmuller G, Strack N, van Helden J, et al. (2005) CYGD: the Comprehensive Yeast Genome Database. Nucleic acids research 33: D364-368.).** The corresponding phenotype of the phenotype number can be found in Table 1.
(PDF)

Table S2 **Yeast Protein-Protein Interaction Network.** The protein-protein interaction were downloaded from STRING (Search Tool for the Retrieval of Interacting Genes/Proteins) (Jensen LJ, Kuhn M, Stark M, Chaffron S, Creevey C, et al. (2009) STRING 8–a global view on proteins and their functional interactions in 630 organisms. Nucleic acids research 37: D412-416.) with the highest confidence, i.e., the confidence scores are not less than 900.
(PDF)

Table S3 **The proteins and the complexes they belong to in yeast.** The information was retrieved from CYGD (the Comprehensive Yeast Genome Database) (Guldener U, Munsterkotter M, Kastenmuller G, Strack N, van Helden J, et al. (2005) CYGD: the Comprehensive Yeast Genome Database. Nucleic acids research 33: D364-368.).
(PDF)

Table S4 **The proteins and the pathways they belong to in yeast.** The information was retrieved from KEGG (Kyoto Encyclopedia of Genes and Genomes) (Kanehisa M, Goto S, Hattori M, Aoki-Kinoshita KF, Itoh M, et al. (2006) From genomics to chemical genomics: new developments in KEGG. Nucleic Acids Res 34: D354-357.).
(PDF)
