# Supplementary material for: Predicting Protein Phenotypes Based on Protein-Protein Interaction Network
Source: PLoS One. 2011 Mar 10;6(3):e17668. doi: 10.1371/journal.pone.0017668 (PMC3053377; doi:10.1371/journal.pone.0017668)
Supplement: Table S1 — The 1,460 proteins with both sequence and phenotype information retrieved from CYGD (the Comprehensive Yeast Genome Database) (Guldener U, Munsterkotter M, Kastenmuller G, Strack N, van Helden J, et al. (2005) CYGD: the Comprehensive Yeast Genome Database. Nucleic acids research 33: D364-368.). The corresponding phenotype of the phenotype number can be found in Table 1. (PDF) [file pone.0017668.s001.pdf]

Table S1. The 1,460 proteins with both sequence and phenotype information retrieved from CYGD (the Comprehensive Yeast Genome Database) (Guldener U, Munsterkotter M, Kastenmuller G, Strack N, van Helden J, et al. (2005) CYGD: the Comprehensive Yeast Genome Database. Nucleic acids research 33: D364-368.). The corresponding phenotype of the phenotype number can be found in Table 1.

Protein	Phenotype Number(s)	Protein	Phenotype Number(s)
YBR143C	10; 4; 5	YHR102W	5
YDR353W	1; 6	YAL029C	3
YCR028C	1; 7; 9	YGL225W	3
YHR184W	3	YNR033W	4
YMR015C	7	YGL055W	1; 5; 7
YLR295C	4	YHR005C	3
YLR005W	8	YJR068W	1; 2; 5; 8
YPL022W	8	YAL062W	5; 6
YOR310C	1	YLR418C	3; 8
YLR355C	4	YNL225C	1; 2; 3; 5
YPR178W	1	YJL146W	2
YPR103W	1; 2; 3; 5	YJR032W	1
YPL172C	1; 5	YPL069C	1; 7
YPR165W	1; 2; 3	YNL221C	1
YJL073W	3	YDR207C	1; 3; 8
YHR124W	2	YOR132W	5
YDR439W	8	YER177W	11
YER120W	4	YMR272C	7
YFL038C	1; 2; 3; 5; 6	YGL035C	4; 8
YMR168C	2	YDR166C	5
YGL167C	4; 5; 6	YPL018W	2; 5
YIL022W	5	YPL057C	5; 6; 7
YOR221C	4	YDR323C	1; 5; 6
YPR072W	1	YLR397C	9
YGR056W	1	YBR256C	4
YOR125C	4	YGR023W	3
YNL007C	1	YNR038W	1
YGL175C	2	YBR110W	10
YBL025W	1	YNL001W	1; 2; 3; 5
YNL025C	2; 4; 5	YDR172W	4; 5
YFR036W	1	YDL017W	2; 8
YPL256C	2	YOR035C	3; 5
YBR069C	11	YDL215C	4
YAL019W	8	YDR195W	1; 5
YNL330C	3; 8; 9	YIL143C	8
YOL018C	5	YBR015C	6; 7
YGR063C	1; 8	YDR168W	2

YIL031W	1; 5	YJR042W	1; 3; 5
YKL096W-A	5; 9	YJR076C	1; 2; 5
YBR109C	2; 5	YAL009W	1; 3; 5
YCR017C	6; 9	YMR243C	6
YKL080W	1; 5; 6	YDR085C	3
YFR028C	2	YIL078W	1
YDR369C	8	YML095C	8
YMR146C	1	YDR159W	1; 5
YJR090C	1; 2; 5; 6	YCL008C	5
YLR447C	5; 6	YDR034C	4
YOR360C	6	YIL068C	5
YJL092W	3; 8	YMR231W	3; 5; 6
YGR144W	8	YLR313C	3
YER015W	4; 5; 7	YEL046C	4
YHR175W	6	YIL119C	1
YDR342C	4	YJR106W	5
YLR372W	1; 5; 7	YLR274W	2; 8
YBR200W	1; 2; 3; 5	YLR357W	1
YOL086C	1	YLR310C	1; 2; 3; 5
YDR173C	1; 3	YNL216W	8
YBR212W	1	YCL035C	6
YMR016C	5	YMR214W	5
YMR241W	4	YGR061C	4
YOL020W	9	YMR294W	1; 2; 5
YIL015W	2	YBL091C	1
YGR072W	10	YEL050C	4; 5
YDR495C	1; 5	YMR190C	8
YLL021W	3; 5	YJL141C	1
YBR036C	6; 7	YPR018W	8
YOL001W	1; 4; 5	YMR116C	1; 5
YDR297W	5; 7	YLL040C	3
YHR172W	2; 5	YLR332W	3
YBL088C	8	YER065C	4
YIL150C	1; 5; 8	YLR337C	1; 5; 6
YLR330W	5	YGR172C	5
YNL079C	1; 3; 5	YLR359W	4
YML064C	2	YPL161C	1; 2; 5
YER022W	1; 8	YDL084W	1
YBR039W	4	YML062C	1
YHR002W	1	YGR078C	1; 3; 5
YER107C	1; 5	YBR272C	8
YPR026W	4	YNL082W	8
YPL092W	9	YJL026W	2
YKL148C	4	YBR201W	5

YDR410C	3	YPL132W	5
YCR077C	1; 2; 8; 9	YDL095W	5
YDL008W	2	YPL053C	10; 5
YPR124W	6	YBL105C	2; 5; 8; 9
YHR004C	1; 3; 5	YGL120C	1
YDL028C	2; 5	YMR173W	5; 6; 9
YBL040C	5	YOR089C	1; 5
YBL015W	3	YDL128W	6
YBR083W	5; 8	YPL002C	3; 4
YHR069C	8	YDR347W	4; 5
YBR045C	3	YOR040W	9
YJR093C	1	YML004C	9
YGR112W	4	YBR279W	1; 5; 8
YBR009C	1; 2; 4; 5; 8	YBR010W	4; 5; 8
YNL192W	3; 5	YHR171W	5; 6
YDR284C	5	YLR299W	5
YGR158C	8	YIL049W	5
YDR160W	9	YJR104C	6
YMR021C	1; 4; 6	YNL112W	1
YCR084C	5	YGR229C	2; 5
YDR507C	1; 2; 5	YOR257W	2; 5
YDR468C	5	YDL164C	2
YJL097W	2	YJR025C	4
YER154W	4	YFR052W	1; 2; 6; 8; 9
YER044C-A	2; 8	YHR137W	1
YKL213C	1; 9	YDR072C	6
YOL022C	1	YBR087W	1; 8
YDL003W	1; 2; 5; 8	YOR208W	1
YLR071C	2; 4	YGL135W	1
YDR345C	4	YCR003W	4
YPL210C	1	YKL196C	5
YNL032W	5; 9	YER026C	4; 7
YHR060W	11; 1; 4; 6	YOR249C	1; 2
YER031C	5	YGR105W	1; 5; 6
YPL148C	4	YNL126W	2; 5
YML060W	8	YKL042W	2
YOR136W	4	YMR198W	2
YIL154C	10; 1; 3; 5; 9	YPR163C	1
YPL177C	6	YDR009W	4
YHR164C	1; 2; 8	YDR080W	5
YGL173C	1; 2; 3; 5; 6; 8	YJL166W	5
YER014W	4; 5	YBR169C	1
YDR021W	10; 1	YDL059C	8
YDR224C	2; 3	YMR275C	1

YDR529C	4	YBR114W	8
YDR225W	1; 2; 3; 4; 9	YPL049C	5
YNL252C	1; 5	YNR045W	4
YBL020W	2	YHL027W	1; 5
YDR337W	4; 5	YOR157C	1
YHR165C	2	YDR182W	1; 2; 5; 6; 8
YDL126C	2; 5	YFL033C	2; 3
YPL040C	4	YHR086W	3
YFL059W	3	YKL124W	11
YBR171W	1; 3; 5	YCR088W	1; 3
YMR055C	2	YDR118W	1
YGL200C	5	YFR004W	1
YLR353W	5; 9	YKL032C	4; 9
YJL057C	6	YLR191W	5
YGL095C	5	YPL255W	2; 5
YLR390W-A	5	YCR086W	2; 3
YJL173C	8	YDR419W	8
YPL008W	2	YMR256C	4
YFR019W	1; 2; 5	YJL134W	7
YMR035W	6; 8	YJR137C	10; 5
YDL078C	7	YDL160C	1; 9
YGL206C	1	YJL115W	1; 8
YDR405W	4	YPL204W	8
YGR047C	8	YBL035C	1
YBR103W	6; 8	YGR197C	8
YPR104C	1	YGR222W	4
YOR099W	5	YBL005W	9
YOR184W	4	YKL025C	5
YMR022W	6	YCR057C	5
YJL005W	1; 2; 3; 4	YOL051W	3
YOL108C	4	YJL063C	4
YOR043W	2; 6	YLR342W	11; 1; 5; 9
YNL027W	3; 6	YPL118W	4
YHR005C-A	5	YMR223W	1
YOR178C	4	YMR039C	4
YDR285W	2; 3	YOR092W	5
YOR355W	4	YOR018W	6
YPL036W	1	YJL053W	5
YOL052C	1; 5; 6	YDR427W	1; 6
YJR122W	4	YHR205W	1; 2
YPL155C	5	YPR185W	6
YOL030W	1	YKR076W	5
YGL145W	5	YNL062C	1
YML094W	1; 5	YMR060C	4

YKL022C	1; 2
YGL100W	1; 3
YOR336W	1; 5
YHR147C	4
YML075C	7
YLR195C	1; 3
YIL146C	5; 9
YGR113W	2; 5
YFR002W	1
YDR432W	1; 5; 8
YPL032C	1; 5
YKL114C	6; 8
YIR011C	1; 2
YHR057C	1
YGL253W	3
YNL201C	4
YJR055W	1
YLR212C	2; 5
YLR229C	1; 3; 5
YBL101C	5
YOL076W	1; 5; 9
YNL277W	9
YLR396C	1; 4; 5
YPR062W	9
YIL128W	1; 3; 4; 8
YJL128C	5; 9
YHR041C	1; 5; 8
YGR120C	1; 5
YDR283C	1
YAL010C	1; 5
YJR060W	4; 5
YOR061W	1; 5; 8
YNL229C	5
YAR007C	3
YLR371W	1; 2; 3; 5; 9
YGR092W	1; 2; 6; 9
YAL040C	2
YKL048C	5
YJL013C	5
YNL189W	1; 2
YJR034W	4; 5
YGL103W	9
YER020W	5
YDR069C	2
YHR118C	2; 8
YIL106W	2; 5
YGL018C	4
YDR142C	5
YLR096W	2
YMR052W	2
YNL029C	5
YER172C	1; 3
YKL057C	1; 5
YMR213W	2
YMR197C	5
YER125W	1; 5; 9
YNL329C	5
YDR488C	1
YDR498C	10; 1; 5
YOR106W	1; 5
YBL080C	4
YMR062C	5
YKL130C	3
YHR014W	2; 3
YDR150W	1; 2; 3
YHR077C	9
YPL021W	5
YGL087C	8
YPL243W	1
YDL040C	1; 3
YOR201C	4
YHR062C	8
YLR292C	2; 3; 5
YEL059C-A	4
YOL006C	2; 8
YOR266W	9
YBR112C	1; 8
YLR319C	1; 5
YPR016C	2
YAL041W	1; 3; 5; 6
YMR302C	1
YIR009W	1
YLR382C	4
YCR093W	2
YHR157W	2; 3; 8
YGR044C	3
YLR026C	5
YPL086C	1; 8

YDR293C	6; 9	YOR153W	9
YBR082C	1; 6; 9	YDL047W	1; 2
YBR196C	2; 4	YNL259C	6
YIL075C	1; 6	YBR166C	6
YBR084C-A	1	YDR508C	9
YLR048W	1	YOR001W	1
YOL004W	2; 3; 8; 9	YDR363W-A	1; 2; 5
YKR031C	3; 4; 5	YJL127C	1; 8
YPL213W	1	YOR133W	9
YBR070C	5	YJR052W	8
YBR176W	5	YLR303W	9
YIL085C	5	YCL014W	5
YJL208C	4; 8	YPR160W	4
YKR082W	1; 3; 5	YHR139C	3
YIR008C	2; 3; 8	YGL090W	3; 8
YCR002C	1; 5	YKL009W	1
YGR055W	9	YLL001W	3; 5
YLR262C	1; 5	YPR168W	1
YKR054C	2; 5	YHR021W-A	5
YNL238W	3; 9	YCR066W	8
YJR075W	10; 5	YGR281W	10; 9
YDR002W	1; 3	YJR059W	6; 9
YPL029W	4	YKL004W	5
YGR104C	1	YNL148C	5
YLR305C	1; 2; 5; 9	YBR294W	4; 6
YGR174C	4	YBL027W	1
YOR204W	1	YNL222W	8
YOR269W	1; 2	YLR150W	4
YKR009C	10; 5	YNL261W	1; 8
YHL043W	5	YBR073W	1; 3; 8
YER155C	1; 2; 5; 9	YIL046W	6
YGR159C	1	YGL227W	6
YDR390C	1; 5	YJR092W	5
YNL005C	4	YLR078C	1; 5
YML048W	10; 4; 5	YOR317W	7
YBR097W	5	YOL091W	3
YLR399C	1; 2; 4; 5; 6; 8	YGL044C	1
YLL050C	1; 5	YBR132C	4
YJL089W	1	YDR212W	1; 5
YBR091C	5	YDL225W	5
YPL042C	2; 4; 5	YDL108W	1; 8
YGR062C	4	YDR164C	5; 7
YDR052C	1; 2	YER008C	1; 4; 5
YDL192W	1; 3; 5; 6	YPR082C	2

YJL125C	1
YPR164W	9
YPL268W	1; 2; 4; 5
YMR167W	2; 3
YGL073W	1; 2; 5
YIL162W	4
YDR457W	1; 2; 8
YNR007C	3; 5
YHR023W	1; 2; 5
YPL076W	1
YMR100W	5
YBR253W	1; 8
YIL084C	3
YDR208W	5
YDR200C	2
YCR089W	3
YGR214W	1; 5
YJL054W	1; 3
YGL070C	1
YNL197C	5
YGL119W	4
YFL016C	1; 4
YDL022W	5
YER056C	9
YBR179C	1; 4; 5
YJL004C	1
YNL162W	9
YJR043C	1; 8
YMR164C	4
YCL004W	1; 5; 9
YGR116W	1; 2; 8
YLR075W	1; 5
YNL291C	3
YER021W	1
YBR159W	1
YDR294C	7
YKL203C	11; 5
YGR171C	4
YER095W	2; 3; 8
YOR098C	1; 2; 5
YDR448W	1
YML112W	1
YNL280C	7
YCL025C	9
YOL143C	4
YPL271W	4
YKL122C	1
YPL203W	5
YLR321C	2
YFR027W	1
YPR133C	1
YAL024C	1; 2; 3
YHL011C	1
YPL121C	2; 8
YER149C	3; 5
YOR316C	6
YPR191W	1; 4
YER176W	10; 5
YGL092W	1; 5
YHL004W	1
YPR141C	2; 5; 8
YLR067C	4
YJL159W	6
YAL025C	1; 2
YOR038C	8
YDR081C	1; 4
YEL036C	1; 5; 7; 9
YLL002W	9
YKL092C	5
YNL031C	8
YJL062W	5
YDR027C	5
YGL076C	1
YML104C	1; 2; 5
YER127W	1
YER129W	9
YER179W	2; 3; 8
YOR030W	5
YGR166W	5
YKL154W	1; 4
YPL043W	1
YKL184W	3; 5
YBL097W	1
YLL009C	4
YOL069W	2
YHR187W	1; 5
YIR019C	5
YOL148C	1; 3; 4; 8

YJL099W	1; 5
YGL252C	4
YDL188C	1; 2; 5
YEL027W	1; 4; 5; 6
YDR436W	5
YHR059W	1
YER159C	1
YIL009C-A	8
YKL145W	2; 8
YIL061C	1
YMR096W	3
YGR180C	1; 2; 4; 5; 8
YDR227W	6; 8
YPL091W	6
YPL078C	4
YLR452C	3
YDL014W	1
YLR287C-A	1
YNL064C	5
YLR139C	4
YNL154C	1; 5
YNL209W	1
YFL037W	1; 3; 5
YPR088C	1; 4
YGL071W	3; 5; 6
YOR008C	10; 5; 9
YGR162W	1
YER162C	8
YOR058C	5
YGL155W	1; 2; 5
YJL014W	1; 2; 5
YBR185C	1; 4
YPL050C	5; 6
YER032W	1
YBR160W	1; 2; 8
YBR265W	7
YML043C	8
YOL115W	1; 5; 8
YAL021C	1; 2; 4; 6; 9
YKL045W	3
YDR483W	1; 5
YPR132W	10
YER109C	5
Q0250	4
YFL001W	1
YBL084C	2; 8
YDR311W	8
YPL240C	1
YNL298W	2; 5
YJL041W	1
YFL003C	2; 3
YMR201C	8
YNR052C	1; 2; 3; 4; 6; 9
YGL249W	2; 8
YCR047C	1; 6
YAL054C	5
YGL058W	10; 1; 8
YNL093W	5
YGL123W	10
YDR054C	1; 2; 5
YDR077W	6
YGR009C	1; 5
YOR334W	4
YOR299W	5
YPL089C	9
YOL145C	1; 2; 3; 5
YER178W	4
YIR030C	5; 9
YJL042W	5
YDL142C	7
YPL140C	1; 4; 6
YJL210W	1
YLR304C	4
YNL138W	1; 5; 6
YOR212W	3
YNR030W	5
YOR005C	2; 3
YHL022C	2; 8
YMR138W	1; 2; 5
YKL134C	4
YLR429W	5
YGL240W	1; 2
YPL242C	1; 2; 5
YLR148W	1; 3; 5; 6
YDL202W	4
YNL287W	1; 5; 9
YKL167C	1; 4
YDR376W	4; 5

YER023W	1; 2
YCR032W	5
YNL084C	1; 3; 5
YJL087C	4
YGR080W	5
YDL092W	1
YNL142W	5
YKL096W	5
YLR377C	4
YDR356W	2; 5
YMR139W	3
YBR023C	5
YPL178W	2
YOR026W	1; 2
YDR176W	1
YAL023C	5
YBR217W	5; 6
YPL239W	1
YOR198C	2; 5
YGR008C	5
YER148W	8
YDR329C	5
YBR146W	4
YPL061W	1
YOR031W	6
YMR235C	1; 5
YBR130C	3
YMR263W	3; 8; 9
YGL105W	1
YBR048W	1
YCR071C	4
YEL024W	4
YPR069C	5
YJL140W	1
YJL081C	8
YOL025W	4; 5
YLR102C	1; 2
YEL032W	2; 8
YNL312W	1; 8
YDL138W	5
YGL061C	2; 5
YNL223W	3
YIL033C	1; 3; 4; 6; 8
YBR173C	3; 6
YGR183C	4
YIL010W	8
YPL175W	1; 5; 7; 8
YAL051W	7
YCL029C	1; 2; 5
YJR094C	2
YOL023W	5
YJR002W	1
YPL174C	2
YPR181C	5
YHR166C	1; 2
YNL021W	8
YNL250W	2; 3; 8
YER003C	4; 7
YBL093C	6
YLR433C	11; 3; 5
YBR131W	6; 9
YOR028C	2
YDL212W	4; 5; 6
YPR024W	1; 4; 5
YLR240W	1; 5
YOR211C	1; 5; 8
YMR047C	1; 5
YKR063C	2
YBR024W	5
YOL067C	4
YCL054W	8
YDR513W	6
YDL200C	1; 8
YDL045W-A	4
YML032C	8
YMR257C	4
YJL174W	1; 3; 5
YKR001C	1; 2; 5
YGL130W	1
YNL090W	5
YDR030C	8
YML010W	8
YDR103W	3
YBL039C	5
YJL077C	6
YOR036W	1; 3; 5
YNL103W	4
YDR499W	2; 8

YCR044C	1; 3; 5; 6	YAR019C	2
YML007W	6; 8	YLR390W	10; 5
YKL056C	1	YNL292W	2
YOR254C	1; 2; 3	YGR121C	9
YBR021W	8	YGR253C	3; 5
YGR082W	1	YML016C	5
YER007W	1; 2; 5	YPR201W	6
YHR208W	2	YGR094W	4
YPL082C	3; 8	YGR262C	1
YIL002C	5	YBR029C	8
YML116W	9	YKL055C	4
YML085C	1; 5	YMR304W	1
YPR200C	6	YDR256C	6
YGL189C	1	YOL049W	1
YGL255W	1; 6	YCR005C	4
YOR322C	5	YKL117W	1
YLR079W	2	YPR035W	4
YKR061W	5	YFL045C	1; 5
YHR119W	3; 5; 8	YPL104W	4
YDL029W	5	YDL042C	8
YIR017C	4; 6	YML110C	4
YFL034C-B	5	YPL269W	2
YGL022W	1; 9	YOL036W	1
YGR218W	9	YIL123W	2
YOR075W	3; 5	YJL061W	1
YOR069W	5	YBR211C	5
YBR106W	4	YHR079C-A	2; 8
YMR238W	5	YCR098C	7
YLR055C	3; 8	YER151C	8
YFR051C	5	YDR099W	11
YGL178W	1; 3; 9	YDR265W	5
YBR243C	10	YHR010W	1
YOR140W	5	YER091C	4
YKL192C	4	YKL139W	1; 8
YDR456W	4	YOR386W	9
YKL110C	5	YDL207W	1
YPL167C	8	YDR129C	5
YBL023C	8	YGR057C	9
YNL236W	1; 5; 8; 9	YKL208W	4
YOR298W	3; 8	YBR136W	2; 8
YLR268W	1; 5	YBR088C	1; 2; 8
YPR086W	1; 8	YHL030W	10; 5
YJL209W	1; 4	YHL020C	7
YHR098C	1	YBL078C	3

YPR120C	2; 3	YML130C	1; 5; 9
YPL106C	1	YLR197W	1
YJL201W	5	YNL054W	1; 5
YDR097C	8	YMR038C	1; 4; 5; 6
YHR107C	1; 2; 5	YGL006W	6
YNR006W	5	YOR217W	1; 2; 8
YDR108W	3; 8	YNL233W	5
YMR234W	10; 5; 9	YBL099W	4
YCR020C	4	YKL112W	8
YLR103C	2; 8	YBL042C	8
YML012W	5	YLR436C	5
YKL085W	4	YDR298C	4
YMR137C	1; 8	YFL026W	3
YOR109W	5	YNL215W	1
YMR065W	3	YBR079C	1; 2
YML121W	1; 6	YDL101C	8
YBR133C	2; 5	YIL118W	1
YBR275C	8	YIL004C	5
YHR038W	1; 4	YLR298C	1
YKL093W	4; 6	YMR106C	1; 8; 9
YNL322C	5	YDL145C	1; 5
YLR451W	9	YBR202W	2; 8
YMR152W	4	YPR101W	1
YGL093W	1	YJL121C	6
YPL145C	1	YJR006W	1; 2; 8
YIL147C	10; 5; 9	YMR145C	4
YER017C	4	YDR328C	2
YPR057W	1	YCL051W	5
YKL185W	5	YEL030W	10; 5; 7
YKL011C	4	YIR004W	5
YML105C	1; 4	YDR364C	1; 2; 8
YNL003C	4	YOR361C	1; 2; 3
YPL241C	1; 2; 5	YMR036C	2
YPL214C	4	YLL041C	4
YOR116C	8	YEL034W	1
YDR440W	8	YNL088W	8; 9
YDR446W	5	YMR179W	8
YLR263W	3; 8	YBR158W	1; 3
YHR200W	9	YKL149C	5
YDL120W	4; 6	YDR226W	1; 4
YCR048W	7	YGR244C	4
YGL040C	4	YIL129C	3
YJL203W	1; 8	YPR119W	1
YDL090C	1; 2	YKR068C	5

YBL022C	4; 5
YHR168W	1
YNL219C	5; 9
YIL140W	5
YDR011W	6
YBR290W	4; 6
YGR036C	1; 5
YHR067W	1
YBL002W	1; 4; 5
YDR251W	1
YFL025C	5
YPL065W	5
YLR442C	2; 8
YOR363C	5
YGR252W	1; 9
YCL005W-A	5
YPR122W	3; 5
YBL008W	8
YJL080C	1; 5
YDL064W	2
YDR322C-A	1; 4; 5
YOL021C	1
YHR001W-A	5
YCR024C	4
YPR159W	1; 5
YLR032W	8
YEL037C	5; 7; 8; 9
YER070W	2
YER164W	8
YDL077C	5
YOR341W	5
YGL116W	2
YPL179W	4
YBR037C	4
YBR057C	2; 3; 8
YKL073W	1; 6
YGL127C	8
YNL271C	2; 3; 5
YKL181W	1
YGR099W	5
YJR045C	6
YER013W	5; 8
YDR079W	4
YDL226C	1; 2

YOL109W	1; 4
YER142C	8
YDR155C	11; 1
YPR199C	6
YIR024C	1; 2
YNR019W	3
YGL048C	2; 8; 9
YNL006W	9
YNL020C	5
YCR021C	1; 6
YOR270C	5
YOL044W	5
YML129C	4
YLR200W	1; 5
YBR078W	5
YJL059W	9
YMR301C	1; 4; 6
YGR041W	5
YKL020C	1
YDL087C	2
YIL126W	1; 2; 4; 5
YDL232W	10; 1; 5; 7
YOR326W	1; 5
YOR259C	1; 2
YBL052C	3
YNL037C	4
YHL031C	1; 5
YBR076W	5
YGL207W	2
YMR094W	1; 2
YDR076W	2; 8
YGR098C	2; 5
YER171W	8
YLL004W	2; 8
YGL181W	5
YLR127C	1; 2; 3
YPR190C	1
YGR216C	1; 5
YAL002W	1; 5
YKL049C	2
YDR331W	7
YPR173C	3; 5; 6
YOR358W	4
YNR010W	1; 2

YFR031C	2
YDL134C	2
YMR273C	5; 6
YBL079W	1
YNL066W	6
YEL063C	8
YGL115W	4; 8
YLR093C	5
YGL191W	4
YCR106W	9
YMR154C	1; 3; 5
YGR152C	5
YCR008W	5; 6
YJR040W	4
YDL185W	1; 6; 9
YJL001W	1
YCR092C	8
YER042W	6
YFL005W	7
YAL035W	1
YDL229W	1
YDR523C	3; 5
YEL003W	1; 5
YGR258C	8
YBR017C	1
YNL135C	11; 1
YOL104C	2; 3
YDR377W	4
YCR034W	11; 1; 3; 5; 6; 7
YLL026W	1
YHR013C	3
YGR213C	9
YAL026C	1; 6
YNL210W	2
YLR223C	1
YJR053W	2
YHR068W	1; 2
YLR244C	1
YLL060C	1; 6
YKL190W	5
YHR132C	10; 5
YPL031C	1; 3; 4; 5
YHR027C	1; 9
YOL116W	10; 4; 5; 9
YKR048C	2; 5
YDL165W	2
YOR017W	4
YKL028W	1; 6
YBR001C	1
YLL008W	1
YGR276C	1
YJR035W	8
YOR278W	1; 4
YOL090W	8
YBR189W	1
YLR288C	5; 8; 9
YDR515W	6
YOR332W	1; 4; 5
YBR123C	5
YPR051W	1; 4
YML008C	1; 2; 5; 7; 9
YLR098C	8
YPL234C	5
YLR314C	1; 2; 5
YGL212W	1; 5
YAL059W	5
YMR133W	2
YHL007C	3; 5
YFL036W	5; 8
YDR025W	10; 1
YCR037C	6
YER057C	6
YCR046C	4
YOR344C	3; 8
YMR078C	1
YHR120W	1; 4
YJR138W	2
YPR113W	3
YDR392W	3
YBL003C	1; 4; 5
YOR330C	4
YCR067C	5
YHL035C	1
YOL139C	3
YLR430W	1
YMR282C	4
YMR307W	5; 9
YJR065C	5; 6

YJL090C	2; 8
YHR206W	6
YBR127C	1; 4; 6
YJR017C	5
YGL213C	3
YBL066C	3
YIR021W	4
YKL165C	3
YJL074C	2
YCR042C	1
YDR123C	2; 3; 7
YIL051C	4; 5
YLR233C	8
YDR213W	6
YBR098W	8
YDR218C	3
YIL115C	1; 5
YMR165C	4; 5
YJR073C	7; 8
YJL023C	4
YLR443W	5
YLR329W	2
YJR021C	2; 8
YDL102W	1; 2; 8
YDR138W	1; 4; 8
YHR141C	9
YOR349W	1; 2; 5
YBR135W	2
YHR129C	5
YPL209C	1; 2; 5
YIR006C	2; 5
YIL142W	1; 5
YOL122C	6
YLR378C	1; 5
YDR238C	5
YPR133W-A	1; 4
YOR231W	4; 6
YIL026C	5
YDR303C	2
YGR155W	4; 5
YDR402C	5
YJL194W	2; 5; 8
YHL038C	4
YGR167W	1
YJR066W	11
YHR047C	4
YDR423C	6
YBL082C	5; 7
YML061C	1; 8
YHR101C	1; 2; 5
YIL153W	11; 6; 7; 9
YGR195W	10; 5; 9
YKR053C	7
YNR003C	10; 5
YBL007C	1; 5
YCL043C	1
YER068W	1; 3
YFL002C	1
YGR108W	2
YPL194W	8
YHR174W	1
YDL055C	5
YGR059W	3
YKL197C	5
YEL013W	1; 5
YKL113C	1; 2; 8; 9
YMR032W	1; 2
YML035C	1
YDR264C	1; 3; 5; 9
YNR026C	5
YAL058W	5
YGL163C	8
YMR284W	1; 2
YAL016W	1; 2; 8
YBR044C	4
YHR051W	1; 6
YKL109W	4
YNL053W	3
YOR351C	2; 3
YLR384C	5
YMR080C	9
YCR028C-A	3; 5
YML078W	1; 4
YOR181W	1; 2; 5; 9
YOL081W	3; 6
YMR037C	6
YGR075C	1
YNR001C	4

YOR014W	1; 2; 5	YER168C	1
YPL153C	2; 8	YPR042C	10; 9
YAL020C	1; 2; 5	YPL254W	1; 4
YMR013C	5	YER173W	2; 8
YDR082W	1	YDL195W	1; 5
YHR193C	4	YNL102W	2
YCR094W	1; 6; 8; 9	YJL165C	10; 5
YMR089C	4	YIR038C	1; 6
YGR118W	10	YBR126C	3; 4; 5
YNL098C	1; 3; 4	YOL147C	4; 5
YMR176W	10; 5; 9	YLR439W	4; 5
YBR107C	2; 5	YDL005C	1; 4
YGR188C	5	YNL055C	4
YER153C	4	YOR195W	2; 5
YDR254W	2; 5	YOR265W	5
YPL154C	6	YKL087C	4
YPL163C	6	YNL106C	5
YJL095W	1; 3; 4; 5; 6	YDR277C	4
YER111C	1; 2; 5	YCR073C	10
YPR198W	1; 3; 4	YFL029C	1; 2; 5
YER141W	4	YDR484W	1
YML001W	5	YHR084W	1; 2; 5; 8
YNL243W	1; 5	YBR081C	1; 3; 4; 7; 8
YBR055C	1	YNL030W	1; 8
YBL061C	3; 5	YML115C	10; 2; 3; 5; 6
YCR081W	6; 9	YGL097W	5
YAL013W	1; 3; 4; 5	YBR192W	4
YNL172W	2; 5	YLL036C	1; 8
YEL019C	1; 8	YDL198C	1
YNL325C	3	YMR280C	4; 8
YIL034C	5	YNL188W	2; 5
YNL186W	8	YLR038C	1; 4
YLR092W	6	YFL008W	2
YBR203W	8	YDR178W	4
YOR160W	1	YNR031C	5; 9
YLR453C	8	YJL204C	1; 3
YOR190W	2	YAR050W	5; 8
YMR216C	1	YAL047C	1; 5
YPL152W	11	YLR208W	5
YLR138W	6	YGL027C	5
YNL307C	1; 5	YCR063W	1
YLR249W	10; 5	YDL006W	1; 3; 4; 5; 9
YDR113C	1; 2	YBR195C	8
YDR375C	4	YNL137C	1; 4

YBR038W	5	YER058W	4
YPL045W	1; 3; 5; 6	YHR030C	1; 4; 5; 6; 9
YOR329C	1; 5	YAL015C	6
YML091C	4	YHR042W	7
YDR414C	5	YJR031C	1
YLL043W	5	YLR025W	1; 3; 4
YFR033C	1; 4; 5	YGL180W	3; 5; 6
YDL220C	1; 2; 8	YHR158C	3; 5
YDL004W	4	YKR095W	8
YPL084W	1; 5; 9	YOR196C	4; 7
YKR004C	5	YBL030C	4
YKL089W	2; 5	YKL007W	5
YGL125W	4	YPL149W	5; 6
YML097C	1; 5	YML031W	1; 2; 5
YPR054W	3; 5	YBR065C	5; 9
YDR217C	2; 8	YGL137W	1; 5
YJL101C	1; 6	YDR074W	1
YBL071W-A	1; 2; 5	YBL043W	5; 9
YKL210W	6	YDR454C	10; 1
YOR122C	1; 5	YLR182W	5
YMR224C	2; 5; 8	YMR308C	1
YBL001C	5	YDR174W	1
YLR175W	1; 2	YHR039C-A	5
YLR237W	9	YPR166C	4; 5
YIR026C	3	YNL327W	2
YLR056W	2; 9	YGL013C	8; 9
YGL028C	5	YDR245W	1; 5; 7
YOL096C	4; 5; 6	YDL058W	1; 5
YGR140W	1; 2	YKR024C	1
YNL239W	9	YGL003C	1; 2
YLR369W	1; 4; 5	YLL039C	1; 3; 6; 9
YLL006W	1; 4; 5	YHR064C	1
YGR217W	1; 6	YDR170C	5; 7
YGL078C	1	YBL024W	10
YBL050W	5	YBL058W	1; 2; 3; 4
YBR026C	5	YGL238W	1
YKR019C	8	YIL063C	1
YJL167W	1	YKR086W	1
YDR343C	4	YDL106C	3
YPR033C	1	YJL006C	1
YOR127W	5	YOR162C	9
YNL076W	5; 9	YBR040W	3
YGL043W	8	YDR420W	5
YNL244C	10	YGR191W	6

YLR362W	3	YDL194W	10; 4; 5; 9
YLR234W	1; 2; 3; 8	YNL241C	4; 6
YHR135C	1; 5	YGL128C	1; 2
YNL139C	1; 8	YFL031W	10; 4; 9
YCR038C	5	YCL063W	6; 9
YPL129W	1; 3; 5	YML102W	8
YER016W	1; 2; 5	YDR028C	1; 4; 5
YDR087C	10; 1	YJL091C	1
YFR029W	9	YDR189W	5
YMR079W	5	YMR287C	4; 5; 8
YJL033W	5	YMR001C	1; 2
YDR388W	3; 5; 6	YCL007C	5
YFL009W	1; 2; 5	YML081C-A	4; 5
YGR274C	1	YER133W	2; 3; 4; 5
YPR019W	1; 2; 8	YCL055W	3
YOR158W	4; 5	YCR009C	3; 4; 5; 6; 9
YIL072W	3; 8	YGL216W	1; 2; 5
YDR477W	2; 4	YMR072W	4
YGL210W	5	YNL151C	1
YBR080C	5	YPL097W	4
YGL008C	9	YMR228W	4
YML057W	3	YIL073C	3
YJR051W	5	YJL196C	7
YPR135W	2; 5; 8	YKL212W	4
YNR023W	1; 4	YDL116W	1; 3; 5
YLR403W	1	YGL106W	2; 5
YDL154W	2; 3	YJR069C	9
YGR109C	2; 3	YNL153C	1; 3; 5
YJL157C	3	YJR046W	8
YML124C	3; 5	YIL048W	3
YML028W	6	YFL039C	1; 2; 5
YMR128W	10; 5	YLR450W	9
YDR125C	5	YGL190C	1; 5
