# Supplementary material for: Predicting Protein Phenotypes Based on Protein-Protein Interaction Network
Source: PLoS One. 2011 Mar 10;6(3):e17668. doi: 10.1371/journal.pone.0017668 (PMC3053377; doi:10.1371/journal.pone.0017668)
Supplement: Table S3 — The proteins and the complexes they belong to in yeast. The information was retrieved from CYGD (the Comprehensive Yeast Genome Database) (Guldener U, Munsterkotter M, Kastenmuller G, Strack N, van Helden J, et al. (2005) CYGD: the Comprehensive Yeast Genome Database. Nucleic acids research 33: D364-368.). (PDF) [file pone.0017668.s003.pdf]

Table S3. The proteins and the complexes they belong to in yeast. The information was retrieved from CYGD (the Comprehensive Yeast Genome Database) (Guldener U, Munsterkotter M, Kastenmuller G, Strack N, van Helden J, et al. (2005) CYGD: the Comprehensive Yeast Genome Database. Nucleic acids research 33: D364-368.).

Protein	Complex
Q0032	Complex Number 530
Q0045	Cytochrome c oxidase (complex IV)
Q0050	Complex Number 169
Q0080	Complex Number 77, probably membrane biogenesis and traffic
Q0080	F0/F1 ATP synthase (complex V)
Q0085	F0/F1 ATP synthase (complex V)
Q0092	Complex Number 537
Q0105	Cytochrome bc1 complex (Ubiquinol-cytochrome c reductase complex, complex III)
Q0120	Mitochondrial splicing complexes
Q0130	F0/F1 ATP synthase (complex V)
Q0140	mitochondrial ribosomal small subunit
Q0140	Mitochondrial ribosomes
Q0250	Cytochrome c oxidase (complex IV)
Q0275	Cytochrome c oxidase (complex IV)
RNA_RNASE-P	RNase P
RNA_TLC1	Telomerase
SNRNA_NME1	RNase MRP
YAL001C	Complex Number 178, probably transcription/DNA maintanance/chromatin structure
YAL001C	TFIIIC
YAL002W	Complex Number 111
YAL002W	Complex Number 77, probably membrane biogenesis and traffic
YAL003W	Complex Number 103, probably protein synthesis turnover
YAL003W	Complex Number 388
YAL003W	eEF1
YAL005C	Complex Number 91, probably protein synthesis turnover
YAL007C	Complex Number 69, probably membrane biogenesis and traffic
YAL007C	COPII
YAL009W	Complex Number 14, probably cell polarity and structure
YAL009W	Nem1p-Spo7p complex
YAL010C	Complex Number 128, probably protein/RNA transport
YAL012W	Complex Number 244
YAL012W	Complex Number 5
YAL012W	Complex Number 51, probably intermediate and energy metabolism
YAL013W	Complex Number 208, probably transcription/DNA maintanance/chromatin structure
YAL015C	Complex Number 207
YAL015C	Complex Number 208
YAL015C	Complex Number 284
YAL016W	Complex Number 151

YAL016W	Complex Number 170, probably signalling
YAL016W	Complex Number 232
YAL016W	Complex Number 233
YAL016W	Complex Number 356
YAL016W	Complex Number 435
YAL016W	Complex Number 460
YAL016W	Complex Number 48
YAL017W	Complex Number 105
YAL017W	Complex Number 161, probably signalling
YAL017W	Complex Number 207, probably transcription/DNA maintanance/chromatin structure
YAL017W	Complex Number 214, probably transcription/DNA maintanance/chromatin structure
YAL017W	Complex Number 533
YAL019W	Complex Number 183, probably transcription/DNA maintanance/chromatin structure
YAL019W	Complex Number 533
YAL021C	CCR4 complex
YAL021C	Complex Number 211, probably transcription/DNA maintanance/chromatin structure
YAL021C	Complex Number 31
YAL021C	Complex Number 65, CCR4 (3)
YAL024C	Complex Number 12, probably cell cycle
YAL024C	Complex Number 170, probably signalling
YAL025C	Complex Number 161
YAL026C	Complex Number 67, probably membrane biogenesis and traffic
YAL027W	Complex Number 136, probably RNA metabolism
YAL027W	Complex Number 257
YAL029C	Actin-associated motorproteins
YAL029C	Complex Number 155
YAL029C	Complex Number 17, probably cell polarity and structure
YAL029C	Complex Number 207, probably transcription/DNA maintanance/chromatin structure
YAL029C	Complex Number 308
YAL029C	Complex Number 345
YAL029C	Complex Number 62
YAL030W	v-SNAREs
YAL032C	Complex Number 143, probably RNA metabolism
YAL033W	Complex Number 154, probably RNA metabolism
YAL033W	RNase MRP
YAL033W	RNase P
YAL034C	Complex Number 214, probably transcription/DNA maintanance/chromatin structure
YAL035W	Complex Number 107, probably protein synthesis turnover
YAL035W	Complex Number 116, probably protein synthesis turnover
YAL035W	Complex Number 135, probably RNA metabolism
YAL035W	Complex Number 140, probably RNA metabolism
YAL035W	Complex Number 169
YAL035W	Complex Number 344
YAL035W	Complex Number 505

YAL035W	Complex Number 517
YAL035W	Complex Number 7, Dbp7/Rrp5 (4)
YAL035W	Complex Number 86, probably protein synthesis turnover
YAL035W	Complex Number 99, probably protein synthesis turnover
YAL036C	Complex Number 104
YAL036C	Complex Number 142, probably RNA metabolism
YAL038W	Pyruvate kinase
YAL040C	Cdc28p complexes
YAL041W	Complex Number 5, probably cell cycle
YAL041W	Complex Number 89, probably protein synthesis turnover
YAL042W	Complex Number 80, probably membrane biogenesis and traffic
YAL042W	COPII
YAL043C	Complex Number 125, probably protein/RNA transport
YAL043C	Complex Number 148, probably RNA metabolism
YAL043C	Complex Number 34, TFIIIC (4)
YAL043C	Complex Number 35, U1 snRNP (7)
YAL043C	Complex Number 36, Ynl313c (1)
YAL043C	Complex Number 56, mRNA cleavage/polyadenylation (10)
YAL043C	pre mRNA3'-end processing factor CFII
YAL043C	pre mRNA polyadenylation factor PFI
YAL044C	Glycine decarboxylase
YAL047C	Complex Number 46
YAL047C	SPB components
YAL048C	Complex Number 230, probably transcription/DNA maintanance/chromatin structure
YAL049C	Complex Number 440
YAL051W	OAF complex
YAL053W	Complex Number 67, probably membrane biogenesis and traffic
YAL059W	Complex Number 171, probably signalling
YAL059W	Complex Number 384
YAL061W	Complex Number 120, probably protein/RNA transport
YAR002C-A	Complex Number 69, probably membrane biogenesis and traffic
YAR002C-A	COPII
YAR002W	Complex Number 139, probably RNA metabolism
YAR002W	Complex Number 376
YAR003W	Complex Number 199, probably transcription/DNA maintanance/chromatin structure
YAR003W	Complex Number 425
YAR007C	Complex Number 14
YAR007C	Complex Number 195, probably transcription/DNA maintanance/chromatin structure
YAR007C	Complex Number 200, probably transcription/DNA maintanance/chromatin structure
YAR007C	Complex Number 202, probably transcription/DNA maintanance/chromatin structure
YAR007C	Complex Number 212, probably transcription/DNA maintanance/chromatin structure
YAR007C	Complex Number 292
YAR007C	Complex Number 293
YAR007C	Replication factor A complex

YAR008W	tRNA splicing
YAR009C	Complex Number 134
YAR014C	Complex Number 106, probably protein synthesis turnover
YAR014C	Complex Number 132
YAR014C	Complex Number 147
YAR018C	Complex Number 106, probably protein synthesis turnover
YAR019C	Complex Number 38
YAR019C	Complex Number 388
YAR019C	Complex Number 389
YAR019C	Complex Number 390
YAR019C	Complex Number 391
YAR019C	Complex Number 79
YAR042W	Complex Number 23, probably intermediate and energy metabolism
YAR042W	Complex Number 59, probably intermediate and energy metabolism
YAR073W	Complex Number 303
YAR073W	Complex Number 404
YAR073W	Complex Number 446
YBL002W	Complex Number 102
YBL002W	Complex Number 119
YBL002W	Complex Number 141
YBL002W	Complex Number 170
YBL002W	Complex Number 186
YBL002W	Complex Number 22
YBL002W	Complex Number 220, probably transcription/DNA maintanance/chromatin structure
YBL002W	Complex Number 261
YBL002W	Complex Number 446
YBL002W	Complex Number 473
YBL002W	Complex Number 474
YBL002W	Complex Number 486
YBL002W	Complex Number 491
YBL002W	Complex Number 507
YBL002W	Complex Number 529
YBL002W	Complex Number 534
YBL002W	Complex Number 535
YBL002W	Complex Number 56
YBL002W	Nucleosomal protein complex
YBL003C	Complex Number 125, probably protein/RNA transport
YBL003C	Complex Number 144, probably RNA metabolism
YBL003C	Complex Number 146
YBL003C	Complex Number 208, probably transcription/DNA maintanance/chromatin structure
YBL003C	Complex Number 233
YBL003C	Complex Number 278
YBL003C	Complex Number 534
YBL003C	Nucleosomal protein complex

YBL004W	Complex Number 104, probably protein synthesis turnover
YBL004W	Complex Number 108, probably protein synthesis turnover
YBL004W	Complex Number 109, probably protein synthesis turnover
YBL004W	Complex Number 125, probably protein/RNA transport
YBL004W	Complex Number 140, probably RNA metabolism
YBL004W	Complex Number 141, probably RNA metabolism
YBL004W	Complex Number 142, probably RNA metabolism
YBL004W	Complex Number 144, probably RNA metabolism
YBL004W	Complex Number 149, probably RNA metabolism
YBL004W	Complex Number 197, probably transcription/DNA maintanance/chromatin structure
YBL004W	Complex Number 204, probably transcription/DNA maintanance/chromatin structure
YBL004W	Complex Number 22
YBL004W	Complex Number 321
YBL004W	Complex Number 412
YBL004W	Complex Number 446
YBL004W	Complex Number 507
YBL007C	Actin-associated proteins
YBL007C	Complex Number 155
YBL007C	Complex Number 156
YBL007C	Complex Number 18, probably cell polarity and structure
YBL008W	Complex Number 150
YBL014C	Core Factor (CF)
YBL015W	Complex Number 267
YBL015W	Complex Number 298
YBL015W	Complex Number 316
YBL015W	Complex Number 341
YBL016W	Complex Number 194
YBL016W	Complex Number 289
YBL016W	STE5-MAPK complex
YBL017C	Complex Number 155
YBL017C	Complex Number 156
YBL017C	Complex Number 227, probably transcription/DNA maintanance/chromatin structure
YBL017C	Complex Number 72, Pep1 (1)
YBL018C	Complex Number 154, probably RNA metabolism
YBL018C	RNase MRP
YBL018C	RNase P
YBL021C	CCAAT-binding factor complex
YBL021C	Complex Number 124
YBL021C	Complex Number 217, probably transcription/DNA maintanance/chromatin structure
YBL022C	Complex Number 152
YBL022C	Complex Number 369
YBL022C	Complex Number 518
YBL022C	Complex Number 62, probably intermediate and energy metabolism
YBL022C	Lon protease complex (homohexamer)

YBL023C	Complex Number 10, probably cell cycle
YBL023C	Complex Number 29, probably intermediate and energy metabolism
YBL023C	Complex Number 82
YBL023C	Pre-replication complex (pre-RC)
YBL023C	Replication complex
YBL024W	Complex Number 152, probably RNA metabolism
YBL025W	Upstream Activation Factor (UAF) complex
YBL026W	Complex Number 138, probably RNA metabolism
YBL026W	Complex Number 147, probably RNA metabolism
YBL026W	Complex Number 163
YBL026W	Complex Number 165
YBL026W	Complex Number 22
YBL026W	Complex Number 507
YBL027W	cytoplasmic ribosomal large subunit
YBL029W	Complex Number 26
YBL030C	Complex Number 111
YBL030C	Complex Number 146
YBL030C	Complex Number 338
YBL032W	Complex Number 142, probably RNA metabolism
YBL034C	Complex Number 17
YBL034C	Complex Number 23, RNA Polymerase III (12)
YBL034C	SPB associated proteins
YBL034C	Tubulin-associated proteins
YBL035C	Complex Number 211, probably transcription/DNA maintanance/chromatin structure
YBL035C	Complex Number 228, probably transcription/DNA maintanance/chromatin structure
YBL035C	DNA polymerase alpha (I) - primase complex
YBL036C	Complex Number 426
YBL037W	AP-2 complex
YBL037W	Complex Number 78, probably membrane biogenesis and traffic
YBL038W	Complex Number 108, probably protein synthesis turnover
YBL038W	mitochondrial ribosomal large subunit
YBL039C	Complex Number 23
YBL039C	Complex Number 328
YBL039C	Complex Number 383
YBL039C	Complex Number 41, probably intermediate and energy metabolism
YBL039C	Complex Number 469
YBL039C	Complex Number 88, Ura7 (1)
YBL039C	CTP synthetase
YBL041W	20S proteasome
YBL041W	Complex Number 110, probably protein synthesis turnover
YBL041W	Complex Number 111, probably protein synthesis turnover
YBL041W	Complex Number 170, probably signalling
YBL041W	Complex Number 238
YBL041W	Complex Number 60, 20S Proteosome (13)

YBL044W	Complex Number 22
YBL044W	Complex Number 507
YBL044W	Complex Number 96, probably protein synthesis turnover
YBL045C	Complex Number 132
YBL045C	Complex Number 186
YBL045C	Complex Number 218
YBL045C	Complex Number 327
YBL045C	Complex Number 338
YBL045C	Complex Number 341
YBL045C	Complex Number 350
YBL045C	Complex Number 362
YBL045C	Complex Number 363
YBL045C	Complex Number 369
YBL045C	Complex Number 388
YBL045C	Complex Number 457
YBL045C	Complex Number 46, probably intermediate and energy metabolism
YBL045C	Complex Number 497
YBL045C	Complex Number 70
YBL045C	Cytochrome bc1 complex (Ubiquinol-cytochrome c reductase complex, complex III)
YBL046W	Complex Number 169, probably signalling
YBL046W	Complex Number 234
YBL047C	Complex Number 132
YBL047C	Complex Number 133
YBL047C	Complex Number 20, probably cell polarity and structure
YBL047C	Complex Number 279
YBL047C	Complex Number 534
YBL047C	Complex Number 535
YBL047C	Complex Number 62
YBL049W	Complex Number 427
YBL049W	Complex Number 437
YBL049W	Complex Number 462
YBL049W	Complex Number 479
YBL050W	Complex Number 71, probably membrane biogenesis and traffic
YBL050W	NSF-SNAP complex
YBL051C	Complex Number 131, probably RNA metabolism
YBL051C	Complex Number 132
YBL056W	Complex Number 176, probably signalling
YBL056W	Complex Number 250
YBL056W	Complex Number 282
YBL056W	Complex Number 283
YBL058W	Complex Number 261
YBL058W	Complex Number 413
YBL058W	Complex Number 431
YBL058W	Complex Number 432

YBL058W	Complex Number 9, probably cell cycle
YBL061C	Complex Number 451
YBL063W	Complex Number 21, probably cell polarity and structure
YBL063W	Kinesin-related motorproteins
YBL064C	Complex Number 212
YBL064C	Complex Number 97, probably protein synthesis turnover
YBL066C	Complex Number 451
YBL072C	cytoplasmic ribosomal small subunit
YBL074C	Complex Number 140, probably RNA metabolism
YBL074C	Complex Number 147, probably RNA metabolism
YBL074C	Complex Number 62, Aar2 (1)
YBL074C	mRNA splicing
YBL075C	Complex Number 135, probably RNA metabolism
YBL075C	Complex Number 148, probably RNA metabolism
YBL076C	Complex Number 121, probably protein/RNA transport
YBL076C	Complex Number 244
YBL076C	Complex Number 316
YBL076C	Complex Number 34, TFIIIC (4)
YBL076C	Complex Number 379
YBL076C	Complex Number 506
YBL076C	Complex Number 62
YBL076C	Complex Number 82
YBL076C	Complex Number 95, probably protein synthesis turnover
YBL078C	Clathrin
YBL078C	Tubulin-associated proteins
YBL079W	Nuclear pore complex (NPC)
YBL080C	Complex Number 540
YBL080C	Mitochondrial translation complexes
YBL084C	Anaphase promoting complex (APC)
YBL084C	Complex Number 3, probably cell cycle
YBL085W	Complex Number 164, probably signalling
YBL087C	Complex Number 64, Bcp1/Rpl23A (3)
YBL087C	Complex Number 85, probably protein synthesis turnover
YBL087C	cytoplasmic ribosomal large subunit
YBL088C	Complex Number 183, probably transcription/DNA maintanance/chromatin structure
YBL088C	Complex Number 204, probably transcription/DNA maintanance/chromatin structure
YBL088C	Complex Number 387
YBL090W	Complex Number 104, probably protein synthesis turnover
YBL090W	mitochondrial ribosomal small subunit
YBL091C	Complex Number 299
YBL092W	cytoplasmic ribosomal large subunit
YBL093C	Complex Number 209, probably transcription/DNA maintanance/chromatin structure
YBL093C	Kornberg's mediator (SRB) complex
YBL099W	F0/F1 ATP synthase (complex V)

YBL103C	RTG complex
YBL104C	Complex Number 170, probably signalling
YBL104C	Complex Number 455
YBL104C	Complex Number 6
YBL104C	Complex Number 82
YBL105C	Complex Number 174, probably signalling
YBL108W	Complex Number 435
YBR001C	Complex Number 161, probably signalling
YBR009C	Complex Number 100
YBR009C	Complex Number 101
YBR009C	Complex Number 132
YBR009C	Complex Number 137
YBR009C	Complex Number 141
YBR009C	Complex Number 157
YBR009C	Complex Number 200
YBR009C	Complex Number 201
YBR009C	Complex Number 204, probably transcription/DNA maintanance/chromatin structure
YBR009C	Complex Number 22
YBR009C	Complex Number 253
YBR009C	Complex Number 261
YBR009C	Complex Number 262
YBR009C	Complex Number 303
YBR009C	Complex Number 315
YBR009C	Complex Number 319
YBR009C	Complex Number 341
YBR009C	Complex Number 386
YBR009C	Complex Number 507
YBR009C	Complex Number 513
YBR009C	Complex Number 530
YBR009C	Complex Number 534
YBR009C	Complex Number 56
YBR009C	Complex Number 74
YBR009C	Nucleosomal protein complex
YBR010W	Complex Number 154
YBR010W	Complex Number 170
YBR010W	Complex Number 183
YBR010W	Complex Number 56
YBR010W	Nucleosomal protein complex
YBR011C	Complex Number 123
YBR011C	Complex Number 132
YBR011C	Complex Number 206
YBR011C	Complex Number 27
YBR011C	Complex Number 278
YBR011C	Complex Number 360

YBR011C	Complex Number 4
YBR011C	Complex Number 55, probably intermediate and energy metabolism
YBR011C	Complex Number 62
YBR011C	Complex Number 88
YBR014C	Complex Number 442
YBR017C	Complex Number 123, probably protein/RNA transport
YBR017C	Complex Number 146
YBR017C	Complex Number 69, Nab2/Kap104 (2)
YBR018C	Complex Number 29
YBR018C	Complex Number 338
YBR018C	Complex Number 360
YBR018C	Complex Number 363
YBR018C	Complex Number 504
YBR018C	Complex Number 505
YBR018C	Complex Number 88
YBR025C	Complex Number 103, probably protein synthesis turnover
YBR025C	Complex Number 205, probably transcription/DNA maintanance/chromatin structure
YBR025C	Complex Number 212, probably transcription/DNA maintanance/chromatin structure
YBR025C	Complex Number 244
YBR025C	Complex Number 341
YBR025C	Complex Number 385
YBR025C	Complex Number 459
YBR025C	Complex Number 89, Ybr025c (1)
YBR028C	Complex Number 435
YBR031W	Complex Number 38, Arx1 Complex (5)
YBR031W	cytoplasmic ribosomal large subunit
YBR035C	Complex Number 90
YBR039W	Complex Number 123
YBR039W	Complex Number 13
YBR039W	Complex Number 26
YBR039W	Complex Number 271
YBR039W	Complex Number 272
YBR039W	Complex Number 283
YBR039W	Complex Number 297
YBR039W	Complex Number 341
YBR039W	Complex Number 353
YBR039W	Complex Number 364
YBR039W	Complex Number 37
YBR039W	Complex Number 374
YBR039W	Complex Number 486
YBR039W	Complex Number 504
YBR039W	Complex Number 505
YBR039W	Complex Number 535
YBR039W	Complex Number 61

YBR039W	Complex Number 69
YBR039W	F0/F1 ATP synthase (complex V)
YBR044C	Complex Number 42, probably intermediate and energy metabolism
YBR048W	cytoplasmic ribosomal small subunit
YBR049C	Complex Number 200, probably transcription/DNA maintanance/chromatin structure
YBR049C	Complex Number 376
YBR049C	Complex Number 77, Reb1 (1)
YBR055C	Complex Number 138, probably RNA metabolism
YBR055C	Complex Number 145, probably RNA metabolism
YBR055C	Complex Number 146, probably RNA metabolism
YBR055C	Complex Number 147, probably RNA metabolism
YBR055C	Complex Number 14, U6-specific snRNP core (14)
YBR055C	Complex Number 244
YBR055C	mRNA splicing
YBR057C	Complex Number 13, probably cell cycle
YBR057C	Complex Number 511
YBR058C	Complex Number 95, probably protein synthesis turnover
YBR059C	Complex Number 239
YBR059C	Complex Number 3
YBR059C	Complex Number 355
YBR060C	Complex Number 10, probably cell cycle
YBR060C	Post-replication complex (Origin recognition complex=ORC)
YBR060C	Pre-replication complex (pre-RC)
YBR060C	Replication complex
YBR060C	Replication initiation complex
YBR061C	Complex Number 85, Trm7 (2)
YBR063C	Complex Number 422
YBR065C	Complex Number 143, probably RNA metabolism
YBR065C	Complex Number 145, probably RNA metabolism
YBR065C	Complex Number 146, probably RNA metabolism
YBR065C	Complex Number 147, probably RNA metabolism
YBR069C	Complex Number 119, probably protein/RNA transport
YBR078W	Complex Number 134
YBR079C	Complex Number 107, probably protein synthesis turnover
YBR079C	Complex Number 135, probably RNA metabolism
YBR079C	Complex Number 149, probably RNA metabolism
YBR079C	Complex Number 189, probably transcription/DNA maintanance/chromatin structure
YBR079C	Complex Number 204, probably transcription/DNA maintanance/chromatin structure
YBR079C	Complex Number 209, probably transcription/DNA maintanance/chromatin structure
YBR079C	Complex Number 379
YBR079C	Complex Number 395
YBR079C	Complex Number 51, eIF3 (7)
YBR079C	Complex Number 535
YBR079C	eIF3

YBR080C	Complex Number 23
YBR080C	Complex Number 338
YBR080C	Complex Number 4
YBR080C	NSF-SNAP complex
YBR081C	Complex Number 112
YBR081C	Complex Number 206, probably transcription/DNA maintanance/chromatin structure
YBR081C	Complex Number 207, probably transcription/DNA maintanance/chromatin structure
YBR081C	Complex Number 209, probably transcription/DNA maintanance/chromatin structure
YBR081C	Complex Number 214, probably transcription/DNA maintanance/chromatin structure
YBR081C	Complex Number 324
YBR081C	Complex Number 382
YBR081C	SAGA complex
YBR081C	SAGA-like complex (SLIK)
YBR082C	Complex Number 411
YBR083W	Complex Number 152
YBR083W	Complex Number 386
YBR084C-A	cytoplasmic ribosomal large subunit
YBR084W	Complex Number 106, probably protein synthesis turnover
YBR084W	Complex Number 140, probably RNA metabolism
YBR084W	Complex Number 142, probably RNA metabolism
YBR084W	Complex Number 149, probably RNA metabolism
YBR084W	Complex Number 152, probably RNA metabolism
YBR084W	Complex Number 183, probably transcription/DNA maintanance/chromatin structure
YBR084W	Complex Number 193, probably transcription/DNA maintanance/chromatin structure
YBR084W	Complex Number 197, probably transcription/DNA maintanance/chromatin structure
YBR084W	Complex Number 204, probably transcription/DNA maintanance/chromatin structure
YBR084W	Complex Number 228, probably transcription/DNA maintanance/chromatin structure
YBR084W	Complex Number 84, probably membrane biogenesis and traffic
YBR084W	Complex Number 86, probably protein synthesis turnover
YBR084W	Complex Number 99, probably protein synthesis turnover
YBR085W	Complex Number 123
YBR085W	Complex Number 155
YBR085W	Complex Number 23
YBR085W	Complex Number 271
YBR085W	Complex Number 272
YBR085W	Complex Number 283
YBR085W	Complex Number 296
YBR085W	Complex Number 364
YBR085W	Complex Number 375
YBR085W	Complex Number 4
YBR085W	Complex Number 435
YBR086C	Complex Number 107, probably protein synthesis turnover
YBR087W	Complex Number 205, probably transcription/DNA maintanance/chromatin structure
YBR087W	Complex Number 228, probably transcription/DNA maintanance/chromatin structure

YBR087W	Complex Number 264
YBR087W	Complex Number 265
YBR087W	Complex Number 299
YBR087W	Complex Number 300
YBR087W	Replication factor C complex
YBR088C	Complex Number 229
YBR088C	Complex Number 230
YBR088C	Complex Number 268
YBR088C	Complex Number 461
YBR088C	Complex Number 50
YBR088C	PCNA
YBR089C-A	Complex Number 200, probably transcription/DNA maintanance/chromatin structure
YBR091C	Tim22p-complex
YBR094W	Complex Number 428
YBR095C	Complex Number 208, probably transcription/DNA maintanance/chromatin structure
YBR097W	Complex Number 176, probably signalling
YBR097W	Intracellular transport complexes
YBR098W	Complex Number 200
YBR101C	Complex Number 132, probably RNA metabolism
YBR102C	Complex Number 81, probably membrane biogenesis and traffic
YBR103W	Complex Number 101, probably protein synthesis turnover
YBR103W	Complex Number 170, probably signalling
YBR103W	Complex Number 219, probably transcription/DNA maintanance/chromatin structure
YBR103W	Complex Number 347
YBR103W	Complex Number 376
YBR105C	Complex Number 148, probably RNA metabolism
YBR105C	Complex Number 437
YBR107C	Chl4 protein complex
YBR107C	Complex Number 221, probably transcription/DNA maintanance/chromatin structure
YBR109C	Complex Number 134, probably RNA metabolism
YBR109C	Complex Number 62
YBR109C	Complex Number 63
YBR109C	Complex Number 64
YBR109C	Complex Number 65
YBR109C	SPB components
YBR112C	Complex Number 405
YBR112C	Tup1/Ssn6 complex
YBR114W	Complex Number 212, probably transcription/DNA maintanance/chromatin structure
YBR114W	Complex Number 230, probably transcription/DNA maintanance/chromatin structure
YBR114W	Complex Number 259
YBR114W	Complex Number 260
YBR114W	Complex Number 261
YBR114W	Complex Number 262
YBR114W	NEF4 complex

YBR115C	Complex Number 171, probably signalling
YBR115C	Complex Number 57, probably intermediate and energy metabolism
YBR115C	L-aminopadipate-semialdehyde dehydrogenase
YBR117C	Complex Number 28, probably intermediate and energy metabolism
YBR118W	Complex Number 103, probably protein synthesis turnover
YBR118W	eEF1
YBR119W	Complex Number 139, probably RNA metabolism
YBR119W	Complex Number 145, probably RNA metabolism
YBR119W	Complex Number 146, probably RNA metabolism
YBR119W	Complex Number 147, probably RNA metabolism
YBR119W	Complex Number 35, U1 snRNP (7)
YBR120C	Complex Number 111
YBR120C	Complex Number 146
YBR120C	Mitochondrial splicing complexes
YBR120C	Mitochondrial translation complexes
YBR121C	Complex Number 244
YBR121C	Complex Number 379
YBR121C	Complex Number 517
YBR122C	Complex Number 108, probably protein synthesis turnover
YBR122C	mitochondrial ribosomal large subunit
YBR123C	Complex Number 178, probably transcription/DNA maintanance/chromatin structure
YBR123C	Complex Number 34, TFIIIC (4)
YBR123C	TFIIIC
YBR125C	Complex Number 132
YBR125C	Complex Number 177, probably signalling
YBR125C	Complex Number 251
YBR125C	Complex Number 451
YBR126C	Complex Number 134
YBR126C	Complex Number 149
YBR126C	Complex Number 164
YBR126C	Complex Number 244
YBR126C	Complex Number 29, probably intermediate and energy metabolism
YBR126C	Complex Number 308
YBR126C	Complex Number 37
YBR126C	Complex Number 379
YBR126C	Complex Number 418
YBR126C	Complex Number 439
YBR126C	Complex Number 451
YBR126C	Complex Number 49
YBR126C	Complex Number 504
YBR126C	Complex Number 77
YBR127C	Complex Number 45, probably intermediate and energy metabolism
YBR127C	H+-transporting ATPase, vacuolar
YBR128C	Complex Number 173, probably signalling

YBR129C	Complex Number 284
YBR130C	Complex Number 17, probably cell polarity and structure
YBR130C	Complex Number 345
YBR130C	Complex Number 62
YBR133C	Complex Number 244
YBR133C	Complex Number 381
YBR135W	Complex Number 58
YBR135W	Complex Number 60
YBR135W	Complex Number 6, probably cell cycle
YBR135W	Complex Number 82
YBR136W	Complex Number 175
YBR136W	Complex Number 176
YBR136W	Complex Number 197, probably transcription/DNA maintanance/chromatin structure
YBR136W	Complex Number 202, probably transcription/DNA maintanance/chromatin structure
YBR136W	Complex Number 212, probably transcription/DNA maintanance/chromatin structure
YBR139W	Complex Number 31, probably intermediate and energy metabolism
YBR139W	Complex Number 434
YBR140C	Complex Number 288
YBR140C	Complex Number 308
YBR142W	Complex Number 149, probably RNA metabolism
YBR142W	Complex Number 480
YBR142W	mRNA splicing
YBR143C	Complex Number 107, probably protein synthesis turnover
YBR143C	Complex Number 197, probably transcription/DNA maintanance/chromatin structure
YBR143C	Complex Number 345
YBR143C	Complex Number 375
YBR143C	Complex Number 82, Translation release factor (2)
YBR143C	eRF1
YBR145W	Complex Number 157
YBR146W	Complex Number 104, probably protein synthesis turnover
YBR146W	mitochondrial ribosomal small subunit
YBR149W	Complex Number 244
YBR149W	D-arabinose dehydrogenase
YBR150C	Complex Number 316
YBR152W	Complex Number 147, probably RNA metabolism
YBR152W	mRNA splicing
YBR154C	Complex Number 19, mRNA cap-binding/eIF4F (8)
YBR154C	Complex Number 210, probably transcription/DNA maintanance/chromatin structure
YBR154C	Complex Number 213, probably transcription/DNA maintanance/chromatin structure
YBR154C	Complex Number 23, RNA Polymerase III (12)
YBR154C	Complex Number 24, RNA Polymerase II (12)
YBR154C	Complex Number 25, RNA polymerase I (7)
YBR154C	Complex Number 314
YBR154C	Complex Number 316

YBR154C	Complex Number 322
YBR154C	Complex Number 37, Dst1 (1)
YBR154C	RNA polymerase I
YBR154C	RNA polymerase II
YBR154C	RNA polymerase III
YBR155W	Complex Number 43, probably intermediate and energy metabolism
YBR155W	Complex Number 67
YBR158W	Complex Number 106, probably protein synthesis turnover
YBR158W	Complex Number 11, probably cell cycle
YBR160W	Cdc28p complexes
YBR160W	Complex Number 101
YBR160W	Complex Number 41
YBR160W	Complex Number 52
YBR160W	Complex Number 58
YBR160W	Complex Number 59
YBR160W	Complex Number 60
YBR160W	Complex Number 61
YBR160W	Complex Number 6, probably cell cycle
YBR162C	Complex Number 79, probably membrane biogenesis and traffic
YBR166C	Complex Number 4
YBR167C	Complex Number 154, probably RNA metabolism
YBR167C	RNase MRP
YBR167C	RNase P
YBR169C	Complex Number 209, probably transcription/DNA maintanance/chromatin structure
YBR169C	Complex Number 215, probably transcription/DNA maintanance/chromatin structure
YBR169C	Complex Number 34, probably intermediate and energy metabolism
YBR170C	Complex Number 413
YBR170C	Complex Number 9, probably cell cycle
YBR171W	Complex Number 72, probably membrane biogenesis and traffic
YBR171W	Sec62-63 complex
YBR172C	Actin-associated motorproteins
YBR173C	20S proteasome
YBR175W	Complex Number 199, probably transcription/DNA maintanance/chromatin structure
YBR175W	Complex Number 429
YBR179C	Complex Number 221
YBR181C	cytoplasmic ribosomal small subunit
YBR184W	Complex Number 271
YBR187W	Complex Number 338
YBR188C	Nuclear splicing complexes/Spliceosome
YBR189W	cytoplasmic ribosomal small subunit
YBR191W	cytoplasmic ribosomal large subunit
YBR193C	Complex Number 209, probably transcription/DNA maintanance/chromatin structure
YBR193C	Kornberg's mediator (SRB) complex
YBR195C	Chromatin assembly complex (CAC)

YBR195C	Complex Number 198
YBR195C	Complex Number 257
YBR196C	Complex Number 136, probably RNA metabolism
YBR196C	Complex Number 65, probably membrane biogenesis and traffic
YBR198C	Complex Number 108, probably protein synthesis turnover
YBR198C	Complex Number 196, probably transcription/DNA maintanance/chromatin structure
YBR198C	Complex Number 207, probably transcription/DNA maintanance/chromatin structure
YBR198C	Complex Number 214, probably transcription/DNA maintanance/chromatin structure
YBR198C	Complex Number 385
YBR198C	SAGA complex
YBR198C	TAFIIs
YBR200W	Actin-associated proteins
YBR200W	Complex Number 5, probably cell cycle
YBR202W	Complex Number 10, probably cell cycle
YBR202W	Complex Number 13
YBR202W	Pre-replication complex (pre-RC)
YBR202W	Replication complex
YBR203W	Complex Number 430
YBR205W	Complex Number 146
YBR205W	Complex Number 417
YBR205W	Complex Number 70, probably membrane biogenesis and traffic
YBR207W	Complex Number 53, probably intermediate and energy metabolism
YBR208C	Complex Number 13
YBR208C	Complex Number 134
YBR208C	Complex Number 334
YBR208C	Complex Number 42, probably intermediate and energy metabolism
YBR211C	Complex Number 221, probably transcription/DNA maintanance/chromatin structure
YBR211C	SPB associated proteins
YBR216C	Complex Number 162, probably signalling
YBR217W	Complex Number 4
YBR218C	Complex Number 316
YBR218C	Complex Number 435
YBR221C	Complex Number 102, probably protein synthesis turnover
YBR221C	Complex Number 135, probably RNA metabolism
YBR221C	Complex Number 165, probably signalling
YBR221C	Complex Number 42, probably intermediate and energy metabolism
YBR221C	Pyruvate dehydrogenase
YBR223C	Complex Number 431
YBR223C	Complex Number 432
YBR225W	Complex Number 132
YBR225W	Complex Number 163, probably signalling
YBR227C	Complex Number 111
YBR227C	Complex Number 388
YBR228W	Complex Number 505

YBR233W-A	Dam1 protein complex
YBR233W	Complex Number 136, probably RNA metabolism
YBR234C	Actin-associated proteins
YBR234C	Complex Number 13
YBR234C	Complex Number 19, probably cell polarity and structure
YBR234C	Complex Number 9
YBR236C	Complex Number 210, probably transcription/DNA maintanance/chromatin structure
YBR237W	mRNA splicing
YBR238C	Complex Number 164, probably signalling
YBR242W	Complex Number 552
YBR245C	Complex Number 127, probably protein/RNA transport
YBR245C	Complex Number 145
YBR245C	Complex Number 189, probably transcription/DNA maintanance/chromatin structure
YBR245C	Complex Number 192, probably transcription/DNA maintanance/chromatin structure
YBR245C	Complex Number 196, probably transcription/DNA maintanance/chromatin structure
YBR245C	Complex Number 200, probably transcription/DNA maintanance/chromatin structure
YBR245C	Complex Number 203, probably transcription/DNA maintanance/chromatin structure
YBR245C	Complex Number 208, probably transcription/DNA maintanance/chromatin structure
YBR245C	Complex Number 220, probably transcription/DNA maintanance/chromatin structure
YBR246W	Complex Number 98, probably protein synthesis turnover
YBR247C	Complex Number 109, probably protein synthesis turnover
YBR247C	Complex Number 125, probably protein/RNA transport
YBR247C	Complex Number 132
YBR247C	Complex Number 133
YBR247C	Complex Number 144, probably RNA metabolism
YBR247C	Complex Number 146
YBR247C	Complex Number 176, probably signalling
YBR247C	Complex Number 198, probably transcription/DNA maintanance/chromatin structure
YBR247C	Complex Number 2
YBR247C	Complex Number 369
YBR247C	Complex Number 379
YBR247C	Complex Number 82
YBR249C	Complex Number 316
YBR249C	Complex Number 541
YBR251W	Complex Number 104, probably protein synthesis turnover
YBR251W	Complex Number 147, probably RNA metabolism
YBR251W	mitochondrial ribosomal small subunit
YBR252W	Complex Number 388
YBR253W	Complex Number 209, probably transcription/DNA maintanance/chromatin structure
YBR253W	Kornberg's mediator (SRB) complex
YBR254C	Complex Number 75, probably membrane biogenesis and traffic
YBR254C	TRAPP (Transport Protein Particle) complex
YBR257W	Complex Number 154, probably RNA metabolism
YBR257W	Complex Number 78, Rnase P (3)

YBR257W	RNase MRP
YBR257W	RNase P
YBR260C	Complex Number 187
YBR260C	Complex Number 35
YBR263W	Complex Number 86, probably protein synthesis turnover
YBR264C	Complex Number 113
YBR264C	Complex Number 547
YBR265W	Complex Number 29, probably intermediate and energy metabolism
YBR267W	Complex Number 149, probably RNA metabolism
YBR267W	Complex Number 41, Nap1 (3)
YBR267W	Complex Number 433
YBR268W	mitochondrial ribosomal large subunit
YBR272C	Complex Number 204, probably transcription/DNA maintanance/chromatin structure
YBR272C	Complex Number 468
YBR274W	Complex Number 53
YBR274W	Complex Number 54
YBR276C	Complex Number 235
YBR278W	Complex Number 190, probably transcription/DNA maintanance/chromatin structure
YBR278W	DNA polymerase epsilon (II)
YBR278W	Replication complex
YBR279W	Complex Number 201, probably transcription/DNA maintanance/chromatin structure
YBR279W	Complex Number 46, SPT16 (8)
YBR280C	Complex Number 434
YBR280C	Complex Number 47
YBR281C	Complex Number 388
YBR281C	Complex Number 389
YBR281C	Complex Number 390
YBR281C	Complex Number 391
YBR281C	Complex Number 521
YBR282W	Complex Number 108, probably protein synthesis turnover
YBR282W	mitochondrial ribosomal large subunit
YBR286W	Complex Number 340
YBR287W	Complex Number 184, probably transcription/DNA maintanance/chromatin structure
YBR288C	AP-3 complex
YBR288C	Complex Number 7
YBR288C	Complex Number 78, probably membrane biogenesis and traffic
YBR289W	Complex Number 109, probably protein synthesis turnover
YBR289W	Complex Number 198, probably transcription/DNA maintanance/chromatin structure
YBR289W	SWI/SNF transcription activator complex
YBR295W	Complex Number 6, probably cell cycle
YCL009C	Alpha-agglutinin anchor
YCL010C	Complex Number 112
YCL010C	Complex Number 207, probably transcription/DNA maintanance/chromatin structure
YCL010C	Complex Number 214, probably transcription/DNA maintanance/chromatin structure

YCL010C	SAGA complex
YCL011C	Complex Number 106
YCL011C	Complex Number 446
YCL011C	Complex Number 74
YCL014W	Complex Number 108, probably protein synthesis turnover
YCL014W	Complex Number 150
YCL014W	Complex Number 486
YCL017C	Complex Number 144
YCL024W	Complex Number 136, probably RNA metabolism
YCL024W	Complex Number 224, probably transcription/DNA maintanance/chromatin structure
YCL028W	Complex Number 142
YCL028W	Complex Number 157
YCL028W	Complex Number 187
YCL028W	Complex Number 299
YCL028W	Complex Number 301
YCL028W	Complex Number 31
YCL028W	Complex Number 313
YCL028W	Complex Number 322
YCL028W	Complex Number 343
YCL028W	Complex Number 364
YCL028W	Complex Number 378
YCL028W	Complex Number 383
YCL028W	Complex Number 416
YCL028W	Complex Number 432
YCL028W	Complex Number 447
YCL029C	Complex Number 154
YCL029C	SPB associated proteins
YCL029C	Tubulin-associated proteins
YCL030C	Complex Number 472
YCL030C	Complex Number 54, probably intermediate and energy metabolism
YCL031C	Complex Number 144, probably RNA metabolism
YCL031C	Complex Number 48, UTP22/RRP7 (2)
YCL035C	Complex Number 341
YCL037C	Complex Number 125, probably protein/RNA transport
YCL037C	Complex Number 138, probably RNA metabolism
YCL037C	Complex Number 139, probably RNA metabolism
YCL037C	Complex Number 140, probably RNA metabolism
YCL037C	Complex Number 142, probably RNA metabolism
YCL039W	Complex Number 427
YCL039W	Complex Number 437
YCL040W	Actin-associated proteins
YCL042W	Complex Number 300
YCL043C	Complex Number 174
YCL043C	Complex Number 34

YCL043C	Complex Number 344
YCL043C	Complex Number 56
YCL043C	Complex Number 96
YCL046W	Complex Number 148, probably RNA metabolism
YCL050C	Complex Number 165
YCL054W	Complex Number 115
YCL054W	Complex Number 149, probably RNA metabolism
YCL054W	Complex Number 475
YCL054W	Complex Number 82
YCL059C	Complex Number 109, probably protein synthesis turnover
YCL059C	Complex Number 125, probably protein/RNA transport
YCL059C	Complex Number 140, probably RNA metabolism
YCL059C	Complex Number 144, probably RNA metabolism
YCL059C	Complex Number 446
YCL059C	Complex Number 6
YCR001W	Complex Number 438
YCR002C	Complex Number 244
YCR002C	Complex Number 295
YCR002C	Complex Number 33
YCR002C	Complex Number 34
YCR002C	Complex Number 35
YCR002C	Complex Number 42
YCR002C	Complex Number 456
YCR002C	Complex Number 4, probably cell cycle
YCR002C	Septin filaments
YCR003W	mitochondrial ribosomal large subunit
YCR008W	Complex Number 335
YCR009C	Complex Number 326
YCR009C	Complex Number 327
YCR009C	Complex Number 338
YCR012W	Complex Number 110, probably protein synthesis turnover
YCR012W	Complex Number 111, probably protein synthesis turnover
YCR012W	Complex Number 202, probably transcription/DNA maintanance/chromatin structure
YCR012W	Complex Number 209, probably transcription/DNA maintanance/chromatin structure
YCR012W	Complex Number 212, probably transcription/DNA maintanance/chromatin structure
YCR012W	Complex Number 27, probably intermediate and energy metabolism
YCR012W	Complex Number 79, probably membrane biogenesis and traffic
YCR014C	Complex Number 231
YCR014C	DNA polymerase beta (IV)
YCR015C	Complex Number 11, probably cell cycle
YCR018C	rRNA splicing
YCR020C-A	Complex Number 117, probably protein synthesis turnover
YCR024C-A	H+-ATPase, plasma mebrane
YCR028C-A	Complex Number 212, probably transcription/DNA maintanance/chromatin structure

YCR028C-A	Complex Number 230, probably transcription/DNA maintanance/chromatin structure
YCR030C	Complex Number 108, probably protein synthesis turnover
YCR030C	Complex Number 18, probably cell polarity and structure
YCR030C	Complex Number 20, probably cell polarity and structure
YCR030C	Complex Number 534
YCR031C	cytoplasmic ribosomal small subunit
YCR033W	Complex Number 101, probably protein synthesis turnover
YCR033W	Complex Number 170, probably signalling
YCR033W	Complex Number 347
YCR033W	Histone deacetylase complexes
YCR034W	1,3-beta-D-glucan synthase
YCR035C	Complex Number 134, probably RNA metabolism
YCR035C	Complex Number 141, probably RNA metabolism
YCR035C	Complex Number 22, Exosome (12)
YCR035C	Complex Number 376
YCR035C	Exosome complex
YCR042C	Complex Number 214, probably transcription/DNA maintanance/chromatin structure
YCR042C	TAFIIs
YCR046C	Complex Number 108, probably protein synthesis turnover
YCR046C	Complex Number 147, probably RNA metabolism
YCR046C	mitochondrial ribosomal large subunit
YCR046C	Mitochondrial ribosomes
YCR048W	Complex Number 517
YCR052W	Complex Number 192, probably transcription/DNA maintanance/chromatin structure
YCR052W	Complex Number 203, probably transcription/DNA maintanance/chromatin structure
YCR052W	Complex Number 32, RSC (13)
YCR052W	RSC complex (Remodel the structure of chromatin)
YCR053W	Complex Number 244
YCR053W	Complex Number 379
YCR053W	Complex Number 380
YCR053W	Complex Number 42
YCR057C	Complex Number 125, probably protein/RNA transport
YCR057C	Complex Number 144, probably RNA metabolism
YCR057C	Complex Number 255
YCR057C	Complex Number 493
YCR057C	Complex Number 87, UTP B (6)
YCR063W	Complex Number 143, probably RNA metabolism
YCR063W	Complex Number 147, probably RNA metabolism
YCR066W	Complex Number 285
YCR066W	Complex Number 410
YCR071C	Complex Number 108, probably protein synthesis turnover
YCR071C	mitochondrial ribosomal large subunit
YCR072C	Complex Number 149, probably RNA metabolism
YCR073C	Complex Number 274

YCR073C	Complex Number 377
YCR076C	Complex Number 338
YCR077C	Complex Number 138, probably RNA metabolism
YCR077C	Complex Number 147, probably RNA metabolism
YCR077C	Complex Number 14, U6-specific snRNP core (14)
YCR077C	Complex Number 163
YCR077C	Complex Number 164
YCR077C	Complex Number 214
YCR077C	Complex Number 215
YCR079W	Complex Number 439
YCR079W	Complex Number 64, probably intermediate and energy metabolism
YCR081W	Complex Number 209, probably transcription/DNA maintanance/chromatin structure
YCR081W	Srb10p complex
YCR084C	Complex Number 1
YCR084C	Complex Number 108
YCR084C	Complex Number 208, probably transcription/DNA maintanance/chromatin structure
YCR084C	Complex Number 405
YCR084C	Tup1/Ssn6 complex
YCR087W	Complex Number 458
YCR088W	Actin-associated proteins
YCR088W	Complex Number 1
YCR088W	Complex Number 132
YCR088W	Complex Number 239
YCR088W	Complex Number 487
YCR088W	Complex Number 525
YCR088W	Complex Number 526
YCR091W	Complex Number 148
YCR092C	Complex Number 196
YCR092C	Complex Number 212, probably transcription/DNA maintanance/chromatin structure
YCR092C	MSH2/MSH3 complex
YCR093W	CCR4 complex
YCR093W	Complex Number 134
YCR093W	Complex Number 211, probably transcription/DNA maintanance/chromatin structure
YCR093W	Complex Number 31
YCR093W	Complex Number 424
YCR093W	Complex Number 65, CCR4 (3)
YCR093W	NOT complex
YDL002C	Complex Number 203, probably transcription/DNA maintanance/chromatin structure
YDL002C	Complex Number 208, probably transcription/DNA maintanance/chromatin structure
YDL003W	Complex Number 172
YDL003W	Complex Number 46
YDL003W	Complex Number 8, probably cell cycle
YDL003W	Sister chromatid cohesion complex
YDL004W	F0/F1 ATP synthase (complex V)

YDL005C	Complex Number 209, probably transcription/DNA maintanance/chromatin structure
YDL005C	Kornberg's mediator (SRB) complex
YDL006W	Complex Number 10
YDL006W	Complex Number 207, probably transcription/DNA maintanance/chromatin structure
YDL006W	Complex Number 214, probably transcription/DNA maintanance/chromatin structure
YDL006W	Complex Number 216
YDL006W	Complex Number 220
YDL006W	Complex Number 249
YDL006W	Complex Number 356
YDL006W	Complex Number 47
YDL007W	19/22S regulator
YDL007W	Complex Number 110, probably protein synthesis turnover
YDL007W	Complex Number 111, probably protein synthesis turnover
YDL007W	Complex Number 202, probably transcription/DNA maintanance/chromatin structure
YDL007W	Complex Number 204, probably transcription/DNA maintanance/chromatin structure
YDL007W	Complex Number 292
YDL007W	Complex Number 318
YDL007W	Complex Number 320
YDL007W	Complex Number 468
YDL007W	Complex Number 50, 19S Proteosome (17)
YDL007W	Complex Number 532
YDL008W	Anaphase promoting complex (APC)
YDL013W	Complex Number 126
YDL014W	Complex Number 101
YDL014W	Complex Number 109, probably protein synthesis turnover
YDL014W	Complex Number 125, probably protein/RNA transport
YDL014W	Complex Number 135, probably RNA metabolism
YDL014W	Complex Number 138, probably RNA metabolism
YDL014W	Complex Number 140, probably RNA metabolism
YDL014W	Complex Number 144, probably RNA metabolism
YDL014W	Complex Number 149, probably RNA metabolism
YDL014W	Complex Number 15, Nop1 (1)
YDL014W	Complex Number 205
YDL014W	Complex Number 214, probably transcription/DNA maintanance/chromatin structure
YDL014W	Complex Number 375
YDL014W	Complex Number 403
YDL014W	Complex Number 475
YDL014W	Complex Number 493
YDL014W	Complex Number 498
YDL014W	Complex Number 91
YDL014W	Complex Number 99, probably protein synthesis turnover
YDL014W	Nop56p/Nop1p complex
YDL014W	rRNA splicing
YDL017W	Complex Number 49

YDL017W	Replication initiation complex
YDL019C	Complex Number 23, probably intermediate and energy metabolism
YDL020C	26S proteasome
YDL022W	Complex Number 257
YDL025C	Complex Number 440
YDL025C	Complex Number 50, 19S Proteosome (17)
YDL029W	Actin-associated proteins
YDL029W	Arp2p/Arp3p complex
YDL029W	Complex Number 13
YDL029W	Complex Number 187
YDL029W	Complex Number 19, probably cell polarity and structure
YDL029W	Complex Number 207
YDL029W	Complex Number 242
YDL029W	Complex Number 29
YDL029W	Complex Number 292
YDL029W	Complex Number 313
YDL029W	Complex Number 320
YDL029W	Complex Number 332
YDL029W	Complex Number 346
YDL029W	Complex Number 353
YDL029W	Complex Number 360
YDL029W	Complex Number 374
YDL029W	Complex Number 378
YDL029W	Complex Number 383
YDL029W	Complex Number 4
YDL029W	Complex Number 9
YDL030W	Complex Number 145, probably RNA metabolism
YDL030W	Complex Number 146, probably RNA metabolism
YDL030W	Complex Number 147, probably RNA metabolism
YDL030W	Prp9p/Prp11p/Prp21p complex
YDL031W	Complex Number 149, probably RNA metabolism
YDL031W	Complex Number 475
YDL031W	Complex Number 56
YDL031W	Complex Number 82
YDL040C	Complex Number 14, probably cell polarity and structure
YDL040C	Complex Number 193, probably transcription/DNA maintanance/chromatin structure
YDL040C	Complex Number 364
YDL040C	Protein N-acetyltransferase
YDL042C	Complex Number 194, probably transcription/DNA maintanance/chromatin structure
YDL042C	Complex Number 351
YDL042C	Complex Number 352
YDL043C	Complex Number 119, probably protein/RNA transport
YDL043C	Complex Number 145, probably RNA metabolism
YDL043C	Complex Number 146, probably RNA metabolism

YDL043C	Complex Number 147, probably RNA metabolism
YDL043C	Complex Number 240
YDL043C	Prp9p/Prp11p/Prp21p complex
YDL044C	Mitochondrial splicing complexes
YDL045W-A	Complex Number 104, probably protein synthesis turnover
YDL045W-A	mitochondrial ribosomal small subunit
YDL047W	Complex Number 12, Sit4 (6)
YDL047W	Complex Number 132
YDL047W	Complex Number 133
YDL047W	Complex Number 157, probably RNA metabolism
YDL047W	Complex Number 1, probably cell cycle
YDL047W	Complex Number 203
YDL047W	Complex Number 2, probably cell cycle
YDL047W	Complex Number 333
YDL047W	Complex Number 334
YDL047W	Complex Number 353
YDL047W	Complex Number 369
YDL047W	Complex Number 40, probably intermediate and energy metabolism
YDL047W	Complex Number 74
YDL048C	tRNA splicing
YDL051W	Complex Number 140, probably RNA metabolism
YDL051W	Complex Number 142, probably RNA metabolism
YDL051W	Complex Number 149, probably RNA metabolism
YDL051W	Complex Number 155, probably RNA metabolism
YDL051W	Complex Number 22
YDL051W	Complex Number 384
YDL051W	Complex Number 458
YDL051W	Complex Number 507
YDL051W	Complex Number 80, SRP (3)
YDL052C	Complex Number 111
YDL052C	Complex Number 417
YDL052C	Complex Number 504
YDL052C	Complex Number 70, probably membrane biogenesis and traffic
YDL055C	Complex Number 41, probably intermediate and energy metabolism
YDL056W	Complex Number 101
YDL056W	Complex Number 223, probably transcription/DNA maintanance/chromatin structure
YDL056W	MBF complex
YDL058W	Complex Number 197, probably transcription/DNA maintanance/chromatin structure
YDL058W	Complex Number 204, probably transcription/DNA maintanance/chromatin structure
YDL058W	Complex Number 228, probably transcription/DNA maintanance/chromatin structure
YDL059C	Complex Number 283
YDL059C	Complex Number 284
YDL060W	Complex Number 109, probably protein synthesis turnover
YDL060W	Complex Number 115, probably protein synthesis turnover

YDL060W	Complex Number 132
YDL060W	Complex Number 133
YDL060W	Complex Number 144, probably RNA metabolism
YDL060W	Complex Number 176, probably signalling
YDL060W	Complex Number 198, probably transcription/DNA maintanance/chromatin structure
YDL060W	Complex Number 288
YDL060W	Complex Number 441
YDL060W	Complex Number 446
YDL061C	cytoplasmic ribosomal small subunit
YDL063C	Complex Number 146
YDL065C	Complex Number 132
YDL065C	Complex Number 138
YDL065C	Complex Number 214
YDL065C	Complex Number 215
YDL065C	Complex Number 462
YDL065C	Complex Number 501
YDL065C	Complex Number 502
YDL067C	Complex Number 47, probably intermediate and energy metabolism
YDL067C	Cytochrome c oxidase (complex IV)
YDL069C	Mitochondrial processing complexes
YDL069C	Mitochondrial translation complexes
YDL070W	Complex Number 179, probably transcription/DNA maintanance/chromatin structure
YDL070W	Complex Number 204, probably transcription/DNA maintanance/chromatin structure
YDL074C	Complex Number 19
YDL075W	cytoplasmic ribosomal large subunit
YDL076C	Complex Number 208, probably transcription/DNA maintanance/chromatin structure
YDL077C	Complex Number 77, probably membrane biogenesis and traffic
YDL077C	Vacuolar assembly complex
YDL078C	Complex Number 244
YDL080C	Complex Number 49
YDL080C	Complex Number 93, probably protein synthesis turnover
YDL081C	cytoplasmic ribosomal large subunit
YDL082W	cytoplasmic ribosomal large subunit
YDL083C	cytoplasmic ribosomal small subunit
YDL084W	Complex Number 106
YDL084W	Complex Number 129
YDL084W	Complex Number 30, Sub2 (1)
YDL085W	other respiration chain complexes
YDL086W	Complex Number 79
YDL087C	Complex Number 139, probably RNA metabolism
YDL087C	Complex Number 145, probably RNA metabolism
YDL087C	Complex Number 146, probably RNA metabolism
YDL087C	Complex Number 147, probably RNA metabolism
YDL087C	Complex Number 77, probably membrane biogenesis and traffic

YDL090C	Complex Number 168, probably signalling
YDL090C	Farnesyltransferase (FTase)
YDL092W	Signal recognition particle (SRP)
YDL097C	19/22S regulator
YDL097C	Complex Number 110, probably protein synthesis turnover
YDL097C	Complex Number 111, probably protein synthesis turnover
YDL097C	Complex Number 152
YDL097C	Complex Number 163
YDL097C	Complex Number 202, probably transcription/DNA maintanance/chromatin structure
YDL097C	Complex Number 204, probably transcription/DNA maintanance/chromatin structure
YDL097C	Complex Number 221
YDL097C	Complex Number 23
YDL097C	Complex Number 318
YDL097C	Complex Number 320
YDL097C	Complex Number 360
YDL097C	Complex Number 4
YDL097C	Complex Number 468
YDL097C	Complex Number 50, 19S Proteosome (17)
YDL098C	Complex Number 138, probably RNA metabolism
YDL098C	Complex Number 147, probably RNA metabolism
YDL100C	Complex Number 121, probably protein/RNA transport
YDL100C	Complex Number 442
YDL101C	Complex Number 130
YDL101C	Complex Number 131
YDL101C	Complex Number 183
YDL101C	Complex Number 257
YDL101C	Complex Number 265
YDL101C	Complex Number 270
YDL101C	Complex Number 275
YDL101C	Complex Number 280
YDL101C	Complex Number 287
YDL101C	Complex Number 323
YDL101C	Complex Number 364
YDL101C	Complex Number 398
YDL101C	Complex Number 497
YDL101C	Complex Number 90
YDL102W	DNA polymerase delta (III)
YDL102W	Replication complex
YDL108W	Complex Number 206, probably transcription/DNA maintanance/chromatin structure
YDL108W	Complex Number 271
YDL108W	Kin28p complex
YDL108W	NEF3 complex
YDL108W	RNA polymerase I
YDL111C	Complex Number 134, probably RNA metabolism

YDL111C	Complex Number 141, probably RNA metabolism
YDL111C	Complex Number 165
YDL111C	Complex Number 22, Exosome (12)
YDL111C	Exosome complex
YDL112W	Complex Number 169, probably signalling
YDL112W	Complex Number 173
YDL112W	Complex Number 347
YDL112W	Complex Number 84, Trm3 (1)
YDL113C	Complex Number 333
YDL115C	Complex Number 210, probably transcription/DNA maintanance/chromatin structure
YDL116W	Complex Number 124, probably protein/RNA transport
YDL116W	Complex Number 209
YDL116W	Complex Number 210
YDL116W	Complex Number 340
YDL116W	NUP84 complex
YDL117W	Complex Number 17
YDL124W	Complex Number 244
YDL124W	Complex Number 379
YDL126C	Complex Number 151, probably RNA metabolism
YDL126C	Complex Number 19, mRNA cap-binding/eIF4F (8)
YDL126C	Complex Number 7, probably cell cycle
YDL126C	Complex Number 9, probably cell cycle
YDL127W	Pho85p complexes
YDL130W	cytoplasmic ribosomal large subunit
YDL132W	Complex Number 119
YDL132W	Complex Number 134
YDL132W	Complex Number 181
YDL132W	Complex Number 357
YDL132W	Complex Number 434
YDL132W	Complex Number 454
YDL132W	Complex Number 47
YDL132W	Complex Number 494
YDL132W	Complex Number 508
YDL132W	Complex Number 516
YDL132W	Complex Number 519
YDL132W	Complex Number 82
YDL132W	SCF-CDC4 complex
YDL132W	SCF-GRR1 complex
YDL132W	SCF-MET30 complex
YDL133C-A	cytoplasmic ribosomal large subunit
YDL134C	Complex Number 170, probably signalling
YDL134C	Complex Number 232
YDL134C	Complex Number 374
YDL134C	Complex Number 435

YDL134C	Complex Number 48
YDL136W	cytoplasmic ribosomal large subunit
YDL137W	Complex Number 11
YDL137W	Complex Number 316
YDL137W	Complex Number 472
YDL137W	Complex Number 484
YDL140C	Complex Number 207, probably transcription/DNA maintanance/chromatin structure
YDL140C	Complex Number 209, probably transcription/DNA maintanance/chromatin structure
YDL140C	Complex Number 210, probably transcription/DNA maintanance/chromatin structure
YDL140C	Complex Number 214, probably transcription/DNA maintanance/chromatin structure
YDL140C	Complex Number 22, Exosome (12)
YDL140C	Complex Number 24, RNA Polymerase II (12)
YDL140C	Complex Number 322
YDL140C	Complex Number 545
YDL140C	Complex Number 95
YDL140C	RNA polymerase II
YDL143W	Chaperonine containing T-complex TRiC (TCP RING Complex)
YDL143W	Complex Number 105, probably protein synthesis turnover
YDL143W	Complex Number 70, probably membrane biogenesis and traffic
YDL145C	Complex Number 108, probably protein synthesis turnover
YDL145C	Complex Number 211, probably transcription/DNA maintanance/chromatin structure
YDL145C	Complex Number 240
YDL145C	Complex Number 250
YDL145C	Complex Number 338
YDL145C	Complex Number 359
YDL145C	Complex Number 381
YDL145C	Complex Number 469
YDL145C	Complex Number 504
YDL145C	Complex Number 535
YDL145C	Complex Number 69
YDL145C	Complex Number 79, probably membrane biogenesis and traffic
YDL145C	Complex Number 87
YDL145C	Complex Number 99
YDL145C	COPI
YDL147W	19/22S regulator
YDL147W	Complex Number 110, probably protein synthesis turnover
YDL147W	Complex Number 111, probably protein synthesis turnover
YDL147W	Complex Number 174
YDL147W	Complex Number 23
YDL147W	Complex Number 318
YDL147W	Complex Number 320
YDL147W	Complex Number 4
YDL147W	Complex Number 468
YDL147W	Complex Number 50, 19S Proteosome (17)

YDL147W	Complex Number 77
YDL147W	Complex Number 87, probably protein synthesis turnover
YDL148C	Complex Number 109, probably protein synthesis turnover
YDL148C	Complex Number 125, probably protein/RNA transport
YDL148C	Complex Number 144, probably RNA metabolism
YDL148C	Complex Number 84
YDL148C	Nuclear pore complex (NPC)
YDL149W	Complex Number 107, probably protein synthesis turnover
YDL150W	Complex Number 213, probably transcription/DNA maintanance/chromatin structure
YDL150W	Complex Number 23, RNA Polymerase III (12)
YDL153C	Complex Number 132
YDL155W	Cdc28p complexes
YDL155W	Complex Number 58
YDL155W	Complex Number 6, probably cell cycle
YDL156W	Complex Number 212, probably transcription/DNA maintanance/chromatin structure
YDL156W	Complex Number 443
YDL159W	Complex Number 152
YDL159W	Complex Number 153
YDL159W	STE5-MAPK complex
YDL160C	CCR4 complex
YDL160C	Complex Number 138, probably RNA metabolism
YDL160C	Complex Number 147, probably RNA metabolism
YDL160C	Complex Number 163
YDL160C	Complex Number 213, probably transcription/DNA maintanance/chromatin structure
YDL160C	Complex Number 214
YDL160C	Complex Number 215
YDL160C	Complex Number 338
YDL160C	Complex Number 505
YDL160C	Complex Number 54, probably intermediate and energy metabolism
YDL160C	Complex Number 70, probably membrane biogenesis and traffic
YDL164C	Complex Number 50
YDL164C	Complex Number 51
YDL164C	DNA ligases
YDL165W	CCR4 complex
YDL165W	Complex Number 211, probably transcription/DNA maintanance/chromatin structure
YDL165W	Complex Number 31
YDL165W	NOT complex
YDL166C	Complex Number 144, probably RNA metabolism
YDL167C	Complex Number 155, probably RNA metabolism
YDL171C	Complex Number 107, probably protein synthesis turnover
YDL171C	Complex Number 10, probably cell cycle
YDL171C	Complex Number 353
YDL171C	Complex Number 371
YDL171C	Complex Number 85, probably protein synthesis turnover

YDL174C	other respiration chain complexes
YDL175C	Complex Number 139, probably RNA metabolism
YDL175C	Complex Number 404
YDL175C	Complex Number 444
YDL175C	Complex Number 9, Mtr4 (3)
YDL178W	Actin-associated proteins
YDL179W	Complex Number 218
YDL181W	F0/F1 ATP synthase (complex V)
YDL184C	cytoplasmic ribosomal large subunit
YDL185W1	Complex Number 124
YDL185W1	Complex Number 149
YDL185W1	Complex Number 206
YDL185W1	Complex Number 240
YDL185W1	Complex Number 258
YDL185W1	Complex Number 267
YDL185W1	Complex Number 271
YDL185W1	Complex Number 272
YDL185W1	Complex Number 275
YDL185W1	Complex Number 316
YDL185W1	Complex Number 347
YDL185W1	Complex Number 349
YDL185W1	Complex Number 38
YDL185W1	Complex Number 437
YDL185W1	Complex Number 493
YDL185W1	Complex Number 511
YDL185W1	Complex Number 540
YDL185W1	Complex Number 77
YDL185W1	Complex Number 92
YDL185W1	H+-transporting ATPase, vacuolar
YDL185W	Complex Number 10, probably cell cycle
YDL185W	Complex Number 173, probably signalling
YDL185W	Complex Number 18, probably cell polarity and structure
YDL185W	Complex Number 211, probably transcription/DNA maintanance/chromatin structure
YDL185W	Complex Number 231, probably transcription/DNA maintanance/chromatin structure
YDL185W	Complex Number 35, probably intermediate and energy metabolism
YDL185W	Complex Number 45, probably intermediate and energy metabolism
YDL185W	H+-transporting ATPase, vacuolar
YDL188C	Complex Number 170, probably signalling
YDL188C	Complex Number 232
YDL188C	Complex Number 233
YDL188C	Complex Number 374
YDL188C	Complex Number 414
YDL188C	Complex Number 435
YDL188C	Complex Number 48

YDL190C	Complex Number 413
YDL190C	Complex Number 543
YDL191W	cytoplasmic ribosomal large subunit
YDL192W	Complex Number 11
YDL192W	Complex Number 182
YDL192W	Complex Number 316
YDL192W	Complex Number 341
YDL192W	Complex Number 367
YDL192W	Complex Number 484
YDL192W	Complex Number 73
YDL192W	COPI
YDL193W	Complex Number 445
YDL195W	Complex Number 124, probably protein/RNA transport
YDL195W	Complex Number 148, probably RNA metabolism
YDL195W	Complex Number 337
YDL195W	Complex Number 339
YDL195W	COPII
YDL200C	Complex Number 182
YDL200C	Complex Number 183
YDL202W	mitochondrial ribosomal large subunit
YDL203C	Complex Number 55, probably intermediate and energy metabolism
YDL204W	Complex Number 146
YDL204W	Complex Number 338
YDL208W	Complex Number 140, probably RNA metabolism
YDL208W	Complex Number 200
YDL208W	Complex Number 5, Gar1/Cbf5 (6)
YDL209C	Complex Number 143, probably RNA metabolism
YDL209C	Complex Number 145, probably RNA metabolism
YDL209C	Complex Number 146, probably RNA metabolism
YDL209C	Complex Number 147, probably RNA metabolism
YDL209C	mRNA splicing
YDL213C	Complex Number 125, probably protein/RNA transport
YDL213C	Complex Number 140, probably RNA metabolism
YDL213C	Complex Number 144, probably RNA metabolism
YDL213C	Complex Number 336
YDL213C	Complex Number 376
YDL213C	Complex Number 446
YDL213C	Complex Number 5, Gar1/Cbf5 (6)
YDL215C	Complex Number 515
YDL216C	Complex Number 87, probably protein synthesis turnover
YDL217C	Tim22p-complex
YDL219W	Complex Number 2, probably cell cycle
YDL220C	Complex Number 279
YDL220C	Complex Number 36

YDL225W	Complex Number 212, probably transcription/DNA maintanance/chromatin structure
YDL225W	Complex Number 33
YDL225W	Complex Number 346
YDL225W	Complex Number 42
YDL225W	Complex Number 437
YDL225W	Complex Number 462
YDL225W	Complex Number 4, probably cell cycle
YDL225W	Septin filaments
YDL232W	Oligosaccharyltransferase
YDL235C	Complex Number 552
YDR001C	Complex Number 161, probably signalling
YDR001C	Complex Number 17
YDR002W	Complex Number 120
YDR002W	Complex Number 146
YDR002W	Complex Number 312
YDR004W	other DNA repair complexes
YDR005C	Complex Number 213, probably transcription/DNA maintanance/chromatin structure
YDR006C	Complex Number 17
YDR009W	Complex Number 134
YDR009W	GAL80 complex
YDR012W	cytoplasmic ribosomal large subunit
YDR013W	0
YDR016C	Dam1 protein complex
YDR017C	Complex Number 17
YDR019C	Glycine decarboxylase
YDR021W	Complex Number 112, probably protein synthesis turnover
YDR021W	Complex Number 380
YDR022C	Complex Number 123
YDR023W	Complex Number 115, probably protein synthesis turnover
YDR023W	Complex Number 132
YDR023W	Complex Number 327
YDR023W	Complex Number 341
YDR023W	Complex Number 350
YDR023W	Complex Number 426
YDR023W	Complex Number 444
YDR023W	Complex Number 535
YDR025W	cytoplasmic ribosomal small subunit
YDR027C	Complex Number 74, probably membrane biogenesis and traffic
YDR028C	Complex Number 115
YDR028C	Complex Number 166, probably signalling
YDR028C	Complex Number 17
YDR028C	Complex Number 29, probably intermediate and energy metabolism
YDR028C	Complex Number 336
YDR028C	Complex Number 9, probably cell cycle

YDR028C	Serine/threonine phoshpoprotein phosphatase
YDR030C	Complex Number 269
YDR030C	Complex Number 270
YDR032C	Complex Number 13
YDR032C	Complex Number 368
YDR032C	Complex Number 388
YDR032C	Complex Number 422
YDR032C	Complex Number 435
YDR032C	Complex Number 49
YDR032C	Complex Number 504
YDR032C	Complex Number 62
YDR036C	Complex Number 104, probably protein synthesis turnover
YDR036C	Complex Number 22
YDR036C	Complex Number 507
YDR037W	Complex Number 103
YDR037W	Complex Number 138, probably RNA metabolism
YDR037W	Complex Number 151, probably RNA metabolism
YDR037W	Complex Number 244
YDR037W	Complex Number 327
YDR041W	Complex Number 104, probably protein synthesis turnover
YDR041W	mitochondrial ribosomal small subunit
YDR044W	Complex Number 207, probably transcription/DNA maintanance/chromatin structure
YDR044W	Complex Number 214, probably transcription/DNA maintanance/chromatin structure
YDR045C	Complex Number 213, probably transcription/DNA maintanance/chromatin structure
YDR049W	Complex Number 413
YDR049W	Complex Number 9, probably cell cycle
YDR050C	Complex Number 34, probably intermediate and energy metabolism
YDR052C	Replication initiation complex
YDR054C	SCF-CDC4 complex
YDR054C	SCF-GRR1 complex
YDR054C	SCF-MET30 complex
YDR060W	Complex Number 11, Noc (3)
YDR060W	Complex Number 140, probably RNA metabolism
YDR060W	Complex Number 149, probably RNA metabolism
YDR060W	Complex Number 7, Dbp7/Rrp5 (4)
YDR062W	Complex Number 123, probably protein/RNA transport
YDR064W	cytoplasmic ribosomal small subunit
YDR069C	26S proteasome
YDR069C	Complex Number 199, probably transcription/DNA maintanance/chromatin structure
YDR069C	Complex Number 42, probably intermediate and energy metabolism
YDR069C	Complex Number 97, probably protein synthesis turnover
YDR071C	Complex Number 146
YDR071C	Complex Number 176, probably signalling
YDR071C	Complex Number 177, probably signalling

YDR071C	Complex Number 250
YDR071C	Complex Number 251
YDR071C	Complex Number 278
YDR071C	Complex Number 451
YDR073W	SWI/SNF transcription activator complex
YDR074W	Complex Number 29, probably intermediate and energy metabolism
YDR074W	Complex Number 485
YDR075W	Complex Number 169, probably signalling
YDR075W	Complex Number 234
YDR076W	Complex Number 282
YDR076W	other DNA repair complexes
YDR080W	Complex Number 290
YDR080W	Complex Number 77, probably membrane biogenesis and traffic
YDR080W	Vacuolar assembly complex
YDR083W	Complex Number 29, Rrp8 (1)
YDR083W	Complex Number 53, Kap95/Srp1 (2)
YDR085C	Complex Number 341
YDR086C	Sec61 complex
YDR087C	Complex Number 101
YDR087C	Complex Number 149, probably RNA metabolism
YDR087C	Complex Number 205
YDR087C	Complex Number 475
YDR087C	Complex Number 69, probably membrane biogenesis and traffic
YDR087C	Complex Number 96
YDR088C	Complex Number 147, probably RNA metabolism
YDR088C	mRNA splicing
YDR091C	Complex Number 107, probably protein synthesis turnover
YDR091C	Complex Number 375
YDR091C	Complex Number 446
YDR091C	Complex Number 51, eIF3 (7)
YDR091C	Complex Number 54, Rli1/Rpp0 (3)
YDR092W	Complex Number 190
YDR092W	Complex Number 191
YDR092W	Complex Number 409
YDR092W	Complex Number 410
YDR097C	Complex Number 197
YDR097C	Complex Number 202, probably transcription/DNA maintanance/chromatin structure
YDR097C	Complex Number 212, probably transcription/DNA maintanance/chromatin structure
YDR097C	MSH2/MSH6 complex
YDR098C	Complex Number 23
YDR098C	Complex Number 471
YDR099W	Complex Number 123, probably protein/RNA transport
YDR099W	Complex Number 161, probably signalling
YDR099W	Complex Number 18

YDR101C	Complex Number 116, probably protein synthesis turnover
YDR101C	Complex Number 149, probably RNA metabolism
YDR101C	Complex Number 17, probably cell polarity and structure
YDR101C	Complex Number 22
YDR101C	Complex Number 38, Arx1 Complex (5)
YDR101C	Complex Number 507
YDR101C	Complex Number 69, probably membrane biogenesis and traffic
YDR101C	Complex Number 86, probably protein synthesis turnover
YDR101C	Complex Number 89
YDR102C	Complex Number 458
YDR103W	STE5-MAPK complex
YDR106W	Complex Number 511
YDR108W	Complex Number 75, probably membrane biogenesis and traffic
YDR108W	TRAPP (Transport Protein Particle) complex
YDR111C	Complex Number 96, probably protein synthesis turnover
YDR113C	Complex Number 219
YDR113C	Sister chromatid separation complex
YDR115W	Complex Number 108, probably protein synthesis turnover
YDR115W	mitochondrial ribosomal large subunit
YDR116C	Complex Number 108, probably protein synthesis turnover
YDR116C	Complex Number 56
YDR116C	mitochondrial ribosomal large subunit
YDR117C	Complex Number 15, Nop1 (1)
YDR117C	Complex Number 16, Ded1 (1)
YDR117C	Complex Number 18, Ydr117c/Rps4b (2)
YDR118W	Anaphase promoting complex (APC)
YDR120C	Complex Number 83, Trm1/Nsa1 (2)
YDR121W	Complex Number 190, probably transcription/DNA maintanance/chromatin structure
YDR121W	Complex Number 220, probably transcription/DNA maintanance/chromatin structure
YDR122W	Complex Number 523
YDR127W	Complex Number 151
YDR127W	Complex Number 195, probably transcription/DNA maintanance/chromatin structure
YDR127W	Complex Number 296
YDR127W	Complex Number 383
YDR127W	Complex Number 4
YDR127W	Complex Number 467
YDR127W	Complex Number 504
YDR127W	Complex Number 69, probably membrane biogenesis and traffic
YDR128W	Complex Number 296
YDR128W	Complex Number 447
YDR128W	Complex Number 504
YDR129C	Actin-associated proteins
YDR129C	Complex Number 103
YDR129C	Complex Number 244

YDR129C	Complex Number 330
YDR129C	Complex Number 97
YDR130C	Complex Number 115
YDR131C	Complex Number 357
YDR131C	Complex Number 448
YDR131C	Complex Number 486
YDR131C	Complex Number 545
YDR137W	Complex Number 549
YDR138W	Complex Number 106
YDR138W	Complex Number 128
YDR138W	Complex Number 129
YDR138W	Complex Number 30, Sub2 (1)
YDR138W	Complex Number 31, TREX (5)
YDR138W	Complex Number 376
YDR138W	Complex Number 429
YDR138W	HPR1 complex
YDR139C	Complex Number 181
YDR140W	Complex Number 36, probably intermediate and energy metabolism
YDR141C	Complex Number 15
YDR141C	Complex Number 316
YDR142C	Complex Number 221
YDR143C	Complex Number 301
YDR143C	Complex Number 332
YDR143C	Complex Number 45
YDR145W	Complex Number 207, probably transcription/DNA maintanance/chromatin structure
YDR145W	Complex Number 214, probably transcription/DNA maintanance/chromatin structure
YDR145W	SAGA complex
YDR145W	TAFIIs
YDR146C	Complex Number 382
YDR148C	2-oxoglutarate dehydrogenase
YDR148C	Complex Number 327
YDR148C	Complex Number 346
YDR148C	Complex Number 39, probably intermediate and energy metabolism
YDR150W	Complex Number 23, probably intermediate and energy metabolism
YDR150W	Complex Number 451
YDR152W	Complex Number 476
YDR152W	Complex Number 58, Gir Complex (2)
YDR155C	Complex Number 101, probably protein synthesis turnover
YDR155C	Complex Number 119
YDR155C	Complex Number 135
YDR155C	Complex Number 136
YDR155C	Complex Number 143
YDR155C	Complex Number 154
YDR155C	Complex Number 183

YDR155C	Complex Number 186
YDR155C	Complex Number 22
YDR155C	Complex Number 258
YDR155C	Complex Number 341
YDR155C	Complex Number 36
YDR155C	Complex Number 366
YDR155C	Complex Number 379
YDR155C	Complex Number 388
YDR155C	Complex Number 491
YDR155C	Complex Number 507
YDR155C	Complex Number 525
YDR155C	Complex Number 529
YDR155C	Complex Number 62
YDR155C	Complex Number 90
YDR156W	RNA polymerase I
YDR158W	Complex Number 244
YDR158W	Complex Number 42
YDR158W	Complex Number 90
YDR162C	Complex Number 216
YDR163W	mRNA splicing
YDR164C	Complex Number 108, probably protein synthesis turnover
YDR164C	Complex Number 65, probably membrane biogenesis and traffic
YDR166C	Complex Number 81, probably membrane biogenesis and traffic
YDR166C	Exocyst complex
YDR167W	Complex Number 207, probably transcription/DNA maintanance/chromatin structure
YDR167W	Complex Number 214, probably transcription/DNA maintanance/chromatin structure
YDR167W	SAGA complex
YDR167W	TAFIIs
YDR168W	Complex Number 74
YDR169C	Complex Number 75
YDR170C	Complex Number 149, probably RNA metabolism
YDR170C	Complex Number 220
YDR170C	Complex Number 291
YDR170C	Complex Number 367
YDR170C	Complex Number 412
YDR170C	Complex Number 70, probably membrane biogenesis and traffic
YDR170C	other coat complexes
YDR170W-A	Complex Number 308
YDR170W-A	Complex Number 58
YDR171W	Complex Number 10, probably cell cycle
YDR171W	Complex Number 119, probably protein/RNA transport
YDR171W	Complex Number 132, probably RNA metabolism
YDR171W	Complex Number 194, probably transcription/DNA maintanance/chromatin structure
YDR171W	Complex Number 42, probably intermediate and energy metabolism

YDR171W	Complex Number 89, probably protein synthesis turnover
YDR172W	Complex Number 197, probably transcription/DNA maintanance/chromatin structure
YDR172W	Complex Number 82, Translation release factor (2)
YDR172W	eRF3
YDR173C	ARG complex
YDR174W	Complex Number 100
YDR174W	Complex Number 136, probably RNA metabolism
YDR174W	Complex Number 22
YDR174W	Complex Number 446
YDR174W	Complex Number 507
YDR175C	Complex Number 104, probably protein synthesis turnover
YDR175C	Complex Number 22
YDR175C	Complex Number 507
YDR175C	mitochondrial ribosomal small subunit
YDR176W	ADA complex
YDR176W	Complex Number 207, probably transcription/DNA maintanance/chromatin structure
YDR176W	Complex Number 214, probably transcription/DNA maintanance/chromatin structure
YDR176W	SAGA complex
YDR177W	Complex Number 407
YDR178W	Succinate dehydrogenase complex (complex II)
YDR179C	Complex Number 87, probably protein synthesis turnover
YDR179C	Signal transduction complexes
YDR180W	Sister chromatid cohesion complex
YDR186C	Complex Number 177, probably signalling
YDR188W	Chaperonine containing T-complex TRiC (TCP RING Complex)
YDR188W	Complex Number 150
YDR188W	Complex Number 211, probably transcription/DNA maintanance/chromatin structure
YDR188W	Complex Number 221
YDR188W	Complex Number 223
YDR188W	Complex Number 243
YDR188W	Complex Number 254
YDR188W	Complex Number 26
YDR188W	Complex Number 269
YDR188W	Complex Number 338
YDR188W	Complex Number 353
YDR188W	Complex Number 370
YDR188W	Complex Number 378
YDR188W	Complex Number 420
YDR188W	Complex Number 421
YDR188W	Complex Number 447
YDR188W	Complex Number 48
YDR188W	Complex Number 520
YDR188W	Complex Number 92, probably protein synthesis turnover
YDR188W	Complex Number 94

YDR189W	Complex Number 71, probably membrane biogenesis and traffic
YDR190C	Complex Number 105
YDR190C	Complex Number 13
YDR190C	Complex Number 150
YDR190C	Complex Number 152
YDR190C	Complex Number 153
YDR190C	Complex Number 187
YDR190C	Complex Number 233
YDR190C	Complex Number 292
YDR190C	Complex Number 31
YDR190C	Complex Number 325
YDR190C	Complex Number 336
YDR190C	Complex Number 388
YDR190C	Complex Number 412
YDR190C	Complex Number 421
YDR190C	Complex Number 501
YDR190C	Complex Number 532
YDR190C	Complex Number 75
YDR192C	Nuclear pore complex (NPC)
YDR194C	Complex Number 185
YDR194C	Complex Number 204
YDR194C	Complex Number 446
YDR194C	Complex Number 458
YDR194C	Complex Number 480
YDR194C	Complex Number 91
YDR194C	Complex Number 96
YDR194C	Mitochondrial splicing complexes
YDR195W	Complex Number 125, probably protein/RNA transport
YDR195W	Complex Number 148, probably RNA metabolism
YDR195W	Complex Number 56, mRNA cleavage/polyadenylation (10)
YDR196C	Complex Number 185, probably transcription/DNA maintanance/chromatin structure
YDR197W	Mitochondrial processing complexes
YDR197W	Mitochondrial translation complexes
YDR198C	Complex Number 162
YDR200C	Complex Number 449
YDR201W	Dam1 protein complex
YDR201W	SPB associated proteins
YDR207C	Complex Number 208, probably transcription/DNA maintanance/chromatin structure
YDR207C	Ume6/Ime1 complex
YDR211W	Complex Number 105, probably protein synthesis turnover
YDR211W	Complex Number 108
YDR211W	Complex Number 109
YDR211W	Complex Number 111
YDR211W	Complex Number 14, probably cell polarity and structure

YDR211W	Complex Number 380
YDR211W	Complex Number 52, tIF2 (7)
YDR211W	Complex Number 88, probably protein synthesis turnover
YDR211W	eIF2B
YDR212W	Chaperonine containing T-complex TRiC (TCP RING Complex)
YDR212W	Complex Number 140
YDR212W	Complex Number 158
YDR212W	Complex Number 223
YDR212W	Complex Number 234
YDR212W	Complex Number 254
YDR212W	Complex Number 26
YDR212W	Complex Number 265
YDR212W	Complex Number 270
YDR212W	Complex Number 338
YDR212W	Complex Number 348
YDR212W	Complex Number 370
YDR212W	Complex Number 378
YDR212W	Complex Number 385
YDR212W	Complex Number 39
YDR212W	Complex Number 400
YDR212W	Complex Number 41
YDR212W	Complex Number 420
YDR212W	Complex Number 48
YDR212W	Complex Number 497
YDR214W	Complex Number 129
YDR214W	Complex Number 182
YDR214W	Complex Number 207
YDR214W	Complex Number 211, probably transcription/DNA maintanance/chromatin structure
YDR214W	Complex Number 264
YDR214W	Complex Number 284
YDR214W	Complex Number 302
YDR214W	Complex Number 316
YDR214W	Complex Number 360
YDR214W	Complex Number 374
YDR214W	Complex Number 378
YDR214W	Complex Number 4
YDR214W	Complex Number 43
YDR214W	Complex Number 90
YDR216W	Complex Number 13
YDR216W	Complex Number 134
YDR216W	Complex Number 146
YDR216W	Complex Number 17
YDR216W	Complex Number 316
YDR216W	Complex Number 504

YDR216W	Complex Number 69
YDR217C	Complex Number 287
YDR218C	Septin filaments
YDR219C	Complex Number 450
YDR224C	Complex Number 101
YDR224C	Complex Number 137
YDR224C	Complex Number 141
YDR224C	Complex Number 150
YDR224C	Complex Number 154
YDR224C	Complex Number 182
YDR224C	Complex Number 183
YDR224C	Complex Number 196
YDR224C	Complex Number 22
YDR224C	Complex Number 294
YDR224C	Complex Number 313
YDR224C	Complex Number 386
YDR224C	Complex Number 441
YDR224C	Complex Number 446
YDR224C	Complex Number 458
YDR224C	Complex Number 507
YDR224C	Complex Number 534
YDR224C	Nucleosomal protein complex
YDR225W	Complex Number 100
YDR225W	Complex Number 137
YDR225W	Complex Number 139, probably RNA metabolism
YDR225W	Complex Number 147, probably RNA metabolism
YDR225W	Complex Number 148, probably RNA metabolism
YDR225W	Complex Number 161
YDR225W	Complex Number 22
YDR225W	Complex Number 507
YDR225W	Complex Number 513
YDR225W	Complex Number 534
YDR225W	Complex Number 56
YDR225W	Nucleosomal protein complex
YDR226W	Complex Number 224
YDR226W	Complex Number 240
YDR226W	Complex Number 37
YDR226W	Complex Number 388
YDR226W	Complex Number 407
YDR226W	Complex Number 426
YDR227W	Complex Number 194, probably transcription/DNA maintanance/chromatin structure
YDR227W	Complex Number 351
YDR227W	Complex Number 352
YDR228C	Complex Number 148, probably RNA metabolism

YDR228C	pre mRNA3'-end processing factor CFI
YDR229W	Complex Number 46
YDR233C	Complex Number 125
YDR235W	Complex Number 139, probably RNA metabolism
YDR235W	Complex Number 145, probably RNA metabolism
YDR235W	Complex Number 146, probably RNA metabolism
YDR235W	Complex Number 147, probably RNA metabolism
YDR235W	Complex Number 35, U1 snRNP (7)
YDR237W	Complex Number 108, probably protein synthesis turnover
YDR237W	mitochondrial ribosomal large subunit
YDR238C	Complex Number 108, probably protein synthesis turnover
YDR238C	Complex Number 164
YDR238C	Complex Number 338
YDR238C	Complex Number 69
YDR238C	Complex Number 79
YDR238C	Complex Number 79, probably membrane biogenesis and traffic
YDR238C	COPI
YDR239C	Complex Number 152
YDR240C	Complex Number 139, probably RNA metabolism
YDR240C	Complex Number 145, probably RNA metabolism
YDR240C	Complex Number 146, probably RNA metabolism
YDR240C	Complex Number 147, probably RNA metabolism
YDR240C	Complex Number 35, U1 snRNP (7)
YDR240C	Complex Number 376
YDR240C	Complex Number 53, Kap95/Srp1 (2)
YDR243C	Complex Number 147, probably RNA metabolism
YDR243C	Complex Number 383
YDR243C	Complex Number 70
YDR243C	mRNA splicing
YDR245W	Complex Number 72, probably membrane biogenesis and traffic
YDR246W	Complex Number 75, probably membrane biogenesis and traffic
YDR246W	TRAPP (Transport Protein Particle) complex
YDR247W	Complex Number 251
YDR247W	Complex Number 451
YDR253C	Met4/Met28/Met32 complex
YDR254W	Chl4 protein complex
YDR254W	Complex Number 221, probably transcription/DNA maintanance/chromatin structure
YDR255C	Complex Number 427
YDR255C	Complex Number 437
YDR256C	Catalase A
YDR260C	Complex Number 383
YDR260C	Complex Number 40
YDR266C	Complex Number 452
YDR267C	Complex Number 453

YDR279W	Complex Number 142
YDR279W	Complex Number 23
YDR280W	Complex Number 134, probably RNA metabolism
YDR280W	Complex Number 141, probably RNA metabolism
YDR280W	Complex Number 22, Exosome (12)
YDR280W	Complex Number 25, RNA polymerase I (7)
YDR280W	Exosome complex
YDR283C	Complex Number 110
YDR283C	Complex Number 147, probably RNA metabolism
YDR285W	Synaptonemal complex (SC)
YDR289C	Complex Number 376
YDR292C	Complex Number 90, probably protein synthesis turnover
YDR292C	Signal recognition particle receptor (SR)
YDR293C	Complex Number 12, Sit4 (6)
YDR293C	Complex Number 150
YDR293C	Complex Number 29
YDR293C	Complex Number 389
YDR293C	Complex Number 59, Ssd1 (1)
YDR295C	Complex Number 212, probably transcription/DNA maintanance/chromatin structure
YDR295C	Complex Number 218, probably transcription/DNA maintanance/chromatin structure
YDR296W	Complex Number 108, probably protein synthesis turnover
YDR298C	Complex Number 37
YDR298C	F0/F1 ATP synthase (complex V)
YDR299W	Complex Number 125, probably protein/RNA transport
YDR299W	Complex Number 144, probably RNA metabolism
YDR299W	Complex Number 49
YDR300C	Complex Number 105, probably protein synthesis turnover
YDR300C	Complex Number 204, probably transcription/DNA maintanance/chromatin structure
YDR300C	Complex Number 258
YDR300C	Complex Number 321
YDR300C	Complex Number 63, probably intermediate and energy metabolism
YDR301W	Complex Number 115
YDR301W	Complex Number 148, probably RNA metabolism
YDR301W	Complex Number 223
YDR301W	Complex Number 56, mRNA cleavage/polyadenylation (10)
YDR301W	pre mRNA3'-end processing factor CFII
YDR303C	Complex Number 200, probably transcription/DNA maintanance/chromatin structure
YDR303C	Complex Number 204, probably transcription/DNA maintanance/chromatin structure
YDR303C	Complex Number 32, RSC (13)
YDR306C	Complex Number 454
YDR308C	Complex Number 209, probably transcription/DNA maintanance/chromatin structure
YDR308C	Kornberg's mediator (SRB) complex
YDR310C	Complex Number 372
YDR311W	Complex Number 202, probably transcription/DNA maintanance/chromatin structure

YDR311W	Complex Number 206, probably transcription/DNA maintanance/chromatin structure
YDR311W	NEF3 complex
YDR311W	RNA polymerase I
YDR312W	Complex Number 82
YDR313C	Complex Number 226
YDR314C	Complex Number 202, probably transcription/DNA maintanance/chromatin structure
YDR316W	Complex Number 455
YDR318W	Complex Number 221, probably transcription/DNA maintanance/chromatin structure
YDR318W	Ctf19 protein complex
YDR320C-A	Dam1 protein complex
YDR322C-A	F0/F1 ATP synthase (complex V)
YDR322W	Complex Number 108, probably protein synthesis turnover
YDR322W	Complex Number 147, probably RNA metabolism
YDR322W	mitochondrial ribosomal large subunit
YDR324C	Complex Number 125, probably protein/RNA transport
YDR324C	Complex Number 141
YDR324C	Complex Number 144, probably RNA metabolism
YDR324C	Complex Number 257
YDR324C	Complex Number 43, UTP A (8)
YDR324C	Complex Number 456
YDR324C	rRNA splicing
YDR326C	Complex Number 267
YDR328C	CBF3 protein complex
YDR328C	Complex Number 119
YDR328C	Complex Number 181
YDR328C	Complex Number 357
YDR328C	Complex Number 430
YDR328C	Complex Number 44
YDR328C	Complex Number 448
YDR328C	Complex Number 450
YDR328C	Complex Number 454
YDR328C	Complex Number 47
YDR328C	Complex Number 494
YDR328C	Complex Number 508
YDR328C	Complex Number 516
YDR328C	Complex Number 519
YDR328C	Complex Number 532
YDR328C	Complex Number 73
YDR328C	Complex Number 82
YDR328C	SCF-CDC4 complex
YDR328C	SCF-GRR1 complex
YDR328C	SCF-MET30 complex
YDR334W	Complex Number 14, U6-specific snRNP core (14)
YDR335W	Complex Number 199

YDR337W	Complex Number 104, probably protein synthesis turnover
YDR337W	Complex Number 124, probably protein/RNA transport
YDR337W	Complex Number 202, probably transcription/DNA maintanance/chromatin structure
YDR337W	Complex Number 206, probably transcription/DNA maintanance/chromatin structure
YDR337W	Complex Number 9, probably cell cycle
YDR337W	mitochondrial ribosomal small subunit
YDR339C	Complex Number 457
YDR341C	Complex Number 103
YDR341C	Complex Number 143
YDR342C	Complex Number 1
YDR342C	Complex Number 111
YDR342C	Complex Number 132
YDR342C	Complex Number 156
YDR342C	Complex Number 158
YDR342C	Complex Number 228
YDR342C	Complex Number 248
YDR342C	Complex Number 296
YDR342C	Complex Number 319
YDR342C	Complex Number 426
YDR342C	Complex Number 437
YDR342C	Complex Number 520
YDR342C	Complex Number 94
YDR343C	Complex Number 152
YDR343C	Complex Number 155
YDR343C	Complex Number 228
YDR343C	Complex Number 248
YDR343C	Complex Number 272
YDR343C	Complex Number 322
YDR343C	Complex Number 336
YDR343C	Complex Number 354
YDR343C	Complex Number 426
YDR343C	Complex Number 95
YDR347W	Complex Number 104, probably protein synthesis turnover
YDR347W	mitochondrial ribosomal small subunit
YDR348C	Complex Number 20, probably cell polarity and structure
YDR350C	Complex Number 115, probably protein synthesis turnover
YDR353W	Complex Number 244
YDR353W	Complex Number 307
YDR353W	Complex Number 330
YDR353W	Complex Number 379
YDR353W	Complex Number 536
YDR353W	Complex Number 71
YDR356W	Complex Number 62
YDR356W	Complex Number 7, probably cell cycle

YDR356W	SPB components
YDR359C	Complex Number 127
YDR359C	Complex Number 191, probably transcription/DNA maintanance/chromatin structure
YDR359C	Complex Number 334
YDR361C	Complex Number 463
YDR361C	Complex Number 64, Bcp1/Rpl23A (3)
YDR361C	Complex Number 85, probably protein synthesis turnover
YDR362C	Complex Number 178, probably transcription/DNA maintanance/chromatin structure
YDR362C	TFIIIC
YDR364C	Complex Number 143, probably RNA metabolism
YDR364C	Complex Number 27, Cbc2/Sto1 (2)
YDR364C	Complex Number 55, Prp19 (4)
YDR364C	mRNA splicing
YDR365C	Complex Number 125, probably protein/RNA transport
YDR365C	Complex Number 144, probably RNA metabolism
YDR365C	Complex Number 42, Ydr365c (1)
YDR365C	Complex Number 458
YDR365C	Complex Number 58, Gir Complex (2)
YDR369C	Complex Number 274
YDR369C	Complex Number 275
YDR369C	Complex Number 422
YDR369C	Complex Number 423
YDR369C	MRE11/RAD50/XRS2 complex
YDR372C	Complex Number 374
YDR377W	F0/F1 ATP synthase (complex V)
YDR378C	Complex Number 138, probably RNA metabolism
YDR378C	Complex Number 147, probably RNA metabolism
YDR378C	Complex Number 14, U6-specific snRNP core (14)
YDR379W	Complex Number 169, probably signalling
YDR381W	Complex Number 102, probably protein synthesis turnover
YDR381W	Complex Number 106
YDR381W	Complex Number 123
YDR381W	Complex Number 136, probably RNA metabolism
YDR381W	Complex Number 170, probably signalling
YDR381W	Complex Number 200
YDR381W	Complex Number 201
YDR381W	Complex Number 211, probably transcription/DNA maintanance/chromatin structure
YDR381W	Complex Number 224, probably transcription/DNA maintanance/chromatin structure
YDR381W	Complex Number 334
YDR381W	Complex Number 397
YDR381W	Complex Number 446
YDR381W	Complex Number 458
YDR381W	Complex Number 56
YDR381W	Complex Number 91

YDR382W	Complex Number 124, probably protein/RNA transport
YDR382W	Complex Number 125, probably protein/RNA transport
YDR382W	Complex Number 144, probably RNA metabolism
YDR382W	Complex Number 149, probably RNA metabolism
YDR382W	Complex Number 152, probably RNA metabolism
YDR382W	cytoplasmic ribosomal large subunit
YDR383C	Complex Number 221, probably transcription/DNA maintanance/chromatin structure
YDR385W	Complex Number 91, eEF2 (2)
YDR385W	eEF2
YDR386W	Complex Number 200
YDR386W	Complex Number 201
YDR388W	Actin-associated proteins
YDR388W	Complex Number 155
YDR388W	Complex Number 156
YDR388W	Complex Number 316
YDR388W	Complex Number 326
YDR388W	Complex Number 327
YDR388W	Complex Number 328
YDR388W	Complex Number 329
YDR390C	Complex Number 114, probably protein synthesis turnover
YDR392W	SAGA complex
YDR392W	SAGA-like complex (SLIK)
YDR394W	19/22S regulator
YDR394W	Complex Number 102, probably protein synthesis turnover
YDR394W	Complex Number 110, probably protein synthesis turnover
YDR394W	Complex Number 111, probably protein synthesis turnover
YDR394W	Complex Number 195, probably transcription/DNA maintanance/chromatin structure
YDR394W	Complex Number 202, probably transcription/DNA maintanance/chromatin structure
YDR394W	Complex Number 204, probably transcription/DNA maintanance/chromatin structure
YDR394W	Complex Number 23
YDR394W	Complex Number 231, probably transcription/DNA maintanance/chromatin structure
YDR394W	Complex Number 265
YDR394W	Complex Number 272
YDR394W	Complex Number 275
YDR394W	Complex Number 284
YDR394W	Complex Number 300
YDR394W	Complex Number 302
YDR394W	Complex Number 320
YDR394W	Complex Number 338
YDR394W	Complex Number 360
YDR394W	Complex Number 4
YDR394W	Complex Number 41, probably intermediate and energy metabolism
YDR394W	Complex Number 468
YDR394W	Complex Number 501

YDR394W	Complex Number 50, 19S Proteosome (17)
YDR394W	Complex Number 77
YDR394W	Complex Number 99
YDR395W	Complex Number 171, probably signalling
YDR395W	Complex Number 384
YDR395W	Complex Number 544
YDR397C	NC2 complex
YDR398W	Complex Number 43, UTP A (8)
YDR398W	Complex Number 459
YDR398W	rRNA splicing
YDR404C	Complex Number 210, probably transcription/DNA maintanance/chromatin structure
YDR404C	Complex Number 24, RNA Polymerase II (12)
YDR404C	RNA polymerase II
YDR405W	Complex Number 108, probably protein synthesis turnover
YDR405W	mitochondrial ribosomal large subunit
YDR407C	Complex Number 121
YDR407C	Complex Number 75, probably membrane biogenesis and traffic
YDR407C	TRAPP (Transport Protein Particle) complex
YDR412W	Complex Number 115
YDR412W	Complex Number 520
YDR412W	Complex Number 94
YDR416W	Complex Number 143, probably RNA metabolism
YDR416W	Complex Number 147, probably RNA metabolism
YDR416W	Complex Number 451
YDR418W	cytoplasmic ribosomal large subunit
YDR422C	Complex Number 166, probably signalling
YDR422C	Complex Number 368
YDR422C	Complex Number 504
YDR422C	SNF1 complex
YDR424C	Complex Number 212
YDR424C	Dynein-complex motorproteins
YDR427W	19/22S regulator
YDR427W	Complex Number 10, probably cell cycle
YDR427W	Complex Number 110, probably protein synthesis turnover
YDR427W	Complex Number 111, probably protein synthesis turnover
YDR427W	Complex Number 195, probably transcription/DNA maintanance/chromatin structure
YDR427W	Complex Number 196, probably transcription/DNA maintanance/chromatin structure
YDR427W	Complex Number 202, probably transcription/DNA maintanance/chromatin structure
YDR427W	Complex Number 204, probably transcription/DNA maintanance/chromatin structure
YDR427W	Complex Number 214, probably transcription/DNA maintanance/chromatin structure
YDR427W	Complex Number 313
YDR427W	Complex Number 318
YDR427W	Complex Number 320
YDR427W	Complex Number 468

YDR427W	Complex Number 50, 19S Proteosome (17)
YDR429C	Complex Number 107, probably protein synthesis turnover
YDR429C	Complex Number 16, Ded1 (1)
YDR429C	Complex Number 189, probably transcription/DNA maintanance/chromatin structure
YDR429C	Complex Number 338
YDR429C	Complex Number 379
YDR429C	Complex Number 51, eIF3 (7)
YDR429C	Complex Number 535
YDR429C	eIF3
YDR430C	Complex Number 42, probably intermediate and energy metabolism
YDR432W	Complex Number 139, probably RNA metabolism
YDR432W	Complex Number 146, probably RNA metabolism
YDR432W	Complex Number 155, probably RNA metabolism
YDR432W	Complex Number 20, Npl3 (1)
YDR432W	Complex Number 227, probably transcription/DNA maintanance/chromatin structure
YDR432W	Complex Number 344
YDR432W	Complex Number 444
YDR432W	Complex Number 446
YDR432W	Complex Number 473
YDR432W	Complex Number 489
YDR432W	Complex Number 517
YDR432W	Complex Number 74
YDR432W	rRNA splicing
YDR436W	Complex Number 116
YDR436W	Complex Number 237
YDR436W	Complex Number 336
YDR443C	Complex Number 209, probably transcription/DNA maintanance/chromatin structure
YDR443C	Srb10p complex
YDR447C	cytoplasmic ribosomal small subunit
YDR448W	ADA complex
YDR448W	Complex Number 112
YDR448W	Complex Number 207, probably transcription/DNA maintanance/chromatin structure
YDR448W	Complex Number 209, probably transcription/DNA maintanance/chromatin structure
YDR448W	Complex Number 214, probably transcription/DNA maintanance/chromatin structure
YDR448W	SAGA complex
YDR448W	SAGA-like complex (SLIK)
YDR449C	Complex Number 107, probably protein synthesis turnover
YDR449C	Complex Number 109, probably protein synthesis turnover
YDR449C	Complex Number 125, probably protein/RNA transport
YDR449C	Complex Number 144, probably RNA metabolism
YDR449C	Complex Number 255
YDR449C	Complex Number 493
YDR449C	Complex Number 87, UTP B (6)
YDR449C	rRNA splicing

YDR450W	cytoplasmic ribosomal small subunit
YDR453C	Complex Number 261
YDR453C	Complex Number 316
YDR453C	Complex Number 341
YDR453C	Complex Number 37
YDR453C	Complex Number 424
YDR453C	Complex Number 504
YDR457W	Complex Number 95
YDR460W	Complex Number 206, probably transcription/DNA maintanance/chromatin structure
YDR460W	Complex Number 271
YDR460W	Complex Number 393
YDR460W	NEF3 complex
YDR460W	RNA polymerase I
YDR462W	Complex Number 108, probably protein synthesis turnover
YDR462W	Complex Number 147, probably RNA metabolism
YDR462W	mitochondrial ribosomal large subunit
YDR463W	tRNA splicing
YDR465C	Complex Number 353
YDR468C	Complex Number 71, probably membrane biogenesis and traffic
YDR468C	t-SNAREs
YDR469W	Complex Number 199, probably transcription/DNA maintanance/chromatin structure
YDR471W	cytoplasmic ribosomal large subunit
YDR472W	Complex Number 75, probably membrane biogenesis and traffic
YDR472W	TRAPP (Transport Protein Particle) complex
YDR473C	Complex Number 145, probably RNA metabolism
YDR473C	Complex Number 146, probably RNA metabolism
YDR473C	Complex Number 147, probably RNA metabolism
YDR473C	Complex Number 14, U6-specific snRNP core (14)
YDR473C	Complex Number 244
YDR473C	Nuclear splicing complexes/Spliceosome
YDR477W	Complex Number 166, probably signalling
YDR477W	Complex Number 289
YDR477W	Complex Number 348
YDR477W	Complex Number 367
YDR477W	Complex Number 540
YDR477W	SNF1 complex
YDR478W	rRNA splicing
YDR480W	Complex Number 152
YDR480W	Complex Number 83
YDR482C	Complex Number 460
YDR482C	Nuclear splicing complexes/Spliceosome
YDR484W	Complex Number 74, probably membrane biogenesis and traffic
YDR488C	Complex Number 212
YDR488C	Dynein-complex motorproteins

YDR489W	0
YDR490C	Complex Number 227
YDR496C	Complex Number 140, probably RNA metabolism
YDR496C	Complex Number 149, probably RNA metabolism
YDR496C	Complex Number 204
YDR496C	Complex Number 214, probably transcription/DNA maintanance/chromatin structure
YDR496C	Complex Number 22
YDR496C	Complex Number 220, probably transcription/DNA maintanance/chromatin structure
YDR496C	Complex Number 375
YDR496C	Complex Number 446
YDR496C	Complex Number 507
YDR496C	Complex Number 56
YDR496C	Complex Number 73, Puf6 (1)
YDR496C	Complex Number 91
YDR496C	Complex Number 96
YDR496C	Complex Number 99, probably protein synthesis turnover
YDR498C	t-SNAREs
YDR499W	Complex Number 157
YDR499W	Complex Number 212, probably transcription/DNA maintanance/chromatin structure
YDR500C	cytoplasmic ribosomal large subunit
YDR502C	Complex Number 123, probably protein/RNA transport
YDR507C	Complex Number 136, probably RNA metabolism
YDR507C	Complex Number 224, probably transcription/DNA maintanance/chromatin structure
YDR507C	Complex Number 251
YDR507C	Complex Number 308
YDR510W	Complex Number 366
YDR516C	Complex Number 354
YDR517W	Complex Number 123
YDR523C	Complex Number 1
YDR523C	Complex Number 374
YDR529C	Complex Number 411
YDR529C	Cytochrome bc1 complex (Ubiquinol-cytochrome c reductase complex, complex III)
YEL002C	Complex Number 417
YEL002C	Complex Number 44, probably intermediate and energy metabolism
YEL002C	Oligosaccharyltransferase
YEL003W	Gim complexes
YEL013W	Complex Number 109
YEL013W	Complex Number 297
YEL015W	Complex Number 132
YEL015W	Complex Number 133
YEL015W	Complex Number 150, probably RNA metabolism
YEL017C-A	H+-ATPase, plasma mebrane
YEL018W	Complex Number 191, probably transcription/DNA maintanance/chromatin structure
YEL020W-A	Tim22p-complex

YEL020W-A	Tim9p/Tim10p-complex
YEL021W	Complex Number 416
YEL022W	Complex Number 123, probably protein/RNA transport
YEL022W	Complex Number 53, probably intermediate and energy metabolism
YEL023C	Complex Number 540
YEL024W	Complex Number 182
YEL024W	Cytochrome bc1 complex (Ubiquinol-cytochrome c reductase complex, complex III)
YEL026W	Complex Number 149, probably RNA metabolism
YEL026W	Complex Number 341
YEL027W	H+-transporting ATPase, vacuolar
YEL030W	Complex Number 206
YEL030W	Complex Number 208
YEL030W	Complex Number 239
YEL030W	Complex Number 267
YEL030W	Complex Number 283
YEL030W	Complex Number 29
YEL030W	Complex Number 36
YEL030W	Complex Number 365
YEL030W	Complex Number 405
YEL030W	Complex Number 49
YEL030W	Complex Number 50
YEL030W	Complex Number 504
YEL030W	Complex Number 51
YEL030W	Complex Number 61
YEL030W	Complex Number 67
YEL030W	Complex Number 91
YEL031W	Complex Number 27, probably intermediate and energy metabolism
YEL032W	Complex Number 82
YEL032W	Pre-replication complex (pre-RC)
YEL032W	Replication complex
YEL034W	Complex Number 132
YEL034W	Complex Number 134
YEL034W	Complex Number 22
YEL034W	Complex Number 244
YEL034W	Complex Number 27
YEL034W	Complex Number 319
YEL034W	Complex Number 320
YEL034W	Complex Number 40
YEL034W	Complex Number 419
YEL034W	Complex Number 435
YEL034W	Complex Number 501
YEL034W	Complex Number 507
YEL034W	Complex Number 58
YEL034W	Complex Number 62

YEL034W	eIF5A
YEL036C	Complex Number 72, probably membrane biogenesis and traffic
YEL037C	Complex Number 202, probably transcription/DNA maintanance/chromatin structure
YEL037C	Complex Number 413
YEL037C	Complex Number 438
YEL037C	NEF2 complex
YEL046C	Complex Number 204, probably transcription/DNA maintanance/chromatin structure
YEL050C	Complex Number 125, probably protein/RNA transport
YEL050C	Complex Number 144, probably RNA metabolism
YEL050C	mitochondrial ribosomal large subunit
YEL051W	Complex Number 109
YEL051W	Complex Number 111
YEL051W	Complex Number 146
YEL051W	Complex Number 274
YEL051W	Complex Number 283
YEL051W	Complex Number 374
YEL051W	Complex Number 45, probably intermediate and energy metabolism
YEL051W	Complex Number 505
YEL051W	H+-transporting ATPase, vacuolar
YEL053C	Complex Number 117, probably protein synthesis turnover
YEL054C	cytoplasmic ribosomal large subunit
YEL055C	Complex Number 123
YEL055C	Complex Number 150
YEL055C	Complex Number 229, probably transcription/DNA maintanance/chromatin structure
YEL055C	Complex Number 412
YEL055C	Complex Number 43, UTP A (8)
YEL055C	Complex Number 446
YEL056W	Complex Number 125
YEL056W	Complex Number 204, probably transcription/DNA maintanance/chromatin structure
YEL056W	HAT B complex
YEL058W	Complex Number 60, probably intermediate and energy metabolism
YEL060C	Complex Number 128
YEL060C	Complex Number 180
YEL060C	Complex Number 187
YEL060C	Complex Number 207
YEL060C	Complex Number 208
YEL060C	Complex Number 283
YEL060C	Complex Number 284
YEL060C	Complex Number 29
YEL060C	Complex Number 338
YEL060C	Complex Number 357
YEL060C	Complex Number 367
YEL060C	Complex Number 374
YEL060C	Complex Number 383

YEL060C	Complex Number 4
YEL060C	Complex Number 434
YEL060C	Complex Number 47
YEL060C	Complex Number 540
YEL060C	Complex Number 69
YEL061C	Complex Number 150
YEL061C	Kinesin-related motorproteins
YEL062W	Complex Number 447
YEL064C	Complex Number 347
YEL071W	Complex Number 154
YEL071W	Complex Number 304
YER002W	Complex Number 123
YER002W	Complex Number 149, probably RNA metabolism
YER002W	Complex Number 520
YER002W	Complex Number 94
YER003C	Complex Number 244
YER003C	Complex Number 327
YER004W	Complex Number 25, RNA polymerase I (7)
YER006W	Complex Number 108, probably protein synthesis turnover
YER006W	Complex Number 132
YER006W	Complex Number 138, probably RNA metabolism
YER006W	Complex Number 140, probably RNA metabolism
YER006W	Complex Number 149, probably RNA metabolism
YER006W	Complex Number 201
YER006W	Complex Number 205
YER006W	Complex Number 22
YER006W	Complex Number 441
YER006W	Complex Number 446
YER006W	Complex Number 469
YER006W	Complex Number 475
YER006W	Complex Number 507
YER006W	Complex Number 520
YER006W	Complex Number 56
YER006W	Complex Number 86, probably protein synthesis turnover
YER006W	Complex Number 94
YER007C-A	Complex Number 137, probably RNA metabolism
YER007W	Complex Number 213
YER008C	Complex Number 81, probably membrane biogenesis and traffic
YER008C	Complex Number 96, probably protein synthesis turnover
YER008C	Exocyst complex
YER009W	Complex Number 341
YER012W	20S proteasome
YER012W	Complex Number 110, probably protein synthesis turnover
YER012W	Complex Number 111, probably protein synthesis turnover

YER012W	Complex Number 170, probably signalling
YER012W	Complex Number 238
YER012W	Complex Number 504
YER012W	Complex Number 60, 20S Proteosome (13)
YER013W	Complex Number 143, probably RNA metabolism
YER013W	mRNA splicing
YER015W	Complex Number 382
YER016W	Tubulin-associated proteins
YER017C	Complex Number 2
YER017C	m-AAA protease complex
YER018C	Complex Number 371
YER018C	Ndc80 protein complex
YER018C	SPB components
YER019C-A	ER protein-translocation complex (Sec complex)
YER020W	Complex Number 118
YER021W	19/22S regulator
YER021W	Complex Number 110, probably protein synthesis turnover
YER021W	Complex Number 111, probably protein synthesis turnover
YER021W	Complex Number 202, probably transcription/DNA maintanance/chromatin structure
YER021W	Complex Number 204, probably transcription/DNA maintanance/chromatin structure
YER021W	Complex Number 316
YER021W	Complex Number 320
YER021W	Complex Number 4
YER021W	Complex Number 468
YER021W	Complex Number 50, 19S Proteosome (17)
YER021W	Complex Number 53, probably intermediate and energy metabolism
YER022W	Complex Number 209, probably transcription/DNA maintanance/chromatin structure
YER022W	Complex Number 33, Mediator (8)
YER022W	Kornberg's mediator (SRB) complex
YER023W	Complex Number 109
YER023W	Complex Number 338
YER025W	Complex Number 105, probably protein synthesis turnover
YER025W	Complex Number 107
YER025W	Complex Number 109
YER025W	Complex Number 111
YER025W	Complex Number 283
YER025W	Complex Number 294
YER025W	Complex Number 316
YER025W	Complex Number 32, RSC (13)
YER025W	Complex Number 371
YER025W	Complex Number 378
YER025W	Complex Number 380
YER025W	Complex Number 388
YER025W	Complex Number 4

YER025W	Complex Number 497
YER025W	Complex Number 51, eIF3 (7)
YER025W	Complex Number 52, tIF2 (7)
YER025W	Complex Number 7, Dbp7/Rrp5 (4)
YER025W	eIF2
YER027C	Complex Number 166, probably signalling
YER027C	Complex Number 25
YER027C	Complex Number 348
YER027C	Complex Number 367
YER027C	SNF1 complex
YER029C	Complex Number 138, probably RNA metabolism
YER029C	Complex Number 140, probably RNA metabolism
YER029C	Complex Number 145, probably RNA metabolism
YER029C	Complex Number 146, probably RNA metabolism
YER029C	Complex Number 147, probably RNA metabolism
YER029C	Complex Number 165
YER030W	Complex Number 509
YER031C	Complex Number 113
YER031C	Complex Number 73, probably membrane biogenesis and traffic
YER036C	Complex Number 116, probably protein synthesis turnover
YER041W	Complex Number 461
YER043C	Complex Number 119
YER043C	Complex Number 123
YER043C	Complex Number 143
YER043C	Complex Number 258
YER043C	Complex Number 341
YER043C	Complex Number 350
YER043C	Complex Number 375
YER043C	Complex Number 435
YER043C	Complex Number 454
YER043C	Complex Number 499
YER043C	Complex Number 520
YER043C	Complex Number 529
YER043C	Complex Number 94
YER047C	Complex Number 202
YER049W	Complex Number 19, mRNA cap-binding/eIF4F (8)
YER049W	Complex Number 2, Yer049w (1)
YER049W	Complex Number 338
YER050C	mitochondrial ribosomal small subunit
YER052C	Complex Number 103
YER052C	Complex Number 120
YER052C	Complex Number 283
YER052C	Complex Number 284
YER052C	Complex Number 308

YER054C	Complex Number 114
YER054C	Serine/threonine phoshpoprotein phosphatase
YER056C-A	cytoplasmic ribosomal large subunit
YER057C	Complex Number 413
YER059W	Complex Number 217
YER062C	Complex Number 272
YER062C	Complex Number 283
YER062C	Complex Number 296
YER062C	Complex Number 364
YER065C	Complex Number 204, probably transcription/DNA maintanance/chromatin structure
YER065C	Isocitrate lyase
YER066C-A	Complex Number 462
YER067W	Complex Number 319
YER068W	CCR4 complex
YER068W	Complex Number 211, probably transcription/DNA maintanance/chromatin structure
YER068W	NOT complex
YER070W	Complex Number 313
YER070W	Complex Number 389
YER070W	Complex Number 79
YER070W	Ribonucleoside-diphosphate reductase
YER071C	Complex Number 16, probably cell polarity and structure
YER073W	Complex Number 277
YER074W	cytoplasmic ribosomal small subunit
YER075C	Complex Number 253
YER077C	Complex Number 505
YER077C	Complex Number 91
YER077C	Complex Number 96
YER078C	Complex Number 201
YER078C	Complex Number 292
YER081W	Complex Number 24, probably intermediate and energy metabolism
YER082C	Complex Number 109, probably protein synthesis turnover
YER082C	Complex Number 125, probably protein/RNA transport
YER082C	Complex Number 144, probably RNA metabolism
YER082C	Complex Number 150
YER082C	Complex Number 375
YER082C	rRNA splicing
YER083C	Complex Number 442
YER084W	Complex Number 56
YER086W	Complex Number 103, probably protein synthesis turnover
YER086W	Complex Number 195, probably transcription/DNA maintanance/chromatin structure
YER086W	Complex Number 41, probably intermediate and energy metabolism
YER086W	Complex Number 79, probably membrane biogenesis and traffic
YER087C-B	Sec61 complex
YER088C	Complex Number 208, probably transcription/DNA maintanance/chromatin structure

YER089C	Complex Number 279
YER090W	Anthranilate synthase
YER090W	Complex Number 30, probably intermediate and energy metabolism
YER090W	Complex Number 43
YER091C	Complex Number 341
YER091C	Complex Number 35
YER091C	Complex Number 520
YER091C	Complex Number 94
YER092W	Complex Number 203, probably transcription/DNA maintanance/chromatin structure
YER093C	Complex Number 152
YER093C	Complex Number 204, probably transcription/DNA maintanance/chromatin structure
YER094C	20S proteasome
YER094C	Complex Number 102, probably protein synthesis turnover
YER094C	Complex Number 110, probably protein synthesis turnover
YER094C	Complex Number 111, probably protein synthesis turnover
YER094C	Complex Number 170, probably signalling
YER094C	Complex Number 238
YER094C	Complex Number 504
YER095W	Complex Number 18, probably cell polarity and structure
YER095W	Complex Number 195, probably transcription/DNA maintanance/chromatin structure
YER095W	Complex Number 276
YER095W	other DNA repair complexes
YER098W	Complex Number 453
YER099C	Complex Number 308
YER100W	Complex Number 412
YER102W	cytoplasmic ribosomal small subunit
YER103W	Complex Number 135, probably RNA metabolism
YER103W	Complex Number 189, probably transcription/DNA maintanance/chromatin structure
YER103W	Complex Number 85, probably protein synthesis turnover
YER105C	Nuclear pore complex (NPC)
YER107C	Complex Number 63, probably intermediate and energy metabolism
YER107C	Nuclear pore complex (NPC)
YER110C	Complex Number 103, probably protein synthesis turnover
YER110C	Complex Number 119, probably protein/RNA transport
YER110C	Complex Number 123, probably protein/RNA transport
YER110C	Complex Number 171, probably signalling
YER110C	Complex Number 17, probably cell polarity and structure
YER110C	Complex Number 195, probably transcription/DNA maintanance/chromatin structure
YER110C	Complex Number 197, probably transcription/DNA maintanance/chromatin structure
YER110C	Complex Number 19, mRNA cap-binding/eIF4F (8)
YER110C	Complex Number 204, probably transcription/DNA maintanance/chromatin structure
YER110C	Complex Number 214, probably transcription/DNA maintanance/chromatin structure
YER110C	Complex Number 35, probably intermediate and energy metabolism
YER110C	Complex Number 39, probably intermediate and energy metabolism

YER110C	Complex Number 41, probably intermediate and energy metabolism
YER110C	Complex Number 53, probably intermediate and energy metabolism
YER110C	Complex Number 69, probably membrane biogenesis and traffic
YER110C	Complex Number 70, probably membrane biogenesis and traffic
YER110C	Complex Number 85, probably protein synthesis turnover
YER110C	Complex Number 8, probably cell cycle
YER111C	Complex Number 279
YER111C	SBF complex
YER112W	Complex Number 138, probably RNA metabolism
YER112W	Complex Number 145, probably RNA metabolism
YER112W	Complex Number 146, probably RNA metabolism
YER112W	Complex Number 147, probably RNA metabolism
YER112W	Complex Number 163
YER112W	Complex Number 164
YER112W	Complex Number 214
YER112W	Complex Number 215
YER112W	mRNA splicing
YER114C	Complex Number 17
YER114C	Complex Number 5, probably cell cycle
YER117W	Complex Number 463
YER117W	cytoplasmic ribosomal large subunit
YER120W	Complex Number 23, probably intermediate and energy metabolism
YER120W	Complex Number 485
YER120W	Complex Number 520
YER120W	Complex Number 94
YER122C	Complex Number 79, probably membrane biogenesis and traffic
YER123W	Casein kinase I
YER123W	Complex Number 435
YER125W	Complex Number 191
YER125W	Complex Number 322
YER125W	Complex Number 323
YER125W	Complex Number 327
YER126C	Complex Number 123
YER126C	Complex Number 149, probably RNA metabolism
YER126C	Complex Number 205
YER126C	Complex Number 22
YER126C	Complex Number 507
YER127W	Complex Number 144, probably RNA metabolism
YER127W	Complex Number 493
YER129W	Complex Number 166, probably signalling
YER131W	cytoplasmic ribosomal small subunit
YER132C	Complex Number 308
YER133W	Complex Number 114
YER133W	Complex Number 115

YER133W	Complex Number 116
YER133W	Complex Number 125, probably protein/RNA transport
YER133W	Complex Number 133
YER133W	Complex Number 148, probably RNA metabolism
YER133W	Complex Number 158, probably signalling
YER133W	Complex Number 184
YER133W	Complex Number 336
YER133W	Complex Number 37
YER133W	Complex Number 465
YER133W	Complex Number 538
YER133W	Complex Number 56, mRNA cleavage/polyadenylation (10)
YER133W	Serine/threonine phoshpoprotein phosphatase
YER136W	Complex Number 103
YER136W	Complex Number 113
YER136W	Complex Number 267
YER136W	Complex Number 418
YER136W	Complex Number 546
YER136W	Complex Number 547
YER136W	Complex Number 549
YER136W	Complex Number 73, probably membrane biogenesis and traffic
YER138C	Complex Number 132
YER138C	Complex Number 308
YER138C	Complex Number 316
YER138C	Complex Number 459
YER138C	Complex Number 58
YER139C	Complex Number 149, probably RNA metabolism
YER142C	Complex Number 169
YER142C	Complex Number 170
YER146W	Complex Number 138, probably RNA metabolism
YER146W	Complex Number 13, U4/U6.U5 tri-snRNP (2)
YER146W	Complex Number 147, probably RNA metabolism
YER146W	Complex Number 14, U6-specific snRNP core (14)
YER147C	Complex Number 8, probably cell cycle
YER148W	Complex Number 192, probably transcription/DNA maintanance/chromatin structure
YER148W	Complex Number 196, probably transcription/DNA maintanance/chromatin structure
YER148W	Complex Number 214, probably transcription/DNA maintanance/chromatin structure
YER148W	Core Factor (CF)
YER148W	TBP
YER148W	TFIIIB
YER153C	Mitochondrial translation complexes
YER155C	Complex Number 104, probably protein synthesis turnover
YER155C	Complex Number 106, probably protein synthesis turnover
YER155C	Complex Number 108, probably protein synthesis turnover
YER155C	Complex Number 109, probably protein synthesis turnover

YER155C	Complex Number 135, probably RNA metabolism
YER155C	Complex Number 149, probably RNA metabolism
YER155C	Complex Number 169, probably signalling
YER155C	Complex Number 198, probably transcription/DNA maintanance/chromatin structure
YER155C	Complex Number 1, probably cell cycle
YER155C	Complex Number 283
YER155C	Complex Number 535
YER157W	Complex Number 209, probably transcription/DNA maintanance/chromatin structure
YER157W	Golgi transport complex
YER159C	NC2 complex
YER160C	Complex Number 308
YER160C	Complex Number 385
YER161C	Complex Number 344
YER161C	Complex Number 375
YER162C	Complex Number 202, probably transcription/DNA maintanance/chromatin structure
YER162C	Complex Number 259
YER162C	NEF2 complex
YER164W	Complex Number 135
YER164W	Complex Number 189, probably transcription/DNA maintanance/chromatin structure
YER164W	Complex Number 46, SPT16 (8)
YER164W	Complex Number 47, Casein Kinase II (4)
YER165W	Complex Number 17, Pab1 (1)
YER165W	Complex Number 244
YER165W	Complex Number 6
YER165W	eIF4E/eIF4G/Pab1p complex
YER165W	other RNA processing components
YER167W	Complex Number 153
YER168C	Complex Number 100, probably protein synthesis turnover
YER171W	Complex Number 180
YER171W	Complex Number 231, probably transcription/DNA maintanance/chromatin structure
YER171W	Complex Number 267
YER171W	Complex Number 271
YER171W	Complex Number 272
YER171W	Complex Number 273
YER171W	Complex Number 393
YER171W	NEF3 complex
YER171W	RNA polymerase I
YER172C	Complex Number 13, U4/U6.U5 tri-snRNP (2)
YER172C	Complex Number 14, U6-specific snRNP core (14)
YER172C	Complex Number 244
YER172C	Complex Number 297
YER172C	mRNA splicing
YER173W	Complex Number 264
YER173W	Complex Number 265

YER174C	Complex Number 23
YER174C	Complex Number 471
YER176W	DNA helicases
YER177W	Complex Number 123, probably protein/RNA transport
YER177W	Complex Number 161, probably signalling
YER177W	Complex Number 166, probably signalling
YER177W	Complex Number 17
YER177W	Complex Number 4, Dbp3/Bmh1/Nsr1 (3)
YER178W	Complex Number 102, probably protein synthesis turnover
YER178W	Complex Number 216
YER178W	Complex Number 338
YER178W	Complex Number 42, probably intermediate and energy metabolism
YER178W	Pyruvate dehydrogenase
YER179W	Complex Number 209, probably transcription/DNA maintanance/chromatin structure
YER179W	Complex Number 84
YER179W	Kornberg's mediator (SRB) complex
YER182W	Complex Number 447
YFL002C	Complex Number 149, probably RNA metabolism
YFL002C	Complex Number 520
YFL002C	Complex Number 94
YFL003C	Complex Number 139, probably RNA metabolism
YFL005W	Complex Number 113
YFL005W	Complex Number 546
YFL005W	Complex Number 89
YFL007W	Complex Number 102, probably protein synthesis turnover
YFL007W	Complex Number 111, probably protein synthesis turnover
YFL007W	Complex Number 170, probably signalling
YFL007W	Complex Number 22
YFL007W	Complex Number 351
YFL007W	Complex Number 352
YFL007W	Complex Number 507
YFL008W	Complex Number 172
YFL008W	Complex Number 229, probably transcription/DNA maintanance/chromatin structure
YFL008W	Complex Number 361
YFL008W	Complex Number 46
YFL008W	Complex Number 8, probably cell cycle
YFL008W	Sister chromatid cohesion complex
YFL009W	Complex Number 357
YFL009W	Complex Number 44
YFL009W	SCF-CDC4 complex
YFL013C	Complex Number 203, probably transcription/DNA maintanance/chromatin structure
YFL014W	Complex Number 136
YFL016C	Complex Number 447
YFL016C	Complex Number 84

YFL017W-A	Complex Number 145, probably RNA metabolism
YFL017W-A	Complex Number 146, probably RNA metabolism
YFL017W-A	Complex Number 147, probably RNA metabolism
YFL018C	2-oxoglutarate dehydrogenase
YFL018C	Complex Number 2
YFL018C	Complex Number 327
YFL018C	Complex Number 33
YFL018C	Complex Number 39, probably intermediate and energy metabolism
YFL018C	Complex Number 42, probably intermediate and energy metabolism
YFL018C	Glycine decarboxylase
YFL018C	Pyruvate dehydrogenase
YFL022C	Complex Number 111
YFL022C	Complex Number 113, probably protein synthesis turnover
YFL022C	Complex Number 341
YFL022C	Complex Number 503
YFL022C	Phenylalaninine-tRNA-ligase
YFL024C	Complex Number 191, probably transcription/DNA maintanance/chromatin structure
YFL024C	Complex Number 334
YFL028C	CCR4 complex
YFL030W	Complex Number 224
YFL030W	Complex Number 90
YFL033C	Complex Number 309
YFL033C	Complex Number 400
YFL034C-A	cytoplasmic ribosomal large subunit
YFL034C-B	Complex Number 192
YFL034C-B	Complex Number 193
YFL034C-B	Complex Number 29
YFL034W	Complex Number 154
YFL034W	Complex Number 464
YFL037W	Tubulins
YFL038C	Complex Number 113
YFL038C	Complex Number 412
YFL038C	Complex Number 546
YFL038C	Complex Number 73, probably membrane biogenesis and traffic
YFL038C	Complex Number 89
YFL039C	Actins
YFL039C	Complex Number 148, probably RNA metabolism
YFL039C	Complex Number 167, probably signalling
YFL039C	Complex Number 17, probably cell polarity and structure
YFL039C	Complex Number 191, probably transcription/DNA maintanance/chromatin structure
YFL039C	Complex Number 203, probably transcription/DNA maintanance/chromatin structure
YFL039C	Complex Number 229, probably transcription/DNA maintanance/chromatin structure
YFL039C	Complex Number 3, probably cell cycle
YFL039C	Complex Number 52, probably intermediate and energy metabolism

YFL039C	Complex Number 52, tIF2 (7)
YFL041W	Complex Number 53, probably intermediate and energy metabolism
YFL042C	Complex Number 316
YFL045C	Complex Number 130
YFL045C	Complex Number 267
YFL045C	Complex Number 283
YFL045C	Complex Number 341
YFL045C	Complex Number 348
YFL045C	Complex Number 35
YFL045C	Complex Number 352
YFL045C	Complex Number 378
YFL045C	Complex Number 388
YFL045C	Complex Number 459
YFL045C	Complex Number 520
YFL045C	Complex Number 8
YFL045C	Complex Number 94
YFL049W	Complex Number 198, probably transcription/DNA maintanance/chromatin structure
YFL053W	Complex Number 455
YFR001W	Complex Number 123
YFR001W	Complex Number 132
YFR001W	Complex Number 149, probably RNA metabolism
YFR001W	Complex Number 200
YFR001W	Complex Number 201
YFR001W	Complex Number 91
YFR002W	NSP1 complex
YFR003C	Complex Number 115
YFR003C	Complex Number 465
YFR004W	19/22S regulator
YFR004W	Complex Number 110, probably protein synthesis turnover
YFR004W	Complex Number 111, probably protein synthesis turnover
YFR004W	Complex Number 300
YFR004W	Complex Number 320
YFR004W	Complex Number 4
YFR004W	Complex Number 41, probably intermediate and energy metabolism
YFR004W	Complex Number 42, probably intermediate and energy metabolism
YFR004W	Complex Number 468
YFR004W	Complex Number 50, 19S Proteosome (17)
YFR006W	Complex Number 204, probably transcription/DNA maintanance/chromatin structure
YFR008W	Complex Number 449
YFR009W	Complex Number 172, probably signalling
YFR009W	Complex Number 195, probably transcription/DNA maintanance/chromatin structure
YFR009W	Complex Number 231, probably transcription/DNA maintanance/chromatin structure
YFR009W	Complex Number 25, probably intermediate and energy metabolism
YFR009W	Complex Number 41, probably intermediate and energy metabolism

YFR009W	Complex Number 70, probably membrane biogenesis and traffic
YFR010W	Complex Number 110, probably protein synthesis turnover
YFR010W	Complex Number 111, probably protein synthesis turnover
YFR010W	Complex Number 50, 19S Proteosome (17)
YFR011C	Complex Number 315
YFR011C	Complex Number 396
YFR013W	Complex Number 192, probably transcription/DNA maintanance/chromatin structure
YFR013W	Complex Number 200, probably transcription/DNA maintanance/chromatin structure
YFR013W	Complex Number 203, probably transcription/DNA maintanance/chromatin structure
YFR013W	Complex Number 208, probably transcription/DNA maintanance/chromatin structure
YFR014C	Complex Number 63
YFR015C	Complex Number 115
YFR015C	Complex Number 41
YFR015C	Complex Number 469
YFR016C	Complex Number 466
YFR017C	Complex Number 17
YFR019W	Complex Number 1, probably cell cycle
YFR019W	Complex Number 209, probably transcription/DNA maintanance/chromatin structure
YFR021W	Complex Number 203
YFR021W	Complex Number 58, probably intermediate and energy metabolism
YFR024C-A	Complex Number 18, probably cell polarity and structure
YFR024C-A	Complex Number 467
YFR028C	Complex Number 194, probably transcription/DNA maintanance/chromatin structure
YFR028C	Complex Number 213, probably transcription/DNA maintanance/chromatin structure
YFR028C	Complex Number 37
YFR028C	Complex Number 520
YFR028C	Complex Number 94
YFR030W	Complex Number 4
YFR030W	Complex Number 41
YFR031C-A	Complex Number 86, probably protein synthesis turnover
YFR031C-A	cytoplasmic ribosomal large subunit
YFR032C-A	cytoplasmic ribosomal large subunit
YFR033C	Cytochrome bc1 complex (Ubiquinol-cytochrome c reductase complex, complex III)
YFR036W	Anaphase promoting complex (APC)
YFR037C	Complex Number 192, probably transcription/DNA maintanance/chromatin structure
YFR037C	Complex Number 200, probably transcription/DNA maintanance/chromatin structure
YFR037C	Complex Number 203, probably transcription/DNA maintanance/chromatin structure
YFR037C	Complex Number 208, probably transcription/DNA maintanance/chromatin structure
YFR037C	Complex Number 212, probably transcription/DNA maintanance/chromatin structure
YFR037C	Complex Number 213, probably transcription/DNA maintanance/chromatin structure
YFR037C	Complex Number 32, RSC (13)
YFR037C	Complex Number 53, Kap95/Srp1 (2)
YFR037C	RSC complex (Remodel the structure of chromatin)
YFR039C	Complex Number 166

YFR040W	Complex Number 12, Sit4 (6)
YFR040W	Complex Number 1, probably cell cycle
YFR040W	Complex Number 203
YFR040W	Complex Number 333
YFR040W	Complex Number 353
YFR044C	Complex Number 304
YFR044C	Complex Number 70, probably membrane biogenesis and traffic
YFR049W	Complex Number 39, probably intermediate and energy metabolism
YFR049W	mitochondrial ribosomal small subunit
YFR050C	20S proteasome
YFR050C	Complex Number 102, probably protein synthesis turnover
YFR050C	Complex Number 110, probably protein synthesis turnover
YFR050C	Complex Number 111, probably protein synthesis turnover
YFR050C	Complex Number 170, probably signalling
YFR051C	Complex Number 108, probably protein synthesis turnover
YFR051C	Complex Number 338
YFR051C	Complex Number 69
YFR051C	Complex Number 79, probably membrane biogenesis and traffic
YFR051C	COPI
YFR052W	19/22S regulator
YFR052W	Complex Number 110, probably protein synthesis turnover
YFR052W	Complex Number 111, probably protein synthesis turnover
YFR052W	Complex Number 156
YFR052W	Complex Number 165
YFR052W	Complex Number 202, probably transcription/DNA maintanance/chromatin structure
YFR052W	Complex Number 204, probably transcription/DNA maintanance/chromatin structure
YFR052W	Complex Number 313
YFR052W	Complex Number 318
YFR052W	Complex Number 320
YFR052W	Complex Number 41, probably intermediate and energy metabolism
YFR052W	Complex Number 50, 19S Proteosome (17)
YFR052W	Complex Number 504
YFR052W	Complex Number 505
YGL001C	Complex Number 10, probably cell cycle
YGL003C	Complex Number 52
YGL004C	Complex Number 320
YGL004C	Complex Number 468
YGL005C	Golgi transport complex
YGL005C	Kinesin-related motorproteins
YGL006W	Complex Number 457
YGL008C	Complex Number 152
YGL008C	Complex Number 336
YGL008C	Complex Number 360
YGL008C	Complex Number 445

YGL008C	Complex Number 458
YGL008C	Complex Number 542
YGL008C	H+-ATPase, plasma mebrane
YGL009C	Complex Number 56, probably intermediate and energy metabolism
YGL011C	20S proteasome
YGL011C	Complex Number 102, probably protein synthesis turnover
YGL011C	Complex Number 110, probably protein synthesis turnover
YGL011C	Complex Number 111, probably protein synthesis turnover
YGL011C	Complex Number 125, probably protein/RNA transport
YGL011C	Complex Number 144, probably RNA metabolism
YGL011C	Complex Number 170, probably signalling
YGL011C	Complex Number 238
YGL011C	Complex Number 60, 20S Proteosome (13)
YGL016W	Complex Number 13
YGL016W	Complex Number 316
YGL016W	Complex Number 4
YGL017W	Complex Number 334
YGL019W	Casein kinase II
YGL019W	Complex Number 101
YGL019W	Complex Number 124, probably protein/RNA transport
YGL019W	Complex Number 144, probably RNA metabolism
YGL019W	Complex Number 166, probably signalling
YGL019W	Complex Number 181, probably transcription/DNA maintanance/chromatin structure
YGL019W	Complex Number 189, probably transcription/DNA maintanance/chromatin structure
YGL019W	Complex Number 201, probably transcription/DNA maintanance/chromatin structure
YGL019W	Complex Number 204, probably transcription/DNA maintanance/chromatin structure
YGL019W	Complex Number 205
YGL019W	Complex Number 208, probably transcription/DNA maintanance/chromatin structure
YGL019W	Complex Number 375
YGL019W	Complex Number 398
YGL019W	Complex Number 458
YGL019W	Complex Number 47, Casein Kinase II (4)
YGL019W	Complex Number 493
YGL019W	Complex Number 56
YGL019W	Complex Number 78, probably membrane biogenesis and traffic
YGL020C	Complex Number 442
YGL022W	Complex Number 44, probably intermediate and energy metabolism
YGL022W	Oligosaccharyltransferase
YGL023C	Complex Number 520
YGL023C	Complex Number 94
YGL025C	Complex Number 209, probably transcription/DNA maintanance/chromatin structure
YGL025C	Kornberg's mediator (SRB) complex
YGL026C	Complex Number 103
YGL026C	Complex Number 111

YGL026C	Complex Number 316
YGL026C	Complex Number 327
YGL030W	cytoplasmic ribosomal large subunit
YGL031C	cytoplasmic ribosomal large subunit
YGL035C	Complex Number 185
YGL036W	Complex Number 107, probably protein synthesis turnover
YGL036W	Complex Number 13, probably cell cycle
YGL036W	Complex Number 149, probably RNA metabolism
YGL043W	Complex Number 37, Dst1 (1)
YGL043W	Complex Number 43, UTP A (8)
YGL044C	Complex Number 148, probably RNA metabolism
YGL044C	pre mRNA3'-end processing factor CFI
YGL048C	19/22S regulator
YGL048C	Complex Number 110, probably protein synthesis turnover
YGL048C	Complex Number 111, probably protein synthesis turnover
YGL048C	Complex Number 149, probably RNA metabolism
YGL048C	Complex Number 195, probably transcription/DNA maintanance/chromatin structure
YGL048C	Complex Number 202, probably transcription/DNA maintanance/chromatin structure
YGL048C	Complex Number 204, probably transcription/DNA maintanance/chromatin structure
YGL048C	Complex Number 231, probably transcription/DNA maintanance/chromatin structure
YGL048C	Complex Number 41, probably intermediate and energy metabolism
YGL048C	Complex Number 50, 19S Proteosome (17)
YGL049C	Complex Number 130, probably RNA metabolism
YGL049C	Complex Number 139, probably RNA metabolism
YGL049C	Complex Number 140, probably RNA metabolism
YGL049C	Complex Number 142, probably RNA metabolism
YGL049C	Complex Number 146, probably RNA metabolism
YGL049C	Complex Number 148, probably RNA metabolism
YGL049C	Complex Number 155, probably RNA metabolism
YGL049C	Complex Number 169
YGL049C	Complex Number 34
YGL049C	Complex Number 344
YGL049C	Complex Number 380
YGL049C	Complex Number 394
YGL049C	Complex Number 517
YGL049C	eIF4F
YGL058W	Complex Number 285
YGL060W	Complex Number 326
YGL061C	Dam1 protein complex
YGL061C	Spindle pole body (SPB)
YGL062W	Complex Number 152
YGL062W	Complex Number 316
YGL062W	Complex Number 35
YGL062W	Complex Number 365

YGL062W	Complex Number 439
YGL064C	Complex Number 108, probably protein synthesis turnover
YGL066W	Complex Number 207, probably transcription/DNA maintanance/chromatin structure
YGL066W	Complex Number 214, probably transcription/DNA maintanance/chromatin structure
YGL066W	SAGA complex
YGL068W	Complex Number 150
YGL068W	Complex Number 22
YGL068W	Complex Number 319
YGL068W	Complex Number 446
YGL068W	Complex Number 507
YGL068W	mitochondrial ribosomal large subunit
YGL070C	Complex Number 210, probably transcription/DNA maintanance/chromatin structure
YGL070C	Complex Number 545
YGL070C	RNA polymerase II
YGL076C	cytoplasmic ribosomal large subunit
YGL078C	Complex Number 15, Nop1 (1)
YGL078C	Complex Number 4, Dbp3/Bmh1/Nsr1 (3)
YGL078C	Complex Number 5, Gar1/Cbf5 (6)
YGL078C	Complex Number 6, Nop58/Sik1 (3)
YGL081W	Complex Number 469
YGL087C	Complex Number 190
YGL087C	Complex Number 191
YGL087C	Complex Number 409
YGL087C	Complex Number 410
YGL090W	Complex Number 159
YGL090W	Complex Number 160
YGL090W	DNA ligase IV
YGL091C	Complex Number 225, probably transcription/DNA maintanance/chromatin structure
YGL092W	Complex Number 124, probably protein/RNA transport
YGL092W	Complex Number 209
YGL092W	Complex Number 340
YGL092W	NUP84 complex
YGL093W	SPB components
YGL094C	Complex Number 153, probably RNA metabolism
YGL095C	Complex Number 71, probably membrane biogenesis and traffic
YGL097W	Complex Number 120
YGL099W	Complex Number 107, probably protein synthesis turnover
YGL099W	Complex Number 149, probably RNA metabolism
YGL099W	Complex Number 68, Lsg1 (1)
YGL099W	Complex Number 86, probably protein synthesis turnover
YGL100W	Complex Number 124, probably protein/RNA transport
YGL100W	Complex Number 210
YGL100W	Complex Number 340
YGL100W	Complex Number 447

YGL100W	Complex Number 44, probably intermediate and energy metabolism
YGL100W	Complex Number 79, probably membrane biogenesis and traffic
YGL100W	Complex Number 82
YGL100W	NUP84 complex
YGL103W	cytoplasmic ribosomal large subunit
YGL104C	Complex Number 56
YGL105W	Complex Number 146
YGL105W	Complex Number 1, Met-glut tRNA synthetase (3)
YGL105W	Complex Number 258
YGL105W	Complex Number 33
YGL105W	Complex Number 341
YGL105W	Complex Number 4
YGL106W	Complex Number 134, probably RNA metabolism
YGL106W	Complex Number 17, probably cell polarity and structure
YGL106W	Complex Number 345
YGL106W	Complex Number 62
YGL111W	Complex Number 115
YGL111W	Complex Number 132
YGL111W	Complex Number 149, probably RNA metabolism
YGL111W	Complex Number 161
YGL111W	Complex Number 205
YGL111W	Complex Number 520
YGL111W	Complex Number 83, Trm1/Nsa1 (2)
YGL111W	Complex Number 94
YGL112C	Complex Number 112
YGL112C	Complex Number 148, probably RNA metabolism
YGL112C	Complex Number 196, probably transcription/DNA maintanance/chromatin structure
YGL112C	Complex Number 207, probably transcription/DNA maintanance/chromatin structure
YGL112C	Complex Number 209, probably transcription/DNA maintanance/chromatin structure
YGL112C	Complex Number 214, probably transcription/DNA maintanance/chromatin structure
YGL112C	SAGA complex
YGL112C	SAGA-like complex (SLIK)
YGL112C	TAFIIs
YGL113W	Pre-replication complex (pre-RC)
YGL115W	Complex Number 166, probably signalling
YGL115W	Complex Number 289
YGL115W	Complex Number 336
YGL115W	Complex Number 348
YGL115W	Complex Number 367
YGL115W	Complex Number 368
YGL115W	Complex Number 37
YGL115W	Complex Number 399
YGL115W	Complex Number 43
YGL115W	SNF1 complex

YGL116W	Complex Number 39
YGL117W	Complex Number 165
YGL120C	Complex Number 108, probably protein synthesis turnover
YGL120C	Complex Number 125, probably protein/RNA transport
YGL120C	Complex Number 135, probably RNA metabolism
YGL120C	Complex Number 138, probably RNA metabolism
YGL120C	Complex Number 14
YGL120C	Complex Number 143, probably RNA metabolism
YGL120C	Complex Number 144, probably RNA metabolism
YGL120C	Complex Number 145, probably RNA metabolism
YGL120C	Complex Number 146, probably RNA metabolism
YGL120C	Complex Number 147, probably RNA metabolism
YGL120C	Complex Number 22
YGL120C	Complex Number 446
YGL120C	Complex Number 507
YGL120C	Complex Number 520
YGL120C	Complex Number 55, probably intermediate and energy metabolism
YGL120C	Complex Number 94
YGL120C	mRNA splicing
YGL121C	Complex Number 16
YGL121C	Complex Number 182
YGL121C	Complex Number 215
YGL121C	Complex Number 233
YGL122C	Complex Number 123, probably protein/RNA transport
YGL122C	Complex Number 146
YGL122C	Complex Number 69, Nab2/Kap104 (2)
YGL123W	cytoplasmic ribosomal small subunit
YGL128C	Complex Number 138, probably RNA metabolism
YGL128C	Complex Number 143, probably RNA metabolism
YGL128C	Complex Number 147, probably RNA metabolism
YGL128C	mRNA splicing
YGL129C	Complex Number 104, probably protein synthesis turnover
YGL129C	mitochondrial ribosomal small subunit
YGL130W	Complex Number 101
YGL130W	Complex Number 135, probably RNA metabolism
YGL130W	mRNA guanylyl transferase (capping complex)
YGL131C	Complex Number 34, probably intermediate and energy metabolism
YGL131C	Complex Number 470
YGL133W	Complex Number 178, probably transcription/DNA maintanance/chromatin structure
YGL133W	Complex Number 192, probably transcription/DNA maintanance/chromatin structure
YGL133W	Complex Number 200, probably transcription/DNA maintanance/chromatin structure
YGL133W	Complex Number 203, probably transcription/DNA maintanance/chromatin structure
YGL133W	Complex Number 222, probably transcription/DNA maintanance/chromatin structure
YGL134W	Pho85p complexes

YGL135W	cytoplasmic ribosomal large subunit
YGL137W	Complex Number 103, probably protein synthesis turnover
YGL137W	Complex Number 108, probably protein synthesis turnover
YGL137W	Complex Number 134
YGL137W	Complex Number 182
YGL137W	Complex Number 211, probably transcription/DNA maintanance/chromatin structure
YGL137W	Complex Number 240
YGL137W	Complex Number 275
YGL137W	Complex Number 283
YGL137W	Complex Number 284
YGL137W	Complex Number 316
YGL137W	Complex Number 338
YGL137W	Complex Number 402
YGL137W	Complex Number 41, probably intermediate and energy metabolism
YGL137W	Complex Number 49
YGL137W	Complex Number 498
YGL137W	Complex Number 69
YGL137W	Complex Number 77
YGL137W	Complex Number 79, probably membrane biogenesis and traffic
YGL137W	COPI
YGL141W	Complex Number 171
YGL141W	Complex Number 62
YGL142C	Complex Number 83, probably membrane biogenesis and traffic
YGL146C	Complex Number 161
YGL146C	Complex Number 321
YGL147C	cytoplasmic ribosomal large subunit
YGL150C	Complex Number 102
YGL150C	Complex Number 203, probably transcription/DNA maintanance/chromatin structure
YGL151W	Complex Number 209, probably transcription/DNA maintanance/chromatin structure
YGL151W	Complex Number 33, Mediator (8)
YGL151W	Complex Number 49
YGL151W	Kornberg's mediator (SRB) complex
YGL154C	Complex Number 57, probably intermediate and energy metabolism
YGL154C	L-aminopadipate-semialdehyde dehydrogenase
YGL155W	Geranylgeranyltransferase I (GGTase I)
YGL156W	Complex Number 154
YGL156W	Complex Number 213, probably transcription/DNA maintanance/chromatin structure
YGL158W	Complex Number 289
YGL162W	Complex Number 139, probably RNA metabolism
YGL162W	Complex Number 142, probably RNA metabolism
YGL162W	Complex Number 143, probably RNA metabolism
YGL162W	Complex Number 145, probably RNA metabolism
YGL162W	Complex Number 146, probably RNA metabolism
YGL162W	Complex Number 147, probably RNA metabolism

YGL162W	Complex Number 155, probably RNA metabolism
YGL162W	Complex Number 200, probably transcription/DNA maintanance/chromatin structure
YGL162W	Complex Number 208, probably transcription/DNA maintanance/chromatin structure
YGL162W	Complex Number 376
YGL163C	Complex Number 281
YGL163C	other DNA repair complexes
YGL171W	Complex Number 125, probably protein/RNA transport
YGL171W	Complex Number 144, probably RNA metabolism
YGL171W	Complex Number 521
YGL172W	NSP1 complex
YGL173C	Complex Number 14, U6-specific snRNP core (14)
YGL173C	Complex Number 15, Nop1 (1)
YGL173C	Complex Number 16, Ded1 (1)
YGL174W	Complex Number 143
YGL174W	Complex Number 21
YGL174W	Complex Number 506
YGL179C	Complex Number 399
YGL179C	Complex Number 68
YGL187C	Complex Number 47, probably intermediate and energy metabolism
YGL187C	Cytochrome c oxidase (complex IV)
YGL189C	cytoplasmic ribosomal small subunit
YGL190C	Complex Number 170, probably signalling
YGL190C	Complex Number 203
YGL190C	Complex Number 204, probably transcription/DNA maintanance/chromatin structure
YGL190C	Complex Number 233
YGL190C	Complex Number 48
YGL191W	Cytochrome c oxidase (complex IV)
YGL192W	Complex Number 70
YGL194C	Complex Number 101, probably protein synthesis turnover
YGL194C	Complex Number 170, probably signalling
YGL195W	Complex Number 118, probably protein/RNA transport
YGL195W	Complex Number 123, probably protein/RNA transport
YGL195W	Complex Number 125, probably protein/RNA transport
YGL195W	Complex Number 129, probably protein/RNA transport
YGL195W	Complex Number 134
YGL195W	Complex Number 134, probably RNA metabolism
YGL195W	Complex Number 144, probably RNA metabolism
YGL195W	Complex Number 149, probably RNA metabolism
YGL195W	Complex Number 158
YGL195W	Complex Number 171, probably signalling
YGL195W	Complex Number 172, probably signalling
YGL195W	Complex Number 17, probably cell polarity and structure
YGL195W	Complex Number 192, probably transcription/DNA maintanance/chromatin structure
YGL195W	Complex Number 196, probably transcription/DNA maintanance/chromatin structure

YGL195W	Complex Number 211, probably transcription/DNA maintanance/chromatin structure
YGL195W	Complex Number 231, probably transcription/DNA maintanance/chromatin structure
YGL195W	Complex Number 412
YGL195W	Complex Number 445
YGL195W	Complex Number 68
YGL195W	Complex Number 70, probably membrane biogenesis and traffic
YGL195W	Complex Number 82, probably membrane biogenesis and traffic
YGL195W	Complex Number 8, probably cell cycle
YGL197W	Complex Number 102, probably protein synthesis turnover
YGL197W	Complex Number 132
YGL197W	Complex Number 133
YGL197W	Complex Number 163, probably signalling
YGL197W	Complex Number 1, probably cell cycle
YGL200C	Complex Number 318
YGL200C	Complex Number 69, probably membrane biogenesis and traffic
YGL200C	COPII
YGL201C	Complex Number 10, probably cell cycle
YGL201C	Pre-replication complex (pre-RC)
YGL201C	Replication complex
YGL205W	Complex Number 447
YGL206C	Clathrin
YGL206C	Complex Number 105, probably protein synthesis turnover
YGL206C	Complex Number 18, probably cell polarity and structure
YGL206C	Complex Number 68, probably membrane biogenesis and traffic
YGL206C	Complex Number 82, probably membrane biogenesis and traffic
YGL207W	Complex Number 11, probably cell cycle
YGL207W	Complex Number 135, probably RNA metabolism
YGL207W	Complex Number 137
YGL207W	Complex Number 189, probably transcription/DNA maintanance/chromatin structure
YGL207W	Complex Number 201, probably transcription/DNA maintanance/chromatin structure
YGL207W	Complex Number 396
YGL207W	Complex Number 451
YGL207W	Complex Number 46, SPT16 (8)
YGL207W	Complex Number 47, Casein Kinase II (4)
YGL207W	Complex Number 534
YGL207W	Complex Number 56
YGL208W	Complex Number 166, probably signalling
YGL208W	Complex Number 348
YGL208W	Complex Number 367
YGL208W	SNF1 complex
YGL210W	Complex Number 113
YGL212W	Vam3/Vam7 vacuolar t-SNARE complex
YGL213C	Complex Number 355
YGL216W	Complex Number 117

YGL216W	Complex Number 21
YGL216W	Kinesin-related motorproteins
YGL220W	Complex Number 471
YGL223C	Golgi transport complex
YGL226C-A	Oligosaccharyltransferase
YGL227W	Complex Number 427
YGL227W	Complex Number 437
YGL233W	Exocyst complex
YGL234W	Complex Number 163
YGL234W	Complex Number 200, probably transcription/DNA maintanance/chromatin structure
YGL234W	Complex Number 298
YGL234W	Complex Number 302
YGL234W	Complex Number 4
YGL234W	Complex Number 402
YGL237C	CCAAT-binding factor complex
YGL237C	Complex Number 123
YGL237C	Complex Number 217, probably transcription/DNA maintanance/chromatin structure
YGL238W	Complex Number 70, probably membrane biogenesis and traffic
YGL240W	Anaphase promoting complex (APC)
YGL240W	Complex Number 3, probably cell cycle
YGL241W	Complex Number 137
YGL241W	Complex Number 196, probably transcription/DNA maintanance/chromatin structure
YGL241W	Complex Number 214, probably transcription/DNA maintanance/chromatin structure
YGL241W	Complex Number 220, probably transcription/DNA maintanance/chromatin structure
YGL241W	Complex Number 224, probably transcription/DNA maintanance/chromatin structure
YGL241W	Complex Number 68
YGL244W	Complex Number 201, probably transcription/DNA maintanance/chromatin structure
YGL244W	Complex Number 324
YGL244W	Complex Number 46, SPT16 (8)
YGL244W	Complex Number 5, Gar1/Cbf5 (6)
YGL245W	Complex Number 103, probably protein synthesis turnover
YGL245W	Complex Number 118
YGL245W	Complex Number 152
YGL245W	Complex Number 170, probably signalling
YGL245W	Complex Number 176
YGL245W	Complex Number 195, probably transcription/DNA maintanance/chromatin structure
YGL245W	Complex Number 1, Met-glut tRNA synthetase (3)
YGL245W	Complex Number 301
YGL245W	Complex Number 316
YGL245W	Complex Number 341
YGL245W	Complex Number 35
YGL245W	Complex Number 388
YGL245W	Complex Number 4
YGL245W	Complex Number 41, probably intermediate and energy metabolism

YGL245W	Complex Number 520
YGL245W	Complex Number 94
YGL246C	Complex Number 157, probably RNA metabolism
YGL246C	Complex Number 9, probably cell cycle
YGL249W	Complex Number 144, probably RNA metabolism
YGL249W	Synaptonemal complex (SC)
YGL251C	Complex Number 34, probably intermediate and energy metabolism
YGL252C	Complex Number 125, probably protein/RNA transport
YGL252C	Complex Number 144, probably RNA metabolism
YGL252C	Complex Number 161, probably signalling
YGL253W	Hexokinase 2
YGL256W	Complex Number 115
YGL256W	Complex Number 146
YGL256W	Complex Number 267
YGL256W	Complex Number 36
YGL256W	Complex Number 379
YGL256W	Complex Number 88
YGL256W	Complex Number 90
YGR002C	Complex Number 382
YGR005C	Complex Number 146
YGR005C	Complex Number 210, probably transcription/DNA maintanance/chromatin structure
YGR005C	Complex Number 24, RNA Polymerase II (12)
YGR005C	TFIIF
YGR006W	Complex Number 147, probably RNA metabolism
YGR006W	mRNA splicing
YGR013W	Complex Number 139, probably RNA metabolism
YGR013W	Complex Number 145, probably RNA metabolism
YGR013W	Complex Number 146, probably RNA metabolism
YGR013W	Complex Number 147, probably RNA metabolism
YGR013W	Complex Number 35, U1 snRNP (7)
YGR013W	Complex Number 77, probably membrane biogenesis and traffic
YGR016W	Complex Number 440
YGR016W	Complex Number 540
YGR017W	Complex Number 101
YGR020C	Complex Number 45, probably intermediate and energy metabolism
YGR020C	H+-transporting ATPase, vacuolar
YGR027C	cytoplasmic ribosomal small subunit
YGR030C	Complex Number 154, probably RNA metabolism
YGR030C	RNase MRP
YGR030C	RNase P
YGR032W	1,3-beta-D-glucan synthase
YGR033C	Complex Number 228
YGR034W	cytoplasmic ribosomal large subunit
YGR040W	Complex Number 152

YGR040W	Complex Number 153
YGR040W	Complex Number 83
YGR040W	STE5-MAPK complex
YGR041W	Complex Number 455
YGR047C	Complex Number 147
YGR047C	Complex Number 178, probably transcription/DNA maintanance/chromatin structure
YGR047C	Complex Number 34, TFIIIC (4)
YGR047C	TFIIIC
YGR048W	Complex Number 9, probably cell cycle
YGR052W	Complex Number 472
YGR054W	Complex Number 109, probably protein synthesis turnover
YGR054W	Complex Number 14, U6-specific snRNP core (14)
YGR054W	Complex Number 198, probably transcription/DNA maintanance/chromatin structure
YGR054W	Complex Number 473
YGR056W	RSC complex (Remodel the structure of chromatin)
YGR059W	Septin filaments
YGR061C	Complex Number 244
YGR063C	Complex Number 210, probably transcription/DNA maintanance/chromatin structure
YGR066C	Complex Number 388
YGR067C	Complex Number 474
YGR072W	Nonsense-mediated mRNA decay pathway complex
YGR074W	Complex Number 139, probably RNA metabolism
YGR074W	Complex Number 145, probably RNA metabolism
YGR074W	Complex Number 146, probably RNA metabolism
YGR074W	Complex Number 147, probably RNA metabolism
YGR074W	mRNA splicing
YGR075C	Complex Number 138, probably RNA metabolism
YGR075C	Complex Number 147, probably RNA metabolism
YGR075C	mRNA splicing
YGR076C	Complex Number 108, probably protein synthesis turnover
YGR076C	mitochondrial ribosomal large subunit
YGR078C	Complex Number 7, probably cell cycle
YGR078C	Gim complexes
YGR080W	Actin-associated proteins
YGR081C	Complex Number 102, probably protein synthesis turnover
YGR081C	Complex Number 109, probably protein synthesis turnover
YGR081C	Complex Number 144, probably RNA metabolism
YGR082W	TOM - transport across the outer membrane
YGR083C	Complex Number 101
YGR083C	Complex Number 105, probably protein synthesis turnover
YGR083C	Complex Number 108
YGR083C	Complex Number 109
YGR083C	Complex Number 111
YGR083C	Complex Number 380

YGR083C	Complex Number 52, tIF2 (7)
YGR083C	eIF2B
YGR084C	Complex Number 104, probably protein synthesis turnover
YGR084C	mitochondrial ribosomal small subunit
YGR085C	cytoplasmic ribosomal large subunit
YGR086C	Complex Number 132
YGR086C	Complex Number 227
YGR086C	Complex Number 228
YGR086C	Complex Number 23, probably intermediate and energy metabolism
YGR086C	Complex Number 257
YGR086C	Complex Number 31
YGR086C	Complex Number 338
YGR086C	Complex Number 341
YGR086C	Complex Number 360
YGR086C	Complex Number 449
YGR086C	Complex Number 541
YGR086C	Complex Number 551
YGR086C	Complex Number 91
YGR087C	Complex Number 119
YGR087C	Complex Number 257
YGR087C	Complex Number 360
YGR087C	Complex Number 426
YGR087C	Complex Number 47
YGR087C	Complex Number 49
YGR087C	Complex Number 517
YGR088W	Catalase T
YGR090W	Complex Number 104, probably protein synthesis turnover
YGR090W	Complex Number 109, probably protein synthesis turnover
YGR090W	Complex Number 125, probably protein/RNA transport
YGR090W	Complex Number 141, probably RNA metabolism
YGR090W	Complex Number 144, probably RNA metabolism
YGR090W	Complex Number 149, probably RNA metabolism
YGR090W	Complex Number 150
YGR090W	Complex Number 156, probably RNA metabolism
YGR090W	Complex Number 169
YGR090W	Complex Number 180, probably transcription/DNA maintanance/chromatin structure
YGR090W	Complex Number 189, probably transcription/DNA maintanance/chromatin structure
YGR090W	Complex Number 193, probably transcription/DNA maintanance/chromatin structure
YGR090W	Complex Number 200
YGR090W	Complex Number 278
YGR090W	Complex Number 279
YGR090W	Complex Number 375
YGR090W	Complex Number 47, Casein Kinase II (4)
YGR090W	Complex Number 48, UTP22/RRP7 (2)

YGR090W	Complex Number 493
YGR090W	Complex Number 517
YGR090W	Complex Number 56
YGR090W	Complex Number 91
YGR090W	Complex Number 95
YGR091W	Complex Number 138, probably RNA metabolism
YGR091W	Complex Number 145, probably RNA metabolism
YGR091W	Complex Number 146, probably RNA metabolism
YGR091W	Complex Number 147, probably RNA metabolism
YGR091W	Complex Number 244
YGR091W	mRNA splicing
YGR092W	CCR4 complex
YGR092W	Complex Number 77
YGR094W	Complex Number 207, probably transcription/DNA maintanance/chromatin structure
YGR094W	Complex Number 212, probably transcription/DNA maintanance/chromatin structure
YGR094W	Complex Number 214, probably transcription/DNA maintanance/chromatin structure
YGR094W	Complex Number 37
YGR094W	Complex Number 41, probably intermediate and energy metabolism
YGR094W	Complex Number 62
YGR095C	Complex Number 105, probably protein synthesis turnover
YGR095C	Complex Number 134, probably RNA metabolism
YGR095C	Complex Number 141, probably RNA metabolism
YGR095C	Complex Number 22, Exosome (12)
YGR095C	Exosome complex
YGR098C	Complex Number 11, probably cell cycle
YGR098C	Sister chromatid separation complex
YGR102C	Complex Number 99, probably protein synthesis turnover
YGR103W	Complex Number 107, probably protein synthesis turnover
YGR103W	Complex Number 115
YGR103W	Complex Number 137
YGR103W	Complex Number 140, probably RNA metabolism
YGR103W	Complex Number 149, probably RNA metabolism
YGR103W	Complex Number 161
YGR103W	Complex Number 171
YGR103W	Complex Number 204
YGR103W	Complex Number 205
YGR103W	Complex Number 22
YGR103W	Complex Number 28, Nop7/Erb1/Ytm1 (3)
YGR103W	Complex Number 446
YGR103W	Complex Number 475
YGR103W	Complex Number 507
YGR103W	Complex Number 520
YGR103W	Complex Number 53, Kap95/Srp1 (2)
YGR103W	Complex Number 56

YGR103W	Complex Number 86, probably protein synthesis turnover
YGR103W	Complex Number 89
YGR103W	Complex Number 91
YGR103W	Complex Number 94
YGR104C	Complex Number 209, probably transcription/DNA maintanance/chromatin structure
YGR104C	Complex Number 33, Mediator (8)
YGR104C	Complex Number 53, Kap95/Srp1 (2)
YGR104C	Kornberg's mediator (SRB) complex
YGR108W	Cdc28p complexes
YGR109C	Cdc28p complexes
YGR111W	Complex Number 435
YGR113W	Complex Number 102, probably protein synthesis turnover
YGR113W	Dam1 protein complex
YGR113W	Spindle pole body (SPB)
YGR116W	Complex Number 181, probably transcription/DNA maintanance/chromatin structure
YGR116W	Complex Number 24, RNA Polymerase II (12)
YGR118W	cytoplasmic ribosomal small subunit
YGR119C	NSP1 complex
YGR120C	Golgi transport complex
YGR123C	Complex Number 175, probably signalling
YGR128C	Complex Number 125, probably protein/RNA transport
YGR128C	Complex Number 144, probably RNA metabolism
YGR128C	Complex Number 43, UTP A (8)
YGR128C	Complex Number 7, Dbp7/Rrp5 (4)
YGR128C	rRNA splicing
YGR130C	Complex Number 336
YGR132C	Complex Number 69, probably membrane biogenesis and traffic
YGR134W	Complex Number 211, probably transcription/DNA maintanance/chromatin structure
YGR135W	20S proteasome
YGR135W	Complex Number 102, probably protein synthesis turnover
YGR135W	Complex Number 110, probably protein synthesis turnover
YGR135W	Complex Number 111, probably protein synthesis turnover
YGR135W	Complex Number 125, probably protein/RNA transport
YGR135W	Complex Number 144, probably RNA metabolism
YGR135W	Complex Number 170, probably signalling
YGR135W	Complex Number 238
YGR135W	Complex Number 308
YGR135W	Complex Number 550
YGR135W	Complex Number 60, 20S Proteosome (13)
YGR136W	Complex Number 322
YGR140W	CBF3 protein complex
YGR142W	Complex Number 338
YGR143W	1,6-beta-D-glucan synthase
YGR143W	Complex Number 75, probably membrane biogenesis and traffic

YGR145W	Complex Number 125, probably protein/RNA transport
YGR145W	Complex Number 14
YGR145W	Complex Number 144, probably RNA metabolism
YGR145W	Complex Number 446
YGR145W	Complex Number 56
YGR145W	Complex Number 96, probably protein synthesis turnover
YGR148C	cytoplasmic ribosomal large subunit
YGR150C	Complex Number 104, probably protein synthesis turnover
YGR150C	Complex Number 446
YGR154C	Complex Number 435
YGR155W	Complex Number 123
YGR155W	Complex Number 132
YGR155W	Complex Number 149, probably RNA metabolism
YGR155W	Complex Number 152
YGR155W	Complex Number 229
YGR155W	Complex Number 296
YGR155W	Complex Number 31
YGR155W	Complex Number 313
YGR155W	Complex Number 341
YGR155W	Complex Number 350
YGR155W	Complex Number 375
YGR155W	Complex Number 388
YGR155W	Complex Number 435
YGR155W	Complex Number 469
YGR155W	Complex Number 48, probably intermediate and energy metabolism
YGR156W	Complex Number 125, probably protein/RNA transport
YGR156W	Complex Number 148, probably RNA metabolism
YGR158C	Complex Number 134, probably RNA metabolism
YGR158C	Complex Number 141, probably RNA metabolism
YGR159C	Complex Number 15, Nop1 (1)
YGR159C	Complex Number 16, Ded1 (1)
YGR159C	Complex Number 17, Pab1 (1)
YGR159C	Complex Number 336
YGR159C	Complex Number 4, Dbp3/Bmh1/Nsr1 (3)
YGR159C	Complex Number 52, tIF2 (7)
YGR159C	Complex Number 5, Gar1/Cbf5 (6)
YGR159C	Complex Number 6, Nop58/Sik1 (3)
YGR159C	rRNA splicing
YGR161C	Complex Number 170, probably signalling
YGR161C	Complex Number 232
YGR161C	Complex Number 233
YGR162W	Complex Number 104, probably protein synthesis turnover
YGR162W	Complex Number 106, probably protein synthesis turnover
YGR162W	Complex Number 130, probably RNA metabolism

YGR162W	Complex Number 139, probably RNA metabolism
YGR162W	Complex Number 140, probably RNA metabolism
YGR162W	Complex Number 142, probably RNA metabolism
YGR162W	Complex Number 146, probably RNA metabolism
YGR162W	Complex Number 150
YGR162W	Complex Number 155, probably RNA metabolism
YGR162W	Complex Number 15, Nop1 (1)
YGR162W	Complex Number 169
YGR162W	Complex Number 16, Ded1 (1)
YGR162W	Complex Number 17, Pab1 (1)
YGR162W	Complex Number 19, mRNA cap-binding/eIF4F (8)
YGR162W	Complex Number 1, Met-glut tRNA synthetase (3)
YGR162W	Complex Number 204
YGR162W	Complex Number 240
YGR162W	Complex Number 34
YGR162W	Complex Number 344
YGR162W	Complex Number 375
YGR162W	Complex Number 380
YGR162W	Complex Number 394
YGR162W	Complex Number 446
YGR162W	Complex Number 517
YGR162W	Complex Number 56
YGR162W	Complex Number 85
YGR162W	eIF4E/eIF4G/Pab1p complex
YGR162W	eIF4F
YGR163W	Complex Number 11, probably cell cycle
YGR165W	Complex Number 444
YGR165W	mitochondrial ribosomal small subunit
YGR166W	Complex Number 75, probably membrane biogenesis and traffic
YGR166W	TRAPP (Transport Protein Particle) complex
YGR167W	Clathrin
YGR167W	Complex Number 68, probably membrane biogenesis and traffic
YGR170W	Complex Number 104, probably protein synthesis turnover
YGR170W	Complex Number 204, probably transcription/DNA maintanance/chromatin structure
YGR173W	Complex Number 476
YGR173W	Complex Number 58, Gir Complex (2)
YGR175C	Complex Number 532
YGR179C	Ctf19 protein complex
YGR180C	Complex Number 226, probably transcription/DNA maintanance/chromatin structure
YGR180C	Ribonucleoside-diphosphate reductase
YGR183C	Cytochrome bc1 complex (Ubiquinol-cytochrome c reductase complex, complex III)
YGR184C	Complex Number 182
YGR184C	Complex Number 221
YGR184C	Complex Number 31

YGR184C	Complex Number 424
YGR184C	Complex Number 469
YGR186W	Complex Number 210, probably transcription/DNA maintanance/chromatin structure
YGR186W	Complex Number 24, RNA Polymerase II (12)
YGR186W	Complex Number 95
YGR186W	TFIIF
YGR187C	Complex Number 223
YGR187C	Complex Number 43, probably intermediate and energy metabolism
YGR187C	Complex Number 91, eEF2 (2)
YGR192C	Complex Number 130, probably RNA metabolism
YGR192C	Complex Number 169, probably signalling
YGR192C	Complex Number 170, probably signalling
YGR192C	Complex Number 194, probably transcription/DNA maintanance/chromatin structure
YGR192C	Complex Number 230, probably transcription/DNA maintanance/chromatin structure
YGR192C	Complex Number 59, probably intermediate and energy metabolism
YGR193C	Complex Number 262
YGR193C	Complex Number 42, probably intermediate and energy metabolism
YGR193C	Complex Number 505
YGR193C	Pyruvate dehydrogenase
YGR194C	Complex Number 105, probably protein synthesis turnover
YGR195W	Complex Number 134, probably RNA metabolism
YGR195W	Complex Number 141, probably RNA metabolism
YGR195W	Complex Number 22, Exosome (12)
YGR195W	Exosome complex
YGR196C	Complex Number 101
YGR196C	Complex Number 525
YGR196C	Complex Number 93, probably protein synthesis turnover
YGR198W	Complex Number 446
YGR200C	Complex Number 216, probably transcription/DNA maintanance/chromatin structure
YGR200C	Complex Number 93
YGR202C	Complex Number 338
YGR202C	Complex Number 376
YGR203W	Complex Number 11, probably cell cycle
YGR204W	Complex Number 103
YGR204W	Complex Number 244
YGR204W	Complex Number 86, probably protein synthesis turnover
YGR205W	Complex Number 250
YGR207C	other respiration chain complexes
YGR209C	Complex Number 487
YGR210C	Complex Number 255
YGR214W	cytoplasmic ribosomal small subunit
YGR215W	Complex Number 104, probably protein synthesis turnover
YGR215W	mitochondrial ribosomal small subunit
YGR218W	Complex Number 119, probably protein/RNA transport

YGR218W	Complex Number 123, probably protein/RNA transport
YGR218W	Complex Number 134
YGR218W	Complex Number 23
YGR218W	Complex Number 4
YGR218W	Complex Number 550
YGR220C	Complex Number 108, probably protein synthesis turnover
YGR220C	mitochondrial ribosomal large subunit
YGR222W	Mitochondrial splicing complexes
YGR222W	Mitochondrial translation complexes
YGR223C	Complex Number 477
YGR232W	19/22S regulator
YGR232W	Complex Number 110, probably protein synthesis turnover
YGR232W	Complex Number 320
YGR232W	Complex Number 379
YGR232W	Complex Number 468
YGR233C	Complex Number 167, probably signalling
YGR233C	Complex Number 224
YGR233C	Complex Number 23
YGR233C	Complex Number 509
YGR233C	Complex Number 68
YGR234W	Complex Number 133, probably RNA metabolism
YGR234W	Complex Number 142
YGR234W	Complex Number 195, probably transcription/DNA maintanance/chromatin structure
YGR234W	Complex Number 200
YGR234W	Complex Number 224
YGR234W	Complex Number 388
YGR234W	Complex Number 67
YGR234W	Complex Number 68, probably membrane biogenesis and traffic
YGR234W	Complex Number 8, probably cell cycle
YGR238C	Complex Number 147
YGR238C	Kel1p/Kel2p complex
YGR240C	Complex Number 119
YGR240C	Complex Number 134
YGR240C	Complex Number 148, probably RNA metabolism
YGR240C	Complex Number 170, probably signalling
YGR240C	Complex Number 176, probably signalling
YGR240C	Complex Number 195, probably transcription/DNA maintanance/chromatin structure
YGR240C	Complex Number 19, probably cell polarity and structure
YGR240C	Complex Number 204, probably transcription/DNA maintanance/chromatin structure
YGR240C	Complex Number 222
YGR240C	Complex Number 243
YGR240C	Complex Number 33, probably intermediate and energy metabolism
YGR240C	Complex Number 341
YGR240C	Complex Number 364

YGR240C	Complex Number 388
YGR240C	Complex Number 4
YGR240C	Complex Number 402
YGR240C	Complex Number 485
YGR240C	Complex Number 61, probably intermediate and energy metabolism
YGR240C	Complex Number 79
YGR240C	Complex Number 7, probably cell cycle
YGR240C	Phosphofructokinase
YGR244C	Succinyl-CoA ligase
YGR245C	Complex Number 149, probably RNA metabolism
YGR245C	Complex Number 41, Nap1 (3)
YGR246C	TFIIIB
YGR250C	Complex Number 240
YGR250C	Complex Number 344
YGR252W	ADA complex
YGR252W	ADA complexes
YGR252W	Complex Number 112
YGR252W	Complex Number 207, probably transcription/DNA maintanance/chromatin structure
YGR252W	Complex Number 209, probably transcription/DNA maintanance/chromatin structure
YGR252W	Complex Number 214, probably transcription/DNA maintanance/chromatin structure
YGR252W	SAGA complex
YGR253C	20S proteasome
YGR253C	Complex Number 170, probably signalling
YGR253C	Complex Number 238
YGR253C	Complex Number 60, 20S Proteosome (13)
YGR253C	Complex Number 95
YGR254W	Complex Number 174, probably signalling
YGR254W	Complex Number 193, probably transcription/DNA maintanance/chromatin structure
YGR254W	Complex Number 50, probably intermediate and energy metabolism
YGR256W	Complex Number 202
YGR256W	Complex Number 341
YGR258C	Complex Number 263
YGR258C	NEF3 complex
YGR261C	AP-3 complex
YGR261C	Complex Number 78, probably membrane biogenesis and traffic
YGR262C	Complex Number 23
YGR263C	Complex Number 65
YGR264C	Complex Number 1, Met-glut tRNA synthetase (3)
YGR264C	Complex Number 200
YGR264C	Complex Number 207, probably transcription/DNA maintanance/chromatin structure
YGR264C	Complex Number 214, probably transcription/DNA maintanance/chromatin structure
YGR264C	Complex Number 376
YGR266W	Complex Number 505
YGR267C	Complex Number 119

YGR267C	Complex Number 123
YGR267C	Complex Number 168
YGR267C	Complex Number 360
YGR267C	Complex Number 406
YGR267C	Complex Number 435
YGR267C	Complex Number 504
YGR267C	Complex Number 535
YGR270W	26S proteasome
YGR270W	Complex Number 15
YGR270W	Complex Number 189, probably transcription/DNA maintanance/chromatin structure
YGR270W	Complex Number 279
YGR274C	Complex Number 196, probably transcription/DNA maintanance/chromatin structure
YGR274C	Complex Number 200, probably transcription/DNA maintanance/chromatin structure
YGR274C	Complex Number 207, probably transcription/DNA maintanance/chromatin structure
YGR274C	Complex Number 214, probably transcription/DNA maintanance/chromatin structure
YGR274C	TAFIIs
YGR275W	Complex Number 32, RSC (13)
YGR278W	Complex Number 143, probably RNA metabolism
YGR278W	Complex Number 147, probably RNA metabolism
YGR278W	mRNA splicing
YGR279C	Complex Number 109
YGR280C	Complex Number 478
YGR281W	Complex Number 149, probably RNA metabolism
YGR281W	Complex Number 170, probably signalling
YGR282C	Complex Number 111
YGR282C	Complex Number 146
YGR282C	Complex Number 194, probably transcription/DNA maintanance/chromatin structure
YGR282C	Complex Number 346
YGR282C	Complex Number 353
YGR283C	Complex Number 5, Gar1/Cbf5 (6)
YGR285C	Complex Number 106, probably protein synthesis turnover
YGR296W	Complex Number 347
YHL001W	cytoplasmic ribosomal large subunit
YHL002W	Complex Number 76, probably membrane biogenesis and traffic
YHL004W	Complex Number 104, probably protein synthesis turnover
YHL004W	mitochondrial ribosomal small subunit
YHL007C	Complex Number 257
YHL010C	Complex Number 479
YHL011C	Complex Number 252
YHL011C	Complex Number 308
YHL015W	cytoplasmic ribosomal small subunit
YHL019C	Complex Number 84, probably membrane biogenesis and traffic
YHL019C	other adaptor like proteins
YHL020C	Complex Number 23, probably intermediate and energy metabolism

YHL021C	Complex Number 210, probably transcription/DNA maintanance/chromatin structure
YHL025W	Complex Number 198, probably transcription/DNA maintanance/chromatin structure
YHL025W	SWI/SNF transcription activator complex
YHL030W	Complex Number 102, probably protein synthesis turnover
YHL030W	Complex Number 110, probably protein synthesis turnover
YHL030W	Complex Number 111, probably protein synthesis turnover
YHL030W	Complex Number 123, probably protein/RNA transport
YHL030W	Complex Number 125, probably protein/RNA transport
YHL030W	Complex Number 134
YHL030W	Complex Number 144, probably RNA metabolism
YHL030W	Complex Number 197, probably transcription/DNA maintanance/chromatin structure
YHL030W	Complex Number 202, probably transcription/DNA maintanance/chromatin structure
YHL030W	Complex Number 231, probably transcription/DNA maintanance/chromatin structure
YHL030W	Complex Number 250
YHL030W	Complex Number 316
YHL030W	Complex Number 329
YHL030W	Complex Number 70, probably membrane biogenesis and traffic
YHL031C	v-SNAREs
YHL032C	Complex Number 105, probably protein synthesis turnover
YHL033C	cytoplasmic ribosomal large subunit
YHL034C	Complex Number 103, probably protein synthesis turnover
YHL034C	Complex Number 142, probably RNA metabolism
YHL034C	rRNA splicing
YHL035C	Complex Number 148, probably RNA metabolism
YHL035C	Complex Number 56
YHL038C	Complex Number 446
YHL038C	Mitochondrial processing complexes
YHL039W	Complex Number 337
YHR001W-A	Cytochrome bc1 complex (Ubiquinol-cytochrome c reductase complex, complex III)
YHR001W	Complex Number 26
YHR004C	Complex Number 14, probably cell polarity and structure
YHR004C	Nem1p-Spo7p complex
YHR005C-A	Mitochondrial splicing complexes
YHR005C-A	Tim22p-complex
YHR005C-A	Tim9p/Tim10p-complex
YHR005C	Complex Number 118
YHR005C	Complex Number 378
YHR005C	Pheromone response pathway
YHR006W	tRNA splicing
YHR008C	Complex Number 258
YHR008C	Complex Number 504
YHR009C	Complex Number 324
YHR009C	Complex Number 440
YHR010W	cytoplasmic ribosomal large subunit

YHR011W	Complex Number 144, probably RNA metabolism
YHR011W	Complex Number 23
YHR011W	Complex Number 234
YHR011W	Complex Number 550
YHR012W	Complex Number 66, probably membrane biogenesis and traffic
YHR012W	Vps35/Vps29/Vps26 complex
YHR013C	Complex Number 193, probably transcription/DNA maintanance/chromatin structure
YHR013C	Protein N-acetyltransferase
YHR014W	Complex Number 373
YHR015W	Complex Number 197, probably transcription/DNA maintanance/chromatin structure
YHR016C	Complex Number 1
YHR016C	Complex Number 18, probably cell polarity and structure
YHR018C	Complex Number 338
YHR018C	Complex Number 379
YHR019C	Complex Number 143
YHR019C	Complex Number 244
YHR019C	Complex Number 379
YHR019C	Complex Number 497
YHR019C	Complex Number 82
YHR020W	Complex Number 119, probably protein/RNA transport
YHR020W	Complex Number 123, probably protein/RNA transport
YHR020W	Complex Number 302
YHR020W	Complex Number 4
YHR020W	Complex Number 511
YHR020W	Complex Number 70, probably membrane biogenesis and traffic
YHR021C	cytoplasmic ribosomal small subunit
YHR023W	Actin-associated motorproteins
YHR023W	Complex Number 17, probably cell polarity and structure
YHR023W	Complex Number 193, probably transcription/DNA maintanance/chromatin structure
YHR023W	Complex Number 214, probably transcription/DNA maintanance/chromatin structure
YHR024C	Complex Number 127, probably protein/RNA transport
YHR024C	Complex Number 195
YHR024C	Complex Number 313
YHR024C	Processing peptidase
YHR026W	H+-transporting ATPase, vacuolar
YHR027C	19/22S regulator
YHR027C	Complex Number 104
YHR027C	Complex Number 134
YHR027C	Complex Number 155
YHR027C	Complex Number 156
YHR027C	Complex Number 179
YHR027C	Complex Number 187
YHR027C	Complex Number 213
YHR027C	Complex Number 23

YHR027C	Complex Number 272
YHR027C	Complex Number 318
YHR027C	Complex Number 320
YHR027C	Complex Number 34
YHR027C	Complex Number 4
YHR027C	Complex Number 41
YHR027C	Complex Number 429
YHR027C	Complex Number 468
YHR027C	Complex Number 50, 19S Proteosome (17)
YHR027C	Complex Number 6
YHR027C	Complex Number 79
YHR027C	Complex Number 95
YHR030C	Complex Number 194
YHR030C	Complex Number 360
YHR030C	Complex Number 363
YHR030C	Complex Number 490
YHR030C	Complex Number 82
YHR033W	Complex Number 152
YHR033W	Complex Number 182
YHR033W	Complex Number 23
YHR033W	Complex Number 234
YHR033W	Complex Number 238
YHR033W	Complex Number 257
YHR033W	Complex Number 282
YHR033W	Complex Number 34
YHR033W	Complex Number 346
YHR033W	Complex Number 374
YHR033W	Complex Number 378
YHR033W	Complex Number 388
YHR033W	Complex Number 4
YHR033W	Complex Number 402
YHR033W	Complex Number 447
YHR033W	Complex Number 48
YHR033W	Complex Number 504
YHR033W	Complex Number 95
YHR039C-A	Complex Number 45, probably intermediate and energy metabolism
YHR039C-A	H+-transporting ATPase, vacuolar
YHR041C	Complex Number 145, probably RNA metabolism
YHR041C	Complex Number 146, probably RNA metabolism
YHR041C	Complex Number 147, probably RNA metabolism
YHR041C	Complex Number 209, probably transcription/DNA maintanance/chromatin structure
YHR041C	Complex Number 33, Mediator (8)
YHR041C	Kornberg's mediator (SRB) complex
YHR043C	Complex Number 35

YHR044C	Complex Number 147
YHR044C	Complex Number 35
YHR044C	Complex Number 369
YHR044C	Complex Number 453
YHR046C	Complex Number 544
YHR051W	Complex Number 205
YHR051W	Complex Number 47, probably intermediate and energy metabolism
YHR051W	Cytochrome c oxidase (complex IV)
YHR052W	Complex Number 115
YHR052W	Complex Number 123
YHR052W	Complex Number 140, probably RNA metabolism
YHR052W	Complex Number 149, probably RNA metabolism
YHR052W	Complex Number 161
YHR052W	Complex Number 204
YHR052W	Complex Number 22
YHR052W	Complex Number 317
YHR052W	Complex Number 39, Cic1 (1)
YHR052W	Complex Number 446
YHR052W	Complex Number 480
YHR052W	Complex Number 507
YHR052W	Complex Number 520
YHR052W	Complex Number 521
YHR052W	Complex Number 552
YHR052W	Complex Number 56
YHR052W	Complex Number 6
YHR052W	Complex Number 7, Dbp7/Rrp5 (4)
YHR052W	Complex Number 82
YHR052W	Complex Number 91
YHR052W	Complex Number 94
YHR058C	Complex Number 209, probably transcription/DNA maintanance/chromatin structure
YHR058C	Kornberg's mediator (SRB) complex
YHR059W	Complex Number 104, probably protein synthesis turnover
YHR059W	Complex Number 22
YHR059W	Complex Number 507
YHR060W	Complex Number 35
YHR060W	ER assembly complex
YHR062C	Complex Number 154, probably RNA metabolism
YHR062C	Complex Number 78, Rnase P (3)
YHR062C	RNase MRP
YHR062C	RNase P
YHR064C	Complex Number 103
YHR064C	Complex Number 106, probably protein synthesis turnover
YHR064C	Complex Number 143
YHR064C	Complex Number 199, probably transcription/DNA maintanance/chromatin structure

YHR064C	Complex Number 244
YHR064C	Complex Number 37
YHR064C	Complex Number 379
YHR064C	Complex Number 82
YHR064C	Complex Number 9
YHR065C	Complex Number 504
YHR065C	rRNA splicing
YHR066W	Complex Number 149, probably RNA metabolism
YHR066W	Complex Number 375
YHR066W	Complex Number 56
YHR066W	Complex Number 82
YHR069C	Complex Number 127, probably protein/RNA transport
YHR069C	Complex Number 134, probably RNA metabolism
YHR069C	Complex Number 141, probably RNA metabolism
YHR069C	Complex Number 22, Exosome (12)
YHR069C	Complex Number 376
YHR069C	Exosome complex
YHR073W	Complex Number 211
YHR074W	Complex Number 71
YHR075C	Complex Number 232
YHR075C	Complex Number 233
YHR075C	mitochondrial ribosomal small subunit
YHR076W	Complex Number 246
YHR076W	Complex Number 267
YHR076W	Complex Number 4
YHR076W	Complex Number 88
YHR077C	Nonsense-mediated mRNA decay pathway complex
YHR081W	Complex Number 141, probably RNA metabolism
YHR081W	Complex Number 22, Exosome (12)
YHR081W	RNA processing complexes
YHR082C	Complex Number 151
YHR084W	Complex Number 152
YHR084W	Complex Number 153
YHR084W	Complex Number 234
YHR084W	Complex Number 386
YHR085W	Complex Number 149, probably RNA metabolism
YHR085W	Complex Number 67, Ipi (3)
YHR086W	Complex Number 139, probably RNA metabolism
YHR086W	Complex Number 146, probably RNA metabolism
YHR086W	Complex Number 376
YHR087W	Complex Number 319
YHR088W	Complex Number 149, probably RNA metabolism
YHR088W	Complex Number 552
YHR089C	Complex Number 140, probably RNA metabolism

YHR089C	Complex Number 146
YHR089C	Complex Number 165
YHR089C	Complex Number 16, Ded1 (1)
YHR089C	Complex Number 205
YHR089C	Complex Number 42, Ydr365c (1)
YHR089C	Complex Number 5, Gar1/Cbf5 (6)
YHR089C	rRNA splicing
YHR096C	Complex Number 353
YHR098C	Complex Number 132
YHR098C	Complex Number 133
YHR099W	ADA complex
YHR099W	Complex Number 112
YHR099W	Complex Number 191, probably transcription/DNA maintanance/chromatin structure
YHR099W	Complex Number 334
YHR099W	Complex Number 35, U1 snRNP (7)
YHR099W	Complex Number 376
YHR099W	Complex Number 382
YHR099W	Complex Number 446
YHR099W	NuA4 complex
YHR099W	SAGA complex
YHR099W	SAGA-like complex (SLIK)
YHR102W	Complex Number 139
YHR102W	Complex Number 360
YHR102W	Complex Number 539
YHR105W	Complex Number 481
YHR107C	Complex Number 33
YHR107C	Complex Number 34
YHR107C	Complex Number 35
YHR107C	Complex Number 42
YHR107C	Complex Number 4, probably cell cycle
YHR107C	Septin filaments
YHR108W	Complex Number 82, probably membrane biogenesis and traffic
YHR112C	Complex Number 316
YHR112C	Complex Number 435
YHR114W	Complex Number 155
YHR114W	Complex Number 156
YHR114W	Complex Number 18, probably cell polarity and structure
YHR115C	Complex Number 482
YHR115C	Complex Number 532
YHR117W	TOM - transport across the outer membrane
YHR118C	Complex Number 10, probably cell cycle
YHR118C	Post-replication complex (Origin recognition complex=ORC)
YHR118C	Pre-replication complex (pre-RC)
YHR118C	Replication complex

YHR118C	Replication initiation complex
YHR119W	Complex Number 199, probably transcription/DNA maintanance/chromatin structure
YHR119W	Complex Number 342
YHR119W	Complex Number 429
YHR120W	Complex Number 195
YHR121W	Complex Number 133, probably RNA metabolism
YHR121W	Complex Number 135, probably RNA metabolism
YHR122W	Complex Number 212, probably transcription/DNA maintanance/chromatin structure
YHR122W	Complex Number 450
YHR128W	Complex Number 37
YHR129C	Dynactin complex
YHR129C	SPB associated proteins
YHR135C	Casein kinase I
YHR135C	Complex Number 435
YHR135C	Complex Number 436
YHR137W	Complex Number 409
YHR137W	Complex Number 410
YHR141C	cytoplasmic ribosomal large subunit
YHR143W-A	Complex Number 200
YHR143W-A	RNA polymerase I
YHR143W-A	RNA polymerase II
YHR143W-A	RNA polymerase III
YHR147C	Complex Number 108, probably protein synthesis turnover
YHR147C	Complex Number 209, probably transcription/DNA maintanance/chromatin structure
YHR147C	mitochondrial ribosomal large subunit
YHR148W	Complex Number 109, probably protein synthesis turnover
YHR148W	Complex Number 125, probably protein/RNA transport
YHR148W	Complex Number 144, probably RNA metabolism
YHR149C	Complex Number 19
YHR152W	Complex Number 372
YHR154W	Complex Number 514
YHR154W	Complex Number 515
YHR156C	Complex Number 147, probably RNA metabolism
YHR156C	Kinesin-related motorproteins
YHR158C	Complex Number 115
YHR158C	Complex Number 12, probably cell cycle
YHR158C	Complex Number 147
YHR158C	Complex Number 170, probably signalling
YHR158C	Complex Number 381
YHR158C	Kel1p/Kel2p complex
YHR164C	Complex Number 202, probably transcription/DNA maintanance/chromatin structure
YHR164C	Complex Number 212, probably transcription/DNA maintanance/chromatin structure
YHR164C	DNA helicases
YHR165C	Complex Number 13, U4/U6.U5 tri-snRNP (2)

YHR165C	Complex Number 147, probably RNA metabolism
YHR165C	Complex Number 369
YHR165C	mRNA splicing
YHR166C	Anaphase promoting complex (APC)
YHR166C	Complex Number 3, probably cell cycle
YHR166C	Complex Number 40
YHR167W	Complex Number 106
YHR167W	Complex Number 31, TREX (5)
YHR169W	Complex Number 125, probably protein/RNA transport
YHR169W	Complex Number 144, probably RNA metabolism
YHR169W	Complex Number 79
YHR170W	Complex Number 149, probably RNA metabolism
YHR170W	Complex Number 179
YHR170W	Complex Number 205
YHR170W	Complex Number 22
YHR170W	Complex Number 319
YHR170W	Complex Number 388
YHR170W	Complex Number 497
YHR170W	Complex Number 507
YHR170W	Complex Number 82
YHR170W	Complex Number 85
YHR171W	Complex Number 310
YHR172W	Complex Number 7, probably cell cycle
YHR172W	gamma-tubulin complex
YHR174W	Complex Number 100, probably protein synthesis turnover
YHR174W	Complex Number 148, probably RNA metabolism
YHR174W	Complex Number 209, probably transcription/DNA maintanance/chromatin structure
YHR174W	Complex Number 229, probably transcription/DNA maintanance/chromatin structure
YHR174W	Complex Number 27, probably intermediate and energy metabolism
YHR174W	Complex Number 34, probably intermediate and energy metabolism
YHR174W	Complex Number 78, probably membrane biogenesis and traffic
YHR179W	Actin-associated proteins
YHR179W	Complex Number 132
YHR179W	Complex Number 22
YHR179W	Complex Number 283
YHR179W	Complex Number 341
YHR179W	Complex Number 360
YHR179W	Complex Number 435
YHR179W	Complex Number 504
YHR179W	Complex Number 507
YHR179W	Complex Number 510
YHR179W	Complex Number 517
YHR179W	Complex Number 525
YHR179W	Complex Number 536

YHR179W	Complex Number 541
YHR179W	Complex Number 79
YHR183W	Complex Number 117
YHR183W	Complex Number 125
YHR183W	Complex Number 182
YHR183W	Complex Number 202
YHR183W	Complex Number 261
YHR183W	Complex Number 316
YHR183W	Complex Number 341
YHR183W	Complex Number 360
YHR183W	Complex Number 375
YHR183W	Complex Number 435
YHR183W	Complex Number 485
YHR183W	Complex Number 520
YHR183W	Complex Number 94
YHR186C	Complex Number 151
YHR186C	Complex Number 336
YHR186C	Complex Number 483
YHR187W	Complex Number 216, probably transcription/DNA maintanance/chromatin structure
YHR188C	Complex Number 484
YHR191C	Complex Number 205, probably transcription/DNA maintanance/chromatin structure
YHR193C	Complex Number 132
YHR193C	Complex Number 360
YHR193C	Complex Number 520
YHR193C	Complex Number 78
YHR193C	Complex Number 94
YHR193C	NAC complex
YHR196W	Complex Number 125, probably protein/RNA transport
YHR196W	Complex Number 144, probably RNA metabolism
YHR196W	Complex Number 43, UTP A (8)
YHR196W	Complex Number 485
YHR196W	Complex Number 5, Gar1/Cbf5 (6)
YHR196W	rRNA splicing
YHR197W	Complex Number 104, probably protein synthesis turnover
YHR197W	Complex Number 149, probably RNA metabolism
YHR197W	Complex Number 22
YHR197W	Complex Number 486
YHR197W	Complex Number 507
YHR197W	Complex Number 67, Ipi (3)
YHR199C	Complex Number 158
YHR199C	Complex Number 487
YHR199C	Complex Number 517
YHR200W	19/22S regulator
YHR200W	Complex Number 110, probably protein synthesis turnover

YHR200W	Complex Number 111, probably protein synthesis turnover
YHR200W	Complex Number 152
YHR200W	Complex Number 202, probably transcription/DNA maintanance/chromatin structure
YHR200W	Complex Number 231, probably transcription/DNA maintanance/chromatin structure
YHR200W	Complex Number 23, probably intermediate and energy metabolism
YHR200W	Complex Number 320
YHR200W	Complex Number 4
YHR200W	Complex Number 41, probably intermediate and energy metabolism
YHR200W	Complex Number 468
YHR200W	Complex Number 50, 19S Proteosome (17)
YHR201C	Complex Number 4
YHR203C	Complex Number 18, Ydr117c/Rps4b (2)
YHR203C	cytoplasmic ribosomal small subunit
YHR204W	Complex Number 96, probably protein synthesis turnover
YHR208W	Complex Number 22, probably intermediate and energy metabolism
YHR208W	Complex Number 244
YHR208W	Complex Number 36
YHR214W-A	Complex Number 402
YHR215W	Complex Number 24, probably intermediate and energy metabolism
YHR216W	Complex Number 169
YHR216W	Complex Number 204
YHR216W	Complex Number 23
YHR216W	Complex Number 375
YHR216W	Complex Number 517
YHR216W	Complex Number 57, Imd (3)
YIL004C	v-SNAREs
YIL007C	Complex Number 488
YIL009C-A	Complex Number 102, probably protein synthesis turnover
YIL009C-A	Telomerase
YIL017C	Complex Number 427
YIL017C	Complex Number 437
YIL018W	cytoplasmic ribosomal large subunit
YIL021W	Complex Number 210, probably transcription/DNA maintanance/chromatin structure
YIL021W	Complex Number 24, RNA Polymerase II (12)
YIL021W	Complex Number 284
YIL021W	Complex Number 322
YIL021W	Complex Number 545
YIL021W	Complex Number 95
YIL021W	RNA polymerase II
YIL022W	TIM - transport across the inner membrane
YIL026C	Complex Number 172
YIL026C	Complex Number 46
YIL026C	Complex Number 8, probably cell cycle
YIL026C	Sister chromatid cohesion complex

YIL028W	Complex Number 17
YIL033C	cAMP-dependent protein kinase
YIL033C	Complex Number 160, probably signalling
YIL033C	Complex Number 180
YIL033C	Complex Number 256
YIL033C	Complex Number 369
YIL033C	Complex Number 400
YIL033C	Complex Number 401
YIL033C	Complex Number 402
YIL033C	Complex Number 435
YIL033C	Complex Number 548
YIL033C	Complex Number 56
YIL033C	Complex Number 95
YIL034C	Actin-associated proteins
YIL034C	Complex Number 16, probably cell polarity and structure
YIL034C	Complex Number 466
YIL035C	Casein kinase II
YIL035C	Complex Number 101
YIL035C	Complex Number 124, probably protein/RNA transport
YIL035C	Complex Number 144, probably RNA metabolism
YIL035C	Complex Number 148, probably RNA metabolism
YIL035C	Complex Number 166, probably signalling
YIL035C	Complex Number 181, probably transcription/DNA maintanance/chromatin structure
YIL035C	Complex Number 189, probably transcription/DNA maintanance/chromatin structure
YIL035C	Complex Number 201, probably transcription/DNA maintanance/chromatin structure
YIL035C	Complex Number 208, probably transcription/DNA maintanance/chromatin structure
YIL035C	Complex Number 375
YIL035C	Complex Number 458
YIL035C	Complex Number 46, SPT16 (8)
YIL035C	Complex Number 47, Casein Kinase II (4)
YIL035C	Complex Number 48, UTP22/RRP7 (2)
YIL035C	Complex Number 493
YIL035C	Complex Number 56
YIL035C	Complex Number 57
YIL035C	Complex Number 87
YIL035C	Complex Number 88
YIL035C	Complex Number 88, probably protein synthesis turnover
YIL037C	Complex Number 341
YIL038C	CCR4 complex
YIL038C	Complex Number 211, probably transcription/DNA maintanance/chromatin structure
YIL038C	NOT complex
YIL046W	Complex Number 181
YIL046W	SCF-MET30 complex
YIL050W	Complex Number 224

YIL052C	cytoplasmic ribosomal large subunit
YIL053W	Complex Number 123
YIL053W	Complex Number 267
YIL053W	Complex Number 272
YIL053W	Complex Number 283
YIL053W	Complex Number 296
YIL055C	Complex Number 15
YIL061C	Complex Number 139, probably RNA metabolism
YIL061C	Complex Number 142, probably RNA metabolism
YIL061C	Complex Number 145, probably RNA metabolism
YIL061C	Complex Number 146, probably RNA metabolism
YIL061C	Complex Number 147, probably RNA metabolism
YIL061C	Complex Number 35, U1 snRNP (7)
YIL061C	Complex Number 369
YIL061C	mRNA splicing
YIL062C	Arp2p/Arp3p complex
YIL062C	Complex Number 13
YIL062C	Complex Number 19, probably cell polarity and structure
YIL063C	Complex Number 448
YIL063C	Complex Number 550
YIL066C	Complex Number 30
YIL066C	Complex Number 313
YIL066C	Ribonucleoside-diphosphate reductase
YIL068C	Complex Number 221
YIL068C	Complex Number 257
YIL068C	Complex Number 81, probably membrane biogenesis and traffic
YIL068C	Exocyst complex
YIL069C	cytoplasmic ribosomal small subunit
YIL070C	Complex Number 123
YIL070C	Complex Number 134, probably RNA metabolism
YIL070C	Complex Number 141
YIL070C	Complex Number 22
YIL070C	Complex Number 226, probably transcription/DNA maintanance/chromatin structure
YIL070C	Complex Number 267
YIL070C	Complex Number 274
YIL070C	Complex Number 369
YIL070C	Complex Number 475
YIL070C	Complex Number 507
YIL070C	Complex Number 534
YIL070C	Complex Number 544
YIL070C	Complex Number 87
YIL070C	Complex Number 88
YIL071C	Complex Number 87, probably protein synthesis turnover
YIL072W	Synaptonemal complex (SC)

YIL074C	Complex Number 24, probably intermediate and energy metabolism
YIL075C	19/22S regulator
YIL075C	Complex Number 50, 19S Proteosome (17)
YIL076W	Complex Number 29
YIL076W	Complex Number 338
YIL076W	Complex Number 69
YIL076W	Complex Number 79, probably membrane biogenesis and traffic
YIL076W	COPI
YIL077C	Complex Number 209, probably transcription/DNA maintanance/chromatin structure
YIL078W	Complex Number 103
YIL078W	Complex Number 341
YIL078W	Complex Number 81, Ths1 (1)
YIL079C	Complex Number 404
YIL079C	Complex Number 489
YIL084C	Complex Number 208, probably transcription/DNA maintanance/chromatin structure
YIL093C	Complex Number 104, probably protein synthesis turnover
YIL093C	Complex Number 22
YIL093C	Complex Number 507
YIL093C	mitochondrial ribosomal small subunit
YIL094C	Complex Number 102, probably protein synthesis turnover
YIL094C	Complex Number 10, probably cell cycle
YIL094C	Complex Number 123, probably protein/RNA transport
YIL094C	Complex Number 187
YIL094C	Complex Number 201, probably transcription/DNA maintanance/chromatin structure
YIL094C	Complex Number 224, probably transcription/DNA maintanance/chromatin structure
YIL094C	Complex Number 328
YIL094C	Complex Number 35, probably intermediate and energy metabolism
YIL094C	Complex Number 360
YIL094C	Complex Number 41, probably intermediate and energy metabolism
YIL094C	Complex Number 504
YIL094C	Complex Number 52, probably intermediate and energy metabolism
YIL094C	Complex Number 68, probably membrane biogenesis and traffic
YIL094C	Complex Number 82, probably membrane biogenesis and traffic
YIL095W	Complex Number 239
YIL097W	Complex Number 427
YIL097W	Complex Number 437
YIL104C	Complex Number 28
YIL105C	Complex Number 173
YIL106W	CCR4 complex
YIL106W	Complex Number 77
YIL106W	Complex Number 78
YIL108W	Complex Number 174
YIL109C	COPII
YIL112W	Complex Number 101, probably protein synthesis turnover

YIL112W	Complex Number 170, probably signalling
YIL112W	Complex Number 347
YIL113W	Complex Number 490
YIL115C	Nuclear pore complex (NPC)
YIL124W	Complex Number 146
YIL124W	Complex Number 338
YIL125W	2-oxoglutarate dehydrogenase
YIL125W	Complex Number 161
YIL125W	Complex Number 312
YIL125W	Complex Number 318
YIL125W	Complex Number 388
YIL125W	Complex Number 39, probably intermediate and energy metabolism
YIL125W	Complex Number 4
YIL125W	Complex Number 439
YIL125W	Complex Number 466
YIL125W	Complex Number 485
YIL125W	Complex Number 79
YIL126W	Complex Number 200, probably transcription/DNA maintanance/chromatin structure
YIL126W	Complex Number 220, probably transcription/DNA maintanance/chromatin structure
YIL126W	Complex Number 32, RSC (13)
YIL126W	Complex Number 528
YIL126W	RSC complex (Remodel the structure of chromatin)
YIL128W	Complex Number 109
YIL128W	Complex Number 13
YIL128W	Complex Number 174
YIL128W	Complex Number 180
YIL128W	Complex Number 231, probably transcription/DNA maintanance/chromatin structure
YIL128W	Complex Number 271
YIL128W	Complex Number 272
YIL128W	Complex Number 316
YIL128W	Complex Number 328
YIL128W	Complex Number 371
YIL128W	Complex Number 4
YIL128W	Complex Number 453
YIL129C	Complex Number 197, probably transcription/DNA maintanance/chromatin structure
YIL129C	Complex Number 225, probably transcription/DNA maintanance/chromatin structure
YIL129C	Complex Number 29
YIL131C	Complex Number 101
YIL133C	cytoplasmic ribosomal large subunit
YIL136W	Complex Number 338
YIL137C	Complex Number 552
YIL138C	Actin-associated proteins
YIL139C	DNA polymerase zeta
YIL142W	Chaperonine containing T-complex TRiC (TCP RING Complex)

YIL142W	Complex Number 140
YIL142W	Complex Number 150
YIL142W	Complex Number 152
YIL142W	Complex Number 1, probably cell cycle
YIL142W	Complex Number 221
YIL142W	Complex Number 223
YIL142W	Complex Number 234
YIL142W	Complex Number 254
YIL142W	Complex Number 26
YIL142W	Complex Number 269
YIL142W	Complex Number 270
YIL142W	Complex Number 32
YIL142W	Complex Number 338
YIL142W	Complex Number 370
YIL142W	Complex Number 378
YIL142W	Complex Number 385
YIL142W	Complex Number 39
YIL142W	Complex Number 420
YIL142W	Complex Number 427
YIL142W	Complex Number 443
YIL142W	Complex Number 48
YIL142W	Complex Number 52
YIL142W	Complex Number 93, probably protein synthesis turnover
YIL143C	Complex Number 266
YIL143C	NEF3 complex
YIL143C	RNA polymerase I
YIL144W	Ndc80 protein complex
YIL144W	SPB associated proteins
YIL147C	Complex Number 359
YIL148W	cytoplasmic ribosomal large subunit
YIL149C	Complex Number 103
YIL149C	Complex Number 392
YIL155C	other respiration chain complexes
YIL156W	Complex Number 329
YIL159W	Complex Number 17
YIL177C	Complex Number 29, probably intermediate and energy metabolism
YIR001C	Complex Number 130, probably RNA metabolism
YIR001C	Complex Number 155, probably RNA metabolism
YIR001C	Complex Number 344
YIR002C	Complex Number 101
YIR002C	Complex Number 169
YIR002C	Complex Number 202, probably transcription/DNA maintanance/chromatin structure
YIR002C	Complex Number 212, probably transcription/DNA maintanance/chromatin structure
YIR003W	Complex Number 16, probably cell polarity and structure

YIR003W	Complex Number 22
YIR003W	Complex Number 507
YIR005W	Complex Number 143
YIR006C	Actin-associated proteins
YIR008C	Complex Number 211, probably transcription/DNA maintanance/chromatin structure
YIR008C	Complex Number 228, probably transcription/DNA maintanance/chromatin structure
YIR008C	DNA polymerase alpha (I) - primase complex
YIR009W	Complex Number 145, probably RNA metabolism
YIR009W	Complex Number 146, probably RNA metabolism
YIR009W	Complex Number 147, probably RNA metabolism
YIR012W	Complex Number 156
YIR015W	RNase P
YIR017C	Cbf1/Met4/Met28 complex
YIR017C	Met4/Met28/Met31 complex
YIR017C	Met4/Met28/Met32 complex
YIR021W	Mitochondrial splicing complexes
YIR022W	Signal peptidase
YIR031C	Complex Number 6, probably cell cycle
YIR032C	Complex Number 29, probably intermediate and energy metabolism
YIR033W	Complex Number 103, probably protein synthesis turnover
YIR034C	Complex Number 168
YIR034C	Complex Number 435
YIR035C	Complex Number 141, probably RNA metabolism
YJL001W	20S proteasome
YJL001W	Complex Number 102, probably protein synthesis turnover
YJL001W	Complex Number 110, probably protein synthesis turnover
YJL001W	Complex Number 111, probably protein synthesis turnover
YJL001W	Complex Number 238
YJL001W	Complex Number 363
YJL001W	Complex Number 60, 20S Proteosome (13)
YJL002C	Complex Number 44, probably intermediate and energy metabolism
YJL002C	Oligosaccharyltransferase
YJL005W	Complex Number 108, probably protein synthesis turnover
YJL005W	Complex Number 149, probably RNA metabolism
YJL005W	Complex Number 167, probably signalling
YJL005W	Complex Number 341
YJL005W	Complex Number 68
YJL005W	Complex Number 76
YJL005W	Complex Number 77
YJL006C	Complex Number 215, probably transcription/DNA maintanance/chromatin structure
YJL006C	Ctk1p complex
YJL006C	TFIIK (CTD kinase)
YJL008C	Chaperonine containing T-complex TRiC (TCP RING Complex)
YJL008C	Complex Number 195, probably transcription/DNA maintanance/chromatin structure

YJL008C	Complex Number 19, probably cell polarity and structure
YJL008C	Complex Number 41, probably intermediate and energy metabolism
YJL008C	Complex Number 8, probably cell cycle
YJL011C	Complex Number 213, probably transcription/DNA maintanance/chromatin structure
YJL011C	Complex Number 23, RNA Polymerase III (12)
YJL011C	Complex Number 25, RNA polymerase I (7)
YJL013C	Complex Number 39
YJL014W	Chaperonine containing T-complex TRiC (TCP RING Complex)
YJL014W	Complex Number 150
YJL014W	Complex Number 221
YJL014W	Complex Number 234
YJL014W	Complex Number 254
YJL014W	Complex Number 26
YJL014W	Complex Number 265
YJL014W	Complex Number 370
YJL014W	Complex Number 378
YJL014W	Complex Number 385
YJL014W	Complex Number 39
YJL014W	Complex Number 420
YJL014W	Complex Number 427
YJL014W	Complex Number 443
YJL014W	Complex Number 48
YJL014W	Complex Number 504
YJL014W	Complex Number 52
YJL016W	Complex Number 198, probably transcription/DNA maintanance/chromatin structure
YJL020C	Complex Number 134
YJL020C	Complex Number 491
YJL024C	AP-3 complex
YJL024C	Complex Number 78, probably membrane biogenesis and traffic
YJL025W	Core Factor (CF)
YJL026W	Complex Number 200, probably transcription/DNA maintanance/chromatin structure
YJL026W	Complex Number 226, probably transcription/DNA maintanance/chromatin structure
YJL026W	Complex Number 258
YJL026W	Complex Number 338
YJL026W	Complex Number 341
YJL026W	Complex Number 350
YJL026W	Complex Number 353
YJL026W	Complex Number 362
YJL026W	Complex Number 389
YJL026W	Complex Number 421
YJL026W	Complex Number 425
YJL026W	Complex Number 459
YJL026W	Complex Number 76
YJL026W	Complex Number 79

YJL026W	Ribonucleoside-diphosphate reductase
YJL029C	Complex Number 148, probably RNA metabolism
YJL029C	Complex Number 74, probably membrane biogenesis and traffic
YJL031C	Complex Number 159, probably signalling
YJL031C	Geranylgeranyltransferase II (GGTase II)
YJL033W	Complex Number 125, probably protein/RNA transport
YJL033W	Complex Number 144, probably RNA metabolism
YJL033W	Complex Number 155, probably RNA metabolism
YJL033W	Complex Number 15, Nop1 (1)
YJL033W	Complex Number 17, Pab1 (1)
YJL033W	Complex Number 43, UTP A (8)
YJL033W	Complex Number 8, Hca4 (1)
YJL034W	ER protein-translocation complex associated proteins
YJL041W	Complex Number 126, probably protein/RNA transport
YJL041W	NSP1 complex
YJL041W	NSP1-NIC96 complex
YJL041W	NSP1-NUP82 complex
YJL042W	Complex Number 115
YJL042W	Complex Number 184
YJL042W	Tubulin-associated proteins
YJL044C	Complex Number 121
YJL045W	Complex Number 467
YJL047C	Complex Number 134
YJL047C	Complex Number 514
YJL047C	Complex Number 515
YJL050W	Complex Number 118, probably protein/RNA transport
YJL050W	Complex Number 129, probably protein/RNA transport
YJL050W	Complex Number 16, Ded1 (1)
YJL050W	Complex Number 17, Pab1 (1)
YJL050W	Complex Number 404
YJL050W	Complex Number 57, Imd (3)
YJL050W	Complex Number 96, probably protein synthesis turnover
YJL050W	Complex Number 9, Mtr4 (3)
YJL053W	Vps35/Vps29/Vps26 complex
YJL054W	Tim22p-complex
YJL060W	Complex Number 15, probably cell polarity and structure
YJL061W	Complex Number 126, probably protein/RNA transport
YJL061W	Complex Number 71, Nup82 (1)
YJL061W	NSP1-NUP82 complex
YJL063C	Complex Number 108, probably protein synthesis turnover
YJL063C	Complex Number 147, probably RNA metabolism
YJL063C	mitochondrial ribosomal large subunit
YJL066C	Complex Number 128, probably protein/RNA transport
YJL068C	Complex Number 492

YJL068C	Complex Number 70
YJL069C	Complex Number 125, probably protein/RNA transport
YJL069C	Complex Number 144, probably RNA metabolism
YJL069C	Complex Number 493
YJL069C	Complex Number 511
YJL069C	Complex Number 87, UTP B (6)
YJL072C	0
YJL074C	Complex Number 172
YJL074C	Complex Number 278
YJL074C	Complex Number 361
YJL074C	Complex Number 41, probably intermediate and energy metabolism
YJL074C	Complex Number 46
YJL074C	Complex Number 469
YJL074C	Complex Number 8, probably cell cycle
YJL074C	Sister chromatid cohesion complex
YJL076W	Complex Number 101
YJL076W	Complex Number 194, probably transcription/DNA maintanance/chromatin structure
YJL076W	Complex Number 336
YJL080C	Complex Number 106, probably protein synthesis turnover
YJL080C	Complex Number 139, probably RNA metabolism
YJL080C	Complex Number 244
YJL080C	Complex Number 341
YJL080C	Complex Number 474
YJL080C	Complex Number 88, probably protein synthesis turnover
YJL081C	Complex Number 123
YJL081C	Complex Number 191, probably transcription/DNA maintanance/chromatin structure
YJL081C	Complex Number 203, probably transcription/DNA maintanance/chromatin structure
YJL081C	Complex Number 334
YJL081C	Complex Number 382
YJL085W	Complex Number 513
YJL085W	Exocyst complex
YJL087C	Complex Number 56
YJL087C	Complex Number 87
YJL087C	tRNA splicing
YJL088W	Ornithine carbamoyltransferase
YJL089W	SNF1 complex
YJL090C	Complex Number 85
YJL090C	Complex Number 86
YJL090C	Pre-replication complex (pre-RC)
YJL090C	Replication complex
YJL092W	Complex Number 130
YJL092W	Complex Number 131
YJL095W	Complex Number 151
YJL095W	Complex Number 187

YJL095W	Complex Number 360
YJL095W	Complex Number 96, probably protein synthesis turnover
YJL096W	mitochondrial ribosomal large subunit
YJL098W	Complex Number 12, Sit4 (6)
YJL098W	Complex Number 132
YJL098W	Complex Number 16, Ded1 (1)
YJL098W	Complex Number 1, probably cell cycle
YJL098W	Complex Number 334
YJL098W	Complex Number 353
YJL098W	Complex Number 369
YJL106W	Complex Number 140
YJL107C	Complex Number 394
YJL109C	Complex Number 125, probably protein/RNA transport
YJL109C	Complex Number 134, probably RNA metabolism
YJL109C	Complex Number 140, probably RNA metabolism
YJL109C	Complex Number 141, probably RNA metabolism
YJL109C	Complex Number 144, probably RNA metabolism
YJL109C	Complex Number 149, probably RNA metabolism
YJL109C	Complex Number 150
YJL109C	Complex Number 43, UTP A (8)
YJL109C	Complex Number 446
YJL109C	Complex Number 485
YJL109C	Complex Number 493
YJL109C	Complex Number 521
YJL109C	Complex Number 82
YJL109C	Complex Number 96
YJL109C	rRNA splicing
YJL111W	Chaperonine containing T-complex TRiC (TCP RING Complex)
YJL111W	Complex Number 92, probably protein synthesis turnover
YJL115W	Complex Number 226, probably transcription/DNA maintanance/chromatin structure
YJL115W	Complex Number 278
YJL115W	Complex Number 279
YJL117W	Complex Number 209
YJL117W	Complex Number 504
YJL117W	Complex Number 69
YJL122W	Complex Number 22
YJL122W	Complex Number 38, Arx1 Complex (5)
YJL122W	Complex Number 39, Cic1 (1)
YJL122W	Complex Number 433
YJL122W	Complex Number 507
YJL122W	Complex Number 7, Dbp7/Rrp5 (4)
YJL122W	Complex Number 89
YJL124C	Complex Number 138, probably RNA metabolism
YJL124C	Complex Number 147, probably RNA metabolism

YJL124C	Complex Number 14, U6-specific snRNP core (14)
YJL124C	Complex Number 163
YJL124C	Complex Number 214
YJL124C	Complex Number 215
YJL128C	Complex Number 216
YJL133W	Mitochondrial splicing complexes
YJL136C	cytoplasmic ribosomal small subunit
YJL138C	Complex Number 104
YJL138C	Complex Number 123
YJL138C	Complex Number 142
YJL138C	Complex Number 142, probably RNA metabolism
YJL138C	Complex Number 19, mRNA cap-binding/eIF4F (8)
YJL138C	Complex Number 212, probably transcription/DNA maintanance/chromatin structure
YJL138C	Complex Number 222
YJL138C	Complex Number 258
YJL138C	Complex Number 267
YJL138C	Complex Number 29, probably intermediate and energy metabolism
YJL138C	Complex Number 341
YJL138C	Complex Number 360
YJL138C	Complex Number 373
YJL138C	Complex Number 380
YJL138C	Complex Number 388
YJL138C	Complex Number 394
YJL138C	Complex Number 4
YJL138C	Complex Number 41, probably intermediate and energy metabolism
YJL138C	Complex Number 435
YJL138C	Complex Number 439
YJL138C	Complex Number 446
YJL138C	Complex Number 497
YJL138C	Complex Number 79
YJL138C	eIF4A
YJL140W	Complex Number 210, probably transcription/DNA maintanance/chromatin structure
YJL140W	Complex Number 24, RNA Polymerase II (12)
YJL140W	RNA polymerase II
YJL141C	Complex Number 424
YJL141C	Complex Number 82
YJL141C	Complex Number 91
YJL143W	Tim17p-complex
YJL148W	Complex Number 23, RNA Polymerase III (12)
YJL148W	Complex Number 24, RNA Polymerase II (12)
YJL148W	Complex Number 25, RNA polymerase I (7)
YJL148W	RNA polymerase I
YJL149W	Complex Number 494
YJL154C	Complex Number 552

YJL154C	Complex Number 66, probably membrane biogenesis and traffic
YJL154C	Vps35/Vps29/Vps26 complex
YJL156C	Complex Number 405
YJL157C	Complex Number 257
YJL157C	Complex Number 99
YJL164C	cAMP-dependent protein kinase
YJL164C	Complex Number 400
YJL164C	Complex Number 401
YJL164C	Complex Number 402
YJL164C	Complex Number 548
YJL165C	Complex Number 122
YJL166W	Cytochrome bc1 complex (Ubiquinol-cytochrome c reductase complex, complex III)
YJL167W	Complex Number 244
YJL167W	Complex Number 267
YJL167W	Complex Number 379
YJL167W	Complex Number 423
YJL167W	Complex Number 5
YJL168C	Complex Number 180, probably transcription/DNA maintanance/chromatin structure
YJL173C	Complex Number 212, probably transcription/DNA maintanance/chromatin structure
YJL173C	Complex Number 296
YJL173C	Replication factor A complex
YJL176C	Complex Number 108, probably protein synthesis turnover
YJL176C	Complex Number 109, probably protein synthesis turnover
YJL176C	Complex Number 14, probably cell polarity and structure
YJL176C	Complex Number 198, probably transcription/DNA maintanance/chromatin structure
YJL176C	SWI/SNF transcription activator complex
YJL177W	cytoplasmic ribosomal large subunit
YJL187C	Complex Number 381
YJL189W	cytoplasmic ribosomal large subunit
YJL190C	cytoplasmic ribosomal small subunit
YJL191W	cytoplasmic ribosomal small subunit
YJL194W	Complex Number 110, probably protein synthesis turnover
YJL194W	Complex Number 111, probably protein synthesis turnover
YJL194W	Complex Number 185, probably transcription/DNA maintanance/chromatin structure
YJL194W	Pre-replication complex (pre-RC)
YJL197W	Complex Number 101
YJL201W	Complex Number 18, probably cell polarity and structure
YJL203W	Complex Number 143, probably RNA metabolism
YJL203W	Complex Number 145, probably RNA metabolism
YJL203W	Complex Number 146, probably RNA metabolism
YJL203W	Complex Number 147, probably RNA metabolism
YJL203W	Prp19p-associated complex
YJL207C	Complex Number 435
YJL207C	Complex Number 458

YJL208C	Complex Number 186, probably transcription/DNA maintanance/chromatin structure
YJL209W	Mitochondrial processing complexes
YJL222W	Complex Number 204, probably transcription/DNA maintanance/chromatin structure
YJR002W	Complex Number 109, probably protein synthesis turnover
YJR002W	Complex Number 125, probably protein/RNA transport
YJR002W	Complex Number 144, probably RNA metabolism
YJR005W	AP-2 complex
YJR005W	Complex Number 78, probably membrane biogenesis and traffic
YJR006W	DNA polymerase delta (III)
YJR006W	Replication complex
YJR007W	Complex Number 101
YJR007W	Complex Number 105, probably protein synthesis turnover
YJR007W	Complex Number 107, probably protein synthesis turnover
YJR007W	Complex Number 115
YJR007W	Complex Number 380
YJR007W	eIF2
YJR009C	Complex Number 170, probably signalling
YJR010C-A	Signal peptidase
YJR014W	Complex Number 137, probably RNA metabolism
YJR016C	Complex Number 304
YJR017C	Complex Number 95
YJR022W	Complex Number 163
YJR022W	Complex Number 165
YJR027W	Complex Number 308
YJR028W	Complex Number 308
YJR029W	Complex Number 13
YJR033C	Complex Number 45, probably intermediate and energy metabolism
YJR035W	Complex Number 267
YJR041C	Complex Number 446
YJR042W	Complex Number 124, probably protein/RNA transport
YJR042W	Complex Number 209
YJR042W	Complex Number 210
YJR042W	Complex Number 340
YJR042W	NUP84 complex
YJR043C	DNA polymerase delta (III)
YJR043C	Replication complex
YJR045C	Complex Number 224, probably transcription/DNA maintanance/chromatin structure
YJR045C	Complex Number 48, probably intermediate and energy metabolism
YJR045C	Complex Number 50, probably intermediate and energy metabolism
YJR045C	Endonuclease SceI, mitochondrial
YJR045C	TIM - transport across the inner membrane
YJR047C	eIF5A
YJR050W	Complex Number 143, probably RNA metabolism
YJR052W	Complex Number 261

YJR052W	Complex Number 286
YJR052W	NEF4 complex
YJR053W	Complex Number 16
YJR053W	Complex Number 388
YJR053W	Complex Number 389
YJR058C	AP-2 complex
YJR058C	Complex Number 78, probably membrane biogenesis and traffic
YJR060W	Cbf1/Met4/Met28 complex
YJR060W	Inner Kinetochor Protein Complex
YJR061W	Complex Number 495
YJR062C	Complex Number 206
YJR063W	Complex Number 213, probably transcription/DNA maintanance/chromatin structure
YJR063W	Complex Number 315
YJR063W	RNA polymerase I
YJR064W	Chaperonine containing T-complex TRiC (TCP RING Complex)
YJR064W	Complex Number 19, probably cell polarity and structure
YJR064W	Complex Number 221
YJR064W	Complex Number 223
YJR064W	Complex Number 243
YJR064W	Complex Number 254
YJR064W	Complex Number 26
YJR064W	Complex Number 370
YJR064W	Complex Number 378
YJR064W	Complex Number 385
YJR064W	Complex Number 39
YJR064W	Complex Number 40, probably intermediate and energy metabolism
YJR064W	Complex Number 48
YJR064W	Complex Number 497
YJR064W	Complex Number 505
YJR065C	Arp2p/Arp3p complex
YJR065C	Complex Number 1
YJR065C	Complex Number 13
YJR065C	Complex Number 19, probably cell polarity and structure
YJR065C	Complex Number 9
YJR068W	Complex Number 174
YJR068W	Complex Number 205, probably transcription/DNA maintanance/chromatin structure
YJR068W	Complex Number 207
YJR068W	Complex Number 264
YJR068W	Complex Number 265
YJR068W	Complex Number 297
YJR068W	Complex Number 298
YJR068W	Complex Number 301
YJR068W	Complex Number 302
YJR068W	Replication factor C complex

YJR070C	Complex Number 32
YJR070C	Complex Number 338
YJR070C	Complex Number 346
YJR070C	Complex Number 459
YJR072C	Complex Number 111
YJR072C	Complex Number 152
YJR072C	Complex Number 187
YJR072C	Complex Number 23
YJR072C	Complex Number 264
YJR072C	Complex Number 353
YJR072C	Complex Number 385
YJR072C	Complex Number 505
YJR072C	Complex Number 77
YJR074W	Complex Number 120
YJR076C	Complex Number 33
YJR076C	Complex Number 34
YJR076C	Complex Number 35
YJR076C	Complex Number 42
YJR076C	Complex Number 4, probably cell cycle
YJR076C	Complex Number 96, probably protein synthesis turnover
YJR076C	Septin filaments
YJR077C	Complex Number 119, probably protein/RNA transport
YJR077C	Complex Number 123, probably protein/RNA transport
YJR077C	Complex Number 230, probably transcription/DNA maintanance/chromatin structure
YJR077C	Complex Number 70, probably membrane biogenesis and traffic
YJR084W	Complex Number 145, probably RNA metabolism
YJR084W	Complex Number 146, probably RNA metabolism
YJR084W	Complex Number 147, probably RNA metabolism
YJR084W	Signal transduction complexes
YJR086W	Pheromone response pathway
YJR089W	Complex Number 49
YJR090C	Complex Number 119
YJR090C	SCF-GRR1 complex
YJR093C	Complex Number 115
YJR093C	Complex Number 125, probably protein/RNA transport
YJR093C	Complex Number 148, probably RNA metabolism
YJR093C	Complex Number 376
YJR093C	pre mRNA polyadenylation factor PFI
YJR094C	Ume6/Ime1 complex
YJR094W-A	cytoplasmic ribosomal large subunit
YJR098C	Complex Number 96, probably protein synthesis turnover
YJR101W	Complex Number 104, probably protein synthesis turnover
YJR101W	mitochondrial ribosomal small subunit
YJR104C	Complex Number 103

YJR104C	Complex Number 224
YJR104C	Complex Number 244
YJR104C	Complex Number 341
YJR104C	Complex Number 435
YJR104C	Complex Number 497
YJR104C	Complex Number 62
YJR104C	Complex Number 90
YJR105W	Complex Number 142
YJR105W	Complex Number 244
YJR105W	Complex Number 353
YJR109C	Arginine-specific carbamoylphosphate synthase
YJR109C	Complex Number 195, probably transcription/DNA maintanance/chromatin structure
YJR109C	Complex Number 298
YJR109C	Complex Number 4
YJR109C	Complex Number 41, probably intermediate and energy metabolism
YJR109C	Complex Number 504
YJR109C	Complex Number 79
YJR110W	Complex Number 496
YJR113C	Complex Number 104, probably protein synthesis turnover
YJR113C	Complex Number 199, probably transcription/DNA maintanance/chromatin structure
YJR113C	mitochondrial ribosomal small subunit
YJR121W	Complex Number 35, probably intermediate and energy metabolism
YJR121W	F0/F1 ATP synthase (complex V)
YJR122W	CCR4 complex
YJR123W	cytoplasmic ribosomal small subunit
YJR127C	Complex Number 93
YJR132W	Complex Number 109
YJR132W	Complex Number 123
YJR132W	Complex Number 134, probably RNA metabolism
YJR132W	Complex Number 158
YJR132W	Complex Number 204, probably transcription/DNA maintanance/chromatin structure
YJR132W	Complex Number 374
YJR132W	Complex Number 445
YJR134C	Complex Number 132
YJR134C	Complex Number 133
YJR135C	Complex Number 221, probably transcription/DNA maintanance/chromatin structure
YJR135C	Ctf3 protein complex
YJR138W	Complex Number 108, probably protein synthesis turnover
YJR138W	Complex Number 155, probably RNA metabolism
YJR138W	Complex Number 170, probably signalling
YJR138W	Complex Number 19, mRNA cap-binding/eIF4F (8)
YJR138W	Complex Number 201, probably transcription/DNA maintanance/chromatin structure
YJR138W	Complex Number 204, probably transcription/DNA maintanance/chromatin structure
YJR138W	Complex Number 29, probably intermediate and energy metabolism

YJR138W	Complex Number 96, probably protein synthesis turnover
YJR139C	Complex Number 329
YJR140C	Complex Number 226, probably transcription/DNA maintanance/chromatin structure
YJR140C	Complex Number 295
YJR140C	Complex Number 365
YJR141W	Complex Number 296
YJR144W	Complex Number 101
YJR144W	Complex Number 201
YJR144W	Complex Number 212, probably transcription/DNA maintanance/chromatin structure
YJR144W	Complex Number 22
YJR144W	Complex Number 245
YJR144W	Complex Number 295
YJR144W	Complex Number 446
YJR144W	Complex Number 507
YJR144W	Complex Number 517
YJR144W	Complex Number 56
YJR145C	cytoplasmic ribosomal small subunit
YJR148W	Complex Number 22, probably intermediate and energy metabolism
YJR152W	Complex Number 250
YJR152W	Complex Number 374
YJR152W	Complex Number 4
YKL003C	Complex Number 104, probably protein synthesis turnover
YKL003C	mitochondrial ribosomal small subunit
YKL006W	cytoplasmic ribosomal large subunit
YKL007W	Actin-associated proteins
YKL007W	Complex Number 16, probably cell polarity and structure
YKL007W	Complex Number 466
YKL009W	Complex Number 146
YKL009W	Complex Number 149, probably RNA metabolism
YKL009W	Complex Number 520
YKL009W	Complex Number 94
YKL010C	Complex Number 138
YKL010C	Complex Number 388
YKL010C	Complex Number 411
YKL010C	Complex Number 6
YKL011C	Complex Number 260
YKL011C	Complex Number 30
YKL012W	Complex Number 139, probably RNA metabolism
YKL012W	Complex Number 145, probably RNA metabolism
YKL012W	Complex Number 146, probably RNA metabolism
YKL012W	Complex Number 147, probably RNA metabolism
YKL012W	Complex Number 35, U1 snRNP (7)
YKL012W	Complex Number 493
YKL012W	mRNA splicing

YKL013C	Arp2p/Arp3p complex
YKL013C	Complex Number 13
YKL013C	Complex Number 19, probably cell polarity and structure
YKL013C	Complex Number 9
YKL014C	Complex Number 140, probably RNA metabolism
YKL014C	Complex Number 149, probably RNA metabolism
YKL014C	Complex Number 446
YKL014C	Complex Number 82
YKL014C	Complex Number 90, Ykl014c (1)
YKL014C	Complex Number 96
YKL016C	Complex Number 37
YKL016C	F0/F1 ATP synthase (complex V)
YKL019W	Complex Number 168, probably signalling
YKL019W	Farnesyltransferase (FTase)
YKL019W	Geranylgeranyltransferase I (GGTase I)
YKL021C	Complex Number 171
YKL022C	Anaphase promoting complex (APC)
YKL022C	Complex Number 200
YKL022C	Complex Number 3, probably cell cycle
YKL022C	Complex Number 423
YKL025C	Complex Number 153, probably RNA metabolism
YKL028W	Complex Number 137, probably RNA metabolism
YKL028W	Complex Number 202, probably transcription/DNA maintanance/chromatin structure
YKL028W	Complex Number 206, probably transcription/DNA maintanance/chromatin structure
YKL028W	TFIIE
YKL029C	Complex Number 338
YKL029C	Complex Number 353
YKL029C	Complex Number 362
YKL041W	Vps4p ATPase complex (Vps protein complex)
YKL042W	SPB components
YKL045W	Complex Number 151
YKL045W	Complex Number 211, probably transcription/DNA maintanance/chromatin structure
YKL045W	Complex Number 228, probably transcription/DNA maintanance/chromatin structure
YKL045W	DNA polymerase alpha (I) - primase complex
YKL048C	Complex Number 92
YKL049C	Inner Kinetochor Protein Complex
YKL052C	Dam1 protein complex
YKL054C	Complex Number 439
YKL054C	Complex Number 504
YKL056C	Complex Number 132
YKL056C	Complex Number 150
YKL056C	Complex Number 182
YKL056C	Complex Number 281
YKL056C	Complex Number 341

YKL056C	Complex Number 35
YKL056C	Complex Number 412
YKL057C	Complex Number 124, probably protein/RNA transport
YKL057C	Complex Number 209
YKL057C	NUP84 complex
YKL058W	Complex Number 182, probably transcription/DNA maintanance/chromatin structure
YKL058W	TFIIA
YKL059C	Complex Number 148, probably RNA metabolism
YKL059C	Complex Number 56, mRNA cleavage/polyadenylation (10)
YKL059C	Nuclear processing complexes
YKL060C	Complex Number 209, probably transcription/DNA maintanance/chromatin structure
YKL060C	Complex Number 213, probably transcription/DNA maintanance/chromatin structure
YKL060C	Complex Number 34, probably intermediate and energy metabolism
YKL062W	Complex Number 442
YKL067W	Complex Number 188, probably transcription/DNA maintanance/chromatin structure
YKL067W	Complex Number 341
YKL067W	Complex Number 388
YKL067W	Complex Number 497
YKL067W	Complex Number 62
YKL068W	Nuclear pore complex (NPC)
YKL073W	Complex Number 182
YKL073W	Complex Number 320
YKL073W	Complex Number 360
YKL073W	Complex Number 535
YKL074C	mRNA splicing
YKL078W	Complex Number 498
YKL078W	mRNA splicing
YKL079W	Actin-associated motorproteins
YKL080W	Complex Number 341
YKL080W	Complex Number 388
YKL080W	Complex Number 45, probably intermediate and energy metabolism
YKL080W	H+-transporting ATPase, vacuolar
YKL081W	Complex Number 103, probably protein synthesis turnover
YKL081W	Complex Number 147
YKL081W	Complex Number 170, probably signalling
YKL081W	Complex Number 181
YKL081W	Complex Number 214, probably transcription/DNA maintanance/chromatin structure
YKL081W	Complex Number 22
YKL081W	Complex Number 267
YKL081W	Complex Number 283
YKL081W	Complex Number 341
YKL081W	Complex Number 350
YKL081W	Complex Number 388
YKL081W	Complex Number 507

YKL081W	Complex Number 64
YKL081W	eEF1
YKL082C	Complex Number 56
YKL085W	Complex Number 114
YKL085W	Complex Number 115
YKL085W	Complex Number 117
YKL085W	Complex Number 132
YKL085W	Complex Number 176
YKL085W	Complex Number 206
YKL085W	Complex Number 22
YKL085W	Complex Number 244
YKL085W	Complex Number 267
YKL085W	Complex Number 27
YKL085W	Complex Number 271
YKL085W	Complex Number 278
YKL085W	Complex Number 281
YKL085W	Complex Number 316
YKL085W	Complex Number 330
YKL085W	Complex Number 39
YKL085W	Complex Number 439
YKL085W	Complex Number 454
YKL085W	Complex Number 476
YKL085W	Complex Number 507
YKL085W	Complex Number 525
YKL085W	Complex Number 536
YKL085W	Complex Number 88
YKL085W	other respiration chain complexes
YKL088W	Complex Number 189, probably transcription/DNA maintanance/chromatin structure
YKL088W	Complex Number 47, Casein Kinase II (4)
YKL088W	Complex Number 48, UTP22/RRP7 (2)
YKL088W	Complex Number 49, Ykl088w (3)
YKL089W	Inner Kinetochor Protein Complex
YKL095W	Complex Number 143, probably RNA metabolism
YKL095W	Complex Number 497
YKL099C	Complex Number 125, probably protein/RNA transport
YKL099C	Complex Number 144, probably RNA metabolism
YKL099C	Complex Number 493
YKL099C	rRNA splicing
YKL101W	Complex Number 125, probably protein/RNA transport
YKL101W	Complex Number 144, probably RNA metabolism
YKL103C	Complex Number 154
YKL103C	Complex Number 221
YKL104C	Complex Number 102, probably protein synthesis turnover
YKL104C	Complex Number 103, probably protein synthesis turnover

YKL104C	Complex Number 110, probably protein synthesis turnover
YKL104C	Complex Number 123, probably protein/RNA transport
YKL104C	Complex Number 13
YKL104C	Complex Number 152
YKL104C	Complex Number 164, probably signalling
YKL104C	Complex Number 195, probably transcription/DNA maintanance/chromatin structure
YKL104C	Complex Number 25, probably intermediate and energy metabolism
YKL104C	Complex Number 316
YKL104C	Complex Number 32, probably intermediate and energy metabolism
YKL104C	Complex Number 35, probably intermediate and energy metabolism
YKL104C	Complex Number 4
YKL104C	Complex Number 41, probably intermediate and energy metabolism
YKL104C	Complex Number 469
YKL104C	Complex Number 499
YKL104C	Complex Number 53
YKL104C	Complex Number 70, probably membrane biogenesis and traffic
YKL104C	Complex Number 85, probably protein synthesis turnover
YKL108W	Complex Number 87
YKL108W	Complex Number 88
YKL109W	CCAAT-binding factor complex
YKL113C	Complex Number 268
YKL113C	Exonucleases
YKL119C	Complex Number 483
YKL119C	Complex Number 63
YKL119C	ER assembly complex
YKL120W	Complex Number 146
YKL122C	Signal recognition particle (SRP)
YKL125W	Complex Number 52, probably intermediate and energy metabolism
YKL127W	Complex Number 284
YKL129C	Actin-associated motorproteins
YKL129C	Complex Number 62
YKL130C	Complex Number 17, probably cell polarity and structure
YKL135C	AP-1 complex
YKL135C	Complex Number 6
YKL135C	Complex Number 84, probably membrane biogenesis and traffic
YKL138C	mitochondrial ribosomal large subunit
YKL139W	Complex Number 215, probably transcription/DNA maintanance/chromatin structure
YKL139W	Complex Number 74
YKL139W	Ctk1p complex
YKL139W	TFIIK (CTD kinase)
YKL140W	Complex Number 133
YKL141W	Succinate dehydrogenase complex (complex II)
YKL142W	Mitochondrial ribosomes
YKL143W	Complex Number 109, probably protein synthesis turnover

YKL143W	Complex Number 132
YKL143W	Complex Number 144, probably RNA metabolism
YKL143W	Complex Number 198, probably transcription/DNA maintanance/chromatin structure
YKL144C	Complex Number 213, probably transcription/DNA maintanance/chromatin structure
YKL144C	Complex Number 23, RNA Polymerase III (12)
YKL144C	Complex Number 24, RNA Polymerase II (12)
YKL144C	Complex Number 25, RNA polymerase I (7)
YKL144C	Complex Number 316
YKL144C	RNA polymerase III
YKL145W	19/22S regulator
YKL145W	Complex Number 110, probably protein synthesis turnover
YKL145W	Complex Number 111, probably protein synthesis turnover
YKL145W	Complex Number 23
YKL145W	Complex Number 318
YKL145W	Complex Number 320
YKL145W	Complex Number 4
YKL145W	Complex Number 468
YKL145W	Complex Number 50, 19S Proteosome (17)
YKL148C	other respiration chain complexes
YKL148C	Succinate dehydrogenase complex (complex II)
YKL150W	Complex Number 37
YKL152C	Complex Number 168, probably signalling
YKL152C	Complex Number 34, probably intermediate and energy metabolism
YKL154W	Complex Number 90, probably protein synthesis turnover
YKL154W	Signal recognition particle receptor (SR)
YKL155C	Complex Number 104, probably protein synthesis turnover
YKL155C	mitochondrial ribosomal small subunit
YKL156W	cytoplasmic ribosomal small subunit
YKL157W	Complex Number 244
YKL161C	Complex Number 499
YKL166C	cAMP-dependent protein kinase
YKL166C	Complex Number 160, probably signalling
YKL166C	Complex Number 227
YKL166C	Complex Number 400
YKL166C	Complex Number 401
YKL166C	Complex Number 402
YKL166C	Complex Number 435
YKL166C	Complex Number 548
YKL167C	Complex Number 108, probably protein synthesis turnover
YKL167C	mitochondrial ribosomal large subunit
YKL168C	Complex Number 495
YKL170W	Complex Number 147, probably RNA metabolism
YKL170W	mitochondrial ribosomal large subunit
YKL172W	Complex Number 149, probably RNA metabolism

YKL172W	Complex Number 204
YKL172W	Complex Number 480
YKL172W	Complex Number 520
YKL172W	Complex Number 91
YKL172W	Complex Number 94
YKL173W	Complex Number 138, probably RNA metabolism
YKL173W	Complex Number 140, probably RNA metabolism
YKL173W	Complex Number 143, probably RNA metabolism
YKL173W	Complex Number 145, probably RNA metabolism
YKL173W	Complex Number 146, probably RNA metabolism
YKL173W	Complex Number 147, probably RNA metabolism
YKL173W	Complex Number 14, U6-specific snRNP core (14)
YKL176C	Complex Number 82
YKL180W	cytoplasmic ribosomal large subunit
YKL182W	Fatty acid synthetase, cytoplasmic
YKL189W	Complex Number 138
YKL189W	Complex Number 139
YKL190W	Calcineurin B
YKL190W	Complex Number 64
YKL190W	Complex Number 66
YKL193C	Complex Number 115
YKL193C	Complex Number 158, probably signalling
YKL193C	Complex Number 237
YKL193C	Complex Number 331
YKL193C	Complex Number 336
YKL193C	Serine/threonine phoshpoprotein phosphatase
YKL195W	Complex Number 1, probably cell cycle
YKL196C	Complex Number 41, probably intermediate and energy metabolism
YKL196C	Complex Number 71, probably membrane biogenesis and traffic
YKL196C	SNAREs
YKL201C	Complex Number 214, probably transcription/DNA maintanance/chromatin structure
YKL204W	Complex Number 338
YKL204W	Complex Number 43
YKL205W	Complex Number 109
YKL205W	Complex Number 412
YKL205W	tRNA splicing
YKL206C	Complex Number 12
YKL206C	Complex Number 238
YKL210W	Complex Number 147
YKL210W	Complex Number 244
YKL210W	Complex Number 283
YKL210W	Complex Number 285
YKL210W	Complex Number 341
YKL210W	Complex Number 406

YKL210W	Complex Number 410
YKL210W	Complex Number 412
YKL210W	Complex Number 435
YKL210W	Complex Number 62
YKL210W	Complex Number 75
YKL211C	Anthranilate synthase
YKL211C	Complex Number 111
YKL211C	Complex Number 13
YKL211C	Complex Number 30, probably intermediate and energy metabolism
YKL211C	Complex Number 69
YKL211C	Complex Number 84
YKL212W	Complex Number 146
YKL213C	Complex Number 9, probably cell cycle
YKL214C	Complex Number 123
YKL214C	Complex Number 139, probably RNA metabolism
YKL215C	Complex Number 500
YKL216W	Complex Number 103
YKL216W	Complex Number 306
YKL218C	Complex Number 316
YKR001C	Complex Number 135, probably RNA metabolism
YKR001C	Complex Number 189, probably transcription/DNA maintanance/chromatin structure
YKR001C	Complex Number 192, probably transcription/DNA maintanance/chromatin structure
YKR001C	Complex Number 196, probably transcription/DNA maintanance/chromatin structure
YKR001C	Complex Number 198, probably transcription/DNA maintanance/chromatin structure
YKR001C	Complex Number 200, probably transcription/DNA maintanance/chromatin structure
YKR001C	Complex Number 203, probably transcription/DNA maintanance/chromatin structure
YKR001C	Complex Number 208, probably transcription/DNA maintanance/chromatin structure
YKR001C	Complex Number 212, probably transcription/DNA maintanance/chromatin structure
YKR001C	Complex Number 214, probably transcription/DNA maintanance/chromatin structure
YKR001C	Complex Number 220, probably transcription/DNA maintanance/chromatin structure
YKR001C	Complex Number 230, probably transcription/DNA maintanance/chromatin structure
YKR001C	Complex Number 424
YKR002W	Complex Number 125, probably protein/RNA transport
YKR002W	Complex Number 148, probably RNA metabolism
YKR002W	Complex Number 376
YKR002W	Complex Number 56, mRNA cleavage/polyadenylation (10)
YKR006C	Complex Number 108, probably protein synthesis turnover
YKR006C	mitochondrial ribosomal large subunit
YKR007W	Complex Number 338
YKR008W	Complex Number 192, probably transcription/DNA maintanance/chromatin structure
YKR008W	Complex Number 203, probably transcription/DNA maintanance/chromatin structure
YKR008W	Complex Number 32, RSC (13)
YKR008W	RSC complex (Remodel the structure of chromatin)
YKR014C	Complex Number 113

YKR014C	Complex Number 418
YKR014C	Complex Number 548
YKR014C	Complex Number 73, probably membrane biogenesis and traffic
YKR017C	Complex Number 479
YKR018C	Complex Number 322
YKR020W	Complex Number 74, probably membrane biogenesis and traffic
YKR024C	Complex Number 10, Mdm20/Nat3 (2)
YKR024C	Complex Number 151
YKR024C	Complex Number 16, Ded1 (1)
YKR024C	Complex Number 204
YKR024C	Complex Number 446
YKR024C	Complex Number 7, Dbp7/Rrp5 (4)
YKR024C	Complex Number 96
YKR025W	Complex Number 213, probably transcription/DNA maintanance/chromatin structure
YKR025W	Complex Number 23, RNA Polymerase III (12)
YKR026C	Complex Number 100
YKR026C	Complex Number 101
YKR026C	Complex Number 105, probably protein synthesis turnover
YKR026C	Complex Number 108
YKR026C	Complex Number 109
YKR026C	Complex Number 111
YKR026C	Complex Number 132
YKR026C	Complex Number 380
YKR026C	eIF2B
YKR028W	Complex Number 123
YKR028W	Complex Number 12, Sit4 (6)
YKR028W	Complex Number 132
YKR028W	Complex Number 142
YKR028W	Complex Number 1, probably cell cycle
YKR028W	Complex Number 353
YKR028W	Complex Number 369
YKR029C	Complex Number 101, probably protein synthesis turnover
YKR029C	Complex Number 170, probably signalling
YKR029C	Complex Number 198
YKR031C	Complex Number 13, probably cell cycle
YKR036C	CCR4 complex
YKR036C	Complex Number 26
YKR037C	Dam1 protein complex
YKR037C	SPB associated proteins
YKR038C	Complex Number 23
YKR046C	Complex Number 111
YKR046C	Complex Number 146
YKR046C	Complex Number 338
YKR046C	Complex Number 374

YKR048C	Complex Number 123
YKR048C	Complex Number 137
YKR048C	Complex Number 149, probably RNA metabolism
YKR048C	Complex Number 152
YKR048C	Complex Number 220, probably transcription/DNA maintanance/chromatin structure
YKR048C	Complex Number 224, probably transcription/DNA maintanance/chromatin structure
YKR048C	Complex Number 232, probably transcription/DNA maintanance/chromatin structure
YKR048C	Complex Number 25
YKR048C	Complex Number 308
YKR048C	Complex Number 404
YKR048C	Complex Number 41, Nap1 (3)
YKR048C	Complex Number 430
YKR048C	Complex Number 497
YKR048C	Complex Number 52
YKR048C	Complex Number 541
YKR051W	Complex Number 505
YKR052C	Mitochondrial splicing complexes
YKR054C	Complex Number 204, probably transcription/DNA maintanance/chromatin structure
YKR054C	Dynein-complex motorproteins
YKR054C	SPB associated proteins
YKR055W	Complex Number 306
YKR057W	cytoplasmic ribosomal small subunit
YKR059W	Complex Number 125, probably protein/RNA transport
YKR059W	Complex Number 131, probably RNA metabolism
YKR059W	Complex Number 132, probably RNA metabolism
YKR059W	Complex Number 144, probably RNA metabolism
YKR059W	Complex Number 209, probably transcription/DNA maintanance/chromatin structure
YKR059W	Complex Number 29, probably intermediate and energy metabolism
YKR059W	Complex Number 41, probably intermediate and energy metabolism
YKR059W	eIF4A
YKR060W	Complex Number 125, probably protein/RNA transport
YKR060W	Complex Number 144, probably RNA metabolism
YKR060W	Complex Number 493
YKR062W	TFIIE
YKR063C	Complex Number 343
YKR067W	Complex Number 338
YKR067W	Complex Number 79, probably membrane biogenesis and traffic
YKR068C	Complex Number 75, probably membrane biogenesis and traffic
YKR068C	TRAPP (Transport Protein Particle) complex
YKR071C	Complex Number 96, probably protein synthesis turnover
YKR072C	Complex Number 49, Ykl088w (3)
YKR079C	Complex Number 186, probably transcription/DNA maintanance/chromatin structure
YKR079C	Complex Number 41, Nap1 (3)
YKR081C	Complex Number 149, probably RNA metabolism

YKR081C	Complex Number 161
YKR081C	Complex Number 200
YKR081C	Complex Number 375
YKR081C	Complex Number 403
YKR081C	Complex Number 446
YKR081C	Complex Number 458
YKR081C	Complex Number 461
YKR081C	Complex Number 475
YKR081C	Complex Number 517
YKR081C	Complex Number 91
YKR081C	Complex Number 96
YKR082W	Complex Number 337
YKR082W	Nuclear pore complex (NPC)
YKR083C	Dam1 protein complex
YKR084C	eEF1
YKR085C	Complex Number 108, probably protein synthesis turnover
YKR085C	mitochondrial ribosomal large subunit
YKR086W	mRNA splicing
YKR091W	Complex Number 6, probably cell cycle
YKR094C	cytoplasmic ribosomal large subunit
YKR095W	Complex Number 209, probably transcription/DNA maintanance/chromatin structure
YKR096W	Complex Number 399
YKR096W	Complex Number 493
YKR101W	Complex Number 350
YLL001W	Complex Number 132
YLL001W	Complex Number 424
YLL004W	Complex Number 10, probably cell cycle
YLL004W	Post-replication complex (Origin recognition complex=ORC)
YLL004W	Pre-replication complex (pre-RC)
YLL004W	Replication complex
YLL004W	Replication initiation complex
YLL008W	Complex Number 140, probably RNA metabolism
YLL008W	Complex Number 149, probably RNA metabolism
YLL008W	Complex Number 204
YLL008W	Complex Number 446
YLL008W	rRNA splicing
YLL010C	Complex Number 247
YLL011W	Complex Number 109, probably protein synthesis turnover
YLL011W	Complex Number 125, probably protein/RNA transport
YLL011W	Complex Number 144, probably RNA metabolism
YLL011W	Complex Number 182
YLL011W	Complex Number 370
YLL013C	Complex Number 13
YLL013C	Complex Number 132

YLL013C	Complex Number 133, probably RNA metabolism
YLL013C	Complex Number 505
YLL018C	Complex Number 146
YLL018C	Complex Number 244
YLL019C	Complex Number 149
YLL019C	Complex Number 534
YLL021W	Complex Number 102, probably protein synthesis turnover
YLL022C	Complex Number 125
YLL022C	Complex Number 204, probably transcription/DNA maintanance/chromatin structure
YLL026W	Complex Number 132
YLL026W	Complex Number 147
YLL026W	Complex Number 206
YLL026W	Complex Number 302
YLL026W	Complex Number 485
YLL026W	Complex Number 500
YLL026W	Complex Number 95
YLL027W	Complex Number 446
YLL029W	Complex Number 471
YLL034C	Complex Number 134
YLL034C	Complex Number 149, probably RNA metabolism
YLL036C	Complex Number 143, probably RNA metabolism
YLL036C	Complex Number 145, probably RNA metabolism
YLL036C	Complex Number 146, probably RNA metabolism
YLL036C	Complex Number 147, probably RNA metabolism
YLL036C	Complex Number 241
YLL036C	Complex Number 497
YLL036C	Complex Number 55, Prp19 (4)
YLL036C	Prp19p-associated complex
YLL039C	Complex Number 119
YLL039C	Complex Number 134
YLL039C	Complex Number 152
YLL039C	Complex Number 181
YLL039C	Complex Number 200
YLL039C	Complex Number 226
YLL039C	Complex Number 256
YLL039C	Complex Number 257
YLL039C	Complex Number 286
YLL039C	Complex Number 329
YLL039C	Complex Number 332
YLL039C	Complex Number 367
YLL039C	Complex Number 413
YLL039C	Complex Number 425
YLL039C	Complex Number 49
YLL039C	Complex Number 508

YLL039C	Complex Number 532
YLL039C	Complex Number 535
YLL040C	Complex Number 134
YLL040C	Complex Number 205, probably transcription/DNA maintanance/chromatin structure
YLL040C	Complex Number 400
YLL040C	Complex Number 481
YLL040C	Complex Number 60, 20S Proteosome (13)
YLL040C	Complex Number 62
YLL041C	Complex Number 374
YLL041C	Succinate dehydrogenase complex (complex II)
YLL045C	Complex Number 38, Arx1 Complex (5)
YLL045C	cytoplasmic ribosomal large subunit
YLL048C	Complex Number 504
YLL050C	Actin-associated proteins
YLL050C	Complex Number 119
YLL050C	Complex Number 22
YLL050C	Complex Number 341
YLL050C	Complex Number 388
YLL050C	Complex Number 466
YLL050C	Complex Number 507
YLL050C	Complex Number 525
YLL050C	Complex Number 62
YLL050C	Complex Number 68
YLL050C	Complex Number 82
YLR002C	Complex Number 11, Noc (3)
YLR002C	Complex Number 149, probably RNA metabolism
YLR002C	Complex Number 16, Ded1 (1)
YLR002C	Complex Number 17, Pab1 (1)
YLR002C	Complex Number 520
YLR002C	Complex Number 56
YLR002C	Complex Number 94
YLR003C	Complex Number 144, probably RNA metabolism
YLR005W	Complex Number 202, probably transcription/DNA maintanance/chromatin structure
YLR005W	Complex Number 206, probably transcription/DNA maintanance/chromatin structure
YLR005W	NEF3 complex
YLR005W	RNA polymerase I
YLR006C	Complex Number 126
YLR006C	Complex Number 256
YLR006C	Complex Number 377
YLR009W	Complex Number 149, probably RNA metabolism
YLR009W	Complex Number 22
YLR009W	Complex Number 507
YLR015W	Complex Number 199, probably transcription/DNA maintanance/chromatin structure
YLR015W	Complex Number 342

YLR016C	Complex Number 506
YLR019W	Complex Number 248
YLR019W	Complex Number 97
YLR024C	Complex Number 520
YLR024C	Complex Number 94
YLR025W	Vps4p ATPase complex (Vps protein complex)
YLR026C	t-SNAREs
YLR027C	Complex Number 341
YLR027C	Complex Number 379
YLR027C	Complex Number 42
YLR029C	cytoplasmic ribosomal large subunit
YLR033W	Complex Number 192, probably transcription/DNA maintanance/chromatin structure
YLR033W	Complex Number 200, probably transcription/DNA maintanance/chromatin structure
YLR033W	Complex Number 203, probably transcription/DNA maintanance/chromatin structure
YLR033W	Complex Number 32, RSC (13)
YLR035C-A	Complex Number 134
YLR035C	Complex Number 110, probably protein synthesis turnover
YLR035C	Complex Number 111, probably protein synthesis turnover
YLR038C	Cytochrome c oxidase (complex IV)
YLR039C	Complex Number 107, probably protein synthesis turnover
YLR039C	Complex Number 549
YLR044C	Complex Number 105, probably protein synthesis turnover
YLR044C	Complex Number 119, probably protein/RNA transport
YLR044C	Complex Number 136, probably RNA metabolism
YLR044C	Complex Number 192, probably transcription/DNA maintanance/chromatin structure
YLR044C	Complex Number 196, probably transcription/DNA maintanance/chromatin structure
YLR044C	Complex Number 211, probably transcription/DNA maintanance/chromatin structure
YLR044C	Complex Number 230, probably transcription/DNA maintanance/chromatin structure
YLR044C	Complex Number 34, probably intermediate and energy metabolism
YLR044C	Complex Number 54, probably intermediate and energy metabolism
YLR044C	Complex Number 9, probably cell cycle
YLR045C	SPB associated proteins
YLR045C	Tubulin-associated proteins
YLR048W	cytoplasmic ribosomal small subunit
YLR055C	Complex Number 207, probably transcription/DNA maintanance/chromatin structure
YLR055C	Complex Number 214, probably transcription/DNA maintanance/chromatin structure
YLR055C	SAGA complex
YLR058C	Complex Number 240
YLR058C	Complex Number 25, probably intermediate and energy metabolism
YLR058C	Complex Number 300
YLR059C	Complex Number 240
YLR059C	Complex Number 275
YLR059C	Complex Number 4
YLR059C	Complex Number 410

YLR059C	Complex Number 68
YLR060W	Complex Number 113, probably protein synthesis turnover
YLR060W	Phenylalaninine-tRNA-ligase
YLR061W	cytoplasmic ribosomal large subunit
YLR067C	Mitochondrial translation complexes
YLR071C	Complex Number 102, probably protein synthesis turnover
YLR071C	Complex Number 111, probably protein synthesis turnover
YLR071C	Complex Number 209, probably transcription/DNA maintanance/chromatin structure
YLR071C	Complex Number 33, Mediator (8)
YLR071C	Complex Number 496
YLR071C	Complex Number 70, probably membrane biogenesis and traffic
YLR071C	Kornberg's mediator (SRB) complex
YLR074C	Complex Number 149, probably RNA metabolism
YLR074C	Complex Number 22
YLR074C	Complex Number 507
YLR075W	cytoplasmic ribosomal large subunit
YLR075W	Cytoplasmic ribosomes
YLR078C	v-SNAREs
YLR079W	Complex Number 6, probably cell cycle
YLR081W	Complex Number 156
YLR086W	Complex Number 127, probably protein/RNA transport
YLR086W	Complex Number 316
YLR086W	Complex Number 506
YLR092W	Complex Number 345
YLR093C	SNAREs
YLR093C	v-SNAREs
YLR096W	Complex Number 147
YLR096W	Complex Number 360
YLR097C	Complex Number 508
YLR100W	Complex Number 338
YLR102C	Anaphase promoting complex (APC)
YLR103C	Complex Number 10, probably cell cycle
YLR103C	Pre-replication complex (pre-RC)
YLR103C	Replication complex
YLR105C	Complex Number 221
YLR105C	Complex Number 32
YLR105C	tRNA splicing
YLR106C	Complex Number 134
YLR106C	Complex Number 140, probably RNA metabolism
YLR106C	Complex Number 149, probably RNA metabolism
YLR106C	Complex Number 204, probably transcription/DNA maintanance/chromatin structure
YLR106C	Complex Number 320
YLR106C	Complex Number 518
YLR106C	Complex Number 95

YLR109W	Complex Number 160
YLR109W	Complex Number 182
YLR109W	Complex Number 183
YLR109W	Complex Number 256
YLR109W	Complex Number 294
YLR109W	Complex Number 319
YLR109W	Complex Number 341
YLR109W	Complex Number 423
YLR109W	Complex Number 424
YLR109W	Complex Number 435
YLR113W	Complex Number 127
YLR113W	Complex Number 289
YLR113W	Complex Number 290
YLR115W	Complex Number 125, probably protein/RNA transport
YLR115W	Complex Number 148, probably RNA metabolism
YLR115W	Complex Number 56, mRNA cleavage/polyadenylation (10)
YLR115W	pre mRNA3'-end processing factor CFII
YLR117C	Complex Number 138, probably RNA metabolism
YLR117C	Complex Number 143, probably RNA metabolism
YLR117C	Complex Number 145, probably RNA metabolism
YLR117C	Complex Number 146, probably RNA metabolism
YLR117C	Complex Number 147, probably RNA metabolism
YLR117C	Complex Number 241
YLR117C	Complex Number 55, Prp19 (4)
YLR127C	Anaphase promoting complex (APC)
YLR127C	Complex Number 3, probably cell cycle
YLR127C	Complex Number 472
YLR129W	Complex Number 125, probably protein/RNA transport
YLR129W	Complex Number 144, probably RNA metabolism
YLR129W	Complex Number 493
YLR129W	Complex Number 511
YLR129W	Complex Number 87, UTP B (6)
YLR131C	Complex Number 96, probably protein synthesis turnover
YLR133W	Complex Number 308
YLR134W	Complex Number 115
YLR134W	Complex Number 119
YLR134W	Complex Number 28
YLR134W	Complex Number 379
YLR134W	Complex Number 435
YLR134W	Complex Number 47
YLR134W	Complex Number 49
YLR136C	Complex Number 91
YLR138W	Complex Number 479
YLR141W	Upstream Activation Factor (UAF) complex

YLR146C	Complex Number 26, probably intermediate and energy metabolism
YLR147C	Complex Number 139, probably RNA metabolism
YLR147C	Complex Number 145, probably RNA metabolism
YLR147C	Complex Number 146, probably RNA metabolism
YLR147C	Complex Number 147, probably RNA metabolism
YLR147C	Complex Number 155, probably RNA metabolism
YLR147C	mRNA splicing
YLR148W	Class C Vps protein complex
YLR148W	Complex Number 220
YLR148W	Complex Number 77, probably membrane biogenesis and traffic
YLR150W	Complex Number 124, probably protein/RNA transport
YLR150W	Complex Number 142, probably RNA metabolism
YLR150W	Complex Number 18, probably cell polarity and structure
YLR152C	Complex Number 54
YLR153C	Complex Number 244
YLR153C	Complex Number 304
YLR153C	Complex Number 379
YLR154C	Complex Number 153
YLR154C	Complex Number 398
YLR163C	Complex Number 127, probably protein/RNA transport
YLR163C	Complex Number 195
YLR163C	Complex Number 313
YLR163C	Processing peptidase
YLR166C	Complex Number 81, probably membrane biogenesis and traffic
YLR166C	Exocyst complex
YLR167W	cytoplasmic ribosomal small subunit
YLR170C	AP-1 complex
YLR170C	Complex Number 84, probably membrane biogenesis and traffic
YLR174W	Complex Number 23
YLR175W	Complex Number 109, probably protein synthesis turnover
YLR175W	Complex Number 125, probably protein/RNA transport
YLR175W	Complex Number 140, probably RNA metabolism
YLR175W	Complex Number 142, probably RNA metabolism
YLR175W	Complex Number 144, probably RNA metabolism
YLR175W	Complex Number 28
YLR175W	Complex Number 446
YLR175W	Complex Number 5, Gar1/Cbf5 (6)
YLR175W	Complex Number 96
YLR175W	Complex Number 99, probably protein synthesis turnover
YLR175W	Tubulin-associated proteins
YLR176C	Complex Number 200, probably transcription/DNA maintanance/chromatin structure
YLR180W	Complex Number 109, probably protein synthesis turnover
YLR180W	Complex Number 123, probably protein/RNA transport
YLR180W	Complex Number 134, probably RNA metabolism

YLR180W	Complex Number 144, probably RNA metabolism
YLR180W	Complex Number 204, probably transcription/DNA maintanance/chromatin structure
YLR180W	Complex Number 211, probably transcription/DNA maintanance/chromatin structure
YLR180W	Complex Number 30, probably intermediate and energy metabolism
YLR180W	Complex Number 39, probably intermediate and energy metabolism
YLR180W	Complex Number 41, probably intermediate and energy metabolism
YLR180W	Complex Number 52, probably intermediate and energy metabolism
YLR180W	Complex Number 54, probably intermediate and energy metabolism
YLR180W	Complex Number 62, probably intermediate and energy metabolism
YLR180W	Complex Number 70, probably membrane biogenesis and traffic
YLR180W	Complex Number 79, probably membrane biogenesis and traffic
YLR182W	Complex Number 223, probably transcription/DNA maintanance/chromatin structure
YLR182W	MBF complex
YLR182W	SBF complex
YLR183C	Complex Number 147, probably RNA metabolism
YLR185W	cytoplasmic ribosomal large subunit
YLR186W	Complex Number 109, probably protein synthesis turnover
YLR186W	Complex Number 125, probably protein/RNA transport
YLR186W	Complex Number 144, probably RNA metabolism
YLR186W	Complex Number 509
YLR186W	Complex Number 510
YLR187W	Complex Number 360
YLR189C	Complex Number 108, probably protein synthesis turnover
YLR192C	Complex Number 107, probably protein synthesis turnover
YLR192C	Complex Number 51, eIF3 (7)
YLR196W	Complex Number 200
YLR196W	Complex Number 22
YLR196W	Complex Number 229, probably transcription/DNA maintanance/chromatin structure
YLR196W	Complex Number 254
YLR196W	Complex Number 40, Pwp1/Brx1/Nop12 (3)
YLR196W	Complex Number 446
YLR196W	Complex Number 507
YLR196W	Complex Number 56
YLR196W	Complex Number 6
YLR196W	Complex Number 91
YLR196W	Complex Number 96
YLR197W	Complex Number 125, probably protein/RNA transport
YLR197W	Complex Number 14
YLR197W	Complex Number 140, probably RNA metabolism
YLR197W	Complex Number 144, probably RNA metabolism
YLR197W	Complex Number 149, probably RNA metabolism
YLR197W	Complex Number 150
YLR197W	Complex Number 205
YLR197W	Complex Number 28

YLR197W	Complex Number 397
YLR197W	Complex Number 404
YLR197W	Complex Number 493
YLR197W	Complex Number 56
YLR197W	Complex Number 6, Nop58/Sik1 (3)
YLR197W	Nop56p/Nop1p complex
YLR199C	Complex Number 238
YLR200W	Complex Number 7, probably cell cycle
YLR200W	Gim complexes
YLR203C	Mitochondrial translation complexes
YLR206W	Complex Number 26
YLR208W	Complex Number 124, probably protein/RNA transport
YLR208W	Complex Number 148, probably RNA metabolism
YLR208W	Complex Number 337
YLR208W	Complex Number 340
YLR208W	Complex Number 44, probably intermediate and energy metabolism
YLR208W	Complex Number 80, probably membrane biogenesis and traffic
YLR208W	COPII
YLR208W	NUP84 complex
YLR210W	Cdc28p complexes
YLR212C	Complex Number 7, probably cell cycle
YLR212C	gamma-tubulin complex
YLR212C	Tubulins
YLR215C	Complex Number 107
YLR216C	Complex Number 23
YLR216C	Complex Number 320
YLR216C	Complex Number 338
YLR216C	Complex Number 360
YLR216C	Complex Number 374
YLR216C	Complex Number 4
YLR216C	Complex Number 402
YLR216C	Complex Number 447
YLR216C	Complex Number 459
YLR216C	Complex Number 71
YLR216C	Complex Number 95
YLR219W	Complex Number 517
YLR221C	Complex Number 148, probably RNA metabolism
YLR221C	Complex Number 149, probably RNA metabolism
YLR222C	Complex Number 125, probably protein/RNA transport
YLR222C	Complex Number 137
YLR222C	Complex Number 144, probably RNA metabolism
YLR222C	Complex Number 203
YLR222C	Complex Number 240
YLR222C	Complex Number 255

YLR222C	Complex Number 333
YLR222C	Complex Number 493
YLR222C	Complex Number 511
YLR222C	Complex Number 87, UTP B (6)
YLR222C	rRNA splicing
YLR223C	rRNA splicing
YLR226W	Complex Number 139, probably RNA metabolism
YLR226W	Complex Number 58
YLR229C	Complex Number 45
YLR231C	Complex Number 49
YLR233C	Complex Number 377
YLR233C	Complex Number 96
YLR233C	Telomerase
YLR234W	Complex Number 212, probably transcription/DNA maintanance/chromatin structure
YLR238W	Complex Number 512
YLR239C	Complex Number 204, probably transcription/DNA maintanance/chromatin structure
YLR241W	Complex Number 424
YLR241W	Complex Number 9
YLR243W	Complex Number 328
YLR247C	Complex Number 513
YLR248W	Complex Number 127
YLR248W	Complex Number 12, Sit4 (6)
YLR248W	Complex Number 290
YLR249W	Complex Number 114, probably protein synthesis turnover
YLR249W	Complex Number 136, probably RNA metabolism
YLR249W	Complex Number 146, probably RNA metabolism
YLR249W	Complex Number 149, probably RNA metabolism
YLR249W	Complex Number 170, probably signalling
YLR249W	Complex Number 17, probably cell polarity and structure
YLR249W	Complex Number 192, probably transcription/DNA maintanance/chromatin structure
YLR249W	Complex Number 194, probably transcription/DNA maintanance/chromatin structure
YLR249W	Complex Number 196, probably transcription/DNA maintanance/chromatin structure
YLR249W	Complex Number 19, mRNA cap-binding/eIF4F (8)
YLR249W	eEF3
YLR258W	Complex Number 114
YLR258W	Complex Number 115
YLR258W	Complex Number 17
YLR258W	Complex Number 491
YLR258W	Complex Number 537
YLR262C	Complex Number 113
YLR262C	Complex Number 549
YLR263W	Complex Number 291
YLR263W	Synaptonemal complex (SC)
YLR264W	cytoplasmic ribosomal small subunit

YLR267W	Complex Number 357
YLR268W	v-SNAREs
YLR270W	Complex Number 424
YLR271W	Complex Number 505
YLR274W	Complex Number 10, probably cell cycle
YLR274W	Complex Number 82
YLR274W	Pre-replication complex (pre-RC)
YLR274W	Replication complex
YLR275W	Complex Number 138, probably RNA metabolism
YLR275W	Complex Number 139, probably RNA metabolism
YLR275W	Complex Number 143, probably RNA metabolism
YLR275W	Complex Number 145, probably RNA metabolism
YLR275W	Complex Number 146, probably RNA metabolism
YLR275W	Complex Number 147, probably RNA metabolism
YLR276C	Complex Number 102, probably protein synthesis turnover
YLR276C	Complex Number 140, probably RNA metabolism
YLR276C	Complex Number 149, probably RNA metabolism
YLR276C	Complex Number 50
YLR277C	Complex Number 125, probably protein/RNA transport
YLR277C	Complex Number 148, probably RNA metabolism
YLR277C	Complex Number 56, mRNA cleavage/polyadenylation (10)
YLR277C	pre mRNA3'-end processing factor CFII
YLR277C	pre mRNA polyadenylation factor PFI
YLR284C	delta3-cis-delta2-trans-enoyl-CoA isomerase
YLR287C-A	cytoplasmic ribosomal small subunit
YLR287C	Complex Number 319
YLR288C	Complex Number 160
YLR288C	Complex Number 177
YLR288C	Complex Number 80
YLR288C	Complex Number 81
YLR288C	Ddc1p-Mec3p complex
YLR289W	Complex Number 111
YLR289W	Complex Number 134
YLR289W	Complex Number 508
YLR289W	Complex Number 542
YLR291C	Complex Number 101
YLR291C	Complex Number 105, probably protein synthesis turnover
YLR291C	Complex Number 108
YLR291C	Complex Number 109
YLR291C	Complex Number 111
YLR291C	Complex Number 52, tIF2 (7)
YLR291C	eIF2B
YLR292C	Complex Number 72, probably membrane biogenesis and traffic
YLR292C	Sec62-63 complex

YLR293C	Complex Number 120
YLR293C	Complex Number 146
YLR293C	Complex Number 312
YLR293C	Complex Number 375
YLR293C	Complex Number 445
YLR295C	F0/F1 ATP synthase (complex V)
YLR298C	Complex Number 145, probably RNA metabolism
YLR298C	Complex Number 146, probably RNA metabolism
YLR298C	Complex Number 147, probably RNA metabolism
YLR298C	mRNA splicing
YLR300W	Complex Number 174
YLR300W	Complex Number 520
YLR300W	Complex Number 94
YLR304C	Complex Number 132
YLR304C	Complex Number 152
YLR304C	Complex Number 161
YLR304C	Complex Number 176
YLR304C	Complex Number 267
YLR304C	Complex Number 272
YLR304C	Complex Number 293
YLR304C	Complex Number 301
YLR304C	Complex Number 341
YLR304C	Complex Number 396
YLR304C	Complex Number 488
YLR304C	Complex Number 520
YLR304C	Complex Number 549
YLR304C	Complex Number 83
YLR304C	Complex Number 94
YLR305C	Complex Number 14, U6-specific snRNP core (14)
YLR305C	Complex Number 23, probably intermediate and energy metabolism
YLR306W	Complex Number 320
YLR306W	Complex Number 408
YLR309C	Complex Number 13
YLR310C	Complex Number 133
YLR310C	Complex Number 163, probably signalling
YLR310C	Complex Number 1, probably cell cycle
YLR310C	Complex Number 308
YLR312W-A	mitochondrial ribosomal large subunit
YLR313C	Complex Number 405
YLR314C	Complex Number 15, probably cell polarity and structure
YLR314C	Complex Number 33
YLR314C	Complex Number 34
YLR314C	Complex Number 35
YLR314C	Complex Number 42

YLR314C	Complex Number 4, probably cell cycle
YLR314C	Septin filaments
YLR318W	Telomerase
YLR319C	Actin-associated proteins
YLR320W	Complex Number 514
YLR320W	Complex Number 515
YLR321C	Complex Number 200, probably transcription/DNA maintanance/chromatin structure
YLR321C	Complex Number 32, RSC (13)
YLR321C	RSC complex (Remodel the structure of chromatin)
YLR325C	cytoplasmic ribosomal large subunit
YLR326W	Complex Number 158
YLR328W	Complex Number 6
YLR331C	Complex Number 49
YLR333C	cytoplasmic ribosomal small subunit
YLR335W	Complex Number 376
YLR335W	Nuclear pore complex (NPC)
YLR336C	Complex Number 459
YLR337C	Actin-associated proteins
YLR337C	Complex Number 155
YLR337C	Complex Number 18, probably cell polarity and structure
YLR340W	Complex Number 319
YLR340W	Complex Number 54, Rli1/Rpp0 (3)
YLR340W	cytoplasmic ribosomal large subunit
YLR342W	1,3-beta-D-glucan synthase
YLR342W	Complex Number 123, probably protein/RNA transport
YLR342W	Complex Number 14, probably cell polarity and structure
YLR342W	Complex Number 23, probably intermediate and energy metabolism
YLR342W	Complex Number 53, probably intermediate and energy metabolism
YLR342W	Complex Number 67, probably membrane biogenesis and traffic
YLR342W	Complex Number 69, probably membrane biogenesis and traffic
YLR342W	Complex Number 70, probably membrane biogenesis and traffic
YLR342W	Complex Number 72, probably membrane biogenesis and traffic
YLR342W	Complex Number 75, probably membrane biogenesis and traffic
YLR342W	Complex Number 80, probably membrane biogenesis and traffic
YLR344W	cytoplasmic ribosomal large subunit
YLR347C	Complex Number 101, probably protein synthesis turnover
YLR347C	Complex Number 102, probably protein synthesis turnover
YLR347C	Complex Number 120
YLR347C	Complex Number 139, probably RNA metabolism
YLR347C	Complex Number 145
YLR347C	Complex Number 158
YLR347C	Complex Number 170, probably signalling
YLR347C	Complex Number 202, probably transcription/DNA maintanance/chromatin structure
YLR347C	Complex Number 206, probably transcription/DNA maintanance/chromatin structure

YLR347C	Complex Number 212, probably transcription/DNA maintanance/chromatin structure
YLR347C	Complex Number 279
YLR347C	Complex Number 311
YLR347C	Complex Number 376
YLR347C	Complex Number 446
YLR347C	Complex Number 46
YLR347C	Complex Number 53, Kap95/Srp1 (2)
YLR352W	Complex Number 47
YLR352W	Complex Number 516
YLR354C	Complex Number 168
YLR354C	Complex Number 194
YLR354C	Complex Number 492
YLR354C	Complex Number 88
YLR355C	Complex Number 157
YLR355C	Complex Number 328
YLR355C	Complex Number 350
YLR355C	Complex Number 360
YLR355C	Complex Number 435
YLR355C	Complex Number 67
YLR357W	Complex Number 192, probably transcription/DNA maintanance/chromatin structure
YLR357W	Complex Number 200, probably transcription/DNA maintanance/chromatin structure
YLR357W	Complex Number 203, probably transcription/DNA maintanance/chromatin structure
YLR357W	Complex Number 32, RSC (13)
YLR357W	Complex Number 5, probably cell cycle
YLR357W	RSC complex (Remodel the structure of chromatin)
YLR359W	Complex Number 235
YLR359W	Complex Number 340
YLR359W	Complex Number 49, probably intermediate and energy metabolism
YLR362W	Complex Number 113
YLR362W	Complex Number 152
YLR362W	Complex Number 153
YLR362W	Complex Number 418
YLR362W	STE5-MAPK complex
YLR367W	cytoplasmic ribosomal small subunit
YLR368W	Complex Number 257
YLR369W	Complex Number 520
YLR369W	Complex Number 94
YLR370C	Arp2p/Arp3p complex
YLR370C	Complex Number 13
YLR370C	Complex Number 19, probably cell polarity and structure
YLR370C	Complex Number 9
YLR371W	Complex Number 108, probably protein synthesis turnover
YLR371W	Complex Number 142, probably RNA metabolism
YLR371W	Complex Number 197, probably transcription/DNA maintanance/chromatin structure

YLR371W	Complex Number 297
YLR371W	Complex Number 34, probably intermediate and energy metabolism
YLR371W	Complex Number 55, probably intermediate and energy metabolism
YLR375W	tRNA splicing
YLR378C	Sec61 complex
YLR381W	Complex Number 221, probably transcription/DNA maintanance/chromatin structure
YLR381W	Ctf3 protein complex
YLR382C	Mitochondrial splicing complexes
YLR383W	Complex Number 303
YLR384C	Complex Number 216, probably transcription/DNA maintanance/chromatin structure
YLR384C	Complex Number 93
YLR386W	Complex Number 197, probably transcription/DNA maintanance/chromatin structure
YLR386W	Complex Number 369
YLR386W	Complex Number 40, probably intermediate and energy metabolism
YLR386W	Complex Number 49
YLR388W	cytoplasmic ribosomal small subunit
YLR389C	Complex Number 16
YLR389C	Complex Number 375
YLR392C	Complex Number 322
YLR395C	Cytochrome c oxidase (complex IV)
YLR396C	Class C Vps protein complex
YLR396C	Complex Number 522
YLR396C	Complex Number 77, probably membrane biogenesis and traffic
YLR397C	Complex Number 22
YLR397C	Complex Number 507
YLR397C	Complex Number 59, probably intermediate and energy metabolism
YLR397C	Complex Number 89
YLR398C	Complex Number 141, probably RNA metabolism
YLR398C	Complex Number 149, probably RNA metabolism
YLR398C	Complex Number 355
YLR399C	Complex Number 179, probably transcription/DNA maintanance/chromatin structure
YLR403W	Complex Number 343
YLR403W	Complex Number 429
YLR403W	Complex Number 56
YLR403W	Complex Number 74
YLR406C	cytoplasmic ribosomal large subunit
YLR409C	Complex Number 125, probably protein/RNA transport
YLR409C	Complex Number 140, probably RNA metabolism
YLR409C	Complex Number 144, probably RNA metabolism
YLR409C	Complex Number 147, probably RNA metabolism
YLR409C	Complex Number 347
YLR409C	Complex Number 493
YLR409C	Complex Number 87, UTP B (6)
YLR410W	Complex Number 40, Pwp1/Brx1/Nop12 (3)

YLR410W	Complex Number 41, Nap1 (3)
YLR410W	Complex Number 44, Vip1 (1)
YLR410W	Complex Number 46, SPT16 (8)
YLR410W	Complex Number 4, Dbp3/Bmh1/Nsr1 (3)
YLR410W	Complex Number 5, Gar1/Cbf5 (6)
YLR411W	Complex Number 29, probably intermediate and energy metabolism
YLR413W	Complex Number 265
YLR413W	Complex Number 470
YLR418C	Complex Number 201, probably transcription/DNA maintanance/chromatin structure
YLR418C	Complex Number 46, SPT16 (8)
YLR418C	RNA polymerase II
YLR421C	Complex Number 110, probably protein synthesis turnover
YLR421C	Complex Number 111, probably protein synthesis turnover
YLR421C	Complex Number 468
YLR422W	Complex Number 467
YLR423C	Complex Number 123
YLR423C	Complex Number 4
YLR423C	Complex Number 530
YLR424W	Complex Number 138, probably RNA metabolism
YLR424W	Complex Number 143, probably RNA metabolism
YLR424W	Complex Number 145, probably RNA metabolism
YLR424W	Complex Number 146, probably RNA metabolism
YLR424W	Complex Number 147, probably RNA metabolism
YLR425W	Complex Number 55, probably intermediate and energy metabolism
YLR427W	Complex Number 517
YLR429W	Actin-associated proteins
YLR429W	Complex Number 197, probably transcription/DNA maintanance/chromatin structure
YLR429W	Complex Number 339
YLR429W	Complex Number 68
YLR430W	Complex Number 115
YLR430W	Complex Number 139, probably RNA metabolism
YLR430W	Complex Number 152
YLR430W	Complex Number 257
YLR432W	Complex Number 106
YLR432W	Complex Number 169
YLR432W	Complex Number 204
YLR432W	Complex Number 244
YLR432W	Complex Number 404
YLR432W	Complex Number 446
YLR432W	Complex Number 458
YLR432W	Complex Number 517
YLR432W	Complex Number 57, Imd (3)
YLR432W	Complex Number 91
YLR433C	Calcineurin B

YLR433C	Complex Number 65
YLR433C	Complex Number 66
YLR435W	Complex Number 86, Tsr2 (1)
YLR436C	Complex Number 96, probably protein synthesis turnover
YLR438C-A	Complex Number 138, probably RNA metabolism
YLR438C-A	Complex Number 14, U6-specific snRNP core (14)
YLR438W	Complex Number 132
YLR438W	Complex Number 143
YLR438W	Complex Number 149
YLR438W	Complex Number 23
YLR438W	Complex Number 257
YLR438W	Complex Number 304
YLR438W	Complex Number 341
YLR438W	Complex Number 379
YLR438W	Complex Number 4
YLR438W	Complex Number 435
YLR438W	Complex Number 510
YLR438W	Complex Number 541
YLR438W	Complex Number 79
YLR438W	Complex Number 95
YLR439W	Complex Number 108, probably protein synthesis turnover
YLR439W	Complex Number 147, probably RNA metabolism
YLR439W	mitochondrial ribosomal large subunit
YLR441C	cytoplasmic ribosomal small subunit
YLR442C	Complex Number 349
YLR442C	Complex Number 350
YLR442C	Complex Number 351
YLR442C	Complex Number 352
YLR447C	Complex Number 156
YLR447C	Complex Number 29
YLR447C	Complex Number 455
YLR447C	Complex Number 45, probably intermediate and energy metabolism
YLR447C	H+-transporting ATPase, vacuolar
YLR448W	cytoplasmic ribosomal large subunit
YLR449W	Complex Number 115
YLR449W	Complex Number 149, probably RNA metabolism
YLR449W	Complex Number 229, probably transcription/DNA maintanance/chromatin structure
YLR449W	Complex Number 461
YLR449W	Complex Number 520
YLR449W	Complex Number 94
YLR450W	Complex Number 356
YLR451W	LEU3 complex
YML001W	Complex Number 113
YML001W	Complex Number 18, probably cell polarity and structure

YML001W	Complex Number 402
YML006C	Complex Number 147
YML006C	Complex Number 367
YML007W	Complex Number 162, probably signalling
YML007W	Complex Number 191, probably transcription/DNA maintanance/chromatin structure
YML007W	Complex Number 207, probably transcription/DNA maintanance/chromatin structure
YML007W	Complex Number 209, probably transcription/DNA maintanance/chromatin structure
YML008C	Complex Number 341
YML008C	S-adenosyl-methionine delta-24-sterol-c-methyltransferase
YML009C	Complex Number 108, probably protein synthesis turnover
YML009C	mitochondrial ribosomal large subunit
YML010W	Complex Number 107, probably protein synthesis turnover
YML010W	Complex Number 169, probably signalling
YML010W	Complex Number 210, probably transcription/DNA maintanance/chromatin structure
YML010W	Complex Number 24, RNA Polymerase II (12)
YML010W	Complex Number 95
YML012W	Complex Number 69, probably membrane biogenesis and traffic
YML012W	Complex Number 83, probably membrane biogenesis and traffic
YML012W	COPII
YML014W	Complex Number 36, probably intermediate and energy metabolism
YML015C	Complex Number 207, probably transcription/DNA maintanance/chromatin structure
YML015C	Complex Number 214, probably transcription/DNA maintanance/chromatin structure
YML015C	TAFIIs
YML016C	Complex Number 236
YML019W	Complex Number 44, probably intermediate and energy metabolism
YML019W	Oligosaccharyltransferase
YML020W	Complex Number 504
YML022W	Complex Number 504
YML024W	cytoplasmic ribosomal small subunit
YML025C	Complex Number 108, probably protein synthesis turnover
YML025C	Complex Number 147, probably RNA metabolism
YML025C	mitochondrial ribosomal large subunit
YML026C	cytoplasmic ribosomal small subunit
YML028W	Complex Number 41, probably intermediate and energy metabolism
YML028W	Complex Number 68, probably membrane biogenesis and traffic
YML029W	Complex Number 518
YML030W	Complex Number 148, probably RNA metabolism
YML032C	Complex Number 195, probably transcription/DNA maintanance/chromatin structure
YML032C	Complex Number 202, probably transcription/DNA maintanance/chromatin structure
YML032C	Complex Number 212, probably transcription/DNA maintanance/chromatin structure
YML032C	Complex Number 277
YML032C	Complex Number 283
YML032C	other DNA repair complexes
YML034W	Sister chromatid separation complex

YML036W	Complex Number 23
YML043C	Core Factor (CF)
YML046W	Complex Number 139, probably RNA metabolism
YML046W	Complex Number 145, probably RNA metabolism
YML046W	Complex Number 146, probably RNA metabolism
YML046W	Complex Number 147, probably RNA metabolism
YML046W	Complex Number 35, U1 snRNP (7)
YML046W	mRNA splicing
YML048W	Complex Number 124
YML048W	Complex Number 353
YML048W	Complex Number 442
YML049C	Complex Number 135
YML049C	Complex Number 139, probably RNA metabolism
YML049C	Complex Number 143, probably RNA metabolism
YML049C	Complex Number 145, probably RNA metabolism
YML049C	Complex Number 146, probably RNA metabolism
YML049C	Complex Number 147, probably RNA metabolism
YML049C	Complex Number 336
YML051W	GAL80 complex
YML054C	other respiration chain complexes
YML055W	Signal peptidase
YML056C	Complex Number 169
YML056C	Complex Number 229
YML056C	Complex Number 23
YML056C	Complex Number 303
YML056C	Complex Number 35
YML056C	Complex Number 404
YML056C	Complex Number 463
YML056C	Complex Number 489
YML056C	Complex Number 517
YML056C	Complex Number 57, Imd (3)
YML056C	Complex Number 91
YML057W	Calcineurin B
YML057W	Complex Number 147
YML057W	Complex Number 151
YML057W	Complex Number 62
YML057W	Complex Number 64
YML057W	Complex Number 66
YML058W	Complex Number 364
YML058W	Complex Number 365
YML059C	Complex Number 165, probably signalling
YML062C	Complex Number 106
YML062C	Complex Number 129
YML062C	Complex Number 30, Sub2 (1)

YML062C	Complex Number 31, TREX (5)
YML062C	Complex Number 376
YML063W	cytoplasmic ribosomal small subunit
YML064C	Complex Number 388
YML064C	Complex Number 389
YML064C	Complex Number 390
YML064C	Complex Number 391
YML064C	Complex Number 505
YML065W	Complex Number 10, probably cell cycle
YML065W	Post-replication complex (Origin recognition complex=ORC)
YML065W	Pre-replication complex (pre-RC)
YML065W	Replication complex
YML065W	Replication initiation complex
YML067C	Complex Number 80, probably membrane biogenesis and traffic
YML069W	Complex Number 201, probably transcription/DNA maintanance/chromatin structure
YML069W	Complex Number 46, SPT16 (8)
YML069W	Complex Number 47, Casein Kinase II (4)
YML069W	Complex Number 534
YML069W	Complex Number 56
YML069W	Complex Number 9
YML071C	Complex Number 105, probably protein synthesis turnover
YML071C	Golgi transport complex
YML072C	Complex Number 383
YML072C	Complex Number 67, probably membrane biogenesis and traffic
YML073C	cytoplasmic ribosomal large subunit
YML074C	Complex Number 115
YML074C	Complex Number 133
YML074C	Complex Number 200
YML074C	Complex Number 369
YML074C	Complex Number 56
YML074C	Complex Number 82
YML077W	Complex Number 75, probably membrane biogenesis and traffic
YML077W	TRAPP (Transport Protein Particle) complex
YML078W	Complex Number 388
YML081C-A	F0/F1 ATP synthase (complex V)
YML085C	Tubulins
YML088W	Complex Number 357
YML088W	Complex Number 519
YML091C	Complex Number 132
YML091C	Complex Number 133
YML091C	Complex Number 150, probably RNA metabolism
YML092C	20S proteasome
YML092C	Complex Number 102, probably protein synthesis turnover
YML092C	Complex Number 110, probably protein synthesis turnover

YML092C	Complex Number 111, probably protein synthesis turnover
YML092C	Complex Number 170, probably signalling
YML092C	Complex Number 238
YML092C	Complex Number 60, 20S Proteosome (13)
YML093W	Complex Number 493
YML094W	Complex Number 7, probably cell cycle
YML094W	Gim complexes
YML095C	Complex Number 136, probably RNA metabolism
YML095C	Complex Number 258
YML095C	NEF1 complex
YML097C	Complex Number 81, probably membrane biogenesis and traffic
YML098W	Complex Number 214, probably transcription/DNA maintanance/chromatin structure
YML098W	TAFIIs
YML099C	ARG complex
YML100W	Complex Number 249
YML100W	Complex Number 29, probably intermediate and energy metabolism
YML100W	Complex Number 308
YML100W	Complex Number 413
YML102W	Chromatin assembly complex (CAC)
YML102W	Complex Number 24
YML103C	Nuclear pore complex (NPC)
YML104C	Intermediate filaments
YML105C	Signal recognition particle (SRP)
YML109W	Complex Number 170, probably signalling
YML109W	Complex Number 347
YML112W	Complex Number 215, probably transcription/DNA maintanance/chromatin structure
YML112W	Complex Number 75
YML112W	Ctk1p complex
YML112W	TFIIK (CTD kinase)
YML114C	Complex Number 214, probably transcription/DNA maintanance/chromatin structure
YML115C	Complex Number 417
YML117W	Complex Number 139, probably RNA metabolism
YML117W	Complex Number 142, probably RNA metabolism
YML117W	Complex Number 155, probably RNA metabolism
YML117W	Complex Number 197, probably transcription/DNA maintanance/chromatin structure
YML120C	other respiration chain complexes
YML123C	Complex Number 152
YML123C	Complex Number 156
YML123C	Complex Number 205
YML123C	Complex Number 322
YML123C	Complex Number 323
YML123C	Complex Number 328
YML123C	Complex Number 329
YML123C	Complex Number 374

YML123C	Complex Number 449
YML123C	Complex Number 512
YML123C	Complex Number 528
YML124C	Complex Number 133, probably RNA metabolism
YML124C	Complex Number 149, probably RNA metabolism
YML124C	Complex Number 231, probably transcription/DNA maintanance/chromatin structure
YML124C	Complex Number 41, probably intermediate and energy metabolism
YML124C	Complex Number 54, probably intermediate and energy metabolism
YML124C	Tubulins
YML126C	Complex Number 170, probably signalling
YML126C	Complex Number 341
YML126C	Complex Number 52, probably intermediate and energy metabolism
YML127W	Complex Number 32, RSC (13)
YML130C	Complex Number 125, probably protein/RNA transport
YML130C	Complex Number 144, probably RNA metabolism
YML130C	Complex Number 44, probably intermediate and energy metabolism
YML130C	Complex Number 80, probably membrane biogenesis and traffic
YMR001C	Complex Number 201
YMR001C	Complex Number 46
YMR005W	Complex Number 214, probably transcription/DNA maintanance/chromatin structure
YMR005W	Complex Number 334
YMR012W	Complex Number 104
YMR012W	Complex Number 107, probably protein synthesis turnover
YMR012W	Complex Number 115
YMR012W	Complex Number 123, probably protein/RNA transport
YMR012W	Complex Number 135, probably RNA metabolism
YMR012W	Complex Number 149, probably RNA metabolism
YMR012W	Complex Number 150
YMR012W	Complex Number 175
YMR012W	Complex Number 195, probably transcription/DNA maintanance/chromatin structure
YMR012W	Complex Number 197, probably transcription/DNA maintanance/chromatin structure
YMR012W	Complex Number 207
YMR012W	Complex Number 21
YMR012W	Complex Number 214, probably transcription/DNA maintanance/chromatin structure
YMR012W	Complex Number 231, probably transcription/DNA maintanance/chromatin structure
YMR012W	Complex Number 240
YMR012W	Complex Number 244
YMR012W	Complex Number 34
YMR012W	Complex Number 341
YMR012W	Complex Number 344
YMR012W	Complex Number 388
YMR012W	Complex Number 389
YMR012W	Complex Number 396
YMR012W	Complex Number 39, probably intermediate and energy metabolism

YMR012W	Complex Number 405
YMR012W	Complex Number 41
YMR012W	Complex Number 41, probably intermediate and energy metabolism
YMR012W	Complex Number 452
YMR012W	Complex Number 474
YMR012W	Complex Number 505
YMR012W	Complex Number 544
YMR012W	Complex Number 55
YMR012W	Complex Number 99
YMR019W	Complex Number 382
YMR021C	Complex Number 204, probably transcription/DNA maintanance/chromatin structure
YMR022W	Complex Number 256
YMR023C	Mitochondrial splicing complexes
YMR023C	Mto1p/Mss1p complex
YMR024W	Complex Number 108, probably protein synthesis turnover
YMR024W	Complex Number 147, probably RNA metabolism
YMR024W	Complex Number 1, probably cell cycle
YMR024W	Complex Number 41, probably intermediate and energy metabolism
YMR024W	mitochondrial ribosomal large subunit
YMR025W	Complex Number 87, probably protein synthesis turnover
YMR027W	Complex Number 145, probably RNA metabolism
YMR028W	Complex Number 232
YMR028W	Complex Number 233
YMR028W	Complex Number 353
YMR028W	Complex Number 92, probably protein synthesis turnover
YMR029C	Complex Number 118
YMR029C	Complex Number 449
YMR029C	Complex Number 512
YMR033W	Complex Number 198, probably transcription/DNA maintanance/chromatin structure
YMR033W	Complex Number 32, RSC (13)
YMR033W	RSC complex (Remodel the structure of chromatin)
YMR033W	SWI/SNF transcription activator complex
YMR035W	Inner membrane protease
YMR036C	Complex Number 186
YMR042W	ARG complex
YMR043W	ARG complex
YMR047C	Complex Number 126, probably protein/RNA transport
YMR047C	Nuclear pore complex (NPC)
YMR049C	Complex Number 115
YMR049C	Complex Number 138, probably RNA metabolism
YMR049C	Complex Number 140, probably RNA metabolism
YMR049C	Complex Number 149, probably RNA metabolism
YMR049C	Complex Number 171
YMR049C	Complex Number 200

YMR049C	Complex Number 28, Nop7/Erb1/Ytm1 (3)
YMR049C	Complex Number 446
YMR049C	Complex Number 480
YMR049C	Complex Number 520
YMR049C	Complex Number 521
YMR049C	Complex Number 552
YMR049C	Complex Number 56
YMR049C	Complex Number 91
YMR049C	Complex Number 94
YMR052W	Complex Number 34, probably intermediate and energy metabolism
YMR053C	Sin3 complex
YMR054W	H+-transporting ATPase, vacuolar
YMR055C	Complex Number 20
YMR056C	Complex Number 271
YMR056C	Complex Number 4
YMR058W	Complex Number 109
YMR058W	Complex Number 13
YMR058W	Complex Number 138
YMR058W	Complex Number 207
YMR058W	Complex Number 4
YMR058W	Complex Number 5
YMR059W	Complex Number 341
YMR059W	tRNA splicing
YMR060C	TOM - transport across the outer membrane
YMR061W	Complex Number 125, probably protein/RNA transport
YMR061W	Complex Number 148, probably RNA metabolism
YMR061W	pre mRNA3'-end processing factor CFI
YMR064W	Complex Number 115, probably protein synthesis turnover
YMR064W	Mitochondrial translation complexes
YMR065W	SPB associated proteins
YMR070W	Complex Number 37
YMR072W	Complex Number 208, probably transcription/DNA maintanance/chromatin structure
YMR075W	Complex Number 208, probably transcription/DNA maintanance/chromatin structure
YMR078C	Complex Number 205, probably transcription/DNA maintanance/chromatin structure
YMR080C	Complex Number 138, probably RNA metabolism
YMR080C	Complex Number 155, probably RNA metabolism
YMR080C	Complex Number 70, Nam7 (1)
YMR080C	Nonsense-mediated mRNA decay pathway complex
YMR083W	Complex Number 365
YMR086W	Complex Number 367
YMR089C	m-AAA protease complex
YMR091C	Complex Number 192, probably transcription/DNA maintanance/chromatin structure
YMR091C	Complex Number 200, probably transcription/DNA maintanance/chromatin structure
YMR091C	Complex Number 203, probably transcription/DNA maintanance/chromatin structure

YMR091C	Complex Number 220, probably transcription/DNA maintanance/chromatin structure
YMR091C	Complex Number 32, RSC (13)
YMR091C	Complex Number 50, 19S Proteosome (17)
YMR092C	Actin-associated proteins
YMR092C	Complex Number 68
YMR093W	Complex Number 109, probably protein synthesis turnover
YMR093W	Complex Number 125, probably protein/RNA transport
YMR093W	Complex Number 144, probably RNA metabolism
YMR093W	Complex Number 43, UTP A (8)
YMR093W	Complex Number 521
YMR094W	CBF3 protein complex
YMR094W	Complex Number 73
YMR100W	Complex Number 520
YMR100W	Complex Number 94
YMR102C	Complex Number 178
YMR104C	Complex Number 540
YMR105C	Complex Number 143
YMR105C	Complex Number 146
YMR105C	Complex Number 244
YMR105C	Complex Number 284
YMR105C	Complex Number 296
YMR105C	Complex Number 298
YMR105C	Complex Number 378
YMR105C	Complex Number 454
YMR105C	Complex Number 511
YMR105C	Complex Number 525
YMR105C	Complex Number 544
YMR105C	Complex Number 60
YMR105C	Complex Number 62
YMR105C	Complex Number 79
YMR105C	Complex Number 90
YMR106C	Complex Number 187, probably transcription/DNA maintanance/chromatin structure
YMR106C	Complex Number 504
YMR106C	Complex Number 505
YMR106C	Ku complex
YMR108W	Alpha-agglutinin anchor
YMR108W	Complex Number 283
YMR108W	Complex Number 392
YMR108W	Complex Number 505
YMR109W	Actin-associated motorproteins
YMR109W	Complex Number 170, probably signalling
YMR109W	Complex Number 62
YMR112C	Complex Number 209, probably transcription/DNA maintanance/chromatin structure
YMR112C	Kornberg's mediator (SRB) complex

YMR116C	Complex Number 106, probably protein synthesis turnover
YMR116C	Complex Number 107, probably protein synthesis turnover
YMR116C	Complex Number 137, probably RNA metabolism
YMR116C	Complex Number 138, probably RNA metabolism
YMR116C	Complex Number 14
YMR116C	Complex Number 142, probably RNA metabolism
YMR116C	Complex Number 144, probably RNA metabolism
YMR116C	Complex Number 193, probably transcription/DNA maintanance/chromatin structure
YMR116C	Complex Number 213, probably transcription/DNA maintanance/chromatin structure
YMR116C	Complex Number 231, probably transcription/DNA maintanance/chromatin structure
YMR116C	Complex Number 68, probably membrane biogenesis and traffic
YMR116C	Complex Number 88, probably protein synthesis turnover
YMR116C	cytoplasmic ribosomal small subunit
YMR117C	Complex Number 371
YMR117C	Ndc80 protein complex
YMR117C	SPB components
YMR120C	Complex Number 244
YMR120C	Complex Number 378
YMR121C	cytoplasmic ribosomal large subunit
YMR125W	Complex Number 27, Cbc2/Sto1 (2)
YMR125W	Complex Number 53, Kap95/Srp1 (2)
YMR125W	mRNA splicing
YMR128W	Complex Number 104, probably protein synthesis turnover
YMR128W	Complex Number 109, probably protein synthesis turnover
YMR128W	Complex Number 125, probably protein/RNA transport
YMR128W	Complex Number 134, probably RNA metabolism
YMR128W	Complex Number 141, probably RNA metabolism
YMR128W	Complex Number 144, probably RNA metabolism
YMR128W	Complex Number 208, probably transcription/DNA maintanance/chromatin structure
YMR128W	Complex Number 66, Ecm16 (1)
YMR129W	Nuclear pore complex (NPC)
YMR131C	Complex Number 26, Rrb1/Rpl3 (2)
YMR135C	Complex Number 278
YMR135C	Complex Number 427
YMR135C	Complex Number 437
YMR137C	Complex Number 245
YMR137C	Complex Number 246
YMR138W	Tubulin-associated proteins
YMR139W	Complex Number 232, probably transcription/DNA maintanance/chromatin structure
YMR139W	Complex Number 308
YMR142C	cytoplasmic ribosomal large subunit
YMR143W	cytoplasmic ribosomal small subunit
YMR144W	Complex Number 101
YMR145C	Complex Number 111

YMR145C	Complex Number 146
YMR145C	Complex Number 353
YMR145C	other respiration chain complexes
YMR146C	Complex Number 107, probably protein synthesis turnover
YMR146C	Complex Number 207
YMR146C	Complex Number 338
YMR146C	Complex Number 379
YMR146C	Complex Number 395
YMR146C	Complex Number 44, probably intermediate and energy metabolism
YMR146C	Complex Number 51, eIF3 (7)
YMR146C	Complex Number 535
YMR146C	eIF3
YMR149W	Complex Number 44, probably intermediate and energy metabolism
YMR149W	Oligosaccharyltransferase
YMR150C	Inner membrane protease
YMR153W	Complex Number 389
YMR155W	Complex Number 347
YMR158W	Complex Number 104, probably protein synthesis turnover
YMR158W	mitochondrial ribosomal small subunit
YMR163C	Complex Number 149, probably RNA metabolism
YMR167W	Complex Number 188
YMR167W	Complex Number 212, probably transcription/DNA maintanance/chromatin structure
YMR167W	Complex Number 276
YMR167W	MLH1/PMS1 complex
YMR168C	CBF3 protein complex
YMR172W	Complex Number 189, probably transcription/DNA maintanance/chromatin structure
YMR172W	Complex Number 56
YMR176W	Complex Number 204, probably transcription/DNA maintanance/chromatin structure
YMR181C	Complex Number 205, probably transcription/DNA maintanance/chromatin structure
YMR183C	t-SNAREs
YMR186W	Complex Number 104, probably protein synthesis turnover
YMR186W	Complex Number 171, probably signalling
YMR186W	Complex Number 175, probably signalling
YMR186W	Complex Number 17, probably cell polarity and structure
YMR186W	Complex Number 195, probably transcription/DNA maintanance/chromatin structure
YMR186W	Complex Number 212, probably transcription/DNA maintanance/chromatin structure
YMR186W	Complex Number 34, probably intermediate and energy metabolism
YMR188C	Complex Number 104, probably protein synthesis turnover
YMR188C	mitochondrial ribosomal small subunit
YMR189W	Glycine decarboxylase
YMR190C	Complex Number 212, probably transcription/DNA maintanance/chromatin structure
YMR190C	Complex Number 429
YMR193W	Complex Number 108, probably protein synthesis turnover
YMR193W	mitochondrial ribosomal large subunit

YMR194W	cytoplasmic ribosomal large subunit
YMR196W	Complex Number 353
YMR197C	Complex Number 71, probably membrane biogenesis and traffic
YMR197C	v-SNAREs
YMR198W	Complex Number 55
YMR198W	Kinesin-related motorproteins
YMR198W	SPB associated proteins
YMR199W	Cdc28p complexes
YMR199W	Complex Number 58
YMR199W	Complex Number 60
YMR199W	Complex Number 6, probably cell cycle
YMR201C	Complex Number 259
YMR201C	Complex Number 260
YMR201C	NEF1 complex
YMR203W	Complex Number 128, probably protein/RNA transport
YMR203W	TOM - transport across the outer membrane
YMR205C	Complex Number 103
YMR205C	Complex Number 196, probably transcription/DNA maintanance/chromatin structure
YMR205C	Complex Number 214, probably transcription/DNA maintanance/chromatin structure
YMR205C	Complex Number 222
YMR205C	Complex Number 244
YMR205C	Complex Number 28
YMR205C	Complex Number 353
YMR205C	Complex Number 61, probably intermediate and energy metabolism
YMR205C	Phosphofructokinase
YMR209C	Complex Number 210
YMR209C	Complex Number 334
YMR213W	Complex Number 143, probably RNA metabolism
YMR213W	Complex Number 147, probably RNA metabolism
YMR213W	Complex Number 241
YMR213W	Complex Number 55, Prp19 (4)
YMR214W	Complex Number 79, probably membrane biogenesis and traffic
YMR218C	Complex Number 121
YMR218C	Complex Number 75, probably membrane biogenesis and traffic
YMR218C	TRAPP (Transport Protein Particle) complex
YMR223W	Complex Number 112
YMR223W	Complex Number 207, probably transcription/DNA maintanance/chromatin structure
YMR223W	Complex Number 349
YMR224C	Complex Number 274
YMR224C	Complex Number 275
YMR224C	Complex Number 423
YMR224C	MRE11/RAD50/XRS2 complex
YMR225C	Complex Number 108, probably protein synthesis turnover
YMR225C	mitochondrial ribosomal large subunit

YMR226C	Complex Number 201
YMR226C	Complex Number 23
YMR226C	Complex Number 261
YMR226C	Complex Number 267
YMR226C	Complex Number 300
YMR226C	Complex Number 350
YMR226C	Complex Number 388
YMR226C	Complex Number 435
YMR226C	Complex Number 504
YMR226C	Complex Number 90
YMR227C	Complex Number 207, probably transcription/DNA maintanance/chromatin structure
YMR227C	Complex Number 214, probably transcription/DNA maintanance/chromatin structure
YMR227C	TAFIIs
YMR229C	Complex Number 17, Pab1 (1)
YMR229C	Complex Number 204
YMR229C	Complex Number 253
YMR229C	Complex Number 305
YMR229C	Complex Number 370
YMR229C	Complex Number 446
YMR229C	Complex Number 475
YMR229C	Complex Number 480
YMR229C	Complex Number 493
YMR229C	Complex Number 56
YMR229C	Complex Number 7, Dbp7/Rrp5 (4)
YMR230W	cytoplasmic ribosomal small subunit
YMR231W	Class C Vps protein complex
YMR231W	Complex Number 77, probably membrane biogenesis and traffic
YMR234W	Complex Number 212, probably transcription/DNA maintanance/chromatin structure
YMR234W	RNase H1
YMR235C	Complex Number 120
YMR235C	Complex Number 312
YMR235C	Complex Number 51, probably intermediate and energy metabolism
YMR235C	Complex Number 549
YMR236W	Complex Number 207, probably transcription/DNA maintanance/chromatin structure
YMR236W	Complex Number 214, probably transcription/DNA maintanance/chromatin structure
YMR236W	SAGA complex
YMR236W	TAFIIs
YMR239C	Complex Number 376
YMR239C	Complex Number 79, Rnt1 (1)
YMR239C	rRNA splicing
YMR240C	Complex Number 145, probably RNA metabolism
YMR240C	Complex Number 146, probably RNA metabolism
YMR240C	Complex Number 147, probably RNA metabolism
YMR241W	Complex Number 111

YMR241W	Complex Number 146
YMR241W	Complex Number 156
YMR242C	cytoplasmic ribosomal large subunit
YMR246W	Complex Number 109
YMR246W	Complex Number 134
YMR246W	Complex Number 174
YMR246W	Complex Number 353
YMR246W	Complex Number 389
YMR246W	Complex Number 43
YMR246W	Complex Number 459
YMR246W	Complex Number 89
YMR246W	Complex Number 97
YMR251W	Complex Number 29, probably intermediate and energy metabolism
YMR256C	Cytochrome c oxidase (complex IV)
YMR257C	Mitochondrial translation complexes
YMR260C	eIF1
YMR261C	Complex Number 29, probably intermediate and energy metabolism
YMR263W	Complex Number 208, probably transcription/DNA maintanance/chromatin structure
YMR263W	HDB complex
YMR266W	Complex Number 341
YMR267W	Complex Number 55, probably intermediate and energy metabolism
YMR268C	Complex Number 138, probably RNA metabolism
YMR268C	Complex Number 147, probably RNA metabolism
YMR268C	Complex Number 14, U6-specific snRNP core (14)
YMR268C	Complex Number 163
YMR268C	mRNA splicing
YMR270C	Upstream Activation Factor (UAF) complex
YMR273C	Complex Number 170, probably signalling
YMR275C	Complex Number 187
YMR275C	Complex Number 322
YMR275C	Complex Number 323
YMR276W	Complex Number 413
YMR282C	Complex Number 115, probably protein synthesis turnover
YMR282C	Mitochondrial translation complexes
YMR284W	Complex Number 187, probably transcription/DNA maintanance/chromatin structure
YMR284W	Complex Number 501
YMR284W	Complex Number 502
YMR284W	Complex Number 503
YMR284W	Ku complex
YMR285C	Complex Number 316
YMR286W	mitochondrial ribosomal large subunit
YMR287C	Complex Number 504
YMR287C	Complex Number 87
YMR287C	mitochondrial 3'-to-5' exoribonuclease (mtEXO)

YMR288W	Complex Number 143, probably RNA metabolism
YMR288W	Complex Number 145, probably RNA metabolism
YMR288W	Complex Number 146, probably RNA metabolism
YMR288W	Complex Number 147, probably RNA metabolism
YMR290C	Complex Number 125, probably protein/RNA transport
YMR290C	Complex Number 140, probably RNA metabolism
YMR290C	Complex Number 149, probably RNA metabolism
YMR290C	Complex Number 152
YMR290C	Complex Number 369
YMR290C	Complex Number 376
YMR290C	Complex Number 388
YMR290C	Complex Number 446
YMR290C	Complex Number 475
YMR290C	Complex Number 517
YMR290C	Complex Number 520
YMR290C	Complex Number 56
YMR290C	Complex Number 69, probably membrane biogenesis and traffic
YMR290C	Complex Number 94
YMR291W	Complex Number 522
YMR294W	Dynactin complex
YMR294W	SPB associated proteins
YMR296C	Complex Number 123, probably protein/RNA transport
YMR300C	Complex Number 125, probably protein/RNA transport
YMR300C	Complex Number 144, probably RNA metabolism
YMR300C	Complex Number 51, probably intermediate and energy metabolism
YMR302C	Complex Number 467
YMR302C	Complex Number 478
YMR302C	rRNA splicing
YMR303C	Complex Number 102
YMR303C	Complex Number 139
YMR303C	Complex Number 157
YMR303C	Complex Number 200
YMR303C	Complex Number 22
YMR303C	Complex Number 302
YMR303C	Complex Number 316
YMR303C	Complex Number 435
YMR303C	Complex Number 444
YMR303C	Complex Number 45
YMR303C	Complex Number 507
YMR303C	Complex Number 508
YMR303C	Complex Number 71
YMR303C	Complex Number 87
YMR304W	Complex Number 164
YMR304W	Complex Number 240

YMR304W	Complex Number 29
YMR304W	Complex Number 381
YMR304W	Complex Number 424
YMR304W	Complex Number 467
YMR304W	Complex Number 52
YMR304W	Complex Number 96, probably protein synthesis turnover
YMR304W	Complex Number 99
YMR307W	Complex Number 132
YMR307W	Complex Number 350
YMR308C	Complex Number 102, probably protein synthesis turnover
YMR308C	Complex Number 120, probably protein/RNA transport
YMR308C	Complex Number 123
YMR308C	Complex Number 141
YMR308C	Complex Number 165, probably signalling
YMR308C	Complex Number 171, probably signalling
YMR308C	Complex Number 204, probably transcription/DNA maintanance/chromatin structure
YMR308C	Complex Number 372
YMR308C	Complex Number 404
YMR308C	Complex Number 487
YMR309C	Complex Number 107, probably protein synthesis turnover
YMR309C	Complex Number 135, probably RNA metabolism
YMR309C	Complex Number 140, probably RNA metabolism
YMR309C	Complex Number 149, probably RNA metabolism
YMR309C	Complex Number 17, probably cell polarity and structure
YMR309C	Complex Number 189, probably transcription/DNA maintanance/chromatin structure
YMR309C	Complex Number 204, probably transcription/DNA maintanance/chromatin structure
YMR309C	Complex Number 379
YMR309C	Complex Number 469
YMR309C	Complex Number 51, eIF3 (7)
YMR309C	Complex Number 535
YMR309C	Complex Number 9
YMR309C	Complex Number 95, probably protein synthesis turnover
YMR309C	Complex Number 99, probably protein synthesis turnover
YMR309C	eIF3
YMR310C	Complex Number 102, probably protein synthesis turnover
YMR310C	Complex Number 140, probably RNA metabolism
YMR310C	Complex Number 142, probably RNA metabolism
YMR310C	Complex Number 5, Gar1/Cbf5 (6)
YMR311C	Complex Number 115
YMR311C	Complex Number 116
YMR311C	Complex Number 237
YMR311C	Serine/threonine phoshpoprotein phosphatase
YMR314W	20S proteasome
YMR314W	Complex Number 102, probably protein synthesis turnover

YMR314W	Complex Number 110, probably protein synthesis turnover
YMR314W	Complex Number 111, probably protein synthesis turnover
YMR314W	Complex Number 170, probably signalling
YMR314W	Complex Number 238
YMR314W	Complex Number 60, 20S Proteosome (13)
YMR315W	Complex Number 103
YMR315W	Complex Number 230, probably transcription/DNA maintanance/chromatin structure
YMR318C	Complex Number 267
YMR318C	Complex Number 350
YMR319C	Complex Number 112
YMR319C	Complex Number 152
YMR319C	Complex Number 16
YMR319C	Complex Number 205
YMR319C	Complex Number 216
YMR319C	Complex Number 253
YMR319C	Complex Number 290
YMR319C	Complex Number 369
YMR319C	Complex Number 4
YMR319C	Complex Number 400
YMR319C	Complex Number 426
YMR319C	Complex Number 449
YMR319C	Complex Number 463
YMR319C	Complex Number 511
YMR319C	Complex Number 512
YMR319C	Complex Number 517
YMR323W	Complex Number 179
YNL002C	Complex Number 140, probably RNA metabolism
YNL002C	Complex Number 149, probably RNA metabolism
YNL002C	Complex Number 520
YNL002C	Complex Number 94
YNL004W	Complex Number 489
YNL004W	Complex Number 74
YNL005C	Complex Number 108, probably protein synthesis turnover
YNL005C	Complex Number 140, probably RNA metabolism
YNL005C	Complex Number 142, probably RNA metabolism
YNL005C	Complex Number 147, probably RNA metabolism
YNL005C	mitochondrial ribosomal large subunit
YNL006W	Complex Number 166
YNL006W	Complex Number 504
YNL007C	Complex Number 181
YNL007C	Complex Number 29
YNL007C	Complex Number 360
YNL007C	Complex Number 505
YNL007C	Complex Number 89, probably protein synthesis turnover

YNL008C	Complex Number 531
YNL014W	Complex Number 117
YNL014W	Complex Number 182
YNL014W	Complex Number 23
YNL014W	Complex Number 296
YNL014W	Complex Number 298
YNL014W	Complex Number 341
YNL014W	Complex Number 37
YNL014W	Complex Number 439
YNL014W	Complex Number 79
YNL014W	Complex Number 95
YNL016W	Complex Number 155, probably RNA metabolism
YNL016W	Complex Number 344
YNL021W	Complex Number 149, probably RNA metabolism
YNL021W	Complex Number 212, probably transcription/DNA maintanance/chromatin structure
YNL021W	Complex Number 218, probably transcription/DNA maintanance/chromatin structure
YNL021W	HDA complex
YNL023C	Complex Number 103, probably protein synthesis turnover
YNL023C	Complex Number 206, probably transcription/DNA maintanance/chromatin structure
YNL023C	Complex Number 98
YNL025C	Complex Number 209, probably transcription/DNA maintanance/chromatin structure
YNL025C	Complex Number 435
YNL025C	Srb10p complex
YNL027W	Complex Number 132
YNL029C	Complex Number 107, probably protein synthesis turnover
YNL030W	Complex Number 101, probably protein synthesis turnover
YNL030W	Complex Number 125, probably protein/RNA transport
YNL030W	Complex Number 135, probably RNA metabolism
YNL030W	Complex Number 142, probably RNA metabolism
YNL030W	Complex Number 144, probably RNA metabolism
YNL030W	Complex Number 148, probably RNA metabolism
YNL030W	Complex Number 170, probably signalling
YNL030W	Complex Number 188, probably transcription/DNA maintanance/chromatin structure
YNL030W	Complex Number 189, probably transcription/DNA maintanance/chromatin structure
YNL030W	Complex Number 191, probably transcription/DNA maintanance/chromatin structure
YNL030W	Complex Number 200, probably transcription/DNA maintanance/chromatin structure
YNL030W	Complex Number 204, probably transcription/DNA maintanance/chromatin structure
YNL030W	Complex Number 69, probably membrane biogenesis and traffic
YNL030W	Complex Number 8, probably cell cycle
YNL030W	Complex Number 99, probably protein synthesis turnover
YNL030W	Nucleosomal protein complex
YNL031C	Nucleosomal protein complex
YNL032W	Complex Number 354
YNL032W	Complex Number 527

YNL035C	Complex Number 146
YNL035C	Complex Number 523
YNL037C	Complex Number 118
YNL037C	Complex Number 134
YNL037C	Complex Number 187
YNL037C	Complex Number 207
YNL037C	Complex Number 328
YNL037C	Complex Number 338
YNL037C	Complex Number 360
YNL037C	Complex Number 374
YNL037C	Complex Number 504
YNL037C	Complex Number 505
YNL037C	Complex Number 508
YNL037C	Complex Number 64
YNL037C	Isocitrate dehydrogenase
YNL038W	Complex Number 22
YNL038W	Complex Number 507
YNL039W	TFIIIB
YNL040W	Complex Number 9
YNL041C	Golgi transport complex
YNL045W	Complex Number 33, probably intermediate and energy metabolism
YNL051W	Golgi transport complex
YNL052W	Complex Number 47, probably intermediate and energy metabolism
YNL052W	Cytochrome c oxidase (complex IV)
YNL053W	Complex Number 194
YNL055C	Complex Number 119
YNL055C	Complex Number 142
YNL055C	Complex Number 168
YNL055C	Complex Number 23
YNL055C	Complex Number 305
YNL055C	Complex Number 320
YNL055C	Complex Number 326
YNL055C	Complex Number 334
YNL055C	Complex Number 435
YNL055C	Complex Number 442
YNL055C	Complex Number 47
YNL055C	Complex Number 504
YNL055C	Complex Number 511
YNL055C	Complex Number 531
YNL055C	Complex Number 535
YNL055C	Complex Number 548
YNL055C	Complex Number 72
YNL055C	Complex Number 84
YNL056W	Complex Number 524

YNL061W	Complex Number 140, probably RNA metabolism
YNL061W	Complex Number 149, probably RNA metabolism
YNL061W	Complex Number 161
YNL061W	Complex Number 171
YNL061W	Complex Number 200
YNL061W	Complex Number 205
YNL061W	Complex Number 310
YNL061W	Complex Number 446
YNL061W	Complex Number 480
YNL061W	Complex Number 520
YNL061W	Complex Number 56
YNL061W	Complex Number 94
YNL062C	eIF3
YNL063W	Complex Number 123
YNL064C	Complex Number 119, probably protein/RNA transport
YNL064C	Complex Number 231, probably transcription/DNA maintanance/chromatin structure
YNL067W	cytoplasmic ribosomal large subunit
YNL068C	Complex Number 102
YNL069C	cytoplasmic ribosomal large subunit
YNL070W	TOM - transport across the outer membrane
YNL071W	Complex Number 42, probably intermediate and energy metabolism
YNL071W	Pyruvate dehydrogenase
YNL073W	Complex Number 35
YNL075W	Complex Number 109, probably protein synthesis turnover
YNL075W	Complex Number 125, probably protein/RNA transport
YNL075W	Complex Number 144, probably RNA metabolism
YNL076W	Complex Number 29, probably intermediate and energy metabolism
YNL078W	Complex Number 224, probably transcription/DNA maintanance/chromatin structure
YNL078W	Complex Number 93
YNL079C	Actin-associated proteins
YNL082W	Complex Number 212, probably transcription/DNA maintanance/chromatin structure
YNL082W	MSH2/MSH3 complex
YNL084C	Complex Number 18, probably cell polarity and structure
YNL085W	Complex Number 111
YNL085W	Complex Number 134
YNL085W	Complex Number 152
YNL085W	Complex Number 195, probably transcription/DNA maintanance/chromatin structure
YNL085W	Complex Number 200
YNL085W	Complex Number 233
YNL085W	Complex Number 266
YNL085W	Complex Number 274
YNL085W	Complex Number 305
YNL085W	Complex Number 313
YNL085W	Complex Number 320

YNL085W	Complex Number 360
YNL085W	Complex Number 474
YNL088W	Complex Number 135, probably RNA metabolism
YNL088W	Complex Number 205, probably transcription/DNA maintanance/chromatin structure
YNL088W	Complex Number 34
YNL088W	Complex Number 397
YNL088W	Complex Number 398
YNL088W	Complex Number 551
YNL088W	Synaptonemal complex (SC)
YNL088W	Topoisomerases
YNL090W	Complex Number 305
YNL093W	Complex Number 418
YNL093W	Complex Number 435
YNL093W	Complex Number 548
YNL094W	Complex Number 525
YNL094W	Complex Number 526
YNL096C	cytoplasmic ribosomal small subunit
YNL097C	Complex Number 208, probably transcription/DNA maintanance/chromatin structure
YNL098C	Complex Number 288
YNL099C	Complex Number 524
YNL099C	Complex Number 527
YNL101W	Complex Number 1, probably cell cycle
YNL102W	Complex Number 211, probably transcription/DNA maintanance/chromatin structure
YNL102W	Complex Number 228, probably transcription/DNA maintanance/chromatin structure
YNL102W	DNA polymerase alpha (I) - primase complex
YNL103W	Cbf1/Met4/Met28 complex
YNL103W	Complex Number 181
YNL103W	Met4/Met28/Met31 complex
YNL103W	Met4/Met28/Met32 complex
YNL106C	Complex Number 142
YNL106C	Complex Number 18, probably cell polarity and structure
YNL107W	Complex Number 191, probably transcription/DNA maintanance/chromatin structure
YNL110C	Complex Number 149, probably RNA metabolism
YNL110C	Complex Number 161
YNL110C	Complex Number 520
YNL110C	Complex Number 94
YNL112W	Complex Number 124, probably protein/RNA transport
YNL112W	Complex Number 142, probably RNA metabolism
YNL112W	Complex Number 56
YNL112W	Complex Number 86, probably protein synthesis turnover
YNL113W	Complex Number 132
YNL113W	Complex Number 213, probably transcription/DNA maintanance/chromatin structure
YNL113W	Complex Number 23, RNA Polymerase III (12)
YNL113W	Complex Number 315

YNL113W	Complex Number 316
YNL113W	Complex Number 486
YNL113W	Complex Number 49
YNL113W	RNA polymerase I
YNL113W	RNA polymerase III
YNL116W	Complex Number 257
YNL116W	Complex Number 482
YNL116W	Complex Number 528
YNL116W	Complex Number 532
YNL118C	Complex Number 132
YNL118C	Complex Number 133
YNL118C	Complex Number 138, probably RNA metabolism
YNL118C	Complex Number 149, probably RNA metabolism
YNL118C	Complex Number 150, probably RNA metabolism
YNL118C	Complex Number 214
YNL118C	Complex Number 215
YNL119W	Complex Number 52, probably intermediate and energy metabolism
YNL121C	TOM - transport across the outer membrane
YNL124W	Complex Number 28
YNL126W	Complex Number 7, probably cell cycle
YNL126W	gamma-tubulin complex
YNL126W	Tubulin-associated proteins
YNL127W	Complex Number 34, probably intermediate and energy metabolism
YNL128W	Complex Number 392
YNL131W	TOM - transport across the outer membrane
YNL132W	Complex Number 109, probably protein synthesis turnover
YNL132W	Complex Number 125, probably protein/RNA transport
YNL132W	Complex Number 140, probably RNA metabolism
YNL132W	Complex Number 144, probably RNA metabolism
YNL132W	Complex Number 149, probably RNA metabolism
YNL132W	Complex Number 150
YNL132W	Complex Number 200
YNL132W	Complex Number 204, probably transcription/DNA maintanance/chromatin structure
YNL132W	Complex Number 370
YNL132W	Complex Number 403
YNL132W	Complex Number 446
YNL132W	Complex Number 473
YNL132W	Complex Number 480
YNL132W	Complex Number 493
YNL132W	Complex Number 517
YNL132W	Complex Number 56
YNL132W	Complex Number 6
YNL132W	Complex Number 74
YNL132W	Complex Number 82

YNL132W	Complex Number 91
YNL132W	Complex Number 96
YNL134C	Complex Number 313
YNL135C	Complex Number 103
YNL135C	Complex Number 206, probably transcription/DNA maintanance/chromatin structure
YNL135C	Complex Number 341
YNL135C	Complex Number 545
YNL135C	Complex Number 98
YNL137C	Complex Number 104, probably protein synthesis turnover
YNL137C	mitochondrial ribosomal small subunit
YNL137C	Mitochondrial ribosomes
YNL138W	Actin-associated proteins
YNL138W	Complex Number 130, probably RNA metabolism
YNL138W	Complex Number 167, probably signalling
YNL138W	Complex Number 372
YNL138W	Complex Number 454
YNL138W	Complex Number 490
YNL138W	Complex Number 511
YNL138W	Complex Number 535
YNL138W	Complex Number 68
YNL138W	Complex Number 70, probably membrane biogenesis and traffic
YNL138W	Complex Number 76
YNL139C	Complex Number 106
YNL139C	Complex Number 128
YNL139C	Complex Number 129
YNL139C	Complex Number 139, probably RNA metabolism
YNL139C	Complex Number 31, TREX (5)
YNL139C	Complex Number 376
YNL139C	Complex Number 435
YNL139C	Complex Number 53, Kap95/Srp1 (2)
YNL141W	Complex Number 434
YNL141W	Complex Number 61, Aah1 (1)
YNL147W	Complex Number 138, probably RNA metabolism
YNL147W	Complex Number 147, probably RNA metabolism
YNL147W	Complex Number 163
YNL147W	Complex Number 215
YNL151C	Complex Number 213, probably transcription/DNA maintanance/chromatin structure
YNL151C	Complex Number 23, RNA Polymerase III (12)
YNL151C	RNA polymerase III
YNL153C	Complex Number 7, probably cell cycle
YNL153C	Gim complexes
YNL154C	Casein kinase I
YNL154C	Complex Number 435
YNL154C	Complex Number 436

YNL154C	Complex Number 48
YNL157W	Complex Number 529
YNL160W	Complex Number 134
YNL161W	Complex Number 192
YNL161W	Complex Number 193
YNL161W	Complex Number 29
YNL162W	cytoplasmic ribosomal large subunit
YNL169C	Complex Number 146
YNL172W	Anaphase promoting complex (APC)
YNL172W	Complex Number 3, probably cell cycle
YNL172W	Complex Number 435
YNL175C	Complex Number 204
YNL175C	Complex Number 22
YNL175C	Complex Number 46
YNL175C	Complex Number 507
YNL177C	mitochondrial ribosomal large subunit
YNL178W	cytoplasmic ribosomal small subunit
YNL180C	Complex Number 231
YNL180C	Complex Number 307
YNL181W	Complex Number 338
YNL182C	Complex Number 149, probably RNA metabolism
YNL182C	Complex Number 486
YNL182C	Complex Number 530
YNL182C	Complex Number 67, Ipi (3)
YNL185C	Complex Number 108, probably protein synthesis turnover
YNL185C	mitochondrial ribosomal large subunit
YNL186W	Complex Number 104, probably protein synthesis turnover
YNL187W	Complex Number 1, probably cell cycle
YNL188W	SPB components
YNL189W	Complex Number 102, probably protein synthesis turnover
YNL189W	Complex Number 134, probably RNA metabolism
YNL189W	Complex Number 139, probably RNA metabolism
YNL189W	Complex Number 141, probably RNA metabolism
YNL189W	Complex Number 170, probably signalling
YNL189W	Complex Number 209, probably transcription/DNA maintanance/chromatin structure
YNL189W	Complex Number 219
YNL189W	Complex Number 279
YNL189W	Complex Number 28
YNL189W	Complex Number 298
YNL189W	Complex Number 303
YNL189W	Complex Number 311
YNL189W	Complex Number 332
YNL189W	Complex Number 351
YNL189W	Complex Number 352

YNL189W	Complex Number 369
YNL189W	Complex Number 375
YNL189W	Complex Number 376
YNL189W	Complex Number 444
YNL189W	Complex Number 46
YNL189W	Complex Number 475
YNL189W	Complex Number 506
YNL189W	Complex Number 53, Kap95/Srp1 (2)
YNL189W	Complex Number 83
YNL189W	Complex Number 85
YNL189W	Complex Number 86
YNL189W	Complex Number 87
YNL189W	Complex Number 88
YNL189W	Complex Number 99
YNL192W	Complex Number 151
YNL199C	Complex Number 308
YNL199C	GCR complex
YNL201C	Complex Number 151
YNL201C	Complex Number 169, probably signalling
YNL201C	Complex Number 234
YNL201C	Complex Number 23, RNA Polymerase III (12)
YNL207W	Complex Number 109, probably protein synthesis turnover
YNL207W	Complex Number 132
YNL207W	Complex Number 198, probably transcription/DNA maintanance/chromatin structure
YNL208W	Complex Number 4
YNL210W	Complex Number 305
YNL210W	Complex Number 451
YNL213C	Complex Number 110
YNL218W	Complex Number 60, probably intermediate and energy metabolism
YNL221C	Complex Number 154, probably RNA metabolism
YNL221C	Complex Number 78, Rnase P (3)
YNL221C	RNase MRP
YNL221C	RNase P
YNL221C	rRNA splicing
YNL222W	Complex Number 148, probably RNA metabolism
YNL222W	Complex Number 56, mRNA cleavage/polyadenylation (10)
YNL223W	Clathrin
YNL223W	Complex Number 38
YNL223W	Tubulin-associated proteins
YNL224C	Complex Number 138, probably RNA metabolism
YNL225C	SPB components
YNL227C	Complex Number 402
YNL227C	Complex Number 548
YNL229C	Complex Number 101

YNL229C	Complex Number 316
YNL230C	Complex Number 91
YNL232W	Complex Number 134, probably RNA metabolism
YNL232W	Complex Number 141, probably RNA metabolism
YNL232W	Complex Number 22, Exosome (12)
YNL236W	Complex Number 108, probably protein synthesis turnover
YNL236W	Complex Number 209, probably transcription/DNA maintanance/chromatin structure
YNL236W	Complex Number 214, probably transcription/DNA maintanance/chromatin structure
YNL236W	Complex Number 33, Mediator (8)
YNL236W	Kornberg's mediator (SRB) complex
YNL238W	Complex Number 16
YNL242W	Complex Number 203
YNL242W	Complex Number 58, probably intermediate and energy metabolism
YNL243W	Actin-associated proteins
YNL243W	Complex Number 18, probably cell polarity and structure
YNL243W	Complex Number 20, probably cell polarity and structure
YNL244C	Complex Number 107, probably protein synthesis turnover
YNL244C	Complex Number 379
YNL244C	eIF3
YNL248C	Complex Number 213, probably transcription/DNA maintanance/chromatin structure
YNL248C	Complex Number 24, RNA Polymerase II (12)
YNL248C	Complex Number 25, RNA polymerase I (7)
YNL248C	Complex Number 316
YNL248C	RNA polymerase I
YNL250W	Complex Number 176, probably signalling
YNL250W	Complex Number 274
YNL250W	Complex Number 275
YNL250W	Complex Number 422
YNL250W	Complex Number 423
YNL250W	Complex Number 424
YNL250W	MRE11/RAD50/XRS2 complex
YNL251C	Complex Number 139, probably RNA metabolism
YNL251C	Complex Number 155, probably RNA metabolism
YNL251C	Complex Number 197, probably transcription/DNA maintanance/chromatin structure
YNL251C	Complex Number 208, probably transcription/DNA maintanance/chromatin structure
YNL252C	Complex Number 108, probably protein synthesis turnover
YNL252C	mitochondrial ribosomal large subunit
YNL253W	Complex Number 106
YNL253W	Complex Number 31, TREX (5)
YNL260C	Complex Number 531
YNL260C	Complex Number 54, Rli1/Rpp0 (3)
YNL261W	Complex Number 10, probably cell cycle
YNL261W	Post-replication complex (Origin recognition complex=ORC)
YNL261W	Pre-replication complex (pre-RC)

YNL261W	Replication complex
YNL261W	Replication initiation complex
YNL262W	Complex Number 140, probably RNA metabolism
YNL262W	Complex Number 142, probably RNA metabolism
YNL262W	Complex Number 155, probably RNA metabolism
YNL262W	Complex Number 190, probably transcription/DNA maintanance/chromatin structure
YNL262W	Complex Number 205, probably transcription/DNA maintanance/chromatin structure
YNL262W	Complex Number 220, probably transcription/DNA maintanance/chromatin structure
YNL262W	Complex Number 56
YNL262W	DNA polymerase epsilon (II)
YNL262W	Replication complex
YNL265C	Complex Number 105, probably protein synthesis turnover
YNL267W	Complex Number 161, probably signalling
YNL271C	Complex Number 112, probably protein synthesis turnover
YNL271C	Complex Number 13
YNL271C	Complex Number 9, probably cell cycle
YNL272C	Complex Number 101
YNL272C	Complex Number 132
YNL278W	Complex Number 71
YNL281W	Complex Number 62
YNL282W	Complex Number 154, probably RNA metabolism
YNL282W	Complex Number 78, Rnase P (3)
YNL282W	RNase MRP
YNL282W	RNase P
YNL284C	Complex Number 108, probably protein synthesis turnover
YNL284C	Complex Number 152, probably RNA metabolism
YNL284C	mitochondrial ribosomal large subunit
YNL287W	Complex Number 125, probably protein/RNA transport
YNL287W	Complex Number 144, probably RNA metabolism
YNL287W	Complex Number 338
YNL287W	Complex Number 69
YNL287W	Complex Number 79, probably membrane biogenesis and traffic
YNL287W	COPI
YNL288W	Complex Number 211, probably transcription/DNA maintanance/chromatin structure
YNL289W	Pho85p complexes
YNL290W	Complex Number 205, probably transcription/DNA maintanance/chromatin structure
YNL290W	Complex Number 228, probably transcription/DNA maintanance/chromatin structure
YNL290W	Complex Number 264
YNL290W	Complex Number 265
YNL290W	Complex Number 297
YNL290W	Complex Number 299
YNL290W	Complex Number 300
YNL290W	Complex Number 64
YNL290W	Replication factor C complex

YNL292W	Complex Number 75, Pus4 (1)
YNL301C	cytoplasmic ribosomal large subunit
YNL302C	cytoplasmic ribosomal small subunit
YNL306W	Complex Number 104, probably protein synthesis turnover
YNL306W	mitochondrial ribosomal small subunit
YNL307C	Complex Number 173
YNL308C	Complex Number 137
YNL308C	Complex Number 14
YNL308C	Complex Number 200
YNL308C	Complex Number 204
YNL308C	Complex Number 316
YNL308C	Complex Number 446
YNL308C	Complex Number 517
YNL308C	Complex Number 56
YNL308C	Complex Number 6
YNL308C	Complex Number 82
YNL309W	Sin3 complex
YNL311C	Complex Number 482
YNL311C	Complex Number 528
YNL311C	Complex Number 532
YNL312W	Complex Number 202, probably transcription/DNA maintanance/chromatin structure
YNL312W	Complex Number 212, probably transcription/DNA maintanance/chromatin structure
YNL312W	Complex Number 294
YNL312W	Complex Number 295
YNL312W	Replication factor A complex
YNL313C	Complex Number 35, probably intermediate and energy metabolism
YNL313C	Complex Number 36, Ynl313c (1)
YNL313C	Complex Number 96, probably protein synthesis turnover
YNL315C	Complex Number 211, probably transcription/DNA maintanance/chromatin structure
YNL317W	Complex Number 125, probably protein/RNA transport
YNL317W	Complex Number 148, probably RNA metabolism
YNL317W	Complex Number 223
YNL317W	Complex Number 56, mRNA cleavage/polyadenylation (10)
YNL323W	Complex Number 158
YNL329C	Complex Number 518
YNL330C	Complex Number 10, probably cell cycle
YNL330C	Complex Number 208, probably transcription/DNA maintanance/chromatin structure
YNL330C	Complex Number 415
YNL330C	HDB complex
YNL330C	Sin3 complex
YNL334C	Complex Number 435
YNR001C	other respiration chain complexes
YNR003C	Complex Number 200, probably transcription/DNA maintanance/chromatin structure
YNR003C	Complex Number 213, probably transcription/DNA maintanance/chromatin structure

YNR003C	Complex Number 23, RNA Polymerase III (12)
YNR003C	Complex Number 316
YNR003C	RNA polymerase III
YNR006W	Complex Number 76, probably membrane biogenesis and traffic
YNR007C	Complex Number 122, probably protein/RNA transport
YNR008W	Complex Number 341
YNR010W	Complex Number 209, probably transcription/DNA maintanance/chromatin structure
YNR010W	Complex Number 72
YNR010W	Kornberg's mediator (SRB) complex
YNR011C	Complex Number 143, probably RNA metabolism
YNR011C	mRNA splicing
YNR016C	Complex Number 1, probably cell cycle
YNR016C	Complex Number 316
YNR016C	Complex Number 346
YNR016C	Complex Number 353
YNR016C	Complex Number 40, probably intermediate and energy metabolism
YNR016C	Complex Number 41, probably intermediate and energy metabolism
YNR016C	Complex Number 459
YNR016C	Complex Number 504
YNR016C	Complex Number 84
YNR017W	Tim17p-complex
YNR019W	Complex Number 10
YNR022C	mitochondrial ribosomal large subunit
YNR023W	Complex Number 198, probably transcription/DNA maintanance/chromatin structure
YNR023W	SWI/SNF transcription activator complex
YNR031C	Complex Number 123
YNR031C	Complex Number 126
YNR031C	Complex Number 128
YNR031C	Complex Number 216
YNR031C	Complex Number 240
YNR031C	Complex Number 256
YNR031C	Complex Number 377
YNR031C	Complex Number 99
YNR035C	Arp2p/Arp3p complex
YNR035C	Complex Number 13
YNR035C	Complex Number 19, probably cell polarity and structure
YNR035C	Complex Number 346
YNR035C	Complex Number 348
YNR035C	Complex Number 9
YNR037C	Complex Number 104, probably protein synthesis turnover
YNR037C	mitochondrial ribosomal small subunit
YNR039C	Complex Number 178
YNR043W	Complex Number 125, probably protein/RNA transport
YNR043W	Complex Number 144, probably RNA metabolism

YNR045W	Mitochondrial translation complexes
YNR046W	Complex Number 36, probably intermediate and energy metabolism
YNR047W	Complex Number 148
YNR050C	Complex Number 36, probably intermediate and energy metabolism
YNR050C	Complex Number 37, probably intermediate and energy metabolism
YNR052C	CCR4 complex
YNR052C	Complex Number 147
YNR052C	Complex Number 211, probably transcription/DNA maintanance/chromatin structure
YNR052C	Complex Number 31
YNR052C	Complex Number 65, CCR4 (3)
YNR052C	rRNA splicing
YNR053C	Complex Number 138, probably RNA metabolism
YNR053C	Complex Number 149, probably RNA metabolism
YNR053C	Complex Number 19, probably cell polarity and structure
YNR053C	Complex Number 505
YNR054C	Complex Number 125, probably protein/RNA transport
YNR054C	Complex Number 144, probably RNA metabolism
YNR054C	Complex Number 458
YNR058W	Complex Number 196
YNR058W	Complex Number 212
YNR058W	Complex Number 216
YNR058W	Complex Number 267
YNR065C	Complex Number 155
YOL001W	Pho85p complexes
YOL004W	Complex Number 101
YOL004W	Complex Number 102
YOL004W	Complex Number 10, probably cell cycle
YOL004W	Complex Number 122, probably protein/RNA transport
YOL004W	Complex Number 192, probably transcription/DNA maintanance/chromatin structure
YOL004W	Complex Number 203, probably transcription/DNA maintanance/chromatin structure
YOL004W	Complex Number 208, probably transcription/DNA maintanance/chromatin structure
YOL004W	Complex Number 214, probably transcription/DNA maintanance/chromatin structure
YOL004W	Complex Number 376
YOL004W	Complex Number 415
YOL004W	HDB complex
YOL004W	Sin3 complex
YOL005C	Complex Number 210, probably transcription/DNA maintanance/chromatin structure
YOL005C	Complex Number 233
YOL005C	Complex Number 545
YOL005C	RNA polymerase II
YOL006C	Complex Number 19, mRNA cap-binding/eIF4F (8)
YOL006C	Complex Number 200, probably transcription/DNA maintanance/chromatin structure
YOL006C	Complex Number 208, probably transcription/DNA maintanance/chromatin structure
YOL006C	Complex Number 220, probably transcription/DNA maintanance/chromatin structure

YOL006C	Complex Number 396
YOL006C	Topoisomerases
YOL010W	Complex Number 109, probably protein synthesis turnover
YOL010W	Complex Number 144, probably RNA metabolism
YOL010W	Complex Number 6
YOL010W	Complex Number 76, Rcl1/Bms1 (2)
YOL012C	Complex Number 224, probably transcription/DNA maintanance/chromatin structure
YOL012C	Complex Number 261
YOL016C	Complex Number 62
YOL017W	Complex Number 192, probably transcription/DNA maintanance/chromatin structure
YOL017W	Complex Number 203, probably transcription/DNA maintanance/chromatin structure
YOL018C	Complex Number 71, probably membrane biogenesis and traffic
YOL018C	t-SNAREs
YOL021C	Complex Number 127, probably protein/RNA transport
YOL021C	Complex Number 134, probably RNA metabolism
YOL021C	Complex Number 141, probably RNA metabolism
YOL021C	Complex Number 144, probably RNA metabolism
YOL021C	Complex Number 22, Exosome (12)
YOL021C	Complex Number 376
YOL021C	Exosome complex
YOL033W	Complex Number 152
YOL038W	20S proteasome
YOL038W	Complex Number 102, probably protein synthesis turnover
YOL038W	Complex Number 110, probably protein synthesis turnover
YOL038W	Complex Number 111, probably protein synthesis turnover
YOL038W	Complex Number 125, probably protein/RNA transport
YOL038W	Complex Number 144, probably RNA metabolism
YOL038W	Complex Number 170, probably signalling
YOL038W	Complex Number 238
YOL038W	Complex Number 60, 20S Proteosome (13)
YOL039W	cytoplasmic ribosomal large subunit
YOL040C	cytoplasmic ribosomal small subunit
YOL041C	Complex Number 125, probably protein/RNA transport
YOL041C	Complex Number 140, probably RNA metabolism
YOL041C	Complex Number 149, probably RNA metabolism
YOL041C	Complex Number 169
YOL041C	Complex Number 185
YOL041C	Complex Number 200
YOL041C	Complex Number 201
YOL041C	Complex Number 22
YOL041C	Complex Number 375
YOL041C	Complex Number 38, Arx1 Complex (5)
YOL041C	Complex Number 40, Pwp1/Brx1/Nop12 (3)
YOL041C	Complex Number 441

YOL041C	Complex Number 446
YOL041C	Complex Number 458
YOL041C	Complex Number 507
YOL041C	Complex Number 517
YOL041C	Complex Number 56
YOL041C	Complex Number 82
YOL041C	Complex Number 8, probably cell cycle
YOL041C	Complex Number 91
YOL044W	Complex Number 263
YOL045W	Complex Number 105
YOL045W	Complex Number 429
YOL045W	Complex Number 533
YOL051W	Complex Number 199
YOL051W	Complex Number 209, probably transcription/DNA maintanance/chromatin structure
YOL051W	Kornberg's mediator (SRB) complex
YOL054W	Complex Number 204, probably transcription/DNA maintanance/chromatin structure
YOL054W	Complex Number 534
YOL058W	Complex Number 327
YOL059W	Complex Number 257
YOL062C	AP-2 complex
YOL062C	Complex Number 78, probably membrane biogenesis and traffic
YOL062C	Complex Number 8
YOL067C	RTG complex
YOL068C	Complex Number 219, probably transcription/DNA maintanance/chromatin structure
YOL069W	Ndc80 protein complex
YOL069W	SPB associated proteins
YOL070C	Complex Number 224, probably transcription/DNA maintanance/chromatin structure
YOL076W	Complex Number 10, Mdm20/Nat3 (2)
YOL076W	Complex Number 15, Nop1 (1)
YOL077C	Complex Number 140, probably RNA metabolism
YOL077C	Complex Number 144, probably RNA metabolism
YOL077C	Complex Number 149, probably RNA metabolism
YOL077C	Complex Number 150
YOL077C	Complex Number 205
YOL077C	Complex Number 254
YOL077C	Complex Number 40, Pwp1/Brx1/Nop12 (3)
YOL077C	Complex Number 520
YOL077C	Complex Number 6
YOL077C	Complex Number 94
YOL077W-A	F0/F1 ATP synthase (complex V)
YOL078W	Complex Number 135, probably RNA metabolism
YOL078W	Complex Number 152
YOL078W	Complex Number 321
YOL078W	Complex Number 426

YOL078W	Complex Number 505
YOL081W	Complex Number 190
YOL081W	Complex Number 308
YOL082W	Complex Number 154
YOL086C	Complex Number 109, probably protein synthesis turnover
YOL086C	Complex Number 114, probably protein synthesis turnover
YOL086C	Complex Number 136, probably RNA metabolism
YOL086C	Complex Number 171, probably signalling
YOL086C	Complex Number 17, probably cell polarity and structure
YOL086C	Complex Number 191, probably transcription/DNA maintanance/chromatin structure
YOL086C	Complex Number 192, probably transcription/DNA maintanance/chromatin structure
YOL086C	Complex Number 194, probably transcription/DNA maintanance/chromatin structure
YOL086C	Complex Number 196, probably transcription/DNA maintanance/chromatin structure
YOL086C	Complex Number 203, probably transcription/DNA maintanance/chromatin structure
YOL086C	Complex Number 209, probably transcription/DNA maintanance/chromatin structure
YOL086C	Complex Number 214, probably transcription/DNA maintanance/chromatin structure
YOL086C	Complex Number 222, probably transcription/DNA maintanance/chromatin structure
YOL086C	Complex Number 230, probably transcription/DNA maintanance/chromatin structure
YOL086C	Complex Number 34, probably intermediate and energy metabolism
YOL086C	Complex Number 53, probably intermediate and energy metabolism
YOL086C	Complex Number 56, probably intermediate and energy metabolism
YOL086C	Complex Number 58, probably intermediate and energy metabolism
YOL086C	Complex Number 65, probably membrane biogenesis and traffic
YOL086C	Complex Number 70, probably membrane biogenesis and traffic
YOL086C	Complex Number 95, probably protein synthesis turnover
YOL087C	Complex Number 535
YOL090W	Complex Number 169
YOL090W	Complex Number 197
YOL090W	Complex Number 202, probably transcription/DNA maintanance/chromatin structure
YOL090W	Complex Number 212, probably transcription/DNA maintanance/chromatin structure
YOL090W	MSH2/MSH3 complex
YOL090W	MSH2/MSH6 complex
YOL094C	Complex Number 205, probably transcription/DNA maintanance/chromatin structure
YOL094C	Complex Number 228, probably transcription/DNA maintanance/chromatin structure
YOL094C	Complex Number 297
YOL094C	Complex Number 300
YOL094C	Complex Number 301
YOL094C	Complex Number 302
YOL094C	Complex Number 469
YOL094C	Complex Number 81
YOL094C	Replication factor C complex
YOL097C	Complex Number 230, probably transcription/DNA maintanance/chromatin structure
YOL098C	Complex Number 41, probably intermediate and energy metabolism
YOL098C	Complex Number 85, probably protein synthesis turnover

YOL100W	Complex Number 228
YOL102C	Complex Number 403
YOL102C	tRNA splicing
YOL103W	Complex Number 122
YOL108C	Complex Number 102, probably protein synthesis turnover
YOL108C	Complex Number 141
YOL111C	Complex Number 453
YOL113W	Complex Number 356
YOL115W	Complex Number 104, probably protein synthesis turnover
YOL115W	Complex Number 404
YOL115W	Complex Number 489
YOL115W	Complex Number 9, Mtr4 (3)
YOL117W	Complex Number 87, probably protein synthesis turnover
YOL120C	cytoplasmic ribosomal large subunit
YOL121C	cytoplasmic ribosomal small subunit
YOL123W	Complex Number 115, probably protein synthesis turnover
YOL123W	Complex Number 123, probably protein/RNA transport
YOL123W	Complex Number 146
YOL123W	pre mRNA3'-end processing factor CFI
YOL124C	Complex Number 36, probably intermediate and energy metabolism
YOL126C	Complex Number 174
YOL127W	cytoplasmic ribosomal large subunit
YOL128C	Complex Number 536
YOL133W	Complex Number 134
YOL133W	Complex Number 181
YOL135C	Complex Number 209, probably transcription/DNA maintanance/chromatin structure
YOL135C	Complex Number 33, Mediator (8)
YOL135C	Kornberg's mediator (SRB) complex
YOL139C	Complex Number 123
YOL139C	Complex Number 138, probably RNA metabolism
YOL139C	Complex Number 142, probably RNA metabolism
YOL139C	Complex Number 146, probably RNA metabolism
YOL139C	Complex Number 147, probably RNA metabolism
YOL139C	Complex Number 19, mRNA cap-binding/eIF4F (8)
YOL139C	Complex Number 200
YOL139C	Complex Number 25
YOL139C	Complex Number 267
YOL139C	Complex Number 327
YOL139C	Complex Number 334
YOL139C	Complex Number 341
YOL139C	Complex Number 380
YOL139C	Complex Number 388
YOL139C	Complex Number 394
YOL139C	Complex Number 43

YOL139C	Complex Number 440
YOL139C	Complex Number 446
YOL139C	Complex Number 447
YOL139C	Complex Number 458
YOL139C	Complex Number 517
YOL139C	Complex Number 540
YOL139C	Complex Number 548
YOL139C	Complex Number 72
YOL139C	Complex Number 76
YOL139C	Complex Number 90
YOL139C	eIF4E
YOL139C	eIF4E/eIF4G/Pab1p complex
YOL142W	Complex Number 105, probably protein synthesis turnover
YOL142W	Complex Number 134, probably RNA metabolism
YOL142W	Complex Number 141, probably RNA metabolism
YOL142W	Complex Number 22, Exosome (12)
YOL145C	Complex Number 134, probably RNA metabolism
YOL145C	Complex Number 135, probably RNA metabolism
YOL145C	Complex Number 201, probably transcription/DNA maintanance/chromatin structure
YOL145C	Complex Number 46, SPT16 (8)
YOL146W	0
YOL148C	ADA complex
YOL148C	ADA complexes
YOL148C	Complex Number 207, probably transcription/DNA maintanance/chromatin structure
YOL148C	Complex Number 214, probably transcription/DNA maintanance/chromatin structure
YOL148C	SAGA complex
YOL149W	Complex Number 132
YOL149W	Complex Number 150, probably RNA metabolism
YOR001W	Complex Number 134, probably RNA metabolism
YOR001W	Complex Number 141, probably RNA metabolism
YOR001W	Complex Number 22, Exosome (12)
YOR001W	Complex Number 26, Rrb1/Rpl3 (2)
YOR001W	Complex Number 376
YOR001W	Complex Number 53, Kap95/Srp1 (2)
YOR001W	Complex Number 9, Mtr4 (3)
YOR005C	Complex Number 160
YOR005C	Complex Number 161
YOR005C	Complex Number 162
YOR005C	DNA ligase IV
YOR007C	Complex Number 29
YOR014W	Complex Number 170, probably signalling
YOR014W	Complex Number 232
YOR014W	Complex Number 233
YOR017W	Complex Number 446

YOR017W	Complex Number 91
YOR018W	Complex Number 289
YOR018W	Complex Number 368
YOR023C	Complex Number 381
YOR026W	Complex Number 537
YOR027W	Complex Number 175
YOR027W	Complex Number 242
YOR027W	Complex Number 266
YOR027W	Complex Number 31
YOR027W	Complex Number 320
YOR027W	Complex Number 338
YOR027W	Complex Number 363
YOR027W	Complex Number 406
YOR027W	Complex Number 454
YOR027W	Complex Number 462
YOR027W	Complex Number 49
YOR027W	Complex Number 504
YOR027W	Complex Number 505
YOR027W	Complex Number 532
YOR028C	Complex Number 513
YOR035C	Complex Number 134, probably RNA metabolism
YOR035C	Complex Number 62
YOR036W	t-SNAREs
YOR038C	Complex Number 137
YOR039W	Casein kinase II
YOR039W	Complex Number 101
YOR039W	Complex Number 144, probably RNA metabolism
YOR039W	Complex Number 189, probably transcription/DNA maintanance/chromatin structure
YOR039W	Complex Number 201, probably transcription/DNA maintanance/chromatin structure
YOR039W	Complex Number 208, probably transcription/DNA maintanance/chromatin structure
YOR039W	Complex Number 467
YOR039W	Complex Number 475
YOR039W	Complex Number 47, Casein Kinase II (4)
YOR039W	Complex Number 48, UTP22/RRP7 (2)
YOR039W	Complex Number 493
YOR039W	Complex Number 56
YOR042W	Complex Number 467
YOR043W	Complex Number 247
YOR043W	Complex Number 419
YOR045W	TOM - transport across the outer membrane
YOR048C	Complex Number 12, Sit4 (6)
YOR048C	Complex Number 157, probably RNA metabolism
YOR048C	Complex Number 86, probably protein synthesis turnover
YOR048C	rRNA splicing

YOR054C	Complex Number 237
YOR054C	Complex Number 49, Ykl088w (3)
YOR056C	Complex Number 109, probably protein synthesis turnover
YOR056C	Complex Number 14
YOR056C	Complex Number 144, probably RNA metabolism
YOR056C	Complex Number 198, probably transcription/DNA maintanance/chromatin structure
YOR056C	Complex Number 441
YOR056C	Complex Number 534
YOR057W	Complex Number 357
YOR057W	SCF-CDC4 complex
YOR058C	Tubulin-associated proteins
YOR059C	Complex Number 144, probably RNA metabolism
YOR061W	Casein kinase II
YOR061W	Complex Number 101
YOR061W	Complex Number 124, probably protein/RNA transport
YOR061W	Complex Number 133
YOR061W	Complex Number 144, probably RNA metabolism
YOR061W	Complex Number 159
YOR061W	Complex Number 181, probably transcription/DNA maintanance/chromatin structure
YOR061W	Complex Number 189, probably transcription/DNA maintanance/chromatin structure
YOR061W	Complex Number 201, probably transcription/DNA maintanance/chromatin structure
YOR061W	Complex Number 208, probably transcription/DNA maintanance/chromatin structure
YOR061W	Complex Number 334
YOR061W	Complex Number 375
YOR061W	Complex Number 398
YOR061W	Complex Number 428
YOR061W	Complex Number 458
YOR061W	Complex Number 46, SPT16 (8)
YOR061W	Complex Number 475
YOR061W	Complex Number 47, Casein Kinase II (4)
YOR061W	Complex Number 493
YOR061W	Complex Number 56
YOR061W	Complex Number 57
YOR063W	Complex Number 26, Rrb1/Rpl3 (2)
YOR063W	cytoplasmic ribosomal large subunit
YOR065W	Cytochrome bc1 complex (Ubiquinol-cytochrome c reductase complex, complex III)
YOR069W	Complex Number 197, probably transcription/DNA maintanance/chromatin structure
YOR069W	Vps5/Vps17 complex
YOR073W	Complex Number 23
YOR075W	t-SNAREs
YOR076C	Complex Number 134, probably RNA metabolism
YOR076C	Complex Number 141, probably RNA metabolism
YOR076C	Complex Number 22, Exosome (12)
YOR078W	Complex Number 125, probably protein/RNA transport

YOR078W	Complex Number 144, probably RNA metabolism
YOR080W	Complex Number 82
YOR085W	Oligosaccharyltransferase
YOR086C	Complex Number 250
YOR086C	Complex Number 4
YOR086C	Complex Number 41, probably intermediate and energy metabolism
YOR089C	Complex Number 113
YOR089C	Complex Number 305
YOR089C	Complex Number 418
YOR089C	Complex Number 435
YOR089C	Complex Number 548
YOR090C	Complex Number 252
YOR090C	Complex Number 493
YOR096W	cytoplasmic ribosomal small subunit
YOR098C	Complex Number 376
YOR098C	Nuclear pore complex (NPC)
YOR100C	Complex Number 198
YOR101W	Complex Number 288
YOR103C	Oligosaccharyltransferase
YOR106W	Vam3/Vam7 vacuolar t-SNARE complex
YOR110W	Complex Number 178, probably transcription/DNA maintanance/chromatin structure
YOR110W	Complex Number 200, probably transcription/DNA maintanance/chromatin structure
YOR110W	Complex Number 211, probably transcription/DNA maintanance/chromatin structure
YOR110W	Complex Number 34, TFIIIC (4)
YOR110W	Complex Number 505
YOR110W	TFIIIC
YOR112W	Complex Number 41, Nap1 (3)
YOR112W	Complex Number 45, Yor112w (1)
YOR115C	Complex Number 75, probably membrane biogenesis and traffic
YOR115C	TRAPP (Transport Protein Particle) complex
YOR116C	Complex Number 137
YOR116C	Complex Number 200, probably transcription/DNA maintanance/chromatin structure
YOR116C	Complex Number 213, probably transcription/DNA maintanance/chromatin structure
YOR116C	Complex Number 220, probably transcription/DNA maintanance/chromatin structure
YOR116C	Complex Number 230
YOR116C	Complex Number 23, RNA Polymerase III (12)
YOR116C	Complex Number 25, RNA polymerase I (7)
YOR116C	Complex Number 316
YOR116C	Complex Number 382
YOR116C	Complex Number 9, probably cell cycle
YOR116C	RNA polymerase III
YOR117W	19/22S regulator
YOR117W	Complex Number 102, probably protein synthesis turnover
YOR117W	Complex Number 110, probably protein synthesis turnover

YOR117W	Complex Number 111, probably protein synthesis turnover
YOR117W	Complex Number 196, probably transcription/DNA maintanance/chromatin structure
YOR117W	Complex Number 204, probably transcription/DNA maintanance/chromatin structure
YOR117W	Complex Number 214, probably transcription/DNA maintanance/chromatin structure
YOR117W	Complex Number 320
YOR117W	Complex Number 35, probably intermediate and energy metabolism
YOR117W	Complex Number 41, probably intermediate and energy metabolism
YOR117W	Complex Number 468
YOR117W	Complex Number 77
YOR120W	Complex Number 244
YOR122C	Actin-associated proteins
YOR123C	Complex Number 201, probably transcription/DNA maintanance/chromatin structure
YOR123C	Complex Number 46, SPT16 (8)
YOR125C	Complex Number 70
YOR132W	Vps5/Vps17 complex
YOR133W	eEF2
YOR136W	Complex Number 195, probably transcription/DNA maintanance/chromatin structure
YOR136W	Complex Number 271
YOR136W	Complex Number 289
YOR136W	Complex Number 316
YOR136W	Complex Number 320
YOR136W	Complex Number 348
YOR136W	Complex Number 373
YOR136W	Complex Number 62, probably intermediate and energy metabolism
YOR136W	Complex Number 70, probably membrane biogenesis and traffic
YOR136W	Complex Number 78
YOR136W	Isocitrate dehydrogenase
YOR141C	Complex Number 203, probably transcription/DNA maintanance/chromatin structure
YOR142W	Complex Number 272
YOR142W	Complex Number 504
YOR142W	Succinyl-CoA ligase
YOR144C	Complex Number 205, probably transcription/DNA maintanance/chromatin structure
YOR144C	Complex Number 301
YOR145C	Complex Number 109, probably protein synthesis turnover
YOR145C	Complex Number 144, probably RNA metabolism
YOR145C	Complex Number 198, probably transcription/DNA maintanance/chromatin structure
YOR145C	Complex Number 493
YOR148C	mRNA splicing
YOR150W	Complex Number 108, probably protein synthesis turnover
YOR150W	Complex Number 197, probably transcription/DNA maintanance/chromatin structure
YOR150W	mitochondrial ribosomal large subunit
YOR151C	Complex Number 195, probably transcription/DNA maintanance/chromatin structure
YOR151C	Complex Number 207, probably transcription/DNA maintanance/chromatin structure
YOR151C	Complex Number 210, probably transcription/DNA maintanance/chromatin structure

YOR151C	Complex Number 214, probably transcription/DNA maintanance/chromatin structure
YOR151C	Complex Number 24, RNA Polymerase II (12)
YOR151C	Complex Number 41, probably intermediate and energy metabolism
YOR151C	Complex Number 91, probably protein synthesis turnover
YOR151C	RNA polymerase II
YOR153W	Complex Number 67, probably membrane biogenesis and traffic
YOR154W	Complex Number 71
YOR155C	Complex Number 188
YOR157C	20S proteasome
YOR157C	Complex Number 170, probably signalling
YOR157C	Complex Number 60, 20S Proteosome (13)
YOR158W	Complex Number 104, probably protein synthesis turnover
YOR158W	mitochondrial ribosomal small subunit
YOR159C	Complex Number 145, probably RNA metabolism
YOR159C	Complex Number 146, probably RNA metabolism
YOR159C	Complex Number 147, probably RNA metabolism
YOR159C	mRNA splicing
YOR160W	Complex Number 122, probably protein/RNA transport
YOR164C	Complex Number 37, probably intermediate and energy metabolism
YOR167C	cytoplasmic ribosomal small subunit
YOR172W	Complex Number 212
YOR173W	Complex Number 206, probably transcription/DNA maintanance/chromatin structure
YOR173W	Complex Number 424
YOR174W	Complex Number 178
YOR174W	Complex Number 208, probably transcription/DNA maintanance/chromatin structure
YOR174W	Complex Number 209, probably transcription/DNA maintanance/chromatin structure
YOR174W	Complex Number 214, probably transcription/DNA maintanance/chromatin structure
YOR174W	Complex Number 33, Mediator (8)
YOR174W	Kornberg's mediator (SRB) complex
YOR176W	Complex Number 146
YOR176W	Complex Number 157
YOR176W	Complex Number 210
YOR176W	Complex Number 232
YOR176W	Complex Number 254
YOR177C	Complex Number 379
YOR177C	Complex Number 435
YOR178C	Complex Number 534
YOR178C	Serine/threonine phoshpoprotein phosphatase
YOR179C	Complex Number 148, probably RNA metabolism
YOR181W	Actin-associated proteins
YOR181W	Complex Number 155
YOR181W	Complex Number 156
YOR181W	Complex Number 18, probably cell polarity and structure
YOR182C	cytoplasmic ribosomal small subunit

YOR184W	Complex Number 406
YOR185C	Complex Number 120
YOR185C	Complex Number 146
YOR185C	Complex Number 312
YOR185C	Complex Number 445
YOR185C	Complex Number 536
YOR187W	Complex Number 334
YOR187W	Complex Number 6
YOR188W	Complex Number 204, probably transcription/DNA maintanance/chromatin structure
YOR191W	Complex Number 310
YOR194C	Complex Number 182, probably transcription/DNA maintanance/chromatin structure
YOR194C	TFIIA
YOR201C	Complex Number 108, probably protein synthesis turnover
YOR201C	Complex Number 96
YOR202W	Complex Number 149, probably RNA metabolism
YOR204W	Complex Number 16, Ded1 (1)
YOR204W	Complex Number 328
YOR204W	Complex Number 444
YOR204W	Complex Number 489
YOR205C	Complex Number 104, probably protein synthesis turnover
YOR206W	Complex Number 11, Noc (3)
YOR206W	Complex Number 140, probably RNA metabolism
YOR206W	Complex Number 144, probably RNA metabolism
YOR206W	Complex Number 149, probably RNA metabolism
YOR206W	Complex Number 15, Nop1 (1)
YOR206W	Complex Number 17, Pab1 (1)
YOR206W	Complex Number 204
YOR206W	Complex Number 205
YOR206W	Complex Number 446
YOR206W	Complex Number 480
YOR206W	Complex Number 517
YOR206W	Complex Number 520
YOR206W	Complex Number 7, Dbp7/Rrp5 (4)
YOR206W	Complex Number 85, probably protein synthesis turnover
YOR206W	Complex Number 94
YOR207C	Complex Number 137
YOR207C	Complex Number 169
YOR207C	Complex Number 189, probably transcription/DNA maintanance/chromatin structure
YOR207C	Complex Number 213, probably transcription/DNA maintanance/chromatin structure
YOR207C	Complex Number 220, probably transcription/DNA maintanance/chromatin structure
YOR207C	Complex Number 23, RNA Polymerase III (12)
YOR207C	Complex Number 315
YOR207C	Complex Number 316
YOR207C	Complex Number 544

YOR207C	RNA polymerase III
YOR208W	Complex Number 126
YOR210W	Complex Number 213, probably transcription/DNA maintanance/chromatin structure
YOR210W	Complex Number 78
YOR210W	RNA polymerase I
YOR210W	RNA polymerase II
YOR210W	RNA polymerase III
YOR212W	Complex Number 378
YOR212W	Pheromone response pathway
YOR213C	RSC complex (Remodel the structure of chromatin)
YOR215C	Complex Number 132
YOR217W	Complex Number 205, probably transcription/DNA maintanance/chromatin structure
YOR217W	Complex Number 228, probably transcription/DNA maintanance/chromatin structure
YOR217W	Complex Number 301
YOR217W	Complex Number 302
YOR217W	Replication factor C complex
YOR220W	Complex Number 190
YOR220W	Complex Number 360
YOR220W	Complex Number 71
YOR221C	Fatty acid synthetase, mitochondrial
YOR224C	Complex Number 210, probably transcription/DNA maintanance/chromatin structure
YOR224C	Complex Number 213, probably transcription/DNA maintanance/chromatin structure
YOR224C	RNA polymerase I
YOR224C	RNA polymerase II
YOR224C	RNA polymerase III
YOR227W	Complex Number 115
YOR227W	Complex Number 149, probably RNA metabolism
YOR227W	Complex Number 538
YOR229W	Complex Number 420
YOR229W	Complex Number 421
YOR229W	Complex Number 56
YOR230W	Complex Number 304
YOR230W	Complex Number 341
YOR230W	Complex Number 420
YOR230W	Complex Number 421
YOR232W	Complex Number 132
YOR232W	Complex Number 182
YOR232W	Complex Number 188
YOR232W	Complex Number 206
YOR232W	Complex Number 281
YOR232W	Complex Number 326
YOR232W	Complex Number 452
YOR232W	Complex Number 465
YOR232W	Complex Number 88

YOR234C	cytoplasmic ribosomal large subunit
YOR243C	Complex Number 104, probably protein synthesis turnover
YOR243C	Complex Number 11, Noc (3)
YOR243C	Complex Number 142, probably RNA metabolism
YOR243C	Complex Number 38, probably intermediate and energy metabolism
YOR244W	Complex Number 191, probably transcription/DNA maintanance/chromatin structure
YOR244W	Complex Number 334
YOR244W	NuA4 complex
YOR249C	Anaphase promoting complex (APC)
YOR250C	Complex Number 148, probably RNA metabolism
YOR250C	pre mRNA3'-end processing factor CFI
YOR254C	Complex Number 72, probably membrane biogenesis and traffic
YOR254C	Sec62-63 complex
YOR257W	SPB components
YOR259C	19/22S regulator
YOR259C	Complex Number 110, probably protein synthesis turnover
YOR259C	Complex Number 111, probably protein synthesis turnover
YOR259C	Complex Number 13
YOR259C	Complex Number 146
YOR259C	Complex Number 320
YOR259C	Complex Number 346
YOR259C	Complex Number 353
YOR259C	Complex Number 459
YOR259C	Complex Number 468
YOR259C	Complex Number 532
YOR260W	Complex Number 105, probably protein synthesis turnover
YOR260W	Complex Number 107
YOR260W	Complex Number 108
YOR260W	Complex Number 109
YOR260W	Complex Number 111
YOR260W	Complex Number 380
YOR260W	Complex Number 52, tIF2 (7)
YOR260W	eIF2B
YOR261C	19/22S regulator
YOR261C	Complex Number 110, probably protein synthesis turnover
YOR261C	Complex Number 111, probably protein synthesis turnover
YOR261C	Complex Number 13
YOR261C	Complex Number 134
YOR261C	Complex Number 165
YOR261C	Complex Number 202, probably transcription/DNA maintanance/chromatin structure
YOR261C	Complex Number 204, probably transcription/DNA maintanance/chromatin structure
YOR261C	Complex Number 271
YOR261C	Complex Number 289
YOR261C	Complex Number 318

YOR261C	Complex Number 320
YOR261C	Complex Number 374
YOR261C	Complex Number 41, probably intermediate and energy metabolism
YOR261C	Complex Number 453
YOR261C	Complex Number 468
YOR261C	Complex Number 50, 19S Proteosome (17)
YOR265W	Tubulin-associated proteins
YOR266W	Complex Number 173
YOR267C	Complex Number 1, probably cell cycle
YOR267C	Complex Number 368
YOR270C	Complex Number 45, probably intermediate and energy metabolism
YOR270C	H+-transporting ATPase, vacuolar
YOR272W	Complex Number 115
YOR272W	Complex Number 123
YOR272W	Complex Number 140, probably RNA metabolism
YOR272W	Complex Number 149, probably RNA metabolism
YOR272W	Complex Number 161
YOR272W	Complex Number 28, Nop7/Erb1/Ytm1 (3)
YOR272W	Complex Number 389
YOR272W	Complex Number 446
YOR272W	Complex Number 475
YOR272W	Complex Number 520
YOR272W	Complex Number 552
YOR272W	Complex Number 91
YOR272W	Complex Number 94
YOR272W	Tubulin-associated proteins
YOR276W	Complex Number 25
YOR276W	eIF4F
YOR283W	Complex Number 421
YOR290C	Complex Number 101, probably protein synthesis turnover
YOR290C	Complex Number 108, probably protein synthesis turnover
YOR290C	Complex Number 109, probably protein synthesis turnover
YOR290C	Complex Number 155, probably RNA metabolism
YOR290C	Complex Number 198, probably transcription/DNA maintanance/chromatin structure
YOR290C	SWI/SNF transcription activator complex
YOR293W	cytoplasmic ribosomal small subunit
YOR299W	Complex Number 363
YOR303W	Arginine-specific carbamoylphosphate synthase
YOR303W	Complex Number 383
YOR304W	Complex Number 145
YOR304W	Complex Number 189, probably transcription/DNA maintanance/chromatin structure
YOR304W	Complex Number 200, probably transcription/DNA maintanance/chromatin structure
YOR304W	Complex Number 203, probably transcription/DNA maintanance/chromatin structure
YOR304W	Complex Number 220, probably transcription/DNA maintanance/chromatin structure

YOR304W	Complex Number 222, probably transcription/DNA maintanance/chromatin structure
YOR308C	Complex Number 138, probably RNA metabolism
YOR308C	Complex Number 140, probably RNA metabolism
YOR308C	Complex Number 145, probably RNA metabolism
YOR308C	Complex Number 146, probably RNA metabolism
YOR308C	Complex Number 147, probably RNA metabolism
YOR308C	Complex Number 198
YOR308C	Complex Number 236
YOR308C	Complex Number 385
YOR310C	Complex Number 125, probably protein/RNA transport
YOR310C	Complex Number 144, probably RNA metabolism
YOR310C	Complex Number 15, Nop1 (1)
YOR310C	Complex Number 16, Ded1 (1)
YOR310C	Complex Number 17, Pab1 (1)
YOR310C	Complex Number 18, Ydr117c/Rps4b (2)
YOR310C	Complex Number 20, Npl3 (1)
YOR310C	Complex Number 21, Rrp9 (1)
YOR310C	Complex Number 25, RNA polymerase I (7)
YOR310C	Complex Number 321
YOR310C	Complex Number 446
YOR310C	Complex Number 480
YOR310C	Complex Number 4, Dbp3/Bmh1/Nsr1 (3)
YOR310C	Complex Number 53, Kap95/Srp1 (2)
YOR310C	Complex Number 5, Gar1/Cbf5 (6)
YOR310C	Complex Number 6, Nop58/Sik1 (3)
YOR310C	Nop56p/Nop1p complex
YOR312C	Complex Number 38, Arx1 Complex (5)
YOR312C	cytoplasmic ribosomal large subunit
YOR317W	Complex Number 111
YOR317W	Complex Number 146
YOR317W	Complex Number 353
YOR317W	Complex Number 439
YOR317W	Complex Number 77
YOR319W	Complex Number 103
YOR319W	Complex Number 135
YOR319W	Complex Number 145, probably RNA metabolism
YOR319W	Complex Number 96
YOR323C	Complex Number 212, probably transcription/DNA maintanance/chromatin structure
YOR323C	Complex Number 439
YOR323C	Complex Number 64, probably intermediate and energy metabolism
YOR326W	Actin-associated motorproteins
YOR326W	Complex Number 134
YOR326W	Complex Number 134, probably RNA metabolism
YOR326W	Complex Number 17, probably cell polarity and structure

YOR326W	Complex Number 221
YOR326W	Complex Number 308
YOR326W	Complex Number 62
YOR326W	Complex Number 96, probably protein synthesis turnover
YOR327C	v-SNAREs
YOR329C	Complex Number 115
YOR330C	DNA polymerase gamma
YOR332W	Complex Number 132
YOR332W	Complex Number 154
YOR332W	Complex Number 206
YOR332W	Complex Number 230, probably transcription/DNA maintanance/chromatin structure
YOR332W	Complex Number 316
YOR332W	Complex Number 341
YOR332W	Complex Number 45, probably intermediate and energy metabolism
YOR332W	Complex Number 90
YOR332W	H+-transporting ATPase, vacuolar
YOR334W	Mitochondrial splicing complexes
YOR335C	Complex Number 103
YOR335C	Complex Number 244
YOR335C	Complex Number 379
YOR335C	Complex Number 63, Ala1 (1)
YOR335C	Complex Number 78
YOR340C	Complex Number 213, probably transcription/DNA maintanance/chromatin structure
YOR340C	Complex Number 25, RNA polymerase I (7)
YOR340C	Complex Number 314
YOR340C	RNA polymerase I
YOR341W	Complex Number 134
YOR341W	Complex Number 149, probably RNA metabolism
YOR341W	Complex Number 213, probably transcription/DNA maintanance/chromatin structure
YOR341W	Complex Number 24, RNA Polymerase II (12)
YOR341W	Complex Number 25, RNA polymerase I (7)
YOR341W	Complex Number 314
YOR341W	Complex Number 315
YOR341W	Complex Number 316
YOR341W	Complex Number 9, probably cell cycle
YOR341W	RNA polymerase I
YOR344C	Complex Number 148, probably RNA metabolism
YOR347C	Pyruvate kinase
YOR349W	Complex Number 170, probably signalling
YOR351C	Complex Number 179
YOR353C	Complex Number 539
YOR356W	Complex Number 38, probably intermediate and energy metabolism
YOR356W	other respiration chain complexes
YOR358W	CCAAT-binding factor complex

YOR358W	Complex Number 123
YOR358W	Complex Number 217, probably transcription/DNA maintanance/chromatin structure
YOR361C	Complex Number 107, probably protein synthesis turnover
YOR361C	Complex Number 149, probably RNA metabolism
YOR361C	Complex Number 189, probably transcription/DNA maintanance/chromatin structure
YOR361C	Complex Number 19, probably cell polarity and structure
YOR361C	Complex Number 204, probably transcription/DNA maintanance/chromatin structure
YOR361C	Complex Number 49, probably intermediate and energy metabolism
YOR361C	Complex Number 51, eIF3 (7)
YOR361C	Complex Number 52, tIF2 (7)
YOR361C	Complex Number 53, Kap95/Srp1 (2)
YOR361C	Complex Number 7, Dbp7/Rrp5 (4)
YOR361C	Complex Number 99, probably protein synthesis turnover
YOR361C	eIF3
YOR362C	20S proteasome
YOR362C	Complex Number 102, probably protein synthesis turnover
YOR362C	Complex Number 111, probably protein synthesis turnover
YOR362C	Complex Number 170, probably signalling
YOR362C	Complex Number 238
YOR362C	Complex Number 328
YOR362C	Complex Number 338
YOR362C	Complex Number 60, 20S Proteosome (13)
YOR363C	OAF complex
YOR367W	Complex Number 1
YOR368W	Complex Number 177
YOR369C	cytoplasmic ribosomal small subunit
YOR370C	Complex Number 343
YOR370C	Complex Number 418
YOR370C	Complex Number 546
YOR370C	Complex Number 547
YOR370C	Complex Number 548
YOR370C	Geranylgeranyltransferase II (GGTase II)
YOR373W	Complex Number 102, probably protein synthesis turnover
YOR373W	SPB components
YOR374W	other respiration chain complexes
YOR378W	Complex Number 50
YOR386W	Complex Number 225
YOR388C	Complex Number 41, probably intermediate and energy metabolism
YPL001W	Complex Number 10, probably cell cycle
YPL001W	Complex Number 125
YPL001W	Complex Number 204, probably transcription/DNA maintanance/chromatin structure
YPL001W	Complex Number 91
YPL001W	HAT B complex
YPL003W	Complex Number 408

YPL003W	Complex Number 414
YPL003W	Complex Number 94, probably protein synthesis turnover
YPL004C	Complex Number 132
YPL004C	Complex Number 227
YPL004C	Complex Number 228
YPL004C	Complex Number 244
YPL004C	Complex Number 257
YPL004C	Complex Number 341
YPL004C	Complex Number 449
YPL004C	Complex Number 510
YPL004C	Complex Number 525
YPL004C	Complex Number 541
YPL004C	Complex Number 551
YPL004C	Complex Number 91
YPL007C	Complex Number 178, probably transcription/DNA maintanance/chromatin structure
YPL009C	Complex Number 205
YPL009C	Complex Number 86, probably protein synthesis turnover
YPL010W	Complex Number 79, probably membrane biogenesis and traffic
YPL010W	COPI
YPL011C	Complex Number 207, probably transcription/DNA maintanance/chromatin structure
YPL011C	Complex Number 214, probably transcription/DNA maintanance/chromatin structure
YPL011C	TAFIIs
YPL012W	Complex Number 102, probably protein synthesis turnover
YPL012W	Complex Number 109, probably protein synthesis turnover
YPL012W	Complex Number 140, probably RNA metabolism
YPL012W	Complex Number 144, probably RNA metabolism
YPL012W	Complex Number 149, probably RNA metabolism
YPL012W	Complex Number 150
YPL012W	Complex Number 198, probably transcription/DNA maintanance/chromatin structure
YPL012W	Complex Number 446
YPL012W	Complex Number 517
YPL012W	Complex Number 82
YPL012W	Complex Number 91
YPL012W	Complex Number 96
YPL013C	Complex Number 104, probably protein synthesis turnover
YPL013C	Complex Number 22
YPL013C	Complex Number 507
YPL013C	mitochondrial ribosomal small subunit
YPL014W	Complex Number 6, probably cell cycle
YPL016W	Complex Number 155, probably RNA metabolism
YPL016W	Complex Number 198, probably transcription/DNA maintanance/chromatin structure
YPL016W	Complex Number 7
YPL016W	SWI/SNF transcription activator complex
YPL018W	Complex Number 221, probably transcription/DNA maintanance/chromatin structure

YPL018W	Complex Number 437
YPL018W	Ctf19 protein complex
YPL020C	Complex Number 102, probably protein synthesis turnover
YPL022W	Complex Number 136, probably RNA metabolism
YPL022W	Complex Number 257
YPL022W	Complex Number 258
YPL022W	Complex Number 259
YPL022W	Complex Number 439
YPL022W	NEF1 complex
YPL026C	Complex Number 358
YPL028W	Complex Number 244
YPL028W	Complex Number 304
YPL028W	Complex Number 358
YPL028W	Complex Number 379
YPL028W	Complex Number 477
YPL029W	Complex Number 429
YPL029W	Complex Number 81
YPL029W	mitochondrial 3'-to-5' exoribonuclease (mtEXO)
YPL029W	Mitochondrial translation complexes
YPL031C	Complex Number 116
YPL031C	Complex Number 217
YPL031C	Complex Number 224
YPL031C	Complex Number 309
YPL031C	Complex Number 335
YPL031C	Complex Number 505
YPL031C	Pho85p complexes
YPL032C	Complex Number 17
YPL036W	Complex Number 118
YPL036W	Complex Number 134
YPL036W	Complex Number 336
YPL036W	H+-ATPase, plasma mebrane
YPL037C	Complex Number 341
YPL037C	Complex Number 497
YPL037C	Complex Number 56
YPL037C	NAC complex
YPL038W	Complex Number 181
YPL038W	Met4/Met28/Met31 complex
YPL040C	Complex Number 20
YPL042C	Complex Number 209, probably transcription/DNA maintanance/chromatin structure
YPL042C	Srb10p complex
YPL043W	Complex Number 137
YPL043W	Complex Number 140, probably RNA metabolism
YPL043W	Complex Number 149, probably RNA metabolism
YPL043W	Complex Number 204

YPL043W	Complex Number 211
YPL043W	Complex Number 517
YPL043W	Complex Number 56
YPL043W	Complex Number 6
YPL043W	Complex Number 9
YPL045W	Class C Vps protein complex
YPL045W	Complex Number 77, probably membrane biogenesis and traffic
YPL046C	Complex Number 286
YPL048W	Complex Number 103, probably protein synthesis turnover
YPL048W	eEF1
YPL049C	Complex Number 152
YPL049C	Complex Number 153
YPL049C	Complex Number 227, probably transcription/DNA maintanance/chromatin structure
YPL050C	Complex Number 417
YPL051W	Complex Number 12
YPL055C	Complex Number 19
YPL066W	Complex Number 55, probably intermediate and energy metabolism
YPL074W	Complex Number 551
YPL075W	GCR complex
YPL078C	Complex Number 132
YPL078C	Complex Number 412
YPL078C	Complex Number 501
YPL078C	F0/F1 ATP synthase (complex V)
YPL079W	cytoplasmic ribosomal large subunit
YPL081W	cytoplasmic ribosomal small subunit
YPL082C	Complex Number 167
YPL082C	Complex Number 192, probably transcription/DNA maintanance/chromatin structure
YPL082C	Complex Number 196, probably transcription/DNA maintanance/chromatin structure
YPL082C	Complex Number 200, probably transcription/DNA maintanance/chromatin structure
YPL082C	Complex Number 203, probably transcription/DNA maintanance/chromatin structure
YPL082C	Complex Number 214, probably transcription/DNA maintanance/chromatin structure
YPL082C	Complex Number 220, probably transcription/DNA maintanance/chromatin structure
YPL082C	Mot1 complex
YPL083C	Complex Number 197, probably transcription/DNA maintanance/chromatin structure
YPL083C	tRNA splicing
YPL085W	Complex Number 124, probably protein/RNA transport
YPL085W	COPII
YPL086C	Complex Number 216, probably transcription/DNA maintanance/chromatin structure
YPL086C	Complex Number 93
YPL086C	RNA polymerase II holoenzyme
YPL090C	cytoplasmic ribosomal small subunit
YPL093W	Complex Number 107, probably protein synthesis turnover
YPL093W	Complex Number 140, probably RNA metabolism
YPL093W	Complex Number 149, probably RNA metabolism

YPL093W	Complex Number 22
YPL093W	Complex Number 507
YPL093W	Complex Number 520
YPL093W	Complex Number 56
YPL093W	Complex Number 6
YPL093W	Complex Number 86, probably protein synthesis turnover
YPL093W	Complex Number 89
YPL093W	Complex Number 91
YPL093W	Complex Number 94
YPL094C	Complex Number 72, probably membrane biogenesis and traffic
YPL094C	Sec62-63 complex
YPL101W	Complex Number 216, probably transcription/DNA maintanance/chromatin structure
YPL104W	Complex Number 225
YPL106C	Complex Number 68, probably membrane biogenesis and traffic
YPL110C	Complex Number 161
YPL110C	Complex Number 387
YPL110C	Complex Number 439
YPL110C	Complex Number 464
YPL110C	Complex Number 478
YPL110C	Complex Number 528
YPL110C	Complex Number 56
YPL111W	Arginase
YPL111W	Complex Number 27
YPL111W	Complex Number 312
YPL111W	Complex Number 32, probably intermediate and energy metabolism
YPL113C	Complex Number 332
YPL115C	Complex Number 15
YPL115C	Complex Number 152
YPL115C	Complex Number 153
YPL118W	Complex Number 104, probably protein synthesis turnover
YPL118W	mitochondrial ribosomal small subunit
YPL120W	Complex Number 173, probably signalling
YPL122C	NEF3 complex
YPL122C	RNA polymerase I
YPL124W	SPB components
YPL126W	Complex Number 125, probably protein/RNA transport
YPL126W	Complex Number 144, probably RNA metabolism
YPL126W	Complex Number 150
YPL126W	Complex Number 202
YPL126W	Complex Number 229, probably transcription/DNA maintanance/chromatin structure
YPL126W	Complex Number 240
YPL126W	Complex Number 43, UTP A (8)
YPL126W	Complex Number 485
YPL126W	Complex Number 493

YPL127C	Complex Number 169
YPL127C	Complex Number 201
YPL128C	Complex Number 214, probably transcription/DNA maintanance/chromatin structure
YPL128C	Complex Number 279
YPL129W	Complex Number 200
YPL129W	Complex Number 201
YPL129W	Complex Number 210, probably transcription/DNA maintanance/chromatin structure
YPL129W	Complex Number 214, probably transcription/DNA maintanance/chromatin structure
YPL129W	Complex Number 24, RNA Polymerase II (12)
YPL129W	Complex Number 334
YPL129W	Kornberg's mediator (SRB) complex
YPL129W	SWI/SNF transcription activator complex
YPL129W	TAFIIs
YPL129W	TFIIF
YPL131W	Complex Number 317
YPL131W	cytoplasmic ribosomal large subunit
YPL135W	Complex Number 144
YPL138C	Complex Number 199, probably transcription/DNA maintanance/chromatin structure
YPL139C	Complex Number 208, probably transcription/DNA maintanance/chromatin structure
YPL139C	Complex Number 376
YPL139C	Complex Number 415
YPL139C	Complex Number 437
YPL140C	Complex Number 187
YPL140C	Complex Number 229
YPL140C	Complex Number 360
YPL140C	Complex Number 39
YPL140C	Complex Number 505
YPL143W	cytoplasmic ribosomal large subunit
YPL146C	Complex Number 404
YPL149W	Complex Number 5
YPL150W	Complex Number 541
YPL151C	Complex Number 138, probably RNA metabolism
YPL151C	Complex Number 143, probably RNA metabolism
YPL151C	Complex Number 145, probably RNA metabolism
YPL151C	Complex Number 146, probably RNA metabolism
YPL151C	Complex Number 147, probably RNA metabolism
YPL151C	Complex Number 243
YPL151C	Complex Number 42, probably intermediate and energy metabolism
YPL152W	Complex Number 170, probably signalling
YPL153C	Complex Number 200
YPL153C	Complex Number 226, probably transcription/DNA maintanance/chromatin structure
YPL153C	Complex Number 278
YPL153C	Complex Number 279
YPL153C	Complex Number 280

YPL155C	Kinesin-related motorproteins
YPL160W	Complex Number 316
YPL160W	Complex Number 32, probably intermediate and energy metabolism
YPL160W	Complex Number 379
YPL160W	Complex Number 439
YPL161C	Complex Number 45
YPL164C	Complex Number 189
YPL164C	Complex Number 392
YPL166W	Complex Number 123
YPL167C	DNA polymerase zeta
YPL170W	Complex Number 542
YPL171C	Complex Number 435
YPL173W	mitochondrial ribosomal large subunit
YPL174C	Dynactin complex
YPL178W	Complex Number 139, probably RNA metabolism
YPL178W	Complex Number 146, probably RNA metabolism
YPL178W	Complex Number 27, Cbc2/Sto1 (2)
YPL178W	Complex Number 53, Kap95/Srp1 (2)
YPL178W	mRNA splicing
YPL179W	Complex Number 331
YPL181W	Complex Number 208, probably transcription/DNA maintanance/chromatin structure
YPL181W	Complex Number 415
YPL183C	Complex Number 85, Trm7 (2)
YPL183W-A	Complex Number 108, probably protein synthesis turnover
YPL183W-A	mitochondrial ribosomal large subunit
YPL190C	Complex Number 118, probably protein/RNA transport
YPL190C	Complex Number 129, probably protein/RNA transport
YPL190C	Complex Number 139, probably RNA metabolism
YPL190C	Complex Number 155, probably RNA metabolism
YPL190C	Complex Number 197, probably transcription/DNA maintanance/chromatin structure
YPL194W	Complex Number 80
YPL194W	Complex Number 81
YPL194W	Ddc1p-Mec3p complex
YPL195W	AP-3 complex
YPL195W	Complex Number 7
YPL195W	Complex Number 78, probably membrane biogenesis and traffic
YPL198W	cytoplasmic ribosomal large subunit
YPL203W	cAMP-dependent protein kinase
YPL203W	Complex Number 160, probably signalling
YPL203W	Complex Number 400
YPL203W	Complex Number 401
YPL203W	Complex Number 435
YPL204W	Casein kinase I
YPL204W	Complex Number 108, probably protein synthesis turnover

YPL204W	Complex Number 109, probably protein synthesis turnover
YPL204W	Complex Number 132
YPL204W	Complex Number 133
YPL204W	Complex Number 176, probably signalling
YPL204W	Complex Number 198, probably transcription/DNA maintanance/chromatin structure
YPL204W	Complex Number 334
YPL207W	Complex Number 229, probably transcription/DNA maintanance/chromatin structure
YPL208W	Complex Number 463
YPL208W	Complex Number 64, Bcp1/Rpl23A (3)
YPL208W	Complex Number 85, probably protein synthesis turnover
YPL210C	Complex Number 80, SRP (3)
YPL210C	Signal recognition particle (SRP)
YPL211W	Complex Number 141
YPL211W	Complex Number 149, probably RNA metabolism
YPL211W	Complex Number 205
YPL211W	Complex Number 69, probably membrane biogenesis and traffic
YPL211W	Complex Number 82
YPL212C	Complex Number 156, probably RNA metabolism
YPL212C	Complex Number 74, Pus1 (1)
YPL213W	Complex Number 143, probably RNA metabolism
YPL213W	Complex Number 145, probably RNA metabolism
YPL213W	Complex Number 146, probably RNA metabolism
YPL213W	Complex Number 147, probably RNA metabolism
YPL213W	Complex Number 39, probably intermediate and energy metabolism
YPL215W	Complex Number 210
YPL217C	Complex Number 125, probably protein/RNA transport
YPL217C	Complex Number 144, probably RNA metabolism
YPL217C	Complex Number 149, probably RNA metabolism
YPL217C	Complex Number 76, Rcl1/Bms1 (2)
YPL217C	Complex Number 82
YPL218W	Complex Number 388
YPL218W	Complex Number 49
YPL218W	COPII
YPL219W	Pho85p complexes
YPL220W	cytoplasmic ribosomal large subunit
YPL222W	Complex Number 69
YPL226W	Complex Number 103, probably protein synthesis turnover
YPL226W	Complex Number 221
YPL228W	Complex Number 135, probably RNA metabolism
YPL228W	mRNA guanylyl transferase (capping complex)
YPL231W	Complex Number 211, probably transcription/DNA maintanance/chromatin structure
YPL231W	Fatty acid synthetase, cytoplasmic
YPL232W	t-SNAREs
YPL233W	Complex Number 119, probably protein/RNA transport

YPL234C	H+-transporting ATPase, vacuolar
YPL235W	Complex Number 101, probably protein synthesis turnover
YPL235W	Complex Number 11, probably cell cycle
YPL235W	Complex Number 128, probably protein/RNA transport
YPL235W	Complex Number 131, probably RNA metabolism
YPL235W	Complex Number 147, probably RNA metabolism
YPL235W	Complex Number 184, probably transcription/DNA maintanance/chromatin structure
YPL235W	Complex Number 189, probably transcription/DNA maintanance/chromatin structure
YPL235W	Complex Number 195, probably transcription/DNA maintanance/chromatin structure
YPL235W	Complex Number 200, probably transcription/DNA maintanance/chromatin structure
YPL235W	Complex Number 203, probably transcription/DNA maintanance/chromatin structure
YPL235W	Complex Number 211, probably transcription/DNA maintanance/chromatin structure
YPL235W	Complex Number 212, probably transcription/DNA maintanance/chromatin structure
YPL235W	Complex Number 214, probably transcription/DNA maintanance/chromatin structure
YPL235W	Complex Number 325
YPL235W	Complex Number 327
YPL235W	Complex Number 346
YPL235W	Complex Number 468
YPL235W	Complex Number 6, Nop58/Sik1 (3)
YPL235W	Complex Number 75, probably membrane biogenesis and traffic
YPL235W	Complex Number 84
YPL235W	Complex Number 8, probably cell cycle
YPL236C	Complex Number 543
YPL237W	Complex Number 101
YPL237W	Complex Number 105, probably protein synthesis turnover
YPL237W	Complex Number 107, probably protein synthesis turnover
YPL237W	Complex Number 109
YPL237W	Complex Number 115
YPL237W	Complex Number 142, probably RNA metabolism
YPL237W	Complex Number 197, probably transcription/DNA maintanance/chromatin structure
YPL237W	Complex Number 380
YPL237W	Complex Number 52, tIF2 (7)
YPL237W	eIF2
YPL240C	Complex Number 175, probably signalling
YPL241C	Tubulin-associated proteins
YPL243W	Complex Number 80, SRP (3)
YPL243W	Signal recognition particle (SRP)
YPL247C	Complex Number 424
YPL248C	GAL80 complex
YPL249C-A	cytoplasmic ribosomal large subunit
YPL249C	Complex Number 328
YPL249C	Complex Number 329
YPL254W	ADA complex
YPL254W	Complex Number 112

YPL254W	Complex Number 207, probably transcription/DNA maintanance/chromatin structure
YPL254W	Complex Number 382
YPL254W	SAGA complex
YPL255W	SPB components
YPL256C	Cdc28p complexes
YPL256C	Complex Number 61
YPL256C	Complex Number 6, probably cell cycle
YPL258C	Complex Number 203
YPL258C	Complex Number 435
YPL258C	Complex Number 447
YPL258C	Complex Number 47
YPL258C	Complex Number 520
YPL258C	Complex Number 94
YPL259C	AP-1 complex
YPL259C	Complex Number 42, probably intermediate and energy metabolism
YPL259C	Complex Number 6
YPL259C	Complex Number 84, probably membrane biogenesis and traffic
YPL262W	Complex Number 258
YPL262W	Complex Number 304
YPL262W	Complex Number 522
YPL262W	other respiration chain complexes
YPL263C	Complex Number 17, Pab1 (1)
YPL263C	Complex Number 20, Npl3 (1)
YPL263C	Complex Number 3, Kel3 (1)
YPL265W	Complex Number 511
YPL266W	Complex Number 109, probably protein synthesis turnover
YPL266W	Complex Number 144, probably RNA metabolism
YPL266W	Complex Number 146
YPL266W	Complex Number 198, probably transcription/DNA maintanance/chromatin structure
YPL271W	F0/F1 ATP synthase (complex V)
YPR003C	Complex Number 505
YPR004C	other respiration chain complexes
YPR007C	Sister chromatid cohesion complex
YPR010C	Complex Number 149, probably RNA metabolism
YPR010C	Complex Number 152
YPR010C	Complex Number 188, probably transcription/DNA maintanance/chromatin structure
YPR010C	Complex Number 195, probably transcription/DNA maintanance/chromatin structure
YPR010C	Complex Number 197, probably transcription/DNA maintanance/chromatin structure
YPR010C	Complex Number 204, probably transcription/DNA maintanance/chromatin structure
YPR010C	Complex Number 213, probably transcription/DNA maintanance/chromatin structure
YPR010C	Complex Number 220, probably transcription/DNA maintanance/chromatin structure
YPR010C	Complex Number 233
YPR010C	Complex Number 25, RNA polymerase I (7)
YPR010C	Complex Number 314

YPR010C	Complex Number 315
YPR010C	Complex Number 316
YPR010C	Complex Number 332
YPR010C	Complex Number 41, probably intermediate and energy metabolism
YPR010C	Complex Number 84, probably membrane biogenesis and traffic
YPR010C	Complex Number 91, probably protein synthesis turnover
YPR010C	Complex Number 9, probably cell cycle
YPR010C	RNA polymerase I
YPR015C	Complex Number 544
YPR016C	Complex Number 123
YPR016C	Complex Number 149, probably RNA metabolism
YPR016C	Complex Number 161
YPR016C	Complex Number 165
YPR016C	Complex Number 171
YPR016C	Complex Number 206, probably transcription/DNA maintanance/chromatin structure
YPR016C	Complex Number 252
YPR016C	Complex Number 310
YPR016C	Complex Number 317
YPR016C	Complex Number 319
YPR016C	Complex Number 446
YPR016C	Complex Number 475
YPR016C	Complex Number 520
YPR016C	Complex Number 56
YPR016C	Complex Number 6
YPR016C	Complex Number 82
YPR016C	Complex Number 94
YPR017C	Complex Number 546
YPR017C	Complex Number 89
YPR018W	Chromatin assembly complex (CAC)
YPR018W	Complex Number 198
YPR018W	Complex Number 24
YPR018W	Complex Number 311
YPR019W	Complex Number 13
YPR019W	Complex Number 332
YPR019W	Pre-replication complex (pre-RC)
YPR019W	Replication complex
YPR020W	F0/F1 ATP synthase (complex V)
YPR023C	Complex Number 191, probably transcription/DNA maintanance/chromatin structure
YPR023C	Complex Number 208, probably transcription/DNA maintanance/chromatin structure
YPR023C	Complex Number 376
YPR024W	Yme1 protease complex
YPR025C	Complex Number 206, probably transcription/DNA maintanance/chromatin structure
YPR025C	Complex Number 272
YPR025C	Kin28p complex

YPR025C	NEF3 complex
YPR025C	RNA polymerase I
YPR029C	AP-1 complex
YPR029C	Complex Number 6
YPR029C	Complex Number 84, probably membrane biogenesis and traffic
YPR030W	Complex Number 17
YPR030W	Complex Number 18
YPR030W	Complex Number 419
YPR032W	Complex Number 202, probably transcription/DNA maintanance/chromatin structure
YPR032W	Complex Number 213, probably transcription/DNA maintanance/chromatin structure
YPR033C	Complex Number 3
YPR034W	Complex Number 152
YPR034W	Complex Number 32, RSC (13)
YPR034W	RSC complex (Remodel the structure of chromatin)
YPR034W	SWI/SNF transcription activator complex
YPR035W	Complex Number 29, probably intermediate and energy metabolism
YPR036W	Complex Number 116, probably protein synthesis turnover
YPR036W	Complex Number 45, probably intermediate and energy metabolism
YPR036W	H+-transporting ATPase, vacuolar
YPR040W	Complex Number 333
YPR040W	Complex Number 334
YPR041W	Complex Number 107, probably protein synthesis turnover
YPR041W	Complex Number 51, eIF3 (7)
YPR041W	eIF5
YPR043W	cytoplasmic ribosomal large subunit
YPR046W	Ctf3 protein complex
YPR047W	Phenylalaninine-tRNA-ligase
YPR049C	Complex Number 4
YPR051W	Complex Number 117, probably protein synthesis turnover
YPR054W	Complex Number 360
YPR054W	Complex Number 362
YPR054W	Complex Number 363
YPR055W	Complex Number 81, probably membrane biogenesis and traffic
YPR055W	Exocyst complex
YPR056W	Complex Number 151, probably RNA metabolism
YPR056W	Complex Number 202, probably transcription/DNA maintanance/chromatin structure
YPR056W	Complex Number 206, probably transcription/DNA maintanance/chromatin structure
YPR056W	RNA polymerase I
YPR057W	Complex Number 139, probably RNA metabolism
YPR057W	Complex Number 145, probably RNA metabolism
YPR057W	Complex Number 146, probably RNA metabolism
YPR057W	Complex Number 147, probably RNA metabolism
YPR057W	mRNA splicing
YPR066W	Complex Number 94, probably protein synthesis turnover

YPR067W	Complex Number 316
YPR069C	Complex Number 123
YPR069C	Complex Number 26, probably intermediate and energy metabolism
YPR069C	Complex Number 37
YPR072W	CCR4 complex
YPR072W	Complex Number 211, probably transcription/DNA maintanance/chromatin structure
YPR072W	NOT complex
YPR073C	Complex Number 167
YPR074C	Complex Number 28, probably intermediate and energy metabolism
YPR077C	Complex Number 204, probably transcription/DNA maintanance/chromatin structure
YPR080W	eEF1
YPR082C	Complex Number 138, probably RNA metabolism
YPR082C	Complex Number 145, probably RNA metabolism
YPR082C	Complex Number 146, probably RNA metabolism
YPR082C	Complex Number 147, probably RNA metabolism
YPR085C	Complex Number 123
YPR086W	Complex Number 107, probably protein synthesis turnover
YPR086W	TFIIB
YPR088C	Complex Number 118, probably protein/RNA transport
YPR088C	Complex Number 26
YPR088C	Complex Number 505
YPR088C	Complex Number 552
YPR088C	Signal recognition particle (SRP)
YPR089W	Complex Number 376
YPR093C	Complex Number 545
YPR100W	Complex Number 108, probably protein synthesis turnover
YPR100W	mitochondrial ribosomal large subunit
YPR101W	Complex Number 143, probably RNA metabolism
YPR101W	Complex Number 145, probably RNA metabolism
YPR101W	Complex Number 146, probably RNA metabolism
YPR101W	Complex Number 147, probably RNA metabolism
YPR101W	Complex Number 241
YPR101W	Prp19p-associated complex
YPR102C	cytoplasmic ribosomal large subunit
YPR103W	20S proteasome
YPR103W	Complex Number 102, probably protein synthesis turnover
YPR103W	Complex Number 110, probably protein synthesis turnover
YPR103W	Complex Number 111, probably protein synthesis turnover
YPR103W	Complex Number 170, probably signalling
YPR103W	Complex Number 238
YPR103W	Complex Number 60, 20S Proteosome (13)
YPR104C	Complex Number 100
YPR105C	Golgi transport complex
YPR107C	Complex Number 148, probably RNA metabolism

YPR108W	19/22S regulator
YPR108W	Complex Number 110, probably protein synthesis turnover
YPR108W	Complex Number 111, probably protein synthesis turnover
YPR108W	Complex Number 187
YPR108W	Complex Number 320
YPR108W	Complex Number 338
YPR108W	Complex Number 4
YPR108W	Complex Number 468
YPR108W	Complex Number 50, 19S Proteosome (17)
YPR108W	Complex Number 64
YPR110C	Complex Number 192, probably transcription/DNA maintanance/chromatin structure
YPR110C	Complex Number 200, probably transcription/DNA maintanance/chromatin structure
YPR110C	Complex Number 203, probably transcription/DNA maintanance/chromatin structure
YPR110C	Complex Number 204, probably transcription/DNA maintanance/chromatin structure
YPR110C	Complex Number 207
YPR110C	Complex Number 213, probably transcription/DNA maintanance/chromatin structure
YPR110C	Complex Number 22, Exosome (12)
YPR110C	Complex Number 232
YPR110C	Complex Number 24, RNA Polymerase II (12)
YPR110C	Complex Number 25, RNA polymerase I (7)
YPR110C	Complex Number 315
YPR110C	Complex Number 316
YPR110C	RNA polymerase I
YPR110C	RNA polymerase III
YPR111W	Complex Number 78
YPR115W	Complex Number 152
YPR115W	Complex Number 96, probably protein synthesis turnover
YPR119W	Cdc28p complexes
YPR119W	Complex Number 381
YPR119W	Complex Number 52
YPR119W	Complex Number 58
YPR119W	Complex Number 59
YPR120C	Cdc28p complexes
YPR120C	Complex Number 58
YPR121W	Complex Number 240
YPR121W	Complex Number 272
YPR121W	Complex Number 435
YPR121W	Complex Number 455
YPR121W	Complex Number 79
YPR122W	Complex Number 78
YPR124W	Complex Number 338
YPR124W	Complex Number 54
YPR131C	Complex Number 10, Mdm20/Nat3 (2)
YPR132W	cytoplasmic ribosomal small subunit

YPR133C	Complex Number 181, probably transcription/DNA maintanance/chromatin structure
YPR133W-A	TOM - transport across the outer membrane
YPR134W	Mitochondrial splicing complexes
YPR135W	Complex Number 259
YPR135W	Complex Number 260
YPR135W	Complex Number 82
YPR137W	Complex Number 125, probably protein/RNA transport
YPR137W	Complex Number 144, probably RNA metabolism
YPR137W	Complex Number 21, Rrp9 (1)
YPR137W	Complex Number 321
YPR141C	Kinesin-related motorproteins
YPR141C	SPB associated proteins
YPR143W	Complex Number 475
YPR144C	Complex Number 109, probably protein synthesis turnover
YPR144C	Complex Number 125, probably protein/RNA transport
YPR144C	Complex Number 144, probably RNA metabolism
YPR159W	1,6-beta-D-glucan synthase
YPR159W	Complex Number 147
YPR159W	Complex Number 318
YPR159W	Complex Number 6
YPR159W	Complex Number 66
YPR160W	Complex Number 105
YPR160W	Complex Number 114
YPR160W	Complex Number 115
YPR160W	Complex Number 132
YPR160W	Complex Number 240
YPR160W	Complex Number 244
YPR160W	Complex Number 273
YPR160W	Complex Number 274
YPR160W	Complex Number 364
YPR160W	Complex Number 368
YPR160W	Complex Number 402
YPR160W	Complex Number 485
YPR160W	Complex Number 505
YPR160W	Complex Number 77
YPR160W	Complex Number 78
YPR161C	Complex Number 139, probably RNA metabolism
YPR162C	Complex Number 10, probably cell cycle
YPR162C	Post-replication complex (Origin recognition complex=ORC)
YPR162C	Pre-replication complex (pre-RC)
YPR162C	Replication complex
YPR162C	Replication initiation complex
YPR163C	eIF4B
YPR164W	Complex Number 134

YPR165W	Complex Number 304
YPR166C	mitochondrial ribosomal small subunit
YPR167C	Complex Number 505
YPR168W	Complex Number 209, probably transcription/DNA maintanance/chromatin structure
YPR168W	Kornberg's mediator (SRB) complex
YPR171W	Complex Number 18, probably cell polarity and structure
YPR173C	Vps4p ATPase complex (Vps protein complex)
YPR175W	Complex Number 190, probably transcription/DNA maintanance/chromatin structure
YPR175W	Complex Number 205, probably transcription/DNA maintanance/chromatin structure
YPR175W	Complex Number 220, probably transcription/DNA maintanance/chromatin structure
YPR175W	Complex Number 505
YPR175W	DNA polymerase epsilon (II)
YPR175W	Replication complex
YPR176C	Complex Number 159, probably signalling
YPR176C	Geranylgeranyltransferase II (GGTase II)
YPR178W	Complex Number 138, probably RNA metabolism
YPR178W	Complex Number 140, probably RNA metabolism
YPR178W	Complex Number 145, probably RNA metabolism
YPR178W	Complex Number 146, probably RNA metabolism
YPR178W	Complex Number 147, probably RNA metabolism
YPR178W	Complex Number 14, U6-specific snRNP core (14)
YPR178W	Complex Number 242
YPR178W	Complex Number 244
YPR178W	mRNA splicing
YPR179C	Complex Number 212, probably transcription/DNA maintanance/chromatin structure
YPR179C	Complex Number 218, probably transcription/DNA maintanance/chromatin structure
YPR180W	Complex Number 114, probably protein synthesis turnover
YPR180W	Complex Number 210, probably transcription/DNA maintanance/chromatin structure
YPR181C	Complex Number 124, probably protein/RNA transport
YPR181C	Complex Number 130
YPR181C	Complex Number 132
YPR181C	Complex Number 133
YPR181C	Complex Number 200
YPR181C	Complex Number 23
YPR181C	COPII
YPR182W	Complex Number 145, probably RNA metabolism
YPR182W	Complex Number 146, probably RNA metabolism
YPR182W	Complex Number 147, probably RNA metabolism
YPR183W	Complex Number 37
YPR183W	Complex Number 84
YPR184W	Complex Number 114
YPR184W	Complex Number 424
YPR186C	TFIIIA
YPR187W	Complex Number 210, probably transcription/DNA maintanance/chromatin structure

YPR187W	Complex Number 213, probably transcription/DNA maintanance/chromatin structure
YPR187W	Complex Number 24, RNA Polymerase II (12)
YPR187W	Complex Number 316
YPR187W	Complex Number 322
YPR187W	RNA polymerase I
YPR187W	RNA polymerase II
YPR187W	RNA polymerase III
YPR188C	Complex Number 135
YPR188C	Complex Number 17, probably cell polarity and structure
YPR189W	Complex Number 106, probably protein synthesis turnover
YPR189W	Complex Number 141, probably RNA metabolism
YPR189W	Complex Number 355
YPR190C	Complex Number 137
YPR190C	Complex Number 169
YPR190C	Complex Number 213, probably transcription/DNA maintanance/chromatin structure
YPR190C	Complex Number 220, probably transcription/DNA maintanance/chromatin structure
YPR190C	Complex Number 23, RNA Polymerase III (12)
YPR190C	Complex Number 316
YPR190C	Complex Number 396
YPR190C	RNA polymerase III
YPR191W	Complex Number 165
YPR191W	Complex Number 186
YPR191W	Complex Number 189
YPR191W	Complex Number 254
YPR191W	Complex Number 267
YPR191W	Complex Number 360
YPR191W	Complex Number 363
YPR191W	Complex Number 369
YPR191W	Complex Number 444
YPR191W	Complex Number 46, probably intermediate and energy metabolism
YPR191W	Complex Number 497
YPR191W	Cytochrome bc1 complex (Ubiquinol-cytochrome c reductase complex, complex III)
