# Supplementary material for: Predicting Protein Phenotypes Based on Protein-Protein Interaction Network
Source: PLoS One. 2011 Mar 10;6(3):e17668. doi: 10.1371/journal.pone.0017668 (PMC3053377; doi:10.1371/journal.pone.0017668)
Supplement: Table S4 — The proteins and the pathways they belong to in yeast. The information was retrieved from KEGG (Kyoto Encyclopedia of Genes and Genomes) (Kanehisa M, Goto S, Hattori M, Aoki-Kinoshita KF, Itoh M, et al. (2006) From genomics to chemical genomics: new developments in KEGG. Nucleic Acids Res 34: D354-357.). (PDF) [file pone.0017668.s004.pdf]

Table S4. The proteins and the pathways they belong to in yeast. The information was retrieved from KEGG (Kyoto Encyclopedia of Genes and Genomes) (Kanehisa M, Goto S, Hattori M, Aoki-Kinoshita KF, Itoh M, et al. (2006) From genomics to chemical genomics: new developments in KEGG. Nucleic Acids Res 34: D354-357.).

Protein	Pathway
YAL038W	sce00010
YAL054C	sce00010
YBR019C	sce00010
YBR145W	sce00010
YBR196C	sce00010
YBR221C	sce00010
YCL040W	sce00010
YCR012W	sce00010
YDL021W	sce00010
YDL080C	sce00010
YDL168W	sce00010
YDR050C	sce00010
YER073W	sce00010
YER178W	sce00010
YFL018C	sce00010
YFR053C	sce00010
YGL253W	sce00010
YGL256W	sce00010
YGR087C	sce00010
YGR192C	sce00010
YGR240C	sce00010
YGR254W	sce00010
YHR174W	sce00010
YJL052W	sce00010
YJR009C	sce00010
YKL060C	sce00010
YKL127W	sce00010
YKL152C	sce00010
YKR043C	sce00010
YKR097W	sce00010
YLR044C	sce00010
YLR153C	sce00010
YLR377C	sce00010
YMR083W	sce00010
YMR099C	sce00010
YMR105C	sce00010
YMR169C	sce00010
YMR170C	sce00010

YMR205C	sce00010
YMR278W	sce00010
YMR303C	sce00010
YMR323W	sce00010
YNL071W	sce00010
YOL086C	sce00010
YOR347C	sce00010
YOR374W	sce00010
YOR393W	sce00010
YPL017C	sce00010
YPL061W	sce00010
YBR218C	sce00020
YBR221C	sce00020
YCR005C	sce00020
YDL066W	sce00020
YDL078C	sce00020
YDR148C	sce00020
YDR178W	sce00020
YER178W	sce00020
YFL018C	sce00020
YGL062W	sce00020
YGR244C	sce00020
YIL125W	sce00020
YJL045W	sce00020
YJL200C	sce00020
YKL085W	sce00020
YKL141W	sce00020
YKL148C	sce00020
YKR097W	sce00020
YLL041C	sce00020
YLR164W	sce00020
YLR174W	sce00020
YLR304C	sce00020
YMR118C	sce00020
YNL009W	sce00020
YNL037C	sce00020
YNL071W	sce00020
YNR001C	sce00020
YOL126C	sce00020
YOR136W	sce00020
YOR142W	sce00020
YPL017C	sce00020
YPL262W	sce00020
YPR001W	sce00020

YBL068W	sce00030
YBR117C	sce00030
YBR196C	sce00030
YCR036W	sce00030
YCR073W-A	sce00030
YDR248C	sce00030
YER099C	sce00030
YGR043C	sce00030
YGR240C	sce00030
YGR248W	sce00030
YGR256W	sce00030
YHL011C	sce00030
YHR163W	sce00030
YHR183W	sce00030
YJL121C	sce00030
YKL060C	sce00030
YKL127W	sce00030
YKL181W	sce00030
YLR354C	sce00030
YLR377C	sce00030
YMR105C	sce00030
YMR205C	sce00030
YMR278W	sce00030
YNL241C	sce00030
YNR034W	sce00030
YOL061W	sce00030
YOR095C	sce00030
YPR074C	sce00030
YGR194C	sce00040
YHL012W	sce00040
YHR104W	sce00040
YJL121C	sce00040
YJR153W	sce00040
YKL035W	sce00040
YLR070C	sce00040
YCL040W	sce00051
YCR105W	sce00051
YCR107W	sce00051
YDL055C	sce00051
YDL243C	sce00051
YDL246C	sce00051
YDR050C	sce00051
YDR368W	sce00051
YEL070W	sce00051

YER003C	sce00051
YFL045C	sce00051
YFL056C	sce00051
YFR053C	sce00051
YGL253W	sce00051
YGR240C	sce00051
YHR104W	sce00051
YIL107C	sce00051
YJL155C	sce00051
YJR155W	sce00051
YJR159W	sce00051
YKL060C	sce00051
YKR009C	sce00051
YLR377C	sce00051
YMR205C	sce00051
YNL331C	sce00051
YOL136C	sce00051
YOR120W	sce00051
YBR018C	sce00052
YBR019C	sce00052
YBR020W	sce00052
YBR299W	sce00052
YCL040W	sce00052
YDR009W	sce00052
YFR053C	sce00052
YGL253W	sce00052
YGR240C	sce00052
YGR287C	sce00052
YGR292W	sce00052
YHL012W	sce00052
YHR104W	sce00052
YIL162W	sce00052
YIL172C	sce00052
YJL216C	sce00052
YJL221C	sce00052
YKL035W	sce00052
YKL127W	sce00052
YMR105C	sce00052
YMR205C	sce00052
YMR278W	sce00052
YOL157C	sce00052
YER061C	sce00061
YKL182W	sce00061
YNR016C	sce00061

YPL231W	sce00061
YBR026C	sce00062
YBR145W	sce00071
YDL168W	sce00071
YDR402C	sce00071
YER015W	sce00071
YER073W	sce00071
YGL205W	sce00071
YGL256W	sce00071
YIL009W	sce00071
YIL160C	sce00071
YMR083W	sce00071
YMR246W	sce00071
YMR303C	sce00071
YOL086C	sce00071
YOR317W	sce00071
YOR374W	sce00071
YPL028W	sce00071
YPL061W	sce00071
YML126C	sce00072
YPL028W	sce00072
YCR048W	sce00100
YGL001C	sce00100
YGL012W	sce00100
YGR060W	sce00100
YGR175C	sce00100
YHR007C	sce00100
YHR072W	sce00100
YHR190W	sce00100
YLR056W	sce00100
YLR100W	sce00100
YML008C	sce00100
YMR015C	sce00100
YMR202W	sce00100
YNL280C	sce00100
YNR019W	sce00100
YGR255C	sce00130
YML110C	sce00130
YNR041C	sce00130
YOL096C	sce00130
YOR125C	sce00130
Q0045	sce00190
Q0065	sce00190
Q0070	sce00190

Q0080	sce00190
Q0085	sce00190
Q0105	sce00190
Q0110	sce00190
Q0115	sce00190
Q0120	sce00190
Q0130	sce00190
Q0250	sce00190
Q0275	sce00190
YBL045C	sce00190
YBL099W	sce00190
YBR011C	sce00190
YBR039W	sce00190
YBR127C	sce00190
YDL004W	sce00190
YDL067C	sce00190
YDL085W	sce00190
YDL185W	sce00190
YDR178W	sce00190
YDR298C	sce00190
YDR377W	sce00190
YDR529C	sce00190
YEL024W	sce00190
YEL027W	sce00190
YEL051W	sce00190
YER141W	sce00190
YFR033C	sce00190
YGL008C	sce00190
YGL187C	sce00190
YGL191W	sce00190
YGR020C	sce00190
YGR183C	sce00190
YHR001W-A	sce00190
YHR026W	sce00190
YHR039C-A	sce00190
YHR051W	sce00190
YIL111W	sce00190
YJL045W	sce00190
YJL166W	sce00190
YJR121W	sce00190
YKL016C	sce00190
YKL080W	sce00190
YKL141W	sce00190
YKL148C	sce00190

YKL192C	sce00190
YLL009C	sce00190
YLL041C	sce00190
YLR038C	sce00190
YLR164W	sce00190
YLR295C	sce00190
YLR395C	sce00190
YLR447C	sce00190
YML081C-A	sce00190
YML120C	sce00190
YMR054W	sce00190
YMR118C	sce00190
YMR145C	sce00190
YMR256C	sce00190
YMR267W	sce00190
YNL052W	sce00190
YOL077W-A	sce00190
YOR065W	sce00190
YOR270C	sce00190
YOR332W	sce00190
YPL036W	sce00190
YPL078C	sce00190
YPL132W	sce00190
YPL172C	sce00190
YPL234C	sce00190
YPL271W	sce00190
YPR020W	sce00190
YPR036W	sce00190
YPR191W	sce00190
YAL038W	sce00230
YAR015W	sce00230
YBL035C	sce00230
YBL068W	sce00230
YBR111C	sce00230
YBR154C	sce00230
YBR278W	sce00230
YCL050C	sce00230
YDL102W	sce00230
YDL140C	sce00230
YDL150W	sce00230
YDL238C	sce00230
YDR045C	sce00230
YDR121W	sce00230
YDR156W	sce00230

YDR226W	sce00230
YDR404C	sce00230
YDR408C	sce00230
YDR441C	sce00230
YDR454C	sce00230
YDR530C	sce00230
YER005W	sce00230
YER070W	sce00230
YER099C	sce00230
YER170W	sce00230
YGL070C	sce00230
YGL234W	sce00230
YGL248W	sce00230
YGR061C	sce00230
YGR180C	sce00230
YHL011C	sce00230
YHR143W-A	sce00230
YHR201C	sce00230
YHR216W	sce00230
YIL021W	sce00230
YIL066C	sce00230
YIR008C	sce00230
YIR027C	sce00230
YIR029W	sce00230
YIR032C	sce00230
YJL005W	sce00230
YJL026W	sce00230
YJL140W	sce00230
YJL148W	sce00230
YJR006W	sce00230
YJR010W	sce00230
YJR043C	sce00230
YJR063W	sce00230
YJR105W	sce00230
YKL001C	sce00230
YKL045W	sce00230
YKL067W	sce00230
YKL127W	sce00230
YKL144C	sce00230
YKL181W	sce00230
YLR028C	sce00230
YLR209C	sce00230
YLR359W	sce00230
YLR432W	sce00230

YML022W	sce00230
YML035C	sce00230
YML056C	sce00230
YMR105C	sce00230
YMR120C	sce00230
YMR217W	sce00230
YMR278W	sce00230
YMR300C	sce00230
YNL102W	sce00230
YNL113W	sce00230
YNL141W	sce00230
YNL151C	sce00230
YNL220W	sce00230
YNL248C	sce00230
YNL262W	sce00230
YNR003C	sce00230
YOL005C	sce00230
YOL061W	sce00230
YOR116C	sce00230
YOR128C	sce00230
YOR151C	sce00230
YOR207C	sce00230
YOR210W	sce00230
YOR224C	sce00230
YOR340C	sce00230
YOR341W	sce00230
YOR347C	sce00230
YOR360C	sce00230
YPR010C	sce00230
YPR110C	sce00230
YPR175W	sce00230
YPR187W	sce00230
YPR190C	sce00230
YBL035C	sce00240
YBL039C	sce00240
YBR154C	sce00240
YBR252W	sce00240
YBR278W	sce00240
YDL102W	sce00240
YDL140C	sce00240
YDL150W	sce00240
YDR045C	sce00240
YDR121W	sce00240
YDR156W	sce00240

YDR353W	sce00240
YDR404C	sce00240
YEL021W	sce00240
YER005W	sce00240
YER070W	sce00240
YGL063W	sce00240
YGL070C	sce00240
YGR180C	sce00240
YHR106W	sce00240
YHR128W	sce00240
YHR143W-A	sce00240
YHR144C	sce00240
YIL021W	sce00240
YIL066C	sce00240
YIR008C	sce00240
YJL026W	sce00240
YJL130C	sce00240
YJL140W	sce00240
YJL148W	sce00240
YJR006W	sce00240
YJR043C	sce00240
YJR057W	sce00240
YJR063W	sce00240
YJR103W	sce00240
YJR109C	sce00240
YKL024C	sce00240
YKL045W	sce00240
YKL067W	sce00240
YKL144C	sce00240
YKL216W	sce00240
YLR209C	sce00240
YLR245C	sce00240
YLR420W	sce00240
YML106W	sce00240
YMR271C	sce00240
YNL102W	sce00240
YNL113W	sce00240
YNL151C	sce00240
YNL248C	sce00240
YNL262W	sce00240
YNR003C	sce00240
YNR012W	sce00240
YOL005C	sce00240
YOR074C	sce00240

YOR116C	sce00240
YOR151C	sce00240
YOR207C	sce00240
YOR210W	sce00240
YOR224C	sce00240
YOR303W	sce00240
YOR340C	sce00240
YOR341W	sce00240
YPR010C	sce00240
YPR062W	sce00240
YPR110C	sce00240
YPR175W	sce00240
YPR187W	sce00240
YPR190C	sce00240
YAL062W	sce00250
YBR006W	sce00250
YDL171C	sce00250
YDL215C	sce00250
YDR111C	sce00250
YDR321W	sce00250
YGR019W	sce00250
YGR124W	sce00250
YHR018C	sce00250
YHR037W	sce00250
YJL130C	sce00250
YJR109C	sce00250
YKL104C	sce00250
YKL106W	sce00250
YLR027C	sce00250
YLR089C	sce00250
YLR155C	sce00250
YLR157C	sce00250
YLR158C	sce00250
YLR160C	sce00250
YLR359W	sce00250
YMR250W	sce00250
YMR300C	sce00250
YNL220W	sce00250
YOL058W	sce00250
YOR303W	sce00250
YOR375C	sce00250
YPR035W	sce00250
YPR145W	sce00250
YAL012W	sce00260

YBR263W	sce00260
YCL064C	sce00260
YCR053W	sce00260
YDR019C	sce00260
YDR158W	sce00260
YDR232W	sce00260
YEL046C	sce00260
YER026C	sce00260
YER052C	sce00260
YER081W	sce00260
YER086W	sce00260
YFL018C	sce00260
YGL026C	sce00260
YGR155W	sce00260
YGR208W	sce00260
YHR025W	sce00260
YIL074C	sce00260
YJR139C	sce00260
YLR058C	sce00260
YMR189W	sce00260
YOR184W	sce00260
YPL017C	sce00260
YAL012W	sce00270
YCL064C	sce00270
YDR158W	sce00270
YDR502C	sce00270
YEL038W	sce00270
YER043C	sce00270
YER052C	sce00270
YER091C	sce00270
YFR055W	sce00270
YGL184C	sce00270
YGL202W	sce00270
YGR012W	sce00270
YGR155W	sce00270
YHR137W	sce00270
YJR024C	sce00270
YJR130C	sce00270
YJR139C	sce00270
YKL106W	sce00270
YLL058W	sce00270
YLR017W	sce00270
YLR027C	sce00270
YLR146C	sce00270

YLR180W	sce00270
YLR303W	sce00270
YML082W	sce00270
YMR009W	sce00270
YNL277W	sce00270
YOL052C	sce00270
YPR069C	sce00270
YPR118W	sce00270
YER073W	sce00280
YFL018C	sce00280
YGR019W	sce00280
YHR208W	sce00280
YIL160C	sce00280
YJR148W	sce00280
YML126C	sce00280
YOR374W	sce00280
YPL017C	sce00280
YPL028W	sce00280
YPL061W	sce00280
YBL076C	sce00290
YBR221C	sce00290
YCL009C	sce00290
YCL018W	sce00290
YCL064C	sce00290
YER086W	sce00290
YER178W	sce00290
YGL009C	sce00290
YGR094W	sce00290
YHR208W	sce00290
YJR016C	sce00290
YJR148W	sce00290
YLR355C	sce00290
YLR382C	sce00290
YMR108W	sce00290
YNL104C	sce00290
YPL040C	sce00290
YPL160W	sce00290
YBR115C	sce00300
YDL131W	sce00300
YDL182W	sce00300
YDR158W	sce00300
YDR234W	sce00300
YER052C	sce00300
YGL154C	sce00300

YGL202W	sce00300
YIL094C	sce00300
YIR034C	sce00300
YJL060W	sce00300
YJR139C	sce00300
YNR050C	sce00300
YBR115C	sce00310
YDR148C	sce00310
YDR440W	sce00310
YER073W	sce00310
YER078C	sce00310
YGL154C	sce00310
YHR119W	sce00310
YIL125W	sce00310
YIR034C	sce00310
YJL168C	sce00310
YJR010C-A	sce00310
YNR050C	sce00310
YOR374W	sce00310
YPL028W	sce00310
YPL061W	sce00310
YAL062W	sce00330
YBR208C	sce00330
YDL215C	sce00330
YDR242W	sce00330
YDR300C	sce00330
YER023W	sce00330
YER069W	sce00330
YER073W	sce00330
YHR018C	sce00330
YHR037W	sce00330
YJL088W	sce00330
YKL106W	sce00330
YKL184W	sce00330
YLR027C	sce00330
YLR142W	sce00330
YLR146C	sce00330
YLR438W	sce00330
YMR062C	sce00330
YOL052C	sce00330
YOL058W	sce00330
YOL140W	sce00330
YOR323C	sce00330
YOR374W	sce00330

YOR375C	sce00330
YPL061W	sce00330
YPL111W	sce00330
YPR035W	sce00330
YPR062W	sce00330
YPR069C	sce00330
YBR248C	sce00340
YCL030C	sce00340
YCL054W	sce00340
YDR465C	sce00340
YER055C	sce00340
YER073W	sce00340
YFR025C	sce00340
YIL020C	sce00340
YIL116W	sce00340
YMR169C	sce00340
YMR170C	sce00340
YOR201C	sce00340
YOR202W	sce00340
YOR374W	sce00340
YPL061W	sce00340
YBR006W	sce00350
YBR145W	sce00350
YCL054W	sce00350
YDL168W	sce00350
YDR465C	sce00350
YGL202W	sce00350
YGL256W	sce00350
YHR137W	sce00350
YIL116W	sce00350
YJL218W	sce00350
YKL106W	sce00350
YLR027C	sce00350
YMR083W	sce00350
YMR169C	sce00350
YMR170C	sce00350
YMR303C	sce00350
YOL086C	sce00350
YOR201C	sce00350
YDR242W	sce00360
YDR380W	sce00360
YGL202W	sce00360
YHR137W	sce00360
YIL116W	sce00360

YKL106W	sce00360
YLR027C	sce00360
YMR169C	sce00360
YMR170C	sce00360
YBL098W	sce00380
YDR242W	sce00380
YDR256C	sce00380
YDR268W	sce00380
YDR402C	sce00380
YER073W	sce00380
YGR088W	sce00380
YIL125W	sce00380
YJR025C	sce00380
YJR078W	sce00380
YLR134W	sce00380
YLR231C	sce00380
YOL097C	sce00380
YOR374W	sce00380
YPL028W	sce00380
YPL061W	sce00380
YBR166C	sce00400
YBR249C	sce00400
YDR007W	sce00400
YDR035W	sce00400
YDR127W	sce00400
YDR354W	sce00400
YER090W	sce00400
YGL026C	sce00400
YGL148W	sce00400
YGL202W	sce00400
YHR137W	sce00400
YIL116W	sce00400
YKL106W	sce00400
YKL211C	sce00400
YLR027C	sce00400
YNL316C	sce00400
YPR060C	sce00400
YER073W	sce00410
YGR019W	sce00410
YIL145C	sce00410
YLR146C	sce00410
YMR250W	sce00410
YOR374W	sce00410
YPL061W	sce00410

YPR069C	sce00410
YLR299W	sce00430
YMR250W	sce00430
YAL012W	sce00450
YCL054W	sce00450
YDR465C	sce00450
YDR502C	sce00450
YER043C	sce00450
YFR055W	sce00450
YGL184C	sce00450
YGR012W	sce00450
YGR155W	sce00450
YGR171C	sce00450
YGR264C	sce00450
YJR010W	sce00450
YJR130C	sce00450
YKL001C	sce00450
YLL058W	sce00450
YLR180W	sce00450
YLR299W	sce00450
YLR303W	sce00450
YML082W	sce00450
YOR201C	sce00450
YBR263W	sce00460
YDR242W	sce00460
YDR321W	sce00460
YLR058C	sce00460
YLR155C	sce00460
YLR157C	sce00460
YLR158C	sce00460
YLR160C	sce00460
YLR299W	sce00460
YBR244W	sce00480
YDL066W	sce00480
YER070W	sce00480
YGR256W	sce00480
YHR183W	sce00480
YIL066C	sce00480
YIR037W	sce00480
YJL026W	sce00480
YJL101C	sce00480
YKL026C	sce00480
YKL184W	sce00480
YKL215C	sce00480

YLR146C	sce00480
YLR174W	sce00480
YLR299W	sce00480
YNL009W	sce00480
YNL241C	sce00480
YOL049W	sce00480
YPL091W	sce00480
YPR069C	sce00480
YBR001C	sce00500
YBR126C	sce00500
YBR196C	sce00500
YBR299W	sce00500
YCL040W	sce00500
YDL037C	sce00500
YDR001C	sce00500
YDR074W	sce00500
YDR261C	sce00500
YEL011W	sce00500
YFR015C	sce00500
YFR053C	sce00500
YGL253W	sce00500
YGR032W	sce00500
YGR282C	sce00500
YGR287C	sce00500
YGR292W	sce00500
YHL012W	sce00500
YIL099W	sce00500
YIL162W	sce00500
YIL172C	sce00500
YIR019C	sce00500
YJL216C	sce00500
YJL221C	sce00500
YJR153W	sce00500
YKL035W	sce00500
YKL127W	sce00500
YLR258W	sce00500
YLR300W	sce00500
YLR342W	sce00500
YML100W	sce00500
YMR105C	sce00500
YMR261C	sce00500
YMR278W	sce00500
YMR306W	sce00500
YOL157C	sce00500

YOR190W	sce00500
YPR026W	sce00500
YPR160W	sce00500
YPR184W	sce00500
ds:H00118	sce00510
ds:H00119	sce00510
gl:G00001	sce00510
gl:G00002	sce00510
gl:G00003	sce00510
gl:G00004	sce00510
gl:G00005	sce00510
gl:G00006	sce00510
gl:G00007	sce00510
gl:G00008	sce00510
gl:G00009	sce00510
gl:G00010	sce00510
gl:G00011	sce00510
gl:G00012	sce00510
gl:G00013	sce00510
gl:G00014	sce00510
gl:G00015	sce00510
gl:G00016	sce00510
gl:G00017	sce00510
gl:G00018	sce00510
gl:G00019	sce00510
gl:G00020	sce00510
gl:G00021	sce00510
gl:G00022	sce00510
gl:G00171	sce00510
gl:G10526	sce00510
gl:G10595	sce00510
gl:G10596	sce00510
gl:G10597	sce00510
gl:G10598	sce00510
gl:G10599	sce00510
YBL020W	sce00510
YBL082C	sce00510
YBR070C	sce00510
YBR110W	sce00510
YBR229C	sce00510
YBR243C	sce00510
YDL232W	sce00510
YEL002C	sce00510
YGL022W	sce00510

YGL027C	sce00510
YGL047W	sce00510
YGL065C	sce00510
YGL226C-A	sce00510
YGR036C	sce00510
YGR227W	sce00510
YJL002C	sce00510
YJR131W	sce00510
YLR057W	sce00510
YML019W	sce00510
YMR013C	sce00510
YMR149W	sce00510
YNL048W	sce00510
YNL219C	sce00510
YNR030W	sce00510
YOR002W	sce00510
YOR067C	sce00510
YOR085W	sce00510
YOR103C	sce00510
YPL227C	sce00510
YPR183W	sce00510
YGL156W	sce00511
gl:G00011	sce00513
gl:G01813	sce00513
gl:G10694	sce00513
gl:G10841	sce00513
gl:G11040	sce00513
gl:G12625	sce00513
gl:G12626	sce00513
gl:G13047	sce00513
gl:G13048	sce00513
gl:G13049	sce00513
gl:G13050	sce00513
YBR015C	sce00513
YDR245W	sce00513
YEL036C	sce00513
YER001W	sce00513
YGL038C	sce00513
YJL183W	sce00513
YJL186W	sce00513
YJR075W	sce00513
YJR131W	sce00513
YLR057W	sce00513
YML115C	sce00513

YPL050C	sce00513
YPL053C	sce00513
ds:H00120	sce00514
YAL023C	sce00514
YBR205W	sce00514
YDL093W	sce00514
YDL095W	sce00514
YDR307W	sce00514
YDR483W	sce00514
YER001W	sce00514
YGL257C	sce00514
YGR199W	sce00514
YIL014W	sce00514
YJR143C	sce00514
YOR099W	sce00514
YOR321W	sce00514
YBR018C	sce00520
YBR019C	sce00520
YBR020W	sce00520
YBR023C	sce00520
YBR038W	sce00520
YBR196C	sce00520
YCL040W	sce00520
YDL055C	sce00520
YDL103C	sce00520
YDR009W	sce00520
YEL058W	sce00520
YER003C	sce00520
YFL017C	sce00520
YFL045C	sce00520
YFR053C	sce00520
YGL253W	sce00520
YHL012W	sce00520
YIL043C	sce00520
YKL035W	sce00520
YKL104C	sce00520
YKL127W	sce00520
YKL150W	sce00520
YLR286C	sce00520
YLR307W	sce00520
YLR308W	sce00520
YMR105C	sce00520
YMR278W	sce00520
YNL192W	sce00520

YBL011W	sce00561
YDL052C	sce00561
YDR058C	sce00561
YER062C	sce00561
YER073W	sce00561
YFL053W	sce00561
YHL032C	sce00561
YHR104W	sce00561
YIL053W	sce00561
YKR067W	sce00561
YML070W	sce00561
YNR008W	sce00561
YOR175C	sce00561
YOR374W	sce00561
YPL061W	sce00561
YDR050C	sce00562
YDR173C	sce00562
YDR208W	sce00562
YDR287W	sce00562
YFR019W	sce00562
YHR046C	sce00562
YJL153C	sce00562
YLR240W	sce00562
YLR305C	sce00562
YNL106C	sce00562
YNL267W	sce00562
YOL065C	sce00562
YOR109W	sce00562
YPL268W	sce00562
YPR113W	sce00562
gl:G00143	sce00563
gl:G00144	sce00563
gl:G00145	sce00563
gl:G00146	sce00563
gl:G00147	sce00563
gl:G00148	sce00563
gl:G00149	sce00563
gl:G10610	sce00563
gl:G10617	sce00563
gl:G12396	sce00563
gl:G13044	sce00563
gl:G13045	sce00563
gl:G13046	sce00563
YBR004C	sce00563

YCL052C	sce00563
YDR302W	sce00563
YDR331W	sce00563
YDR434W	sce00563
YDR437W	sce00563
YFL025C	sce00563
YGL142C	sce00563
YGR216C	sce00563
YHR188C	sce00563
YJL091C	sce00563
YJR013W	sce00563
YKL165C	sce00563
YLL031C	sce00563
YLR088W	sce00563
YLR459W	sce00563
YMR281W	sce00563
YNL038W	sce00563
YOR149C	sce00563
YPL076W	sce00563
YPL096C-A	sce00563
YPL175W	sce00563
YBL011W	sce00564
YBR029C	sce00564
YCL004W	sce00564
YDL022W	sce00564
YDL052C	sce00564
YDL142C	sce00564
YDR147W	sce00564
YER026C	sce00564
YGR007W	sce00564
YGR110W	sce00564
YGR157W	sce00564
YGR170W	sce00564
YGR202C	sce00564
YHR123W	sce00564
YIL124W	sce00564
YIL155C	sce00564
YKR031C	sce00564
YKR067W	sce00564
YLR133W	sce00564
YMR006C	sce00564
YMR008C	sce00564
YNL130C	sce00564
YNL169C	sce00564

YOL011W	sce00564
YOL059W	sce00564
YOR175C	sce00564
YPL206C	sce00564
YPR113W	sce00564
YPR140W	sce00564
YHR123W	sce00565
YIL124W	sce00565
YKR031C	sce00565
YNL130C	sce00565
YOR175C	sce00565
YBR244W	sce00590
YIR037W	sce00590
YKL026C	sce00590
YLR299W	sce00590
YNL045W	sce00590
YGL205W	sce00592
YIL160C	sce00592
YBR183W	sce00600
YBR265W	sce00600
YDR062W	sce00600
YDR294C	sce00600
YDR297W	sce00600
YHL003C	sce00600
YJL134W	sce00600
YKL008C	sce00600
YKR053C	sce00600
YLR260W	sce00600
YMR296C	sce00600
YOR171C	sce00600
YPL087W	sce00600
YAL038W	sce00620
YAL054C	sce00620
YBL015W	sce00620
YBR218C	sce00620
YBR221C	sce00620
YDL078C	sce00620
YDL131W	sce00620
YDL174C	sce00620
YDL182W	sce00620
YDR272W	sce00620
YER073W	sce00620
YER178W	sce00620
YFL018C	sce00620

YGL062W	sce00620
YHR104W	sce00620
YIR031C	sce00620
YKL029C	sce00620
YKL085W	sce00620
YKR097W	sce00620
YLR153C	sce00620
YML004C	sce00620
YML054C	sce00620
YNL071W	sce00620
YNL104C	sce00620
YNL117W	sce00620
YNR016C	sce00620
YOL126C	sce00620
YOR040W	sce00620
YOR347C	sce00620
YOR374W	sce00620
YPL017C	sce00620
YPL028W	sce00620
YPL061W	sce00620
YBR084W	sce00630
YCR005C	sce00630
YDL078C	sce00630
YER065C	sce00630
YGR204W	sce00630
YIR031C	sce00630
YJL200C	sce00630
YKL085W	sce00630
YLR304C	sce00630
YNL117W	sce00630
YNR001C	sce00630
YOL126C	sce00630
YOR388C	sce00630
YPR001W	sce00630
YPR006C	sce00630
YAL054C	sce00640
YER073W	sce00640
YGR019W	sce00640
YGR244C	sce00640
YLR153C	sce00640
YNR016C	sce00640
YOR142W	sce00640
YOR374W	sce00640
YPL028W	sce00640

YPL061W	sce00640
YPR002W	sce00640
YAL060W	sce00650
YAL061W	sce00650
YBR006W	sce00650
YBR221C	sce00650
YCL009C	sce00650
YCR105W	sce00650
YCR107W	sce00650
YDL243C	sce00650
YDR368W	sce00650
YER073W	sce00650
YER178W	sce00650
YFL056C	sce00650
YGR019W	sce00650
YHR104W	sce00650
YJR155W	sce00650
YKR009C	sce00650
YML126C	sce00650
YMR108W	sce00650
YMR250W	sce00650
YNL331C	sce00650
YOR120W	sce00650
YOR374W	sce00650
YPL028W	sce00650
YPL061W	sce00650
YBL013W	sce00670
YBR084W	sce00670
YBR263W	sce00670
YDR019C	sce00670
YDR408C	sce00670
YER183C	sce00670
YGL125W	sce00670
YGR204W	sce00670
YKR080W	sce00670
YLR028C	sce00670
YLR058C	sce00670
YMR120C	sce00670
YOR074C	sce00670
YOR236W	sce00670
YPL023C	sce00670
YBR263W	sce00680
YDL168W	sce00680
YDR256C	sce00680

YGL125W	sce00680
YGR088W	sce00680
YJL068C	sce00680
YLR058C	sce00680
YOR388C	sce00680
YPL023C	sce00680
YCL017C	sce00730
YOL055C	sce00730
YOR143C	sce00730
YPL214C	sce00730
YPL258C	sce00730
YAR071W	sce00740
YBL033C	sce00740
YBR092C	sce00740
YBR093C	sce00740
YBR153W	sce00740
YBR256C	sce00740
YDL024C	sce00740
YDL045C	sce00740
YDR236C	sce00740
YDR487C	sce00740
YHR215W	sce00740
YOL143C	sce00740
YPR073C	sce00740
YBR035C	sce00750
YCR053W	sce00750
YEL029C	sce00750
YFL059W	sce00750
YFL060C	sce00750
YMR095C	sce00750
YMR096W	sce00750
YNL334C	sce00750
YNR027W	sce00750
YOR184W	sce00750
YPR127W	sce00750
YFR047C	sce00760
YGL037C	sce00760
YGR010W	sce00760
YHR074W	sce00760
YLR209C	sce00760
YLR328W	sce00760
YOR209C	sce00760
YBR176W	sce00770
YCL009C	sce00770

YDR196C	sce00770
YDR531W	sce00770
YHR063C	sce00770
YHR208W	sce00770
YIL145C	sce00770
YJR016C	sce00770
YJR148W	sce00770
YLR355C	sce00770
YMR108W	sce00770
YDL141W	sce00780
YER078C	sce00780
YGR286C	sce00780
YJR010C-A	sce00780
YNR057C	sce00780
YNR058W	sce00780
YJL046W	sce00785
YLR239C	sce00785
YOR196C	sce00785
YDR481C	sce00790
YGR267C	sce00790
YKL132C	sce00790
YMR113W	sce00790
YNL256W	sce00790
YNR033W	sce00790
YOR236W	sce00790
YOR241W	sce00790
YAL039C	sce00860
YBR213W	sce00860
YDL205C	sce00860
YDR044W	sce00860
YDR047W	sce00860
YDR232W	sce00860
YER014W	sce00860
YER141W	sce00860
YGL040C	sce00860
YGL245W	sce00860
YKL087C	sce00860
YKR069W	sce00860
YOL033W	sce00860
YOR176W	sce00860
YOR278W	sce00860
YPL172C	sce00860
YBR002C	sce00900
YBR003W	sce00900

YJL167W	sce00900
YLR450W	sce00900
YML075C	sce00900
YML126C	sce00900
YMR101C	sce00900
YMR208W	sce00900
YMR220W	sce00900
YNR043W	sce00900
YPL028W	sce00900
YPL069C	sce00900
YPL117C	sce00900
YER073W	sce00903
YJL218W	sce00903
YKR009C	sce00903
YOR374W	sce00903
YPL061W	sce00903
YAL012W	sce00910
YAL062W	sce00910
YDL171C	sce00910
YDL215C	sce00910
YDR019C	sce00910
YDR321W	sce00910
YFR055W	sce00910
YGL184C	sce00910
YGR124W	sce00910
YLR155C	sce00910
YLR157C	sce00910
YLR158C	sce00910
YLR160C	sce00910
YOR375C	sce00910
YPR035W	sce00910
YPR145W	sce00910
YCL050C	sce00920
YFR030W	sce00920
YFR055W	sce00920
YGL184C	sce00920
YGR012W	sce00920
YJR010W	sce00920
YJR130C	sce00920
YJR137C	sce00920
YKL001C	sce00920
YLL058W	sce00920
YLR303W	sce00920
YML082W	sce00920

YNL277W	sce00920
YOL064C	sce00920
YPR167C	sce00920
YBL013W	sce00970
YBL076C	sce00970
YBL080C	sce00970
YBR121C	sce00970
YCR024C	sce00970
YDR023W	sce00970
YDR037W	sce00970
YDR268W	sce00970
YDR341C	sce00970
YER087W	sce00970
YFL022C	sce00970
YGL245W	sce00970
YGR094W	sce00970
YGR171C	sce00970
YGR185C	sce00970
YGR264C	sce00970
YHR011W	sce00970
YHR019C	sce00970
YHR020W	sce00970
YHR091C	sce00970
YIL078W	sce00970
YKL194C	sce00970
YLL018C	sce00970
YLR060W	sce00970
YLR382C	sce00970
YMR293C	sce00970
YNL073W	sce00970
YNL247W	sce00970
YOL033W	sce00970
YOL097C	sce00970
YOR168W	sce00970
YOR335C	sce00970
YPL040C	sce00970
YPL097W	sce00970
YPL104W	sce00970
YPL160W	sce00970
YPR033C	sce00970
YPR047W	sce00970
YPR081C	sce00970
YBR159W	sce01040
YCR034W	sce01040

YDL015C	sce01040
YGL055W	sce01040
YGL205W	sce01040
YJL097W	sce01040
YJL196C	sce01040
YJR019C	sce01040
YLR372W	sce01040
gl:G00001	sce01100
gl:G00002	sce01100
gl:G00003	sce01100
gl:G00004	sce01100
gl:G00005	sce01100
gl:G00006	sce01100
gl:G00007	sce01100
gl:G00008	sce01100
gl:G00009	sce01100
gl:G00011	sce01100
gl:G00012	sce01100
gl:G00013	sce01100
gl:G00014	sce01100
gl:G00015	sce01100
gl:G00016	sce01100
gl:G00017	sce01100
gl:G00018	sce01100
gl:G00019	sce01100
gl:G00020	sce01100
gl:G00021	sce01100
gl:G00022	sce01100
gl:G00023	sce01100
gl:G00024	sce01100
gl:G00025	sce01100
gl:G00026	sce01100
gl:G00027	sce01100
gl:G00028	sce01100
gl:G00029	sce01100
gl:G00030	sce01100
gl:G00031	sce01100
gl:G00033	sce01100
gl:G00034	sce01100
gl:G00035	sce01100
gl:G00036	sce01100
gl:G00037	sce01100
gl:G00039	sce01100
gl:G00040	sce01100

gl:G00042	sce01100
gl:G00043	sce01100
gl:G00044	sce01100
gl:G00045	sce01100
gl:G00046	sce01100
gl:G00047	sce01100
gl:G00048	sce01100
gl:G00050	sce01100
gl:G00052	sce01100
gl:G00054	sce01100
gl:G00055	sce01100
gl:G00056	sce01100
gl:G00057	sce01100
gl:G00058	sce01100
gl:G00059	sce01100
gl:G00060	sce01100
gl:G00062	sce01100
gl:G00063	sce01100
gl:G00066	sce01100
gl:G00067	sce01100
gl:G00077	sce01100
gl:G00078	sce01100
gl:G00093	sce01100
gl:G00094	sce01100
gl:G00095	sce01100
gl:G00097	sce01100
gl:G00098	sce01100
gl:G00099	sce01100
gl:G00108	sce01100
gl:G00109	sce01100
gl:G00110	sce01100
gl:G00111	sce01100
gl:G00112	sce01100
gl:G00113	sce01100
gl:G00114	sce01100
gl:G00115	sce01100
gl:G00116	sce01100
gl:G00117	sce01100
gl:G00118	sce01100
gl:G00119	sce01100
gl:G00120	sce01100
gl:G00121	sce01100
gl:G00122	sce01100
gl:G00123	sce01100

gl:G00124	sce01100
gl:G00125	sce01100
gl:G00126	sce01100
gl:G00127	sce01100
gl:G00128	sce01100
gl:G00129	sce01100
gl:G00130	sce01100
gl:G00131	sce01100
gl:G00132	sce01100
gl:G00143	sce01100
gl:G00144	sce01100
gl:G00145	sce01100
gl:G00146	sce01100
gl:G00147	sce01100
gl:G00148	sce01100
gl:G00149	sce01100
gl:G00154	sce01100
gl:G00155	sce01100
gl:G00156	sce01100
gl:G00157	sce01100
gl:G00158	sce01100
gl:G00159	sce01100
gl:G00160	sce01100
gl:G00162	sce01100
gl:G00163	sce01100
gl:G00164	sce01100
gl:G00169	sce01100
gl:G00170	sce01100
gl:G00171	sce01100
gl:G00872	sce01100
gl:G01813	sce01100
gl:G02632	sce01100
gl:G06780	sce01100
gl:G08421	sce01100
gl:G09660	sce01100
gl:G10595	sce01100
gl:G10596	sce01100
gl:G10597	sce01100
gl:G10598	sce01100
gl:G10599	sce01100
gl:G10611	sce01100
gl:G10694	sce01100
gl:G10841	sce01100
gl:G11040	sce01100

gl:G12336	sce01100
gl:G12625	sce01100
gl:G12626	sce01100
gl:G13031	sce01100
gl:G13032	sce01100
gl:G13033	sce01100
gl:G13034	sce01100
gl:G13035	sce01100
gl:G13036	sce01100
gl:G13037	sce01100
gl:G13038	sce01100
gl:G13039	sce01100
gl:G13040	sce01100
gl:G13041	sce01100
gl:G13042	sce01100
gl:G13043	sce01100
gl:G13044	sce01100
gl:G13045	sce01100
gl:G13046	sce01100
gl:G13047	sce01100
gl:G13048	sce01100
gl:G13049	sce01100
gl:G13050	sce01100
Q0045	sce01100
Q0065	sce01100
Q0070	sce01100
Q0080	sce01100
Q0085	sce01100
Q0105	sce01100
Q0110	sce01100
Q0115	sce01100
Q0120	sce01100
Q0130	sce01100
Q0250	sce01100
Q0275	sce01100
YAL012W	sce01100
YAL038W	sce01100
YAL054C	sce01100
YAL062W	sce01100
YAR015W	sce01100
YBL011W	sce01100
YBL033C	sce01100
YBL035C	sce01100
YBL039C	sce01100

YBL045C	sce01100
YBL068W	sce01100
YBL080C	sce01100
YBL082C	sce01100
YBL098W	sce01100
YBL099W	sce01100
YBR004C	sce01100
YBR006W	sce01100
YBR015C	sce01100
YBR018C	sce01100
YBR019C	sce01100
YBR020W	sce01100
YBR026C	sce01100
YBR029C	sce01100
YBR035C	sce01100
YBR039W	sce01100
YBR070C	sce01100
YBR084W	sce01100
YBR110W	sce01100
YBR115C	sce01100
YBR117C	sce01100
YBR127C	sce01100
YBR145W	sce01100
YBR153W	sce01100
YBR154C	sce01100
YBR166C	sce01100
YBR176W	sce01100
YBR196C	sce01100
YBR208C	sce01100
YBR213W	sce01100
YBR218C	sce01100
YBR221C	sce01100
YBR229C	sce01100
YBR243C	sce01100
YBR248C	sce01100
YBR249C	sce01100
YBR252W	sce01100
YBR256C	sce01100
YBR263W	sce01100
YBR265W	sce01100
YBR278W	sce01100
YBR299W	sce01100
YCL004W	sce01100
YCL009C	sce01100

YCL018W	sce01100
YCL030C	sce01100
YCL040W	sce01100
YCL050C	sce01100
YCL052C	sce01100
YCL064C	sce01100
YCR005C	sce01100
YCR012W	sce01100
YCR053W	sce01100
YCR073W-A	sce01100
YCR105W	sce01100
YCR107W	sce01100
YDL004W	sce01100
YDL021W	sce01100
YDL037C	sce01100
YDL045C	sce01100
YDL052C	sce01100
YDL055C	sce01100
YDL066W	sce01100
YDL067C	sce01100
YDL078C	sce01100
YDL080C	sce01100
YDL086W	sce01100
YDL102W	sce01100
YDL103C	sce01100
YDL131W	sce01100
YDL140C	sce01100
YDL141W	sce01100
YDL142C	sce01100
YDL150W	sce01100
YDL168W	sce01100
YDL171C	sce01100
YDL182W	sce01100
YDL185W	sce01100
YDL205C	sce01100
YDL215C	sce01100
YDL232W	sce01100
YDL238C	sce01100
YDL243C	sce01100
YDL246C	sce01100
YDR007W	sce01100
YDR009W	sce01100
YDR019C	sce01100
YDR035W	sce01100

YDR044W	sce01100
YDR045C	sce01100
YDR050C	sce01100
YDR058C	sce01100
YDR062W	sce01100
YDR111C	sce01100
YDR121W	sce01100
YDR127W	sce01100
YDR147W	sce01100
YDR148C	sce01100
YDR156W	sce01100
YDR158W	sce01100
YDR178W	sce01100
YDR196C	sce01100
YDR208W	sce01100
YDR226W	sce01100
YDR232W	sce01100
YDR234W	sce01100
YDR236C	sce01100
YDR245W	sce01100
YDR248C	sce01100
YDR256C	sce01100
YDR287W	sce01100
YDR294C	sce01100
YDR297W	sce01100
YDR298C	sce01100
YDR300C	sce01100
YDR302W	sce01100
YDR321W	sce01100
YDR331W	sce01100
YDR354W	sce01100
YDR368W	sce01100
YDR377W	sce01100
YDR380W	sce01100
YDR404C	sce01100
YDR408C	sce01100
YDR434W	sce01100
YDR437W	sce01100
YDR441C	sce01100
YDR454C	sce01100
YDR481C	sce01100
YDR502C	sce01100
YDR529C	sce01100
YDR531W	sce01100

YEL002C	sce01100
YEL011W	sce01100
YEL021W	sce01100
YEL024W	sce01100
YEL027W	sce01100
YEL029C	sce01100
YEL036C	sce01100
YEL046C	sce01100
YEL051W	sce01100
YER001W	sce01100
YER003C	sce01100
YER014W	sce01100
YER015W	sce01100
YER023W	sce01100
YER026C	sce01100
YER043C	sce01100
YER052C	sce01100
YER055C	sce01100
YER061C	sce01100
YER062C	sce01100
YER065C	sce01100
YER069W	sce01100
YER070W	sce01100
YER073W	sce01100
YER078C	sce01100
YER081W	sce01100
YER086W	sce01100
YER090W	sce01100
YER091C	sce01100
YER099C	sce01100
YER141W	sce01100
YER170W	sce01100
YER178W	sce01100
YER183C	sce01100
YFL018C	sce01100
YFL025C	sce01100
YFL045C	sce01100
YFL053W	sce01100
YFL056C	sce01100
YFR014C	sce01100
YFR025C	sce01100
YFR030W	sce01100
YFR033C	sce01100
YFR047C	sce01100

YFR053C	sce01100
YFR055W	sce01100
YGL001C	sce01100
YGL012W	sce01100
YGL022W	sce01100
YGL026C	sce01100
YGL027C	sce01100
YGL037C	sce01100
YGL038C	sce01100
YGL040C	sce01100
YGL047W	sce01100
YGL062W	sce01100
YGL065C	sce01100
YGL070C	sce01100
YGL125W	sce01100
YGL142C	sce01100
YGL148W	sce01100
YGL154C	sce01100
YGL184C	sce01100
YGL187C	sce01100
YGL191W	sce01100
YGL202W	sce01100
YGL205W	sce01100
YGL226C-A	sce01100
YGL234W	sce01100
YGL245W	sce01100
YGL253W	sce01100
YGL256W	sce01100
YGR007W	sce01100
YGR010W	sce01100
YGR012W	sce01100
YGR019W	sce01100
YGR020C	sce01100
YGR043C	sce01100
YGR060W	sce01100
YGR061C	sce01100
YGR087C	sce01100
YGR088W	sce01100
YGR124W	sce01100
YGR155W	sce01100
YGR157W	sce01100
YGR170W	sce01100
YGR175C	sce01100
YGR180C	sce01100

YGR183C	sce01100
YGR192C	sce01100
YGR194C	sce01100
YGR202C	sce01100
YGR204W	sce01100
YGR208W	sce01100
YGR216C	sce01100
YGR227W	sce01100
YGR240C	sce01100
YGR244C	sce01100
YGR248W	sce01100
YGR254W	sce01100
YGR255C	sce01100
YGR256W	sce01100
YGR267C	sce01100
YGR286C	sce01100
YGR287C	sce01100
YGR292W	sce01100
YHL003C	sce01100
YHL011C	sce01100
YHL012W	sce01100
YHL032C	sce01100
YHR001W-A	sce01100
YHR007C	sce01100
YHR018C	sce01100
YHR025W	sce01100
YHR026W	sce01100
YHR037W	sce01100
YHR039C-A	sce01100
YHR046C	sce01100
YHR051W	sce01100
YHR063C	sce01100
YHR072W	sce01100
YHR074W	sce01100
YHR104W	sce01100
YHR123W	sce01100
YHR128W	sce01100
YHR137W	sce01100
YHR143W-A	sce01100
YHR144C	sce01100
YHR163W	sce01100
YHR174W	sce01100
YHR183W	sce01100
YHR188C	sce01100

YHR190W	sce01100
YHR208W	sce01100
YHR216W	sce01100
YIL009W	sce01100
YIL020C	sce01100
YIL021W	sce01100
YIL053W	sce01100
YIL066C	sce01100
YIL074C	sce01100
YIL094C	sce01100
YIL099W	sce01100
YIL111W	sce01100
YIL116W	sce01100
YIL124W	sce01100
YIL125W	sce01100
YIL139C	sce01100
YIL145C	sce01100
YIL160C	sce01100
YIL162W	sce01100
YIL172C	sce01100
YIR008C	sce01100
YIR019C	sce01100
YIR027C	sce01100
YIR029W	sce01100
YIR031C	sce01100
YIR032C	sce01100
YIR034C	sce01100
YJL002C	sce01100
YJL026W	sce01100
YJL045W	sce01100
YJL046W	sce01100
YJL052W	sce01100
YJL088W	sce01100
YJL091C	sce01100
YJL101C	sce01100
YJL121C	sce01100
YJL130C	sce01100
YJL140W	sce01100
YJL148W	sce01100
YJL153C	sce01100
YJL166W	sce01100
YJL167W	sce01100
YJL183W	sce01100
YJL186W	sce01100

YJL200C	sce01100
YJL216C	sce01100
YJL221C	sce01100
YJR006W	sce01100
YJR009C	sce01100
YJR010C-A	sce01100
YJR010W	sce01100
YJR013W	sce01100
YJR016C	sce01100
YJR024C	sce01100
YJR025C	sce01100
YJR043C	sce01100
YJR057W	sce01100
YJR063W	sce01100
YJR075W	sce01100
YJR078W	sce01100
YJR103W	sce01100
YJR105W	sce01100
YJR109C	sce01100
YJR121W	sce01100
YJR130C	sce01100
YJR131W	sce01100
YJR137C	sce01100
YJR139C	sce01100
YJR148W	sce01100
YJR153W	sce01100
YJR155W	sce01100
YJR159W	sce01100
YKL001C	sce01100
YKL008C	sce01100
YKL016C	sce01100
YKL024C	sce01100
YKL035W	sce01100
YKL045W	sce01100
YKL060C	sce01100
YKL067W	sce01100
YKL080W	sce01100
YKL085W	sce01100
YKL104C	sce01100
YKL106W	sce01100
YKL127W	sce01100
YKL132C	sce01100
YKL141W	sce01100
YKL144C	sce01100

YKL148C	sce01100
YKL152C	sce01100
YKL165C	sce01100
YKL181W	sce01100
YKL182W	sce01100
YKL184W	sce01100
YKL192C	sce01100
YKL211C	sce01100
YKL216W	sce01100
YKR009C	sce01100
YKR031C	sce01100
YKR043C	sce01100
YKR067W	sce01100
YKR069W	sce01100
YKR080W	sce01100
YKR097W	sce01100
YLL009C	sce01100
YLL031C	sce01100
YLL041C	sce01100
YLL058W	sce01100
YLR017W	sce01100
YLR027C	sce01100
YLR028C	sce01100
YLR038C	sce01100
YLR044C	sce01100
YLR056W	sce01100
YLR057W	sce01100
YLR058C	sce01100
YLR070C	sce01100
YLR088W	sce01100
YLR089C	sce01100
YLR100W	sce01100
YLR133W	sce01100
YLR134W	sce01100
YLR142W	sce01100
YLR146C	sce01100
YLR153C	sce01100
YLR155C	sce01100
YLR157C	sce01100
YLR158C	sce01100
YLR160C	sce01100
YLR164W	sce01100
YLR174W	sce01100
YLR180W	sce01100

YLR209C	sce01100
YLR231C	sce01100
YLR239C	sce01100
YLR240W	sce01100
YLR245C	sce01100
YLR260W	sce01100
YLR295C	sce01100
YLR299W	sce01100
YLR303W	sce01100
YLR304C	sce01100
YLR305C	sce01100
YLR328W	sce01100
YLR354C	sce01100
YLR355C	sce01100
YLR359W	sce01100
YLR377C	sce01100
YLR395C	sce01100
YLR420W	sce01100
YLR432W	sce01100
YLR438W	sce01100
YLR447C	sce01100
YLR450W	sce01100
YLR459W	sce01100
YML008C	sce01100
YML019W	sce01100
YML022W	sce01100
YML035C	sce01100
YML054C	sce01100
YML056C	sce01100
YML070W	sce01100
YML075C	sce01100
YML081C-A	sce01100
YML082W	sce01100
YML106W	sce01100
YML110C	sce01100
YML115C	sce01100
YML126C	sce01100
YMR009W	sce01100
YMR013C	sce01100
YMR015C	sce01100
YMR054W	sce01100
YMR062C	sce01100
YMR083W	sce01100
YMR105C	sce01100

YMR108W	sce01100
YMR113W	sce01100
YMR118C	sce01100
YMR120C	sce01100
YMR149W	sce01100
YMR169C	sce01100
YMR170C	sce01100
YMR189W	sce01100
YMR202W	sce01100
YMR205C	sce01100
YMR208W	sce01100
YMR217W	sce01100
YMR220W	sce01100
YMR246W	sce01100
YMR250W	sce01100
YMR256C	sce01100
YMR271C	sce01100
YMR278W	sce01100
YMR281W	sce01100
YMR293C	sce01100
YMR296C	sce01100
YMR300C	sce01100
YMR303C	sce01100
YMR323W	sce01100
YNL009W	sce01100
YNL037C	sce01100
YNL038W	sce01100
YNL045W	sce01100
YNL048W	sce01100
YNL052W	sce01100
YNL071W	sce01100
YNL102W	sce01100
YNL104C	sce01100
YNL106C	sce01100
YNL113W	sce01100
YNL117W	sce01100
YNL130C	sce01100
YNL141W	sce01100
YNL151C	sce01100
YNL169C	sce01100
YNL219C	sce01100
YNL220W	sce01100
YNL241C	sce01100
YNL248C	sce01100

YNL256W	sce01100
YNL262W	sce01100
YNL267W	sce01100
YNL277W	sce01100
YNL280C	sce01100
YNL316C	sce01100
YNL331C	sce01100
YNR001C	sce01100
YNR003C	sce01100
YNR012W	sce01100
YNR016C	sce01100
YNR027W	sce01100
YNR030W	sce01100
YNR034W	sce01100
YNR041C	sce01100
YNR043W	sce01100
YNR050C	sce01100
YNR057C	sce01100
YNR058W	sce01100
YOL005C	sce01100
YOL016C	sce01100
YOL033W	sce01100
YOL049W	sce01100
YOL052C	sce01100
YOL055C	sce01100
YOL058W	sce01100
YOL061W	sce01100
YOL064C	sce01100
YOL065C	sce01100
YOL077W-A	sce01100
YOL086C	sce01100
YOL096C	sce01100
YOL126C	sce01100
YOL140W	sce01100
YOL143C	sce01100
YOL157C	sce01100
YOR002W	sce01100
YOR065W	sce01100
YOR067C	sce01100
YOR074C	sce01100
YOR085W	sce01100
YOR095C	sce01100
YOR103C	sce01100
YOR109W	sce01100

YOR116C	sce01100
YOR120W	sce01100
YOR125C	sce01100
YOR128C	sce01100
YOR136W	sce01100
YOR142W	sce01100
YOR143C	sce01100
YOR151C	sce01100
YOR168W	sce01100
YOR171C	sce01100
YOR175C	sce01100
YOR176W	sce01100
YOR184W	sce01100
YOR196C	sce01100
YOR202W	sce01100
YOR207C	sce01100
YOR209C	sce01100
YOR210W	sce01100
YOR224C	sce01100
YOR236W	sce01100
YOR241W	sce01100
YOR270C	sce01100
YOR274W	sce01100
YOR278W	sce01100
YOR303W	sce01100
YOR317W	sce01100
YOR323C	sce01100
YOR330C	sce01100
YOR332W	sce01100
YOR340C	sce01100
YOR341W	sce01100
YOR347C	sce01100
YOR374W	sce01100
YOR375C	sce01100
YOR388C	sce01100
YOR393W	sce01100
YPL017C	sce01100
YPL023C	sce01100
YPL028W	sce01100
YPL050C	sce01100
YPL053C	sce01100
YPL061W	sce01100
YPL069C	sce01100
YPL076W	sce01100

YPL078C	sce01100
YPL096C-A	sce01100
YPL111W	sce01100
YPL117C	sce01100
YPL132W	sce01100
YPL167C	sce01100
YPL172C	sce01100
YPL175W	sce01100
YPL214C	sce01100
YPL227C	sce01100
YPL231W	sce01100
YPL234C	sce01100
YPL258C	sce01100
YPL262W	sce01100
YPL268W	sce01100
YPL271W	sce01100
YPR001W	sce01100
YPR006C	sce01100
YPR010C	sce01100
YPR020W	sce01100
YPR035W	sce01100
YPR036W	sce01100
YPR060C	sce01100
YPR062W	sce01100
YPR069C	sce01100
YPR074C	sce01100
YPR110C	sce01100
YPR113W	sce01100
YPR118W	sce01100
YPR127W	sce01100
YPR145W	sce01100
YPR167C	sce01100
YPR175W	sce01100
YPR183W	sce01100
YPR187W	sce01100
YPR190C	sce01100
YPR191W	sce01100
YAL038W	sce01110
YAL054C	sce01110
YAR015W	sce01110
YBL068W	sce01110
YBR002C	sce01110
YBR003W	sce01110
YBR019C	sce01110

YBR084W	sce01110
YBR115C	sce01110
YBR117C	sce01110
YBR145W	sce01110
YBR176W	sce01110
YBR196C	sce01110
YBR213W	sce01110
YBR221C	sce01110
YBR248C	sce01110
YBR249C	sce01110
YBR263W	sce01110
YCL009C	sce01110
YCL018W	sce01110
YCL030C	sce01110
YCL040W	sce01110
YCL064C	sce01110
YCR005C	sce01110
YCR012W	sce01110
YCR073W-A	sce01110
YDL021W	sce01110
YDL055C	sce01110
YDL066W	sce01110
YDL078C	sce01110
YDL080C	sce01110
YDL168W	sce01110
YDL171C	sce01110
YDL205C	sce01110
YDR007W	sce01110
YDR035W	sce01110
YDR047W	sce01110
YDR050C	sce01110
YDR127W	sce01110
YDR148C	sce01110
YDR158W	sce01110
YDR178W	sce01110
YDR226W	sce01110
YDR248C	sce01110
YDR287W	sce01110
YDR310C	sce01110
YDR321W	sce01110
YDR354W	sce01110
YDR408C	sce01110
YDR502C	sce01110
YEL046C	sce01110

YER003C	sce01110
YER014W	sce01110
YER023W	sce01110
YER052C	sce01110
YER055C	sce01110
YER069W	sce01110
YER073W	sce01110
YER086W	sce01110
YER090W	sce01110
YER091C	sce01110
YER099C	sce01110
YER141W	sce01110
YER170W	sce01110
YER178W	sce01110
YFL018C	sce01110
YFL045C	sce01110
YFR025C	sce01110
YFR053C	sce01110
YFR055W	sce01110
YGL009C	sce01110
YGL026C	sce01110
YGL040C	sce01110
YGL148W	sce01110
YGL154C	sce01110
YGL184C	sce01110
YGL234W	sce01110
YGL245W	sce01110
YGL253W	sce01110
YGL256W	sce01110
YGR043C	sce01110
YGR061C	sce01110
YGR087C	sce01110
YGR124W	sce01110
YGR175C	sce01110
YGR192C	sce01110
YGR204W	sce01110
YGR240C	sce01110
YGR244C	sce01110
YGR248W	sce01110
YGR254W	sce01110
YGR255C	sce01110
YGR256W	sce01110
YHL011C	sce01110
YHL012W	sce01110

YHR007C	sce01110
YHR018C	sce01110
YHR046C	sce01110
YHR063C	sce01110
YHR072W	sce01110
YHR163W	sce01110
YHR174W	sce01110
YHR183W	sce01110
YHR188C	sce01110
YHR190W	sce01110
YHR208W	sce01110
YHR216W	sce01110
YIL020C	sce01110
YIL116W	sce01110
YIL125W	sce01110
YIL145C	sce01110
YIL160C	sce01110
YIR034C	sce01110
YJL045W	sce01110
YJL052W	sce01110
YJL088W	sce01110
YJL121C	sce01110
YJL153C	sce01110
YJL167W	sce01110
YJL200C	sce01110
YJR009C	sce01110
YJR016C	sce01110
YJR130C	sce01110
YJR139C	sce01110
YJR148W	sce01110
YKL035W	sce01110
YKL060C	sce01110
YKL067W	sce01110
YKL085W	sce01110
YKL106W	sce01110
YKL127W	sce01110
YKL141W	sce01110
YKL148C	sce01110
YKL152C	sce01110
YKL181W	sce01110
YKL184W	sce01110
YKR009C	sce01110
YKR043C	sce01110
YKR069W	sce01110

YKR097W	sce01110
YLL041C	sce01110
YLL058W	sce01110
YLR027C	sce01110
YLR028C	sce01110
YLR044C	sce01110
YLR058C	sce01110
YLR142W	sce01110
YLR153C	sce01110
YLR155C	sce01110
YLR157C	sce01110
YLR158C	sce01110
YLR160C	sce01110
YLR164W	sce01110
YLR174W	sce01110
YLR180W	sce01110
YLR209C	sce01110
YLR304C	sce01110
YLR354C	sce01110
YLR355C	sce01110
YLR359W	sce01110
YLR377C	sce01110
YLR432W	sce01110
YLR438W	sce01110
YLR450W	sce01110
YML008C	sce01110
YML035C	sce01110
YML056C	sce01110
YML075C	sce01110
YML082W	sce01110
YML110C	sce01110
YML126C	sce01110
YMR062C	sce01110
YMR083W	sce01110
YMR101C	sce01110
YMR105C	sce01110
YMR108W	sce01110
YMR118C	sce01110
YMR120C	sce01110
YMR205C	sce01110
YMR208W	sce01110
YMR220W	sce01110
YMR278W	sce01110
YMR300C	sce01110

YMR303C	sce01110
YMR323W	sce01110
YNL009W	sce01110
YNL037C	sce01110
YNL071W	sce01110
YNL104C	sce01110
YNL241C	sce01110
YNL280C	sce01110
YNL316C	sce01110
YNR001C	sce01110
YNR034W	sce01110
YNR041C	sce01110
YNR043W	sce01110
YNR050C	sce01110
YOL033W	sce01110
YOL058W	sce01110
YOL061W	sce01110
YOL086C	sce01110
YOL096C	sce01110
YOL126C	sce01110
YOL140W	sce01110
YOR095C	sce01110
YOR125C	sce01110
YOR128C	sce01110
YOR136W	sce01110
YOR142W	sce01110
YOR176W	sce01110
YOR202W	sce01110
YOR274W	sce01110
YOR278W	sce01110
YOR347C	sce01110
YOR374W	sce01110
YOR393W	sce01110
YPL017C	sce01110
YPL028W	sce01110
YPL061W	sce01110
YPL069C	sce01110
YPL111W	sce01110
YPL117C	sce01110
YPL172C	sce01110
YPL262W	sce01110
YPR001W	sce01110
YPR060C	sce01110
YPR074C	sce01110

YPR145W	sce01110
YBL027W	sce03010
YBL072C	sce03010
YBL087C	sce03010
YBL092W	sce03010
YBR031W	sce03010
YBR048W	sce03010
YBR084C-A	sce03010
YBR181C	sce03010
YBR189W	sce03010
YBR191W	sce03010
YBR251W	sce03010
YCR031C	sce03010
YDL061C	sce03010
YDL075W	sce03010
YDL081C	sce03010
YDL082W	sce03010
YDL083C	sce03010
YDL130W	sce03010
YDL133C-A	sce03010
YDL136W	sce03010
YDL184C	sce03010
YDL191W	sce03010
YDR012W	sce03010
YDR025W	sce03010
YDR064W	sce03010
YDR382W	sce03010
YDR418W	sce03010
YDR447C	sce03010
YDR450W	sce03010
YDR471W	sce03010
YDR500C	sce03010
YEL054C	sce03010
YER056C-A	sce03010
YER074W	sce03010
YER102W	sce03010
YER117W	sce03010
YER131W	sce03010
YFL034C-A	sce03010
YFR031C-A	sce03010
YFR032C-A	sce03010
YGL030W	sce03010
YGL031C	sce03010
YGL076C	sce03010

YGL103W	sce03010
YGL123W	sce03010
YGL135W	sce03010
YGL147C	sce03010
YGL189C	sce03010
YGR027C	sce03010
YGR034W	sce03010
YGR085C	sce03010
YGR118W	sce03010
YGR148C	sce03010
YGR214W	sce03010
YHL001W	sce03010
YHL004W	sce03010
YHL015W	sce03010
YHL033C	sce03010
YHR010W	sce03010
YHR021C	sce03010
YHR141C	sce03010
YHR203C	sce03010
YIL018W	sce03010
YIL052C	sce03010
YIL069C	sce03010
YIL133C	sce03010
YIL148W	sce03010
YJL136C	sce03010
YJL177W	sce03010
YJL189W	sce03010
YJL190C	sce03010
YJL191W	sce03010
YJR094W-A	sce03010
YJR113C	sce03010
YJR123W	sce03010
YJR145C	sce03010
YKL006W	sce03010
YKL156W	sce03010
YKL180W	sce03010
YKR057W	sce03010
YKR094C	sce03010
YLL045C	sce03010
YLR009W	sce03010
YLR029C	sce03010
YLR048W	sce03010
YLR061W	sce03010
YLR075W	sce03010

YLR167W	sce03010
YLR185W	sce03010
YLR264W	sce03010
YLR287C-A	sce03010
YLR325C	sce03010
YLR333C	sce03010
YLR340W	sce03010
YLR344W	sce03010
YLR367W	sce03010
YLR388W	sce03010
YLR406C	sce03010
YLR441C	sce03010
YLR448W	sce03010
YML024W	sce03010
YML026C	sce03010
YML063W	sce03010
YML073C	sce03010
YMR121C	sce03010
YMR142C	sce03010
YMR143W	sce03010
YMR194W	sce03010
YMR230W	sce03010
YMR242C	sce03010
YNL002C	sce03010
YNL067W	sce03010
YNL069C	sce03010
YNL081C	sce03010
YNL096C	sce03010
YNL162W	sce03010
YNL178W	sce03010
YNL301C	sce03010
YNL302C	sce03010
YOL039W	sce03010
YOL040C	sce03010
YOL120C	sce03010
YOL121C	sce03010
YOL127W	sce03010
YOR063W	sce03010
YOR096W	sce03010
YOR150W	sce03010
YOR167C	sce03010
YOR182C	sce03010
YOR234C	sce03010
YOR293W	sce03010

YOR312C	sce03010
YOR369C	sce03010
YPL079W	sce03010
YPL081W	sce03010
YPL090C	sce03010
YPL131W	sce03010
YPL143W	sce03010
YPL198W	sce03010
YPL220W	sce03010
YPL249C-A	sce03010
YPR043W	sce03010
YPR102C	sce03010
YPR132W	sce03010
YAL021C	sce03018
YBL026W	sce03018
YCR035C	sce03018
YCR077C	sce03018
YCR093W	sce03018
YDL111C	sce03018
YDL160C	sce03018
YDL165W	sce03018
YDL175C	sce03018
YDR280W	sce03018
YDR378C	sce03018
YEL015W	sce03018
YER068W	sce03018
YER112W	sce03018
YER146W	sce03018
YFL028C	sce03018
YGL173C	sce03018
YGL213C	sce03018
YGR095C	sce03018
YGR158C	sce03018
YGR195W	sce03018
YGR254W	sce03018
YHR069C	sce03018
YHR081W	sce03018
YHR174W	sce03018
YIL038C	sce03018
YIL079C	sce03018
YJL050W	sce03018
YJL124C	sce03018
YJR022W	sce03018
YJR045C	sce03018

YKR002W	sce03018
YLR187W	sce03018
YLR259C	sce03018
YLR270W	sce03018
YLR398C	sce03018
YLR438C-A	sce03018
YMR323W	sce03018
YNL118C	sce03018
YNL147W	sce03018
YNL232W	sce03018
YNL278W	sce03018
YNL288W	sce03018
YNL299W	sce03018
YNR024W	sce03018
YNR052C	sce03018
YOL021C	sce03018
YOL115W	sce03018
YOL142W	sce03018
YOL149W	sce03018
YOR001W	sce03018
YOR048C	sce03018
YOR076C	sce03018
YOR393W	sce03018
YPR189W	sce03018
YBR154C	sce03020
YDL140C	sce03020
YDL150W	sce03020
YDR045C	sce03020
YDR156W	sce03020
YDR404C	sce03020
YGL070C	sce03020
YHR143W-A	sce03020
YIL021W	sce03020
YJL140W	sce03020
YJL148W	sce03020
YJR063W	sce03020
YKL144C	sce03020
YNL113W	sce03020
YNL151C	sce03020
YNL248C	sce03020
YNR003C	sce03020
YOL005C	sce03020
YOR116C	sce03020
YOR151C	sce03020

YOR207C	sce03020
YOR210W	sce03020
YOR224C	sce03020
YOR340C	sce03020
YOR341W	sce03020
YPR010C	sce03020
YPR110C	sce03020
YPR187W	sce03020
YPR190C	sce03020
YBR198C	sce03022
YCR042C	sce03022
YDR145W	sce03022
YDR167W	sce03022
YDR311W	sce03022
YER148W	sce03022
YGL112C	sce03022
YGR005C	sce03022
YGR186W	sce03022
YGR274C	sce03022
YKL028W	sce03022
YKL058W	sce03022
YKR062W	sce03022
YLR005W	sce03022
YML015C	sce03022
YML098W	sce03022
YMR227C	sce03022
YMR236W	sce03022
YOR194C	sce03022
YPL122C	sce03022
YPL129W	sce03022
YPR056W	sce03022
YPR086W	sce03022
YAR007C	sce03030
YBL023C	sce03030
YBL035C	sce03030
YBR087W	sce03030
YBR088C	sce03030
YBR202W	sce03030
YBR278W	sce03030
YDL102W	sce03030
YDL164C	sce03030
YDR121W	sce03030
YEL032W	sce03030
YGL201C	sce03030

YHR164C	sce03030
YIR008C	sce03030
YJR006W	sce03030
YJR043C	sce03030
YJR068W	sce03030
YKL045W	sce03030
YKL113C	sce03030
YLR274W	sce03030
YMR234W	sce03030
YNL072W	sce03030
YNL102W	sce03030
YNL262W	sce03030
YNL290W	sce03030
YNL312W	sce03030
YOL094C	sce03030
YOR217W	sce03030
YPR019W	sce03030
YPR175W	sce03030
YAL005C	sce03040
YAL032C	sce03040
YBL026W	sce03040
YBL075C	sce03040
YBR055C	sce03040
YBR065C	sce03040
YBR237W	sce03040
YCR063W	sce03040
YDL030W	sce03040
YDL043C	sce03040
YDL084W	sce03040
YDL098C	sce03040
YDR088C	sce03040
YDR163W	sce03040
YDR243C	sce03040
YDR364C	sce03040
YDR378C	sce03040
YDR381W	sce03040
YDR416W	sce03040
YDR473C	sce03040
YEL026W	sce03040
YER013W	sce03040
YER029C	sce03040
YER103W	sce03040
YER112W	sce03040
YER146W	sce03040

YER172C	sce03040
YFL017W-A	sce03040
YFR005C	sce03040
YGL120C	sce03040
YGR006W	sce03040
YGR074W	sce03040
YGR075C	sce03040
YGR091W	sce03040
YHR165C	sce03040
YIL061C	sce03040
YJR022W	sce03040
YJR050W	sce03040
YKL012W	sce03040
YKL173W	sce03040
YKR086W	sce03040
YLL024C	sce03040
YLL036C	sce03040
YLR117C	sce03040
YLR147C	sce03040
YLR275W	sce03040
YLR438C-A	sce03040
YML049C	sce03040
YMR125W	sce03040
YMR213W	sce03040
YMR240C	sce03040
YNL112W	sce03040
YNL138W-A	sce03040
YNL139C	sce03040
YNL147W	sce03040
YNL253W	sce03040
YNR011C	sce03040
YOR159C	sce03040
YOR308C	sce03040
YOR319W	sce03040
YPL151C	sce03040
YPL178W	sce03040
YPL213W	sce03040
YPR082C	sce03040
YPR094W	sce03040
YPR178W	sce03040
YPR182W	sce03040
YBL041W	sce03050
YBR173C	sce03050
YDL007W	sce03050

YDL097C	sce03050
YDL147W	sce03050
YDR363W-A	sce03050
YDR394W	sce03050
YDR427W	sce03050
YER012W	sce03050
YER021W	sce03050
YER094C	sce03050
YFL007W	sce03050
YFR004W	sce03050
YFR050C	sce03050
YFR052W	sce03050
YGL011C	sce03050
YGL048C	sce03050
YGR135W	sce03050
YGR253C	sce03050
YHR027C	sce03050
YHR200W	sce03050
YIL075C	sce03050
YJL001W	sce03050
YKL145W	sce03050
YLR421C	sce03050
YML092C	sce03050
YMR314W	sce03050
YOL038W	sce03050
YOR117W	sce03050
YOR157C	sce03050
YOR259C	sce03050
YOR261C	sce03050
YOR362C	sce03050
YPR103W	sce03050
YPR108W	sce03050
YBR283C	sce03060
YDL092W	sce03060
YDR086C	sce03060
YDR292C	sce03060
YER019C-A	sce03060
YER087C-B	sce03060
YER154W	sce03060
YIR022W	sce03060
YJL034W	sce03060
YKL154W	sce03060
YLR066W	sce03060
YLR378C	sce03060

YML055W	sce03060
YML105C	sce03060
YMR035W	sce03060
YMR150C	sce03060
YOR254C	sce03060
YPL094C	sce03060
YPL210C	sce03060
YPL243W	sce03060
YPR088C	sce03060
YAL015C	sce03410
YBL019W	sce03410
YBR088C	sce03410
YBR278W	sce03410
YDL102W	sce03410
YDL164C	sce03410
YDR121W	sce03410
YER142C	sce03410
YJR006W	sce03410
YJR043C	sce03410
YKL113C	sce03410
YKL114C	sce03410
YML021C	sce03410
YML060W	sce03410
YNL262W	sce03410
YOL043C	sce03410
YPR175W	sce03410
YAR007C	sce03420
YBR087W	sce03420
YBR088C	sce03420
YBR278W	sce03420
YDL102W	sce03420
YDL108W	sce03420
YDL164C	sce03420
YDR030C	sce03420
YDR079C-A	sce03420
YDR121W	sce03420
YDR311W	sce03420
YDR460W	sce03420
YEL037C	sce03420
YER162C	sce03420
YER171W	sce03420
YGR258C	sce03420
YIL143C	sce03420
YJL092W	sce03420

YJR006W	sce03420
YJR035W	sce03420
YJR043C	sce03420
YJR068W	sce03420
YLR005W	sce03420
YML095C	sce03420
YMR201C	sce03420
YNL262W	sce03420
YNL290W	sce03420
YNL312W	sce03420
YOL094C	sce03420
YOL133W	sce03420
YOR217W	sce03420
YPL022W	sce03420
YPL122C	sce03420
YPR025C	sce03420
YPR056W	sce03420
YPR175W	sce03420
YAR007C	sce03430
YBR087W	sce03430
YBR088C	sce03430
YCR092C	sce03430
YDL102W	sce03430
YDL164C	sce03430
YDR097C	sce03430
YJL092W	sce03430
YJR006W	sce03430
YJR043C	sce03430
YJR068W	sce03430
YMR167W	sce03430
YNL290W	sce03430
YNL312W	sce03430
YOL090W	sce03430
YOL094C	sce03430
YOR033C	sce03430
YOR217W	sce03430
YPL164C	sce03430
YAR007C	sce03440
YBR073W	sce03440
YDL059C	sce03440
YDL102W	sce03440
YDR004W	sce03440
YDR076W	sce03440
YDR363W-A	sce03440

YDR369C	sce03440
YDR386W	sce03440
YER095W	sce03440
YGL163C	sce03440
YJR006W	sce03440
YJR043C	sce03440
YLR234W	sce03440
YML032C	sce03440
YMR190C	sce03440
YMR224C	sce03440
YNL250W	sce03440
YNL312W	sce03440
YCR014C	sce03450
YDR369C	sce03450
YGL090W	sce03450
YKL113C	sce03450
YLR265C	sce03450
YMR106C	sce03450
YMR224C	sce03450
YMR284W	sce03450
YNL250W	sce03450
YOR005C	sce03450
YAL041W	sce04011
YBL016W	sce04011
YBL105C	sce04011
YBR083W	sce04011
YBR200W	sce04011
YCL027W	sce04011
YCR073C	sce04011
YDL159W	sce04011
YDL235C	sce04011
YDR103W	sce04011
YDR461W	sce04011
YDR480W	sce04011
YER111C	sce04011
YER118C	sce04011
YFL026W	sce04011
YGL089C	sce04011
YGR032W	sce04011
YGR040W	sce04011
YGR088W	sce04011
YHL007C	sce04011
YHR005C	sce04011
YHR030C	sce04011

YHR084W	sce04011
YIL147C	sce04011
YJL095W	sce04011
YJL128C	sce04011
YJL157C	sce04011
YJR086W	sce04011
YKL062W	sce04011
YKL178C	sce04011
YKR095W	sce04011
YLR006C	sce04011
YLR113W	sce04011
YLR182W	sce04011
YLR229C	sce04011
YLR332W	sce04011
YLR342W	sce04011
YLR362W	sce04011
YML004C	sce04011
YMR037C	sce04011
YMR043W	sce04011
YNL053W	sce04011
YNL098C	sce04011
YNL145W	sce04011
YNL271C	sce04011
YNL283C	sce04011
YNR031C	sce04011
YOL105C	sce04011
YOR008C	sce04011
YOR212W	sce04011
YOR231W	sce04011
YPL049C	sce04011
YPL089C	sce04011
YPL187W	sce04011
YPR165W	sce04011
YBL105C	sce04070
YBR029C	sce04070
YBR109C	sce04070
YDR208W	sce04070
YDR287W	sce04070
YFR019W	sce04070
YHR046C	sce04070
YLR240W	sce04070
YLR305C	sce04070
YNL106C	sce04070
YNL267W	sce04070

YOL065C	sce04070
YOR109W	sce04070
YPL268W	sce04070
YPR113W	sce04070
YAL016W	sce04111
YAL024C	sce04111
YAL040C	sce04111
YAR019C	sce04111
YBL016W	sce04111
YBL023C	sce04111
YBL084C	sce04111
YBL097W	sce04111
YBR060C	sce04111
YBR093C	sce04111
YBR112C	sce04111
YBR133C	sce04111
YBR135W	sce04111
YBR136W	sce04111
YBR160W	sce04111
YBR202W	sce04111
YBR274W	sce04111
YCL061C	sce04111
YCR084C	sce04111
YDL003W	sce04111
YDL008W	sce04111
YDL017W	sce04111
YDL028C	sce04111
YDL056W	sce04111
YDL101C	sce04111
YDL106C	sce04111
YDL127W	sce04111
YDL132W	sce04111
YDL134C	sce04111
YDL155W	sce04111
YDL188C	sce04111
YDR052C	sce04111
YDR110W	sce04111
YDR113C	sce04111
YDR118W	sce04111
YDR146C	sce04111
YDR180W	sce04111
YDR217C	sce04111
YDR260C	sce04111
YDR325W	sce04111

YDR328C	sce04111
YDR451C	sce04111
YDR499W	sce04111
YDR507C	sce04111
YEL032W	sce04111
YER111C	sce04111
YER147C	sce04111
YER173W	sce04111
YFL008W	sce04111
YFL009W	sce04111
YFL029C	sce04111
YFR028C	sce04111
YFR031C	sce04111
YFR034C	sce04111
YFR036W	sce04111
YGL003C	sce04111
YGL086W	sce04111
YGL116W	sce04111
YGL190C	sce04111
YGL201C	sce04111
YGL240W	sce04111
YGR092W	sce04111
YGR098C	sce04111
YGR108W	sce04111
YGR109C	sce04111
YGR113W	sce04111
YGR188C	sce04111
YGR233C	sce04111
YHR118C	sce04111
YHR152W	sce04111
YHR166C	sce04111
YIL026C	sce04111
YIL046W	sce04111
YIL106W	sce04111
YJL013C	sce04111
YJL030W	sce04111
YJL074C	sce04111
YJL076W	sce04111
YJL157C	sce04111
YJL187C	sce04111
YJL194W	sce04111
YJR046W	sce04111
YJR053W	sce04111
YJR090C	sce04111

YKL022C	sce04111
YKL101W	sce04111
YLL004W	sce04111
YLR079W	sce04111
YLR086W	sce04111
YLR102C	sce04111
YLR103C	sce04111
YLR127C	sce04111
YLR176C	sce04111
YLR182W	sce04111
YLR210W	sce04111
YLR272C	sce04111
YLR274W	sce04111
YLR288C	sce04111
YML027W	sce04111
YML064C	sce04111
YML065W	sce04111
YMR001C	sce04111
YMR036C	sce04111
YMR043W	sce04111
YMR055C	sce04111
YMR199W	sce04111
YNL172W	sce04111
YNL261W	sce04111
YNL289W	sce04111
YOL001W	sce04111
YOL133W	sce04111
YOR026W	sce04111
YOR083W	sce04111
YOR195W	sce04111
YOR249C	sce04111
YOR368W	sce04111
YPL031C	sce04111
YPL153C	sce04111
YPL194W	sce04111
YPL256C	sce04111
YPR019W	sce04111
YPR111W	sce04111
YPR119W	sce04111
YPR120C	sce04111
YPR162C	sce04111
YAL016W	sce04113
YAL040C	sce04113
YAR019C	sce04113

YBL023C	sce04113
YBL084C	sce04113
YBR045C	sce04113
YBR060C	sce04113
YBR136W	sce04113
YBR160W	sce04113
YBR202W	sce04113
YBR274W	sce04113
YCL067C	sce04113
YCR039C	sce04113
YCR096C	sce04113
YCR097W	sce04113
YDL008W	sce04113
YDL017W	sce04113
YDL035C	sce04113
YDL134C	sce04113
YDL138W	sce04113
YDL155W	sce04113
YDL188C	sce04113
YDL194W	sce04113
YDL245C	sce04113
YDR052C	sce04113
YDR113C	sce04113
YDR118W	sce04113
YDR207C	sce04113
YDR217C	sce04113
YDR260C	sce04113
YDR285W	sce04113
YDR310C	sce04113
YDR342C	sce04113
YDR343C	sce04113
YDR345C	sce04113
YDR439W	sce04113
YDR477W	sce04113
YDR523C	sce04113
YEL032W	sce04113
YEL069C	sce04113
YER020W	sce04113
YER106W	sce04113
YER111C	sce04113
YER133W	sce04113
YER173W	sce04113
YER179W	sce04113
YFL008W	sce04113

YFL011W	sce04113
YFL033C	sce04113
YFR028C	sce04113
YFR036W	sce04113
YGL086W	sce04113
YGL116W	sce04113
YGL201C	sce04113
YGL240W	sce04113
YGR044C	sce04113
YGR059W	sce04113
YGR098C	sce04113
YGR108W	sce04113
YGR109C	sce04113
YGR188C	sce04113
YGR225W	sce04113
YHL022C	sce04113
YHR092C	sce04113
YHR094C	sce04113
YHR096C	sce04113
YHR118C	sce04113
YHR124W	sce04113
YHR152W	sce04113
YHR166C	sce04113
YHR184W	sce04113
YIL026C	sce04113
YIL072W	sce04113
YIR025W	sce04113
YJL005W	sce04113
YJL030W	sce04113
YJL074C	sce04113
YJL106W	sce04113
YJL164C	sce04113
YJL187C	sce04113
YJL194W	sce04113
YJL214W	sce04113
YJL219W	sce04113
YJR046W	sce04113
YJR094C	sce04113
YJR158W	sce04113
YKL022C	sce04113
YKL062W	sce04113
YKL166C	sce04113
YKL203C	sce04113
YLL004W	sce04113

YLR079W	sce04113
YLR081W	sce04113
YLR102C	sce04113
YLR103C	sce04113
YLR127C	sce04113
YLR182W	sce04113
YLR210W	sce04113
YLR263W	sce04113
YLR274W	sce04113
YLR288C	sce04113
YML065W	sce04113
YMR001C	sce04113
YMR011W	sce04113
YMR016C	sce04113
YMR037C	sce04113
YMR139W	sce04113
YNL098C	sce04113
YNL172W	sce04113
YNL261W	sce04113
YNR072W	sce04113
YOL156W	sce04113
YOR014W	sce04113
YOR073W	sce04113
YOR195W	sce04113
YOR249C	sce04113
YOR313C	sce04113
YOR351C	sce04113
YOR368W	sce04113
YPL153C	sce04113
YPL194W	sce04113
YPL203W	sce04113
YPL256C	sce04113
YPR007C	sce04113
YPR019W	sce04113
YPR120C	sce04113
YPR162C	sce04113
YBL084C	sce04120
YBR082C	sce04120
YDL008W	sce04120
YDL064W	sce04120
YDL132W	sce04120
YDL190C	sce04120
YDR030C	sce04120
YDR054C	sce04120

YDR059C	sce04120
YDR092W	sce04120
YDR118W	sce04120
YDR177W	sce04120
YDR260C	sce04120
YDR328C	sce04120
YDR390C	sce04120
YDR457W	sce04120
YEL012W	sce04120
YER100W	sce04120
YER125W	sce04120
YFL009W	sce04120
YFR036W	sce04120
YGL003C	sce04120
YGL058W	sce04120
YGL116W	sce04120
YGL141W	sce04120
YGL240W	sce04120
YGR003W	sce04120
YHR166C	sce04120
YIL046W	sce04120
YJR090C	sce04120
YKL010C	sce04120
YKL022C	sce04120
YKL210W	sce04120
YLL036C	sce04120
YLR102C	sce04120
YLR127C	sce04120
YLR306W	sce04120
YMR022W	sce04120
YNL172W	sce04120
YOL013C	sce04120
YOL133W	sce04120
YOR249C	sce04120
YOR339C	sce04120
YPL046C	sce04120
YPR066W	sce04120
YPR180W	sce04120
YAL014C	sce04130
YAL030W	sce04130
YDR468C	sce04130
YDR498C	sce04130
YGL098W	sce04130
YGL212W	sce04130

YGR009C	sce04130
YHL031C	sce04130
YIL004C	sce04130
YKL006C-A	sce04130
YKL196C	sce04130
YLR026C	sce04130
YLR078C	sce04130
YLR268W	sce04130
YMR017W	sce04130
YMR183C	sce04130
YMR197C	sce04130
YOL018C	sce04130
YOR036W	sce04130
YOR075W	sce04130
YOR106W	sce04130
YOR327C	sce04130
YPL232W	sce04130
YBL078C	sce04140
YBR097W	sce04140
YBR128C	sce04140
YBR217W	sce04140
YEL013W	sce04140
YGL180W	sce04140
YHR171W	sce04140
YLL042C	sce04140
YLR240W	sce04140
YLR423C	sce04140
YMR159C	sce04140
YNL223W	sce04140
YNR007C	sce04140
YPL120W	sce04140
YPL149W	sce04140
YPR049C	sce04140
YPR185W	sce04140
gl:G00110	sce04144
YAL005C	sce04144
YBL037W	sce04144
YBL075C	sce04144
YCL008C	sce04144
YDL226C	sce04144
YDR069C	sce04144
YDR208W	sce04144
YDR486C	sce04144
YER103W	sce04144

YER122C	sce04144
YER125W	sce04144
YER144C	sce04144
YGL206C	sce04144
YGR044C	sce04144
YGR206W	sce04144
YHL002W	sce04144
YIL044C	sce04144
YJR058C	sce04144
YJR102C	sce04144
YKL002W	sce04144
YKL041W	sce04144
YKR031C	sce04144
YKR035W-A	sce04144
YLL001W	sce04144
YLL024C	sce04144
YLR025W	sce04144
YLR181C	sce04144
YLR229C	sce04144
YLR417W	sce04144
YML001W	sce04144
YMR077C	sce04144
YNR006W	sce04144
YOR275C	sce04144
YPL002C	sce04144
YPL065W	sce04144
YPR173C	sce04144
YAL058W	sce04145
YBR127C	sce04145
YBR283C	sce04145
YDL185W	sce04145
YDR086C	sce04145
YDR488C	sce04145
YEL027W	sce04145
YEL051W	sce04145
YER019C-A	sce04145
YER087C-B	sce04145
YFL037W	sce04145
YFL039C	sce04145
YFR019W	sce04145
YGR020C	sce04145
YHR026W	sce04145
YHR039C-A	sce04145
YKL080W	sce04145

YKR054C	sce04145
YLR240W	sce04145
YLR268W	sce04145
YLR378C	sce04145
YML001W	sce04145
YML085C	sce04145
YMR054W	sce04145
YNR006W	sce04145
YOR036W	sce04145
YOR075W	sce04145
YOR106W	sce04145
YOR270C	sce04145
YOR332W	sce04145
YPL234C	sce04145
YPR036W	sce04145
YPR079W	sce04145
YAR035W	sce04146
YDL065C	sce04146
YDL066W	sce04146
YDR142C	sce04146
YDR244W	sce04146
YDR256C	sce04146
YDR265W	sce04146
YDR329C	sce04146
YDR347W	sce04146
YEL020C	sce04146
YER015W	sce04146
YGL153W	sce04146
YGL205W	sce04146
YGR088W	sce04146
YHR008C	sce04146
YIL009W	sce04146
YJL210W	sce04146
YJR101W	sce04146
YJR104C	sce04146
YKL197C	sce04146
YLR174W	sce04146
YLR191W	sce04146
YLR251W	sce04146
YML042W	sce04146
YMR026C	sce04146
YMR208W	sce04146
YMR246W	sce04146
YNL009W	sce04146

YNL202W	sce04146
YNL329C	sce04146
YOR317W	sce04146
