# Supplementary material for: Predicting Protein Phenotypes Based on Protein-Protein Interaction Network
Source: PLoS One. 2011 Mar 10;6(3):e17668. doi: 10.1371/journal.pone.0017668 (PMC3053377; doi:10.1371/journal.pone.0017668)
Supplement: Table S2 — Yeast Protein-Protein Interaction Network. The protein-protein interaction were downloaded from STRING (Search Tool for the Retrieval of Interacting Genes/Proteins) (Jensen LJ, Kuhn M, Stark M, Chaffron S, Creevey C, et al. (2009) STRING 8–a global view on proteins and their functional interactions in 630 organisms. Nucleic acids research 37: D412-416.) with the highest confidence, i.e., the confidence scores are not less than 900. (PDF) [file pone.0017668.s002.pdf]

Table S2. Yeast Protein-Protein Interaction Network. The protein-protein interaction were downloaded from STRING (Search Tool for the Retrieval of Interacting Genes/Proteins) (Jensen LJ, Kuhn M, Stark M, Chaffron S, Creevey C, et al. (2009) STRING 8--a global view on proteins and their functional interactions in 630 organisms. Nucleic acids research 37: D412-416.) with the highest confidence, i.e., the confidence scores are not less than 900.

Protein	Protein	Confidence Score
Q0045	Q0105	982
Q0045	Q0250	999
Q0045	Q0275	999
Q0045	YBL045C	900
Q0045	YDL044C	952
Q0045	YDL067C	984
Q0045	YER141W	942
Q0045	YGL064C	903
Q0045	YGL187C	997
Q0045	YGL191W	978
Q0045	YGR112W	950
Q0045	YGR222W	989
Q0045	YHR051W	998
Q0045	YIL111W	995
Q0045	YLR038C	983
Q0045	YLR203C	999
Q0045	YLR395C	995
Q0045	YML129C	998
Q0045	YMR256C	997
Q0045	YNL052W	995
Q0045	YPL132W	982
Q0045	YPL172C	991
Q0065	Q0250	966
Q0065	Q0275	954
Q0070	Q0250	991
Q0070	Q0275	991
Q0070	YPL172C	932
Q0080	Q0085	970
Q0080	Q0130	965
Q0080	YBL099W	999
Q0080	YJR121W	999
Q0080	YPL078C	999
Q0085	Q0080	970
Q0085	Q0130	996
Q0085	YBL099W	999
Q0085	YBR039W	981
Q0085	YDL004W	901

Q0085	YDR298C	994
Q0085	YDR377W	972
Q0085	YER154W	986
Q0085	YJR121W	999
Q0085	YKL016C	957
Q0085	YLR393W	986
Q0085	YML081C-A	957
Q0085	YPL078C	999
Q0105	Q0045	982
Q0105	YBL045C	999
Q0105	YDL044C	941
Q0105	YDR375C	986
Q0105	YDR529C	999
Q0105	YEL024W	999
Q0105	YFR033C	999
Q0105	YGR183C	999
Q0105	YHL038C	988
Q0105	YHR001W-A	989
Q0105	YJL166W	999
Q0105	YJL209W	927
Q0105	YJR048W	989
Q0105	YOR017W	931
Q0105	YOR065W	999
Q0105	YPR191W	999
Q0115	YIR021W	996
Q0120	YBL045C	999
Q0120	YDR529C	999
Q0120	YEL024W	999
Q0120	YJL166W	999
Q0120	YLR382C	992
Q0120	YOR065W	999
Q0120	YPL160W	976
Q0130	Q0080	965
Q0130	Q0085	996
Q0130	YBL099W	999
Q0130	YER154W	988
Q0130	YJR121W	999
Q0130	YMR064W	903
Q0130	YMR282C	936
Q0130	YPL078C	999
Q0140	YBL090W	962
Q0140	YBR146W	962
Q0140	YBR251W	962
Q0140	YDL045W-A	962

Q0140	YDR036C	962
Q0140	YDR041W	962
Q0140	YDR175C	962
Q0140	YDR337W	962
Q0140	YDR347W	962
Q0140	YER050C	962
Q0140	YFR049W	962
Q0140	YGL129C	962
Q0140	YGR084C	962
Q0140	YGR165W	962
Q0140	YGR215W	962
Q0140	YHL004W	991
Q0140	YHR075C	962
Q0140	YIL093C	962
Q0140	YJR101W	962
Q0140	YJR113C	962
Q0140	YKL003C	962
Q0140	YKL155C	992
Q0140	YMR158W	962
Q0140	YMR188C	962
Q0140	YNL081C	962
Q0140	YNL137C	962
Q0140	YNL306W	962
Q0140	YNR036C	962
Q0140	YNR037C	962
Q0140	YOR158W	962
Q0140	YPL013C	962
Q0140	YPL029W	943
Q0140	YPL118W	962
Q0140	YPR166C	968
Q0250	Q0045	999
Q0250	Q0065	966
Q0250	Q0070	991
Q0250	Q0275	999
Q0250	YBL045C	966
Q0250	YBR024W	943
Q0250	YBR037C	995
Q0250	YDL067C	978
Q0250	YDL107W	922
Q0250	YDR231C	922
Q0250	YER154W	998
Q0250	YGL187C	978
Q0250	YGL191W	978
Q0250	YGR062C	922

Q0250	YHR051W	999
Q0250	YIL111W	978
Q0250	YJR121W	923
Q0250	YLR038C	988
Q0250	YLR395C	978
Q0250	YMR256C	978
Q0250	YMR257C	999
Q0250	YNL052W	978
Q0250	YPL132W	982
Q0250	YPL172C	976
Q0275	Q0045	999
Q0275	Q0065	954
Q0275	Q0070	991
Q0275	Q0250	999
Q0275	YBL090W	912
Q0275	YDL067C	978
Q0275	YDR494W	912
Q0275	YER153C	999
Q0275	YER154W	982
Q0275	YGL187C	990
Q0275	YGL191W	978
Q0275	YGR222W	999
Q0275	YHR051W	994
Q0275	YIL111W	978
Q0275	YLR038C	988
Q0275	YLR395C	978
Q0275	YMR256C	986
Q0275	YNL052W	978
Q0275	YNR045W	999
Q0275	YPL118W	912
Q0275	YPL132W	979
Q0275	YPL172C	966
R0020C	R0040C	984
R0040C	R0020C	984
YAL001C	YBR123C	999
YAL001C	YDR362C	999
YAL001C	YER148W	978
YAL001C	YGR047C	999
YAL001C	YGR246C	978
YAL001C	YHR143W-A	997
YAL001C	YOR110W	999
YAL001C	YPL007C	999
YAL002W	YDR495C	993
YAL002W	YLR148W	999

YAL002W	YLR396C	999
YAL002W	YMR231W	999
YAL002W	YOR089C	984
YAL002W	YPL045W	999
YAL003W	YBR118W	999
YAL003W	YBR191W	976
YAL003W	YDL075W	954
YAL003W	YDR064W	989
YAL003W	YDR382W	955
YAL003W	YEL034W	978
YAL003W	YGL123W	908
YAL003W	YGR285C	973
YAL003W	YHL033C	976
YAL003W	YHR064C	998
YAL003W	YIL052C	968
YAL003W	YKL006W	903
YAL003W	YKL081W	999
YAL003W	YKL180W	979
YAL003W	YLR048W	978
YAL003W	YLR061W	942
YAL003W	YLR075W	902
YAL003W	YLR249W	998
YAL003W	YLR340W	908
YAL003W	YLR388W	954
YAL003W	YLR448W	964
YAL003W	YNL178W	925
YAL003W	YPL037C	988
YAL003W	YPL048W	996
YAL003W	YPR080W	999
YAL005C	YAR002W	928
YAL005C	YBL005W	912
YAL005C	YBL075C	999
YAL005C	YBR101C	999
YAL005C	YBR155W	992
YAL005C	YCL028W	965
YAL005C	YDL229W	993
YAL005C	YDR172W	999
YAL005C	YER103W	997
YAL005C	YER161C	976
YAL005C	YGL073W	999
YAL005C	YIL016W	977
YAL005C	YJL034W	905
YAL005C	YJR032W	999
YAL005C	YLL024C	999

YAL005C	YLL026W	997
YAL005C	YMR186W	959
YAL005C	YNL007C	999
YAL005C	YNL064C	999
YAL005C	YNL209W	979
YAL005C	YNL229C	944
YAL005C	YOR027W	999
YAL005C	YOR232W	924
YAL005C	YPL106C	999
YAL005C	YPL240C	991
YAL007C	YAR002C-A	999
YAL007C	YDR087C	925
YAL007C	YDR100W	923
YAL007C	YGL200C	999
YAL007C	YLR342W	901
YAL007C	YML012W	999
YAL008W	YPL186C	913
YAL009W	YDL040C	922
YAL009W	YDL116W	991
YAL009W	YGL100W	918
YAL009W	YHR004C	999
YAL009W	YKL057C	918
YAL009W	YMR165C	914
YAL010C	YGR132C	909
YAL010C	YGR231C	907
YAL010C	YHR083W	921
YAL010C	YHR194W	905
YAL010C	YLL006W	998
YAL010C	YMR060C	988
YAL010C	YMR203W	944
YAL010C	YNL026W	932
YAL010C	YOL009C	990
YAL010C	YOR147W	905
YAL011W	YBR175W	910
YAL011W	YBR231C	999
YAL011W	YDL074C	969
YAL011W	YDR190C	999
YAL011W	YDR334W	999
YAL011W	YDR485C	999
YAL011W	YEL018W	972
YAL011W	YFL039C	961
YAL011W	YGL043W	974
YAL011W	YGL058W	961
YAL011W	YGL070C	907

YAL011W	YGL127C	977
YAL011W	YGL244W	918
YAL011W	YGR002C	988
YAL011W	YJL081C	999
YAL011W	YJL168C	961
YAL011W	YLR015W	920
YAL011W	YLR055C	929
YAL011W	YLR085C	999
YAL011W	YLR385C	954
YAL011W	YLR418C	939
YAL011W	YML041C	997
YAL011W	YML094W	930
YAL011W	YNL107W	999
YAL011W	YOL012C	995
YAL011W	YOR026W	902
YAL011W	YOR123C	908
YAL011W	YOR349W	920
YAL011W	YPL055C	968
YAL011W	YPL235W	999
YAL011W	YPL241C	918
YAL012W	YCL064C	905
YAL012W	YFR055W	925
YAL012W	YGR012W	978
YAL012W	YGR155W	996
YAL012W	YJR130C	942
YAL012W	YLR027C	901
YAL012W	YLR303W	940
YAL012W	YML082W	917
YAL012W	YMR300C	988
YAL012W	YNL247W	906
YAL013W	YBR095C	999
YAL013W	YBR103W	943
YAL013W	YCR033W	954
YAL013W	YDL076C	999
YAL013W	YDR207C	984
YAL013W	YGL194C	933
YAL013W	YIL084C	999
YAL013W	YJL168C	960
YAL013W	YKL185W	993
YAL013W	YKR029C	947
YAL013W	YMR263W	999
YAL013W	YNL097C	999
YAL013W	YNL330C	999
YAL013W	YOL004W	999

YAL013W	YOL012C	910
YAL013W	YPL139C	999
YAL013W	YPL181W	999
YAL014C	YAL030W	915
YAL014C	YER133W	965
YAL014C	YKL196C	975
YAL014C	YMR197C	982
YAL014C	YOR036W	981
YAL015C	YKL114C	993
YAL015C	YML060W	962
YAL015C	YML105C	954
YAL015C	YMR201C	905
YAL015C	YOL043C	986
YAL015C	YPL022W	933
YAL016W	YAL024C	989
YAL016W	YDL134C	999
YAL016W	YDL188C	999
YAL016W	YGL190C	999
YAL016W	YGR161C	996
YAL016W	YHR158C	958
YAL016W	YIL153W	967
YAL016W	YML109W	999
YAL016W	YMR028W	925
YAL016W	YMR273C	999
YAL016W	YNL127W	965
YAL016W	YOR014W	999
YAL016W	YPL152W	999
YAL017W	YDR099W	974
YAL017W	YGL112C	923
YAL017W	YMR227C	903
YAL017W	YOL045W	987
YAL020C	YBL071W-A	998
YAL020C	YKR048C	972
YAL020C	YPL086C	987
YAL021C	YBR279W	991
YAL021C	YCL061C	970
YAL021C	YCR093W	999
YAL021C	YDL101C	977
YAL021C	YDL160C	997
YAL021C	YDL165W	999
YAL021C	YDR138W	929
YAL021C	YDR252W	943
YAL021C	YDR392W	970
YAL021C	YER068W	999

YAL021C	YER111C	987
YAL021C	YER148W	983
YAL021C	YER165W	951
YAL021C	YFL028C	920
YAL021C	YGL094C	936
YAL021C	YGL178W	999
YAL021C	YGL207W	935
YAL021C	YGR092W	999
YAL021C	YGR104C	926
YAL021C	YGR116W	998
YAL021C	YGR134W	999
YAL021C	YIL038C	999
YAL021C	YJL127C	999
YAL021C	YLR226W	905
YAL021C	YLR418C	992
YAL021C	YML010W	944
YAL021C	YML032C	979
YAL021C	YNL288W	999
YAL021C	YNR052C	999
YAL021C	YOL051W	926
YAL021C	YPR072W	999
YAL023C	YDL095W	999
YAL023C	YJR143C	994
YAL023C	YOR008C	982
YAL023C	YOR321W	927
YAL023C	YPL240C	919
YAL024C	YAL016W	989
YAL024C	YAL041W	920
YAL024C	YAR019C	997
YAL024C	YBR160W	943
YAL024C	YFR028C	999
YAL024C	YGR098C	967
YAL024C	YHL007C	999
YAL024C	YHR158C	999
YAL024C	YIL106W	942
YAL024C	YIR009W	927
YAL024C	YJR043C	919
YAL024C	YLR229C	990
YAL024C	YML064C	999
YAL024C	YMR055C	999
YAL024C	YNL098C	996
YAL024C	YPL240C	919
YAL024C	YPR119W	987
YAL025C	YKL021C	942

YAL026C	YAL053W	994
YAL026C	YCR094W	999
YAL026C	YDL192W	996
YAL026C	YDR170C	983
YAL026C	YEL022W	987
YAL026C	YER166W	926
YAL026C	YLR447C	984
YAL026C	YNL243W	911
YAL026C	YNL323W	980
YAL026C	YOR115C	910
YAL026C	YPL048W	976
YAL027W	YCL024W	925
YAL027W	YDR174W	901
YAL027W	YDR381W	929
YAL027W	YDR507C	994
YAL027W	YML095C	999
YAL027W	YPL022W	999
YAL029C	YBL032W	994
YAL029C	YBR109C	999
YAL029C	YBR130C	999
YAL029C	YFL039C	999
YAL029C	YGL106W	999
YAL029C	YHR023W	999
YAL029C	YKL130C	999
YAL029C	YKL185W	991
YAL029C	YMR186W	933
YAL029C	YOR035C	998
YAL029C	YOR326W	999
YAL030W	YAL014C	915
YAL030W	YBL050W	982
YAL030W	YDR164C	987
YAL030W	YDR189W	998
YAL030W	YDR468C	998
YAL030W	YER143W	975
YAL030W	YGR009C	999
YAL030W	YGR142W	944
YAL030W	YJL036W	987
YAL030W	YJL204C	989
YAL030W	YMR017W	901
YAL030W	YMR183C	999
YAL030W	YMR197C	962
YAL030W	YNR049C	997
YAL030W	YOL018C	989
YAL030W	YOR036W	998

YAL030W	YOR106W	978
YAL030W	YOR327C	999
YAL030W	YPL232W	999
YAL032C	YDL209C	999
YAL032C	YDR364C	999
YAL032C	YDR416W	999
YAL032C	YER013W	932
YAL032C	YER172C	992
YAL032C	YGL120C	988
YAL032C	YGL128C	999
YAL032C	YGR278W	999
YAL032C	YHR165C	992
YAL032C	YJL203W	968
YAL032C	YJR050W	968
YAL032C	YKL095W	980
YAL032C	YKL173W	998
YAL032C	YLL036C	999
YAL032C	YLR117C	999
YAL032C	YLR345W	975
YAL032C	YML049C	999
YAL032C	YMR213W	999
YAL032C	YMR288W	999
YAL032C	YOR156C	916
YAL032C	YPL151C	999
YAL032C	YPL213W	999
YAL032C	YPR101W	989
YAL033W	YBL018C	999
YAL033W	YBR167C	999
YAL033W	YBR257W	999
YAL033W	YDR478W	952
YAL033W	YGR030C	999
YAL033W	YHR062C	999
YAL033W	YIR015W	982
YAL033W	YLR145W	979
YAL033W	YNL221C	999
YAL033W	YNL282W	999
YAL034C	YER110C	995
YAL034C	YGR274C	933
YAL034C	YOL004W	902
YAL034W-A	YBR107C	976
YAL034W-A	YBR211C	996
YAL034W-A	YDR254W	976
YAL034W-A	YDR318W	976
YAL034W-A	YER018C	999

YAL034W-A	YGL093W	961
YAL034W-A	YGR179C	963
YAL034W-A	YIL144W	995
YAL034W-A	YIR010W	999
YAL034W-A	YJL030W	979
YAL034W-A	YJR112W	999
YAL034W-A	YKL049C	989
YAL034W-A	YKL089W	980
YAL034W-A	YLR045C	902
YAL034W-A	YMR117C	998
YAL034W-A	YOL069W	993
YAL034W-A	YPL018W	995
YAL034W-A	YPL233W	999
YAL034W-A	YPR046W	995
YAL035W	YBL072C	908
YAL035W	YBR031W	998
YAL035W	YBR079C	985
YAL035W	YBR084C-A	913
YAL035W	YBR084W	956
YAL035W	YBR181C	940
YAL035W	YBR189W	923
YAL035W	YBR191W	928
YAL035W	YCR031C	930
YAL035W	YDL014W	996
YAL035W	YDL061C	921
YAL035W	YDL075W	930
YAL035W	YDL082W	993
YAL035W	YDL136W	912
YAL035W	YDR012W	929
YAL035W	YDR060W	941
YAL035W	YDR064W	968
YAL035W	YDR091C	974
YAL035W	YDR382W	931
YAL035W	YDR418W	998
YAL035W	YDR429C	976
YAL035W	YDR471W	916
YAL035W	YDR496C	999
YAL035W	YDR500C	914
YAL035W	YEL054C	922
YAL035W	YER025W	988
YAL035W	YER036C	981
YAL035W	YER056C-A	919
YAL035W	YER102W	998
YAL035W	YER117W	911

YAL035W	YFR031C-A	997
YAL035W	YGL030W	963
YAL035W	YGL076C	998
YAL035W	YGL103W	918
YAL035W	YGL123W	936
YAL035W	YGL130W	939
YAL035W	YGR027C	909
YAL035W	YGR085C	903
YAL035W	YGR118W	928
YAL035W	YGR148C	915
YAL035W	YGR214W	923
YAL035W	YHL001W	924
YAL035W	YHL015W	913
YAL035W	YHL033C	941
YAL035W	YHR010W	929
YAL035W	YHR052W	946
YAL035W	YHR203C	916
YAL035W	YIL018W	999
YAL035W	YIL052C	925
YAL035W	YIL148W	912
YAL035W	YJL122W	964
YAL035W	YJL177W	929
YAL035W	YJL190C	994
YAL035W	YJL191W	918
YAL035W	YJR094W-A	916
YAL035W	YJR123W	994
YAL035W	YJR145C	993
YAL035W	YKL006W	929
YAL035W	YKL156W	918
YAL035W	YKL180W	923
YAL035W	YKR024C	940
YAL035W	YLL045C	993
YAL035W	YLR029C	924
YAL035W	YLR048W	941
YAL035W	YLR061W	929
YAL035W	YLR075W	928
YAL035W	YLR175W	915
YAL035W	YLR185W	914
YAL035W	YLR192C	970
YAL035W	YLR264W	954
YAL035W	YLR340W	934
YAL035W	YLR344W	917
YAL035W	YLR367W	924
YAL035W	YLR388W	923

YAL035W	YLR448W	931
YAL035W	YML024W	924
YAL035W	YML063W	918
YAL035W	YMR142C	924
YAL035W	YMR146C	972
YAL035W	YMR194W	938
YAL035W	YMR242C	914
YAL035W	YMR260C	988
YAL035W	YMR309C	998
YAL035W	YNL067W	912
YAL035W	YNL096C	914
YAL035W	YNL178W	998
YAL035W	YNL301C	917
YAL035W	YOL127W	918
YAL035W	YOR063W	995
YAL035W	YOR096W	919
YAL035W	YOR167C	945
YAL035W	YOR234C	915
YAL035W	YOR293W	912
YAL035W	YOR361C	999
YAL035W	YPL081W	912
YAL035W	YPL090C	940
YAL035W	YPL131W	923
YAL035W	YPL143W	923
YAL035W	YPL198W	998
YAL035W	YPL220W	914
YAL035W	YPR041W	997
YAL035W	YPR102C	916
YAL035W	YPR132W	923
YAL036C	YDR152W	976
YAL036C	YGR162W	923
YAL036C	YOR091W	995
YAL038W	YBR218C	991
YAL038W	YBR221C	924
YAL038W	YCR012W	964
YAL038W	YDL174C	923
YAL038W	YDR050C	930
YAL038W	YDR226W	906
YAL038W	YDR454C	901
YAL038W	YER170W	905
YAL038W	YER178W	908
YAL038W	YGL062W	991
YAL038W	YGR240C	960
YAL038W	YGR254W	983

YAL038W	YHR174W	982
YAL038W	YJL005W	940
YAL038W	YJL148W	923
YAL038W	YJL164C	983
YAL038W	YKL029C	984
YAL038W	YKL060C	944
YAL038W	YKL110C	940
YAL038W	YKR097W	993
YAL038W	YLR044C	965
YAL038W	YLR377C	927
YAL038W	YMR205C	948
YAL038W	YMR323W	929
YAL038W	YNL102W	911
YAL038W	YNL307C	984
YAL038W	YNR001C	901
YAL038W	YOR347C	921
YAL038W	YOR393W	929
YAL038W	YPR010C	912
YAL038W	YPR187W	914
YAL039C	YER141W	901
YAL039C	YPL172C	931
YAL040C	YBR160W	999
YAL040C	YDL047W	993
YAL040C	YDL132W	916
YAL040C	YER111C	999
YAL040C	YER167W	999
YAL040C	YGR040W	906
YAL040C	YJL157C	999
YAL040C	YLR079W	998
YAL040C	YLR182W	996
YAL040C	YMR199W	997
YAL040C	YNL064C	908
YAL040C	YNL197C	987
YAL040C	YOR083W	997
YAL040C	YPL256C	999
YAL041W	YAL024C	920
YAL041W	YBL016W	912
YAL041W	YBR160W	922
YAL041W	YBR200W	999
YAL041W	YCR009C	998
YAL041W	YDL159W	901
YAL041W	YDR103W	998
YAL041W	YDR309C	979
YAL041W	YER114C	998

YAL041W	YGL233W	908
YAL041W	YGR014W	991
YAL041W	YGR152C	999
YAL041W	YGR221C	923
YAL041W	YHL007C	992
YAL041W	YHR061C	979
YAL041W	YJL157C	999
YAL041W	YJL187C	940
YAL041W	YJR086W	907
YAL041W	YLR229C	999
YAL041W	YLR362W	915
YAL041W	YMR232W	998
YAL041W	YNL007C	994
YAL041W	YNL293W	970
YAL041W	YNL298W	992
YAL041W	YOR188W	995
YAL041W	YOR212W	999
YAL041W	YPL115C	992
YAL041W	YPL161C	966
YAL041W	YPR165W	947
YAL042W	YHR060W	920
YAL042W	YKL119C	920
YAL042W	YML067C	999
YAL042W	YML130C	977
YAL043C	YBL076C	972
YAL043C	YBR119W	970
YAL043C	YDR046C	940
YAL043C	YDR195W	999
YAL043C	YDR228C	965
YAL043C	YDR301W	999
YAL043C	YER133W	999
YAL043C	YGR156W	999
YAL043C	YJL033W	961
YAL043C	YJR093C	999
YAL043C	YKL018W	999
YAL043C	YKL059C	999
YAL043C	YKR002W	999
YAL043C	YLR115W	999
YAL043C	YLR277C	999
YAL043C	YMR061W	999
YAL043C	YNL222W	999
YAL043C	YNL313C	940
YAL043C	YNL317W	999
YAL043C	YOR179C	999

YAL043C	YPL008W	929
YAL043C	YPR107C	999
YAL044C	YDR019C	999
YAL044C	YFL018C	988
YAL044C	YGL125W	929
YAL044C	YKR080W	900
YAL044C	YMR189W	999
YAL044C	YPL023C	913
YAL047C	YGR218W	972
YAL047C	YHR172W	999
YAL047C	YLR045C	998
YAL047C	YLR212C	999
YAL047C	YMR001C	997
YAL047C	YNL126W	999
YAL047C	YNL188W	977
YAL047C	YOR373W	962
YAL048C	YDL135C	904
YAL048C	YER136W	902
YAL048C	YLR190W	928
YAL048C	YLR229C	911
YAL048C	YNR051C	983
YAL048C	YPL154C	900
YAL048C	YPR165W	903
YAL049C	YNL094W	952
YAL049C	YPL240C	902
YAL051W	YLL004W	998
YAL051W	YOR363C	999
YAL053W	YAL026C	994
YAL053W	YBR199W	928
YAL053W	YOR153W	998
YAL054C	YBL015W	905
YAL054C	YER073W	901
YAL054C	YIR031C	907
YAL054C	YLR153C	952
YAL054C	YMR170C	900
YAL054C	YNL071W	915
YAL054C	YNL117W	907
YAL054C	YNR016C	902
YAL054C	YOR374W	901
YAL054C	YPL028W	915
YAL054C	YPL061W	900
YAL055W	YDR244W	906
YAL055W	YDR265W	931
YAL055W	YGR133W	996

YAL055W	YKL197C	902
YAL055W	YMR026C	913
YAL055W	YNL329C	917
YAL055W	YOL044W	981
YAL056W	YBR140C	963
YAL056W	YDL035C	908
YAL056W	YER020W	998
YAL056W	YJL005W	964
YAL056W	YOL081W	977
YAL056W	YOR371C	971
YAL058W	YBR229C	924
YAL058W	YCL052C	922
YAL058W	YEL002C	907
YAL058W	YFL025C	901
YAL058W	YHR101C	923
YAL058W	YJL034W	933
YAL058W	YJR117W	913
YAL058W	YPL227C	923
YAL059W	YBR115C	933
YAL059W	YDR395W	969
YAL059W	YMR186W	939
YAL062W	YDL171C	991
YAL062W	YFR055W	905
YAL062W	YGR124W	904
YAL062W	YLR089C	913
YAL062W	YMR250W	905
YAL062W	YOL033W	914
YAL062W	YOR375C	999
YAL062W	YPR035W	989
YAL062W	YPR145W	903
YAL063C	YAR050W	928
YAL063C	YCR032W	939
YAL063C	YHR211W	920
YAL063C	YIR019C	913
YAL063C	YKR102W	904
YAR002C-A	YAL007C	999
YAR002C-A	YGL054C	981
YAR002C-A	YGL200C	999
YAR002C-A	YML012W	999
YAR002W	YAL005C	928
YAR002W	YBL079W	940
YAR002W	YBR111W-A	908
YAR002W	YDL088C	947
YAR002W	YDL116W	978

YAR002W	YDL207W	976
YAR002W	YDL229W	928
YAR002W	YDR076W	926
YAR002W	YDR159W	989
YAR002W	YDR192C	945
YAR002W	YDR334W	914
YAR002W	YDR381W	941
YAR002W	YER110C	998
YAR002W	YER116C	915
YAR002W	YER165W	928
YAR002W	YFR002W	936
YAR002W	YGL086W	993
YAR002W	YGL097W	978
YAR002W	YGL100W	947
YAR002W	YGL172W	939
YAR002W	YGR119C	987
YAR002W	YIL031W	911
YAR002W	YKL057C	963
YAR002W	YKL068W	948
YAR002W	YKL113C	965
YAR002W	YKL186C	981
YAR002W	YKR082W	982
YAR002W	YLR018C	949
YAR002W	YLR293C	904
YAR002W	YLR335W	999
YAR002W	YLR347C	999
YAR002W	YML032C	990
YAR002W	YML041C	912
YAR002W	YMR047C	990
YAR002W	YMR153W	942
YAR002W	YNL189W	999
YAR002W	YNL250W	917
YAR002W	YOL012C	989
YAR002W	YOL072W	981
YAR002W	YOR098C	999
YAR002W	YOR257W	961
YAR002W	YPL020C	999
YAR002W	YPL153C	939
YAR002W	YPL169C	998
YAR003W	YBR175W	999
YAR003W	YBR258C	999
YAR003W	YDL020C	965
YAR003W	YDR334W	900
YAR003W	YDR363W-A	930

YAR003W	YDR469W	999
YAR003W	YFR010W	954
YAR003W	YHR119W	999
YAR003W	YHR200W	980
YAR003W	YKL018W	999
YAR003W	YLR015W	999
YAR003W	YML041C	903
YAR003W	YOL012C	976
YAR003W	YPL138C	999
YAR007C	YBL023C	992
YAR007C	YBL035C	991
YAR007C	YBL088C	959
YAR007C	YBR060C	908
YAR007C	YBR088C	957
YAR007C	YBR136W	999
YAR007C	YBR160W	910
YAR007C	YBR202W	942
YAR007C	YDL102W	923
YAR007C	YDR004W	940
YAR007C	YDR097C	986
YAR007C	YDR311W	977
YAR007C	YDR460W	977
YAR007C	YDR499W	997
YAR007C	YEL032W	915
YAR007C	YEL037C	901
YAR007C	YER095W	999
YAR007C	YER162C	988
YAR007C	YER171W	989
YAR007C	YGL201C	928
YAR007C	YGR258C	989
YAR007C	YHR164C	999
YAR007C	YIL143C	989
YAR007C	YIR002C	990
YAR007C	YIR008C	934
YAR007C	YJL173C	999
YAR007C	YJR006W	914
YAR007C	YKL045W	948
YAR007C	YLR005W	977
YAR007C	YLR103C	986
YAR007C	YLR274W	987
YAR007C	YLR288C	964
YAR007C	YML032C	999
YAR007C	YML095C	999
YAR007C	YMR201C	989

YAR007C	YMR224C	990
YAR007C	YMR234W	909
YAR007C	YNL102W	955
YAR007C	YNL250W	973
YAR007C	YNL262W	989
YAR007C	YNL312W	999
YAR007C	YOL090W	992
YAR007C	YOR368W	919
YAR007C	YPL022W	999
YAR007C	YPR019W	939
YAR007C	YPR175W	984
YAR008W	YBL051C	998
YAR008W	YLR105C	999
YAR008W	YMR059W	998
YAR008W	YPL083C	999
YAR014C	YCR073W-A	947
YAR014C	YDR195W	985
YAR014C	YER133W	999
YAR014C	YER155C	981
YAR014C	YGR285C	950
YAR014C	YHR064C	950
YAR014C	YHR158C	935
YAR014C	YKL193C	932
YAR014C	YPL209C	952
YAR015W	YDR408C	977
YAR015W	YGL234W	987
YAR015W	YLR359W	984
YAR015W	YMR300C	939
YAR015W	YOR128C	998
YAR015W	YPL240C	923
YAR018C	YGR285C	950
YAR018C	YHR064C	950
YAR019C	YAL024C	997
YAR019C	YDL220C	949
YAR019C	YFR028C	999
YAR019C	YGR092W	999
YAR019C	YGR152C	980
YAR019C	YHR152W	999
YAR019C	YIL106W	998
YAR019C	YJL076W	991
YAR019C	YJL194W	999
YAR019C	YLR079W	999
YAR019C	YML064C	999
YAR019C	YMR001C	999

YAR019C	YNL098C	967
YAR019C	YOR101W	967
YAR019C	YPL242C	929
YAR019C	YPR111W	974
YAR019C	YPR119W	999
YAR027W	YAR028W	925
YAR027W	YPL020C	924
YAR028W	YAR027W	925
YAR033W	YGL051W	997
YAR035W	YCR005C	933
YAR035W	YNL071W	905
YAR035W	YOR100C	951
YAR042W	YDL019C	910
YAR042W	YER120W	971
YAR042W	YHR195W	989
YAR050W	YAL063C	928
YAR050W	YCR032W	914
YAR050W	YER109C	979
YAR050W	YHR211W	926
YAR050W	YIR019C	921
YAR050W	YKR102W	903
YAR071W	YBR093C	967
YAR071W	YHR215W	999
YAR073W	YAR075W	943
YAR073W	YGR159C	998
YAR073W	YHR216W	990
YAR073W	YLR432W	980
YAR073W	YML056C	965
YAR075W	YAR073W	943
YBL002W	YBL003C	999
YBL002W	YBL046W	926
YBL002W	YBR009C	999
YBL002W	YBR010W	965
YBL002W	YDR075W	926
YBL002W	YDR121W	986
YBL002W	YDR224C	973
YBL002W	YDR225W	999
YBL002W	YGL241W	999
YBL002W	YHL007C	910
YBL002W	YKL213C	916
YBL002W	YKR048C	997
YBL002W	YML069W	984
YBL002W	YMR072W	986
YBL002W	YMR091C	947

YBL002W	YNL030W	961
YBL002W	YNL201C	926
YBL002W	YOL006C	903
YBL002W	YOL012C	999
YBL002W	YPR175W	983
YBL003C	YBL002W	999
YBL003C	YBL088C	951
YBL003C	YBR009C	998
YBL003C	YBR010W	986
YBL003C	YBR245C	996
YBL003C	YDR075W	926
YBL003C	YDR217C	930
YBL003C	YDR224C	999
YBL003C	YDR225W	997
YBL003C	YGL241W	999
YBL003C	YKR048C	999
YBL003C	YMR072W	989
YBL003C	YNL030W	986
YBL003C	YNL201C	955
YBL003C	YOL012C	995
YBL003C	YOR244W	966
YBL003C	YPL082C	983
YBL004W	YBL072C	931
YBL004W	YBR048W	931
YBL004W	YBR189W	931
YBL004W	YBR247C	999
YBL004W	YCL059C	999
YBL004W	YCR057C	999
YBL004W	YDL014W	995
YBL004W	YDL148C	999
YBL004W	YDL213C	993
YBL004W	YDR064W	935
YBL004W	YDR280W	923
YBL004W	YDR299W	998
YBL004W	YDR324C	997
YBL004W	YDR449C	999
YBL004W	YER082C	999
YBL004W	YER102W	929
YBL004W	YGL120C	960
YBL004W	YGR090W	999
YBL004W	YGR128C	997
YBL004W	YGR145W	997
YBL004W	YHR148W	997
YBL004W	YHR196W	999

YBL004W	YJL069C	954
YBL004W	YJL109C	999
YBL004W	YJR002W	999
YBL004W	YKR060W	997
YBL004W	YLL011W	998
YBL004W	YLR003C	969
YBL004W	YLR129W	997
YBL004W	YLR186W	998
YBL004W	YLR197W	997
YBL004W	YLR222C	997
YBL004W	YLR367W	976
YBL004W	YLR409C	998
YBL004W	YMR093W	997
YBL004W	YMR128W	997
YBL004W	YMR229C	997
YBL004W	YNL110C	920
YBL004W	YNL132W	994
YBL004W	YOL010W	999
YBL004W	YOR078W	980
YBL004W	YOR096W	935
YBL004W	YOR145C	997
YBL004W	YPL012W	994
YBL004W	YPL043W	998
YBL004W	YPL126W	997
YBL004W	YPL217C	998
YBL004W	YPL266W	997
YBL004W	YPR137W	999
YBL004W	YPR144C	999
YBL005W	YAL005C	912
YBL005W	YDR011W	987
YBL005W	YDR406W	935
YBL005W	YGL013C	999
YBL005W	YGR281W	934
YBL005W	YHR178W	916
YBL005W	YJL219W	966
YBL005W	YNL121C	971
YBL005W	YOL156W	963
YBL005W	YOR153W	999
YBL005W	YOR328W	925
YBL005W-A	YDR170W-A	970
YBL005W-A	YMR046C	998
YBL006C	YCR020W-B	981
YBL006C	YDR303C	981
YBL006C	YHR056C	956

YBL006C	YIL126W	960
YBL006C	YMR033W	960
YBL006C	YMR091C	982
YBL006C	YPL240C	919
YBL007C	YCL034W	976
YBL007C	YCR009C	955
YBL007C	YCR088W	999
YBL007C	YDR172W	994
YBL007C	YDR388W	999
YBL007C	YER133W	994
YBL007C	YFR024C-A	999
YBL007C	YGL181W	939
YBL007C	YGR229C	918
YBL007C	YGR268C	924
YBL007C	YHR016C	999
YBL007C	YHR114W	991
YBL007C	YIR006C	999
YBL007C	YJL020C	998
YBL007C	YJL201W	970
YBL007C	YLL039C	956
YBL007C	YLR337C	999
YBL007C	YNL020C	979
YBL007C	YNL084C	987
YBL007C	YNL094W	924
YBL007C	YNL106C	987
YBL007C	YNL238W	980
YBL007C	YNL243W	984
YBL007C	YOR181W	999
YBL007C	YPR171W	993
YBL008W	YBR009C	902
YBL008W	YBR215W	999
YBL008W	YBR289W	918
YBL008W	YDR224C	919
YBL008W	YGR063C	992
YBL008W	YGR116W	932
YBL008W	YJL115W	999
YBL008W	YJR140C	998
YBL008W	YML010W	922
YBL008W	YML069W	925
YBL008W	YML102W	994
YBL008W	YOR038C	999
YBL008W	YPR018W	999
YBL010C	YKR022C	932
YBL013W	YBR084W	956

YBL013W	YDR494W	939
YBL014C	YCL048W	998
YBL014C	YCR024C	998
YBL014C	YER148W	998
YBL014C	YJL025W	999
YBL014C	YKL125W	993
YBL014C	YLR141W	923
YBL014C	YML043C	999
YBL015W	YAL054C	905
YBL015W	YIR031C	909
YBL015W	YLR153C	905
YBL015W	YNL117W	908
YBL016W	YAL041W	912
YBL016W	YBR083W	999
YBL016W	YBR200W	920
YBL016W	YCL027W	999
YBL016W	YDL159W	999
YBL016W	YDR103W	999
YBL016W	YDR480W	999
YBL016W	YER020W	926
YBL016W	YER075C	998
YBL016W	YFL026W	914
YBL016W	YGL178W	947
YBL016W	YGR040W	999
YBL016W	YHR005C	999
YBL016W	YHR030C	907
YBL016W	YHR084W	999
YBL016W	YJL128C	922
YBL016W	YJL157C	999
YBL016W	YJR086W	955
YBL016W	YKL178C	910
YBL016W	YLR362W	999
YBL016W	YLR452C	998
YBL016W	YMR052W	919
YBL016W	YMR199W	909
YBL016W	YMR232W	998
YBL016W	YNL053W	999
YBL016W	YNL098C	930
YBL016W	YNL271C	988
YBL016W	YOR212W	999
YBL016W	YPL049C	999
YBL016W	YPL089C	911
YBL016W	YPL256C	909
YBL017C	YJL053W	993

YBL017C	YJL154C	998
YBL017C	YOR132W	906
YBL017C	YPL049C	929
YBL017C	YPR079W	969
YBL018C	YAL033W	999
YBL018C	YBR167C	997
YBL018C	YBR257W	999
YBL018C	YGR030C	999
YBL018C	YHR062C	999
YBL018C	YIR015W	949
YBL018C	YNL221C	999
YBL018C	YNL282W	999
YBL019W	YBR252W	988
YBL019W	YKL114C	999
YBL019W	YML021C	986
YBL019W	YML095C	992
YBL019W	YMR156C	948
YBL019W	YMR201C	981
YBL019W	YPL022W	999
YBL020W	YNL048W	933
YBL021C	YGL237C	999
YBL021C	YHR187W	976
YBL021C	YKL109W	997
YBL021C	YOR358W	999
YBL022C	YBR227C	964
YBL022C	YOR136W	927
YBL023C	YAR007C	992
YBL023C	YBR060C	997
YBL023C	YBR126C	938
YBL023C	YBR160W	984
YBL023C	YBR202W	999
YBL023C	YCL061C	971
YBL023C	YDL017W	999
YBL023C	YDR052C	999
YBL023C	YDR489W	980
YBL023C	YEL032W	999
YBL023C	YGL201C	998
YBL023C	YHR118C	977
YBL023C	YIL150C	999
YBL023C	YIR008C	901
YBL023C	YJL194W	999
YBL023C	YJR046W	998
YBL023C	YKL045W	901
YBL023C	YLL004W	983

YBL023C	YLR103C	999
YBL023C	YLR274W	999
YBL023C	YML065W	996
YBL023C	YNL102W	954
YBL023C	YNL261W	998
YBL023C	YNL262W	993
YBL023C	YNL312W	903
YBL023C	YPL001W	999
YBL023C	YPR019W	999
YBL023C	YPR162C	922
YBL023C	YPR175W	960
YBL024W	YDL201W	945
YBL024W	YDR165W	912
YBL025W	YDL113C	956
YBL025W	YLR141W	999
YBL025W	YMR270C	999
YBL025W	YOR295W	953
YBL026W	YBR055C	999
YBL026W	YBR154C	905
YBL026W	YCR020C-A	994
YBL026W	YCR077C	999
YBL026W	YDL098C	987
YBL026W	YDL160C	999
YBL026W	YDR378C	999
YBL026W	YDR404C	984
YBL026W	YDR473C	993
YBL026W	YEL026W	947
YBL026W	YER029C	999
YBL026W	YER112W	999
YBL026W	YER146W	999
YBL026W	YER172C	999
YBL026W	YFL017W-A	991
YBL026W	YGL070C	910
YBL026W	YGL173C	999
YBL026W	YGR074W	999
YBL026W	YGR075C	992
YBL026W	YGR091W	998
YBL026W	YGR158C	973
YBL026W	YHR165C	999
YBL026W	YIL021W	907
YBL026W	YJL124C	999
YBL026W	YJL140W	984
YBL026W	YJR022W	999
YBL026W	YKL173W	999

YBL026W	YLR147C	999
YBL026W	YLR275W	999
YBL026W	YLR438C-A	999
YBL026W	YMR268C	999
YBL026W	YMR288W	913
YBL026W	YNL147W	999
YBL026W	YOL005C	917
YBL026W	YOL149W	998
YBL026W	YOR159C	998
YBL026W	YOR210W	917
YBL026W	YOR224C	933
YBL026W	YOR308C	996
YBL026W	YPL178W	911
YBL026W	YPR082C	998
YBL026W	YPR178W	997
YBL026W	YPR182W	997
YBL026W	YPR187W	919
YBL027W	YBL072C	920
YBL027W	YBL087C	997
YBL027W	YBL092W	999
YBL027W	YBR031W	998
YBL027W	YBR048W	998
YBL027W	YBR084C-A	901
YBL027W	YBR118W	908
YBL027W	YBR181C	997
YBL027W	YBR189W	902
YBL027W	YBR191W	999
YBL027W	YCR031C	995
YBL027W	YDL061C	948
YBL027W	YDL075W	999
YBL027W	YDL081C	928
YBL027W	YDL082W	996
YBL027W	YDL083C	960
YBL027W	YDL136W	999
YBL027W	YDL191W	916
YBL027W	YDR012W	999
YBL027W	YDR025W	966
YBL027W	YDR064W	996
YBL027W	YDR382W	995
YBL027W	YDR385W	914
YBL027W	YDR418W	990
YBL027W	YDR447C	996
YBL027W	YDR450W	921
YBL027W	YDR471W	995

YBL027W	YDR500C	902
YBL027W	YEL054C	910
YBL027W	YER056C-A	904
YBL027W	YER074W	994
YBL027W	YER102W	920
YBL027W	YER117W	975
YBL027W	YER131W	907
YBL027W	YFL034C-A	916
YBL027W	YFR031C-A	999
YBL027W	YFR032C-A	998
YBL027W	YGL014W	978
YBL027W	YGL030W	996
YBL027W	YGL031C	999
YBL027W	YGL076C	992
YBL027W	YGL103W	999
YBL027W	YGL123W	999
YBL027W	YGL135W	990
YBL027W	YGL147C	999
YBL027W	YGL189C	907
YBL027W	YGR027C	913
YBL027W	YGR034W	999
YBL027W	YGR085C	998
YBL027W	YGR118W	922
YBL027W	YGR148C	912
YBL027W	YGR214W	920
YBL027W	YHL001W	997
YBL027W	YHL015W	921
YBL027W	YHL033C	999
YBL027W	YHR021C	921
YBL027W	YHR141C	999
YBL027W	YHR203C	971
YBL027W	YIL018W	973
YBL027W	YIL052C	904
YBL027W	YIL069C	902
YBL027W	YIL133C	999
YBL027W	YIL148W	906
YBL027W	YJL136C	922
YBL027W	YJL177W	998
YBL027W	YJL189W	927
YBL027W	YJL190C	969
YBL027W	YJL191W	916
YBL027W	YJR094W-A	992
YBL027W	YJR123W	997
YBL027W	YJR145C	971

YBL027W	YKL006W	918
YBL027W	YKL156W	921
YBL027W	YKL180W	999
YBL027W	YKR057W	922
YBL027W	YKR094C	906
YBL027W	YLL045C	995
YBL027W	YLR029C	999
YBL027W	YLR048W	920
YBL027W	YLR061W	919
YBL027W	YLR075W	999
YBL027W	YLR150W	978
YBL027W	YLR167W	901
YBL027W	YLR185W	989
YBL027W	YLR264W	909
YBL027W	YLR287C-A	997
YBL027W	YLR325C	901
YBL027W	YLR333C	912
YBL027W	YLR340W	966
YBL027W	YLR344W	999
YBL027W	YLR367W	969
YBL027W	YLR388W	954
YBL027W	YLR406C	919
YBL027W	YLR441C	996
YBL027W	YLR448W	996
YBL027W	YML024W	920
YBL027W	YML026C	921
YBL027W	YML063W	996
YBL027W	YML073C	996
YBL027W	YMR121C	933
YBL027W	YMR142C	921
YBL027W	YMR194W	995
YBL027W	YMR230W	918
YBL027W	YMR242C	996
YBL027W	YMR260C	905
YBL027W	YNL067W	980
YBL027W	YNL069C	996
YBL027W	YNL096C	997
YBL027W	YNL162W	924
YBL027W	YNL178W	999
YBL027W	YNL301C	999
YBL027W	YNL302C	939
YBL027W	YNR037C	931
YBL027W	YOL039W	912
YBL027W	YOL040C	999

YBL027W	YOL120C	933
YBL027W	YOL121C	939
YBL027W	YOL127W	999
YBL027W	YOR063W	999
YBL027W	YOR096W	996
YBL027W	YOR133W	914
YBL027W	YOR167C	909
YBL027W	YOR234C	953
YBL027W	YOR293W	918
YBL027W	YOR312C	996
YBL027W	YOR369C	915
YBL027W	YPL079W	923
YBL027W	YPL081W	902
YBL027W	YPL090C	994
YBL027W	YPL131W	999
YBL027W	YPL143W	953
YBL027W	YPL198W	930
YBL027W	YPL220W	914
YBL027W	YPL249C-A	996
YBL027W	YPR043W	932
YBL027W	YPR080W	908
YBL027W	YPR102C	997
YBL027W	YPR132W	922
YBL030C	YBR085W	993
YBL030C	YMR056C	977
YBL030C	YNL083W	980
YBL030C	YPR024W	905
YBL032W	YAL029C	994
YBL032W	YGL049C	988
YBL032W	YGR162W	981
YBL032W	YHR135C	996
YBL032W	YMR310C	950
YBL032W	YOL139C	962
YBL033C	YBR153W	994
YBL033C	YBR256C	924
YBL033C	YDR487C	995
YBL033C	YOL143C	906
YBL034C	YEL003W	951
YBL034C	YEL061C	933
YBL034C	YFL037W	997
YBL034C	YGL173C	964
YBL034C	YGR078C	951
YBL034C	YGR113W	958
YBL034C	YGR188C	944

YBL034C	YKL144C	940
YBL034C	YLR200W	975
YBL034C	YML094W	976
YBL034C	YML124C	964
YBL034C	YNL153C	951
YBL034C	YNR003C	946
YBL035C	YAR007C	991
YBL035C	YBR060C	932
YBL035C	YBR087W	997
YBL035C	YDL017W	918
YBL035C	YDR082W	998
YBL035C	YGL207W	981
YBL035C	YIL150C	989
YBL035C	YIR008C	999
YBL035C	YJR043C	905
YBL035C	YJR068W	970
YBL035C	YKL045W	999
YBL035C	YML065W	933
YBL035C	YML069W	981
YBL035C	YNL102W	999
YBL035C	YNL290W	998
YBL035C	YNR052C	987
YBL035C	YOL094C	996
YBL035C	YOR217W	998
YBL037W	YJR005W	996
YBL037W	YJR058C	999
YBL037W	YNL270C	962
YBL037W	YOL062C	999
YBL038W	YBR122C	972
YBL038W	YCL014W	942
YBL038W	YCR046C	992
YBL038W	YCR071C	972
YBL038W	YDR116C	995
YBL038W	YDR164C	927
YBL038W	YDR237W	999
YBL038W	YDR296W	992
YBL038W	YDR322W	997
YBL038W	YDR462W	997
YBL038W	YEL050C	953
YBL038W	YGR220C	999
YBL038W	YHR147C	972
YBL038W	YJL063C	999
YBL038W	YKL167C	972
YBL038W	YKL170W	942

YBL038W	YKR006C	988
YBL038W	YKR085C	998
YBL038W	YLR069C	918
YBL038W	YLR312W-A	972
YBL038W	YLR439W	998
YBL038W	YML025C	999
YBL038W	YMR024W	997
YBL038W	YMR193W	972
YBL038W	YNL005C	999
YBL038W	YNL185C	955
YBL038W	YNL252C	974
YBL038W	YNL284C	999
YBL038W	YNR036C	937
YBL038W	YNR037C	934
YBL038W	YOR150W	999
YBL038W	YPR100W	933
YBL039C	YBL105C	987
YBL039C	YDL140C	900
YBL039C	YFR004W	939
YBL039C	YJL148W	922
YBL039C	YJR063W	923
YBL039C	YJR103W	982
YBL039C	YKL067W	921
YBL039C	YMR217W	932
YBL039C	YNL113W	910
YBL039C	YNL151C	915
YBL039C	YNL248C	962
YBL039C	YOR151C	977
YBL039C	YOR388C	916
YBL039C	YPR010C	915
YBL039C	YPR110C	970
YBL039C	YPR190C	918
YBL040C	YCR067C	914
YBL040C	YDR077W	901
YBL040C	YHR079C	900
YBL040C	YLR026C	901
YBL041W	YBR173C	985
YBL041W	YCR073C	998
YBL041W	YDL007W	905
YBL041W	YDL020C	998
YBL041W	YDL097C	952
YBL041W	YDL147W	996
YBL041W	YDR394W	905
YBL041W	YDR427W	916

YBL041W	YER012W	999
YBL041W	YER021W	998
YBL041W	YER094C	999
YBL041W	YER105C	998
YBL041W	YFL007W	991
YBL041W	YFR004W	972
YBL041W	YFR050C	999
YBL041W	YFR052W	911
YBL041W	YGL011C	999
YBL041W	YGL048C	959
YBL041W	YGR135W	999
YBL041W	YGR253C	999
YBL041W	YHR027C	926
YBL041W	YHR200W	997
YBL041W	YIL007C	999
YBL041W	YIL075C	906
YBL041W	YJL001W	999
YBL041W	YKL145W	904
YBL041W	YLL039C	902
YBL041W	YML092C	999
YBL041W	YMR308C	962
YBL041W	YMR314W	999
YBL041W	YOL038W	999
YBL041W	YOR117W	909
YBL041W	YOR157C	999
YBL041W	YOR259C	920
YBL041W	YOR261C	910
YBL041W	YOR362C	999
YBL041W	YPR103W	999
YBL041W	YPR108W	931
YBL042C	YER021W	900
YBL045C	Q0045	900
YBL045C	Q0105	999
YBL045C	Q0120	999
YBL045C	Q0250	966
YBL045C	YDL074C	990
YBL045C	YDL189W	996
YBL045C	YDR529C	999
YBL045C	YEL024W	999
YBL045C	YFR033C	999
YBL045C	YGL187C	978
YBL045C	YGR183C	999
YBL045C	YHR001W-A	990
YBL045C	YJL143W	969

YBL045C	YJL166W	999
YBL045C	YJR048W	990
YBL045C	YLR090W	998
YBL045C	YNR017W	969
YBL045C	YOR065W	999
YBL045C	YPR191W	999
YBL046W	YBL002W	926
YBL046W	YDL112W	925
YBL046W	YDR075W	999
YBL046W	YDR224C	936
YBL046W	YDR293C	926
YBL046W	YGR063C	969
YBL046W	YML010W	999
YBL046W	YNL201C	999
YBL047C	YCR030C	999
YBL047C	YDL161W	947
YBL047C	YDR348C	936
YBL047C	YGR241C	973
YBL047C	YNL243W	921
YBL049W	YDR255C	929
YBL049W	YGL227W	929
YBL049W	YIL017C	929
YBL049W	YIL097W	929
YBL049W	YMR135C	921
YBL050W	YAL030W	982
YBL050W	YBR080C	999
YBL050W	YDR189W	994
YBL050W	YDR468C	967
YBL050W	YGL095C	996
YBL050W	YGL212W	988
YBL050W	YGR009C	924
YBL050W	YKL196C	999
YBL050W	YLR026C	999
YBL050W	YLR078C	962
YBL050W	YLR093C	999
YBL050W	YLR268W	999
YBL050W	YML001W	957
YBL050W	YMR197C	999
YBL050W	YOL018C	977
YBL050W	YOR106W	999
YBL050W	YPL232W	984
YBL051C	YAR008W	998
YBL051C	YBR136W	978
YBL051C	YDL165W	974

YBL051C	YJL138C	936
YBL051C	YKR059W	936
YBL051C	YMR186W	919
YBL051C	YPL240C	919
YBL052C	YGL207W	997
YBL052C	YGR252W	994
YBL052C	YJR082C	954
YBL052C	YOL012C	953
YBL052C	YOR064C	999
YBL052C	YPL129W	999
YBL052C	YPR031W	999
YBL056W	YBR097W	919
YBL056W	YDL060W	945
YBL056W	YDR071C	999
YBL056W	YER089C	948
YBL056W	YIL147C	976
YBL056W	YOR208W	939
YBL057C	YMR276W	951
YBL058W	YDL126C	999
YBL058W	YDL190C	992
YBL058W	YDR411C	929
YBL058W	YER133W	919
YBL058W	YFL044C	988
YBL058W	YKL213C	999
YBL060W	YOR094W	990
YBL061C	YBR023C	999
YBL061C	YGR229C	947
YBL061C	YLR207W	999
YBL061C	YLR342W	964
YBL061C	YNL233W	984
YBL063W	YEL061C	999
YBL063W	YOR058C	984
YBL063W	YPR141C	984
YBL064C	YDR069C	919
YBL066C	YML091C	929
YBL067C	YOL087C	998
YBL068W	YBR276C	996
YBL068W	YCR036W	914
YBL068W	YDL188C	946
YBL068W	YER099C	998
YBL068W	YHL011C	978
YBL068W	YIL076W	930
YBL068W	YKL181W	999
YBL068W	YML092C	967

YBL068W	YML106W	911
YBL068W	YMR300C	914
YBL068W	YOL061W	994
YBL068W	YOR095C	911
YBL068W	YPR074C	900
YBL069W	YGL008C	928
YBL071W-A	YAL020C	998
YBL071W-A	YDR385W	935
YBL071W-A	YGR200C	952
YBL071W-A	YIL103W	999
YBL071W-A	YKL191W	999
YBL071W-A	YLR384C	987
YBL071W-A	YOL121C	915
YBL071W-A	YOR096W	901
YBL071W-A	YPL086C	989
YBL072C	YAL035W	908
YBL072C	YBL004W	931
YBL072C	YBL027W	920
YBL072C	YBL087C	928
YBL072C	YBL092W	925
YBL072C	YBR031W	995
YBL072C	YBR048W	996
YBL072C	YBR079C	998
YBL072C	YBR084C-A	920
YBL072C	YBR118W	909
YBL072C	YBR181C	993
YBL072C	YBR189W	996
YBL072C	YBR191W	920
YBL072C	YBR247C	989
YBL072C	YCL011C	927
YBL072C	YCL037C	978
YBL072C	YCL059C	998
YBL072C	YCR031C	997
YBL072C	YDL014W	964
YBL072C	YDL060W	978
YBL072C	YDL061C	902
YBL072C	YDL075W	919
YBL072C	YDL081C	926
YBL072C	YDL082W	995
YBL072C	YDL083C	954
YBL072C	YDL136W	994
YBL072C	YDL148C	969
YBL072C	YDL191W	916
YBL072C	YDR012W	928

YBL072C	YDR025W	996
YBL072C	YDR064W	998
YBL072C	YDR324C	929
YBL072C	YDR382W	918
YBL072C	YDR385W	912
YBL072C	YDR418W	996
YBL072C	YDR429C	915
YBL072C	YDR447C	995
YBL072C	YDR449C	980
YBL072C	YDR450W	933
YBL072C	YDR471W	993
YBL072C	YDR500C	911
YBL072C	YEL054C	915
YBL072C	YER025W	931
YBL072C	YER074W	996
YBL072C	YER102W	996
YBL072C	YER117W	928
YBL072C	YER131W	910
YBL072C	YFL034C-A	913
YBL072C	YFR031C-A	994
YBL072C	YFR032C-A	929
YBL072C	YGL030W	933
YBL072C	YGL031C	904
YBL072C	YGL076C	995
YBL072C	YGL103W	993
YBL072C	YGL120C	938
YBL072C	YGL123W	906
YBL072C	YGL135W	995
YBL072C	YGL147C	997
YBL072C	YGL189C	910
YBL072C	YGR027C	921
YBL072C	YGR034W	920
YBL072C	YGR054W	974
YBL072C	YGR085C	994
YBL072C	YGR090W	940
YBL072C	YGR118W	928
YBL072C	YGR145W	927
YBL072C	YGR148C	904
YBL072C	YGR214W	933
YBL072C	YHL001W	996
YBL072C	YHL015W	998
YBL072C	YHL033C	996
YBL072C	YHL034C	982
YBL072C	YHR010W	900

YBL072C	YHR021C	931
YBL072C	YHR141C	924
YBL072C	YHR203C	996
YBL072C	YIL018W	915
YBL072C	YIL069C	996
YBL072C	YIL133C	921
YBL072C	YIL148W	906
YBL072C	YJL136C	929
YBL072C	YJL177W	994
YBL072C	YJL189W	920
YBL072C	YJL190C	996
YBL072C	YJL191W	919
YBL072C	YJR002W	933
YBL072C	YJR007W	903
YBL072C	YJR094W-A	926
YBL072C	YJR123W	996
YBL072C	YJR145C	997
YBL072C	YKL006W	918
YBL072C	YKL156W	931
YBL072C	YKL180W	994
YBL072C	YKR057W	929
YBL072C	YKR094C	906
YBL072C	YLL011W	927
YBL072C	YLL045C	994
YBL072C	YLR029C	994
YBL072C	YLR048W	925
YBL072C	YLR061W	915
YBL072C	YLR075W	997
YBL072C	YLR129W	929
YBL072C	YLR167W	900
YBL072C	YLR185W	911
YBL072C	YLR186W	931
YBL072C	YLR192C	917
YBL072C	YLR197W	933
YBL072C	YLR264W	909
YBL072C	YLR287C-A	992
YBL072C	YLR325C	915
YBL072C	YLR333C	920
YBL072C	YLR340W	997
YBL072C	YLR344W	920
YBL072C	YLR367W	912
YBL072C	YLR388W	902
YBL072C	YLR406C	919
YBL072C	YLR441C	997

YBL072C	YLR448W	995
YBL072C	YML024W	927
YBL072C	YML026C	921
YBL072C	YML063W	995
YBL072C	YML073C	995
YBL072C	YMR093W	931
YBL072C	YMR116C	988
YBL072C	YMR121C	915
YBL072C	YMR128W	937
YBL072C	YMR142C	926
YBL072C	YMR146C	997
YBL072C	YMR194W	995
YBL072C	YMR229C	937
YBL072C	YMR230W	929
YBL072C	YMR242C	995
YBL072C	YMR260C	907
YBL072C	YMR290C	940
YBL072C	YMR309C	913
YBL072C	YNL067W	922
YBL072C	YNL069C	920
YBL072C	YNL096C	994
YBL072C	YNL132W	962
YBL072C	YNL162W	924
YBL072C	YNL178W	997
YBL072C	YNL301C	924
YBL072C	YNL302C	923
YBL072C	YNL308C	980
YBL072C	YOL039W	911
YBL072C	YOL040C	944
YBL072C	YOL120C	924
YBL072C	YOL121C	923
YBL072C	YOL127W	994
YBL072C	YOL139C	916
YBL072C	YOR063W	994
YBL072C	YOR096W	996
YBL072C	YOR133W	912
YBL072C	YOR167C	909
YBL072C	YOR234C	925
YBL072C	YOR293W	929
YBL072C	YOR312C	997
YBL072C	YOR361C	997
YBL072C	YOR369C	923
YBL072C	YPL012W	942
YBL072C	YPL079W	920

YBL072C	YPL081W	917
YBL072C	YPL090C	995
YBL072C	YPL126W	931
YBL072C	YPL131W	994
YBL072C	YPL143W	925
YBL072C	YPL198W	995
YBL072C	YPL220W	916
YBL072C	YPL249C-A	927
YBL072C	YPR043W	926
YBL072C	YPR080W	909
YBL072C	YPR102C	917
YBL072C	YPR132W	928
YBL072C	YPR144C	980
YBL074C	YBR055C	995
YBL074C	YER029C	995
YBL074C	YER172C	992
YBL074C	YGR074W	994
YBL074C	YHR165C	999
YBL074C	YKL173W	999
YBL074C	YLR275W	995
YBL074C	YOR308C	990
YBL074C	YPR178W	977
YBL075C	YAL005C	999
YBL075C	YDL229W	906
YBL075C	YER103W	986
YBL075C	YGL073W	913
YBL075C	YJL034W	906
YBL075C	YLL024C	997
YBL075C	YNL007C	968
YBL075C	YNL064C	998
YBL075C	YNL209W	906
YBL075C	YOR027W	964
YBL075C	YOR232W	927
YBL075C	YOR250C	932
YBL076C	YAL043C	972
YBL076C	YBR058C	920
YBL076C	YBR123C	940
YBL076C	YDL100C	932
YBL076C	YDR037W	905
YBL076C	YDR341C	911
YBL076C	YGL105W	914
YBL076C	YGR264C	979
YBL076C	YHR020W	951
YBL076C	YLL018C	922

YBL076C	YOR168W	924
YBL076C	YPL040C	913
YBL076C	YPL160W	975
YBL078C	YBR217W	986
YBL078C	YHR171W	999
YBL078C	YKL103C	911
YBL078C	YMR159C	940
YBL078C	YNL223W	999
YBL078C	YNR007C	999
YBL078C	YOL082W	976
YBL078C	YPL149W	980
YBL079W	YAR002W	940
YBL079W	YDL088C	998
YBL079W	YDL116W	972
YBL079W	YDL207W	962
YBL079W	YDR159W	968
YBL079W	YER105C	997
YBL079W	YER107C	929
YBL079W	YFR002W	994
YBL079W	YGL086W	922
YBL079W	YGL092W	959
YBL079W	YGL100W	905
YBL079W	YGL172W	942
YBL079W	YGR119C	993
YBL079W	YIL115C	930
YBL079W	YJL030W	917
YBL079W	YJL041W	941
YBL079W	YJL061W	989
YBL079W	YKL057C	989
YBL079W	YKL068W	994
YBL079W	YKR082W	974
YBL079W	YLR018C	991
YBL079W	YLR208W	920
YBL079W	YLR335W	998
YBL079W	YLR347C	991
YBL079W	YML103C	988
YBL079W	YMR047C	993
YBL079W	YMR129W	999
YBL079W	YMR153W	999
YBL079W	YMR308C	920
YBL079W	YOR098C	998
YBL079W	YOR257W	961
YBL079W	YPL169C	968
YBL080C	YHR019C	917

YBL080C	YMR293C	999
YBL080C	YOR168W	916
YBL082C	YGL022W	918
YBL082C	YNL048W	997
YBL082C	YNL219C	984
YBL082C	YPR183W	909
YBL084C	YBR082C	917
YBL084C	YBR135W	930
YBL084C	YBR160W	994
YBL084C	YDL008W	999
YBL084C	YDL028C	929
YBL084C	YDR113C	980
YBL084C	YDR118W	999
YBL084C	YDR260C	999
YBL084C	YDR311W	916
YBL084C	YFR036W	999
YBL084C	YGL003C	999
YBL084C	YGL116W	999
YBL084C	YGL240W	999
YBL084C	YHR166C	999
YBL084C	YIR025W	999
YBL084C	YJL030W	991
YBL084C	YKL022C	999
YBL084C	YLL039C	902
YBL084C	YLR005W	906
YBL084C	YLR102C	999
YBL084C	YLR127C	999
YBL084C	YMR001C	999
YBL084C	YNL172W	999
YBL084C	YOR249C	999
YBL084C	YPR056W	912
YBL084C	YPR119W	983
YBL085W	YBR200W	999
YBL085W	YBR238C	994
YBL085W	YER114C	982
YBL085W	YER124C	924
YBL085W	YIL118W	990
YBL085W	YKR055W	988
YBL085W	YLR229C	912
YBL085W	YML109W	958
YBL087C	YBL027W	997
YBL087C	YBL072C	928
YBL087C	YBL092W	998
YBL087C	YBR031W	977

YBL087C	YBR048W	975
YBL087C	YBR084C-A	975
YBL087C	YBR118W	917
YBL087C	YBR181C	977
YBL087C	YBR189W	930
YBL087C	YBR191W	992
YBL087C	YCR031C	961
YBL087C	YDL061C	960
YBL087C	YDL075W	990
YBL087C	YDL081C	964
YBL087C	YDL082W	959
YBL087C	YDL136W	990
YBL087C	YDL171C	924
YBL087C	YDL191W	914
YBL087C	YDR012W	997
YBL087C	YDR025W	975
YBL087C	YDR041W	967
YBL087C	YDR064W	956
YBL087C	YDR361C	999
YBL087C	YDR382W	918
YBL087C	YDR385W	928
YBL087C	YDR418W	996
YBL087C	YDR447C	966
YBL087C	YDR450W	927
YBL087C	YDR471W	901
YBL087C	YDR500C	909
YBL087C	YEL054C	921
YBL087C	YER056C-A	901
YBL087C	YER074W	916
YBL087C	YER102W	928
YBL087C	YER117W	999
YBL087C	YER131W	929
YBL087C	YFL034C-A	910
YBL087C	YFR031C-A	999
YBL087C	YFR032C-A	940
YBL087C	YGL030W	933
YBL087C	YGL031C	993
YBL087C	YGL076C	994
YBL087C	YGL103W	994
YBL087C	YGL111W	968
YBL087C	YGL123W	983
YBL087C	YGL135W	990
YBL087C	YGL147C	998
YBL087C	YGL189C	916

YBL087C	YGR027C	939
YBL087C	YGR034W	989
YBL087C	YGR085C	987
YBL087C	YGR103W	974
YBL087C	YGR118W	930
YBL087C	YGR148C	920
YBL087C	YGR214W	944
YBL087C	YHL001W	933
YBL087C	YHL015W	911
YBL087C	YHL033C	991
YBL087C	YHR010W	901
YBL087C	YHR021C	930
YBL087C	YHR141C	996
YBL087C	YHR203C	975
YBL087C	YIL018W	981
YBL087C	YIL052C	901
YBL087C	YIL069C	916
YBL087C	YIL133C	996
YBL087C	YIL148W	906
YBL087C	YJL136C	931
YBL087C	YJL177W	970
YBL087C	YJL189W	923
YBL087C	YJL190C	998
YBL087C	YJL191W	958
YBL087C	YJR094W-A	992
YBL087C	YJR123W	965
YBL087C	YJR145C	975
YBL087C	YKL006W	930
YBL087C	YKL156W	930
YBL087C	YKL170W	935
YBL087C	YKL180W	997
YBL087C	YKR057W	931
YBL087C	YKR094C	906
YBL087C	YLL045C	959
YBL087C	YLR029C	992
YBL087C	YLR048W	928
YBL087C	YLR061W	912
YBL087C	YLR075W	995
YBL087C	YLR167W	901
YBL087C	YLR185W	990
YBL087C	YLR264W	904
YBL087C	YLR325C	961
YBL087C	YLR333C	935
YBL087C	YLR340W	971

YBL087C	YLR344W	997
YBL087C	YLR367W	972
YBL087C	YLR388W	966
YBL087C	YLR406C	915
YBL087C	YLR441C	970
YBL087C	YLR448W	933
YBL087C	YML024W	928
YBL087C	YML026C	927
YBL087C	YML063W	931
YBL087C	YML073C	955
YBL087C	YMR121C	942
YBL087C	YMR142C	936
YBL087C	YMR194W	929
YBL087C	YMR230W	925
YBL087C	YMR242C	974
YBL087C	YMR260C	913
YBL087C	YMR290C	972
YBL087C	YNL067W	981
YBL087C	YNL069C	944
YBL087C	YNL081C	967
YBL087C	YNL096C	932
YBL087C	YNL162W	920
YBL087C	YNL178W	990
YBL087C	YNL301C	991
YBL087C	YNL302C	922
YBL087C	YNR037C	980
YBL087C	YOL039W	922
YBL087C	YOL040C	988
YBL087C	YOL098C	921
YBL087C	YOL120C	923
YBL087C	YOL121C	962
YBL087C	YOL127W	998
YBL087C	YOR063W	999
YBL087C	YOR096W	906
YBL087C	YOR133W	927
YBL087C	YOR167C	925
YBL087C	YOR234C	929
YBL087C	YOR293W	925
YBL087C	YOR312C	932
YBL087C	YOR369C	980
YBL087C	YPL079W	936
YBL087C	YPL081W	916
YBL087C	YPL090C	901
YBL087C	YPL093W	972

YBL087C	YPL131W	998
YBL087C	YPL143W	930
YBL087C	YPL198W	934
YBL087C	YPL208W	999
YBL087C	YPL220W	911
YBL087C	YPL249C-A	930
YBL087C	YPR043W	933
YBL087C	YPR080W	925
YBL087C	YPR102C	998
YBL087C	YPR132W	930
YBL088C	YAR007C	959
YBL088C	YBL003C	951
YBL088C	YBR087W	918
YBL088C	YBR136W	999
YBL088C	YBR274W	989
YBL088C	YBR275C	952
YBL088C	YDL101C	992
YBL088C	YDL220C	999
YBL088C	YDR113C	926
YBL088C	YDR217C	995
YBL088C	YDR225W	948
YBL088C	YDR369C	999
YBL088C	YDR499W	993
YBL088C	YER095W	993
YBL088C	YER173W	989
YBL088C	YGL150C	938
YBL088C	YJL115W	914
YBL088C	YLR288C	989
YBL088C	YLR318W	993
YBL088C	YLR453C	953
YBL088C	YMR190C	903
YBL088C	YMR224C	999
YBL088C	YNL216W	934
YBL088C	YNL250W	945
YBL088C	YNL312W	955
YBL088C	YOR189W	914
YBL088C	YPL153C	999
YBL088C	YPL194W	999
YBL090W	Q0140	962
YBL090W	Q0275	912
YBL090W	YBR146W	988
YBL090W	YBR251W	999
YBL090W	YDL045W-A	986
YBL090W	YDR036C	972

YBL090W	YDR041W	988
YBL090W	YDR175C	988
YBL090W	YDR337W	988
YBL090W	YDR347W	988
YBL090W	YGL129C	988
YBL090W	YGR084C	974
YBL090W	YGR165W	988
YBL090W	YGR215W	988
YBL090W	YHL004W	999
YBL090W	YIL093C	988
YBL090W	YJR101W	988
YBL090W	YJR113C	988
YBL090W	YKL155C	988
YBL090W	YMR158W	974
YBL090W	YMR188C	988
YBL090W	YNL137C	988
YBL090W	YNL306W	988
YBL090W	YNR037C	987
YBL090W	YOR158W	988
YBL090W	YPL013C	996
YBL090W	YPL118W	995
YBL091C	YLR244C	982
YBL092W	YBL027W	999
YBL092W	YBL072C	925
YBL092W	YBL087C	998
YBL092W	YBR031W	999
YBL092W	YBR048W	976
YBL092W	YBR084C-A	986
YBL092W	YBR118W	911
YBL092W	YBR181C	998
YBL092W	YBR189W	957
YBL092W	YBR191W	999
YBL092W	YCR031C	944
YBL092W	YDL061C	970
YBL092W	YDL075W	999
YBL092W	YDL081C	975
YBL092W	YDL082W	997
YBL092W	YDL083C	969
YBL092W	YDL130W	911
YBL092W	YDL136W	999
YBL092W	YDL191W	926
YBL092W	YDR012W	998
YBL092W	YDR025W	976
YBL092W	YDR064W	999

YBL092W	YDR382W	997
YBL092W	YDR385W	914
YBL092W	YDR418W	994
YBL092W	YDR447C	997
YBL092W	YDR450W	944
YBL092W	YDR471W	998
YBL092W	YDR500C	908
YBL092W	YEL054C	936
YBL092W	YER056C-A	920
YBL092W	YER074W	995
YBL092W	YER102W	925
YBL092W	YER117W	979
YBL092W	YER131W	947
YBL092W	YFL034C-A	917
YBL092W	YFR031C-A	999
YBL092W	YFR032C-A	999
YBL092W	YGL014W	978
YBL092W	YGL030W	998
YBL092W	YGL031C	999
YBL092W	YGL076C	996
YBL092W	YGL103W	999
YBL092W	YGL123W	983
YBL092W	YGL135W	993
YBL092W	YGL147C	999
YBL092W	YGL189C	906
YBL092W	YGR027C	967
YBL092W	YGR034W	999
YBL092W	YGR085C	999
YBL092W	YGR118W	998
YBL092W	YGR148C	947
YBL092W	YGR214W	968
YBL092W	YHL001W	998
YBL092W	YHL015W	976
YBL092W	YHL033C	995
YBL092W	YHR010W	930
YBL092W	YHR021C	926
YBL092W	YHR029C	972
YBL092W	YHR141C	999
YBL092W	YHR203C	984
YBL092W	YIL018W	971
YBL092W	YIL052C	933
YBL092W	YIL069C	907
YBL092W	YIL133C	995
YBL092W	YIL148W	915

YBL092W	YJL136C	953
YBL092W	YJL177W	999
YBL092W	YJL189W	919
YBL092W	YJL190C	986
YBL092W	YJL191W	943
YBL092W	YJR094W-A	999
YBL092W	YJR123W	999
YBL092W	YJR145C	984
YBL092W	YKL006W	974
YBL092W	YKL156W	935
YBL092W	YKL180W	999
YBL092W	YKR057W	944
YBL092W	YKR094C	915
YBL092W	YLL045C	971
YBL092W	YLR029C	999
YBL092W	YLR048W	952
YBL092W	YLR061W	934
YBL092W	YLR075W	996
YBL092W	YLR167W	905
YBL092W	YLR185W	990
YBL092W	YLR264W	938
YBL092W	YLR287C-A	997
YBL092W	YLR325C	915
YBL092W	YLR333C	943
YBL092W	YLR340W	982
YBL092W	YLR344W	998
YBL092W	YLR367W	984
YBL092W	YLR388W	982
YBL092W	YLR406C	937
YBL092W	YLR441C	996
YBL092W	YLR448W	997
YBL092W	YML024W	949
YBL092W	YML026C	944
YBL092W	YML063W	998
YBL092W	YML073C	996
YBL092W	YMR121C	909
YBL092W	YMR142C	942
YBL092W	YMR194W	997
YBL092W	YMR230W	948
YBL092W	YMR242C	955
YBL092W	YMR260C	907
YBL092W	YNL067W	990
YBL092W	YNL069C	997
YBL092W	YNL096C	998

YBL092W	YNL162W	941
YBL092W	YNL178W	995
YBL092W	YNL301C	999
YBL092W	YNL302C	946
YBL092W	YNR037C	944
YBL092W	YOL039W	944
YBL092W	YOL040C	999
YBL092W	YOL120C	926
YBL092W	YOL121C	958
YBL092W	YOL127W	999
YBL092W	YOR063W	999
YBL092W	YOR096W	996
YBL092W	YOR133W	914
YBL092W	YOR167C	946
YBL092W	YOR182C	905
YBL092W	YOR234C	948
YBL092W	YOR293W	956
YBL092W	YOR312C	940
YBL092W	YOR369C	938
YBL092W	YPL079W	965
YBL092W	YPL081W	929
YBL092W	YPL090C	912
YBL092W	YPL131W	998
YBL092W	YPL143W	948
YBL092W	YPL198W	955
YBL092W	YPL220W	937
YBL092W	YPL249C-A	938
YBL092W	YPR043W	939
YBL092W	YPR080W	911
YBL092W	YPR102C	999
YBL092W	YPR132W	935
YBL093C	YBR193C	999
YBL093C	YBR253W	999
YBL093C	YCR081W	999
YBL093C	YDL005C	999
YBL093C	YDL140C	984
YBL093C	YDR308C	999
YBL093C	YDR443C	999
YBL093C	YDR477W	977
YBL093C	YER022W	999
YBL093C	YGL025C	999
YBL093C	YGL035C	919
YBL093C	YGL127C	999
YBL093C	YGL151W	999

YBL093C	YGR104C	999
YBL093C	YHR041C	999
YBL093C	YHR058C	999
YBL093C	YJR047C	929
YBL093C	YLR071C	999
YBL093C	YML007W	974
YBL093C	YMR042W	962
YBL093C	YMR112C	999
YBL093C	YNL025C	998
YBL093C	YNL236W	999
YBL093C	YNR010W	999
YBL093C	YOL051W	999
YBL093C	YOL135C	999
YBL093C	YOR014W	926
YBL093C	YOR140W	958
YBL093C	YOR174W	999
YBL093C	YPL042C	999
YBL093C	YPL129W	965
YBL093C	YPR070W	999
YBL093C	YPR168W	999
YBL097W	YDR325W	999
YBL097W	YFR031C	999
YBL097W	YLR086W	999
YBL097W	YLR272C	999
YBL098W	YLR231C	989
YBL099W	Q0080	999
YBL099W	Q0085	999
YBL099W	Q0130	999
YBL099W	YBR011C	906
YBL099W	YBR039W	999
YBL099W	YDL004W	999
YBL099W	YDL181W	999
YBL099W	YDR298C	999
YBL099W	YDR377W	999
YBL099W	YJL180C	938
YBL099W	YJR121W	999
YBL099W	YKL016C	999
YBL099W	YLR295C	999
YBL099W	YML081C-A	999
YBL099W	YMR267W	902
YBL099W	YNL315C	963
YBL099W	YOR065W	931
YBL099W	YPL078C	999
YBL099W	YPL271W	999

YBL099W	YPR020W	999
YBL099W	YPR024W	983
YBL100W-A	YCL020W	998
YBL100W-B	YCL019W	966
YBL101C	YER125W	988
YBL101C	YGR229C	920
YBL101C	YOL122C	922
YBL101C	YOR124C	930
YBL102W	YDR495C	932
YBL102W	YGL137W	903
YBL102W	YLR026C	995
YBL102W	YMR292W	996
YBL103C	YDR099W	953
YBL103C	YER177W	954
YBL103C	YFR021W	989
YBL103C	YNL006W	902
YBL103C	YOL067C	999
YBL103C	YOL108C	971
YBL103C	YPL100W	956
YBL105C	YBL039C	987
YBL105C	YBR109C	909
YBL105C	YCR017C	917
YBL105C	YDR356W	969
YBL105C	YEL002C	909
YBL105C	YEL034W	979
YBL105C	YER111C	999
YBL105C	YER167W	980
YBL105C	YGL022W	928
YBL105C	YGL178W	978
YBL105C	YGR032W	912
YBL105C	YGR229C	995
YBL105C	YGR254W	997
YBL105C	YHR030C	981
YBL105C	YHR206W	908
YBL105C	YIL105C	980
YBL105C	YIL126W	982
YBL105C	YJL002C	910
YBL105C	YJL095W	999
YBL105C	YKL203C	991
YBL105C	YLL021W	922
YBL105C	YLR305C	955
YBL105C	YLR342W	986
YBL105C	YLR371W	947
YBL105C	YML109W	992

YBL105C	YMR149W	910
YBL105C	YNL047C	968
YBL105C	YNL271C	932
YBL105C	YOL105C	934
YBL105C	YOR008C	999
YBL105C	YOR231W	997
YBL105C	YPR106W	928
YBL105C	YPR159W	913
YBL105C	YPR165W	999
YBL106C	YBR102C	981
YBL106C	YGR009C	919
YBL106C	YHR023W	931
YBL106C	YIL118W	934
YBL106C	YOR197W	920
YBL106C	YPR032W	972
YBL107W-A	YCR018C-A	950
YBL107W-A	YGR122C-A	966
YBL113C	YIR019C	900
YBL113C	YJL225C	909
YBR001C	YDR001C	921
YBR001C	YDR074W	939
YBR001C	YDR099W	963
YBR001C	YPR026W	986
YBR002C	YBR003W	910
YBR002C	YJL167W	976
YBR002C	YMR013C	994
YBR002C	YMR101C	997
YBR002C	YPR183W	936
YBR003W	YBR002C	910
YBR003W	YDR204W	901
YBR003W	YGL119W	926
YBR003W	YGR255C	916
YBR003W	YML110C	971
YBR003W	YNR041C	995
YBR003W	YOL096C	926
YBR004C	YNL158W	942
YBR005W	YDL212W	919
YBR005W	YER125W	986
YBR006W	YDL170W	957
YBR006W	YDL210W	932
YBR006W	YGR019W	997
YBR006W	YIR023W	920
YBR006W	YMR250W	938
YBR009C	YBL002W	999

YBR009C	YBL003C	998
YBR009C	YBL008W	902
YBR009C	YBR010W	999
YBR009C	YBR093C	941
YBR009C	YBR245C	995
YBR009C	YCR084C	930
YBR009C	YDL042C	999
YBR009C	YDL139C	901
YBR009C	YDR190C	982
YBR009C	YDR224C	999
YBR009C	YDR225W	999
YBR009C	YDR227W	998
YBR009C	YDR303C	992
YBR009C	YEL056W	999
YBR009C	YER110C	958
YBR009C	YFL024C	979
YBR009C	YGL207W	988
YBR009C	YGR252W	999
YBR009C	YHR187W	904
YBR009C	YJL081C	920
YBR009C	YJL115W	999
YBR009C	YKL049C	999
YBR009C	YKR001C	970
YBR009C	YKR101W	940
YBR009C	YLL022C	999
YBR009C	YLR176C	911
YBR009C	YLR357W	975
YBR009C	YLR399C	999
YBR009C	YLR442C	999
YBR009C	YLR449W	936
YBR009C	YML010W	906
YBR009C	YML069W	997
YBR009C	YMR072W	989
YBR009C	YMR127C	990
YBR009C	YMR308C	991
YBR009C	YNL030W	999
YBR009C	YNL031C	999
YBR009C	YNL206C	928
YBR009C	YOL012C	999
YBR009C	YOL054W	997
YBR009C	YOR244W	999
YBR009C	YOR290C	997
YBR009C	YOR304W	982
YBR009C	YPL001W	999

YBR009C	YPL082C	975
YBR009C	YPL086C	920
YBR009C	YPL127C	984
YBR009C	YPL153C	912
YBR009C	YPR018W	987
YBR009C	YPR052C	956
YBR010W	YBL002W	965
YBR010W	YBL003C	986
YBR010W	YBR009C	999
YBR010W	YBR245C	935
YBR010W	YCR084C	976
YBR010W	YDL042C	976
YBR010W	YDR224C	966
YBR010W	YDR225W	999
YBR010W	YDR227W	976
YBR010W	YDR440W	999
YBR010W	YDR477W	976
YBR010W	YEL056W	984
YBR010W	YER110C	970
YBR010W	YGL058W	982
YBR010W	YGL207W	924
YBR010W	YGR252W	999
YBR010W	YHR119W	971
YBR010W	YJL115W	999
YBR010W	YJL168C	982
YBR010W	YJR119C	976
YBR010W	YKL049C	968
YBR010W	YKR048C	938
YBR010W	YLL002W	998
YBR010W	YLL022C	997
YBR010W	YLR141W	968
YBR010W	YLR399C	991
YBR010W	YLR442C	999
YBR010W	YML069W	917
YBR010W	YMR308C	984
YBR010W	YNL030W	999
YBR010W	YNL031C	998
YBR010W	YNL206C	953
YBR010W	YNL330C	903
YBR010W	YOL012C	995
YBR010W	YOR038C	909
YBR010W	YPL209C	976
YBR010W	YPR018W	985
YBR010W	YPR052C	980

YBR011C	YBL099W	906
YBR011C	YBR039W	910
YBR011C	YDL004W	911
YBR011C	YDL203C	999
YBR011C	YEL027W	961
YBR011C	YGR020C	909
YBR011C	YHR026W	970
YBR011C	YHR039C-A	900
YBR011C	YJR121W	906
YBR011C	YLR371W	999
YBR011C	YLR425W	999
YBR011C	YMR267W	975
YBR011C	YOR332W	900
YBR011C	YPL066W	999
YBR011C	YPL234C	961
YBR012W-A	YJR028W	998
YBR012W-B	YPL257W-B	998
YBR017C	YDR062W	962
YBR017C	YDR192C	933
YBR017C	YDR225W	966
YBR017C	YEL022W	966
YBR017C	YER110C	997
YBR017C	YGL092W	965
YBR017C	YGL122C	999
YBR017C	YGR005C	909
YBR017C	YGR119C	965
YBR017C	YJR077C	988
YBR017C	YKL068W	982
YBR017C	YLR293C	995
YBR017C	YMR047C	928
YBR017C	YOL123W	999
YBR017C	YOR160W	950
YBR017C	YPR093C	977
YBR018C	YBR019C	999
YBR018C	YBR020W	997
YBR018C	YDR009W	998
YBR018C	YKL035W	960
YBR018C	YLR081W	995
YBR018C	YML051W	972
YBR018C	YMR105C	922
YBR018C	YPL248C	953
YBR019C	YBR018C	999
YBR019C	YBR020W	998
YBR019C	YDR009W	988

YBR019C	YDR432W	996
YBR019C	YFR053C	914
YBR019C	YGL253W	909
YBR019C	YGR116W	979
YBR019C	YHL012W	902
YBR019C	YKL035W	931
YBR019C	YLR081W	994
YBR019C	YML051W	972
YBR019C	YPL248C	934
YBR020W	YBR018C	997
YBR020W	YBR019C	998
YBR020W	YDR009W	999
YBR020W	YHR104W	909
YBR020W	YLR081W	993
YBR020W	YML051W	999
YBR020W	YPL248C	994
YBR021W	YEL063C	973
YBR021W	YJR066W	980
YBR021W	YML052W	975
YBR023C	YBL061C	999
YBR023C	YBR038W	996
YBR023C	YDR153C	973
YBR023C	YGR229C	977
YBR023C	YGR282C	901
YBR023C	YJL099W	998
YBR023C	YKR027W	903
YBR023C	YLR330W	968
YBR023C	YLR342W	968
YBR023C	YML115C	902
YBR023C	YMR237W	903
YBR023C	YMR307W	991
YBR023C	YNL233W	982
YBR023C	YNL298W	971
YBR023C	YOL003C	918
YBR024W	Q0250	943
YBR024W	YLL009C	973
YBR025C	YDL185W	970
YBR025C	YGL195W	966
YBR025C	YJR121W	999
YBR025C	YKL081W	991
YBR027C	YLR353W	998
YBR029C	YPR113W	963
YBR031W	YAL035W	998
YBR031W	YBL027W	998

YBR031W	YBL072C	995
YBR031W	YBL087C	977
YBR031W	YBL092W	999
YBR031W	YBR048W	998
YBR031W	YBR084C-A	978
YBR031W	YBR118W	920
YBR031W	YBR142W	934
YBR031W	YBR181C	995
YBR031W	YBR189W	999
YBR031W	YBR191W	998
YBR031W	YCL059C	943
YBR031W	YCR031C	999
YBR031W	YDL061C	969
YBR031W	YDL075W	997
YBR031W	YDL081C	956
YBR031W	YDL082W	997
YBR031W	YDL083C	967
YBR031W	YDL130W	935
YBR031W	YDL136W	996
YBR031W	YDL191W	931
YBR031W	YDR012W	999
YBR031W	YDR025W	998
YBR031W	YDR064W	984
YBR031W	YDR101C	940
YBR031W	YDR382W	999
YBR031W	YDR385W	929
YBR031W	YDR418W	999
YBR031W	YDR447C	996
YBR031W	YDR450W	925
YBR031W	YDR471W	997
YBR031W	YDR496C	933
YBR031W	YDR500C	932
YBR031W	YEL054C	938
YBR031W	YER056C-A	935
YBR031W	YER074W	996
YBR031W	YER102W	995
YBR031W	YER117W	977
YBR031W	YER131W	940
YBR031W	YFL034C-A	925
YBR031W	YFR031C-A	999
YBR031W	YFR032C-A	935
YBR031W	YGL030W	999
YBR031W	YGL031C	997
YBR031W	YGL076C	969

YBR031W	YGL103W	998
YBR031W	YGL123W	994
YBR031W	YGL135W	996
YBR031W	YGL147C	999
YBR031W	YGL189C	921
YBR031W	YGR027C	956
YBR031W	YGR034W	998
YBR031W	YGR085C	999
YBR031W	YGR118W	960
YBR031W	YGR148C	960
YBR031W	YGR214W	983
YBR031W	YHL001W	998
YBR031W	YHL015W	958
YBR031W	YHL033C	999
YBR031W	YHR010W	948
YBR031W	YHR021C	927
YBR031W	YHR141C	996
YBR031W	YHR203C	999
YBR031W	YIL018W	999
YBR031W	YIL052C	950
YBR031W	YIL069C	996
YBR031W	YIL133C	997
YBR031W	YIL148W	921
YBR031W	YJL136C	953
YBR031W	YJL177W	999
YBR031W	YJL189W	927
YBR031W	YJL190C	998
YBR031W	YJL191W	977
YBR031W	YJR094W-A	952
YBR031W	YJR123W	999
YBR031W	YJR145C	998
YBR031W	YKL006W	979
YBR031W	YKL156W	942
YBR031W	YKL180W	999
YBR031W	YKR057W	938
YBR031W	YKR081C	938
YBR031W	YKR094C	911
YBR031W	YLL045C	999
YBR031W	YLR029C	998
YBR031W	YLR048W	976
YBR031W	YLR061W	964
YBR031W	YLR075W	998
YBR031W	YLR167W	914
YBR031W	YLR185W	999

YBR031W	YLR264W	955
YBR031W	YLR287C-A	995
YBR031W	YLR333C	926
YBR031W	YLR340W	992
YBR031W	YLR344W	982
YBR031W	YLR367W	975
YBR031W	YLR388W	976
YBR031W	YLR406C	931
YBR031W	YLR441C	997
YBR031W	YLR448W	999
YBR031W	YML024W	955
YBR031W	YML026C	925
YBR031W	YML063W	998
YBR031W	YML073C	997
YBR031W	YMR121C	949
YBR031W	YMR142C	940
YBR031W	YMR194W	997
YBR031W	YMR230W	946
YBR031W	YMR242C	997
YBR031W	YMR260C	927
YBR031W	YMR290C	938
YBR031W	YNL067W	987
YBR031W	YNL069C	997
YBR031W	YNL096C	997
YBR031W	YNL112W	974
YBR031W	YNL132W	945
YBR031W	YNL162W	939
YBR031W	YNL178W	999
YBR031W	YNL301C	997
YBR031W	YNL302C	930
YBR031W	YNR037C	935
YBR031W	YOL039W	954
YBR031W	YOL040C	986
YBR031W	YOL077C	943
YBR031W	YOL120C	999
YBR031W	YOL121C	930
YBR031W	YOL127W	999
YBR031W	YOR063W	999
YBR031W	YOR096W	998
YBR031W	YOR133W	937
YBR031W	YOR167C	934
YBR031W	YOR182C	912
YBR031W	YOR234C	955
YBR031W	YOR293W	951

YBR031W	YOR312C	997
YBR031W	YOR369C	947
YBR031W	YPL012W	941
YBR031W	YPL037C	924
YBR031W	YPL079W	954
YBR031W	YPL081W	940
YBR031W	YPL090C	995
YBR031W	YPL131W	996
YBR031W	YPL143W	958
YBR031W	YPL198W	997
YBR031W	YPL220W	997
YBR031W	YPL249C-A	997
YBR031W	YPR043W	939
YBR031W	YPR080W	920
YBR031W	YPR102C	988
YBR031W	YPR132W	941
YBR034C	YDL014W	919
YBR034C	YDR432W	999
YBR034C	YGL122C	976
YBR034C	YHL034C	981
YBR034C	YHR089C	920
YBR034C	YMR125W	997
YBR034C	YOL123W	999
YBR035C	YEL029C	947
YBR035C	YFL060C	908
YBR035C	YMR095C	908
YBR035C	YMR096W	911
YBR035C	YNL334C	908
YBR035C	YNR027W	936
YBR036C	YBR161W	989
YBR036C	YDL015C	980
YBR036C	YDR062W	982
YBR036C	YDR072C	941
YBR036C	YDR208W	983
YBR036C	YDR297W	995
YBR036C	YEL027W	947
YBR036C	YER120W	909
YBR036C	YIL105C	990
YBR036C	YKL004W	981
YBR036C	YKL203C	920
YBR036C	YLR305C	915
YBR036C	YMR272C	997
YBR036C	YMR296C	968
YBR036C	YPL057C	999

YBR037C	Q0250	995
YBR037C	YER141W	930
YBR037C	YLL009C	998
YBR037C	YPL132W	932
YBR038W	YBR023C	996
YBR039W	Q0085	981
YBR039W	YBL099W	999
YBR039W	YBR011C	910
YBR039W	YDL004W	999
YBR039W	YDL130W-A	914
YBR039W	YDL181W	917
YBR039W	YDR298C	999
YBR039W	YJR121W	999
YBR039W	YKL016C	961
YBR039W	YLR295C	917
YBR039W	YML081C-A	934
YBR039W	YMR267W	903
YBR039W	YPL078C	999
YBR039W	YPL271W	997
YBR039W	YPR024W	984
YBR041W	YDL052C	938
YBR041W	YGL205W	903
YBR041W	YGR229C	923
YBR041W	YHR072W	985
YBR041W	YIL124W	951
YBR041W	YKL094W	917
YBR041W	YKL140W	960
YBR041W	YLL012W	929
YBR041W	YMR246W	974
YBR041W	YOR317W	974
YBR044C	YKL141W	925
YBR045C	YER133W	999
YBR045C	YOR178C	906
YBR047W	YPL240C	919
YBR048W	YBL004W	931
YBR048W	YBL027W	998
YBR048W	YBL072C	996
YBR048W	YBL087C	975
YBR048W	YBL092W	976
YBR048W	YBR031W	998
YBR048W	YBR084C-A	982
YBR048W	YBR118W	912
YBR048W	YBR181C	961
YBR048W	YBR189W	997

YBR048W	YBR191W	941
YBR048W	YBR247C	975
YBR048W	YCL011C	925
YBR048W	YCL059C	996
YBR048W	YCR031C	999
YBR048W	YDL014W	938
YBR048W	YDL060W	981
YBR048W	YDL061C	996
YBR048W	YDL075W	924
YBR048W	YDL081C	904
YBR048W	YDL082W	914
YBR048W	YDL083C	997
YBR048W	YDL136W	955
YBR048W	YDL148C	976
YBR048W	YDL191W	928
YBR048W	YDR012W	998
YBR048W	YDR025W	995
YBR048W	YDR064W	999
YBR048W	YDR299W	983
YBR048W	YDR365C	977
YBR048W	YDR382W	924
YBR048W	YDR385W	989
YBR048W	YDR418W	997
YBR048W	YDR429C	916
YBR048W	YDR447C	996
YBR048W	YDR450W	999
YBR048W	YDR471W	996
YBR048W	YDR500C	965
YBR048W	YEL054C	926
YBR048W	YER025W	998
YBR048W	YER056C-A	921
YBR048W	YER074W	996
YBR048W	YER102W	998
YBR048W	YER117W	987
YBR048W	YER131W	926
YBR048W	YER165W	915
YBR048W	YFR031C-A	998
YBR048W	YFR032C-A	924
YBR048W	YGL030W	999
YBR048W	YGL031C	909
YBR048W	YGL076C	997
YBR048W	YGL103W	956
YBR048W	YGL123W	998
YBR048W	YGL135W	996

YBR048W	YGL147C	971
YBR048W	YGL189C	914
YBR048W	YGR027C	924
YBR048W	YGR034W	998
YBR048W	YGR085C	998
YBR048W	YGR090W	977
YBR048W	YGR118W	997
YBR048W	YGR148C	919
YBR048W	YGR214W	993
YBR048W	YHL001W	997
YBR048W	YHL015W	992
YBR048W	YHL033C	996
YBR048W	YHR010W	914
YBR048W	YHR021C	918
YBR048W	YHR141C	910
YBR048W	YHR203C	999
YBR048W	YIL018W	998
YBR048W	YIL052C	918
YBR048W	YIL069C	995
YBR048W	YIL133C	995
YBR048W	YIL148W	947
YBR048W	YJL136C	914
YBR048W	YJL177W	999
YBR048W	YJL189W	956
YBR048W	YJL190C	999
YBR048W	YJL191W	944
YBR048W	YJR094W-A	929
YBR048W	YJR123W	999
YBR048W	YJR145C	998
YBR048W	YKL006W	937
YBR048W	YKL156W	930
YBR048W	YKL170W	907
YBR048W	YKL180W	999
YBR048W	YKR057W	914
YBR048W	YKR059W	939
YBR048W	YLL045C	993
YBR048W	YLR029C	997
YBR048W	YLR048W	955
YBR048W	YLR061W	935
YBR048W	YLR075W	996
YBR048W	YLR167W	935
YBR048W	YLR185W	967
YBR048W	YLR264W	919
YBR048W	YLR287C-A	996

YBR048W	YLR325C	917
YBR048W	YLR333C	912
YBR048W	YLR340W	997
YBR048W	YLR344W	976
YBR048W	YLR367W	999
YBR048W	YLR388W	975
YBR048W	YLR406C	900
YBR048W	YLR441C	996
YBR048W	YLR448W	997
YBR048W	YML024W	934
YBR048W	YML026C	921
YBR048W	YML063W	996
YBR048W	YML073C	996
YBR048W	YMR121C	914
YBR048W	YMR142C	912
YBR048W	YMR194W	997
YBR048W	YMR229C	925
YBR048W	YMR230W	918
YBR048W	YMR242C	997
YBR048W	YMR260C	912
YBR048W	YMR290C	938
YBR048W	YMR309C	913
YBR048W	YNL067W	976
YBR048W	YNL069C	995
YBR048W	YNL081C	904
YBR048W	YNL096C	997
YBR048W	YNL132W	969
YBR048W	YNL162W	910
YBR048W	YNL178W	999
YBR048W	YNL301C	975
YBR048W	YNL302C	915
YBR048W	YNL308C	980
YBR048W	YNR037C	953
YBR048W	YNR054C	970
YBR048W	YOL039W	909
YBR048W	YOL040C	998
YBR048W	YOL120C	920
YBR048W	YOL121C	935
YBR048W	YOL127W	998
YBR048W	YOR063W	999
YBR048W	YOR096W	997
YBR048W	YOR133W	931
YBR048W	YOR204W	931
YBR048W	YOR234C	936

YBR048W	YOR293W	926
YBR048W	YOR312C	997
YBR048W	YOR369C	932
YBR048W	YPL012W	931
YBR048W	YPL079W	925
YBR048W	YPL081W	999
YBR048W	YPL090C	995
YBR048W	YPL131W	998
YBR048W	YPL143W	936
YBR048W	YPL198W	994
YBR048W	YPL217C	939
YBR048W	YPL220W	956
YBR048W	YPL249C-A	914
YBR048W	YPR041W	915
YBR048W	YPR043W	943
YBR048W	YPR080W	900
YBR048W	YPR102C	974
YBR048W	YPR132W	959
YBR049C	YDR303C	974
YBR049C	YGL234W	918
YBR049C	YKR001C	956
YBR049C	YLR176C	978
YBR049C	YLR357W	983
YBR049C	YOL006C	918
YBR049C	YPL082C	973
YBR050C	YDR028C	998
YBR050C	YDR099W	936
YBR050C	YER133W	999
YBR052C	YCR004C	999
YBR052C	YDR032C	999
YBR052C	YDR076W	904
YBR055C	YBL026W	999
YBR055C	YBL074C	995
YBR055C	YBR152W	998
YBR055C	YCR077C	976
YBR055C	YDL030W	993
YBR055C	YDL043C	985
YBR055C	YDL098C	976
YBR055C	YDL160C	972
YBR055C	YDR283C	984
YBR055C	YDR378C	993
YBR055C	YDR473C	999
YBR055C	YER029C	999
YBR055C	YER112W	993

YBR055C	YER146W	998
YBR055C	YER172C	999
YBR055C	YFL017W-A	997
YBR055C	YGR013W	990
YBR055C	YGR074W	999
YBR055C	YGR075C	984
YBR055C	YGR091W	999
YBR055C	YGR271W	901
YBR055C	YHR165C	999
YBR055C	YJL124C	974
YBR055C	YJL203W	976
YBR055C	YJR022W	962
YBR055C	YKL173W	999
YBR055C	YLL036C	994
YBR055C	YLR147C	999
YBR055C	YLR275W	999
YBR055C	YLR424W	976
YBR055C	YLR438C-A	981
YBR055C	YML046W	990
YBR055C	YML049C	997
YBR055C	YMR240C	997
YBR055C	YMR268C	980
YBR055C	YMR288W	998
YBR055C	YNL147W	998
YBR055C	YOR159C	999
YBR055C	YOR308C	998
YBR055C	YOR319W	920
YBR055C	YPL213W	978
YBR055C	YPR082C	999
YBR055C	YPR178W	999
YBR055C	YPR182W	999
YBR057C	YGL036W	992
YBR057C	YKR031C	996
YBR058C	YBL076C	920
YBR058C	YKL213C	903
YBR058C	YOL020W	903
YBR058C-A	YDR062W	998
YBR058C-A	YMR296C	941
YBR059C	YER144C	933
YBR060C	YAR007C	908
YBR060C	YBL023C	997
YBR060C	YBL035C	932
YBR060C	YBR160W	998
YBR060C	YBR202W	999

YBR060C	YDL017W	999
YBR060C	YDR052C	999
YBR060C	YDR227W	999
YBR060C	YDR310C	971
YBR060C	YEL032W	964
YBR060C	YEL056W	915
YBR060C	YFR028C	999
YBR060C	YGL086W	999
YBR060C	YGL201C	953
YBR060C	YHR118C	999
YBR060C	YIL150C	998
YBR060C	YJL194W	999
YBR060C	YJR046W	982
YBR060C	YKR101W	987
YBR060C	YLL004W	999
YBR060C	YLR103C	997
YBR060C	YLR274W	999
YBR060C	YML065W	999
YBR060C	YMR036C	999
YBR060C	YMR127C	987
YBR060C	YMR190C	915
YBR060C	YNL102W	946
YBR060C	YNL261W	999
YBR060C	YPL001W	999
YBR060C	YPR019W	999
YBR060C	YPR162C	999
YBR061C	YMR259C	991
YBR061C	YPL183C	976
YBR065C	YDL209C	988
YBR065C	YDR088C	997
YBR065C	YDR364C	984
YBR065C	YER172C	995
YBR065C	YHR165C	995
YBR065C	YKR086W	912
YBR065C	YLL036C	999
YBR065C	YLR117C	999
YBR065C	YMR213W	997
YBR065C	YPL151C	999
YBR065C	YPR101W	988
YBR065C	YPR182W	915
YBR066C	YDR477W	976
YBR069C	YDL043C	925
YBR069C	YDR007W	983
YBR069C	YDR171W	947

YBR069C	YGR218W	979
YBR069C	YHR020W	961
YBR069C	YJR077C	948
YBR069C	YNL064C	969
YBR069C	YOR048C	960
YBR069C	YPL233W	993
YBR070C	YBR110W	968
YBR070C	YBR243C	973
YBR070C	YGL047W	999
YBR072W	YBR127C	984
YBR072W	YNL064C	963
YBR072W	YPR184W	980
YBR073W	YDL059C	965
YBR073W	YDR004W	965
YBR073W	YDR076W	966
YBR073W	YDR369C	970
YBR073W	YER095W	999
YBR073W	YER179W	996
YBR073W	YGL163C	999
YBR073W	YML032C	993
YBR073W	YMR224C	994
YBR073W	YNL250W	968
YBR077C	YGL023C	960
YBR077C	YGR163W	998
YBR077C	YJR066W	970
YBR077C	YKL143W	957
YBR077C	YKR007W	999
YBR077C	YML121W	999
YBR078W	YJL062W	996
YBR078W	YMR307W	980
YBR079C	YAL035W	985
YBR079C	YBL072C	998
YBR079C	YBR181C	906
YBR079C	YBR189W	929
YBR079C	YCR031C	923
YBR079C	YDL061C	918
YBR079C	YDR025W	997
YBR079C	YDR064W	995
YBR079C	YDR091C	994
YBR079C	YDR429C	999
YBR079C	YER025W	999
YBR079C	YER036C	906
YBR079C	YER102W	997
YBR079C	YER131W	916

YBR079C	YER165W	941
YBR079C	YGL123W	923
YBR079C	YGR027C	915
YBR079C	YGR118W	916
YBR079C	YGR162W	939
YBR079C	YGR214W	994
YBR079C	YHL015W	915
YBR079C	YHR203C	913
YBR079C	YJL191W	921
YBR079C	YJR007W	999
YBR079C	YJR047C	962
YBR079C	YJR123W	925
YBR079C	YJR145C	915
YBR079C	YKL156W	916
YBR079C	YKR059W	910
YBR079C	YLR048W	945
YBR079C	YLR192C	999
YBR079C	YLR264W	917
YBR079C	YLR367W	911
YBR079C	YLR388W	919
YBR079C	YML024W	913
YBR079C	YML063W	921
YBR079C	YMR146C	999
YBR079C	YMR260C	911
YBR079C	YMR309C	999
YBR079C	YNL096C	914
YBR079C	YNL178W	914
YBR079C	YNL244C	999
YBR079C	YOL121C	943
YBR079C	YOL139C	918
YBR079C	YOR096W	994
YBR079C	YOR293W	914
YBR079C	YOR361C	999
YBR079C	YPL001W	955
YBR079C	YPL081W	997
YBR079C	YPL090C	906
YBR079C	YPL237W	999
YBR079C	YPR041W	999
YBR079C	YPR086W	998
YBR079C	YPR132W	909
YBR079C	YPR163C	915
YBR080C	YBL050W	999
YBR080C	YDR164C	959
YBR080C	YGL212W	950

YBR080C	YKL196C	930
YBR080C	YLR026C	944
YBR080C	YLR043C	976
YBR080C	YLR093C	951
YBR080C	YLR268W	981
YBR080C	YMR197C	910
YBR080C	YMR292W	923
YBR080C	YNL015W	976
YBR080C	YOR106W	988
YBR081C	YBR111W-A	998
YBR081C	YBR198C	999
YBR081C	YCL010C	999
YBR081C	YDR145W	999
YBR081C	YDR167W	999
YBR081C	YDR176W	999
YBR081C	YDR392W	999
YBR081C	YDR448W	999
YBR081C	YEL009C	976
YBR081C	YEL020W-A	975
YBR081C	YER148W	987
YBR081C	YER164W	991
YBR081C	YGL043W	903
YBR081C	YGL066W	999
YBR081C	YGL112C	999
YBR081C	YGR252W	999
YBR081C	YGR274C	988
YBR081C	YHR099W	999
YBR081C	YLR055C	999
YBR081C	YML007W	997
YBR081C	YML015C	988
YBR081C	YML114C	923
YBR081C	YMR223W	999
YBR081C	YMR227C	988
YBR081C	YMR236W	999
YBR081C	YOL148C	999
YBR081C	YPL011C	988
YBR081C	YPL047W	999
YBR081C	YPL129W	923
YBR081C	YPL254W	999
YBR082C	YBL084C	917
YBR082C	YBR160W	916
YBR082C	YDL008W	998
YBR082C	YDL190C	904
YBR082C	YDR059C	999

YBR082C	YDR177W	997
YBR082C	YDR244W	939
YBR082C	YEL037C	980
YBR082C	YER012W	983
YBR082C	YER068W	992
YBR082C	YER125W	909
YBR082C	YFR010W	906
YBR082C	YGL058W	978
YBR082C	YGL116W	933
YBR082C	YGL240W	979
YBR082C	YIL148W	911
YBR082C	YKL010C	958
YBR082C	YKL022C	928
YBR082C	YKL210W	957
YBR082C	YLL039C	995
YBR082C	YLR127C	926
YBR082C	YLR167W	911
YBR082C	YOR339C	906
YBR082C	YPR103W	999
YBR083W	YBL016W	999
YBR083W	YER109C	998
YBR083W	YFL009W	956
YBR083W	YGR040W	992
YBR083W	YHR084W	999
YBR083W	YIR019C	969
YBR083W	YLR362W	954
YBR083W	YMR164C	991
YBR083W	YMR199W	948
YBR083W	YNL098C	987
YBR083W	YOR212W	943
YBR083W	YPL049C	937
YBR084C-A	YAL035W	913
YBR084C-A	YBL027W	901
YBR084C-A	YBL072C	920
YBR084C-A	YBL087C	975
YBR084C-A	YBL092W	986
YBR084C-A	YBR031W	978
YBR084C-A	YBR048W	982
YBR084C-A	YBR118W	930
YBR084C-A	YBR181C	970
YBR084C-A	YBR189W	939
YBR084C-A	YBR191W	983
YBR084C-A	YCR031C	983
YBR084C-A	YDL061C	974

YBR084C-A	YDL075W	995
YBR084C-A	YDL081C	928
YBR084C-A	YDL082W	922
YBR084C-A	YDL130W	930
YBR084C-A	YDL136W	999
YBR084C-A	YDL191W	916
YBR084C-A	YDL192W	964
YBR084C-A	YDR012W	981
YBR084C-A	YDR025W	966
YBR084C-A	YDR051C	940
YBR084C-A	YDR064W	985
YBR084C-A	YDR382W	976
YBR084C-A	YDR385W	914
YBR084C-A	YDR418W	958
YBR084C-A	YDR447C	920
YBR084C-A	YDR450W	921
YBR084C-A	YDR471W	938
YBR084C-A	YDR500C	999
YBR084C-A	YEL054C	910
YBR084C-A	YER056C-A	993
YBR084C-A	YER074W	902
YBR084C-A	YER102W	999
YBR084C-A	YER117W	999
YBR084C-A	YER131W	918
YBR084C-A	YFL034C-A	916
YBR084C-A	YFR031C-A	973
YBR084C-A	YFR032C-A	932
YBR084C-A	YGL030W	975
YBR084C-A	YGL031C	912
YBR084C-A	YGL076C	926
YBR084C-A	YGL103W	988
YBR084C-A	YGL123W	994
YBR084C-A	YGL135W	914
YBR084C-A	YGL147C	994
YBR084C-A	YGL189C	907
YBR084C-A	YGR027C	937
YBR084C-A	YGR034W	990
YBR084C-A	YGR085C	985
YBR084C-A	YGR118W	974
YBR084C-A	YGR148C	942
YBR084C-A	YGR214W	945
YBR084C-A	YHL001W	948
YBR084C-A	YHL015W	955
YBR084C-A	YHL033C	978

YBR084C-A	YHR010W	926
YBR084C-A	YHR021C	921
YBR084C-A	YHR141C	924
YBR084C-A	YHR203C	999
YBR084C-A	YIL018W	973
YBR084C-A	YIL052C	995
YBR084C-A	YIL069C	902
YBR084C-A	YIL132C	938
YBR084C-A	YIL133C	935
YBR084C-A	YIL148W	999
YBR084C-A	YJL136C	940
YBR084C-A	YJL177W	988
YBR084C-A	YJL189W	947
YBR084C-A	YJL190C	969
YBR084C-A	YJL191W	955
YBR084C-A	YJR094W-A	993
YBR084C-A	YJR123W	976
YBR084C-A	YJR145C	971
YBR084C-A	YKL006W	973
YBR084C-A	YKL156W	962
YBR084C-A	YKL180W	993
YBR084C-A	YKR057W	922
YBR084C-A	YKR059W	999
YBR084C-A	YKR094C	906
YBR084C-A	YLL045C	924
YBR084C-A	YLR029C	998
YBR084C-A	YLR048W	980
YBR084C-A	YLR061W	998
YBR084C-A	YLR075W	998
YBR084C-A	YLR167W	999
YBR084C-A	YLR185W	999
YBR084C-A	YLR264W	957
YBR084C-A	YLR325C	901
YBR084C-A	YLR333C	912
YBR084C-A	YLR340W	987
YBR084C-A	YLR344W	999
YBR084C-A	YLR367W	975
YBR084C-A	YLR388W	990
YBR084C-A	YLR406C	919
YBR084C-A	YLR441C	925
YBR084C-A	YLR448W	977
YBR084C-A	YML024W	994
YBR084C-A	YML026C	921
YBR084C-A	YML063W	954

YBR084C-A	YML073C	924
YBR084C-A	YMR121C	933
YBR084C-A	YMR142C	921
YBR084C-A	YMR194W	983
YBR084C-A	YMR230W	918
YBR084C-A	YMR242C	929
YBR084C-A	YMR260C	905
YBR084C-A	YNL067W	986
YBR084C-A	YNL069C	997
YBR084C-A	YNL096C	913
YBR084C-A	YNL162W	924
YBR084C-A	YNL178W	998
YBR084C-A	YNL301C	999
YBR084C-A	YNL302C	939
YBR084C-A	YNR037C	931
YBR084C-A	YOL039W	912
YBR084C-A	YOL040C	979
YBR084C-A	YOL120C	933
YBR084C-A	YOL121C	939
YBR084C-A	YOL127W	994
YBR084C-A	YOR063W	991
YBR084C-A	YOR096W	954
YBR084C-A	YOR133W	970
YBR084C-A	YOR167C	909
YBR084C-A	YOR234C	969
YBR084C-A	YOR293W	933
YBR084C-A	YOR312C	946
YBR084C-A	YOR369C	915
YBR084C-A	YPL037C	940
YBR084C-A	YPL079W	939
YBR084C-A	YPL081W	912
YBR084C-A	YPL090C	902
YBR084C-A	YPL131W	997
YBR084C-A	YPL143W	977
YBR084C-A	YPL198W	930
YBR084C-A	YPL220W	999
YBR084C-A	YPL249C-A	950
YBR084C-A	YPR043W	997
YBR084C-A	YPR080W	908
YBR084C-A	YPR102C	982
YBR084C-A	YPR132W	999
YBR084W	YAL035W	956
YBR084W	YBL013W	956
YBR084W	YBR263W	994

YBR084W	YDR019C	955
YBR084W	YDR408C	928
YBR084W	YDR471W	972
YBR084W	YER183C	910
YBR084W	YFR031C-A	993
YBR084W	YGL099W	945
YBR084W	YGL125W	933
YBR084W	YGR204W	964
YBR084W	YHR010W	972
YBR084W	YKR080W	924
YBR084W	YLR028C	909
YBR084W	YLR058C	994
YBR084W	YLR075W	976
YBR084W	YLR340W	974
YBR084W	YMR113W	913
YBR084W	YMR120C	908
YBR084W	YOR063W	977
YBR084W	YOR074C	939
YBR084W	YOR236W	909
YBR085W	YBL030C	993
YBR085W	YDR129C	919
YBR086C	YGR142W	982
YBR086C	YIL026C	921
YBR086C	YJL059W	908
YBR086C	YKL130C	989
YBR087W	YBL035C	997
YBR087W	YBL088C	918
YBR087W	YBR088C	999
YBR087W	YER173W	999
YBR087W	YIR008C	918
YBR087W	YJL115W	995
YBR087W	YJR068W	999
YBR087W	YMR078C	999
YBR087W	YMR181C	903
YBR087W	YNL102W	914
YBR087W	YNL290W	999
YBR087W	YOL094C	999
YBR087W	YOR144C	999
YBR087W	YOR217W	999
YBR087W	YPL153C	996
YBR088C	YAR007C	957
YBR088C	YBR087W	999
YBR088C	YCR066W	999
YBR088C	YCR092C	997

YBR088C	YDL064W	996
YBR088C	YDL102W	999
YBR088C	YDL164C	999
YBR088C	YDR092W	983
YBR088C	YDR097C	999
YBR088C	YDR409W	921
YBR088C	YDR419W	999
YBR088C	YDR510W	996
YBR088C	YFR027W	955
YBR088C	YGL058W	999
YBR088C	YGL087C	999
YBR088C	YHR164C	927
YBR088C	YIL150C	929
YBR088C	YIR008C	949
YBR088C	YJL092W	999
YBR088C	YJL115W	923
YBR088C	YJR006W	999
YBR088C	YJR043C	999
YBR088C	YJR068W	999
YBR088C	YKL045W	904
YBR088C	YKL113C	999
YBR088C	YLL039C	999
YBR088C	YLR032W	999
YBR088C	YML021C	954
YBR088C	YML032C	996
YBR088C	YML102W	996
YBR088C	YMR078C	974
YBR088C	YMR167W	987
YBR088C	YNL102W	990
YBR088C	YNL218W	972
YBR088C	YNL250W	908
YBR088C	YNL262W	963
YBR088C	YNL290W	999
YBR088C	YNL312W	903
YBR088C	YNR052C	903
YBR088C	YOL090W	999
YBR088C	YOL094C	999
YBR088C	YOR144C	959
YBR088C	YOR217W	999
YBR088C	YPL167C	902
YBR088C	YPR018W	976
YBR089C-A	YBR245C	975
YBR089C-A	YCR084C	972
YBR089C-A	YFR013W	987

YBR089C-A	YGL133W	970
YBR089C-A	YGL207W	993
YBR089C-A	YGR047C	907
YBR089C-A	YJL026W	911
YBR089C-A	YLL021W	915
YBR089C-A	YLR033W	978
YBR089C-A	YLR321C	916
YBR089C-A	YOR290C	938
YBR089C-A	YPL082C	969
YBR089C-A	YPR052C	999
YBR091C	YDL217C	999
YBR091C	YEL020W-A	997
YBR091C	YGR181W	969
YBR091C	YHR005C-A	999
YBR091C	YJL054W	999
YBR091C	YJR135W-A	972
YBR091C	YNR017W	969
YBR091C	YOR297C	992
YBR092C	YGR233C	953
YBR093C	YAR071W	967
YBR093C	YBR009C	941
YBR093C	YDL106C	999
YBR093C	YFR034C	990
YBR093C	YGR233C	958
YBR093C	YOL001W	935
YBR093C	YPL031C	970
YBR094W	YEL015W	999
YBR094W	YJR043C	924
YBR094W	YMR190C	946
YBR094W	YOL149W	993
YBR094W	YOR144C	959
YBR095C	YAL013W	999
YBR095C	YCR033W	961
YBR095C	YDL076C	999
YBR095C	YDR207C	998
YBR095C	YGL194C	931
YBR095C	YIL084C	999
YBR095C	YKL185W	998
YBR095C	YKR029C	947
YBR095C	YMR075W	986
YBR095C	YMR263W	999
YBR095C	YNL097C	999
YBR095C	YNL330C	999
YBR095C	YOL004W	999

YBR095C	YOL012C	913
YBR095C	YPL139C	999
YBR095C	YPL181W	999
YBR095C	YPR023C	997
YBR097W	YBL056W	919
YBR097W	YBR128C	999
YBR097W	YHR005C	987
YBR097W	YLR240W	999
YBR097W	YLR360W	999
YBR097W	YPL120W	999
YBR098W	YDL154W	934
YBR098W	YDR363W	944
YBR098W	YDR386W	999
YBR098W	YHR014W	981
YBR098W	YJL092W	991
YBR098W	YKL113C	955
YBR098W	YKL114C	900
YBR098W	YLR234W	997
YBR098W	YLR373C	927
YBR098W	YML095C	933
YBR098W	YMR190C	999
YBR098W	YOR144C	965
YBR098W	YPL022W	923
YBR098W	YPL024W	998
YBR101C	YAL005C	999
YBR101C	YBR155W	925
YBR101C	YDL229W	975
YBR101C	YDR171W	922
YBR101C	YHR064C	903
YBR101C	YJL138C	925
YBR101C	YKR059W	925
YBR101C	YLL026W	913
YBR101C	YNL064C	926
YBR101C	YNL209W	974
YBR101C	YOR027W	929
YBR101C	YPL106C	998
YBR102C	YBL106C	981
YBR102C	YDR166C	999
YBR102C	YER008C	999
YBR102C	YGL233W	999
YBR102C	YHR165C	983
YBR102C	YIL061C	921
YBR102C	YIL068C	999
YBR102C	YJL085W	999

YBR102C	YLR078C	962
YBR102C	YLR166C	999
YBR102C	YML097C	945
YBR102C	YPR032W	978
YBR102C	YPR055W	999
YBR103W	YAL013W	943
YBR103W	YCR033W	999
YBR103W	YDR155C	999
YBR103W	YDR207C	914
YBR103W	YDR227W	983
YBR103W	YDR310C	926
YBR103W	YGL194C	999
YBR103W	YIL084C	923
YBR103W	YIL112W	999
YBR103W	YJL168C	961
YBR103W	YKR029C	999
YBR103W	YLR347C	998
YBR103W	YLR442C	922
YBR103W	YMR263W	960
YBR103W	YMR273C	994
YBR103W	YNL097C	930
YBR103W	YNL330C	927
YBR103W	YOL004W	937
YBR103W	YOL012C	968
YBR103W	YOL068C	999
YBR103W	YPL139C	948
YBR103W	YPL181W	974
YBR105C	YCL039W	981
YBR105C	YDL082W	984
YBR105C	YDR255C	981
YBR105C	YGL227W	999
YBR105C	YIL017C	999
YBR105C	YIL097W	999
YBR105C	YMR135C	999
YBR106W	YDR245W	990
YBR106W	YEL036C	990
YBR106W	YGL055W	984
YBR106W	YGL167C	973
YBR106W	YJL117W	962
YBR106W	YJL183W	991
YBR106W	YJR075W	990
YBR106W	YPL050C	990
YBR107C	YAL034W-A	976
YBR107C	YBR211C	999

YBR107C	YDR254W	999
YBR107C	YDR318W	999
YBR107C	YDR383C	985
YBR107C	YDR421W	918
YBR107C	YEL061C	939
YBR107C	YER016W	980
YBR107C	YGL071W	951
YBR107C	YGL086W	924
YBR107C	YGR179C	999
YBR107C	YGR188C	948
YBR107C	YJL030W	998
YBR107C	YJR135C	999
YBR107C	YLR381W	999
YBR107C	YML085C	911
YBR107C	YMR117C	983
YBR107C	YPL018W	999
YBR107C	YPL233W	906
YBR107C	YPR046W	999
YBR108W	YCR009C	999
YBR108W	YDR388W	999
YBR109C	YAL029C	999
YBR109C	YBL105C	909
YBR109C	YBR130C	998
YBR109C	YCR065W	991
YBR109C	YDR208W	940
YBR109C	YDR356W	999
YBR109C	YFL039C	994
YBR109C	YFR014C	999
YBR109C	YGL043W	976
YBR109C	YGL106W	999
YBR109C	YGL242C	986
YBR109C	YGR036C	938
YBR109C	YHR023W	973
YBR109C	YJR065C	903
YBR109C	YKL007W	970
YBR109C	YKL129C	994
YBR109C	YLL040C	974
YBR109C	YLR212C	917
YBR109C	YLR429W	983
YBR109C	YLR433C	999
YBR109C	YML057W	999
YBR109C	YMR109W	999
YBR109C	YMR186W	932
YBR109C	YMR250W	972

YBR109C	YNR035C	999
YBR109C	YOL016C	999
YBR109C	YOR326W	999
YBR109C	YPL124W	972
YBR109C	YPL242C	995
YBR109C	YPR171W	968
YBR110W	YBR070C	968
YBR110W	YBR243C	948
YBR110W	YDL055C	995
YBR110W	YGL047W	968
YBR110W	YGL065C	999
YBR110W	YMR199W	918
YBR110W	YNL048W	970
YBR110W	YPL256C	917
YBR110W	YPR183W	979
YBR111W-A	YAR002W	908
YBR111W-A	YBR081C	998
YBR111W-A	YBR198C	959
YBR111W-A	YCL010C	959
YBR111W-A	YDR145W	992
YBR111W-A	YDR159W	999
YBR111W-A	YDR167W	959
YBR111W-A	YDR176W	992
YBR111W-A	YDR392W	959
YBR111W-A	YDR448W	998
YBR111W-A	YGL066W	997
YBR111W-A	YGL086W	908
YBR111W-A	YGL112C	959
YBR111W-A	YGR252W	959
YBR111W-A	YHR099W	998
YBR111W-A	YJL030W	908
YBR111W-A	YLR055C	959
YBR111W-A	YMR223W	999
YBR111W-A	YMR236W	959
YBR111W-A	YOL072W	999
YBR111W-A	YOL148C	959
YBR111W-A	YOR098C	936
YBR111W-A	YOR257W	999
YBR111W-A	YPL047W	998
YBR111W-A	YPL169C	971
YBR111W-A	YPL254W	966
YBR112C	YCL067C	994
YBR112C	YCR084C	999
YBR112C	YDL194W	982

YBR112C	YDR477W	994
YBR112C	YGL035C	999
YBR112C	YGL071W	940
YBR112C	YGL115W	935
YBR112C	YGL194C	996
YBR112C	YHL025W	954
YBR112C	YHR117W	999
YBR112C	YJL168C	971
YBR112C	YKL038W	996
YBR112C	YLR113W	986
YBR112C	YLR176C	992
YBR112C	YMR011W	900
YBR112C	YNL167C	978
YBR112C	YNL330C	996
YBR112C	YOR027W	928
YBR112C	YOR140W	936
YBR112C	YOR290C	945
YBR112C	YPL181W	936
YBR112C	YPR065W	959
YBR112C	YPR068C	914
YBR114W	YCR028C-A	980
YBR114W	YDR097C	919
YBR114W	YEL037C	998
YBR114W	YER162C	997
YBR114W	YER171W	965
YBR114W	YGL163C	918
YBR114W	YGR003W	984
YBR114W	YGR258C	971
YBR114W	YHL024W	936
YBR114W	YIL128W	946
YBR114W	YIL143C	908
YBR114W	YJR035W	999
YBR114W	YJR052W	999
YBR114W	YKL112W	902
YBR114W	YML095C	971
YBR114W	YMR190C	979
YBR114W	YMR201C	996
YBR114W	YMR315W	932
YBR114W	YNL312W	919
YBR114W	YPL022W	971
YBR114W	YPL046C	998
YBR114W	YPL154C	921
YBR115C	YAL059W	933
YBR115C	YDR392W	976

YBR115C	YGL154C	999
YBR115C	YNR050C	930
YBR117C	YBR196C	937
YBR117C	YCR036W	926
YBR117C	YDR050C	905
YBR117C	YGR043C	959
YBR117C	YHL011C	900
YBR117C	YJL121C	975
YBR117C	YKL060C	933
YBR117C	YLR354C	959
YBR117C	YLR377C	924
YBR117C	YOR095C	934
YBR117C	YPR074C	999
YBR118W	YAL003W	999
YBR118W	YBL027W	908
YBR118W	YBL072C	909
YBR118W	YBL087C	917
YBR118W	YBL092W	911
YBR118W	YBR031W	920
YBR118W	YBR048W	912
YBR118W	YBR084C-A	930
YBR118W	YBR189W	901
YBR118W	YBR191W	916
YBR118W	YCR031C	907
YBR118W	YDL075W	905
YBR118W	YDL081C	914
YBR118W	YDL082W	910
YBR118W	YDL136W	909
YBR118W	YDR012W	920
YBR118W	YDR025W	900
YBR118W	YDR041W	924
YBR118W	YDR064W	929
YBR118W	YDR382W	923
YBR118W	YDR385W	946
YBR118W	YDR418W	907
YBR118W	YDR447C	910
YBR118W	YDR450W	907
YBR118W	YDR500C	916
YBR118W	YEL054C	907
YBR118W	YER074W	904
YBR118W	YER102W	939
YBR118W	YER117W	935
YBR118W	YER125W	939
YBR118W	YER131W	904

YBR118W	YFL034C-A	902
YBR118W	YFR031C-A	905
YBR118W	YFR032C-A	906
YBR118W	YGL030W	939
YBR118W	YGL076C	913
YBR118W	YGL123W	925
YBR118W	YGL135W	957
YBR118W	YGL147C	907
YBR118W	YGL189C	904
YBR118W	YGR027C	908
YBR118W	YGR034W	905
YBR118W	YGR085C	907
YBR118W	YGR118W	932
YBR118W	YGR214W	911
YBR118W	YHL001W	907
YBR118W	YHL015W	978
YBR118W	YHL033C	919
YBR118W	YHR021C	908
YBR118W	YHR141C	910
YBR118W	YHR203C	946
YBR118W	YIL018W	905
YBR118W	YIL069C	904
YBR118W	YIL133C	907
YBR118W	YIL148W	916
YBR118W	YJL136C	910
YBR118W	YJL177W	901
YBR118W	YJL189W	903
YBR118W	YJL191W	907
YBR118W	YJR094W-A	909
YBR118W	YJR123W	964
YBR118W	YJR145C	922
YBR118W	YKL001C	923
YBR118W	YKL006W	907
YBR118W	YKL081W	994
YBR118W	YKL156W	908
YBR118W	YKL170W	912
YBR118W	YKL180W	911
YBR118W	YKR057W	910
YBR118W	YKR094C	900
YBR118W	YLL045C	906
YBR118W	YLR029C	936
YBR118W	YLR048W	921
YBR118W	YLR061W	903
YBR118W	YLR075W	919

YBR118W	YLR167W	917
YBR118W	YLR185W	915
YBR118W	YLR249W	992
YBR118W	YLR264W	902
YBR118W	YLR333C	907
YBR118W	YLR340W	931
YBR118W	YLR344W	914
YBR118W	YLR406C	905
YBR118W	YLR441C	916
YBR118W	YLR448W	911
YBR118W	YML024W	920
YBR118W	YML026C	907
YBR118W	YML063W	916
YBR118W	YML073C	909
YBR118W	YMR121C	936
YBR118W	YMR142C	909
YBR118W	YMR194W	908
YBR118W	YMR230W	909
YBR118W	YMR242C	916
YBR118W	YNL067W	907
YBR118W	YNL069C	906
YBR118W	YNL081C	912
YBR118W	YNL096C	903
YBR118W	YNL162W	910
YBR118W	YNL178W	940
YBR118W	YNL301C	926
YBR118W	YNL302C	907
YBR118W	YNR037C	933
YBR118W	YOL039W	904
YBR118W	YOL040C	916
YBR118W	YOL120C	911
YBR118W	YOL121C	908
YBR118W	YOL127W	904
YBR118W	YOR063W	941
YBR118W	YOR096W	903
YBR118W	YOR133W	951
YBR118W	YOR167C	902
YBR118W	YOR234C	912
YBR118W	YOR293W	909
YBR118W	YOR312C	916
YBR118W	YOR369C	911
YBR118W	YPL079W	907
YBR118W	YPL081W	901
YBR118W	YPL131W	912

YBR118W	YPL143W	912
YBR118W	YPL198W	913
YBR118W	YPL220W	926
YBR118W	YPL249C-A	909
YBR118W	YPR043W	919
YBR118W	YPR080W	986
YBR118W	YPR102C	907
YBR118W	YPR132W	944
YBR119W	YAL043C	970
YBR119W	YDL087C	999
YBR119W	YDR235W	999
YBR119W	YDR240C	999
YBR119W	YER029C	998
YBR119W	YFL017W-A	994
YBR119W	YGR013W	999
YBR119W	YGR074W	995
YBR119W	YHR086W	999
YBR119W	YIL061C	999
YBR119W	YKL012W	999
YBR119W	YLR147C	996
YBR119W	YLR275W	995
YBR119W	YLR298C	999
YBR119W	YML046W	999
YBR119W	YMR125W	999
YBR119W	YOR159C	987
YBR119W	YPL178W	999
YBR119W	YPR057W	974
YBR119W	YPR182W	995
YBR121C	YPR081C	989
YBR122C	YBL038W	972
YBR122C	YCR046C	992
YBR122C	YCR071C	972
YBR122C	YDR116C	974
YBR122C	YDR237W	974
YBR122C	YDR296W	992
YBR122C	YDR322W	974
YBR122C	YDR405W	970
YBR122C	YDR462W	972
YBR122C	YGR220C	997
YBR122C	YHR147C	968
YBR122C	YJL063C	972
YBR122C	YKL167C	972
YBR122C	YKR006C	972
YBR122C	YKR085C	972

YBR122C	YLR439W	974
YBR122C	YML025C	991
YBR122C	YMR024W	972
YBR122C	YMR193W	972
YBR122C	YNL005C	974
YBR122C	YNL252C	974
YBR122C	YNL284C	928
YBR122C	YOR150W	973
YBR123C	YAL001C	999
YBR123C	YBL076C	940
YBR123C	YDR362C	999
YBR123C	YGR047C	999
YBR123C	YGR246C	984
YBR123C	YKL068W	913
YBR123C	YOR110W	999
YBR123C	YPL007C	999
YBR125C	YDR071C	999
YBR125C	YDR186C	999
YBR125C	YDR247W	944
YBR126C	YBL023C	938
YBR126C	YBR265W	929
YBR126C	YDR001C	935
YBR126C	YDR074W	999
YBR126C	YER155C	962
YBR126C	YGL035C	976
YBR126C	YGL253W	993
YBR126C	YIL177C	906
YBR126C	YIR032C	906
YBR126C	YKL035W	932
YBR126C	YLL026W	919
YBR126C	YLR258W	931
YBR126C	YLR411W	906
YBR126C	YML100W	999
YBR126C	YMR105C	911
YBR126C	YMR251W	906
YBR126C	YMR261C	999
YBR126C	YPR035W	935
YBR127C	YBR072W	984
YBR127C	YDL185W	999
YBR127C	YDR202C	975
YBR127C	YDR328C	929
YBR127C	YEL027W	980
YBR127C	YEL051W	999
YBR127C	YGR020C	999

YBR127C	YGR092W	911
YBR127C	YHR026W	904
YBR127C	YHR039C-A	999
YBR127C	YJR033C	999
YBR127C	YKL080W	999
YBR127C	YLR447C	987
YBR127C	YMR054W	999
YBR127C	YOR270C	999
YBR127C	YOR332W	996
YBR127C	YPL234C	977
YBR127C	YPR036W	999
YBR128C	YBR097W	999
YBR128C	YER040W	907
YBR128C	YLR240W	999
YBR128C	YLR360W	983
YBR128C	YPL120W	999
YBR130C	YAL029C	999
YBR130C	YBR109C	998
YBR130C	YFL039C	942
YBR130C	YGL106W	999
YBR130C	YKL130C	999
YBR130C	YKL185W	957
YBR130C	YOR035C	907
YBR131W	YGL124C	999
YBR131W	YGR198W	918
YBR131W	YML001W	997
YBR133C	YHL007C	943
YBR133C	YJL187C	999
YBR133C	YKL101W	995
YBR135W	YBL084C	930
YBR135W	YBR160W	999
YBR135W	YBR295W	901
YBR135W	YDL155W	999
YBR135W	YFR005C	901
YBR135W	YGR108W	995
YBR135W	YHR166C	929
YBR135W	YJL194W	998
YBR135W	YKL022C	929
YBR135W	YKR091W	996
YBR135W	YLR079W	999
YBR135W	YLR210W	907
YBR135W	YMR199W	999
YBR135W	YPL014W	901
YBR135W	YPL256C	999

YBR135W	YPR119W	999
YBR135W	YPR120C	997
YBR136W	YAR007C	999
YBR136W	YBL051C	978
YBR136W	YBL088C	999
YBR136W	YBR274W	997
YBR136W	YDL101C	972
YBR136W	YDL220C	999
YBR136W	YDR052C	915
YBR136W	YDR076W	970
YBR136W	YDR097C	968
YBR136W	YDR113C	954
YBR136W	YDR217C	999
YBR136W	YDR369C	920
YBR136W	YDR499W	999
YBR136W	YER089C	935
YBR136W	YER095W	971
YBR136W	YER173W	946
YBR136W	YGL150C	962
YBR136W	YGR180C	914
YBR136W	YHR031C	986
YBR136W	YHR154W	952
YBR136W	YHR164C	927
YBR136W	YJL090C	919
YBR136W	YJL092W	975
YBR136W	YJL115W	995
YBR136W	YJL173C	996
YBR136W	YLR288C	986
YBR136W	YML032C	994
YBR136W	YML058W	999
YBR136W	YMR199W	976
YBR136W	YMR224C	963
YBR136W	YMR284W	988
YBR136W	YNL312W	999
YBR136W	YOR033C	930
YBR136W	YOR189W	905
YBR136W	YPL153C	999
YBR136W	YPL194W	997
YBR136W	YPL256C	967
YBR136W	YPR120C	984
YBR136W	YPR135W	908
YBR137W	YOR007C	999
YBR140C	YAL056W	963
YBR140C	YJL005W	987

YBR140C	YLR310C	992
YBR140C	YNL098C	999
YBR140C	YOL081W	978
YBR140C	YOR101W	998
YBR140C	YOR371C	963
YBR140C	YPL106C	970
YBR142W	YBR031W	934
YBR142W	YDL014W	923
YBR142W	YDL031W	985
YBR142W	YDR060W	998
YBR142W	YDR496C	999
YBR142W	YER102W	978
YBR142W	YGL076C	937
YBR142W	YGL111W	903
YBR142W	YGL135W	940
YBR142W	YGR085C	938
YBR142W	YHR052W	996
YBR142W	YHR066W	968
YBR142W	YJL050W	919
YBR142W	YJR145C	925
YBR142W	YKL014C	980
YBR142W	YLL008W	984
YBR142W	YLL045C	968
YBR142W	YLR029C	947
YBR142W	YLR276C	984
YBR142W	YLR398C	966
YBR142W	YML073C	941
YBR142W	YMR049C	998
YBR142W	YMR229C	984
YBR142W	YMR290C	999
YBR142W	YNL002C	999
YBR142W	YNL061W	998
YBR142W	YOL041C	998
YBR142W	YOR063W	940
YBR142W	YOR272W	999
YBR142W	YPL043W	999
YBR142W	YPL093W	918
YBR142W	YPL131W	981
YBR142W	YPL198W	964
YBR142W	YPL211W	980
YBR142W	YPL249C-A	982
YBR143C	YDR140W	926
YBR143C	YDR172W	999
YBR143C	YLR386W	921

YBR143C	YLR429W	904
YBR143C	YMR080C	995
YBR143C	YPL237W	972
YBR145W	YER073W	901
YBR145W	YGL256W	979
YBR145W	YMR169C	900
YBR145W	YMR170C	900
YBR145W	YOR374W	901
YBR145W	YPL061W	900
YBR146W	Q0140	962
YBR146W	YBL090W	988
YBR146W	YBR251W	999
YBR146W	YDR036C	999
YBR146W	YDR041W	999
YBR146W	YDR175C	988
YBR146W	YDR337W	996
YBR146W	YDR347W	988
YBR146W	YER050C	986
YBR146W	YGL123W	917
YBR146W	YGL129C	988
YBR146W	YGR084C	988
YBR146W	YGR165W	988
YBR146W	YGR215W	988
YBR146W	YHL004W	999
YBR146W	YIL093C	988
YBR146W	YJL063C	946
YBR146W	YJR101W	988
YBR146W	YJR113C	998
YBR146W	YKL003C	986
YBR146W	YKL155C	997
YBR146W	YMR158W	974
YBR146W	YMR188C	989
YBR146W	YNL081C	998
YBR146W	YNL137C	988
YBR146W	YNL306W	988
YBR146W	YNR036C	988
YBR146W	YNR037C	999
YBR146W	YOR150W	975
YBR146W	YOR158W	988
YBR146W	YOR204W	986
YBR146W	YPL013C	999
YBR146W	YPL118W	988
YBR152W	YBR055C	998
YBR152W	YDL030W	921

YBR152W	YDL043C	934
YBR152W	YDL098C	999
YBR152W	YDR473C	999
YBR152W	YER029C	998
YBR152W	YER172C	989
YBR152W	YGR075C	999
YBR152W	YGR091W	997
YBR152W	YIR009W	937
YBR152W	YJL203W	931
YBR152W	YKL173W	915
YBR152W	YML049C	945
YBR152W	YMR240C	982
YBR152W	YMR288W	916
YBR152W	YOR308C	999
YBR152W	YPL213W	928
YBR152W	YPR178W	999
YBR153W	YBL033C	994
YBR153W	YOL066C	965
YBR154C	YBL026W	905
YBR154C	YBR279W	927
YBR154C	YDL115C	996
YBR154C	YDL140C	999
YBR154C	YDL150W	999
YBR154C	YDR045C	997
YBR154C	YDR156W	998
YBR154C	YDR404C	999
YBR154C	YEL026W	936
YBR154C	YER022W	981
YBR154C	YER029C	915
YBR154C	YER139C	911
YBR154C	YER148W	935
YBR154C	YFL017W-A	947
YBR154C	YFL023W	990
YBR154C	YGL043W	999
YBR154C	YGL070C	999
YBR154C	YGR005C	999
YBR154C	YGR063C	987
YBR154C	YGR074W	923
YBR154C	YGR104C	983
YBR154C	YGR186W	999
YBR154C	YHR143W-A	999
YBR154C	YIL021W	999
YBR154C	YIL143C	915
YBR154C	YJL011C	998

YBR154C	YJL140W	999
YBR154C	YJL148W	999
YBR154C	YJR063W	999
YBR154C	YJR138W	929
YBR154C	YKL028W	919
YBR154C	YKL144C	999
YBR154C	YKR008W	913
YBR154C	YKR025W	997
YBR154C	YKR062W	948
YBR154C	YLR071C	980
YBR154C	YLR147C	912
YBR154C	YML010W	999
YBR154C	YMR277W	950
YBR154C	YMR288W	904
YBR154C	YNL113W	999
YBR154C	YNL151C	997
YBR154C	YNL248C	999
YBR154C	YNR003C	999
YBR154C	YOL005C	999
YBR154C	YOL135C	913
YBR154C	YOR116C	999
YBR154C	YOR151C	999
YBR154C	YOR207C	999
YBR154C	YOR210W	999
YBR154C	YOR224C	999
YBR154C	YOR340C	999
YBR154C	YOR341W	999
YBR154C	YPL129W	999
YBR154C	YPR010C	999
YBR154C	YPR086W	980
YBR154C	YPR110C	999
YBR154C	YPR168W	938
YBR154C	YPR182W	961
YBR154C	YPR187W	999
YBR154C	YPR190C	999
YBR155W	YAL005C	992
YBR155W	YBR101C	925
YBR155W	YDL229W	940
YBR155W	YGR187C	990
YBR155W	YJR032W	999
YBR155W	YKL117W	950
YBR155W	YLL026W	993
YBR155W	YLR216C	950
YBR155W	YMR186W	999

YBR155W	YNL209W	907
YBR155W	YPL106C	966
YBR155W	YPL240C	999
YBR156C	YER082C	935
YBR156C	YFR028C	998
YBR156C	YGL061C	923
YBR156C	YGR113W	987
YBR156C	YGR140W	920
YBR156C	YJR089W	999
YBR156C	YPL209C	999
YBR158W	YGR285C	977
YBR158W	YHR064C	985
YBR158W	YMR001C	973
YBR159W	YCR034W	909
YBR159W	YDR211W	988
YBR159W	YER025W	981
YBR159W	YGR083C	988
YBR159W	YJR007W	962
YBR159W	YKR026C	983
YBR159W	YLR291C	978
YBR159W	YNL265C	962
YBR159W	YOR101W	962
YBR159W	YPL237W	962
YBR160W	YAL024C	943
YBR160W	YAL040C	999
YBR160W	YAL041W	922
YBR160W	YAR007C	910
YBR160W	YBL023C	984
YBR160W	YBL084C	994
YBR160W	YBR060C	998
YBR160W	YBR082C	916
YBR160W	YBR135W	999
YBR160W	YBR202W	908
YBR160W	YBR274W	909
YBR160W	YDL017W	999
YBR160W	YDL056W	999
YBR160W	YDL106C	971
YBR160W	YDL108W	980
YBR160W	YDL140C	967
YBR160W	YDL155W	999
YBR160W	YDL179W	966
YBR160W	YDR113C	986
YBR160W	YDR130C	965
YBR160W	YDR146C	992

YBR160W	YDR168W	990
YBR160W	YDR356W	992
YBR160W	YDR460W	919
YBR160W	YDR507C	947
YBR160W	YEL032W	991
YBR160W	YER012W	904
YBR160W	YER041W	916
YBR160W	YER059W	989
YBR160W	YER111C	999
YBR160W	YFL029C	999
YBR160W	YFR028C	980
YBR160W	YFR046C	916
YBR160W	YFR052W	900
YBR160W	YGL003C	999
YBR160W	YGL058W	976
YBR160W	YGL073W	908
YBR160W	YGL075C	916
YBR160W	YGL116W	997
YBR160W	YGL190C	943
YBR160W	YGL215W	943
YBR160W	YGL240W	981
YBR160W	YGR108W	999
YBR160W	YGR109C	999
YBR160W	YHL007C	979
YBR160W	YHL022C	914
YBR160W	YHR014W	971
YBR160W	YHR030C	996
YBR160W	YHR118C	998
YBR160W	YHR166C	999
YBR160W	YIL050W	963
YBR160W	YIL150C	908
YBR160W	YIR031C	906
YBR160W	YJL157C	999
YBR160W	YJL187C	999
YBR160W	YJL194W	999
YBR160W	YJR076C	906
YBR160W	YJR090C	973
YBR160W	YKL022C	999
YBR160W	YKL042W	971
YBR160W	YKL048C	992
YBR160W	YKL101W	982
YBR160W	YKL108W	956
YBR160W	YKL185W	936
YBR160W	YKR077W	941

YBR160W	YLR079W	999
YBR160W	YLR102C	984
YBR160W	YLR103C	929
YBR160W	YLR127C	912
YBR160W	YLR182W	999
YBR160W	YLR210W	999
YBR160W	YLR274W	909
YBR160W	YLR425W	928
YBR160W	YML032C	976
YBR160W	YML065W	992
YBR160W	YML109W	993
YBR160W	YMR001C	999
YBR160W	YMR036C	999
YBR160W	YMR199W	999
YBR160W	YMR273C	977
YBR160W	YNL064C	918
YBR160W	YNL068C	975
YBR160W	YNL102W	964
YBR160W	YNL197C	918
YBR160W	YNL261W	986
YBR160W	YNL262W	913
YBR160W	YNL289W	930
YBR160W	YNL309W	999
YBR160W	YNL312W	910
YBR160W	YOR083W	996
YBR160W	YOR372C	999
YBR160W	YPL194W	916
YBR160W	YPL219W	949
YBR160W	YPL256C	999
YBR160W	YPL267W	961
YBR160W	YPL269W	997
YBR160W	YPR025C	907
YBR160W	YPR119W	999
YBR160W	YPR120C	999
YBR160W	YPR175W	994
YBR161W	YBR036C	989
YBR161W	YMR251W-A	932
YBR161W	YPL057C	977
YBR162C	YCR012W	962
YBR162C	YDL145C	962
YBR162C	YDL192W	962
YBR162C	YDR238C	994
YBR162C	YER122C	962
YBR162C	YFR051C	962

YBR162C	YGL137W	962
YBR162C	YIL076W	962
YBR162C	YMR214W	994
YBR162C	YNL287W	996
YBR162C	YPL010W	962
YBR162W-A	YLR064W	923
YBR164C	YDR184C	967
YBR164C	YER122C	922
YBR164C	YFR040W	967
YBR164C	YGL005C	938
YBR164C	YHR004C	905
YBR164C	YKL212W	937
YBR164C	YLR039C	920
YBR164C	YLR262C	996
YBR164C	YLR309C	988
YBR164C	YML071C	933
YBR164C	YNL041C	956
YBR164C	YNL051W	959
YBR164C	YNL297C	998
YBR164C	YOL018C	931
YBR164C	YOR070C	997
YBR165W	YDR002W	921
YBR165W	YDR054C	989
YBR166C	YPR060C	950
YBR167C	YAL033W	999
YBR167C	YBL018C	997
YBR167C	YBR257W	999
YBR167C	YDR478W	991
YBR167C	YGR030C	999
YBR167C	YHR062C	999
YBR167C	YIR015W	952
YBR167C	YLR145W	952
YBR167C	YNL221C	999
YBR167C	YNL282W	999
YBR168W	YDL065C	986
YBR168W	YHR150W	936
YBR169C	YDR349C	946
YBR169C	YLL026W	918
YBR169C	YNL007C	959
YBR169C	YNL064C	905
YBR169C	YPL106C	999
YBR170C	YBR201W	977
YBR170C	YDL126C	999
YBR170C	YDL190C	993

YBR170C	YDR411C	971
YBR170C	YFL044C	930
YBR170C	YGL055W	925
YBR170C	YGR048W	999
YBR170C	YIL030C	997
YBR170C	YIR033W	991
YBR170C	YKL020C	998
YBR170C	YKL213C	954
YBR170C	YLR207W	970
YBR170C	YML013W	998
YBR170C	YMR022W	976
YBR170C	YMR264W	996
YBR170C	YOL013C	995
YBR171W	YCL001W	968
YBR171W	YDR086C	996
YBR171W	YER087C-B	997
YBR171W	YJL034W	994
YBR171W	YLR292C	999
YBR171W	YLR378C	997
YBR171W	YOR254C	999
YBR171W	YPL094C	999
YBR172C	YKL074C	968
YBR172C	YLR116W	945
YBR173C	YBL041W	985
YBR173C	YDR419W	972
YBR173C	YER012W	929
YBR173C	YER094C	929
YBR173C	YFL007W	916
YBR173C	YGL011C	999
YBR173C	YGR135W	927
YBR173C	YGR253C	917
YBR173C	YJL001W	954
YBR173C	YKL206C	925
YBR173C	YLR199C	964
YBR173C	YML091C	928
YBR173C	YML092C	929
YBR173C	YMR314W	929
YBR173C	YOL038W	958
YBR173C	YOR056C	906
YBR173C	YOR157C	992
YBR173C	YOR362C	917
YBR173C	YPR103W	999
YBR175W	YAL011W	910
YBR175W	YAR003W	999

YBR175W	YBR231C	902
YBR175W	YBR258C	971
YBR175W	YDL020C	965
YBR175W	YDR334W	937
YBR175W	YDR469W	935
YBR175W	YGR135W	943
YBR175W	YHR119W	999
YBR175W	YJL168C	952
YBR175W	YKL018W	997
YBR175W	YLR015W	999
YBR175W	YLR085C	949
YBR175W	YML041C	952
YBR175W	YOL012C	990
YBR175W	YPL138C	999
YBR176W	YHR063C	958
YBR176W	YIL145C	984
YBR176W	YJR148W	905
YBR176W	YLR291C	931
YBR179C	YDR470C	999
YBR179C	YIL065C	976
YBR179C	YJL112W	999
YBR179C	YLL001W	999
YBR179C	YLR368W	989
YBR179C	YOR211C	999
YBR181C	YAL035W	940
YBR181C	YBL027W	997
YBR181C	YBL072C	993
YBR181C	YBL087C	977
YBR181C	YBL092W	998
YBR181C	YBR031W	995
YBR181C	YBR048W	961
YBR181C	YBR079C	906
YBR181C	YBR084C-A	970
YBR181C	YBR189W	915
YBR181C	YBR191W	997
YBR181C	YCR031C	995
YBR181C	YDL075W	997
YBR181C	YDL081C	927
YBR181C	YDL082W	997
YBR181C	YDL083C	957
YBR181C	YDL136W	994
YBR181C	YDR012W	995
YBR181C	YDR025W	996
YBR181C	YDR064W	959

YBR181C	YDR382W	953
YBR181C	YDR385W	906
YBR181C	YDR418W	994
YBR181C	YDR429C	934
YBR181C	YDR447C	998
YBR181C	YDR450W	907
YBR181C	YDR471W	995
YBR181C	YER025W	961
YBR181C	YER074W	994
YBR181C	YER102W	900
YBR181C	YER117W	901
YBR181C	YER131W	915
YBR181C	YFL034C-A	900
YBR181C	YFR031C-A	997
YBR181C	YFR032C-A	917
YBR181C	YGL014W	972
YBR181C	YGL030W	998
YBR181C	YGL031C	998
YBR181C	YGL076C	996
YBR181C	YGL103W	995
YBR181C	YGL123W	997
YBR181C	YGL135W	902
YBR181C	YGL147C	998
YBR181C	YGR027C	917
YBR181C	YGR034W	996
YBR181C	YGR085C	997
YBR181C	YGR118W	999
YBR181C	YGR148C	917
YBR181C	YGR214W	919
YBR181C	YHL001W	995
YBR181C	YHL015W	913
YBR181C	YHL033C	994
YBR181C	YHR021C	903
YBR181C	YHR141C	998
YBR181C	YHR203C	995
YBR181C	YIL018W	995
YBR181C	YIL069C	995
YBR181C	YIL133C	998
YBR181C	YIL148W	900
YBR181C	YJL136C	906
YBR181C	YJL177W	995
YBR181C	YJL189W	963
YBR181C	YJL190C	983
YBR181C	YJR007W	913

YBR181C	YJR094W-A	903
YBR181C	YJR123W	998
YBR181C	YJR145C	906
YBR181C	YKL006W	904
YBR181C	YKL126W	980
YBR181C	YKL156W	903
YBR181C	YKL180W	995
YBR181C	YKR057W	906
YBR181C	YKR059W	915
YBR181C	YKR094C	900
YBR181C	YLL045C	966
YBR181C	YLR029C	996
YBR181C	YLR048W	906
YBR181C	YLR061W	901
YBR181C	YLR075W	997
YBR181C	YLR264W	921
YBR181C	YLR287C-A	994
YBR181C	YLR325C	937
YBR181C	YLR333C	916
YBR181C	YLR340W	923
YBR181C	YLR344W	997
YBR181C	YLR406C	900
YBR181C	YLR441C	998
YBR181C	YLR448W	998
YBR181C	YML024W	903
YBR181C	YML026C	907
YBR181C	YML063W	995
YBR181C	YML073C	996
YBR181C	YMR121C	923
YBR181C	YMR142C	919
YBR181C	YMR146C	934
YBR181C	YMR194W	995
YBR181C	YMR230W	901
YBR181C	YMR242C	998
YBR181C	YMR260C	932
YBR181C	YNL067W	922
YBR181C	YNL069C	996
YBR181C	YNL096C	996
YBR181C	YNL162W	902
YBR181C	YNL178W	997
YBR181C	YNL301C	995
YBR181C	YNL302C	907
YBR181C	YOL039W	901
YBR181C	YOL040C	997

YBR181C	YOL120C	906
YBR181C	YOL121C	956
YBR181C	YOL127W	996
YBR181C	YOL139C	925
YBR181C	YOR063W	993
YBR181C	YOR096W	993
YBR181C	YOR133W	906
YBR181C	YOR167C	933
YBR181C	YOR234C	904
YBR181C	YOR293W	911
YBR181C	YOR312C	995
YBR181C	YOR361C	908
YBR181C	YOR369C	953
YBR181C	YPL079W	919
YBR181C	YPL090C	994
YBR181C	YPL143W	903
YBR181C	YPL198W	907
YBR181C	YPL220W	994
YBR181C	YPL249C-A	902
YBR181C	YPR041W	919
YBR181C	YPR043W	903
YBR181C	YPR052C	968
YBR181C	YPR067W	970
YBR181C	YPR102C	961
YBR181C	YPR132W	904
YBR181C	YPR163C	912
YBR182C	YHL027W	909
YBR182C	YHR030C	929
YBR182C	YLR113W	998
YBR182C	YPL089C	986
YBR183W	YHL003C	997
YBR183W	YKL008C	993
YBR185C	YER017C	980
YBR185C	YER154W	992
YBR185C	YMR089C	980
YBR186W	YDR285W	998
YBR186W	YER179W	973
YBR186W	YGL249W	947
YBR188C	YDR416W	999
YBR188C	YGR129W	979
YBR188C	YJR050W	999
YBR188C	YKR086W	928
YBR188C	YLL036C	999
YBR188C	YLR117C	999

YBR188C	YMR213W	999
YBR188C	YPL151C	968
YBR188C	YPR101W	998
YBR189W	YAL035W	923
YBR189W	YBL004W	931
YBR189W	YBL027W	902
YBR189W	YBL072C	996
YBR189W	YBL087C	930
YBR189W	YBL092W	957
YBR189W	YBR031W	999
YBR189W	YBR048W	997
YBR189W	YBR079C	929
YBR189W	YBR084C-A	939
YBR189W	YBR118W	901
YBR189W	YBR181C	915
YBR189W	YBR191W	999
YBR189W	YBR247C	990
YBR189W	YCL011C	925
YBR189W	YCL037C	978
YBR189W	YCR031C	999
YBR189W	YDL014W	958
YBR189W	YDL060W	993
YBR189W	YDL061C	991
YBR189W	YDL075W	976
YBR189W	YDL081C	905
YBR189W	YDL082W	969
YBR189W	YDL083C	986
YBR189W	YDL130W	935
YBR189W	YDL136W	947
YBR189W	YDL148C	929
YBR189W	YDL191W	920
YBR189W	YDR012W	989
YBR189W	YDR025W	996
YBR189W	YDR064W	999
YBR189W	YDR382W	999
YBR189W	YDR418W	999
YBR189W	YDR447C	994
YBR189W	YDR449C	929
YBR189W	YDR450W	998
YBR189W	YDR471W	999
YBR189W	YDR500C	953
YBR189W	YEL054C	923
YBR189W	YER025W	968
YBR189W	YER056C-A	981

YBR189W	YER074W	996
YBR189W	YER102W	998
YBR189W	YER117W	948
YBR189W	YER131W	994
YBR189W	YER165W	937
YBR189W	YFL034C-A	906
YBR189W	YFR031C-A	918
YBR189W	YFR032C-A	918
YBR189W	YGL030W	999
YBR189W	YGL031C	998
YBR189W	YGL076C	981
YBR189W	YGL103W	999
YBR189W	YGL123W	998
YBR189W	YGL135W	993
YBR189W	YGL147C	996
YBR189W	YGL173C	925
YBR189W	YGL189C	917
YBR189W	YGR027C	999
YBR189W	YGR034W	916
YBR189W	YGR081C	985
YBR189W	YGR085C	999
YBR189W	YGR090W	977
YBR189W	YGR118W	997
YBR189W	YGR148C	996
YBR189W	YGR162W	919
YBR189W	YGR214W	999
YBR189W	YHL001W	999
YBR189W	YHL015W	999
YBR189W	YHL033C	999
YBR189W	YHR010W	992
YBR189W	YHR021C	917
YBR189W	YHR141C	933
YBR189W	YHR203C	998
YBR189W	YIL018W	901
YBR189W	YIL052C	997
YBR189W	YIL069C	996
YBR189W	YIL133C	998
YBR189W	YIL148W	939
YBR189W	YJL136C	970
YBR189W	YJL177W	999
YBR189W	YJL189W	938
YBR189W	YJL190C	997
YBR189W	YJL191W	999
YBR189W	YJR002W	986

YBR189W	YJR094W-A	971
YBR189W	YJR123W	999
YBR189W	YJR145C	996
YBR189W	YKL006W	999
YBR189W	YKL156W	995
YBR189W	YKL180W	999
YBR189W	YKR057W	922
YBR189W	YKR059W	931
YBR189W	YLL045C	998
YBR189W	YLR029C	999
YBR189W	YLR048W	999
YBR189W	YLR061W	999
YBR189W	YLR075W	999
YBR189W	YLR167W	931
YBR189W	YLR185W	949
YBR189W	YLR186W	958
YBR189W	YLR197W	941
YBR189W	YLR264W	999
YBR189W	YLR287C-A	902
YBR189W	YLR325C	927
YBR189W	YLR333C	943
YBR189W	YLR340W	998
YBR189W	YLR344W	965
YBR189W	YLR367W	999
YBR189W	YLR388W	995
YBR189W	YLR406C	904
YBR189W	YLR441C	997
YBR189W	YLR448W	999
YBR189W	YML024W	992
YBR189W	YML026C	969
YBR189W	YML056C	976
YBR189W	YML063W	999
YBR189W	YML073C	994
YBR189W	YMR116C	987
YBR189W	YMR121C	922
YBR189W	YMR142C	958
YBR189W	YMR194W	999
YBR189W	YMR230W	960
YBR189W	YMR242C	995
YBR189W	YMR260C	908
YBR189W	YNL067W	997
YBR189W	YNL069C	997
YBR189W	YNL096C	997
YBR189W	YNL132W	961

YBR189W	YNL162W	914
YBR189W	YNL178W	999
YBR189W	YNL207W	976
YBR189W	YNL301C	998
YBR189W	YNL302C	919
YBR189W	YOL039W	946
YBR189W	YOL040C	996
YBR189W	YOL120C	904
YBR189W	YOL121C	931
YBR189W	YOL127W	999
YBR189W	YOL139C	915
YBR189W	YOR063W	999
YBR189W	YOR096W	999
YBR189W	YOR133W	914
YBR189W	YOR145C	981
YBR189W	YOR167C	981
YBR189W	YOR182C	902
YBR189W	YOR204W	931
YBR189W	YOR234C	995
YBR189W	YOR293W	985
YBR189W	YOR312C	997
YBR189W	YOR361C	934
YBR189W	YOR369C	950
YBR189W	YPL012W	931
YBR189W	YPL079W	980
YBR189W	YPL081W	999
YBR189W	YPL090C	995
YBR189W	YPL131W	998
YBR189W	YPL143W	985
YBR189W	YPL198W	931
YBR189W	YPL220W	943
YBR189W	YPL237W	951
YBR189W	YPL249C-A	942
YBR189W	YPL266W	978
YBR189W	YPR041W	914
YBR189W	YPR043W	933
YBR189W	YPR080W	901
YBR189W	YPR102C	999
YBR189W	YPR132W	950
YBR189W	YPR163C	923
YBR191W	YAL003W	976
YBR191W	YAL035W	928
YBR191W	YBL027W	999
YBR191W	YBL072C	920

YBR191W	YBL087C	992
YBR191W	YBL092W	999
YBR191W	YBR031W	998
YBR191W	YBR048W	941
YBR191W	YBR084C-A	983
YBR191W	YBR118W	916
YBR191W	YBR181C	997
YBR191W	YBR189W	999
YBR191W	YCR031C	999
YBR191W	YDL061C	999
YBR191W	YDL075W	999
YBR191W	YDL081C	934
YBR191W	YDL082W	997
YBR191W	YDL083C	963
YBR191W	YDL130W	950
YBR191W	YDL136W	999
YBR191W	YDL191W	913
YBR191W	YDL192W	956
YBR191W	YDR012W	998
YBR191W	YDR025W	917
YBR191W	YDR064W	999
YBR191W	YDR382W	999
YBR191W	YDR385W	913
YBR191W	YDR418W	999
YBR191W	YDR447C	996
YBR191W	YDR450W	921
YBR191W	YDR471W	999
YBR191W	YDR500C	984
YBR191W	YEL054C	919
YBR191W	YER056C-A	999
YBR191W	YER074W	995
YBR191W	YER102W	986
YBR191W	YER117W	993
YBR191W	YER131W	974
YBR191W	YFL034C-A	914
YBR191W	YFR031C-A	999
YBR191W	YFR032C-A	998
YBR191W	YGL014W	978
YBR191W	YGL030W	999
YBR191W	YGL031C	996
YBR191W	YGL076C	996
YBR191W	YGL103W	999
YBR191W	YGL123W	999
YBR191W	YGL135W	991

YBR191W	YGL147C	999
YBR191W	YGL189C	910
YBR191W	YGR027C	999
YBR191W	YGR034W	995
YBR191W	YGR085C	999
YBR191W	YGR118W	999
YBR191W	YGR148C	999
YBR191W	YGR214W	999
YBR191W	YHL001W	999
YBR191W	YHL015W	998
YBR191W	YHL033C	999
YBR191W	YHR010W	996
YBR191W	YHR021C	926
YBR191W	YHR141C	999
YBR191W	YHR203C	986
YBR191W	YIL018W	914
YBR191W	YIL052C	999
YBR191W	YIL069C	907
YBR191W	YIL133C	991
YBR191W	YIL148W	984
YBR191W	YJL136C	980
YBR191W	YJL177W	999
YBR191W	YJL189W	956
YBR191W	YJL190C	928
YBR191W	YJL191W	983
YBR191W	YJR094W-A	999
YBR191W	YJR123W	999
YBR191W	YJR145C	943
YBR191W	YKL006W	999
YBR191W	YKL156W	998
YBR191W	YKL180W	999
YBR191W	YKR057W	925
YBR191W	YKR094C	906
YBR191W	YLL045C	948
YBR191W	YLR029C	999
YBR191W	YLR048W	999
YBR191W	YLR060W	940
YBR191W	YLR061W	999
YBR191W	YLR075W	999
YBR191W	YLR167W	973
YBR191W	YLR185W	997
YBR191W	YLR249W	944
YBR191W	YLR264W	999
YBR191W	YLR287C-A	997

YBR191W	YLR325C	909
YBR191W	YLR333C	937
YBR191W	YLR340W	996
YBR191W	YLR344W	999
YBR191W	YLR367W	985
YBR191W	YLR388W	999
YBR191W	YLR406C	921
YBR191W	YLR441C	996
YBR191W	YLR448W	999
YBR191W	YML024W	999
YBR191W	YML026C	921
YBR191W	YML063W	999
YBR191W	YML073C	996
YBR191W	YMR121C	917
YBR191W	YMR142C	952
YBR191W	YMR194W	999
YBR191W	YMR230W	961
YBR191W	YMR242C	996
YBR191W	YMR260C	912
YBR191W	YNL067W	997
YBR191W	YNL069C	995
YBR191W	YNL096C	997
YBR191W	YNL162W	927
YBR191W	YNL178W	999
YBR191W	YNL209W	976
YBR191W	YNL301C	998
YBR191W	YNL302C	919
YBR191W	YOL039W	941
YBR191W	YOL040C	945
YBR191W	YOL120C	929
YBR191W	YOL121C	919
YBR191W	YOL127W	999
YBR191W	YOR063W	999
YBR191W	YOR096W	999
YBR191W	YOR133W	935
YBR191W	YOR167C	966
YBR191W	YOR234C	998
YBR191W	YOR293W	985
YBR191W	YOR312C	946
YBR191W	YOR369C	928
YBR191W	YPL037C	999
YBR191W	YPL079W	996
YBR191W	YPL081W	952
YBR191W	YPL090C	902

YBR191W	YPL131W	999
YBR191W	YPL143W	998
YBR191W	YPL198W	926
YBR191W	YPL220W	990
YBR191W	YPL249C-A	960
YBR191W	YPR043W	966
YBR191W	YPR080W	907
YBR191W	YPR102C	999
YBR191W	YPR132W	994
YBR193C	YBL093C	999
YBR193C	YBR198C	952
YBR193C	YBR253W	999
YBR193C	YCR081W	974
YBR193C	YDL005C	999
YBR193C	YDL140C	982
YBR193C	YDR308C	999
YBR193C	YDR443C	997
YBR193C	YER022W	999
YBR193C	YER148W	984
YBR193C	YGL025C	999
YBR193C	YGL127C	999
YBR193C	YGL151W	997
YBR193C	YGL253W	904
YBR193C	YGR104C	999
YBR193C	YHR041C	999
YBR193C	YHR058C	999
YBR193C	YIL021W	975
YBR193C	YLR071C	999
YBR193C	YML007W	974
YBR193C	YMR112C	999
YBR193C	YNL236W	999
YBR193C	YNR010W	999
YBR193C	YOL051W	999
YBR193C	YOL135C	999
YBR193C	YOR174W	999
YBR193C	YPL042C	989
YBR193C	YPL129W	965
YBR193C	YPR070W	999
YBR193C	YPR168W	999
YBR194W	YPR152C	991
YBR195C	YJL115W	983
YBR195C	YJL141C	904
YBR195C	YJR043C	919
YBR195C	YJR140C	991

YBR195C	YML102W	999
YBR195C	YNL098C	999
YBR195C	YNL183C	984
YBR195C	YNL330C	905
YBR195C	YOR038C	922
YBR195C	YPR018W	999
YBR196C	YBR117C	937
YBR196C	YCL040W	917
YBR196C	YDL215C	946
YBR196C	YDR050C	987
YBR196C	YFR053C	936
YBR196C	YGL229C	900
YBR196C	YGL253W	923
YBR196C	YGR240C	961
YBR196C	YKL127W	913
YBR196C	YLR044C	979
YBR196C	YLR249W	968
YBR196C	YLR377C	979
YBR196C	YMR105C	923
YBR196C	YMR186W	972
YBR196C	YMR205C	962
YBR196C	YNL241C	998
YBR196C	YOL086C	972
YBR196C	YPR074C	942
YBR198C	YBR081C	999
YBR198C	YBR111W-A	959
YBR198C	YBR193C	952
YBR198C	YCL010C	999
YBR198C	YCR042C	999
YBR198C	YDL140C	903
YBR198C	YDR145W	999
YBR198C	YDR167W	999
YBR198C	YDR176W	999
YBR198C	YDR216W	974
YBR198C	YDR392W	999
YBR198C	YDR404C	900
YBR198C	YDR448W	999
YBR198C	YEL009C	995
YBR198C	YER148W	999
YBR198C	YER164W	980
YBR198C	YGL066W	999
YBR198C	YGL070C	900
YBR198C	YGL112C	999
YBR198C	YGL127C	923

YBR198C	YGR005C	940
YBR198C	YGR252W	999
YBR198C	YGR274C	999
YBR198C	YHR041C	996
YBR198C	YHR099W	999
YBR198C	YKL058W	982
YBR198C	YLR055C	999
YBR198C	YML015C	999
YBR198C	YML098W	999
YBR198C	YML114C	999
YBR198C	YMR005W	999
YBR198C	YMR223W	999
YBR198C	YMR227C	999
YBR198C	YMR236W	999
YBR198C	YOL148C	999
YBR198C	YOR174W	943
YBR198C	YOR194C	900
YBR198C	YOR210W	901
YBR198C	YPL011C	999
YBR198C	YPL047W	999
YBR198C	YPL129W	997
YBR198C	YPL254W	999
YBR198C	YPR070W	912
YBR198C	YPR086W	904
YBR198C	YPR187W	900
YBR199W	YAL053W	928
YBR199W	YPL221W	926
YBR200W	YAL041W	999
YBR200W	YBL016W	920
YBR200W	YBL085W	999
YBR200W	YDL159W	922
YBR200W	YDR103W	998
YBR200W	YDR166C	914
YBR200W	YER114C	999
YBR200W	YER155C	967
YBR200W	YFL039C	996
YBR200W	YGL116W	976
YBR200W	YGL233W	997
YBR200W	YGR152C	996
YBR200W	YGR229C	901
YBR200W	YHL007C	999
YBR200W	YIL118W	999
YBR200W	YJL157C	999
YBR200W	YJL187C	989

YBR200W	YKR055W	937
YBR200W	YLR229C	999
YBR200W	YLR362W	940
YBR200W	YMR232W	957
YBR200W	YNL298W	995
YBR200W	YOR181W	994
YBR200W	YOR188W	998
YBR200W	YOR212W	926
YBR200W	YPR055W	974
YBR201W	YBR170C	977
YBR201W	YDL126C	999
YBR201W	YDR057W	978
YBR201W	YDR411C	954
YBR201W	YGR048W	988
YBR201W	YLR207W	999
YBR201W	YML013W	995
YBR201W	YML029W	999
YBR201W	YOL013C	999
YBR202W	YAR007C	942
YBR202W	YBL023C	999
YBR202W	YBR060C	999
YBR202W	YBR160W	908
YBR202W	YDL017W	989
YBR202W	YDR052C	976
YBR202W	YDR489W	980
YBR202W	YEL032W	999
YBR202W	YGL001C	938
YBR202W	YGL201C	999
YBR202W	YIL150C	999
YBR202W	YIR008C	905
YBR202W	YJL194W	999
YBR202W	YJR046W	983
YBR202W	YKL045W	906
YBR202W	YLR103C	999
YBR202W	YLR274W	999
YBR202W	YML065W	990
YBR202W	YMR043W	979
YBR202W	YNL102W	945
YBR202W	YNL261W	999
YBR202W	YNL262W	903
YBR202W	YPR019W	999
YBR202W	YPR162C	901
YBR205W	YHR105W	941
YBR207W	YFL041W	996

YBR208C	YBR218C	930
YBR208C	YBR221C	919
YBR208C	YGL062W	930
YBR208C	YIR023W	951
YBR208C	YIR029W	944
YBR208C	YIR032C	988
YBR208C	YKR034W	916
YBR208C	YNL314W	945
YBR211C	YAL034W-A	996
YBR211C	YBR107C	999
YBR211C	YDR254W	999
YBR211C	YDR318W	999
YBR211C	YDR383C	999
YBR211C	YDR394W	999
YBR211C	YGL060W	981
YBR211C	YGR179C	999
YBR211C	YIR010W	906
YBR211C	YJR112W	980
YBR211C	YJR135C	999
YBR211C	YKL138C-A	963
YBR211C	YLR315W	999
YBR211C	YLR381W	999
YBR211C	YPL018W	999
YBR211C	YPL233W	977
YBR211C	YPR046W	998
YBR213W	YKR069W	993
YBR215W	YBL008W	999
YBR215W	YER161C	908
YBR215W	YJL115W	916
YBR215W	YJR140C	999
YBR215W	YOR038C	999
YBR216C	YIR037W	944
YBR216C	YML007W	998
YBR217W	YBL078C	986
YBR217W	YHR171W	999
YBR217W	YLL042C	989
YBR217W	YMR159C	999
YBR217W	YNL223W	942
YBR217W	YNR007C	999
YBR217W	YPL149W	999
YBR218C	YAL038W	991
YBR218C	YBR208C	930
YBR218C	YBR221C	905
YBR218C	YCR005C	927

YBR218C	YDL078C	970
YBR218C	YDR111C	905
YBR218C	YER065C	933
YBR218C	YER178W	907
YBR218C	YGL062W	979
YBR218C	YGR240C	908
YBR218C	YGR254W	907
YBR218C	YHR174W	907
YBR218C	YKL085W	961
YBR218C	YKL106W	903
YBR218C	YKR097W	991
YBR218C	YLR027C	912
YBR218C	YLR089C	914
YBR218C	YLR377C	920
YBR218C	YMR205C	910
YBR218C	YNL117W	978
YBR218C	YNR001C	920
YBR218C	YOL126C	948
YBR218C	YOR347C	915
YBR218C	YPR001W	902
YBR221C	YAL038W	924
YBR221C	YBR208C	919
YBR221C	YBR218C	905
YBR221C	YDL174C	902
YBR221C	YDR430C	999
YBR221C	YER178W	999
YBR221C	YFL018C	999
YBR221C	YGL062W	905
YBR221C	YGR193C	999
YBR221C	YLR418C	927
YBR221C	YML059C	978
YBR221C	YNL071W	999
YBR221C	YOR347C	909
YBR221C	YPL017C	962
YBR222C	YEL020C	999
YBR223C	YDR217C	976
YBR223C	YDR386W	900
YBR223C	YKL114C	964
YBR223C	YML032C	978
YBR223C	YOL006C	993
YBR223C	YPL022W	999
YBR225W	YGL197W	999
YBR225W	YLR310C	999
YBR227C	YBL022C	964

YBR227C	YDR258C	931
YBR227C	YOR232W	900
YBR228W	YDR363W	945
YBR228W	YLR135W	999
YBR228W	YLR234W	950
YBR228W	YMR190C	996
YBR228W	YPL024W	978
YBR229C	YAL058W	924
YBR229C	YCL043C	923
YBR229C	YDR221W	999
YBR229C	YDR389W	905
YBR229C	YFL025C	913
YBR229C	YGL027C	989
YBR229C	YHR101C	957
YBR229C	YML067C	969
YBR229C	YMR200W	975
YBR231C	YAL011W	999
YBR231C	YBR175W	902
YBR231C	YDL074C	964
YBR231C	YDR190C	999
YBR231C	YDR334W	999
YBR231C	YDR392W	907
YBR231C	YDR485C	999
YBR231C	YEL018W	973
YBR231C	YFL039C	999
YBR231C	YGL043W	972
YBR231C	YGL127C	975
YBR231C	YGL244W	916
YBR231C	YGR002C	999
YBR231C	YJL081C	999
YBR231C	YLR015W	909
YBR231C	YLR055C	931
YBR231C	YLR085C	999
YBR231C	YLR385C	999
YBR231C	YLR399C	997
YBR231C	YLR418C	942
YBR231C	YML041C	998
YBR231C	YML094W	926
YBR231C	YNL107W	999
YBR231C	YNL136W	900
YBR231C	YOL012C	971
YBR231C	YOR123C	910
YBR231C	YOR349W	908
YBR231C	YPL055C	964

YBR231C	YPL235W	999
YBR231C	YPL241C	909
YBR233W-A	YDR016C	998
YBR233W-A	YGL061C	963
YBR233W-A	YGR113W	963
YBR233W-A	YKL052C	961
YBR233W-A	YKL138C-A	978
YBR233W-A	YKR037C	992
YBR233W-A	YKR083C	959
YBR234C	YDL029W	999
YBR234C	YIL062C	999
YBR234C	YIR006C	966
YBR234C	YJR065C	999
YBR234C	YKL013C	999
YBR234C	YLR370C	999
YBR234C	YLR429W	981
YBR234C	YLR453C	944
YBR234C	YMR109W	994
YBR234C	YNR035C	999
YBR234C	YOR181W	997
YBR236C	YDL108W	925
YBR236C	YDL140C	997
YBR236C	YDR502C	983
YBR236C	YGL130W	934
YBR236C	YLR180W	983
YBR236C	YML010W	997
YBR236C	YOR151C	983
YBR237W	YMR240C	948
YBR237W	YMR288W	908
YBR237W	YNL286W	990
YBR238C	YBL085W	994
YBR240C	YDL080C	993
YBR243C	YBR070C	973
YBR243C	YBR110W	948
YBR243C	YGL047W	973
YBR243C	YGR036C	956
YBR243C	YMR013C	984
YBR243C	YPL227C	974
YBR243C	YPR183W	968
YBR244W	YIR037W	971
YBR244W	YLR299W	901
YBR244W	YOL049W	910
YBR244W	YPL091W	979
YBR245C	YBL003C	996

YBR245C	YBR009C	995
YBR245C	YBR010W	935
YBR245C	YBR089C-A	975
YBR245C	YCR052W	999
YBR245C	YDL002C	957
YBR245C	YDR190C	978
YBR245C	YDR303C	999
YBR245C	YDR334W	956
YBR245C	YDR359C	931
YBR245C	YER164W	997
YBR245C	YFL039C	974
YBR245C	YFR013W	999
YBR245C	YFR037C	999
YBR245C	YGL043W	942
YBR245C	YGL133W	999
YBR245C	YGL207W	971
YBR245C	YGR056W	962
YBR245C	YHR090C	931
YBR245C	YIL126W	999
YBR245C	YKR001C	999
YBR245C	YKR008W	999
YBR245C	YLR033W	999
YBR245C	YLR095C	971
YBR245C	YLR176C	984
YBR245C	YLR321C	962
YBR245C	YLR357W	999
YBR245C	YML127W	962
YBR245C	YMR044W	972
YBR245C	YMR072W	977
YBR245C	YMR091C	999
YBR245C	YNL030W	994
YBR245C	YNL031C	912
YBR245C	YOL012C	978
YBR245C	YOL017W	998
YBR245C	YOR207C	970
YBR245C	YOR304W	999
YBR245C	YOR372C	903
YBR245C	YPL082C	999
YBR245C	YPR034W	957
YBR247C	YBL004W	999
YBR247C	YBL072C	989
YBR247C	YBR048W	975
YBR247C	YBR189W	990
YBR247C	YCL059C	999

YBR247C	YCR057C	994
YBR247C	YDL014W	999
YBR247C	YDL060W	999
YBR247C	YDL148C	999
YBR247C	YDL153C	918
YBR247C	YDR064W	990
YBR247C	YDR299W	958
YBR247C	YDR324C	996
YBR247C	YDR398W	963
YBR247C	YDR449C	999
YBR247C	YER082C	999
YBR247C	YGL120C	923
YBR247C	YGL171W	967
YBR247C	YGR081C	999
YBR247C	YGR090W	993
YBR247C	YGR128C	967
YBR247C	YGR145W	999
YBR247C	YHR148W	999
YBR247C	YHR169W	978
YBR247C	YHR196W	958
YBR247C	YJL010C	985
YBR247C	YJL109C	983
YBR247C	YJR002W	999
YBR247C	YJR145C	989
YBR247C	YKL099C	958
YBR247C	YKL143W	999
YBR247C	YKR060W	963
YBR247C	YLL011W	999
YBR247C	YLR003C	914
YBR247C	YLR129W	992
YBR247C	YLR175W	995
YBR247C	YLR186W	999
YBR247C	YLR197W	974
YBR247C	YLR222C	975
YBR247C	YLR409C	978
YBR247C	YML093W	916
YBR247C	YMR093W	999
YBR247C	YMR128W	999
YBR247C	YMR229C	976
YBR247C	YMR290C	923
YBR247C	YNL075W	999
YBR247C	YNL132W	999
YBR247C	YNL178W	988
YBR247C	YNL207W	999

YBR247C	YOL010W	996
YBR247C	YOR004W	919
YBR247C	YOR056C	999
YBR247C	YOR078W	981
YBR247C	YOR096W	931
YBR247C	YOR145C	981
YBR247C	YPL012W	999
YBR247C	YPL126W	968
YBR247C	YPL204W	999
YBR247C	YPL217C	999
YBR247C	YPL266W	999
YBR247C	YPR112C	943
YBR247C	YPR137W	971
YBR247C	YPR144C	999
YBR248C	YCL030C	968
YBR248C	YDL106C	934
YBR248C	YER055C	960
YBR248C	YIL020C	930
YBR248C	YIL116W	906
YBR248C	YLR028C	918
YBR248C	YMR120C	919
YBR248C	YOR202W	996
YBR249C	YDR127W	919
YBR250W	YNL012W	990
YBR251W	Q0140	962
YBR251W	YBL090W	999
YBR251W	YBR146W	999
YBR251W	YDL045W-A	947
YBR251W	YDR036C	983
YBR251W	YDR041W	999
YBR251W	YDR175C	999
YBR251W	YDR337W	999
YBR251W	YDR347W	999
YBR251W	YGL129C	999
YBR251W	YGR084C	999
YBR251W	YGR165W	988
YBR251W	YGR215W	999
YBR251W	YHL004W	999
YBR251W	YHR059W	947
YBR251W	YIL093C	999
YBR251W	YJR101W	999
YBR251W	YJR113C	999
YBR251W	YKL003C	999
YBR251W	YKL155C	999

YBR251W	YMR158W	974
YBR251W	YMR188C	988
YBR251W	YNL081C	934
YBR251W	YNL137C	999
YBR251W	YNL186W	932
YBR251W	YNL306W	999
YBR251W	YNR036C	965
YBR251W	YNR037C	999
YBR251W	YOR158W	988
YBR251W	YOR205C	906
YBR251W	YOR243C	952
YBR251W	YPL013C	999
YBR251W	YPL118W	999
YBR252W	YBL019W	988
YBR252W	YHR144C	971
YBR252W	YJR057W	941
YBR252W	YKL067W	908
YBR252W	YKL114C	988
YBR252W	YML021C	998
YBR252W	YOR074C	956
YBR253W	YBL093C	999
YBR253W	YBR193C	999
YBR253W	YBR279W	964
YBR253W	YCR081W	999
YBR253W	YDL005C	999
YBR253W	YDL108W	936
YBR253W	YDL140C	999
YBR253W	YDR167W	991
YBR253W	YDR308C	999
YBR253W	YDR443C	999
YBR253W	YDR448W	995
YBR253W	YER022W	999
YBR253W	YER148W	958
YBR253W	YGL025C	999
YBR253W	YGL112C	977
YBR253W	YGL127C	999
YBR253W	YGL151W	999
YBR253W	YGL244W	970
YBR253W	YGR005C	983
YBR253W	YGR104C	999
YBR253W	YHR041C	999
YBR253W	YHR058C	999
YBR253W	YLR071C	999
YBR253W	YLR418C	970

YBR253W	YMR112C	999
YBR253W	YNL025C	993
YBR253W	YNL236W	999
YBR253W	YNR010W	999
YBR253W	YOL051W	999
YBR253W	YOL135C	999
YBR253W	YOL145C	970
YBR253W	YOR123C	964
YBR253W	YOR174W	999
YBR253W	YPL042C	999
YBR253W	YPL129W	991
YBR253W	YPR056W	984
YBR253W	YPR070W	999
YBR253W	YPR168W	999
YBR254C	YDR108W	999
YBR254C	YDR246W	999
YBR254C	YDR407C	999
YBR254C	YDR472W	999
YBR254C	YGR143W	933
YBR254C	YGR166W	999
YBR254C	YKR068C	999
YBR254C	YML077W	999
YBR254C	YMR218C	999
YBR254C	YOR115C	999
YBR256C	YBL033C	924
YBR256C	YDR487C	968
YBR256C	YOL143C	996
YBR256C	YPR073C	926
YBR257W	YAL033W	999
YBR257W	YBL018C	999
YBR257W	YBR167C	999
YBR257W	YDR478W	999
YBR257W	YGR030C	999
YBR257W	YHR062C	999
YBR257W	YIR015W	999
YBR257W	YLR145W	995
YBR257W	YNL221C	999
YBR257W	YNL282W	999
YBR258C	YAR003W	999
YBR258C	YBR175W	971
YBR258C	YDR469W	999
YBR258C	YHR119W	999
YBR258C	YJL168C	952
YBR258C	YKL018W	994

YBR258C	YLR015W	999
YBR258C	YPL138C	999
YBR260C	YGR196C	998
YBR260C	YIL118W	998
YBR260C	YKR055W	998
YBR260C	YLR332W	968
YBR260C	YLR337C	983
YBR260C	YMR032W	926
YBR260C	YNL090W	996
YBR260C	YOR008C	998
YBR260C	YOR181W	993
YBR260C	YPR165W	950
YBR263W	YBR084W	994
YBR263W	YCL064C	906
YBR263W	YDR019C	977
YBR263W	YDR023W	916
YBR263W	YDR232W	915
YBR263W	YDR408C	927
YBR263W	YEL046C	999
YBR263W	YER086W	909
YBR263W	YFL030W	983
YBR263W	YGL125W	933
YBR263W	YGR155W	934
YBR263W	YGR204W	999
YBR263W	YGR208W	966
YBR263W	YKR080W	932
YBR263W	YLR028C	908
YBR263W	YLR058C	985
YBR263W	YMR120C	906
YBR263W	YMR189W	962
YBR263W	YOR074C	940
YBR263W	YOR236W	926
YBR263W	YOR241W	912
YBR263W	YPL023C	900
YBR264C	YER136W	995
YBR265W	YBR126C	929
YBR267W	YDR101C	999
YBR267W	YDR507C	948
YBR267W	YGL099W	999
YBR267W	YHR170W	999
YBR267W	YJL122W	971
YBR267W	YKR048C	997
YBR267W	YKR079C	970
YBR267W	YLR387C	959

YBR267W	YNL227C	920
YBR267W	YPR016C	949
YBR270C	YFR050C	999
YBR270C	YIL105C	986
YBR272C	YDL007W	971
YBR272C	YDL097C	984
YBR272C	YDR174W	957
YBR272C	YDR317W	952
YBR272C	YDR394W	978
YBR272C	YDR427W	995
YBR272C	YEL056W	987
YBR272C	YFR052W	983
YBR272C	YGL048C	978
YBR272C	YGR258C	989
YBR272C	YLL022C	981
YBR272C	YNL082W	916
YBR272C	YOL054W	977
YBR272C	YOR117W	994
YBR272C	YOR261C	988
YBR272C	YPL001W	983
YBR272C	YPL167C	924
YBR273C	YDL126C	992
YBR273C	YDR411C	971
YBR273C	YKL213C	993
YBR274W	YBL088C	989
YBR274W	YBR136W	997
YBR274W	YBR160W	909
YBR274W	YDL101C	997
YBR274W	YDL220C	961
YBR274W	YDR113C	999
YBR274W	YDR499W	973
YBR274W	YJL164C	920
YBR274W	YJL187C	921
YBR274W	YMR036C	905
YBR274W	YMR284W	995
YBR274W	YNL234W	957
YBR274W	YPL203W	923
YBR274W	YPR119W	911
YBR275C	YBL088C	952
YBR275C	YLR453C	993
YBR275C	YNL216W	998
YBR276C	YBL068W	996
YBR276C	YKL181W	998
YBR276C	YOL061W	960

YBR278W	YDL102W	960
YBR278W	YDR121W	999
YBR278W	YJL090C	945
YBR278W	YNL262W	999
YBR278W	YPR175W	999
YBR279W	YAL021C	991
YBR279W	YBR154C	927
YBR279W	YBR253W	964
YBR279W	YDL056W	945
YBR279W	YDL108W	946
YBR279W	YDL140C	999
YBR279W	YDR138W	999
YBR279W	YDR167W	965
YBR279W	YDR308C	964
YBR279W	YDR443C	907
YBR279W	YER022W	972
YBR279W	YER111C	991
YBR279W	YER148W	952
YBR279W	YFL029C	963
YBR279W	YGL019W	966
YBR279W	YGL043W	938
YBR279W	YGL058W	913
YBR279W	YGL070C	969
YBR279W	YGL127C	926
YBR279W	YGL207W	999
YBR279W	YGL244W	999
YBR279W	YGR005C	998
YBR279W	YGR063C	947
YBR279W	YGR104C	999
YBR279W	YGR186W	938
YBR279W	YHR041C	973
YBR279W	YIL035C	985
YBR279W	YJL168C	986
YBR279W	YKL139W	914
YBR279W	YLR071C	919
YBR279W	YLR182W	960
YBR279W	YLR226W	977
YBR279W	YLR418C	999
YBR279W	YML010W	998
YBR279W	YML069W	999
YBR279W	YMR247C	900
YBR279W	YOL051W	997
YBR279W	YOL145C	999
YBR279W	YOR039W	972

YBR279W	YOR061W	985
YBR279W	YOR123C	999
YBR279W	YOR151C	927
YBR279W	YPL129W	984
YBR279W	YPR187W	929
YBR280C	YDL132W	998
YBR280C	YDR054C	923
YBR280C	YDR328C	990
YBR280C	YIL046W	910
YBR280C	YNL141W	999
YBR280C	YPL240C	919
YBR281C	YNL191W	990
YBR282W	YCR046C	991
YBR282W	YDR116C	970
YBR282W	YDR237W	970
YBR282W	YDR296W	970
YBR282W	YDR322W	970
YBR282W	YLR439W	970
YBR282W	YMR024W	970
YBR282W	YMR193W	972
YBR282W	YNL005C	973
YBR282W	YNL252C	970
YBR282W	YNL284C	934
YBR283C	YDR086C	977
YBR283C	YER019C-A	978
YBR283C	YER087C-B	975
YBR283C	YJL034W	920
YBR283C	YLR378C	986
YBR288C	YGL095C	916
YBR288C	YGR261C	999
YBR288C	YJL004C	947
YBR288C	YJL024C	997
YBR288C	YLR093C	921
YBR288C	YPL195W	999
YBR289W	YBL008W	918
YBR289W	YDR073W	999
YBR289W	YDR224C	949
YBR289W	YEL009C	999
YBR289W	YFL049W	999
YBR289W	YGR116W	952
YBR289W	YGR252W	976
YBR289W	YHL025W	999
YBR289W	YJL176C	999
YBR289W	YMR033W	999

YBR289W	YNR023W	999
YBR289W	YOR038C	923
YBR289W	YOR290C	999
YBR289W	YPL016W	999
YBR289W	YPL129W	999
YBR289W	YPR034W	999
YBR290W	YCL001W	985
YBR290W	YER125W	987
YBR290W	YGL012W	940
YBR290W	YHR008C	957
YBR290W	YHR050W	947
YBR290W	YJR104C	999
YBR290W	YKL034W	939
YBR290W	YMR123W	919
YBR290W	YOL122C	957
YBR290W	YPL176C	943
YBR290W	YPL240C	970
YBR293W	YCL069W	906
YBR295W	YBR135W	901
YBR296C	YML123C	987
YBR299W	YGR292W	947
YCL001W	YBR171W	968
YCL001W	YBR290W	985
YCL001W	YJL002C	944
YCL001W	YJR131W	955
YCL001W	YML071C	909
YCL001W	YNR026C	959
YCL001W	YOR085W	907
YCL001W	YPL227C	905
YCL004W	YDL142C	970
YCL004W	YER026C	968
YCL004W	YOR336W	942
YCL004W	YPR113W	922
YCL008C	YGR206W	999
YCL008C	YHL002W	983
YCL008C	YKL041W	907
YCL008C	YLR119W	999
YCL008C	YLR417W	957
YCL008C	YNR006W	998
YCL008C	YPL065W	999
YCL009C	YCL018W	974
YCL009C	YER086W	922
YCL009C	YLR355C	991
YCL009C	YMR108W	999

YCL010C	YBR081C	999
YCL010C	YBR111W-A	959
YCL010C	YBR198C	999
YCL010C	YDR145W	999
YCL010C	YDR167W	999
YCL010C	YDR176W	999
YCL010C	YDR392W	999
YCL010C	YDR448W	999
YCL010C	YER164W	980
YCL010C	YGL066W	999
YCL010C	YGL112C	999
YCL010C	YGR252W	999
YCL010C	YHR099W	997
YCL010C	YLR055C	999
YCL010C	YMR223W	999
YCL010C	YMR236W	999
YCL010C	YOL148C	999
YCL010C	YPL047W	999
YCL010C	YPL254W	999
YCL011C	YBL072C	927
YCL011C	YBR048W	925
YCL011C	YBR189W	925
YCL011C	YCL037C	974
YCL011C	YDL014W	938
YCL011C	YDL084W	999
YCL011C	YDR025W	978
YCL011C	YDR064W	929
YCL011C	YDR138W	999
YCL011C	YDR381W	999
YCL011C	YDR432W	998
YCL011C	YER102W	925
YCL011C	YGL049C	972
YCL011C	YGL120C	979
YCL011C	YGL173C	976
YCL011C	YGR162W	976
YCL011C	YHL034C	979
YCL011C	YHR167W	999
YCL011C	YHR203C	923
YCL011C	YJR145C	936
YCL011C	YKL139W	979
YCL011C	YLL045C	929
YCL011C	YLR432W	985
YCL011C	YML062C	999
YCL011C	YMR125W	976

YCL011C	YNL004W	910
YCL011C	YNL139C	999
YCL011C	YNL253W	998
YCL011C	YOL139C	977
YCL011C	YOR204W	978
YCL011C	YPL081W	972
YCL014W	YBL038W	942
YCL014W	YHR107C	937
YCL014W	YIL140W	948
YCL014W	YKR085C	913
YCL014W	YLR353W	924
YCL014W	YMR186W	907
YCL014W	YPL256C	962
YCL014W	YPR119W	997
YCL016C	YCL061C	934
YCL016C	YER016W	967
YCL016C	YHR191C	999
YCL016C	YJL092W	946
YCL016C	YJL168C	952
YCL016C	YJR043C	923
YCL016C	YMR048W	997
YCL016C	YMR078C	977
YCL016C	YNL273W	956
YCL016C	YNL298W	930
YCL017C	YDL033C	902
YCL017C	YDL120W	999
YCL017C	YER048W-A	999
YCL017C	YGL018C	953
YCL017C	YJR104C	991
YCL017C	YLL027W	903
YCL017C	YLR369W	972
YCL017C	YOR226C	989
YCL017C	YPL135W	999
YCL017C	YPL252C	989
YCL018W	YCL009C	974
YCL018W	YCL064C	928
YCL018W	YDR160W	991
YCL018W	YEL009C	909
YCL018W	YER086W	924
YCL018W	YFR029W	939
YCL018W	YGL009C	999
YCL018W	YHR208W	911
YCL018W	YJR148W	931
YCL018W	YLR355C	968

YCL018W	YLR451W	985
YCL018W	YMR108W	995
YCL018W	YNL104C	913
YCL019W	YBL100W-B	966
YCL019W	YDR261W-A	998
YCL019W	YDR261W-B	998
YCL020W	YBL100W-A	998
YCL024W	YAL027W	925
YCL024W	YCR002C	982
YCL024W	YDR507C	999
YCL024W	YHR107C	986
YCL024W	YJR076C	983
YCL024W	YKL101W	919
YCL024W	YKR048C	999
YCL024W	YLR314C	983
YCL025C	YJR090C	922
YCL027W	YBL016W	999
YCL027W	YCL032W	923
YCL027W	YCR009C	990
YCL027W	YDR103W	999
YCL027W	YER118C	999
YCL027W	YER149C	999
YCL027W	YGR040W	999
YCL027W	YIL159W	999
YCL027W	YJL157C	998
YCL027W	YLL021W	999
YCL027W	YLR330W	999
YCL027W	YMR232W	999
YCL027W	YNL271C	999
YCL028W	YAL005C	965
YCL028W	YDR172W	973
YCL028W	YLL026W	955
YCL028W	YNL007C	999
YCL028W	YNL064C	978
YCL028W	YNL229C	904
YCL029C	YDL155W	962
YCL029C	YEL061C	992
YCL029C	YER016W	999
YCL029C	YGL086W	939
YCL029C	YGR113W	979
YCL029C	YGR188C	951
YCL029C	YJL030W	939
YCL029C	YJR112W	976
YCL029C	YLL003W	904

YCL029C	YLR045C	999
YCL029C	YML085C	976
YCL029C	YMR117C	984
YCL029C	YOR026W	928
YCL029C	YOR058C	974
YCL029C	YOR269W	947
YCL029C	YOR349W	965
YCL029C	YPL155C	994
YCL029C	YPL241C	965
YCL029C	YPL269W	999
YCL030C	YBR248C	968
YCL030C	YDR392W	988
YCL030C	YEL009C	965
YCL030C	YER055C	999
YCL030C	YER161C	985
YCL030C	YIL020C	962
YCL030C	YIL116W	998
YCL030C	YKR099W	974
YCL030C	YLR055C	912
YCL030C	YNL216W	923
YCL030C	YOR202W	999
YCL030C	YPR033C	960
YCL031C	YDR299W	938
YCL031C	YGL019W	989
YCL031C	YGR090W	999
YCL031C	YIL035C	999
YCL031C	YJL010C	983
YCL031C	YKR060W	910
YCL031C	YLR003C	975
YCL031C	YLR175W	909
YCL031C	YLR223C	995
YCL031C	YOR039W	991
YCL031C	YOR061W	989
YCL031C	YPL217C	922
YCL032W	YCL027W	923
YCL032W	YDR103W	998
YCL032W	YDR309C	905
YCL032W	YER118C	987
YCL032W	YHL007C	914
YCL032W	YHR061C	905
YCL032W	YLR006C	975
YCL032W	YLR113W	990
YCL032W	YLR229C	993
YCL032W	YLR362W	999

YCL032W	YOR212W	999
YCL032W	YPR075C	928
YCL033C	YER042W	995
YCL034W	YBL007C	976
YCL034W	YHR016C	937
YCL034W	YOR094W	987
YCL034W	YOR181W	995
YCL035C	YDR513W	984
YCL035C	YGR209C	934
YCL035C	YJL026W	900
YCL035C	YPL091W	927
YCL037C	YBL072C	978
YCL037C	YBR189W	978
YCL037C	YCL011C	974
YCL037C	YDL014W	987
YCL037C	YDL083C	978
YCL037C	YGL049C	970
YCL037C	YGL120C	993
YCL037C	YGL173C	974
YCL037C	YGR162W	992
YCL037C	YHL034C	978
YCL037C	YIL118W	909
YCL037C	YIL138C	922
YCL037C	YJL190C	978
YCL037C	YJR145C	978
YCL037C	YLR175W	980
YCL037C	YLR256W	976
YCL037C	YML056C	972
YCL037C	YMR125W	995
YCL037C	YMR230W	972
YCL037C	YMR290C	978
YCL037C	YNL064C	924
YCL037C	YNL079C	912
YCL037C	YOL139C	980
YCL037C	YOR096W	925
YCL037C	YPL131W	978
YCL038C	YKL146W	906
YCL038C	YNL101W	903
YCL039W	YBR105C	981
YCL039W	YDR255C	995
YCL039W	YGL227W	999
YCL039W	YIL017C	995
YCL039W	YIL097W	995
YCL039W	YMR135C	998

YCL040W	YBR196C	917
YCL040W	YDL035C	967
YCL040W	YDR516C	964
YCL040W	YFR053C	998
YCL040W	YGL253W	997
YCL040W	YMR105C	962
YCL043C	YBR229C	923
YCL043C	YDR518W	978
YCL043C	YHR079C	915
YCL043C	YHR204W	999
YCL043C	YIR037W	965
YCL043C	YML130C	993
YCL043C	YOL088C	982
YCL043C	YOR288C	976
YCL044C	YPR024W	991
YCL045C	YJL196C	923
YCL045C	YML051W	912
YCL046W	YGL115W	918
YCL048W	YBL014C	998
YCL048W	YCR024C	998
YCL050C	YDR530C	902
YCL050C	YEL071W	998
YCL051W	YDR099W	961
YCL051W	YNL161W	981
YCL051W	YOR370C	918
YCL052C	YAL058W	922
YCL052C	YEL060C	908
YCL052C	YJR013W	929
YCL054W	YDL031W	996
YCL054W	YER006W	998
YCL054W	YER165W	993
YCL054W	YGL111W	976
YCL054W	YGR103W	988
YCL054W	YKL172W	977
YCL054W	YKR081C	939
YCL054W	YLL008W	981
YCL054W	YMR290C	978
YCL054W	YNL002C	980
YCL054W	YNL061W	979
YCL054W	YNL110C	999
YCL054W	YOR310C	927
YCL054W	YPL093W	978
YCL054W	YPL211W	975
YCL055W	YHR084W	982

YCL055W	YPR141C	905
YCL056C	YDL157C	954
YCL059C	YBL004W	999
YCL059C	YBL072C	998
YCL059C	YBR031W	943
YCL059C	YBR048W	996
YCL059C	YBR247C	999
YCL059C	YCR031C	938
YCL059C	YCR057C	998
YCL059C	YDL014W	999
YCL059C	YDL060W	914
YCL059C	YDL148C	999
YCL059C	YDL153C	975
YCL059C	YDL213C	998
YCL059C	YDR299W	999
YCL059C	YDR324C	997
YCL059C	YDR339C	938
YCL059C	YDR449C	999
YCL059C	YER082C	999
YCL059C	YER102W	955
YCL059C	YGL078C	903
YCL059C	YGL120C	968
YCL059C	YGL171W	983
YCL059C	YGR081C	915
YCL059C	YGR090W	985
YCL059C	YGR128C	923
YCL059C	YGR145W	999
YCL059C	YGR214W	943
YCL059C	YHR148W	999
YCL059C	YHR169W	931
YCL059C	YHR203C	998
YCL059C	YIL019W	991
YCL059C	YIL035C	911
YCL059C	YJL010C	911
YCL059C	YJL069C	952
YCL059C	YJL109C	997
YCL059C	YJR002W	999
YCL059C	YJR123W	943
YCL059C	YJR145C	998
YCL059C	YKL099C	914
YCL059C	YKR060W	996
YCL059C	YLL011W	999
YCL059C	YLR003C	952
YCL059C	YLR129W	975

YCL059C	YLR175W	999
YCL059C	YLR186W	999
YCL059C	YLR197W	999
YCL059C	YLR222C	979
YCL059C	YLR409C	999
YCL059C	YML093W	915
YCL059C	YMR093W	997
YCL059C	YMR128W	999
YCL059C	YMR229C	999
YCL059C	YMR290C	998
YCL059C	YNL075W	934
YCL059C	YNL110C	934
YCL059C	YNL132W	999
YCL059C	YNL178W	951
YCL059C	YNL308C	999
YCL059C	YOL010W	920
YCL059C	YOR004W	941
YCL059C	YOR039W	905
YCL059C	YOR061W	910
YCL059C	YOR078W	970
YCL059C	YOR096W	953
YCL059C	YPL012W	967
YCL059C	YPL126W	995
YCL059C	YPL217C	999
YCL059C	YPL266W	906
YCL059C	YPR102C	943
YCL059C	YPR112C	925
YCL059C	YPR137W	999
YCL059C	YPR144C	999
YCL061C	YAL021C	970
YCL061C	YBL023C	971
YCL061C	YCL016C	934
YCL061C	YDL220C	989
YCL061C	YDR075W	950
YCL061C	YDR217C	998
YCL061C	YER016W	906
YCL061C	YER095W	914
YCL061C	YER173W	963
YCL061C	YHR031C	998
YCL061C	YJL092W	998
YCL061C	YJL115W	915
YCL061C	YJL194W	952
YCL061C	YJR043C	959
YCL061C	YKL113C	943

YCL061C	YLR103C	904
YCL061C	YMR048W	997
YCL061C	YMR190C	998
YCL061C	YMR198W	915
YCL061C	YMR284W	968
YCL061C	YNL273W	999
YCL061C	YOL012C	911
YCL061C	YOR144C	954
YCL061C	YOR368W	949
YCL061C	YPL008W	902
YCL061C	YPL153C	996
YCL061C	YPR135W	912
YCL063W	YEL013W	999
YCL063W	YFR021W	992
YCL063W	YGL106W	936
YCL063W	YOR326W	999
YCL063W	YPL240C	919
YCL064C	YAL012W	905
YCL064C	YBR263W	906
YCL064C	YCL018W	928
YCL064C	YEL046C	903
YCL064C	YGR155W	908
YCL064C	YLR058C	907
YCL064C	YLR098C	943
YCL064C	YMR108W	901
YCL065W	YCR041W	964
YCL066W	YCL067C	989
YCL066W	YCR097W	958
YCL066W	YHR084W	975
YCL066W	YMR043W	992
YCL067C	YBR112C	994
YCL067C	YCL066W	989
YCL067C	YCR084C	999
YCL067C	YCR097W	999
YCL067C	YJL170C	922
YCL067C	YMR043W	999
YCL069W	YBR293W	906
YCL069W	YMR088C	967
YCR002C	YCL024W	982
YCR002C	YDL225W	999
YCR002C	YDR507C	998
YCR002C	YHR107C	999
YCR002C	YJR076C	999
YCR002C	YJR092W	999

YCR002C	YLL021W	989
YCR002C	YLR314C	999
YCR002C	YNL233W	995
YCR002C	YPL242C	939
YCR004C	YBR052C	999
YCR004C	YDR032C	999
YCR005C	YAR035W	933
YCR005C	YBR218C	927
YCR005C	YDL078C	990
YCR005C	YDL120W	905
YCR005C	YER024W	927
YCR005C	YGL062W	928
YCR005C	YGL252C	990
YCR005C	YIR031C	930
YCR005C	YJL200C	930
YCR005C	YKL085W	992
YCR005C	YLR304C	955
YCR005C	YML042W	920
YCR005C	YNL117W	925
YCR005C	YNR001C	999
YCR005C	YOL067C	944
YCR005C	YOL126C	927
YCR008W	YJL165C	937
YCR009C	YAL041W	998
YCR009C	YBL007C	955
YCR009C	YBR108W	999
YCR009C	YCL027W	990
YCR009C	YCR088W	997
YCR009C	YDR297W	978
YCR009C	YDR388W	999
YCR009C	YFL039C	998
YCR009C	YHR030C	968
YCR009C	YLL021W	926
YCR009C	YLR372W	986
YCR009C	YLR429W	985
YCR009C	YMR192W	995
YCR009C	YMR232W	999
YCR009C	YNL094W	919
YCR009C	YOR089C	963
YCR009C	YOR181W	981
YCR009C	YPL004C	968
YCR009C	YPL057C	980
YCR009C	YPL249C	995
YCR012W	YAL038W	964

YCR012W	YBR162C	962
YCR012W	YDL021W	900
YCR012W	YDL145C	930
YCR012W	YDR050C	998
YCR012W	YGR192C	999
YCR012W	YGR254W	999
YCR012W	YHR174W	999
YCR012W	YJL052W	996
YCR012W	YJR009C	992
YCR012W	YKL060C	999
YCR012W	YKL152C	915
YCR012W	YLR044C	997
YCR012W	YOL086C	987
YCR014C	YDL007W	970
YCR014C	YDL147W	970
YCR014C	YDR108W	966
YCR014C	YDR394W	970
YCR014C	YDR427W	970
YCR014C	YFR004W	970
YCR014C	YFR052W	970
YCR014C	YHR027C	970
YCR014C	YKL113C	928
YCR014C	YKL145W	970
YCR014C	YLR421C	970
YCR014C	YOR005C	912
YCR014C	YOR259C	970
YCR015C	YGR163W	941
YCR017C	YBL105C	917
YCR017C	YER167W	987
YCR017C	YGR229C	982
YCR017C	YJL010C	999
YCR018C	YDR087C	992
YCR018C-A	YBL107W-A	950
YCR018C-A	YGR122C-A	974
YCR020C-A	YBL026W	994
YCR020C-A	YDR378C	984
YCR020C-A	YEL053C	999
YCR020C-A	YER112W	984
YCR020C-A	YER146W	984
YCR020C-A	YFR037C	940
YCR020C-A	YGL173C	983
YCR020C-A	YJL124C	984
YCR020C-A	YJR022W	994
YCR020C-A	YLR438C-A	984

YCR020C-A	YNL147W	984
YCR020C-A	YPR051W	999
YCR020W-B	YBL006C	981
YCR020W-B	YCR052W	999
YCR020W-B	YDR303C	999
YCR020W-B	YFR037C	999
YCR020W-B	YGR056W	999
YCR020W-B	YGR275W	994
YCR020W-B	YHR056C	999
YCR020W-B	YIL126W	999
YCR020W-B	YKR008W	999
YCR020W-B	YLR033W	999
YCR020W-B	YLR321C	998
YCR020W-B	YLR357W	999
YCR020W-B	YML127W	999
YCR020W-B	YMR033W	998
YCR020W-B	YMR091C	999
YCR020W-B	YPR034W	998
YCR021C	YJL059W	978
YCR024C	YBL014C	998
YCR024C	YCL048W	998
YCR024C	YGR124W	906
YCR024C	YPR145W	905
YCR026C	YEL016C	906
YCR027C	YGR142W	979
YCR027C	YJR066W	931
YCR027C	YKL203C	991
YCR027C	YOL083W	978
YCR028C-A	YBR114W	980
YCR028C-A	YDR097C	958
YCR028C-A	YJL173C	998
YCR028C-A	YML032C	999
YCR028C-A	YMR234W	909
YCR028C-A	YNL312W	998
YCR030C	YBL047C	999
YCR031C	YAL035W	930
YCR031C	YBL027W	995
YCR031C	YBL072C	997
YCR031C	YBL087C	961
YCR031C	YBL092W	944
YCR031C	YBR031W	999
YCR031C	YBR048W	999
YCR031C	YBR079C	923
YCR031C	YBR084C-A	983

YCR031C	YBR118W	907
YCR031C	YBR181C	995
YCR031C	YBR189W	999
YCR031C	YBR191W	999
YCR031C	YCL059C	938
YCR031C	YDL014W	951
YCR031C	YDL061C	999
YCR031C	YDL075W	998
YCR031C	YDL081C	928
YCR031C	YDL082W	996
YCR031C	YDL083C	998
YCR031C	YDL130W	950
YCR031C	YDL136W	997
YCR031C	YDL148C	929
YCR031C	YDL191W	920
YCR031C	YDR012W	982
YCR031C	YDR025W	997
YCR031C	YDR041W	957
YCR031C	YDR064W	999
YCR031C	YDR382W	999
YCR031C	YDR385W	989
YCR031C	YDR418W	999
YCR031C	YDR447C	996
YCR031C	YDR450W	999
YCR031C	YDR471W	998
YCR031C	YDR500C	952
YCR031C	YEL054C	931
YCR031C	YER025W	945
YCR031C	YER056C-A	972
YCR031C	YER074W	995
YCR031C	YER102W	961
YCR031C	YER117W	974
YCR031C	YER131W	952
YCR031C	YER165W	946
YCR031C	YFL034C-A	919
YCR031C	YFR031C-A	996
YCR031C	YFR032C-A	937
YCR031C	YGL030W	999
YCR031C	YGL031C	996
YCR031C	YGL076C	998
YCR031C	YGL103W	999
YCR031C	YGL123W	999
YCR031C	YGL135W	996
YCR031C	YGL147C	996

YCR031C	YGL189C	921
YCR031C	YGR027C	988
YCR031C	YGR034W	996
YCR031C	YGR085C	998
YCR031C	YGR118W	997
YCR031C	YGR148C	971
YCR031C	YGR162W	914
YCR031C	YGR214W	999
YCR031C	YHL001W	999
YCR031C	YHL015W	999
YCR031C	YHL033C	999
YCR031C	YHR010W	971
YCR031C	YHR021C	935
YCR031C	YHR141C	996
YCR031C	YHR203C	998
YCR031C	YIL018W	997
YCR031C	YIL052C	993
YCR031C	YIL069C	918
YCR031C	YIL133C	998
YCR031C	YIL148W	953
YCR031C	YJL136C	959
YCR031C	YJL177W	999
YCR031C	YJL189W	936
YCR031C	YJL190C	999
YCR031C	YJL191W	999
YCR031C	YJR007W	901
YCR031C	YJR094W-A	976
YCR031C	YJR123W	999
YCR031C	YJR145C	930
YCR031C	YKL006W	997
YCR031C	YKL156W	975
YCR031C	YKL170W	965
YCR031C	YKL180W	999
YCR031C	YKR057W	932
YCR031C	YKR059W	945
YCR031C	YKR094C	909
YCR031C	YLL045C	996
YCR031C	YLR029C	999
YCR031C	YLR048W	995
YCR031C	YLR061W	995
YCR031C	YLR075W	998
YCR031C	YLR150W	956
YCR031C	YLR167W	933
YCR031C	YLR185W	955

YCR031C	YLR264W	965
YCR031C	YLR287C-A	995
YCR031C	YLR325C	914
YCR031C	YLR333C	929
YCR031C	YLR340W	999
YCR031C	YLR344W	965
YCR031C	YLR367W	959
YCR031C	YLR388W	997
YCR031C	YLR406C	925
YCR031C	YLR441C	996
YCR031C	YLR448W	999
YCR031C	YML024W	994
YCR031C	YML026C	979
YCR031C	YML063W	998
YCR031C	YML073C	996
YCR031C	YMR121C	925
YCR031C	YMR142C	997
YCR031C	YMR146C	914
YCR031C	YMR194W	999
YCR031C	YMR230W	943
YCR031C	YMR242C	996
YCR031C	YMR260C	945
YCR031C	YMR309C	903
YCR031C	YNL067W	978
YCR031C	YNL069C	998
YCR031C	YNL081C	994
YCR031C	YNL096C	996
YCR031C	YNL132W	936
YCR031C	YNL162W	928
YCR031C	YNL178W	999
YCR031C	YNL301C	997
YCR031C	YNL302C	998
YCR031C	YNR037C	985
YCR031C	YOL039W	935
YCR031C	YOL040C	994
YCR031C	YOL120C	909
YCR031C	YOL121C	927
YCR031C	YOL127W	999
YCR031C	YOR063W	999
YCR031C	YOR096W	999
YCR031C	YOR133W	925
YCR031C	YOR167C	939
YCR031C	YOR182C	908
YCR031C	YOR204W	929

YCR031C	YOR234C	999
YCR031C	YOR293W	966
YCR031C	YOR312C	997
YCR031C	YOR361C	920
YCR031C	YOR369C	942
YCR031C	YPL079W	957
YCR031C	YPL081W	998
YCR031C	YPL090C	994
YCR031C	YPL131W	980
YCR031C	YPL143W	999
YCR031C	YPL198W	930
YCR031C	YPL220W	964
YCR031C	YPL237W	925
YCR031C	YPL249C-A	997
YCR031C	YPR041W	905
YCR031C	YPR043W	941
YCR031C	YPR080W	907
YCR031C	YPR102C	988
YCR031C	YPR132W	954
YCR031C	YPR163C	910
YCR032W	YAL063C	939
YCR032W	YAR050W	914
YCR032W	YGR179C	935
YCR032W	YML097C	927
YCR032W	YPL069C	926
YCR033W	YAL013W	954
YCR033W	YBR095C	961
YCR033W	YBR103W	999
YCR033W	YDR155C	999
YCR033W	YDR207C	911
YCR033W	YDR310C	926
YCR033W	YGL194C	999
YCR033W	YIL084C	923
YCR033W	YIL112W	999
YCR033W	YJL168C	979
YCR033W	YKR029C	999
YCR033W	YMR263W	913
YCR033W	YMR273C	993
YCR033W	YNL097C	924
YCR033W	YOL004W	916
YCR033W	YOL068C	998
YCR033W	YPL181W	955
YCR034W	YBR159W	909
YCR034W	YDL015C	987

YCR034W	YLR372W	999
YCR034W	YMR202W	912
YCR035C	YDL111C	999
YCR035C	YDR280W	999
YCR035C	YGR095C	999
YCR035C	YGR158C	999
YCR035C	YGR195W	999
YCR035C	YHR069C	999
YCR035C	YHR081W	999
YCR035C	YNL189W	990
YCR035C	YNL232W	999
YCR035C	YNR024W	960
YCR035C	YOL021C	999
YCR035C	YOL142W	999
YCR035C	YOR001W	999
YCR035C	YOR076C	999
YCR035C	YPL211W	941
YCR036W	YBL068W	914
YCR036W	YBR117C	926
YCR036W	YER099C	914
YCR036W	YHL011C	915
YCR036W	YOR095C	961
YCR036W	YPR074C	935
YCR037C	YJL117W	998
YCR037C	YML123C	998
YCR038C	YGL130W	900
YCR038C	YGR041W	948
YCR038C	YGR152C	969
YCR038C	YIL140W	931
YCR038C	YKL092C	968
YCR038C	YLR353W	954
YCR041W	YCL065W	964
YCR042C	YBR198C	999
YCR042C	YDR145W	991
YCR042C	YDR167W	999
YCR042C	YER022W	909
YCR042C	YER148W	999
YCR042C	YGL112C	999
YCR042C	YGR005C	970
YCR042C	YGR104C	908
YCR042C	YGR186W	979
YCR042C	YGR274C	999
YCR042C	YKL081W	919
YCR042C	YML015C	999

YCR042C	YML098W	999
YCR042C	YML114C	999
YCR042C	YMR005W	999
YCR042C	YMR227C	999
YCR042C	YMR236W	996
YCR042C	YOL051W	984
YCR042C	YPL011C	999
YCR042C	YPL129W	999
YCR042C	YPR056W	984
YCR043C	YEL036C	907
YCR044C	YDR182W	987
YCR044C	YJL171C	913
YCR046C	YBL038W	992
YCR046C	YBR122C	992
YCR046C	YBR282W	991
YCR046C	YCR071C	999
YCR046C	YDR116C	999
YCR046C	YDR237W	992
YCR046C	YDR296W	992
YCR046C	YDR322W	993
YCR046C	YDR462W	992
YCR046C	YGR220C	997
YCR046C	YHR147C	991
YCR046C	YJL063C	992
YCR046C	YKL167C	992
YCR046C	YKR006C	992
YCR046C	YKR085C	992
YCR046C	YLR312W-A	991
YCR046C	YLR439W	993
YCR046C	YML025C	992
YCR046C	YMR024W	992
YCR046C	YMR193W	992
YCR046C	YNL005C	992
YCR046C	YNL177C	998
YCR046C	YNL252C	993
YCR046C	YNL284C	916
YCR046C	YNR022C	915
YCR046C	YOR150W	991
YCR046C	YPR100W	921
YCR048W	YLR242C	919
YCR048W	YNR019W	999
YCR052W	YBR245C	999
YCR052W	YCR020W-B	999
YCR052W	YDR303C	999

YCR052W	YER148W	923
YCR052W	YFR037C	999
YCR052W	YGR056W	999
YCR052W	YGR275W	999
YCR052W	YHR056C	996
YCR052W	YIL126W	999
YCR052W	YKR008W	999
YCR052W	YLR033W	999
YCR052W	YLR321C	999
YCR052W	YLR357W	999
YCR052W	YML127W	999
YCR052W	YMR033W	999
YCR052W	YMR091C	999
YCR052W	YPR034W	999
YCR053W	YEL046C	932
YCR053W	YER086W	919
YCR053W	YHR025W	994
YCR053W	YIL078W	937
YCR053W	YJR139C	923
YCR053W	YOR184W	933
YCR057C	YBL004W	999
YCR057C	YBR247C	994
YCR057C	YCL059C	998
YCR057C	YDL014W	999
YCR057C	YDL060W	918
YCR057C	YDL148C	999
YCR057C	YDR064W	980
YCR057C	YDR299W	982
YCR057C	YDR324C	999
YCR057C	YDR449C	999
YCR057C	YER082C	999
YCR057C	YGL011C	974
YCR057C	YGL120C	991
YCR057C	YGL171W	990
YCR057C	YGR090W	999
YCR057C	YGR128C	999
YCR057C	YGR145W	986
YCR057C	YHR148W	999
YCR057C	YHR169W	979
YCR057C	YHR196W	999
YCR057C	YJL010C	985
YCR057C	YJL069C	999
YCR057C	YJL109C	999
YCR057C	YJR002W	999

YCR057C	YJR145C	979
YCR057C	YKL099C	992
YCR057C	YKR060W	999
YCR057C	YLL011W	999
YCR057C	YLR003C	980
YCR057C	YLR129W	999
YCR057C	YLR175W	975
YCR057C	YLR186W	999
YCR057C	YLR197W	999
YCR057C	YLR222C	999
YCR057C	YLR409C	999
YCR057C	YMR093W	999
YCR057C	YMR128W	999
YCR057C	YMR229C	983
YCR057C	YMR290C	972
YCR057C	YNL075W	999
YCR057C	YNL132W	999
YCR057C	YNR054C	961
YCR057C	YOL010W	972
YCR057C	YOR078W	983
YCR057C	YOR145C	998
YCR057C	YOR310C	994
YCR057C	YPL012W	972
YCR057C	YPL126W	999
YCR057C	YPL217C	999
YCR057C	YPL266W	999
YCR057C	YPR112C	967
YCR057C	YPR137W	999
YCR057C	YPR144C	999
YCR060W	YDR190C	995
YCR060W	YHR034C	999
YCR060W	YOR310C	921
YCR060W	YPL235W	999
YCR060W	YPL240C	996
YCR063W	YDL030W	988
YCR063W	YDL209C	988
YCR063W	YER172C	903
YCR063W	YGL128C	968
YCR063W	YHR165C	999
YCR063W	YLL036C	999
YCR063W	YLR117C	970
YCR063W	YML049C	994
YCR063W	YMR213W	999
YCR063W	YMR240C	971

YCR063W	YNL138W-A	936
YCR063W	YPL151C	990
YCR063W	YPR094W	929
YCR063W	YPR101W	988
YCR065W	YBR109C	991
YCR065W	YDR113C	912
YCR065W	YJL030W	984
YCR065W	YOL012C	900
YCR066W	YBR088C	999
YCR066W	YDL064W	996
YCR066W	YDL074C	991
YCR066W	YDL102W	997
YCR066W	YDR092W	999
YCR066W	YDR409W	999
YCR066W	YDR419W	936
YCR066W	YDR440W	976
YCR066W	YER095W	985
YCR066W	YER162C	942
YCR066W	YER171W	955
YCR066W	YGL058W	999
YCR066W	YGL087C	994
YCR066W	YGR184C	962
YCR066W	YGR258C	976
YCR066W	YJL092W	999
YCR066W	YJR006W	984
YCR066W	YJR043C	915
YCR066W	YLR032W	999
YCR066W	YML032C	997
YCR066W	YML095C	952
YCR066W	YNL218W	993
YCR066W	YOR128C	906
YCR066W	YOR346W	928
YCR066W	YPL022W	999
YCR066W	YPL167C	998
YCR067C	YBL040C	914
YCR067C	YDR057W	923
YCR067C	YPL085W	989
YCR067C	YPL218W	998
YCR067C	YPR181C	983
YCR069W	YDL003W	962
YCR071C	YBL038W	972
YCR071C	YBR122C	972
YCR071C	YCR046C	999
YCR071C	YDR116C	996

YCR071C	YDR237W	974
YCR071C	YDR296W	992
YCR071C	YDR322W	988
YCR071C	YDR405W	988
YCR071C	YDR462W	972
YCR071C	YGR220C	998
YCR071C	YJL063C	987
YCR071C	YKL167C	972
YCR071C	YKR006C	972
YCR071C	YKR085C	972
YCR071C	YLR312W-A	988
YCR071C	YLR439W	989
YCR071C	YML025C	972
YCR071C	YMR024W	988
YCR071C	YMR193W	972
YCR071C	YNL005C	974
YCR071C	YNL252C	974
YCR071C	YNL284C	969
YCR071C	YOR150W	970
YCR072C	YDR101C	993
YCR072C	YER006W	999
YCR072C	YER126C	999
YCR072C	YGR245C	999
YCR072C	YHR197W	999
YCR072C	YKL009W	972
YCR072C	YLR074C	999
YCR072C	YLR106C	999
YCR072C	YLR325C	970
YCR072C	YNL182C	999
YCR072C	YNR053C	993
YCR072C	YPL093W	990
YCR072C	YPR016C	995
YCR073C	YBL041W	998
YCR073C	YHR027C	998
YCR073C	YJL128C	923
YCR073C	YLR006C	983
YCR073W-A	YAR014C	947
YCR073W-A	YDR248C	930
YCR073W-A	YER133W	997
YCR073W-A	YGR256W	945
YCR073W-A	YHR183W	946
YCR073W-A	YKL205W	954
YCR073W-A	YLR028C	998
YCR073W-A	YNL241C	981

YCR073W-A	YNR034W	922
YCR075C	YKR007W	977
YCR076C	YER094C	982
YCR077C	YBL026W	999
YCR077C	YBR055C	976
YCR077C	YDL160C	999
YCR077C	YDL165W	913
YCR077C	YDR378C	999
YCR077C	YDR473C	974
YCR077C	YER112W	999
YCR077C	YER146W	999
YCR077C	YER165W	998
YCR077C	YER172C	976
YCR077C	YGL173C	999
YCR077C	YGR091W	974
YCR077C	YHR165C	976
YCR077C	YJL124C	999
YCR077C	YJL140W	976
YCR077C	YJR022W	996
YCR077C	YKL173W	976
YCR077C	YLR147C	997
YCR077C	YLR275W	997
YCR077C	YLR438C-A	999
YCR077C	YMR186W	963
YCR077C	YMR268C	998
YCR077C	YNL088W	906
YCR077C	YNL118C	988
YCR077C	YNL145W	983
YCR077C	YNL147W	999
YCR077C	YOL149W	997
YCR077C	YOR308C	977
YCR077C	YPL240C	919
YCR077C	YPR082C	974
YCR077C	YPR178W	976
YCR079W	YGL180W	910
YCR079W	YOR323C	999
YCR081W	YBL093C	999
YCR081W	YBR193C	974
YCR081W	YBR253W	999
YCR081W	YDL005C	978
YCR081W	YDR308C	997
YCR081W	YDR443C	999
YCR081W	YER022W	998
YCR081W	YER111C	921

YCR081W	YGL025C	995
YCR081W	YGL127C	996
YCR081W	YGL151W	997
YCR081W	YGR104C	998
YCR081W	YHR041C	992
YCR081W	YHR058C	974
YCR081W	YLR071C	995
YCR081W	YLR182W	925
YCR081W	YML007W	974
YCR081W	YMR112C	974
YCR081W	YNL025C	999
YCR081W	YNL236W	998
YCR081W	YNR010W	985
YCR081W	YOL051W	998
YCR081W	YOL135C	999
YCR081W	YOR140W	979
YCR081W	YOR174W	974
YCR081W	YPL042C	999
YCR081W	YPR070W	997
YCR081W	YPR168W	983
YCR082W	YOR023C	970
YCR083W	YDR353W	979
YCR083W	YHR106W	997
YCR083W	YIL066C	900
YCR083W	YJL026W	901
YCR083W	YPL091W	987
YCR083W	YPR167C	909
YCR084C	YBR009C	930
YCR084C	YBR010W	976
YCR084C	YBR089C-A	972
YCR084C	YBR112C	999
YCR084C	YCL067C	999
YCR084C	YDR308C	997
YCR084C	YDR477W	993
YCR084C	YGL025C	981
YCR084C	YGL035C	986
YCR084C	YIL066C	930
YCR084C	YLR071C	935
YCR084C	YLR113W	980
YCR084C	YLR176C	992
YCR084C	YLR418C	961
YCR084C	YMR043W	973
YCR084C	YNL021W	993
YCR084C	YNL025C	990

YCR084C	YNL167C	996
YCR084C	YNL236W	971
YCR084C	YNL330C	998
YCR084C	YOL004W	947
YCR084C	YPL042C	994
YCR084C	YPL228W	974
YCR084C	YPR065W	959
YCR084C	YPR068C	908
YCR086W	YDL089W	980
YCR086W	YDR309C	905
YCR086W	YDR439W	999
YCR086W	YER106W	997
YCR086W	YFL008W	927
YCR086W	YHR061C	905
YCR086W	YHR152W	957
YCR086W	YKR010C	997
YCR086W	YOR264W	984
YCR087C-A	YDR510W	914
YCR088W	YBL007C	999
YCR088W	YCR009C	997
YCR088W	YDL029W	993
YCR088W	YDR129C	999
YCR088W	YDR388W	999
YCR088W	YFL039C	999
YCR088W	YFR024C-A	903
YCR088W	YGL206C	900
YCR088W	YHR016C	946
YCR088W	YIL002C	933
YCR088W	YIL095W	999
YCR088W	YIR003W	982
YCR088W	YJR065C	952
YCR088W	YLR429W	994
YCR088W	YNL020C	987
YCR088W	YNL094W	996
YCR088W	YNL106C	996
YCR088W	YNL138W	999
YCR088W	YNL243W	998
YCR088W	YNL298W	946
YCR088W	YOR181W	987
YCR088W	YOR284W	991
YCR088W	YOR329C	904
YCR088W	YPL004C	968
YCR089W	YIR019C	909
YCR089W	YNL325C	918

YCR092C	YBR088C	997
YCR092C	YDL059C	977
YCR092C	YDL102W	900
YCR092C	YDR097C	999
YCR092C	YMR167W	998
YCR092C	YNL082W	999
YCR092C	YOL090W	999
YCR092C	YOR033C	942
YCR092C	YPL022W	968
YCR093W	YAL021C	999
YCR093W	YDL165W	999
YCR093W	YDR103W	982
YCR093W	YDR392W	987
YCR093W	YER068W	999
YCR093W	YER148W	977
YCR093W	YGR134W	999
YCR093W	YHR084W	901
YCR093W	YHR206W	965
YCR093W	YIL038C	999
YCR093W	YLR055C	982
YCR093W	YNL288W	999
YCR093W	YNR052C	999
YCR093W	YOR212W	916
YCR093W	YPR072W	999
YCR094W	YAL026C	999
YCR094W	YDL192W	914
YCR094W	YDR484W	927
YCR094W	YER166W	970
YCR094W	YIL048W	912
YCR094W	YJL204C	973
YCR094W	YKL129C	900
YCR094W	YMR109W	900
YCR094W	YNL323W	995
YCR095C	YHL029C	999
YCR095C	YNL032W	999
YCR095C	YNL056W	929
YCR095C	YNL099C	999
YCR096C	YCR097W	999
YCR096C	YMR043W	999
YCR097W	YCL066W	958
YCR097W	YCL067C	999
YCR097W	YCR096C	999
YDL002C	YBR245C	957
YDL002C	YDR190C	996

YDL002C	YER092W	999
YDL002C	YFL013C	999
YDL002C	YFL039C	998
YDL002C	YGL133W	980
YDL002C	YGL150C	999
YDL002C	YJL081C	999
YDL002C	YLR052W	999
YDL002C	YLR357W	953
YDL002C	YNL059C	997
YDL002C	YNL215W	994
YDL002C	YOR141C	999
YDL002C	YOR304W	990
YDL002C	YPL129W	991
YDL002C	YPL235W	998
YDL003W	YCR069W	962
YDL003W	YDR014W	932
YDL003W	YDR113C	993
YDL003W	YDR180W	987
YDL003W	YER147C	991
YDL003W	YFL008W	999
YDL003W	YFR027W	948
YDL003W	YGR098C	999
YDL003W	YIL026C	999
YDL003W	YJL074C	999
YDL003W	YML034W	908
YDL003W	YMR001C	999
YDL003W	YMR076C	940
YDL003W	YOR195W	937
YDL003W	YPR007C	959
YDL004W	Q0085	901
YDL004W	YBL099W	999
YDL004W	YBR011C	911
YDL004W	YBR039W	999
YDL004W	YDL130W-A	910
YDL004W	YDL181W	922
YDL004W	YDR298C	995
YDL004W	YDR377W	906
YDL004W	YJR121W	999
YDL004W	YKL016C	949
YDL004W	YLR295C	908
YDL004W	YML081C-A	925
YDL004W	YMR267W	903
YDL004W	YPL078C	999
YDL004W	YPL271W	997

YDL005C	YBL093C	999
YDL005C	YBR193C	999
YDL005C	YBR253W	999
YDL005C	YCR081W	978
YDL005C	YDL140C	937
YDL005C	YDR308C	999
YDL005C	YDR443C	999
YDL005C	YER022W	999
YDL005C	YGL025C	999
YDL005C	YGL127C	999
YDL005C	YGL151W	999
YDL005C	YGR104C	999
YDL005C	YHR041C	999
YDL005C	YHR058C	999
YDL005C	YIL021W	975
YDL005C	YJL140W	910
YDL005C	YKL028W	984
YDL005C	YLR071C	999
YDL005C	YMR112C	999
YDL005C	YNL236W	999
YDL005C	YNR010W	999
YDL005C	YOL051W	999
YDL005C	YOL135C	999
YDL005C	YOR151C	923
YDL005C	YOR174W	999
YDL005C	YPL042C	997
YDL005C	YPL129W	965
YDL005C	YPR070W	999
YDL005C	YPR168W	999
YDL006W	YDR162C	999
YDL006W	YDR167W	906
YDL006W	YIL147C	976
YDL006W	YJL128C	990
YDL006W	YLR113W	998
YDL006W	YNR031C	973
YDL006W	YOR208W	998
YDL007W	YBL041W	905
YDL007W	YBR272C	971
YDL007W	YCR014C	970
YDL007W	YDL020C	962
YDL007W	YDL097C	999
YDL007W	YDL147W	999
YDL007W	YDR363W-A	999
YDL007W	YDR394W	999

YDL007W	YDR427W	999
YDL007W	YEL037C	991
YDL007W	YER012W	999
YDL007W	YER021W	999
YDL007W	YER094C	912
YDL007W	YFR004W	999
YDL007W	YFR010W	999
YDL007W	YFR050C	911
YDL007W	YFR052W	999
YDL007W	YGL004C	967
YDL007W	YGL048C	999
YDL007W	YGL141W	983
YDL007W	YGR232W	998
YDL007W	YHL030W	999
YDL007W	YHR027C	999
YDL007W	YHR200W	999
YDL007W	YIL007C	903
YDL007W	YIL075C	999
YDL007W	YKL145W	999
YDL007W	YLL039C	988
YDL007W	YLR421C	999
YDL007W	YMR191W	987
YDL007W	YMR314W	997
YDL007W	YOR117W	999
YDL007W	YOR157C	905
YDL007W	YOR259C	999
YDL007W	YOR261C	999
YDL007W	YPR103W	908
YDL007W	YPR108W	999
YDL008W	YBL084C	999
YDL008W	YBR082C	998
YDL008W	YDR118W	999
YDL008W	YDR260C	999
YDL008W	YFR036W	999
YDL008W	YGL116W	921
YDL008W	YGL240W	999
YDL008W	YHR166C	999
YDL008W	YIR025W	982
YDL008W	YKL022C	999
YDL008W	YLR102C	999
YDL008W	YLR127C	999
YDL008W	YMR001C	910
YDL008W	YNL172W	999
YDL008W	YOR249C	999

YDL013W	YDL042C	971
YDL013W	YDL064W	972
YDL013W	YDR320C	954
YDL013W	YDR510W	999
YDL013W	YER116C	999
YDL013W	YFR004W	970
YDL013W	YGR140W	929
YDL013W	YHR156C	904
YDL013W	YKL145W	970
YDL013W	YLR006C	929
YDL013W	YLR234W	926
YDL013W	YLR421C	970
YDL013W	YML032C	971
YDL013W	YMR190C	999
YDL013W	YOR156C	997
YDL013W	YOR259C	970
YDL013W	YPL020C	994
YDL013W	YPL024W	979
YDL013W	YPL082C	904
YDL014W	YAL035W	996
YDL014W	YBL004W	995
YDL014W	YBL072C	964
YDL014W	YBR034C	919
YDL014W	YBR048W	938
YDL014W	YBR142W	923
YDL014W	YBR189W	958
YDL014W	YBR247C	999
YDL014W	YCL011C	938
YDL014W	YCL037C	987
YDL014W	YCL059C	999
YDL014W	YCR031C	951
YDL014W	YCR057C	999
YDL014W	YDL060W	954
YDL014W	YDL082W	949
YDL014W	YDL083C	947
YDL014W	YDL148C	998
YDL014W	YDL208W	991
YDL014W	YDR025W	956
YDL014W	YDR060W	991
YDL014W	YDR064W	968
YDL014W	YDR117C	940
YDL014W	YDR299W	998
YDL014W	YDR324C	995
YDL014W	YDR365C	912

YDL014W	YDR447C	956
YDL014W	YDR449C	997
YDL014W	YDR496C	970
YDL014W	YEL026W	999
YDL014W	YER082C	999
YDL014W	YER102W	936
YDL014W	YGL011C	915
YDL014W	YGL030W	954
YDL014W	YGL078C	971
YDL014W	YGL120C	999
YDL014W	YGL173C	940
YDL014W	YGR085C	948
YDL014W	YGR090W	999
YDL014W	YGR103W	973
YDL014W	YGR128C	995
YDL014W	YGR145W	995
YDL014W	YGR159C	984
YDL014W	YGR162W	936
YDL014W	YHL034C	929
YDL014W	YHR089C	950
YDL014W	YHR148W	998
YDL014W	YHR196W	972
YDL014W	YHR203C	936
YDL014W	YIL018W	937
YDL014W	YIL133C	937
YDL014W	YJL010C	983
YDL014W	YJL033W	999
YDL014W	YJL069C	926
YDL014W	YJL109C	998
YDL014W	YJL190C	946
YDL014W	YJR002W	999
YDL014W	YJR145C	964
YDL014W	YKR060W	997
YDL014W	YLL008W	929
YDL014W	YLL011W	999
YDL014W	YLL045C	962
YDL014W	YLR003C	942
YDL014W	YLR029C	950
YDL014W	YLR075W	957
YDL014W	YLR129W	995
YDL014W	YLR175W	999
YDL014W	YLR186W	995
YDL014W	YLR197W	999
YDL014W	YLR222C	994

YDL014W	YLR409C	997
YDL014W	YML063W	955
YDL014W	YMR093W	995
YDL014W	YMR128W	994
YDL014W	YMR229C	998
YDL014W	YMR239C	916
YDL014W	YMR290C	997
YDL014W	YMR309C	977
YDL014W	YNL061W	995
YDL014W	YNL069C	938
YDL014W	YNL075W	979
YDL014W	YNL132W	995
YDL014W	YNL308C	998
YDL014W	YNR054C	919
YDL014W	YOL010W	984
YDL014W	YOL076W	940
YDL014W	YOL077C	969
YDL014W	YOR063W	961
YDL014W	YOR078W	932
YDL014W	YOR096W	970
YDL014W	YOR145C	913
YDL014W	YOR206W	997
YDL014W	YOR310C	999
YDL014W	YPL012W	995
YDL014W	YPL043W	984
YDL014W	YPL126W	995
YDL014W	YPL131W	960
YDL014W	YPL198W	941
YDL014W	YPL217C	995
YDL014W	YPL266W	937
YDL014W	YPR112C	935
YDL014W	YPR137W	999
YDL014W	YPR144C	998
YDL015C	YBR036C	980
YDL015C	YCR034W	987
YDL015C	YHR195W	992
YDL015C	YLR372W	990
YDL017W	YBL023C	999
YDL017W	YBL035C	918
YDL017W	YBR060C	999
YDL017W	YBR160W	999
YDL017W	YBR202W	989
YDL017W	YDL132W	976
YDL017W	YDR052C	999

YDL017W	YEL032W	999
YDL017W	YGL201C	998
YDL017W	YIL150C	955
YDL017W	YJL194W	971
YDL017W	YLR103C	981
YDL017W	YLR274W	999
YDL017W	YML065W	916
YDL017W	YNL102W	986
YDL017W	YNL261W	911
YDL017W	YNL262W	918
YDL017W	YPL153C	997
YDL017W	YPR019W	999
YDL017W	YPR120C	909
YDL018C	YHR110W	995
YDL018C	YML012W	989
YDL019C	YAR042W	910
YDL019C	YER120W	975
YDL019C	YMR109W	913
YDL020C	YAR003W	965
YDL020C	YBL041W	998
YDL020C	YBR175W	965
YDL020C	YDL007W	962
YDL020C	YDL074C	912
YDL020C	YGL058W	985
YDL020C	YGR135W	950
YDL020C	YHR200W	903
YDL020C	YIL075C	996
YDL020C	YLR021W	969
YDL020C	YLR024C	998
YDL020C	YMR100W	983
YDL020C	YPL144W	970
YDL020C	YPR103W	999
YDL021W	YCR012W	900
YDL022W	YER062C	972
YDL022W	YIL053W	961
YDL022W	YIL155C	989
YDL022W	YLL043W	951
YDL022W	YLR336C	907
YDL022W	YML070W	934
YDL022W	YNR031C	976
YDL022W	YOL059W	999
YDL022W	YPL061W	950
YDL025C	YDL097C	940
YDL025C	YHR027C	927

YDL028C	YBL084C	929
YDL028C	YDR168W	998
YDL028C	YEL061C	979
YDL028C	YGL086W	984
YDL028C	YGL116W	956
YDL028C	YGR113W	999
YDL028C	YGR188C	999
YDL028C	YIL106W	987
YDL028C	YIL144W	963
YDL028C	YJL030W	998
YDL028C	YKL022C	929
YDL028C	YKL042W	988
YDL028C	YMR055C	984
YDL028C	YOR026W	978
YDL028C	YPL209C	984
YDL029W	YBR234C	999
YDL029W	YCR088W	993
YDL029W	YER125W	958
YDL029W	YIL062C	999
YDL029W	YIL095W	971
YDL029W	YIR006C	998
YDL029W	YJR064W	935
YDL029W	YJR065C	999
YDL029W	YKL013C	999
YDL029W	YKL129C	973
YDL029W	YLR370C	999
YDL029W	YLR429W	994
YDL029W	YMR109W	998
YDL029W	YNL084C	908
YDL029W	YNL298W	935
YDL029W	YNR035C	999
YDL029W	YOR035C	965
YDL029W	YOR098C	927
YDL029W	YOR181W	999
YDL030W	YBR055C	993
YDL030W	YBR152W	921
YDL030W	YCR063W	988
YDL030W	YDL043C	999
YDL030W	YDL098C	976
YDL030W	YDL209C	974
YDL030W	YDR416W	974
YDL030W	YDR473C	981
YDL030W	YER029C	997
YDL030W	YER172C	993

YDL030W	YFL017W-A	982
YDL030W	YGL128C	974
YDL030W	YGR074W	999
YDL030W	YGR091W	976
YDL030W	YHR165C	998
YDL030W	YIR005W	977
YDL030W	YIR009W	999
YDL030W	YJL203W	999
YDL030W	YKL173W	978
YDL030W	YLL036C	998
YDL030W	YLR117C	976
YDL030W	YLR147C	996
YDL030W	YLR275W	992
YDL030W	YLR424W	974
YDL030W	YML046W	976
YDL030W	YML049C	999
YDL030W	YMR125W	994
YDL030W	YMR240C	999
YDL030W	YMR288W	999
YDL030W	YNL286W	962
YDL030W	YOL133W	962
YDL030W	YOR159C	977
YDL030W	YOR308C	976
YDL030W	YOR319W	999
YDL030W	YPL151C	976
YDL030W	YPL213W	999
YDL030W	YPR082C	976
YDL030W	YPR101W	974
YDL030W	YPR178W	978
YDL030W	YPR182W	975
YDL031W	YBR142W	985
YDL031W	YCL054W	996
YDL031W	YDR060W	984
YDL031W	YDR087C	998
YDL031W	YDR101C	989
YDL031W	YDR496C	992
YDL031W	YER006W	999
YDL031W	YER126C	983
YDL031W	YFL002C	996
YDL031W	YFR001W	987
YDL031W	YGL111W	982
YDL031W	YGR103W	999
YDL031W	YHR052W	996
YDL031W	YHR066W	974

YDL031W	YHR088W	981
YDL031W	YHR197W	984
YDL031W	YJL050W	918
YDL031W	YKL009W	985
YDL031W	YKL172W	996
YDL031W	YKR081C	999
YDL031W	YLL008W	996
YDL031W	YLL034C	986
YDL031W	YLL045C	942
YDL031W	YLR002C	991
YDL031W	YLR029C	931
YDL031W	YLR276C	998
YDL031W	YLR449W	980
YDL031W	YMR049C	981
YDL031W	YMR290C	998
YDL031W	YNL002C	993
YDL031W	YNL061W	999
YDL031W	YNL110C	999
YDL031W	YNL182C	981
YDL031W	YOL077C	987
YDL031W	YOL127W	944
YDL031W	YOR063W	937
YDL031W	YOR206W	988
YDL031W	YOR272W	992
YDL031W	YPL043W	994
YDL031W	YPL093W	979
YDL031W	YPL131W	956
YDL031W	YPL198W	939
YDL031W	YPL211W	989
YDL031W	YPR016C	999
YDL033C	YCL017C	902
YDL033C	YJL168C	952
YDL035C	YAL056W	908
YDL035C	YCL040W	967
YDL035C	YER020W	999
YDL035C	YFR053C	967
YDL035C	YGL253W	967
YDL035C	YHR205W	906
YDL035C	YNL098C	991
YDL035C	YOR371C	905
YDL040C	YAL009W	922
YDL040C	YHR013C	999
YDL040C	YKR101W	990
YDL040C	YMR116C	956

YDL040C	YOR253W	990
YDL040C	YPL198W	972
YDL042C	YBR009C	999
YDL042C	YBR010W	976
YDL042C	YDL013W	971
YDL042C	YDR110W	999
YDL042C	YDR227W	999
YDL042C	YDR285W	986
YDL042C	YDR310C	999
YDL042C	YDR363W	980
YDL042C	YDR400W	911
YDL042C	YDR440W	991
YDL042C	YFR028C	999
YDL042C	YGL037C	971
YDL042C	YGR010W	992
YDL042C	YGR192C	969
YDL042C	YGR282C	941
YDL042C	YHR119W	996
YDL042C	YIL150C	996
YDL042C	YJL076W	999
YDL042C	YJL194W	976
YDL042C	YKR101W	999
YDL042C	YLR209C	916
YDL042C	YLR328W	991
YDL042C	YLR442C	999
YDL042C	YML032C	909
YDL042C	YML109W	939
YDL042C	YMR127C	906
YDL042C	YNL030W	999
YDL042C	YNL031C	976
YDL042C	YNL129W	919
YDL042C	YNL216W	958
YDL042C	YNL330C	994
YDL042C	YOL017W	978
YDL042C	YOL068C	996
YDL042C	YOR244W	908
YDL043C	YBR055C	985
YDL043C	YBR069C	925
YDL043C	YBR152W	934
YDL043C	YDL030W	999
YDL043C	YER029C	996
YDL043C	YHR165C	981
YDL043C	YJL203W	999
YDL043C	YKL074C	928

YDL043C	YKL173W	974
YDL043C	YLR147C	990
YDL043C	YLR275W	991
YDL043C	YML049C	993
YDL043C	YMR125W	975
YDL043C	YMR240C	999
YDL043C	YMR288W	994
YDL043C	YNL210W	967
YDL043C	YNL286W	912
YDL043C	YOR319W	992
YDL043C	YPL213W	998
YDL043C	YPR178W	987
YDL044C	Q0045	952
YDL044C	Q0105	941
YDL044C	YFL036W	930
YDL044C	YLR067C	939
YDL044C	YLR139C	999
YDL044C	YMR257C	910
YDL045C	YDR236C	971
YDL045W-A	Q0140	962
YDL045W-A	YBL090W	986
YDL045W-A	YBR251W	947
YDL045W-A	YDR175C	986
YDL045W-A	YIL093C	986
YDL045W-A	YJR113C	986
YDL045W-A	YNL306W	986
YDL047W	YAL040C	993
YDL047W	YDL134C	996
YDL047W	YDL188C	972
YDL047W	YDL219W	927
YDL047W	YDR293C	997
YDL047W	YDR477W	943
YDL047W	YER040W	997
YDL047W	YER155C	993
YDL047W	YFR040W	999
YDL047W	YGL197W	926
YDL047W	YGR161C	999
YDL047W	YIL153W	999
YDL047W	YJL098W	999
YDL047W	YJL141C	960
YDL047W	YJR066W	900
YDL047W	YKL110C	941
YDL047W	YKL203C	972
YDL047W	YKR028W	999

YDL047W	YKR072C	998
YDL047W	YLR138W	987
YDL047W	YLR248W	940
YDL047W	YLR310C	933
YDL047W	YLR384C	970
YDL047W	YMR028W	999
YDL047W	YNL229C	965
YDL047W	YOR048C	946
YDL047W	YOR054C	981
YDL047W	YOR267C	920
YDL047W	YPL152W	986
YDL049C	YJL174W	925
YDL051W	YDL092W	998
YDL051W	YDR120C	924
YDL051W	YDR378C	919
YDL051W	YDR395W	996
YDL051W	YER146W	948
YDL051W	YGR074W	906
YDL051W	YGR162W	994
YDL051W	YGR276C	904
YDL051W	YJL125C	929
YDL051W	YJR022W	953
YDL051W	YKL122C	994
YDL051W	YML105C	995
YDL051W	YNL147W	913
YDL051W	YNL262W	960
YDL051W	YPL210C	999
YDL051W	YPL243W	999
YDL051W	YPR088C	999
YDL052C	YBR041W	938
YDL052C	YDL093W	919
YDL052C	YDR170C	926
YDL052C	YFR009W	963
YDL052C	YIL124W	908
YDL052C	YLR071C	924
YDL052C	YNL138W	901
YDL052C	YOR175C	999
YDL053C	YGR178C	997
YDL053C	YHR121W	998
YDL055C	YBR110W	995
YDL055C	YDR510W	921
YDL055C	YFL045C	973
YDL055C	YGL065C	902
YDL055C	YMR278W	914

YDL055C	YNL149C	980
YDL055C	YOR360C	909
YDL055C	YPR183W	997
YDL056W	YBR160W	999
YDL056W	YBR279W	945
YDL056W	YER111C	999
YDL056W	YHR206W	925
YDL056W	YIL101C	925
YDL056W	YLR182W	999
YDL056W	YOR083W	939
YDL056W	YPR119W	960
YDL058W	YDL099W	901
YDL058W	YDR189W	998
YDL058W	YDR517W	943
YDL058W	YFL038C	998
YDL058W	YGR120C	942
YDL058W	YIL004C	993
YDL058W	YKL196C	930
YDL058W	YLR078C	943
YDL058W	YLR268W	992
YDL058W	YOR216C	999
YDL059C	YBR073W	965
YDL059C	YCR092C	977
YDL059C	YDR004W	986
YDL059C	YDR076W	971
YDL059C	YDR369C	969
YDL059C	YER095W	999
YDL059C	YGL163C	971
YDL059C	YJL092W	946
YDL059C	YKL113C	994
YDL059C	YML032C	999
YDL059C	YMR224C	970
YDL059C	YNL250W	979
YDL059C	YNL312W	925
YDL059C	YPL022W	988
YDL059C	YPR164W	913
YDL060W	YBL056W	945
YDL060W	YBL072C	978
YDL060W	YBR048W	981
YDL060W	YBR189W	993
YDL060W	YBR247C	999
YDL060W	YCL059C	914
YDL060W	YCR057C	918
YDL060W	YDL014W	954

YDL060W	YDL148C	968
YDL060W	YDL153C	914
YDL060W	YDR064W	937
YDL060W	YDR299W	935
YDL060W	YDR324C	923
YDL060W	YDR447C	982
YDL060W	YDR449C	910
YDL060W	YER006W	929
YDL060W	YER074W	981
YDL060W	YER102W	978
YDL060W	YGL171W	909
YDL060W	YGR054W	972
YDL060W	YGR081C	999
YDL060W	YGR090W	960
YDL060W	YGR145W	921
YDL060W	YHR148W	917
YDL060W	YHR169W	912
YDL060W	YIL069C	985
YDL060W	YJL010C	986
YDL060W	YJR002W	921
YDL060W	YJR145C	982
YDL060W	YKL143W	999
YDL060W	YLL011W	905
YDL060W	YLR129W	914
YDL060W	YLR186W	903
YDL060W	YLR197W	906
YDL060W	YLR222C	914
YDL060W	YLR409C	916
YDL060W	YML093W	910
YDL060W	YMR093W	954
YDL060W	YMR290C	903
YDL060W	YNL075W	912
YDL060W	YNL132W	923
YDL060W	YNL207W	999
YDL060W	YOL010W	946
YDL060W	YOR056C	999
YDL060W	YOR096W	935
YDL060W	YOR145C	999
YDL060W	YPL012W	999
YDL060W	YPL126W	915
YDL060W	YPL204W	997
YDL060W	YPL266W	999
YDL060W	YPR112C	931
YDL060W	YPR137W	914

YDL060W	YPR144C	914
YDL061C	YAL035W	921
YDL061C	YBL027W	948
YDL061C	YBL072C	902
YDL061C	YBL087C	960
YDL061C	YBL092W	970
YDL061C	YBR031W	969
YDL061C	YBR048W	996
YDL061C	YBR079C	918
YDL061C	YBR084C-A	974
YDL061C	YBR189W	991
YDL061C	YBR191W	999
YDL061C	YCR031C	999
YDL061C	YDL075W	997
YDL061C	YDL081C	910
YDL061C	YDL082W	917
YDL061C	YDL083C	912
YDL061C	YDL130W	941
YDL061C	YDL136W	955
YDL061C	YDL191W	906
YDL061C	YDR012W	980
YDL061C	YDR025W	959
YDL061C	YDR064W	999
YDL061C	YDR382W	999
YDL061C	YDR385W	989
YDL061C	YDR418W	982
YDL061C	YDR447C	911
YDL061C	YDR450W	990
YDL061C	YDR471W	969
YDL061C	YDR500C	978
YDL061C	YEL054C	922
YDL061C	YER025W	953
YDL061C	YER056C-A	996
YDL061C	YER074W	910
YDL061C	YER102W	964
YDL061C	YER117W	986
YDL061C	YER131W	968
YDL061C	YER165W	931
YDL061C	YFL034C-A	911
YDL061C	YFR031C-A	935
YDL061C	YFR032C-A	910
YDL061C	YGL030W	998
YDL061C	YGL031C	949
YDL061C	YGL076C	945

YDL061C	YGL103W	994
YDL061C	YGL123W	999
YDL061C	YGL135W	910
YDL061C	YGL147C	950
YDL061C	YGL189C	912
YDL061C	YGR027C	994
YDL061C	YGR034W	962
YDL061C	YGR085C	985
YDL061C	YGR118W	999
YDL061C	YGR148C	989
YDL061C	YGR214W	997
YDL061C	YHL001W	996
YDL061C	YHL015W	999
YDL061C	YHL033C	995
YDL061C	YHR010W	973
YDL061C	YHR021C	912
YDL061C	YHR141C	913
YDL061C	YHR203C	985
YDL061C	YIL018W	935
YDL061C	YIL052C	999
YDL061C	YIL069C	910
YDL061C	YIL133C	913
YDL061C	YIL148W	965
YDL061C	YJL136C	960
YDL061C	YJL177W	984
YDL061C	YJL189W	944
YDL061C	YJL190C	995
YDL061C	YJL191W	982
YDL061C	YJR094W-A	981
YDL061C	YJR123W	999
YDL061C	YJR145C	963
YDL061C	YKL006W	997
YDL061C	YKL156W	980
YDL061C	YKL180W	998
YDL061C	YKR057W	915
YDL061C	YKR059W	952
YDL061C	YLL045C	923
YDL061C	YLR029C	965
YDL061C	YLR048W	995
YDL061C	YLR061W	998
YDL061C	YLR075W	980
YDL061C	YLR167W	960
YDL061C	YLR185W	961
YDL061C	YLR264W	995

YDL061C	YLR287C-A	900
YDL061C	YLR325C	911
YDL061C	YLR333C	922
YDL061C	YLR340W	978
YDL061C	YLR344W	987
YDL061C	YLR367W	995
YDL061C	YLR388W	999
YDL061C	YLR406C	903
YDL061C	YLR441C	916
YDL061C	YLR448W	979
YDL061C	YML024W	997
YDL061C	YML026C	906
YDL061C	YML063W	973
YDL061C	YML073C	907
YDL061C	YMR121C	911
YDL061C	YMR142C	922
YDL061C	YMR146C	900
YDL061C	YMR194W	994
YDL061C	YMR230W	979
YDL061C	YMR242C	911
YDL061C	YMR260C	903
YDL061C	YNL067W	986
YDL061C	YNL069C	913
YDL061C	YNL096C	915
YDL061C	YNL162W	913
YDL061C	YNL178W	999
YDL061C	YNL301C	953
YDL061C	YNL302C	912
YDL061C	YNR037C	910
YDL061C	YOL039W	923
YDL061C	YOL040C	996
YDL061C	YOL121C	912
YDL061C	YOL127W	996
YDL061C	YOR063W	988
YDL061C	YOR096W	976
YDL061C	YOR133W	921
YDL061C	YOR167C	967
YDL061C	YOR182C	900
YDL061C	YOR234C	970
YDL061C	YOR293W	968
YDL061C	YOR312C	922
YDL061C	YOR361C	914
YDL061C	YOR369C	925
YDL061C	YPL037C	968

YDL061C	YPL079W	958
YDL061C	YPL081W	993
YDL061C	YPL131W	992
YDL061C	YPL143W	978
YDL061C	YPL198W	912
YDL061C	YPL220W	966
YDL061C	YPL237W	921
YDL061C	YPL249C-A	939
YDL061C	YPR043W	941
YDL061C	YPR102C	990
YDL061C	YPR132W	964
YDL064W	YBR088C	996
YDL064W	YCR066W	996
YDL064W	YDL013W	972
YDL064W	YDR409W	999
YDL064W	YDR510W	999
YDL064W	YER016W	968
YDL064W	YER095W	996
YDL064W	YER116C	973
YDL064W	YGR140W	929
YDL064W	YIL031W	900
YDL064W	YJL092W	986
YDL064W	YLR032W	985
YDL064W	YML032C	971
YDL064W	YMR168C	972
YDL064W	YOL006C	980
YDL064W	YOR156C	997
YDL064W	YPL269W	966
YDL065C	YBR168W	986
YDL065C	YDR244W	904
YDL065C	YDR265W	989
YDL065C	YDR329C	999
YDL065C	YGL153W	979
YDL065C	YJL210W	934
YDL065C	YKR001C	974
YDL065C	YLR191W	999
YDL065C	YLR324W	986
YDL065C	YMR026C	919
YDL065C	YNL214W	990
YDL065C	YNL307C	938
YDL065C	YOL147C	999
YDL065C	YPL112C	998
YDL066W	YJL034W	958
YDL066W	YJL200C	904

YDL066W	YLR174W	904
YDL066W	YLR304C	909
YDL066W	YNL009W	903
YDL066W	YNL037C	984
YDL066W	YOR136W	984
YDL067C	Q0045	984
YDL067C	Q0250	978
YDL067C	Q0275	978
YDL067C	YGL187C	999
YDL067C	YGL191W	978
YDL067C	YHR051W	999
YDL067C	YIL111W	978
YDL067C	YLR038C	990
YDL067C	YLR395C	978
YDL067C	YMR256C	990
YDL067C	YNL052W	998
YDL070W	YER133W	955
YDL070W	YLR399C	999
YDL070W	YMR227C	928
YDL072C	YER120W	923
YDL072C	YOR254C	921
YDL074C	YAL011W	969
YDL074C	YBL045C	990
YDL074C	YBR231C	964
YDL074C	YCR066W	991
YDL074C	YDL020C	912
YDL074C	YDR334W	984
YDL074C	YDR359C	931
YDL074C	YDR485C	967
YDL074C	YFR010W	901
YDL074C	YGL058W	999
YDL074C	YGR200C	942
YDL074C	YHR200W	913
YDL074C	YJL168C	986
YDL074C	YKL139W	952
YDL074C	YKL213C	909
YDL074C	YLR032W	989
YDL074C	YLR085C	978
YDL074C	YLR384C	942
YDL074C	YML041C	978
YDL074C	YMR312W	942
YDL074C	YOL012C	999
YDL074C	YPL055C	999
YDL074C	YPL086C	952

YDL074C	YPL101W	942
YDL075W	YAL003W	954
YDL075W	YAL035W	930
YDL075W	YBL027W	999
YDL075W	YBL072C	919
YDL075W	YBL087C	990
YDL075W	YBL092W	999
YDL075W	YBR031W	997
YDL075W	YBR048W	924
YDL075W	YBR084C-A	995
YDL075W	YBR118W	905
YDL075W	YBR181C	997
YDL075W	YBR189W	976
YDL075W	YBR191W	999
YDL075W	YCR031C	998
YDL075W	YDL061C	997
YDL075W	YDL081C	937
YDL075W	YDL082W	996
YDL075W	YDL083C	966
YDL075W	YDL130W	954
YDL075W	YDL136W	999
YDL075W	YDL191W	913
YDL075W	YDL192W	998
YDL075W	YDR012W	999
YDL075W	YDR025W	900
YDL075W	YDR064W	999
YDL075W	YDR101C	983
YDL075W	YDR382W	999
YDL075W	YDR385W	909
YDL075W	YDR418W	999
YDL075W	YDR447C	996
YDL075W	YDR450W	920
YDL075W	YDR471W	998
YDL075W	YDR500C	996
YDL075W	YEL054C	924
YDL075W	YER006W	931
YDL075W	YER056C-A	999
YDL075W	YER074W	995
YDL075W	YER102W	992
YDL075W	YER117W	996
YDL075W	YER131W	943
YDL075W	YFL034C-A	918
YDL075W	YFR031C-A	999
YDL075W	YFR032C-A	996

YDL075W	YGL014W	948
YDL075W	YGL030W	999
YDL075W	YGL031C	992
YDL075W	YGL076C	999
YDL075W	YGL103W	999
YDL075W	YGL123W	999
YDL075W	YGL135W	999
YDL075W	YGL147C	999
YDL075W	YGL189C	909
YDL075W	YGR027C	974
YDL075W	YGR034W	996
YDL075W	YGR085C	999
YDL075W	YGR103W	930
YDL075W	YGR118W	999
YDL075W	YGR148C	993
YDL075W	YGR214W	976
YDL075W	YHL001W	999
YDL075W	YHL015W	989
YDL075W	YHL033C	999
YDL075W	YHR010W	979
YDL075W	YHR021C	922
YDL075W	YHR141C	999
YDL075W	YHR203C	993
YDL075W	YIL018W	996
YDL075W	YIL052C	999
YDL075W	YIL069C	902
YDL075W	YIL133C	999
YDL075W	YIL148W	981
YDL075W	YJL136C	968
YDL075W	YJL177W	999
YDL075W	YJL189W	981
YDL075W	YJL190C	996
YDL075W	YJL191W	972
YDL075W	YJR094W-A	999
YDL075W	YJR123W	999
YDL075W	YJR145C	928
YDL075W	YKL006W	998
YDL075W	YKL156W	977
YDL075W	YKL180W	999
YDL075W	YKR057W	927
YDL075W	YKR094C	907
YDL075W	YLL045C	996
YDL075W	YLR029C	999
YDL075W	YLR048W	999

YDL075W	YLR061W	999
YDL075W	YLR075W	999
YDL075W	YLR167W	975
YDL075W	YLR185W	999
YDL075W	YLR249W	923
YDL075W	YLR264W	980
YDL075W	YLR287C-A	995
YDL075W	YLR333C	919
YDL075W	YLR340W	999
YDL075W	YLR344W	999
YDL075W	YLR367W	999
YDL075W	YLR388W	999
YDL075W	YLR406C	908
YDL075W	YLR441C	921
YDL075W	YLR448W	999
YDL075W	YML024W	999
YDL075W	YML026C	920
YDL075W	YML063W	998
YDL075W	YML073C	996
YDL075W	YMR121C	910
YDL075W	YMR142C	927
YDL075W	YMR194W	999
YDL075W	YMR230W	953
YDL075W	YMR242C	996
YDL075W	YMR260C	917
YDL075W	YNL002C	931
YDL075W	YNL067W	968
YDL075W	YNL069C	996
YDL075W	YNL096C	996
YDL075W	YNL110C	976
YDL075W	YNL162W	934
YDL075W	YNL178W	999
YDL075W	YNL209W	966
YDL075W	YNL301C	999
YDL075W	YNL302C	916
YDL075W	YOL039W	928
YDL075W	YOL040C	996
YDL075W	YOL120C	914
YDL075W	YOL121C	917
YDL075W	YOL127W	999
YDL075W	YOR063W	999
YDL075W	YOR096W	999
YDL075W	YOR133W	940
YDL075W	YOR167C	941

YDL075W	YOR182C	909
YDL075W	YOR234C	999
YDL075W	YOR293W	966
YDL075W	YOR312C	996
YDL075W	YOR369C	910
YDL075W	YPL012W	929
YDL075W	YPL037C	999
YDL075W	YPL079W	970
YDL075W	YPL081W	925
YDL075W	YPL090C	995
YDL075W	YPL131W	999
YDL075W	YPL143W	981
YDL075W	YPL198W	994
YDL075W	YPL220W	999
YDL075W	YPL249C-A	997
YDL075W	YPR043W	968
YDL075W	YPR067W	970
YDL075W	YPR080W	905
YDL075W	YPR102C	998
YDL075W	YPR132W	996
YDL076C	YAL013W	999
YDL076C	YBR095C	999
YDL076C	YDR207C	999
YDL076C	YER088C	988
YDL076C	YIL084C	999
YDL076C	YKL185W	993
YDL076C	YLR098C	957
YDL076C	YMR075W	985
YDL076C	YMR128W	962
YDL076C	YMR263W	999
YDL076C	YNL097C	999
YDL076C	YNL330C	999
YDL076C	YOL004W	999
YDL076C	YPL139C	999
YDL076C	YPL181W	999
YDL077C	YDR080W	999
YDL077C	YGL212W	997
YDL077C	YKL196C	920
YDL077C	YLR093C	925
YDL077C	YLR148W	999
YDL077C	YLR396C	999
YDL077C	YML001W	998
YDL077C	YMR197C	938
YDL077C	YMR231W	999

YDL077C	YOR106W	944
YDL077C	YPL045W	999
YDL077C	YPL240C	919
YDL078C	YBR218C	970
YDL078C	YCR005C	990
YDL078C	YDR244W	999
YDL078C	YER073W	923
YDL078C	YGL062W	951
YDL078C	YIR031C	900
YDL078C	YKL029C	907
YDL078C	YKL106W	902
YDL078C	YKR097W	951
YDL078C	YLR027C	912
YDL078C	YNL117W	900
YDL078C	YNR001C	913
YDL078C	YOL126C	973
YDL078C	YPL262W	937
YDL079C	YKL127W	927
YDL079C	YMR037C	902
YDL079C	YMR139W	980
YDL080C	YBR240C	993
YDL080C	YDR081C	946
YDL080C	YOR374W	935
YDL081C	YBL027W	928
YDL081C	YBL072C	926
YDL081C	YBL087C	964
YDL081C	YBL092W	975
YDL081C	YBR031W	956
YDL081C	YBR048W	904
YDL081C	YBR084C-A	928
YDL081C	YBR118W	914
YDL081C	YBR181C	927
YDL081C	YBR189W	905
YDL081C	YBR191W	934
YDL081C	YCR031C	928
YDL081C	YDL061C	910
YDL081C	YDL075W	937
YDL081C	YDL082W	951
YDL081C	YDL130W	912
YDL081C	YDL136W	916
YDL081C	YDL191W	916
YDL081C	YDR012W	952
YDL081C	YDR025W	904
YDL081C	YDR064W	972

YDL081C	YDR382W	999
YDL081C	YDR385W	998
YDL081C	YDR418W	988
YDL081C	YDR447C	949
YDL081C	YDR450W	938
YDL081C	YDR471W	910
YDL081C	YDR500C	908
YDL081C	YEL054C	927
YDL081C	YER056C-A	908
YDL081C	YER074W	903
YDL081C	YER102W	926
YDL081C	YER117W	923
YDL081C	YER131W	908
YDL081C	YFL034C-A	917
YDL081C	YFR031C-A	971
YDL081C	YFR032C-A	947
YDL081C	YGL030W	925
YDL081C	YGL031C	918
YDL081C	YGL076C	949
YDL081C	YGL135W	938
YDL081C	YGL147C	954
YDL081C	YGL189C	908
YDL081C	YGR027C	941
YDL081C	YGR034W	953
YDL081C	YGR085C	933
YDL081C	YGR118W	939
YDL081C	YGR214W	932
YDL081C	YHL001W	938
YDL081C	YHL015W	963
YDL081C	YHL033C	943
YDL081C	YHR010W	910
YDL081C	YHR021C	926
YDL081C	YHR141C	951
YDL081C	YHR203C	943
YDL081C	YIL018W	932
YDL081C	YIL052C	908
YDL081C	YIL069C	903
YDL081C	YIL133C	954
YDL081C	YIL148W	913
YDL081C	YJL136C	943
YDL081C	YJL177W	937
YDL081C	YJL189W	914
YDL081C	YJL190C	955
YDL081C	YJL191W	921

YDL081C	YJR094W-A	939
YDL081C	YJR123W	933
YDL081C	YJR145C	943
YDL081C	YKL006W	937
YDL081C	YKL156W	926
YDL081C	YKL180W	937
YDL081C	YKR057W	943
YDL081C	YKR094C	913
YDL081C	YLL045C	961
YDL081C	YLR029C	907
YDL081C	YLR048W	932
YDL081C	YLR061W	921
YDL081C	YLR075W	960
YDL081C	YLR167W	905
YDL081C	YLR185W	963
YDL081C	YLR264W	926
YDL081C	YLR287C-A	902
YDL081C	YLR325C	924
YDL081C	YLR333C	933
YDL081C	YLR340W	999
YDL081C	YLR344W	941
YDL081C	YLR367W	912
YDL081C	YLR388W	911
YDL081C	YLR406C	937
YDL081C	YLR441C	938
YDL081C	YLR448W	936
YDL081C	YML024W	939
YDL081C	YML026C	938
YDL081C	YML063W	929
YDL081C	YML073C	942
YDL081C	YMR121C	917
YDL081C	YMR142C	929
YDL081C	YMR194W	938
YDL081C	YMR230W	934
YDL081C	YMR242C	961
YDL081C	YMR260C	905
YDL081C	YNL067W	935
YDL081C	YNL069C	949
YDL081C	YNL096C	935
YDL081C	YNL162W	945
YDL081C	YNL178W	968
YDL081C	YNL301C	931
YDL081C	YNL302C	928
YDL081C	YOL039W	998

YDL081C	YOL040C	976
YDL081C	YOL120C	931
YDL081C	YOL121C	944
YDL081C	YOL127W	909
YDL081C	YOR063W	914
YDL081C	YOR096W	927
YDL081C	YOR133W	916
YDL081C	YOR167C	937
YDL081C	YOR182C	902
YDL081C	YOR234C	926
YDL081C	YOR293W	935
YDL081C	YOR312C	939
YDL081C	YOR369C	968
YDL081C	YPL079W	933
YDL081C	YPL081W	905
YDL081C	YPL090C	910
YDL081C	YPL131W	905
YDL081C	YPL143W	926
YDL081C	YPL198W	938
YDL081C	YPL220W	938
YDL081C	YPL249C-A	975
YDL081C	YPR043W	939
YDL081C	YPR080W	914
YDL081C	YPR102C	939
YDL081C	YPR132W	939
YDL082W	YAL035W	993
YDL082W	YBL027W	996
YDL082W	YBL072C	995
YDL082W	YBL087C	959
YDL082W	YBL092W	997
YDL082W	YBR031W	997
YDL082W	YBR048W	914
YDL082W	YBR084C-A	922
YDL082W	YBR105C	984
YDL082W	YBR118W	910
YDL082W	YBR181C	997
YDL082W	YBR189W	969
YDL082W	YBR191W	997
YDL082W	YCR031C	996
YDL082W	YDL014W	949
YDL082W	YDL061C	917
YDL082W	YDL075W	996
YDL082W	YDL081C	951
YDL082W	YDL083C	970

YDL082W	YDL136W	996
YDL082W	YDL191W	916
YDL082W	YDR012W	997
YDL082W	YDR025W	995
YDL082W	YDR064W	997
YDL082W	YDR382W	996
YDL082W	YDR385W	914
YDL082W	YDR418W	997
YDL082W	YDR447C	997
YDL082W	YDR450W	920
YDL082W	YDR471W	996
YDL082W	YDR500C	907
YDL082W	YEL054C	997
YDL082W	YER074W	995
YDL082W	YER102W	928
YDL082W	YER117W	929
YDL082W	YER131W	952
YDL082W	YFL034C-A	912
YDL082W	YFR031C-A	998
YDL082W	YFR032C-A	996
YDL082W	YGL014W	956
YDL082W	YGL030W	997
YDL082W	YGL031C	997
YDL082W	YGL076C	998
YDL082W	YGL103W	996
YDL082W	YGL123W	938
YDL082W	YGL135W	996
YDL082W	YGL147C	998
YDL082W	YGL189C	914
YDL082W	YGR027C	944
YDL082W	YGR034W	996
YDL082W	YGR085C	997
YDL082W	YGR103W	936
YDL082W	YGR118W	941
YDL082W	YGR148C	938
YDL082W	YGR214W	959
YDL082W	YHL001W	997
YDL082W	YHL015W	925
YDL082W	YHL033C	996
YDL082W	YHR010W	996
YDL082W	YHR021C	928
YDL082W	YHR141C	998
YDL082W	YHR203C	996
YDL082W	YHR216W	974

YDL082W	YIL018W	996
YDL082W	YIL052C	910
YDL082W	YIL069C	912
YDL082W	YIL133C	997
YDL082W	YIL148W	905
YDL082W	YJL136C	937
YDL082W	YJL177W	996
YDL082W	YJL189W	921
YDL082W	YJL190C	998
YDL082W	YJL191W	998
YDL082W	YJR094W-A	921
YDL082W	YJR123W	998
YDL082W	YJR145C	994
YDL082W	YKL006W	941
YDL082W	YKL156W	936
YDL082W	YKL172W	927
YDL082W	YKL180W	996
YDL082W	YKR057W	928
YDL082W	YKR094C	905
YDL082W	YLL045C	998
YDL082W	YLR029C	997
YDL082W	YLR048W	945
YDL082W	YLR061W	924
YDL082W	YLR075W	996
YDL082W	YLR167W	902
YDL082W	YLR185W	908
YDL082W	YLR264W	921
YDL082W	YLR287C-A	995
YDL082W	YLR325C	939
YDL082W	YLR333C	932
YDL082W	YLR340W	997
YDL082W	YLR344W	911
YDL082W	YLR367W	931
YDL082W	YLR388W	918
YDL082W	YLR406C	915
YDL082W	YLR441C	997
YDL082W	YLR448W	997
YDL082W	YML024W	930
YDL082W	YML026C	920
YDL082W	YML063W	997
YDL082W	YML073C	997
YDL082W	YMR121C	932
YDL082W	YMR142C	998
YDL082W	YMR194W	996

YDL082W	YMR230W	938
YDL082W	YMR242C	998
YDL082W	YMR260C	908
YDL082W	YMR290C	947
YDL082W	YNL067W	943
YDL082W	YNL069C	996
YDL082W	YNL096C	996
YDL082W	YNL110C	975
YDL082W	YNL162W	923
YDL082W	YNL178W	998
YDL082W	YNL301C	996
YDL082W	YNL302C	923
YDL082W	YOL039W	947
YDL082W	YOL040C	953
YDL082W	YOL041C	927
YDL082W	YOL077C	950
YDL082W	YOL120C	998
YDL082W	YOL121C	949
YDL082W	YOL127W	996
YDL082W	YOR063W	996
YDL082W	YOR096W	995
YDL082W	YOR133W	914
YDL082W	YOR167C	923
YDL082W	YOR234C	998
YDL082W	YOR272W	940
YDL082W	YOR293W	941
YDL082W	YOR312C	996
YDL082W	YOR369C	959
YDL082W	YPL079W	951
YDL082W	YPL081W	945
YDL082W	YPL090C	995
YDL082W	YPL093W	987
YDL082W	YPL131W	931
YDL082W	YPL143W	929
YDL082W	YPL198W	997
YDL082W	YPL220W	995
YDL082W	YPL249C-A	997
YDL082W	YPR043W	921
YDL082W	YPR080W	910
YDL082W	YPR102C	958
YDL082W	YPR132W	930
YDL083C	YBL027W	960
YDL083C	YBL072C	954
YDL083C	YBL092W	969

YDL083C	YBR031W	967
YDL083C	YBR048W	997
YDL083C	YBR181C	957
YDL083C	YBR189W	986
YDL083C	YBR191W	963
YDL083C	YCL037C	978
YDL083C	YCR031C	998
YDL083C	YDL014W	947
YDL083C	YDL061C	912
YDL083C	YDL075W	966
YDL083C	YDL082W	970
YDL083C	YDL136W	967
YDL083C	YDR064W	997
YDL083C	YDR382W	965
YDL083C	YDR385W	909
YDL083C	YDR418W	966
YDL083C	YDR447C	970
YDL083C	YDR450W	966
YDL083C	YDR471W	960
YDL083C	YEL026W	966
YDL083C	YER074W	962
YDL083C	YER102W	952
YDL083C	YER131W	977
YDL083C	YFR031C-A	961
YDL083C	YFR032C-A	975
YDL083C	YGL030W	982
YDL083C	YGL031C	956
YDL083C	YGL076C	975
YDL083C	YGL103W	962
YDL083C	YGL120C	927
YDL083C	YGL123W	998
YDL083C	YGL135W	967
YDL083C	YGL147C	965
YDL083C	YGR027C	981
YDL083C	YGR034W	964
YDL083C	YGR085C	968
YDL083C	YGR118W	998
YDL083C	YGR214W	962
YDL083C	YHL001W	968
YDL083C	YHL015W	998
YDL083C	YHL033C	962
YDL083C	YHL034C	978
YDL083C	YHR141C	965
YDL083C	YHR203C	968

YDL083C	YIL018W	962
YDL083C	YIL069C	967
YDL083C	YIL133C	991
YDL083C	YJL177W	964
YDL083C	YJL190C	996
YDL083C	YJR123W	998
YDL083C	YJR145C	948
YDL083C	YKL180W	964
YDL083C	YLL045C	949
YDL083C	YLR029C	972
YDL083C	YLR075W	970
YDL083C	YLR287C-A	952
YDL083C	YLR340W	975
YDL083C	YLR441C	964
YDL083C	YLR448W	971
YDL083C	YML063W	966
YDL083C	YML073C	970
YDL083C	YMR142C	965
YDL083C	YMR194W	969
YDL083C	YMR230W	982
YDL083C	YMR242C	972
YDL083C	YNL069C	990
YDL083C	YNL096C	965
YDL083C	YNL178W	998
YDL083C	YNL301C	965
YDL083C	YOL040C	998
YDL083C	YOL127W	961
YDL083C	YOR063W	966
YDL083C	YOR096W	955
YDL083C	YOR204W	931
YDL083C	YPL081W	999
YDL083C	YPL131W	951
YDL083C	YPL249C-A	972
YDL084W	YCL011C	999
YDL084W	YDR138W	999
YDL084W	YDR159W	941
YDL084W	YDR381W	999
YDL084W	YDR432W	968
YDL084W	YDR510W	920
YDL084W	YER063W	988
YDL084W	YHR167W	999
YDL084W	YKL074C	997
YDL084W	YKL214C	994
YDL084W	YLR418C	916

YDL084W	YML062C	999
YDL084W	YMR314W	998
YDL084W	YNL004W	998
YDL084W	YNL139C	999
YDL084W	YNL253W	982
YDL084W	YOR001W	998
YDL085W	YDR050C	902
YDL085W	YEL047C	907
YDL085W	YIL155C	983
YDL085W	YMR145C	970
YDL087C	YBR119W	999
YDL087C	YDR235W	999
YDL087C	YDR240C	999
YDL087C	YER029C	999
YDL087C	YFL017W-A	983
YDL087C	YGR013W	999
YDL087C	YGR074W	988
YDL087C	YHR086W	999
YDL087C	YHR165C	993
YDL087C	YIL061C	999
YDL087C	YKL012W	999
YDL087C	YKL173W	997
YDL087C	YLR147C	999
YDL087C	YLR275W	999
YDL087C	YLR298C	999
YDL087C	YML046W	999
YDL087C	YML049C	999
YDL087C	YMR125W	999
YDL087C	YMR288W	964
YDL087C	YOL139C	901
YDL087C	YPL178W	999
YDL087C	YPR182W	999
YDL088C	YAR002W	947
YDL088C	YBL079W	998
YDL088C	YDL116W	939
YDL088C	YDL207W	976
YDL088C	YDR159W	966
YDL088C	YDR192C	989
YDL088C	YER105C	948
YDL088C	YER107C	937
YDL088C	YFR002W	975
YDL088C	YGL100W	948
YDL088C	YGR119C	905
YDL088C	YIL115C	938

YDL088C	YJL061W	941
YDL088C	YKL057C	947
YDL088C	YKL068W	983
YDL088C	YKR082W	941
YDL088C	YLR018C	995
YDL088C	YLR335W	936
YDL088C	YLR347C	997
YDL088C	YML031W	989
YDL088C	YML103C	965
YDL088C	YMR047C	993
YDL088C	YMR129W	997
YDL088C	YMR153W	992
YDL088C	YMR308C	916
YDL088C	YOR257W	961
YDL088C	YPL125W	915
YDL088C	YPL169C	905
YDL089W	YCR086W	980
YDL089W	YLR324W	957
YDL089W	YML034W	998
YDL090C	YDR461W	932
YDL090C	YKL019W	999
YDL092W	YDL051W	998
YDL092W	YKL122C	999
YDL092W	YML105C	999
YDL092W	YPL210C	999
YDL092W	YPL243W	999
YDL092W	YPR088C	999
YDL093W	YDL052C	919
YDL095W	YAL023C	999
YDL095W	YJR143C	989
YDL095W	YOR002W	948
YDL095W	YOR321W	979
YDL095W	YPR183W	918
YDL097C	YBL041W	952
YDL097C	YBR272C	984
YDL097C	YDL007W	999
YDL097C	YDL025C	940
YDL097C	YDL147W	999
YDL097C	YDR363W-A	999
YDL097C	YDR394W	999
YDL097C	YDR427W	999
YDL097C	YEL009C	900
YDL097C	YEL037C	982
YDL097C	YER012W	999

YDL097C	YER021W	999
YDL097C	YER094C	945
YDL097C	YFR004W	999
YDL097C	YFR010W	999
YDL097C	YFR050C	954
YDL097C	YFR052W	999
YDL097C	YGL004C	945
YDL097C	YGL011C	929
YDL097C	YGL048C	999
YDL097C	YGR135W	944
YDL097C	YGR232W	998
YDL097C	YGR253C	933
YDL097C	YHL030W	999
YDL097C	YHR027C	999
YDL097C	YHR200W	999
YDL097C	YIL075C	999
YDL097C	YJL001W	915
YDL097C	YKL145W	999
YDL097C	YLL039C	972
YDL097C	YLR421C	999
YDL097C	YML092C	929
YDL097C	YMR314W	932
YDL097C	YNL298W	919
YDL097C	YOL038W	979
YDL097C	YOR117W	999
YDL097C	YOR157C	941
YDL097C	YOR259C	999
YDL097C	YOR261C	999
YDL097C	YOR362C	928
YDL097C	YPR103W	924
YDL097C	YPR108W	999
YDL098C	YBL026W	987
YDL098C	YBR055C	976
YDL098C	YBR152W	999
YDL098C	YDL030W	976
YDL098C	YDR378C	989
YDL098C	YDR473C	993
YDL098C	YER029C	994
YDL098C	YER172C	999
YDL098C	YGR074W	976
YDL098C	YGR075C	999
YDL098C	YGR091W	974
YDL098C	YHR165C	978
YDL098C	YJL203W	974

YDL098C	YKL173W	994
YDL098C	YLR147C	996
YDL098C	YLR275W	976
YDL098C	YML049C	976
YDL098C	YMR240C	974
YDL098C	YMR268C	970
YDL098C	YMR288W	976
YDL098C	YNL147W	999
YDL098C	YOR308C	989
YDL098C	YPL213W	974
YDL098C	YPR082C	989
YDL098C	YPR178W	999
YDL099W	YDL058W	901
YDL099W	YDL137W	923
YDL099W	YDR517W	999
YDL100C	YBL076C	932
YDL100C	YDL137W	911
YDL100C	YER019C-A	903
YDL100C	YER083C	999
YDL100C	YGL020C	998
YDL100C	YGL054C	910
YDL100C	YJR040W	910
YDL100C	YLR268W	953
YDL100C	YOL018C	954
YDL101C	YAL021C	977
YDL101C	YBL088C	992
YDL101C	YBR136W	972
YDL101C	YBR274W	997
YDL101C	YDL220C	908
YDL101C	YDR075W	926
YDL101C	YDR113C	998
YDL101C	YDR217C	969
YDL101C	YGR180C	941
YDL101C	YHR135C	974
YDL101C	YIL066C	955
YDL101C	YKL025C	983
YDL101C	YML032C	991
YDL101C	YML058W	999
YDL101C	YNL201C	919
YDL101C	YNR052C	911
YDL101C	YPL153C	999
YDL101C	YPL269W	919
YDL101C	YPR018W	974
YDL101C	YPR120C	941

YDL102W	YAR007C	923
YDL102W	YBR088C	999
YDL102W	YBR278W	960
YDL102W	YCR066W	997
YDL102W	YCR092C	900
YDL102W	YDR097C	982
YDL102W	YFL029C	941
YDL102W	YHR164C	998
YDL102W	YIR008C	964
YDL102W	YJR006W	999
YDL102W	YJR043C	999
YDL102W	YKL045W	901
YDL102W	YKL113C	999
YDL102W	YML032C	997
YDL102W	YMR167W	979
YDL102W	YNL082W	998
YDL102W	YNL102W	998
YDL102W	YNL218W	947
YDL102W	YNL250W	973
YDL102W	YNL262W	999
YDL102W	YNL312W	903
YDL102W	YOL090W	999
YDL102W	YOR033C	988
YDL102W	YOR217W	911
YDL102W	YPL167C	971
YDL103C	YEL058W	995
YDL103C	YFL017C	947
YDL105W	YDR288W	963
YDL105W	YLR007W	904
YDL105W	YOL034W	987
YDL106C	YBR093C	999
YDL106C	YBR160W	971
YDL106C	YBR248C	934
YDL106C	YDR146C	999
YDL106C	YFR034C	999
YDL106C	YGR233C	922
YDL106C	YJR048W	903
YDL106C	YKR099W	999
YDL106C	YLR058C	931
YDL106C	YOL001W	983
YDL107W	Q0250	922
YDL107W	YGR062C	983
YDL108W	YBR160W	980
YDL108W	YBR236C	925

YDL108W	YBR253W	936
YDL108W	YBR279W	946
YDL108W	YDL125C	990
YDL108W	YDL140C	999
YDL108W	YDL155W	903
YDL108W	YDR168W	995
YDL108W	YDR311W	996
YDL108W	YDR460W	999
YDL108W	YER022W	962
YDL108W	YER148W	911
YDL108W	YER171W	999
YDL108W	YFL029C	996
YDL108W	YGL130W	984
YDL108W	YGL244W	984
YDL108W	YGR104C	928
YDL108W	YGR108W	900
YDL108W	YHR041C	984
YDL108W	YIL143C	923
YDL108W	YLR005W	970
YDL108W	YLR071C	987
YDL108W	YLR210W	901
YDL108W	YML010W	982
YDL108W	YMR277W	952
YDL108W	YNL222W	916
YDL108W	YNL236W	986
YDL108W	YOL051W	913
YDL108W	YOL145C	925
YDL108W	YOR151C	986
YDL108W	YOR174W	997
YDL108W	YPL122C	962
YDL108W	YPR025C	999
YDL108W	YPR056W	982
YDL108W	YPR119W	901
YDL111C	YCR035C	999
YDL111C	YDR280W	999
YDL111C	YGR095C	999
YDL111C	YGR158C	999
YDL111C	YGR195W	999
YDL111C	YHR069C	999
YDL111C	YHR081W	987
YDL111C	YNL189W	986
YDL111C	YNL232W	999
YDL111C	YNR024W	959
YDL111C	YOL021C	999

YDL111C	YOL142W	999
YDL111C	YOR001W	999
YDL111C	YOR076C	999
YDL111C	YOR326W	966
YDL111C	YPR189W	981
YDL112W	YBL046W	925
YDL112W	YKL182W	960
YDL113C	YBL025W	956
YDL113C	YDR425W	940
YDL113C	YJL036W	999
YDL113C	YLR423C	903
YDL113C	YPR049C	984
YDL115C	YBR154C	996
YDL115C	YDL140C	996
YDL115C	YDR404C	997
YDL115C	YGL070C	997
YDL115C	YGR005C	987
YDL115C	YGR186W	987
YDL115C	YIL021W	999
YDL115C	YJL140W	997
YDL115C	YOL005C	997
YDL115C	YOR151C	997
YDL115C	YOR210W	996
YDL115C	YOR224C	997
YDL115C	YPR187W	996
YDL116W	YAL009W	991
YDL116W	YAR002W	978
YDL116W	YBL079W	972
YDL116W	YDL088C	939
YDL116W	YDL207W	984
YDL116W	YDR159W	969
YDL116W	YDR192C	997
YDL116W	YER105C	975
YDL116W	YFR002W	970
YDL116W	YGL092W	999
YDL116W	YGL100W	999
YDL116W	YGL172W	998
YDL116W	YGR119C	995
YDL116W	YHR004C	952
YDL116W	YIL115C	972
YDL116W	YJL041W	991
YDL116W	YJL061W	949
YDL116W	YJR042W	999
YDL116W	YKL057C	999

YDL116W	YKL068W	999
YDL116W	YKL186C	996
YDL116W	YKR082W	999
YDL116W	YLR018C	958
YDL116W	YLR208W	999
YDL116W	YLR335W	956
YDL116W	YLR347C	989
YDL116W	YML107C	952
YDL116W	YMR047C	999
YDL116W	YMR129W	925
YDL116W	YMR153W	952
YDL116W	YOR098C	928
YDL116W	YOR257W	961
YDL116W	YPL020C	925
YDL116W	YPL169C	999
YDL117W	YDR099W	922
YDL117W	YHR023W	913
YDL117W	YMR032W	983
YDL117W	YPL242C	951
YDL120W	YCL017C	999
YDL120W	YCR005C	905
YDL120W	YGL071W	951
YDL120W	YJL133W	992
YDL120W	YKL134C	980
YDL120W	YKR052C	992
YDL120W	YLL041C	903
YDL120W	YLR304C	972
YDL120W	YLR369W	978
YDL120W	YOR176W	990
YDL120W	YPL135W	999
YDL122W	YKR094C	968
YDL122W	YLR167W	968
YDL123W	YER125W	927
YDL125C	YDL108W	990
YDL125C	YFL029C	933
YDL126C	YBL058W	999
YDL126C	YBR170C	999
YDL126C	YBR201W	999
YDL126C	YBR273C	992
YDL126C	YDL132W	965
YDL126C	YDL190C	999
YDL126C	YDR049W	998
YDL126C	YDR330W	947
YDL126C	YDR411C	999

YDL126C	YEL037C	963
YDL126C	YFL044C	993
YDL126C	YGL108C	920
YDL126C	YGR048W	999
YDL126C	YGR162W	940
YDL126C	YIL030C	999
YDL126C	YIR033W	919
YDL126C	YJL048C	975
YDL126C	YKL020C	984
YDL126C	YKL213C	999
YDL126C	YLL039C	996
YDL126C	YLR207W	976
YDL126C	YLR450W	972
YDL126C	YML013W	999
YDL126C	YMR067C	997
YDL126C	YMR264W	921
YDL126C	YOL013C	999
YDL126C	YOR075W	903
YDL126C	YPL256C	994
YDL127W	YDR146C	942
YDL127W	YDR388W	976
YDL127W	YFR034C	936
YDL127W	YHR030C	969
YDL127W	YPL031C	999
YDL128W	YGL006W	999
YDL128W	YLR433C	928
YDL130W	YBL092W	911
YDL130W	YBR031W	935
YDL130W	YBR084C-A	930
YDL130W	YBR189W	935
YDL130W	YBR191W	950
YDL130W	YCR031C	950
YDL130W	YDL061C	941
YDL130W	YDL075W	954
YDL130W	YDL081C	912
YDL130W	YDL136W	918
YDL130W	YDL191W	904
YDL130W	YDR012W	939
YDL130W	YDR064W	954
YDL130W	YDR382W	999
YDL130W	YDR385W	997
YDL130W	YDR418W	991
YDL130W	YDR447C	906
YDL130W	YDR450W	902

YDL130W	YDR471W	927
YDL130W	YDR500C	936
YDL130W	YEL054C	916
YDL130W	YER056C-A	939
YDL130W	YER074W	903
YDL130W	YER102W	928
YDL130W	YER117W	930
YDL130W	YER131W	908
YDL130W	YFL034C-A	911
YDL130W	YFR032C-A	901
YDL130W	YGL030W	955
YDL130W	YGL076C	913
YDL130W	YGL103W	937
YDL130W	YGL123W	943
YDL130W	YGL135W	915
YDL130W	YGL189C	908
YDL130W	YGR027C	923
YDL130W	YGR034W	912
YDL130W	YGR085C	923
YDL130W	YGR118W	941
YDL130W	YGR148C	919
YDL130W	YGR214W	924
YDL130W	YHL001W	951
YDL130W	YHL015W	929
YDL130W	YHL033C	948
YDL130W	YHR010W	925
YDL130W	YHR021C	902
YDL130W	YHR141C	917
YDL130W	YHR203C	941
YDL130W	YIL052C	948
YDL130W	YIL069C	903
YDL130W	YIL133C	914
YDL130W	YIL148W	923
YDL130W	YJL136C	929
YDL130W	YJL177W	937
YDL130W	YJL189W	916
YDL130W	YJL190C	902
YDL130W	YJL191W	943
YDL130W	YJR094W-A	937
YDL130W	YJR123W	942
YDL130W	YJR145C	917
YDL130W	YKL006W	955
YDL130W	YKL156W	923
YDL130W	YKL180W	954

YDL130W	YKR057W	913
YDL130W	YLL045C	914
YDL130W	YLR029C	925
YDL130W	YLR048W	948
YDL130W	YLR061W	958
YDL130W	YLR075W	948
YDL130W	YLR167W	923
YDL130W	YLR185W	937
YDL130W	YLR264W	925
YDL130W	YLR287C-A	902
YDL130W	YLR340W	999
YDL130W	YLR344W	954
YDL130W	YLR367W	917
YDL130W	YLR388W	957
YDL130W	YLR406C	907
YDL130W	YLR441C	906
YDL130W	YLR448W	943
YDL130W	YML024W	942
YDL130W	YML026C	902
YDL130W	YML063W	921
YDL130W	YML073C	902
YDL130W	YMR121C	907
YDL130W	YMR194W	943
YDL130W	YMR230W	913
YDL130W	YMR242C	906
YDL130W	YMR260C	905
YDL130W	YNL067W	919
YDL130W	YNL069C	914
YDL130W	YNL096C	920
YDL130W	YNL162W	917
YDL130W	YNL178W	959
YDL130W	YNL301C	923
YDL130W	YNL302C	904
YDL130W	YOL039W	999
YDL130W	YOL040C	901
YDL130W	YOL121C	904
YDL130W	YOL127W	941
YDL130W	YOR063W	932
YDL130W	YOR096W	943
YDL130W	YOR133W	911
YDL130W	YOR167C	901
YDL130W	YOR182C	902
YDL130W	YOR234C	925
YDL130W	YOR293W	924

YDL130W	YOR312C	906
YDL130W	YOR369C	920
YDL130W	YPL079W	915
YDL130W	YPL081W	905
YDL130W	YPL131W	955
YDL130W	YPL143W	928
YDL130W	YPL198W	913
YDL130W	YPL220W	935
YDL130W	YPL249C-A	928
YDL130W	YPR043W	920
YDL130W	YPR102C	916
YDL130W	YPR132W	928
YDL130W-A	YBR039W	914
YDL130W-A	YDL004W	910
YDL130W-A	YDL181W	954
YDL130W-A	YPL271W	911
YDL131W	YDL182W	999
YDL131W	YDR234W	969
YDL131W	YNL104C	905
YDL132W	YAL040C	916
YDL132W	YBR280C	998
YDL132W	YDL017W	976
YDL132W	YDL126C	965
YDL132W	YDL155W	962
YDL132W	YDR054C	999
YDL132W	YDR131C	969
YDL132W	YDR139C	999
YDL132W	YDR328C	999
YDL132W	YFL009W	999
YDL132W	YGL249W	983
YDL132W	YGL252C	966
YDL132W	YIL046W	999
YDL132W	YJL047C	999
YDL132W	YJL149W	999
YDL132W	YJL187C	946
YDL132W	YJR090C	999
YDL132W	YLR079W	997
YDL132W	YLR097C	979
YDL132W	YLR100W	983
YDL132W	YLR128W	999
YDL132W	YLR352W	985
YDL132W	YLR368W	982
YDL132W	YML088W	999
YDL132W	YMR199W	987

YDL132W	YOL133W	999
YDL132W	YOR080W	999
YDL132W	YPL003W	946
YDL132W	YPL256C	999
YDL134C	YAL016W	999
YDL134C	YDL047W	996
YDL134C	YDL188C	999
YDL134C	YGL190C	999
YDL134C	YGR161C	988
YDL134C	YHR075C	968
YDL134C	YIL153W	982
YDL134C	YKL203C	904
YDL134C	YML109W	999
YDL134C	YMR001C	967
YDL134C	YMR028W	999
YDL134C	YMR036C	902
YDL134C	YMR273C	999
YDL134C	YOR014W	999
YDL134C	YPL152W	999
YDL135C	YAL048C	904
YDL135C	YER136W	952
YDL135C	YKR055W	987
YDL135C	YLR229C	999
YDL135C	YPL115C	938
YDL135C	YPR165W	999
YDL136W	YAL035W	912
YDL136W	YBL027W	999
YDL136W	YBL072C	994
YDL136W	YBL087C	990
YDL136W	YBL092W	999
YDL136W	YBR031W	996
YDL136W	YBR048W	955
YDL136W	YBR084C-A	999
YDL136W	YBR118W	909
YDL136W	YBR181C	994
YDL136W	YBR189W	947
YDL136W	YBR191W	999
YDL136W	YCR031C	997
YDL136W	YDL061C	955
YDL136W	YDL075W	999
YDL136W	YDL081C	916
YDL136W	YDL082W	996
YDL136W	YDL083C	967
YDL136W	YDL130W	918

YDL136W	YDL191W	901
YDL136W	YDR012W	999
YDL136W	YDR025W	928
YDL136W	YDR064W	998
YDL136W	YDR382W	997
YDL136W	YDR418W	995
YDL136W	YDR447C	996
YDL136W	YDR450W	912
YDL136W	YDR471W	997
YDL136W	YDR500C	999
YDL136W	YEL054C	935
YDL136W	YER056C-A	981
YDL136W	YER074W	996
YDL136W	YER102W	999
YDL136W	YER117W	999
YDL136W	YER131W	937
YDL136W	YFL034C-A	912
YDL136W	YFR031C-A	999
YDL136W	YFR032C-A	998
YDL136W	YGL014W	972
YDL136W	YGL030W	999
YDL136W	YGL031C	999
YDL136W	YGL076C	999
YDL136W	YGL103W	999
YDL136W	YGL123W	954
YDL136W	YGL135W	999
YDL136W	YGL147C	999
YDL136W	YGL189C	918
YDL136W	YGR027C	945
YDL136W	YGR034W	996
YDL136W	YGR085C	996
YDL136W	YGR118W	999
YDL136W	YGR148C	941
YDL136W	YGR214W	941
YDL136W	YHL001W	997
YDL136W	YHL015W	960
YDL136W	YHL033C	999
YDL136W	YHR010W	939
YDL136W	YHR021C	927
YDL136W	YHR141C	999
YDL136W	YHR203C	999
YDL136W	YIL018W	996
YDL136W	YIL052C	990
YDL136W	YIL069C	922

YDL136W	YIL133C	999
YDL136W	YIL148W	999
YDL136W	YJL136C	940
YDL136W	YJL177W	997
YDL136W	YJL189W	936
YDL136W	YJL190C	921
YDL136W	YJL191W	948
YDL136W	YJR094W-A	998
YDL136W	YJR123W	998
YDL136W	YJR145C	911
YDL136W	YKL006W	973
YDL136W	YKL156W	981
YDL136W	YKL180W	999
YDL136W	YKR057W	930
YDL136W	YKR059W	974
YDL136W	YKR094C	902
YDL136W	YLL045C	995
YDL136W	YLR029C	994
YDL136W	YLR048W	957
YDL136W	YLR061W	971
YDL136W	YLR075W	999
YDL136W	YLR167W	999
YDL136W	YLR185W	999
YDL136W	YLR264W	963
YDL136W	YLR287C-A	994
YDL136W	YLR325C	929
YDL136W	YLR333C	916
YDL136W	YLR340W	952
YDL136W	YLR344W	999
YDL136W	YLR367W	946
YDL136W	YLR388W	977
YDL136W	YLR406C	997
YDL136W	YLR441C	995
YDL136W	YLR448W	998
YDL136W	YML024W	986
YDL136W	YML026C	912
YDL136W	YML063W	998
YDL136W	YML073C	996
YDL136W	YMR121C	929
YDL136W	YMR142C	995
YDL136W	YMR194W	998
YDL136W	YMR230W	937
YDL136W	YMR242C	996
YDL136W	YMR260C	907

YDL136W	YNL067W	998
YDL136W	YNL069C	996
YDL136W	YNL096C	996
YDL136W	YNL162W	926
YDL136W	YNL178W	977
YDL136W	YNL301C	999
YDL136W	YNL302C	998
YDL136W	YOL039W	905
YDL136W	YOL040C	997
YDL136W	YOL120C	923
YDL136W	YOL121C	925
YDL136W	YOL127W	999
YDL136W	YOR063W	999
YDL136W	YOR096W	997
YDL136W	YOR133W	939
YDL136W	YOR167C	916
YDL136W	YOR182C	905
YDL136W	YOR234C	964
YDL136W	YOR293W	957
YDL136W	YOR312C	998
YDL136W	YOR369C	937
YDL136W	YPL079W	924
YDL136W	YPL081W	928
YDL136W	YPL090C	995
YDL136W	YPL131W	996
YDL136W	YPL143W	999
YDL136W	YPL198W	926
YDL136W	YPL220W	999
YDL136W	YPL249C-A	997
YDL136W	YPR043W	983
YDL136W	YPR080W	905
YDL136W	YPR102C	993
YDL136W	YPR132W	999
YDL137W	YDL099W	923
YDL137W	YDL100C	911
YDL137W	YDL192W	999
YDL137W	YDL226C	988
YDL137W	YDR170C	933
YDL137W	YDR238C	949
YDL137W	YDR358W	989
YDL137W	YEL022W	992
YDL137W	YER122C	985
YDL137W	YGL137W	992
YDL137W	YHR108W	989

YDL137W	YHR188C	929
YDL137W	YJR031C	998
YDL137W	YNL287W	949
YDL137W	YPL010W	905
YDL137W	YPR095C	905
YDL138W	YDL194W	999
YDL138W	YDR277C	997
YDL138W	YHR135C	981
YDL138W	YOR047C	952
YDL139C	YBR009C	901
YDL139C	YGR140W	978
YDL139C	YKL049C	999
YDL140C	YBL039C	900
YDL140C	YBL093C	984
YDL140C	YBR154C	999
YDL140C	YBR160W	967
YDL140C	YBR193C	982
YDL140C	YBR198C	903
YDL140C	YBR236C	997
YDL140C	YBR253W	999
YDL140C	YBR279W	999
YDL140C	YDL005C	937
YDL140C	YDL108W	999
YDL140C	YDL115C	996
YDL140C	YDR059C	967
YDL140C	YDR167W	985
YDL140C	YDR228C	999
YDL140C	YDR289C	918
YDL140C	YDR301W	971
YDL140C	YDR308C	935
YDL140C	YDR404C	999
YDL140C	YDR432W	975
YDL140C	YDR443C	984
YDL140C	YEL026W	910
YDL140C	YER022W	999
YDL140C	YER029C	910
YDL140C	YER125W	999
YDL140C	YER139C	997
YDL140C	YER148W	997
YDL140C	YER171W	917
YDL140C	YGL025C	936
YDL140C	YGL043W	999
YDL140C	YGL070C	999
YDL140C	YGL127C	982

YDL140C	YGL130W	999
YDL140C	YGL244W	982
YDL140C	YGR005C	999
YDL140C	YGR063C	999
YDL140C	YGR097W	986
YDL140C	YGR104C	999
YDL140C	YGR116W	997
YDL140C	YGR186W	999
YDL140C	YGR200C	958
YDL140C	YHR041C	999
YDL140C	YHR058C	999
YDL140C	YHR143W-A	999
YDL140C	YIL021W	999
YDL140C	YIL143C	944
YDL140C	YJL140W	999
YDL140C	YJL168C	999
YDL140C	YJR017C	999
YDL140C	YJR035W	929
YDL140C	YJR103W	926
YDL140C	YKL028W	938
YDL140C	YKL058W	932
YDL140C	YKL110C	992
YDL140C	YKL139W	997
YDL140C	YKR062W	987
YDL140C	YLR005W	984
YDL140C	YLR015W	970
YDL140C	YLR071C	999
YDL140C	YLR113W	976
YDL140C	YLR147C	915
YDL140C	YLR275W	905
YDL140C	YLR384C	960
YDL140C	YLR418C	999
YDL140C	YLR430W	968
YDL140C	YML010W	999
YDL140C	YML095C	910
YDL140C	YMR061W	996
YDL140C	YMR277W	999
YDL140C	YNL098C	976
YDL140C	YNL222W	980
YDL140C	YNL236W	986
YDL140C	YNL251C	999
YDL140C	YNR010W	982
YDL140C	YOL005C	999
YDL140C	YOL051W	997

YDL140C	YOL135C	988
YDL140C	YOL145C	996
YDL140C	YOR001W	988
YDL140C	YOR116C	980
YDL140C	YOR151C	999
YDL140C	YOR174W	975
YDL140C	YOR194C	906
YDL140C	YOR207C	985
YDL140C	YOR210W	999
YDL140C	YOR224C	999
YDL140C	YPL022W	909
YDL140C	YPL042C	998
YDL140C	YPL129W	999
YDL140C	YPL228W	976
YDL140C	YPL248C	967
YDL140C	YPR025C	937
YDL140C	YPR056W	986
YDL140C	YPR086W	999
YDL140C	YPR093C	998
YDL140C	YPR133C	953
YDL140C	YPR161C	976
YDL140C	YPR168W	950
YDL140C	YPR187W	999
YDL142C	YCL004W	970
YDL143W	YDR188W	999
YDL143W	YDR212W	943
YDL143W	YFL037W	910
YDL143W	YFL039C	984
YDL143W	YGL195W	972
YDL143W	YGR202C	962
YDL143W	YIL142W	999
YDL143W	YJL008C	996
YDL143W	YJL014W	999
YDL143W	YJL111W	999
YDL143W	YJR064W	996
YDL143W	YKL029C	975
YDL143W	YML094W	900
YDL143W	YNL153C	901
YDL143W	YNL212W	962
YDL143W	YOR281C	999
YDL145C	YBR162C	962
YDL145C	YCR012W	930
YDL145C	YDL192W	980
YDL145C	YDL226C	919

YDL145C	YDR238C	999
YDL145C	YEL002C	972
YDL145C	YER122C	999
YDL145C	YFL048C	976
YDL145C	YFR051C	999
YDL145C	YGL051W	967
YDL145C	YGL137W	999
YDL145C	YIL076W	999
YDL145C	YJR031C	977
YDL145C	YKL144C	940
YDL145C	YLR078C	903
YDL145C	YNL258C	967
YDL145C	YNL284C	917
YDL145C	YNL287W	999
YDL145C	YNR006W	908
YDL145C	YPL010W	999
YDL146W	YHR114W	929
YDL146W	YKL070W	904
YDL147W	YBL041W	996
YDL147W	YCR014C	970
YDL147W	YDL007W	999
YDL147W	YDL097C	999
YDL147W	YDL216C	999
YDL147W	YDR069C	998
YDL147W	YDR073W	998
YDL147W	YDR179C	999
YDL147W	YDR363W-A	999
YDL147W	YDR394W	999
YDL147W	YDR427W	999
YDL147W	YER012W	999
YDL147W	YER021W	999
YDL147W	YER094C	960
YDL147W	YFL007W	971
YDL147W	YFR004W	999
YDL147W	YFR010W	999
YDL147W	YFR050C	973
YDL147W	YFR052W	999
YDL147W	YGL004C	964
YDL147W	YGL011C	995
YDL147W	YGL048C	999
YDL147W	YGL141W	954
YDL147W	YGR135W	939
YDL147W	YGR232W	998
YDL147W	YGR253C	940

YDL147W	YHL030W	999
YDL147W	YHR027C	999
YDL147W	YHR200W	999
YDL147W	YIL071C	997
YDL147W	YIL075C	999
YDL147W	YJL001W	993
YDL147W	YKL145W	999
YDL147W	YLL039C	971
YDL147W	YLR421C	999
YDL147W	YML092C	935
YDL147W	YMR025W	999
YDL147W	YMR191W	987
YDL147W	YMR314W	999
YDL147W	YOL038W	981
YDL147W	YOL117W	986
YDL147W	YOR117W	999
YDL147W	YOR157C	942
YDL147W	YOR259C	999
YDL147W	YOR261C	999
YDL147W	YOR362C	931
YDL147W	YPL026C	999
YDL147W	YPR103W	998
YDL147W	YPR108W	999
YDL148C	YBL004W	999
YDL148C	YBL072C	969
YDL148C	YBR048W	976
YDL148C	YBR189W	929
YDL148C	YBR247C	999
YDL148C	YCL059C	999
YDL148C	YCR031C	929
YDL148C	YCR057C	999
YDL148C	YDL014W	998
YDL148C	YDL060W	968
YDL148C	YDL153C	947
YDL148C	YDR064W	933
YDL148C	YDR299W	999
YDL148C	YDR324C	998
YDL148C	YDR449C	998
YDL148C	YER082C	999
YDL148C	YGL171W	923
YDL148C	YGR090W	999
YDL148C	YGR128C	999
YDL148C	YGR145W	998
YDL148C	YHR148W	998

YDL148C	YHR169W	910
YDL148C	YHR196W	991
YDL148C	YIL069C	979
YDL148C	YJL010C	910
YDL148C	YJL109C	997
YDL148C	YJR002W	999
YDL148C	YJR145C	987
YDL148C	YKR060W	997
YDL148C	YLL011W	997
YDL148C	YLR003C	911
YDL148C	YLR129W	997
YDL148C	YLR186W	999
YDL148C	YLR197W	997
YDL148C	YLR222C	991
YDL148C	YLR409C	913
YDL148C	YML093W	959
YDL148C	YMR093W	997
YDL148C	YMR128W	999
YDL148C	YMR229C	999
YDL148C	YMR290C	972
YDL148C	YNL075W	912
YDL148C	YNL132W	999
YDL148C	YNL308C	989
YDL148C	YOL010W	999
YDL148C	YOR004W	912
YDL148C	YOR078W	967
YDL148C	YOR096W	982
YDL148C	YOR310C	999
YDL148C	YPL012W	998
YDL148C	YPL126W	999
YDL148C	YPL217C	999
YDL148C	YPR112C	920
YDL148C	YPR137W	991
YDL148C	YPR144C	999
YDL149W	YFR021W	998
YDL149W	YGL180W	958
YDL149W	YJL178C	998
YDL149W	YLR431C	999
YDL149W	YNL242W	992
YDL149W	YPR049C	999
YDL149W	YPR185W	935
YDL150W	YBR154C	999
YDL150W	YDR045C	998
YDL150W	YDR156W	921

YDL150W	YDR293C	923
YDL150W	YER148W	965
YDL150W	YGR047C	999
YDL150W	YHR143W-A	960
YDL150W	YJL011C	999
YDL150W	YJL148W	922
YDL150W	YJR063W	921
YDL150W	YKL144C	999
YDL150W	YKR025W	999
YDL150W	YNL039W	960
YDL150W	YNL113W	999
YDL150W	YNL151C	999
YDL150W	YNL248C	908
YDL150W	YNR003C	999
YDL150W	YOR116C	999
YDL150W	YOR207C	999
YDL150W	YOR210W	998
YDL150W	YOR224C	999
YDL150W	YOR340C	921
YDL150W	YOR341W	921
YDL150W	YPR010C	921
YDL150W	YPR110C	999
YDL150W	YPR187W	999
YDL150W	YPR190C	999
YDL153C	YBR247C	918
YDL153C	YCL059C	975
YDL153C	YDL060W	914
YDL153C	YDL148C	947
YDL153C	YDR299W	915
YDL153C	YDR324C	923
YDL153C	YDR398W	910
YDL153C	YDR449C	911
YDL153C	YER082C	922
YDL153C	YGL120C	935
YDL153C	YGL171W	946
YDL153C	YGR128C	915
YDL153C	YGR145W	923
YDL153C	YHR148W	910
YDL153C	YHR169W	916
YDL153C	YJL109C	912
YDL153C	YJR002W	996
YDL153C	YKR060W	910
YDL153C	YLL011W	910
YDL153C	YLR003C	929

YDL153C	YLR129W	919
YDL153C	YLR175W	909
YDL153C	YLR197W	916
YDL153C	YLR222C	933
YDL153C	YLR409C	923
YDL153C	YML093W	916
YDL153C	YMR229C	918
YDL153C	YMR290C	915
YDL153C	YNL075W	918
YDL153C	YNL132W	915
YDL153C	YOR004W	922
YDL153C	YOR145C	911
YDL153C	YOR310C	923
YDL153C	YPL126W	913
YDL153C	YPR112C	917
YDL153C	YPR137W	909
YDL153C	YPR144C	909
YDL154W	YBR098W	934
YDL154W	YFL003C	957
YDL154W	YLR394W	934
YDL154W	YMR167W	961
YDL154W	YMR190C	945
YDL154W	YNL082W	905
YDL154W	YPL164C	950
YDL155W	YBR135W	999
YDL155W	YBR160W	999
YDL155W	YCL029C	962
YDL155W	YDL108W	903
YDL155W	YDL132W	962
YDL155W	YDR146C	962
YDL155W	YFL009W	962
YDL155W	YGL003C	999
YDL155W	YGL197W	962
YDL155W	YGR092W	962
YDL155W	YGR108W	913
YDL155W	YIL106W	962
YDL155W	YJL187C	949
YDL155W	YLR079W	999
YDL155W	YLR131C	962
YDL155W	YLR210W	960
YDL155W	YLR310C	978
YDL155W	YMR036C	948
YDL155W	YNL161W	962
YDL155W	YPL267W	962

YDL155W	YPR025C	920
YDL155W	YPR119W	996
YDL156W	YDR097C	934
YDL156W	YJL173C	992
YDL156W	YNL312W	997
YDL157C	YCL056C	954
YDL159W	YAL041W	901
YDL159W	YBL016W	999
YDL159W	YBR200W	922
YDL159W	YDR103W	999
YDL159W	YFL026W	966
YDL159W	YGR040W	999
YDL159W	YHR005C	992
YDL159W	YHR030C	925
YDL159W	YHR084W	999
YDL159W	YJL128C	918
YDL159W	YJR086W	964
YDL159W	YLR362W	999
YDL159W	YMR232W	998
YDL159W	YOR212W	999
YDL160C	YAL021C	997
YDL160C	YBL026W	999
YDL160C	YBR055C	972
YDL160C	YCR077C	999
YDL160C	YDR378C	989
YDL160C	YEL015W	999
YDL160C	YER112W	994
YDL160C	YGL173C	998
YDL160C	YGL178W	930
YDL160C	YGR091W	972
YDL160C	YHR084W	996
YDL160C	YJL124C	999
YDL160C	YKL173W	974
YDL160C	YNL118C	983
YDL160C	YNL147W	999
YDL160C	YNR052C	999
YDL160C	YOL149W	999
YDL160C	YOR204W	916
YDL160C	YOR276W	985
YDL160C	YOR308C	972
YDL160C	YPR082C	972
YDL160C	YPR110C	918
YDL160C	YPR129W	907
YDL160C	YPR178W	972

YDL161W	YBL047C	947
YDL161W	YGL206C	996
YDL161W	YIR006C	995
YDL164C	YBR088C	999
YDL164C	YER173W	967
YDL164C	YHR164C	943
YDL164C	YKL113C	940
YDL165W	YAL021C	999
YDL165W	YBL051C	974
YDL165W	YCR077C	913
YDL165W	YCR093W	999
YDL165W	YDR448W	901
YDL165W	YER068W	999
YDL165W	YIL038C	999
YDL165W	YNL288W	999
YDL165W	YNR052C	999
YDL165W	YOR212W	900
YDL165W	YPR072W	999
YDL166C	YJL191W	902
YDL168W	YER073W	916
YDL168W	YGL256W	981
YDL168W	YJL068C	997
YDL168W	YMR169C	915
YDL168W	YMR170C	915
YDL168W	YOR374W	916
YDL168W	YPL061W	915
YDL170W	YBR006W	957
YDL170W	YDL210W	954
YDL170W	YGR019W	960
YDL170W	YIR023W	954
YDL171C	YAL062W	991
YDL171C	YBL087C	924
YDL171C	YJR109C	913
YDL171C	YKL104C	924
YDL171C	YMR217W	924
YDL171C	YMR300C	907
YDL171C	YOR303W	925
YDL171C	YOR375C	991
YDL171C	YPR035W	986
YDL171C	YPR086W	904
YDL174C	YAL038W	923
YDL174C	YBR221C	902
YDL174C	YML054C	984
YDL175C	YDR432W	980

YDL175C	YIL079C	999
YDL175C	YJL050W	999
YDL175C	YLR430W	996
YDL175C	YNL251C	997
YDL175C	YNL299W	964
YDL175C	YOL115W	999
YDL175C	YPL190C	981
YDL176W	YGL227W	918
YDL176W	YMR135C	916
YDL178W	YFL039C	999
YDL179W	YBR160W	966
YDL179W	YDR146C	918
YDL179W	YDR388W	906
YDL179W	YER059W	986
YDL179W	YGL215W	970
YDL179W	YPL031C	999
YDL179W	YPL219W	981
YDL179W	YPL256C	946
YDL181W	YBL099W	999
YDL181W	YBR039W	917
YDL181W	YDL004W	922
YDL181W	YDL130W-A	954
YDL181W	YDR298C	920
YDL181W	YGR008C	957
YDL181W	YJR121W	999
YDL181W	YKL016C	937
YDL181W	YPL078C	999
YDL181W	YPR024W	903
YDL182W	YDL131W	999
YDL182W	YDR234W	970
YDL182W	YNL104C	918
YDL184C	YER146W	969
YDL184C	YJL124C	969
YDL185W	YBR025C	970
YDL185W	YBR127C	999
YDL185W	YEL027W	994
YDL185W	YEL051W	999
YDL185W	YER005W	900
YDL185W	YFR009W	962
YDL185W	YGR020C	999
YDL185W	YHR026W	901
YDL185W	YHR039C-A	999
YDL185W	YJR033C	994
YDL185W	YJR121W	980

YDL185W	YKL080W	999
YDL185W	YLR447C	984
YDL185W	YMR054W	999
YDL185W	YOR270C	999
YDL185W	YOR332W	999
YDL185W	YPL234C	994
YDL185W	YPR036W	999
YDL188C	YAL016W	999
YDL188C	YBL068W	946
YDL188C	YDL047W	972
YDL188C	YDL134C	999
YDL188C	YER094C	946
YDL188C	YER099C	946
YDL188C	YGL011C	944
YDL188C	YGL190C	999
YDL188C	YGR135W	923
YDL188C	YGR161C	909
YDL188C	YHR075C	909
YDL188C	YJR009C	933
YDL188C	YML092C	942
YDL188C	YML109W	999
YDL188C	YMR001C	967
YDL188C	YMR028W	999
YDL188C	YMR273C	999
YDL188C	YOL038W	923
YDL188C	YOL061W	946
YDL188C	YOR014W	999
YDL188C	YPL152W	999
YDL188C	YPR040W	941
YDL189W	YBL045C	996
YDL189W	YPR191W	929
YDL190C	YBL058W	992
YDL190C	YBR082C	904
YDL190C	YBR170C	993
YDL190C	YDL126C	999
YDL190C	YEL037C	999
YDL190C	YFL044C	988
YDL190C	YGR048W	949
YDL190C	YHR200W	980
YDL190C	YKL010C	909
YDL190C	YKL213C	995
YDL190C	YLL039C	999
YDL190C	YMR276W	993
YDL191W	YBL027W	916

YDL191W	YBL072C	916
YDL191W	YBL087C	914
YDL191W	YBL092W	926
YDL191W	YBR031W	931
YDL191W	YBR048W	928
YDL191W	YBR084C-A	916
YDL191W	YBR189W	920
YDL191W	YBR191W	913
YDL191W	YCR031C	920
YDL191W	YDL061C	906
YDL191W	YDL075W	913
YDL191W	YDL081C	916
YDL191W	YDL082W	916
YDL191W	YDL130W	904
YDL191W	YDL136W	901
YDL191W	YDR012W	931
YDL191W	YDR025W	928
YDL191W	YDR064W	944
YDL191W	YDR382W	913
YDL191W	YDR418W	935
YDL191W	YDR447C	926
YDL191W	YDR450W	912
YDL191W	YDR471W	908
YDL191W	YDR500C	918
YDL191W	YEL054C	935
YDL191W	YER056C-A	905
YDL191W	YER074W	922
YDL191W	YER102W	916
YDL191W	YER117W	914
YDL191W	YER131W	918
YDL191W	YFL034C-A	912
YDL191W	YFR031C-A	911
YDL191W	YFR032C-A	938
YDL191W	YGL030W	958
YDL191W	YGL031C	916
YDL191W	YGL076C	926
YDL191W	YGL103W	903
YDL191W	YGL123W	913
YDL191W	YGL135W	918
YDL191W	YGL147C	922
YDL191W	YGL189C	918
YDL191W	YGR027C	917
YDL191W	YGR034W	932
YDL191W	YGR085C	911

YDL191W	YGR118W	922
YDL191W	YGR148C	916
YDL191W	YGR214W	914
YDL191W	YHL001W	930
YDL191W	YHL015W	929
YDL191W	YHL033C	924
YDL191W	YHR010W	908
YDL191W	YHR021C	927
YDL191W	YHR141C	926
YDL191W	YHR203C	911
YDL191W	YIL018W	911
YDL191W	YIL052C	905
YDL191W	YIL069C	922
YDL191W	YIL133C	918
YDL191W	YIL148W	902
YDL191W	YJL136C	930
YDL191W	YJL177W	916
YDL191W	YJL189W	919
YDL191W	YJL190C	921
YDL191W	YJL191W	920
YDL191W	YJR094W-A	923
YDL191W	YJR123W	935
YDL191W	YJR145C	911
YDL191W	YKL006W	931
YDL191W	YKL156W	927
YDL191W	YKL180W	916
YDL191W	YKR057W	930
YDL191W	YKR094C	902
YDL191W	YLL045C	925
YDL191W	YLR029C	929
YDL191W	YLR048W	914
YDL191W	YLR061W	913
YDL191W	YLR075W	939
YDL191W	YLR185W	919
YDL191W	YLR264W	905
YDL191W	YLR287C-A	905
YDL191W	YLR325C	929
YDL191W	YLR333C	916
YDL191W	YLR340W	934
YDL191W	YLR344W	932
YDL191W	YLR367W	921
YDL191W	YLR388W	907
YDL191W	YLR406C	913
YDL191W	YLR441C	921

YDL191W	YLR448W	924
YDL191W	YML024W	926
YDL191W	YML026C	912
YDL191W	YML063W	920
YDL191W	YML073C	922
YDL191W	YMR121C	929
YDL191W	YMR142C	914
YDL191W	YMR194W	926
YDL191W	YMR230W	929
YDL191W	YMR242C	922
YDL191W	YMR260C	907
YDL191W	YNL067W	923
YDL191W	YNL069C	917
YDL191W	YNL096C	935
YDL191W	YNL162W	926
YDL191W	YNL178W	944
YDL191W	YNL301C	923
YDL191W	YNL302C	925
YDL191W	YOL039W	905
YDL191W	YOL040C	952
YDL191W	YOL120C	923
YDL191W	YOL121C	925
YDL191W	YOL127W	925
YDL191W	YOR063W	952
YDL191W	YOR096W	935
YDL191W	YOR167C	905
YDL191W	YOR182C	905
YDL191W	YOR234C	928
YDL191W	YOR293W	929
YDL191W	YOR312C	922
YDL191W	YOR369C	937
YDL191W	YPL079W	912
YDL191W	YPL081W	920
YDL191W	YPL131W	940
YDL191W	YPL143W	929
YDL191W	YPL198W	926
YDL191W	YPL220W	918
YDL191W	YPL249C-A	927
YDL191W	YPR043W	923
YDL191W	YPR102C	911
YDL191W	YPR132W	922
YDL192W	YAL026C	996
YDL192W	YBR084C-A	964
YDL192W	YBR162C	962

YDL192W	YBR191W	956
YDL192W	YCR094W	914
YDL192W	YDL075W	998
YDL192W	YDL137W	999
YDL192W	YDL145C	980
YDL192W	YDL226C	999
YDL192W	YDR064W	966
YDL192W	YDR170C	999
YDL192W	YDR238C	997
YDL192W	YDR320C	991
YDL192W	YDR358W	915
YDL192W	YDR524C	984
YDL192W	YEL022W	999
YDL192W	YEL027W	964
YDL192W	YER056C-A	964
YDL192W	YER117W	952
YDL192W	YER122C	999
YDL192W	YFL038C	975
YDL192W	YFR051C	931
YDL192W	YGL103W	901
YDL192W	YGL123W	915
YDL192W	YGL137W	996
YDL192W	YGL206C	982
YDL192W	YGR118W	901
YDL192W	YHR108W	986
YDL192W	YHR188C	929
YDL192W	YIL004C	998
YDL192W	YIL044C	984
YDL192W	YIL052C	972
YDL192W	YJL099W	996
YDL192W	YJR031C	999
YDL192W	YJR094W-A	944
YDL192W	YLR061W	942
YDL192W	YLR075W	982
YDL192W	YLR078C	998
YDL192W	YLR167W	966
YDL192W	YLR268W	993
YDL192W	YLR330W	996
YDL192W	YLR388W	940
YDL192W	YML024W	978
YDL192W	YMR194W	938
YDL192W	YNL178W	976
YDL192W	YNL267W	979
YDL192W	YNL287W	997

YDL192W	YOL018C	959
YDL192W	YOR216C	983
YDL192W	YPL010W	942
YDL192W	YPL037C	999
YDL192W	YPL240C	919
YDL192W	YPR132W	980
YDL194W	YBR112C	982
YDL194W	YDL138W	999
YDL194W	YDR277C	997
YDL194W	YDR345C	974
YDL194W	YGL252C	902
YDL194W	YHR094C	995
YDL194W	YIL162W	901
YDL194W	YJR090C	954
YDL194W	YKL038W	999
YDL194W	YMR011W	973
YDL194W	YOR047C	953
YDL194W	YPL026C	985
YDL195W	YDL212W	967
YDL195W	YGL200C	913
YDL195W	YHR098C	997
YDL195W	YIL004C	958
YDL195W	YIL109C	999
YDL195W	YLR208W	999
YDL195W	YNL049C	969
YDL195W	YPL085W	999
YDL195W	YPL218W	997
YDL195W	YPR181C	999
YDL197C	YPL240C	919
YDL198C	YPL240C	919
YDL199C	YPL240C	970
YDL201W	YBL024W	945
YDL201W	YDR165W	999
YDL201W	YOL115W	965
YDL201W	YOR001W	969
YDL203C	YBR011C	999
YDL203C	YER125W	985
YDL203C	YGR058W	925
YDL203C	YLR371W	999
YDL204W	YGR088W	948
YDL205C	YDR047W	975
YDL205C	YDR232W	950
YDL205C	YGL040C	999
YDL205C	YKR069W	983

YDL205C	YOR278W	995
YDL207W	YAR002W	976
YDL207W	YBL079W	962
YDL207W	YDL088C	976
YDL207W	YDL116W	984
YDL207W	YDR159W	966
YDL207W	YDR192C	999
YDL207W	YDR315C	994
YDL207W	YEL024W	976
YDL207W	YER105C	962
YDL207W	YER107C	997
YDL207W	YFR002W	972
YDL207W	YGL092W	992
YDL207W	YGL100W	976
YDL207W	YGL172W	962
YDL207W	YGR119C	962
YDL207W	YIL115C	999
YDL207W	YJL039C	962
YDL207W	YJL041W	962
YDL207W	YJL061W	996
YDL207W	YJR042W	962
YDL207W	YKL057C	962
YDL207W	YKL068W	999
YDL207W	YKR082W	962
YDL207W	YLR018C	977
YDL207W	YLR347C	989
YDL207W	YML103C	962
YDL207W	YMR047C	995
YDL207W	YMR153W	974
YDL207W	YMR255W	999
YDL207W	YOR046C	999
YDL207W	YOR098C	991
YDL207W	YOR257W	961
YDL207W	YPL020C	988
YDL207W	YPL169C	967
YDL208W	YDL014W	991
YDL208W	YGL078C	984
YDL208W	YGR283C	946
YDL208W	YHR072W-A	999
YDL208W	YHR089C	999
YDL208W	YIL104C	924
YDL208W	YKL014C	932
YDL208W	YLR175W	999
YDL208W	YMR049C	920

YDL208W	YMR310C	999
YDL208W	YNL124W	999
YDL209C	YAL032C	999
YDL209C	YBR065C	988
YDL209C	YCR063W	988
YDL209C	YDL030W	974
YDL209C	YDR364C	998
YDL209C	YDR416W	999
YDL209C	YER172C	996
YDL209C	YGL120C	990
YDL209C	YGL128C	974
YDL209C	YGR278W	996
YDL209C	YHR165C	999
YDL209C	YJR050W	965
YDL209C	YKL173W	997
YDL209C	YLL036C	999
YDL209C	YLR117C	999
YDL209C	YLR424W	992
YDL209C	YML049C	993
YDL209C	YMR125W	982
YDL209C	YMR213W	999
YDL209C	YMR288W	976
YDL209C	YPL151C	999
YDL209C	YPL213W	998
YDL209C	YPR101W	995
YDL210W	YBR006W	932
YDL210W	YDL170W	954
YDL210W	YGR019W	937
YDL210W	YIR023W	930
YDL210W	YKR034W	967
YDL210W	YKR039W	923
YDL210W	YOR348C	950
YDL212W	YBR005W	919
YDL212W	YDL195W	967
YDL212W	YDR003W	931
YDL212W	YDR508C	954
YDL212W	YIL109C	966
YDL212W	YKL124W	990
YDL212W	YKR039W	995
YDL212W	YLR208W	986
YDL212W	YPR181C	977
YDL213C	YBL004W	993
YDL213C	YCL059C	998
YDL213C	YHR089C	996

YDL213C	YLR186W	937
YDL213C	YOL010W	920
YDL215C	YBR196C	946
YDL215C	YER040W	947
YDL215C	YKR066C	919
YDL215C	YMR250W	983
YDL215C	YOR375C	986
YDL215C	YPR035W	904
YDL216C	YDL147W	999
YDL216C	YDR179C	999
YDL216C	YIL071C	999
YDL216C	YJR084W	997
YDL216C	YMR025W	999
YDL216C	YOL117W	999
YDL217C	YBR091C	999
YDL217C	YEL020W-A	979
YDL217C	YHR005C-A	999
YDL217C	YJL054W	999
YDL217C	YOR297C	999
YDL219W	YDL047W	927
YDL220C	YAR019C	949
YDL220C	YBL088C	999
YDL220C	YBR136W	999
YDL220C	YBR274W	961
YDL220C	YCL061C	989
YDL220C	YDL101C	908
YDL220C	YDR082W	999
YDL220C	YDR113C	975
YDL220C	YDR143C	990
YDL220C	YDR206W	936
YDL220C	YDR217C	999
YDL220C	YDR369C	914
YDL220C	YER173W	997
YDL220C	YER177W	936
YDL220C	YGR092W	973
YDL220C	YGR262C	949
YDL220C	YHR077C	950
YDL220C	YIL009C-A	990
YDL220C	YJR053W	940
YDL220C	YLR010C	996
YDL220C	YLR150W	950
YDL220C	YLR233C	999
YDL220C	YLR288C	948
YDL220C	YLR318W	990

YDL220C	YML032C	954
YDL220C	YML036W	956
YDL220C	YML061C	998
YDL220C	YMR106C	999
YDL220C	YMR224C	990
YDL220C	YMR284W	999
YDL220C	YNL102W	999
YDL220C	YNL250W	988
YDL220C	YOR033C	996
YDL220C	YPL153C	997
YDL220C	YPL194W	956
YDL222C	YJL117W	921
YDL223C	YNR032C-A	939
YDL224C	YIL038C	923
YDL224C	YNL197C	993
YDL225W	YCR002C	999
YDL225W	YDR013W	962
YDL225W	YDR507C	999
YDL225W	YDR510W	985
YDL225W	YHR107C	999
YDL225W	YJR076C	999
YDL225W	YLL021W	956
YDL225W	YLR314C	999
YDL225W	YNL166C	923
YDL225W	YNL233W	957
YDL225W	YNL298W	912
YDL225W	YPL031C	953
YDL226C	YDL137W	988
YDL226C	YDL145C	919
YDL226C	YDL192W	999
YDL226C	YDR077W	976
YDL226C	YDR129C	933
YDL226C	YDR264C	976
YDL226C	YER031C	963
YDL226C	YER122C	999
YDL226C	YFL039C	908
YDL226C	YGL137W	947
YDL226C	YGR199W	923
YDL226C	YIL044C	999
YDL226C	YIL076W	907
YDL226C	YMR079W	995
YDL226C	YNL243W	918
YDL226C	YNL287W	905
YDL229W	YAL005C	993

YDL229W	YAR002W	928
YDL229W	YBL075C	906
YDL229W	YBR101C	975
YDL229W	YBR155W	940
YDL229W	YDR172W	984
YDL229W	YDR457W	954
YDL229W	YER103W	905
YDL229W	YGL073W	975
YDL229W	YHR064C	966
YDL229W	YIL016W	979
YDL229W	YJL034W	907
YDL229W	YLL024C	971
YDL229W	YLL026W	973
YDL229W	YMR186W	925
YDL229W	YNL064C	985
YDL229W	YNL209W	999
YDL229W	YOR027W	936
YDL229W	YOR232W	917
YDL229W	YPL106C	998
YDL229W	YPL240C	924
YDL232W	YEL002C	999
YDL232W	YER087C-B	968
YDL232W	YGL022W	999
YDL232W	YGL226C-A	999
YDL232W	YJL002C	999
YDL232W	YLR378C	981
YDL232W	YML019W	999
YDL232W	YMR149W	999
YDL232W	YOR085W	999
YDL232W	YOR103C	999
YDL234C	YLL039C	956
YDL235C	YHR206W	999
YDL235C	YIL147C	999
YDL235C	YJL128C	936
YDL235C	YLR006C	999
YDL235C	YLR113W	938
YDL235C	YNR031C	937
YDL238C	YDR441C	912
YDL238C	YLR209C	963
YDL238C	YML022W	920
YDL239C	YHR184W	996
YDL239C	YIL144W	926
YDL239C	YLR098C	924
YDL239C	YNL225C	956

YDL239C	YOL091W	997
YDL239C	YOR373W	956
YDL239C	YPL240C	919
YDL240W	YPR165W	995
YDL246C	YHR104W	900
YDL246C	YJR037W	998
YDL246C	YJR159W	998
YDL246C	YPL031C	970
YDR001C	YBR001C	921
YDR001C	YBR126C	935
YDR001C	YDR074W	964
YDR001C	YDR099W	999
YDR001C	YER177W	999
YDR001C	YLR270W	988
YDR001C	YPR026W	983
YDR002W	YBR165W	921
YDR002W	YER009W	902
YDR002W	YGL097W	992
YDR002W	YGR218W	998
YDR002W	YLR293C	999
YDR002W	YLR335W	999
YDR002W	YMR235C	978
YDR002W	YNL189W	915
YDR003W	YDL212W	931
YDR004W	YAR007C	940
YDR004W	YBR073W	965
YDR004W	YDL059C	986
YDR004W	YDR076W	999
YDR004W	YDR369C	971
YDR004W	YER095W	999
YDR004W	YER173W	983
YDR004W	YGL163C	973
YDR004W	YJL092W	997
YDR004W	YKL113C	989
YDR004W	YML032C	997
YDR004W	YMR190C	975
YDR004W	YMR224C	993
YDR004W	YNL250W	979
YDR004W	YOR144C	984
YDR005C	YDR045C	941
YDR005C	YDR335W	918
YDR005C	YGR246C	936
YDR005C	YJL011C	941
YDR005C	YKL144C	941

YDR005C	YNL151C	941
YDR005C	YNR003C	997
YDR005C	YOR116C	999
YDR005C	YOR207C	998
YDR005C	YPR110C	997
YDR005C	YPR190C	999
YDR007W	YBR069C	983
YDR007W	YDR354W	996
YDR007W	YGL026C	992
YDR007W	YKL211C	998
YDR007W	YOL020W	999
YDR009W	YBR018C	998
YDR009W	YBR019C	988
YDR009W	YBR020W	999
YDR009W	YML051W	999
YDR009W	YPL248C	983
YDR011W	YBL005W	987
YDR011W	YDR406W	909
YDR011W	YGL013C	997
YDR011W	YGR281W	991
YDR011W	YIL013C	906
YDR011W	YML007W	930
YDR011W	YOR153W	999
YDR011W	YOR162C	954
YDR011W	YOR328W	909
YDR011W	YPL058C	910
YDR012W	YAL035W	929
YDR012W	YBL027W	999
YDR012W	YBL072C	928
YDR012W	YBL087C	997
YDR012W	YBL092W	998
YDR012W	YBR031W	999
YDR012W	YBR048W	998
YDR012W	YBR084C-A	981
YDR012W	YBR118W	920
YDR012W	YBR181C	995
YDR012W	YBR189W	989
YDR012W	YBR191W	998
YDR012W	YCR031C	982
YDR012W	YDL061C	980
YDR012W	YDL075W	999
YDR012W	YDL081C	952
YDR012W	YDL082W	997
YDR012W	YDL130W	939

YDR012W	YDL136W	999
YDR012W	YDL191W	931
YDR012W	YDR025W	998
YDR012W	YDR064W	992
YDR012W	YDR091C	900
YDR012W	YDR382W	999
YDR012W	YDR385W	929
YDR012W	YDR418W	999
YDR012W	YDR447C	996
YDR012W	YDR450W	925
YDR012W	YDR471W	998
YDR012W	YDR496C	939
YDR012W	YDR500C	946
YDR012W	YEL054C	938
YDR012W	YER056C-A	962
YDR012W	YER074W	995
YDR012W	YER102W	996
YDR012W	YER117W	979
YDR012W	YER131W	957
YDR012W	YFL034C-A	925
YDR012W	YFR031C-A	999
YDR012W	YFR032C-A	935
YDR012W	YGL030W	990
YDR012W	YGL031C	994
YDR012W	YGL076C	996
YDR012W	YGL103W	999
YDR012W	YGL123W	998
YDR012W	YGL135W	999
YDR012W	YGL147C	999
YDR012W	YGL189C	921
YDR012W	YGR027C	968
YDR012W	YGR034W	998
YDR012W	YGR085C	999
YDR012W	YGR118W	965
YDR012W	YGR148C	956
YDR012W	YGR214W	994
YDR012W	YHL001W	994
YDR012W	YHL015W	970
YDR012W	YHL033C	999
YDR012W	YHR010W	960
YDR012W	YHR021C	927
YDR012W	YHR141C	993
YDR012W	YHR203C	999
YDR012W	YIL018W	998

YDR012W	YIL052C	970
YDR012W	YIL069C	917
YDR012W	YIL133C	999
YDR012W	YIL148W	929
YDR012W	YJL136C	954
YDR012W	YJL177W	999
YDR012W	YJL189W	929
YDR012W	YJL190C	965
YDR012W	YJL191W	984
YDR012W	YJR094W-A	996
YDR012W	YJR123W	999
YDR012W	YJR145C	998
YDR012W	YKL006W	990
YDR012W	YKL156W	962
YDR012W	YKL180W	999
YDR012W	YKR057W	938
YDR012W	YKR081C	935
YDR012W	YKR094C	911
YDR012W	YLL045C	999
YDR012W	YLR029C	999
YDR012W	YLR048W	983
YDR012W	YLR061W	975
YDR012W	YLR075W	997
YDR012W	YLR167W	923
YDR012W	YLR185W	993
YDR012W	YLR264W	972
YDR012W	YLR287C-A	912
YDR012W	YLR333C	929
YDR012W	YLR340W	999
YDR012W	YLR344W	998
YDR012W	YLR367W	981
YDR012W	YLR388W	990
YDR012W	YLR406C	931
YDR012W	YLR441C	996
YDR012W	YLR448W	999
YDR012W	YML024W	968
YDR012W	YML026C	925
YDR012W	YML063W	998
YDR012W	YML073C	997
YDR012W	YMR121C	949
YDR012W	YMR142C	939
YDR012W	YMR194W	998
YDR012W	YMR230W	946
YDR012W	YMR242C	997

YDR012W	YMR260C	927
YDR012W	YMR290C	933
YDR012W	YNL061W	938
YDR012W	YNL067W	988
YDR012W	YNL069C	997
YDR012W	YNL096C	942
YDR012W	YNL162W	939
YDR012W	YNL178W	999
YDR012W	YNL301C	999
YDR012W	YNL302C	930
YDR012W	YNR037C	935
YDR012W	YOL039W	954
YDR012W	YOL040C	986
YDR012W	YOL041C	982
YDR012W	YOL120C	998
YDR012W	YOL121C	945
YDR012W	YOL127W	999
YDR012W	YOR063W	999
YDR012W	YOR096W	999
YDR012W	YOR133W	941
YDR012W	YOR167C	940
YDR012W	YOR182C	912
YDR012W	YOR234C	969
YDR012W	YOR293W	959
YDR012W	YOR312C	944
YDR012W	YOR369C	940
YDR012W	YPL079W	958
YDR012W	YPL081W	939
YDR012W	YPL090C	997
YDR012W	YPL131W	999
YDR012W	YPL143W	969
YDR012W	YPL198W	997
YDR012W	YPL220W	998
YDR012W	YPL249C-A	941
YDR012W	YPR043W	948
YDR012W	YPR080W	920
YDR012W	YPR102C	999
YDR012W	YPR132W	945
YDR013W	YDL225W	962
YDR013W	YDR489W	999
YDR013W	YGL113W	991
YDR013W	YHR107C	962
YDR013W	YJL005W	962
YDR013W	YJL072C	999

YDR013W	YJL090C	989
YDR013W	YJR076C	962
YDR013W	YLR103C	942
YDR013W	YLR274W	901
YDR013W	YOL146W	999
YDR013W	YPR135W	906
YDR013W	YPR175W	975
YDR014W	YDL003W	932
YDR014W	YFL008W	936
YDR014W	YFR027W	937
YDR014W	YJL074C	924
YDR014W-A	YER095W	997
YDR014W-A	YER179W	932
YDR016C	YBR233W-A	998
YDR016C	YDR201W	999
YDR016C	YDR320C-A	999
YDR016C	YGL061C	999
YDR016C	YGR113W	999
YDR016C	YGR188C	977
YDR016C	YIL144W	983
YDR016C	YJR135C	963
YDR016C	YKL052C	999
YDR016C	YKL138C-A	991
YDR016C	YKR037C	999
YDR016C	YKR083C	999
YDR017C	YDR099W	987
YDR017C	YDR173C	917
YDR017C	YDR315C	919
YDR017C	YLR410W	977
YDR019C	YAL044C	999
YDR019C	YBR084W	955
YDR019C	YBR263W	977
YDR019C	YFL018C	993
YDR019C	YGL125W	928
YDR019C	YGR204W	957
YDR019C	YLR058C	981
YDR019C	YMR189W	999
YDR019C	YOR074C	926
YDR019C	YPL017C	921
YDR019C	YPL023C	913
YDR020C	YNR012W	988
YDR022C	YGL180W	950
YDR022C	YKL103C	951
YDR022C	YLR423C	999

YDR022C	YMR198W	909
YDR022C	YPL166W	999
YDR023W	YBR263W	916
YDR023W	YDR064W	908
YDR023W	YGR155W	947
YDR023W	YGR208W	922
YDR023W	YGR239C	996
YDR023W	YHR011W	913
YDR025W	YBL027W	966
YDR025W	YBL072C	996
YDR025W	YBL087C	975
YDR025W	YBL092W	976
YDR025W	YBR031W	998
YDR025W	YBR048W	995
YDR025W	YBR079C	997
YDR025W	YBR084C-A	966
YDR025W	YBR118W	900
YDR025W	YBR181C	996
YDR025W	YBR189W	996
YDR025W	YBR191W	917
YDR025W	YCL011C	978
YDR025W	YCR031C	997
YDR025W	YDL014W	956
YDR025W	YDL061C	959
YDR025W	YDL075W	900
YDR025W	YDL081C	904
YDR025W	YDL082W	995
YDR025W	YDL136W	928
YDR025W	YDL191W	928
YDR025W	YDR012W	998
YDR025W	YDR064W	996
YDR025W	YDR418W	926
YDR025W	YDR429C	917
YDR025W	YDR447C	918
YDR025W	YDR450W	921
YDR025W	YDR471W	996
YDR025W	YDR500C	900
YDR025W	YEL054C	926
YDR025W	YER025W	914
YDR025W	YER074W	996
YDR025W	YER102W	995
YDR025W	YER117W	975
YDR025W	YER131W	914
YDR025W	YER165W	901

YDR025W	YFR031C-A	998
YDR025W	YFR032C-A	924
YDR025W	YGL030W	996
YDR025W	YGL031C	909
YDR025W	YGL076C	996
YDR025W	YGL103W	947
YDR025W	YGL123W	983
YDR025W	YGL135W	904
YDR025W	YGL147C	971
YDR025W	YGL189C	914
YDR025W	YGR027C	913
YDR025W	YGR034W	972
YDR025W	YGR054W	972
YDR025W	YGR085C	998
YDR025W	YGR118W	922
YDR025W	YGR145W	978
YDR025W	YGR148C	909
YDR025W	YGR214W	927
YDR025W	YHL001W	921
YDR025W	YHL015W	920
YDR025W	YHL033C	902
YDR025W	YHR021C	918
YDR025W	YHR141C	910
YDR025W	YHR203C	998
YDR025W	YIL018W	998
YDR025W	YIL069C	995
YDR025W	YJL136C	914
YDR025W	YJL177W	966
YDR025W	YJL190C	998
YDR025W	YJL191W	927
YDR025W	YJR094W-A	916
YDR025W	YJR123W	998
YDR025W	YJR145C	998
YDR025W	YKL006W	920
YDR025W	YKL156W	918
YDR025W	YKL170W	907
YDR025W	YKL180W	967
YDR025W	YKR057W	914
YDR025W	YLL045C	993
YDR025W	YLR029C	996
YDR025W	YLR048W	916
YDR025W	YLR075W	996
YDR025W	YLR150W	978
YDR025W	YLR185W	900

YDR025W	YLR325C	917
YDR025W	YLR333C	912
YDR025W	YLR340W	927
YDR025W	YLR344W	972
YDR025W	YLR367W	974
YDR025W	YLR388W	964
YDR025W	YLR406C	900
YDR025W	YLR441C	996
YDR025W	YLR448W	918
YDR025W	YML024W	918
YDR025W	YML026C	921
YDR025W	YML056C	974
YDR025W	YML063W	996
YDR025W	YML073C	916
YDR025W	YMR121C	914
YDR025W	YMR142C	912
YDR025W	YMR194W	913
YDR025W	YMR230W	918
YDR025W	YMR242C	919
YDR025W	YMR260C	912
YDR025W	YMR290C	933
YDR025W	YMR309C	997
YDR025W	YNL067W	972
YDR025W	YNL069C	995
YDR025W	YNL081C	904
YDR025W	YNL096C	929
YDR025W	YNL132W	969
YDR025W	YNL162W	910
YDR025W	YNL178W	999
YDR025W	YNL301C	920
YDR025W	YNL302C	915
YDR025W	YNL308C	979
YDR025W	YNR037C	953
YDR025W	YOL040C	989
YDR025W	YOL120C	920
YDR025W	YOL121C	915
YDR025W	YOL127W	977
YDR025W	YOR063W	999
YDR025W	YOR096W	997
YDR025W	YOR204W	962
YDR025W	YOR234C	924
YDR025W	YOR293W	918
YDR025W	YOR312C	996
YDR025W	YOR361C	997

YDR025W	YOR369C	932
YDR025W	YPL079W	916
YDR025W	YPL081W	997
YDR025W	YPL090C	994
YDR025W	YPL131W	998
YDR025W	YPL143W	924
YDR025W	YPL198W	996
YDR025W	YPL220W	904
YDR025W	YPL249C-A	914
YDR025W	YPR041W	919
YDR025W	YPR043W	916
YDR025W	YPR080W	900
YDR025W	YPR102C	999
YDR025W	YPR132W	922
YDR026C	YDR110W	991
YDR027C	YDR468C	905
YDR027C	YDR484W	999
YDR027C	YJL029C	999
YDR027C	YKR020W	999
YDR027C	YLR262C	978
YDR027C	YOL018C	996
YDR028C	YBR050C	998
YDR028C	YDR099W	999
YDR028C	YDR216W	990
YDR028C	YDR477W	999
YDR028C	YER027C	999
YDR028C	YER129W	954
YDR028C	YER133W	999
YDR028C	YER177W	999
YDR028C	YGL115W	999
YDR028C	YGL253W	973
YDR028C	YGR252W	920
YDR028C	YIL033C	909
YDR028C	YJR090C	997
YDR028C	YKL062W	942
YDR028C	YLR377C	942
YDR028C	YMR235C	975
YDR028C	YMR239C	927
YDR028C	YOR178C	942
YDR030C	YJR035W	945
YDR030C	YOR151C	935
YDR032C	YBR052C	999
YDR032C	YCR004C	999
YDR035W	YDR127W	929

YDR035W	YEL009C	925
YDR036C	Q0140	962
YDR036C	YBL090W	972
YDR036C	YBR146W	999
YDR036C	YBR251W	983
YDR036C	YDR041W	999
YDR036C	YDR175C	999
YDR036C	YDR337W	999
YDR036C	YDR347W	999
YDR036C	YGL129C	999
YDR036C	YGR084C	999
YDR036C	YHL004W	999
YDR036C	YIL093C	999
YDR036C	YJR101W	972
YDR036C	YJR113C	999
YDR036C	YKL155C	999
YDR036C	YMR188C	999
YDR036C	YNL137C	999
YDR036C	YNL306W	999
YDR036C	YPL118W	999
YDR037W	YBL076C	905
YDR037W	YDR211W	926
YDR037W	YDR283C	938
YDR037W	YDR341C	909
YDR037W	YGR083C	917
YDR037W	YGR264C	918
YDR037W	YHR020W	912
YDR037W	YHR091C	904
YDR037W	YJL124C	931
YDR037W	YLL018C	911
YDR037W	YOR168W	944
YDR037W	YPL160W	912
YDR037W	YPR056W	934
YDR038C	YDR040C	910
YDR040C	YDR038C	910
YDR040C	YDR436W	979
YDR040C	YDR456W	954
YDR040C	YLR019W	904
YDR040C	YLR138W	958
YDR040C	YML006C	928
YDR040C	YML016C	984
YDR040C	YPR005C	914
YDR041W	Q0140	962
YDR041W	YBL087C	967

YDR041W	YBL090W	988
YDR041W	YBR118W	924
YDR041W	YBR146W	999
YDR041W	YBR251W	999
YDR041W	YCR031C	957
YDR041W	YDR036C	999
YDR041W	YDR175C	988
YDR041W	YDR337W	998
YDR041W	YDR347W	996
YDR041W	YDR450W	912
YDR041W	YER050C	986
YDR041W	YER117W	967
YDR041W	YFR031C-A	961
YDR041W	YGL123W	983
YDR041W	YGL129C	998
YDR041W	YGR084C	995
YDR041W	YGR085C	965
YDR041W	YGR165W	988
YDR041W	YGR215W	988
YDR041W	YHL004W	999
YDR041W	YHL015W	929
YDR041W	YIL018W	961
YDR041W	YIL093C	988
YDR041W	YJL190C	900
YDR041W	YJL191W	952
YDR041W	YJR101W	999
YDR041W	YJR113C	999
YDR041W	YJR123W	966
YDR041W	YKL155C	999
YDR041W	YKL170W	976
YDR041W	YLR048W	901
YDR041W	YLR367W	900
YDR041W	YML026C	912
YDR041W	YMR158W	974
YDR041W	YMR188C	998
YDR041W	YNL081C	995
YDR041W	YNL137C	996
YDR041W	YNL178W	997
YDR041W	YNL306W	999
YDR041W	YNR036C	990
YDR041W	YNR037C	999
YDR041W	YOR158W	988
YDR041W	YOR204W	987
YDR041W	YPL013C	998

YDR041W	YPL118W	999
YDR041W	YPR080W	924
YDR041W	YPR102C	966
YDR043C	YDR477W	996
YDR043C	YHL027W	970
YDR043C	YIL035C	932
YDR043C	YOR061W	941
YDR044W	YDR047W	989
YDR044W	YER014W	931
YDR044W	YGL112C	930
YDR044W	YPR065W	945
YDR045C	YBR154C	997
YDR045C	YDL150W	998
YDR045C	YDR005C	941
YDR045C	YDR156W	921
YDR045C	YHR143W-A	960
YDR045C	YJL011C	999
YDR045C	YJL148W	921
YDR045C	YJR063W	931
YDR045C	YKL144C	998
YDR045C	YKR025W	998
YDR045C	YNL113W	998
YDR045C	YNL151C	998
YDR045C	YNL248C	919
YDR045C	YNR003C	999
YDR045C	YOR116C	999
YDR045C	YOR207C	999
YDR045C	YOR210W	901
YDR045C	YOR224C	997
YDR045C	YOR340C	921
YDR045C	YOR341W	929
YDR045C	YPR010C	928
YDR045C	YPR110C	965
YDR045C	YPR187W	902
YDR045C	YPR190C	998
YDR046C	YAL043C	940
YDR047W	YDL205C	975
YDR047W	YDR044W	989
YDR047W	YGL040C	976
YDR047W	YKR069W	905
YDR047W	YNL244C	959
YDR047W	YOR176W	961
YDR047W	YOR278W	904
YDR047W	YPL237W	911

YDR049W	YDL126C	998
YDR050C	YAL038W	930
YDR050C	YBR117C	905
YDR050C	YBR196C	987
YDR050C	YCR012W	998
YDR050C	YDL085W	902
YDR050C	YGR192C	993
YDR050C	YGR254W	922
YDR050C	YHR174W	942
YDR050C	YJL052W	989
YDR050C	YJR009C	989
YDR050C	YKL060C	999
YDR050C	YMR323W	914
YDR050C	YOL086C	970
YDR050C	YOR347C	930
YDR050C	YOR393W	914
YDR050C	YPR074C	906
YDR051C	YBR084C-A	940
YDR051C	YER048W-A	935
YDR051C	YER117W	956
YDR051C	YFL038C	956
YDR051C	YGR193C	998
YDR051C	YLR167W	938
YDR051C	YPR132W	998
YDR052C	YBL023C	999
YDR052C	YBR060C	999
YDR052C	YBR136W	915
YDR052C	YBR202W	976
YDR052C	YDL017W	999
YDR052C	YEL032W	981
YDR052C	YLL004W	995
YDR052C	YLR274W	999
YDR052C	YMR001C	985
YDR052C	YPL153C	999
YDR052C	YPR019W	983
YDR052C	YPR120C	908
YDR054C	YBR165W	989
YDR054C	YBR280C	923
YDR054C	YDL132W	999
YDR054C	YDR328C	999
YDR054C	YER125W	962
YDR054C	YFL009W	999
YDR054C	YGL252C	999
YDR054C	YIL046W	999

YDR054C	YJL047C	998
YDR054C	YJL157C	950
YDR054C	YJL187C	973
YDR054C	YJL194W	947
YDR054C	YJR090C	999
YDR054C	YLL021W	964
YDR054C	YLL039C	980
YDR054C	YLR079W	999
YDR054C	YMR199W	918
YDR054C	YNL098C	966
YDR054C	YOL133W	999
YDR054C	YOR080W	979
YDR054C	YPL003W	901
YDR054C	YPL256C	938
YDR057W	YBR201W	978
YDR057W	YCR067C	923
YDR057W	YDR144C	911
YDR057W	YER157W	907
YDR057W	YHR204W	956
YDR057W	YJL034W	991
YDR057W	YLR207W	999
YDR057W	YML013W	931
YDR057W	YML029W	983
YDR057W	YMR297W	995
YDR057W	YOL013C	997
YDR059C	YBR082C	999
YDR059C	YDL140C	967
YDR059C	YER068W	945
YDR059C	YER125W	977
YDR059C	YKL010C	915
YDR059C	YLL039C	905
YDR059C	YOR339C	904
YDR059C	YPR103W	999
YDR060W	YAL035W	941
YDR060W	YBR142W	998
YDR060W	YDL014W	991
YDR060W	YDL031W	984
YDR060W	YDR087C	998
YDR060W	YDR496C	972
YDR060W	YER006W	948
YDR060W	YFL002C	976
YDR060W	YGL076C	938
YDR060W	YGL111W	978
YDR060W	YGR103W	999

YDR060W	YGR195W	914
YDR060W	YHR052W	999
YDR060W	YHR066W	986
YDR060W	YHR088W	978
YDR060W	YIL133C	939
YDR060W	YKL172W	990
YDR060W	YKR024C	966
YDR060W	YKR081C	994
YDR060W	YLL008W	978
YDR060W	YLL034C	976
YDR060W	YLL045C	941
YDR060W	YLR002C	999
YDR060W	YLR029C	940
YDR060W	YLR221C	914
YDR060W	YLR276C	998
YDR060W	YLR398C	984
YDR060W	YMR049C	998
YDR060W	YMR229C	999
YDR060W	YMR290C	997
YDR060W	YNL002C	998
YDR060W	YNL061W	997
YDR060W	YNL110C	981
YDR060W	YNL301C	936
YDR060W	YOL041C	998
YDR060W	YOL077C	999
YDR060W	YOR063W	944
YDR060W	YOR206W	999
YDR060W	YOR272W	999
YDR060W	YPL012W	997
YDR060W	YPL043W	999
YDR060W	YPL093W	952
YDR060W	YPL131W	959
YDR060W	YPL211W	999
YDR062W	YBR017C	962
YDR062W	YBR036C	982
YDR062W	YBR058C-A	998
YDR062W	YDR099W	917
YDR062W	YER177W	966
YDR062W	YMR296C	999
YDR062W	YOR171C	903
YDR064W	YAL003W	989
YDR064W	YAL035W	968
YDR064W	YBL004W	935
YDR064W	YBL027W	996

YDR064W	YBL072C	998
YDR064W	YBL087C	956
YDR064W	YBL092W	999
YDR064W	YBR031W	984
YDR064W	YBR048W	999
YDR064W	YBR079C	995
YDR064W	YBR084C-A	985
YDR064W	YBR118W	929
YDR064W	YBR181C	959
YDR064W	YBR189W	999
YDR064W	YBR191W	999
YDR064W	YBR247C	990
YDR064W	YCL011C	929
YDR064W	YCR031C	999
YDR064W	YCR057C	980
YDR064W	YDL014W	968
YDR064W	YDL060W	937
YDR064W	YDL061C	999
YDR064W	YDL075W	999
YDR064W	YDL081C	972
YDR064W	YDL082W	997
YDR064W	YDL083C	997
YDR064W	YDL130W	954
YDR064W	YDL136W	998
YDR064W	YDL148C	933
YDR064W	YDL191W	944
YDR064W	YDL192W	966
YDR064W	YDR012W	992
YDR064W	YDR023W	908
YDR064W	YDR025W	996
YDR064W	YDR382W	999
YDR064W	YDR385W	992
YDR064W	YDR418W	999
YDR064W	YDR429C	906
YDR064W	YDR447C	997
YDR064W	YDR449C	933
YDR064W	YDR450W	995
YDR064W	YDR471W	999
YDR064W	YDR500C	985
YDR064W	YEL054C	953
YDR064W	YER025W	968
YDR064W	YER056C-A	999
YDR064W	YER074W	995
YDR064W	YER082C	931

YDR064W	YER102W	999
YDR064W	YER117W	987
YDR064W	YER131W	980
YDR064W	YER165W	955
YDR064W	YFL034C-A	928
YDR064W	YFR031C-A	996
YDR064W	YFR032C-A	973
YDR064W	YGL014W	944
YDR064W	YGL030W	999
YDR064W	YGL031C	964
YDR064W	YGL076C	984
YDR064W	YGL103W	999
YDR064W	YGL120C	940
YDR064W	YGL123W	999
YDR064W	YGL135W	995
YDR064W	YGL147C	997
YDR064W	YGL189C	941
YDR064W	YGR027C	997
YDR064W	YGR034W	996
YDR064W	YGR054W	976
YDR064W	YGR085C	999
YDR064W	YGR090W	981
YDR064W	YGR118W	999
YDR064W	YGR145W	929
YDR064W	YGR148C	997
YDR064W	YGR162W	926
YDR064W	YGR214W	999
YDR064W	YHL001W	999
YDR064W	YHL015W	999
YDR064W	YHL033C	999
YDR064W	YHL034C	935
YDR064W	YHR010W	985
YDR064W	YHR021C	956
YDR064W	YHR141C	996
YDR064W	YHR148W	933
YDR064W	YHR203C	999
YDR064W	YIL018W	995
YDR064W	YIL052C	999
YDR064W	YIL069C	997
YDR064W	YIL133C	996
YDR064W	YIL148W	978
YDR064W	YJL136C	985
YDR064W	YJL138C	904
YDR064W	YJL177W	999

YDR064W	YJL189W	981
YDR064W	YJL190C	999
YDR064W	YJL191W	999
YDR064W	YJR002W	935
YDR064W	YJR094W-A	998
YDR064W	YJR123W	999
YDR064W	YJR145C	996
YDR064W	YKL006W	999
YDR064W	YKL156W	997
YDR064W	YKL180W	999
YDR064W	YKR057W	958
YDR064W	YKR059W	967
YDR064W	YKR094C	916
YDR064W	YLL011W	929
YDR064W	YLL045C	997
YDR064W	YLR029C	999
YDR064W	YLR048W	999
YDR064W	YLR060W	979
YDR064W	YLR061W	999
YDR064W	YLR075W	999
YDR064W	YLR129W	931
YDR064W	YLR167W	969
YDR064W	YLR185W	985
YDR064W	YLR186W	943
YDR064W	YLR197W	948
YDR064W	YLR249W	954
YDR064W	YLR264W	999
YDR064W	YLR287C-A	993
YDR064W	YLR325C	965
YDR064W	YLR333C	963
YDR064W	YLR340W	999
YDR064W	YLR344W	997
YDR064W	YLR367W	999
YDR064W	YLR388W	999
YDR064W	YLR406C	940
YDR064W	YLR432W	982
YDR064W	YLR441C	999
YDR064W	YLR448W	999
YDR064W	YML024W	999
YDR064W	YML026C	998
YDR064W	YML063W	999
YDR064W	YML073C	997
YDR064W	YMR093W	933
YDR064W	YMR116C	993

YDR064W	YMR121C	941
YDR064W	YMR128W	931
YDR064W	YMR142C	998
YDR064W	YMR194W	999
YDR064W	YMR230W	999
YDR064W	YMR242C	997
YDR064W	YMR260C	933
YDR064W	YNL067W	997
YDR064W	YNL069C	996
YDR064W	YNL096C	997
YDR064W	YNL132W	949
YDR064W	YNL162W	948
YDR064W	YNL178W	999
YDR064W	YNL207W	980
YDR064W	YNL209W	970
YDR064W	YNL301C	999
YDR064W	YNL302C	949
YDR064W	YNL308C	981
YDR064W	YOL039W	953
YDR064W	YOL040C	999
YDR064W	YOL120C	949
YDR064W	YOL121C	967
YDR064W	YOL127W	999
YDR064W	YOR063W	999
YDR064W	YOR096W	999
YDR064W	YOR133W	954
YDR064W	YOR145C	980
YDR064W	YOR167C	983
YDR064W	YOR182C	903
YDR064W	YOR234C	998
YDR064W	YOR293W	987
YDR064W	YOR310C	993
YDR064W	YOR312C	998
YDR064W	YOR361C	932
YDR064W	YOR369C	975
YDR064W	YPL012W	936
YDR064W	YPL037C	999
YDR064W	YPL079W	991
YDR064W	YPL081W	999
YDR064W	YPL090C	996
YDR064W	YPL126W	933
YDR064W	YPL131W	999
YDR064W	YPL143W	999
YDR064W	YPL198W	997

YDR064W	YPL217C	942
YDR064W	YPL220W	998
YDR064W	YPL237W	927
YDR064W	YPL249C-A	998
YDR064W	YPL266W	944
YDR064W	YPR043W	974
YDR064W	YPR080W	929
YDR064W	YPR102C	998
YDR064W	YPR132W	988
YDR064W	YPR144C	981
YDR064W	YPR163C	911
YDR064W	YPR189W	981
YDR067C	YHL029C	976
YDR068W	YDR483W	958
YDR069C	YBL064C	919
YDR069C	YDL147W	998
YDR069C	YKL002W	981
YDR069C	YKL041W	995
YDR069C	YKR035W-A	931
YDR069C	YLL039C	993
YDR069C	YLR025W	999
YDR069C	YMR077C	912
YDR069C	YNR006W	972
YDR069C	YPL084W	999
YDR069C	YPL154C	997
YDR069C	YPR103W	999
YDR069C	YPR173C	988
YDR071C	YBL056W	999
YDR071C	YBR125C	999
YDR071C	YER089C	999
YDR071C	YPL153C	921
YDR072C	YBR036C	941
YDR073W	YBR289W	999
YDR073W	YDL147W	998
YDR073W	YFL049W	956
YDR073W	YHL025W	999
YDR073W	YJL176C	999
YDR073W	YMR033W	992
YDR073W	YNR023W	998
YDR073W	YOR290C	999
YDR073W	YPL016W	992
YDR073W	YPL129W	977
YDR073W	YPR034W	998
YDR074W	YBR001C	939

YDR074W	YBR126C	999
YDR074W	YDR001C	964
YDR074W	YKL035W	934
YDR074W	YKL128C	912
YDR074W	YLR258W	948
YDR074W	YML100W	999
YDR074W	YMR261C	999
YDR074W	YPR026W	924
YDR075W	YBL002W	926
YDR075W	YBL003C	926
YDR075W	YBL046W	999
YDR075W	YCL061C	950
YDR075W	YDL101C	926
YDR075W	YDR224C	936
YDR075W	YDR225W	936
YDR075W	YDR293C	926
YDR075W	YER089C	944
YDR075W	YHR031C	953
YDR075W	YIL153W	996
YDR075W	YKL113C	910
YDR075W	YML010W	999
YDR075W	YML032C	932
YDR075W	YMR028W	997
YDR075W	YMR048W	905
YDR075W	YNL201C	999
YDR075W	YPL153C	917
YDR075W	YPR040W	936
YDR076W	YAR002W	926
YDR076W	YBR052C	904
YDR076W	YBR073W	966
YDR076W	YBR136W	970
YDR076W	YDL059C	971
YDR076W	YDR004W	999
YDR076W	YDR217C	965
YDR076W	YDR369C	969
YDR076W	YER095W	999
YDR076W	YGL058W	911
YDR076W	YGL090W	921
YDR076W	YGL163C	989
YDR076W	YJL092W	931
YDR076W	YJR043C	963
YDR076W	YKL113C	988
YDR076W	YKR082W	973
YDR076W	YLR032W	919

YDR076W	YLR234W	978
YDR076W	YML032C	998
YDR076W	YMR224C	964
YDR076W	YNL250W	972
YDR076W	YOR005C	910
YDR076W	YOR144C	982
YDR076W	YPL020C	966
YDR076W	YPL153C	992
YDR076W	YPL167C	905
YDR077W	YBL040C	901
YDR077W	YDL226C	976
YDR077W	YEL065W	975
YDR077W	YGL028C	920
YDR077W	YGR279C	909
YDR077W	YLR075W	974
YDR078C	YHL006C	994
YDR078C	YLR234W	913
YDR078C	YLR376C	999
YDR078C	YMR190C	978
YDR079C-A	YDR311W	988
YDR079C-A	YGL070C	939
YDR079C-A	YPL122C	999
YDR079C-A	YPR056W	996
YDR080W	YDL077C	999
YDR080W	YGL212W	986
YDR080W	YGR013W	950
YDR080W	YLR148W	999
YDR080W	YLR396C	999
YDR080W	YML001W	941
YDR080W	YMR197C	934
YDR080W	YMR231W	999
YDR080W	YOR089C	919
YDR080W	YOR106W	997
YDR080W	YPL045W	999
YDR080W	YPL195W	976
YDR081C	YDL080C	946
YDR081C	YLR044C	955
YDR081C	YLR134W	937
YDR082W	YBL035C	998
YDR082W	YDL220C	999
YDR082W	YDR217C	918
YDR082W	YIL009C-A	916
YDR082W	YLR010C	999
YDR082W	YLR318W	999

YDR082W	YNL102W	969
YDR083W	YLR347C	940
YDR083W	YNL189W	958
YDR084C	YGL161C	999
YDR084C	YGL198W	999
YDR085C	YFL026W	924
YDR085C	YHR107C	999
YDR085C	YPL242C	905
YDR086C	YBR171W	996
YDR086C	YBR283C	977
YDR086C	YEL002C	972
YDR086C	YER019C-A	993
YDR086C	YER087C-B	999
YDR086C	YJL034W	921
YDR086C	YLR292C	917
YDR086C	YLR378C	999
YDR086C	YMR149W	982
YDR086C	YOR254C	979
YDR086C	YPL094C	946
YDR087C	YAL007C	925
YDR087C	YCR018C	992
YDR087C	YDL031W	998
YDR087C	YDR060W	998
YDR087C	YDR496C	979
YDR087C	YER006W	976
YDR087C	YFL002C	974
YDR087C	YGL111W	996
YDR087C	YGR085C	923
YDR087C	YGR103W	992
YDR087C	YHR052W	999
YDR087C	YHR066W	971
YDR087C	YHR088W	995
YDR087C	YKL009W	976
YDR087C	YKR081C	999
YDR087C	YLL008W	996
YDR087C	YLL034C	974
YDR087C	YMR049C	995
YDR087C	YMR290C	992
YDR087C	YNL002C	996
YDR087C	YNL061W	999
YDR087C	YNL110C	952
YDR087C	YOL077C	998
YDR087C	YOR063W	994
YDR087C	YOR206W	995

YDR087C	YOR272W	998
YDR087C	YPL093W	936
YDR087C	YPL211W	995
YDR087C	YPR016C	999
YDR088C	YBR065C	997
YDR088C	YDR364C	999
YDR088C	YER013W	953
YDR088C	YER172C	924
YDR088C	YGR006W	999
YDR088C	YHR165C	998
YDR088C	YKR086W	999
YDR088C	YLR117C	932
YDR091C	YAL035W	974
YDR091C	YBR079C	994
YDR091C	YDR012W	900
YDR091C	YLR192C	999
YDR091C	YLR340W	957
YDR091C	YML063W	914
YDR091C	YMR146C	968
YDR091C	YMR309C	999
YDR091C	YNL260C	974
YDR091C	YOR096W	985
YDR091C	YPR041W	997
YDR092W	YBR088C	983
YDR092W	YCR066W	999
YDR092W	YDR419W	959
YDR092W	YER125W	933
YDR092W	YGL087C	999
YDR092W	YIL139C	901
YDR092W	YJL092W	973
YDR092W	YLR032W	999
YDR092W	YPL167C	996
YDR093W	YER166W	983
YDR093W	YNL323W	997
YDR096W	YFL023W	929
YDR096W	YFL033C	954
YDR096W	YHR205W	922
YDR096W	YLR310C	971
YDR096W	YMR219W	905
YDR096W	YOR156C	900
YDR097C	YAR007C	986
YDR097C	YBR088C	999
YDR097C	YBR114W	919
YDR097C	YBR136W	968

YDR097C	YCR028C-A	958
YDR097C	YCR092C	999
YDR097C	YDL102W	982
YDR097C	YDL156W	934
YDR097C	YDR499W	912
YDR097C	YIR002C	984
YDR097C	YJL173C	933
YDR097C	YML032C	976
YDR097C	YML060W	997
YDR097C	YMR167W	999
YDR097C	YNL082W	998
YDR097C	YNL262W	908
YDR097C	YNL312W	985
YDR097C	YOL090W	999
YDR097C	YOR033C	955
YDR097C	YPL022W	924
YDR097C	YPL164C	991
YDR098C	YGL071W	999
YDR098C	YGL220W	996
YDR098C	YGR262C	941
YDR098C	YIL128W	996
YDR099W	YAL017W	974
YDR099W	YBL103C	953
YDR099W	YBR001C	963
YDR099W	YBR050C	936
YDR099W	YCL051W	961
YDR099W	YDL117W	922
YDR099W	YDR001C	999
YDR099W	YDR017C	987
YDR099W	YDR028C	999
YDR099W	YDR062W	917
YDR099W	YDR130C	999
YDR099W	YDR164C	948
YDR099W	YDR309C	954
YDR099W	YDR373W	976
YDR099W	YDR477W	973
YDR099W	YER040W	945
YDR099W	YER133W	946
YDR099W	YER177W	999
YDR099W	YFL021W	937
YDR099W	YFL039C	903
YDR099W	YGL003C	992
YDR099W	YGL206C	922
YDR099W	YGL252C	999

YDR099W	YHL007C	932
YDR099W	YJL141C	991
YDR099W	YJL164C	962
YDR099W	YJR090C	938
YDR099W	YKL062W	996
YDR099W	YKL168C	940
YDR099W	YLR177W	986
YDR099W	YLR310C	974
YDR099W	YMR037C	996
YDR099W	YNL042W	982
YDR099W	YNL199C	938
YDR099W	YNL267W	999
YDR099W	YNL293W	957
YDR099W	YPL256C	962
YDR099W	YPL267W	977
YDR100W	YAL007C	923
YDR100W	YLR039C	961
YDR100W	YLR262C	921
YDR100W	YMR071C	952
YDR100W	YPL020C	906
YDR101C	YBR031W	940
YDR101C	YBR267W	999
YDR101C	YCR072C	993
YDR101C	YDL031W	989
YDR101C	YDL075W	983
YDR101C	YDR192C	956
YDR101C	YER006W	999
YDR101C	YER036C	967
YDR101C	YER107C	933
YDR101C	YER126C	999
YDR101C	YGL030W	935
YDR101C	YGL099W	999
YDR101C	YGL103W	940
YDR101C	YGR103W	996
YDR101C	YGR245C	999
YDR101C	YHR052W	996
YDR101C	YHR170W	999
YDR101C	YHR197W	996
YDR101C	YJL122W	999
YDR101C	YKL009W	992
YDR101C	YKL068W	984
YDR101C	YKR081C	998
YDR101C	YLL045C	996
YDR101C	YLR009W	984

YDR101C	YLR074C	939
YDR101C	YLR106C	977
YDR101C	YLR397C	940
YDR101C	YMR308C	972
YDR101C	YNL002C	994
YDR101C	YNL110C	998
YDR101C	YNL182C	993
YDR101C	YNL227C	951
YDR101C	YNL248C	938
YDR101C	YNR053C	985
YDR101C	YOR063W	993
YDR101C	YOR312C	940
YDR101C	YPL093W	999
YDR101C	YPL169C	985
YDR101C	YPL211W	987
YDR101C	YPL249C-A	983
YDR101C	YPR016C	998
YDR103W	YAL041W	998
YDR103W	YBL016W	999
YDR103W	YBR200W	998
YDR103W	YCL027W	999
YDR103W	YCL032W	998
YDR103W	YCR093W	982
YDR103W	YDL159W	999
YDR103W	YFL026W	994
YDR103W	YGR040W	999
YDR103W	YHL007C	999
YDR103W	YHR005C	993
YDR103W	YHR084W	989
YDR103W	YJL157C	985
YDR103W	YJR086W	999
YDR103W	YKL178C	920
YDR103W	YLR113W	936
YDR103W	YLR362W	999
YDR103W	YLR452C	978
YDR103W	YOR212W	999
YDR103W	YOR326W	971
YDR105C	YOL129W	927
YDR106W	YHR129C	982
YDR106W	YPL174C	976
YDR108W	YBR254C	999
YDR108W	YCR014C	966
YDR108W	YDR246W	999
YDR108W	YDR407C	999

YDR108W	YDR472W	999
YDR108W	YEL043W	921
YDR108W	YGR166W	992
YDR108W	YGR234W	901
YDR108W	YKR068C	999
YDR108W	YML077W	999
YDR108W	YMR010W	911
YDR108W	YMR218C	999
YDR108W	YNL044W	903
YDR108W	YOR115C	999
YDR108W	YOR259C	970
YDR110W	YDL042C	999
YDR110W	YDR026C	991
YDR110W	YFR028C	990
YDR110W	YHR031C	910
YDR110W	YHR152W	958
YDR110W	YHR164C	987
YDR110W	YJL076W	999
YDR110W	YKR010C	932
YDR110W	YMR190C	975
YDR111C	YBR218C	905
YDR111C	YGL062W	905
YDR111C	YJL101C	907
YDR111C	YLR089C	903
YDR111C	YPR035W	930
YDR113C	YBL084C	980
YDR113C	YBL088C	926
YDR113C	YBR136W	954
YDR113C	YBR160W	986
YDR113C	YBR274W	999
YDR113C	YCR065W	912
YDR113C	YDL003W	993
YDR113C	YDL101C	998
YDR113C	YDL220C	975
YDR113C	YEL061C	983
YDR113C	YER143W	946
YDR113C	YER173W	933
YDR113C	YFR036W	928
YDR113C	YGL003C	998
YDR113C	YGL116W	999
YDR113C	YGR098C	999
YDR113C	YGR225W	999
YDR113C	YHR166C	997
YDR113C	YJL187C	925

YDR113C	YKL022C	998
YDR113C	YLR102C	955
YDR113C	YLR127C	982
YDR113C	YMR001C	960
YDR113C	YOR058C	918
YDR113C	YPL153C	998
YDR113C	YPR119W	998
YDR113C	YPR120C	968
YDR115W	YKL142W	959
YDR116C	YBL038W	995
YDR116C	YBR122C	974
YDR116C	YBR282W	970
YDR116C	YCR046C	999
YDR116C	YCR071C	996
YDR116C	YDR237W	992
YDR116C	YDR296W	992
YDR116C	YDR322W	974
YDR116C	YDR405W	987
YDR116C	YDR462W	972
YDR116C	YGL068W	930
YDR116C	YGR220C	999
YDR116C	YHL004W	913
YDR116C	YHR147C	995
YDR116C	YJL041W	930
YDR116C	YJL063C	996
YDR116C	YKL167C	972
YDR116C	YKR006C	972
YDR116C	YKR085C	972
YDR116C	YLR312W-A	988
YDR116C	YLR439W	988
YDR116C	YML025C	994
YDR116C	YMR024W	988
YDR116C	YMR193W	974
YDR116C	YNL005C	989
YDR116C	YNL185C	999
YDR116C	YNL252C	974
YDR116C	YNL284C	983
YDR116C	YNR036C	912
YDR116C	YOR150W	993
YDR117C	YDL014W	940
YDR117C	YHR203C	940
YDR117C	YJR145C	940
YDR117C	YOR204W	927
YDR118W	YBL084C	999

YDR118W	YDL008W	999
YDR118W	YDR260C	999
YDR118W	YFR036W	999
YDR118W	YGL240W	999
YDR118W	YHR166C	999
YDR118W	YIR025W	999
YDR118W	YKL022C	999
YDR118W	YLR102C	999
YDR118W	YLR127C	999
YDR118W	YNL172W	999
YDR118W	YOR249C	999
YDR118W	YPR119W	968
YDR120C	YDL051W	924
YDR120C	YGL111W	962
YDR120C	YNR046W	918
YDR120C	YOL093W	902
YDR120C	YOL124C	984
YDR121W	YBL002W	986
YDR121W	YBR278W	999
YDR121W	YGL133W	999
YDR121W	YJL065C	999
YDR121W	YJL090C	900
YDR121W	YNL262W	999
YDR121W	YOR304W	999
YDR121W	YPR175W	999
YDR122W	YIL118W	979
YDR122W	YLR229C	968
YDR123C	YDR207C	918
YDR123C	YFL031W	938
YDR123C	YGL238W	983
YDR123C	YHL020C	999
YDR123C	YJL153C	997
YDR123C	YKL135C	982
YDR123C	YOL108C	999
YDR123C	YPR086W	978
YDR127W	YBR249C	919
YDR127W	YDR035W	929
YDR127W	YER095W	902
YDR127W	YGL148W	915
YDR127W	YGR234W	920
YDR127W	YGR240C	999
YDR127W	YKL104C	992
YDR127W	YLR180W	973
YDR127W	YMR186W	996

YDR128W	YLR208W	900
YDR129C	YBR085W	919
YDR129C	YCR088W	999
YDR129C	YDL226C	933
YDR129C	YDR353W	905
YDR129C	YER155C	908
YDR129C	YFL039C	999
YDR129C	YIL034C	997
YDR129C	YJL020C	961
YDR129C	YKL007W	955
YDR129C	YLR429W	985
YDR129C	YNL243W	992
YDR129C	YOR181W	919
YDR129C	YOR367W	998
YDR130C	YBR160W	965
YDR130C	YDR099W	999
YDR130C	YER133W	994
YDR130C	YER177W	985
YDR131C	YDL132W	969
YDR135C	YLL015W	996
YDR135C	YLR425W	931
YDR135C	YPR201W	998
YDR137W	YHR135C	909
YDR137W	YLR039C	999
YDR137W	YLR262C	975
YDR137W	YNL154C	909
YDR138W	YAL021C	929
YDR138W	YBR279W	999
YDR138W	YCL011C	999
YDR138W	YDL084W	999
YDR138W	YDR381W	999
YDR138W	YER063W	987
YDR138W	YGL025C	920
YDR138W	YGL127C	996
YDR138W	YGR063C	930
YDR138W	YGR104C	926
YDR138W	YHR041C	994
YDR138W	YHR167W	999
YDR138W	YJR052W	967
YDR138W	YKR010C	908
YDR138W	YLR418C	966
YDR138W	YML062C	999
YDR138W	YMR125W	963
YDR138W	YNL004W	999

YDR138W	YNL139C	999
YDR138W	YNL253W	999
YDR138W	YNR016C	930
YDR138W	YOL006C	999
YDR138W	YOL051W	947
YDR138W	YOR001W	995
YDR138W	YOR151C	973
YDR138W	YPL169C	998
YDR139C	YDL132W	999
YDR139C	YDR328C	991
YDR139C	YGR003W	912
YDR139C	YJL047C	910
YDR139C	YJR099W	905
YDR139C	YLR306W	988
YDR139C	YPR066W	947
YDR140W	YBR143C	926
YDR140W	YNR046W	999
YDR141C	YNL297C	999
YDR142C	YDR244W	999
YDR142C	YDR265W	926
YDR142C	YGL153W	999
YDR142C	YGR077C	924
YDR142C	YGR239C	999
YDR142C	YHR160C	999
YDR142C	YIL160C	999
YDR142C	YJL210W	956
YDR142C	YLR191W	999
YDR142C	YMR026C	932
YDR142C	YNL214W	999
YDR143C	YDL220C	990
YDR143C	YDR227W	998
YDR143C	YGL207W	998
YDR143C	YLL039C	913
YDR143C	YLR442C	985
YDR143C	YPR019W	929
YDR144C	YDR057W	911
YDR144C	YLR120C	996
YDR144C	YNL238W	998
YDR145W	YBR081C	999
YDR145W	YBR111W-A	992
YDR145W	YBR198C	999
YDR145W	YCL010C	999
YDR145W	YCR042C	991
YDR145W	YDR167W	999

YDR145W	YDR176W	999
YDR145W	YDR392W	999
YDR145W	YDR448W	999
YDR145W	YEL009C	912
YDR145W	YER148W	999
YDR145W	YER164W	980
YDR145W	YGL066W	999
YDR145W	YGL112C	999
YDR145W	YGL127C	909
YDR145W	YGL150C	923
YDR145W	YGR252W	999
YDR145W	YGR274C	999
YDR145W	YHR041C	921
YDR145W	YHR099W	999
YDR145W	YLR055C	999
YDR145W	YLR182W	923
YDR145W	YML015C	999
YDR145W	YML098W	999
YDR145W	YML114C	999
YDR145W	YMR005W	999
YDR145W	YMR223W	999
YDR145W	YMR227C	999
YDR145W	YMR236W	999
YDR145W	YNL236W	921
YDR145W	YNR010W	954
YDR145W	YOL051W	977
YDR145W	YOL148C	999
YDR145W	YOR174W	969
YDR145W	YPL011C	999
YDR145W	YPL047W	999
YDR145W	YPL129W	994
YDR145W	YPL248C	992
YDR145W	YPL254W	999
YDR145W	YPR070W	909
YDR145W	YPR072W	983
YDR145W	YPR086W	978
YDR146C	YBR160W	992
YDR146C	YDL106C	999
YDR146C	YDL127W	942
YDR146C	YDL155W	962
YDR146C	YDL179W	918
YDR146C	YDR335W	972
YDR146C	YER022W	973
YDR146C	YER111C	977

YDR146C	YFR028C	998
YDR146C	YGL003C	999
YDR146C	YGR108W	960
YDR146C	YIL131C	975
YDR146C	YJL194W	999
YDR146C	YLR079W	999
YDR146C	YLR131C	965
YDR146C	YLR286C	923
YDR146C	YMR043W	930
YDR146C	YNL068C	978
YDR146C	YNL327W	925
YDR146C	YNL330C	976
YDR146C	YOL001W	937
YDR146C	YOL004W	999
YDR146C	YOR290C	996
YDR146C	YPL031C	996
YDR146C	YPR119W	999
YDR148C	YDR510W	901
YDR148C	YFL018C	999
YDR148C	YFR049W	990
YDR148C	YGR244C	993
YDR148C	YIL125W	999
YDR148C	YMR072W	909
YDR148C	YOR142W	993
YDR148C	YPL017C	972
YDR150W	YDR488C	985
YDR150W	YEL061C	975
YDR150W	YER016W	995
YDR150W	YER120W	934
YDR150W	YGL216W	973
YDR150W	YLL001W	938
YDR150W	YML085C	922
YDR150W	YML124C	901
YDR150W	YNL271C	980
YDR150W	YPL269W	999
YDR151C	YJR017C	969
YDR152W	YAL036C	976
YDR152W	YDR365C	940
YDR152W	YGL195W	913
YDR152W	YGR173W	999
YDR152W	YPR106W	938
YDR153C	YBR023C	973
YDR153C	YGL206C	995
YDR153C	YHR108W	999

YDR153C	YJR125C	997
YDR153C	YKL135C	960
YDR153C	YNR006W	905
YDR155C	YBR103W	999
YDR155C	YCR033W	999
YDR155C	YDR304C	927
YDR155C	YGL194C	999
YDR155C	YHR057C	917
YDR155C	YIL112W	999
YDR155C	YJL106W	954
YDR155C	YJL168C	952
YDR155C	YJR017C	981
YDR155C	YKR029C	999
YDR155C	YML078W	977
YDR155C	YOL068C	996
YDR156W	YBR154C	998
YDR156W	YDL150W	921
YDR156W	YDR045C	921
YDR156W	YHR143W-A	960
YDR156W	YJL148W	999
YDR156W	YJR063W	999
YDR156W	YKL144C	921
YDR156W	YNL113W	998
YDR156W	YNL151C	921
YDR156W	YNL248C	999
YDR156W	YNR003C	921
YDR156W	YOL006C	959
YDR156W	YOR116C	921
YDR156W	YOR207C	921
YDR156W	YOR210W	998
YDR156W	YOR340C	999
YDR156W	YOR341W	999
YDR156W	YPR010C	998
YDR156W	YPR110C	998
YDR156W	YPR187W	998
YDR156W	YPR190C	921
YDR158W	YER052C	996
YDR158W	YJR139C	997
YDR159W	YAR002W	989
YDR159W	YBL079W	968
YDR159W	YBR111W-A	999
YDR159W	YDL084W	941
YDR159W	YDL088C	966
YDR159W	YDL116W	969

YDR159W	YDL207W	966
YDR159W	YDR192C	905
YDR159W	YDR381W	941
YDR159W	YDR448W	935
YDR159W	YER105C	966
YDR159W	YER107C	993
YDR159W	YFL039C	976
YDR159W	YFR002W	966
YDR159W	YGL086W	915
YDR159W	YGL100W	966
YDR159W	YGL172W	966
YDR159W	YGR119C	966
YDR159W	YIL063C	915
YDR159W	YIL115C	966
YDR159W	YJL030W	917
YDR159W	YJL039C	966
YDR159W	YJL041W	999
YDR159W	YJL061W	972
YDR159W	YJR042W	966
YDR159W	YKL057C	973
YDR159W	YKL068W	969
YDR159W	YKL186C	999
YDR159W	YKR082W	969
YDR159W	YKR095W	949
YDR159W	YLR018C	974
YDR159W	YLR208W	966
YDR159W	YLR347C	966
YDR159W	YML103C	972
YDR159W	YMR047C	999
YDR159W	YMR129W	924
YDR159W	YMR153W	966
YDR159W	YOL012C	964
YDR159W	YOL072W	999
YDR159W	YOR046C	952
YDR159W	YOR098C	993
YDR159W	YOR257W	997
YDR159W	YPL125W	921
YDR159W	YPL169C	999
YDR160W	YCL018W	991
YDR160W	YFR029W	997
YDR160W	YJL156C	997
YDR160W	YKR039W	997
YDR160W	YNL008C	906
YDR160W	YNL159C	906

YDR162C	YDL006W	999
YDR162C	YHL007C	991
YDR162C	YJL095W	952
YDR162C	YJL128C	998
YDR162C	YKR048C	984
YDR162C	YMR139W	925
YDR162C	YOR208W	909
YDR162C	YOR231W	909
YDR163W	YPL240C	919
YDR164C	YAL030W	987
YDR164C	YBL038W	927
YDR164C	YBR080C	959
YDR164C	YDR099W	948
YDR164C	YDR166C	994
YDR164C	YER008C	985
YDR164C	YER177W	948
YDR164C	YFL005W	996
YDR164C	YGL233W	998
YDR164C	YGR009C	999
YDR164C	YIL068C	981
YDR164C	YJL085W	994
YDR164C	YLR026C	940
YDR164C	YLR166C	998
YDR164C	YLR396C	905
YDR164C	YMR079W	932
YDR164C	YMR183C	999
YDR164C	YNL272C	981
YDR164C	YNR049C	999
YDR164C	YOL018C	921
YDR164C	YOR327C	999
YDR164C	YPL232W	999
YDR164C	YPR055W	991
YDR165W	YBL024W	912
YDR165W	YDL201W	999
YDR165W	YOR243C	906
YDR166C	YBR102C	999
YDR166C	YBR200W	914
YDR166C	YDR164C	994
YDR166C	YER008C	999
YDR166C	YFL005W	998
YDR166C	YGL233W	999
YDR166C	YIL068C	999
YDR166C	YJL085W	999
YDR166C	YLR166C	999

YDR166C	YLR229C	941
YDR166C	YNL272C	995
YDR166C	YOR326W	987
YDR166C	YPR032W	966
YDR166C	YPR055W	999
YDR167W	YBR081C	999
YDR167W	YBR111W-A	959
YDR167W	YBR198C	999
YDR167W	YBR253W	991
YDR167W	YBR279W	965
YDR167W	YCL010C	999
YDR167W	YCR042C	999
YDR167W	YDL006W	906
YDR167W	YDL140C	985
YDR167W	YDR145W	999
YDR167W	YDR176W	999
YDR167W	YDR308C	991
YDR167W	YDR392W	999
YDR167W	YDR404C	911
YDR167W	YDR448W	999
YDR167W	YER022W	978
YDR167W	YER148W	999
YDR167W	YER164W	980
YDR167W	YGL025C	991
YDR167W	YGL066W	999
YDR167W	YGL070C	910
YDR167W	YGL112C	999
YDR167W	YGR097W	982
YDR167W	YGR252W	999
YDR167W	YGR274C	999
YDR167W	YHR041C	978
YDR167W	YHR099W	999
YDR167W	YIL021W	908
YDR167W	YKL028W	902
YDR167W	YKL058W	939
YDR167W	YLR055C	999
YDR167W	YLR418C	965
YDR167W	YML015C	999
YDR167W	YML098W	999
YDR167W	YML114C	999
YDR167W	YMR005W	999
YDR167W	YMR223W	999
YDR167W	YMR227C	999
YDR167W	YMR236W	999

YDR167W	YNR010W	995
YDR167W	YOL135C	992
YDR167W	YOL145C	965
YDR167W	YOL148C	999
YDR167W	YOR151C	977
YDR167W	YOR174W	991
YDR167W	YOR194C	904
YDR167W	YOR207C	954
YDR167W	YOR210W	912
YDR167W	YOR224C	908
YDR167W	YPL011C	999
YDR167W	YPL047W	999
YDR167W	YPL129W	999
YDR167W	YPL248C	992
YDR167W	YPL254W	999
YDR167W	YPR056W	907
YDR167W	YPR070W	994
YDR167W	YPR086W	994
YDR167W	YPR168W	991
YDR167W	YPR187W	918
YDR168W	YBR160W	990
YDR168W	YDL028C	998
YDR168W	YDL108W	995
YDR168W	YGR040W	972
YDR168W	YJR032W	977
YDR168W	YKL117W	966
YDR168W	YLR113W	982
YDR168W	YLR362W	999
YDR168W	YMR186W	999
YDR168W	YNL281W	943
YDR168W	YOR027W	999
YDR168W	YOR061W	933
YDR168W	YPL106C	914
YDR168W	YPL240C	999
YDR169C	YOL004W	904
YDR170C	YAL026C	983
YDR170C	YDL052C	926
YDR170C	YDL137W	933
YDR170C	YDL192W	999
YDR170C	YFL038C	978
YDR170C	YFR044C	935
YDR170C	YGL238W	922
YDR170C	YJR031C	912
YDR170C	YLR342W	988

YDR170C	YNL238W	988
YDR170C	YNL287W	924
YDR170C	YOR094W	966
YDR170C	YPR181C	978
YDR170W-A	YBL005W-A	970
YDR171W	YBR069C	947
YDR171W	YBR101C	922
YDR171W	YGL153W	927
YDR171W	YNL007C	935
YDR171W	YNL085W	954
YDR171W	YOR151C	969
YDR171W	YPR035W	984
YDR172W	YAL005C	999
YDR172W	YBL007C	994
YDR172W	YBR143C	999
YDR172W	YCL028W	973
YDR172W	YDL229W	984
YDR172W	YDR385W	900
YDR172W	YER103W	909
YDR172W	YER165W	999
YDR172W	YER176W	967
YDR172W	YGR072W	994
YDR172W	YHR015W	994
YDR172W	YHR077C	996
YDR172W	YLL024C	977
YDR172W	YLL026W	999
YDR172W	YLR344W	980
YDR172W	YML068W	982
YDR172W	YML117W	924
YDR172W	YMR080C	999
YDR172W	YNL007C	993
YDR172W	YNL064C	999
YDR172W	YNL209W	906
YDR172W	YNL229C	927
YDR172W	YNL243W	970
YDR172W	YOL149W	971
YDR172W	YOR046C	906
YDR172W	YOR069W	916
YDR172W	YPL106C	980
YDR173C	YDR017C	917
YDR173C	YDR315C	999
YDR173C	YJR066W	901
YDR173C	YML099C	963
YDR173C	YMR042W	999

YDR173C	YMR043W	999
YDR173C	YPL268W	984
YDR174W	YAL027W	901
YDR174W	YBR272C	957
YDR174W	YDR317W	951
YDR174W	YGR258C	990
YDR174W	YJL076W	954
YDR174W	YJL148W	913
YDR174W	YJL177W	976
YDR174W	YLR234W	971
YDR174W	YML015C	982
YDR174W	YMR242C	972
YDR174W	YNL135C	974
YDR174W	YNL248C	996
YDR174W	YOR194C	905
YDR175C	Q0140	962
YDR175C	YBL090W	988
YDR175C	YBR146W	988
YDR175C	YBR251W	999
YDR175C	YDL045W-A	986
YDR175C	YDR036C	999
YDR175C	YDR041W	988
YDR175C	YDR337W	988
YDR175C	YDR347W	988
YDR175C	YGL129C	988
YDR175C	YGR084C	974
YDR175C	YGR165W	988
YDR175C	YGR215W	988
YDR175C	YHL004W	997
YDR175C	YIL093C	988
YDR175C	YJR101W	988
YDR175C	YJR113C	988
YDR175C	YKL155C	997
YDR175C	YMR158W	974
YDR175C	YMR188C	988
YDR175C	YNL081C	986
YDR175C	YNL137C	988
YDR175C	YNL306W	988
YDR175C	YNR037C	987
YDR175C	YOR158W	988
YDR175C	YPL013C	997
YDR175C	YPL118W	988
YDR176W	YBR081C	999
YDR176W	YBR111W-A	992

YDR176W	YBR198C	999
YDR176W	YCL010C	999
YDR176W	YDR145W	999
YDR176W	YDR167W	999
YDR176W	YDR392W	999
YDR176W	YDR448W	999
YDR176W	YEL009C	913
YDR176W	YER148W	999
YDR176W	YER151C	976
YDR176W	YER164W	980
YDR176W	YGL066W	999
YDR176W	YGL112C	999
YDR176W	YGL221C	924
YDR176W	YGR252W	999
YDR176W	YGR274C	998
YDR176W	YHR041C	978
YDR176W	YHR099W	999
YDR176W	YLR055C	999
YDR176W	YMR223W	999
YDR176W	YMR227C	988
YDR176W	YMR236W	999
YDR176W	YOL148C	999
YDR176W	YOR023C	966
YDR176W	YPL011C	988
YDR176W	YPL016W	974
YDR176W	YPL047W	999
YDR176W	YPL254W	999
YDR177W	YBR082C	997
YDR177W	YER012W	927
YDR177W	YPR103W	999
YDR178W	YJL045W	950
YDR178W	YKL141W	999
YDR178W	YKL148C	995
YDR178W	YLL041C	991
YDR178W	YPL262W	948
YDR179C	YDL147W	999
YDR179C	YDL216C	999
YDR179C	YIL071C	999
YDR179C	YJR084W	995
YDR179C	YMR025W	999
YDR179C	YOL117W	999
YDR180W	YDL003W	987
YDR180W	YER147C	999
YDR180W	YFL008W	989

YDR180W	YIL026C	994
YDR180W	YJL074C	989
YDR181C	YDR227W	909
YDR181C	YJL115W	998
YDR181C	YKR101W	933
YDR181C	YMR127C	999
YDR181C	YOR213C	999
YDR182W	YCR044C	987
YDR182W	YGR217W	983
YDR182W	YOL122C	990
YDR183W	YGR078C	982
YDR183W	YOR212W	950
YDR184C	YBR164C	967
YDR184C	YIR009W	927
YDR184C	YKR072C	918
YDR184C	YLR319C	968
YDR186C	YBR125C	999
YDR188W	YDL143W	999
YDR188W	YDR212W	943
YDR188W	YFL037W	910
YDR188W	YFR009W	968
YDR188W	YIL142W	999
YDR188W	YJL008C	936
YDR188W	YJL014W	997
YDR188W	YJL111W	999
YDR188W	YJR064W	927
YDR188W	YKL029C	973
YDR188W	YKL104C	970
YDR188W	YKL203C	984
YDR188W	YML094W	902
YDR188W	YML124C	915
YDR188W	YMR028W	927
YDR188W	YNL153C	901
YDR188W	YOR281C	995
YDR188W	YOR297C	987
YDR189W	YAL030W	998
YDR189W	YBL050W	994
YDR189W	YDL058W	998
YDR189W	YER157W	987
YDR189W	YFL038C	999
YDR189W	YGR120C	987
YDR189W	YIL004C	999
YDR189W	YKL196C	993
YDR189W	YLR026C	999

YDR189W	YLR078C	999
YDR189W	YLR262C	999
YDR189W	YLR268W	999
YDR189W	YNL258C	996
YDR189W	YOR075W	999
YDR189W	YOR327C	998
YDR189W	YPR105C	979
YDR190C	YAL011W	999
YDR190C	YBR009C	982
YDR190C	YBR231C	999
YDR190C	YBR245C	978
YDR190C	YCR060W	995
YDR190C	YDL002C	996
YDR190C	YDR334W	999
YDR190C	YDR485C	999
YDR190C	YER092W	993
YDR190C	YER148W	914
YDR190C	YFL013C	994
YDR190C	YFL039C	999
YDR190C	YGL150C	999
YDR190C	YGR002C	999
YDR190C	YGR163W	909
YDR190C	YHR034C	999
YDR190C	YHR099W	971
YDR190C	YJL081C	999
YDR190C	YLR052W	990
YDR190C	YLR085C	998
YDR190C	YLR385C	998
YDR190C	YLR399C	970
YDR190C	YML041C	999
YDR190C	YMR072W	977
YDR190C	YNL059C	996
YDR190C	YNL107W	999
YDR190C	YNL215W	997
YDR190C	YOL012C	997
YDR190C	YOR141C	997
YDR190C	YOR304W	970
YDR190C	YPL129W	991
YDR190C	YPL235W	999
YDR191W	YOR025W	976
YDR192C	YAR002W	945
YDR192C	YBR017C	933
YDR192C	YDL088C	989
YDR192C	YDL116W	997

YDR192C	YDL207W	999
YDR192C	YDR101C	956
YDR192C	YDR159W	905
YDR192C	YDR315C	920
YDR192C	YER107C	996
YDR192C	YER110C	992
YDR192C	YGL092W	993
YDR192C	YGL172W	985
YDR192C	YGR119C	988
YDR192C	YGR218W	998
YDR192C	YIL115C	918
YDR192C	YJL061W	939
YDR192C	YJR042W	961
YDR192C	YKL057C	988
YDR192C	YKL068W	988
YDR192C	YKR082W	985
YDR192C	YLR018C	934
YDR192C	YLR293C	964
YDR192C	YLR335W	974
YDR192C	YLR347C	999
YDR192C	YMR047C	995
YDR192C	YMR255W	998
YDR192C	YNL189W	924
YDR192C	YOR046C	998
YDR192C	YOR098C	963
YDR192C	YOR257W	961
YDR192C	YPL020C	989
YDR192C	YPL169C	954
YDR194C	YPL029W	904
YDR195W	YAL043C	999
YDR195W	YAR014C	985
YDR195W	YDR301W	999
YDR195W	YER032W	991
YDR195W	YER133W	999
YDR195W	YGR156W	999
YDR195W	YJL033W	960
YDR195W	YJR093C	999
YDR195W	YKL018W	999
YDR195W	YKL059C	999
YDR195W	YKR002W	999
YDR195W	YLR115W	999
YDR195W	YLR277C	999
YDR195W	YLR448W	983
YDR195W	YMR061W	991

YDR195W	YMR242C	981
YDR195W	YNL222W	999
YDR195W	YNL317W	999
YDR195W	YOR179C	999
YDR195W	YPR107C	999
YDR196C	YGR277C	978
YDR197W	YGL119W	904
YDR200C	YFR008W	959
YDR200C	YLR238W	902
YDR200C	YMR052W	997
YDR200C	YNL127W	991
YDR201W	YDR016C	999
YDR201W	YDR320C-A	984
YDR201W	YGL061C	999
YDR201W	YGR113W	999
YDR201W	YIL144W	983
YDR201W	YKL052C	999
YDR201W	YKL138C-A	996
YDR201W	YKR037C	999
YDR201W	YKR083C	999
YDR201W	YOL069W	962
YDR201W	YPL209C	929
YDR202C	YBR127C	975
YDR202C	YDR328C	998
YDR202C	YJR033C	999
YDR202C	YOR332W	960
YDR204W	YBR003W	901
YDR204W	YGL119W	901
YDR204W	YGR255C	919
YDR204W	YLR201C	942
YDR204W	YML110C	914
YDR204W	YOL096C	997
YDR204W	YOR125C	986
YDR205W	YIL023C	918
YDR205W	YNR039C	995
YDR206W	YDL220C	936
YDR206W	YOL149W	976
YDR207C	YAL013W	984
YDR207C	YBR095C	998
YDR207C	YBR103W	914
YDR207C	YCR033W	911
YDR207C	YDL076C	999
YDR207C	YDR123C	918
YDR207C	YGL194C	924

YDR207C	YHL020C	911
YDR207C	YIL084C	999
YDR207C	YJL106W	967
YDR207C	YJL153C	910
YDR207C	YJR094C	999
YDR207C	YKL185W	983
YDR207C	YKR029C	914
YDR207C	YMR042W	932
YDR207C	YMR139W	995
YDR207C	YMR263W	984
YDR207C	YNL097C	988
YDR207C	YNL307C	930
YDR207C	YNL330C	999
YDR207C	YOL004W	999
YDR207C	YOR304W	935
YDR207C	YPL139C	993
YDR207C	YPL181W	998
YDR208W	YBR036C	983
YDR208W	YBR109C	940
YDR208W	YDR361C	942
YDR208W	YER110C	942
YDR208W	YIL105C	908
YDR208W	YKL203C	913
YDR208W	YLR305C	991
YDR208W	YLR371W	932
YDR208W	YMR109W	917
YDR208W	YPL268W	941
YDR211W	YBR159W	988
YDR211W	YDR037W	926
YDR211W	YER025W	999
YDR211W	YFL039C	940
YDR211W	YGR083C	999
YDR211W	YJR007W	999
YDR211W	YKR026C	999
YDR211W	YLR291C	999
YDR211W	YNL265C	987
YDR211W	YOR260W	999
YDR211W	YPL237W	999
YDR211W	YPR010C	972
YDR212W	YDL143W	943
YDR212W	YDR188W	943
YDR212W	YFL037W	989
YDR212W	YFL039C	984
YDR212W	YIL142W	999

YDR212W	YJL008C	932
YDR212W	YJL014W	998
YDR212W	YJL111W	924
YDR212W	YJR064W	924
YDR212W	YML085C	988
YDR212W	YML094W	900
YDR213W	YHR007C	909
YDR213W	YHR165C	976
YDR213W	YML083C	902
YDR213W	YMR202W	995
YDR214W	YGL195W	963
YDR214W	YJL030W	900
YDR214W	YLR216C	971
YDR214W	YMR186W	999
YDR214W	YNL281W	997
YDR214W	YOR027W	951
YDR214W	YPL240C	999
YDR216W	YBR198C	974
YDR216W	YDR028C	990
YDR216W	YDR256C	953
YDR216W	YDR448W	937
YDR216W	YDR477W	999
YDR216W	YGR252W	936
YDR216W	YMR280C	997
YDR216W	YMR303C	996
YDR216W	YOR363C	993
YDR216W	YPR086W	987
YDR217C	YBL003C	930
YDR217C	YBL088C	995
YDR217C	YBR136W	999
YDR217C	YBR223C	976
YDR217C	YCL061C	998
YDR217C	YDL101C	969
YDR217C	YDL220C	999
YDR217C	YDR076W	965
YDR217C	YDR082W	918
YDR217C	YDR225W	925
YDR217C	YER095W	994
YDR217C	YER173W	999
YDR217C	YGL175C	965
YDR217C	YHR031C	987
YDR217C	YHR134W	969
YDR217C	YHR154W	985
YDR217C	YJL047C	975

YDR217C	YJL115W	974
YDR217C	YJR043C	959
YDR217C	YKL113C	999
YDR217C	YKL213C	966
YDR217C	YLL002W	966
YDR217C	YLR288C	970
YDR217C	YLR318W	906
YDR217C	YML032C	994
YDR217C	YMR048W	954
YDR217C	YMR106C	913
YDR217C	YMR190C	993
YDR217C	YMR284W	986
YDR217C	YNL261W	998
YDR217C	YNL273W	989
YDR217C	YOR033C	976
YDR217C	YOR217W	967
YDR217C	YOR368W	976
YDR217C	YPL153C	999
YDR218C	YJR076C	980
YDR218C	YPL161C	954
YDR219C	YDR328C	985
YDR219C	YHR117W	986
YDR219C	YLR368W	930
YDR219C	YNL121C	907
YDR221W	YBR229C	999
YDR223W	YJL141C	930
YDR223W	YPR104C	911
YDR224C	YBL002W	973
YDR224C	YBL003C	999
YDR224C	YBL008W	919
YDR224C	YBL046W	936
YDR224C	YBR009C	999
YDR224C	YBR010W	966
YDR224C	YBR289W	949
YDR224C	YDR075W	936
YDR224C	YDR225W	999
YDR224C	YDR303C	987
YDR224C	YDR448W	940
YDR224C	YGL133W	982
YDR224C	YGL241W	999
YDR224C	YGR063C	912
YDR224C	YHL007C	909
YDR224C	YIL126W	999
YDR224C	YJL127C	934

YDR224C	YKR048C	990
YDR224C	YLR357W	989
YDR224C	YLR442C	968
YDR224C	YML032C	910
YDR224C	YMR308C	962
YDR224C	YNL201C	936
YDR224C	YOL012C	999
YDR224C	YOR038C	933
YDR224C	YOR116C	978
YDR224C	YOR304W	975
YDR224C	YPL082C	985
YDR224C	YPR052C	919
YDR225W	YBL002W	999
YDR225W	YBL003C	997
YDR225W	YBL088C	948
YDR225W	YBR009C	999
YDR225W	YBR010W	999
YDR225W	YBR017C	966
YDR225W	YDR075W	936
YDR225W	YDR217C	925
YDR225W	YDR224C	999
YDR225W	YDR243C	918
YDR225W	YDR448W	919
YDR225W	YER110C	966
YDR225W	YGL207W	989
YDR225W	YGL241W	999
YDR225W	YGR002C	912
YDR225W	YGR252W	956
YDR225W	YHR191C	924
YDR225W	YJL081C	994
YDR225W	YJL127C	984
YDR225W	YKR048C	999
YDR225W	YLR347C	992
YDR225W	YLR357W	974
YDR225W	YLR442C	968
YDR225W	YML069W	932
YDR225W	YMR308C	992
YDR225W	YNL030W	998
YDR225W	YNL031C	994
YDR225W	YNL201C	936
YDR225W	YOL012C	995
YDR225W	YOR038C	932
YDR225W	YOR244W	926
YDR226W	YAL038W	906

YDR226W	YDR441C	901
YDR226W	YHR136C	920
YDR226W	YJR105W	975
YDR226W	YKL067W	951
YDR226W	YLR359W	900
YDR226W	YML022W	905
YDR226W	YOR347C	905
YDR227W	YBR009C	998
YDR227W	YBR010W	976
YDR227W	YBR060C	999
YDR227W	YBR103W	983
YDR227W	YDL042C	999
YDR227W	YDR143C	998
YDR227W	YDR181C	909
YDR227W	YDR310C	989
YDR227W	YER151C	996
YDR227W	YFL023W	900
YDR227W	YKR101W	999
YDR227W	YLR442C	999
YDR227W	YLR453C	947
YDR227W	YML032C	926
YDR227W	YML109W	995
YDR227W	YMR106C	957
YDR227W	YMR273C	966
YDR227W	YMR284W	998
YDR227W	YNL186W	999
YDR227W	YNL216W	999
YDR227W	YOR213C	906
YDR228C	YAL043C	965
YDR228C	YDL140C	999
YDR228C	YDR301W	993
YDR228C	YER165W	917
YDR228C	YGL044C	999
YDR228C	YHR216W	975
YDR228C	YKL059C	998
YDR228C	YKR002W	993
YDR228C	YLR115W	999
YDR228C	YLR277C	999
YDR228C	YMR061W	999
YDR228C	YOR250C	999
YDR228C	YPR049C	905
YDR229W	YLR396C	987
YDR229W	YML001W	922
YDR231C	Q0250	922

YDR231C	YNL192W	997
YDR232W	YBR263W	915
YDR232W	YDL205C	950
YDR232W	YGL040C	993
YDR232W	YGR286C	930
YDR232W	YLR058C	915
YDR233C	YER087C-B	982
YDR233C	YFL038C	956
YDR233C	YIL068C	967
YDR233C	YNL044W	924
YDR233C	YPL037C	938
YDR233C	YPL154C	992
YDR234W	YDL131W	969
YDR234W	YDL182W	970
YDR234W	YIL094C	970
YDR235W	YBR119W	999
YDR235W	YDL087C	999
YDR235W	YDR240C	999
YDR235W	YER029C	997
YDR235W	YGR013W	999
YDR235W	YGR074W	988
YDR235W	YHR086W	999
YDR235W	YIL061C	999
YDR235W	YKL012W	999
YDR235W	YLR147C	988
YDR235W	YLR275W	988
YDR235W	YLR298C	999
YDR235W	YML046W	999
YDR235W	YML049C	974
YDR235W	YMR125W	999
YDR235W	YPL178W	970
YDR235W	YPR182W	999
YDR236C	YDL045C	971
YDR237W	YBL038W	999
YDR237W	YBR122C	974
YDR237W	YBR282W	970
YDR237W	YCR046C	992
YDR237W	YCR071C	974
YDR237W	YDR116C	992
YDR237W	YDR296W	993
YDR237W	YDR322W	989
YDR237W	YDR405W	970
YDR237W	YDR462W	999
YDR237W	YEL050C	915

YDR237W	YGR220C	999
YDR237W	YHR147C	998
YDR237W	YJL063C	998
YDR237W	YKL167C	974
YDR237W	YKR006C	974
YDR237W	YKR085C	974
YDR237W	YLR312W-A	977
YDR237W	YLR439W	974
YDR237W	YML025C	998
YDR237W	YMR024W	974
YDR237W	YMR193W	974
YDR237W	YNL005C	994
YDR237W	YNL185C	993
YDR237W	YNL252C	989
YDR237W	YNL284C	999
YDR237W	YOR150W	996
YDR238C	YBR162C	994
YDR238C	YDL137W	949
YDR238C	YDL145C	999
YDR238C	YDL192W	997
YDR238C	YER122C	999
YDR238C	YFR051C	999
YDR238C	YGL137W	999
YDR238C	YIL004C	906
YDR238C	YIL076W	998
YDR238C	YLR078C	911
YDR238C	YLR268W	904
YDR238C	YNL284C	917
YDR238C	YNL287W	999
YDR238C	YPL010W	999
YDR240C	YBR119W	999
YDR240C	YDL087C	999
YDR240C	YDR235W	999
YDR240C	YER029C	996
YDR240C	YFL017W-A	990
YDR240C	YGR013W	999
YDR240C	YGR074W	999
YDR240C	YHR086W	999
YDR240C	YHR099W	940
YDR240C	YHR165C	991
YDR240C	YIL061C	999
YDR240C	YKL012W	999
YDR240C	YKL074C	984
YDR240C	YLR147C	996

YDR240C	YLR275W	996
YDR240C	YLR298C	999
YDR240C	YLR347C	965
YDR240C	YML046W	999
YDR240C	YMR125W	999
YDR240C	YNL210W	994
YDR240C	YPL178W	999
YDR240C	YPR182W	986
YDR243C	YDR225W	918
YDR243C	YER172C	980
YDR243C	YHR165C	999
YDR243C	YKL173W	990
YDR243C	YLR183C	981
YDR243C	YMR268C	930
YDR244W	YAL055W	906
YDR244W	YBR082C	939
YDR244W	YDL065C	904
YDR244W	YDL078C	999
YDR244W	YDR142C	999
YDR244W	YDR265W	999
YDR244W	YDR329C	917
YDR244W	YGL153W	999
YDR244W	YGR077C	999
YDR244W	YGR133W	991
YDR244W	YJL210W	995
YDR244W	YKL197C	997
YDR244W	YLR191W	999
YDR244W	YLR284C	914
YDR244W	YML042W	984
YDR244W	YMR026C	999
YDR244W	YMR177W	999
YDR244W	YNL214W	999
YDR244W	YNL329C	994
YDR244W	YOL044W	966
YDR244W	YOL147C	951
YDR245W	YBR106W	990
YDR245W	YEL036C	999
YDR245W	YGR132C	982
YDR245W	YGR231C	980
YDR245W	YIL034C	906
YDR245W	YJL183W	999
YDR245W	YJR075W	999
YDR245W	YLR342W	981
YDR245W	YML115C	953

YDR245W	YPL050C	999
YDR246W	YBR254C	999
YDR246W	YDR108W	999
YDR246W	YDR407C	999
YDR246W	YDR472W	999
YDR246W	YFL038C	985
YDR246W	YGR166W	999
YDR246W	YKR068C	999
YDR246W	YML077W	999
YDR246W	YMR218C	999
YDR246W	YOR115C	999
YDR247W	YBR125C	944
YDR247W	YKR072C	911
YDR248C	YCR073W-A	930
YDR248C	YGR248W	925
YDR248C	YGR256W	967
YDR248C	YHR163W	940
YDR248C	YHR183W	966
YDR248C	YNR034W	914
YDR248C	YOR095C	922
YDR252W	YAL021C	943
YDR252W	YGR134W	978
YDR252W	YHR193C	999
YDR252W	YPL037C	999
YDR253C	YIL046W	966
YDR253C	YIR017C	950
YDR253C	YJR060W	957
YDR253C	YNL103W	992
YDR253C	YPL038W	996
YDR254W	YAL034W-A	976
YDR254W	YBR107C	999
YDR254W	YBR211C	999
YDR254W	YDR318W	999
YDR254W	YDR383C	996
YDR254W	YEL061C	939
YDR254W	YER016W	982
YDR254W	YGL060W	981
YDR254W	YGL086W	971
YDR254W	YGR113W	983
YDR254W	YGR140W	999
YDR254W	YGR179C	999
YDR254W	YGR188C	936
YDR254W	YIR010W	906
YDR254W	YJL030W	998

YDR254W	YJR135C	999
YDR254W	YKL089W	978
YDR254W	YLR315W	997
YDR254W	YLR381W	999
YDR254W	YMR094W	999
YDR254W	YMR117C	983
YDR254W	YMR168C	994
YDR254W	YPL018W	999
YDR254W	YPL233W	906
YDR254W	YPR046W	992
YDR255C	YBL049W	929
YDR255C	YBR105C	981
YDR255C	YCL039W	995
YDR255C	YGL227W	999
YDR255C	YIL017C	999
YDR255C	YIL097W	999
YDR255C	YMR135C	999
YDR256C	YDR216W	953
YDR256C	YGR088W	925
YDR256C	YHR008C	995
YDR256C	YJR104C	987
YDR258C	YBR227C	931
YDR260C	YBL084C	999
YDR260C	YDL008W	999
YDR260C	YDR118W	999
YDR260C	YFR036W	999
YDR260C	YGL240W	999
YDR260C	YHR166C	999
YDR260C	YIR025W	999
YDR260C	YKL022C	999
YDR260C	YLR102C	999
YDR260C	YLR127C	999
YDR260C	YNL172W	999
YDR260C	YOR249C	999
YDR261W-A	YCL019W	998
YDR261W-A	YDR261W-B	998
YDR261W-B	YCL019W	998
YDR261W-B	YDR261W-A	998
YDR263C	YHR120W	909
YDR264C	YDL226C	976
YDR264C	YKL178C	909
YDR264C	YNL154C	983
YDR264C	YOR036W	913
YDR264C	YOR171C	902

YDR264C	YOR212W	999
YDR265W	YAL055W	931
YDR265W	YDL065C	989
YDR265W	YDR142C	926
YDR265W	YDR244W	999
YDR265W	YGL153W	994
YDR265W	YGR077C	963
YDR265W	YGR133W	970
YDR265W	YJL210W	994
YDR265W	YKL197C	902
YDR265W	YLR191W	998
YDR265W	YMR026C	999
YDR265W	YNL214W	993
YDR265W	YOL044W	969
YDR267C	YHR122W	986
YDR267C	YIL128W	984
YDR270W	YGL071W	940
YDR270W	YMR058W	960
YDR270W	YNL259C	999
YDR270W	YPL057C	993
YDR272W	YML004C	992
YDR273W	YFR052W	934
YDR276C	YPL240C	919
YDR277C	YDL138W	997
YDR277C	YDL194W	997
YDR277C	YDR345C	976
YDR277C	YHR094C	984
YDR277C	YKL038W	976
YDR277C	YMR011W	909
YDR277C	YOR047C	976
YDR277C	YPL240C	919
YDR279W	YDR386W	913
YDR279W	YLR154C	998
YDR279W	YMR190C	962
YDR279W	YNL072W	999
YDR280W	YBL004W	923
YDR280W	YCR035C	999
YDR280W	YDL111C	999
YDR280W	YGR090W	923
YDR280W	YGR095C	999
YDR280W	YGR158C	999
YDR280W	YGR195W	999
YDR280W	YHR069C	999
YDR280W	YHR081W	999

YDR280W	YJL109C	926
YDR280W	YLR398C	967
YDR280W	YMR128W	946
YDR280W	YMR229C	959
YDR280W	YNL189W	993
YDR280W	YNL232W	999
YDR280W	YNL248C	964
YDR280W	YNR024W	959
YDR280W	YOL021C	999
YDR280W	YOL142W	999
YDR280W	YOR001W	999
YDR280W	YOR076C	999
YDR280W	YPR189W	997
YDR283C	YBR055C	984
YDR283C	YDR037W	938
YDR283C	YEL009C	999
YDR283C	YER133W	920
YDR283C	YFR009W	994
YDR283C	YGL195W	999
YDR283C	YGR083C	999
YDR283C	YGR091W	984
YDR283C	YJR007W	994
YDR283C	YKL173W	943
YDR283C	YKL204W	928
YDR283C	YKR026C	999
YDR283C	YLR291C	998
YDR283C	YMR116C	984
YDR283C	YMR186W	986
YDR283C	YOR308C	978
YDR283C	YPL240C	994
YDR283C	YPR178W	977
YDR285W	YBR186W	998
YDR285W	YDL042C	986
YDR285W	YDR440W	947
YDR285W	YER179W	963
YDR285W	YKL201C	999
YDR285W	YLR263W	999
YDR285W	YLR309C	999
YDR285W	YLR394W	914
YDR285W	YOR216C	956
YDR285W	YOR351C	985
YDR287W	YJL153C	933
YDR288W	YDL105W	963
YDR288W	YLR007W	920

YDR288W	YOL034W	996
YDR289C	YDL140C	918
YDR289C	YGL246C	924
YDR289C	YOL012C	904
YDR289C	YOR048C	997
YDR289C	YOR151C	917
YDR292C	YKL154W	999
YDR293C	YBL046W	926
YDR293C	YDL047W	997
YDR293C	YDL150W	923
YDR293C	YDR075W	926
YDR293C	YER033C	926
YDR293C	YER111C	939
YDR293C	YER155C	912
YDR293C	YIL129C	994
YDR293C	YJL095W	913
YDR293C	YJR066W	972
YDR293C	YKR028W	976
YDR293C	YLR262C	980
YDR293C	YML109W	941
YDR293C	YNL161W	999
YDR293C	YNL201C	926
YDR294C	YDR297W	907
YDR294C	YJL134W	999
YDR294C	YKL008C	998
YDR294C	YKR053C	946
YDR294C	YLR260W	966
YDR294C	YNL169C	928
YDR294C	YOR171C	999
YDR295C	YNL021W	999
YDR295C	YOL090W	988
YDR295C	YPR179C	999
YDR296W	YBL038W	992
YDR296W	YBR122C	992
YDR296W	YBR282W	970
YDR296W	YCR046C	992
YDR296W	YCR071C	992
YDR296W	YDR116C	992
YDR296W	YDR237W	993
YDR296W	YDR322W	992
YDR296W	YDR405W	970
YDR296W	YDR462W	992
YDR296W	YGR220C	997
YDR296W	YHR120W	906

YDR296W	YHR147C	970
YDR296W	YJL063C	991
YDR296W	YKL011C	907
YDR296W	YKL167C	993
YDR296W	YKR006C	993
YDR296W	YKR085C	992
YDR296W	YLR312W-A	991
YDR296W	YLR439W	993
YDR296W	YML025C	992
YDR296W	YMR024W	990
YDR296W	YMR193W	974
YDR296W	YNL005C	992
YDR296W	YNL177C	979
YDR296W	YNL185C	933
YDR296W	YNL252C	992
YDR296W	YNL284C	917
YDR296W	YOR150W	992
YDR297W	YBR036C	995
YDR297W	YCR009C	978
YDR297W	YDR294C	907
YDR297W	YDR346C	921
YDR297W	YGL001C	909
YDR297W	YJL134W	999
YDR297W	YJR088C	901
YDR297W	YKL008C	964
YDR297W	YKR053C	945
YDR297W	YLR260W	943
YDR297W	YOR171C	997
YDR298C	Q0085	994
YDR298C	YBL099W	999
YDR298C	YBR039W	999
YDR298C	YDL004W	995
YDR298C	YDL181W	920
YDR298C	YJR121W	999
YDR298C	YKL016C	999
YDR298C	YPL078C	999
YDR298C	YPL271W	912
YDR299W	YBL004W	998
YDR299W	YBR048W	983
YDR299W	YBR247C	958
YDR299W	YCL031C	938
YDR299W	YCL059C	999
YDR299W	YCR057C	982
YDR299W	YDL014W	998

YDR299W	YDL060W	935
YDR299W	YDL148C	999
YDR299W	YDL153C	915
YDR299W	YDR324C	951
YDR299W	YDR449C	948
YDR299W	YER082C	944
YDR299W	YER127W	998
YDR299W	YGL019W	914
YDR299W	YGL171W	947
YDR299W	YGR081C	923
YDR299W	YGR090W	992
YDR299W	YGR128C	931
YDR299W	YGR145W	999
YDR299W	YHR148W	998
YDR299W	YHR169W	946
YDR299W	YHR196W	945
YDR299W	YIL035C	914
YDR299W	YJL010C	986
YDR299W	YJL033W	993
YDR299W	YJL069C	931
YDR299W	YJL109C	931
YDR299W	YJR002W	998
YDR299W	YJR145C	978
YDR299W	YKR060W	998
YDR299W	YLL011W	946
YDR299W	YLR003C	943
YDR299W	YLR129W	940
YDR299W	YLR175W	922
YDR299W	YLR186W	998
YDR299W	YLR197W	931
YDR299W	YLR208W	904
YDR299W	YLR222C	948
YDR299W	YLR409C	932
YDR299W	YMR093W	926
YDR299W	YMR128W	922
YDR299W	YMR229C	914
YDR299W	YNL075W	945
YDR299W	YNL132W	999
YDR299W	YNL308C	978
YDR299W	YNR054C	970
YDR299W	YOL010W	934
YDR299W	YOR039W	922
YDR299W	YOR061W	914
YDR299W	YOR078W	944

YDR299W	YOR096W	941
YDR299W	YOR145C	942
YDR299W	YPL012W	922
YDR299W	YPL085W	921
YDR299W	YPL126W	998
YDR299W	YPL217C	998
YDR299W	YPL266W	922
YDR299W	YPR112C	940
YDR299W	YPR137W	951
YDR299W	YPR144C	998
YDR299W	YPR181C	903
YDR300C	YER023W	972
YDR300C	YGR194C	927
YDR300C	YMR062C	912
YDR300C	YNL265C	912
YDR300C	YOR323C	999
YDR301W	YAL043C	999
YDR301W	YDL140C	971
YDR301W	YDR195W	999
YDR301W	YDR228C	993
YDR301W	YER133W	999
YDR301W	YGL044C	967
YDR301W	YGR156W	999
YDR301W	YJR093C	999
YDR301W	YKL018W	999
YDR301W	YKL059C	999
YDR301W	YKR002W	999
YDR301W	YLR115W	999
YDR301W	YLR277C	999
YDR301W	YMR061W	999
YDR301W	YNL222W	987
YDR301W	YNL317W	999
YDR301W	YOR250C	953
YDR301W	YPR107C	999
YDR302W	YJL062W	951
YDR302W	YLL031C	963
YDR302W	YLR088W	951
YDR302W	YOR149C	947
YDR303C	YBL006C	981
YDR303C	YBR009C	992
YDR303C	YBR049C	974
YDR303C	YBR245C	999
YDR303C	YCR020W-B	999
YDR303C	YCR052W	999

YDR303C	YDR224C	987
YDR303C	YFR037C	999
YDR303C	YGR056W	999
YDR303C	YGR275W	995
YDR303C	YHR056C	999
YDR303C	YIL126W	999
YDR303C	YKR001C	981
YDR303C	YKR008W	999
YDR303C	YLR033W	999
YDR303C	YLR176C	987
YDR303C	YLR321C	999
YDR303C	YLR357W	999
YDR303C	YML127W	999
YDR303C	YMR033W	999
YDR303C	YMR091C	999
YDR303C	YPL082C	991
YDR303C	YPR034W	999
YDR304C	YDR155C	927
YDR304C	YHR057C	912
YDR304C	YML078W	962
YDR306C	YDR328C	952
YDR307W	YDR349C	923
YDR307W	YER145C	998
YDR308C	YBL093C	999
YDR308C	YBR193C	999
YDR308C	YBR253W	999
YDR308C	YBR279W	964
YDR308C	YCR081W	997
YDR308C	YCR084C	997
YDR308C	YDL005C	999
YDR308C	YDL140C	935
YDR308C	YDR167W	991
YDR308C	YDR443C	998
YDR308C	YDR448W	960
YDR308C	YEL009C	999
YDR308C	YER022W	999
YDR308C	YER148W	972
YDR308C	YGL025C	999
YDR308C	YGL127C	999
YDR308C	YGL151W	999
YDR308C	YGL244W	970
YDR308C	YGR104C	999
YDR308C	YHR041C	999
YDR308C	YHR058C	999

YDR308C	YLR071C	999
YDR308C	YLR182W	924
YDR308C	YLR418C	970
YDR308C	YMR112C	998
YDR308C	YNL025C	901
YDR308C	YNL236W	999
YDR308C	YNR010W	999
YDR308C	YOL051W	999
YDR308C	YOL135C	999
YDR308C	YOL145C	970
YDR308C	YOR123C	964
YDR308C	YOR174W	999
YDR308C	YPL042C	999
YDR308C	YPL129W	994
YDR308C	YPL248C	920
YDR308C	YPR070W	999
YDR308C	YPR168W	999
YDR309C	YAL041W	979
YDR309C	YCL032W	905
YDR309C	YCR086W	905
YDR309C	YDR099W	954
YDR309C	YGR221C	921
YDR309C	YHL007C	964
YDR309C	YHR061C	999
YDR309C	YHR107C	992
YDR309C	YHR149C	921
YDR309C	YIL135C	927
YDR309C	YKL082C	940
YDR309C	YLR229C	999
YDR309C	YML109W	905
YDR309C	YNL074C	921
YDR309C	YNL271C	990
YDR309C	YNL298W	996
YDR309C	YOR127W	905
YDR309C	YOR188W	910
YDR309C	YPL256C	933
YDR310C	YBR060C	971
YDR310C	YBR103W	926
YDR310C	YCR033W	926
YDR310C	YDL042C	999
YDR310C	YDR227W	989
YDR310C	YHR124W	945
YDR310C	YIL112W	964
YDR310C	YJL106W	980

YDR310C	YKR029C	955
YDR310C	YLR442C	999
YDR310C	YNL261W	966
YDR310C	YOL068C	999
YDR310C	YOR279C	999
YDR310C	YPL240C	919
YDR311W	YAR007C	977
YDR311W	YBL084C	916
YDR311W	YDL108W	996
YDR311W	YDR079C-A	988
YDR311W	YDR460W	997
YDR311W	YEL037C	984
YDR311W	YER162C	978
YDR311W	YER171W	999
YDR311W	YGR120C	905
YDR311W	YGR258C	999
YDR311W	YIL143C	994
YDR311W	YKL028W	996
YDR311W	YLR005W	999
YDR311W	YML095C	984
YDR311W	YMR201C	997
YDR311W	YNL135C	982
YDR311W	YNL312W	978
YDR311W	YOL051W	959
YDR311W	YOL082W	952
YDR311W	YOR123C	911
YDR311W	YPL022W	983
YDR311W	YPL122C	999
YDR311W	YPR025C	993
YDR311W	YPR056W	999
YDR312W	YHR066W	944
YDR312W	YPR143W	906
YDR314C	YEL037C	945
YDR314C	YML011C	931
YDR315C	YDL207W	994
YDR315C	YDR017C	919
YDR315C	YDR173C	999
YDR315C	YDR192C	920
YDR315C	YLR410W	944
YDR315C	YOR046C	985
YDR316W	YER154W	923
YDR317W	YBR272C	952
YDR317W	YDR174W	951
YDR317W	YGR258C	938

YDR318W	YAL034W-A	976
YDR318W	YBR107C	999
YDR318W	YBR211C	999
YDR318W	YDR254W	999
YDR318W	YDR383C	998
YDR318W	YEL061C	990
YDR318W	YER016W	975
YDR318W	YER018C	978
YDR318W	YGL060W	991
YDR318W	YGL086W	952
YDR318W	YGR140W	987
YDR318W	YGR179C	999
YDR318W	YGR188C	978
YDR318W	YIL144W	989
YDR318W	YIR010W	976
YDR318W	YJL030W	999
YDR318W	YJR112W	976
YDR318W	YJR135C	999
YDR318W	YKL049C	924
YDR318W	YKL089W	998
YDR318W	YKR037C	983
YDR318W	YLR315W	999
YDR318W	YLR381W	999
YDR318W	YMR094W	988
YDR318W	YMR117C	996
YDR318W	YMR168C	954
YDR318W	YOL069W	990
YDR318W	YOR195W	941
YDR318W	YOR349W	926
YDR318W	YPL018W	999
YDR318W	YPL233W	906
YDR318W	YPR046W	999
YDR320C	YDL013W	954
YDR320C	YDL192W	991
YDR320C	YGL206C	979
YDR320C	YGR167W	976
YDR320C	YGR261C	915
YDR320C	YHR004C	921
YDR320C	YHR027C	970
YDR320C	YIL044C	911
YDR320C	YIR004W	978
YDR320C	YJL004C	915
YDR320C	YJL024C	903
YDR320C	YJR097W	978

YDR320C	YLR423C	980
YDR320C	YMR214W	924
YDR320C	YMR274C	917
YDR320C	YNL007C	978
YDR320C	YNL064C	978
YDR320C	YPL051W	949
YDR320C	YPL195W	911
YDR320C	YPR049C	935
YDR320C-A	YDR016C	999
YDR320C-A	YDR201W	984
YDR320C-A	YER018C	963
YDR320C-A	YGL061C	984
YDR320C-A	YGR113W	957
YDR320C-A	YKL052C	998
YDR320C-A	YKL138C-A	991
YDR320C-A	YKR037C	996
YDR320C-A	YKR083C	991
YDR320C-A	YPR046W	963
YDR321W	YHR019C	932
YDR321W	YJL130C	917
YDR322C-A	YJR121W	998
YDR322C-A	YPL078C	998
YDR322W	YBL038W	997
YDR322W	YBR122C	974
YDR322W	YBR282W	970
YDR322W	YCR046C	993
YDR322W	YCR071C	988
YDR322W	YDR116C	974
YDR322W	YDR237W	989
YDR322W	YDR296W	992
YDR322W	YDR405W	988
YDR322W	YDR462W	999
YDR322W	YGR220C	997
YDR322W	YHR147C	970
YDR322W	YJL041W	915
YDR322W	YJL063C	974
YDR322W	YKL167C	974
YDR322W	YKR006C	974
YDR322W	YKR085C	974
YDR322W	YLR312W-A	987
YDR322W	YLR439W	974
YDR322W	YML025C	993
YDR322W	YMR024W	976
YDR322W	YMR193W	976

YDR322W	YNL005C	990
YDR322W	YNL185C	935
YDR322W	YNL252C	989
YDR322W	YNL284C	997
YDR322W	YOR150W	972
YDR323C	YGL095C	999
YDR323C	YLR148W	988
YDR323C	YLR240W	947
YDR323C	YLR396C	946
YDR323C	YMR231W	989
YDR323C	YOR036W	992
YDR323C	YOR089C	991
YDR324C	YBL004W	997
YDR324C	YBL072C	929
YDR324C	YBR247C	996
YDR324C	YCL059C	997
YDR324C	YCR057C	999
YDR324C	YDL014W	995
YDR324C	YDL060W	923
YDR324C	YDL148C	998
YDR324C	YDL153C	923
YDR324C	YDR299W	951
YDR324C	YDR398W	999
YDR324C	YDR449C	999
YDR324C	YEL055C	990
YDR324C	YER082C	999
YDR324C	YGL011C	967
YDR324C	YGL171W	916
YDR324C	YGR090W	999
YDR324C	YGR128C	999
YDR324C	YGR145W	953
YDR324C	YHR148W	998
YDR324C	YHR169W	912
YDR324C	YHR196W	999
YDR324C	YJL010C	988
YDR324C	YJL069C	999
YDR324C	YJL109C	999
YDR324C	YJR002W	999
YDR324C	YKR060W	997
YDR324C	YLL011W	997
YDR324C	YLR129W	988
YDR324C	YLR175W	915
YDR324C	YLR186W	997
YDR324C	YLR197W	997

YDR324C	YLR222C	997
YDR324C	YLR409C	999
YDR324C	YML093W	910
YDR324C	YMR093W	999
YDR324C	YMR128W	999
YDR324C	YMR229C	972
YDR324C	YNL075W	999
YDR324C	YNL132W	996
YDR324C	YOL010W	916
YDR324C	YOR078W	990
YDR324C	YOR145C	997
YDR324C	YOR310C	984
YDR324C	YPL126W	999
YDR324C	YPL217C	998
YDR324C	YPL266W	999
YDR324C	YPR112C	933
YDR324C	YPR137W	999
YDR324C	YPR144C	999
YDR325W	YBL097W	999
YDR325W	YFR031C	999
YDR325W	YLR086W	998
YDR325W	YLR272C	999
YDR328C	YBR127C	929
YDR328C	YBR280C	990
YDR328C	YDL132W	999
YDR328C	YDR054C	999
YDR328C	YDR139C	991
YDR328C	YDR202C	998
YDR328C	YDR219C	985
YDR328C	YDR306C	952
YDR328C	YFL009W	999
YDR328C	YGR140W	999
YDR328C	YGR188C	985
YDR328C	YIL046W	999
YDR328C	YJL149W	999
YDR328C	YJL187C	907
YDR328C	YJL204C	999
YDR328C	YJR033C	994
YDR328C	YJR053W	972
YDR328C	YJR060W	990
YDR328C	YJR090C	999
YDR328C	YKR014C	999
YDR328C	YLR079W	980
YDR328C	YLR097C	999

YDR328C	YLR224W	996
YDR328C	YLR352W	998
YDR328C	YLR368W	975
YDR328C	YML088W	999
YDR328C	YMR094W	999
YDR328C	YMR168C	999
YDR328C	YMR199W	976
YDR328C	YMR258C	999
YDR328C	YNL311C	924
YDR328C	YOL133W	999
YDR328C	YOR057W	999
YDR328C	YOR080W	998
YDR328C	YPL003W	901
YDR328C	YPL240C	962
YDR328C	YPL256C	999
YDR329C	YDL065C	999
YDR329C	YDR244W	917
YDR329C	YGL153W	945
YDR329C	YLR191W	944
YDR329C	YPL112C	998
YDR330W	YDL126C	947
YDR330W	YKL213C	931
YDR331W	YDR434W	998
YDR331W	YFL025C	942
YDR331W	YHR188C	999
YDR331W	YJL062W	927
YDR331W	YLR088W	999
YDR331W	YLR459W	992
YDR334W	YAL011W	999
YDR334W	YAR002W	914
YDR334W	YAR003W	900
YDR334W	YBR175W	937
YDR334W	YBR231C	999
YDR334W	YBR245C	956
YDR334W	YDL074C	984
YDR334W	YDR190C	999
YDR334W	YDR359C	929
YDR334W	YDR392W	919
YDR334W	YDR485C	999
YDR334W	YEL003W	917
YDR334W	YEL018W	985
YDR334W	YER092W	908
YDR334W	YFL024C	949
YDR334W	YFL039C	999

YDR334W	YGL043W	925
YDR334W	YGL058W	963
YDR334W	YGL127C	907
YDR334W	YGL244W	906
YDR334W	YGR002C	999
YDR334W	YJL030W	906
YDR334W	YJL081C	999
YDR334W	YJL168C	986
YDR334W	YLR015W	907
YDR334W	YLR055C	928
YDR334W	YLR085C	999
YDR334W	YLR385C	999
YDR334W	YLR399C	996
YDR334W	YLR418C	963
YDR334W	YLR438C-A	949
YDR334W	YML041C	999
YDR334W	YML094W	912
YDR334W	YMR263W	914
YDR334W	YNL107W	999
YDR334W	YNL136W	961
YDR334W	YNL153C	900
YDR334W	YOL012C	999
YDR334W	YOR026W	903
YDR334W	YOR123C	913
YDR334W	YOR244W	927
YDR334W	YOR349W	951
YDR334W	YPL055C	976
YDR334W	YPL235W	999
YDR334W	YPL241C	931
YDR335W	YDR005C	918
YDR335W	YDR146C	972
YDR335W	YDR477W	947
YDR335W	YER111C	977
YDR335W	YFR034C	998
YDR335W	YGL035C	947
YDR335W	YGR128C	905
YDR335W	YJL061W	984
YDR335W	YKL068W	948
YDR335W	YLR182W	972
YDR335W	YLR293C	976
YDR335W	YMR037C	956
YDR335W	YMR047C	953
YDR335W	YPL042C	994
YDR337W	Q0140	962

YDR337W	YBL090W	988
YDR337W	YBR146W	996
YDR337W	YBR251W	999
YDR337W	YDR036C	999
YDR337W	YDR041W	998
YDR337W	YDR175C	988
YDR337W	YDR347W	988
YDR337W	YGL129C	988
YDR337W	YGR084C	974
YDR337W	YGR165W	988
YDR337W	YGR215W	988
YDR337W	YHL004W	999
YDR337W	YIL093C	988
YDR337W	YJR101W	988
YDR337W	YJR113C	995
YDR337W	YKL155C	997
YDR337W	YMR158W	974
YDR337W	YMR188C	988
YDR337W	YNL137C	988
YDR337W	YNL306W	989
YDR337W	YNR036C	941
YDR337W	YNR037C	996
YDR337W	YOR158W	988
YDR337W	YPL013C	999
YDR337W	YPL118W	988
YDR339C	YCL059C	938
YDR339C	YEL026W	914
YDR339C	YHR148W	905
YDR339C	YJR002W	984
YDR339C	YLR003C	912
YDR339C	YLR186W	902
YDR339C	YOR145C	904
YDR339C	YPR112C	912
YDR341C	YBL076C	911
YDR341C	YDR037W	909
YDR341C	YGR264C	926
YDR341C	YHR018C	916
YDR341C	YHR091C	990
YDR341C	YLL018C	902
YDR341C	YOR168W	904
YDR341C	YPL040C	902
YDR341C	YPL160W	915
YDR345C	YDL194W	974
YDR345C	YDR277C	976

YDR345C	YJL129C	967
YDR345C	YJR090C	902
YDR345C	YKR050W	967
YDR346C	YDR297W	921
YDR346C	YHR112C	980
YDR346C	YJL134W	922
YDR347W	Q0140	962
YDR347W	YBL090W	988
YDR347W	YBR146W	988
YDR347W	YBR251W	999
YDR347W	YDR036C	999
YDR347W	YDR041W	996
YDR347W	YDR175C	988
YDR347W	YDR337W	988
YDR347W	YGL129C	988
YDR347W	YGR084C	974
YDR347W	YGR165W	988
YDR347W	YGR215W	988
YDR347W	YHL004W	999
YDR347W	YIL093C	997
YDR347W	YJR101W	997
YDR347W	YJR113C	988
YDR347W	YKL155C	997
YDR347W	YMR158W	974
YDR347W	YMR188C	988
YDR347W	YNL137C	988
YDR347W	YNL306W	988
YDR347W	YNR037C	987
YDR347W	YOR158W	998
YDR347W	YPL013C	996
YDR347W	YPL118W	988
YDR348C	YBL047C	936
YDR348C	YMR295C	962
YDR349C	YBR169C	946
YDR349C	YDR307W	923
YDR349C	YGR199W	923
YDR349C	YLR430W	952
YDR351W	YGR229C	923
YDR351W	YLR330W	926
YDR353W	YCR083W	979
YDR353W	YDR129C	905
YDR353W	YGR209C	999
YDR353W	YHR106W	999
YDR353W	YHR206W	940

YDR353W	YLR043C	996
YDR353W	YML028W	952
YDR353W	YNL180C	993
YDR353W	YPL091W	994
YDR354W	YDR007W	996
YDR354W	YER090W	994
YDR354W	YGL026C	978
YDR354W	YKL211C	999
YDR356W	YBL105C	969
YDR356W	YBR109C	999
YDR356W	YBR160W	992
YDR356W	YFL037W	909
YDR356W	YHR172W	999
YDR356W	YKL042W	999
YDR356W	YLR212C	999
YDR356W	YNL126W	999
YDR356W	YNL225C	993
YDR356W	YOR008C	969
YDR356W	YOR257W	969
YDR356W	YOR373W	968
YDR356W	YPL124W	998
YDR357C	YGL079W	935
YDR357C	YKL061W	992
YDR358W	YDL137W	989
YDR358W	YDL192W	915
YDR358W	YGL206C	976
YDR358W	YHR108W	999
YDR358W	YLL039C	939
YDR359C	YBR245C	931
YDR359C	YDL074C	931
YDR359C	YDR334W	929
YDR359C	YEL018W	999
YDR359C	YFL024C	999
YDR359C	YFL039C	991
YDR359C	YGL058W	928
YDR359C	YGL086W	903
YDR359C	YGR002C	999
YDR359C	YGR252W	973
YDR359C	YHR090C	999
YDR359C	YHR099W	999
YDR359C	YJL030W	936
YDR359C	YJL081C	999
YDR359C	YJL168C	987
YDR359C	YJR043C	980

YDR359C	YJR082C	996
YDR359C	YLR015W	914
YDR359C	YML007W	992
YDR359C	YNL107W	999
YDR359C	YNL136W	999
YDR359C	YOL012C	976
YDR359C	YOR244W	999
YDR359C	YPL055C	924
YDR359C	YPR023C	999
YDR361C	YBL087C	999
YDR361C	YDR208W	942
YDR361C	YER117W	999
YDR361C	YPL208W	999
YDR362C	YAL001C	999
YDR362C	YBR123C	999
YDR362C	YGR047C	999
YDR362C	YOR110W	999
YDR362C	YPL007C	999
YDR363W	YBR098W	944
YDR363W	YBR228W	945
YDR363W	YDL042C	980
YDR363W	YDR386W	909
YDR363W	YHR031C	952
YDR363W	YJL092W	956
YDR363W	YJR043C	917
YDR363W	YMR190C	977
YDR363W	YOL017W	917
YDR363W-A	YAR003W	930
YDR363W-A	YDL007W	999
YDR363W-A	YDL097C	999
YDR363W-A	YDL147W	999
YDR363W-A	YDR394W	999
YDR363W-A	YDR427W	999
YDR363W-A	YER021W	999
YDR363W-A	YFR004W	999
YDR363W-A	YFR010W	997
YDR363W-A	YFR052W	999
YDR363W-A	YGL048C	999
YDR363W-A	YGL141W	954
YDR363W-A	YGL233W	903
YDR363W-A	YGR232W	954
YDR363W-A	YHR027C	999
YDR363W-A	YHR200W	999
YDR363W-A	YIL075C	999

YDR363W-A	YJR084W	968
YDR363W-A	YKL145W	999
YDR363W-A	YLR166C	910
YDR363W-A	YLR421C	999
YDR363W-A	YMR191W	987
YDR363W-A	YOL012C	943
YDR363W-A	YOR117W	999
YDR363W-A	YOR259C	999
YDR363W-A	YOR261C	999
YDR363W-A	YPR055W	906
YDR363W-A	YPR108W	999
YDR364C	YAL032C	999
YDR364C	YBR065C	984
YDR364C	YDL209C	998
YDR364C	YDR088C	999
YDR364C	YDR369C	992
YDR364C	YDR416W	999
YDR364C	YER013W	945
YDR364C	YER172C	980
YDR364C	YGR006W	999
YDR364C	YGR129W	990
YDR364C	YGR278W	968
YDR364C	YHR165C	999
YDR364C	YKL095W	980
YDR364C	YKL173W	981
YDR364C	YKR086W	999
YDR364C	YLL036C	999
YDR364C	YLR117C	999
YDR364C	YLR150W	956
YDR364C	YML085C	982
YDR364C	YMR125W	966
YDR364C	YMR213W	995
YDR364C	YPL129W	962
YDR364C	YPL151C	970
YDR364C	YPL178W	956
YDR364C	YPR101W	988
YDR365C	YBR048W	977
YDR365C	YDL014W	912
YDR365C	YDR152W	940
YDR365C	YGR145W	992
YDR365C	YHR089C	973
YDR365C	YJL033W	988
YDR365C	YNR054C	999
YDR368W	YHR104W	973

YDR369C	YBL088C	999
YDR369C	YBR073W	970
YDR369C	YBR136W	920
YDR369C	YDL059C	969
YDR369C	YDL220C	914
YDR369C	YDR004W	971
YDR369C	YDR076W	969
YDR369C	YDR364C	992
YDR369C	YER095W	971
YDR369C	YGL090W	999
YDR369C	YGL163C	972
YDR369C	YHR031C	911
YDR369C	YJL092W	993
YDR369C	YJR043C	938
YDR369C	YKL113C	993
YDR369C	YML032C	996
YDR369C	YMR224C	999
YDR369C	YNL250W	999
YDR369C	YOR033C	998
YDR369C	YPL153C	946
YDR369C	YPR119W	904
YDR373W	YDR099W	976
YDR373W	YER177W	977
YDR373W	YNL267W	999
YDR375C	Q0105	986
YDR375C	YDR529C	921
YDR375C	YEL024W	936
YDR375C	YFR033C	984
YDR375C	YHR001W-A	949
YDR375C	YOR065W	917
YDR376W	YPL252C	949
YDR377W	Q0085	972
YDR377W	YBL099W	999
YDR377W	YDL004W	906
YDR377W	YJR121W	999
YDR377W	YKL016C	973
YDR377W	YML081C-A	984
YDR377W	YPL078C	999
YDR378C	YBL026W	999
YDR378C	YBR055C	993
YDR378C	YCR020C-A	984
YDR378C	YCR077C	999
YDR378C	YDL051W	919
YDR378C	YDL098C	989

YDR378C	YDL160C	989
YDR378C	YDR473C	999
YDR378C	YER029C	997
YDR378C	YER112W	999
YDR378C	YER146W	999
YDR378C	YER172C	999
YDR378C	YGL173C	999
YDR378C	YGR074W	931
YDR378C	YGR091W	999
YDR378C	YHR165C	999
YDR378C	YJL124C	999
YDR378C	YJR022W	999
YDR378C	YKL173W	993
YDR378C	YKL188C	966
YDR378C	YLR147C	999
YDR378C	YLR275W	999
YDR378C	YLR438C-A	999
YDR378C	YMR268C	999
YDR378C	YNL147W	999
YDR378C	YOL149W	971
YDR378C	YOR308C	997
YDR378C	YPR082C	997
YDR378C	YPR178W	996
YDR378C	YPR182W	900
YDR379W	YHR107C	945
YDR379W	YLR229C	971
YDR379W	YNL201C	977
YDR379W	YNL298W	921
YDR379W	YOR127W	999
YDR380W	YGL202W	948
YDR380W	YHR137W	969
YDR381W	YAL027W	929
YDR381W	YAR002W	941
YDR381W	YCL011C	999
YDR381W	YDL084W	999
YDR381W	YDR138W	999
YDR381W	YDR159W	941
YDR381W	YDR432W	991
YDR381W	YER110C	967
YDR381W	YER165W	962
YDR381W	YGL049C	948
YDR381W	YGL099W	930
YDR381W	YGL122C	976
YDR381W	YGL173C	930

YDR381W	YGR162W	948
YDR381W	YHR127W	976
YDR381W	YHR167W	998
YDR381W	YIL149C	934
YDR381W	YKL214C	993
YDR381W	YKR048C	983
YDR381W	YKR095W	934
YDR381W	YLR249W	969
YDR381W	YML062C	999
YDR381W	YMR237W	930
YDR381W	YMR308C	999
YDR381W	YNL004W	999
YDR381W	YNL061W	930
YDR381W	YNL112W	948
YDR381W	YNL139C	997
YDR381W	YNL308C	930
YDR381W	YOR080W	913
YDR381W	YOR294W	930
YDR381W	YPL169C	999
YDR381W	YPR094W	908
YDR382W	YAL003W	955
YDR382W	YAL035W	931
YDR382W	YBL027W	995
YDR382W	YBL072C	918
YDR382W	YBL087C	918
YDR382W	YBL092W	997
YDR382W	YBR031W	999
YDR382W	YBR048W	924
YDR382W	YBR084C-A	976
YDR382W	YBR118W	923
YDR382W	YBR181C	953
YDR382W	YBR189W	999
YDR382W	YBR191W	999
YDR382W	YCR031C	999
YDR382W	YDL061C	999
YDR382W	YDL075W	999
YDR382W	YDL081C	999
YDR382W	YDL082W	996
YDR382W	YDL083C	965
YDR382W	YDL130W	999
YDR382W	YDL136W	997
YDR382W	YDL191W	913
YDR382W	YDR012W	999
YDR382W	YDR064W	999

YDR382W	YDR385W	990
YDR382W	YDR418W	999
YDR382W	YDR447C	996
YDR382W	YDR450W	924
YDR382W	YDR471W	999
YDR382W	YDR500C	977
YDR382W	YEL054C	932
YDR382W	YER056C-A	996
YDR382W	YER074W	995
YDR382W	YER102W	982
YDR382W	YER117W	980
YDR382W	YER131W	953
YDR382W	YFL034C-A	924
YDR382W	YFR031C-A	995
YDR382W	YFR032C-A	998
YDR382W	YGL030W	999
YDR382W	YGL031C	942
YDR382W	YGL076C	998
YDR382W	YGL103W	999
YDR382W	YGL123W	999
YDR382W	YGL135W	996
YDR382W	YGL147C	918
YDR382W	YGL189C	917
YDR382W	YGR027C	986
YDR382W	YGR034W	996
YDR382W	YGR085C	999
YDR382W	YGR118W	997
YDR382W	YGR148C	994
YDR382W	YGR214W	994
YDR382W	YHL001W	999
YDR382W	YHL015W	986
YDR382W	YHL033C	999
YDR382W	YHR010W	980
YDR382W	YHR021C	920
YDR382W	YHR141C	996
YDR382W	YHR203C	985
YDR382W	YIL018W	995
YDR382W	YIL052C	999
YDR382W	YIL069C	996
YDR382W	YIL133C	996
YDR382W	YIL148W	970
YDR382W	YJL136C	981
YDR382W	YJL177W	999
YDR382W	YJL189W	973

YDR382W	YJL190C	995
YDR382W	YJL191W	993
YDR382W	YJR094W-A	986
YDR382W	YJR123W	999
YDR382W	YJR145C	941
YDR382W	YKL006W	999
YDR382W	YKL156W	974
YDR382W	YKL180W	999
YDR382W	YKR057W	932
YDR382W	YKR094C	907
YDR382W	YLL045C	998
YDR382W	YLR029C	999
YDR382W	YLR048W	999
YDR382W	YLR061W	999
YDR382W	YLR075W	999
YDR382W	YLR167W	968
YDR382W	YLR185W	976
YDR382W	YLR249W	940
YDR382W	YLR264W	994
YDR382W	YLR287C-A	995
YDR382W	YLR333C	932
YDR382W	YLR340W	999
YDR382W	YLR344W	994
YDR382W	YLR367W	968
YDR382W	YLR388W	999
YDR382W	YLR406C	938
YDR382W	YLR441C	996
YDR382W	YLR448W	999
YDR382W	YML024W	997
YDR382W	YML026C	924
YDR382W	YML063W	998
YDR382W	YML073C	996
YDR382W	YMR121C	935
YDR382W	YMR142C	932
YDR382W	YMR194W	999
YDR382W	YMR230W	949
YDR382W	YMR242C	996
YDR382W	YMR260C	909
YDR382W	YNL067W	972
YDR382W	YNL069C	996
YDR382W	YNL096C	996
YDR382W	YNL162W	945
YDR382W	YNL178W	999
YDR382W	YNL209W	966

YDR382W	YNL301C	999
YDR382W	YNL302C	926
YDR382W	YOL039W	998
YDR382W	YOL040C	939
YDR382W	YOL120C	912
YDR382W	YOL121C	927
YDR382W	YOL127W	999
YDR382W	YOR063W	999
YDR382W	YOR096W	999
YDR382W	YOR133W	944
YDR382W	YOR167C	957
YDR382W	YOR182C	914
YDR382W	YOR234C	999
YDR382W	YOR293W	967
YDR382W	YOR312C	996
YDR382W	YOR369C	935
YDR382W	YPL037C	999
YDR382W	YPL079W	972
YDR382W	YPL081W	937
YDR382W	YPL090C	901
YDR382W	YPL131W	999
YDR382W	YPL143W	982
YDR382W	YPL198W	940
YDR382W	YPL220W	999
YDR382W	YPL249C-A	997
YDR382W	YPR043W	963
YDR382W	YPR080W	913
YDR382W	YPR102C	993
YDR382W	YPR132W	976
YDR383C	YBR107C	985
YDR383C	YBR211C	999
YDR383C	YDR254W	996
YDR383C	YDR318W	998
YDR383C	YER016W	979
YDR383C	YGR179C	999
YDR383C	YIR010W	902
YDR383C	YJR135C	998
YDR383C	YLR315W	999
YDR383C	YPL018W	995
YDR383C	YPL233W	901
YDR383C	YPR046W	930
YDR385W	YBL027W	914
YDR385W	YBL071W-A	935
YDR385W	YBL072C	912

YDR385W	YBL087C	928
YDR385W	YBL092W	914
YDR385W	YBR031W	929
YDR385W	YBR048W	989
YDR385W	YBR084C-A	914
YDR385W	YBR118W	946
YDR385W	YBR181C	906
YDR385W	YBR191W	913
YDR385W	YCR031C	989
YDR385W	YDL061C	989
YDR385W	YDL075W	909
YDR385W	YDL081C	998
YDR385W	YDL082W	914
YDR385W	YDL083C	909
YDR385W	YDL130W	997
YDR385W	YDR012W	929
YDR385W	YDR064W	992
YDR385W	YDR172W	900
YDR385W	YDR382W	990
YDR385W	YDR418W	901
YDR385W	YDR447C	909
YDR385W	YDR450W	990
YDR385W	YEL054C	901
YDR385W	YER102W	912
YDR385W	YER117W	914
YDR385W	YFL034C-A	909
YDR385W	YFR031C-A	913
YDR385W	YFR032C-A	923
YDR385W	YGL030W	961
YDR385W	YGL076C	913
YDR385W	YGL103W	906
YDR385W	YGL123W	991
YDR385W	YGL135W	906
YDR385W	YGL147C	910
YDR385W	YGR027C	907
YDR385W	YGR034W	909
YDR385W	YGR085C	916
YDR385W	YGR118W	995
YDR385W	YGR187C	980
YDR385W	YGR214W	991
YDR385W	YHL001W	908
YDR385W	YHL015W	996
YDR385W	YHL033C	903
YDR385W	YHR021C	912

YDR385W	YHR141C	913
YDR385W	YHR203C	912
YDR385W	YIL018W	913
YDR385W	YIL103W	994
YDR385W	YIL133C	911
YDR385W	YIL148W	903
YDR385W	YJL136C	911
YDR385W	YJL138C	962
YDR385W	YJL177W	901
YDR385W	YJL189W	908
YDR385W	YJL190C	989
YDR385W	YJL191W	901
YDR385W	YJR094W-A	918
YDR385W	YJR123W	997
YDR385W	YJR145C	912
YDR385W	YKL006W	908
YDR385W	YKL060C	968
YDR385W	YKL152C	970
YDR385W	YKL156W	912
YDR385W	YKL180W	901
YDR385W	YKL191W	996
YDR385W	YKR057W	911
YDR385W	YKR094C	903
YDR385W	YLL045C	903
YDR385W	YLR048W	916
YDR385W	YLR061W	911
YDR385W	YLR172C	967
YDR385W	YLR249W	972
YDR385W	YLR264W	905
YDR385W	YLR333C	907
YDR385W	YLR340W	999
YDR385W	YLR344W	909
YDR385W	YLR367W	901
YDR385W	YLR406C	909
YDR385W	YLR441C	919
YDR385W	YLR448W	919
YDR385W	YML024W	909
YDR385W	YML026C	912
YDR385W	YML063W	918
YDR385W	YML073C	917
YDR385W	YMR083W	972
YDR385W	YMR142C	914
YDR385W	YMR194W	911
YDR385W	YMR230W	910

YDR385W	YMR242C	917
YDR385W	YNL067W	911
YDR385W	YNL069C	910
YDR385W	YNL096C	902
YDR385W	YNL162W	913
YDR385W	YNL178W	993
YDR385W	YNL301C	928
YDR385W	YNL302C	907
YDR385W	YOL039W	997
YDR385W	YOL040C	991
YDR385W	YOL086C	968
YDR385W	YOL120C	916
YDR385W	YOL121C	907
YDR385W	YOL127W	907
YDR385W	YOR063W	930
YDR385W	YOR096W	902
YDR385W	YOR133W	999
YDR385W	YOR167C	905
YDR385W	YOR234C	913
YDR385W	YOR293W	910
YDR385W	YOR312C	917
YDR385W	YOR369C	901
YDR385W	YPL079W	913
YDR385W	YPL081W	989
YDR385W	YPL090C	906
YDR385W	YPL131W	919
YDR385W	YPL143W	913
YDR385W	YPL198W	913
YDR385W	YPL220W	906
YDR385W	YPL249C-A	912
YDR385W	YPR043W	918
YDR385W	YPR080W	947
YDR385W	YPR102C	916
YDR385W	YPR132W	955
YDR386W	YBR098W	999
YDR386W	YBR223C	900
YDR386W	YDR279W	913
YDR386W	YDR363W	909
YDR386W	YER095W	964
YDR386W	YGL163C	951
YDR386W	YJL092W	995
YDR386W	YKL113C	957
YDR386W	YLR234W	996
YDR386W	YLR373C	929

YDR386W	YML095C	938
YDR386W	YMR190C	999
YDR386W	YOR144C	957
YDR386W	YPL022W	944
YDR386W	YPL024W	998
YDR388W	YBL007C	999
YDR388W	YBR108W	999
YDR388W	YCR009C	999
YDR388W	YCR088W	999
YDR388W	YDL127W	976
YDR388W	YDL179W	906
YDR388W	YER125W	955
YDR388W	YFL039C	999
YDR388W	YGL060W	986
YDR388W	YGL181W	914
YDR388W	YGR229C	921
YDR388W	YGR268C	917
YDR388W	YHR016C	907
YDR388W	YHR030C	962
YDR388W	YIR003W	919
YDR388W	YJL020C	933
YDR388W	YJR083C	980
YDR388W	YLR144C	999
YDR388W	YLR337C	997
YDR388W	YLR372W	915
YDR388W	YML052W	906
YDR388W	YML058W	901
YDR388W	YMR109W	995
YDR388W	YMR192W	999
YDR388W	YNL084C	965
YDR388W	YNL094W	999
YDR388W	YNL138W	984
YDR388W	YOR089C	970
YDR388W	YOR181W	999
YDR388W	YOR326W	976
YDR388W	YPL031C	997
YDR388W	YPL249C	999
YDR388W	YPR171W	995
YDR389W	YBR229C	905
YDR389W	YFL039C	968
YDR389W	YKL203C	998
YDR389W	YLR371W	974
YDR389W	YMR200W	983
YDR389W	YPR165W	996

YDR390C	YDR510W	999
YDR390C	YIL031W	929
YDR390C	YKR002W	996
YDR390C	YPR180W	999
YDR392W	YAL021C	970
YDR392W	YBR081C	999
YDR392W	YBR111W-A	959
YDR392W	YBR115C	976
YDR392W	YBR198C	999
YDR392W	YBR231C	907
YDR392W	YCL010C	999
YDR392W	YCL030C	988
YDR392W	YCR093W	987
YDR392W	YDR145W	999
YDR392W	YDR167W	999
YDR392W	YDR176W	999
YDR392W	YDR334W	919
YDR392W	YDR448W	999
YDR392W	YDR485C	904
YDR392W	YEL009C	961
YDR392W	YER148W	999
YDR392W	YER164W	983
YDR392W	YGL066W	999
YDR392W	YGL070C	960
YDR392W	YGL112C	999
YDR392W	YGR252W	999
YDR392W	YGR274C	991
YDR392W	YHR099W	999
YDR392W	YKL058W	979
YDR392W	YLR055C	999
YDR392W	YLR085C	916
YDR392W	YML041C	904
YDR392W	YMR223W	999
YDR392W	YMR236W	999
YDR392W	YNL107W	917
YDR392W	YOL012C	922
YDR392W	YOL148C	999
YDR392W	YPL047W	999
YDR392W	YPL082C	999
YDR392W	YPL240C	919
YDR392W	YPL248C	950
YDR392W	YPL254W	999
YDR394W	YBL041W	905
YDR394W	YBR211C	999

YDR394W	YBR272C	978
YDR394W	YCR014C	970
YDR394W	YDL007W	999
YDR394W	YDL097C	999
YDR394W	YDL147W	999
YDR394W	YDR363W-A	999
YDR394W	YDR427W	999
YDR394W	YEL037C	995
YDR394W	YER012W	999
YDR394W	YER021W	999
YDR394W	YER094C	913
YDR394W	YFL007W	956
YDR394W	YFR004W	999
YDR394W	YFR010W	999
YDR394W	YFR050C	910
YDR394W	YFR052W	999
YDR394W	YGL004C	932
YDR394W	YGL048C	999
YDR394W	YGL141W	954
YDR394W	YGR232W	999
YDR394W	YGR253C	910
YDR394W	YHL030W	998
YDR394W	YHR027C	999
YDR394W	YHR200W	999
YDR394W	YIL007C	902
YDR394W	YIL075C	998
YDR394W	YJR109C	967
YDR394W	YKL145W	999
YDR394W	YLR421C	999
YDR394W	YMR191W	987
YDR394W	YMR216C	998
YDR394W	YMR314W	997
YDR394W	YOL038W	998
YDR394W	YOR117W	999
YDR394W	YOR157C	903
YDR394W	YOR259C	999
YDR394W	YOR261C	999
YDR394W	YOR362C	909
YDR394W	YPR103W	918
YDR394W	YPR108W	999
YDR394W	YPR131C	950
YDR395W	YAL059W	969
YDR395W	YDL051W	996
YDR395W	YKL068W	913

YDR397C	YER022W	912
YDR397C	YER148W	991
YDR397C	YER159C	999
YDR397C	YNL236W	973
YDR398W	YBR247C	963
YDR398W	YDL153C	910
YDR398W	YDR324C	999
YDR398W	YEL055C	992
YDR398W	YGL120C	911
YDR398W	YGL171W	914
YDR398W	YGR128C	999
YDR398W	YHR148W	911
YDR398W	YHR169W	922
YDR398W	YHR196W	999
YDR398W	YJL010C	983
YDR398W	YJL069C	909
YDR398W	YJL109C	999
YDR398W	YJR002W	986
YDR398W	YLL011W	914
YDR398W	YLR129W	926
YDR398W	YLR175W	919
YDR398W	YLR186W	914
YDR398W	YLR197W	915
YDR398W	YLR222C	923
YDR398W	YLR409C	914
YDR398W	YML093W	912
YDR398W	YMR093W	999
YDR398W	YMR229C	917
YDR398W	YMR290C	921
YDR398W	YNL075W	918
YDR398W	YOR004W	920
YDR398W	YOR145C	917
YDR398W	YOR310C	922
YDR398W	YPL126W	999
YDR398W	YPR144C	911
YDR399W	YDR454C	976
YDR399W	YOR128C	960
YDR400W	YDL042C	911
YDR400W	YGL037C	933
YDR400W	YHR128W	928
YDR400W	YLR209C	994
YDR400W	YLR245C	934
YDR400W	YNL129W	993
YDR400W	YPR062W	929

YDR402C	YDR403W	908
YDR403W	YDR402C	908
YDR404C	YBL026W	984
YDR404C	YBR154C	999
YDR404C	YBR198C	900
YDR404C	YDL115C	997
YDR404C	YDL140C	999
YDR404C	YDR167W	911
YDR404C	YEL026W	907
YDR404C	YER022W	981
YDR404C	YER148W	987
YDR404C	YER171W	925
YDR404C	YGL043W	995
YDR404C	YGL070C	999
YDR404C	YGL112C	901
YDR404C	YGL127C	906
YDR404C	YGR005C	999
YDR404C	YGR063C	999
YDR404C	YGR186W	999
YDR404C	YHR058C	901
YDR404C	YHR143W-A	999
YDR404C	YIL021W	999
YDR404C	YIL143C	923
YDR404C	YJL140W	999
YDR404C	YJR035W	901
YDR404C	YKL058W	904
YDR404C	YLR071C	980
YDR404C	YLR275W	900
YDR404C	YML010W	999
YDR404C	YMR277W	956
YDR404C	YOL005C	999
YDR404C	YOL135C	912
YDR404C	YOR116C	932
YDR404C	YOR151C	999
YDR404C	YOR207C	927
YDR404C	YOR210W	999
YDR404C	YOR224C	999
YDR404C	YPL129W	999
YDR404C	YPL178W	901
YDR404C	YPR086W	939
YDR404C	YPR168W	938
YDR404C	YPR187W	999
YDR405W	YBR122C	970
YDR405W	YCR071C	988

YDR405W	YDR116C	987
YDR405W	YDR237W	970
YDR405W	YDR296W	970
YDR405W	YDR322W	988
YDR405W	YER154W	916
YDR405W	YGR076C	981
YDR405W	YGR220C	966
YDR405W	YKL167C	988
YDR405W	YKR006C	970
YDR405W	YLR439W	987
YDR405W	YML025C	992
YDR405W	YMR024W	987
YDR405W	YNL005C	986
YDR405W	YNL185C	931
YDR406W	YBL005W	935
YDR406W	YDR011W	909
YDR406W	YGL013C	984
YDR406W	YGR281W	920
YDR406W	YIL013C	908
YDR406W	YOR153W	984
YDR406W	YOR328W	903
YDR407C	YBR254C	999
YDR407C	YDR108W	999
YDR407C	YDR246W	999
YDR407C	YDR472W	999
YDR407C	YER031C	936
YDR407C	YGR166W	999
YDR407C	YKR068C	999
YDR407C	YML077W	999
YDR407C	YMR218C	999
YDR407C	YOR115C	999
YDR408C	YAR015W	977
YDR408C	YBR084W	928
YDR408C	YBR263W	927
YDR408C	YGL234W	999
YDR408C	YGR061C	961
YDR408C	YGR204W	974
YDR408C	YKR080W	941
YDR408C	YLR028C	980
YDR408C	YLR058C	924
YDR408C	YMR120C	979
YDR408C	YMR300C	940
YDR408C	YOR128C	996
YDR409W	YBR088C	921

YDR409W	YCR066W	999
YDR409W	YDL064W	999
YDR409W	YDR510W	998
YDR409W	YEL019C	954
YDR409W	YIL031W	926
YDR409W	YLR032W	976
YDR409W	YLR314C	989
YDR409W	YML032C	957
YDR409W	YOR156C	999
YDR410C	YNL098C	970
YDR410C	YOL110W	923
YDR411C	YBL058W	929
YDR411C	YBR170C	971
YDR411C	YBR201W	954
YDR411C	YBR273C	971
YDR411C	YDL126C	999
YDR411C	YML013W	996
YDR412W	YER133W	984
YDR415C	YKL210W	999
YDR416W	YAL032C	999
YDR416W	YBR188C	999
YDR416W	YDL030W	974
YDR416W	YDL209C	999
YDR416W	YDR364C	999
YDR416W	YDR420W	938
YDR416W	YER013W	922
YDR416W	YER172C	992
YDR416W	YGL120C	974
YDR416W	YGL128C	972
YDR416W	YGR129W	999
YDR416W	YGR278W	997
YDR416W	YHR165C	999
YDR416W	YIL143C	900
YDR416W	YJR050W	999
YDR416W	YKL095W	998
YDR416W	YKL173W	976
YDR416W	YLL036C	999
YDR416W	YLR117C	999
YDR416W	YLR424W	994
YDR416W	YML049C	976
YDR416W	YMR213W	999
YDR416W	YMR288W	974
YDR416W	YPL151C	999
YDR416W	YPL213W	998

YDR416W	YPR101W	999
YDR418W	YAL035W	998
YDR418W	YBL027W	990
YDR418W	YBL072C	996
YDR418W	YBL087C	996
YDR418W	YBL092W	994
YDR418W	YBR031W	999
YDR418W	YBR048W	997
YDR418W	YBR084C-A	958
YDR418W	YBR118W	907
YDR418W	YBR181C	994
YDR418W	YBR189W	999
YDR418W	YBR191W	999
YDR418W	YCR031C	999
YDR418W	YDL061C	982
YDR418W	YDL075W	999
YDR418W	YDL081C	988
YDR418W	YDL082W	997
YDR418W	YDL083C	966
YDR418W	YDL130W	991
YDR418W	YDL136W	995
YDR418W	YDL191W	935
YDR418W	YDR012W	999
YDR418W	YDR025W	926
YDR418W	YDR064W	999
YDR418W	YDR382W	999
YDR418W	YDR385W	901
YDR418W	YDR447C	996
YDR418W	YDR450W	913
YDR418W	YDR471W	999
YDR418W	YDR500C	963
YDR418W	YEL054C	986
YDR418W	YER056C-A	987
YDR418W	YER074W	924
YDR418W	YER102W	968
YDR418W	YER117W	963
YDR418W	YER131W	964
YDR418W	YFL034C-A	920
YDR418W	YFR031C-A	999
YDR418W	YFR032C-A	967
YDR418W	YGL030W	999
YDR418W	YGL031C	995
YDR418W	YGL076C	999
YDR418W	YGL103W	999

YDR418W	YGL123W	999
YDR418W	YGL135W	999
YDR418W	YGL147C	999
YDR418W	YGL189C	923
YDR418W	YGR027C	987
YDR418W	YGR034W	931
YDR418W	YGR085C	999
YDR418W	YGR118W	996
YDR418W	YGR148C	995
YDR418W	YGR214W	999
YDR418W	YHL001W	999
YDR418W	YHL015W	983
YDR418W	YHL033C	999
YDR418W	YHR010W	999
YDR418W	YHR021C	930
YDR418W	YHR141C	999
YDR418W	YHR203C	998
YDR418W	YIL018W	995
YDR418W	YIL052C	997
YDR418W	YIL069C	965
YDR418W	YIL133C	999
YDR418W	YIL148W	951
YDR418W	YJL136C	974
YDR418W	YJL177W	999
YDR418W	YJL189W	931
YDR418W	YJL190C	996
YDR418W	YJL191W	982
YDR418W	YJR094W-A	997
YDR418W	YJR123W	999
YDR418W	YJR145C	926
YDR418W	YKL006W	999
YDR418W	YKL156W	969
YDR418W	YKL180W	999
YDR418W	YKR057W	932
YDR418W	YKR094C	906
YDR418W	YLL045C	998
YDR418W	YLR029C	997
YDR418W	YLR048W	999
YDR418W	YLR061W	995
YDR418W	YLR075W	999
YDR418W	YLR150W	978
YDR418W	YLR167W	978
YDR418W	YLR185W	996
YDR418W	YLR264W	978

YDR418W	YLR287C-A	912
YDR418W	YLR325C	924
YDR418W	YLR333C	931
YDR418W	YLR340W	999
YDR418W	YLR344W	997
YDR418W	YLR367W	970
YDR418W	YLR388W	996
YDR418W	YLR406C	924
YDR418W	YLR441C	996
YDR418W	YLR448W	999
YDR418W	YML024W	996
YDR418W	YML026C	913
YDR418W	YML063W	999
YDR418W	YML073C	996
YDR418W	YMR121C	928
YDR418W	YMR142C	945
YDR418W	YMR194W	999
YDR418W	YMR230W	954
YDR418W	YMR242C	997
YDR418W	YMR260C	922
YDR418W	YNL067W	994
YDR418W	YNL069C	996
YDR418W	YNL096C	943
YDR418W	YNL162W	930
YDR418W	YNL178W	999
YDR418W	YNL301C	995
YDR418W	YNL302C	929
YDR418W	YOL039W	990
YDR418W	YOL040C	950
YDR418W	YOL120C	913
YDR418W	YOL121C	929
YDR418W	YOL127W	999
YDR418W	YOR063W	999
YDR418W	YOR096W	999
YDR418W	YOR133W	920
YDR418W	YOR167C	948
YDR418W	YOR182C	912
YDR418W	YOR234C	979
YDR418W	YOR293W	989
YDR418W	YOR312C	996
YDR418W	YOR369C	952
YDR418W	YPL037C	970
YDR418W	YPL079W	974
YDR418W	YPL081W	968

YDR418W	YPL131W	999
YDR418W	YPL143W	983
YDR418W	YPL198W	943
YDR418W	YPL220W	999
YDR418W	YPL249C-A	997
YDR418W	YPR043W	942
YDR418W	YPR080W	907
YDR418W	YPR102C	999
YDR418W	YPR132W	964
YDR419W	YBR088C	999
YDR419W	YBR173C	972
YDR419W	YCR066W	936
YDR419W	YDR092W	959
YDR419W	YER142C	976
YDR419W	YFR027W	961
YDR419W	YGL058W	924
YDR419W	YGL087C	999
YDR419W	YIL139C	966
YDR419W	YJL092W	957
YDR419W	YJR043C	989
YDR419W	YLR032W	976
YDR419W	YML060W	998
YDR419W	YMR201C	982
YDR419W	YOR346W	999
YDR419W	YPL167C	999
YDR420W	YDR416W	938
YDR421W	YBR107C	918
YDR421W	YHR137W	928
YDR422C	YDR477W	999
YDR422C	YER027C	999
YDR422C	YER129W	999
YDR422C	YGL115W	999
YDR422C	YGL208W	999
YDR422C	YJL089W	957
YDR422C	YKL048C	946
YDR422C	YMR280C	929
YDR423C	YML007W	996
YDR424C	YDR488C	948
YDR424C	YIL115C	978
YDR424C	YJL041W	978
YDR424C	YJL061W	978
YDR424C	YKR082W	954
YDR425W	YDL113C	940
YDR425W	YJL036W	999

YDR427W	YBL041W	916
YDR427W	YBR272C	995
YDR427W	YCR014C	970
YDR427W	YDL007W	999
YDR427W	YDL097C	999
YDR427W	YDL147W	999
YDR427W	YDR363W-A	999
YDR427W	YDR394W	999
YDR427W	YEL037C	998
YDR427W	YER012W	999
YDR427W	YER021W	999
YDR427W	YER094C	921
YDR427W	YFL007W	969
YDR427W	YFR004W	999
YDR427W	YFR010W	999
YDR427W	YFR050C	915
YDR427W	YFR052W	999
YDR427W	YGL004C	961
YDR427W	YGL048C	999
YDR427W	YGL141W	954
YDR427W	YGR067C	998
YDR427W	YGR135W	901
YDR427W	YGR232W	999
YDR427W	YGR253C	916
YDR427W	YHL030W	998
YDR427W	YHR027C	999
YDR427W	YHR200W	999
YDR427W	YIL075C	999
YDR427W	YJL001W	900
YDR427W	YKL145W	999
YDR427W	YLL039C	970
YDR427W	YLR076C	998
YDR427W	YLR386W	998
YDR427W	YLR421C	999
YDR427W	YML092C	900
YDR427W	YMR191W	987
YDR427W	YMR314W	998
YDR427W	YNL244C	999
YDR427W	YOL038W	973
YDR427W	YOR117W	999
YDR427W	YOR157C	928
YDR427W	YOR259C	999
YDR427W	YOR261C	999
YDR427W	YOR362C	918

YDR427W	YPR103W	926
YDR427W	YPR108W	999
YDR429C	YAL035W	976
YDR429C	YBL072C	915
YDR429C	YBR048W	916
YDR429C	YBR079C	999
YDR429C	YBR181C	934
YDR429C	YDR025W	917
YDR429C	YDR064W	906
YDR429C	YDR447C	901
YDR429C	YER025W	987
YDR429C	YER074W	919
YDR429C	YER102W	997
YDR429C	YER131W	903
YDR429C	YFL017C	922
YDR429C	YGL123W	903
YDR429C	YGL189C	903
YDR429C	YGR214W	900
YDR429C	YIL069C	904
YDR429C	YJL190C	914
YDR429C	YJL191W	919
YDR429C	YJR123W	927
YDR429C	YLR048W	916
YDR429C	YLR192C	999
YDR429C	YLR367W	914
YDR429C	YLR441C	904
YDR429C	YML024W	916
YDR429C	YML063W	919
YDR429C	YMR146C	999
YDR429C	YMR230W	923
YDR429C	YMR309C	999
YDR429C	YNL096C	901
YDR429C	YNL178W	904
YDR429C	YNL244C	999
YDR429C	YOL040C	900
YDR429C	YOR096W	916
YDR429C	YOR204W	998
YDR429C	YOR293W	901
YDR429C	YOR361C	999
YDR429C	YPR041W	999
YDR429C	YPR086W	974
YDR429C	YPR163C	986
YDR430C	YBR221C	999
YDR430C	YER178W	987

YDR430C	YFL018C	985
YDR430C	YGR193C	987
YDR430C	YNL071W	971
YDR432W	YBR019C	996
YDR432W	YBR034C	999
YDR432W	YCL011C	998
YDR432W	YDL084W	968
YDR432W	YDL140C	975
YDR432W	YDL175C	980
YDR432W	YDR381W	991
YDR432W	YER133W	936
YDR432W	YGL044C	976
YDR432W	YGL049C	970
YDR432W	YGL173C	973
YDR432W	YHR086W	983
YDR432W	YIL021W	975
YDR432W	YMR125W	999
YDR432W	YMR216C	999
YDR432W	YNL016W	976
YDR432W	YOL123W	999
YDR432W	YOR048C	963
YDR432W	YOR160W	999
YDR432W	YOR204W	963
YDR432W	YOR310C	940
YDR432W	YPL169C	962
YDR432W	YPL178W	999
YDR432W	YPL263C	940
YDR434W	YDR331W	998
YDR434W	YHR188C	999
YDR434W	YLR088W	997
YDR434W	YLR459W	989
YDR435C	YGL190C	932
YDR435C	YOR014W	914
YDR436W	YDR040C	979
YDR436W	YHR030C	970
YDR436W	YKR072C	958
YDR436W	YML016C	999
YDR437W	YGR216C	910
YDR437W	YPL076W	986
YDR439W	YCR086W	999
YDR439W	YER106W	979
YDR439W	YJL076W	911
YDR439W	YKR010C	943
YDR440W	YBR010W	999

YDR440W	YCR066W	976
YDR440W	YDL042C	991
YDR440W	YDR285W	947
YDR440W	YER179W	946
YDR440W	YHR119W	958
YDR440W	YJL168C	954
YDR440W	YJR043C	906
YDR440W	YLR442C	969
YDR440W	YNL031C	999
YDR441C	YDL238C	912
YDR441C	YDR226W	901
YDR441C	YDR454C	919
YDR441C	YJR105W	918
YDR441C	YLR028C	901
YDR441C	YLR209C	913
YDR441C	YLR359W	913
YDR441C	YML022W	991
YDR441C	YML035C	909
YDR441C	YMR120C	902
YDR441C	YMR217W	909
YDR443C	YBL093C	999
YDR443C	YBR193C	997
YDR443C	YBR253W	999
YDR443C	YBR279W	907
YDR443C	YCR081W	999
YDR443C	YDL005C	999
YDR443C	YDL140C	984
YDR443C	YDR308C	998
YDR443C	YDR477W	971
YDR443C	YER022W	999
YDR443C	YFL028C	914
YDR443C	YGL025C	998
YDR443C	YGL035C	982
YDR443C	YGL070C	928
YDR443C	YGL127C	999
YDR443C	YGL151W	998
YDR443C	YGR097W	910
YDR443C	YGR104C	999
YDR443C	YHR041C	997
YDR443C	YHR058C	997
YDR443C	YJL140W	922
YDR443C	YJL164C	965
YDR443C	YLR071C	999
YDR443C	YML007W	974

YDR443C	YML098W	909
YDR443C	YMR112C	997
YDR443C	YNL025C	999
YDR443C	YNL098C	962
YDR443C	YNL236W	999
YDR443C	YNR010W	997
YDR443C	YOL051W	999
YDR443C	YOL135C	999
YDR443C	YOR123C	916
YDR443C	YOR140W	943
YDR443C	YOR174W	997
YDR443C	YPL042C	999
YDR443C	YPL129W	916
YDR443C	YPL240C	919
YDR443C	YPR070W	999
YDR443C	YPR168W	998
YDR446W	YJL194W	982
YDR446W	YOR156C	983
YDR447C	YBL027W	996
YDR447C	YBL072C	995
YDR447C	YBL087C	966
YDR447C	YBL092W	997
YDR447C	YBR031W	996
YDR447C	YBR048W	996
YDR447C	YBR084C-A	920
YDR447C	YBR118W	910
YDR447C	YBR181C	998
YDR447C	YBR189W	994
YDR447C	YBR191W	996
YDR447C	YCR031C	996
YDR447C	YDL014W	956
YDR447C	YDL060W	982
YDR447C	YDL061C	911
YDR447C	YDL075W	996
YDR447C	YDL081C	949
YDR447C	YDL082W	997
YDR447C	YDL083C	970
YDR447C	YDL130W	906
YDR447C	YDL136W	996
YDR447C	YDL191W	926
YDR447C	YDR012W	996
YDR447C	YDR025W	918
YDR447C	YDR064W	997
YDR447C	YDR382W	996

YDR447C	YDR385W	909
YDR447C	YDR418W	996
YDR447C	YDR429C	901
YDR447C	YDR450W	996
YDR447C	YDR471W	995
YDR447C	YDR500C	916
YDR447C	YEL054C	930
YDR447C	YER056C-A	924
YDR447C	YER074W	995
YDR447C	YER102W	995
YDR447C	YER117W	928
YDR447C	YER131W	997
YDR447C	YFL034C-A	911
YDR447C	YFR031C-A	997
YDR447C	YFR032C-A	997
YDR447C	YGL014W	954
YDR447C	YGL030W	997
YDR447C	YGL031C	996
YDR447C	YGL076C	997
YDR447C	YGL103W	997
YDR447C	YGL123W	914
YDR447C	YGL135W	996
YDR447C	YGL147C	998
YDR447C	YGL189C	921
YDR447C	YGL246C	974
YDR447C	YGR027C	937
YDR447C	YGR034W	998
YDR447C	YGR085C	996
YDR447C	YGR118W	998
YDR447C	YGR148C	910
YDR447C	YGR214W	998
YDR447C	YHL001W	996
YDR447C	YHL015W	938
YDR447C	YHL033C	996
YDR447C	YHR010W	907
YDR447C	YHR021C	929
YDR447C	YHR141C	998
YDR447C	YHR203C	996
YDR447C	YIL018W	996
YDR447C	YIL052C	924
YDR447C	YIL069C	996
YDR447C	YIL133C	998
YDR447C	YIL148W	906
YDR447C	YJL136C	936

YDR447C	YJL177W	996
YDR447C	YJL189W	908
YDR447C	YJL190C	998
YDR447C	YJL191W	998
YDR447C	YJR094W-A	932
YDR447C	YJR123W	997
YDR447C	YJR145C	995
YDR447C	YKL006W	933
YDR447C	YKL156W	929
YDR447C	YKL180W	996
YDR447C	YKR057W	936
YDR447C	YKR094C	906
YDR447C	YLL045C	996
YDR447C	YLR029C	996
YDR447C	YLR048W	925
YDR447C	YLR061W	914
YDR447C	YLR075W	996
YDR447C	YLR150W	956
YDR447C	YLR167W	901
YDR447C	YLR185W	917
YDR447C	YLR192C	914
YDR447C	YLR264W	915
YDR447C	YLR287C-A	995
YDR447C	YLR325C	965
YDR447C	YLR333C	929
YDR447C	YLR340W	947
YDR447C	YLR344W	997
YDR447C	YLR367W	998
YDR447C	YLR388W	911
YDR447C	YLR406C	923
YDR447C	YLR441C	998
YDR447C	YLR448W	996
YDR447C	YML024W	984
YDR447C	YML026C	919
YDR447C	YML063W	996
YDR447C	YML073C	997
YDR447C	YMR121C	920
YDR447C	YMR142C	997
YDR447C	YMR194W	996
YDR447C	YMR230W	932
YDR447C	YMR242C	998
YDR447C	YMR260C	905
YDR447C	YNL067W	924
YDR447C	YNL069C	997

YDR447C	YNL096C	997
YDR447C	YNL132W	945
YDR447C	YNL162W	935
YDR447C	YNL178W	998
YDR447C	YNL301C	996
YDR447C	YNL302C	998
YDR447C	YOL039W	913
YDR447C	YOL040C	998
YDR447C	YOL120C	921
YDR447C	YOL121C	956
YDR447C	YOL127W	995
YDR447C	YOL139C	916
YDR447C	YOR063W	995
YDR447C	YOR096W	995
YDR447C	YOR133W	909
YDR447C	YOR167C	914
YDR447C	YOR182C	902
YDR447C	YOR234C	998
YDR447C	YOR293W	932
YDR447C	YOR312C	996
YDR447C	YOR369C	971
YDR447C	YPL079W	932
YDR447C	YPL081W	919
YDR447C	YPL090C	995
YDR447C	YPL131W	914
YDR447C	YPL143W	930
YDR447C	YPL198W	995
YDR447C	YPL220W	996
YDR447C	YPL237W	900
YDR447C	YPL249C-A	997
YDR447C	YPR043W	932
YDR447C	YPR080W	910
YDR447C	YPR102C	933
YDR447C	YPR132W	933
YDR448W	YBR081C	999
YDR448W	YBR111W-A	998
YDR448W	YBR198C	999
YDR448W	YBR253W	995
YDR448W	YCL010C	999
YDR448W	YDL165W	901
YDR448W	YDR145W	999
YDR448W	YDR159W	935
YDR448W	YDR167W	999
YDR448W	YDR176W	999

YDR448W	YDR216W	937
YDR448W	YDR224C	940
YDR448W	YDR225W	919
YDR448W	YDR308C	960
YDR448W	YDR392W	999
YDR448W	YEL009C	999
YDR448W	YER022W	957
YDR448W	YER148W	999
YDR448W	YER164W	993
YDR448W	YGL066W	999
YDR448W	YGL112C	999
YDR448W	YGL151W	994
YDR448W	YGR104C	915
YDR448W	YGR252W	999
YDR448W	YGR274C	999
YDR448W	YHR041C	999
YDR448W	YHR058C	986
YDR448W	YHR079C	935
YDR448W	YHR099W	999
YDR448W	YLR055C	999
YDR448W	YML007W	985
YDR448W	YML015C	996
YDR448W	YMR223W	999
YDR448W	YMR236W	999
YDR448W	YNL236W	992
YDR448W	YOL072W	935
YDR448W	YOL135C	997
YDR448W	YOL148C	999
YDR448W	YOR023C	950
YDR448W	YOR290C	976
YDR448W	YPL011C	994
YDR448W	YPL016W	972
YDR448W	YPL047W	999
YDR448W	YPL248C	986
YDR448W	YPL254W	999
YDR448W	YPR070W	994
YDR449C	YBL004W	999
YDR449C	YBL072C	980
YDR449C	YBR189W	929
YDR449C	YBR247C	999
YDR449C	YCL059C	999
YDR449C	YCR057C	999
YDR449C	YDL014W	997
YDR449C	YDL060W	910

YDR449C	YDL148C	998
YDR449C	YDL153C	911
YDR449C	YDR064W	933
YDR449C	YDR299W	948
YDR449C	YDR324C	999
YDR449C	YER082C	999
YDR449C	YGL171W	999
YDR449C	YGR090W	999
YDR449C	YGR128C	999
YDR449C	YGR145W	999
YDR449C	YHR148W	999
YDR449C	YHR169W	915
YDR449C	YHR196W	999
YDR449C	YJL010C	985
YDR449C	YJL069C	999
YDR449C	YJL109C	999
YDR449C	YJR002W	999
YDR449C	YJR145C	929
YDR449C	YKR060W	998
YDR449C	YLL011W	999
YDR449C	YLR003C	972
YDR449C	YLR129W	999
YDR449C	YLR186W	999
YDR449C	YLR197W	997
YDR449C	YLR222C	999
YDR449C	YLR409C	999
YDR449C	YML093W	911
YDR449C	YMR093W	999
YDR449C	YMR128W	997
YDR449C	YMR229C	972
YDR449C	YNL075W	997
YDR449C	YNL132W	999
YDR449C	YOL010W	919
YDR449C	YOR004W	910
YDR449C	YOR078W	972
YDR449C	YOR096W	933
YDR449C	YOR145C	997
YDR449C	YOR310C	987
YDR449C	YPL126W	999
YDR449C	YPL217C	998
YDR449C	YPL266W	997
YDR449C	YPR112C	930
YDR449C	YPR137W	999
YDR449C	YPR144C	999

YDR450W	YBL027W	921
YDR450W	YBL072C	933
YDR450W	YBL087C	927
YDR450W	YBL092W	944
YDR450W	YBR031W	925
YDR450W	YBR048W	999
YDR450W	YBR084C-A	921
YDR450W	YBR118W	907
YDR450W	YBR181C	907
YDR450W	YBR189W	998
YDR450W	YBR191W	921
YDR450W	YCR031C	999
YDR450W	YDL061C	990
YDR450W	YDL075W	920
YDR450W	YDL081C	938
YDR450W	YDL082W	920
YDR450W	YDL083C	966
YDR450W	YDL130W	902
YDR450W	YDL136W	912
YDR450W	YDL191W	912
YDR450W	YDR012W	925
YDR450W	YDR025W	921
YDR450W	YDR041W	912
YDR450W	YDR064W	995
YDR450W	YDR382W	924
YDR450W	YDR385W	990
YDR450W	YDR418W	913
YDR450W	YDR447C	996
YDR450W	YDR471W	903
YDR450W	YDR500C	901
YDR450W	YEL054C	913
YDR450W	YER056C-A	944
YDR450W	YER074W	997
YDR450W	YER102W	921
YDR450W	YER117W	927
YDR450W	YER131W	903
YDR450W	YER165W	901
YDR450W	YFL034C-A	912
YDR450W	YFR031C-A	924
YDR450W	YFR032C-A	934
YDR450W	YGL030W	912
YDR450W	YGL076C	920
YDR450W	YGL103W	913
YDR450W	YGL123W	995

YDR450W	YGL135W	949
YDR450W	YGL147C	998
YDR450W	YGL189C	903
YDR450W	YGR027C	915
YDR450W	YGR034W	998
YDR450W	YGR085C	996
YDR450W	YGR118W	992
YDR450W	YGR214W	994
YDR450W	YHL001W	921
YDR450W	YHL015W	993
YDR450W	YHL033C	922
YDR450W	YHR010W	903
YDR450W	YHR021C	918
YDR450W	YHR141C	926
YDR450W	YHR203C	924
YDR450W	YIL018W	996
YDR450W	YIL052C	902
YDR450W	YIL069C	997
YDR450W	YIL133C	997
YDR450W	YIL148W	906
YDR450W	YJL136C	923
YDR450W	YJL177W	995
YDR450W	YJL189W	908
YDR450W	YJL190C	991
YDR450W	YJL191W	979
YDR450W	YJR094W-A	920
YDR450W	YJR123W	999
YDR450W	YJR145C	924
YDR450W	YKL006W	920
YDR450W	YKL156W	918
YDR450W	YKL170W	948
YDR450W	YKL180W	995
YDR450W	YKR057W	923
YDR450W	YKR094C	906
YDR450W	YLL045C	922
YDR450W	YLR029C	995
YDR450W	YLR048W	943
YDR450W	YLR061W	914
YDR450W	YLR075W	997
YDR450W	YLR167W	900
YDR450W	YLR185W	901
YDR450W	YLR192C	918
YDR450W	YLR264W	912
YDR450W	YLR287C-A	901

YDR450W	YLR333C	914
YDR450W	YLR340W	937
YDR450W	YLR344W	924
YDR450W	YLR367W	919
YDR450W	YLR388W	907
YDR450W	YLR406C	920
YDR450W	YLR441C	918
YDR450W	YLR448W	921
YDR450W	YML024W	919
YDR450W	YML026C	901
YDR450W	YML063W	995
YDR450W	YML073C	919
YDR450W	YMR121C	908
YDR450W	YMR142C	998
YDR450W	YMR146C	915
YDR450W	YMR194W	996
YDR450W	YMR230W	917
YDR450W	YMR242C	996
YDR450W	YMR260C	903
YDR450W	YNL067W	922
YDR450W	YNL069C	998
YDR450W	YNL081C	918
YDR450W	YNL096C	924
YDR450W	YNL162W	926
YDR450W	YNL178W	999
YDR450W	YNL301C	922
YDR450W	YNL302C	914
YDR450W	YNR037C	950
YDR450W	YOL039W	913
YDR450W	YOL040C	995
YDR450W	YOL120C	922
YDR450W	YOL121C	914
YDR450W	YOL127W	996
YDR450W	YOR063W	928
YDR450W	YOR096W	924
YDR450W	YOR133W	912
YDR450W	YOR167C	912
YDR450W	YOR182C	901
YDR450W	YOR204W	976
YDR450W	YOR234C	920
YDR450W	YOR293W	917
YDR450W	YOR312C	925
YDR450W	YOR369C	914
YDR450W	YPL079W	921

YDR450W	YPL081W	996
YDR450W	YPL090C	907
YDR450W	YPL131W	912
YDR450W	YPL143W	997
YDR450W	YPL198W	920
YDR450W	YPL220W	918
YDR450W	YPL237W	906
YDR450W	YPL249C-A	918
YDR450W	YPR043W	920
YDR450W	YPR080W	907
YDR450W	YPR102C	934
YDR450W	YPR132W	927
YDR451C	YMR043W	920
YDR452W	YGR159C	998
YDR452W	YHR201C	985
YDR453C	YKL086W	911
YDR453C	YML028W	980
YDR454C	YAL038W	901
YDR454C	YDR399W	976
YDR454C	YDR441C	919
YDR454C	YGL248W	906
YDR454C	YKL067W	906
YDR454C	YML013W	960
YDR454C	YML022W	933
YDR454C	YMR217W	919
YDR454C	YOR347C	900
YDR456W	YDR040C	954
YDR456W	YJL044C	979
YDR456W	YJL094C	944
YDR456W	YLR138W	977
YDR456W	YNL321W	926
YDR457W	YDL229W	954
YDR457W	YGR285C	908
YDR457W	YHR064C	979
YDR457W	YLL039C	972
YDR457W	YML074C	976
YDR458C	YNL280C	998
YDR460W	YAR007C	977
YDR460W	YBR160W	919
YDR460W	YDL108W	999
YDR460W	YDR311W	997
YDR460W	YER162C	978
YDR460W	YER171W	999
YDR460W	YGL127C	946

YDR460W	YGR258C	981
YDR460W	YIL143C	995
YDR460W	YLR005W	999
YDR460W	YML095C	980
YDR460W	YMR201C	981
YDR460W	YNL312W	978
YDR460W	YPL022W	980
YDR460W	YPL122C	995
YDR460W	YPR025C	999
YDR460W	YPR056W	999
YDR460W	YPR119W	918
YDR461W	YDL090C	932
YDR461W	YPR122W	906
YDR462W	YBL038W	997
YDR462W	YBR122C	972
YDR462W	YCR046C	992
YDR462W	YCR071C	972
YDR462W	YDR116C	972
YDR462W	YDR237W	999
YDR462W	YDR296W	992
YDR462W	YDR322W	999
YDR462W	YGR220C	997
YDR462W	YJL063C	972
YDR462W	YKL167C	972
YDR462W	YKR006C	972
YDR462W	YKR085C	972
YDR462W	YLR439W	972
YDR462W	YML025C	972
YDR462W	YMR024W	972
YDR462W	YNL005C	989
YDR462W	YNL252C	988
YDR462W	YNL284C	999
YDR462W	YOR150W	970
YDR463W	YJL156C	957
YDR464W	YDR473C	980
YDR464W	YHR165C	903
YDR465C	YGL172W	916
YDR465C	YGR119C	923
YDR465C	YKL068W	918
YDR468C	YAL030W	998
YDR468C	YBL050W	967
YDR468C	YDR027C	905
YDR468C	YDR484W	907
YDR468C	YGL095C	999

YDR468C	YJL029C	913
YDR468C	YKR020W	999
YDR468C	YLR026C	999
YDR468C	YLR262C	982
YDR468C	YMR197C	999
YDR468C	YOL018C	999
YDR468C	YOR036W	998
YDR468C	YOR327C	999
YDR469W	YAR003W	999
YDR469W	YBR175W	935
YDR469W	YBR258C	999
YDR469W	YHR119W	999
YDR469W	YJL168C	952
YDR469W	YKL018W	986
YDR469W	YLR015W	999
YDR469W	YOL012C	958
YDR469W	YPL138C	999
YDR470C	YBR179C	999
YDR470C	YLL001W	940
YDR470C	YOR211C	999
YDR471W	YAL035W	916
YDR471W	YBL027W	995
YDR471W	YBL072C	993
YDR471W	YBL087C	901
YDR471W	YBL092W	998
YDR471W	YBR031W	997
YDR471W	YBR048W	996
YDR471W	YBR084C-A	938
YDR471W	YBR084W	972
YDR471W	YBR181C	995
YDR471W	YBR189W	999
YDR471W	YBR191W	999
YDR471W	YCR031C	998
YDR471W	YDL061C	969
YDR471W	YDL075W	998
YDR471W	YDL081C	910
YDR471W	YDL082W	996
YDR471W	YDL083C	960
YDR471W	YDL130W	927
YDR471W	YDL136W	997
YDR471W	YDL191W	908
YDR471W	YDR012W	998
YDR471W	YDR025W	996
YDR471W	YDR064W	999

YDR471W	YDR382W	999
YDR471W	YDR418W	999
YDR471W	YDR447C	995
YDR471W	YDR450W	903
YDR471W	YDR496C	933
YDR471W	YDR500C	944
YDR471W	YEL054C	917
YDR471W	YER006W	933
YDR471W	YER056C-A	978
YDR471W	YER074W	995
YDR471W	YER102W	941
YDR471W	YER117W	943
YDR471W	YER131W	967
YDR471W	YFL034C-A	911
YDR471W	YFR031C-A	995
YDR471W	YFR032C-A	995
YDR471W	YGL014W	956
YDR471W	YGL030W	999
YDR471W	YGL031C	998
YDR471W	YGL076C	997
YDR471W	YGL103W	999
YDR471W	YGL123W	966
YDR471W	YGL135W	996
YDR471W	YGL147C	996
YDR471W	YGL189C	911
YDR471W	YGR027C	988
YDR471W	YGR034W	995
YDR471W	YGR085C	998
YDR471W	YGR118W	996
YDR471W	YGR148C	991
YDR471W	YGR214W	997
YDR471W	YHL001W	999
YDR471W	YHL015W	999
YDR471W	YHL033C	999
YDR471W	YHR010W	973
YDR471W	YHR021C	905
YDR471W	YHR141C	996
YDR471W	YHR203C	998
YDR471W	YIL018W	995
YDR471W	YIL052C	996
YDR471W	YIL069C	996
YDR471W	YIL133C	996
YDR471W	YIL148W	948
YDR471W	YJL136C	943

YDR471W	YJL177W	999
YDR471W	YJL189W	931
YDR471W	YJL190C	996
YDR471W	YJL191W	963
YDR471W	YJR094W-A	956
YDR471W	YJR123W	999
YDR471W	YJR145C	994
YDR471W	YKL006W	999
YDR471W	YKL156W	974
YDR471W	YKL180W	999
YDR471W	YKR057W	912
YDR471W	YKR081C	937
YDR471W	YLL045C	997
YDR471W	YLR029C	998
YDR471W	YLR048W	977
YDR471W	YLR061W	991
YDR471W	YLR075W	999
YDR471W	YLR150W	956
YDR471W	YLR167W	939
YDR471W	YLR185W	946
YDR471W	YLR264W	972
YDR471W	YLR287C-A	995
YDR471W	YLR333C	919
YDR471W	YLR340W	949
YDR471W	YLR344W	999
YDR471W	YLR367W	967
YDR471W	YLR388W	987
YDR471W	YLR406C	997
YDR471W	YLR441C	995
YDR471W	YLR448W	999
YDR471W	YML024W	977
YDR471W	YML026C	903
YDR471W	YML063W	999
YDR471W	YML073C	995
YDR471W	YMR121C	912
YDR471W	YMR142C	996
YDR471W	YMR194W	998
YDR471W	YMR230W	943
YDR471W	YMR242C	996
YDR471W	YMR290C	946
YDR471W	YNL061W	933
YDR471W	YNL067W	993
YDR471W	YNL069C	996
YDR471W	YNL096C	996

YDR471W	YNL110C	975
YDR471W	YNL112W	976
YDR471W	YNL162W	920
YDR471W	YNL178W	999
YDR471W	YNL301C	997
YDR471W	YNL302C	907
YDR471W	YOL039W	916
YDR471W	YOL040C	995
YDR471W	YOL077C	943
YDR471W	YOL121C	907
YDR471W	YOL127W	999
YDR471W	YOR063W	998
YDR471W	YOR096W	999
YDR471W	YOR133W	913
YDR471W	YOR167C	940
YDR471W	YOR182C	910
YDR471W	YOR234C	987
YDR471W	YOR293W	990
YDR471W	YOR312C	996
YDR471W	YOR369C	915
YDR471W	YPL079W	961
YDR471W	YPL081W	955
YDR471W	YPL090C	995
YDR471W	YPL093W	936
YDR471W	YPL131W	963
YDR471W	YPL143W	973
YDR471W	YPL198W	996
YDR471W	YPL211W	938
YDR471W	YPL220W	997
YDR471W	YPL249C-A	997
YDR471W	YPR043W	921
YDR471W	YPR102C	993
YDR471W	YPR132W	950
YDR472W	YBR254C	999
YDR472W	YDR108W	999
YDR472W	YDR246W	999
YDR472W	YDR407C	999
YDR472W	YFL038C	933
YDR472W	YGR166W	999
YDR472W	YKR068C	999
YDR472W	YML077W	999
YDR472W	YMR218C	999
YDR472W	YOR115C	999
YDR473C	YBL026W	993

YDR473C	YBR055C	999
YDR473C	YBR152W	999
YDR473C	YCR077C	974
YDR473C	YDL030W	981
YDR473C	YDL098C	993
YDR473C	YDR378C	999
YDR473C	YDR464W	980
YDR473C	YER029C	996
YDR473C	YER112W	996
YDR473C	YER146W	972
YDR473C	YER172C	999
YDR473C	YFL017W-A	961
YDR473C	YGR074W	997
YDR473C	YGR075C	995
YDR473C	YGR091W	999
YDR473C	YHR165C	999
YDR473C	YJL124C	979
YDR473C	YJL203W	980
YDR473C	YJR022W	931
YDR473C	YKL173W	999
YDR473C	YLR147C	999
YDR473C	YLR275W	997
YDR473C	YLR305C	940
YDR473C	YLR438C-A	999
YDR473C	YML049C	978
YDR473C	YMR240C	974
YDR473C	YMR288W	976
YDR473C	YNL147W	974
YDR473C	YOR159C	987
YDR473C	YOR308C	997
YDR473C	YPR082C	994
YDR473C	YPR178W	999
YDR477W	YBL093C	977
YDR477W	YBR010W	976
YDR477W	YBR066C	976
YDR477W	YBR112C	994
YDR477W	YCR084C	993
YDR477W	YDL047W	943
YDR477W	YDR028C	999
YDR477W	YDR043C	996
YDR477W	YDR099W	973
YDR477W	YDR216W	999
YDR477W	YDR335W	947
YDR477W	YDR422C	999

YDR477W	YDR443C	971
YDR477W	YDR507C	907
YDR477W	YER027C	999
YDR477W	YER040W	933
YDR477W	YER129W	999
YDR477W	YER133W	990
YDR477W	YER177W	990
YDR477W	YGL035C	999
YDR477W	YGL073W	987
YDR477W	YGL115W	999
YDR477W	YGL208W	999
YDR477W	YGL253W	995
YDR477W	YGR252W	918
YDR477W	YHR135C	970
YDR477W	YIL162W	945
YDR477W	YIR019C	934
YDR477W	YJL089W	999
YDR477W	YJL153C	921
YDR477W	YKL048C	983
YDR477W	YKL101W	908
YDR477W	YKL139W	900
YDR477W	YLL039C	935
YDR477W	YLR258W	961
YDR477W	YML006C	998
YDR477W	YMR037C	992
YDR477W	YMR140W	988
YDR477W	YMR280C	998
YDR477W	YNL025C	984
YDR477W	YNL236W	971
YDR477W	YNL257C	976
YDR477W	YNR016C	995
YDR477W	YOL136C	976
YDR477W	YOR047C	999
YDR477W	YOR178C	900
YDR477W	YOR360C	995
YDR477W	YPL031C	997
YDR477W	YPL042C	995
YDR477W	YPL248C	937
YDR477W	YPR185W	903
YDR478W	YAL033W	952
YDR478W	YBR167C	991
YDR478W	YBR257W	999
YDR478W	YGR030C	991
YDR478W	YHR062C	999

YDR478W	YIR015W	952
YDR478W	YLR145W	955
YDR478W	YNL221C	999
YDR478W	YNL282W	952
YDR478W	YPR119W	956
YDR479C	YKR001C	911
YDR479C	YLR324W	956
YDR479C	YPL112C	931
YDR480W	YBL016W	999
YDR480W	YGR040W	999
YDR480W	YHR084W	999
YDR480W	YLR304C	923
YDR480W	YNR026C	925
YDR480W	YPL049C	999
YDR481C	YFR034C	931
YDR482C	YPL213W	906
YDR483W	YDR068W	958
YDR484W	YCR094W	927
YDR484W	YDR027C	999
YDR484W	YDR468C	907
YDR484W	YFL039C	991
YDR484W	YJL029C	999
YDR484W	YKR020W	999
YDR484W	YLR262C	995
YDR485C	YAL011W	999
YDR485C	YBR231C	999
YDR485C	YDL074C	967
YDR485C	YDR190C	999
YDR485C	YDR334W	999
YDR485C	YDR392W	904
YDR485C	YEL003W	902
YDR485C	YEL018W	972
YDR485C	YER092W	917
YDR485C	YFL039C	961
YDR485C	YGL043W	972
YDR485C	YGL058W	961
YDR485C	YGL070C	902
YDR485C	YGL127C	986
YDR485C	YGL244W	906
YDR485C	YGR002C	994
YDR485C	YJL030W	916
YDR485C	YJL081C	998
YDR485C	YLR015W	907
YDR485C	YLR055C	927

YDR485C	YLR085C	999
YDR485C	YLR385C	994
YDR485C	YLR418C	946
YDR485C	YML041C	998
YDR485C	YML094W	963
YDR485C	YNL107W	999
YDR485C	YNL136W	915
YDR485C	YOL012C	999
YDR485C	YOR026W	902
YDR485C	YOR123C	909
YDR485C	YOR349W	947
YDR485C	YPL055C	968
YDR485C	YPL235W	999
YDR485C	YPL241C	952
YDR486C	YKL041W	999
YDR486C	YKR035W-A	981
YDR486C	YLR025W	999
YDR486C	YLR181C	999
YDR486C	YNL265C	906
YDR487C	YBL033C	995
YDR487C	YBR256C	968
YDR487C	YOL143C	998
YDR488C	YDR150W	985
YDR488C	YDR424C	948
YDR488C	YEL061C	923
YDR488C	YER016W	968
YDR488C	YKR054C	954
YDR488C	YPL174C	957
YDR488C	YPL269W	982
YDR489W	YBL023C	980
YDR489W	YBR202W	980
YDR489W	YDR013W	999
YDR489W	YEL032W	980
YDR489W	YGL113W	963
YDR489W	YGL201C	980
YDR489W	YJL072C	999
YDR489W	YJL090C	990
YDR489W	YLR103C	993
YDR489W	YLR274W	992
YDR489W	YOL146W	999
YDR489W	YPR019W	980
YDR489W	YPR135W	930
YDR490C	YGR086C	999
YDR490C	YHR205W	946

YDR490C	YKL126W	999
YDR490C	YMR104C	987
YDR490C	YOL100W	999
YDR490C	YPL004C	999
YDR490C	YPL240C	919
YDR492W	YJL056C	931
YDR494W	Q0275	912
YDR494W	YBL013W	939
YDR494W	YGL107C	941
YDR494W	YOL023W	937
YDR495C	YAL002W	993
YDR495C	YBL102W	932
YDR495C	YLR148W	991
YDR495C	YLR396C	986
YDR495C	YMR231W	992
YDR495C	YPL045W	987
YDR496C	YAL035W	999
YDR496C	YBR031W	933
YDR496C	YBR142W	999
YDR496C	YDL014W	970
YDR496C	YDL031W	992
YDR496C	YDR012W	939
YDR496C	YDR060W	972
YDR496C	YDR087C	979
YDR496C	YDR471W	933
YDR496C	YER006W	979
YDR496C	YFL002C	994
YDR496C	YGL030W	933
YDR496C	YGL076C	931
YDR496C	YGL111W	994
YDR496C	YGL173C	983
YDR496C	YHR052W	996
YDR496C	YHR066W	993
YDR496C	YHR088W	995
YDR496C	YHR089C	947
YDR496C	YHR170W	904
YDR496C	YIL018W	933
YDR496C	YIL133C	933
YDR496C	YJR145C	933
YDR496C	YKL009W	966
YDR496C	YKL014C	988
YDR496C	YKL130C	916
YDR496C	YKR081C	996
YDR496C	YLL008W	995

YDR496C	YLL034C	989
YDR496C	YLL045C	945
YDR496C	YLR002C	981
YDR496C	YLR029C	935
YDR496C	YLR175W	997
YDR496C	YLR197W	950
YDR496C	YLR276C	991
YDR496C	YLR449W	991
YDR496C	YML073C	935
YDR496C	YMR049C	991
YDR496C	YMR229C	984
YDR496C	YMR242C	944
YDR496C	YMR290C	983
YDR496C	YNL002C	987
YDR496C	YNL061W	999
YDR496C	YNL110C	976
YDR496C	YOL077C	989
YDR496C	YOL127W	946
YDR496C	YOR063W	948
YDR496C	YOR206W	983
YDR496C	YOR272W	993
YDR496C	YPL012W	939
YDR496C	YPL043W	999
YDR496C	YPL093W	990
YDR496C	YPL131W	967
YDR496C	YPL198W	969
YDR496C	YPL211W	993
YDR496C	YPL220W	939
YDR496C	YPR016C	998
YDR498C	YGL098W	999
YDR498C	YGL145W	999
YDR498C	YLR268W	999
YDR498C	YLR440C	999
YDR498C	YNL258C	999
YDR498C	YOR075W	999
YDR499W	YAR007C	997
YDR499W	YBL088C	993
YDR499W	YBR136W	999
YDR499W	YBR274W	973
YDR499W	YDR097C	912
YDR499W	YML058W	999
YDR499W	YPL153C	973
YDR500C	YAL035W	914
YDR500C	YBL027W	902

YDR500C	YBL072C	911
YDR500C	YBL087C	909
YDR500C	YBL092W	908
YDR500C	YBR031W	932
YDR500C	YBR048W	965
YDR500C	YBR084C-A	999
YDR500C	YBR118W	916
YDR500C	YBR189W	953
YDR500C	YBR191W	984
YDR500C	YCR031C	952
YDR500C	YDL061C	978
YDR500C	YDL075W	996
YDR500C	YDL081C	908
YDR500C	YDL082W	907
YDR500C	YDL130W	936
YDR500C	YDL136W	999
YDR500C	YDL191W	918
YDR500C	YDR012W	946
YDR500C	YDR025W	900
YDR500C	YDR064W	985
YDR500C	YDR382W	977
YDR500C	YDR418W	963
YDR500C	YDR447C	916
YDR500C	YDR450W	901
YDR500C	YDR471W	944
YDR500C	YEL054C	921
YDR500C	YER056C-A	996
YDR500C	YER074W	917
YDR500C	YER102W	999
YDR500C	YER117W	999
YDR500C	YER131W	931
YDR500C	YFL034C-A	911
YDR500C	YFR032C-A	926
YDR500C	YGL030W	985
YDR500C	YGL076C	916
YDR500C	YGL103W	964
YDR500C	YGL123W	967
YDR500C	YGL135W	912
YDR500C	YGL147C	904
YDR500C	YGL189C	917
YDR500C	YGR027C	939
YDR500C	YGR034W	913
YDR500C	YGR085C	920
YDR500C	YGR118W	972

YDR500C	YGR148C	932
YDR500C	YGR214W	939
YDR500C	YHL001W	958
YDR500C	YHL015W	947
YDR500C	YHL033C	972
YDR500C	YHR010W	939
YDR500C	YHR021C	916
YDR500C	YHR026W	937
YDR500C	YHR141C	917
YDR500C	YHR203C	999
YDR500C	YIL052C	996
YDR500C	YIL069C	917
YDR500C	YIL133C	913
YDR500C	YIL148W	999
YDR500C	YJL136C	936
YDR500C	YJL177W	965
YDR500C	YJL189W	929
YDR500C	YJL190C	909
YDR500C	YJL191W	961
YDR500C	YJR094W-A	994
YDR500C	YJR123W	966
YDR500C	YJR145C	912
YDR500C	YKL006W	967
YDR500C	YKL156W	973
YDR500C	YKL180W	976
YDR500C	YKR057W	921
YDR500C	YKR059W	998
YDR500C	YKR094C	900
YDR500C	YLL045C	912
YDR500C	YLR029C	936
YDR500C	YLR048W	971
YDR500C	YLR061W	995
YDR500C	YLR075W	989
YDR500C	YLR167W	999
YDR500C	YLR185W	999
YDR500C	YLR264W	963
YDR500C	YLR287C-A	906
YDR500C	YLR325C	917
YDR500C	YLR333C	910
YDR500C	YLR340W	972
YDR500C	YLR344W	986
YDR500C	YLR367W	931
YDR500C	YLR388W	997
YDR500C	YLR406C	910

YDR500C	YLR441C	911
YDR500C	YLR448W	971
YDR500C	YML024W	996
YDR500C	YML026C	901
YDR500C	YML063W	944
YDR500C	YML073C	912
YDR500C	YMR121C	913
YDR500C	YMR142C	906
YDR500C	YMR194W	994
YDR500C	YMR230W	928
YDR500C	YMR242C	909
YDR500C	YNL067W	936
YDR500C	YNL069C	913
YDR500C	YNL096C	920
YDR500C	YNL162W	917
YDR500C	YNL178W	975
YDR500C	YNL301C	999
YDR500C	YNL302C	911
YDR500C	YOL040C	923
YDR500C	YOL121C	911
YDR500C	YOL127W	978
YDR500C	YOR063W	948
YDR500C	YOR096W	961
YDR500C	YOR133W	960
YDR500C	YOR167C	912
YDR500C	YOR182C	906
YDR500C	YOR234C	954
YDR500C	YOR293W	936
YDR500C	YOR312C	944
YDR500C	YOR369C	929
YDR500C	YPL037C	963
YDR500C	YPL079W	923
YDR500C	YPL081W	924
YDR500C	YPL131W	970
YDR500C	YPL143W	967
YDR500C	YPL198W	916
YDR500C	YPL220W	999
YDR500C	YPL249C-A	956
YDR500C	YPR043W	996
YDR500C	YPR102C	927
YDR500C	YPR132W	999
YDR502C	YBR236C	983
YDR502C	YER091C	938
YDR502C	YGR264C	907

YDR502C	YLR180W	999
YDR502C	YLR186W	918
YDR503C	YER113C	927
YDR505C	YNL102W	925
YDR507C	YAL027W	994
YDR507C	YBR160W	947
YDR507C	YBR267W	948
YDR507C	YCL024W	999
YDR507C	YCR002C	998
YDR507C	YDL225W	999
YDR507C	YDR477W	907
YDR507C	YHR107C	999
YDR507C	YJL187C	999
YDR507C	YJR076C	998
YDR507C	YKL048C	985
YDR507C	YKL101W	987
YDR507C	YKR048C	999
YDR507C	YLR229C	997
YDR507C	YLR314C	999
YDR507C	YNL078W	917
YDR507C	YNL298W	979
YDR507C	YPR119W	912
YDR508C	YDL212W	954
YDR508C	YPR025C	998
YDR510W	YBR088C	996
YDR510W	YCR087C-A	914
YDR510W	YDL013W	999
YDR510W	YDL055C	921
YDR510W	YDL064W	999
YDR510W	YDL084W	920
YDR510W	YDL225W	985
YDR510W	YDR148C	901
YDR510W	YDR390C	999
YDR510W	YDR409W	998
YDR510W	YER032W	926
YDR510W	YER047C	942
YDR510W	YER116C	994
YDR510W	YFL039C	977
YDR510W	YGL026C	921
YDR510W	YGL250W	942
YDR510W	YHR134W	984
YDR510W	YIL031W	981
YDR510W	YJL092W	996
YDR510W	YJR076C	990

YDR510W	YKL032C	942
YDR510W	YLR314C	999
YDR510W	YML032C	902
YDR510W	YMR111C	942
YDR510W	YMR186W	939
YDR510W	YNL042W	921
YDR510W	YNL078W	945
YDR510W	YNL088W	997
YDR510W	YOL034W	942
YDR510W	YOR156C	999
YDR510W	YOR191W	949
YDR510W	YPL020C	999
YDR510W	YPL082C	935
YDR510W	YPR180W	998
YDR513W	YCL035C	984
YDR513W	YGR209C	914
YDR513W	YJL026W	902
YDR513W	YPL091W	930
YDR516C	YCL040W	964
YDR516C	YFR053C	923
YDR516C	YNR050C	925
YDR517W	YDL058W	943
YDR517W	YDL099W	999
YDR517W	YHR098C	984
YDR517W	YIL109C	999
YDR517W	YNL049C	989
YDR517W	YPR181C	999
YDR518W	YCL043C	978
YDR518W	YIL005W	951
YDR524C	YDL192W	984
YDR529C	Q0105	999
YDR529C	Q0120	999
YDR529C	YBL045C	999
YDR529C	YDR375C	921
YDR529C	YEL024W	999
YDR529C	YEL039C	908
YDR529C	YFR033C	999
YDR529C	YGR183C	999
YDR529C	YHR001W-A	994
YDR529C	YJL166W	999
YDR529C	YJR048W	990
YDR529C	YOR065W	999
YDR529C	YPR191W	999
YDR530C	YCL050C	902

YDR532C	YGL093W	926
YDR532C	YHR079C	904
YEL002C	YAL058W	907
YEL002C	YBL105C	909
YEL002C	YDL145C	972
YEL002C	YDL232W	999
YEL002C	YDR086C	972
YEL002C	YER087C-B	995
YEL002C	YGL022W	999
YEL002C	YGL027C	936
YEL002C	YGL226C-A	999
YEL002C	YGR227W	990
YEL002C	YJL002C	999
YEL002C	YLR378C	981
YEL002C	YML019W	999
YEL002C	YMR149W	999
YEL002C	YNL219C	965
YEL002C	YOR002W	961
YEL002C	YOR085W	999
YEL002C	YOR103C	999
YEL002C	YPL227C	991
YEL003W	YBL034C	951
YEL003W	YDR334W	917
YEL003W	YDR485C	902
YEL003W	YER016W	929
YEL003W	YFL037W	960
YEL003W	YFL039C	963
YEL003W	YGL086W	930
YEL003W	YGR078C	999
YEL003W	YHR191C	948
YEL003W	YIL142W	982
YEL003W	YJL013C	915
YEL003W	YJL014W	982
YEL003W	YJL030W	931
YEL003W	YJL179W	999
YEL003W	YLR200W	999
YEL003W	YLR212C	901
YEL003W	YML041C	917
YEL003W	YML085C	982
YEL003W	YML094W	999
YEL003W	YML124C	960
YEL003W	YNL153C	999
YEL003W	YOL012C	928
YEL003W	YOR265W	913

YEL003W	YPL240C	919
YEL005C	YEL013W	996
YEL009C	YBR081C	976
YEL009C	YBR198C	995
YEL009C	YBR289W	999
YEL009C	YCL018W	909
YEL009C	YCL030C	965
YEL009C	YDL097C	900
YEL009C	YDR035W	925
YEL009C	YDR145W	912
YEL009C	YDR176W	913
YEL009C	YDR283C	999
YEL009C	YDR308C	999
YEL009C	YDR392W	961
YEL009C	YDR448W	999
YEL009C	YER022W	973
YEL009C	YER040W	987
YEL009C	YFL009W	976
YEL009C	YGL112C	975
YEL009C	YGL195W	972
YEL009C	YGR252W	974
YEL009C	YHR041C	997
YEL009C	YHR099W	975
YEL009C	YHR200W	978
YEL009C	YKR026C	960
YEL009C	YMR236W	995
YEL009C	YNR023W	988
YEL009C	YOL051W	991
YEL009C	YOR202W	970
YEL009C	YOR260W	990
YEL009C	YOR290C	999
YEL009C	YOR298C-A	910
YEL009C	YOR375C	928
YEL009C	YPL016W	996
YEL009C	YPL031C	984
YEL009C	YPL042C	995
YEL011W	YFR015C	996
YEL011W	YJL137C	989
YEL011W	YKR058W	989
YEL011W	YLR258W	997
YEL011W	YPR160W	991
YEL011W	YPR184W	926
YEL013W	YCL063W	999
YEL013W	YEL005C	996

YEL013W	YER123W	928
YEL013W	YFL039C	912
YEL013W	YHR195W	998
YEL013W	YKL196C	992
YEL013W	YKR007W	951
YEL013W	YMR015C	923
YEL013W	YNL326C	992
YEL013W	YOR106W	904
YEL013W	YPR149W	998
YEL013W	YPR185W	999
YEL015W	YBR094W	999
YEL015W	YDL160C	999
YEL015W	YGL173C	980
YEL015W	YHR121W	931
YEL015W	YJL124C	955
YEL015W	YJR022W	930
YEL015W	YNL118C	999
YEL015W	YOL149W	999
YEL015W	YOR167C	959
YEL015W	YPR129W	958
YEL016C	YCR026C	906
YEL018W	YAL011W	972
YEL018W	YBR231C	973
YEL018W	YDR334W	985
YEL018W	YDR359C	999
YEL018W	YDR485C	972
YEL018W	YFL024C	999
YEL018W	YFL039C	999
YEL018W	YGR002C	999
YEL018W	YHR090C	999
YEL018W	YHR099W	998
YEL018W	YJL081C	999
YEL018W	YJR082C	999
YEL018W	YLR085C	969
YEL018W	YML041C	973
YEL018W	YNL107W	999
YEL018W	YNL136W	999
YEL018W	YOL012C	991
YEL018W	YOR244W	999
YEL018W	YPR023C	999
YEL019C	YDR409W	954
YEL019C	YLR383W	938
YEL019C	YOL034W	996
YEL019C	YOR156C	989

YEL020C	YBR222C	999
YEL020C	YMR108W	916
YEL020W-A	YBR081C	975
YEL020W-A	YBR091C	997
YEL020W-A	YDL217C	979
YEL020W-A	YGR181W	997
YEL020W-A	YHR005C-A	999
YEL020W-A	YJR135W-A	997
YEL020W-A	YKL195W	998
YEL020W-A	YNR017W	967
YEL020W-A	YOR297C	932
YEL021W	YHR128W	996
YEL021W	YKL024C	912
YEL021W	YKL216W	994
YEL021W	YLR014C	986
YEL021W	YLR420W	928
YEL021W	YML106W	989
YEL021W	YMR271C	965
YEL021W	YNR012W	958
YEL022W	YAL026C	987
YEL022W	YBR017C	966
YEL022W	YDL137W	992
YEL022W	YDL192W	999
YEL022W	YER019C-A	923
YEL022W	YER110C	972
YEL022W	YGR218W	968
YEL022W	YHR020W	968
YEL022W	YJR031C	996
YEL022W	YJR077C	999
YEL022W	YKR030W	933
YEL022W	YMR012W	960
YEL022W	YNL287W	985
YEL022W	YOR094W	989
YEL024W	Q0105	999
YEL024W	Q0120	999
YEL024W	YBL045C	999
YEL024W	YDL207W	976
YEL024W	YDR375C	936
YEL024W	YDR529C	999
YEL024W	YEL039C	936
YEL024W	YER107C	968
YEL024W	YFR033C	999
YEL024W	YGR183C	999
YEL024W	YHR001W-A	995

YEL024W	YJL166W	999
YEL024W	YJR048W	994
YEL024W	YOR065W	999
YEL024W	YPR191W	999
YEL026W	YBL026W	947
YEL026W	YBR154C	936
YEL026W	YDL014W	999
YEL026W	YDL083C	966
YEL026W	YDL140C	910
YEL026W	YDR339C	914
YEL026W	YDR404C	907
YEL026W	YER029C	936
YEL026W	YER172C	999
YEL026W	YFL017W-A	943
YEL026W	YGL030W	952
YEL026W	YGL070C	912
YEL026W	YGL120C	966
YEL026W	YGR074W	944
YEL026W	YHR143W-A	921
YEL026W	YHR148W	908
YEL026W	YHR165C	921
YEL026W	YIL021W	900
YEL026W	YJL010C	906
YEL026W	YJR002W	984
YEL026W	YKL180W	952
YEL026W	YLR147C	991
YEL026W	YLR175W	980
YEL026W	YLR186W	935
YEL026W	YLR197W	999
YEL026W	YLR222C	911
YEL026W	YLR275W	915
YEL026W	YMR229C	919
YEL026W	YMR288W	901
YEL026W	YMR290C	934
YEL026W	YOL005C	904
YEL026W	YOR145C	943
YEL026W	YOR159C	909
YEL026W	YOR207C	914
YEL026W	YOR210W	957
YEL026W	YOR224C	931
YEL026W	YOR310C	999
YEL026W	YPL012W	909
YEL026W	YPL217C	962
YEL026W	YPL266W	917

YEL026W	YPR082C	949
YEL026W	YPR137W	949
YEL026W	YPR182W	939
YEL026W	YPR187W	936
YEL027W	YBR011C	961
YEL027W	YBR036C	947
YEL027W	YBR127C	980
YEL027W	YDL185W	994
YEL027W	YDL192W	964
YEL027W	YEL051W	928
YEL027W	YGR020C	923
YEL027W	YGR105W	976
YEL027W	YHR026W	998
YEL027W	YHR039C-A	919
YEL027W	YKL080W	920
YEL027W	YKL190W	957
YEL027W	YLR447C	946
YEL027W	YMR054W	901
YEL027W	YOR270C	999
YEL027W	YOR332W	946
YEL027W	YPL037C	964
YEL027W	YPL234C	994
YEL027W	YPR036W	970
YEL029C	YBR035C	947
YEL030W	YNR017W	906
YEL030W	YOR232W	999
YEL031W	YGL020C	954
YEL031W	YGL167C	986
YEL031W	YGR229C	919
YEL031W	YJL062W	943
YEL031W	YLR039C	964
YEL031W	YPL069C	908
YEL032W	YAR007C	915
YEL032W	YBL023C	999
YEL032W	YBR060C	964
YEL032W	YBR160W	991
YEL032W	YBR202W	999
YEL032W	YDL017W	999
YEL032W	YDR052C	981
YEL032W	YDR489W	980
YEL032W	YGL201C	990
YEL032W	YIL150C	989
YEL032W	YIR008C	907
YEL032W	YJL194W	993

YEL032W	YJR046W	983
YEL032W	YLR103C	999
YEL032W	YLR274W	999
YEL032W	YML065W	990
YEL032W	YNL102W	946
YEL032W	YNL312W	901
YEL032W	YPR019W	999
YEL032W	YPR119W	923
YEL032W	YPR162C	903
YEL034W	YAL003W	978
YEL034W	YBL105C	979
YEL034W	YER165W	914
YEL034W	YHL033C	921
YEL034W	YHR068W	998
YEL034W	YJR047C	993
YEL034W	YJR070C	988
YEL034W	YLR340W	953
YEL034W	YNL283C	981
YEL034W	YOL105C	982
YEL034W	YOR008C	978
YEL036C	YBR106W	990
YEL036C	YCR043C	907
YEL036C	YDR245W	999
YEL036C	YER001W	929
YEL036C	YFL048C	907
YEL036C	YGR132C	995
YEL036C	YGR231C	990
YEL036C	YJL183W	999
YEL036C	YJR075W	999
YEL036C	YLR026C	987
YEL036C	YLR268W	933
YEL036C	YLR342W	975
YEL036C	YML115C	970
YEL036C	YPL050C	999
YEL036C	YPL094C	963
YEL037C	YAR007C	901
YEL037C	YBR082C	980
YEL037C	YBR114W	998
YEL037C	YDL007W	991
YEL037C	YDL097C	982
YEL037C	YDL126C	963
YEL037C	YDL190C	999
YEL037C	YDR311W	984
YEL037C	YDR314C	945

YEL037C	YDR394W	995
YEL037C	YDR427W	998
YEL037C	YER012W	999
YEL037C	YER021W	983
YEL037C	YER143W	999
YEL037C	YER162C	999
YEL037C	YER171W	929
YEL037C	YFR052W	982
YEL037C	YGL048C	999
YEL037C	YGL058W	977
YEL037C	YGR258C	955
YEL037C	YHL030W	983
YEL037C	YHR027C	999
YEL037C	YHR200W	999
YEL037C	YIL075C	961
YEL037C	YIL143C	988
YEL037C	YJR052W	999
YEL037C	YKL145W	999
YEL037C	YLL039C	999
YEL037C	YLR079W	999
YEL037C	YML011C	982
YEL037C	YML095C	937
YEL037C	YMR201C	998
YEL037C	YMR276W	999
YEL037C	YNL312W	928
YEL037C	YOR117W	992
YEL037C	YOR257W	900
YEL037C	YOR259C	998
YEL037C	YOR261C	981
YEL037C	YPL022W	944
YEL037C	YPL096W	999
YEL037C	YPR025C	900
YEL037C	YPR032W	905
YEL038W	YJR024C	985
YEL038W	YMR009W	934
YEL039C	YDR529C	908
YEL039C	YEL024W	936
YEL039C	YJR048W	986
YEL039C	YOR065W	953
YEL040W	YGR189C	985
YEL041W	YJR049C	998
YEL041W	YPL188W	998
YEL042W	YER005W	992
YEL043W	YDR108W	921

YEL043W	YGL126W	919
YEL043W	YGR089W	953
YEL044W	YGL150C	940
YEL046C	YBR263W	999
YEL046C	YCL064C	903
YEL046C	YCR053W	932
YEL046C	YLR058C	999
YEL046C	YMR189W	900
YEL046C	YOR184W	901
YEL046C	YOR241W	920
YEL047C	YDL085W	907
YEL048C	YPL240C	919
YEL050C	YBL038W	953
YEL050C	YDR237W	915
YEL050C	YGL055W	901
YEL050C	YGR220C	981
YEL050C	YHR147C	954
YEL050C	YJL063C	955
YEL050C	YKL170W	907
YEL050C	YNL185C	904
YEL050C	YNL284C	921
YEL050C	YNR037C	924
YEL051W	YBR127C	999
YEL051W	YDL185W	999
YEL051W	YEL027W	928
YEL051W	YGR020C	999
YEL051W	YHR026W	951
YEL051W	YHR039C-A	999
YEL051W	YJR033C	933
YEL051W	YKL080W	999
YEL051W	YLR447C	998
YEL051W	YMR054W	999
YEL051W	YOR270C	999
YEL051W	YOR332W	999
YEL051W	YPL234C	940
YEL051W	YPR036W	999
YEL053C	YCR020C-A	999
YEL053C	YPL240C	919
YEL053C	YPR051W	999
YEL054C	YAL035W	922
YEL054C	YBL027W	910
YEL054C	YBL072C	915
YEL054C	YBL087C	921
YEL054C	YBL092W	936

YEL054C	YBR031W	938
YEL054C	YBR048W	926
YEL054C	YBR084C-A	910
YEL054C	YBR118W	907
YEL054C	YBR189W	923
YEL054C	YBR191W	919
YEL054C	YCR031C	931
YEL054C	YDL061C	922
YEL054C	YDL075W	924
YEL054C	YDL081C	927
YEL054C	YDL082W	997
YEL054C	YDL130W	916
YEL054C	YDL136W	935
YEL054C	YDL191W	935
YEL054C	YDR012W	938
YEL054C	YDR025W	926
YEL054C	YDR064W	953
YEL054C	YDR382W	932
YEL054C	YDR385W	901
YEL054C	YDR418W	986
YEL054C	YDR447C	930
YEL054C	YDR450W	913
YEL054C	YDR471W	917
YEL054C	YDR500C	921
YEL054C	YER056C-A	915
YEL054C	YER074W	924
YEL054C	YER102W	997
YEL054C	YER117W	921
YEL054C	YER131W	923
YEL054C	YFL034C-A	920
YEL054C	YFR031C-A	920
YEL054C	YFR032C-A	938
YEL054C	YGL030W	998
YEL054C	YGL031C	909
YEL054C	YGL076C	937
YEL054C	YGL123W	921
YEL054C	YGL135W	961
YEL054C	YGL147C	916
YEL054C	YGL189C	923
YEL054C	YGR027C	917
YEL054C	YGR034W	931
YEL054C	YGR085C	912
YEL054C	YGR118W	927
YEL054C	YGR148C	909

YEL054C	YGR214W	910
YEL054C	YHL001W	928
YEL054C	YHL015W	935
YEL054C	YHL033C	935
YEL054C	YHR010W	997
YEL054C	YHR021C	930
YEL054C	YHR141C	930
YEL054C	YHR203C	926
YEL054C	YIL018W	906
YEL054C	YIL052C	915
YEL054C	YIL069C	965
YEL054C	YIL133C	930
YEL054C	YIL148W	906
YEL054C	YJL136C	932
YEL054C	YJL177W	926
YEL054C	YJL189W	900
YEL054C	YJL190C	920
YEL054C	YJL191W	931
YEL054C	YJR094W-A	923
YEL054C	YJR123W	925
YEL054C	YJR145C	998
YEL054C	YKL006W	927
YEL054C	YKL156W	930
YEL054C	YKL180W	926
YEL054C	YKR057W	932
YEL054C	YKR094C	906
YEL054C	YLL045C	936
YEL054C	YLR029C	928
YEL054C	YLR048W	910
YEL054C	YLR061W	923
YEL054C	YLR075W	987
YEL054C	YLR167W	901
YEL054C	YLR185W	921
YEL054C	YLR264W	910
YEL054C	YLR287C-A	912
YEL054C	YLR325C	924
YEL054C	YLR333C	916
YEL054C	YLR340W	978
YEL054C	YLR344W	931
YEL054C	YLR367W	920
YEL054C	YLR388W	922
YEL054C	YLR406C	924
YEL054C	YLR441C	928
YEL054C	YLR448W	928

YEL054C	YML024W	930
YEL054C	YML026C	913
YEL054C	YML063W	998
YEL054C	YML073C	926
YEL054C	YMR121C	928
YEL054C	YMR142C	918
YEL054C	YMR194W	924
YEL054C	YMR230W	934
YEL054C	YMR242C	934
YEL054C	YMR260C	922
YEL054C	YNL067W	916
YEL054C	YNL069C	929
YEL054C	YNL096C	943
YEL054C	YNL162W	930
YEL054C	YNL178W	953
YEL054C	YNL301C	913
YEL054C	YNL302C	929
YEL054C	YOL040C	950
YEL054C	YOL120C	913
YEL054C	YOL121C	929
YEL054C	YOL127W	933
YEL054C	YOR063W	998
YEL054C	YOR096W	943
YEL054C	YOR133W	901
YEL054C	YOR167C	910
YEL054C	YOR182C	912
YEL054C	YOR234C	931
YEL054C	YOR293W	970
YEL054C	YOR312C	933
YEL054C	YOR369C	946
YEL054C	YPL079W	919
YEL054C	YPL081W	923
YEL054C	YPL131W	928
YEL054C	YPL143W	931
YEL054C	YPL198W	937
YEL054C	YPL220W	961
YEL054C	YPL249C-A	997
YEL054C	YPR043W	923
YEL054C	YPR080W	907
YEL054C	YPR102C	996
YEL054C	YPR132W	927
YEL055C	YDR324C	990
YEL055C	YDR398W	992
YEL055C	YGL043W	940

YEL055C	YGR128C	999
YEL055C	YHR196W	999
YEL055C	YJL109C	982
YEL055C	YLR196W	926
YEL055C	YMR093W	988
YEL055C	YPL126W	987
YEL056W	YBR009C	999
YEL056W	YBR010W	984
YEL056W	YBR060C	915
YEL056W	YBR272C	987
YEL056W	YHR118C	999
YEL056W	YLL004W	999
YEL056W	YLL022C	999
YEL056W	YML065W	999
YEL056W	YNL030W	999
YEL056W	YNL031C	999
YEL056W	YNL261W	999
YEL056W	YOL054W	994
YEL056W	YPL001W	999
YEL056W	YPR023C	916
YEL056W	YPR162C	999
YEL058W	YDL103C	995
YEL058W	YFL017C	994
YEL058W	YNL218W	999
YEL059C-A	YMR150C	990
YEL060C	YCL052C	908
YEL060C	YMR297W	965
YEL060C	YPL154C	983
YEL061C	YBL034C	933
YEL061C	YBL063W	999
YEL061C	YBR107C	939
YEL061C	YCL029C	992
YEL061C	YDL028C	979
YEL061C	YDR113C	983
YEL061C	YDR150W	975
YEL061C	YDR254W	939
YEL061C	YDR318W	990
YEL061C	YDR488C	923
YEL061C	YER007W	956
YEL061C	YER177W	943
YEL061C	YFL037W	945
YEL061C	YGL086W	997
YEL061C	YGL116W	988
YEL061C	YGL216W	965

YEL061C	YGR113W	995
YEL061C	YGR188C	984
YEL061C	YGR229C	995
YEL061C	YHR129C	901
YEL061C	YJL013C	981
YEL061C	YJL030W	999
YEL061C	YJR135C	939
YEL061C	YKR054C	999
YEL061C	YLL003W	976
YEL061C	YLL039C	967
YEL061C	YLR045C	942
YEL061C	YLR381W	939
YEL061C	YLR418C	932
YEL061C	YMR055C	996
YEL061C	YMR198W	951
YEL061C	YMR294W	972
YEL061C	YOL069W	962
YEL061C	YOR026W	939
YEL061C	YOR058C	999
YEL061C	YOR195W	998
YEL061C	YOR269W	909
YEL061C	YOR349W	987
YEL061C	YPL018W	989
YEL061C	YPL155C	992
YEL061C	YPL174C	974
YEL061C	YPL209C	997
YEL061C	YPL241C	976
YEL061C	YPR046W	966
YEL061C	YPR119W	979
YEL061C	YPR141C	999
YEL063C	YBR021W	973
YEL063C	YJL059W	957
YEL063C	YML052W	978
YEL063C	YPL240C	902
YEL064C	YPL240C	919
YEL065W	YDR077W	975
YEL065W	YMR058W	954
YEL066W	YPL070W	984
YEL068C	YKL002W	944
YEL071W	YCL050C	998
YEL075C	YEL076C	961
YEL075C	YER189W	944
YEL076C	YEL075C	961
YEL077C	YHL050C	929

YER001W	YEL036C	929
YER001W	YGL038C	992
YER002W	YOR294W	998
YER002W	YPR016C	958
YER003C	YFL045C	986
YER003C	YFR053C	906
YER003C	YGL253W	903
YER003C	YIL107C	900
YER003C	YJL155C	919
YER003C	YMR278W	951
YER004W	YJL148W	940
YER005W	YDL185W	900
YER005W	YEL042W	992
YER005W	YER044C	914
YER005W	YOR216C	923
YER005W	YPR036W	915
YER006W	YCL054W	998
YER006W	YCR072C	999
YER006W	YDL031W	999
YER006W	YDL060W	929
YER006W	YDL075W	931
YER006W	YDR060W	948
YER006W	YDR087C	976
YER006W	YDR101C	999
YER006W	YDR471W	933
YER006W	YDR496C	979
YER006W	YER126C	999
YER006W	YFL002C	993
YER006W	YFR001W	987
YER006W	YGL030W	942
YER006W	YGL111W	987
YER006W	YGL135W	931
YER006W	YGR085C	941
YER006W	YGR103W	999
YER006W	YGR245C	999
YER006W	YHR010W	983
YER006W	YHR052W	999
YER006W	YHR088W	993
YER006W	YHR197W	999
YER006W	YKL009W	998
YER006W	YKL172W	985
YER006W	YKR081C	999
YER006W	YLL008W	983
YER006W	YLL034C	983

YER006W	YLL045C	946
YER006W	YLR002C	999
YER006W	YLR074C	978
YER006W	YLR106C	998
YER006W	YLR276C	982
YER006W	YLR325C	978
YER006W	YLR406C	974
YER006W	YLR449W	993
YER006W	YMR049C	999
YER006W	YMR290C	999
YER006W	YNL002C	998
YER006W	YNL061W	999
YER006W	YNL110C	999
YER006W	YNL182C	993
YER006W	YNR053C	996
YER006W	YOL077C	994
YER006W	YOL127W	954
YER006W	YOR063W	948
YER006W	YOR206W	999
YER006W	YOR272W	998
YER006W	YPL043W	997
YER006W	YPL093W	999
YER006W	YPL131W	967
YER006W	YPL146C	934
YER006W	YPL198W	942
YER006W	YPL211W	998
YER006W	YPL249C-A	929
YER006W	YPR016C	999
YER007C-A	YJR014W	999
YER007C-A	YKL028W	931
YER007W	YEL061C	956
YER007W	YFL037W	995
YER007W	YGL086W	964
YER007W	YJL030W	964
YER007W	YML085C	999
YER007W	YML124C	987
YER007W	YNL148C	993
YER007W	YOR265W	999
YER007W	YOR349W	999
YER008C	YBR102C	999
YER008C	YDR164C	985
YER008C	YDR166C	999
YER008C	YFL005W	997
YER008C	YFL038C	941

YER008C	YGL233W	999
YER008C	YGR009C	997
YER008C	YIL068C	999
YER008C	YJL085W	999
YER008C	YLR166C	999
YER008C	YLR229C	998
YER008C	YMR183C	993
YER008C	YNL272C	941
YER008C	YOR122C	991
YER008C	YPR032W	998
YER008C	YPR055W	999
YER008C	YPR165W	976
YER009W	YDR002W	902
YER009W	YJL041W	976
YER009W	YJR074W	969
YER009W	YLR264W	929
YER009W	YLR293C	998
YER009W	YLR347C	967
YER009W	YNL189W	916
YER009W	YOR185C	986
YER011W	YGR103W	929
YER011W	YGR104C	938
YER011W	YLR150W	940
YER011W	YLR347C	989
YER011W	YNL139C	938
YER011W	YOR001W	923
YER011W	YOR310C	923
YER012W	YBL041W	999
YER012W	YBR082C	983
YER012W	YBR160W	904
YER012W	YBR173C	929
YER012W	YDL007W	999
YER012W	YDL097C	999
YER012W	YDL147W	999
YER012W	YDR177W	927
YER012W	YDR394W	999
YER012W	YDR427W	999
YER012W	YEL037C	999
YER012W	YER021W	999
YER012W	YER094C	999
YER012W	YFL007W	992
YER012W	YFR004W	999
YER012W	YFR010W	999
YER012W	YFR050C	999

YER012W	YFR052W	999
YER012W	YGL011C	999
YER012W	YGL048C	999
YER012W	YGR135W	999
YER012W	YGR232W	956
YER012W	YGR253C	999
YER012W	YHR027C	999
YER012W	YHR200W	999
YER012W	YIL075C	999
YER012W	YIL148W	900
YER012W	YJL001W	999
YER012W	YKL145W	999
YER012W	YKR094C	900
YER012W	YLL039C	900
YER012W	YLR421C	999
YER012W	YML092C	999
YER012W	YMR314W	999
YER012W	YOL038W	999
YER012W	YOR117W	999
YER012W	YOR157C	999
YER012W	YOR259C	999
YER012W	YOR261C	999
YER012W	YOR362C	999
YER012W	YPR103W	999
YER012W	YPR108W	999
YER012W	YPR120C	929
YER013W	YAL032C	932
YER013W	YDR088C	953
YER013W	YDR364C	945
YER013W	YDR416W	922
YER013W	YHR165C	948
YER013W	YLR117C	999
YER013W	YML085C	910
YER013W	YMR213W	989
YER014W	YDR044W	931
YER015W	YGL205W	933
YER015W	YLR195C	932
YER015W	YPL147W	935
YER016W	YBR107C	980
YER016W	YCL016C	967
YER016W	YCL029C	999
YER016W	YCL061C	906
YER016W	YDL064W	968
YER016W	YDR150W	995

YER016W	YDR254W	982
YER016W	YDR318W	975
YER016W	YDR383C	979
YER016W	YDR488C	968
YER016W	YEL003W	929
YER016W	YER177W	962
YER016W	YFR050C	999
YER016W	YGL061C	974
YER016W	YGL086W	999
YER016W	YGL216W	973
YER016W	YGR113W	968
YER016W	YGR188C	991
YER016W	YGR218W	902
YER016W	YHR129C	984
YER016W	YHR134W	977
YER016W	YHR191C	989
YER016W	YJL013C	993
YER016W	YJL030W	994
YER016W	YJR053W	988
YER016W	YJR060W	965
YER016W	YJR135C	978
YER016W	YKR037C	944
YER016W	YKR054C	998
YER016W	YLL049W	953
YER016W	YLR045C	999
YER016W	YLR085C	941
YER016W	YLR315W	982
YER016W	YLR381W	949
YER016W	YML085C	988
YER016W	YML094W	938
YER016W	YML124C	910
YER016W	YMR048W	949
YER016W	YMR055C	986
YER016W	YMR078C	923
YER016W	YMR117C	983
YER016W	YMR294W	996
YER016W	YNL153C	940
YER016W	YOR026W	988
YER016W	YOR058C	971
YER016W	YOR073W	965
YER016W	YOR156C	974
YER016W	YOR195W	994
YER016W	YOR269W	990
YER016W	YPL018W	933

YER016W	YPL155C	961
YER016W	YPL269W	999
YER016W	YPR046W	951
YER016W	YPR141C	933
YER017C	YBR185C	980
YER017C	YGR132C	973
YER017C	YLR450W	978
YER017C	YMR089C	999
YER018C	YAL034W-A	999
YER018C	YDR318W	978
YER018C	YDR320C-A	963
YER018C	YFR046C	963
YER018C	YGL060W	981
YER018C	YGL086W	962
YER018C	YIL144W	999
YER018C	YIR010W	993
YER018C	YJR112W	963
YER018C	YKR037C	963
YER018C	YMR117C	999
YER018C	YOL069W	999
YER018C	YPR046W	963
YER019C-A	YBR283C	978
YER019C-A	YDL100C	903
YER019C-A	YDR086C	993
YER019C-A	YEL022W	923
YER019C-A	YER087C-B	999
YER019C-A	YJR010C-A	954
YER019C-A	YLR378C	991
YER019C-A	YML055W	922
YER019C-A	YPL094C	908
YER019C-A	YPL210C	923
YER020W	YAL056W	998
YER020W	YBL016W	926
YER020W	YDL035C	999
YER020W	YHR005C	993
YER020W	YHR205W	976
YER020W	YJL005W	975
YER020W	YJL106W	976
YER020W	YNL098C	999
YER020W	YOR107W	955
YER020W	YOR371C	967
YER021W	YBL041W	998
YER021W	YBL042C	900
YER021W	YDL007W	999

YER021W	YDL097C	999
YER021W	YDL147W	999
YER021W	YDR363W-A	999
YER021W	YDR394W	999
YER021W	YDR427W	999
YER021W	YEL037C	983
YER021W	YER012W	999
YER021W	YER094C	944
YER021W	YFR004W	999
YER021W	YFR010W	999
YER021W	YFR050C	962
YER021W	YFR052W	999
YER021W	YGL011C	927
YER021W	YGL048C	999
YER021W	YGR135W	934
YER021W	YGR232W	999
YER021W	YGR253C	934
YER021W	YHL030W	993
YER021W	YHR027C	999
YER021W	YHR200W	999
YER021W	YIL075C	998
YER021W	YJL001W	978
YER021W	YKL145W	999
YER021W	YLL039C	971
YER021W	YLR421C	992
YER021W	YML092C	933
YER021W	YMR191W	972
YER021W	YMR314W	998
YER021W	YOL038W	999
YER021W	YOR117W	998
YER021W	YOR157C	939
YER021W	YOR259C	999
YER021W	YOR261C	999
YER021W	YOR362C	959
YER021W	YPR103W	922
YER021W	YPR108W	999
YER022W	YBL093C	999
YER022W	YBR154C	981
YER022W	YBR193C	999
YER022W	YBR253W	999
YER022W	YBR279W	972
YER022W	YCR042C	909
YER022W	YCR081W	998
YER022W	YDL005C	999

YER022W	YDL108W	962
YER022W	YDL140C	999
YER022W	YDR146C	973
YER022W	YDR167W	978
YER022W	YDR308C	999
YER022W	YDR397C	912
YER022W	YDR404C	981
YER022W	YDR443C	999
YER022W	YDR448W	957
YER022W	YEL009C	973
YER022W	YER148W	995
YER022W	YGL025C	999
YER022W	YGL070C	981
YER022W	YGL127C	999
YER022W	YGL151W	999
YER022W	YGL244W	970
YER022W	YGR005C	995
YER022W	YGR104C	999
YER022W	YGR186W	999
YER022W	YHR041C	999
YER022W	YHR058C	999
YER022W	YHR143W-A	981
YER022W	YIL021W	981
YER022W	YJL140W	981
YER022W	YLR071C	999
YER022W	YLR418C	970
YER022W	YMR112C	999
YER022W	YMR236W	978
YER022W	YNL236W	999
YER022W	YNR010W	999
YER022W	YOL005C	996
YER022W	YOL051W	999
YER022W	YOL135C	999
YER022W	YOL145C	970
YER022W	YOR123C	964
YER022W	YOR151C	981
YER022W	YOR174W	999
YER022W	YOR210W	981
YER022W	YOR224C	981
YER022W	YPL042C	999
YER022W	YPL129W	998
YER022W	YPL248C	997
YER022W	YPR056W	984
YER022W	YPR070W	999

YER022W	YPR168W	999
YER022W	YPR187W	981
YER023W	YDR300C	972
YER023W	YHR037W	937
YER023W	YNL229C	927
YER023W	YOR323C	985
YER023W	YPL070W	923
YER024W	YCR005C	927
YER025W	YAL035W	988
YER025W	YBL072C	931
YER025W	YBR048W	998
YER025W	YBR079C	999
YER025W	YBR159W	981
YER025W	YBR181C	961
YER025W	YBR189W	968
YER025W	YCR031C	945
YER025W	YDL061C	953
YER025W	YDR025W	914
YER025W	YDR064W	968
YER025W	YDR211W	999
YER025W	YDR429C	987
YER025W	YER102W	948
YER025W	YER131W	938
YER025W	YER165W	964
YER025W	YGL123W	954
YER025W	YGR027C	938
YER025W	YGR083C	999
YER025W	YGR118W	972
YER025W	YGR162W	931
YER025W	YGR214W	974
YER025W	YHL015W	950
YER025W	YHR203C	953
YER025W	YIL126W	923
YER025W	YIL133C	915
YER025W	YJL136C	916
YER025W	YJL191W	948
YER025W	YJR007W	999
YER025W	YJR123W	964
YER025W	YKL156W	954
YER025W	YKL181W	940
YER025W	YKR026C	999
YER025W	YKR059W	949
YER025W	YLR048W	994
YER025W	YLR167W	918

YER025W	YLR192C	905
YER025W	YLR215C	999
YER025W	YLR264W	974
YER025W	YLR291C	999
YER025W	YLR367W	952
YER025W	YLR388W	961
YER025W	YLR441C	901
YER025W	YML024W	952
YER025W	YML063W	972
YER025W	YMR146C	999
YER025W	YMR230W	913
YER025W	YMR309C	999
YER025W	YNL178W	939
YER025W	YOL139C	900
YER025W	YOR096W	953
YER025W	YOR167C	944
YER025W	YOR260W	999
YER025W	YOR293W	936
YER025W	YOR361C	976
YER025W	YOR369C	926
YER025W	YPL081W	920
YER025W	YPL090C	961
YER025W	YPL237W	999
YER025W	YPR041W	999
YER025W	YPR132W	932
YER025W	YPR163C	917
YER026C	YCL004W	968
YER026C	YGR208W	904
YER026C	YPR113W	905
YER027C	YDR028C	999
YER027C	YDR422C	999
YER027C	YDR477W	999
YER027C	YER129W	999
YER027C	YGL115W	999
YER027C	YGL208W	999
YER027C	YJL089W	999
YER027C	YJL217W	922
YER027C	YKL048C	960
YER027C	YMR280C	928
YER029C	YBL026W	999
YER029C	YBL074C	995
YER029C	YBR055C	999
YER029C	YBR119W	998
YER029C	YBR152W	998

YER029C	YBR154C	915
YER029C	YDL030W	997
YER029C	YDL043C	996
YER029C	YDL087C	999
YER029C	YDL098C	994
YER029C	YDL140C	910
YER029C	YDR235W	997
YER029C	YDR240C	996
YER029C	YDR378C	997
YER029C	YDR473C	996
YER029C	YEL026W	936
YER029C	YER112W	999
YER029C	YER146W	930
YER029C	YER172C	999
YER029C	YFL017W-A	999
YER029C	YGL070C	947
YER029C	YGL120C	982
YER029C	YGL128C	992
YER029C	YGR013W	996
YER029C	YGR074W	999
YER029C	YGR075C	974
YER029C	YGR091W	999
YER029C	YHR086W	998
YER029C	YHR165C	999
YER029C	YIL061C	997
YER029C	YIR009W	994
YER029C	YJL203W	993
YER029C	YJR022W	999
YER029C	YKL012W	996
YER029C	YKL173W	999
YER029C	YLR117C	998
YER029C	YLR147C	999
YER029C	YLR275W	999
YER029C	YLR298C	999
YER029C	YLR424W	990
YER029C	YLR438C-A	994
YER029C	YML046W	997
YER029C	YML049C	999
YER029C	YMR125W	999
YER029C	YMR240C	999
YER029C	YMR288W	999
YER029C	YNL147W	986
YER029C	YOL005C	910
YER029C	YOR151C	900

YER029C	YOR159C	999
YER029C	YOR210W	929
YER029C	YOR224C	939
YER029C	YOR308C	989
YER029C	YOR319W	973
YER029C	YPL151C	993
YER029C	YPL157W	984
YER029C	YPL178W	961
YER029C	YPL213W	999
YER029C	YPR082C	999
YER029C	YPR101W	995
YER029C	YPR178W	996
YER029C	YPR182W	999
YER029C	YPR187W	928
YER031C	YDL226C	963
YER031C	YDR407C	936
YER031C	YER136W	999
YER031C	YFL038C	979
YER031C	YGL198W	913
YER031C	YGL210W	995
YER031C	YGR166W	926
YER031C	YGR172C	999
YER031C	YJL204C	933
YER031C	YMR218C	999
YER031C	YNL044W	955
YER031C	YNL263C	988
YER031C	YNL267W	924
YER031C	YNL293W	906
YER032W	YDR195W	991
YER032W	YDR510W	926
YER032W	YER161C	924
YER032W	YER165W	905
YER032W	YGR140W	929
YER032W	YGR178C	928
YER032W	YMR117C	939
YER033C	YDR293C	926
YER033C	YNL161W	914
YER035W	YNL118C	992
YER035W	YOL149W	928
YER036C	YAL035W	981
YER036C	YBR079C	906
YER036C	YDR101C	967
YER036C	YHL033C	915
YER036C	YPR036W	921

YER040W	YBR128C	907
YER040W	YDL047W	997
YER040W	YDL215C	947
YER040W	YDR099W	945
YER040W	YDR477W	933
YER040W	YEL009C	987
YER040W	YFL021W	999
YER040W	YGL073W	908
YER040W	YGR218W	924
YER040W	YIR023W	961
YER040W	YIR032C	987
YER040W	YJR066W	999
YER040W	YJR152W	945
YER040W	YKL203C	964
YER040W	YKR039W	972
YER040W	YNL006W	979
YER040W	YNL032W	906
YER040W	YNL142W	935
YER040W	YNL183C	952
YER040W	YNL189W	987
YER040W	YNL229C	999
YER040W	YNL314W	921
YER040W	YOR348C	904
YER040W	YOR375C	932
YER040W	YPL254W	961
YER040W	YPR035W	977
YER041W	YBR160W	916
YER042W	YCL033C	995
YER042W	YGR209C	917
YER042W	YIR037W	995
YER042W	YLR043C	903
YER042W	YPL048W	923
YER043C	YFL028C	999
YER043C	YGR155W	919
YER044C	YER005W	914
YER044C	YGL001C	989
YER044C	YGL012W	999
YER044C	YGR060W	998
YER044C	YHR007C	999
YER044C	YHR072W	999
YER044C	YHR190W	999
YER044C	YLR056W	999
YER044C	YLR100W	998
YER044C	YLR228C	908

YER044C	YML008C	999
YER044C	YMR202W	999
YER044C	YNL280C	999
YER044C-A	YHR014W	983
YER044C-A	YHR157W	940
YER044C-A	YJR021C	969
YER044C-A	YLR329W	940
YER044C-A	YMR133W	998
YER047C	YDR510W	942
YER047C	YGR140W	929
YER048W-A	YCL017C	999
YER048W-A	YDR051C	935
YER049W	YER165W	910
YER049W	YLR249W	972
YER050C	Q0140	962
YER050C	YBR146W	986
YER050C	YDR041W	986
YER050C	YGL129C	986
YER050C	YKL155C	918
YER050C	YNR036C	999
YER050C	YOR158W	986
YER050C	YPL118W	986
YER052C	YDR158W	996
YER052C	YJR139C	998
YER052C	YNL135C	998
YER054C	YER133W	999
YER054C	YLR258W	976
YER054C	YML016C	960
YER055C	YBR248C	960
YER055C	YCL030C	999
YER055C	YIL116W	990
YER055C	YOR202W	973
YER056C-A	YAL035W	919
YER056C-A	YBL027W	904
YER056C-A	YBL087C	901
YER056C-A	YBL092W	920
YER056C-A	YBR031W	935
YER056C-A	YBR048W	921
YER056C-A	YBR084C-A	993
YER056C-A	YBR189W	981
YER056C-A	YBR191W	999
YER056C-A	YCR031C	972
YER056C-A	YDL061C	996
YER056C-A	YDL075W	999

YER056C-A	YDL081C	908
YER056C-A	YDL130W	939
YER056C-A	YDL136W	981
YER056C-A	YDL191W	905
YER056C-A	YDL192W	964
YER056C-A	YDR012W	962
YER056C-A	YDR064W	999
YER056C-A	YDR382W	996
YER056C-A	YDR418W	987
YER056C-A	YDR447C	924
YER056C-A	YDR450W	944
YER056C-A	YDR471W	978
YER056C-A	YDR500C	996
YER056C-A	YEL054C	915
YER056C-A	YER074W	923
YER056C-A	YER102W	993
YER056C-A	YER117W	996
YER056C-A	YER131W	953
YER056C-A	YFL034C-A	911
YER056C-A	YFR031C-A	903
YER056C-A	YFR032C-A	906
YER056C-A	YGL030W	999
YER056C-A	YGL031C	931
YER056C-A	YGL076C	939
YER056C-A	YGL103W	996
YER056C-A	YGL123W	994
YER056C-A	YGL135W	913
YER056C-A	YGL189C	910
YER056C-A	YGR027C	973
YER056C-A	YGR034W	911
YER056C-A	YGR085C	972
YER056C-A	YGR118W	999
YER056C-A	YGR148C	995
YER056C-A	YGR214W	987
YER056C-A	YHL001W	994
YER056C-A	YHL015W	986
YER056C-A	YHL033C	998
YER056C-A	YHR010W	978
YER056C-A	YHR021C	903
YER056C-A	YHR141C	916
YER056C-A	YHR203C	994
YER056C-A	YIL018W	903
YER056C-A	YIL052C	999
YER056C-A	YIL069C	906

YER056C-A	YIL133C	913
YER056C-A	YIL148W	991
YER056C-A	YJL136C	965
YER056C-A	YJL177W	985
YER056C-A	YJL189W	952
YER056C-A	YJL190C	900
YER056C-A	YJL191W	972
YER056C-A	YJR094W-A	999
YER056C-A	YJR123W	998
YER056C-A	YJR145C	913
YER056C-A	YKL006W	999
YER056C-A	YKL156W	993
YER056C-A	YKL180W	999
YER056C-A	YKR057W	909
YER056C-A	YLL045C	922
YER056C-A	YLR029C	969
YER056C-A	YLR048W	997
YER056C-A	YLR060W	944
YER056C-A	YLR061W	999
YER056C-A	YLR075W	994
YER056C-A	YLR167W	994
YER056C-A	YLR185W	987
YER056C-A	YLR264W	993
YER056C-A	YLR287C-A	907
YER056C-A	YLR333C	914
YER056C-A	YLR340W	980
YER056C-A	YLR344W	998
YER056C-A	YLR367W	970
YER056C-A	YLR388W	999
YER056C-A	YLR406C	910
YER056C-A	YLR441C	905
YER056C-A	YLR448W	999
YER056C-A	YML024W	999
YER056C-A	YML026C	902
YER056C-A	YML063W	993
YER056C-A	YML073C	911
YER056C-A	YMR121C	908
YER056C-A	YMR142C	923
YER056C-A	YMR194W	999
YER056C-A	YMR230W	944
YER056C-A	YMR242C	903
YER056C-A	YMR260C	902
YER056C-A	YNL067W	978
YER056C-A	YNL069C	913

YER056C-A	YNL096C	921
YER056C-A	YNL162W	916
YER056C-A	YNL178W	999
YER056C-A	YNL209W	976
YER056C-A	YNL301C	987
YER056C-A	YNL302C	905
YER056C-A	YOL039W	913
YER056C-A	YOL040C	900
YER056C-A	YOL121C	905
YER056C-A	YOL127W	997
YER056C-A	YOR063W	991
YER056C-A	YOR096W	997
YER056C-A	YOR133W	932
YER056C-A	YOR167C	941
YER056C-A	YOR182C	907
YER056C-A	YOR234C	993
YER056C-A	YOR293W	970
YER056C-A	YOR312C	923
YER056C-A	YOR369C	912
YER056C-A	YPL037C	999
YER056C-A	YPL079W	964
YER056C-A	YPL081W	933
YER056C-A	YPL131W	991
YER056C-A	YPL143W	980
YER056C-A	YPL198W	910
YER056C-A	YPL220W	993
YER056C-A	YPL249C-A	957
YER056C-A	YPR043W	961
YER056C-A	YPR102C	985
YER056C-A	YPR132W	994
YER059W	YBR160W	989
YER059W	YDL179W	986
YER059W	YGL215W	964
YER059W	YJL084C	952
YER059W	YLR190W	978
YER059W	YMR311C	988
YER059W	YPL031C	999
YER059W	YPL046C	924
YER059W	YPL219W	975
YER059W	YPL256C	942
YER061C	YKL055C	948
YER061C	YKL192C	984
YER062C	YDL022W	972
YER062C	YIL053W	969

YER062C	YPL201C	992
YER063W	YDL084W	988
YER063W	YDR138W	987
YER063W	YGL181W	974
YER063W	YOR333C	998
YER065C	YBR218C	933
YER065C	YIR031C	983
YER065C	YLR304C	934
YER065C	YNL117W	997
YER068W	YAL021C	999
YER068W	YBR082C	992
YER068W	YCR093W	999
YER068W	YDL165W	999
YER068W	YDR059C	945
YER068W	YGR134W	908
YER068W	YGR252W	970
YER068W	YIL038C	999
YER068W	YLR226W	984
YER068W	YML098W	918
YER068W	YNL288W	998
YER068W	YNR052C	999
YER068W	YPL037C	918
YER068W	YPL042C	912
YER068W	YPR025C	979
YER068W	YPR072W	999
YER069W	YJL071W	992
YER069W	YJR109C	923
YER069W	YMR062C	950
YER069W	YOL140W	954
YER070W	YGR180C	999
YER070W	YGR209C	989
YER070W	YIL066C	999
YER070W	YJL026W	999
YER070W	YJR057W	915
YER070W	YKL024C	900
YER070W	YKL067W	913
YER070W	YLR043C	989
YER070W	YML058W	999
YER070W	YOR330C	976
YER070W	YPL091W	988
YER071C	YIR003W	999
YER071C	YKL007W	999
YER072W	YJL012C	918
YER073W	YAL054C	901

YER073W	YBR145W	901
YER073W	YDL078C	923
YER073W	YDL168W	916
YER073W	YGL256W	956
YER073W	YLR153C	903
YER073W	YMR083W	901
YER073W	YMR169C	910
YER073W	YMR170C	909
YER073W	YMR303C	902
YER073W	YOL086C	901
YER073W	YOR374W	904
YER074W	YBL027W	994
YER074W	YBL072C	996
YER074W	YBL087C	916
YER074W	YBL092W	995
YER074W	YBR031W	996
YER074W	YBR048W	996
YER074W	YBR084C-A	902
YER074W	YBR118W	904
YER074W	YBR181C	994
YER074W	YBR189W	996
YER074W	YBR191W	995
YER074W	YCR031C	995
YER074W	YDL060W	981
YER074W	YDL061C	910
YER074W	YDL075W	995
YER074W	YDL081C	903
YER074W	YDL082W	995
YER074W	YDL083C	962
YER074W	YDL130W	903
YER074W	YDL136W	996
YER074W	YDL191W	922
YER074W	YDR012W	995
YER074W	YDR025W	996
YER074W	YDR064W	995
YER074W	YDR382W	995
YER074W	YDR418W	924
YER074W	YDR429C	919
YER074W	YDR447C	995
YER074W	YDR450W	997
YER074W	YDR471W	995
YER074W	YDR500C	917
YER074W	YEL054C	924
YER074W	YER056C-A	923

YER074W	YER102W	994
YER074W	YER117W	916
YER074W	YER131W	921
YER074W	YFL034C-A	904
YER074W	YFR031C-A	995
YER074W	YFR032C-A	996
YER074W	YGL014W	950
YER074W	YGL030W	997
YER074W	YGL031C	906
YER074W	YGL076C	996
YER074W	YGL103W	995
YER074W	YGL123W	913
YER074W	YGL135W	995
YER074W	YGL147C	995
YER074W	YGL189C	921
YER074W	YGR027C	916
YER074W	YGR034W	995
YER074W	YGR085C	995
YER074W	YGR118W	998
YER074W	YGR148C	906
YER074W	YGR214W	909
YER074W	YHL001W	995
YER074W	YHL015W	905
YER074W	YHL033C	995
YER074W	YHR010W	907
YER074W	YHR021C	918
YER074W	YHR141C	995
YER074W	YHR203C	995
YER074W	YIL018W	995
YER074W	YIL052C	906
YER074W	YIL069C	995
YER074W	YIL133C	995
YER074W	YJL136C	922
YER074W	YJL177W	994
YER074W	YJL190C	995
YER074W	YJL191W	918
YER074W	YJR094W-A	917
YER074W	YJR123W	997
YER074W	YJR145C	993
YER074W	YKL006W	916
YER074W	YKL156W	918
YER074W	YKL180W	995
YER074W	YKR057W	922
YER074W	YLL045C	994

YER074W	YLR029C	996
YER074W	YLR048W	909
YER074W	YLR061W	905
YER074W	YLR075W	996
YER074W	YLR150W	952
YER074W	YLR185W	918
YER074W	YLR264W	900
YER074W	YLR287C-A	994
YER074W	YLR325C	929
YER074W	YLR333C	915
YER074W	YLR340W	995
YER074W	YLR344W	905
YER074W	YLR367W	905
YER074W	YLR388W	911
YER074W	YLR406C	902
YER074W	YLR441C	995
YER074W	YLR448W	996
YER074W	YML024W	918
YER074W	YML026C	901
YER074W	YML063W	995
YER074W	YML073C	996
YER074W	YMR121C	915
YER074W	YMR142C	995
YER074W	YMR194W	996
YER074W	YMR230W	920
YER074W	YMR242C	996
YER074W	YMR260C	905
YER074W	YNL067W	905
YER074W	YNL069C	995
YER074W	YNL096C	995
YER074W	YNL162W	913
YER074W	YNL178W	996
YER074W	YNL301C	994
YER074W	YNL302C	931
YER074W	YOL040C	997
YER074W	YOL120C	902
YER074W	YOL121C	932
YER074W	YOL127W	997
YER074W	YOR063W	994
YER074W	YOR096W	997
YER074W	YOR167C	900
YER074W	YOR182C	901
YER074W	YOR234C	998
YER074W	YOR293W	920

YER074W	YOR312C	995
YER074W	YOR369C	937
YER074W	YPL079W	906
YER074W	YPL081W	917
YER074W	YPL090C	994
YER074W	YPL131W	931
YER074W	YPL143W	924
YER074W	YPL198W	996
YER074W	YPL220W	906
YER074W	YPL249C-A	996
YER074W	YPR043W	917
YER074W	YPR080W	909
YER074W	YPR102C	905
YER074W	YPR132W	922
YER074W-A	YGR172C	931
YER074W-A	YNL263C	934
YER075C	YBL016W	998
YER075C	YHR030C	994
YER075C	YLR113W	999
YER075C	YOR208W	999
YER081W	YHR215W	989
YER081W	YIL074C	999
YER081W	YOR184W	986
YER082C	YBL004W	999
YER082C	YBR156C	935
YER082C	YBR247C	999
YER082C	YCL059C	999
YER082C	YCR057C	999
YER082C	YDL014W	999
YER082C	YDL148C	999
YER082C	YDL153C	922
YER082C	YDR064W	931
YER082C	YDR299W	944
YER082C	YDR324C	999
YER082C	YDR449C	999
YER082C	YFR028C	912
YER082C	YGR090W	999
YER082C	YGR128C	999
YER082C	YGR145W	997
YER082C	YHR148W	997
YER082C	YHR196W	999
YER082C	YJL010C	983
YER082C	YJL109C	999
YER082C	YJR002W	999

YER082C	YKR060W	997
YER082C	YLL011W	999
YER082C	YLR003C	975
YER082C	YLR129W	999
YER082C	YLR186W	997
YER082C	YLR197W	999
YER082C	YLR222C	999
YER082C	YLR409C	999
YER082C	YMR093W	999
YER082C	YMR128W	997
YER082C	YMR229C	972
YER082C	YMR290C	980
YER082C	YNL132W	999
YER082C	YOR078W	991
YER082C	YOR096W	929
YER082C	YOR310C	908
YER082C	YPL012W	913
YER082C	YPL126W	999
YER082C	YPL217C	999
YER082C	YPL266W	997
YER082C	YPR112C	987
YER082C	YPR137W	999
YER082C	YPR144C	997
YER083C	YDL100C	999
YER083C	YGL020C	977
YER083C	YOR070C	925
YER086W	YBR263W	909
YER086W	YCL009C	922
YER086W	YCL018W	924
YER086W	YCR053W	919
YER086W	YGL245W	962
YER086W	YGR155W	908
YER086W	YIL078W	922
YER086W	YJR016C	938
YER086W	YJR048W	909
YER086W	YKL029C	967
YER086W	YLR259C	982
YER086W	YLR355C	932
YER086W	YMR108W	990
YER086W	YOR133W	960
YER087C-B	YBR171W	997
YER087C-B	YBR283C	975
YER087C-B	YDL232W	968
YER087C-B	YDR086C	999

YER087C-B	YDR233C	982
YER087C-B	YEL002C	995
YER087C-B	YER019C-A	999
YER087C-B	YGL022W	977
YER087C-B	YGL233W	985
YER087C-B	YJL002C	978
YER087C-B	YJR010C-A	948
YER087C-B	YLR292C	992
YER087C-B	YLR378C	999
YER087C-B	YML055W	922
YER087C-B	YOR085W	979
YER087C-B	YOR103C	979
YER087C-B	YOR254C	997
YER087C-B	YPL094C	979
YER088C	YDL076C	988
YER088C	YGR121C	916
YER088C	YNL142W	914
YER089C	YBL056W	948
YER089C	YBR136W	935
YER089C	YDR071C	999
YER089C	YDR075W	944
YER089C	YHR079C	992
YER089C	YLR113W	965
YER089C	YNL053W	912
YER089C	YOR208W	979
YER089C	YPL153C	999
YER090W	YDR354W	994
YER090W	YGL026C	975
YER090W	YGL148W	947
YER090W	YKL211C	999
YER091C	YDR502C	938
YER091C	YFR055W	974
YER091C	YGR264C	950
YER091C	YJR130C	906
YER091C	YLR180W	995
YER091C	YLR303W	964
YER091C	YML082W	902
YER091C	YOR241W	918
YER092W	YDL002C	999
YER092W	YDR190C	993
YER092W	YDR334W	908
YER092W	YDR485C	917
YER092W	YFL013C	998
YER092W	YGL150C	999

YER092W	YJL081C	986
YER092W	YLR052W	998
YER092W	YLR423C	992
YER092W	YNL059C	928
YER092W	YOR141C	998
YER092W	YOR304W	942
YER092W	YPL129W	997
YER092W	YPL235W	991
YER093C	YFL039C	911
YER093C	YHR030C	921
YER093C	YHR186C	908
YER093C	YIL105C	996
YER093C	YJL058C	977
YER093C	YJR066W	922
YER093C	YKL203C	999
YER093C	YMR068W	998
YER093C	YMR104C	951
YER093C	YNL006W	999
YER093C	YNL047C	978
YER093C	YOL078W	999
YER094C	YBL041W	999
YER094C	YBR173C	929
YER094C	YCR076C	982
YER094C	YDL007W	912
YER094C	YDL097C	945
YER094C	YDL147W	960
YER094C	YDL188C	946
YER094C	YDR394W	913
YER094C	YDR427W	921
YER094C	YER012W	999
YER094C	YER021W	944
YER094C	YFL007W	999
YER094C	YFR004W	939
YER094C	YFR050C	999
YER094C	YFR052W	944
YER094C	YGL011C	999
YER094C	YGL048C	919
YER094C	YGR135W	999
YER094C	YGR253C	999
YER094C	YHL007C	942
YER094C	YHR027C	936
YER094C	YHR200W	999
YER094C	YJL001W	999
YER094C	YKL145W	912

YER094C	YLL039C	970
YER094C	YML092C	999
YER094C	YMR314W	999
YER094C	YOL038W	999
YER094C	YOR117W	913
YER094C	YOR157C	999
YER094C	YOR259C	913
YER094C	YOR261C	928
YER094C	YOR362C	999
YER094C	YPR103W	999
YER094C	YPR108W	942
YER094C	YPR187W	922
YER095W	YAR007C	999
YER095W	YBL088C	993
YER095W	YBR073W	999
YER095W	YBR136W	971
YER095W	YCL061C	914
YER095W	YCR066W	985
YER095W	YDL059C	999
YER095W	YDL064W	996
YER095W	YDR004W	999
YER095W	YDR014W-A	997
YER095W	YDR076W	999
YER095W	YDR127W	902
YER095W	YDR217C	994
YER095W	YDR369C	971
YER095W	YDR386W	964
YER095W	YER173W	998
YER095W	YER179W	997
YER095W	YGL033W	945
YER095W	YGL058W	988
YER095W	YGL163C	999
YER095W	YHR031C	971
YER095W	YJL092W	999
YER095W	YJR043C	966
YER095W	YKL113C	999
YER095W	YKR082W	954
YER095W	YLR032W	991
YER095W	YLR234W	990
YER095W	YLR288C	972
YER095W	YML028W	998
YER095W	YML032C	999
YER095W	YML061C	963
YER095W	YMR167W	924

YER095W	YMR190C	999
YER095W	YMR224C	973
YER095W	YMR284W	923
YER095W	YNL085W	916
YER095W	YNL218W	961
YER095W	YNL250W	999
YER095W	YOR144C	984
YER095W	YOR368W	916
YER095W	YPL020C	969
YER095W	YPL022W	999
YER095W	YPL024W	996
YER095W	YPL153C	969
YER095W	YPL167C	964
YER099C	YBL068W	998
YER099C	YCR036W	914
YER099C	YDL188C	946
YER099C	YGL011C	954
YER099C	YHL011C	903
YER099C	YKL181W	998
YER099C	YMR300C	914
YER099C	YOL061W	999
YER099C	YOR095C	911
YER099C	YPR074C	900
YER100W	YER125W	952
YER100W	YIL030C	938
YER100W	YMR022W	999
YER102W	YAL035W	998
YER102W	YBL004W	929
YER102W	YBL027W	920
YER102W	YBL072C	996
YER102W	YBL087C	928
YER102W	YBL092W	925
YER102W	YBR031W	995
YER102W	YBR048W	998
YER102W	YBR079C	997
YER102W	YBR084C-A	999
YER102W	YBR118W	939
YER102W	YBR142W	978
YER102W	YBR181C	900
YER102W	YBR189W	998
YER102W	YBR191W	986
YER102W	YCL011C	925
YER102W	YCL059C	955
YER102W	YCR031C	961

YER102W	YDL014W	936
YER102W	YDL060W	978
YER102W	YDL061C	964
YER102W	YDL075W	992
YER102W	YDL081C	926
YER102W	YDL082W	928
YER102W	YDL083C	952
YER102W	YDL130W	928
YER102W	YDL136W	999
YER102W	YDL191W	916
YER102W	YDR012W	996
YER102W	YDR025W	995
YER102W	YDR064W	999
YER102W	YDR382W	982
YER102W	YDR385W	912
YER102W	YDR418W	968
YER102W	YDR429C	997
YER102W	YDR447C	995
YER102W	YDR450W	921
YER102W	YDR471W	941
YER102W	YDR500C	999
YER102W	YEL054C	997
YER102W	YER025W	948
YER102W	YER056C-A	993
YER102W	YER074W	994
YER102W	YER117W	999
YER102W	YER131W	924
YER102W	YER165W	919
YER102W	YFL034C-A	913
YER102W	YFR031C-A	915
YER102W	YFR032C-A	929
YER102W	YGL030W	998
YER102W	YGL031C	904
YER102W	YGL076C	995
YER102W	YGL103W	998
YER102W	YGL123W	978
YER102W	YGL135W	916
YER102W	YGL147C	994
YER102W	YGL173C	923
YER102W	YGL189C	910
YER102W	YGR027C	950
YER102W	YGR034W	920
YER102W	YGR085C	996
YER102W	YGR090W	927

YER102W	YGR118W	984
YER102W	YGR148C	950
YER102W	YGR214W	964
YER102W	YHL001W	961
YER102W	YHL015W	962
YER102W	YHL033C	980
YER102W	YHL034C	995
YER102W	YHR010W	938
YER102W	YHR021C	931
YER102W	YHR026W	950
YER102W	YHR089C	970
YER102W	YHR141C	924
YER102W	YHR203C	999
YER102W	YIL018W	994
YER102W	YIL052C	995
YER102W	YIL069C	995
YER102W	YIL133C	994
YER102W	YIL148W	999
YER102W	YJL136C	943
YER102W	YJL177W	969
YER102W	YJL189W	936
YER102W	YJL190C	996
YER102W	YJL191W	954
YER102W	YJR002W	929
YER102W	YJR007W	903
YER102W	YJR094W-A	982
YER102W	YJR123W	998
YER102W	YJR145C	996
YER102W	YKL006W	976
YER102W	YKL156W	964
YER102W	YKL180W	980
YER102W	YKR057W	929
YER102W	YKR059W	999
YER102W	YKR094C	906
YER102W	YLL045C	994
YER102W	YLR029C	996
YER102W	YLR048W	984
YER102W	YLR061W	994
YER102W	YLR075W	997
YER102W	YLR150W	973
YER102W	YLR167W	999
YER102W	YLR185W	999
YER102W	YLR192C	997
YER102W	YLR264W	958

YER102W	YLR325C	915
YER102W	YLR333C	920
YER102W	YLR340W	979
YER102W	YLR344W	989
YER102W	YLR367W	998
YER102W	YLR388W	993
YER102W	YLR406C	919
YER102W	YLR441C	995
YER102W	YLR448W	998
YER102W	YML024W	999
YER102W	YML026C	921
YER102W	YML056C	976
YER102W	YML063W	998
YER102W	YML073C	995
YER102W	YMR121C	915
YER102W	YMR128W	925
YER102W	YMR142C	926
YER102W	YMR146C	997
YER102W	YMR194W	983
YER102W	YMR229C	923
YER102W	YMR230W	929
YER102W	YMR242C	927
YER102W	YMR260C	907
YER102W	YMR290C	937
YER102W	YMR309C	997
YER102W	YNL067W	956
YER102W	YNL069C	920
YER102W	YNL096C	916
YER102W	YNL132W	935
YER102W	YNL162W	924
YER102W	YNL178W	999
YER102W	YNL301C	999
YER102W	YNL302C	923
YER102W	YOL039W	911
YER102W	YOL040C	944
YER102W	YOL120C	924
YER102W	YOL121C	923
YER102W	YOL127W	998
YER102W	YOL139C	997
YER102W	YOR063W	997
YER102W	YOR096W	997
YER102W	YOR133W	990
YER102W	YOR167C	909
YER102W	YOR204W	929

YER102W	YOR234C	951
YER102W	YOR293W	944
YER102W	YOR312C	998
YER102W	YOR369C	923
YER102W	YPL012W	929
YER102W	YPL037C	946
YER102W	YPL079W	935
YER102W	YPL081W	998
YER102W	YPL090C	995
YER102W	YPL131W	998
YER102W	YPL143W	965
YER102W	YPL198W	995
YER102W	YPL220W	999
YER102W	YPL237W	910
YER102W	YPL249C-A	952
YER102W	YPR043W	993
YER102W	YPR080W	909
YER102W	YPR102C	948
YER102W	YPR132W	999
YER103W	YAL005C	997
YER103W	YBL075C	986
YER103W	YDL229W	905
YER103W	YDR172W	909
YER103W	YGL073W	912
YER103W	YJL034W	905
YER103W	YJR132W	960
YER103W	YLL024C	997
YER103W	YLL026W	959
YER103W	YLR403W	913
YER103W	YNL007C	975
YER103W	YNL064C	993
YER103W	YNL077W	927
YER103W	YNL209W	905
YER103W	YOR027W	977
YER103W	YOR039W	902
YER103W	YOR232W	931
YER103W	YPL240C	929
YER104W	YJL173C	988
YER104W	YNL312W	966
YER105C	YBL041W	998
YER105C	YBL079W	997
YER105C	YDL088C	948
YER105C	YDL116W	975
YER105C	YDL207W	962

YER105C	YDR159W	966
YER105C	YFR002W	992
YER105C	YFR050C	999
YER105C	YGL086W	932
YER105C	YGL092W	993
YER105C	YGL100W	959
YER105C	YGL172W	943
YER105C	YGR119C	944
YER105C	YIL115C	957
YER105C	YJL030W	990
YER105C	YJL041W	942
YER105C	YJL061W	984
YER105C	YJR042W	959
YER105C	YKL057C	959
YER105C	YKL068W	956
YER105C	YKR082W	959
YER105C	YLR208W	959
YER105C	YML103C	998
YER105C	YMR047C	962
YER105C	YMR129W	998
YER105C	YMR153W	995
YER105C	YOR257W	961
YER105C	YPL169C	966
YER106W	YCR086W	997
YER106W	YDR439W	979
YER106W	YPL204W	987
YER107C	YBL079W	929
YER107C	YDL088C	937
YER107C	YDL207W	997
YER107C	YDR101C	933
YER107C	YDR159W	993
YER107C	YDR192C	996
YER107C	YEL024W	968
YER107C	YFR002W	999
YER107C	YGL092W	985
YER107C	YGL100W	900
YER107C	YGL172W	947
YER107C	YGR119C	989
YER107C	YIL115C	999
YER107C	YJL030W	915
YER107C	YJL039C	964
YER107C	YJL041W	995
YER107C	YJL061W	999
YER107C	YKL057C	929

YER107C	YKL068W	999
YER107C	YLR018C	987
YER107C	YLR208W	907
YER107C	YLR347C	974
YER107C	YMR047C	999
YER107C	YMR129W	954
YER107C	YOR098C	926
YER107C	YOR257W	961
YER107C	YPL169C	997
YER109C	YAR050W	979
YER109C	YBR083W	998
YER109C	YGR040W	909
YER109C	YHR084W	998
YER109C	YKR102W	981
YER109C	YMR164C	999
YER109C	YOL116W	989
YER109C	YPL203W	922
YER110C	YAL034C	995
YER110C	YAR002W	998
YER110C	YBR009C	958
YER110C	YBR010W	970
YER110C	YBR017C	997
YER110C	YDR192C	992
YER110C	YDR208W	942
YER110C	YDR225W	966
YER110C	YDR381W	967
YER110C	YEL022W	972
YER110C	YFR002W	965
YER110C	YGL016W	942
YER110C	YGL172W	961
YER110C	YGL195W	970
YER110C	YGR119C	998
YER110C	YGR162W	940
YER110C	YIL115C	971
YER110C	YJL039C	915
YER110C	YJL041W	980
YER110C	YJL115W	907
YER110C	YJR123W	979
YER110C	YKL057C	928
YER110C	YKL068W	990
YER110C	YLR293C	999
YER110C	YLR335W	999
YER110C	YLR347C	999
YER110C	YLR441C	978

YER110C	YML007W	976
YER110C	YML103C	935
YER110C	YMR047C	985
YER110C	YMR129W	930
YER110C	YNL030W	945
YER110C	YNL031C	970
YER110C	YNL189W	982
YER110C	YOR098C	997
YER110C	YPL020C	927
YER110C	YPL169C	922
YER110C	YPR093C	977
YER110C	YPR174C	964
YER111C	YAL021C	987
YER111C	YAL040C	999
YER111C	YBL105C	999
YER111C	YBR160W	999
YER111C	YBR279W	991
YER111C	YCR081W	921
YER111C	YDL056W	999
YER111C	YDR146C	977
YER111C	YDR293C	939
YER111C	YDR335W	977
YER111C	YER167W	991
YER111C	YGL173C	917
YER111C	YGL207W	923
YER111C	YGR044C	982
YER111C	YGR229C	938
YER111C	YHR030C	999
YER111C	YIL101C	916
YER111C	YKR077W	942
YER111C	YLR182W	999
YER111C	YLR418C	969
YER111C	YMR199W	957
YER111C	YNL236W	995
YER111C	YNL289W	954
YER111C	YNL298W	951
YER111C	YOR083W	998
YER111C	YPL055C	937
YER111C	YPL089C	939
YER111C	YPL153C	985
YER111C	YPL256C	999
YER111C	YPR119W	999
YER111C	YPR120C	996
YER112W	YBL026W	999

YER112W	YBR055C	993
YER112W	YCR020C-A	984
YER112W	YCR077C	999
YER112W	YDL160C	994
YER112W	YDR378C	999
YER112W	YDR473C	996
YER112W	YER029C	999
YER112W	YER146W	999
YER112W	YER172C	999
YER112W	YFL017W-A	987
YER112W	YGL173C	999
YER112W	YGR074W	981
YER112W	YGR091W	996
YER112W	YHR165C	990
YER112W	YJL124C	999
YER112W	YJR022W	999
YER112W	YKL173W	992
YER112W	YLR147C	998
YER112W	YLR264W	966
YER112W	YLR275W	999
YER112W	YLR438C-A	999
YER112W	YMR268C	998
YER112W	YNL147W	999
YER112W	YOL149W	997
YER112W	YOR197W	917
YER112W	YOR308C	993
YER112W	YPR082C	993
YER112W	YPR178W	993
YER113C	YDR503C	927
YER114C	YAL041W	998
YER114C	YBL085W	982
YER114C	YBR200W	999
YER114C	YER124C	967
YER114C	YIL118W	990
YER114C	YKR055W	988
YER114C	YLR229C	901
YER116C	YAR002W	915
YER116C	YDL013W	999
YER116C	YDL064W	973
YER116C	YDR510W	994
YER116C	YKR082W	940
YER116C	YLR234W	930
YER116C	YML032C	994
YER116C	YMR190C	999

YER116C	YOR156C	996
YER116C	YPL020C	973
YER116C	YPL024W	905
YER116C	YPL082C	906
YER117W	YAL035W	911
YER117W	YBL027W	975
YER117W	YBL072C	928
YER117W	YBL087C	999
YER117W	YBL092W	979
YER117W	YBR031W	977
YER117W	YBR048W	987
YER117W	YBR084C-A	999
YER117W	YBR118W	935
YER117W	YBR181C	901
YER117W	YBR189W	948
YER117W	YBR191W	993
YER117W	YCR031C	974
YER117W	YDL061C	986
YER117W	YDL075W	996
YER117W	YDL081C	923
YER117W	YDL082W	929
YER117W	YDL130W	930
YER117W	YDL136W	999
YER117W	YDL191W	914
YER117W	YDL192W	952
YER117W	YDR012W	979
YER117W	YDR025W	975
YER117W	YDR041W	967
YER117W	YDR051C	956
YER117W	YDR064W	987
YER117W	YDR361C	999
YER117W	YDR382W	980
YER117W	YDR385W	914
YER117W	YDR418W	963
YER117W	YDR447C	928
YER117W	YDR450W	927
YER117W	YDR471W	943
YER117W	YDR500C	999
YER117W	YEL054C	921
YER117W	YER056C-A	996
YER117W	YER074W	916
YER117W	YER102W	999
YER117W	YER131W	926
YER117W	YFL034C-A	910

YER117W	YFR031C-A	981
YER117W	YFR032C-A	940
YER117W	YGL030W	979
YER117W	YGL031C	999
YER117W	YGL076C	931
YER117W	YGL103W	987
YER117W	YGL123W	995
YER117W	YGL135W	911
YER117W	YGL147C	977
YER117W	YGL189C	916
YER117W	YGR027C	949
YER117W	YGR034W	977
YER117W	YGR085C	990
YER117W	YGR118W	983
YER117W	YGR148C	944
YER117W	YGR214W	950
YER117W	YHL001W	964
YER117W	YHL015W	954
YER117W	YHL033C	970
YER117W	YHR010W	938
YER117W	YHR021C	930
YER117W	YHR141C	920
YER117W	YHR203C	999
YER117W	YIL018W	981
YER117W	YIL052C	995
YER117W	YIL069C	916
YER117W	YIL133C	925
YER117W	YIL148W	999
YER117W	YJL136C	954
YER117W	YJL177W	986
YER117W	YJL189W	947
YER117W	YJL190C	972
YER117W	YJL191W	973
YER117W	YJR094W-A	998
YER117W	YJR123W	987
YER117W	YJR145C	975
YER117W	YKL006W	980
YER117W	YKL156W	967
YER117W	YKL170W	935
YER117W	YKL180W	993
YER117W	YKR057W	931
YER117W	YKR059W	998
YER117W	YKR094C	906
YER117W	YLL045C	910

YER117W	YLR029C	946
YER117W	YLR048W	984
YER117W	YLR061W	996
YER117W	YLR075W	998
YER117W	YLR167W	999
YER117W	YLR185W	999
YER117W	YLR264W	966
YER117W	YLR325C	929
YER117W	YLR333C	923
YER117W	YLR340W	989
YER117W	YLR344W	998
YER117W	YLR367W	983
YER117W	YLR388W	998
YER117W	YLR406C	915
YER117W	YLR441C	932
YER117W	YLR448W	977
YER117W	YML024W	997
YER117W	YML026C	927
YER117W	YML063W	959
YER117W	YML073C	931
YER117W	YMR121C	927
YER117W	YMR142C	928
YER117W	YMR194W	982
YER117W	YMR230W	933
YER117W	YMR242C	932
YER117W	YMR260C	913
YER117W	YNL067W	987
YER117W	YNL069C	928
YER117W	YNL081C	967
YER117W	YNL096C	906
YER117W	YNL162W	920
YER117W	YNL178W	999
YER117W	YNL301C	999
YER117W	YNL302C	922
YER117W	YNR037C	980
YER117W	YOL039W	911
YER117W	YOL040C	986
YER117W	YOL120C	923
YER117W	YOL121C	923
YER117W	YOL127W	996
YER117W	YOR063W	999
YER117W	YOR096W	957
YER117W	YOR133W	971
YER117W	YOR167C	916

YER117W	YOR234C	960
YER117W	YOR293W	942
YER117W	YOR312C	957
YER117W	YOR369C	934
YER117W	YPL037C	971
YER117W	YPL079W	940
YER117W	YPL081W	916
YER117W	YPL090C	901
YER117W	YPL131W	995
YER117W	YPL143W	972
YER117W	YPL198W	934
YER117W	YPL220W	999
YER117W	YPL249C-A	955
YER117W	YPR043W	999
YER117W	YPR080W	917
YER117W	YPR102C	990
YER117W	YPR132W	999
YER118C	YCL027W	999
YER118C	YCL032W	987
YER118C	YGR014W	998
YER118C	YHL007C	991
YER118C	YIL147C	960
YER118C	YJL062W	921
YER118C	YJL128C	999
YER118C	YLR006C	998
YER118C	YLR113W	999
YER118C	YLR229C	973
YER118C	YLR362W	999
YER118C	YNR031C	995
YER118C	YOR181W	939
YER120W	YAR042W	971
YER120W	YBR036C	909
YER120W	YDL019C	975
YER120W	YDL072C	923
YER120W	YDR150W	934
YER120W	YFL031W	972
YER120W	YGL238W	922
YER120W	YHL020C	999
YER120W	YIL090W	945
YER120W	YJL153C	986
YER120W	YLR305C	996
YER120W	YMR299C	905
YER122C	YBR162C	962
YER122C	YBR164C	922

YER122C	YDL137W	985
YER122C	YDL145C	999
YER122C	YDL192W	999
YER122C	YDL226C	999
YER122C	YDR238C	999
YER122C	YGL137W	999
YER122C	YIL004C	953
YER122C	YLR078C	955
YER122C	YLR268W	952
YER122C	YNL287W	999
YER122C	YPL137C	923
YER123W	YEL013W	928
YER123W	YHR135C	985
YER124C	YBL085W	924
YER124C	YER114C	967
YER124C	YGL028C	999
YER124C	YHR143W	999
YER125W	YBL101C	988
YER125W	YBR005W	986
YER125W	YBR082C	909
YER125W	YBR118W	939
YER125W	YBR290W	987
YER125W	YDL029W	958
YER125W	YDL123W	927
YER125W	YDL140C	999
YER125W	YDL203C	985
YER125W	YDR054C	962
YER125W	YDR059C	977
YER125W	YDR092W	933
YER125W	YDR388W	955
YER125W	YER100W	952
YER125W	YER151C	909
YER125W	YER177W	939
YER125W	YFL039C	908
YER125W	YFR022W	993
YER125W	YGL144C	991
YER125W	YGR068C	991
YER125W	YGR136W	921
YER125W	YGR268C	901
YER125W	YHL002W	964
YER125W	YHL024W	975
YER125W	YHR131C	998
YER125W	YIL148W	995
YER125W	YIR006C	903

YER125W	YIR033W	999
YER125W	YJL084C	998
YER125W	YJL151C	999
YER125W	YJR017C	916
YER125W	YKL020C	999
YER125W	YKL034W	950
YER125W	YKL176C	911
YER125W	YKR021W	991
YER125W	YKR039W	949
YER125W	YLL039C	999
YER125W	YLR392C	971
YER125W	YML111W	998
YER125W	YMR022W	979
YER125W	YMR275C	999
YER125W	YMR316W	969
YER125W	YNL084C	995
YER125W	YNL183C	968
YER125W	YNL243W	917
YER125W	YOL013C	901
YER125W	YOR018W	939
YER125W	YOR124C	999
YER125W	YOR138C	999
YER125W	YOR322C	979
YER125W	YPL084W	987
YER125W	YPL176C	968
YER125W	YPR030W	985
YER126C	YCR072C	999
YER126C	YDL031W	983
YER126C	YDR101C	999
YER126C	YER006W	999
YER126C	YGL029W	910
YER126C	YGR103W	989
YER126C	YGR245C	999
YER126C	YHR170W	901
YER126C	YHR197W	999
YER126C	YKL009W	999
YER126C	YKR081C	999
YER126C	YLR009W	996
YER126C	YLR074C	999
YER126C	YLR325C	970
YER126C	YMR049C	999
YER126C	YMR290C	999
YER126C	YNL002C	999
YER126C	YNL110C	999

YER126C	YNR053C	999
YER126C	YOR063W	953
YER126C	YPL093W	999
YER126C	YPR016C	999
YER127W	YDR299W	998
YER127W	YGR145W	977
YER127W	YKR002W	917
YER128W	YPR173C	908
YER129W	YDR028C	954
YER129W	YDR422C	999
YER129W	YDR477W	999
YER129W	YER027C	999
YER129W	YGL035C	918
YER129W	YGL115W	999
YER129W	YGL179C	996
YER129W	YGL208W	974
YER129W	YKL048C	999
YER131W	YBL027W	907
YER131W	YBL072C	910
YER131W	YBL087C	929
YER131W	YBL092W	947
YER131W	YBR031W	940
YER131W	YBR048W	926
YER131W	YBR079C	916
YER131W	YBR084C-A	918
YER131W	YBR118W	904
YER131W	YBR181C	915
YER131W	YBR189W	994
YER131W	YBR191W	974
YER131W	YCR031C	952
YER131W	YDL061C	968
YER131W	YDL075W	943
YER131W	YDL081C	908
YER131W	YDL082W	952
YER131W	YDL083C	977
YER131W	YDL130W	908
YER131W	YDL136W	937
YER131W	YDL191W	918
YER131W	YDR012W	957
YER131W	YDR025W	914
YER131W	YDR064W	980
YER131W	YDR382W	953
YER131W	YDR418W	964
YER131W	YDR429C	903

YER131W	YDR447C	997
YER131W	YDR450W	903
YER131W	YDR471W	967
YER131W	YDR500C	931
YER131W	YEL054C	923
YER131W	YER025W	938
YER131W	YER056C-A	953
YER131W	YER074W	921
YER131W	YER102W	924
YER131W	YER117W	926
YER131W	YER165W	911
YER131W	YFL034C-A	912
YER131W	YFR032C-A	922
YER131W	YGL030W	970
YER131W	YGL031C	990
YER131W	YGL076C	971
YER131W	YGL103W	955
YER131W	YGL123W	948
YER131W	YGL135W	914
YER131W	YGL147C	952
YER131W	YGL189C	902
YER131W	YGR027C	999
YER131W	YGR034W	997
YER131W	YGR085C	962
YER131W	YGR118W	970
YER131W	YGR148C	975
YER131W	YGR214W	976
YER131W	YHL001W	979
YER131W	YHL015W	969
YER131W	YHL033C	966
YER131W	YHR010W	974
YER131W	YHR021C	921
YER131W	YHR141C	935
YER131W	YHR203C	925
YER131W	YIL052C	970
YER131W	YIL069C	921
YER131W	YIL133C	924
YER131W	YIL148W	915
YER131W	YJL136C	935
YER131W	YJL177W	970
YER131W	YJL189W	919
YER131W	YJL190C	931
YER131W	YJL191W	960
YER131W	YJR094W-A	940

YER131W	YJR123W	977
YER131W	YJR145C	913
YER131W	YKL006W	979
YER131W	YKL156W	959
YER131W	YKL180W	979
YER131W	YKR057W	922
YER131W	YKR059W	909
YER131W	YLL045C	957
YER131W	YLR029C	979
YER131W	YLR048W	964
YER131W	YLR061W	960
YER131W	YLR075W	948
YER131W	YLR150W	968
YER131W	YLR167W	915
YER131W	YLR185W	930
YER131W	YLR264W	973
YER131W	YLR287C-A	905
YER131W	YLR325C	931
YER131W	YLR330W	980
YER131W	YLR333C	976
YER131W	YLR340W	933
YER131W	YLR344W	930
YER131W	YLR367W	971
YER131W	YLR388W	978
YER131W	YLR406C	909
YER131W	YLR435W	997
YER131W	YLR441C	927
YER131W	YLR448W	959
YER131W	YML024W	946
YER131W	YML026C	903
YER131W	YML063W	986
YER131W	YML073C	916
YER131W	YMR121C	930
YER131W	YMR142C	932
YER131W	YMR194W	998
YER131W	YMR230W	951
YER131W	YMR242C	934
YER131W	YMR260C	907
YER131W	YNL067W	988
YER131W	YNL069C	911
YER131W	YNL096C	934
YER131W	YNL162W	919
YER131W	YNL178W	957
YER131W	YNL301C	924

YER131W	YNL302C	921
YER131W	YOL039W	923
YER131W	YOL040C	931
YER131W	YOL120C	908
YER131W	YOL121C	933
YER131W	YOL127W	973
YER131W	YOR063W	960
YER131W	YOR096W	971
YER131W	YOR167C	963
YER131W	YOR182C	905
YER131W	YOR234C	971
YER131W	YOR293W	975
YER131W	YOR312C	930
YER131W	YOR361C	915
YER131W	YOR369C	952
YER131W	YPL079W	953
YER131W	YPL081W	951
YER131W	YPL131W	935
YER131W	YPL143W	963
YER131W	YPL198W	923
YER131W	YPL220W	924
YER131W	YPL237W	948
YER131W	YPL249C-A	932
YER131W	YPR043W	921
YER131W	YPR080W	904
YER131W	YPR102C	993
YER131W	YPR132W	929
YER131W	YPR163C	915
YER133W	YAL014C	965
YER133W	YAL043C	999
YER133W	YAR014C	999
YER133W	YBL007C	994
YER133W	YBL058W	919
YER133W	YBR045C	999
YER133W	YBR050C	999
YER133W	YCR073W-A	997
YER133W	YDL070W	955
YER133W	YDR028C	999
YER133W	YDR099W	946
YER133W	YDR130C	994
YER133W	YDR195W	999
YER133W	YDR283C	920
YER133W	YDR301W	999
YER133W	YDR412W	984

YER133W	YDR432W	936
YER133W	YDR477W	990
YER133W	YER054C	999
YER133W	YER177W	998
YER133W	YFL023W	975
YER133W	YFR003C	999
YER133W	YGL253W	952
YER133W	YGR113W	962
YER133W	YGR156W	999
YER133W	YIL095W	948
YER133W	YIR006C	982
YER133W	YJL042W	987
YER133W	YJR090C	927
YER133W	YJR093C	999
YER133W	YKL018W	999
YER133W	YKL059C	999
YER133W	YKL193C	999
YER133W	YKR002W	999
YER133W	YLR028C	995
YER133W	YLR115W	999
YER133W	YLR258W	998
YER133W	YLR263W	997
YER133W	YLR277C	999
YER133W	YLR430W	978
YER133W	YMR037C	930
YER133W	YMR140W	949
YER133W	YMR311C	999
YER133W	YNL222W	989
YER133W	YNL233W	999
YER133W	YNL317W	999
YER133W	YOR178C	999
YER133W	YOR179C	995
YER133W	YOR227W	957
YER133W	YOR329C	999
YER133W	YOR351C	920
YER133W	YPL031C	905
YER133W	YPL074W	965
YER133W	YPL137C	998
YER133W	YPL209C	999
YER133W	YPR107C	999
YER136W	YAL048C	902
YER136W	YBR264C	995
YER136W	YDL135C	952
YER136W	YER031C	999

YER136W	YFL005W	999
YER136W	YFL038C	999
YER136W	YGL210W	957
YER136W	YGR172C	956
YER136W	YKR014C	998
YER136W	YLR229C	910
YER136W	YLR262C	997
YER136W	YLR362W	915
YER136W	YML001W	999
YER136W	YOR089C	999
YER136W	YPR165W	936
YER138C	YER160C	909
YER139C	YBR154C	911
YER139C	YDL140C	997
YER139C	YGL070C	906
YER139C	YLR418C	970
YER140W	YOR154W	999
YER141W	Q0045	942
YER141W	YAL039C	901
YER141W	YBR037C	930
YER141W	YPL132W	978
YER141W	YPL172C	995
YER141W	YPL252C	982
YER142C	YDR419W	976
YER142C	YIR002C	968
YER142C	YKL114C	988
YER142C	YPL167C	997
YER143W	YAL030W	975
YER143W	YDR113C	946
YER143W	YEL037C	999
YER143W	YGR009C	985
YER143W	YJR143C	998
YER143W	YLL039C	998
YER143W	YML088W	988
YER143W	YMR055C	941
YER143W	YOR327C	995
YER144C	YBR059C	933
YER144C	YOR157C	999
YER145C	YDR307W	998
YER145C	YGL071W	993
YER145C	YLR214W	937
YER145C	YMR058W	999
YER145C	YMR319C	955
YER145C	YOR357C	924

YER146W	YBL026W	999
YER146W	YBR055C	998
YER146W	YCR020C-A	984
YER146W	YCR077C	999
YER146W	YDL051W	948
YER146W	YDL184C	969
YER146W	YDR378C	999
YER146W	YDR473C	972
YER146W	YER029C	930
YER146W	YER112W	999
YER146W	YER172C	997
YER146W	YGL173C	999
YER146W	YGR074W	944
YER146W	YHR165C	999
YER146W	YJL124C	999
YER146W	YJR022W	999
YER146W	YKL173W	998
YER146W	YKL188C	946
YER146W	YLR147C	993
YER146W	YLR275W	995
YER146W	YLR438C-A	999
YER146W	YMR268C	999
YER146W	YNL145W	969
YER146W	YNL147W	999
YER146W	YOL149W	971
YER146W	YPR178W	999
YER146W	YPR182W	974
YER147C	YDL003W	991
YER147C	YDR180W	999
YER148W	YAL001C	978
YER148W	YAL021C	983
YER148W	YBL014C	998
YER148W	YBR081C	987
YER148W	YBR154C	935
YER148W	YBR193C	984
YER148W	YBR198C	999
YER148W	YBR253W	958
YER148W	YBR279W	952
YER148W	YCR042C	999
YER148W	YCR052W	923
YER148W	YCR093W	977
YER148W	YDL108W	911
YER148W	YDL140C	997
YER148W	YDL150W	965

YER148W	YDR145W	999
YER148W	YDR167W	999
YER148W	YDR176W	999
YER148W	YDR190C	914
YER148W	YDR308C	972
YER148W	YDR392W	999
YER148W	YDR397C	991
YER148W	YDR404C	987
YER148W	YDR448W	999
YER148W	YER022W	995
YER148W	YER159C	999
YER148W	YER171W	904
YER148W	YFR034C	958
YER148W	YGL048C	982
YER148W	YGL070C	922
YER148W	YGL112C	999
YER148W	YGL207W	911
YER148W	YGL241W	999
YER148W	YGL244W	999
YER148W	YGR047C	966
YER148W	YGR104C	998
YER148W	YGR246C	999
YER148W	YGR252W	999
YER148W	YGR274C	999
YER148W	YHR041C	999
YER148W	YHR058C	910
YER148W	YHR099W	980
YER148W	YIL021W	988
YER148W	YIL126W	926
YER148W	YIL143C	956
YER148W	YJL025W	993
YER148W	YJL140W	985
YER148W	YKL028W	905
YER148W	YKL058W	999
YER148W	YLR033W	923
YER148W	YLR055C	999
YER148W	YLR182W	954
YER148W	YLR399C	930
YER148W	YLR418C	998
YER148W	YML015C	999
YER148W	YML043C	993
YER148W	YML098W	999
YER148W	YML114C	999
YER148W	YML123C	903

YER148W	YMR005W	999
YER148W	YMR186W	963
YER148W	YMR227C	999
YER148W	YMR236W	999
YER148W	YMR270C	998
YER148W	YNL039W	999
YER148W	YNL113W	915
YER148W	YNR003C	909
YER148W	YOL051W	985
YER148W	YOL135C	903
YER148W	YOL145C	958
YER148W	YOL148C	999
YER148W	YOR039W	949
YER148W	YOR047C	996
YER148W	YOR123C	952
YER148W	YOR151C	933
YER148W	YOR174W	944
YER148W	YOR194C	999
YER148W	YOR207C	904
YER148W	YOR210W	928
YER148W	YOR224C	910
YER148W	YOR298C-A	978
YER148W	YPL007C	939
YER148W	YPL011C	999
YER148W	YPL082C	999
YER148W	YPL101W	923
YER148W	YPL122C	980
YER148W	YPL129W	999
YER148W	YPL235W	916
YER148W	YPL240C	919
YER148W	YPL248C	999
YER148W	YPL254W	994
YER148W	YPR034W	923
YER148W	YPR052C	967
YER148W	YPR056W	986
YER148W	YPR086W	999
YER148W	YPR110C	918
YER148W	YPR168W	902
YER148W	YPR187W	934
YER149C	YCL027W	999
YER149C	YIL159W	999
YER149C	YLL021W	999
YER149C	YLR319C	994
YER149C	YLR330W	999

YER149C	YLR362W	901
YER149C	YMR124W	923
YER149C	YMR232W	998
YER149C	YNL271C	972
YER149C	YNL298W	966
YER149C	YPL115C	908
YER151C	YDR176W	976
YER151C	YDR227W	996
YER151C	YER125W	909
YER151C	YGL043W	995
YER151C	YGR063C	970
YER151C	YJR043C	924
YER151C	YLL024C	970
YER151C	YNR051C	999
YER151C	YOL012C	970
YER151C	YOL082W	990
YER151C	YPL240C	919
YER151C	YPR181C	902
YER153C	Q0275	999
YER153C	YGR222W	959
YER153C	YKL003C	932
YER153C	YLR067C	910
YER153C	YOR017W	911
YER153C	YOR158W	998
YER154W	Q0085	986
YER154W	Q0130	988
YER154W	Q0250	998
YER154W	Q0275	982
YER154W	YBR185C	992
YER154W	YDR316W	923
YER154W	YDR405W	916
YER154W	YLR289W	923
YER154W	YOR065W	981
YER154W	YPR024W	903
YER155C	YAR014C	981
YER155C	YBR126C	962
YER155C	YBR200W	967
YER155C	YDL047W	993
YER155C	YDR129C	908
YER155C	YDR293C	912
YER155C	YFL039C	972
YER155C	YGL190C	912
YER155C	YGR229C	920
YER155C	YJR090C	901

YER155C	YKR090W	914
YER155C	YLR229C	989
YER155C	YLR371W	994
YER155C	YNL079C	989
YER155C	YNL090W	967
YER155C	YNL180C	954
YER155C	YNL289W	957
YER155C	YOR188W	994
YER155C	YOR326W	932
YER155C	YPL115C	983
YER155C	YPR165W	997
YER157W	YDR057W	907
YER157W	YDR189W	987
YER157W	YFL038C	995
YER157W	YGL005C	999
YER157W	YGL038C	919
YER157W	YGL223C	999
YER157W	YGR120C	999
YER157W	YKL196C	973
YER157W	YLR268W	972
YER157W	YML071C	999
YER157W	YNL041C	999
YER157W	YNL051W	999
YER157W	YOR216C	997
YER157W	YPR105C	999
YER159C	YDR397C	999
YER159C	YER148W	999
YER159C	YNL236W	982
YER159C	YOR046C	960
YER159C	YPL082C	960
YER160C	YER138C	909
YER161C	YAL005C	976
YER161C	YBR215W	908
YER161C	YCL030C	985
YER161C	YER032W	924
YER161C	YGL201C	980
YER161C	YGR252W	968
YER161C	YHR166C	976
YER161C	YLL024C	976
YER161C	YLR418C	980
YER161C	YPL016W	991
YER162C	YAR007C	988
YER162C	YBR114W	997
YER162C	YCR066W	942

YER162C	YDR311W	978
YER162C	YDR460W	978
YER162C	YEL037C	999
YER162C	YER171W	997
YER162C	YGL058W	944
YER162C	YGR258C	997
YER162C	YIL128W	942
YER162C	YIL143C	991
YER162C	YJR052W	999
YER162C	YKL114C	921
YER162C	YLR005W	978
YER162C	YML011C	960
YER162C	YML095C	997
YER162C	YMR201C	999
YER162C	YNL312W	991
YER162C	YPL022W	997
YER164W	YBR081C	991
YER164W	YBR198C	980
YER164W	YBR245C	997
YER164W	YCL010C	980
YER164W	YDR145W	980
YER164W	YDR167W	980
YER164W	YDR176W	980
YER164W	YDR392W	983
YER164W	YDR448W	993
YER164W	YGL019W	999
YER164W	YGL112C	980
YER164W	YGL207W	999
YER164W	YGL244W	990
YER164W	YGR252W	996
YER164W	YHR099W	980
YER164W	YHR119W	924
YER164W	YIL035C	999
YER164W	YJL168C	986
YER164W	YLR055C	924
YER164W	YML010W	908
YER164W	YML069W	999
YER164W	YMR172W	968
YER164W	YMR223W	980
YER164W	YMR236W	980
YER164W	YOL148C	995
YER164W	YOR039W	999
YER164W	YOR061W	999
YER164W	YOR304W	994

YER164W	YPL254W	980
YER165W	YAL021C	951
YER165W	YAR002W	928
YER165W	YBR048W	915
YER165W	YBR079C	941
YER165W	YBR189W	937
YER165W	YCL054W	993
YER165W	YCR031C	946
YER165W	YCR077C	998
YER165W	YDL061C	931
YER165W	YDR025W	901
YER165W	YDR064W	955
YER165W	YDR172W	999
YER165W	YDR228C	917
YER165W	YDR381W	962
YER165W	YDR450W	901
YER165W	YEL034W	914
YER165W	YER025W	964
YER165W	YER032W	905
YER165W	YER049W	910
YER165W	YER102W	919
YER165W	YER131W	911
YER165W	YFL002C	901
YER165W	YGL049C	999
YER165W	YGL094C	915
YER165W	YGL120C	976
YER165W	YGL123W	962
YER165W	YGR027C	918
YER165W	YGR118W	921
YER165W	YGR159C	923
YER165W	YGR162W	999
YER165W	YGR178C	988
YER165W	YGR214W	955
YER165W	YGR218W	974
YER165W	YHL015W	931
YER165W	YHL034C	975
YER165W	YHR021C	908
YER165W	YHR203C	929
YER165W	YIR001C	908
YER165W	YJL033W	949
YER165W	YJL050W	931
YER165W	YJL080C	986
YER165W	YJL124C	944
YER165W	YJL136C	902

YER165W	YJL138C	903
YER165W	YJL189W	936
YER165W	YJL191W	936
YER165W	YJR007W	919
YER165W	YJR123W	954
YER165W	YJR145C	907
YER165W	YKL025C	939
YER165W	YKL068W	913
YER165W	YKL156W	925
YER165W	YKR057W	902
YER165W	YKR059W	925
YER165W	YLR002C	927
YER165W	YLR048W	974
YER165W	YLR167W	911
YER165W	YLR264W	918
YER165W	YLR367W	914
YER165W	YLR388W	938
YER165W	YLR441C	907
YER165W	YML024W	923
YER165W	YML026C	901
YER165W	YML063W	929
YER165W	YML117W	972
YER165W	YMR125W	992
YER165W	YMR229C	940
YER165W	YMR230W	902
YER165W	YMR260C	946
YER165W	YNL096C	903
YER165W	YNL178W	946
YER165W	YNL302C	901
YER165W	YNR052C	986
YER165W	YOL121C	901
YER165W	YOL139C	999
YER165W	YOL149W	988
YER165W	YOR001W	972
YER165W	YOR046C	920
YER165W	YOR096W	935
YER165W	YOR206W	933
YER165W	YOR293W	917
YER165W	YOR310C	939
YER165W	YOR361C	928
YER165W	YPL169C	941
YER165W	YPL190C	973
YER165W	YPL263C	940
YER165W	YPR132W	912

YER165W	YPR163C	919
YER166W	YAL026C	926
YER166W	YCR094W	970
YER166W	YDR093W	983
YER166W	YNL323W	997
YER167W	YAL040C	999
YER167W	YBL105C	980
YER167W	YCR017C	987
YER167W	YER111C	991
YER167W	YGR229C	982
YER167W	YLR182W	934
YER167W	YMR199W	970
YER167W	YOR083W	980
YER167W	YPL256C	970
YER168C	YKL205W	916
YER170W	YAL038W	905
YER170W	YJR105W	916
YER170W	YKL067W	933
YER170W	YML022W	903
YER170W	YOR347C	905
YER171W	YAR007C	989
YER171W	YBR114W	965
YER171W	YCR066W	955
YER171W	YDL108W	999
YER171W	YDL140C	917
YER171W	YDR311W	999
YER171W	YDR404C	925
YER171W	YDR460W	999
YER171W	YEL037C	929
YER171W	YER148W	904
YER171W	YER162C	997
YER171W	YFR009W	940
YER171W	YGL025C	913
YER171W	YGL043W	909
YER171W	YGL058W	959
YER171W	YGL070C	925
YER171W	YGL163C	913
YER171W	YGR258C	999
YER171W	YIL021W	921
YER171W	YIL128W	999
YER171W	YIL143C	999
YER171W	YJR035W	988
YER171W	YJR052W	958
YER171W	YKL058W	907

YER171W	YKL113C	919
YER171W	YLR005W	999
YER171W	YLR032W	906
YER171W	YML032C	999
YER171W	YML095C	997
YER171W	YMR201C	999
YER171W	YNL064C	934
YER171W	YNL236W	917
YER171W	YNL250W	921
YER171W	YNL312W	987
YER171W	YOL005C	902
YER171W	YOL051W	917
YER171W	YOL090W	969
YER171W	YOR123C	916
YER171W	YOR151C	908
YER171W	YOR210W	927
YER171W	YOR224C	921
YER171W	YOR341W	900
YER171W	YPL022W	997
YER171W	YPL122C	999
YER171W	YPL167C	984
YER171W	YPR025C	996
YER171W	YPR056W	996
YER171W	YPR070W	907
YER171W	YPR086W	922
YER171W	YPR110C	901
YER171W	YPR187W	925
YER172C	YAL032C	992
YER172C	YBL026W	999
YER172C	YBL074C	992
YER172C	YBR055C	999
YER172C	YBR065C	995
YER172C	YBR152W	989
YER172C	YCR063W	903
YER172C	YCR077C	976
YER172C	YDL030W	993
YER172C	YDL098C	999
YER172C	YDL209C	996
YER172C	YDR088C	924
YER172C	YDR243C	980
YER172C	YDR364C	980
YER172C	YDR378C	999
YER172C	YDR416W	992
YER172C	YDR473C	999

YER172C	YEL026W	999
YER172C	YER029C	999
YER172C	YER112W	999
YER172C	YER146W	997
YER172C	YFL017W-A	999
YER172C	YGL120C	995
YER172C	YGL128C	992
YER172C	YGR013W	994
YER172C	YGR074W	999
YER172C	YGR075C	999
YER172C	YGR091W	999
YER172C	YGR278W	992
YER172C	YHR156C	933
YER172C	YHR165C	999
YER172C	YIL061C	986
YER172C	YJL124C	976
YER172C	YJL203W	993
YER172C	YJR022W	999
YER172C	YKL173W	999
YER172C	YKR086W	967
YER172C	YLL036C	998
YER172C	YLR117C	998
YER172C	YLR147C	999
YER172C	YLR275W	999
YER172C	YLR424W	993
YER172C	YLR438C-A	998
YER172C	YML046W	994
YER172C	YML049C	999
YER172C	YMR125W	998
YER172C	YMR213W	996
YER172C	YMR240C	999
YER172C	YMR288W	999
YER172C	YNL147W	999
YER172C	YNR011C	940
YER172C	YOR159C	999
YER172C	YOR308C	999
YER172C	YOR319W	968
YER172C	YPL151C	996
YER172C	YPL213W	998
YER172C	YPR082C	999
YER172C	YPR101W	997
YER172C	YPR178W	999
YER172C	YPR182W	999
YER173W	YBL088C	989

YER173W	YBR087W	999
YER173W	YBR136W	946
YER173W	YCL061C	963
YER173W	YDL164C	967
YER173W	YDL220C	997
YER173W	YDR004W	983
YER173W	YDR113C	933
YER173W	YDR217C	999
YER173W	YER095W	998
YER173W	YER179W	909
YER173W	YHR154W	970
YER173W	YJL115W	976
YER173W	YJR043C	975
YER173W	YJR068W	999
YER173W	YKL113C	999
YER173W	YLR288C	946
YER173W	YML032C	937
YER173W	YMR078C	999
YER173W	YMR190C	999
YER173W	YNL250W	901
YER173W	YNL290W	999
YER173W	YOL094C	999
YER173W	YOR144C	999
YER173W	YOR217W	999
YER173W	YPL022W	911
YER173W	YPL153C	986
YER173W	YPL167C	920
YER173W	YPL194W	999
YER174C	YGL071W	998
YER174C	YGL220W	966
YER174C	YGR262C	998
YER176W	YDR172W	967
YER177W	YBL103C	954
YER177W	YDL220C	936
YER177W	YDR001C	999
YER177W	YDR028C	999
YER177W	YDR062W	966
YER177W	YDR099W	999
YER177W	YDR130C	985
YER177W	YDR164C	948
YER177W	YDR373W	977
YER177W	YDR477W	990
YER177W	YEL061C	943
YER177W	YER016W	962

YER177W	YER125W	939
YER177W	YER133W	998
YER177W	YFL039C	905
YER177W	YFR017C	911
YER177W	YGL003C	992
YER177W	YGL078C	940
YER177W	YGL115W	956
YER177W	YGL206C	930
YER177W	YGL252C	997
YER177W	YHL007C	937
YER177W	YJL141C	993
YER177W	YJL164C	985
YER177W	YKL168C	928
YER177W	YLR028C	989
YER177W	YLR177W	940
YER177W	YLR310C	975
YER177W	YNL076W	994
YER177W	YNL236W	909
YER177W	YNL267W	995
YER177W	YOR244W	939
YER177W	YPL267W	997
YER178W	YAL038W	908
YER178W	YBR218C	907
YER178W	YBR221C	999
YER178W	YDR430C	987
YER178W	YFL018C	998
YER178W	YGL062W	907
YER178W	YGR193C	990
YER178W	YGR229C	955
YER178W	YIL042C	984
YER178W	YLR044C	931
YER178W	YLR089C	916
YER178W	YMR308C	924
YER178W	YNL071W	999
YER178W	YOR090C	923
YER178W	YOR347C	905
YER178W	YPL017C	958
YER179W	YBR073W	996
YER179W	YBR186W	973
YER179W	YDR014W-A	932
YER179W	YDR285W	963
YER179W	YDR440W	946
YER179W	YER095W	997
YER179W	YER173W	909

YER179W	YGL033W	982
YER179W	YGL163C	997
YER179W	YGL183C	956
YER179W	YHL022C	997
YER179W	YHR124W	937
YER179W	YIL072W	972
YER179W	YLR263W	955
YER179W	YML032C	977
YER179W	YOR351C	962
YER179W	YOR368W	996
YER179W	YPL121C	942
YER180C	YGL026C	904
YER183C	YBR084W	910
YER183C	YGR204W	981
YER189W	YEL075C	944
YER189W	YFL064C	901
YFL002C	YDL031W	996
YFL002C	YDR060W	976
YFL002C	YDR087C	974
YFL002C	YDR496C	994
YFL002C	YER006W	993
YFL002C	YER165W	901
YFL002C	YFR001W	992
YFL002C	YGL111W	976
YFL002C	YGR103W	981
YFL002C	YHR052W	997
YFL002C	YHR088W	993
YFL002C	YHR197W	996
YFL002C	YKL009W	978
YFL002C	YKL172W	976
YFL002C	YKR081C	974
YFL002C	YLL008W	980
YFL002C	YLL034C	974
YFL002C	YLR002C	994
YFL002C	YLR449W	992
YFL002C	YMR049C	995
YFL002C	YMR290C	987
YFL002C	YNL002C	993
YFL002C	YNL061W	999
YFL002C	YNL110C	997
YFL002C	YOL077C	992
YFL002C	YOL127W	941
YFL002C	YOR063W	942
YFL002C	YOR206W	994

YFL002C	YOR272W	993
YFL002C	YPL093W	989
YFL002C	YPL131W	959
YFL002C	YPL211W	993
YFL002C	YPR016C	976
YFL003C	YDL154W	957
YFL003C	YLR035C	920
YFL003C	YLR394W	924
YFL003C	YMR167W	974
YFL003C	YNL082W	926
YFL003C	YPL164C	966
YFL005W	YDR164C	996
YFL005W	YDR166C	998
YFL005W	YER008C	997
YFL005W	YER136W	999
YFL005W	YFL038C	974
YFL005W	YGL106W	963
YFL005W	YGL233W	999
YFL005W	YGR009C	999
YFL005W	YGR172C	970
YFL005W	YIL118W	996
YFL005W	YJL085W	995
YFL005W	YKL079W	991
YFL005W	YLR166C	991
YFL005W	YLR229C	941
YFL005W	YNL263C	979
YFL005W	YNL272C	999
YFL005W	YNL293W	997
YFL005W	YNR049C	984
YFL005W	YOL112W	931
YFL005W	YOR070C	948
YFL005W	YOR326W	999
YFL005W	YOR370C	929
YFL005W	YPR017C	999
YFL005W	YPR032W	988
YFL005W	YPR055W	978
YFL007W	YBL041W	991
YFL007W	YBR173C	916
YFL007W	YDL147W	971
YFL007W	YDR394W	956
YFL007W	YDR427W	969
YFL007W	YER012W	992
YFL007W	YER094C	999
YFL007W	YFR004W	973

YFL007W	YFR050C	999
YFL007W	YGL011C	999
YFL007W	YGL048C	991
YFL007W	YGR135W	990
YFL007W	YGR232W	973
YFL007W	YGR253C	996
YFL007W	YHL030W	991
YFL007W	YIL075C	940
YFL007W	YJL001W	999
YFL007W	YKL168C	984
YFL007W	YLR421C	970
YFL007W	YML092C	999
YFL007W	YMR314W	992
YFL007W	YOL038W	998
YFL007W	YOR117W	942
YFL007W	YOR259C	973
YFL007W	YOR261C	973
YFL007W	YOR362C	991
YFL007W	YPR103W	998
YFL008W	YCR086W	927
YFL008W	YDL003W	999
YFL008W	YDR014W	936
YFL008W	YDR180W	989
YFL008W	YFR031C	967
YFL008W	YGR098C	900
YFL008W	YIL026C	999
YFL008W	YIL144W	903
YFL008W	YJL074C	999
YFL008W	YMR001C	993
YFL008W	YMR076C	959
YFL008W	YNL262W	964
YFL008W	YPR007C	984
YFL009W	YBR083W	956
YFL009W	YDL132W	999
YFL009W	YDL155W	962
YFL009W	YDR054C	999
YFL009W	YDR328C	999
YFL009W	YEL009C	976
YFL009W	YFL031W	942
YFL009W	YGR049W	935
YFL009W	YHR052W	978
YFL009W	YHR206W	926
YFL009W	YIL046W	986
YFL009W	YJL194W	999

YFL009W	YJR090C	932
YFL009W	YLR079W	999
YFL009W	YOL133W	999
YFL009W	YOR080W	975
YFL009W	YPL003W	905
YFL009W	YPL256C	981
YFL009W	YPR120C	939
YFL010C	YLL039C	911
YFL011W	YPL240C	970
YFL012W	YHR114W	998
YFL012W	YPL111W	998
YFL013C	YDL002C	999
YFL013C	YDR190C	994
YFL013C	YER092W	998
YFL013C	YFL039C	996
YFL013C	YGL150C	999
YFL013C	YJL081C	997
YFL013C	YLR052W	984
YFL013C	YNL059C	996
YFL013C	YNL215W	998
YFL013C	YOR141C	998
YFL013C	YPL129W	990
YFL013C	YPL235W	991
YFL016C	YGL018C	951
YFL016C	YIL157C	963
YFL016C	YJR045C	999
YFL016C	YNL328C	991
YFL016C	YOR232W	995
YFL017C	YDL103C	947
YFL017C	YDR429C	922
YFL017C	YEL058W	994
YFL017C	YKL104C	988
YFL017C	YOR362C	943
YFL017W-A	YBL026W	991
YFL017W-A	YBR055C	997
YFL017W-A	YBR119W	994
YFL017W-A	YBR154C	947
YFL017W-A	YDL030W	982
YFL017W-A	YDL087C	983
YFL017W-A	YDR240C	990
YFL017W-A	YDR473C	961
YFL017W-A	YEL026W	943
YFL017W-A	YER029C	999
YFL017W-A	YER112W	987

YFL017W-A	YER172C	999
YFL017W-A	YGL070C	958
YFL017W-A	YGR013W	987
YFL017W-A	YGR074W	999
YFL017W-A	YGR091W	929
YFL017W-A	YHR165C	986
YFL017W-A	YIL061C	990
YFL017W-A	YJL124C	904
YFL017W-A	YJL203W	918
YFL017W-A	YJR084W	922
YFL017W-A	YKL012W	984
YFL017W-A	YKL173W	996
YFL017W-A	YLL036C	961
YFL017W-A	YLR147C	999
YFL017W-A	YLR275W	999
YFL017W-A	YLR298C	992
YFL017W-A	YML049C	987
YFL017W-A	YMR125W	994
YFL017W-A	YMR240C	998
YFL017W-A	YMR288W	990
YFL017W-A	YOR151C	902
YFL017W-A	YOR159C	999
YFL017W-A	YOR210W	971
YFL017W-A	YOR224C	935
YFL017W-A	YOR308C	939
YFL017W-A	YPL178W	905
YFL017W-A	YPL213W	985
YFL017W-A	YPR082C	985
YFL017W-A	YPR182W	998
YFL017W-A	YPR187W	905
YFL018C	YAL044C	988
YFL018C	YBR221C	999
YFL018C	YDR019C	993
YFL018C	YDR148C	999
YFL018C	YDR430C	985
YFL018C	YER178W	998
YFL018C	YFR049W	994
YFL018C	YGR193C	999
YFL018C	YIL125W	999
YFL018C	YKR080W	905
YFL018C	YMR189W	998
YFL018C	YNL071W	999
YFL021W	YDR099W	937
YFL021W	YER040W	999

YFL021W	YIL008W	900
YFL021W	YIR032C	904
YFL021W	YJR066W	961
YFL021W	YMR186W	919
YFL021W	YNL229C	995
YFL021W	YPL240C	919
YFL022C	YIL116W	900
YFL022C	YLR060W	999
YFL022C	YNL316C	920
YFL023W	YBR154C	990
YFL023W	YDR096W	929
YFL023W	YDR227W	900
YFL023W	YER133W	975
YFL023W	YLR200W	999
YFL023W	YMR190C	900
YFL023W	YMR219W	928
YFL024C	YBR009C	979
YFL024C	YDR334W	949
YFL024C	YDR359C	999
YFL024C	YEL018W	999
YFL024C	YFL039C	999
YFL024C	YGR002C	999
YFL024C	YHR090C	999
YFL024C	YHR099W	999
YFL024C	YJL081C	999
YFL024C	YJR082C	994
YFL024C	YML007W	991
YFL024C	YNL030W	956
YFL024C	YNL107W	999
YFL024C	YNL136W	998
YFL024C	YOR244W	999
YFL024C	YPR023C	999
YFL025C	YAL058W	901
YFL025C	YBR229C	913
YFL025C	YDR331W	942
YFL025C	YJL024C	913
YFL025C	YJL062W	921
YFL025C	YMR010W	911
YFL025C	YMR307W	911
YFL025C	YPL227C	903
YFL026W	YBL016W	914
YFL026W	YDL159W	966
YFL026W	YDR085C	924
YFL026W	YDR103W	994

YFL026W	YGL089C	995
YFL026W	YHL007C	909
YFL026W	YHR005C	999
YFL026W	YHR084W	999
YFL026W	YIL015W	986
YFL026W	YJL192C	909
YFL026W	YJR086W	968
YFL026W	YKL178C	995
YFL026W	YKL209C	999
YFL026W	YLR362W	962
YFL026W	YLR452C	999
YFL026W	YNL276C	998
YFL026W	YOR212W	999
YFL026W	YPL187W	995
YFL027C	YFL038C	910
YFL028C	YAL021C	920
YFL028C	YDR443C	914
YFL028C	YER043C	999
YFL029C	YBR160W	999
YFL029C	YBR279W	963
YFL029C	YDL102W	941
YFL029C	YDL108W	996
YFL029C	YDL125C	933
YFL029C	YKL139W	921
YFL029C	YNL222W	966
YFL029C	YOL145C	966
YFL029C	YPR054W	986
YFL029C	YPR119W	934
YFL030W	YBR263W	983
YFL030W	YLR058C	983
YFL030W	YML054C	901
YFL031W	YDR123C	938
YFL031W	YER120W	972
YFL031W	YFL009W	942
YFL031W	YGR227W	903
YFL031W	YGR252W	930
YFL031W	YHR079C	995
YFL031W	YJL034W	956
YFL031W	YKL073W	903
YFL031W	YLR133W	922
YFL031W	YMR022W	908
YFL033C	YDR096W	954
YFL033C	YGL073W	950
YFL033C	YGL248W	900

YFL033C	YHR205W	999
YFL033C	YIL033C	960
YFL033C	YIL038C	906
YFL033C	YJL164C	902
YFL033C	YKL062W	997
YFL033C	YMR037C	997
YFL033C	YNL098C	971
YFL033C	YOL001W	900
YFL033C	YPL031C	987
YFL034C-A	YBL027W	916
YFL034C-A	YBL072C	913
YFL034C-A	YBL087C	910
YFL034C-A	YBL092W	917
YFL034C-A	YBR031W	925
YFL034C-A	YBR084C-A	916
YFL034C-A	YBR118W	902
YFL034C-A	YBR181C	900
YFL034C-A	YBR189W	906
YFL034C-A	YBR191W	914
YFL034C-A	YCR031C	919
YFL034C-A	YDL061C	911
YFL034C-A	YDL075W	918
YFL034C-A	YDL081C	917
YFL034C-A	YDL082W	912
YFL034C-A	YDL130W	911
YFL034C-A	YDL136W	912
YFL034C-A	YDL191W	912
YFL034C-A	YDR012W	925
YFL034C-A	YDR064W	928
YFL034C-A	YDR382W	924
YFL034C-A	YDR385W	909
YFL034C-A	YDR418W	920
YFL034C-A	YDR447C	911
YFL034C-A	YDR450W	912
YFL034C-A	YDR471W	911
YFL034C-A	YDR500C	911
YFL034C-A	YEL054C	920
YFL034C-A	YER056C-A	911
YFL034C-A	YER074W	904
YFL034C-A	YER102W	913
YFL034C-A	YER117W	910
YFL034C-A	YER131W	912
YFL034C-A	YFR032C-A	920
YFL034C-A	YGL030W	919

YFL034C-A	YGL076C	920
YFL034C-A	YGL135W	920
YFL034C-A	YGL147C	911
YFL034C-A	YGL189C	912
YFL034C-A	YGR034W	920
YFL034C-A	YGR085C	913
YFL034C-A	YGR118W	913
YFL034C-A	YGR214W	904
YFL034C-A	YHL001W	915
YFL034C-A	YHL015W	919
YFL034C-A	YHL033C	921
YFL034C-A	YHR010W	911
YFL034C-A	YHR021C	915
YFL034C-A	YHR141C	926
YFL034C-A	YHR203C	927
YFL034C-A	YIL052C	911
YFL034C-A	YIL069C	904
YFL034C-A	YIL133C	925
YFL034C-A	YIL148W	904
YFL034C-A	YJL136C	920
YFL034C-A	YJL177W	913
YFL034C-A	YJL189W	925
YFL034C-A	YJL190C	907
YFL034C-A	YJL191W	919
YFL034C-A	YJR094W-A	909
YFL034C-A	YJR123W	910
YFL034C-A	YJR145C	927
YFL034C-A	YKL006W	916
YFL034C-A	YKL156W	928
YFL034C-A	YKL180W	913
YFL034C-A	YKR057W	920
YFL034C-A	YKR094C	904
YFL034C-A	YLL045C	921
YFL034C-A	YLR029C	909
YFL034C-A	YLR048W	904
YFL034C-A	YLR061W	905
YFL034C-A	YLR075W	900
YFL034C-A	YLR185W	911
YFL034C-A	YLR264W	917
YFL034C-A	YLR287C-A	906
YFL034C-A	YLR340W	920
YFL034C-A	YLR344W	920
YFL034C-A	YLR367W	907
YFL034C-A	YLR388W	912

YFL034C-A	YLR406C	918
YFL034C-A	YLR441C	918
YFL034C-A	YLR448W	914
YFL034C-A	YML024W	911
YFL034C-A	YML026C	912
YFL034C-A	YML063W	918
YFL034C-A	YML073C	913
YFL034C-A	YMR121C	909
YFL034C-A	YMR142C	911
YFL034C-A	YMR194W	926
YFL034C-A	YMR230W	917
YFL034C-A	YMR242C	914
YFL034C-A	YMR260C	912
YFL034C-A	YNL067W	911
YFL034C-A	YNL069C	924
YFL034C-A	YNL096C	921
YFL034C-A	YNL162W	926
YFL034C-A	YNL178W	928
YFL034C-A	YNL301C	903
YFL034C-A	YNL302C	915
YFL034C-A	YOL040C	910
YFL034C-A	YOL120C	903
YFL034C-A	YOL121C	915
YFL034C-A	YOL127W	921
YFL034C-A	YOR063W	901
YFL034C-A	YOR096W	921
YFL034C-A	YOR133W	909
YFL034C-A	YOR167C	907
YFL034C-A	YOR182C	906
YFL034C-A	YOR234C	925
YFL034C-A	YOR293W	926
YFL034C-A	YOR312C	914
YFL034C-A	YOR369C	918
YFL034C-A	YPL079W	913
YFL034C-A	YPL081W	906
YFL034C-A	YPL090C	900
YFL034C-A	YPL131W	906
YFL034C-A	YPL143W	914
YFL034C-A	YPL198W	920
YFL034C-A	YPL220W	920
YFL034C-A	YPL249C-A	919
YFL034C-A	YPR043W	909
YFL034C-A	YPR080W	902
YFL034C-A	YPR102C	913

YFL034C-A	YPR132W	913
YFL034C-B	YHR102W	956
YFL034C-B	YIL129C	956
YFL034C-B	YLR131C	967
YFL034C-B	YNL161W	999
YFL034W	YHL019C	989
YFL036W	YDL044C	930
YFL036W	YMR228W	999
YFL037W	YBL034C	997
YFL037W	YDL143W	910
YFL037W	YDR188W	910
YFL037W	YDR212W	989
YFL037W	YDR356W	909
YFL037W	YEL003W	960
YFL037W	YEL061C	945
YFL037W	YER007W	995
YFL037W	YGR078C	946
YFL037W	YGR218W	958
YFL037W	YIL142W	911
YFL037W	YJL008C	958
YFL037W	YJL013C	924
YFL037W	YJL030W	942
YFL037W	YJL042W	904
YFL037W	YJL111W	915
YFL037W	YJR091C	982
YFL037W	YKR054C	941
YFL037W	YLR045C	997
YFL037W	YLR200W	982
YFL037W	YML085C	999
YFL037W	YML094W	960
YFL037W	YML124C	931
YFL037W	YMR138W	989
YFL037W	YNL148C	977
YFL037W	YNL223W	983
YFL037W	YOR265W	999
YFL037W	YOR349W	999
YFL037W	YPL241C	903
YFL037W	YPR141C	920
YFL038C	YDL058W	998
YFL038C	YDL192W	975
YFL038C	YDR051C	956
YFL038C	YDR170C	978
YFL038C	YDR189W	999
YFL038C	YDR233C	956

YFL038C	YDR246W	985
YFL038C	YDR472W	933
YFL038C	YER008C	941
YFL038C	YER031C	979
YFL038C	YER136W	999
YFL038C	YER157W	995
YFL038C	YFL005W	974
YFL038C	YFL027C	910
YFL038C	YGL198W	900
YFL038C	YGR120C	988
YFL038C	YGR172C	999
YFL038C	YIL004C	999
YFL038C	YKR068C	999
YFL038C	YLR026C	999
YFL038C	YLR078C	991
YFL038C	YLR268W	999
YFL038C	YML077W	998
YFL038C	YMR218C	932
YFL038C	YNL263C	987
YFL038C	YNL287W	908
YFL038C	YOR070C	999
YFL038C	YOR307C	985
YFL038C	YOR370C	999
YFL038C	YPL249C	932
YFL038C	YPR017C	999
YFL038C	YPR176C	999
YFL039C	YAL011W	961
YFL039C	YAL029C	999
YFL039C	YBR109C	994
YFL039C	YBR130C	942
YFL039C	YBR200W	996
YFL039C	YBR231C	999
YFL039C	YBR245C	974
YFL039C	YCR009C	998
YFL039C	YCR088W	999
YFL039C	YDL002C	998
YFL039C	YDL143W	984
YFL039C	YDL178W	999
YFL039C	YDL226C	908
YFL039C	YDR099W	903
YFL039C	YDR129C	999
YFL039C	YDR159W	976
YFL039C	YDR190C	999
YFL039C	YDR211W	940

YFL039C	YDR212W	984
YFL039C	YDR334W	999
YFL039C	YDR359C	991
YFL039C	YDR388W	999
YFL039C	YDR389W	968
YFL039C	YDR484W	991
YFL039C	YDR485C	961
YFL039C	YDR510W	977
YFL039C	YEL003W	963
YFL039C	YEL013W	912
YFL039C	YEL018W	999
YFL039C	YER093C	911
YFL039C	YER125W	908
YFL039C	YER155C	972
YFL039C	YER177W	905
YFL039C	YFL013C	996
YFL039C	YFL024C	999
YFL039C	YGL106W	993
YFL039C	YGL150C	999
YFL039C	YGR002C	999
YFL039C	YGR078C	966
YFL039C	YGR080W	996
YFL039C	YHL007C	975
YFL039C	YHR023W	994
YFL039C	YHR090C	994
YFL039C	YHR179W	968
YFL039C	YIR006C	986
YFL039C	YJL005W	982
YFL039C	YJL008C	916
YFL039C	YJL081C	999
YFL039C	YJL111W	922
YFL039C	YJL179W	963
YFL039C	YKL007W	975
YFL039C	YKL129C	959
YFL039C	YKL203C	908
YFL039C	YKL212W	991
YFL039C	YLL050C	999
YFL039C	YLR052W	996
YFL039C	YLR085C	967
YFL039C	YLR200W	967
YFL039C	YLR229C	937
YFL039C	YLR319C	999
YFL039C	YLR337C	999
YFL039C	YLR385C	961

YFL039C	YLR429W	998
YFL039C	YML041C	999
YFL039C	YML094W	970
YFL039C	YMR032W	934
YFL039C	YMR072W	978
YFL039C	YMR092C	999
YFL039C	YMR109W	974
YFL039C	YNL059C	991
YFL039C	YNL079C	997
YFL039C	YNL107W	999
YFL039C	YNL136W	987
YFL039C	YNL138W	999
YFL039C	YNL153C	963
YFL039C	YNL189W	909
YFL039C	YNL243W	999
YFL039C	YNL267W	940
YFL039C	YNL271C	999
YFL039C	YOL012C	997
YFL039C	YOR035C	941
YFL039C	YOR122C	999
YFL039C	YOR141C	998
YFL039C	YOR181W	999
YFL039C	YOR244W	999
YFL039C	YOR326W	999
YFL039C	YOR367W	998
YFL039C	YPL129W	999
YFL039C	YPL235W	999
YFL039C	YPL240C	902
YFL039C	YPL242C	989
YFL039C	YPR023C	969
YFL039C	YPR171W	972
YFL041W	YBR207W	996
YFL041W	YLR034C	944
YFL041W	YMR058W	999
YFL041W	YMR319C	914
YFL042C	YMR303C	904
YFL044C	YBL058W	988
YFL044C	YBR170C	930
YFL044C	YDL126C	993
YFL044C	YDL190C	988
YFL044C	YGR048W	930
YFL044C	YKL213C	930
YFL045C	YDL055C	973
YFL045C	YER003C	986

YFL045C	YGL167C	949
YFL045C	YGL195W	962
YFL046W	YGR284C	998
YFL047W	YNL180C	948
YFL048C	YDL145C	976
YFL048C	YEL036C	907
YFL048C	YGL137W	919
YFL048C	YIL053W	935
YFL048C	YLR080W	999
YFL048C	YLR250W	998
YFL048C	YNL287W	976
YFL049W	YBR289W	999
YFL049W	YDR073W	956
YFL049W	YHL025W	999
YFL049W	YJL176C	999
YFL049W	YMR033W	999
YFL049W	YNR023W	999
YFL049W	YOR290C	999
YFL049W	YPL016W	999
YFL049W	YPR034W	999
YFL050C	YOL130W	977
YFL052W	YKL157W	980
YFL053W	YML070W	976
YFL054C	YHL032C	954
YFL054C	YPL240C	919
YFL059W	YFL060C	994
YFL059W	YHR029C	935
YFL059W	YMR095C	999
YFL059W	YMR096W	999
YFL059W	YNL333W	999
YFL059W	YNL334C	997
YFL060C	YBR035C	908
YFL060C	YFL059W	994
YFL060C	YMR095C	993
YFL060C	YMR096W	997
YFL060C	YNL333W	993
YFL060C	YNL334C	957
YFL062W	YGR295C	994
YFL064C	YER189W	901
YFL064C	YFL065C	906
YFL065C	YFL064C	906
YFR001W	YDL031W	987
YFR001W	YER006W	987
YFR001W	YFL002C	992

YFR001W	YGR103W	953
YFR001W	YHR052W	993
YFR001W	YKL172W	969
YFR001W	YKL185W	933
YFR001W	YLL008W	992
YFR001W	YLL045C	981
YFR001W	YLR002C	992
YFR001W	YLR449W	923
YFR001W	YMR290C	990
YFR001W	YNL002C	990
YFR001W	YNL061W	999
YFR001W	YOL077C	987
YFR001W	YOR063W	982
YFR001W	YOR206W	932
YFR001W	YPL093W	984
YFR001W	YPR016C	999
YFR002W	YAR002W	936
YFR002W	YBL079W	994
YFR002W	YDL088C	975
YFR002W	YDL116W	970
YFR002W	YDL207W	972
YFR002W	YDR159W	966
YFR002W	YER105C	992
YFR002W	YER107C	999
YFR002W	YER110C	965
YFR002W	YGL092W	984
YFR002W	YGL100W	938
YFR002W	YGL172W	999
YFR002W	YGR119C	999
YFR002W	YIL115C	958
YFR002W	YJL039C	998
YFR002W	YJL041W	999
YFR002W	YJL061W	990
YFR002W	YJR042W	930
YFR002W	YKL057C	991
YFR002W	YKL068W	995
YFR002W	YKR082W	929
YFR002W	YLR018C	940
YFR002W	YLR335W	963
YFR002W	YLR347C	998
YFR002W	YML031W	990
YFR002W	YML103C	999
YFR002W	YMR047C	999
YFR002W	YMR129W	999

YFR002W	YMR153W	999
YFR002W	YMR308C	915
YFR002W	YOR098C	926
YFR002W	YOR257W	961
YFR002W	YPL169C	972
YFR003C	YER133W	999
YFR003C	YKL193C	996
YFR003C	YML016C	998
YFR003C	YPL209C	995
YFR004W	YBL039C	939
YFR004W	YBL041W	972
YFR004W	YCR014C	970
YFR004W	YDL007W	999
YFR004W	YDL013W	970
YFR004W	YDL097C	999
YFR004W	YDL147W	999
YFR004W	YDR363W-A	999
YFR004W	YDR394W	999
YFR004W	YDR427W	999
YFR004W	YER012W	999
YFR004W	YER021W	999
YFR004W	YER094C	939
YFR004W	YFL007W	973
YFR004W	YFR010W	999
YFR004W	YFR050C	969
YFR004W	YFR052W	999
YFR004W	YGL004C	955
YFR004W	YGL011C	927
YFR004W	YGL048C	999
YFR004W	YGL141W	954
YFR004W	YGR135W	935
YFR004W	YGR232W	998
YFR004W	YGR253C	955
YFR004W	YHL030W	999
YFR004W	YHR027C	999
YFR004W	YHR200W	999
YFR004W	YIL075C	999
YFR004W	YIL148W	900
YFR004W	YIR011C	928
YFR004W	YJL001W	922
YFR004W	YKL145W	999
YFR004W	YKR094C	900
YFR004W	YLL039C	919
YFR004W	YLR421C	998

YFR004W	YML092C	928
YFR004W	YMR191W	987
YFR004W	YMR314W	998
YFR004W	YOL038W	940
YFR004W	YOR117W	999
YFR004W	YOR157C	938
YFR004W	YOR259C	999
YFR004W	YOR261C	999
YFR004W	YOR362C	956
YFR004W	YPR103W	960
YFR004W	YPR108W	999
YFR005C	YBR135W	901
YFR006W	YMR176W	907
YFR008W	YDR200C	959
YFR008W	YMR029C	981
YFR008W	YMR052W	998
YFR009W	YDL052C	963
YFR009W	YDL185W	962
YFR009W	YDR188W	968
YFR009W	YDR283C	994
YFR009W	YER171W	940
YFR009W	YGL195W	999
YFR009W	YKL029C	972
YFR009W	YKL104C	966
YFR009W	YLR180W	973
YFR010W	YAR003W	954
YFR010W	YBR082C	906
YFR010W	YDL007W	999
YFR010W	YDL074C	901
YFR010W	YDL097C	999
YFR010W	YDL147W	999
YFR010W	YDR363W-A	997
YFR010W	YDR394W	999
YFR010W	YDR427W	999
YFR010W	YER012W	999
YFR010W	YER021W	999
YFR010W	YFR004W	999
YFR010W	YFR052W	994
YFR010W	YGL048C	999
YFR010W	YGL141W	996
YFR010W	YGR232W	992
YFR010W	YHL030W	999
YFR010W	YHR027C	999
YFR010W	YHR200W	999

YFR010W	YIL075C	999
YFR010W	YKL145W	999
YFR010W	YLL039C	994
YFR010W	YLR421C	992
YFR010W	YMR314W	985
YFR010W	YOR117W	988
YFR010W	YOR259C	999
YFR010W	YOR261C	999
YFR010W	YPL240C	934
YFR010W	YPR108W	999
YFR011C	YNL113W	934
YFR013W	YBR089C-A	987
YFR013W	YBR245C	999
YFR013W	YGL133W	990
YFR013W	YLR095C	913
YFR013W	YOL004W	954
YFR013W	YOR304W	912
YFR013W	YPL082C	988
YFR014C	YBR109C	999
YFR014C	YOL016C	963
YFR014C	YPL058C	909
YFR015C	YEL011W	996
YFR015C	YHL012W	901
YFR015C	YJL137C	997
YFR015C	YKL035W	922
YFR015C	YKR058W	995
YFR015C	YLR258W	999
YFR015C	YPR160W	949
YFR017C	YER177W	911
YFR019W	YFR021W	968
YFR019W	YKL212W	993
YFR019W	YLR240W	943
YFR019W	YLR386W	990
YFR019W	YNL054W	914
YFR019W	YNL106C	945
YFR019W	YNL325C	991
YFR019W	YOR109W	956
YFR021W	YBL103C	989
YFR021W	YCL063W	992
YFR021W	YDL149W	998
YFR021W	YFR019W	968
YFR021W	YFR040W	967
YFR021W	YLR386W	970
YFR021W	YNL242W	999

YFR021W	YNL325C	902
YFR022W	YER125W	993
YFR024C-A	YBL007C	999
YFR024C-A	YCR088W	903
YFR024C-A	YGL181W	969
YFR024C-A	YGR268C	981
YFR024C-A	YHR016C	929
YFR024C-A	YHR114W	999
YFR024C-A	YLR144C	903
YFR024C-A	YNL094W	937
YFR024C-A	YNL243W	994
YFR024C-A	YOR042W	997
YFR024C-A	YOR181W	999
YFR024C-A	YPR171W	977
YFR027W	YBR088C	955
YFR027W	YDL003W	948
YFR027W	YDR014W	937
YFR027W	YDR419W	961
YFR027W	YIL026C	907
YFR027W	YJL019W	979
YFR027W	YJL074C	955
YFR027W	YMR076C	991
YFR027W	YMR078C	987
YFR027W	YPL008W	977
YFR028C	YAL024C	999
YFR028C	YAR019C	999
YFR028C	YBR060C	999
YFR028C	YBR156C	998
YFR028C	YBR160W	980
YFR028C	YDL042C	999
YFR028C	YDR110W	990
YFR028C	YDR146C	998
YFR028C	YER082C	912
YFR028C	YGL003C	998
YFR028C	YGR092W	999
YFR028C	YGR098C	966
YFR028C	YHR152W	944
YFR028C	YJL076W	999
YFR028C	YJL194W	999
YFR028C	YJR053W	939
YFR028C	YJR063W	974
YFR028C	YKL108W	906
YFR028C	YKR010C	996
YFR028C	YLR079W	999

YFR028C	YLR182W	983
YFR028C	YML064C	999
YFR028C	YMR001C	999
YFR028C	YMR055C	999
YFR028C	YNL261W	998
YFR028C	YOR195W	947
YFR028C	YOR340C	942
YFR028C	YOR341W	905
YFR028C	YPR110C	903
YFR028C	YPR119W	988
YFR029W	YCL018W	939
YFR029W	YDR160W	997
YFR029W	YJL156C	999
YFR029W	YKR039W	954
YFR030W	YJR137C	998
YFR030W	YPR167C	970
YFR031C	YBL097W	999
YFR031C	YDR325W	999
YFR031C	YFL008W	967
YFR031C	YIL031W	903
YFR031C	YIL144W	902
YFR031C	YKL068W	913
YFR031C	YLR086W	999
YFR031C	YLR272C	999
YFR031C-A	YAL035W	997
YFR031C-A	YBL027W	999
YFR031C-A	YBL072C	994
YFR031C-A	YBL087C	999
YFR031C-A	YBL092W	999
YFR031C-A	YBR031W	999
YFR031C-A	YBR048W	998
YFR031C-A	YBR084C-A	973
YFR031C-A	YBR084W	993
YFR031C-A	YBR118W	905
YFR031C-A	YBR181C	997
YFR031C-A	YBR189W	918
YFR031C-A	YBR191W	999
YFR031C-A	YCR031C	996
YFR031C-A	YDL061C	935
YFR031C-A	YDL075W	999
YFR031C-A	YDL081C	971
YFR031C-A	YDL082W	998
YFR031C-A	YDL083C	961
YFR031C-A	YDL136W	999

YFR031C-A	YDL191W	911
YFR031C-A	YDR012W	999
YFR031C-A	YDR025W	998
YFR031C-A	YDR041W	961
YFR031C-A	YDR064W	996
YFR031C-A	YDR382W	995
YFR031C-A	YDR385W	913
YFR031C-A	YDR418W	999
YFR031C-A	YDR447C	997
YFR031C-A	YDR450W	924
YFR031C-A	YDR471W	995
YFR031C-A	YEL054C	920
YFR031C-A	YER056C-A	903
YFR031C-A	YER074W	995
YFR031C-A	YER102W	915
YFR031C-A	YER117W	981
YFR031C-A	YFR032C-A	950
YFR031C-A	YGL014W	954
YFR031C-A	YGL030W	996
YFR031C-A	YGL031C	999
YFR031C-A	YGL076C	999
YFR031C-A	YGL099W	957
YFR031C-A	YGL103W	999
YFR031C-A	YGL123W	999
YFR031C-A	YGL135W	999
YFR031C-A	YGL147C	999
YFR031C-A	YGR027C	925
YFR031C-A	YGR034W	998
YFR031C-A	YGR085C	999
YFR031C-A	YGR103W	993
YFR031C-A	YGR118W	916
YFR031C-A	YGR148C	923
YFR031C-A	YGR214W	929
YFR031C-A	YHL001W	995
YFR031C-A	YHL015W	904
YFR031C-A	YHL033C	999
YFR031C-A	YHR010W	996
YFR031C-A	YHR021C	904
YFR031C-A	YHR141C	999
YFR031C-A	YHR203C	998
YFR031C-A	YIL018W	996
YFR031C-A	YIL052C	903
YFR031C-A	YIL069C	905
YFR031C-A	YIL133C	999

YFR031C-A	YIL148W	901
YFR031C-A	YJL136C	912
YFR031C-A	YJL177W	998
YFR031C-A	YJL189W	908
YFR031C-A	YJL190C	999
YFR031C-A	YJL191W	998
YFR031C-A	YJR094W-A	992
YFR031C-A	YJR123W	998
YFR031C-A	YJR145C	997
YFR031C-A	YKL006W	910
YFR031C-A	YKL156W	904
YFR031C-A	YKL170W	970
YFR031C-A	YKL180W	999
YFR031C-A	YKR057W	912
YFR031C-A	YKR094C	901
YFR031C-A	YLL045C	997
YFR031C-A	YLR029C	999
YFR031C-A	YLR048W	913
YFR031C-A	YLR061W	901
YFR031C-A	YLR075W	999
YFR031C-A	YLR150W	954
YFR031C-A	YLR167W	959
YFR031C-A	YLR185W	989
YFR031C-A	YLR264W	917
YFR031C-A	YLR287C-A	995
YFR031C-A	YLR325C	929
YFR031C-A	YLR333C	923
YFR031C-A	YLR340W	997
YFR031C-A	YLR344W	999
YFR031C-A	YLR367W	999
YFR031C-A	YLR388W	948
YFR031C-A	YLR406C	912
YFR031C-A	YLR441C	997
YFR031C-A	YLR448W	996
YFR031C-A	YML024W	913
YFR031C-A	YML026C	924
YFR031C-A	YML063W	996
YFR031C-A	YML073C	997
YFR031C-A	YMR121C	940
YFR031C-A	YMR142C	997
YFR031C-A	YMR194W	995
YFR031C-A	YMR230W	904
YFR031C-A	YMR242C	998
YFR031C-A	YNL067W	973

YFR031C-A	YNL069C	998
YFR031C-A	YNL081C	950
YFR031C-A	YNL096C	996
YFR031C-A	YNL162W	920
YFR031C-A	YNL178W	999
YFR031C-A	YNL301C	999
YFR031C-A	YNL302C	997
YFR031C-A	YNR037C	982
YFR031C-A	YOL039W	934
YFR031C-A	YOL040C	999
YFR031C-A	YOL120C	998
YFR031C-A	YOL121C	951
YFR031C-A	YOL127W	999
YFR031C-A	YOR063W	999
YFR031C-A	YOR096W	993
YFR031C-A	YOR133W	913
YFR031C-A	YOR167C	929
YFR031C-A	YOR204W	941
YFR031C-A	YOR234C	907
YFR031C-A	YOR293W	905
YFR031C-A	YOR312C	996
YFR031C-A	YOR369C	967
YFR031C-A	YPL079W	929
YFR031C-A	YPL081W	912
YFR031C-A	YPL090C	995
YFR031C-A	YPL093W	953
YFR031C-A	YPL131W	998
YFR031C-A	YPL143W	998
YFR031C-A	YPL198W	997
YFR031C-A	YPL220W	995
YFR031C-A	YPL249C-A	996
YFR031C-A	YPR043W	929
YFR031C-A	YPR080W	905
YFR031C-A	YPR102C	998
YFR031C-A	YPR132W	916
YFR032C-A	YBL027W	998
YFR032C-A	YBL072C	929
YFR032C-A	YBL087C	940
YFR032C-A	YBL092W	999
YFR032C-A	YBR031W	935
YFR032C-A	YBR048W	924
YFR032C-A	YBR084C-A	932
YFR032C-A	YBR118W	906
YFR032C-A	YBR181C	917

YFR032C-A	YBR189W	918
YFR032C-A	YBR191W	998
YFR032C-A	YCR031C	937
YFR032C-A	YDL061C	910
YFR032C-A	YDL075W	996
YFR032C-A	YDL081C	947
YFR032C-A	YDL082W	996
YFR032C-A	YDL083C	975
YFR032C-A	YDL130W	901
YFR032C-A	YDL136W	998
YFR032C-A	YDL191W	938
YFR032C-A	YDR012W	935
YFR032C-A	YDR025W	924
YFR032C-A	YDR064W	973
YFR032C-A	YDR382W	998
YFR032C-A	YDR385W	923
YFR032C-A	YDR418W	967
YFR032C-A	YDR447C	997
YFR032C-A	YDR450W	934
YFR032C-A	YDR471W	995
YFR032C-A	YDR500C	926
YFR032C-A	YEL054C	938
YFR032C-A	YER056C-A	906
YFR032C-A	YER074W	996
YFR032C-A	YER102W	929
YFR032C-A	YER117W	940
YFR032C-A	YER131W	922
YFR032C-A	YFL034C-A	920
YFR032C-A	YFR031C-A	950
YFR032C-A	YGL014W	976
YFR032C-A	YGL030W	950
YFR032C-A	YGL031C	997
YFR032C-A	YGL076C	938
YFR032C-A	YGL103W	995
YFR032C-A	YGL123W	938
YFR032C-A	YGL135W	928
YFR032C-A	YGL147C	935
YFR032C-A	YGL189C	996
YFR032C-A	YGR027C	936
YFR032C-A	YGR034W	996
YFR032C-A	YGR085C	997
YFR032C-A	YGR118W	998
YFR032C-A	YGR148C	905
YFR032C-A	YGR214W	933

YFR032C-A	YHL001W	946
YFR032C-A	YHL015W	970
YFR032C-A	YHL033C	935
YFR032C-A	YHR010W	907
YFR032C-A	YHR021C	951
YFR032C-A	YHR141C	941
YFR032C-A	YHR203C	940
YFR032C-A	YIL018W	903
YFR032C-A	YIL052C	906
YFR032C-A	YIL069C	921
YFR032C-A	YIL133C	924
YFR032C-A	YIL148W	915
YFR032C-A	YJL136C	948
YFR032C-A	YJL177W	996
YFR032C-A	YJL189W	913
YFR032C-A	YJL190C	928
YFR032C-A	YJL191W	937
YFR032C-A	YJR094W-A	933
YFR032C-A	YJR123W	966
YFR032C-A	YJR145C	940
YFR032C-A	YKL006W	946
YFR032C-A	YKL046C	958
YFR032C-A	YKL156W	951
YFR032C-A	YKL180W	934
YFR032C-A	YKR057W	948
YFR032C-A	YKR094C	915
YFR032C-A	YLL045C	935
YFR032C-A	YLR029C	996
YFR032C-A	YLR048W	933
YFR032C-A	YLR061W	923
YFR032C-A	YLR075W	993
YFR032C-A	YLR167W	959
YFR032C-A	YLR185W	927
YFR032C-A	YLR264W	922
YFR032C-A	YLR287C-A	997
YFR032C-A	YLR325C	949
YFR032C-A	YLR333C	934
YFR032C-A	YLR340W	968
YFR032C-A	YLR344W	938
YFR032C-A	YLR367W	928
YFR032C-A	YLR388W	910
YFR032C-A	YLR406C	935
YFR032C-A	YLR441C	927
YFR032C-A	YLR448W	997

YFR032C-A	YML024W	949
YFR032C-A	YML026C	934
YFR032C-A	YML063W	927
YFR032C-A	YML073C	935
YFR032C-A	YMR121C	907
YFR032C-A	YMR142C	926
YFR032C-A	YMR194W	997
YFR032C-A	YMR230W	940
YFR032C-A	YMR242C	997
YFR032C-A	YMR260C	943
YFR032C-A	YNL067W	935
YFR032C-A	YNL069C	923
YFR032C-A	YNL096C	998
YFR032C-A	YNL162W	941
YFR032C-A	YNL178W	971
YFR032C-A	YNL301C	927
YFR032C-A	YNL302C	933
YFR032C-A	YOL039W	913
YFR032C-A	YOL040C	999
YFR032C-A	YOL120C	927
YFR032C-A	YOL121C	934
YFR032C-A	YOL127W	997
YFR032C-A	YOR063W	957
YFR032C-A	YOR096W	936
YFR032C-A	YOR133W	923
YFR032C-A	YOR167C	922
YFR032C-A	YOR182C	905
YFR032C-A	YOR234C	948
YFR032C-A	YOR293W	941
YFR032C-A	YOR312C	939
YFR032C-A	YOR369C	936
YFR032C-A	YPL079W	928
YFR032C-A	YPL081W	918
YFR032C-A	YPL090C	917
YFR032C-A	YPL131W	904
YFR032C-A	YPL143W	948
YFR032C-A	YPL193W	924
YFR032C-A	YPL198W	938
YFR032C-A	YPL220W	928
YFR032C-A	YPL249C-A	950
YFR032C-A	YPR043W	933
YFR032C-A	YPR080W	906
YFR032C-A	YPR102C	936
YFR032C-A	YPR132W	935

YFR033C	Q0105	999
YFR033C	YBL045C	999
YFR033C	YDR375C	984
YFR033C	YDR529C	999
YFR033C	YEL024W	999
YFR033C	YGR183C	999
YFR033C	YHR001W-A	999
YFR033C	YJL166W	999
YFR033C	YJR048W	989
YFR033C	YLR075W	931
YFR033C	YOR065W	999
YFR033C	YPR191W	999
YFR034C	YBR093C	990
YFR034C	YDL106C	999
YFR034C	YDL127W	936
YFR034C	YDR335W	998
YFR034C	YDR481C	931
YFR034C	YER148W	958
YFR034C	YGL134W	941
YFR034C	YGR233C	999
YFR034C	YJL012C	911
YFR034C	YMR308C	996
YFR034C	YNL289W	936
YFR034C	YOL001W	999
YFR034C	YOL108C	970
YFR034C	YOR290C	975
YFR034C	YPL031C	999
YFR034C	YPR086W	967
YFR036W	YBL084C	999
YFR036W	YDL008W	999
YFR036W	YDR113C	928
YFR036W	YDR118W	999
YFR036W	YDR260C	999
YFR036W	YGL116W	969
YFR036W	YGL240W	999
YFR036W	YHR166C	999
YFR036W	YIR025W	998
YFR036W	YKL022C	999
YFR036W	YLR102C	999
YFR036W	YLR127C	999
YFR036W	YMR001C	912
YFR036W	YNL172W	999
YFR036W	YOR249C	999
YFR036W	YPL240C	919

YFR036W	YPR119W	916
YFR037C	YBR245C	999
YFR037C	YCR020C-A	940
YFR037C	YCR020W-B	999
YFR037C	YCR052W	999
YFR037C	YDR303C	999
YFR037C	YGL133W	970
YFR037C	YGR056W	999
YFR037C	YGR275W	999
YFR037C	YHR056C	999
YFR037C	YIL126W	999
YFR037C	YKR001C	970
YFR037C	YKR008W	999
YFR037C	YLR033W	999
YFR037C	YLR321C	999
YFR037C	YLR347C	964
YFR037C	YLR357W	999
YFR037C	YML127W	999
YFR037C	YMR033W	999
YFR037C	YMR072W	978
YFR037C	YMR091C	999
YFR037C	YNL030W	968
YFR037C	YNR003C	981
YFR037C	YOR290C	976
YFR037C	YOR304W	972
YFR037C	YPL082C	972
YFR037C	YPR034W	999
YFR040W	YBR164C	967
YFR040W	YDL047W	999
YFR040W	YFR021W	967
YFR040W	YGL056C	955
YFR040W	YJL098W	976
YFR040W	YKR028W	976
YFR040W	YPR040W	986
YFR041C	YJL034W	917
YFR041C	YMR214W	900
YFR041C	YOR254C	992
YFR042W	YPR159W	998
YFR044C	YDR170C	935
YFR044C	YNL191W	927
YFR046C	YBR160W	916
YFR046C	YER018C	963
YFR046C	YMR117C	993
YFR047C	YGR010W	906

YFR047C	YLR328W	907
YFR047C	YOR209C	990
YFR048W	YKL065C	998
YFR049W	Q0140	962
YFR049W	YDR148C	990
YFR049W	YFL018C	994
YFR049W	YIL125W	984
YFR050C	YBL041W	999
YFR050C	YBR270C	999
YFR050C	YDL007W	911
YFR050C	YDL097C	954
YFR050C	YDL147W	973
YFR050C	YDR394W	910
YFR050C	YDR427W	915
YFR050C	YER012W	999
YFR050C	YER016W	999
YFR050C	YER021W	962
YFR050C	YER094C	999
YFR050C	YER105C	999
YFR050C	YFL007W	999
YFR050C	YFR004W	969
YFR050C	YFR052W	935
YFR050C	YGL011C	999
YFR050C	YGL048C	933
YFR050C	YGR067C	999
YFR050C	YGR135W	999
YFR050C	YGR253C	999
YFR050C	YHR027C	911
YFR050C	YHR052W	984
YFR050C	YHR200W	999
YFR050C	YIL007C	932
YFR050C	YIL075C	945
YFR050C	YIL148W	900
YFR050C	YJL001W	999
YFR050C	YJL047C	998
YFR050C	YKL145W	912
YFR050C	YKR094C	900
YFR050C	YLL039C	905
YFR050C	YML091C	937
YFR050C	YML092C	999
YFR050C	YMR314W	999
YFR050C	YNL253W	999
YFR050C	YOL038W	999
YFR050C	YOR117W	908

YFR050C	YOR157C	999
YFR050C	YOR259C	910
YFR050C	YOR261C	992
YFR050C	YOR362C	999
YFR050C	YPR103W	999
YFR050C	YPR108W	947
YFR051C	YBR162C	962
YFR051C	YDL145C	999
YFR051C	YDL192W	931
YFR051C	YDR238C	999
YFR051C	YGL137W	999
YFR051C	YGL145W	942
YFR051C	YIL076W	999
YFR051C	YKR067W	956
YFR051C	YNL258C	987
YFR051C	YNL287W	999
YFR051C	YPL010W	999
YFR052W	YBL041W	911
YFR052W	YBR160W	900
YFR052W	YBR272C	983
YFR052W	YCR014C	970
YFR052W	YDL007W	999
YFR052W	YDL097C	999
YFR052W	YDL147W	999
YFR052W	YDR273W	934
YFR052W	YDR363W-A	999
YFR052W	YDR394W	999
YFR052W	YDR427W	999
YFR052W	YEL037C	982
YFR052W	YER012W	999
YFR052W	YER021W	999
YFR052W	YER094C	944
YFR052W	YFR004W	999
YFR052W	YFR010W	994
YFR052W	YFR050C	935
YFR052W	YGL011C	905
YFR052W	YGL048C	999
YFR052W	YGR135W	909
YFR052W	YGR232W	998
YFR052W	YGR253C	909
YFR052W	YHL030W	999
YFR052W	YHR027C	999
YFR052W	YHR200W	999
YFR052W	YIL075C	999

YFR052W	YKL145W	999
YFR052W	YLL039C	915
YFR052W	YLR421C	999
YFR052W	YML092C	955
YFR052W	YMR036C	907
YFR052W	YMR191W	987
YFR052W	YMR314W	999
YFR052W	YOL038W	910
YFR052W	YOR056C	982
YFR052W	YOR117W	999
YFR052W	YOR157C	910
YFR052W	YOR259C	999
YFR052W	YOR261C	999
YFR052W	YOR362C	903
YFR052W	YPR103W	927
YFR052W	YPR108W	999
YFR053C	YBR019C	914
YFR053C	YBR196C	936
YFR053C	YCL040W	998
YFR053C	YDL035C	967
YFR053C	YDR516C	923
YFR053C	YER003C	906
YFR053C	YGL253W	999
YFR053C	YGR240C	907
YFR053C	YJL155C	911
YFR053C	YLR377C	916
YFR053C	YMR105C	964
YFR053C	YMR205C	909
YFR055W	YAL012W	925
YFR055W	YAL062W	905
YFR055W	YER091C	974
YFR055W	YGR012W	944
YFR055W	YLR303W	908
YFR055W	YOR375C	906
YGL001C	YBR202W	938
YGL001C	YDR297W	909
YGL001C	YER044C	989
YGL001C	YGL012W	998
YGL001C	YGR060W	999
YGL001C	YGR175C	999
YGL001C	YHR007C	999
YGL001C	YHR072W	999
YGL001C	YHR190W	999
YGL001C	YLR056W	998

YGL001C	YLR100W	999
YGL001C	YML008C	999
YGL001C	YMR202W	998
YGL001C	YNL280C	998
YGL003C	YBL084C	999
YGL003C	YBR160W	999
YGL003C	YDL155W	999
YGL003C	YDR099W	992
YGL003C	YDR113C	998
YGL003C	YDR146C	999
YGL003C	YER177W	992
YGL003C	YFR028C	998
YGL003C	YGL116W	991
YGL003C	YGL240W	994
YGL003C	YGR108W	963
YGL003C	YHR152W	914
YGL003C	YHR166C	998
YGL003C	YJL030W	953
YGL003C	YJL187C	990
YGL003C	YJL194W	999
YGL003C	YKL022C	998
YGL003C	YKL101W	999
YGL003C	YLR079W	999
YGL003C	YLR210W	968
YGL003C	YMR001C	999
YGL003C	YPL256C	995
YGL003C	YPL267W	999
YGL003C	YPR119W	999
YGL003C	YPR120C	999
YGL004C	YDL007W	967
YGL004C	YDL097C	945
YGL004C	YDL147W	964
YGL004C	YDR394W	932
YGL004C	YDR427W	961
YGL004C	YFR004W	955
YGL004C	YGR232W	980
YGL004C	YHR200W	941
YGL004C	YKL145W	967
YGL004C	YLR421C	907
YGL004C	YOR117W	949
YGL005C	YBR164C	938
YGL005C	YER157W	999
YGL005C	YGL054C	901
YGL005C	YGL223C	999

YGL005C	YGR120C	999
YGL005C	YIL109C	907
YGL005C	YML071C	999
YGL005C	YMR292W	907
YGL005C	YNL041C	999
YGL005C	YNL051W	999
YGL005C	YOR070C	965
YGL005C	YPL051W	929
YGL005C	YPR105C	999
YGL006W	YDL128W	999
YGL006W	YKL190W	959
YGL006W	YLR093C	991
YGL006W	YNL027W	903
YGL008C	YBL069W	928
YGL008C	YHR098C	984
YGL008C	YIL005W	997
YGL008C	YIL109C	981
YGL008C	YJL192C	909
YGL008C	YJR059W	996
YGL008C	YMR296C	919
YGL008C	YNL243W	941
YGL008C	YOR153W	952
YGL009C	YCL018W	999
YGL009C	YLR355C	986
YGL009C	YLR451W	924
YGL009C	YMR108W	968
YGL009C	YNL104C	993
YGL009C	YOR108W	939
YGL011C	YBL041W	999
YGL011C	YBR173C	999
YGL011C	YCR057C	974
YGL011C	YDL014W	915
YGL011C	YDL097C	929
YGL011C	YDL147W	995
YGL011C	YDL188C	944
YGL011C	YDR324C	967
YGL011C	YER012W	999
YGL011C	YER021W	927
YGL011C	YER094C	999
YGL011C	YER099C	954
YGL011C	YFL007W	999
YGL011C	YFR004W	927
YGL011C	YFR050C	999
YGL011C	YFR052W	905

YGL011C	YGL048C	999
YGL011C	YGR135W	999
YGL011C	YGR253C	999
YGL011C	YHR027C	911
YGL011C	YHR052W	984
YGL011C	YHR200W	998
YGL011C	YIL075C	915
YGL011C	YJL001W	999
YGL011C	YLL039C	970
YGL011C	YML092C	999
YGL011C	YMR314W	999
YGL011C	YOL038W	999
YGL011C	YOR078W	967
YGL011C	YOR157C	999
YGL011C	YOR362C	999
YGL011C	YPR103W	999
YGL011C	YPR108W	901
YGL012W	YBR290W	940
YGL012W	YER044C	999
YGL012W	YGL001C	998
YGL012W	YGR060W	998
YGL012W	YGR175C	999
YGL012W	YHR007C	999
YGL012W	YLR100W	998
YGL012W	YML008C	999
YGL012W	YMR015C	999
YGL012W	YNL280C	999
YGL013C	YBL005W	999
YGL013C	YDR011W	997
YGL013C	YDR406W	984
YGL013C	YGR281W	959
YGL013C	YHR178W	911
YGL013C	YJL219W	962
YGL013C	YLR056W	976
YGL013C	YOL156W	959
YGL013C	YOR153W	999
YGL013C	YOR328W	982
YGL014W	YBL027W	978
YGL014W	YBL092W	978
YGL014W	YBR181C	972
YGL014W	YBR191W	978
YGL014W	YDL075W	948
YGL014W	YDL082W	956
YGL014W	YDL136W	972

YGL014W	YDR064W	944
YGL014W	YDR447C	954
YGL014W	YDR471W	956
YGL014W	YER074W	950
YGL014W	YFR031C-A	954
YGL014W	YFR032C-A	976
YGL014W	YGL103W	954
YGL014W	YGR034W	972
YGL014W	YHL001W	948
YGL014W	YHR029C	970
YGL014W	YHR141C	978
YGL014W	YJL177W	950
YGL014W	YLR287C-A	978
YGL014W	YLR448W	952
YGL014W	YML063W	956
YGL014W	YML073C	958
YGL014W	YMR194W	950
YGL014W	YMR242C	958
YGL014W	YNL096C	978
YGL014W	YNL301C	978
YGL014W	YNL302C	972
YGL014W	YNR052C	976
YGL014W	YOL040C	978
YGL014W	YOL127W	966
YGL014W	YPR129W	903
YGL015C	YLR319C	943
YGL016W	YER110C	942
YGL016W	YKL058W	919
YGL016W	YLR335W	990
YGL016W	YMR047C	985
YGL016W	YOR098C	990
YGL016W	YOR185C	903
YGL016W	YOR194C	920
YGL016W	YOR229W	905
YGL016W	YOR230W	901
YGL018C	YCL017C	953
YGL018C	YFL016C	951
YGL018C	YGR285C	908
YGL018C	YJR104C	988
YGL018C	YLR369W	996
YGL018C	YMR214W	917
YGL018C	YNL007C	900
YGL018C	YNL064C	904
YGL018C	YNL227C	907

YGL018C	YPL135W	999
YGL018C	YPL252C	932
YGL019W	YBR279W	966
YGL019W	YCL031C	989
YGL019W	YDR299W	914
YGL019W	YER164W	999
YGL019W	YGL207W	999
YGL019W	YGL244W	966
YGL019W	YGR090W	999
YGL019W	YGR116W	933
YGL019W	YIL035C	999
YGL019W	YJL069C	933
YGL019W	YKL160W	987
YGL019W	YLR079W	909
YGL019W	YLR418C	991
YGL019W	YML069W	999
YGL019W	YMR172W	996
YGL019W	YOL145C	994
YGL019W	YOR039W	999
YGL019W	YOR061W	999
YGL019W	YOR123C	966
YGL019W	YPL217C	915
YGL020C	YDL100C	998
YGL020C	YEL031W	954
YGL020C	YER083C	977
YGL022W	YBL082C	918
YGL022W	YBL105C	928
YGL022W	YDL232W	999
YGL022W	YEL002C	999
YGL022W	YER087C-B	977
YGL022W	YGL226C-A	999
YGL022W	YJL002C	999
YGL022W	YJL174W	904
YGL022W	YLR378C	986
YGL022W	YML019W	999
YGL022W	YMR149W	999
YGL022W	YOR085W	999
YGL022W	YOR103C	999
YGL023C	YBR077C	960
YGL023C	YGR163W	996
YGL023C	YML121W	996
YGL025C	YBL093C	999
YGL025C	YBR193C	999
YGL025C	YBR253W	999

YGL025C	YCR081W	995
YGL025C	YCR084C	981
YGL025C	YDL005C	999
YGL025C	YDL140C	936
YGL025C	YDR138W	920
YGL025C	YDR167W	991
YGL025C	YDR308C	999
YGL025C	YDR443C	998
YGL025C	YER022W	999
YGL025C	YER171W	913
YGL025C	YGL127C	999
YGL025C	YGL151W	999
YGL025C	YGR104C	999
YGL025C	YHR041C	999
YGL025C	YHR058C	999
YGL025C	YIL021W	975
YGL025C	YJL140W	923
YGL025C	YKL028W	984
YGL025C	YLR071C	999
YGL025C	YLR418C	915
YGL025C	YML007W	974
YGL025C	YMR112C	998
YGL025C	YNL236W	999
YGL025C	YNR010W	999
YGL025C	YOL051W	999
YGL025C	YOL135C	999
YGL025C	YOR151C	922
YGL025C	YOR174W	999
YGL025C	YPL042C	995
YGL025C	YPL129W	991
YGL025C	YPR070W	999
YGL025C	YPR168W	999
YGL026C	YDR007W	992
YGL026C	YDR354W	978
YGL026C	YDR510W	921
YGL026C	YER090W	975
YGL026C	YER180C	904
YGL026C	YGL202W	958
YGL026C	YKL211C	995
YGL027C	YBR229C	989
YGL027C	YEL002C	936
YGL027C	YIL022W	998
YGL027C	YMR214W	928
YGL027C	YNL322C	959

YGL027C	YOR067C	938
YGL027C	YOR085W	990
YGL027C	YPR159W	931
YGL028C	YDR077W	920
YGL028C	YER124C	999
YGL028C	YHR143W	999
YGL028C	YLR286C	910
YGL028C	YNL066W	968
YGL028C	YNL300W	913
YGL028C	YNL327W	907
YGL029W	YER126C	910
YGL029W	YOR294W	925
YGL030W	YAL035W	963
YGL030W	YBL027W	996
YGL030W	YBL072C	933
YGL030W	YBL087C	933
YGL030W	YBL092W	998
YGL030W	YBR031W	999
YGL030W	YBR048W	999
YGL030W	YBR084C-A	975
YGL030W	YBR118W	939
YGL030W	YBR181C	998
YGL030W	YBR189W	999
YGL030W	YBR191W	999
YGL030W	YCR031C	999
YGL030W	YDL014W	954
YGL030W	YDL061C	998
YGL030W	YDL075W	999
YGL030W	YDL081C	925
YGL030W	YDL082W	997
YGL030W	YDL083C	982
YGL030W	YDL130W	955
YGL030W	YDL136W	999
YGL030W	YDL191W	958
YGL030W	YDR012W	990
YGL030W	YDR025W	996
YGL030W	YDR064W	999
YGL030W	YDR101C	935
YGL030W	YDR382W	999
YGL030W	YDR385W	961
YGL030W	YDR418W	999
YGL030W	YDR447C	997
YGL030W	YDR450W	912
YGL030W	YDR471W	999

YGL030W	YDR496C	933
YGL030W	YDR500C	985
YGL030W	YEL026W	952
YGL030W	YEL054C	998
YGL030W	YER006W	942
YGL030W	YER056C-A	999
YGL030W	YER074W	997
YGL030W	YER102W	998
YGL030W	YER117W	979
YGL030W	YER131W	970
YGL030W	YFL034C-A	919
YGL030W	YFR031C-A	996
YGL030W	YFR032C-A	950
YGL030W	YGL031C	998
YGL030W	YGL076C	998
YGL030W	YGL103W	999
YGL030W	YGL123W	998
YGL030W	YGL135W	996
YGL030W	YGL147C	997
YGL030W	YGL189C	935
YGL030W	YGR027C	999
YGL030W	YGR034W	997
YGL030W	YGR085C	999
YGL030W	YGR103W	953
YGL030W	YGR118W	999
YGL030W	YGR148C	997
YGL030W	YGR214W	998
YGL030W	YHL001W	999
YGL030W	YHL015W	998
YGL030W	YHL033C	999
YGL030W	YHR010W	999
YGL030W	YHR021C	947
YGL030W	YHR052W	977
YGL030W	YHR141C	997
YGL030W	YHR203C	999
YGL030W	YIL018W	995
YGL030W	YIL052C	999
YGL030W	YIL069C	997
YGL030W	YIL133C	997
YGL030W	YIL148W	975
YGL030W	YJL136C	981
YGL030W	YJL177W	999
YGL030W	YJL189W	980
YGL030W	YJL190C	997

YGL030W	YJL191W	998
YGL030W	YJR094W-A	998
YGL030W	YJR123W	999
YGL030W	YJR145C	927
YGL030W	YKL006W	999
YGL030W	YKL156W	996
YGL030W	YKL180W	999
YGL030W	YKL181W	966
YGL030W	YKR057W	949
YGL030W	YKR094C	912
YGL030W	YLL045C	998
YGL030W	YLR029C	999
YGL030W	YLR048W	997
YGL030W	YLR061W	999
YGL030W	YLR075W	999
YGL030W	YLR150W	956
YGL030W	YLR167W	971
YGL030W	YLR185W	980
YGL030W	YLR264W	995
YGL030W	YLR287C-A	996
YGL030W	YLR325C	998
YGL030W	YLR333C	951
YGL030W	YLR340W	998
YGL030W	YLR344W	996
YGL030W	YLR367W	999
YGL030W	YLR388W	999
YGL030W	YLR406C	930
YGL030W	YLR441C	997
YGL030W	YLR448W	999
YGL030W	YML024W	999
YGL030W	YML026C	912
YGL030W	YML063W	999
YGL030W	YML073C	997
YGL030W	YML106W	946
YGL030W	YMR121C	943
YGL030W	YMR142C	997
YGL030W	YMR194W	999
YGL030W	YMR230W	968
YGL030W	YMR242C	997
YGL030W	YMR260C	942
YGL030W	YNL002C	935
YGL030W	YNL067W	993
YGL030W	YNL069C	997
YGL030W	YNL096C	945

YGL030W	YNL110C	979
YGL030W	YNL132W	944
YGL030W	YNL162W	938
YGL030W	YNL178W	999
YGL030W	YNL209W	942
YGL030W	YNL301C	973
YGL030W	YNL302C	936
YGL030W	YOL039W	923
YGL030W	YOL040C	964
YGL030W	YOL077C	952
YGL030W	YOL120C	925
YGL030W	YOL121C	937
YGL030W	YOL127W	999
YGL030W	YOR063W	999
YGL030W	YOR096W	999
YGL030W	YOR133W	965
YGL030W	YOR167C	961
YGL030W	YOR182C	911
YGL030W	YOR204W	931
YGL030W	YOR234C	997
YGL030W	YOR293W	983
YGL030W	YOR312C	948
YGL030W	YOR369C	964
YGL030W	YPL037C	999
YGL030W	YPL079W	976
YGL030W	YPL081W	964
YGL030W	YPL090C	909
YGL030W	YPL093W	935
YGL030W	YPL131W	998
YGL030W	YPL143W	997
YGL030W	YPL198W	945
YGL030W	YPL220W	999
YGL030W	YPL249C-A	998
YGL030W	YPR016C	955
YGL030W	YPR043W	965
YGL030W	YPR080W	940
YGL030W	YPR102C	992
YGL030W	YPR132W	995
YGL031C	YBL027W	999
YGL031C	YBL072C	904
YGL031C	YBL087C	993
YGL031C	YBL092W	999
YGL031C	YBR031W	997
YGL031C	YBR048W	909

YGL031C	YBR084C-A	912
YGL031C	YBR181C	998
YGL031C	YBR189W	998
YGL031C	YBR191W	996
YGL031C	YCR031C	996
YGL031C	YDL061C	949
YGL031C	YDL075W	992
YGL031C	YDL081C	918
YGL031C	YDL082W	997
YGL031C	YDL083C	956
YGL031C	YDL136W	999
YGL031C	YDL191W	916
YGL031C	YDR012W	994
YGL031C	YDR025W	909
YGL031C	YDR064W	964
YGL031C	YDR382W	942
YGL031C	YDR418W	995
YGL031C	YDR447C	996
YGL031C	YDR471W	998
YGL031C	YEL054C	909
YGL031C	YER056C-A	931
YGL031C	YER074W	906
YGL031C	YER102W	904
YGL031C	YER117W	999
YGL031C	YER131W	990
YGL031C	YFR031C-A	999
YGL031C	YFR032C-A	997
YGL031C	YGL030W	998
YGL031C	YGL076C	999
YGL031C	YGL103W	999
YGL031C	YGL123W	952
YGL031C	YGL135W	989
YGL031C	YGL147C	996
YGL031C	YGL189C	907
YGL031C	YGR027C	983
YGL031C	YGR034W	996
YGL031C	YGR085C	998
YGL031C	YGR118W	999
YGL031C	YGR148C	999
YGL031C	YGR214W	999
YGL031C	YHL001W	966
YGL031C	YHL015W	953
YGL031C	YHL033C	999
YGL031C	YHR010W	965

YGL031C	YHR021C	907
YGL031C	YHR141C	999
YGL031C	YIL018W	908
YGL031C	YIL052C	960
YGL031C	YIL069C	906
YGL031C	YIL133C	992
YGL031C	YJL136C	934
YGL031C	YJL177W	997
YGL031C	YJL189W	914
YGL031C	YJL190C	951
YGL031C	YJL191W	930
YGL031C	YJR094W-A	991
YGL031C	YJR123W	998
YGL031C	YKL006W	966
YGL031C	YKL156W	935
YGL031C	YKL180W	999
YGL031C	YKR057W	904
YGL031C	YLL045C	956
YGL031C	YLR029C	995
YGL031C	YLR048W	952
YGL031C	YLR061W	939
YGL031C	YLR075W	999
YGL031C	YLR185W	989
YGL031C	YLR264W	952
YGL031C	YLR325C	938
YGL031C	YLR333C	943
YGL031C	YLR340W	937
YGL031C	YLR342W	970
YGL031C	YLR344W	991
YGL031C	YLR367W	944
YGL031C	YLR388W	953
YGL031C	YLR441C	996
YGL031C	YLR448W	939
YGL031C	YML024W	938
YGL031C	YML063W	998
YGL031C	YML073C	995
YGL031C	YMR121C	931
YGL031C	YMR142C	937
YGL031C	YMR194W	996
YGL031C	YMR230W	952
YGL031C	YMR242C	997
YGL031C	YMR260C	903
YGL031C	YNL067W	970
YGL031C	YNL069C	996

YGL031C	YNL096C	927
YGL031C	YNL162W	904
YGL031C	YNL178W	955
YGL031C	YNL301C	991
YGL031C	YNL302C	907
YGL031C	YOL039W	932
YGL031C	YOL040C	921
YGL031C	YOL120C	917
YGL031C	YOL121C	928
YGL031C	YOL127W	999
YGL031C	YOR063W	999
YGL031C	YOR096W	953
YGL031C	YOR167C	963
YGL031C	YOR234C	952
YGL031C	YOR293W	970
YGL031C	YOR312C	910
YGL031C	YOR369C	956
YGL031C	YPL079W	963
YGL031C	YPL081W	936
YGL031C	YPL131W	994
YGL031C	YPL143W	937
YGL031C	YPL198W	908
YGL031C	YPL249C-A	995
YGL031C	YPR043W	907
YGL031C	YPR080W	904
YGL031C	YPR102C	999
YGL031C	YPR132W	910
YGL033W	YER095W	945
YGL033W	YER179W	982
YGL033W	YGL183C	999
YGL033W	YLR263W	968
YGL033W	YOR351C	959
YGL035C	YBL093C	919
YGL035C	YBR112C	999
YGL035C	YBR126C	976
YGL035C	YCR084C	986
YGL035C	YDR335W	947
YGL035C	YDR443C	982
YGL035C	YDR477W	999
YGL035C	YER129W	918
YGL035C	YGL115W	980
YGL035C	YGL209W	996
YGL035C	YGL253W	998
YGL035C	YIL162W	960

YGL035C	YJL089W	942
YGL035C	YMR280C	939
YGL035C	YNL025C	912
YGL035C	YNL167C	907
YGL036W	YBR057C	992
YGL036W	YKR031C	907
YGL037C	YDL042C	971
YGL037C	YDR400W	933
YGL037C	YOR209C	987
YGL038C	YER001W	992
YGL038C	YER157W	919
YGL038C	YHL007C	976
YGL038C	YHR206W	958
YGL040C	YDL205C	999
YGL040C	YDR047W	976
YGL040C	YDR232W	993
YGL040C	YGR060W	974
YGL040C	YOR278W	961
YGL043W	YAL011W	974
YGL043W	YBR081C	903
YGL043W	YBR109C	976
YGL043W	YBR154C	999
YGL043W	YBR231C	972
YGL043W	YBR245C	942
YGL043W	YBR279W	938
YGL043W	YDL140C	999
YGL043W	YDR334W	925
YGL043W	YDR404C	995
YGL043W	YDR485C	972
YGL043W	YEL055C	940
YGL043W	YER151C	995
YGL043W	YER171W	909
YGL043W	YGL070C	999
YGL043W	YGL127C	996
YGL043W	YGL224C	926
YGL043W	YGR005C	998
YGL043W	YGR063C	999
YGL043W	YGR104C	981
YGL043W	YGR186W	998
YGL043W	YGR258C	984
YGL043W	YHR059W	914
YGL043W	YHR143W-A	989
YGL043W	YIL021W	999
YGL043W	YIL143C	908

YGL043W	YJL140W	999
YGL043W	YJL168C	905
YGL043W	YJR017C	951
YGL043W	YJR035W	988
YGL043W	YKL139W	909
YGL043W	YLR005W	916
YGL043W	YLR418C	908
YGL043W	YML010W	989
YGL043W	YML041C	972
YGL043W	YML094W	925
YGL043W	YNL236W	973
YGL043W	YOL005C	999
YGL043W	YOL012C	973
YGL043W	YOR151C	999
YGL043W	YOR210W	995
YGL043W	YOR224C	995
YGL043W	YOR290C	912
YGL043W	YPL129W	985
YGL043W	YPR187W	998
YGL044C	YDR228C	999
YGL044C	YDR301W	967
YGL044C	YDR432W	976
YGL044C	YGR047C	984
YGL044C	YIR011C	911
YGL044C	YKR002W	942
YGL044C	YMR039C	926
YGL044C	YMR061W	999
YGL044C	YNL044W	996
YGL044C	YOR001W	937
YGL044C	YOR250C	999
YGL045W	YGR229C	936
YGL045W	YJL168C	952
YGL045W	YPR173C	907
YGL047W	YBR070C	999
YGL047W	YBR110W	968
YGL047W	YBR243C	973
YGL048C	YBL041W	959
YGL048C	YBR272C	978
YGL048C	YDL007W	999
YGL048C	YDL097C	999
YGL048C	YDL147W	999
YGL048C	YDR363W-A	999
YGL048C	YDR394W	999
YGL048C	YDR427W	999

YGL048C	YEL037C	999
YGL048C	YER012W	999
YGL048C	YER021W	999
YGL048C	YER094C	919
YGL048C	YER148W	982
YGL048C	YFL007W	991
YGL048C	YFR004W	999
YGL048C	YFR010W	999
YGL048C	YFR050C	933
YGL048C	YFR052W	999
YGL048C	YGL011C	999
YGL048C	YGL141W	954
YGL048C	YGL207W	997
YGL048C	YGR135W	916
YGL048C	YGR232W	999
YGL048C	YGR253C	934
YGL048C	YHL030W	997
YGL048C	YHR027C	999
YGL048C	YHR200W	999
YGL048C	YIL007C	905
YGL048C	YIL075C	999
YGL048C	YIL148W	913
YGL048C	YJL001W	950
YGL048C	YKL010C	983
YGL048C	YKL145W	999
YGL048C	YLR167W	915
YGL048C	YLR421C	999
YGL048C	YML092C	957
YGL048C	YMR191W	987
YGL048C	YMR314W	967
YGL048C	YNL244C	999
YGL048C	YOL038W	932
YGL048C	YOR117W	999
YGL048C	YOR157C	953
YGL048C	YOR259C	999
YGL048C	YOR261C	999
YGL048C	YOR362C	972
YGL048C	YPL248C	999
YGL048C	YPR103W	999
YGL048C	YPR108W	999
YGL049C	YBL032W	988
YGL049C	YCL011C	972
YGL049C	YCL037C	970
YGL049C	YDR381W	948

YGL049C	YDR432W	970
YGL049C	YER165W	999
YGL049C	YGL173C	972
YGL049C	YGR162W	999
YGL049C	YHL034C	977
YGL049C	YIR001C	999
YGL049C	YJL138C	995
YGL049C	YJL190C	997
YGL049C	YJR007W	984
YGL049C	YKR059W	997
YGL049C	YLR175W	992
YGL049C	YML117W	968
YGL049C	YMR104C	979
YGL049C	YMR125W	998
YGL049C	YMR309C	948
YGL049C	YNL244C	980
YGL049C	YNL251C	991
YGL049C	YOL139C	999
YGL049C	YOR276W	938
YGL049C	YPL178W	914
YGL049C	YPL190C	970
YGL049C	YPR041W	977
YGL050W	YML005W	936
YGL051W	YAR033W	997
YGL051W	YDL145C	967
YGL051W	YNL287W	967
YGL054C	YAR002C-A	981
YGL054C	YDL100C	910
YGL054C	YGL005C	901
YGL054C	YGL200C	937
YGL054C	YIL140W	938
YGL054C	YJL004C	919
YGL054C	YML071C	921
YGL054C	YNL258C	917
YGL054C	YOR070C	909
YGL054C	YOR216C	943
YGL054C	YPL051W	923
YGL055W	YBR106W	984
YGL055W	YBR170C	925
YGL055W	YEL050C	901
YGL055W	YIR033W	991
YGL055W	YKL020C	991
YGL056C	YFR040W	955
YGL058W	YAL011W	961

YGL058W	YBR010W	982
YGL058W	YBR082C	978
YGL058W	YBR088C	999
YGL058W	YBR160W	976
YGL058W	YBR279W	913
YGL058W	YCR066W	999
YGL058W	YDL020C	985
YGL058W	YDL074C	999
YGL058W	YDR076W	911
YGL058W	YDR334W	963
YGL058W	YDR359C	928
YGL058W	YDR419W	924
YGL058W	YDR485C	961
YGL058W	YEL037C	977
YGL058W	YER095W	988
YGL058W	YER162C	944
YGL058W	YER171W	959
YGL058W	YGR063C	929
YGL058W	YGR184C	999
YGL058W	YGR258C	956
YGL058W	YJL092W	999
YGL058W	YJR052W	915
YGL058W	YKL139W	935
YGL058W	YLR024C	999
YGL058W	YLR032W	998
YGL058W	YLR085C	959
YGL058W	YML028W	977
YGL058W	YML032C	999
YGL058W	YML041C	957
YGL058W	YMR100W	959
YGL058W	YMR137C	948
YGL058W	YNL218W	921
YGL058W	YOL012C	993
YGL058W	YOR346W	925
YGL058W	YPL022W	993
YGL058W	YPL167C	998
YGL058W	YPR103W	999
YGL060W	YBR211C	981
YGL060W	YDR254W	981
YGL060W	YDR318W	991
YGL060W	YDR388W	986
YGL060W	YER018C	981
YGL060W	YGR179C	991
YGL060W	YIL144W	989

YGL060W	YJL030W	991
YGL060W	YJR135C	981
YGL060W	YKL049C	901
YGL060W	YLR381W	981
YGL060W	YOL069W	990
YGL060W	YPL018W	991
YGL060W	YPL233W	981
YGL060W	YPR046W	981
YGL061C	YBR156C	923
YGL061C	YBR233W-A	963
YGL061C	YDR016C	999
YGL061C	YDR201W	999
YGL061C	YDR320C-A	984
YGL061C	YER016W	974
YGL061C	YGR113W	999
YGL061C	YGR140W	932
YGL061C	YJL030W	979
YGL061C	YKL052C	999
YGL061C	YKL138C-A	996
YGL061C	YKR037C	999
YGL061C	YKR083C	999
YGL061C	YLR045C	963
YGL061C	YPL209C	983
YGL062W	YAL038W	991
YGL062W	YBR208C	930
YGL062W	YBR218C	979
YGL062W	YBR221C	905
YGL062W	YCR005C	928
YGL062W	YDL078C	951
YGL062W	YDR111C	905
YGL062W	YER178W	907
YGL062W	YGR240C	908
YGL062W	YGR254W	907
YGL062W	YHR174W	907
YGL062W	YKL085W	958
YGL062W	YKL106W	903
YGL062W	YKR097W	991
YGL062W	YLR027C	912
YGL062W	YLR089C	903
YGL062W	YLR377C	924
YGL062W	YMR205C	910
YGL062W	YNL117W	968
YGL062W	YNR001C	920
YGL062W	YOL126C	948

YGL062W	YOR347C	915
YGL062W	YPR001W	902
YGL063W	YNL292W	926
YGL064C	Q0045	903
YGL065C	YBR110W	999
YGL065C	YDL055C	902
YGL065C	YNL048W	989
YGL066W	YBR081C	999
YGL066W	YBR111W-A	997
YGL066W	YBR198C	999
YGL066W	YCL010C	999
YGL066W	YDR145W	999
YGL066W	YDR167W	999
YGL066W	YDR176W	999
YGL066W	YDR392W	999
YGL066W	YDR448W	999
YGL066W	YGL112C	999
YGL066W	YGR252W	999
YGL066W	YGR274C	988
YGL066W	YHR099W	999
YGL066W	YHR191C	926
YGL066W	YLR055C	999
YGL066W	YML015C	988
YGL066W	YMR223W	999
YGL066W	YMR236W	999
YGL066W	YOL148C	999
YGL066W	YPL011C	988
YGL066W	YPL047W	999
YGL066W	YPL254W	999
YGL068W	YDR116C	930
YGL068W	YHR147C	999
YGL068W	YJL063C	999
YGL068W	YNL185C	999
YGL070C	YAL011W	907
YGL070C	YBL026W	910
YGL070C	YBR154C	999
YGL070C	YBR198C	900
YGL070C	YBR279W	969
YGL070C	YDL115C	997
YGL070C	YDL140C	999
YGL070C	YDR079C-A	939
YGL070C	YDR167W	910
YGL070C	YDR392W	960
YGL070C	YDR404C	999

YGL070C	YDR443C	928
YGL070C	YDR485C	902
YGL070C	YEL026W	912
YGL070C	YER022W	981
YGL070C	YER029C	947
YGL070C	YER139C	906
YGL070C	YER148W	922
YGL070C	YER171W	925
YGL070C	YFL017W-A	958
YGL070C	YGL043W	999
YGL070C	YGL112C	901
YGL070C	YGL127C	987
YGL070C	YGR005C	999
YGL070C	YGR063C	987
YGL070C	YGR074W	941
YGL070C	YGR104C	988
YGL070C	YGR186W	999
YGL070C	YGR252W	961
YGL070C	YHR058C	917
YGL070C	YHR059W	915
YGL070C	YHR143W-A	999
YGL070C	YIL021W	999
YGL070C	YIL143C	922
YGL070C	YJL140W	999
YGL070C	YJR035W	998
YGL070C	YJR063W	955
YGL070C	YKL028W	997
YGL070C	YKL058W	917
YGL070C	YLR147C	950
YGL070C	YML010W	998
YGL070C	YMR277W	950
YGL070C	YMR288W	911
YGL070C	YNL113W	914
YGL070C	YNL236W	918
YGL070C	YOL005C	999
YGL070C	YOL051W	965
YGL070C	YOR116C	999
YGL070C	YOR151C	999
YGL070C	YOR207C	913
YGL070C	YOR210W	999
YGL070C	YOR224C	999
YGL070C	YPL129W	999
YGL070C	YPL178W	907
YGL070C	YPR056W	900

YGL070C	YPR086W	994
YGL070C	YPR168W	904
YGL070C	YPR182W	957
YGL070C	YPR187W	999
YGL071W	YBR107C	951
YGL071W	YBR112C	940
YGL071W	YDL120W	951
YGL071W	YDR098C	999
YGL071W	YDR270W	940
YGL071W	YER145C	993
YGL071W	YER174C	998
YGL071W	YGL160W	942
YGL071W	YKL220C	995
YGL071W	YLL051C	948
YGL071W	YLR047C	942
YGL071W	YLR214W	994
YGL071W	YMR058W	999
YGL071W	YMR308C	973
YGL071W	YMR319C	958
YGL071W	YNR060W	954
YGL071W	YOL152W	945
YGL071W	YOR381W	963
YGL071W	YOR384W	945
YGL071W	YPL202C	999
YGL071W	YPR052C	947
YGL073W	YAL005C	999
YGL073W	YBL075C	913
YGL073W	YBR160W	908
YGL073W	YDL229W	975
YGL073W	YDR477W	987
YGL073W	YER040W	908
YGL073W	YER103W	912
YGL073W	YFL033C	950
YGL073W	YHR053C	923
YGL073W	YJL141C	972
YGL073W	YKL062W	992
YGL073W	YLL024C	978
YGL073W	YMR037C	998
YGL073W	YMR186W	999
YGL073W	YNL209W	974
YGL073W	YOR027W	960
YGL073W	YOR178C	909
YGL073W	YPL106C	971
YGL073W	YPL240C	969

YGL075C	YBR160W	916
YGL075C	YJL019W	968
YGL075C	YMR117C	995
YGL075C	YNL107W	967
YGL075C	YPL255W	994
YGL076C	YAL035W	998
YGL076C	YBL027W	992
YGL076C	YBL072C	995
YGL076C	YBL087C	994
YGL076C	YBL092W	996
YGL076C	YBR031W	969
YGL076C	YBR048W	997
YGL076C	YBR084C-A	926
YGL076C	YBR118W	913
YGL076C	YBR142W	937
YGL076C	YBR181C	996
YGL076C	YBR189W	981
YGL076C	YBR191W	996
YGL076C	YCR031C	998
YGL076C	YDL061C	945
YGL076C	YDL075W	999
YGL076C	YDL081C	949
YGL076C	YDL082W	998
YGL076C	YDL083C	975
YGL076C	YDL130W	913
YGL076C	YDL136W	999
YGL076C	YDL191W	926
YGL076C	YDR012W	996
YGL076C	YDR025W	996
YGL076C	YDR060W	938
YGL076C	YDR064W	984
YGL076C	YDR382W	998
YGL076C	YDR385W	913
YGL076C	YDR418W	999
YGL076C	YDR447C	997
YGL076C	YDR450W	920
YGL076C	YDR471W	997
YGL076C	YDR496C	931
YGL076C	YDR500C	916
YGL076C	YEL054C	937
YGL076C	YER056C-A	939
YGL076C	YER074W	996
YGL076C	YER102W	995
YGL076C	YER117W	931

YGL076C	YER131W	971
YGL076C	YFL034C-A	920
YGL076C	YFR031C-A	999
YGL076C	YFR032C-A	938
YGL076C	YGL030W	998
YGL076C	YGL031C	999
YGL076C	YGL103W	999
YGL076C	YGL111W	937
YGL076C	YGL123W	971
YGL076C	YGL135W	999
YGL076C	YGL147C	999
YGL076C	YGL189C	923
YGL076C	YGR027C	976
YGL076C	YGR034W	997
YGL076C	YGR085C	998
YGL076C	YGR103W	954
YGL076C	YGR118W	960
YGL076C	YGR148C	969
YGL076C	YGR214W	981
YGL076C	YHL001W	999
YGL076C	YHL015W	971
YGL076C	YHL033C	999
YGL076C	YHR010W	956
YGL076C	YHR021C	931
YGL076C	YHR088W	937
YGL076C	YHR141C	994
YGL076C	YHR203C	940
YGL076C	YIL018W	995
YGL076C	YIL052C	953
YGL076C	YIL069C	996
YGL076C	YIL133C	999
YGL076C	YIL148W	906
YGL076C	YJL136C	954
YGL076C	YJL177W	998
YGL076C	YJL189W	919
YGL076C	YJL190C	998
YGL076C	YJL191W	963
YGL076C	YJR094W-A	993
YGL076C	YJR123W	999
YGL076C	YJR145C	995
YGL076C	YKL006W	978
YGL076C	YKL156W	956
YGL076C	YKL180W	999
YGL076C	YKR057W	935

YGL076C	YKR081C	938
YGL076C	YKR094C	906
YGL076C	YLL045C	999
YGL076C	YLR002C	927
YGL076C	YLR029C	999
YGL076C	YLR048W	979
YGL076C	YLR061W	960
YGL076C	YLR075W	999
YGL076C	YLR167W	900
YGL076C	YLR185W	991
YGL076C	YLR264W	954
YGL076C	YLR287C-A	908
YGL076C	YLR325C	941
YGL076C	YLR333C	945
YGL076C	YLR340W	961
YGL076C	YLR344W	993
YGL076C	YLR367W	962
YGL076C	YLR388W	959
YGL076C	YLR406C	926
YGL076C	YLR432W	979
YGL076C	YLR441C	997
YGL076C	YLR448W	998
YGL076C	YML024W	952
YGL076C	YML026C	920
YGL076C	YML056C	978
YGL076C	YML063W	999
YGL076C	YML073C	997
YGL076C	YMR121C	938
YGL076C	YMR142C	998
YGL076C	YMR194W	997
YGL076C	YMR230W	956
YGL076C	YMR242C	998
YGL076C	YMR260C	918
YGL076C	YMR290C	951
YGL076C	YNL067W	968
YGL076C	YNL069C	997
YGL076C	YNL096C	960
YGL076C	YNL132W	936
YGL076C	YNL162W	935
YGL076C	YNL178W	998
YGL076C	YNL301C	999
YGL076C	YNL302C	928
YGL076C	YNR037C	901
YGL076C	YOL039W	945

YGL076C	YOL040C	955
YGL076C	YOL041C	933
YGL076C	YOL120C	998
YGL076C	YOL121C	948
YGL076C	YOL127W	999
YGL076C	YOR063W	999
YGL076C	YOR096W	998
YGL076C	YOR133W	913
YGL076C	YOR167C	954
YGL076C	YOR182C	908
YGL076C	YOR206W	940
YGL076C	YOR234C	966
YGL076C	YOR293W	960
YGL076C	YOR312C	997
YGL076C	YOR369C	966
YGL076C	YPL079W	971
YGL076C	YPL081W	952
YGL076C	YPL090C	907
YGL076C	YPL093W	943
YGL076C	YPL131W	996
YGL076C	YPL143W	952
YGL076C	YPL198W	999
YGL076C	YPL220W	996
YGL076C	YPL249C-A	997
YGL076C	YPR043W	929
YGL076C	YPR080W	914
YGL076C	YPR102C	997
YGL076C	YPR132W	933
YGL078C	YCL059C	903
YGL078C	YDL014W	971
YGL078C	YDL208W	984
YGL078C	YER177W	940
YGL078C	YGR159C	944
YGL078C	YHR089C	986
YGL078C	YLR175W	975
YGL078C	YLR197W	980
YGL079W	YDR357C	935
YGL079W	YGR113W	905
YGL082W	YMR246W	921
YGL084C	YHR039C	913
YGL086W	YAR002W	993
YGL086W	YBL079W	922
YGL086W	YBR060C	999
YGL086W	YBR107C	924

YGL086W	YBR111W-A	908
YGL086W	YCL029C	939
YGL086W	YDL028C	984
YGL086W	YDR159W	915
YGL086W	YDR254W	971
YGL086W	YDR318W	952
YGL086W	YDR359C	903
YGL086W	YEL003W	930
YGL086W	YEL061C	997
YGL086W	YER007W	964
YGL086W	YER016W	999
YGL086W	YER018C	962
YGL086W	YER105C	932
YGL086W	YGL116W	997
YGL086W	YGR063C	931
YGL086W	YGR188C	999
YGL086W	YGR218W	911
YGL086W	YHR191C	939
YGL086W	YIL144W	918
YGL086W	YJL013C	978
YGL086W	YJL030W	999
YGL086W	YJR053W	917
YGL086W	YJR060W	983
YGL086W	YJR135C	968
YGL086W	YKL057C	931
YGL086W	YKR082W	931
YGL086W	YLR381W	967
YGL086W	YML124C	964
YGL086W	YMR055C	994
YGL086W	YMR117C	983
YGL086W	YMR153W	999
YGL086W	YOL072W	915
YGL086W	YOR026W	999
YGL086W	YOR195W	993
YGL086W	YOR257W	932
YGL086W	YOR349W	946
YGL086W	YPL008W	965
YGL086W	YPL018W	967
YGL086W	YPL241C	946
YGL086W	YPR046W	944
YGL086W	YPR141C	991
YGL087C	YBR088C	999
YGL087C	YCR066W	994
YGL087C	YDR092W	999

YGL087C	YDR419W	999
YGL087C	YJL092W	976
YGL087C	YJR006W	979
YGL087C	YJR043C	986
YGL087C	YLR032W	999
YGL087C	YMR140W	995
YGL087C	YPL167C	999
YGL089C	YFL026W	995
YGL089C	YPL187W	906
YGL090W	YDR076W	921
YGL090W	YDR369C	999
YGL090W	YLR265C	999
YGL090W	YLR424W	974
YGL090W	YOR005C	999
YGL091C	YIL003W	995
YGL091C	YIL129C	909
YGL091C	YNL240C	943
YGL092W	YBL079W	959
YGL092W	YBR017C	965
YGL092W	YDL116W	999
YGL092W	YDL207W	992
YGL092W	YDR192C	993
YGL092W	YER105C	993
YGL092W	YER107C	985
YGL092W	YFR002W	984
YGL092W	YGL100W	999
YGL092W	YGL172W	996
YGL092W	YGR119C	994
YGL092W	YIL115C	922
YGL092W	YJL041W	984
YGL092W	YJL061W	934
YGL092W	YJR042W	999
YGL092W	YKL057C	999
YGL092W	YKL068W	999
YGL092W	YKL186C	982
YGL092W	YKR082W	984
YGL092W	YLR208W	999
YGL092W	YLR347C	999
YGL092W	YML103C	977
YGL092W	YMR047C	999
YGL092W	YMR129W	928
YGL092W	YMR308C	963
YGL092W	YOR098C	931
YGL092W	YOR257W	961

YGL092W	YPL169C	992
YGL093W	YAL034W-A	961
YGL093W	YDR532C	926
YGL093W	YIL144W	942
YGL093W	YIR010W	941
YGL094C	YAL021C	936
YGL094C	YER165W	915
YGL094C	YKL025C	999
YGL095C	YBL050W	996
YGL095C	YBR288C	916
YGL095C	YDR323C	999
YGL095C	YDR468C	999
YGL095C	YKL196C	920
YGL095C	YMR197C	980
YGL095C	YOL018C	999
YGL095C	YOR036W	998
YGL095C	YOR089C	957
YGL097W	YAR002W	978
YGL097W	YDR002W	992
YGL097W	YGR218W	990
YGL097W	YHR082C	916
YGL097W	YIL063C	937
YGL097W	YJR074W	920
YGL097W	YLR293C	999
YGL097W	YLR335W	998
YGL097W	YML121W	998
YGL097W	YNL189W	903
YGL097W	YOL021C	943
YGL097W	YOR185C	999
YGL098W	YDR498C	999
YGL098W	YGL145W	999
YGL098W	YKL196C	998
YGL098W	YLR268W	999
YGL098W	YLR440C	999
YGL098W	YNL258C	999
YGL098W	YOR075W	999
YGL099W	YBR084W	945
YGL099W	YBR267W	999
YGL099W	YDR101C	999
YGL099W	YDR381W	930
YGL099W	YFR031C-A	957
YGL099W	YGR245C	976
YGL099W	YHR170W	999
YGL099W	YIL018W	946

YGL099W	YLL045C	977
YGL099W	YLR075W	982
YGL099W	YMR194W	985
YGL099W	YOR063W	991
YGL099W	YPR016C	998
YGL100W	YAL009W	918
YGL100W	YAR002W	947
YGL100W	YBL079W	905
YGL100W	YDL088C	948
YGL100W	YDL116W	999
YGL100W	YDL207W	976
YGL100W	YDR159W	966
YGL100W	YER105C	959
YGL100W	YER107C	900
YGL100W	YFR002W	938
YGL100W	YGL092W	999
YGL100W	YIL115C	949
YGL100W	YJL002C	962
YGL100W	YJL041W	961
YGL100W	YJR042W	999
YGL100W	YKL057C	999
YGL100W	YKL186C	992
YGL100W	YKR082W	988
YGL100W	YLR018C	950
YGL100W	YLR208W	999
YGL100W	YLR347C	987
YGL100W	YMR047C	998
YGL100W	YMR153W	944
YGL100W	YOR257W	961
YGL100W	YPL125W	905
YGL100W	YPL169C	999
YGL103W	YAL035W	918
YGL103W	YBL027W	999
YGL103W	YBL072C	993
YGL103W	YBL087C	994
YGL103W	YBL092W	999
YGL103W	YBR031W	998
YGL103W	YBR048W	956
YGL103W	YBR084C-A	988
YGL103W	YBR181C	995
YGL103W	YBR189W	999
YGL103W	YBR191W	999
YGL103W	YCR031C	999
YGL103W	YDL061C	994

YGL103W	YDL075W	999
YGL103W	YDL082W	996
YGL103W	YDL083C	962
YGL103W	YDL130W	937
YGL103W	YDL136W	999
YGL103W	YDL191W	903
YGL103W	YDL192W	901
YGL103W	YDR012W	999
YGL103W	YDR025W	947
YGL103W	YDR064W	999
YGL103W	YDR101C	940
YGL103W	YDR382W	999
YGL103W	YDR385W	906
YGL103W	YDR418W	999
YGL103W	YDR447C	997
YGL103W	YDR450W	913
YGL103W	YDR471W	999
YGL103W	YDR500C	964
YGL103W	YER056C-A	996
YGL103W	YER074W	995
YGL103W	YER102W	998
YGL103W	YER117W	987
YGL103W	YER131W	955
YGL103W	YFR031C-A	999
YGL103W	YFR032C-A	995
YGL103W	YGL014W	954
YGL103W	YGL030W	999
YGL103W	YGL031C	999
YGL103W	YGL076C	999
YGL103W	YGL123W	999
YGL103W	YGL135W	999
YGL103W	YGL147C	999
YGL103W	YGR027C	994
YGL103W	YGR034W	997
YGL103W	YGR085C	999
YGL103W	YGR118W	999
YGL103W	YGR148C	999
YGL103W	YGR214W	999
YGL103W	YHL001W	999
YGL103W	YHL015W	999
YGL103W	YHL033C	999
YGL103W	YHR010W	999
YGL103W	YHR052W	943
YGL103W	YHR141C	999

YGL103W	YHR203C	999
YGL103W	YIL018W	997
YGL103W	YIL052C	999
YGL103W	YIL069C	996
YGL103W	YIL133C	999
YGL103W	YIL148W	971
YGL103W	YJL136C	967
YGL103W	YJL177W	999
YGL103W	YJL189W	957
YGL103W	YJL190C	998
YGL103W	YJL191W	975
YGL103W	YJR094W-A	999
YGL103W	YJR123W	999
YGL103W	YJR145C	997
YGL103W	YKL006W	999
YGL103W	YKL156W	993
YGL103W	YKL172W	927
YGL103W	YKL180W	999
YGL103W	YKR081C	929
YGL103W	YLL045C	995
YGL103W	YLR029C	999
YGL103W	YLR048W	999
YGL103W	YLR061W	999
YGL103W	YLR075W	999
YGL103W	YLR167W	970
YGL103W	YLR185W	996
YGL103W	YLR264W	997
YGL103W	YLR287C-A	995
YGL103W	YLR325C	997
YGL103W	YLR333C	929
YGL103W	YLR340W	999
YGL103W	YLR342W	974
YGL103W	YLR344W	999
YGL103W	YLR367W	988
YGL103W	YLR388W	999
YGL103W	YLR406C	997
YGL103W	YLR441C	995
YGL103W	YLR448W	999
YGL103W	YML024W	999
YGL103W	YML026C	913
YGL103W	YML063W	999
YGL103W	YML072C	975
YGL103W	YML073C	995
YGL103W	YMR121C	905

YGL103W	YMR142C	996
YGL103W	YMR194W	999
YGL103W	YMR230W	953
YGL103W	YMR242C	995
YGL103W	YNL067W	997
YGL103W	YNL069C	995
YGL103W	YNL096C	995
YGL103W	YNL110C	979
YGL103W	YNL162W	914
YGL103W	YNL178W	999
YGL103W	YNL301C	999
YGL103W	YNL302C	997
YGL103W	YNR037C	917
YGL103W	YOL039W	919
YGL103W	YOL040C	997
YGL103W	YOL120C	903
YGL103W	YOL121C	906
YGL103W	YOL127W	999
YGL103W	YOR063W	999
YGL103W	YOR096W	999
YGL103W	YOR133W	925
YGL103W	YOR167C	960
YGL103W	YOR234C	995
YGL103W	YOR293W	994
YGL103W	YOR312C	996
YGL103W	YOR369C	909
YGL103W	YPL012W	943
YGL103W	YPL037C	998
YGL103W	YPL079W	974
YGL103W	YPL081W	960
YGL103W	YPL090C	995
YGL103W	YPL093W	936
YGL103W	YPL131W	999
YGL103W	YPL143W	994
YGL103W	YPL198W	993
YGL103W	YPL220W	998
YGL103W	YPL249C-A	997
YGL103W	YPR043W	946
YGL103W	YPR102C	999
YGL103W	YPR132W	973
YGL105W	YBL076C	914
YGL105W	YGL245W	999
YGL105W	YGR264C	999
YGL105W	YKL205W	999

YGL105W	YLL018C	930
YGL105W	YOR168W	914
YGL106W	YAL029C	999
YGL106W	YBR109C	999
YGL106W	YBR130C	999
YGL106W	YCL063W	936
YGL106W	YFL005W	963
YGL106W	YFL039C	993
YGL106W	YGL210W	940
YGL106W	YHR023W	999
YGL106W	YKL130C	992
YGL106W	YLR429W	986
YGL106W	YOR326W	999
YGL106W	YPL242C	999
YGL106W	YPR171W	984
YGL106W	YPR188C	926
YGL107C	YDR494W	941
YGL108C	YDL126C	920
YGL111W	YBL087C	968
YGL111W	YBR142W	903
YGL111W	YCL054W	976
YGL111W	YDL031W	982
YGL111W	YDR060W	978
YGL111W	YDR087C	996
YGL111W	YDR120C	962
YGL111W	YDR496C	994
YGL111W	YER006W	987
YGL111W	YFL002C	976
YGL111W	YGL076C	937
YGL111W	YGR103W	999
YGL111W	YHR052W	994
YGL111W	YHR066W	996
YGL111W	YHR088W	999
YGL111W	YHR197W	988
YGL111W	YKL009W	988
YGL111W	YKL021C	962
YGL111W	YKL172W	986
YGL111W	YKR081C	989
YGL111W	YLL008W	989
YGL111W	YLL034C	998
YGL111W	YLR002C	984
YGL111W	YLR276C	986
YGL111W	YMR049C	999
YGL111W	YMR290C	993

YGL111W	YNL002C	988
YGL111W	YNL061W	998
YGL111W	YNL110C	999
YGL111W	YOL077C	997
YGL111W	YOL127W	933
YGL111W	YOR063W	933
YGL111W	YOR206W	994
YGL111W	YOR272W	999
YGL111W	YPL043W	985
YGL111W	YPL093W	999
YGL111W	YPL131W	964
YGL111W	YPL211W	995
YGL111W	YPR016C	998
YGL112C	YAL017W	923
YGL112C	YBR081C	999
YGL112C	YBR111W-A	959
YGL112C	YBR198C	999
YGL112C	YBR253W	977
YGL112C	YCL010C	999
YGL112C	YCR042C	999
YGL112C	YDR044W	930
YGL112C	YDR145W	999
YGL112C	YDR167W	999
YGL112C	YDR176W	999
YGL112C	YDR392W	999
YGL112C	YDR404C	901
YGL112C	YDR448W	999
YGL112C	YEL009C	975
YGL112C	YER148W	999
YGL112C	YER164W	980
YGL112C	YGL066W	999
YGL112C	YGL070C	901
YGL112C	YGR094W	980
YGL112C	YGR252W	999
YGL112C	YGR274C	999
YGL112C	YHR041C	997
YGL112C	YHR099W	999
YGL112C	YIL021W	900
YGL112C	YIL143C	900
YGL112C	YKL028W	904
YGL112C	YLR055C	999
YGL112C	YML007W	985
YGL112C	YML015C	999
YGL112C	YML098W	999

YGL112C	YML114C	999
YGL112C	YMR005W	999
YGL112C	YMR043W	923
YGL112C	YMR223W	999
YGL112C	YMR227C	999
YGL112C	YMR236W	999
YGL112C	YNL236W	997
YGL112C	YOL005C	901
YGL112C	YOL135C	975
YGL112C	YOL148C	999
YGL112C	YOR210W	901
YGL112C	YOR224C	900
YGL112C	YPL011C	999
YGL112C	YPL047W	999
YGL112C	YPL129W	995
YGL112C	YPL248C	999
YGL112C	YPL254W	999
YGL112C	YPR070W	998
YGL112C	YPR072W	983
YGL112C	YPR086W	938
YGL112C	YPR187W	901
YGL113W	YDR013W	991
YGL113W	YDR489W	963
YGL113W	YJL072C	947
YGL113W	YJL090C	999
YGL113W	YLR103C	998
YGL113W	YOL146W	965
YGL115W	YBR112C	935
YGL115W	YCL046W	918
YGL115W	YDR028C	999
YGL115W	YDR422C	999
YGL115W	YDR477W	999
YGL115W	YER027C	999
YGL115W	YER129W	999
YGL115W	YER177W	956
YGL115W	YGL035C	980
YGL115W	YGL158W	923
YGL115W	YGL208W	999
YGL115W	YJL089W	907
YGL115W	YML110C	916
YGL115W	YMR207C	914
YGL115W	YMR280C	987
YGL115W	YNR016C	953
YGL115W	YOR018W	983

YGL115W	YOR047C	999
YGL115W	YPL046C	949
YGL116W	YBL084C	999
YGL116W	YBR082C	933
YGL116W	YBR160W	997
YGL116W	YBR200W	976
YGL116W	YDL008W	921
YGL116W	YDL028C	956
YGL116W	YDR113C	999
YGL116W	YEL061C	988
YGL116W	YFR036W	969
YGL116W	YGL003C	991
YGL116W	YGL086W	997
YGL116W	YGL240W	976
YGL116W	YGR188C	997
YGL116W	YHR166C	999
YGL116W	YJL013C	999
YGL116W	YJL030W	999
YGL116W	YKL022C	996
YGL116W	YKL101W	996
YGL116W	YLR102C	980
YGL116W	YLR127C	922
YGL116W	YMR001C	992
YGL116W	YMR055C	962
YGL116W	YNL172W	920
YGL116W	YOR026W	999
YGL116W	YPL203W	903
YGL116W	YPL209C	938
YGL116W	YPL267W	934
YGL116W	YPR119W	998
YGL116W	YPR120C	987
YGL119W	YBR003W	926
YGL119W	YDR197W	904
YGL119W	YDR204W	901
YGL119W	YGR255C	916
YGL119W	YLR201C	992
YGL119W	YML110C	934
YGL119W	YNR041C	904
YGL119W	YOL008W	970
YGL119W	YOL096C	920
YGL119W	YOR125C	912
YGL120C	YAL032C	988
YGL120C	YBL004W	960
YGL120C	YBL072C	938

YGL120C	YBR247C	923
YGL120C	YCL011C	979
YGL120C	YCL037C	993
YGL120C	YCL059C	968
YGL120C	YCR057C	991
YGL120C	YDL014W	999
YGL120C	YDL083C	927
YGL120C	YDL153C	935
YGL120C	YDL209C	990
YGL120C	YDR064W	940
YGL120C	YDR398W	911
YGL120C	YDR416W	974
YGL120C	YEL026W	966
YGL120C	YER029C	982
YGL120C	YER165W	976
YGL120C	YER172C	995
YGL120C	YGL128C	999
YGL120C	YGL171W	915
YGL120C	YGL173C	991
YGL120C	YGR090W	960
YGL120C	YGR128C	922
YGL120C	YGR159C	990
YGL120C	YHR165C	998
YGL120C	YHR169W	931
YGL120C	YHR196W	960
YGL120C	YJL010C	983
YGL120C	YJL069C	910
YGL120C	YJL109C	966
YGL120C	YJL190C	936
YGL120C	YJR002W	918
YGL120C	YJR145C	937
YGL120C	YKL095W	980
YGL120C	YKL173W	999
YGL120C	YKR022C	995
YGL120C	YLL011W	915
YGL120C	YLL036C	998
YGL120C	YLR117C	999
YGL120C	YLR129W	923
YGL120C	YLR175W	978
YGL120C	YLR186W	918
YGL120C	YLR197W	999
YGL120C	YLR222C	941
YGL120C	YLR409C	923
YGL120C	YLR424W	999

YGL120C	YML093W	965
YGL120C	YMR093W	967
YGL120C	YMR128W	983
YGL120C	YMR213W	919
YGL120C	YMR229C	975
YGL120C	YMR290C	981
YGL120C	YNL075W	921
YGL120C	YNL132W	997
YGL120C	YNL224C	998
YGL120C	YOR004W	920
YGL120C	YOR063W	942
YGL120C	YOR096W	939
YGL120C	YOR145C	922
YGL120C	YOR310C	999
YGL120C	YPL012W	964
YGL120C	YPL131W	935
YGL120C	YPL151C	999
YGL120C	YPL217C	966
YGL120C	YPR101W	974
YGL120C	YPR144C	909
YGL121C	YOR371C	919
YGL122C	YBR017C	999
YGL122C	YBR034C	976
YGL122C	YDR381W	976
YGL122C	YKR095W	995
YGL122C	YMR255W	998
YGL122C	YNL016W	979
YGL122C	YOL123W	998
YGL123W	YAL003W	908
YGL123W	YAL035W	936
YGL123W	YBL027W	999
YGL123W	YBL072C	906
YGL123W	YBL087C	983
YGL123W	YBL092W	983
YGL123W	YBR031W	994
YGL123W	YBR048W	998
YGL123W	YBR079C	923
YGL123W	YBR084C-A	994
YGL123W	YBR118W	925
YGL123W	YBR146W	917
YGL123W	YBR181C	997
YGL123W	YBR189W	998
YGL123W	YBR191W	999
YGL123W	YCR031C	999

YGL123W	YDL061C	999
YGL123W	YDL075W	999
YGL123W	YDL082W	938
YGL123W	YDL083C	998
YGL123W	YDL130W	943
YGL123W	YDL136W	954
YGL123W	YDL191W	913
YGL123W	YDL192W	915
YGL123W	YDR012W	998
YGL123W	YDR025W	983
YGL123W	YDR041W	983
YGL123W	YDR064W	999
YGL123W	YDR382W	999
YGL123W	YDR385W	991
YGL123W	YDR418W	999
YGL123W	YDR429C	903
YGL123W	YDR447C	914
YGL123W	YDR450W	995
YGL123W	YDR471W	966
YGL123W	YDR500C	967
YGL123W	YEL054C	921
YGL123W	YER025W	954
YGL123W	YER056C-A	994
YGL123W	YER074W	913
YGL123W	YER102W	978
YGL123W	YER117W	995
YGL123W	YER131W	948
YGL123W	YER165W	962
YGL123W	YFR031C-A	999
YGL123W	YFR032C-A	938
YGL123W	YGL030W	998
YGL123W	YGL031C	952
YGL123W	YGL076C	971
YGL123W	YGL103W	999
YGL123W	YGL147C	986
YGL123W	YGL189C	918
YGL123W	YGR027C	975
YGL123W	YGR034W	976
YGL123W	YGR085C	999
YGL123W	YGR118W	999
YGL123W	YGR148C	995
YGL123W	YGR162W	915
YGL123W	YGR214W	999
YGL123W	YGR220C	905

YGL123W	YHL001W	999
YGL123W	YHL015W	999
YGL123W	YHL033C	999
YGL123W	YHR010W	978
YGL123W	YHR021C	913
YGL123W	YHR203C	995
YGL123W	YIL018W	978
YGL123W	YIL052C	999
YGL123W	YIL069C	913
YGL123W	YIL133C	902
YGL123W	YIL148W	965
YGL123W	YJL136C	955
YGL123W	YJL177W	999
YGL123W	YJL189W	936
YGL123W	YJL190C	999
YGL123W	YJL191W	996
YGL123W	YJR094W-A	975
YGL123W	YJR113C	902
YGL123W	YJR123W	999
YGL123W	YJR145C	976
YGL123W	YKL006W	999
YGL123W	YKL139W	989
YGL123W	YKL156W	969
YGL123W	YKL170W	982
YGL123W	YKL180W	999
YGL123W	YKR057W	906
YGL123W	YKR059W	959
YGL123W	YLL045C	931
YGL123W	YLR029C	991
YGL123W	YLR048W	999
YGL123W	YLR061W	999
YGL123W	YLR075W	999
YGL123W	YLR167W	983
YGL123W	YLR185W	973
YGL123W	YLR249W	948
YGL123W	YLR264W	995
YGL123W	YLR325C	914
YGL123W	YLR333C	927
YGL123W	YLR340W	999
YGL123W	YLR344W	995
YGL123W	YLR367W	994
YGL123W	YLR388W	999
YGL123W	YLR441C	998
YGL123W	YLR448W	995

YGL123W	YML024W	994
YGL123W	YML026C	956
YGL123W	YML063W	987
YGL123W	YML073C	998
YGL123W	YMR121C	933
YGL123W	YMR142C	939
YGL123W	YMR194W	995
YGL123W	YMR230W	938
YGL123W	YMR242C	998
YGL123W	YMR260C	930
YGL123W	YNL067W	994
YGL123W	YNL069C	902
YGL123W	YNL081C	997
YGL123W	YNL096C	912
YGL123W	YNL178W	999
YGL123W	YNL209W	978
YGL123W	YNL301C	978
YGL123W	YNL302C	914
YGL123W	YNR036C	978
YGL123W	YNR037C	996
YGL123W	YOL039W	931
YGL123W	YOL040C	999
YGL123W	YOL120C	931
YGL123W	YOL121C	914
YGL123W	YOL127W	998
YGL123W	YOR063W	999
YGL123W	YOR096W	998
YGL123W	YOR133W	944
YGL123W	YOR167C	976
YGL123W	YOR234C	977
YGL123W	YOR293W	963
YGL123W	YOR312C	945
YGL123W	YOR361C	916
YGL123W	YOR369C	952
YGL123W	YPL037C	999
YGL123W	YPL079W	975
YGL123W	YPL081W	999
YGL123W	YPL131W	999
YGL123W	YPL143W	982
YGL123W	YPL198W	951
YGL123W	YPL220W	972
YGL123W	YPL237W	920
YGL123W	YPL249C-A	933
YGL123W	YPR043W	947

YGL123W	YPR080W	925
YGL123W	YPR102C	998
YGL123W	YPR132W	980
YGL123W	YPR163C	914
YGL124C	YBR131W	999
YGL124C	YGR198W	918
YGL124C	YML001W	921
YGL124C	YOR106W	907
YGL125W	YAL044C	929
YGL125W	YBR084W	933
YGL125W	YBR263W	933
YGL125W	YDR019C	928
YGL125W	YGR204W	936
YGL125W	YKR080W	904
YGL125W	YLR058C	934
YGL125W	YML082W	935
YGL125W	YMR189W	916
YGL125W	YOR074C	957
YGL125W	YOR241W	931
YGL125W	YPL023C	999
YGL126W	YEL043W	919
YGL126W	YOR165W	923
YGL127C	YAL011W	977
YGL127C	YBL093C	999
YGL127C	YBR193C	999
YGL127C	YBR198C	923
YGL127C	YBR231C	975
YGL127C	YBR253W	999
YGL127C	YBR279W	926
YGL127C	YCR081W	996
YGL127C	YDL005C	999
YGL127C	YDL140C	982
YGL127C	YDR138W	996
YGL127C	YDR145W	909
YGL127C	YDR308C	999
YGL127C	YDR334W	907
YGL127C	YDR404C	906
YGL127C	YDR443C	999
YGL127C	YDR460W	946
YGL127C	YDR485C	986
YGL127C	YER022W	999
YGL127C	YGL025C	999
YGL127C	YGL043W	996
YGL127C	YGL070C	987

YGL127C	YGL151W	998
YGL127C	YGR104C	999
YGL127C	YGR186W	919
YGL127C	YGR274C	977
YGL127C	YHR041C	999
YGL127C	YHR058C	999
YGL127C	YHR059W	923
YGL127C	YIL021W	928
YGL127C	YJL140W	946
YGL127C	YJL168C	965
YGL127C	YKR062W	915
YGL127C	YLR071C	999
YGL127C	YLR418C	938
YGL127C	YML041C	987
YGL127C	YMR005W	960
YGL127C	YMR112C	992
YGL127C	YMR236W	924
YGL127C	YNL236W	999
YGL127C	YNR010W	999
YGL127C	YOL012C	942
YGL127C	YOL051W	999
YGL127C	YOL135C	999
YGL127C	YOR151C	983
YGL127C	YOR174W	999
YGL127C	YOR210W	907
YGL127C	YOR224C	905
YGL127C	YPL042C	999
YGL127C	YPL129W	934
YGL127C	YPR070W	999
YGL127C	YPR168W	999
YGL127C	YPR187W	906
YGL128C	YAL032C	999
YGL128C	YCR063W	968
YGL128C	YDL030W	974
YGL128C	YDL209C	974
YGL128C	YDR416W	972
YGL128C	YER029C	992
YGL128C	YER172C	992
YGL128C	YGL120C	999
YGL128C	YGR285C	945
YGL128C	YHR165C	999
YGL128C	YKL173W	993
YGL128C	YKR022C	997
YGL128C	YLL036C	997

YGL128C	YLR117C	998
YGL128C	YLR275W	992
YGL128C	YLR424W	999
YGL128C	YML049C	976
YGL128C	YMR213W	999
YGL128C	YMR288W	974
YGL128C	YNL007C	940
YGL128C	YPL151C	990
YGL128C	YPR101W	972
YGL129C	Q0140	962
YGL129C	YBL090W	988
YGL129C	YBR146W	988
YGL129C	YBR251W	999
YGL129C	YDR036C	999
YGL129C	YDR041W	998
YGL129C	YDR175C	988
YGL129C	YDR337W	988
YGL129C	YDR347W	988
YGL129C	YER050C	986
YGL129C	YGR084C	974
YGL129C	YGR165W	988
YGL129C	YGR215W	988
YGL129C	YHL004W	999
YGL129C	YIL093C	988
YGL129C	YJR101W	988
YGL129C	YJR113C	988
YGL129C	YKL003C	986
YGL129C	YKL155C	999
YGL129C	YMR158W	974
YGL129C	YMR188C	988
YGL129C	YNL137C	988
YGL129C	YNL306W	988
YGL129C	YNR037C	987
YGL129C	YOR158W	988
YGL129C	YOR204W	982
YGL129C	YPL013C	997
YGL129C	YPL118W	988
YGL130W	YAL035W	939
YGL130W	YBR236C	934
YGL130W	YCR038C	900
YGL130W	YDL108W	984
YGL130W	YDL140C	999
YGL130W	YGL173C	976
YGL130W	YML010W	977

YGL130W	YML109W	983
YGL130W	YMR273C	976
YGL130W	YPL228W	999
YGL131C	YMR176W	943
YGL131C	YNL127W	965
YGL133W	YBR089C-A	970
YGL133W	YBR245C	999
YGL133W	YDL002C	980
YGL133W	YDR121W	999
YGL133W	YDR224C	982
YGL133W	YFR013W	990
YGL133W	YFR037C	970
YGL133W	YIL126W	970
YGL133W	YJL065C	999
YGL133W	YJL092W	962
YGL133W	YKR001C	968
YGL133W	YLR176C	963
YGL133W	YOR304W	999
YGL133W	YPL082C	968
YGL134W	YFR034C	941
YGL134W	YLR258W	996
YGL134W	YMR311C	963
YGL134W	YOL096C	910
YGL134W	YOR178C	903
YGL134W	YPL031C	999
YGL135W	YBL027W	990
YGL135W	YBL072C	995
YGL135W	YBL087C	990
YGL135W	YBL092W	993
YGL135W	YBR031W	996
YGL135W	YBR048W	996
YGL135W	YBR084C-A	914
YGL135W	YBR118W	957
YGL135W	YBR142W	940
YGL135W	YBR181C	902
YGL135W	YBR189W	993
YGL135W	YBR191W	991
YGL135W	YCR031C	996
YGL135W	YDL061C	910
YGL135W	YDL075W	999
YGL135W	YDL081C	938
YGL135W	YDL082W	996
YGL135W	YDL083C	967
YGL135W	YDL130W	915

YGL135W	YDL136W	999
YGL135W	YDL191W	918
YGL135W	YDR012W	999
YGL135W	YDR025W	904
YGL135W	YDR064W	995
YGL135W	YDR382W	996
YGL135W	YDR385W	906
YGL135W	YDR418W	999
YGL135W	YDR447C	996
YGL135W	YDR450W	949
YGL135W	YDR471W	996
YGL135W	YDR500C	912
YGL135W	YEL054C	961
YGL135W	YER006W	931
YGL135W	YER056C-A	913
YGL135W	YER074W	995
YGL135W	YER102W	916
YGL135W	YER117W	911
YGL135W	YER131W	914
YGL135W	YFL034C-A	920
YGL135W	YFR031C-A	999
YGL135W	YFR032C-A	928
YGL135W	YGL030W	996
YGL135W	YGL031C	989
YGL135W	YGL076C	999
YGL135W	YGL103W	999
YGL135W	YGL147C	999
YGL135W	YGL189C	914
YGL135W	YGR027C	912
YGL135W	YGR034W	996
YGL135W	YGR085C	999
YGL135W	YGR118W	921
YGL135W	YGR214W	909
YGL135W	YHL001W	996
YGL135W	YHL015W	938
YGL135W	YHL033C	999
YGL135W	YHR010W	915
YGL135W	YHR021C	920
YGL135W	YHR052W	940
YGL135W	YHR141C	999
YGL135W	YHR203C	996
YGL135W	YIL018W	995
YGL135W	YIL052C	913
YGL135W	YIL069C	906

YGL135W	YIL133C	999
YGL135W	YIL148W	905
YGL135W	YJL136C	929
YGL135W	YJL177W	996
YGL135W	YJL189W	905
YGL135W	YJL190C	996
YGL135W	YJL191W	925
YGL135W	YJR094W-A	991
YGL135W	YJR123W	999
YGL135W	YJR145C	930
YGL135W	YKL006W	921
YGL135W	YKL156W	920
YGL135W	YKL180W	999
YGL135W	YKR057W	929
YGL135W	YKR094C	905
YGL135W	YLL045C	997
YGL135W	YLR029C	999
YGL135W	YLR048W	909
YGL135W	YLR061W	923
YGL135W	YLR075W	999
YGL135W	YLR167W	900
YGL135W	YLR185W	990
YGL135W	YLR264W	914
YGL135W	YLR276C	981
YGL135W	YLR287C-A	996
YGL135W	YLR333C	911
YGL135W	YLR340W	999
YGL135W	YLR344W	992
YGL135W	YLR367W	912
YGL135W	YLR388W	911
YGL135W	YLR406C	925
YGL135W	YLR441C	996
YGL135W	YLR448W	996
YGL135W	YML024W	923
YGL135W	YML026C	918
YGL135W	YML056C	979
YGL135W	YML063W	996
YGL135W	YML073C	996
YGL135W	YMR121C	914
YGL135W	YMR142C	909
YGL135W	YMR194W	996
YGL135W	YMR229C	935
YGL135W	YMR230W	926
YGL135W	YMR242C	996

YGL135W	YMR260C	913
YGL135W	YMR290C	971
YGL135W	YNL002C	932
YGL135W	YNL067W	914
YGL135W	YNL069C	996
YGL135W	YNL096C	931
YGL135W	YNL132W	944
YGL135W	YNL162W	932
YGL135W	YNL178W	997
YGL135W	YNL301C	999
YGL135W	YNL302C	922
YGL135W	YNR037C	900
YGL135W	YOL039W	908
YGL135W	YOL040C	944
YGL135W	YOL077C	940
YGL135W	YOL120C	910
YGL135W	YOL121C	923
YGL135W	YOL127W	999
YGL135W	YOR063W	999
YGL135W	YOR096W	995
YGL135W	YOR133W	906
YGL135W	YOR167C	914
YGL135W	YOR182C	910
YGL135W	YOR204W	929
YGL135W	YOR234C	918
YGL135W	YOR293W	926
YGL135W	YOR312C	996
YGL135W	YOR369C	925
YGL135W	YPL012W	940
YGL135W	YPL079W	916
YGL135W	YPL081W	908
YGL135W	YPL090C	902
YGL135W	YPL093W	933
YGL135W	YPL131W	999
YGL135W	YPL143W	998
YGL135W	YPL198W	997
YGL135W	YPL211W	938
YGL135W	YPL220W	999
YGL135W	YPL249C-A	996
YGL135W	YPR043W	922
YGL135W	YPR080W	906
YGL135W	YPR102C	991
YGL135W	YPR132W	921
YGL136C	YHR168W	941

YGL137W	YBL102W	903
YGL137W	YBR162C	962
YGL137W	YDL137W	992
YGL137W	YDL145C	999
YGL137W	YDL192W	996
YGL137W	YDL226C	947
YGL137W	YDR238C	999
YGL137W	YER122C	999
YGL137W	YFL048C	919
YGL137W	YFR051C	999
YGL137W	YGL145W	919
YGL137W	YGL245W	960
YGL137W	YIL004C	975
YGL137W	YIL076W	999
YGL137W	YJR031C	977
YGL137W	YLR078C	976
YGL137W	YLR268W	988
YGL137W	YNL287W	999
YGL137W	YNR006W	901
YGL137W	YPL010W	999
YGL141W	YDL007W	983
YGL141W	YDL147W	954
YGL141W	YDR363W-A	954
YGL141W	YDR394W	954
YGL141W	YDR427W	954
YGL141W	YFR004W	954
YGL141W	YFR010W	996
YGL141W	YGL048C	954
YGL141W	YGR232W	954
YGL141W	YHL030W	954
YGL141W	YHR200W	932
YGL141W	YIL075C	932
YGL141W	YLR421C	954
YGL141W	YOR117W	954
YGL141W	YOR259C	954
YGL141W	YOR261C	954
YGL142C	YML012W	933
YGL143C	YNL063W	972
YGL144C	YER125W	991
YGL144C	YML111W	976
YGL144C	YMR275C	974
YGL144C	YNL307C	968
YGL144C	YOR253W	918
YGL145W	YDR498C	999

YGL145W	YFR051C	942
YGL145W	YGL098W	999
YGL145W	YGL137W	919
YGL145W	YKL196C	966
YGL145W	YLR268W	999
YGL145W	YLR440C	999
YGL145W	YNL258C	999
YGL145W	YNL287W	905
YGL145W	YOR075W	999
YGL147C	YBL027W	999
YGL147C	YBL072C	997
YGL147C	YBL087C	998
YGL147C	YBL092W	999
YGL147C	YBR031W	999
YGL147C	YBR048W	971
YGL147C	YBR084C-A	994
YGL147C	YBR118W	907
YGL147C	YBR181C	998
YGL147C	YBR189W	996
YGL147C	YBR191W	999
YGL147C	YCR031C	996
YGL147C	YDL061C	950
YGL147C	YDL075W	999
YGL147C	YDL081C	954
YGL147C	YDL082W	998
YGL147C	YDL083C	965
YGL147C	YDL136W	999
YGL147C	YDL191W	922
YGL147C	YDR012W	999
YGL147C	YDR025W	971
YGL147C	YDR064W	997
YGL147C	YDR382W	918
YGL147C	YDR385W	910
YGL147C	YDR418W	999
YGL147C	YDR447C	998
YGL147C	YDR450W	998
YGL147C	YDR471W	996
YGL147C	YDR500C	904
YGL147C	YEL054C	916
YGL147C	YER074W	995
YGL147C	YER102W	994
YGL147C	YER117W	977
YGL147C	YER131W	952
YGL147C	YFL034C-A	911

YGL147C	YFR031C-A	999
YGL147C	YFR032C-A	935
YGL147C	YGL030W	997
YGL147C	YGL031C	996
YGL147C	YGL076C	999
YGL147C	YGL103W	999
YGL147C	YGL123W	986
YGL147C	YGL135W	999
YGL147C	YGL189C	908
YGL147C	YGR027C	950
YGL147C	YGR034W	999
YGL147C	YGR085C	999
YGL147C	YGR118W	933
YGL147C	YGR148C	949
YGL147C	YGR214W	956
YGL147C	YHL001W	996
YGL147C	YHL015W	998
YGL147C	YHL033C	999
YGL147C	YHR010W	918
YGL147C	YHR021C	928
YGL147C	YHR141C	999
YGL147C	YHR203C	973
YGL147C	YIL018W	998
YGL147C	YIL052C	912
YGL147C	YIL069C	905
YGL147C	YIL133C	999
YGL147C	YIL148W	904
YGL147C	YJL136C	933
YGL147C	YJL177W	998
YGL147C	YJL189W	915
YGL147C	YJL190C	999
YGL147C	YJL191W	922
YGL147C	YJR094W-A	993
YGL147C	YJR123W	998
YGL147C	YJR145C	973
YGL147C	YKL006W	939
YGL147C	YKL009W	926
YGL147C	YKL156W	928
YGL147C	YKL170W	900
YGL147C	YKL180W	999
YGL147C	YKR057W	926
YGL147C	YKR094C	904
YGL147C	YLL045C	999
YGL147C	YLR029C	999

YGL147C	YLR048W	939
YGL147C	YLR061W	913
YGL147C	YLR075W	994
YGL147C	YLR185W	989
YGL147C	YLR264W	923
YGL147C	YLR325C	958
YGL147C	YLR333C	945
YGL147C	YLR340W	998
YGL147C	YLR344W	997
YGL147C	YLR367W	979
YGL147C	YLR388W	963
YGL147C	YLR406C	997
YGL147C	YLR441C	977
YGL147C	YLR448W	996
YGL147C	YML024W	924
YGL147C	YML026C	922
YGL147C	YML063W	997
YGL147C	YML073C	997
YGL147C	YMR121C	951
YGL147C	YMR142C	952
YGL147C	YMR194W	996
YGL147C	YMR230W	934
YGL147C	YMR242C	999
YGL147C	YMR260C	915
YGL147C	YMR290C	936
YGL147C	YNL067W	932
YGL147C	YNL069C	998
YGL147C	YNL096C	997
YGL147C	YNL162W	925
YGL147C	YNL178W	999
YGL147C	YNL301C	993
YGL147C	YNL302C	918
YGL147C	YNR037C	938
YGL147C	YOL039W	923
YGL147C	YOL040C	999
YGL147C	YOL120C	935
YGL147C	YOL121C	958
YGL147C	YOL127W	999
YGL147C	YOR063W	999
YGL147C	YOR096W	997
YGL147C	YOR133W	910
YGL147C	YOR167C	936
YGL147C	YOR234C	938
YGL147C	YOR293W	936

YGL147C	YOR312C	929
YGL147C	YOR369C	979
YGL147C	YPL079W	950
YGL147C	YPL081W	923
YGL147C	YPL090C	909
YGL147C	YPL093W	931
YGL147C	YPL131W	999
YGL147C	YPL143W	928
YGL147C	YPL198W	997
YGL147C	YPL220W	914
YGL147C	YPL249C-A	996
YGL147C	YPR016C	964
YGL147C	YPR043W	939
YGL147C	YPR080W	907
YGL147C	YPR102C	999
YGL147C	YPR132W	923
YGL148W	YDR127W	915
YGL148W	YER090W	947
YGL148W	YKL211C	955
YGL150C	YBL088C	938
YGL150C	YBR136W	962
YGL150C	YDL002C	999
YGL150C	YDR145W	923
YGL150C	YDR190C	999
YGL150C	YEL044W	940
YGL150C	YER092W	999
YGL150C	YFL013C	999
YGL150C	YFL039C	999
YGL150C	YGR274C	923
YGL150C	YJL081C	999
YGL150C	YLR052W	999
YGL150C	YNL059C	999
YGL150C	YNL215W	981
YGL150C	YOR141C	999
YGL150C	YOR189W	996
YGL150C	YPL129W	999
YGL150C	YPL235W	999
YGL151W	YBL093C	999
YGL151W	YBR193C	997
YGL151W	YBR253W	999
YGL151W	YCR081W	997
YGL151W	YDL005C	999
YGL151W	YDR308C	999
YGL151W	YDR443C	998

YGL151W	YDR448W	994
YGL151W	YER022W	999
YGL151W	YGL025C	999
YGL151W	YGL127C	998
YGL151W	YGR104C	999
YGL151W	YHR041C	998
YGL151W	YHR058C	997
YGL151W	YLR071C	998
YGL151W	YML007W	992
YGL151W	YMR112C	997
YGL151W	YNL025C	999
YGL151W	YNL236W	999
YGL151W	YNR010W	998
YGL151W	YOL051W	999
YGL151W	YOL135C	999
YGL151W	YOR174W	997
YGL151W	YPL042C	995
YGL151W	YPR070W	999
YGL151W	YPR168W	999
YGL153W	YDL065C	979
YGL153W	YDR142C	999
YGL153W	YDR171W	927
YGL153W	YDR244W	999
YGL153W	YDR265W	994
YGL153W	YDR329C	945
YGL153W	YGR077C	992
YGL153W	YGR239C	902
YGL153W	YHR016C	928
YGL153W	YHR160C	906
YGL153W	YJL210W	995
YGL153W	YLR191W	999
YGL153W	YMR026C	999
YGL153W	YNL214W	999
YGL153W	YNL329C	959
YGL153W	YOL044W	969
YGL153W	YOL147C	921
YGL154C	YBR115C	999
YGL154C	YGL254W	914
YGL154C	YNR050C	939
YGL155W	YKL019W	999
YGL155W	YLR229C	940
YGL155W	YPR165W	983
YGL156W	YKL103C	963
YGL156W	YOL082W	979

YGL156W	YPR110C	928
YGL158W	YGL115W	923
YGL158W	YLR113W	936
YGL158W	YOR018W	929
YGL160W	YGL071W	942
YGL161C	YDR084C	999
YGL161C	YGL198W	999
YGL161C	YGL212W	942
YGL161C	YGR172C	982
YGL161C	YHR105W	941
YGL161C	YPL095C	998
YGL162W	YKL183W	998
YGL163C	YBR073W	999
YGL163C	YBR114W	918
YGL163C	YDL059C	971
YGL163C	YDR004W	973
YGL163C	YDR076W	989
YGL163C	YDR369C	972
YGL163C	YDR386W	951
YGL163C	YER095W	999
YGL163C	YER171W	913
YGL163C	YER179W	997
YGL163C	YJL092W	999
YGL163C	YJR043C	968
YGL163C	YKL113C	989
YGL163C	YKR082W	946
YGL163C	YLR032W	979
YGL163C	YLR234W	995
YGL163C	YML032C	985
YGL163C	YML061C	955
YGL163C	YMR106C	909
YGL163C	YMR190C	945
YGL163C	YMR224C	973
YGL163C	YNL250W	986
YGL163C	YOR005C	941
YGL163C	YOR144C	985
YGL163C	YPL020C	966
YGL163C	YPL022W	945
YGL164C	YLR293C	903
YGL166W	YHR053C	975
YGL166W	YOR210W	900
YGL167C	YBR106W	973
YGL167C	YEL031W	986
YGL167C	YFL045C	949

YGL167C	YHR008C	993
YGL167C	YHR050W	939
YGL167C	YHR191C	901
YGL167C	YJR104C	999
YGL167C	YLR220W	986
YGL167C	YOL122C	909
YGL168W	YHR191C	901
YGL170C	YOL091W	991
YGL170C	YOR177C	939
YGL171W	YBR247C	967
YGL171W	YCL059C	983
YGL171W	YCR057C	990
YGL171W	YDL060W	909
YGL171W	YDL148C	923
YGL171W	YDL153C	946
YGL171W	YDR299W	947
YGL171W	YDR324C	916
YGL171W	YDR398W	914
YGL171W	YDR449C	999
YGL171W	YGL120C	915
YGL171W	YGL173C	949
YGL171W	YGR090W	994
YGL171W	YGR128C	935
YGL171W	YGR145W	997
YGL171W	YHR073W	944
YGL171W	YHR148W	997
YGL171W	YHR196W	910
YGL171W	YJL010C	985
YGL171W	YJL069C	911
YGL171W	YJL109C	911
YGL171W	YJR002W	963
YGL171W	YKR060W	920
YGL171W	YLL011W	932
YGL171W	YLR003C	978
YGL171W	YLR129W	998
YGL171W	YLR175W	916
YGL171W	YLR186W	997
YGL171W	YLR197W	923
YGL171W	YLR222C	951
YGL171W	YLR409C	937
YGL171W	YML093W	922
YGL171W	YMR093W	959
YGL171W	YMR128W	916
YGL171W	YMR229C	995

YGL171W	YNL075W	936
YGL171W	YNL132W	929
YGL171W	YOL010W	922
YGL171W	YOR004W	947
YGL171W	YOR078W	952
YGL171W	YOR145C	916
YGL171W	YPL012W	916
YGL171W	YPL126W	935
YGL171W	YPL217C	938
YGL171W	YPR112C	936
YGL171W	YPR137W	912
YGL171W	YPR144C	921
YGL172W	YAR002W	939
YGL172W	YBL079W	942
YGL172W	YDL116W	998
YGL172W	YDL207W	962
YGL172W	YDR159W	966
YGL172W	YDR192C	985
YGL172W	YDR465C	916
YGL172W	YER105C	943
YGL172W	YER107C	947
YGL172W	YER110C	961
YGL172W	YFR002W	999
YGL172W	YGL092W	996
YGL172W	YGR119C	999
YGL172W	YGR218W	933
YGL172W	YIL115C	921
YGL172W	YJL039C	949
YGL172W	YJL041W	999
YGL172W	YJL061W	988
YGL172W	YJR042W	998
YGL172W	YKL057C	993
YGL172W	YKL068W	986
YGL172W	YKR082W	998
YGL172W	YLR335W	998
YGL172W	YLR347C	999
YGL172W	YML092C	999
YGL172W	YML103C	943
YGL172W	YMR047C	999
YGL172W	YMR129W	968
YGL172W	YMR255W	934
YGL172W	YOR098C	948
YGL172W	YOR257W	961
YGL172W	YPL125W	961

YGL173C	YBL026W	999
YGL173C	YBL034C	964
YGL173C	YBR189W	925
YGL173C	YCL011C	976
YGL173C	YCL037C	974
YGL173C	YCR020C-A	983
YGL173C	YCR077C	999
YGL173C	YDL014W	940
YGL173C	YDL160C	998
YGL173C	YDR378C	999
YGL173C	YDR381W	930
YGL173C	YDR432W	973
YGL173C	YDR496C	983
YGL173C	YEL015W	980
YGL173C	YER102W	923
YGL173C	YER111C	917
YGL173C	YER112W	999
YGL173C	YER146W	999
YGL173C	YGL049C	972
YGL173C	YGL120C	991
YGL173C	YGL130W	976
YGL173C	YGL171W	949
YGL173C	YGL213C	994
YGL173C	YGL246C	914
YGL173C	YGR054W	999
YGL173C	YGR074W	903
YGL173C	YGR086C	984
YGL173C	YGR162W	987
YGL173C	YHL034C	990
YGL173C	YHR073W	925
YGL173C	YJL124C	999
YGL173C	YJR022W	999
YGL173C	YJR145C	925
YGL173C	YKL023W	990
YGL173C	YKL188C	946
YGL173C	YLR147C	909
YGL173C	YLR175W	990
YGL173C	YLR275W	929
YGL173C	YLR398C	999
YGL173C	YLR438C-A	999
YGL173C	YML056C	975
YGL173C	YMR080C	970
YGL173C	YMR268C	966
YGL173C	YMR290C	927

YGL173C	YNL118C	997
YGL173C	YNL147W	999
YGL173C	YOL139C	998
YGL173C	YOL149W	994
YGL173C	YOR048C	996
YGL173C	YOR204W	923
YGL173C	YPL004C	988
YGL173C	YPR140W	958
YGL173C	YPR178W	976
YGL173C	YPR182W	906
YGL173C	YPR189W	992
YGL174W	YIR005W	999
YGL174W	YKL074C	969
YGL174W	YLR016C	981
YGL174W	YMR240C	974
YGL174W	YMR288W	974
YGL175C	YDR217C	965
YGL175C	YHL022C	990
YGL175C	YKL113C	984
YGL175C	YLR263W	939
YGL175C	YMR190C	935
YGL178W	YAL021C	999
YGL178W	YBL016W	947
YGL178W	YBL105C	978
YGL178W	YDL160C	930
YGL178W	YLR150W	970
YGL178W	YLR452C	999
YGL178W	YNR052C	999
YGL179C	YER129W	996
YGL179C	YKL048C	982
YGL180W	YCR079W	910
YGL180W	YDL149W	958
YGL180W	YDR022C	950
YGL180W	YLR423C	999
YGL180W	YLR431C	946
YGL180W	YPL166W	964
YGL180W	YPR049C	999
YGL180W	YPR185W	999
YGL181W	YBL007C	939
YGL181W	YDR388W	914
YGL181W	YER063W	974
YGL181W	YFR024C-A	969
YGL181W	YIL122W	942
YGL181W	YIR006C	915

YGL181W	YOR140W	963
YGL183C	YER179W	956
YGL183C	YGL033W	999
YGL183C	YHL022C	963
YGL183C	YIL072W	936
YGL183C	YLR263W	939
YGL184C	YGR155W	974
YGL184C	YJR130C	941
YGL186C	YMR096W	930
YGL187C	Q0045	997
YGL187C	Q0250	978
YGL187C	Q0275	990
YGL187C	YBL045C	978
YGL187C	YDL067C	999
YGL187C	YGL191W	992
YGL187C	YHR051W	998
YGL187C	YIL111W	978
YGL187C	YLR038C	981
YGL187C	YLR395C	982
YGL187C	YMR256C	989
YGL187C	YNL052W	997
YGL189C	YBL027W	907
YGL189C	YBL072C	910
YGL189C	YBL087C	916
YGL189C	YBL092W	906
YGL189C	YBR031W	921
YGL189C	YBR048W	914
YGL189C	YBR084C-A	907
YGL189C	YBR118W	904
YGL189C	YBR189W	917
YGL189C	YBR191W	910
YGL189C	YCR031C	921
YGL189C	YDL061C	912
YGL189C	YDL075W	909
YGL189C	YDL081C	908
YGL189C	YDL082W	914
YGL189C	YDL130W	908
YGL189C	YDL136W	918
YGL189C	YDL191W	918
YGL189C	YDR012W	921
YGL189C	YDR025W	914
YGL189C	YDR064W	941
YGL189C	YDR382W	917
YGL189C	YDR418W	923

YGL189C	YDR429C	903
YGL189C	YDR447C	921
YGL189C	YDR450W	903
YGL189C	YDR471W	911
YGL189C	YDR500C	917
YGL189C	YEL054C	923
YGL189C	YER056C-A	910
YGL189C	YER074W	921
YGL189C	YER102W	910
YGL189C	YER117W	916
YGL189C	YER131W	902
YGL189C	YFL034C-A	912
YGL189C	YFR032C-A	996
YGL189C	YGL030W	935
YGL189C	YGL031C	907
YGL189C	YGL076C	923
YGL189C	YGL123W	918
YGL189C	YGL135W	914
YGL189C	YGL147C	908
YGL189C	YGR027C	916
YGL189C	YGR034W	910
YGL189C	YGR085C	901
YGL189C	YGR118W	921
YGL189C	YGR148C	907
YGL189C	YGR214W	905
YGL189C	YHL001W	922
YGL189C	YHL015W	912
YGL189C	YHL033C	911
YGL189C	YHR010W	911
YGL189C	YHR021C	921
YGL189C	YHR141C	919
YGL189C	YHR203C	913
YGL189C	YIL052C	910
YGL189C	YIL069C	921
YGL189C	YIL133C	919
YGL189C	YJL136C	921
YGL189C	YJL177W	905
YGL189C	YJL190C	914
YGL189C	YJL191W	921
YGL189C	YJR094W-A	921
YGL189C	YJR123W	933
YGL189C	YJR145C	913
YGL189C	YKL006W	922
YGL189C	YKL156W	921

YGL189C	YKL180W	905
YGL189C	YKR057W	922
YGL189C	YLL045C	911
YGL189C	YLR029C	921
YGL189C	YLR048W	905
YGL189C	YLR061W	915
YGL189C	YLR075W	934
YGL189C	YLR185W	918
YGL189C	YLR264W	902
YGL189C	YLR287C-A	905
YGL189C	YLR325C	921
YGL189C	YLR333C	915
YGL189C	YLR340W	919
YGL189C	YLR344W	910
YGL189C	YLR367W	914
YGL189C	YLR388W	912
YGL189C	YLR406C	909
YGL189C	YLR435W	983
YGL189C	YLR441C	919
YGL189C	YLR448W	917
YGL189C	YML024W	921
YGL189C	YML026C	903
YGL189C	YML063W	918
YGL189C	YML073C	916
YGL189C	YMR121C	922
YGL189C	YMR142C	912
YGL189C	YMR194W	920
YGL189C	YMR230W	925
YGL189C	YMR242C	920
YGL189C	YMR260C	907
YGL189C	YNL067W	908
YGL189C	YNL069C	911
YGL189C	YNL096C	926
YGL189C	YNL162W	919
YGL189C	YNL178W	935
YGL189C	YNL301C	908
YGL189C	YNL302C	921
YGL189C	YOL040C	931
YGL189C	YOL120C	908
YGL189C	YOL121C	921
YGL189C	YOL127W	926
YGL189C	YOR063W	916
YGL189C	YOR096W	926
YGL189C	YOR167C	902

YGL189C	YOR182C	905
YGL189C	YOR234C	920
YGL189C	YOR293W	925
YGL189C	YOR312C	920
YGL189C	YOR369C	942
YGL189C	YPL079W	910
YGL189C	YPL081W	917
YGL189C	YPL131W	921
YGL189C	YPL143W	921
YGL189C	YPL198W	923
YGL189C	YPL220W	914
YGL189C	YPL249C-A	920
YGL189C	YPR043W	921
YGL189C	YPR080W	904
YGL189C	YPR102C	901
YGL189C	YPR132W	921
YGL190C	YAL016W	999
YGL190C	YBR160W	943
YGL190C	YDL134C	999
YGL190C	YDL188C	999
YGL190C	YDR435C	932
YGL190C	YER155C	912
YGL190C	YGR098C	913
YGL190C	YJL187C	907
YGL190C	YJL194W	971
YGL190C	YJR138W	901
YGL190C	YKL022C	907
YGL190C	YKL048C	905
YGL190C	YML109W	999
YGL190C	YMR036C	984
YGL190C	YMR273C	999
YGL190C	YOR014W	989
YGL190C	YPL152W	976
YGL191W	Q0045	978
YGL191W	Q0250	978
YGL191W	Q0275	978
YGL191W	YDL067C	978
YGL191W	YGL187C	992
YGL191W	YHR051W	999
YGL191W	YIL111W	978
YGL191W	YLR038C	991
YGL191W	YLR395C	981
YGL191W	YMR256C	981
YGL191W	YNL052W	998

YGL192W	YJR094C	914
YGL194C	YAL013W	933
YGL194C	YBR095C	931
YGL194C	YBR103W	999
YGL194C	YBR112C	996
YGL194C	YCR033W	999
YGL194C	YDR155C	999
YGL194C	YDR207C	924
YGL194C	YIL084C	924
YGL194C	YIL112W	999
YGL194C	YJL168C	979
YGL194C	YKR029C	999
YGL194C	YMR263W	977
YGL194C	YMR273C	989
YGL194C	YNL021W	994
YGL194C	YNL097C	932
YGL194C	YNL330C	998
YGL194C	YOL004W	967
YGL194C	YOL012C	976
YGL194C	YOL068C	994
YGL194C	YPL181W	975
YGL194C	YPR068C	931
YGL195W	YBR025C	966
YGL195W	YDL143W	972
YGL195W	YDR152W	913
YGL195W	YDR214W	963
YGL195W	YDR283C	999
YGL195W	YEL009C	972
YGL195W	YER110C	970
YGL195W	YFL045C	962
YGL195W	YFR009W	999
YGL195W	YIL094C	973
YGL195W	YKL029C	974
YGL195W	YKL104C	999
YGL195W	YLR180W	976
YGL195W	YOR136W	975
YGL197W	YBR225W	999
YGL197W	YDL047W	926
YGL197W	YDL155W	962
YGL197W	YJL098W	992
YGL197W	YLR310C	999
YGL197W	YMR308C	913
YGL197W	YNL307C	910
YGL198W	YDR084C	999

YGL198W	YER031C	913
YGL198W	YFL038C	900
YGL198W	YGL161C	999
YGL198W	YGR172C	958
YGL198W	YLR262C	910
YGL198W	YNL263C	941
YGL200C	YAL007C	999
YGL200C	YAR002C-A	999
YGL200C	YDL195W	913
YGL200C	YGL054C	937
YGL200C	YIL109C	970
YGL200C	YKL073W	911
YGL200C	YLR208W	987
YGL200C	YML012W	999
YGL200C	YMR307W	923
YGL200C	YOR016C	988
YGL200C	YPL218W	974
YGL200C	YPR181C	980
YGL201C	YAR007C	928
YGL201C	YBL023C	998
YGL201C	YBR060C	953
YGL201C	YBR202W	999
YGL201C	YDL017W	998
YGL201C	YDR489W	980
YGL201C	YEL032W	990
YGL201C	YER161C	980
YGL201C	YIL150C	999
YGL201C	YIR008C	904
YGL201C	YJL194W	990
YGL201C	YJR046W	983
YGL201C	YKL045W	901
YGL201C	YLR103C	999
YGL201C	YLR274W	996
YGL201C	YML065W	991
YGL201C	YNL102W	941
YGL201C	YNL261W	926
YGL201C	YPR019W	999
YGL201C	YPR162C	904
YGL202W	YDR380W	948
YGL202W	YGL026C	958
YGL203C	YMR123W	915
YGL205W	YBR041W	903
YGL205W	YER015W	933
YGL205W	YIL009W	901

YGL205W	YIL160C	941
YGL205W	YKL188C	916
YGL205W	YKR009C	988
YGL205W	YMR246W	901
YGL205W	YOR317W	926
YGL205W	YPL028W	905
YGL205W	YPL147W	920
YGL206C	YCR088W	900
YGL206C	YDL161W	996
YGL206C	YDL192W	982
YGL206C	YDR099W	922
YGL206C	YDR153C	995
YGL206C	YDR320C	979
YGL206C	YDR358W	976
YGL206C	YER177W	930
YGL206C	YGR167W	999
YGL206C	YGR241C	956
YGL206C	YHR108W	999
YGL206C	YHR161C	956
YGL206C	YIL094C	928
YGL206C	YJR058C	918
YGL206C	YJR125C	998
YGL206C	YKL135C	998
YGL206C	YKR001C	924
YGL206C	YKR026C	907
YGL206C	YLL039C	961
YGL206C	YLR170C	996
YGL206C	YLR206W	974
YGL206C	YNL106C	989
YGL206C	YNL238W	935
YGL206C	YNL243W	979
YGL206C	YOR036W	968
YGL206C	YOR109W	992
YGL206C	YOR181W	928
YGL206C	YOR329C	985
YGL206C	YPL259C	988
YGL206C	YPR029C	988
YGL207W	YAL021C	935
YGL207W	YBL035C	981
YGL207W	YBL052C	997
YGL207W	YBR009C	988
YGL207W	YBR010W	924
YGL207W	YBR089C-A	993
YGL207W	YBR245C	971

YGL207W	YBR279W	999
YGL207W	YDR143C	998
YGL207W	YDR225W	989
YGL207W	YER111C	923
YGL207W	YER148W	911
YGL207W	YER164W	999
YGL207W	YGL019W	999
YGL207W	YGL048C	997
YGL207W	YGL244W	999
YGL207W	YGR063C	962
YGL207W	YGR116W	918
YGL207W	YGR252W	966
YGL207W	YGR270W	986
YGL207W	YHR164C	963
YGL207W	YIL021W	946
YGL207W	YIL035C	999
YGL207W	YIR008C	981
YGL207W	YJL168C	985
YGL207W	YKL028W	908
YGL207W	YKL045W	981
YGL207W	YKR062W	908
YGL207W	YLR182W	958
YGL207W	YLR418C	999
YGL207W	YML010W	973
YGL207W	YML069W	999
YGL207W	YNL030W	928
YGL207W	YNL031C	939
YGL207W	YNL102W	999
YGL207W	YOL012C	993
YGL207W	YOL054W	999
YGL207W	YOL145C	999
YGL207W	YOR039W	999
YGL207W	YOR061W	999
YGL207W	YOR123C	999
YGL207W	YPL256C	910
YGL207W	YPR052C	999
YGL207W	YPR135W	908
YGL208W	YDR422C	999
YGL208W	YDR477W	999
YGL208W	YER027C	999
YGL208W	YER129W	974
YGL208W	YGL115W	999
YGL208W	YKL048C	928
YGL209W	YGL035C	996

YGL210W	YER031C	995
YGL210W	YER136W	957
YGL210W	YGL106W	940
YGL210W	YGR172C	956
YGL210W	YLR330W	903
YGL210W	YMR218C	986
YGL210W	YNL272C	940
YGL212W	YBL050W	988
YGL212W	YBR080C	950
YGL212W	YDL077C	997
YGL212W	YDR080W	986
YGL212W	YGL161C	942
YGL212W	YKL196C	997
YGL212W	YLR093C	999
YGL212W	YLR148W	999
YGL212W	YLR396C	999
YGL212W	YML001W	969
YGL212W	YMR197C	999
YGL212W	YMR231W	992
YGL212W	YNL263C	962
YGL212W	YOR036W	999
YGL212W	YOR106W	999
YGL212W	YPL045W	986
YGL212W	YPL240C	919
YGL212W	YPL246C	941
YGL213C	YGL173C	994
YGL213C	YHL022C	999
YGL213C	YHR069C	951
YGL213C	YHR157W	935
YGL213C	YJL124C	908
YGL213C	YLR398C	999
YGL213C	YNL232W	907
YGL213C	YOR076C	993
YGL213C	YPR189W	999
YGL215W	YBR160W	943
YGL215W	YDL179W	970
YGL215W	YER059W	964
YGL215W	YPL031C	997
YGL215W	YPL219W	961
YGL215W	YPL256C	946
YGL216W	YDR150W	973
YGL216W	YEL061C	965
YGL216W	YER016W	973
YGL216W	YJL030W	983

YGL216W	YKR054C	995
YGL216W	YLR254C	955
YGL216W	YMR198W	924
YGL216W	YMR294W	939
YGL216W	YOR269W	931
YGL216W	YPL155C	984
YGL216W	YPR141C	998
YGL219C	YHR194W	917
YGL219C	YOR147W	917
YGL220W	YDR098C	996
YGL220W	YER174C	966
YGL220W	YLL029W	974
YGL221C	YDR176W	924
YGL222C	YNL118C	935
YGL222C	YOL149W	929
YGL223C	YER157W	999
YGL223C	YGL005C	999
YGL223C	YGR120C	999
YGL223C	YIL004C	966
YGL223C	YLR026C	964
YGL223C	YML071C	999
YGL223C	YNL041C	999
YGL223C	YNL051W	999
YGL223C	YPR105C	999
YGL224C	YGL043W	926
YGL226C-A	YDL232W	999
YGL226C-A	YEL002C	999
YGL226C-A	YGL022W	999
YGL226C-A	YGR227W	988
YGL226C-A	YHR039C	921
YGL226C-A	YJL002C	999
YGL226C-A	YLR378C	996
YGL226C-A	YML019W	999
YGL226C-A	YMR149W	999
YGL226C-A	YOR085W	999
YGL226C-A	YOR103C	999
YGL226C-A	YPL227C	938
YGL227W	YBL049W	929
YGL227W	YBR105C	999
YGL227W	YCL039W	999
YGL227W	YDL176W	918
YGL227W	YDR255C	999
YGL227W	YIL017C	999
YGL227W	YIL097W	999

YGL227W	YMR135C	999
YGL229C	YBR196C	900
YGL233W	YAL041W	908
YGL233W	YBR102C	999
YGL233W	YBR200W	997
YGL233W	YDR164C	998
YGL233W	YDR166C	999
YGL233W	YDR363W-A	903
YGL233W	YER008C	999
YGL233W	YER087C-B	985
YGL233W	YFL005W	999
YGL233W	YGR009C	996
YGL233W	YIL068C	999
YGL233W	YJL085W	998
YGL233W	YLR166C	999
YGL233W	YLR229C	991
YGL233W	YNL272C	999
YGL233W	YNR049C	998
YGL233W	YOR326W	984
YGL233W	YPL232W	910
YGL233W	YPR017C	960
YGL233W	YPR032W	999
YGL233W	YPR055W	999
YGL234W	YAR015W	987
YGL234W	YBR049C	918
YGL234W	YDR408C	999
YGL234W	YGR061C	998
YGL234W	YLR028C	915
YGL234W	YLR359W	945
YGL234W	YMR120C	914
YGL234W	YMR300C	998
YGL234W	YOR128C	999
YGL236C	YMR023C	997
YGL237C	YBL021C	999
YGL237C	YKL109W	997
YGL237C	YLR256W	940
YGL237C	YMR312W	955
YGL237C	YOR358W	999
YGL238W	YDR123C	983
YGL238W	YDR170C	922
YGL238W	YER120W	922
YGL238W	YLR293C	999
YGL238W	YLR335W	970
YGL238W	YNL189W	999

YGL240W	YBL084C	999
YGL240W	YBR082C	979
YGL240W	YBR160W	981
YGL240W	YDL008W	999
YGL240W	YDR118W	999
YGL240W	YDR260C	999
YGL240W	YFR036W	999
YGL240W	YGL003C	994
YGL240W	YGL116W	976
YGL240W	YGR229C	936
YGL240W	YHR166C	999
YGL240W	YIR025W	999
YGL240W	YKL022C	999
YGL240W	YLL039C	903
YGL240W	YLR102C	999
YGL240W	YLR127C	999
YGL240W	YNL098C	964
YGL240W	YNL172W	999
YGL240W	YOR249C	999
YGL240W	YPL209C	984
YGL241W	YBL002W	999
YGL241W	YBL003C	999
YGL241W	YDR224C	999
YGL241W	YDR225W	999
YGL241W	YER148W	999
YGL241W	YHR088W	921
YGL241W	YKR048C	999
YGL241W	YLR293C	966
YGL241W	YOL012C	995
YGL241W	YPR086W	972
YGL241W	YPR093C	980
YGL242C	YBR109C	986
YGL242C	YMR109W	999
YGL244W	YAL011W	918
YGL244W	YBR231C	916
YGL244W	YBR253W	970
YGL244W	YBR279W	999
YGL244W	YDL108W	984
YGL244W	YDL140C	982
YGL244W	YDR308C	970
YGL244W	YDR334W	906
YGL244W	YDR485C	906
YGL244W	YER022W	970
YGL244W	YER148W	999

YGL244W	YER164W	990
YGL244W	YGL019W	966
YGL244W	YGL207W	999
YGL244W	YGR063C	989
YGL244W	YGR104C	996
YGL244W	YGR186W	927
YGL244W	YHR041C	993
YGL244W	YIL035C	997
YGL244W	YJL168C	987
YGL244W	YKL139W	995
YGL244W	YLR085C	946
YGL244W	YLR175W	923
YGL244W	YLR418C	999
YGL244W	YML010W	995
YGL244W	YML041C	920
YGL244W	YML069W	999
YGL244W	YMR247C	908
YGL244W	YMR277W	975
YGL244W	YOL012C	986
YGL244W	YOL051W	970
YGL244W	YOL145C	999
YGL244W	YOR039W	968
YGL244W	YOR061W	968
YGL244W	YOR123C	999
YGL244W	YOR151C	979
YGL244W	YPL129W	908
YGL245W	YER086W	962
YGL245W	YGL105W	999
YGL245W	YGL137W	960
YGL245W	YGR124W	919
YGL245W	YGR162W	946
YGL245W	YGR264C	999
YGL245W	YOL033W	922
YGL245W	YPR010C	975
YGL246C	YDR289C	924
YGL246C	YDR447C	974
YGL246C	YGL173C	914
YGL246C	YLL045C	975
YGL246C	YLR398C	927
YGL246C	YOR048C	999
YGL248W	YDR454C	906
YGL248W	YFL033C	900
YGL248W	YJL005W	964
YGL248W	YNL098C	995

YGL248W	YOR360C	999
YGL249W	YBR186W	947
YGL249W	YDL132W	983
YGL249W	YLR263W	930
YGL249W	YOR351C	933
YGL250W	YDR510W	942
YGL251C	YHR086W	904
YGL251C	YNL127W	943
YGL252C	YCR005C	990
YGL252C	YDL132W	966
YGL252C	YDL194W	902
YGL252C	YDR054C	999
YGL252C	YDR099W	999
YGL252C	YER177W	997
YGL252C	YJR066W	973
YGL252C	YLR027C	903
YGL252C	YNL006W	947
YGL252C	YNL076W	999
YGL252C	YNR001C	928
YGL252C	YOL067C	983
YGL253W	YBR019C	909
YGL253W	YBR126C	993
YGL253W	YBR193C	904
YGL253W	YBR196C	923
YGL253W	YCL040W	997
YGL253W	YDL035C	967
YGL253W	YDR028C	973
YGL253W	YDR477W	995
YGL253W	YER003C	903
YGL253W	YER133W	952
YGL253W	YFR053C	999
YGL253W	YGL035C	998
YGL253W	YGR240C	929
YGL253W	YJL153C	909
YGL253W	YJL155C	906
YGL253W	YJR090C	967
YGL253W	YKL038W	986
YGL253W	YLR377C	951
YGL253W	YMR205C	906
YGL254W	YGL154C	914
YGL254W	YJR090C	993
YGL254W	YPL092W	991
YGL255W	YJL056C	997
YGL255W	YKL175W	949

YGL255W	YLR130C	996
YGL256W	YBR145W	979
YGL256W	YDL168W	981
YGL256W	YER073W	956
YGL256W	YGR087C	900
YGL256W	YHL032C	930
YGL256W	YLR044C	901
YGL256W	YLR134W	902
YGL256W	YMR083W	980
YGL256W	YMR169C	956
YGL256W	YMR170C	924
YGL256W	YMR303C	980
YGL256W	YOL086C	979
YGL256W	YOR374W	966
YGL256W	YPL061W	924
YGL258W	YOR387C	999
YGR002C	YAL011W	988
YGR002C	YBR231C	999
YGR002C	YDR190C	999
YGR002C	YDR225W	912
YGR002C	YDR334W	999
YGR002C	YDR359C	999
YGR002C	YDR485C	994
YGR002C	YEL018W	999
YGR002C	YFL024C	999
YGR002C	YFL039C	999
YGR002C	YHR090C	999
YGR002C	YJL081C	999
YGR002C	YJR082C	996
YGR002C	YLR085C	999
YGR002C	YLR385C	999
YGR002C	YLR399C	994
YGR002C	YML041C	997
YGR002C	YNL107W	999
YGR002C	YNL136W	998
YGR002C	YOL012C	999
YGR002C	YOR244W	999
YGR002C	YPL235W	999
YGR002C	YPR023C	998
YGR003W	YBR114W	984
YGR003W	YDR139C	912
YGR003W	YIL001W	999
YGR003W	YJR052W	984
YGR003W	YOL133W	998

YGR003W	YPL046C	945
YGR004W	YLR324W	909
YGR004W	YPR028W	998
YGR005C	YBR017C	909
YGR005C	YBR154C	999
YGR005C	YBR198C	940
YGR005C	YBR253W	983
YGR005C	YBR279W	998
YGR005C	YCR042C	970
YGR005C	YDL115C	987
YGR005C	YDL140C	999
YGR005C	YDR404C	999
YGR005C	YER022W	995
YGR005C	YGL043W	998
YGR005C	YGL070C	999
YGR005C	YGR063C	987
YGR005C	YGR104C	999
YGR005C	YGR186W	999
YGR005C	YHR041C	978
YGR005C	YHR143W-A	920
YGR005C	YIL021W	999
YGR005C	YJL140W	999
YGR005C	YLR005W	980
YGR005C	YLR418C	998
YGR005C	YML010W	999
YGR005C	YMR140W	998
YGR005C	YMR277W	958
YGR005C	YOL005C	999
YGR005C	YOL051W	991
YGR005C	YOR151C	999
YGR005C	YOR210W	998
YGR005C	YOR224C	999
YGR005C	YPL129W	999
YGR005C	YPR056W	984
YGR005C	YPR086W	984
YGR005C	YPR187W	999
YGR006W	YDR088C	999
YGR006W	YDR364C	999
YGR006W	YHR165C	988
YGR006W	YKR086W	922
YGR008C	YDL181W	957
YGR009C	YAL030W	999
YGR009C	YBL050W	924
YGR009C	YBL106C	919

YGR009C	YDR164C	999
YGR009C	YER008C	997
YGR009C	YER143W	985
YGR009C	YFL005W	999
YGR009C	YGL233W	996
YGR009C	YIL068C	994
YGR009C	YIL118W	997
YGR009C	YLR166C	992
YGR009C	YLR229C	937
YGR009C	YMR017W	973
YGR009C	YMR183C	999
YGR009C	YNR049C	999
YGR009C	YOR326W	990
YGR009C	YOR327C	999
YGR009C	YPL232W	999
YGR009C	YPR032W	999
YGR009C	YPR055W	994
YGR010W	YDL042C	992
YGR010W	YFR047C	906
YGR010W	YLR328W	999
YGR010W	YLR438W	991
YGR010W	YNL129W	989
YGR010W	YOR209C	960
YGR012W	YAL012W	978
YGR012W	YFR055W	944
YGR012W	YLR303W	966
YGR012W	YNL247W	909
YGR013W	YBR055C	990
YGR013W	YBR119W	999
YGR013W	YDL087C	999
YGR013W	YDR080W	950
YGR013W	YDR235W	999
YGR013W	YDR240C	999
YGR013W	YER029C	996
YGR013W	YER172C	994
YGR013W	YFL017W-A	987
YGR013W	YGR074W	999
YGR013W	YHR086W	999
YGR013W	YHR165C	989
YGR013W	YIL061C	999
YGR013W	YKL012W	999
YGR013W	YKL173W	989
YGR013W	YLR147C	999
YGR013W	YLR275W	999

YGR013W	YLR298C	999
YGR013W	YML046W	999
YGR013W	YML049C	976
YGR013W	YMR125W	999
YGR013W	YPL178W	999
YGR013W	YPR057W	970
YGR013W	YPR178W	976
YGR013W	YPR182W	999
YGR014W	YAL041W	991
YGR014W	YER118C	998
YGR014W	YIL169C	901
YGR014W	YIR019C	920
YGR014W	YLR113W	905
YGR014W	YLR229C	918
YGR014W	YMR317W	956
YGR014W	YPL211W	900
YGR017W	YLR410W	994
YGR019W	YBR006W	997
YGR019W	YDL170W	960
YGR019W	YDL210W	937
YGR019W	YHR124W	998
YGR019W	YIR023W	932
YGR019W	YMR250W	987
YGR019W	YOR374W	900
YGR020C	YBR011C	909
YGR020C	YBR127C	999
YGR020C	YDL185W	999
YGR020C	YEL027W	923
YGR020C	YEL051W	999
YGR020C	YHR026W	965
YGR020C	YHR039C-A	999
YGR020C	YKL080W	909
YGR020C	YLR447C	999
YGR020C	YMR054W	999
YGR020C	YOR270C	999
YGR020C	YOR332W	999
YGR020C	YPL234C	920
YGR020C	YPR036W	999
YGR021W	YHR165C	976
YGR023W	YLR332W	993
YGR023W	YNL327W	999
YGR023W	YPR165W	913
YGR027C	YAL035W	909
YGR027C	YBL027W	913

YGR027C	YBL072C	921
YGR027C	YBL087C	939
YGR027C	YBL092W	967
YGR027C	YBR031W	956
YGR027C	YBR048W	924
YGR027C	YBR079C	915
YGR027C	YBR084C-A	937
YGR027C	YBR118W	908
YGR027C	YBR181C	917
YGR027C	YBR189W	999
YGR027C	YBR191W	999
YGR027C	YCR031C	988
YGR027C	YDL061C	994
YGR027C	YDL075W	974
YGR027C	YDL081C	941
YGR027C	YDL082W	944
YGR027C	YDL083C	981
YGR027C	YDL130W	923
YGR027C	YDL136W	945
YGR027C	YDL191W	917
YGR027C	YDR012W	968
YGR027C	YDR025W	913
YGR027C	YDR064W	997
YGR027C	YDR382W	986
YGR027C	YDR385W	907
YGR027C	YDR418W	987
YGR027C	YDR447C	937
YGR027C	YDR450W	915
YGR027C	YDR471W	988
YGR027C	YDR500C	939
YGR027C	YEL054C	917
YGR027C	YER025W	938
YGR027C	YER056C-A	973
YGR027C	YER074W	916
YGR027C	YER102W	950
YGR027C	YER117W	949
YGR027C	YER131W	999
YGR027C	YER165W	918
YGR027C	YFR031C-A	925
YGR027C	YFR032C-A	936
YGR027C	YGL030W	999
YGR027C	YGL031C	983
YGR027C	YGL076C	976
YGR027C	YGL103W	994

YGR027C	YGL123W	975
YGR027C	YGL135W	912
YGR027C	YGL147C	950
YGR027C	YGL189C	916
YGR027C	YGR034W	924
YGR027C	YGR085C	994
YGR027C	YGR118W	998
YGR027C	YGR148C	999
YGR027C	YGR214W	998
YGR027C	YHL001W	999
YGR027C	YHL015W	994
YGR027C	YHL033C	997
YGR027C	YHR010W	976
YGR027C	YHR021C	921
YGR027C	YHR141C	935
YGR027C	YHR203C	932
YGR027C	YIL018W	913
YGR027C	YIL052C	999
YGR027C	YIL069C	916
YGR027C	YIL133C	922
YGR027C	YIL148W	938
YGR027C	YJL136C	974
YGR027C	YJL177W	999
YGR027C	YJL189W	934
YGR027C	YJL190C	928
YGR027C	YJL191W	980
YGR027C	YJR094W-A	975
YGR027C	YJR123W	999
YGR027C	YJR145C	905
YGR027C	YKL006W	998
YGR027C	YKL156W	980
YGR027C	YKL180W	999
YGR027C	YKR057W	931
YGR027C	YKR059W	923
YGR027C	YLL045C	967
YGR027C	YLR029C	994
YGR027C	YLR048W	982
YGR027C	YLR061W	989
YGR027C	YLR075W	973
YGR027C	YLR167W	938
YGR027C	YLR185W	932
YGR027C	YLR264W	994
YGR027C	YLR287C-A	997
YGR027C	YLR325C	942

YGR027C	YLR333C	964
YGR027C	YLR340W	968
YGR027C	YLR344W	963
YGR027C	YLR367W	973
YGR027C	YLR388W	999
YGR027C	YLR406C	906
YGR027C	YLR441C	928
YGR027C	YLR448W	981
YGR027C	YML024W	984
YGR027C	YML026C	915
YGR027C	YML063W	995
YGR027C	YML073C	924
YGR027C	YMR121C	913
YGR027C	YMR142C	947
YGR027C	YMR194W	982
YGR027C	YMR230W	954
YGR027C	YMR242C	944
YGR027C	YMR260C	901
YGR027C	YNL067W	997
YGR027C	YNL069C	908
YGR027C	YNL096C	918
YGR027C	YNL162W	919
YGR027C	YNL178W	996
YGR027C	YNL301C	941
YGR027C	YNL302C	922
YGR027C	YOL039W	947
YGR027C	YOL040C	955
YGR027C	YOL120C	917
YGR027C	YOL121C	931
YGR027C	YOL127W	999
YGR027C	YOR063W	979
YGR027C	YOR096W	981
YGR027C	YOR133W	907
YGR027C	YOR167C	970
YGR027C	YOR234C	980
YGR027C	YOR293W	979
YGR027C	YOR312C	941
YGR027C	YOR361C	914
YGR027C	YOR369C	948
YGR027C	YPL079W	972
YGR027C	YPL081W	947
YGR027C	YPL090C	900
YGR027C	YPL131W	960
YGR027C	YPL143W	978

YGR027C	YPL198W	927
YGR027C	YPL220W	941
YGR027C	YPL237W	947
YGR027C	YPL249C-A	945
YGR027C	YPR043W	940
YGR027C	YPR080W	923
YGR027C	YPR102C	999
YGR027C	YPR132W	947
YGR027C	YPR163C	913
YGR028W	YJL020C	935
YGR029W	YKL084W	902
YGR029W	YKL195W	999
YGR030C	YAL033W	999
YGR030C	YBL018C	999
YGR030C	YBR167C	999
YGR030C	YBR257W	999
YGR030C	YDR478W	991
YGR030C	YHR062C	999
YGR030C	YIR015W	995
YGR030C	YLR145W	980
YGR030C	YNL221C	999
YGR030C	YNL282W	952
YGR032W	YBL105C	912
YGR032W	YGR282C	924
YGR032W	YKL035W	900
YGR032W	YLR342W	999
YGR032W	YPR054W	953
YGR032W	YPR165W	999
YGR033C	YIL022W	966
YGR033C	YJL143W	999
YGR033C	YLR008C	980
YGR033C	YMR203W	963
YGR033C	YNL131W	999
YGR033C	YNR017W	999
YGR033C	YPL063W	999
YGR034W	YBL027W	999
YGR034W	YBL072C	920
YGR034W	YBL087C	989
YGR034W	YBL092W	999
YGR034W	YBR031W	998
YGR034W	YBR048W	998
YGR034W	YBR084C-A	990
YGR034W	YBR118W	905
YGR034W	YBR181C	996

YGR034W	YBR189W	916
YGR034W	YBR191W	995
YGR034W	YCR031C	996
YGR034W	YDL061C	962
YGR034W	YDL075W	996
YGR034W	YDL081C	953
YGR034W	YDL082W	996
YGR034W	YDL083C	964
YGR034W	YDL130W	912
YGR034W	YDL136W	996
YGR034W	YDL191W	932
YGR034W	YDR012W	998
YGR034W	YDR025W	972
YGR034W	YDR064W	996
YGR034W	YDR382W	996
YGR034W	YDR385W	909
YGR034W	YDR418W	931
YGR034W	YDR447C	998
YGR034W	YDR450W	998
YGR034W	YDR471W	995
YGR034W	YDR500C	913
YGR034W	YEL054C	931
YGR034W	YER056C-A	911
YGR034W	YER074W	995
YGR034W	YER102W	920
YGR034W	YER117W	977
YGR034W	YER131W	997
YGR034W	YFL034C-A	920
YGR034W	YFR031C-A	998
YGR034W	YFR032C-A	996
YGR034W	YGL014W	972
YGR034W	YGL030W	997
YGR034W	YGL031C	996
YGR034W	YGL076C	997
YGR034W	YGL103W	997
YGR034W	YGL123W	976
YGR034W	YGL135W	996
YGR034W	YGL147C	999
YGR034W	YGL189C	910
YGR034W	YGR027C	924
YGR034W	YGR085C	999
YGR034W	YGR118W	924
YGR034W	YGR148C	923
YGR034W	YGR214W	921

YGR034W	YHL001W	995
YGR034W	YHL015W	944
YGR034W	YHL033C	996
YGR034W	YHR010W	913
YGR034W	YHR021C	925
YGR034W	YHR141C	998
YGR034W	YHR203C	999
YGR034W	YIL018W	998
YGR034W	YIL052C	911
YGR034W	YIL069C	995
YGR034W	YIL133C	998
YGR034W	YIL148W	908
YGR034W	YJL136C	931
YGR034W	YJL177W	998
YGR034W	YJL189W	905
YGR034W	YJL190C	999
YGR034W	YJL191W	925
YGR034W	YJR094W-A	938
YGR034W	YJR123W	996
YGR034W	YJR145C	979
YGR034W	YKL006W	924
YGR034W	YKL156W	925
YGR034W	YKL180W	998
YGR034W	YKR057W	931
YGR034W	YKR094C	908
YGR034W	YLL045C	997
YGR034W	YLR029C	997
YGR034W	YLR048W	911
YGR034W	YLR061W	924
YGR034W	YLR075W	997
YGR034W	YLR167W	902
YGR034W	YLR185W	913
YGR034W	YLR264W	916
YGR034W	YLR287C-A	995
YGR034W	YLR325C	950
YGR034W	YLR333C	919
YGR034W	YLR340W	998
YGR034W	YLR344W	997
YGR034W	YLR367W	974
YGR034W	YLR388W	969
YGR034W	YLR406C	927
YGR034W	YLR441C	956
YGR034W	YLR448W	996
YGR034W	YML024W	923

YGR034W	YML026C	924
YGR034W	YML063W	996
YGR034W	YML073C	996
YGR034W	YMR121C	942
YGR034W	YMR142C	995
YGR034W	YMR194W	995
YGR034W	YMR230W	927
YGR034W	YMR242C	998
YGR034W	YMR260C	917
YGR034W	YNL067W	982
YGR034W	YNL069C	997
YGR034W	YNL096C	996
YGR034W	YNL162W	936
YGR034W	YNL178W	999
YGR034W	YNL301C	996
YGR034W	YNL302C	997
YGR034W	YNR037C	933
YGR034W	YOL039W	910
YGR034W	YOL040C	999
YGR034W	YOL120C	925
YGR034W	YOL121C	949
YGR034W	YOL127W	999
YGR034W	YOR063W	999
YGR034W	YOR096W	995
YGR034W	YOR133W	909
YGR034W	YOR167C	929
YGR034W	YOR182C	908
YGR034W	YOR234C	998
YGR034W	YOR293W	936
YGR034W	YOR312C	996
YGR034W	YOR369C	959
YGR034W	YPL079W	933
YGR034W	YPL081W	907
YGR034W	YPL090C	995
YGR034W	YPL131W	988
YGR034W	YPL143W	923
YGR034W	YPL198W	934
YGR034W	YPL220W	996
YGR034W	YPL249C-A	996
YGR034W	YPR043W	938
YGR034W	YPR080W	905
YGR034W	YPR102C	984
YGR034W	YPR132W	924
YGR035C	YKL002W	944

YGR036C	YBR109C	938
YGR036C	YBR243C	956
YGR036C	YMR013C	978
YGR036C	YPL227C	956
YGR036C	YPR183W	952
YGR038W	YGR089W	923
YGR040W	YAL040C	906
YGR040W	YBL016W	999
YGR040W	YBR083W	992
YGR040W	YCL027W	999
YGR040W	YDL159W	999
YGR040W	YDR103W	999
YGR040W	YDR168W	972
YGR040W	YDR480W	999
YGR040W	YER109C	909
YGR040W	YHR030C	907
YGR040W	YHR084W	999
YGR040W	YJL128C	987
YGR040W	YLR362W	999
YGR040W	YLR452C	991
YGR040W	YNL053W	968
YGR040W	YOR212W	985
YGR040W	YPL049C	999
YGR040W	YPL089C	924
YGR041W	YCR038C	948
YGR041W	YLR353W	999
YGR041W	YOR299W	910
YGR041W	YOR301W	991
YGR043C	YBR117C	959
YGR043C	YLR354C	910
YGR043C	YPR074C	958
YGR044C	YER111C	982
YGR044C	YHR014W	902
YGR044C	YLR182W	982
YGR047C	YAL001C	999
YGR047C	YBR089C-A	907
YGR047C	YBR123C	999
YGR047C	YDL150W	999
YGR047C	YDR362C	999
YGR047C	YER148W	966
YGR047C	YGL044C	984
YGR047C	YGR246C	999
YGR047C	YHR143W-A	984
YGR047C	YLR115W	928

YGR047C	YLR277C	928
YGR047C	YNL039W	999
YGR047C	YNR003C	984
YGR047C	YOR110W	999
YGR047C	YOR250C	916
YGR047C	YPL007C	999
YGR047C	YPR052C	956
YGR048W	YBR170C	999
YGR048W	YBR201W	988
YGR048W	YDL126C	999
YGR048W	YDL190C	949
YGR048W	YFL044C	930
YGR048W	YGR178C	903
YGR048W	YIR033W	935
YGR048W	YKL020C	918
YGR048W	YKL213C	965
YGR048W	YKR002W	973
YGR048W	YML013W	950
YGR049W	YFL009W	935
YGR054W	YBL072C	974
YGR054W	YDL060W	972
YGR054W	YDR025W	972
YGR054W	YDR064W	976
YGR054W	YGL173C	999
YGR054W	YMR290C	974
YGR054W	YNL207W	997
YGR054W	YOR063W	976
YGR054W	YOR204W	909
YGR056W	YBR245C	962
YGR056W	YCR020W-B	999
YGR056W	YCR052W	999
YGR056W	YDR303C	999
YGR056W	YFR037C	999
YGR056W	YGR275W	988
YGR056W	YHR056C	999
YGR056W	YIL126W	999
YGR056W	YKR008W	999
YGR056W	YLR033W	999
YGR056W	YLR321C	999
YGR056W	YLR357W	999
YGR056W	YML127W	999
YGR056W	YMR033W	999
YGR056W	YMR091C	999
YGR056W	YPR034W	999

YGR057C	YKL015W	981
YGR057C	YKL176C	992
YGR057C	YLR208W	991
YGR058W	YDL203C	925
YGR058W	YGR136W	931
YGR058W	YNL047C	903
YGR060W	YER044C	998
YGR060W	YGL001C	999
YGR060W	YGL012W	998
YGR060W	YGL040C	974
YGR060W	YGR175C	999
YGR060W	YHR007C	999
YGR060W	YHR072W	999
YGR060W	YHR190W	999
YGR060W	YLR056W	999
YGR060W	YLR100W	999
YGR060W	YML008C	999
YGR060W	YMR015C	999
YGR060W	YMR202W	999
YGR060W	YNL280C	930
YGR060W	YOR278W	975
YGR061C	YDR408C	961
YGR061C	YGL234W	998
YGR061C	YLR386W	955
YGR061C	YMR300C	983
YGR061C	YOR128C	931
YGR061C	YOR158W	929
YGR062C	Q0250	922
YGR062C	YDL107W	983
YGR062C	YOR266W	978
YGR063C	YBL008W	992
YGR063C	YBL046W	969
YGR063C	YBR154C	987
YGR063C	YBR279W	947
YGR063C	YDL140C	999
YGR063C	YDR138W	930
YGR063C	YDR224C	912
YGR063C	YDR404C	999
YGR063C	YER151C	970
YGR063C	YGL043W	999
YGR063C	YGL058W	929
YGR063C	YGL070C	987
YGR063C	YGL086W	931
YGR063C	YGL207W	962

YGR063C	YGL244W	989
YGR063C	YGR005C	987
YGR063C	YGR116W	999
YGR063C	YGR140W	959
YGR063C	YGR186W	990
YGR063C	YIL021W	991
YGR063C	YJL140W	987
YGR063C	YJR017C	950
YGR063C	YKL139W	901
YGR063C	YML010W	999
YGR063C	YML069W	959
YGR063C	YML095C	937
YGR063C	YNL201C	969
YGR063C	YOL005C	987
YGR063C	YOR151C	998
YGR063C	YOR195W	917
YGR063C	YPL240C	919
YGR063C	YPR133C	962
YGR063C	YPR161C	975
YGR063C	YPR187W	987
YGR067C	YDR427W	998
YGR067C	YFR050C	999
YGR068C	YER125W	991
YGR070W	YLR229C	964
YGR070W	YPR165W	995
YGR071C	YPL128C	987
YGR072W	YDR172W	994
YGR072W	YHR077C	999
YGR072W	YMR080C	999
YGR072W	YNL244C	929
YGR072W	YOL149W	937
YGR074W	YBL026W	999
YGR074W	YBL074C	994
YGR074W	YBR055C	999
YGR074W	YBR119W	995
YGR074W	YBR154C	923
YGR074W	YDL030W	999
YGR074W	YDL051W	906
YGR074W	YDL087C	988
YGR074W	YDL098C	976
YGR074W	YDR235W	988
YGR074W	YDR240C	999
YGR074W	YDR378C	931
YGR074W	YDR473C	997

YGR074W	YEL026W	944
YGR074W	YER029C	999
YGR074W	YER112W	981
YGR074W	YER146W	944
YGR074W	YER172C	999
YGR074W	YFL017W-A	999
YGR074W	YGL070C	941
YGR074W	YGL173C	903
YGR074W	YGR013W	999
YGR074W	YGR091W	999
YGR074W	YHR086W	997
YGR074W	YHR165C	999
YGR074W	YIL061C	993
YGR074W	YJL124C	921
YGR074W	YJL203W	999
YGR074W	YJR022W	999
YGR074W	YKL012W	997
YGR074W	YKL173W	999
YGR074W	YKL183W	958
YGR074W	YLR147C	999
YGR074W	YLR275W	999
YGR074W	YLR298C	999
YGR074W	YLR438C-A	903
YGR074W	YML046W	999
YGR074W	YML049C	997
YGR074W	YMR125W	999
YGR074W	YMR240C	998
YGR074W	YMR288W	913
YGR074W	YNL147W	948
YGR074W	YOL005C	914
YGR074W	YOR151C	901
YGR074W	YOR159C	999
YGR074W	YOR210W	950
YGR074W	YOR224C	941
YGR074W	YOR308C	989
YGR074W	YPL178W	906
YGR074W	YPL213W	999
YGR074W	YPR057W	986
YGR074W	YPR082C	997
YGR074W	YPR178W	978
YGR074W	YPR182W	999
YGR074W	YPR187W	938
YGR075C	YBL026W	992
YGR075C	YBR055C	984

YGR075C	YBR152W	999
YGR075C	YDL098C	999
YGR075C	YDR473C	995
YGR075C	YER029C	974
YGR075C	YER172C	999
YGR075C	YGR091W	972
YGR075C	YHR165C	982
YGR075C	YKL173W	974
YGR075C	YOR308C	972
YGR075C	YPR178W	972
YGR076C	YDR405W	981
YGR076C	YGR220C	950
YGR077C	YDR142C	924
YGR077C	YDR244W	999
YGR077C	YDR265W	963
YGR077C	YGL153W	992
YGR077C	YJL210W	993
YGR077C	YKL197C	912
YGR077C	YLR191W	993
YGR077C	YMR026C	992
YGR077C	YNL214W	993
YGR078C	YBL034C	951
YGR078C	YDR183W	982
YGR078C	YEL003W	999
YGR078C	YFL037W	946
YGR078C	YFL039C	966
YGR078C	YHR191C	902
YGR078C	YIL142W	900
YGR078C	YJL179W	999
YGR078C	YJR064W	901
YGR078C	YLR200W	999
YGR078C	YML085C	997
YGR078C	YML094W	999
YGR078C	YML124C	999
YGR078C	YNL153C	999
YGR078C	YOL012C	974
YGR078C	YOR265W	997
YGR080W	YFL039C	996
YGR080W	YIL034C	936
YGR080W	YKL007W	999
YGR080W	YLL050C	944
YGR081C	YBR189W	985
YGR081C	YBR247C	999
YGR081C	YCL059C	915

YGR081C	YDL060W	999
YGR081C	YDR299W	923
YGR081C	YJR002W	910
YGR081C	YMR229C	954
YGR081C	YMR308C	996
YGR081C	YNL075W	910
YGR081C	YPL012W	999
YGR081C	YPL217C	920
YGR082W	YMR060C	972
YGR082W	YMR203W	999
YGR082W	YNL070W	989
YGR082W	YNL121C	999
YGR082W	YNL131W	999
YGR082W	YOR045W	958
YGR082W	YPR133W-A	991
YGR083C	YBR159W	988
YGR083C	YDR037W	917
YGR083C	YDR211W	999
YGR083C	YDR283C	999
YGR083C	YER025W	999
YGR083C	YJR007W	999
YGR083C	YKR026C	999
YGR083C	YLR291C	999
YGR083C	YNL265C	987
YGR083C	YOR260W	999
YGR083C	YPL237W	999
YGR084C	Q0140	962
YGR084C	YBL090W	974
YGR084C	YBR146W	988
YGR084C	YBR251W	999
YGR084C	YDR036C	999
YGR084C	YDR041W	995
YGR084C	YDR175C	974
YGR084C	YDR337W	974
YGR084C	YDR347W	974
YGR084C	YGL129C	974
YGR084C	YGR165W	974
YGR084C	YGR215W	974
YGR084C	YHL004W	999
YGR084C	YIL093C	974
YGR084C	YJR101W	974
YGR084C	YJR113C	974
YGR084C	YKL003C	970
YGR084C	YKL155C	974

YGR084C	YMR158W	974
YGR084C	YMR188C	988
YGR084C	YNL137C	988
YGR084C	YNL306W	974
YGR084C	YNR037C	972
YGR084C	YOR158W	988
YGR084C	YPL013C	993
YGR084C	YPL118W	974
YGR085C	YAL035W	903
YGR085C	YBL027W	998
YGR085C	YBL072C	994
YGR085C	YBL087C	987
YGR085C	YBL092W	999
YGR085C	YBR031W	999
YGR085C	YBR048W	998
YGR085C	YBR084C-A	985
YGR085C	YBR118W	907
YGR085C	YBR142W	938
YGR085C	YBR181C	997
YGR085C	YBR189W	999
YGR085C	YBR191W	999
YGR085C	YCR031C	998
YGR085C	YDL014W	948
YGR085C	YDL061C	985
YGR085C	YDL075W	999
YGR085C	YDL081C	933
YGR085C	YDL082W	997
YGR085C	YDL083C	968
YGR085C	YDL130W	923
YGR085C	YDL136W	996
YGR085C	YDL191W	911
YGR085C	YDR012W	999
YGR085C	YDR025W	998
YGR085C	YDR041W	965
YGR085C	YDR064W	999
YGR085C	YDR087C	923
YGR085C	YDR382W	999
YGR085C	YDR385W	916
YGR085C	YDR418W	999
YGR085C	YDR447C	996
YGR085C	YDR450W	996
YGR085C	YDR471W	998
YGR085C	YDR500C	920
YGR085C	YEL054C	912

YGR085C	YER006W	941
YGR085C	YER056C-A	972
YGR085C	YER074W	995
YGR085C	YER102W	996
YGR085C	YER117W	990
YGR085C	YER131W	962
YGR085C	YFL034C-A	913
YGR085C	YFR031C-A	999
YGR085C	YFR032C-A	997
YGR085C	YGL030W	999
YGR085C	YGL031C	998
YGR085C	YGL076C	998
YGR085C	YGL103W	999
YGR085C	YGL123W	999
YGR085C	YGL135W	999
YGR085C	YGL147C	999
YGR085C	YGL189C	901
YGR085C	YGR027C	994
YGR085C	YGR034W	999
YGR085C	YGR118W	994
YGR085C	YGR148C	999
YGR085C	YGR214W	978
YGR085C	YHL001W	999
YGR085C	YHL015W	999
YGR085C	YHL033C	999
YGR085C	YHR010W	969
YGR085C	YHR021C	920
YGR085C	YHR141C	997
YGR085C	YHR147C	912
YGR085C	YHR203C	999
YGR085C	YIL018W	999
YGR085C	YIL052C	994
YGR085C	YIL069C	996
YGR085C	YIL133C	997
YGR085C	YIL148W	927
YGR085C	YJL136C	981
YGR085C	YJL177W	999
YGR085C	YJL189W	944
YGR085C	YJL190C	999
YGR085C	YJL191W	977
YGR085C	YJR094W-A	972
YGR085C	YJR123W	999
YGR085C	YJR145C	998
YGR085C	YKL006W	996

YGR085C	YKL156W	960
YGR085C	YKL170W	981
YGR085C	YKL180W	999
YGR085C	YKR057W	921
YGR085C	YKR081C	990
YGR085C	YKR094C	905
YGR085C	YLL045C	998
YGR085C	YLR029C	998
YGR085C	YLR048W	995
YGR085C	YLR061W	981
YGR085C	YLR075W	999
YGR085C	YLR150W	961
YGR085C	YLR167W	931
YGR085C	YLR185W	921
YGR085C	YLR264W	979
YGR085C	YLR325C	916
YGR085C	YLR333C	932
YGR085C	YLR340W	999
YGR085C	YLR344W	993
YGR085C	YLR367W	999
YGR085C	YLR388W	997
YGR085C	YLR406C	917
YGR085C	YLR432W	978
YGR085C	YLR441C	996
YGR085C	YLR448W	999
YGR085C	YML024W	978
YGR085C	YML026C	934
YGR085C	YML063W	999
YGR085C	YML073C	996
YGR085C	YMR121C	925
YGR085C	YMR142C	997
YGR085C	YMR194W	998
YGR085C	YMR230W	961
YGR085C	YMR242C	997
YGR085C	YMR260C	912
YGR085C	YMR290C	940
YGR085C	YNL061W	955
YGR085C	YNL067W	997
YGR085C	YNL069C	997
YGR085C	YNL081C	962
YGR085C	YNL096C	996
YGR085C	YNL112W	976
YGR085C	YNL132W	938
YGR085C	YNL162W	922

YGR085C	YNL178W	999
YGR085C	YNL301C	997
YGR085C	YNL302C	918
YGR085C	YNR037C	982
YGR085C	YOL039W	940
YGR085C	YOL040C	999
YGR085C	YOL120C	922
YGR085C	YOL121C	928
YGR085C	YOL127W	999
YGR085C	YOR063W	999
YGR085C	YOR096W	999
YGR085C	YOR133W	916
YGR085C	YOR167C	955
YGR085C	YOR198C	982
YGR085C	YOR204W	933
YGR085C	YOR234C	999
YGR085C	YOR293W	972
YGR085C	YOR294W	996
YGR085C	YOR312C	996
YGR085C	YOR369C	925
YGR085C	YPL079W	988
YGR085C	YPL081W	934
YGR085C	YPL090C	997
YGR085C	YPL093W	937
YGR085C	YPL131W	999
YGR085C	YPL143W	999
YGR085C	YPL198W	997
YGR085C	YPL220W	997
YGR085C	YPL249C-A	997
YGR085C	YPR043W	937
YGR085C	YPR080W	907
YGR085C	YPR102C	999
YGR085C	YPR132W	939
YGR086C	YDR490C	999
YGR086C	YGL173C	984
YGR086C	YKL142W	999
YGR086C	YLR429W	983
YGR086C	YML052W	946
YGR086C	YOL100W	992
YGR086C	YPL004C	999
YGR087C	YGL256W	900
YGR087C	YLR044C	969
YGR087C	YLR134W	969
YGR088W	YDL204W	948

YGR088W	YDR256C	925
YGR088W	YHR008C	993
YGR088W	YJR104C	983
YGR089W	YEL043W	953
YGR089W	YGR038W	923
YGR089W	YGR120C	923
YGR089W	YOR224C	911
YGR090W	YBL004W	999
YGR090W	YBL072C	940
YGR090W	YBR048W	977
YGR090W	YBR189W	977
YGR090W	YBR247C	993
YGR090W	YCL031C	999
YGR090W	YCL059C	985
YGR090W	YCR057C	999
YGR090W	YDL014W	999
YGR090W	YDL060W	960
YGR090W	YDL148C	999
YGR090W	YDR064W	981
YGR090W	YDR280W	923
YGR090W	YDR299W	992
YGR090W	YDR324C	999
YGR090W	YDR449C	999
YGR090W	YER082C	999
YGR090W	YER102W	927
YGR090W	YGL019W	999
YGR090W	YGL120C	960
YGR090W	YGL171W	994
YGR090W	YGR128C	999
YGR090W	YGR145W	997
YGR090W	YHR148W	999
YGR090W	YHR196W	982
YGR090W	YIL035C	999
YGR090W	YJL010C	983
YGR090W	YJL069C	999
YGR090W	YJL109C	999
YGR090W	YJR002W	999
YGR090W	YJR145C	980
YGR090W	YKL088W	956
YGR090W	YKR060W	994
YGR090W	YLR003C	991
YGR090W	YLR129W	999
YGR090W	YLR186W	999
YGR090W	YLR197W	997

YGR090W	YLR222C	997
YGR090W	YLR223C	999
YGR090W	YLR409C	999
YGR090W	YMR093W	997
YGR090W	YMR128W	999
YGR090W	YMR229C	999
YGR090W	YMR290C	992
YGR090W	YNL132W	999
YGR090W	YOR004W	923
YGR090W	YOR039W	999
YGR090W	YOR061W	999
YGR090W	YOR063W	945
YGR090W	YOR078W	982
YGR090W	YOR096W	981
YGR090W	YOR145C	990
YGR090W	YOR206W	905
YGR090W	YOR312C	976
YGR090W	YPL012W	994
YGR090W	YPL126W	999
YGR090W	YPL217C	998
YGR090W	YPL266W	993
YGR090W	YPR112C	924
YGR090W	YPR137W	983
YGR090W	YPR144C	999
YGR091W	YBL026W	998
YGR091W	YBR055C	999
YGR091W	YBR152W	997
YGR091W	YCR077C	974
YGR091W	YDL030W	976
YGR091W	YDL098C	974
YGR091W	YDL160C	972
YGR091W	YDR283C	984
YGR091W	YDR378C	999
YGR091W	YDR473C	999
YGR091W	YER029C	999
YGR091W	YER112W	996
YGR091W	YER172C	999
YGR091W	YFL017W-A	929
YGR091W	YGR074W	999
YGR091W	YGR075C	972
YGR091W	YHR165C	999
YGR091W	YJL124C	975
YGR091W	YJL203W	974
YGR091W	YJR022W	963

YGR091W	YKL173W	999
YGR091W	YLR147C	998
YGR091W	YLR275W	999
YGR091W	YLR424W	974
YGR091W	YLR438C-A	954
YGR091W	YML046W	990
YGR091W	YML049C	978
YGR091W	YMR240C	976
YGR091W	YMR288W	976
YGR091W	YNL147W	988
YGR091W	YOR308C	999
YGR091W	YPL213W	976
YGR091W	YPR082C	999
YGR091W	YPR178W	999
YGR091W	YPR182W	928
YGR092W	YAL021C	999
YGR092W	YAR019C	999
YGR092W	YBR127C	911
YGR092W	YDL155W	962
YGR092W	YDL220C	973
YGR092W	YFR028C	999
YGR092W	YHR152W	999
YGR092W	YIL106W	999
YGR092W	YJL076W	999
YGR092W	YLR079W	999
YGR092W	YML064C	998
YGR092W	YMR001C	999
YGR092W	YMR055C	934
YGR092W	YNL098C	971
YGR092W	YNR052C	985
YGR092W	YOR117W	911
YGR092W	YPL242C	910
YGR092W	YPR111W	999
YGR092W	YPR119W	998
YGR093W	YKL149C	998
YGR094W	YGL112C	980
YGR094W	YHR208W	911
YGR094W	YIL143C	901
YGR094W	YJR148W	913
YGR094W	YMR113W	925
YGR094W	YMR234W	908
YGR094W	YOR151C	915
YGR094W	YOR207C	911
YGR094W	YPR010C	916

YGR094W	YPR110C	912
YGR094W	YPR187W	911
YGR095C	YCR035C	999
YGR095C	YDL111C	999
YGR095C	YDR280W	999
YGR095C	YGR158C	999
YGR095C	YGR195W	999
YGR095C	YHR069C	999
YGR095C	YHR081W	999
YGR095C	YNL189W	993
YGR095C	YNL232W	999
YGR095C	YNL265C	913
YGR095C	YNR024W	959
YGR095C	YOL021C	999
YGR095C	YOL142W	999
YGR095C	YOR001W	999
YGR095C	YOR076C	999
YGR097W	YDL140C	986
YGR097W	YDR167W	982
YGR097W	YDR443C	910
YGR097W	YGR274C	960
YGR097W	YHR206W	921
YGR097W	YML098W	911
YGR097W	YMR005W	965
YGR097W	YNL025C	934
YGR097W	YNR010W	911
YGR098C	YAL024C	967
YGR098C	YDL003W	999
YGR098C	YDR113C	999
YGR098C	YFL008W	900
YGR098C	YFR028C	966
YGR098C	YGL190C	913
YGR098C	YGR163W	941
YGR098C	YHR152W	917
YGR098C	YJL074C	928
YGR098C	YMR001C	979
YGR098C	YOR195W	991
YGR099W	YKL033W	966
YGR099W	YPL235W	959
YGR100W	YKL209C	936
YGR100W	YLR262C	979
YGR101W	YKR066C	976
YGR101W	YOL052C	908
YGR101W	YOR211C	996

YGR103W	YBL087C	974
YGR103W	YCL054W	988
YGR103W	YDL014W	973
YGR103W	YDL031W	999
YGR103W	YDL075W	930
YGR103W	YDL082W	936
YGR103W	YDR060W	999
YGR103W	YDR087C	992
YGR103W	YDR101C	996
YGR103W	YER006W	999
YGR103W	YER011W	929
YGR103W	YER126C	989
YGR103W	YFL002C	981
YGR103W	YFR001W	953
YGR103W	YFR031C-A	993
YGR103W	YGL030W	953
YGR103W	YGL076C	954
YGR103W	YGL111W	999
YGR103W	YGR245C	999
YGR103W	YHR052W	999
YGR103W	YHR066W	997
YGR103W	YHR088W	997
YGR103W	YHR197W	998
YGR103W	YIL018W	996
YGR103W	YIR026C	926
YGR103W	YKL009W	998
YGR103W	YKL021C	989
YGR103W	YKL172W	984
YGR103W	YKR081C	999
YGR103W	YLL008W	999
YGR103W	YLL045C	991
YGR103W	YLR002C	996
YGR103W	YLR009W	997
YGR103W	YLR029C	937
YGR103W	YLR074C	960
YGR103W	YLR106C	992
YGR103W	YLR347C	973
YGR103W	YMR049C	999
YGR103W	YMR131C	991
YGR103W	YMR229C	986
YGR103W	YMR290C	999
YGR103W	YNL002C	999
YGR103W	YNL061W	997
YGR103W	YNL110C	999

YGR103W	YNL182C	989
YGR103W	YNL189W	969
YGR103W	YNR053C	998
YGR103W	YOL077C	999
YGR103W	YOL127W	999
YGR103W	YOR063W	999
YGR103W	YOR206W	998
YGR103W	YOR272W	999
YGR103W	YPL043W	999
YGR103W	YPL093W	999
YGR103W	YPL131W	999
YGR103W	YPL211W	999
YGR103W	YPL249C-A	925
YGR103W	YPR016C	999
YGR104C	YAL021C	926
YGR104C	YBL093C	999
YGR104C	YBR154C	983
YGR104C	YBR193C	999
YGR104C	YBR253W	999
YGR104C	YBR279W	999
YGR104C	YCR042C	908
YGR104C	YCR081W	998
YGR104C	YDL005C	999
YGR104C	YDL108W	928
YGR104C	YDL140C	999
YGR104C	YDR138W	926
YGR104C	YDR308C	999
YGR104C	YDR443C	999
YGR104C	YDR448W	915
YGR104C	YER011W	938
YGR104C	YER022W	999
YGR104C	YER148W	998
YGR104C	YGL025C	999
YGR104C	YGL043W	981
YGR104C	YGL070C	988
YGR104C	YGL127C	999
YGR104C	YGL151W	999
YGR104C	YGL244W	996
YGR104C	YGR005C	999
YGR104C	YHR041C	999
YGR104C	YHR058C	999
YGR104C	YHR143W-A	983
YGR104C	YIL021W	996
YGR104C	YJL140W	983

YGR104C	YLR071C	999
YGR104C	YLR182W	906
YGR104C	YLR418C	998
YGR104C	YMR112C	999
YGR104C	YNL025C	987
YGR104C	YNL189W	923
YGR104C	YNL236W	999
YGR104C	YNR010W	999
YGR104C	YOL005C	983
YGR104C	YOL051W	999
YGR104C	YOL135C	999
YGR104C	YOL145C	970
YGR104C	YOR123C	964
YGR104C	YOR151C	986
YGR104C	YOR174W	999
YGR104C	YOR224C	983
YGR104C	YPL042C	999
YGR104C	YPL129W	977
YGR104C	YPR056W	984
YGR104C	YPR070W	999
YGR104C	YPR168W	999
YGR104C	YPR187W	983
YGR105W	YEL027W	976
YGR105W	YLR447C	981
YGR105W	YMR123W	940
YGR105W	YOR270C	984
YGR105W	YPL234C	976
YGR108W	YBR135W	995
YGR108W	YBR160W	999
YGR108W	YDL108W	900
YGR108W	YDL155W	913
YGR108W	YDR146C	960
YGR108W	YGL003C	963
YGR108W	YJL187C	954
YGR108W	YLR079W	915
YGR108W	YLR210W	916
YGR108W	YMR001C	988
YGR108W	YMR036C	948
YGR108W	YPL256C	983
YGR108W	YPR025C	900
YGR108W	YPR119W	999
YGR109C	YBR160W	999
YGR109C	YJL194W	948
YGR109C	YLR079W	959

YGR109C	YPL256C	978
YGR109C	YPR120C	999
YGR112W	Q0045	950
YGR112W	YHR051W	900
YGR112W	YIL157C	985
YGR112W	YJL168C	952
YGR112W	YLR203C	999
YGR112W	YML129C	988
YGR113W	YBL034C	958
YGR113W	YBR156C	987
YGR113W	YBR233W-A	963
YGR113W	YCL029C	979
YGR113W	YDL028C	999
YGR113W	YDR016C	999
YGR113W	YDR201W	999
YGR113W	YDR254W	983
YGR113W	YDR320C-A	957
YGR113W	YEL061C	995
YGR113W	YER016W	968
YGR113W	YER133W	962
YGR113W	YGL061C	999
YGR113W	YGL079W	905
YGR113W	YGR140W	997
YGR113W	YGR179C	983
YGR113W	YGR188C	996
YGR113W	YIL144W	994
YGR113W	YJL030W	979
YGR113W	YJR089W	968
YGR113W	YJR135C	983
YGR113W	YKL049C	967
YGR113W	YKL052C	999
YGR113W	YKL138C-A	999
YGR113W	YKR037C	999
YGR113W	YKR083C	998
YGR113W	YLR045C	996
YGR113W	YLR423C	939
YGR113W	YMR094W	983
YGR113W	YMR117C	983
YGR113W	YMR168C	983
YGR113W	YOR195W	984
YGR113W	YPL018W	999
YGR113W	YPL209C	996
YGR113W	YPR046W	983
YGR116W	YAL021C	998

YGR116W	YBL008W	932
YGR116W	YBR019C	979
YGR116W	YBR289W	952
YGR116W	YDL140C	997
YGR116W	YGL019W	933
YGR116W	YGL207W	918
YGR116W	YGR063C	999
YGR116W	YHL025W	952
YGR116W	YIL035C	934
YGR116W	YML010W	999
YGR116W	YML069W	964
YGR116W	YOR061W	934
YGR116W	YOR290C	990
YGR116W	YPR133C	999
YGR118W	YAL035W	928
YGR118W	YBL027W	922
YGR118W	YBL072C	928
YGR118W	YBL087C	930
YGR118W	YBL092W	998
YGR118W	YBR031W	960
YGR118W	YBR048W	997
YGR118W	YBR079C	916
YGR118W	YBR084C-A	974
YGR118W	YBR118W	932
YGR118W	YBR181C	999
YGR118W	YBR189W	997
YGR118W	YBR191W	999
YGR118W	YCR031C	997
YGR118W	YDL061C	999
YGR118W	YDL075W	999
YGR118W	YDL081C	939
YGR118W	YDL082W	941
YGR118W	YDL083C	998
YGR118W	YDL130W	941
YGR118W	YDL136W	999
YGR118W	YDL191W	922
YGR118W	YDL192W	901
YGR118W	YDR012W	965
YGR118W	YDR025W	922
YGR118W	YDR064W	999
YGR118W	YDR382W	997
YGR118W	YDR385W	995
YGR118W	YDR418W	996
YGR118W	YDR447C	998

YGR118W	YDR450W	992
YGR118W	YDR471W	996
YGR118W	YDR500C	972
YGR118W	YEL054C	927
YGR118W	YER025W	972
YGR118W	YER056C-A	999
YGR118W	YER074W	998
YGR118W	YER102W	984
YGR118W	YER117W	983
YGR118W	YER131W	970
YGR118W	YER165W	921
YGR118W	YFL034C-A	913
YGR118W	YFR031C-A	916
YGR118W	YFR032C-A	998
YGR118W	YGL030W	999
YGR118W	YGL031C	999
YGR118W	YGL076C	960
YGR118W	YGL103W	999
YGR118W	YGL123W	999
YGR118W	YGL135W	921
YGR118W	YGL147C	933
YGR118W	YGL189C	921
YGR118W	YGR027C	998
YGR118W	YGR034W	924
YGR118W	YGR085C	994
YGR118W	YGR148C	999
YGR118W	YGR214W	999
YGR118W	YHL001W	999
YGR118W	YHL015W	999
YGR118W	YHL033C	999
YGR118W	YHR010W	993
YGR118W	YHR021C	932
YGR118W	YHR141C	938
YGR118W	YHR203C	978
YGR118W	YIL018W	916
YGR118W	YIL052C	999
YGR118W	YIL069C	922
YGR118W	YIL133C	933
YGR118W	YIL148W	969
YGR118W	YJL136C	974
YGR118W	YJL177W	999
YGR118W	YJL189W	980
YGR118W	YJL190C	991
YGR118W	YJL191W	973

YGR118W	YJR094W-A	995
YGR118W	YJR123W	999
YGR118W	YJR145C	925
YGR118W	YKL006W	999
YGR118W	YKL156W	992
YGR118W	YKL180W	999
YGR118W	YKR057W	933
YGR118W	YKR059W	964
YGR118W	YKR094C	907
YGR118W	YLL045C	938
YGR118W	YLR029C	981
YGR118W	YLR048W	999
YGR118W	YLR061W	999
YGR118W	YLR075W	998
YGR118W	YLR167W	972
YGR118W	YLR185W	970
YGR118W	YLR264W	995
YGR118W	YLR287C-A	997
YGR118W	YLR325C	929
YGR118W	YLR333C	944
YGR118W	YLR340W	985
YGR118W	YLR344W	994
YGR118W	YLR367W	986
YGR118W	YLR388W	999
YGR118W	YLR406C	921
YGR118W	YLR441C	998
YGR118W	YLR448W	998
YGR118W	YML024W	999
YGR118W	YML026C	927
YGR118W	YML063W	999
YGR118W	YML073C	998
YGR118W	YMR121C	920
YGR118W	YMR142C	951
YGR118W	YMR194W	999
YGR118W	YMR230W	964
YGR118W	YMR242C	960
YGR118W	YMR260C	923
YGR118W	YNL067W	998
YGR118W	YNL069C	931
YGR118W	YNL096C	923
YGR118W	YNL162W	929
YGR118W	YNL178W	999
YGR118W	YNL301C	975
YGR118W	YNL302C	932

YGR118W	YOL039W	930
YGR118W	YOL040C	999
YGR118W	YOL120C	924
YGR118W	YOL121C	933
YGR118W	YOL127W	999
YGR118W	YOR063W	997
YGR118W	YOR096W	997
YGR118W	YOR133W	964
YGR118W	YOR167C	968
YGR118W	YOR182C	900
YGR118W	YOR234C	995
YGR118W	YOR293W	984
YGR118W	YOR312C	945
YGR118W	YOR361C	914
YGR118W	YOR369C	940
YGR118W	YPL037C	980
YGR118W	YPL079W	980
YGR118W	YPL081W	995
YGR118W	YPL090C	904
YGR118W	YPL131W	982
YGR118W	YPL143W	997
YGR118W	YPL198W	932
YGR118W	YPL220W	977
YGR118W	YPL237W	943
YGR118W	YPL249C-A	954
YGR118W	YPR043W	960
YGR118W	YPR080W	932
YGR118W	YPR102C	999
YGR118W	YPR132W	985
YGR119C	YAR002W	987
YGR119C	YBL079W	993
YGR119C	YBR017C	965
YGR119C	YDL088C	905
YGR119C	YDL116W	995
YGR119C	YDL207W	962
YGR119C	YDR159W	966
YGR119C	YDR192C	988
YGR119C	YDR465C	923
YGR119C	YER105C	944
YGR119C	YER107C	989
YGR119C	YER110C	998
YGR119C	YFR002W	999
YGR119C	YGL092W	994
YGR119C	YGL172W	999

YGR119C	YGR120C	948
YGR119C	YGR218W	943
YGR119C	YIL115C	931
YGR119C	YJL041W	999
YGR119C	YJL061W	991
YGR119C	YJR042W	976
YGR119C	YKL057C	983
YGR119C	YKL068W	999
YGR119C	YKR082W	961
YGR119C	YLR018C	980
YGR119C	YLR335W	999
YGR119C	YLR347C	999
YGR119C	YML103C	949
YGR119C	YMR047C	995
YGR119C	YMR129W	970
YGR119C	YMR153W	916
YGR119C	YMR308C	981
YGR119C	YOL072W	924
YGR119C	YOR098C	981
YGR119C	YOR257W	961
YGR119C	YPL125W	965
YGR119C	YPL169C	994
YGR120C	YDL058W	942
YGR120C	YDR189W	987
YGR120C	YDR311W	905
YGR120C	YER157W	999
YGR120C	YFL038C	988
YGR120C	YGL005C	999
YGR120C	YGL223C	999
YGR120C	YGR089W	923
YGR120C	YGR119C	948
YGR120C	YIL004C	985
YGR120C	YKL196C	928
YGR120C	YLR026C	964
YGR120C	YLR262C	964
YGR120C	YLR268W	923
YGR120C	YLR423C	970
YGR120C	YML071C	999
YGR120C	YNL041C	999
YGR120C	YNL051W	999
YGR120C	YOR216C	983
YGR120C	YPR105C	999
YGR121C	YER088C	916
YGR121C	YJR147W	922

YGR121C	YNL142W	999
YGR121C	YOR032C	908
YGR121C	YPR138C	998
YGR122C-A	YBL107W-A	966
YGR122C-A	YCR018C-A	974
YGR122W	YHL027W	906
YGR122W	YLR025W	914
YGR123C	YKL117W	967
YGR123C	YMR186W	991
YGR123C	YNR032W	979
YGR123C	YPL240C	994
YGR124W	YAL062W	904
YGR124W	YCR024C	906
YGR124W	YGL245W	919
YGR124W	YHR019C	979
YGR124W	YLL018C	922
YGR124W	YMR250W	906
YGR124W	YOL058W	939
YGR124W	YOR375C	903
YGR124W	YPR035W	919
YGR124W	YPR145W	999
YGR128C	YBL004W	997
YGR128C	YBR247C	967
YGR128C	YCL059C	923
YGR128C	YCR057C	999
YGR128C	YDL014W	995
YGR128C	YDL148C	999
YGR128C	YDL153C	915
YGR128C	YDR299W	931
YGR128C	YDR324C	999
YGR128C	YDR335W	905
YGR128C	YDR398W	999
YGR128C	YDR449C	999
YGR128C	YEL055C	999
YGR128C	YER082C	999
YGR128C	YGL120C	922
YGR128C	YGL171W	935
YGR128C	YGR090W	999
YGR128C	YGR145W	912
YGR128C	YHR148W	997
YGR128C	YHR169W	925
YGR128C	YHR196W	999
YGR128C	YJL010C	983
YGR128C	YJL069C	985

YGR128C	YJL109C	999
YGR128C	YJR002W	999
YGR128C	YKL099C	912
YGR128C	YKL205W	944
YGR128C	YKR060W	917
YGR128C	YLL011W	998
YGR128C	YLR003C	922
YGR128C	YLR129W	998
YGR128C	YLR175W	929
YGR128C	YLR186W	997
YGR128C	YLR197W	998
YGR128C	YLR222C	999
YGR128C	YLR293C	900
YGR128C	YLR409C	991
YGR128C	YML093W	920
YGR128C	YMR093W	999
YGR128C	YMR128W	997
YGR128C	YMR229C	996
YGR128C	YMR290C	990
YGR128C	YNL075W	950
YGR128C	YNL132W	999
YGR128C	YOL010W	914
YGR128C	YOR004W	930
YGR128C	YOR078W	990
YGR128C	YOR145C	937
YGR128C	YOR310C	987
YGR128C	YPL012W	998
YGR128C	YPL126W	999
YGR128C	YPL217C	998
YGR128C	YPL266W	974
YGR128C	YPR112C	924
YGR128C	YPR137W	912
YGR128C	YPR144C	999
YGR129W	YBR188C	979
YGR129W	YDR364C	990
YGR129W	YDR416W	999
YGR129W	YJR050W	997
YGR129W	YLL036C	998
YGR129W	YLR117C	999
YGR129W	YMR213W	998
YGR129W	YPR101W	990
YGR130C	YMR031C	995
YGR132C	YAL010C	909
YGR132C	YDR245W	982

YGR132C	YEL036C	995
YGR132C	YER017C	973
YGR132C	YGR231C	999
YGR132C	YJL183W	993
YGR132C	YLL006W	908
YGR132C	YMR089C	974
YGR132C	YNL098C	928
YGR132C	YOL009C	918
YGR132C	YPL050C	993
YGR133W	YAL055W	996
YGR133W	YDR244W	991
YGR133W	YDR265W	970
YGR133W	YKL197C	931
YGR133W	YNL329C	930
YGR134W	YAL021C	999
YGR134W	YCR093W	999
YGR134W	YDR252W	978
YGR134W	YER068W	908
YGR134W	YJR011C	997
YGR134W	YNL288W	999
YGR134W	YNR052C	999
YGR134W	YPR072W	934
YGR135W	YBL041W	999
YGR135W	YBR173C	927
YGR135W	YBR175W	943
YGR135W	YDL020C	950
YGR135W	YDL097C	944
YGR135W	YDL147W	939
YGR135W	YDL188C	923
YGR135W	YDR427W	901
YGR135W	YER012W	999
YGR135W	YER021W	934
YGR135W	YER094C	999
YGR135W	YFL007W	990
YGR135W	YFR004W	935
YGR135W	YFR050C	999
YGR135W	YFR052W	909
YGR135W	YGL011C	999
YGR135W	YGL048C	916
YGR135W	YGR253C	999
YGR135W	YHR027C	911
YGR135W	YHR200W	999
YGR135W	YIL075C	901
YGR135W	YJL001W	999

YGR135W	YLL039C	961
YGR135W	YML092C	999
YGR135W	YMR314W	999
YGR135W	YNL244C	999
YGR135W	YNL288W	927
YGR135W	YNL298W	963
YGR135W	YOL038W	999
YGR135W	YOR157C	999
YGR135W	YOR261C	990
YGR135W	YOR362C	999
YGR135W	YPL144W	967
YGR135W	YPR103W	999
YGR135W	YPR108W	913
YGR136W	YER125W	921
YGR136W	YGR058W	931
YGR136W	YOR181W	981
YGR140W	YBR156C	920
YGR140W	YDL013W	929
YGR140W	YDL064W	929
YGR140W	YDL139C	978
YGR140W	YDR254W	999
YGR140W	YDR318W	987
YGR140W	YDR328C	999
YGR140W	YER032W	929
YGR140W	YER047C	929
YGR140W	YGL061C	932
YGR140W	YGR063C	959
YGR140W	YGR113W	997
YGR140W	YGR179C	986
YGR140W	YGR188C	928
YGR140W	YIL144W	991
YGR140W	YJL030W	911
YGR140W	YJR060W	999
YGR140W	YJR089W	999
YGR140W	YJR135C	981
YGR140W	YKL049C	993
YGR140W	YKL052C	978
YGR140W	YKL089W	998
YGR140W	YLR045C	987
YGR140W	YLR381W	981
YGR140W	YMR094W	999
YGR140W	YMR168C	999
YGR140W	YNL078W	929
YGR140W	YNL307C	990

YGR140W	YOL069W	978
YGR140W	YOR195W	954
YGR140W	YPL018W	999
YGR140W	YPL209C	999
YGR140W	YPL233W	906
YGR140W	YPR046W	981
YGR142W	YAL030W	944
YGR142W	YBR086C	982
YGR142W	YCR027C	979
YGR142W	YJL036W	952
YGR142W	YJL059W	998
YGR142W	YNL263C	997
YGR143W	YBR254C	933
YGR143W	YPR159W	996
YGR145W	YBL004W	997
YGR145W	YBL072C	927
YGR145W	YBR247C	999
YGR145W	YCL059C	999
YGR145W	YCR057C	986
YGR145W	YDL014W	995
YGR145W	YDL060W	921
YGR145W	YDL148C	998
YGR145W	YDL153C	923
YGR145W	YDR025W	978
YGR145W	YDR064W	929
YGR145W	YDR299W	999
YGR145W	YDR324C	953
YGR145W	YDR365C	992
YGR145W	YDR449C	999
YGR145W	YER082C	997
YGR145W	YER127W	977
YGR145W	YGL171W	997
YGR145W	YGR090W	997
YGR145W	YGR128C	912
YGR145W	YHR148W	997
YGR145W	YHR169W	915
YGR145W	YHR196W	916
YGR145W	YIL035C	928
YGR145W	YJL010C	989
YGR145W	YJL033W	981
YGR145W	YJR002W	924
YGR145W	YKR060W	997
YGR145W	YLL011W	997
YGR145W	YLR003C	912

YGR145W	YLR129W	997
YGR145W	YLR186W	997
YGR145W	YLR222C	915
YGR145W	YLR409C	997
YGR145W	YML093W	911
YGR145W	YMR093W	997
YGR145W	YMR194W	925
YGR145W	YNL075W	909
YGR145W	YNL132W	999
YGR145W	YNR054C	997
YGR145W	YOR004W	910
YGR145W	YOR096W	929
YGR145W	YPL126W	997
YGR145W	YPL217C	998
YGR145W	YPR112C	950
YGR145W	YPR137W	923
YGR145W	YPR144C	999
YGR148C	YAL035W	915
YGR148C	YBL027W	912
YGR148C	YBL072C	904
YGR148C	YBL087C	920
YGR148C	YBL092W	947
YGR148C	YBR031W	960
YGR148C	YBR048W	919
YGR148C	YBR084C-A	942
YGR148C	YBR181C	917
YGR148C	YBR189W	996
YGR148C	YBR191W	999
YGR148C	YCR031C	971
YGR148C	YDL061C	989
YGR148C	YDL075W	993
YGR148C	YDL082W	938
YGR148C	YDL130W	919
YGR148C	YDL136W	941
YGR148C	YDL191W	916
YGR148C	YDR012W	956
YGR148C	YDR025W	909
YGR148C	YDR064W	997
YGR148C	YDR382W	994
YGR148C	YDR418W	995
YGR148C	YDR447C	910
YGR148C	YDR471W	991
YGR148C	YDR500C	932
YGR148C	YEL054C	909

YGR148C	YER056C-A	995
YGR148C	YER074W	906
YGR148C	YER102W	950
YGR148C	YER117W	944
YGR148C	YER131W	975
YGR148C	YFR031C-A	923
YGR148C	YFR032C-A	905
YGR148C	YGL030W	997
YGR148C	YGL031C	999
YGR148C	YGL076C	969
YGR148C	YGL103W	999
YGR148C	YGL123W	995
YGR148C	YGL147C	949
YGR148C	YGL189C	907
YGR148C	YGR027C	999
YGR148C	YGR034W	923
YGR148C	YGR085C	999
YGR148C	YGR118W	999
YGR148C	YGR214W	999
YGR148C	YHL001W	998
YGR148C	YHL015W	982
YGR148C	YHL033C	999
YGR148C	YHR010W	982
YGR148C	YHR021C	907
YGR148C	YHR141C	926
YGR148C	YHR203C	934
YGR148C	YIL018W	908
YGR148C	YIL052C	999
YGR148C	YIL069C	906
YGR148C	YIL133C	920
YGR148C	YIL148W	934
YGR148C	YJL136C	979
YGR148C	YJL177W	979
YGR148C	YJL189W	935
YGR148C	YJL190C	925
YGR148C	YJL191W	964
YGR148C	YJR094W-A	968
YGR148C	YJR123W	999
YGR148C	YKL006W	999
YGR148C	YKL156W	970
YGR148C	YKL157W	980
YGR148C	YKL180W	999
YGR148C	YKR057W	904
YGR148C	YLL045C	956

YGR148C	YLR029C	985
YGR148C	YLR048W	981
YGR148C	YLR061W	993
YGR148C	YLR075W	990
YGR148C	YLR167W	939
YGR148C	YLR185W	927
YGR148C	YLR264W	972
YGR148C	YLR325C	932
YGR148C	YLR333C	952
YGR148C	YLR340W	975
YGR148C	YLR344W	974
YGR148C	YLR367W	966
YGR148C	YLR388W	997
YGR148C	YLR441C	925
YGR148C	YLR448W	996
YGR148C	YML024W	997
YGR148C	YML063W	998
YGR148C	YML073C	906
YGR148C	YMR121C	919
YGR148C	YMR142C	952
YGR148C	YMR194W	994
YGR148C	YMR230W	948
YGR148C	YMR242C	931
YGR148C	YMR260C	903
YGR148C	YNL067W	983
YGR148C	YNL069C	902
YGR148C	YNL096C	928
YGR148C	YNL162W	904
YGR148C	YNL178W	999
YGR148C	YNL301C	942
YGR148C	YNL302C	907
YGR148C	YOL039W	929
YGR148C	YOL040C	922
YGR148C	YOL120C	917
YGR148C	YOL121C	918
YGR148C	YOL127W	997
YGR148C	YOR063W	983
YGR148C	YOR096W	996
YGR148C	YOR133W	909
YGR148C	YOR167C	961
YGR148C	YOR234C	977
YGR148C	YOR293W	980
YGR148C	YOR312C	921
YGR148C	YOR369C	940

YGR148C	YPL037C	939
YGR148C	YPL079W	994
YGR148C	YPL081W	951
YGR148C	YPL131W	979
YGR148C	YPL143W	974
YGR148C	YPL198W	908
YGR148C	YPL220W	939
YGR148C	YPL249C-A	921
YGR148C	YPR043W	922
YGR148C	YPR102C	999
YGR148C	YPR132W	947
YGR152C	YAL041W	999
YGR152C	YAR019C	980
YGR152C	YBR200W	996
YGR152C	YCR038C	969
YGR152C	YKL092C	995
YGR152C	YLR229C	979
YGR152C	YLR310C	940
YGR152C	YLR353W	916
YGR152C	YNL098C	995
YGR152C	YOR127W	970
YGR152C	YPL161C	900
YGR155W	YAL012W	996
YGR155W	YBR263W	934
YGR155W	YCL064C	908
YGR155W	YDR023W	947
YGR155W	YER043C	919
YGR155W	YER086W	908
YGR155W	YGL184C	974
YGR155W	YJR130C	955
YGR155W	YLR058C	920
YGR155W	YLR303W	969
YGR156W	YAL043C	999
YGR156W	YDR195W	999
YGR156W	YDR301W	999
YGR156W	YER133W	999
YGR156W	YHL033C	976
YGR156W	YJL033W	959
YGR156W	YJR093C	999
YGR156W	YKL018W	995
YGR156W	YKL059C	999
YGR156W	YKR002W	999
YGR156W	YLR115W	999
YGR156W	YLR277C	995

YGR156W	YLR448W	978
YGR156W	YMR061W	995
YGR156W	YNL222W	995
YGR156W	YNL317W	999
YGR156W	YOR179C	999
YGR156W	YPR107C	999
YGR157W	YJR073C	998
YGR157W	YLR450W	919
YGR158C	YBL026W	973
YGR158C	YCR035C	999
YGR158C	YDL111C	999
YGR158C	YDR280W	999
YGR158C	YGR095C	999
YGR158C	YGR195W	999
YGR158C	YHR069C	999
YGR158C	YHR081W	986
YGR158C	YJR022W	949
YGR158C	YLR345W	974
YGR158C	YNL189W	970
YGR158C	YNL232W	999
YGR158C	YNR024W	959
YGR158C	YOL021C	999
YGR158C	YOL142W	999
YGR158C	YOR001W	999
YGR158C	YOR076C	999
YGR158C	YPR189W	978
YGR159C	YAR073W	998
YGR159C	YDL014W	984
YGR159C	YDR452W	998
YGR159C	YER165W	923
YGR159C	YGL078C	944
YGR159C	YGL120C	990
YGR159C	YHR132C	998
YGR159C	YLR175W	940
YGR159C	YLR197W	950
YGR159C	YLR410W	931
YGR159C	YOR204W	923
YGR159C	YOR310C	948
YGR159C	YPL237W	931
YGR161C	YAL016W	996
YGR161C	YDL047W	999
YGR161C	YDL134C	988
YGR161C	YDL188C	909
YGR161C	YJL098W	995

YGR162W	YAL036C	923
YGR162W	YBL032W	981
YGR162W	YBR079C	939
YGR162W	YBR189W	919
YGR162W	YCL011C	976
YGR162W	YCL037C	992
YGR162W	YCR031C	914
YGR162W	YDL014W	936
YGR162W	YDL051W	994
YGR162W	YDL126C	940
YGR162W	YDR064W	926
YGR162W	YDR381W	948
YGR162W	YER025W	931
YGR162W	YER110C	940
YGR162W	YER165W	999
YGR162W	YGL049C	999
YGR162W	YGL123W	915
YGR162W	YGL173C	987
YGR162W	YGL245W	946
YGR162W	YGR214W	929
YGR162W	YHL034C	993
YGR162W	YIL133C	944
YGR162W	YIR001C	999
YGR162W	YJL138C	999
YGR162W	YJL190C	997
YGR162W	YJL191W	909
YGR162W	YJR007W	978
YGR162W	YJR123W	911
YGR162W	YJR138W	940
YGR162W	YJR145C	916
YGR162W	YKL156W	911
YGR162W	YKR059W	999
YGR162W	YLR048W	932
YGR162W	YLR249W	958
YGR162W	YLR264W	912
YGR162W	YLR388W	910
YGR162W	YLR441C	913
YGR162W	YML063W	911
YGR162W	YMR104C	979
YGR162W	YMR125W	999
YGR162W	YMR230W	916
YGR162W	YMR260C	980
YGR162W	YMR309C	919
YGR162W	YNL096C	914

YGR162W	YNL178W	910
YGR162W	YOL006C	940
YGR162W	YOL139C	999
YGR162W	YOR096W	912
YGR162W	YOR204W	998
YGR162W	YOR243C	902
YGR162W	YOR276W	999
YGR162W	YOR293W	910
YGR162W	YOR361C	942
YGR162W	YPR041W	977
YGR162W	YPR163C	987
YGR163W	YBR077C	998
YGR163W	YCR015C	941
YGR163W	YDR190C	909
YGR163W	YGL023C	996
YGR163W	YGR098C	941
YGR163W	YGR203W	941
YGR163W	YIL063C	913
YGR163W	YJR066W	977
YGR163W	YKL143W	952
YGR163W	YKR007W	999
YGR163W	YML121W	999
YGR163W	YPL235W	972
YGR165W	Q0140	962
YGR165W	YBL090W	988
YGR165W	YBR146W	988
YGR165W	YBR251W	988
YGR165W	YDR041W	988
YGR165W	YDR175C	988
YGR165W	YDR337W	988
YGR165W	YDR347W	988
YGR165W	YGL129C	988
YGR165W	YGR084C	974
YGR165W	YHL004W	997
YGR165W	YIL070C	986
YGR165W	YIL093C	988
YGR165W	YJR101W	988
YGR165W	YJR113C	988
YGR165W	YKL155C	988
YGR165W	YMR158W	997
YGR165W	YMR188C	995
YGR165W	YNL137C	997
YGR165W	YNL306W	988
YGR165W	YOR158W	988

YGR165W	YPL013C	998
YGR165W	YPL118W	988
YGR166W	YBR254C	999
YGR166W	YDR108W	992
YGR166W	YDR246W	999
YGR166W	YDR407C	999
YGR166W	YDR472W	999
YGR166W	YER031C	926
YGR166W	YGR229C	923
YGR166W	YJL174W	970
YGR166W	YKR068C	999
YGR166W	YML077W	999
YGR166W	YMR218C	999
YGR166W	YOR115C	999
YGR166W	YOR197W	998
YGR166W	YPR159W	990
YGR167W	YDR320C	976
YGR167W	YGL206C	999
YGR167W	YJR125C	996
YGR167W	YNL243W	999
YGR167W	YOR181W	903
YGR170W	YGR199W	923
YGR170W	YNL169C	995
YGR170W	YNR051C	923
YGR172C	YER031C	999
YGR172C	YER074W-A	931
YGR172C	YER136W	956
YGR172C	YFL005W	970
YGR172C	YFL038C	999
YGR172C	YGL161C	982
YGR172C	YGL198W	958
YGR172C	YGL210W	956
YGR172C	YKR014C	954
YGR172C	YLR208W	974
YGR172C	YLR262C	993
YGR172C	YNL044W	997
YGR172C	YNL263C	999
YGR172C	YNR026C	969
YGR172C	YOR089C	990
YGR172C	YPR028W	985
YGR172C	YPR181C	969
YGR173W	YDR152W	999
YGR174C	YPL215W	994
YGR175C	YGL001C	999

YGR175C	YGL012W	999
YGR175C	YGR060W	999
YGR175C	YHR007C	937
YGR175C	YHR020W	961
YGR175C	YHR072W	962
YGR175C	YHR190W	964
YGR175C	YMR015C	999
YGR175C	YMR202W	998
YGR175C	YNL280C	999
YGR178C	YDL053C	997
YGR178C	YER032W	928
YGR178C	YER165W	988
YGR178C	YGR048W	903
YGR178C	YHR121W	999
YGR178C	YLR216C	913
YGR178C	YNL085W	982
YGR178C	YOR297C	924
YGR178C	YPL049C	913
YGR179C	YAL034W-A	963
YGR179C	YBR107C	999
YGR179C	YBR211C	999
YGR179C	YCR032W	935
YGR179C	YDR254W	999
YGR179C	YDR318W	999
YGR179C	YDR383C	999
YGR179C	YGL060W	991
YGR179C	YGR113W	983
YGR179C	YGR140W	986
YGR179C	YHR166C	907
YGR179C	YIL144W	937
YGR179C	YIR010W	975
YGR179C	YJL030W	926
YGR179C	YJR135C	999
YGR179C	YKL049C	995
YGR179C	YLR315W	999
YGR179C	YLR381W	999
YGR179C	YMR168C	987
YGR179C	YOL069W	944
YGR179C	YOR026W	919
YGR179C	YOR195W	941
YGR179C	YPL018W	999
YGR179C	YPL209C	973
YGR179C	YPL233W	991
YGR179C	YPR046W	998

YGR180C	YBR136W	914
YGR180C	YDL101C	941
YGR180C	YER070W	999
YGR180C	YGR209C	900
YGR180C	YIL066C	999
YGR180C	YJL026W	999
YGR180C	YJL115W	975
YGR180C	YJR057W	911
YGR180C	YLR043C	900
YGR180C	YNL133C	921
YGR180C	YOR229W	995
YGR180C	YOR230W	999
YGR180C	YPL240C	919
YGR181W	YBR091C	969
YGR181W	YEL020W-A	997
YGR181W	YHR005C-A	986
YGR181W	YJR135W-A	999
YGR181W	YKL195W	996
YGR181W	YNR017W	999
YGR183C	Q0105	999
YGR183C	YBL045C	999
YGR183C	YDR529C	999
YGR183C	YEL024W	999
YGR183C	YFR033C	999
YGR183C	YHR001W-A	996
YGR183C	YJL166W	999
YGR183C	YJR048W	990
YGR183C	YOR065W	999
YGR183C	YPR191W	999
YGR184C	YCR066W	962
YGR184C	YGL058W	999
YGR184C	YPL177C	998
YGR184C	YPR103W	998
YGR185C	YGR229C	997
YGR185C	YNL230C	998
YGR185C	YPL097W	908
YGR186W	YBR154C	999
YGR186W	YBR279W	938
YGR186W	YCR042C	979
YGR186W	YDL115C	987
YGR186W	YDL140C	999
YGR186W	YDR404C	999
YGR186W	YER022W	999
YGR186W	YGL043W	998

YGR186W	YGL070C	999
YGR186W	YGL127C	919
YGR186W	YGL244W	927
YGR186W	YGR005C	999
YGR186W	YGR063C	990
YGR186W	YIL021W	999
YGR186W	YJL140W	998
YGR186W	YKL028W	984
YGR186W	YKR062W	984
YGR186W	YLR418C	946
YGR186W	YML010W	990
YGR186W	YMR277W	999
YGR186W	YOL005C	998
YGR186W	YOL051W	917
YGR186W	YOL145C	930
YGR186W	YOR123C	932
YGR186W	YOR151C	999
YGR186W	YOR210W	998
YGR186W	YOR224C	998
YGR186W	YPL129W	999
YGR186W	YPR056W	984
YGR186W	YPR086W	998
YGR186W	YPR187W	999
YGR187C	YBR155W	990
YGR187C	YDR385W	980
YGR187C	YJL111W	921
YGR187C	YOR133W	953
YGR187C	YOR281C	913
YGR188C	YBL034C	944
YGR188C	YBR107C	948
YGR188C	YCL029C	951
YGR188C	YDL028C	999
YGR188C	YDR016C	977
YGR188C	YDR254W	936
YGR188C	YDR318W	978
YGR188C	YDR328C	985
YGR188C	YEL061C	984
YGR188C	YER016W	991
YGR188C	YGL086W	999
YGR188C	YGL116W	997
YGR188C	YGR113W	996
YGR188C	YGR140W	928
YGR188C	YIL144W	933
YGR188C	YIR010W	963

YGR188C	YJL013C	999
YGR188C	YJL030W	999
YGR188C	YJR053W	917
YGR188C	YJR135C	971
YGR188C	YLR381W	919
YGR188C	YML085C	984
YGR188C	YMR055C	995
YGR188C	YMR078C	933
YGR188C	YMR094W	935
YGR188C	YMR168C	964
YGR188C	YOL069W	900
YGR188C	YOR026W	999
YGR188C	YOR073W	993
YGR188C	YOR195W	927
YGR188C	YPL008W	927
YGR188C	YPL018W	991
YGR188C	YPL209C	914
YGR188C	YPR046W	990
YGR189C	YEL040W	985
YGR192C	YCR012W	999
YGR192C	YDL042C	969
YGR192C	YDR050C	993
YGR192C	YJR009C	969
YGR192C	YKL060C	999
YGR192C	YKL152C	999
YGR193C	YBR221C	999
YGR193C	YDR051C	998
YGR193C	YDR430C	987
YGR193C	YER178W	990
YGR193C	YFL018C	999
YGR193C	YNL071W	987
YGR194C	YDR300C	927
YGR194C	YLR070C	973
YGR194C	YNL265C	920
YGR195W	YCR035C	999
YGR195W	YDL111C	999
YGR195W	YDR060W	914
YGR195W	YDR280W	999
YGR195W	YGR095C	999
YGR195W	YGR158C	999
YGR195W	YHR069C	999
YGR195W	YHR081W	992
YGR195W	YLR398C	969
YGR195W	YNL189W	995

YGR195W	YNL232W	999
YGR195W	YNR024W	959
YGR195W	YOL021C	999
YGR195W	YOL142W	999
YGR195W	YOR001W	999
YGR195W	YOR076C	999
YGR195W	YOR326W	965
YGR196C	YBR260C	998
YGR198W	YBR131W	918
YGR198W	YGL124C	918
YGR199W	YDL226C	923
YGR199W	YDR349C	923
YGR199W	YGR170W	923
YGR200C	YBL071W-A	952
YGR200C	YDL074C	942
YGR200C	YDL140C	958
YGR200C	YGR252W	947
YGR200C	YHR187W	999
YGR200C	YKL110C	999
YGR200C	YLR384C	999
YGR200C	YMR312W	999
YGR200C	YNL272C	972
YGR200C	YPL086C	999
YGR200C	YPL101W	999
YGR202C	YDL143W	962
YGR202C	YLR133W	987
YGR202C	YLR347C	966
YGR202C	YMR079W	996
YGR202C	YNL130C	986
YGR202C	YNL189W	960
YGR203W	YGR163W	941
YGR204W	YBR084W	964
YGR204W	YBR263W	999
YGR204W	YDR019C	957
YGR204W	YDR408C	974
YGR204W	YER183C	981
YGR204W	YGL125W	936
YGR204W	YKR080W	936
YGR204W	YKR092C	955
YGR204W	YLR028C	959
YGR204W	YLR058C	999
YGR204W	YMR113W	915
YGR204W	YMR120C	956
YGR204W	YOR074C	938

YGR204W	YOR236W	909
YGR206W	YCL008C	999
YGR206W	YLR119W	999
YGR206W	YPL065W	999
YGR207C	YOR356W	997
YGR207C	YPR004C	999
YGR208W	YBR263W	966
YGR208W	YDR023W	922
YGR208W	YER026C	904
YGR208W	YHR011W	912
YGR208W	YJL076W	935
YGR208W	YLR058C	966
YGR208W	YOR184W	989
YGR209C	YCL035C	934
YGR209C	YDR353W	999
YGR209C	YDR513W	914
YGR209C	YER042W	917
YGR209C	YER070W	989
YGR209C	YGR180C	900
YGR209C	YHR106W	988
YGR209C	YHR206W	998
YGR209C	YIL066C	989
YGR209C	YJL026W	991
YGR209C	YKL067W	910
YGR209C	YLR043C	999
YGR209C	YML007W	984
YGR209C	YPL091W	999
YGR209C	YPR167C	989
YGR211W	YLR216C	913
YGR213C	YHR134W	904
YGR214W	YAL035W	923
YGR214W	YBL027W	920
YGR214W	YBL072C	933
YGR214W	YBL087C	944
YGR214W	YBL092W	968
YGR214W	YBR031W	983
YGR214W	YBR048W	993
YGR214W	YBR079C	994
YGR214W	YBR084C-A	945
YGR214W	YBR118W	911
YGR214W	YBR181C	919
YGR214W	YBR189W	999
YGR214W	YBR191W	999
YGR214W	YCL059C	943

YGR214W	YCR031C	999
YGR214W	YDL061C	997
YGR214W	YDL075W	976
YGR214W	YDL081C	932
YGR214W	YDL082W	959
YGR214W	YDL083C	962
YGR214W	YDL130W	924
YGR214W	YDL136W	941
YGR214W	YDL191W	914
YGR214W	YDR012W	994
YGR214W	YDR025W	927
YGR214W	YDR064W	999
YGR214W	YDR382W	994
YGR214W	YDR385W	991
YGR214W	YDR418W	999
YGR214W	YDR429C	900
YGR214W	YDR447C	998
YGR214W	YDR450W	994
YGR214W	YDR471W	997
YGR214W	YDR500C	939
YGR214W	YEL054C	910
YGR214W	YER025W	974
YGR214W	YER056C-A	987
YGR214W	YER074W	909
YGR214W	YER102W	964
YGR214W	YER117W	950
YGR214W	YER131W	976
YGR214W	YER165W	955
YGR214W	YFL034C-A	904
YGR214W	YFR031C-A	929
YGR214W	YFR032C-A	933
YGR214W	YGL030W	998
YGR214W	YGL031C	999
YGR214W	YGL076C	981
YGR214W	YGL103W	999
YGR214W	YGL123W	999
YGR214W	YGL135W	909
YGR214W	YGL147C	956
YGR214W	YGL189C	905
YGR214W	YGR027C	998
YGR214W	YGR034W	921
YGR214W	YGR085C	978
YGR214W	YGR118W	999
YGR214W	YGR148C	999

YGR214W	YGR162W	929
YGR214W	YHL001W	998
YGR214W	YHL015W	999
YGR214W	YHL033C	999
YGR214W	YHR010W	979
YGR214W	YHR021C	926
YGR214W	YHR141C	927
YGR214W	YHR203C	956
YGR214W	YIL018W	913
YGR214W	YIL052C	997
YGR214W	YIL069C	909
YGR214W	YIL133C	961
YGR214W	YIL148W	941
YGR214W	YJL136C	975
YGR214W	YJL177W	999
YGR214W	YJL189W	939
YGR214W	YJL190C	992
YGR214W	YJL191W	995
YGR214W	YJR094W-A	972
YGR214W	YJR123W	999
YGR214W	YJR145C	913
YGR214W	YKL006W	999
YGR214W	YKL156W	977
YGR214W	YKL180W	999
YGR214W	YKR057W	952
YGR214W	YKR059W	938
YGR214W	YKR094C	905
YGR214W	YLL045C	967
YGR214W	YLR029C	998
YGR214W	YLR048W	999
YGR214W	YLR061W	995
YGR214W	YLR075W	978
YGR214W	YLR167W	929
YGR214W	YLR185W	937
YGR214W	YLR192C	915
YGR214W	YLR264W	982
YGR214W	YLR325C	918
YGR214W	YLR333C	948
YGR214W	YLR340W	984
YGR214W	YLR344W	971
YGR214W	YLR367W	978
YGR214W	YLR388W	991
YGR214W	YLR406C	911
YGR214W	YLR441C	943

YGR214W	YLR448W	999
YGR214W	YML024W	992
YGR214W	YML026C	945
YGR214W	YML063W	999
YGR214W	YML073C	924
YGR214W	YMR121C	918
YGR214W	YMR142C	962
YGR214W	YMR194W	993
YGR214W	YMR230W	961
YGR214W	YMR242C	939
YGR214W	YMR260C	909
YGR214W	YMR309C	952
YGR214W	YNL067W	997
YGR214W	YNL069C	956
YGR214W	YNL096C	925
YGR214W	YNL162W	917
YGR214W	YNL178W	999
YGR214W	YNL209W	909
YGR214W	YNL301C	962
YGR214W	YNL302C	916
YGR214W	YOL039W	952
YGR214W	YOL040C	994
YGR214W	YOL120C	927
YGR214W	YOL121C	927
YGR214W	YOL127W	999
YGR214W	YOR063W	997
YGR214W	YOR096W	994
YGR214W	YOR133W	932
YGR214W	YOR167C	954
YGR214W	YOR234C	995
YGR214W	YOR293W	991
YGR214W	YOR312C	939
YGR214W	YOR361C	955
YGR214W	YOR369C	937
YGR214W	YPL079W	980
YGR214W	YPL081W	998
YGR214W	YPL090C	906
YGR214W	YPL131W	984
YGR214W	YPL143W	994
YGR214W	YPL198W	925
YGR214W	YPL220W	945
YGR214W	YPL237W	937
YGR214W	YPL249C-A	945
YGR214W	YPR043W	937

YGR214W	YPR080W	912
YGR214W	YPR102C	997
YGR214W	YPR132W	949
YGR214W	YPR163C	920
YGR215W	Q0140	962
YGR215W	YBL090W	988
YGR215W	YBR146W	988
YGR215W	YBR251W	999
YGR215W	YDR041W	988
YGR215W	YDR175C	988
YGR215W	YDR337W	988
YGR215W	YDR347W	988
YGR215W	YGL129C	988
YGR215W	YGR084C	974
YGR215W	YHL004W	999
YGR215W	YIL093C	988
YGR215W	YJR101W	988
YGR215W	YJR113C	988
YGR215W	YKL003C	986
YGR215W	YKL155C	997
YGR215W	YMR158W	972
YGR215W	YMR188C	987
YGR215W	YNL137C	988
YGR215W	YNL306W	988
YGR215W	YNR037C	987
YGR215W	YOR158W	988
YGR215W	YPL118W	988
YGR216C	YDR437W	910
YGR216C	YNL038W	952
YGR216C	YPL076W	991
YGR216C	YPL175W	991
YGR217W	YDR182W	983
YGR217W	YLL021W	904
YGR217W	YNL291C	993
YGR218W	YAL047C	972
YGR218W	YBR069C	979
YGR218W	YDR002W	998
YGR218W	YDR192C	998
YGR218W	YEL022W	968
YGR218W	YER016W	902
YGR218W	YER040W	924
YGR218W	YER165W	974
YGR218W	YFL037W	958
YGR218W	YGL086W	911

YGR218W	YGL097W	990
YGR218W	YGL172W	933
YGR218W	YGR119C	943
YGR218W	YHR020W	999
YGR218W	YHR170W	999
YGR218W	YIL063C	999
YGR218W	YIL115C	999
YGR218W	YJR066W	924
YGR218W	YJR077C	981
YGR218W	YKL068W	913
YGR218W	YKL143W	999
YGR218W	YKL145W	973
YGR218W	YLR113W	953
YGR218W	YLR131C	974
YGR218W	YLR180W	969
YGR218W	YLR293C	999
YGR218W	YML007W	987
YGR218W	YMR047C	956
YGR218W	YMR235C	997
YGR218W	YNL189W	964
YGR218W	YOL123W	982
YGR218W	YOR046C	998
YGR218W	YOR048C	938
YGR218W	YPL120W	908
YGR220C	YBL038W	999
YGR220C	YBR122C	997
YGR220C	YCR046C	997
YGR220C	YCR071C	998
YGR220C	YDR116C	999
YGR220C	YDR237W	999
YGR220C	YDR296W	997
YGR220C	YDR322W	997
YGR220C	YDR405W	966
YGR220C	YDR462W	997
YGR220C	YEL050C	981
YGR220C	YGL123W	905
YGR220C	YGR076C	950
YGR220C	YHR147C	990
YGR220C	YJL063C	999
YGR220C	YKL167C	998
YGR220C	YKL170W	962
YGR220C	YKR006C	998
YGR220C	YKR085C	997
YGR220C	YLR069C	934

YGR220C	YLR189C	913
YGR220C	YLR312W-A	996
YGR220C	YLR439W	999
YGR220C	YML025C	999
YGR220C	YMR193W	997
YGR220C	YNL005C	998
YGR220C	YNL185C	970
YGR220C	YNL252C	993
YGR220C	YNL284C	977
YGR220C	YNR036C	904
YGR220C	YNR037C	976
YGR220C	YOR150W	985
YGR220C	YPL183W-A	926
YGR221C	YAL041W	923
YGR221C	YDR309C	921
YGR221C	YHR061C	923
YGR222W	Q0045	989
YGR222W	Q0275	999
YGR222W	YER153C	959
YGR222W	YLR067C	909
YGR222W	YMR257C	944
YGR222W	YNR045W	960
YGR224W	YPL058C	921
YGR225W	YDR113C	999
YGR225W	YIR025W	999
YGR227W	YEL002C	990
YGR227W	YFL031W	903
YGR227W	YGL226C-A	988
YGR227W	YJL002C	984
YGR227W	YOR067C	994
YGR227W	YOR085W	989
YGR229C	YBL007C	918
YGR229C	YBL061C	947
YGR229C	YBL101C	920
YGR229C	YBL105C	995
YGR229C	YBR023C	977
YGR229C	YBR041W	923
YGR229C	YBR200W	901
YGR229C	YCR017C	982
YGR229C	YDR351W	923
YGR229C	YDR388W	921
YGR229C	YEL031W	919
YGR229C	YEL061C	995
YGR229C	YER111C	938

YGR229C	YER155C	920
YGR229C	YER167W	982
YGR229C	YER178W	955
YGR229C	YGL045W	936
YGR229C	YGL240W	936
YGR229C	YGR166W	923
YGR229C	YGR185C	997
YGR229C	YHR030C	998
YGR229C	YHR129C	923
YGR229C	YHR142W	953
YGR229C	YHR191C	985
YGR229C	YHR200W	918
YGR229C	YIL041W	907
YGR229C	YJL095W	939
YGR229C	YJL099W	922
YGR229C	YJL183W	920
YGR229C	YKL048C	920
YGR229C	YKL190W	967
YGR229C	YLR039C	918
YGR229C	YLR332W	984
YGR229C	YLR342W	940
YGR229C	YLR371W	983
YGR229C	YLR384C	918
YGR229C	YML117W	931
YGR229C	YMR116C	911
YGR229C	YMR294W	987
YGR229C	YMR307W	921
YGR229C	YNL233W	920
YGR229C	YNL271C	972
YGR229C	YNL322C	922
YGR229C	YOL003C	931
YGR229C	YOR028C	971
YGR229C	YOR030W	908
YGR229C	YPL089C	993
YGR229C	YPL240C	944
YGR231C	YAL010C	907
YGR231C	YDR245W	980
YGR231C	YEL036C	990
YGR231C	YGR132C	999
YGR231C	YJL183W	995
YGR231C	YLL006W	906
YGR231C	YLR342W	972
YGR231C	YNL098C	908
YGR231C	YOL009C	917

YGR231C	YPL050C	994
YGR232W	YDL007W	998
YGR232W	YDL097C	998
YGR232W	YDL147W	998
YGR232W	YDR363W-A	954
YGR232W	YDR394W	999
YGR232W	YDR427W	999
YGR232W	YER012W	956
YGR232W	YER021W	999
YGR232W	YFL007W	973
YGR232W	YFR004W	998
YGR232W	YFR010W	992
YGR232W	YFR052W	998
YGR232W	YGL004C	980
YGR232W	YGL048C	999
YGR232W	YGL141W	954
YGR232W	YHL030W	997
YGR232W	YHR027C	999
YGR232W	YHR200W	999
YGR232W	YIL075C	999
YGR232W	YIL112W	999
YGR232W	YKL145W	999
YGR232W	YLL039C	903
YGR232W	YLR421C	999
YGR232W	YOR117W	999
YGR232W	YOR259C	998
YGR232W	YOR261C	999
YGR232W	YPR108W	998
YGR233C	YBR092C	953
YGR233C	YBR093C	958
YGR233C	YDL106C	922
YGR233C	YFR034C	999
YGR233C	YHR071W	992
YGR233C	YIL050W	996
YGR233C	YJL005W	998
YGR233C	YNL138W	999
YGR233C	YOL001W	999
YGR233C	YPL031C	999
YGR234W	YDR108W	901
YGR234W	YDR127W	920
YGR234W	YNL085W	950
YGR238C	YHR158C	999
YGR239C	YDR023W	996
YGR239C	YDR142C	999

YGR239C	YGL153W	902
YGR239C	YHR160C	995
YGR239C	YIL160C	967
YGR239C	YLR191W	979
YGR240C	YAL038W	960
YGR240C	YBR196C	961
YGR240C	YBR218C	908
YGR240C	YDR127W	999
YGR240C	YFR053C	907
YGR240C	YGL062W	908
YGR240C	YGL253W	929
YGR240C	YIL107C	981
YGR240C	YJL155C	967
YGR240C	YKL060C	949
YGR240C	YKR097W	901
YGR240C	YLR180W	975
YGR240C	YLR212C	917
YGR240C	YLR377C	955
YGR240C	YMR205C	999
YGR240C	YMR223W	902
YGR240C	YOL136C	976
YGR240C	YPR074C	909
YGR241C	YBL047C	973
YGR241C	YGL206C	956
YGR241C	YIR006C	968
YGR241C	YNL092W	911
YGR241C	YPR178W	998
YGR244C	YDR148C	993
YGR244C	YIL125W	969
YGR244C	YJL045W	957
YGR244C	YKL148C	959
YGR244C	YLL041C	976
YGR244C	YOR142W	999
YGR245C	YCR072C	999
YGR245C	YDR101C	999
YGR245C	YER006W	999
YGR245C	YER126C	999
YGR245C	YGL099W	976
YGR245C	YGR103W	999
YGR245C	YHR170W	956
YGR245C	YHR197W	999
YGR245C	YKL009W	986
YGR245C	YKR048C	999
YGR245C	YKR081C	992

YGR245C	YLR074C	984
YGR245C	YLR106C	999
YGR245C	YNL002C	999
YGR245C	YNL110C	999
YGR245C	YNL182C	999
YGR245C	YNR053C	999
YGR245C	YOR063W	935
YGR245C	YPL093W	999
YGR245C	YPR016C	999
YGR246C	YAL001C	978
YGR246C	YBR123C	984
YGR246C	YDR005C	936
YGR246C	YER148W	999
YGR246C	YGR047C	999
YGR246C	YJL011C	990
YGR246C	YNL039W	999
YGR246C	YNR003C	998
YGR246C	YOR116C	901
YGR248W	YDR248C	925
YGR248W	YGR256W	987
YGR248W	YHR183W	987
YGR248W	YKL205W	939
YGR248W	YNL241C	994
YGR250C	YIR001C	999
YGR252W	YBL052C	994
YGR252W	YBR009C	999
YGR252W	YBR010W	999
YGR252W	YBR081C	999
YGR252W	YBR111W-A	959
YGR252W	YBR198C	999
YGR252W	YBR289W	976
YGR252W	YCL010C	999
YGR252W	YDR028C	920
YGR252W	YDR145W	999
YGR252W	YDR167W	999
YGR252W	YDR176W	999
YGR252W	YDR216W	936
YGR252W	YDR225W	956
YGR252W	YDR359C	973
YGR252W	YDR392W	999
YGR252W	YDR448W	999
YGR252W	YDR477W	918
YGR252W	YEL009C	974
YGR252W	YER068W	970

YGR252W	YER148W	999
YGR252W	YER161C	968
YGR252W	YER164W	996
YGR252W	YFL031W	930
YGR252W	YGL066W	999
YGR252W	YGL070C	961
YGR252W	YGL112C	999
YGR252W	YGL207W	966
YGR252W	YGR200C	947
YGR252W	YGR274C	942
YGR252W	YHR041C	980
YGR252W	YHR079C	955
YGR252W	YHR099W	999
YGR252W	YJL115W	952
YGR252W	YKL110C	930
YGR252W	YKR008W	976
YGR252W	YLR055C	999
YGR252W	YLR384C	944
YGR252W	YLR442C	911
YGR252W	YMR223W	999
YGR252W	YMR227C	997
YGR252W	YMR236W	999
YGR252W	YNL030W	999
YGR252W	YNL031C	999
YGR252W	YNL236W	997
YGR252W	YNL330C	998
YGR252W	YOL004W	987
YGR252W	YOL012C	998
YGR252W	YOL148C	999
YGR252W	YOR023C	965
YGR252W	YOR244W	958
YGR252W	YOR290C	999
YGR252W	YPL016W	970
YGR252W	YPL047W	999
YGR252W	YPL086C	996
YGR252W	YPL248C	910
YGR252W	YPL254W	999
YGR253C	YBL041W	999
YGR253C	YBR173C	917
YGR253C	YDL097C	933
YGR253C	YDL147W	940
YGR253C	YDR394W	910
YGR253C	YDR427W	916
YGR253C	YER012W	999

YGR253C	YER021W	934
YGR253C	YER094C	999
YGR253C	YFL007W	996
YGR253C	YFR004W	955
YGR253C	YFR050C	999
YGR253C	YFR052W	909
YGR253C	YGL011C	999
YGR253C	YGL048C	934
YGR253C	YGR135W	999
YGR253C	YHR027C	920
YGR253C	YHR200W	948
YGR253C	YIL007C	903
YGR253C	YIL075C	901
YGR253C	YJL001W	999
YGR253C	YML092C	999
YGR253C	YMR314W	999
YGR253C	YOL038W	999
YGR253C	YOR157C	999
YGR253C	YOR259C	913
YGR253C	YOR261C	906
YGR253C	YOR362C	999
YGR253C	YPL144W	980
YGR253C	YPR103W	999
YGR253C	YPR108W	918
YGR254W	YAL038W	983
YGR254W	YBL105C	997
YGR254W	YBR218C	907
YGR254W	YCR012W	999
YGR254W	YDR050C	922
YGR254W	YGL062W	907
YGR254W	YHR013C	928
YGR254W	YHR174W	999
YGR254W	YKL152C	938
YGR254W	YKR097W	960
YGR254W	YMR323W	902
YGR254W	YNR001C	917
YGR254W	YOR347C	931
YGR254W	YOR393W	902
YGR254W	YPL281C	902
YGR255C	YBR003W	916
YGR255C	YDR204W	919
YGR255C	YGL119W	916
YGR255C	YLR201C	951
YGR255C	YML110C	994

YGR255C	YNR041C	907
YGR255C	YOL096C	992
YGR256W	YCR073W-A	945
YGR256W	YDR248C	967
YGR256W	YGR248W	987
YGR256W	YHR163W	991
YGR256W	YHR183W	927
YGR256W	YJL121C	919
YGR256W	YNL241C	973
YGR256W	YNR034W	937
YGR256W	YOR095C	936
YGR258C	YAR007C	989
YGR258C	YBR114W	971
YGR258C	YBR272C	989
YGR258C	YCR066W	976
YGR258C	YDR174W	990
YGR258C	YDR311W	999
YGR258C	YDR317W	938
YGR258C	YDR460W	981
YGR258C	YEL037C	955
YGR258C	YER162C	997
YGR258C	YER171W	999
YGR258C	YGL043W	984
YGR258C	YGL058W	956
YGR258C	YIL128W	934
YGR258C	YIL143C	999
YGR258C	YJR035W	993
YGR258C	YJR052W	970
YGR258C	YKL113C	948
YGR258C	YKL114C	904
YGR258C	YLR005W	997
YGR258C	YML032C	992
YGR258C	YML095C	998
YGR258C	YMR201C	998
YGR258C	YNL312W	991
YGR258C	YOL090W	950
YGR258C	YPL022W	997
YGR261C	YBR288C	999
YGR261C	YDR320C	915
YGR261C	YJL024C	999
YGR261C	YOR042W	919
YGR261C	YOR070C	911
YGR261C	YPL195W	999
YGR262C	YDL220C	949

YGR262C	YDR098C	941
YGR262C	YER174C	998
YGR262C	YJL184W	999
YGR262C	YKR038C	999
YGR262C	YKR095W-A	993
YGR262C	YML036W	999
YGR264C	YBL076C	979
YGR264C	YDR037W	918
YGR264C	YDR341C	926
YGR264C	YDR502C	907
YGR264C	YER091C	950
YGR264C	YGL105W	999
YGR264C	YGL245W	999
YGR264C	YHR020W	925
YGR264C	YHR091C	900
YGR264C	YLL018C	935
YGR264C	YLR180W	907
YGR264C	YOR168W	948
YGR264C	YPL040C	950
YGR264C	YPL160W	954
YGR266W	YOR153W	949
YGR267C	YIR034C	909
YGR267C	YKL067W	925
YGR268C	YBL007C	924
YGR268C	YDR388W	917
YGR268C	YER125W	901
YGR268C	YFR024C-A	981
YGR268C	YHR016C	956
YGR268C	YOR124C	950
YGR268C	YOR138C	990
YGR270W	YGL207W	986
YGR270W	YOR038C	927
YGR271W	YBR055C	901
YGR271W	YHR165C	913
YGR271W	YLR147C	901
YGR271W	YLR275W	904
YGR271W	YPR082C	903
YGR274C	YAL034C	933
YGR274C	YBR081C	988
YGR274C	YBR198C	999
YGR274C	YCR042C	999
YGR274C	YDR145W	999
YGR274C	YDR167W	999
YGR274C	YDR176W	998

YGR274C	YDR392W	991
YGR274C	YDR448W	999
YGR274C	YER148W	999
YGR274C	YGL066W	988
YGR274C	YGL112C	999
YGR274C	YGL127C	977
YGR274C	YGL150C	923
YGR274C	YGR097W	960
YGR274C	YGR252W	942
YGR274C	YJR140C	923
YGR274C	YKL058W	984
YGR274C	YLR055C	931
YGR274C	YML015C	999
YGR274C	YML098W	999
YGR274C	YML114C	999
YGR274C	YMR005W	999
YGR274C	YMR227C	999
YGR274C	YMR236W	999
YGR274C	YNL216W	959
YGR274C	YNL236W	975
YGR274C	YOL051W	920
YGR274C	YOL148C	931
YGR274C	YOR174W	943
YGR274C	YOR194C	984
YGR274C	YPL011C	999
YGR274C	YPL129W	999
YGR274C	YPR070W	912
YGR274C	YPR086W	991
YGR275W	YCR020W-B	994
YGR275W	YCR052W	999
YGR275W	YDR303C	995
YGR275W	YFR037C	999
YGR275W	YGR056W	988
YGR275W	YHL025W	929
YGR275W	YIL126W	999
YGR275W	YJL176C	997
YGR275W	YKR008W	995
YGR275W	YLR033W	999
YGR275W	YLR321C	999
YGR275W	YLR357W	999
YGR275W	YML127W	995
YGR275W	YMR033W	999
YGR275W	YMR091C	999
YGR275W	YOR290C	990

YGR275W	YPR034W	999
YGR276C	YDL051W	904
YGR276C	YLR059C	940
YGR277C	YDR196C	978
YGR278W	YAL032C	999
YGR278W	YDL209C	996
YGR278W	YDR364C	968
YGR278W	YDR416W	997
YGR278W	YER172C	992
YGR278W	YHR165C	998
YGR278W	YKL095W	929
YGR278W	YKL173W	997
YGR278W	YLL036C	999
YGR278W	YLR117C	997
YGR278W	YML049C	974
YGR278W	YMR213W	999
YGR278W	YMR288W	993
YGR278W	YPL151C	997
YGR279C	YDR077W	909
YGR279C	YMR305C	995
YGR280C	YHR072W-A	996
YGR280C	YKR092C	975
YGR281W	YBL005W	934
YGR281W	YDR011W	991
YGR281W	YDR406W	920
YGR281W	YGL013C	959
YGR281W	YOR153W	999
YGR281W	YOR328W	922
YGR282C	YBR023C	901
YGR282C	YDL042C	941
YGR282C	YGR032W	924
YGR282C	YIL162W	911
YGR282C	YLR300W	909
YGR283C	YDL208W	946
YGR283C	YHR089C	984
YGR283C	YLR175W	946
YGR283C	YMR310C	999
YGR284C	YFL046W	998
YGR284C	YHR079C	916
YGR284C	YML012W	906
YGR285C	YAL003W	973
YGR285C	YAR014C	950
YGR285C	YAR018C	950
YGR285C	YBR158W	977

YGR285C	YDR457W	908
YGR285C	YGL018C	908
YGR285C	YGL128C	945
YGR285C	YHR064C	999
YGR285C	YJL080C	999
YGR285C	YMR116C	946
YGR285C	YNL227C	908
YGR285C	YOR254C	918
YGR285C	YPR189W	997
YGR286C	YDR232W	930
YGR286C	YNR057C	985
YGR286C	YNR058W	986
YGR289C	YPL240C	902
YGR292W	YBR299W	947
YGR295C	YFL062W	994
YHL001W	YAL035W	924
YHL001W	YBL027W	997
YHL001W	YBL072C	996
YHL001W	YBL087C	933
YHL001W	YBL092W	998
YHL001W	YBR031W	998
YHL001W	YBR048W	997
YHL001W	YBR084C-A	948
YHL001W	YBR118W	907
YHL001W	YBR181C	995
YHL001W	YBR189W	999
YHL001W	YBR191W	999
YHL001W	YCR031C	999
YHL001W	YDL061C	996
YHL001W	YDL075W	999
YHL001W	YDL081C	938
YHL001W	YDL082W	997
YHL001W	YDL083C	968
YHL001W	YDL130W	951
YHL001W	YDL136W	997
YHL001W	YDL191W	930
YHL001W	YDR012W	994
YHL001W	YDR025W	921
YHL001W	YDR064W	999
YHL001W	YDR382W	999
YHL001W	YDR385W	908
YHL001W	YDR418W	999
YHL001W	YDR447C	996
YHL001W	YDR450W	921

YHL001W	YDR471W	999
YHL001W	YDR500C	958
YHL001W	YEL054C	928
YHL001W	YER056C-A	994
YHL001W	YER074W	995
YHL001W	YER102W	961
YHL001W	YER117W	964
YHL001W	YER131W	979
YHL001W	YFL034C-A	915
YHL001W	YFR031C-A	995
YHL001W	YFR032C-A	946
YHL001W	YGL014W	948
YHL001W	YGL030W	999
YHL001W	YGL031C	966
YHL001W	YGL076C	999
YHL001W	YGL103W	999
YHL001W	YGL123W	999
YHL001W	YGL135W	996
YHL001W	YGL147C	996
YHL001W	YGL189C	922
YHL001W	YGR027C	999
YHL001W	YGR034W	995
YHL001W	YGR085C	999
YHL001W	YGR118W	999
YHL001W	YGR148C	998
YHL001W	YGR214W	998
YHL001W	YHL015W	992
YHL001W	YHL033C	999
YHL001W	YHR010W	991
YHL001W	YHR021C	928
YHL001W	YHR141C	998
YHL001W	YHR203C	998
YHL001W	YIL018W	994
YHL001W	YIL052C	999
YHL001W	YIL069C	996
YHL001W	YIL133C	996
YHL001W	YIL148W	951
YHL001W	YJL136C	982
YHL001W	YJL177W	999
YHL001W	YJL189W	958
YHL001W	YJL190C	997
YHL001W	YJL191W	997
YHL001W	YJR094W-A	977
YHL001W	YJR123W	999

YHL001W	YJR145C	924
YHL001W	YKL006W	999
YHL001W	YKL156W	995
YHL001W	YKL180W	999
YHL001W	YKR057W	930
YHL001W	YKR094C	906
YHL001W	YLL045C	998
YHL001W	YLR029C	992
YHL001W	YLR048W	999
YHL001W	YLR061W	998
YHL001W	YLR075W	999
YHL001W	YLR167W	940
YHL001W	YLR185W	953
YHL001W	YLR264W	997
YHL001W	YLR287C-A	994
YHL001W	YLR325C	924
YHL001W	YLR333C	952
YHL001W	YLR340W	999
YHL001W	YLR344W	978
YHL001W	YLR367W	981
YHL001W	YLR388W	999
YHL001W	YLR406C	918
YHL001W	YLR441C	995
YHL001W	YLR448W	999
YHL001W	YML024W	995
YHL001W	YML026C	921
YHL001W	YML063W	999
YHL001W	YML073C	996
YHL001W	YMR121C	925
YHL001W	YMR142C	998
YHL001W	YMR194W	999
YHL001W	YMR230W	960
YHL001W	YMR242C	996
YHL001W	YMR260C	911
YHL001W	YNL067W	994
YHL001W	YNL069C	995
YHL001W	YNL096C	942
YHL001W	YNL162W	931
YHL001W	YNL178W	999
YHL001W	YNL301C	964
YHL001W	YNL302C	925
YHL001W	YOL039W	953
YHL001W	YOL040C	998
YHL001W	YOL120C	922

YHL001W	YOL121C	925
YHL001W	YOL127W	999
YHL001W	YOR063W	999
YHL001W	YOR096W	999
YHL001W	YOR133W	925
YHL001W	YOR167C	970
YHL001W	YOR182C	903
YHL001W	YOR234C	997
YHL001W	YOR293W	976
YHL001W	YOR312C	996
YHL001W	YOR369C	949
YHL001W	YPL037C	950
YHL001W	YPL079W	978
YHL001W	YPL081W	957
YHL001W	YPL090C	994
YHL001W	YPL131W	978
YHL001W	YPL143W	986
YHL001W	YPL198W	997
YHL001W	YPL220W	997
YHL001W	YPL249C-A	997
YHL001W	YPR043W	943
YHL001W	YPR080W	907
YHL001W	YPR102C	995
YHL001W	YPR132W	963
YHL002W	YCL008C	983
YHL002W	YER125W	964
YHL002W	YIL156W	933
YHL002W	YKL213C	951
YHL002W	YNR006W	999
YHL003C	YBR183W	997
YHL003C	YKL008C	999
YHL003C	YLR350W	907
YHL003C	YMR272C	945
YHL003C	YMR298W	999
YHL003C	YPL087W	997
YHL004W	Q0140	991
YHL004W	YBL090W	999
YHL004W	YBR146W	999
YHL004W	YBR251W	999
YHL004W	YDR036C	999
YHL004W	YDR041W	999
YHL004W	YDR116C	913
YHL004W	YDR175C	997
YHL004W	YDR337W	999

YHL004W	YDR347W	999
YHL004W	YGL129C	999
YHL004W	YGR084C	999
YHL004W	YGR165W	997
YHL004W	YGR215W	999
YHL004W	YIL093C	999
YHL004W	YJL063C	948
YHL004W	YJR101W	999
YHL004W	YJR113C	999
YHL004W	YKL003C	900
YHL004W	YKL155C	999
YHL004W	YMR158W	999
YHL004W	YMR188C	999
YHL004W	YNL081C	904
YHL004W	YNL137C	999
YHL004W	YNL306W	999
YHL004W	YNR036C	986
YHL004W	YNR037C	999
YHL004W	YOR150W	962
YHL004W	YOR158W	999
YHL004W	YOR204W	985
YHL004W	YOR243C	915
YHL004W	YPL013C	999
YHL004W	YPL118W	999
YHL006C	YDR078C	994
YHL006C	YLR234W	982
YHL006C	YLR376C	989
YHL006C	YMR190C	979
YHL006C	YPL024W	908
YHL007C	YAL024C	999
YHL007C	YAL041W	992
YHL007C	YBL002W	910
YHL007C	YBR133C	943
YHL007C	YBR160W	979
YHL007C	YBR200W	999
YHL007C	YCL032W	914
YHL007C	YDR099W	932
YHL007C	YDR103W	999
YHL007C	YDR162C	991
YHL007C	YDR224C	909
YHL007C	YDR309C	964
YHL007C	YER094C	942
YHL007C	YER118C	991
YHL007C	YER177W	937

YHL007C	YFL026W	909
YHL007C	YFL039C	975
YHL007C	YGL038C	976
YHL007C	YHR005C	922
YHL007C	YHR030C	901
YHL007C	YHR061C	965
YHL007C	YHR084W	999
YHL007C	YHR152W	956
YHL007C	YJR086W	966
YHL007C	YKL178C	986
YHL007C	YLL039C	970
YHL007C	YLR006C	967
YHL007C	YLR229C	999
YHL007C	YLR362W	999
YHL007C	YNL098C	984
YHL007C	YNL298W	999
YHL007C	YOL113W	986
YHL007C	YOR212W	999
YHL007C	YPL256C	996
YHL011C	YBL068W	978
YHL011C	YBR117C	900
YHL011C	YCR036W	915
YHL011C	YER099C	903
YHL011C	YHR030C	976
YHL011C	YKL181W	999
YHL011C	YMR300C	914
YHL011C	YOL061W	987
YHL011C	YOR043W	968
YHL011C	YOR095C	914
YHL011C	YPR074C	901
YHL012W	YBR019C	902
YHL012W	YFR015C	901
YHL012W	YLR258W	901
YHL012W	YPR160W	903
YHL015W	YAL035W	913
YHL015W	YBL027W	921
YHL015W	YBL072C	998
YHL015W	YBL087C	911
YHL015W	YBL092W	976
YHL015W	YBR031W	958
YHL015W	YBR048W	992
YHL015W	YBR079C	915
YHL015W	YBR084C-A	955
YHL015W	YBR118W	978

YHL015W	YBR181C	913
YHL015W	YBR189W	999
YHL015W	YBR191W	998
YHL015W	YCR031C	999
YHL015W	YDL061C	999
YHL015W	YDL075W	989
YHL015W	YDL081C	963
YHL015W	YDL082W	925
YHL015W	YDL083C	998
YHL015W	YDL130W	929
YHL015W	YDL136W	960
YHL015W	YDL191W	929
YHL015W	YDR012W	970
YHL015W	YDR025W	920
YHL015W	YDR041W	929
YHL015W	YDR064W	999
YHL015W	YDR382W	986
YHL015W	YDR385W	996
YHL015W	YDR418W	983
YHL015W	YDR447C	938
YHL015W	YDR450W	993
YHL015W	YDR471W	999
YHL015W	YDR500C	947
YHL015W	YEL054C	935
YHL015W	YER025W	950
YHL015W	YER056C-A	986
YHL015W	YER074W	905
YHL015W	YER102W	962
YHL015W	YER117W	954
YHL015W	YER131W	969
YHL015W	YER165W	931
YHL015W	YFL034C-A	919
YHL015W	YFR031C-A	904
YHL015W	YFR032C-A	970
YHL015W	YGL030W	998
YHL015W	YGL031C	953
YHL015W	YGL076C	971
YHL015W	YGL103W	999
YHL015W	YGL123W	999
YHL015W	YGL135W	938
YHL015W	YGL147C	998
YHL015W	YGL189C	912
YHL015W	YGR027C	994
YHL015W	YGR034W	944

YHL015W	YGR085C	999
YHL015W	YGR118W	999
YHL015W	YGR148C	982
YHL015W	YGR214W	999
YHL015W	YHL001W	992
YHL015W	YHL033C	997
YHL015W	YHR010W	976
YHL015W	YHR021C	939
YHL015W	YHR141C	939
YHL015W	YHR203C	969
YHL015W	YIL018W	904
YHL015W	YIL052C	999
YHL015W	YIL069C	905
YHL015W	YIL133C	930
YHL015W	YIL148W	952
YHL015W	YJL136C	967
YHL015W	YJL138C	904
YHL015W	YJL177W	988
YHL015W	YJL189W	933
YHL015W	YJL190C	999
YHL015W	YJL191W	965
YHL015W	YJR007W	907
YHL015W	YJR094W-A	971
YHL015W	YJR123W	999
YHL015W	YJR145C	942
YHL015W	YKL006W	996
YHL015W	YKL156W	979
YHL015W	YKL180W	999
YHL015W	YKR057W	944
YHL015W	YKR059W	942
YHL015W	YKR094C	917
YHL015W	YLL039C	908
YHL015W	YLL045C	955
YHL015W	YLR029C	973
YHL015W	YLR048W	995
YHL015W	YLR061W	986
YHL015W	YLR075W	988
YHL015W	YLR167W	947
YHL015W	YLR185W	944
YHL015W	YLR264W	995
YHL015W	YLR287C-A	911
YHL015W	YLR333C	941
YHL015W	YLR340W	999
YHL015W	YLR344W	979

YHL015W	YLR367W	979
YHL015W	YLR388W	991
YHL015W	YLR406C	932
YHL015W	YLR441C	915
YHL015W	YLR448W	982
YHL015W	YML024W	984
YHL015W	YML026C	941
YHL015W	YML063W	995
YHL015W	YML073C	998
YHL015W	YMR121C	915
YHL015W	YMR142C	926
YHL015W	YMR146C	922
YHL015W	YMR194W	981
YHL015W	YMR230W	998
YHL015W	YMR242C	998
YHL015W	YMR260C	922
YHL015W	YNL067W	992
YHL015W	YNL069C	929
YHL015W	YNL096C	945
YHL015W	YNL162W	939
YHL015W	YNL178W	999
YHL015W	YNL301C	954
YHL015W	YNL302C	939
YHL015W	YOL039W	936
YHL015W	YOL040C	999
YHL015W	YOL120C	927
YHL015W	YOL121C	940
YHL015W	YOL127W	999
YHL015W	YOL139C	920
YHL015W	YOR063W	997
YHL015W	YOR096W	999
YHL015W	YOR133W	972
YHL015W	YOR167C	965
YHL015W	YOR182C	911
YHL015W	YOR198C	990
YHL015W	YOR234C	979
YHL015W	YOR293W	979
YHL015W	YOR312C	949
YHL015W	YOR361C	911
YHL015W	YOR369C	917
YHL015W	YPL079W	962
YHL015W	YPL081W	992
YHL015W	YPL090C	913
YHL015W	YPL131W	994

YHL015W	YPL143W	981
YHL015W	YPL198W	940
YHL015W	YPL220W	966
YHL015W	YPL237W	922
YHL015W	YPL249C-A	955
YHL015W	YPR043W	941
YHL015W	YPR080W	978
YHL015W	YPR102C	989
YHL015W	YPR132W	976
YHL018W	YLR291C	942
YHL018W	YNL113W	915
YHL018W	YPL070W	959
YHL019C	YFL034W	989
YHL019C	YKL135C	999
YHL019C	YLR170C	999
YHL019C	YPL259C	985
YHL019C	YPR029C	999
YHL020C	YDR123C	999
YHL020C	YDR207C	911
YHL020C	YER120W	999
YHL020C	YJL153C	979
YHL020C	YMR165C	911
YHL020C	YOL004W	992
YHL020C	YOL108C	998
YHL021C	YJL140W	917
YHL022C	YBR160W	914
YHL022C	YER179W	997
YHL022C	YGL175C	990
YHL022C	YGL183C	963
YHL022C	YGL213C	999
YHL022C	YHR014W	999
YHL022C	YHR079C-A	989
YHL022C	YHR157W	998
YHL022C	YIR025W	976
YHL022C	YLR329W	999
YHL022C	YML032C	911
YHL022C	YNL250W	997
YHL022C	YPL178W	976
YHL022C	YPR007C	901
YHL024W	YBR114W	936
YHL024W	YER125W	975
YHL024W	YJL106W	921
YHL025W	YBR112C	954
YHL025W	YBR289W	999

YHL025W	YDR073W	999
YHL025W	YFL049W	999
YHL025W	YGR116W	952
YHL025W	YGR275W	929
YHL025W	YJL176C	999
YHL025W	YMR033W	999
YHL025W	YNR023W	999
YHL025W	YOR290C	999
YHL025W	YPL016W	999
YHL025W	YPL129W	999
YHL025W	YPR034W	999
YHL027W	YBR182C	909
YHL027W	YDR043C	970
YHL027W	YGR122W	906
YHL027W	YJL056C	904
YHL027W	YJR102C	939
YHL027W	YLR025W	970
YHL027W	YLR417W	935
YHL027W	YMR077C	946
YHL027W	YMR154C	991
YHL027W	YOR030W	933
YHL027W	YOR275C	995
YHL027W	YPL002C	910
YHL027W	YPL065W	933
YHL027W	YPL084W	928
YHL029C	YCR095C	999
YHL029C	YDR067C	976
YHL029C	YNL032W	999
YHL029C	YNL056W	923
YHL029C	YNL099C	998
YHL030W	YDL007W	999
YHL030W	YDL097C	999
YHL030W	YDL147W	999
YHL030W	YDR394W	998
YHL030W	YDR427W	998
YHL030W	YEL037C	983
YHL030W	YER021W	993
YHL030W	YFL007W	991
YHL030W	YFR004W	999
YHL030W	YFR010W	999
YHL030W	YFR052W	999
YHL030W	YGL048C	997
YHL030W	YGL141W	954
YHL030W	YGR232W	997

YHL030W	YHR027C	977
YHL030W	YHR200W	999
YHL030W	YIL075C	978
YHL030W	YJL001W	924
YHL030W	YKL145W	998
YHL030W	YLR421C	999
YHL030W	YML092C	974
YHL030W	YMR314W	974
YHL030W	YOL038W	997
YHL030W	YOR117W	997
YHL030W	YOR259C	998
YHL030W	YOR261C	999
YHL030W	YPR103W	999
YHL030W	YPR108W	992
YHL031C	YIL004C	999
YHL031C	YJL004C	953
YHL031C	YKL006C-A	999
YHL031C	YKL179C	934
YHL031C	YKL196C	999
YHL031C	YLR026C	999
YHL031C	YLR039C	959
YHL031C	YLR078C	999
YHL031C	YLR262C	944
YHL031C	YLR268W	999
YHL031C	YNL041C	909
YHL031C	YOR036W	998
YHL031C	YOR070C	994
YHL031C	YPL051W	945
YHL032C	YFL054C	954
YHL032C	YGL256W	930
YHL032C	YIL155C	999
YHL032C	YMR303C	945
YHL033C	YAL003W	976
YHL033C	YAL035W	941
YHL033C	YBL027W	999
YHL033C	YBL072C	996
YHL033C	YBL087C	991
YHL033C	YBL092W	995
YHL033C	YBR031W	999
YHL033C	YBR048W	996
YHL033C	YBR084C-A	978
YHL033C	YBR118W	919
YHL033C	YBR181C	994
YHL033C	YBR189W	999

YHL033C	YBR191W	999
YHL033C	YCR031C	999
YHL033C	YDL061C	995
YHL033C	YDL075W	999
YHL033C	YDL081C	943
YHL033C	YDL082W	996
YHL033C	YDL083C	962
YHL033C	YDL130W	948
YHL033C	YDL136W	999
YHL033C	YDL191W	924
YHL033C	YDR012W	999
YHL033C	YDR025W	902
YHL033C	YDR064W	999
YHL033C	YDR382W	999
YHL033C	YDR385W	903
YHL033C	YDR418W	999
YHL033C	YDR447C	996
YHL033C	YDR450W	922
YHL033C	YDR471W	999
YHL033C	YDR500C	972
YHL033C	YEL034W	921
YHL033C	YEL054C	935
YHL033C	YER036C	915
YHL033C	YER056C-A	998
YHL033C	YER074W	995
YHL033C	YER102W	980
YHL033C	YER117W	970
YHL033C	YER131W	966
YHL033C	YFL034C-A	921
YHL033C	YFR031C-A	999
YHL033C	YFR032C-A	935
YHL033C	YGL030W	999
YHL033C	YGL031C	999
YHL033C	YGL076C	999
YHL033C	YGL103W	999
YHL033C	YGL123W	999
YHL033C	YGL135W	999
YHL033C	YGL147C	999
YHL033C	YGL189C	911
YHL033C	YGR027C	997
YHL033C	YGR034W	996
YHL033C	YGR085C	999
YHL033C	YGR118W	999
YHL033C	YGR148C	999

YHL033C	YGR156W	976
YHL033C	YGR214W	999
YHL033C	YHL001W	999
YHL033C	YHL015W	997
YHL033C	YHR010W	976
YHL033C	YHR021C	923
YHL033C	YHR141C	999
YHL033C	YHR170W	979
YHL033C	YHR203C	999
YHL033C	YIL018W	995
YHL033C	YIL052C	999
YHL033C	YIL069C	908
YHL033C	YIL133C	999
YHL033C	YIL148W	968
YHL033C	YJL136C	981
YHL033C	YJL177W	999
YHL033C	YJL189W	941
YHL033C	YJL190C	996
YHL033C	YJL191W	987
YHL033C	YJR094W-A	998
YHL033C	YJR123W	999
YHL033C	YJR145C	995
YHL033C	YKL006W	999
YHL033C	YKL156W	974
YHL033C	YKL180W	999
YHL033C	YKR002W	979
YHL033C	YKR057W	932
YHL033C	YKR094C	908
YHL033C	YLL045C	999
YHL033C	YLR029C	999
YHL033C	YLR048W	999
YHL033C	YLR060W	904
YHL033C	YLR061W	998
YHL033C	YLR075W	999
YHL033C	YLR167W	962
YHL033C	YLR185W	995
YHL033C	YLR249W	938
YHL033C	YLR264W	994
YHL033C	YLR287C-A	914
YHL033C	YLR333C	927
YHL033C	YLR340W	997
YHL033C	YLR344W	998
YHL033C	YLR367W	971
YHL033C	YLR388W	999

YHL033C	YLR398C	974
YHL033C	YLR406C	929
YHL033C	YLR441C	995
YHL033C	YLR448W	999
YHL033C	YML024W	999
YHL033C	YML026C	922
YHL033C	YML063W	999
YHL033C	YML073C	996
YHL033C	YMR121C	924
YHL033C	YMR142C	998
YHL033C	YMR194W	999
YHL033C	YMR230W	947
YHL033C	YMR242C	996
YHL033C	YMR260C	913
YHL033C	YNL067W	997
YHL033C	YNL069C	996
YHL033C	YNL096C	942
YHL033C	YNL162W	937
YHL033C	YNL178W	999
YHL033C	YNL209W	940
YHL033C	YNL301C	997
YHL033C	YNL302C	920
YHL033C	YOL039W	944
YHL033C	YOL040C	937
YHL033C	YOL120C	917
YHL033C	YOL121C	920
YHL033C	YOL127W	999
YHL033C	YOR063W	999
YHL033C	YOR096W	999
YHL033C	YOR133W	935
YHL033C	YOR167C	945
YHL033C	YOR182C	914
YHL033C	YOR234C	979
YHL033C	YOR293W	971
YHL033C	YOR294W	984
YHL033C	YOR312C	996
YHL033C	YOR369C	933
YHL033C	YPL037C	999
YHL033C	YPL079W	977
YHL033C	YPL081W	953
YHL033C	YPL090C	994
YHL033C	YPL131W	999
YHL033C	YPL143W	982
YHL033C	YPL198W	997

YHL033C	YPL220W	999
YHL033C	YPL249C-A	952
YHL033C	YPR043W	948
YHL033C	YPR080W	906
YHL033C	YPR102C	999
YHL033C	YPR132W	974
YHL034C	YBL072C	982
YHL034C	YBR034C	981
YHL034C	YCL011C	979
YHL034C	YCL037C	978
YHL034C	YDL014W	929
YHL034C	YDL083C	978
YHL034C	YDR064W	935
YHL034C	YER102W	995
YHL034C	YER165W	975
YHL034C	YGL049C	977
YHL034C	YGL173C	990
YHL034C	YGR162W	993
YHL034C	YJL190C	981
YHL034C	YJR123W	982
YHL034C	YJR145C	996
YHL034C	YLL045C	987
YHL034C	YMR125W	979
YHL034C	YOL139C	998
YHL034C	YOR096W	939
YHL034C	YOR312C	995
YHL034C	YPL131W	981
YHL034C	YPR080W	956
YHL036W	YPL240C	902
YHL038C	Q0105	988
YHL050C	YEL077C	929
YHL050C	YHR219W	976
YHL050C	YJL225C	998
YHR001W	YPR173C	978
YHR001W-A	Q0105	989
YHR001W-A	YBL045C	990
YHR001W-A	YDR375C	949
YHR001W-A	YDR529C	994
YHR001W-A	YEL024W	995
YHR001W-A	YFR033C	999
YHR001W-A	YGR183C	996
YHR001W-A	YJL166W	994
YHR001W-A	YJR048W	910
YHR001W-A	YLR395C	938

YHR001W-A	YOR065W	999
YHR001W-A	YPR191W	991
YHR004C	YAL009W	999
YHR004C	YBR164C	905
YHR004C	YDL116W	952
YHR004C	YDR320C	921
YHR004C	YJR042W	918
YHR004C	YKL057C	918
YHR004C	YKL212W	901
YHR004C	YMR165C	942
YHR004C	YPL195W	905
YHR005C	YBL016W	999
YHR005C	YBR097W	987
YHR005C	YDL159W	992
YHR005C	YDR103W	993
YHR005C	YER020W	993
YHR005C	YFL026W	999
YHR005C	YHL007C	922
YHR005C	YHR084W	980
YHR005C	YJL080C	975
YHR005C	YJL197W	905
YHR005C	YJR086W	999
YHR005C	YKL178C	997
YHR005C	YLR240W	935
YHR005C	YLR362W	999
YHR005C	YLR452C	999
YHR005C	YML104C	925
YHR005C	YMR069W	918
YHR005C	YOR107W	970
YHR005C	YOR212W	999
YHR005C	YOR301W	944
YHR005C-A	YBR091C	999
YHR005C-A	YDL217C	999
YHR005C-A	YEL020W-A	999
YHR005C-A	YGR181W	986
YHR005C-A	YJL054W	960
YHR005C-A	YJR135W-A	986
YHR005C-A	YNR017W	978
YHR005C-A	YOR297C	998
YHR007C	YDR213W	909
YHR007C	YER044C	999
YHR007C	YGL001C	999
YHR007C	YGL012W	999
YHR007C	YGR060W	999

YHR007C	YGR175C	937
YHR007C	YHR042W	968
YHR007C	YHR072W	999
YHR007C	YHR190W	999
YHR007C	YLR056W	980
YHR007C	YLR100W	999
YHR007C	YML008C	999
YHR007C	YMR202W	999
YHR007C	YNL280C	999
YHR007C	YOR070C	928
YHR007C	YPL170W	949
YHR008C	YBR290W	957
YHR008C	YDR256C	995
YHR008C	YGL167C	993
YHR008C	YGR088W	993
YHR008C	YHR205W	912
YHR008C	YJR104C	999
YHR010W	YAL035W	929
YHR010W	YBL072C	900
YHR010W	YBL087C	901
YHR010W	YBL092W	930
YHR010W	YBR031W	948
YHR010W	YBR048W	914
YHR010W	YBR084C-A	926
YHR010W	YBR084W	972
YHR010W	YBR189W	992
YHR010W	YBR191W	996
YHR010W	YCR031C	971
YHR010W	YDL061C	973
YHR010W	YDL075W	979
YHR010W	YDL081C	910
YHR010W	YDL082W	996
YHR010W	YDL130W	925
YHR010W	YDL136W	939
YHR010W	YDL191W	908
YHR010W	YDR012W	960
YHR010W	YDR064W	985
YHR010W	YDR382W	980
YHR010W	YDR418W	999
YHR010W	YDR447C	907
YHR010W	YDR450W	903
YHR010W	YDR471W	973
YHR010W	YDR500C	939
YHR010W	YEL054C	997

YHR010W	YER006W	983
YHR010W	YER056C-A	978
YHR010W	YER074W	907
YHR010W	YER102W	938
YHR010W	YER117W	938
YHR010W	YER131W	974
YHR010W	YFL034C-A	911
YHR010W	YFR031C-A	996
YHR010W	YFR032C-A	907
YHR010W	YGL030W	999
YHR010W	YGL031C	965
YHR010W	YGL076C	956
YHR010W	YGL103W	999
YHR010W	YGL123W	978
YHR010W	YGL135W	915
YHR010W	YGL147C	918
YHR010W	YGL189C	911
YHR010W	YGR027C	976
YHR010W	YGR034W	913
YHR010W	YGR085C	969
YHR010W	YGR118W	993
YHR010W	YGR148C	982
YHR010W	YGR214W	979
YHR010W	YHL001W	991
YHR010W	YHL015W	976
YHR010W	YHL033C	976
YHR010W	YHR021C	905
YHR010W	YHR052W	992
YHR010W	YHR141C	928
YHR010W	YHR203C	949
YHR010W	YIL018W	995
YHR010W	YIL052C	998
YHR010W	YIL069C	907
YHR010W	YIL133C	915
YHR010W	YIL148W	939
YHR010W	YJL136C	951
YHR010W	YJL177W	973
YHR010W	YJL189W	924
YHR010W	YJL190C	903
YHR010W	YJL191W	962
YHR010W	YJR094W-A	955
YHR010W	YJR123W	979
YHR010W	YJR145C	993
YHR010W	YKL006W	997

YHR010W	YKL156W	975
YHR010W	YKL180W	999
YHR010W	YKR057W	912
YHR010W	YLL045C	995
YHR010W	YLR029C	978
YHR010W	YLR048W	987
YHR010W	YLR061W	994
YHR010W	YLR075W	965
YHR010W	YLR167W	932
YHR010W	YLR185W	935
YHR010W	YLR264W	978
YHR010W	YLR287C-A	910
YHR010W	YLR333C	919
YHR010W	YLR340W	942
YHR010W	YLR344W	959
YHR010W	YLR367W	999
YHR010W	YLR388W	990
YHR010W	YLR406C	913
YHR010W	YLR441C	905
YHR010W	YLR448W	969
YHR010W	YML024W	975
YHR010W	YML026C	903
YHR010W	YML063W	986
YHR010W	YML073C	906
YHR010W	YMR121C	912
YHR010W	YMR142C	922
YHR010W	YMR194W	998
YHR010W	YMR230W	954
YHR010W	YMR242C	905
YHR010W	YMR290C	935
YHR010W	YNL067W	967
YHR010W	YNL069C	996
YHR010W	YNL096C	922
YHR010W	YNL162W	920
YHR010W	YNL178W	977
YHR010W	YNL301C	955
YHR010W	YNL302C	907
YHR010W	YNR053C	974
YHR010W	YOL039W	923
YHR010W	YOL040C	902
YHR010W	YOL120C	914
YHR010W	YOL121C	928
YHR010W	YOL127W	998
YHR010W	YOR063W	998

YHR010W	YOR096W	999
YHR010W	YOR133W	913
YHR010W	YOR167C	963
YHR010W	YOR182C	910
YHR010W	YOR234C	974
YHR010W	YOR293W	980
YHR010W	YOR312C	998
YHR010W	YOR369C	925
YHR010W	YPL079W	962
YHR010W	YPL081W	937
YHR010W	YPL093W	982
YHR010W	YPL131W	956
YHR010W	YPL143W	967
YHR010W	YPL198W	997
YHR010W	YPL220W	944
YHR010W	YPL249C-A	998
YHR010W	YPR043W	922
YHR010W	YPR102C	995
YHR010W	YPR132W	941
YHR010W	YPR189W	975
YHR011W	YDR023W	913
YHR011W	YGR208W	912
YHR012W	YJL053W	999
YHR012W	YJL154C	999
YHR012W	YOR069W	999
YHR012W	YOR132W	999
YHR013C	YDL040C	999
YHR013C	YGR254W	928
YHR013C	YKR101W	941
YHR013C	YMR116C	957
YHR013C	YOR253W	999
YHR014W	YBR098W	981
YHR014W	YBR160W	971
YHR014W	YER044C-A	983
YHR014W	YGR044C	902
YHR014W	YHL022C	999
YHR014W	YHR086W	911
YHR014W	YHR152W	927
YHR014W	YHR157W	928
YHR014W	YHR185C	912
YHR014W	YJR021C	986
YHR014W	YLR329W	998
YHR014W	YML032C	949
YHR014W	YMR133W	951

YHR014W	YNL250W	998
YHR014W	YOR195W	910
YHR014W	YOR351C	998
YHR015W	YDR172W	994
YHR016C	YBL007C	999
YHR016C	YCL034W	937
YHR016C	YCR088W	946
YHR016C	YDR388W	907
YHR016C	YFR024C-A	929
YHR016C	YGL153W	928
YHR016C	YGR268C	956
YHR016C	YOR042W	976
YHR016C	YOR181W	997
YHR018C	YDR341C	916
YHR018C	YHR091C	902
YHR018C	YJL088W	983
YHR018C	YLR359W	972
YHR018C	YMR062C	963
YHR018C	YOL058W	999
YHR018C	YPL111W	970
YHR019C	YBL080C	917
YHR019C	YDR321W	932
YHR019C	YGR124W	979
YHR019C	YMR293C	915
YHR019C	YPR145W	930
YHR020W	YBL076C	951
YHR020W	YBR069C	961
YHR020W	YDR037W	912
YHR020W	YEL022W	968
YHR020W	YGR175C	961
YHR020W	YGR218W	999
YHR020W	YGR264C	925
YHR020W	YJR077C	968
YHR020W	YKL104C	972
YHR020W	YLL018C	918
YHR020W	YLR180W	975
YHR020W	YOR048C	948
YHR020W	YOR168W	927
YHR020W	YPL160W	978
YHR021C	YBL027W	921
YHR021C	YBL072C	931
YHR021C	YBL087C	930
YHR021C	YBL092W	926
YHR021C	YBR031W	927

YHR021C	YBR048W	918
YHR021C	YBR084C-A	921
YHR021C	YBR118W	908
YHR021C	YBR181C	903
YHR021C	YBR189W	917
YHR021C	YBR191W	926
YHR021C	YCR031C	935
YHR021C	YDL061C	912
YHR021C	YDL075W	922
YHR021C	YDL081C	926
YHR021C	YDL082W	928
YHR021C	YDL130W	902
YHR021C	YDL136W	927
YHR021C	YDL191W	927
YHR021C	YDR012W	927
YHR021C	YDR025W	918
YHR021C	YDR064W	956
YHR021C	YDR382W	920
YHR021C	YDR385W	912
YHR021C	YDR418W	930
YHR021C	YDR447C	929
YHR021C	YDR450W	918
YHR021C	YDR471W	905
YHR021C	YDR500C	916
YHR021C	YEL054C	930
YHR021C	YER056C-A	903
YHR021C	YER074W	918
YHR021C	YER102W	931
YHR021C	YER117W	930
YHR021C	YER131W	921
YHR021C	YER165W	908
YHR021C	YFL034C-A	915
YHR021C	YFR031C-A	904
YHR021C	YFR032C-A	951
YHR021C	YGL030W	947
YHR021C	YGL031C	907
YHR021C	YGL076C	931
YHR021C	YGL123W	913
YHR021C	YGL135W	920
YHR021C	YGL147C	928
YHR021C	YGL189C	921
YHR021C	YGR027C	921
YHR021C	YGR034W	925
YHR021C	YGR085C	920

YHR021C	YGR118W	932
YHR021C	YGR148C	907
YHR021C	YGR214W	926
YHR021C	YHL001W	928
YHR021C	YHL015W	939
YHR021C	YHL033C	923
YHR021C	YHR010W	905
YHR021C	YHR141C	949
YHR021C	YHR203C	922
YHR021C	YIL018W	904
YHR021C	YIL052C	903
YHR021C	YIL069C	918
YHR021C	YIL133C	924
YHR021C	YIL148W	905
YHR021C	YJL136C	937
YHR021C	YJL177W	919
YHR021C	YJL189W	908
YHR021C	YJL190C	921
YHR021C	YJL191W	935
YHR021C	YJR007W	948
YHR021C	YJR094W-A	931
YHR021C	YJR123W	943
YHR021C	YJR145C	922
YHR021C	YKL006W	927
YHR021C	YKL156W	901
YHR021C	YKL180W	919
YHR021C	YKR057W	937
YHR021C	YKR094C	905
YHR021C	YLL045C	923
YHR021C	YLR029C	917
YHR021C	YLR048W	926
YHR021C	YLR061W	918
YHR021C	YLR075W	927
YHR021C	YLR167W	900
YHR021C	YLR185W	916
YHR021C	YLR264W	913
YHR021C	YLR287C-A	905
YHR021C	YLR325C	931
YHR021C	YLR333C	920
YHR021C	YLR340W	940
YHR021C	YLR344W	925
YHR021C	YLR367W	921
YHR021C	YLR388W	913
YHR021C	YLR406C	922

YHR021C	YLR441C	928
YHR021C	YLR448W	932
YHR021C	YML024W	929
YHR021C	YML026C	918
YHR021C	YML063W	928
YHR021C	YML073C	967
YHR021C	YMR121C	917
YHR021C	YMR142C	927
YHR021C	YMR194W	930
YHR021C	YMR230W	931
YHR021C	YMR242C	931
YHR021C	YMR260C	926
YHR021C	YNL067W	929
YHR021C	YNL069C	922
YHR021C	YNL096C	921
YHR021C	YNL162W	949
YHR021C	YNL178W	950
YHR021C	YNL301C	922
YHR021C	YNL302C	968
YHR021C	YOL039W	904
YHR021C	YOL040C	959
YHR021C	YOL120C	922
YHR021C	YOL121C	934
YHR021C	YOL127W	924
YHR021C	YOR063W	934
YHR021C	YOR096W	921
YHR021C	YOR133W	912
YHR021C	YOR167C	912
YHR021C	YOR182C	905
YHR021C	YOR234C	932
YHR021C	YOR293W	931
YHR021C	YOR312C	931
YHR021C	YOR369C	934
YHR021C	YPL079W	926
YHR021C	YPL081W	917
YHR021C	YPL090C	903
YHR021C	YPL131W	922
YHR021C	YPL143W	932
YHR021C	YPL198W	930
YHR021C	YPL220W	920
YHR021C	YPL249C-A	931
YHR021C	YPR043W	931
YHR021C	YPR080W	908
YHR021C	YPR102C	920

YHR021C	YPR132W	932
YHR023W	YAL029C	999
YHR023W	YBL106C	931
YHR023W	YBR109C	973
YHR023W	YDL117W	913
YHR023W	YFL039C	994
YHR023W	YGL106W	999
YHR023W	YMR001C	903
YHR023W	YMR032W	951
YHR023W	YOR326W	999
YHR023W	YPL242C	997
YHR023W	YPR032W	981
YHR023W	YPR188C	999
YHR024C	YLR163C	999
YHR025W	YCR053W	994
YHR025W	YJR139C	988
YHR026W	YBR011C	970
YHR026W	YBR127C	904
YHR026W	YDL185W	901
YHR026W	YDR500C	937
YHR026W	YEL027W	998
YHR026W	YEL051W	951
YHR026W	YER102W	950
YHR026W	YGR020C	965
YHR026W	YHR039C-A	919
YHR026W	YHR203C	974
YHR026W	YKL080W	939
YHR026W	YLR185W	944
YHR026W	YLR447C	951
YHR026W	YMR307W	912
YHR026W	YNL301C	930
YHR026W	YOR270C	959
YHR026W	YOR332W	957
YHR026W	YPL220W	966
YHR026W	YPL234C	993
YHR026W	YPR036W	924
YHR026W	YPR132W	906
YHR027C	YBL041W	926
YHR027C	YCR014C	970
YHR027C	YCR073C	998
YHR027C	YDL007W	999
YHR027C	YDL025C	927
YHR027C	YDL097C	999
YHR027C	YDL147W	999

YHR027C	YDR320C	970
YHR027C	YDR363W-A	999
YHR027C	YDR394W	999
YHR027C	YDR427W	999
YHR027C	YEL037C	999
YHR027C	YER012W	999
YHR027C	YER021W	999
YHR027C	YER094C	936
YHR027C	YFR004W	999
YHR027C	YFR010W	999
YHR027C	YFR050C	911
YHR027C	YFR052W	999
YHR027C	YGL011C	911
YHR027C	YGL048C	999
YHR027C	YGR135W	911
YHR027C	YGR232W	999
YHR027C	YGR253C	920
YHR027C	YHL030W	977
YHR027C	YHR200W	999
YHR027C	YIL007C	921
YHR027C	YIL075C	999
YHR027C	YIL148W	904
YHR027C	YJL001W	927
YHR027C	YKL145W	999
YHR027C	YKL171W	999
YHR027C	YKR094C	904
YHR027C	YLL039C	993
YHR027C	YLR167W	900
YHR027C	YLR421C	999
YHR027C	YML092C	908
YHR027C	YMR091C	964
YHR027C	YMR191W	972
YHR027C	YMR276W	981
YHR027C	YMR314W	931
YHR027C	YNL244C	999
YHR027C	YOL004W	940
YHR027C	YOL038W	932
YHR027C	YOR117W	999
YHR027C	YOR157C	926
YHR027C	YOR259C	999
YHR027C	YOR261C	999
YHR027C	YOR362C	999
YHR027C	YPL240C	939
YHR027C	YPR103W	935

YHR027C	YPR108W	999
YHR029C	YBL092W	972
YHR029C	YFL059W	935
YHR029C	YGL014W	970
YHR029C	YHR141C	972
YHR029C	YLR287C-A	972
YHR029C	YMR096W	958
YHR029C	YNL333W	935
YHR030C	YBL016W	907
YHR030C	YBL105C	981
YHR030C	YBR160W	996
YHR030C	YBR182C	929
YHR030C	YCR009C	968
YHR030C	YDL127W	969
YHR030C	YDL159W	925
YHR030C	YDR388W	962
YHR030C	YDR436W	970
YHR030C	YER075C	994
YHR030C	YER093C	921
YHR030C	YER111C	999
YHR030C	YGR040W	907
YHR030C	YGR229C	998
YHR030C	YHL007C	901
YHR030C	YHL011C	976
YHR030C	YHR107C	918
YHR030C	YIL113W	999
YHR030C	YIL119C	902
YHR030C	YJL095W	999
YHR030C	YJL128C	905
YHR030C	YJR066W	992
YHR030C	YKL161C	977
YHR030C	YKL190W	990
YHR030C	YKL203C	946
YHR030C	YKL212W	980
YHR030C	YLL021W	999
YHR030C	YLL043W	987
YHR030C	YLR182W	999
YHR030C	YLR342W	965
YHR030C	YLR442C	995
YHR030C	YML016C	996
YHR030C	YMR036C	985
YHR030C	YMR199W	909
YHR030C	YMR307W	925
YHR030C	YNL053W	999

YHR030C	YNL079C	923
YHR030C	YNL271C	988
YHR030C	YNL289W	969
YHR030C	YOR008C	959
YHR030C	YOR208W	980
YHR030C	YOR231W	999
YHR030C	YOR326W	915
YHR030C	YPL089C	999
YHR030C	YPL140C	999
YHR030C	YPR165W	990
YHR031C	YBR136W	986
YHR031C	YCL061C	998
YHR031C	YDR075W	953
YHR031C	YDR110W	910
YHR031C	YDR217C	987
YHR031C	YDR363W	952
YHR031C	YDR369C	911
YHR031C	YER095W	971
YHR031C	YHR164C	980
YHR031C	YJL092W	999
YHR031C	YLR234W	911
YHR031C	YLR288C	975
YHR031C	YML032C	997
YHR031C	YML058W	970
YHR031C	YML061C	979
YHR031C	YMR190C	999
YHR031C	YMR224C	987
YHR031C	YNL250W	987
YHR031C	YNL261W	999
YHR031C	YNL262W	974
YHR031C	YPL024W	955
YHR031C	YPL153C	933
YHR031C	YPR120C	933
YHR033W	YOR323C	983
YHR034C	YCR060W	999
YHR034C	YDR190C	999
YHR034C	YOR310C	976
YHR034C	YPL146C	945
YHR034C	YPL193W	912
YHR034C	YPL235W	999
YHR037W	YER023W	937
YHR037W	YKL015W	955
YHR037W	YLR142W	999
YHR037W	YLR438W	947

YHR037W	YOR375C	924
YHR037W	YPR035W	953
YHR039C	YGL084C	913
YHR039C	YGL226C-A	921
YHR039C-A	YBR011C	900
YHR039C-A	YBR127C	999
YHR039C-A	YDL185W	999
YHR039C-A	YEL027W	919
YHR039C-A	YEL051W	999
YHR039C-A	YGR020C	999
YHR039C-A	YHR026W	919
YHR039C-A	YKL080W	954
YHR039C-A	YLR447C	991
YHR039C-A	YMR054W	999
YHR039C-A	YOR270C	999
YHR039C-A	YOR332W	999
YHR039C-A	YPL234C	918
YHR039C-A	YPR036W	999
YHR041C	YBL093C	999
YHR041C	YBR193C	999
YHR041C	YBR198C	996
YHR041C	YBR253W	999
YHR041C	YBR279W	973
YHR041C	YCR081W	992
YHR041C	YDL005C	999
YHR041C	YDL108W	984
YHR041C	YDL140C	999
YHR041C	YDR138W	994
YHR041C	YDR145W	921
YHR041C	YDR167W	978
YHR041C	YDR176W	978
YHR041C	YDR308C	999
YHR041C	YDR443C	997
YHR041C	YDR448W	999
YHR041C	YEL009C	997
YHR041C	YER022W	999
YHR041C	YER148W	999
YHR041C	YGL025C	999
YHR041C	YGL112C	997
YHR041C	YGL127C	999
YHR041C	YGL151W	998
YHR041C	YGL244W	993
YHR041C	YGR005C	978
YHR041C	YGR104C	999

YHR041C	YGR252W	980
YHR041C	YHR058C	999
YHR041C	YIL021W	978
YHR041C	YJL140W	912
YHR041C	YKR062W	978
YHR041C	YLR071C	999
YHR041C	YLR182W	906
YHR041C	YLR418C	970
YHR041C	YML007W	992
YHR041C	YML098W	910
YHR041C	YMR112C	998
YHR041C	YMR227C	911
YHR041C	YMR236W	978
YHR041C	YNL236W	999
YHR041C	YNR010W	999
YHR041C	YOL051W	999
YHR041C	YOL135C	999
YHR041C	YOL145C	970
YHR041C	YOL148C	974
YHR041C	YOR123C	964
YHR041C	YOR174W	998
YHR041C	YPL042C	991
YHR041C	YPL129W	965
YHR041C	YPR056W	984
YHR041C	YPR070W	999
YHR041C	YPR168W	998
YHR042W	YHR007C	968
YHR042W	YJR137C	916
YHR042W	YPR167C	909
YHR046C	YJL153C	957
YHR050W	YBR290W	947
YHR050W	YGL167C	939
YHR050W	YOL122C	978
YHR051W	Q0045	998
YHR051W	Q0250	999
YHR051W	Q0275	994
YHR051W	YDL067C	999
YHR051W	YGL187C	998
YHR051W	YGL191W	999
YHR051W	YGR112W	900
YHR051W	YIL111W	978
YHR051W	YLR038C	985
YHR051W	YLR395C	982
YHR051W	YMR256C	992

YHR051W	YNL052W	999
YHR051W	YPL172C	905
YHR052W	YAL035W	946
YHR052W	YBR142W	996
YHR052W	YDL031W	996
YHR052W	YDR060W	999
YHR052W	YDR087C	999
YHR052W	YDR101C	996
YHR052W	YDR496C	996
YHR052W	YER006W	999
YHR052W	YFL002C	997
YHR052W	YFL009W	978
YHR052W	YFR001W	993
YHR052W	YFR050C	984
YHR052W	YGL011C	984
YHR052W	YGL030W	977
YHR052W	YGL103W	943
YHR052W	YGL111W	994
YHR052W	YGL135W	940
YHR052W	YGR103W	999
YHR052W	YHR010W	992
YHR052W	YHR066W	987
YHR052W	YHR088W	998
YHR052W	YHR197W	998
YHR052W	YJL122W	988
YHR052W	YKL009W	999
YHR052W	YKL021C	948
YHR052W	YKL172W	999
YHR052W	YKR081C	999
YHR052W	YLL008W	997
YHR052W	YLL034C	987
YHR052W	YLL045C	944
YHR052W	YLR002C	998
YHR052W	YLR009W	968
YHR052W	YLR074C	925
YHR052W	YLR106C	992
YHR052W	YLR276C	988
YHR052W	YLR449W	982
YHR052W	YMR049C	999
YHR052W	YMR229C	999
YHR052W	YMR242C	942
YHR052W	YMR290C	997
YHR052W	YNL002C	999
YHR052W	YNL061W	999

YHR052W	YNL110C	999
YHR052W	YNL182C	996
YHR052W	YNL293W	904
YHR052W	YOL041C	994
YHR052W	YOL077C	997
YHR052W	YOL112W	905
YHR052W	YOL127W	945
YHR052W	YOR063W	979
YHR052W	YOR206W	999
YHR052W	YOR272W	999
YHR052W	YOR310C	969
YHR052W	YPL012W	951
YHR052W	YPL043W	999
YHR052W	YPL093W	998
YHR052W	YPL131W	990
YHR052W	YPL211W	998
YHR052W	YPR016C	998
YHR052W	YPR103W	998
YHR053C	YGL073W	923
YHR053C	YGL166W	975
YHR053C	YJR104C	993
YHR053C	YOR031W	993
YHR056C	YBL006C	956
YHR056C	YCR020W-B	999
YHR056C	YCR052W	996
YHR056C	YDR303C	999
YHR056C	YFR037C	999
YHR056C	YGR056W	999
YHR056C	YIL126W	998
YHR056C	YKR008W	996
YHR056C	YLR033W	999
YHR056C	YLR321C	996
YHR056C	YLR357W	999
YHR056C	YML127W	996
YHR056C	YMR033W	997
YHR056C	YMR091C	999
YHR056C	YPR034W	996
YHR057C	YDR155C	917
YHR057C	YDR304C	912
YHR057C	YML078W	918
YHR058C	YBL093C	999
YHR058C	YBR193C	999
YHR058C	YBR253W	999
YHR058C	YCR081W	974

YHR058C	YDL005C	999
YHR058C	YDL140C	999
YHR058C	YDR308C	999
YHR058C	YDR404C	901
YHR058C	YDR443C	997
YHR058C	YDR448W	986
YHR058C	YER022W	999
YHR058C	YER148W	910
YHR058C	YGL025C	999
YHR058C	YGL070C	917
YHR058C	YGL127C	999
YHR058C	YGL151W	997
YHR058C	YGR104C	999
YHR058C	YHR041C	999
YHR058C	YIL021W	984
YHR058C	YLR071C	999
YHR058C	YML007W	992
YHR058C	YMR112C	998
YHR058C	YNL236W	998
YHR058C	YNR010W	999
YHR058C	YOL051W	999
YHR058C	YOL135C	999
YHR058C	YOR151C	915
YHR058C	YOR174W	999
YHR058C	YOR210W	901
YHR058C	YOR224C	907
YHR058C	YPL042C	995
YHR058C	YPL129W	965
YHR058C	YPR070W	999
YHR058C	YPR168W	999
YHR058C	YPR187W	901
YHR059W	YBR251W	947
YHR059W	YGL043W	914
YHR059W	YGL070C	915
YHR059W	YGL127C	923
YHR060W	YAL042W	920
YHR060W	YKL119C	997
YHR060W	YLR447C	979
YHR060W	YOR270C	992
YHR061C	YAL041W	979
YHR061C	YCL032W	905
YHR061C	YCR086W	905
YHR061C	YDR309C	999
YHR061C	YGR221C	923

YHR061C	YHL007C	965
YHR061C	YHR107C	991
YHR061C	YHR149C	923
YHR061C	YIL135C	928
YHR061C	YKL082C	934
YHR061C	YLR229C	999
YHR061C	YML109W	970
YHR061C	YMR055C	989
YHR061C	YMR238W	916
YHR061C	YNL074C	923
YHR061C	YNL271C	949
YHR061C	YNL298W	997
YHR061C	YOR188W	912
YHR061C	YPL161C	905
YHR061C	YPL256C	933
YHR062C	YAL033W	999
YHR062C	YBL018C	999
YHR062C	YBR167C	999
YHR062C	YBR257W	999
YHR062C	YDR478W	999
YHR062C	YGR030C	999
YHR062C	YIR015W	989
YHR062C	YLR145W	956
YHR062C	YNL221C	999
YHR062C	YNL282W	999
YHR063C	YBR176W	958
YHR063C	YIL145C	955
YHR064C	YAL003W	998
YHR064C	YAR014C	950
YHR064C	YAR018C	950
YHR064C	YBR101C	903
YHR064C	YBR158W	985
YHR064C	YDL229W	966
YHR064C	YDR457W	979
YHR064C	YGR285C	999
YHR064C	YLR249W	987
YHR064C	YNL209W	983
YHR066W	YBR142W	968
YHR066W	YDL031W	974
YHR066W	YDR060W	986
YHR066W	YDR087C	971
YHR066W	YDR312W	944
YHR066W	YDR496C	993
YHR066W	YGL111W	996

YHR066W	YGR103W	997
YHR066W	YHR052W	987
YHR066W	YHR088W	996
YHR066W	YKL014C	986
YHR066W	YKL082C	933
YHR066W	YKL172W	996
YHR066W	YKR081C	998
YHR066W	YLL008W	996
YHR066W	YLR276C	993
YHR066W	YMR049C	993
YHR066W	YMR290C	996
YHR066W	YNL002C	996
YHR066W	YNL061W	996
YHR066W	YOR206W	980
YHR066W	YOR212W	976
YHR066W	YPL043W	996
YHR066W	YPL093W	997
YHR066W	YPL211W	994
YHR066W	YPR016C	982
YHR068W	YEL034W	998
YHR068W	YJR047C	991
YHR068W	YJR070C	989
YHR068W	YKL135C	998
YHR069C	YCR035C	999
YHR069C	YDL111C	999
YHR069C	YDR280W	999
YHR069C	YGL213C	951
YHR069C	YGR095C	999
YHR069C	YGR158C	999
YHR069C	YGR195W	999
YHR069C	YHR081W	999
YHR069C	YJL050W	943
YHR069C	YLR163C	977
YHR069C	YNL189W	979
YHR069C	YNL232W	999
YHR069C	YNR024W	998
YHR069C	YOL021C	999
YHR069C	YOL142W	999
YHR069C	YOR001W	999
YHR069C	YOR076C	999
YHR071W	YGR233C	992
YHR071W	YPL031C	999
YHR072W	YBR041W	985
YHR072W	YER044C	999

YHR072W	YGL001C	999
YHR072W	YGR060W	999
YHR072W	YGR175C	962
YHR072W	YHR007C	999
YHR072W	YKL140W	946
YHR072W	YLL012W	988
YHR072W	YLR056W	998
YHR072W	YLR100W	999
YHR072W	YML008C	999
YHR072W	YMR015C	999
YHR072W-A	YDL208W	999
YHR072W-A	YGR280C	996
YHR072W-A	YHR089C	999
YHR072W-A	YLR175W	999
YHR072W-A	YNL124W	999
YHR073W	YGL171W	944
YHR073W	YGL173C	925
YHR074W	YJR109C	901
YHR075C	Q0140	962
YHR075C	YDL134C	968
YHR075C	YDL188C	909
YHR077C	YDL220C	950
YHR077C	YDR172W	996
YHR077C	YGR072W	999
YHR077C	YMR080C	999
YHR077C	YNL244C	919
YHR079C	YBL040C	900
YHR079C	YCL043C	915
YHR079C	YDR448W	935
YHR079C	YDR532C	904
YHR079C	YER089C	992
YHR079C	YFL031W	995
YHR079C	YGR252W	955
YHR079C	YGR284C	916
YHR079C	YJL034W	999
YHR079C	YKL073W	977
YHR079C	YKL206C	916
YHR079C	YLR208W	990
YHR079C	YLR361C	925
YHR079C	YMR022W	915
YHR079C	YNR026C	991
YHR079C	YOL031C	959
YHR079C	YOL148C	994
YHR079C	YPL085W	957

YHR079C	YPR181C	993
YHR079C-A	YHL022C	989
YHR079C-A	YPL121C	953
YHR081W	YCR035C	999
YHR081W	YDL111C	987
YHR081W	YDR280W	999
YHR081W	YGR095C	999
YHR081W	YGR158C	986
YHR081W	YGR195W	992
YHR081W	YHR069C	999
YHR081W	YNL232W	999
YHR081W	YOL021C	999
YHR081W	YOL142W	999
YHR081W	YOR001W	999
YHR081W	YOR076C	992
YHR081W	YPL240C	919
YHR082C	YGL097W	916
YHR083W	YAL010C	921
YHR083W	YMR060C	999
YHR083W	YNL026W	999
YHR084W	YBL016W	999
YHR084W	YBR083W	999
YHR084W	YCL055W	982
YHR084W	YCL066W	975
YHR084W	YCR093W	901
YHR084W	YDL159W	999
YHR084W	YDL160C	996
YHR084W	YDR103W	989
YHR084W	YDR480W	999
YHR084W	YER109C	998
YHR084W	YFL026W	999
YHR084W	YGR040W	999
YHR084W	YHL007C	999
YHR084W	YHR005C	980
YHR084W	YIL049W	909
YHR084W	YIR019C	994
YHR084W	YJL157C	954
YHR084W	YJR086W	973
YHR084W	YKR101W	940
YHR084W	YLR362W	999
YHR084W	YMR043W	999
YHR084W	YMR164C	989
YHR084W	YMR308C	972
YHR084W	YNL098C	997

YHR084W	YNL271C	925
YHR084W	YOR212W	983
YHR084W	YOR276W	981
YHR084W	YPL049C	999
YHR085W	YHR197W	999
YHR085W	YKL009W	972
YHR085W	YLR074C	922
YHR085W	YLR106C	998
YHR085W	YNL182C	999
YHR085W	YNR053C	990
YHR085W	YPL093W	998
YHR086W	YBR119W	999
YHR086W	YDL087C	999
YHR086W	YDR235W	999
YHR086W	YDR240C	999
YHR086W	YDR432W	983
YHR086W	YER029C	998
YHR086W	YGL251C	904
YHR086W	YGR013W	999
YHR086W	YGR074W	997
YHR086W	YHR014W	911
YHR086W	YIL061C	999
YHR086W	YJR021C	983
YHR086W	YKL012W	999
YHR086W	YLR147C	999
YHR086W	YLR275W	999
YHR086W	YLR298C	999
YHR086W	YML046W	999
YHR086W	YMR125W	999
YHR086W	YPL178W	999
YHR086W	YPL213W	948
YHR087W	YLR022C	903
YHR087W	YPR131C	925
YHR088W	YDL031W	981
YHR088W	YDR060W	978
YHR088W	YDR087C	995
YHR088W	YDR496C	995
YHR088W	YER006W	993
YHR088W	YFL002C	993
YHR088W	YGL076C	937
YHR088W	YGL111W	999
YHR088W	YGL241W	921
YHR088W	YGR103W	997
YHR088W	YHR052W	998

YHR088W	YHR066W	996
YHR088W	YHR197W	985
YHR088W	YJR132W	923
YHR088W	YKL009W	983
YHR088W	YKL172W	982
YHR088W	YLL008W	989
YHR088W	YLL034C	979
YHR088W	YLL045C	941
YHR088W	YLR002C	983
YHR088W	YLR449W	980
YHR088W	YMR049C	976
YHR088W	YMR290C	999
YHR088W	YNL002C	989
YHR088W	YNL061W	999
YHR088W	YNL110C	999
YHR088W	YOL077C	997
YHR088W	YOL127W	933
YHR088W	YOR063W	933
YHR088W	YOR206W	995
YHR088W	YOR272W	997
YHR088W	YPL043W	985
YHR088W	YPL093W	989
YHR088W	YPL125W	984
YHR088W	YPL131W	960
YHR088W	YPL198W	927
YHR088W	YPL211W	981
YHR088W	YPR016C	999
YHR089C	YBR034C	920
YHR089C	YDL014W	950
YHR089C	YDL208W	999
YHR089C	YDL213C	996
YHR089C	YDR365C	973
YHR089C	YDR496C	947
YHR089C	YER102W	970
YHR089C	YGL078C	986
YHR089C	YGR283C	984
YHR089C	YHR072W-A	999
YHR089C	YKL014C	954
YHR089C	YLR029C	979
YHR089C	YLR175W	999
YHR089C	YMR310C	983
YHR089C	YNL124W	987
YHR089C	YNL308C	990
YHR089C	YOR204W	941

YHR089C	YPL012W	969
YHR089C	YPL043W	973
YHR090C	YBR245C	931
YHR090C	YDR359C	999
YHR090C	YEL018W	999
YHR090C	YFL024C	999
YHR090C	YFL039C	994
YHR090C	YGR002C	999
YHR090C	YHR099W	998
YHR090C	YJL081C	999
YHR090C	YJR082C	994
YHR090C	YNL107W	999
YHR090C	YNL136W	999
YHR090C	YOR244W	999
YHR090C	YPR023C	997
YHR091C	YDR037W	904
YHR091C	YDR341C	990
YHR091C	YGR264C	900
YHR091C	YHR018C	902
YHR092C	YJR090C	917
YHR094C	YDL194W	995
YHR094C	YDR277C	984
YHR094C	YJL129C	967
YHR094C	YJR090C	993
YHR094C	YKL038W	941
YHR094C	YKR050W	967
YHR094C	YML048W	951
YHR098C	YDL195W	997
YHR098C	YDR517W	984
YHR098C	YGL008C	984
YHR098C	YIL109C	998
YHR098C	YLR208W	998
YHR098C	YNL049C	919
YHR098C	YPL218W	955
YHR098C	YPR181C	999
YHR099W	YBR081C	999
YHR099W	YBR111W-A	998
YHR099W	YBR198C	999
YHR099W	YCL010C	997
YHR099W	YDR145W	999
YHR099W	YDR167W	999
YHR099W	YDR176W	999
YHR099W	YDR190C	971
YHR099W	YDR240C	940

YHR099W	YDR359C	999
YHR099W	YDR392W	999
YHR099W	YDR448W	999
YHR099W	YEL009C	975
YHR099W	YEL018W	998
YHR099W	YER148W	980
YHR099W	YER164W	980
YHR099W	YFL024C	999
YHR099W	YGL066W	999
YHR099W	YGL112C	999
YHR099W	YGR252W	999
YHR099W	YHR090C	998
YHR099W	YJL081C	999
YHR099W	YLR055C	999
YHR099W	YMR223W	999
YHR099W	YMR227C	923
YHR099W	YMR236W	999
YHR099W	YNL107W	999
YHR099W	YNL136W	947
YHR099W	YOL148C	999
YHR099W	YOR244W	999
YHR099W	YPL047W	999
YHR099W	YPL082C	972
YHR099W	YPL235W	963
YHR099W	YPL248C	986
YHR099W	YPL254W	999
YHR099W	YPR023C	996
YHR101C	YAL058W	923
YHR101C	YBR229C	957
YHR101C	YMR200W	969
YHR102W	YFL034C-B	956
YHR102W	YIL129C	940
YHR102W	YKL189W	995
YHR102W	YNL161W	976
YHR102W	YOR257W	988
YHR102W	YOR353C	999
YHR104W	YBR020W	909
YHR104W	YDL246C	900
YHR104W	YDR368W	973
YHR104W	YJR159W	933
YHR104W	YLR070C	973
YHR104W	YML004C	991
YHR104W	YOR120W	976
YHR105W	YBR205W	941

YHR105W	YGL161C	941
YHR105W	YNL263C	979
YHR105W	YOR034C	957
YHR105W	YPL246C	955
YHR106W	YCR083W	997
YHR106W	YDR353W	999
YHR106W	YGR209C	988
YHR106W	YLR043C	976
YHR106W	YPL091W	990
YHR107C	YCL014W	937
YHR107C	YCL024W	986
YHR107C	YCR002C	999
YHR107C	YDL225W	999
YHR107C	YDR013W	962
YHR107C	YDR085C	999
YHR107C	YDR309C	992
YHR107C	YDR379W	945
YHR107C	YDR507C	999
YHR107C	YHR030C	918
YHR107C	YHR061C	991
YHR107C	YJL187C	996
YHR107C	YJR076C	999
YHR107C	YJR092W	935
YHR107C	YKL048C	973
YHR107C	YKL101W	998
YHR107C	YLL021W	988
YHR107C	YLR229C	987
YHR107C	YLR314C	999
YHR107C	YMR001C	984
YHR107C	YNL166C	988
YHR107C	YNL233W	991
YHR107C	YNL298W	998
YHR107C	YPL115C	949
YHR107C	YPL161C	965
YHR107C	YPL242C	961
YHR108W	YDL137W	989
YHR108W	YDL192W	986
YHR108W	YDR153C	999
YHR108W	YDR358W	999
YHR108W	YGL206C	999
YHR108W	YIL094C	956
YHR108W	YJR125C	971
YHR108W	YLL039C	939
YHR108W	YOR109W	974

YHR109W	YJR139C	998
YHR110W	YDL018C	995
YHR111W	YIL008W	999
YHR111W	YNL298W	940
YHR112C	YDR346C	980
YHR113W	YKL103C	974
YHR114W	YBL007C	991
YHR114W	YDL146W	929
YHR114W	YFL012W	998
YHR114W	YFR024C-A	999
YHR114W	YJL020C	936
YHR114W	YKL129C	936
YHR114W	YLR337C	984
YHR114W	YMR109W	999
YHR114W	YOR181W	999
YHR114W	YPR154W	999
YHR115C	YLR215C	984
YHR115C	YNL116W	945
YHR117W	YBR112C	999
YHR117W	YDR219C	986
YHR117W	YLR072W	998
YHR117W	YNL121C	970
YHR118C	YBL023C	977
YHR118C	YBR060C	999
YHR118C	YBR160W	998
YHR118C	YEL056W	999
YHR118C	YJL194W	999
YHR118C	YJR046W	997
YHR118C	YLL004W	999
YHR118C	YLR079W	952
YHR118C	YLR274W	956
YHR118C	YML065W	999
YHR118C	YMR036C	998
YHR118C	YNL261W	999
YHR118C	YPL001W	999
YHR118C	YPR018W	998
YHR118C	YPR120C	998
YHR118C	YPR162C	999
YHR119W	YAR003W	999
YHR119W	YBR010W	971
YHR119W	YBR175W	999
YHR119W	YBR258C	999
YHR119W	YDL042C	996
YHR119W	YDR440W	958

YHR119W	YDR469W	999
YHR119W	YER164W	924
YHR119W	YJR119C	902
YHR119W	YKL018W	999
YHR119W	YLR015W	999
YHR119W	YLR288C	995
YHR119W	YLR442C	981
YHR119W	YPL138C	999
YHR120W	YDR263C	909
YHR120W	YDR296W	906
YHR120W	YKL011C	901
YHR121W	YDL053C	998
YHR121W	YEL015W	931
YHR121W	YGR178C	999
YHR121W	YLL013C	938
YHR122W	YDR267C	986
YHR122W	YIL128W	999
YHR122W	YNL240C	971
YHR123W	YNL130C	973
YHR124W	YDR310C	945
YHR124W	YER179W	937
YHR124W	YGR019W	998
YHR124W	YIL112W	904
YHR124W	YJL106W	999
YHR127W	YDR381W	976
YHR128W	YDR400W	928
YHR128W	YEL021W	996
YHR128W	YHR144C	909
YHR128W	YLR245C	930
YHR128W	YNR012W	992
YHR128W	YPR062W	996
YHR129C	YDR106W	982
YHR129C	YEL061C	901
YHR129C	YER016W	984
YHR129C	YGR229C	923
YHR129C	YLR210W	910
YHR129C	YMR294W	999
YHR129C	YNL271C	988
YHR129C	YPL174C	999
YHR129C	YPL269W	998
YHR131C	YER125W	998
YHR131C	YOR061W	980
YHR132C	YGR159C	998
YHR133C	YLR450W	927

YHR134W	YDR217C	969
YHR134W	YDR510W	984
YHR134W	YER016W	977
YHR134W	YGR213C	904
YHR134W	YHR156C	913
YHR134W	YML032C	970
YHR134W	YMR190C	983
YHR134W	YNL201C	928
YHR134W	YNL273W	919
YHR134W	YPL269W	975
YHR135C	YBL032W	996
YHR135C	YDL101C	974
YHR135C	YDL138W	981
YHR135C	YDR137W	909
YHR135C	YDR477W	970
YHR135C	YER123W	985
YHR135C	YLR133W	973
YHR135C	YNL154C	999
YHR135C	YPL240C	902
YHR136C	YDR226W	920
YHR137W	YDR380W	969
YHR137W	YDR421W	928
YHR138C	YNL015W	942
YHR141C	YBL027W	999
YHR141C	YBL072C	924
YHR141C	YBL087C	996
YHR141C	YBL092W	999
YHR141C	YBR031W	996
YHR141C	YBR048W	910
YHR141C	YBR084C-A	924
YHR141C	YBR118W	910
YHR141C	YBR181C	998
YHR141C	YBR189W	933
YHR141C	YBR191W	999
YHR141C	YCR031C	996
YHR141C	YDL061C	913
YHR141C	YDL075W	999
YHR141C	YDL081C	951
YHR141C	YDL082W	998
YHR141C	YDL083C	965
YHR141C	YDL130W	917
YHR141C	YDL136W	999
YHR141C	YDL191W	926
YHR141C	YDR012W	993

YHR141C	YDR025W	910
YHR141C	YDR064W	996
YHR141C	YDR382W	996
YHR141C	YDR385W	913
YHR141C	YDR418W	999
YHR141C	YDR447C	998
YHR141C	YDR450W	926
YHR141C	YDR471W	996
YHR141C	YDR500C	917
YHR141C	YEL054C	930
YHR141C	YER056C-A	916
YHR141C	YER074W	995
YHR141C	YER102W	924
YHR141C	YER117W	920
YHR141C	YER131W	935
YHR141C	YFL034C-A	926
YHR141C	YFR031C-A	999
YHR141C	YFR032C-A	941
YHR141C	YGL014W	978
YHR141C	YGL030W	997
YHR141C	YGL031C	999
YHR141C	YGL076C	994
YHR141C	YGL103W	999
YHR141C	YGL135W	999
YHR141C	YGL147C	999
YHR141C	YGL189C	919
YHR141C	YGR027C	935
YHR141C	YGR034W	998
YHR141C	YGR085C	997
YHR141C	YGR118W	938
YHR141C	YGR148C	926
YHR141C	YGR214W	927
YHR141C	YHL001W	998
YHR141C	YHL015W	939
YHR141C	YHL033C	999
YHR141C	YHR010W	928
YHR141C	YHR021C	949
YHR141C	YHR029C	972
YHR141C	YHR203C	946
YHR141C	YIL018W	920
YHR141C	YIL052C	916
YHR141C	YIL069C	913
YHR141C	YIL133C	999
YHR141C	YIL148W	908

YHR141C	YJL136C	948
YHR141C	YJL177W	995
YHR141C	YJL189W	918
YHR141C	YJL190C	999
YHR141C	YJL191W	928
YHR141C	YJR094W-A	992
YHR141C	YJR123W	996
YHR141C	YJR145C	946
YHR141C	YKL006W	942
YHR141C	YKL156W	943
YHR141C	YKL180W	999
YHR141C	YKR057W	937
YHR141C	YKR094C	908
YHR141C	YLL045C	967
YHR141C	YLR029C	992
YHR141C	YLR048W	917
YHR141C	YLR061W	931
YHR141C	YLR075W	999
YHR141C	YLR167W	902
YHR141C	YLR185W	991
YHR141C	YLR264W	932
YHR141C	YLR287C-A	997
YHR141C	YLR325C	946
YHR141C	YLR333C	929
YHR141C	YLR340W	939
YHR141C	YLR344W	993
YHR141C	YLR367W	928
YHR141C	YLR388W	914
YHR141C	YLR406C	998
YHR141C	YLR441C	997
YHR141C	YLR448W	997
YHR141C	YML024W	935
YHR141C	YML026C	926
YHR141C	YML063W	997
YHR141C	YML073C	997
YHR141C	YMR121C	933
YHR141C	YMR142C	937
YHR141C	YMR194W	996
YHR141C	YMR230W	942
YHR141C	YMR242C	998
YHR141C	YMR260C	913
YHR141C	YNL067W	940
YHR141C	YNL069C	997
YHR141C	YNL096C	998

YHR141C	YNL162W	910
YHR141C	YNL178W	946
YHR141C	YNL301C	999
YHR141C	YNL302C	998
YHR141C	YOL039W	913
YHR141C	YOL040C	998
YHR141C	YOL120C	923
YHR141C	YOL121C	949
YHR141C	YOL127W	999
YHR141C	YOR063W	999
YHR141C	YOR096W	936
YHR141C	YOR133W	913
YHR141C	YOR167C	943
YHR141C	YOR182C	915
YHR141C	YOR234C	938
YHR141C	YOR293W	946
YHR141C	YOR312C	929
YHR141C	YOR369C	954
YHR141C	YPL079W	950
YHR141C	YPL081W	923
YHR141C	YPL090C	902
YHR141C	YPL131W	992
YHR141C	YPL143W	928
YHR141C	YPL198W	935
YHR141C	YPL220W	932
YHR141C	YPL249C-A	931
YHR141C	YPR043W	930
YHR141C	YPR080W	910
YHR141C	YPR102C	995
YHR141C	YPR132W	929
YHR141C	YPR189W	909
YHR142W	YGR229C	953
YHR142W	YHR176W	917
YHR142W	YJL099W	932
YHR143W	YER124C	999
YHR143W	YGL028C	999
YHR143W	YLR286C	935
YHR143W	YNL066W	981
YHR143W	YNL300W	912
YHR143W-A	YAL001C	997
YHR143W-A	YBR154C	999
YHR143W-A	YDL140C	999
YHR143W-A	YDL150W	960
YHR143W-A	YDR045C	960

YHR143W-A	YDR156W	960
YHR143W-A	YDR404C	999
YHR143W-A	YEL026W	921
YHR143W-A	YER022W	981
YHR143W-A	YGL043W	989
YHR143W-A	YGL070C	999
YHR143W-A	YGR005C	920
YHR143W-A	YGR047C	984
YHR143W-A	YGR104C	983
YHR143W-A	YIL021W	999
YHR143W-A	YJL011C	960
YHR143W-A	YJL140W	998
YHR143W-A	YJL148W	961
YHR143W-A	YJR063W	999
YHR143W-A	YKL028W	918
YHR143W-A	YKL144C	960
YHR143W-A	YKR025W	960
YHR143W-A	YKR062W	930
YHR143W-A	YML010W	961
YHR143W-A	YMR277W	950
YHR143W-A	YNL039W	960
YHR143W-A	YNL113W	981
YHR143W-A	YNL151C	960
YHR143W-A	YNL248C	954
YHR143W-A	YNR003C	960
YHR143W-A	YOL005C	998
YHR143W-A	YOL051W	959
YHR143W-A	YOR116C	961
YHR143W-A	YOR151C	999
YHR143W-A	YOR207C	992
YHR143W-A	YOR210W	999
YHR143W-A	YOR224C	999
YHR143W-A	YOR340C	999
YHR143W-A	YOR341W	960
YHR143W-A	YPL129W	965
YHR143W-A	YPR010C	999
YHR143W-A	YPR110C	958
YHR143W-A	YPR168W	931
YHR143W-A	YPR187W	999
YHR143W-A	YPR190C	960
YHR144C	YBR252W	971
YHR144C	YHR128W	909
YHR144C	YOR074C	981
YHR146W	YMR196W	929

YHR146W	YPL154C	994
YHR147C	YBL038W	972
YHR147C	YBR122C	968
YHR147C	YCR046C	991
YHR147C	YDR116C	995
YHR147C	YDR237W	998
YHR147C	YDR296W	970
YHR147C	YDR322W	970
YHR147C	YEL050C	954
YHR147C	YGL068W	999
YHR147C	YGR085C	912
YHR147C	YGR220C	990
YHR147C	YJL063C	981
YHR147C	YKL170W	963
YHR147C	YLR069C	939
YHR147C	YLR439W	970
YHR147C	YML025C	902
YHR147C	YMR193W	968
YHR147C	YNL005C	988
YHR147C	YNL185C	974
YHR147C	YNL252C	972
YHR147C	YNL284C	994
YHR147C	YNR036C	903
YHR147C	YOR150W	952
YHR147C	YPR102C	914
YHR148W	YBL004W	997
YHR148W	YBR247C	999
YHR148W	YCL059C	999
YHR148W	YCR057C	999
YHR148W	YDL014W	998
YHR148W	YDL060W	917
YHR148W	YDL148C	998
YHR148W	YDL153C	910
YHR148W	YDR064W	933
YHR148W	YDR299W	998
YHR148W	YDR324C	998
YHR148W	YDR339C	905
YHR148W	YDR398W	911
YHR148W	YDR449C	999
YHR148W	YEL026W	908
YHR148W	YER082C	997
YHR148W	YGL171W	997
YHR148W	YGR090W	999
YHR148W	YGR128C	997

YHR148W	YGR145W	997
YHR148W	YHR169W	939
YHR148W	YHR196W	999
YHR148W	YJL069C	996
YHR148W	YJL109C	999
YHR148W	YJR002W	999
YHR148W	YJR145C	932
YHR148W	YKR060W	997
YHR148W	YLL011W	998
YHR148W	YLR003C	976
YHR148W	YLR129W	998
YHR148W	YLR175W	901
YHR148W	YLR186W	998
YHR148W	YLR197W	997
YHR148W	YLR222C	998
YHR148W	YLR409C	997
YHR148W	YML093W	916
YHR148W	YMR093W	998
YHR148W	YMR128W	997
YHR148W	YMR229C	911
YHR148W	YMR290C	914
YHR148W	YNL075W	999
YHR148W	YNL132W	995
YHR148W	YOL010W	926
YHR148W	YOR004W	924
YHR148W	YOR078W	992
YHR148W	YOR096W	938
YHR148W	YOR145C	998
YHR148W	YOR310C	974
YHR148W	YPL012W	912
YHR148W	YPL126W	999
YHR148W	YPL217C	998
YHR148W	YPR112C	921
YHR148W	YPR137W	997
YHR148W	YPR144C	998
YHR149C	YDR309C	921
YHR149C	YHR061C	923
YHR149C	YML109W	949
YHR149C	YMR307W	973
YHR149C	YNL238W	973
YHR150W	YBR168W	936
YHR150W	YKR001C	911
YHR150W	YPL112C	931
YHR152W	YAR019C	999

YHR152W	YCR086W	957
YHR152W	YDR110W	958
YHR152W	YFR028C	944
YHR152W	YGL003C	914
YHR152W	YGR092W	999
YHR152W	YGR098C	917
YHR152W	YHL007C	956
YHR152W	YHR014W	927
YHR152W	YJL076W	927
YHR152W	YMR001C	987
YHR152W	YMR308C	933
YHR152W	YOR195W	938
YHR153C	YIL073C	985
YHR154W	YBR136W	952
YHR154W	YDR217C	985
YHR154W	YER173W	970
YHR154W	YJL092W	947
YHR154W	YLR135W	997
YHR154W	YLR320W	993
YHR154W	YLR442C	975
YHR154W	YMR190C	999
YHR156C	YDL013W	904
YHR156C	YER172C	933
YHR156C	YHR134W	913
YHR156C	YHR165C	999
YHR156C	YIL026C	981
YHR156C	YOR156C	904
YHR156C	YPL240C	919
YHR157W	YER044C-A	940
YHR157W	YGL213C	935
YHR157W	YHL022C	998
YHR157W	YHR014W	928
YHR157W	YIL072W	992
YHR157W	YLR329W	999
YHR157W	YMR133W	947
YHR158C	YAL016W	958
YHR158C	YAL024C	999
YHR158C	YAR014C	935
YHR158C	YGR238C	999
YHR158C	YLL021W	929
YHR158C	YLR452C	952
YHR158C	YPR119W	984
YHR160C	YDR142C	999
YHR160C	YGL153W	906

YHR160C	YGR239C	995
YHR160C	YIL160C	994
YHR160C	YLR191W	980
YHR161C	YGL206C	956
YHR161C	YIR006C	968
YHR163W	YDR248C	940
YHR163W	YGR256W	991
YHR163W	YHR183W	997
YHR163W	YKL205W	946
YHR163W	YNL241C	994
YHR164C	YAR007C	999
YHR164C	YBR088C	927
YHR164C	YBR136W	927
YHR164C	YDL102W	998
YHR164C	YDL164C	943
YHR164C	YDR110W	987
YHR164C	YGL207W	963
YHR164C	YHR031C	980
YHR164C	YIR008C	909
YHR164C	YJR006W	927
YHR164C	YJR043C	984
YHR164C	YKL045W	930
YHR164C	YKL113C	999
YHR164C	YLR442C	905
YHR164C	YML061C	944
YHR164C	YMR190C	999
YHR164C	YNL102W	945
YHR164C	YNL312W	998
YHR164C	YOR033C	987
YHR164C	YOR144C	969
YHR164C	YPR135W	988
YHR165C	YAL032C	992
YHR165C	YBL026W	999
YHR165C	YBL074C	999
YHR165C	YBR055C	999
YHR165C	YBR065C	995
YHR165C	YBR102C	983
YHR165C	YCR063W	999
YHR165C	YCR077C	976
YHR165C	YDL030W	998
YHR165C	YDL043C	981
YHR165C	YDL087C	993
YHR165C	YDL098C	978
YHR165C	YDL209C	999

YHR165C	YDR088C	998
YHR165C	YDR213W	976
YHR165C	YDR240C	991
YHR165C	YDR243C	999
YHR165C	YDR364C	999
YHR165C	YDR378C	999
YHR165C	YDR416W	999
YHR165C	YDR464W	903
YHR165C	YDR473C	999
YHR165C	YEL026W	921
YHR165C	YER013W	948
YHR165C	YER029C	999
YHR165C	YER112W	990
YHR165C	YER146W	999
YHR165C	YER172C	999
YHR165C	YFL017W-A	986
YHR165C	YGL120C	998
YHR165C	YGL128C	999
YHR165C	YGR006W	988
YHR165C	YGR013W	989
YHR165C	YGR021W	976
YHR165C	YGR074W	999
YHR165C	YGR075C	982
YHR165C	YGR091W	999
YHR165C	YGR271W	913
YHR165C	YGR278W	998
YHR165C	YHR156C	999
YHR165C	YIL061C	999
YHR165C	YIR004W	976
YHR165C	YJL124C	981
YHR165C	YJL203W	993
YHR165C	YJR022W	991
YHR165C	YKL012W	949
YHR165C	YKL152C	976
YHR165C	YKL173W	999
YHR165C	YKR022C	997
YHR165C	YKR086W	998
YHR165C	YLL036C	999
YHR165C	YLR117C	999
YHR165C	YLR147C	999
YHR165C	YLR275W	999
YHR165C	YLR424W	999
YHR165C	YLR438C-A	935
YHR165C	YML046W	998

YHR165C	YML049C	999
YHR165C	YMR125W	998
YHR165C	YMR213W	999
YHR165C	YMR240C	999
YHR165C	YMR288W	998
YHR165C	YNL147W	992
YHR165C	YOR151C	903
YHR165C	YOR159C	999
YHR165C	YOR308C	999
YHR165C	YOR319W	921
YHR165C	YPL151C	992
YHR165C	YPL178W	915
YHR165C	YPL213W	999
YHR165C	YPR082C	998
YHR165C	YPR101W	991
YHR165C	YPR178W	999
YHR165C	YPR182W	999
YHR166C	YBL084C	999
YHR166C	YBR135W	929
YHR166C	YBR160W	999
YHR166C	YDL008W	999
YHR166C	YDR113C	997
YHR166C	YDR118W	999
YHR166C	YDR260C	999
YHR166C	YER161C	976
YHR166C	YFR036W	999
YHR166C	YGL003C	998
YHR166C	YGL116W	999
YHR166C	YGL240W	999
YHR166C	YGR179C	907
YHR166C	YIR025W	999
YHR166C	YKL022C	999
YHR166C	YLL039C	906
YHR166C	YLR102C	999
YHR166C	YLR127C	999
YHR166C	YMR001C	989
YHR166C	YNL172W	999
YHR166C	YOR249C	999
YHR166C	YPR119W	999
YHR167W	YCL011C	999
YHR167W	YDL084W	999
YHR167W	YDR138W	999
YHR167W	YDR381W	998
YHR167W	YML062C	999

YHR167W	YNL004W	999
YHR167W	YNL139C	999
YHR167W	YNL253W	999
YHR168W	YGL136C	941
YHR169W	YBR247C	978
YHR169W	YCL059C	931
YHR169W	YCR057C	979
YHR169W	YDL060W	912
YHR169W	YDL148C	910
YHR169W	YDL153C	916
YHR169W	YDR299W	946
YHR169W	YDR324C	912
YHR169W	YDR398W	922
YHR169W	YDR449C	915
YHR169W	YGL120C	931
YHR169W	YGR128C	925
YHR169W	YGR145W	915
YHR169W	YHR148W	939
YHR169W	YHR196W	911
YHR169W	YJL069C	921
YHR169W	YJL109C	917
YHR169W	YJR002W	947
YHR169W	YKR060W	920
YHR169W	YLL011W	945
YHR169W	YLR003C	914
YHR169W	YLR129W	938
YHR169W	YLR175W	917
YHR169W	YLR186W	922
YHR169W	YLR197W	911
YHR169W	YLR222C	964
YHR169W	YLR409C	947
YHR169W	YML093W	916
YHR169W	YMR093W	953
YHR169W	YMR128W	912
YHR169W	YMR229C	921
YHR169W	YNL075W	955
YHR169W	YNL132W	916
YHR169W	YNR054C	965
YHR169W	YOL010W	929
YHR169W	YOR004W	965
YHR169W	YOR078W	915
YHR169W	YOR145C	955
YHR169W	YPL012W	918
YHR169W	YPL126W	918

YHR169W	YPL217C	930
YHR169W	YPR112C	924
YHR169W	YPR137W	914
YHR169W	YPR144C	924
YHR170W	YBR267W	999
YHR170W	YDR101C	999
YHR170W	YDR496C	904
YHR170W	YER126C	901
YHR170W	YGL099W	999
YHR170W	YGR218W	999
YHR170W	YGR245C	956
YHR170W	YHL033C	979
YHR170W	YHR197W	938
YHR170W	YJR042W	982
YHR170W	YKL009W	954
YHR170W	YKR081C	998
YHR170W	YLR009W	915
YHR170W	YLR075W	999
YHR170W	YLR196W	978
YHR170W	YLR222C	909
YHR170W	YMR080C	999
YHR170W	YMR290C	966
YHR170W	YOL077C	958
YHR170W	YPL093W	926
YHR170W	YPL169C	919
YHR171W	YBL078C	999
YHR171W	YBR217W	999
YHR171W	YLL042C	999
YHR171W	YMR159C	930
YHR171W	YNL223W	955
YHR171W	YNR007C	999
YHR171W	YPL149W	992
YHR172W	YAL047C	999
YHR172W	YDR356W	999
YHR172W	YLR212C	999
YHR172W	YNL126W	999
YHR174W	YAL038W	982
YHR174W	YBR218C	907
YHR174W	YCR012W	999
YHR174W	YDR050C	942
YHR174W	YGL062W	907
YHR174W	YGR254W	999
YHR174W	YKL060C	997
YHR174W	YKL152C	942

YHR174W	YKR097W	960
YHR174W	YLR044C	998
YHR174W	YMR323W	901
YHR174W	YNR001C	917
YHR174W	YOR347C	930
YHR174W	YOR393W	902
YHR174W	YPL281C	902
YHR175W	YLR246W	923
YHR175W	YPR124W	957
YHR176W	YHR142W	917
YHR176W	YHR204W	923
YHR176W	YPL240C	919
YHR178W	YBL005W	916
YHR178W	YGL013C	911
YHR179W	YFL039C	968
YHR179W	YPL171C	999
YHR183W	YCR073W-A	946
YHR183W	YDR248C	966
YHR183W	YGR248W	987
YHR183W	YGR256W	927
YHR183W	YHR163W	997
YHR183W	YJL121C	965
YHR183W	YNL241C	989
YHR183W	YNR034W	937
YHR183W	YOR095C	931
YHR184W	YDL239C	996
YHR185C	YHR014W	912
YHR185C	YIL007C	993
YHR185C	YPL204W	977
YHR186C	YER093C	908
YHR186C	YJR066W	999
YHR186C	YKL203C	995
YHR186C	YMR028W	900
YHR186C	YMR104C	973
YHR186C	YMR273C	967
YHR186C	YNL006W	998
YHR186C	YPL180W	990
YHR187W	YBL021C	976
YHR187W	YBR009C	904
YHR187W	YGR200C	999
YHR187W	YKL110C	999
YHR187W	YLR384C	999
YHR187W	YMR312W	999
YHR187W	YPL086C	999

YHR187W	YPL101W	999
YHR188C	YDL137W	929
YHR188C	YDL192W	929
YHR188C	YDR331W	999
YHR188C	YDR434W	999
YHR188C	YJR015W	904
YHR188C	YLR088W	999
YHR188C	YLR459W	934
YHR190W	YER044C	999
YHR190W	YGL001C	999
YHR190W	YGR060W	999
YHR190W	YGR175C	964
YHR190W	YHR007C	999
YHR190W	YJL167W	926
YHR190W	YLR056W	999
YHR190W	YLR100W	999
YHR190W	YML008C	999
YHR190W	YPL069C	923
YHR191C	YCL016C	999
YHR191C	YDR225W	924
YHR191C	YEL003W	948
YHR191C	YER016W	989
YHR191C	YGL066W	926
YHR191C	YGL086W	939
YHR191C	YGL167C	901
YHR191C	YGL168W	901
YHR191C	YGR078C	902
YHR191C	YGR229C	985
YHR191C	YJL030W	967
YHR191C	YJL092W	914
YHR191C	YMR048W	996
YHR191C	YMR078C	999
YHR191C	YNL153C	952
YHR191C	YNL273W	992
YHR191C	YNL298W	962
YHR191C	YOR195W	953
YHR191C	YPL008W	999
YHR191C	YPL253C	923
YHR191C	YPR119W	978
YHR191C	YPR135W	992
YHR191C	YPR141C	946
YHR193C	YDR252W	999
YHR193C	YML062C	913
YHR193C	YNL112W	974

YHR193C	YPL037C	999
YHR194W	YAL010C	905
YHR194W	YGL219C	917
YHR194W	YLL006W	905
YHR194W	YOL009C	913
YHR194W	YOR147W	975
YHR195W	YAR042W	989
YHR195W	YDL015C	992
YHR195W	YEL013W	998
YHR195W	YLR183C	935
YHR196W	YBL004W	999
YHR196W	YBR247C	958
YHR196W	YCR057C	999
YHR196W	YDL014W	972
YHR196W	YDL148C	991
YHR196W	YDR299W	945
YHR196W	YDR324C	999
YHR196W	YDR398W	999
YHR196W	YDR449C	999
YHR196W	YEL055C	999
YHR196W	YER082C	999
YHR196W	YGL120C	960
YHR196W	YGL171W	910
YHR196W	YGR090W	982
YHR196W	YGR128C	999
YHR196W	YGR145W	916
YHR196W	YHR148W	999
YHR196W	YHR169W	911
YHR196W	YJL010C	983
YHR196W	YJL033W	940
YHR196W	YJL109C	999
YHR196W	YJR002W	999
YHR196W	YLL011W	999
YHR196W	YLR003C	973
YHR196W	YLR129W	999
YHR196W	YLR175W	996
YHR196W	YLR186W	999
YHR196W	YLR197W	999
YHR196W	YLR222C	919
YHR196W	YLR409C	910
YHR196W	YML093W	938
YHR196W	YMR093W	999
YHR196W	YMR229C	972
YHR196W	YMR290C	980

YHR196W	YNL132W	977
YHR196W	YOL010W	971
YHR196W	YOR004W	915
YHR196W	YOR078W	971
YHR196W	YOR145C	970
YHR196W	YOR310C	985
YHR196W	YPL012W	972
YHR196W	YPL126W	999
YHR196W	YPL217C	999
YHR196W	YPL266W	989
YHR196W	YPR112C	932
YHR196W	YPR137W	999
YHR196W	YPR144C	999
YHR197W	YCR072C	999
YHR197W	YDL031W	984
YHR197W	YDR101C	996
YHR197W	YER006W	999
YHR197W	YER126C	999
YHR197W	YFL002C	996
YHR197W	YGL111W	988
YHR197W	YGR103W	998
YHR197W	YGR245C	999
YHR197W	YHR052W	998
YHR197W	YHR085W	999
YHR197W	YHR088W	985
YHR197W	YHR170W	938
YHR197W	YKL009W	991
YHR197W	YKR081C	999
YHR197W	YLL008W	989
YHR197W	YLL034C	981
YHR197W	YLR002C	991
YHR197W	YLR074C	983
YHR197W	YLR106C	999
YHR197W	YLR423C	906
YHR197W	YMR049C	983
YHR197W	YMR290C	992
YHR197W	YNL002C	994
YHR197W	YNL061W	988
YHR197W	YNL110C	999
YHR197W	YNL182C	999
YHR197W	YNR053C	995
YHR197W	YOL077C	996
YHR197W	YOL127W	963
YHR197W	YOR063W	933

YHR197W	YOR206W	980
YHR197W	YOR272W	992
YHR197W	YPL093W	999
YHR197W	YPL211W	993
YHR197W	YPR016C	999
YHR200W	YAR003W	980
YHR200W	YBL041W	997
YHR200W	YDL007W	999
YHR200W	YDL020C	903
YHR200W	YDL074C	913
YHR200W	YDL097C	999
YHR200W	YDL147W	999
YHR200W	YDL190C	980
YHR200W	YDR363W-A	999
YHR200W	YDR394W	999
YHR200W	YDR427W	999
YHR200W	YEL009C	978
YHR200W	YEL037C	999
YHR200W	YER012W	999
YHR200W	YER021W	999
YHR200W	YER094C	999
YHR200W	YFR004W	999
YHR200W	YFR010W	999
YHR200W	YFR050C	999
YHR200W	YFR052W	999
YHR200W	YGL004C	941
YHR200W	YGL011C	998
YHR200W	YGL048C	999
YHR200W	YGL141W	932
YHR200W	YGR135W	999
YHR200W	YGR229C	918
YHR200W	YGR232W	999
YHR200W	YGR253C	948
YHR200W	YHL030W	999
YHR200W	YHR027C	999
YHR200W	YIL075C	999
YHR200W	YIL148W	940
YHR200W	YJL001W	914
YHR200W	YKL145W	999
YHR200W	YKR094C	933
YHR200W	YLL039C	961
YHR200W	YLR021W	967
YHR200W	YLR079W	999
YHR200W	YLR167W	932

YHR200W	YLR421C	999
YHR200W	YML092C	998
YHR200W	YMR191W	987
YHR200W	YMR276W	998
YHR200W	YMR314W	999
YHR200W	YNL298W	988
YHR200W	YOL038W	996
YHR200W	YOR117W	999
YHR200W	YOR157C	981
YHR200W	YOR259C	999
YHR200W	YOR261C	999
YHR200W	YOR362C	965
YHR200W	YPL144W	967
YHR200W	YPR103W	999
YHR200W	YPR108W	999
YHR201C	YDR452W	985
YHR203C	YAL035W	916
YHR203C	YBL027W	971
YHR203C	YBL072C	996
YHR203C	YBL087C	975
YHR203C	YBL092W	984
YHR203C	YBR031W	999
YHR203C	YBR048W	999
YHR203C	YBR079C	913
YHR203C	YBR084C-A	999
YHR203C	YBR118W	946
YHR203C	YBR181C	995
YHR203C	YBR189W	998
YHR203C	YBR191W	986
YHR203C	YCL011C	923
YHR203C	YCL059C	998
YHR203C	YCR031C	998
YHR203C	YDL014W	936
YHR203C	YDL061C	985
YHR203C	YDL075W	993
YHR203C	YDL081C	943
YHR203C	YDL082W	996
YHR203C	YDL083C	968
YHR203C	YDL130W	941
YHR203C	YDL136W	999
YHR203C	YDL191W	911
YHR203C	YDR012W	999
YHR203C	YDR025W	998
YHR203C	YDR064W	999

YHR203C	YDR117C	940
YHR203C	YDR382W	985
YHR203C	YDR385W	912
YHR203C	YDR418W	998
YHR203C	YDR447C	996
YHR203C	YDR450W	924
YHR203C	YDR471W	998
YHR203C	YDR500C	999
YHR203C	YEL054C	926
YHR203C	YER025W	953
YHR203C	YER056C-A	994
YHR203C	YER074W	995
YHR203C	YER102W	999
YHR203C	YER117W	999
YHR203C	YER131W	925
YHR203C	YER165W	929
YHR203C	YFL034C-A	927
YHR203C	YFR031C-A	998
YHR203C	YFR032C-A	940
YHR203C	YGL030W	999
YHR203C	YGL076C	940
YHR203C	YGL103W	999
YHR203C	YGL123W	995
YHR203C	YGL135W	996
YHR203C	YGL147C	973
YHR203C	YGL189C	913
YHR203C	YGR027C	932
YHR203C	YGR034W	999
YHR203C	YGR085C	999
YHR203C	YGR118W	978
YHR203C	YGR148C	934
YHR203C	YGR214W	956
YHR203C	YHL001W	998
YHR203C	YHL015W	969
YHR203C	YHL033C	999
YHR203C	YHR010W	949
YHR203C	YHR021C	922
YHR203C	YHR026W	974
YHR203C	YHR141C	946
YHR203C	YHR216W	972
YHR203C	YIL018W	998
YHR203C	YIL052C	992
YHR203C	YIL069C	995
YHR203C	YIL133C	996

YHR203C	YIL148W	999
YHR203C	YJL033W	972
YHR203C	YJL136C	942
YHR203C	YJL177W	999
YHR203C	YJL189W	931
YHR203C	YJL190C	998
YHR203C	YJL191W	999
YHR203C	YJR007W	902
YHR203C	YJR094W-A	980
YHR203C	YJR123W	998
YHR203C	YJR145C	996
YHR203C	YKL006W	978
YHR203C	YKL156W	962
YHR203C	YKL180W	999
YHR203C	YKR057W	930
YHR203C	YKR059W	999
YHR203C	YKR094C	910
YHR203C	YLL045C	997
YHR203C	YLR029C	997
YHR203C	YLR048W	984
YHR203C	YLR060W	953
YHR203C	YLR061W	996
YHR203C	YLR075W	999
YHR203C	YLR167W	999
YHR203C	YLR175W	991
YHR203C	YLR185W	999
YHR203C	YLR197W	929
YHR203C	YLR264W	958
YHR203C	YLR287C-A	906
YHR203C	YLR333C	905
YHR203C	YLR340W	984
YHR203C	YLR344W	995
YHR203C	YLR367W	980
YHR203C	YLR388W	997
YHR203C	YLR406C	929
YHR203C	YLR432W	976
YHR203C	YLR441C	996
YHR203C	YLR448W	999
YHR203C	YML024W	995
YHR203C	YML026C	924
YHR203C	YML063W	998
YHR203C	YML073C	996
YHR203C	YMR121C	914
YHR203C	YMR142C	995

YHR203C	YMR194W	999
YHR203C	YMR230W	928
YHR203C	YMR242C	997
YHR203C	YMR260C	913
YHR203C	YMR290C	938
YHR203C	YNL067W	984
YHR203C	YNL069C	997
YHR203C	YNL096C	933
YHR203C	YNL132W	935
YHR203C	YNL162W	946
YHR203C	YNL178W	999
YHR203C	YNL301C	999
YHR203C	YNL302C	921
YHR203C	YNR037C	939
YHR203C	YOL010W	972
YHR203C	YOL039W	910
YHR203C	YOL040C	982
YHR203C	YOL041C	925
YHR203C	YOL120C	916
YHR203C	YOL121C	922
YHR203C	YOL127W	999
YHR203C	YOR063W	999
YHR203C	YOR096W	998
YHR203C	YOR133W	994
YHR203C	YOR167C	916
YHR203C	YOR182C	906
YHR203C	YOR234C	957
YHR203C	YOR293W	943
YHR203C	YOR310C	981
YHR203C	YOR312C	997
YHR203C	YOR369C	923
YHR203C	YPL012W	931
YHR203C	YPL037C	935
YHR203C	YPL079W	956
YHR203C	YPL081W	921
YHR203C	YPL090C	995
YHR203C	YPL131W	994
YHR203C	YPL143W	964
YHR203C	YPL198W	997
YHR203C	YPL220W	999
YHR203C	YPL237W	910
YHR203C	YPL249C-A	998
YHR203C	YPR043W	996
YHR203C	YPR052C	968

YHR203C	YPR080W	922
YHR203C	YPR102C	983
YHR203C	YPR132W	999
YHR204W	YCL043C	999
YHR204W	YDR057W	956
YHR204W	YHR176W	923
YHR205W	YDL035C	906
YHR205W	YDR096W	922
YHR205W	YDR490C	946
YHR205W	YER020W	976
YHR205W	YFL033C	999
YHR205W	YHR008C	912
YHR205W	YJL005W	932
YHR205W	YJL164C	978
YHR205W	YJR066W	994
YHR205W	YKL062W	943
YHR205W	YKL093W	903
YHR205W	YLR310C	980
YHR205W	YMR037C	944
YHR205W	YNL098C	987
YHR205W	YNL167C	975
YHR205W	YPR104C	908
YHR206W	YBL105C	908
YHR206W	YCR093W	965
YHR206W	YDL056W	925
YHR206W	YDL235C	999
YHR206W	YDR353W	940
YHR206W	YFL009W	926
YHR206W	YGL038C	958
YHR206W	YGR097W	921
YHR206W	YGR209C	998
YHR206W	YIL147C	999
YHR206W	YJL174W	983
YHR206W	YLR229C	971
YHR206W	YML007W	997
YHR206W	YNL027W	973
YHR206W	YPL040C	914
YHR206W	YPL091W	909
YHR206W	YPR165W	981
YHR208W	YCL018W	911
YHR208W	YGR094W	911
YHR208W	YJR016C	943
YHR208W	YJR148W	999
YHR208W	YMR108W	911

YHR208W	YNL104C	902
YHR211W	YAL063C	920
YHR211W	YAR050W	926
YHR211W	YIL169C	922
YHR211W	YIR019C	956
YHR211W	YKR102W	904
YHR214W-A	YKL166C	905
YHR214W-A	YPL203W	901
YHR215W	YAR071W	999
YHR215W	YER081W	989
YHR216W	YAR073W	990
YHR216W	YDL082W	974
YHR216W	YDR228C	975
YHR216W	YHR203C	972
YHR216W	YLR028C	902
YHR216W	YLR432W	999
YHR216W	YML056C	999
YHR216W	YMR120C	901
YHR216W	YMR142C	970
YHR216W	YMR217W	988
YHR216W	YNL220W	930
YHR216W	YOR250C	990
YHR219W	YHL050C	976
YHR219W	YJL225C	960
YIL001W	YGR003W	999
YIL001W	YOL133W	931
YIL002C	YCR088W	933
YIL002C	YJL083W	965
YIL002C	YKL203C	911
YIL002C	YKL212W	951
YIL002C	YKR019C	927
YIL002C	YLR371W	990
YIL002C	YNL106C	999
YIL002C	YNL267W	959
YIL002C	YOR008C	936
YIL002C	YOR109W	997
YIL003W	YGL091C	995
YIL003W	YNL240C	990
YIL004C	YDL058W	993
YIL004C	YDL192W	998
YIL004C	YDL195W	958
YIL004C	YDR189W	999
YIL004C	YDR238C	906
YIL004C	YER122C	953

YIL004C	YFL038C	999
YIL004C	YGL137W	975
YIL004C	YGL223C	966
YIL004C	YGR120C	985
YIL004C	YHL031C	999
YIL004C	YIL109C	999
YIL004C	YKL006C-A	999
YIL004C	YKL196C	999
YIL004C	YKR068C	912
YIL004C	YLR026C	999
YIL004C	YLR078C	999
YIL004C	YLR093C	999
YIL004C	YLR268W	999
YIL004C	YNL287W	998
YIL004C	YOR327C	998
YIL004C	YPL218W	999
YIL004C	YPR181C	999
YIL005W	YDR518W	951
YIL005W	YGL008C	997
YIL005W	YMR186W	955
YIL006W	YPL240C	919
YIL007C	YBL041W	999
YIL007C	YDL007W	903
YIL007C	YDR394W	902
YIL007C	YFR050C	932
YIL007C	YGL048C	905
YIL007C	YGR253C	903
YIL007C	YHR027C	921
YIL007C	YHR185C	993
YIL007C	YKL145W	903
YIL007C	YOR117W	974
YIL007C	YOR259C	911
YIL007C	YOR362C	902
YIL008W	YFL021W	900
YIL008W	YHR111W	999
YIL008W	YLR109W	929
YIL009C-A	YDL220C	990
YIL009C-A	YDR082W	916
YIL009C-A	YLR318W	999
YIL009C-A	YML092C	912
YIL009W	YGL205W	901
YIL011W	YMR201C	904
YIL013C	YDR011W	906
YIL013C	YDR406W	908

YIL013C	YOR011W	984
YIL013C	YOR153W	907
YIL013C	YOR328W	910
YIL015W	YFL026W	986
YIL015W	YKL209C	958
YIL015W	YOR212W	919
YIL016W	YAL005C	977
YIL016W	YDL229W	979
YIL016W	YLR173W	998
YIL016W	YMR047C	912
YIL017C	YBL049W	929
YIL017C	YBR105C	999
YIL017C	YCL039W	995
YIL017C	YDR255C	999
YIL017C	YGL227W	999
YIL017C	YIL097W	999
YIL017C	YMR135C	999
YIL018W	YAL035W	999
YIL018W	YBL027W	973
YIL018W	YBL072C	915
YIL018W	YBL087C	981
YIL018W	YBL092W	971
YIL018W	YBR031W	999
YIL018W	YBR048W	998
YIL018W	YBR084C-A	973
YIL018W	YBR118W	905
YIL018W	YBR181C	995
YIL018W	YBR189W	901
YIL018W	YBR191W	914
YIL018W	YCR031C	997
YIL018W	YDL014W	937
YIL018W	YDL061C	935
YIL018W	YDL075W	996
YIL018W	YDL081C	932
YIL018W	YDL082W	996
YIL018W	YDL083C	962
YIL018W	YDL136W	996
YIL018W	YDL191W	911
YIL018W	YDR012W	998
YIL018W	YDR025W	998
YIL018W	YDR041W	961
YIL018W	YDR064W	995
YIL018W	YDR382W	995
YIL018W	YDR385W	913

YIL018W	YDR418W	995
YIL018W	YDR447C	996
YIL018W	YDR450W	996
YIL018W	YDR471W	995
YIL018W	YDR496C	933
YIL018W	YEL054C	906
YIL018W	YER056C-A	903
YIL018W	YER074W	995
YIL018W	YER102W	994
YIL018W	YER117W	981
YIL018W	YFR031C-A	996
YIL018W	YFR032C-A	903
YIL018W	YGL030W	995
YIL018W	YGL031C	908
YIL018W	YGL076C	995
YIL018W	YGL099W	946
YIL018W	YGL103W	997
YIL018W	YGL123W	978
YIL018W	YGL135W	995
YIL018W	YGL147C	998
YIL018W	YGR027C	913
YIL018W	YGR034W	998
YIL018W	YGR085C	999
YIL018W	YGR103W	996
YIL018W	YGR118W	916
YIL018W	YGR148C	908
YIL018W	YGR214W	913
YIL018W	YHL001W	994
YIL018W	YHL015W	904
YIL018W	YHL033C	995
YIL018W	YHR010W	995
YIL018W	YHR021C	904
YIL018W	YHR141C	920
YIL018W	YHR203C	998
YIL018W	YIL052C	903
YIL018W	YIL069C	995
YIL018W	YIL133C	996
YIL018W	YIL148W	901
YIL018W	YJL136C	912
YIL018W	YJL177W	998
YIL018W	YJL189W	908
YIL018W	YJL190C	998
YIL018W	YJL191W	934
YIL018W	YJR094W-A	929

YIL018W	YJR123W	997
YIL018W	YJR145C	998
YIL018W	YKL156W	904
YIL018W	YKL170W	970
YIL018W	YKL180W	998
YIL018W	YKR057W	912
YIL018W	YKR094C	901
YIL018W	YLL045C	996
YIL018W	YLR029C	996
YIL018W	YLR048W	913
YIL018W	YLR061W	901
YIL018W	YLR075W	997
YIL018W	YLR264W	917
YIL018W	YLR333C	913
YIL018W	YLR340W	997
YIL018W	YLR344W	970
YIL018W	YLR367W	961
YIL018W	YLR388W	948
YIL018W	YLR406C	912
YIL018W	YLR441C	996
YIL018W	YLR448W	996
YIL018W	YML024W	913
YIL018W	YML026C	924
YIL018W	YML063W	995
YIL018W	YML073C	996
YIL018W	YMR121C	926
YIL018W	YMR142C	995
YIL018W	YMR194W	996
YIL018W	YMR230W	904
YIL018W	YMR242C	996
YIL018W	YMR290C	938
YIL018W	YNL002C	931
YIL018W	YNL061W	931
YIL018W	YNL067W	973
YIL018W	YNL069C	996
YIL018W	YNL081C	950
YIL018W	YNL132W	936
YIL018W	YNL162W	920
YIL018W	YNL178W	999
YIL018W	YNL301C	996
YIL018W	YNL302C	911
YIL018W	YNR037C	982
YIL018W	YOL039W	913
YIL018W	YOL040C	999

YIL018W	YOL120C	998
YIL018W	YOL121C	911
YIL018W	YOL127W	999
YIL018W	YOR063W	999
YIL018W	YOR096W	996
YIL018W	YOR133W	913
YIL018W	YOR167C	916
YIL018W	YOR204W	942
YIL018W	YOR234C	907
YIL018W	YOR293W	905
YIL018W	YOR312C	996
YIL018W	YPL079W	914
YIL018W	YPL081W	901
YIL018W	YPL090C	994
YIL018W	YPL131W	987
YIL018W	YPL143W	997
YIL018W	YPL198W	996
YIL018W	YPL220W	995
YIL018W	YPL249C-A	996
YIL018W	YPR016C	927
YIL018W	YPR043W	999
YIL018W	YPR080W	905
YIL018W	YPR102C	983
YIL018W	YPR132W	916
YIL019W	YCL059C	991
YIL019W	YKL099C	939
YIL020C	YBR248C	930
YIL020C	YCL030C	962
YIL021W	YBL026W	907
YIL021W	YBR154C	999
YIL021W	YBR193C	975
YIL021W	YDL005C	975
YIL021W	YDL115C	999
YIL021W	YDL140C	999
YIL021W	YDR167W	908
YIL021W	YDR404C	999
YIL021W	YDR432W	975
YIL021W	YEL026W	900
YIL021W	YER022W	981
YIL021W	YER148W	988
YIL021W	YER171W	921
YIL021W	YGL025C	975
YIL021W	YGL043W	999
YIL021W	YGL070C	999

YIL021W	YGL112C	900
YIL021W	YGL127C	928
YIL021W	YGL207W	946
YIL021W	YGR005C	999
YIL021W	YGR063C	991
YIL021W	YGR104C	996
YIL021W	YGR186W	999
YIL021W	YHR041C	978
YIL021W	YHR058C	984
YIL021W	YHR143W-A	999
YIL021W	YIL143C	934
YIL021W	YJL140W	999
YIL021W	YJL168C	972
YIL021W	YJR035W	900
YIL021W	YKL028W	978
YIL021W	YKL058W	902
YIL021W	YLR071C	999
YIL021W	YML010W	999
YIL021W	YMR186W	963
YIL021W	YMR277W	965
YIL021W	YNL113W	999
YIL021W	YNL236W	907
YIL021W	YNR010W	982
YIL021W	YOL005C	999
YIL021W	YOL051W	964
YIL021W	YOL135C	978
YIL021W	YOR116C	941
YIL021W	YOR151C	999
YIL021W	YOR174W	975
YIL021W	YOR207C	938
YIL021W	YOR210W	999
YIL021W	YOR224C	999
YIL021W	YPL129W	999
YIL021W	YPL178W	983
YIL021W	YPL240C	919
YIL021W	YPR086W	939
YIL021W	YPR168W	936
YIL021W	YPR187W	999
YIL022W	YGL027C	998
YIL022W	YGR033C	966
YIL022W	YJL104W	999
YIL022W	YJL143W	999
YIL022W	YJR045C	999
YIL022W	YKR065C	928

YIL022W	YLR008C	999
YIL022W	YLR369W	969
YIL022W	YNL328C	910
YIL022W	YNR017W	999
YIL022W	YOR232W	999
YIL023C	YDR205W	918
YIL023C	YOR316C	904
YIL026C	YBR086C	921
YIL026C	YDL003W	999
YIL026C	YDR180W	994
YIL026C	YFL008W	999
YIL026C	YFR027W	907
YIL026C	YHR156C	981
YIL026C	YJL074C	999
YIL026C	YMR001C	992
YIL026C	YNR053C	927
YIL030C	YBR170C	997
YIL030C	YDL126C	999
YIL030C	YER100W	938
YIL030C	YML013W	999
YIL030C	YMR022W	997
YIL030C	YMR061W	990
YIL030C	YMR123W	913
YIL030C	YMR264W	999
YIL030C	YOL013C	991
YIL031W	YAR002W	911
YIL031W	YDL064W	900
YIL031W	YDR390C	929
YIL031W	YDR409W	926
YIL031W	YDR510W	981
YIL031W	YFR031C	903
YIL031W	YMR076C	901
YIL031W	YNL088W	954
YIL031W	YPL020C	997
YIL033C	YDR028C	909
YIL033C	YFL033C	960
YIL033C	YJL005W	999
YIL033C	YJL164C	999
YIL033C	YKL062W	916
YIL033C	YKL166C	999
YIL033C	YMR273C	963
YIL033C	YNL093W	911
YIL033C	YNL098C	999
YIL033C	YOR101W	998

YIL033C	YPL106C	975
YIL033C	YPL203W	999
YIL034C	YDR129C	997
YIL034C	YDR245W	906
YIL034C	YGR080W	936
YIL034C	YIR003W	999
YIL034C	YKL007W	999
YIL034C	YLL050C	953
YIL035C	YBR279W	985
YIL035C	YCL031C	999
YIL035C	YCL059C	911
YIL035C	YDR043C	932
YIL035C	YDR299W	914
YIL035C	YER164W	999
YIL035C	YGL019W	999
YIL035C	YGL207W	999
YIL035C	YGL244W	997
YIL035C	YGR090W	999
YIL035C	YGR116W	934
YIL035C	YGR145W	928
YIL035C	YIL084C	991
YIL035C	YJL069C	908
YIL035C	YJR093C	976
YIL035C	YKL088W	999
YIL035C	YKL160W	987
YIL035C	YLR079W	909
YIL035C	YLR197W	906
YIL035C	YLR418C	995
YIL035C	YML069W	999
YIL035C	YMR172W	997
YIL035C	YMR229C	933
YIL035C	YMR290C	910
YIL035C	YNL088W	914
YIL035C	YNL132W	901
YIL035C	YOL145C	999
YIL035C	YOR039W	999
YIL035C	YOR061W	999
YIL035C	YOR123C	968
YIL035C	YPR143W	920
YIL036W	YLR113W	905
YIL038C	YAL021C	999
YIL038C	YCR093W	999
YIL038C	YDL165W	999
YIL038C	YDL224C	923

YIL038C	YER068W	999
YIL038C	YFL033C	906
YIL038C	YNL091W	927
YIL038C	YNL288W	997
YIL038C	YNR052C	999
YIL038C	YPR072W	999
YIL040W	YOL012C	944
YIL041W	YGR229C	907
YIL041W	YPL240C	902
YIL042C	YER178W	984
YIL042C	YOR090C	912
YIL043C	YNL111C	902
YIL044C	YDL192W	984
YIL044C	YDL226C	999
YIL044C	YDR320C	911
YIL044C	YOR089C	915
YIL045W	YLR258W	983
YIL046W	YBR280C	910
YIL046W	YDL132W	999
YIL046W	YDR054C	999
YIL046W	YDR253C	966
YIL046W	YDR328C	999
YIL046W	YFL009W	986
YIL046W	YJL187C	998
YIL046W	YJR060W	912
YIL046W	YJR090C	953
YIL046W	YNL103W	999
YIL046W	YOL133W	991
YIL047C	YOR212W	978
YIL048W	YCR094W	912
YIL048W	YNL297C	936
YIL049W	YHR084W	909
YIL049W	YLR362W	912
YIL049W	YNL098C	902
YIL050W	YBR160W	963
YIL050W	YGR233C	996
YIL050W	YJL084C	987
YIL050W	YMR311C	967
YIL050W	YPL031C	999
YIL052C	YAL003W	968
YIL052C	YAL035W	925
YIL052C	YBL027W	904
YIL052C	YBL087C	901
YIL052C	YBL092W	933

YIL052C	YBR031W	950
YIL052C	YBR048W	918
YIL052C	YBR084C-A	995
YIL052C	YBR189W	997
YIL052C	YBR191W	999
YIL052C	YCR031C	993
YIL052C	YDL061C	999
YIL052C	YDL075W	999
YIL052C	YDL081C	908
YIL052C	YDL082W	910
YIL052C	YDL130W	948
YIL052C	YDL136W	990
YIL052C	YDL191W	905
YIL052C	YDL192W	972
YIL052C	YDR012W	970
YIL052C	YDR064W	999
YIL052C	YDR382W	999
YIL052C	YDR418W	997
YIL052C	YDR447C	924
YIL052C	YDR450W	902
YIL052C	YDR471W	996
YIL052C	YDR500C	996
YIL052C	YEL054C	915
YIL052C	YER056C-A	999
YIL052C	YER074W	906
YIL052C	YER102W	995
YIL052C	YER117W	995
YIL052C	YER131W	970
YIL052C	YFL034C-A	911
YIL052C	YFR031C-A	903
YIL052C	YFR032C-A	906
YIL052C	YGL030W	999
YIL052C	YGL031C	960
YIL052C	YGL076C	953
YIL052C	YGL103W	999
YIL052C	YGL123W	999
YIL052C	YGL135W	913
YIL052C	YGL147C	912
YIL052C	YGL189C	910
YIL052C	YGR027C	999
YIL052C	YGR034W	911
YIL052C	YGR085C	994
YIL052C	YGR118W	999
YIL052C	YGR148C	999

YIL052C	YGR214W	997
YIL052C	YHL001W	999
YIL052C	YHL015W	999
YIL052C	YHL033C	999
YIL052C	YHR010W	998
YIL052C	YHR021C	903
YIL052C	YHR141C	916
YIL052C	YHR203C	992
YIL052C	YIL018W	903
YIL052C	YIL069C	906
YIL052C	YIL133C	913
YIL052C	YIL148W	994
YIL052C	YJL136C	976
YIL052C	YJL177W	995
YIL052C	YJL189W	961
YIL052C	YJL190C	900
YIL052C	YJL191W	980
YIL052C	YJR094W-A	999
YIL052C	YJR123W	999
YIL052C	YJR145C	913
YIL052C	YKL006W	999
YIL052C	YKL062W	923
YIL052C	YKL156W	997
YIL052C	YKL180W	999
YIL052C	YKR057W	909
YIL052C	YLL045C	934
YIL052C	YLR029C	992
YIL052C	YLR048W	999
YIL052C	YLR061W	999
YIL052C	YLR075W	999
YIL052C	YLR167W	994
YIL052C	YLR185W	978
YIL052C	YLR264W	997
YIL052C	YLR287C-A	907
YIL052C	YLR333C	931
YIL052C	YLR340W	995
YIL052C	YLR344W	999
YIL052C	YLR367W	979
YIL052C	YLR388W	999
YIL052C	YLR406C	910
YIL052C	YLR441C	905
YIL052C	YLR448W	999
YIL052C	YML024W	999
YIL052C	YML026C	902

YIL052C	YML063W	998
YIL052C	YML073C	911
YIL052C	YMR121C	908
YIL052C	YMR142C	923
YIL052C	YMR194W	999
YIL052C	YMR230W	952
YIL052C	YMR242C	903
YIL052C	YMR260C	902
YIL052C	YNL067W	995
YIL052C	YNL069C	913
YIL052C	YNL096C	921
YIL052C	YNL162W	916
YIL052C	YNL178W	999
YIL052C	YNL209W	978
YIL052C	YNL301C	977
YIL052C	YNL302C	905
YIL052C	YOL039W	921
YIL052C	YOL040C	901
YIL052C	YOL121C	905
YIL052C	YOL127W	999
YIL052C	YOR063W	996
YIL052C	YOR096W	999
YIL052C	YOR133W	930
YIL052C	YOR167C	954
YIL052C	YOR182C	907
YIL052C	YOR234C	993
YIL052C	YOR293W	991
YIL052C	YOR312C	928
YIL052C	YOR369C	912
YIL052C	YPL037C	999
YIL052C	YPL079W	977
YIL052C	YPL081W	939
YIL052C	YPL131W	997
YIL052C	YPL143W	996
YIL052C	YPL198W	910
YIL052C	YPL220W	994
YIL052C	YPL240C	920
YIL052C	YPL249C-A	954
YIL052C	YPR043W	960
YIL052C	YPR102C	999
YIL052C	YPR132W	994
YIL053W	YDL022W	961
YIL053W	YER062C	969
YIL053W	YFL048C	935

YIL053W	YMR303C	927
YIL053W	YPL201C	934
YIL053W	YPL240C	919
YIL061C	YBR102C	921
YIL061C	YBR119W	999
YIL061C	YDL087C	999
YIL061C	YDR235W	999
YIL061C	YDR240C	999
YIL061C	YER029C	997
YIL061C	YER172C	986
YIL061C	YFL017W-A	990
YIL061C	YGR013W	999
YIL061C	YGR074W	993
YIL061C	YHR086W	999
YIL061C	YHR165C	999
YIL061C	YKL012W	999
YIL061C	YKL173W	908
YIL061C	YLR147C	993
YIL061C	YLR275W	993
YIL061C	YLR298C	999
YIL061C	YML046W	999
YIL061C	YML049C	976
YIL061C	YMR125W	999
YIL061C	YPL178W	999
YIL061C	YPR057W	968
YIL061C	YPR182W	993
YIL062C	YBR234C	999
YIL062C	YDL029W	999
YIL062C	YJR065C	999
YIL062C	YKL013C	999
YIL062C	YLR370C	999
YIL062C	YLR429W	979
YIL062C	YMR109W	948
YIL062C	YNR035C	999
YIL062C	YOR181W	971
YIL063C	YDR159W	915
YIL063C	YGL097W	937
YIL063C	YGR163W	913
YIL063C	YGR218W	999
YIL063C	YKL057C	984
YIL063C	YKL143W	925
YIL063C	YLR293C	999
YIL063C	YLR335W	908
YIL063C	YML121W	907

YIL063C	YMR047C	962
YIL063C	YMR235C	944
YIL065C	YBR179C	976
YIL065C	YJL112W	999
YIL065C	YKR036C	999
YIL065C	YLL001W	997
YIL065C	YPL240C	919
YIL066C	YCR083W	900
YIL066C	YCR084C	930
YIL066C	YDL101C	955
YIL066C	YER070W	999
YIL066C	YGR180C	999
YIL066C	YGR209C	989
YIL066C	YJL026W	999
YIL066C	YJR057W	914
YIL066C	YKL024C	900
YIL066C	YKL067W	909
YIL066C	YLR043C	989
YIL066C	YPL091W	988
YIL068C	YBR102C	999
YIL068C	YDR164C	981
YIL068C	YDR166C	999
YIL068C	YDR233C	967
YIL068C	YER008C	999
YIL068C	YGL233W	999
YIL068C	YGR009C	994
YIL068C	YJL085W	999
YIL068C	YKR001C	977
YIL068C	YLR166C	999
YIL068C	YNR049C	918
YIL068C	YPR055W	999
YIL069C	YBL027W	902
YIL069C	YBL072C	996
YIL069C	YBL087C	916
YIL069C	YBL092W	907
YIL069C	YBR031W	996
YIL069C	YBR048W	995
YIL069C	YBR084C-A	902
YIL069C	YBR118W	904
YIL069C	YBR181C	995
YIL069C	YBR189W	996
YIL069C	YBR191W	907
YIL069C	YCR031C	918
YIL069C	YDL060W	985

YIL069C	YDL061C	910
YIL069C	YDL075W	902
YIL069C	YDL081C	903
YIL069C	YDL082W	912
YIL069C	YDL083C	967
YIL069C	YDL130W	903
YIL069C	YDL136W	922
YIL069C	YDL148C	979
YIL069C	YDL191W	922
YIL069C	YDR012W	917
YIL069C	YDR025W	995
YIL069C	YDR064W	997
YIL069C	YDR382W	996
YIL069C	YDR418W	965
YIL069C	YDR429C	904
YIL069C	YDR447C	996
YIL069C	YDR450W	997
YIL069C	YDR471W	996
YIL069C	YDR500C	917
YIL069C	YEL054C	965
YIL069C	YER056C-A	906
YIL069C	YER074W	995
YIL069C	YER102W	995
YIL069C	YER117W	916
YIL069C	YER131W	921
YIL069C	YFL034C-A	904
YIL069C	YFR031C-A	905
YIL069C	YFR032C-A	921
YIL069C	YGL030W	997
YIL069C	YGL031C	906
YIL069C	YGL076C	996
YIL069C	YGL103W	996
YIL069C	YGL123W	913
YIL069C	YGL135W	906
YIL069C	YGL147C	905
YIL069C	YGL189C	921
YIL069C	YGR027C	916
YIL069C	YGR034W	995
YIL069C	YGR085C	996
YIL069C	YGR118W	922
YIL069C	YGR148C	906
YIL069C	YGR214W	909
YIL069C	YHL001W	996
YIL069C	YHL015W	905

YIL069C	YHL033C	908
YIL069C	YHR010W	907
YIL069C	YHR021C	918
YIL069C	YHR141C	913
YIL069C	YHR203C	995
YIL069C	YIL018W	995
YIL069C	YIL052C	906
YIL069C	YIL133C	995
YIL069C	YJL136C	922
YIL069C	YJL177W	995
YIL069C	YJL190C	995
YIL069C	YJL191W	997
YIL069C	YJR002W	925
YIL069C	YJR094W-A	917
YIL069C	YJR123W	997
YIL069C	YJR145C	996
YIL069C	YKL006W	916
YIL069C	YKL156W	918
YIL069C	YKL180W	995
YIL069C	YKR057W	922
YIL069C	YLL045C	994
YIL069C	YLR029C	996
YIL069C	YLR048W	909
YIL069C	YLR061W	905
YIL069C	YLR075W	931
YIL069C	YLR185W	918
YIL069C	YLR264W	900
YIL069C	YLR287C-A	901
YIL069C	YLR325C	929
YIL069C	YLR333C	915
YIL069C	YLR340W	911
YIL069C	YLR344W	905
YIL069C	YLR367W	997
YIL069C	YLR388W	911
YIL069C	YLR406C	902
YIL069C	YLR441C	918
YIL069C	YLR448W	921
YIL069C	YML024W	997
YIL069C	YML026C	997
YIL069C	YML063W	996
YIL069C	YML073C	996
YIL069C	YMR121C	915
YIL069C	YMR142C	910
YIL069C	YMR194W	922

YIL069C	YMR230W	920
YIL069C	YMR242C	996
YIL069C	YMR260C	905
YIL069C	YNL067W	905
YIL069C	YNL069C	995
YIL069C	YNL096C	920
YIL069C	YNL132W	964
YIL069C	YNL162W	913
YIL069C	YNL178W	996
YIL069C	YNL301C	902
YIL069C	YNL302C	931
YIL069C	YOL040C	945
YIL069C	YOL120C	902
YIL069C	YOL121C	932
YIL069C	YOL127W	995
YIL069C	YOR063W	994
YIL069C	YOR096W	996
YIL069C	YOR167C	900
YIL069C	YOR182C	901
YIL069C	YOR204W	960
YIL069C	YOR234C	923
YIL069C	YOR293W	920
YIL069C	YOR312C	995
YIL069C	YOR369C	937
YIL069C	YPL079W	906
YIL069C	YPL081W	997
YIL069C	YPL090C	994
YIL069C	YPL131W	931
YIL069C	YPL143W	924
YIL069C	YPL198W	996
YIL069C	YPL220W	906
YIL069C	YPL249C-A	923
YIL069C	YPR043W	917
YIL069C	YPR080W	909
YIL069C	YPR102C	905
YIL069C	YPR132W	922
YIL070C	YGR165W	986
YIL070C	YIL105C	927
YIL070C	YJL115W	935
YIL070C	YML054C	942
YIL070C	YNL137C	996
YIL070C	YOR326W	924
YIL071C	YDL147W	997
YIL071C	YDL216C	999

YIL071C	YDR179C	999
YIL071C	YJR084W	958
YIL071C	YMR025W	999
YIL071C	YOL117W	999
YIL071C	YOR361C	915
YIL072W	YER179W	972
YIL072W	YGL183C	936
YIL072W	YHR157W	992
YIL072W	YLR263W	999
YIL072W	YOR351C	999
YIL073C	YHR153C	985
YIL074C	YER081W	999
YIL074C	YNL311C	958
YIL074C	YOR184W	986
YIL075C	YBL041W	906
YIL075C	YDL007W	999
YIL075C	YDL020C	996
YIL075C	YDL097C	999
YIL075C	YDL147W	999
YIL075C	YDR363W-A	999
YIL075C	YDR394W	998
YIL075C	YDR427W	999
YIL075C	YEL037C	961
YIL075C	YER012W	999
YIL075C	YER021W	998
YIL075C	YFL007W	940
YIL075C	YFR004W	999
YIL075C	YFR010W	999
YIL075C	YFR050C	945
YIL075C	YFR052W	999
YIL075C	YGL011C	915
YIL075C	YGL048C	999
YIL075C	YGL141W	932
YIL075C	YGR135W	901
YIL075C	YGR232W	999
YIL075C	YGR253C	901
YIL075C	YHL030W	978
YIL075C	YHR027C	999
YIL075C	YHR200W	999
YIL075C	YJL001W	946
YIL075C	YKL145W	999
YIL075C	YLL039C	972
YIL075C	YLR180W	988
YIL075C	YLR421C	999

YIL075C	YMR191W	987
YIL075C	YMR314W	997
YIL075C	YOL038W	938
YIL075C	YOR117W	998
YIL075C	YOR157C	909
YIL075C	YOR259C	998
YIL075C	YOR261C	999
YIL075C	YPR103W	940
YIL075C	YPR108W	999
YIL076W	YBL068W	930
YIL076W	YBR162C	962
YIL076W	YDL145C	999
YIL076W	YDL226C	907
YIL076W	YDR238C	998
YIL076W	YFR051C	999
YIL076W	YGL137W	999
YIL076W	YNL287W	999
YIL076W	YNR006W	905
YIL076W	YPL010W	999
YIL076W	YPL240C	919
YIL077C	YOL135C	917
YIL078W	YCR053W	937
YIL078W	YER086W	922
YIL078W	YNL135C	917
YIL079C	YDL175C	999
YIL079C	YJL050W	997
YIL079C	YNL299W	975
YIL079C	YOL115W	999
YIL083C	YKL088W	915
YIL083C	YKR072C	907
YIL084C	YAL013W	999
YIL084C	YBR095C	999
YIL084C	YBR103W	923
YIL084C	YCR033W	923
YIL084C	YDL076C	999
YIL084C	YDR207C	999
YIL084C	YGL194C	924
YIL084C	YIL035C	991
YIL084C	YKL185W	993
YIL084C	YKR029C	922
YIL084C	YMR075W	993
YIL084C	YMR128W	922
YIL084C	YMR263W	999
YIL084C	YNL097C	999

YIL084C	YNL330C	999
YIL084C	YOL004W	999
YIL084C	YPL139C	999
YIL084C	YPL181W	999
YIL084C	YPR023C	993
YIL090W	YER120W	945
YIL093C	Q0140	962
YIL093C	YBL090W	988
YIL093C	YBR146W	988
YIL093C	YBR251W	999
YIL093C	YDL045W-A	986
YIL093C	YDR036C	999
YIL093C	YDR041W	988
YIL093C	YDR175C	988
YIL093C	YDR337W	988
YIL093C	YDR347W	997
YIL093C	YGL129C	988
YIL093C	YGR084C	974
YIL093C	YGR165W	988
YIL093C	YGR215W	988
YIL093C	YHL004W	999
YIL093C	YJR101W	988
YIL093C	YJR113C	988
YIL093C	YKL155C	997
YIL093C	YMR158W	974
YIL093C	YMR188C	988
YIL093C	YNL081C	986
YIL093C	YNL137C	988
YIL093C	YNL306W	988
YIL093C	YNR037C	987
YIL093C	YOR158W	988
YIL093C	YPL013C	997
YIL093C	YPL118W	988
YIL094C	YDR234W	970
YIL094C	YGL195W	973
YIL094C	YGL206C	928
YIL094C	YHR108W	956
YIL094C	YJL200C	923
YIL094C	YLR304C	934
YIL094C	YNL037C	923
YIL094C	YOR136W	998
YIL094C	YPL140C	911
YIL095W	YCR088W	999
YIL095W	YDL029W	971

YIL095W	YER133W	948
YIL095W	YIR006C	999
YIL095W	YNL020C	985
YIL095W	YNL084C	955
YIL095W	YNL243W	939
YIL095W	YOR329C	991
YIL097W	YBL049W	929
YIL097W	YBR105C	999
YIL097W	YCL039W	995
YIL097W	YDR255C	999
YIL097W	YGL227W	999
YIL097W	YIL017C	999
YIL097W	YMR135C	999
YIL098C	YJL180C	999
YIL098C	YPL134C	929
YIL099W	YPR160W	985
YIL099W	YPR184W	985
YIL101C	YDL056W	925
YIL101C	YER111C	916
YIL101C	YPR119W	936
YIL103W	YBL071W-A	999
YIL103W	YDR385W	994
YIL103W	YKL191W	999
YIL103W	YOR133W	982
YIL104C	YDL208W	924
YIL104C	YNL124W	953
YIL105C	YBL105C	980
YIL105C	YBR036C	990
YIL105C	YBR270C	986
YIL105C	YDR208W	908
YIL105C	YER093C	996
YIL105C	YIL070C	927
YIL105C	YJL058C	980
YIL105C	YKL203C	998
YIL105C	YLR433C	976
YIL105C	YMR068W	999
YIL105C	YNL047C	999
YIL105C	YPL059W	987
YIL106W	YAL024C	942
YIL106W	YAR019C	998
YIL106W	YDL028C	987
YIL106W	YDL155W	962
YIL106W	YGR092W	999
YIL106W	YJL076W	981

YIL106W	YML064C	926
YIL106W	YMR001C	982
YIL106W	YPR111W	999
YIL107C	YER003C	900
YIL107C	YGR240C	981
YIL107C	YLR377C	978
YIL107C	YMR205C	959
YIL109C	YDL195W	999
YIL109C	YDL212W	966
YIL109C	YDR517W	999
YIL109C	YGL005C	907
YIL109C	YGL008C	981
YIL109C	YGL200C	970
YIL109C	YHR098C	998
YIL109C	YIL004C	999
YIL109C	YJL004C	990
YIL109C	YLR026C	996
YIL109C	YLR208W	996
YIL109C	YLR268W	999
YIL109C	YNL049C	999
YIL109C	YNL051W	913
YIL109C	YPL085W	999
YIL109C	YPL218W	999
YIL109C	YPR181C	999
YIL111W	Q0045	995
YIL111W	Q0250	978
YIL111W	Q0275	978
YIL111W	YDL067C	978
YIL111W	YGL187C	978
YIL111W	YGL191W	978
YIL111W	YHR051W	978
YIL111W	YLR038C	978
YIL111W	YLR395C	978
YIL111W	YMR256C	978
YIL112W	YBR103W	999
YIL112W	YCR033W	999
YIL112W	YDR155C	999
YIL112W	YDR310C	964
YIL112W	YGL194C	999
YIL112W	YGR232W	999
YIL112W	YHR124W	904
YIL112W	YJL168C	952
YIL112W	YKR029C	999
YIL112W	YMR273C	993

YIL112W	YOL068C	999
YIL113W	YHR030C	999
YIL113W	YOR231W	932
YIL114C	YNL055C	977
YIL115C	YBL079W	930
YIL115C	YDL088C	938
YIL115C	YDL116W	972
YIL115C	YDL207W	999
YIL115C	YDR159W	966
YIL115C	YDR192C	918
YIL115C	YDR424C	978
YIL115C	YER105C	957
YIL115C	YER107C	999
YIL115C	YER110C	971
YIL115C	YFR002W	958
YIL115C	YGL092W	922
YIL115C	YGL100W	949
YIL115C	YGL172W	921
YIL115C	YGR119C	931
YIL115C	YGR218W	999
YIL115C	YJL041W	999
YIL115C	YJL061W	999
YIL115C	YJR042W	992
YIL115C	YKL057C	995
YIL115C	YKL186C	977
YIL115C	YKR082W	981
YIL115C	YLR018C	989
YIL115C	YLR208W	945
YIL115C	YLR293C	972
YIL115C	YLR347C	999
YIL115C	YMR047C	999
YIL115C	YMR125W	983
YIL115C	YMR129W	942
YIL115C	YMR255W	940
YIL115C	YMR308C	963
YIL115C	YOR046C	999
YIL115C	YOR098C	912
YIL115C	YOR257W	961
YIL115C	YPL169C	997
YIL116W	YBR248C	906
YIL116W	YCL030C	998
YIL116W	YER055C	990
YIL116W	YFL022C	900
YIL116W	YLR027C	904

YIL116W	YNL316C	954
YIL116W	YOR202W	997
YIL118W	YBL085W	990
YIL118W	YBL106C	934
YIL118W	YBR200W	999
YIL118W	YBR260C	998
YIL118W	YCL037C	909
YIL118W	YDR122W	979
YIL118W	YER114C	990
YIL118W	YFL005W	996
YIL118W	YGR009C	997
YIL118W	YJL085W	999
YIL118W	YKR055W	979
YIL118W	YLR229C	996
YIL118W	YLR337C	907
YIL118W	YNL271C	982
YIL118W	YOR008C	995
YIL118W	YOR326W	985
YIL118W	YPR032W	998
YIL119C	YHR030C	902
YIL119C	YNL098C	987
YIL121W	YLL028W	936
YIL122W	YGL181W	942
YIL122W	YJL095W	909
YIL122W	YNL053W	922
YIL123W	YIL129C	971
YIL123W	YNL161W	994
YIL124W	YBR041W	951
YIL124W	YDL052C	908
YIL125W	YDR148C	999
YIL125W	YFL018C	999
YIL125W	YFR049W	984
YIL125W	YGR244C	969
YIL125W	YLL041C	908
YIL125W	YOR136W	980
YIL125W	YOR142W	966
YIL125W	YPL017C	963
YIL126W	YBL006C	960
YIL126W	YBL105C	982
YIL126W	YBR245C	999
YIL126W	YCR020W-B	999
YIL126W	YCR052W	999
YIL126W	YDR224C	999
YIL126W	YDR303C	999

YIL126W	YER025W	923
YIL126W	YER148W	926
YIL126W	YFR037C	999
YIL126W	YGL133W	970
YIL126W	YGR056W	999
YIL126W	YGR275W	999
YIL126W	YHR056C	998
YIL126W	YJL106W	985
YIL126W	YJR094C	997
YIL126W	YKR001C	982
YIL126W	YKR008W	999
YIL126W	YLR033W	999
YIL126W	YLR176C	932
YIL126W	YLR321C	999
YIL126W	YLR357W	999
YIL126W	YML127W	999
YIL126W	YMR033W	999
YIL126W	YMR091C	999
YIL126W	YOR304W	976
YIL126W	YPL082C	991
YIL126W	YPL129W	993
YIL126W	YPR034W	999
YIL128W	YBR114W	946
YIL128W	YDR098C	996
YIL128W	YDR267C	984
YIL128W	YER162C	942
YIL128W	YER171W	999
YIL128W	YGR258C	934
YIL128W	YHR122W	999
YIL128W	YIL143C	931
YIL128W	YJR052W	945
YIL128W	YML095C	940
YIL128W	YMR201C	958
YIL128W	YNL312W	910
YIL128W	YPL022W	932
YIL128W	YPL122C	906
YIL129C	YDR293C	994
YIL129C	YFL034C-B	956
YIL129C	YGL091C	909
YIL129C	YHR102W	940
YIL129C	YIL123W	971
YIL129C	YNL161W	999
YIL130W	YPR052C	998
YIL131C	YDR146C	975

YIL131C	YKR101W	991
YIL131C	YLR131C	939
YIL131C	YMR043W	963
YIL131C	YNL068C	999
YIL131C	YOR372C	950
YIL131C	YPR119W	997
YIL131C	YPR120C	932
YIL132C	YBR084C-A	938
YIL132C	YLR185W	901
YIL132C	YLR376C	994
YIL132C	YMR190C	960
YIL133C	YBL027W	999
YIL133C	YBL072C	921
YIL133C	YBL087C	996
YIL133C	YBL092W	995
YIL133C	YBR031W	997
YIL133C	YBR048W	995
YIL133C	YBR084C-A	935
YIL133C	YBR118W	907
YIL133C	YBR181C	998
YIL133C	YBR189W	998
YIL133C	YBR191W	991
YIL133C	YCR031C	998
YIL133C	YDL014W	937
YIL133C	YDL061C	913
YIL133C	YDL075W	999
YIL133C	YDL081C	954
YIL133C	YDL082W	997
YIL133C	YDL083C	991
YIL133C	YDL130W	914
YIL133C	YDL136W	999
YIL133C	YDL191W	918
YIL133C	YDR012W	999
YIL133C	YDR060W	939
YIL133C	YDR064W	996
YIL133C	YDR382W	996
YIL133C	YDR385W	911
YIL133C	YDR418W	999
YIL133C	YDR447C	998
YIL133C	YDR450W	997
YIL133C	YDR471W	996
YIL133C	YDR496C	933
YIL133C	YDR500C	913
YIL133C	YEL054C	930

YIL133C	YER025W	915
YIL133C	YER056C-A	913
YIL133C	YER074W	995
YIL133C	YER102W	994
YIL133C	YER117W	925
YIL133C	YER131W	924
YIL133C	YFL034C-A	925
YIL133C	YFR031C-A	999
YIL133C	YFR032C-A	924
YIL133C	YGL030W	997
YIL133C	YGL031C	992
YIL133C	YGL076C	999
YIL133C	YGL103W	999
YIL133C	YGL123W	902
YIL133C	YGL135W	999
YIL133C	YGL147C	999
YIL133C	YGL189C	919
YIL133C	YGR027C	922
YIL133C	YGR034W	998
YIL133C	YGR085C	997
YIL133C	YGR118W	933
YIL133C	YGR148C	920
YIL133C	YGR162W	944
YIL133C	YGR214W	961
YIL133C	YHL001W	996
YIL133C	YHL015W	930
YIL133C	YHL033C	999
YIL133C	YHR010W	915
YIL133C	YHR021C	924
YIL133C	YHR141C	999
YIL133C	YHR203C	996
YIL133C	YIL018W	996
YIL133C	YIL052C	913
YIL133C	YIL069C	995
YIL133C	YIL148W	908
YIL133C	YJL136C	932
YIL133C	YJL177W	996
YIL133C	YJL189W	917
YIL133C	YJL190C	999
YIL133C	YJL191W	965
YIL133C	YJR007W	900
YIL133C	YJR094W-A	992
YIL133C	YJR123W	996
YIL133C	YJR145C	995

YIL133C	YKL006W	924
YIL133C	YKL156W	924
YIL133C	YKL180W	999
YIL133C	YKR057W	932
YIL133C	YKR094C	908
YIL133C	YLL045C	999
YIL133C	YLR029C	999
YIL133C	YLR048W	952
YIL133C	YLR061W	929
YIL133C	YLR075W	999
YIL133C	YLR167W	903
YIL133C	YLR185W	990
YIL133C	YLR197W	939
YIL133C	YLR264W	918
YIL133C	YLR287C-A	910
YIL133C	YLR325C	953
YIL133C	YLR333C	923
YIL133C	YLR340W	997
YIL133C	YLR344W	994
YIL133C	YLR367W	913
YIL133C	YLR388W	913
YIL133C	YLR406C	928
YIL133C	YLR441C	998
YIL133C	YLR448W	996
YIL133C	YML024W	925
YIL133C	YML026C	998
YIL133C	YML063W	996
YIL133C	YML073C	997
YIL133C	YMR121C	940
YIL133C	YMR142C	996
YIL133C	YMR194W	996
YIL133C	YMR230W	928
YIL133C	YMR242C	999
YIL133C	YMR260C	927
YIL133C	YMR290C	938
YIL133C	YNL067W	952
YIL133C	YNL069C	999
YIL133C	YNL096C	998
YIL133C	YNL132W	936
YIL133C	YNL162W	935
YIL133C	YNL178W	997
YIL133C	YNL301C	999
YIL133C	YNL302C	923
YIL133C	YOL039W	911

YIL133C	YOL040C	950
YIL133C	YOL041C	964
YIL133C	YOL077C	949
YIL133C	YOL120C	998
YIL133C	YOL121C	960
YIL133C	YOL127W	999
YIL133C	YOL139C	925
YIL133C	YOR063W	999
YIL133C	YOR096W	997
YIL133C	YOR133W	911
YIL133C	YOR167C	930
YIL133C	YOR182C	910
YIL133C	YOR204W	931
YIL133C	YOR206W	941
YIL133C	YOR234C	924
YIL133C	YOR293W	928
YIL133C	YOR312C	996
YIL133C	YOR369C	974
YIL133C	YPL079W	933
YIL133C	YPL081W	962
YIL133C	YPL131W	999
YIL133C	YPL143W	998
YIL133C	YPL198W	997
YIL133C	YPL220W	932
YIL133C	YPL237W	905
YIL133C	YPL249C-A	996
YIL133C	YPR043W	934
YIL133C	YPR080W	907
YIL133C	YPR102C	994
YIL133C	YPR132W	933
YIL133C	YPR163C	914
YIL135C	YDR309C	927
YIL135C	YHR061C	928
YIL138C	YCL037C	922
YIL138C	YNL079C	999
YIL138C	YOL076W	910
YIL139C	YDR092W	901
YIL139C	YDR419W	966
YIL139C	YOR346W	999
YIL139C	YPL167C	999
YIL140W	YCL014W	948
YIL140W	YCR038C	931
YIL140W	YGL054C	938
YIL140W	YJR092W	946

YIL140W	YJR143C	981
YIL140W	YLR229C	992
YIL140W	YLR353W	928
YIL142W	YDL143W	999
YIL142W	YDR188W	999
YIL142W	YDR212W	999
YIL142W	YEL003W	982
YIL142W	YFL037W	911
YIL142W	YGR078C	900
YIL142W	YJL008C	940
YIL142W	YJL014W	997
YIL142W	YJL111W	999
YIL142W	YJR064W	921
YIL142W	YLR105C	923
YIL142W	YLR200W	982
YIL142W	YML094W	900
YIL142W	YNL153C	907
YIL142W	YNL212W	987
YIL142W	YOR281C	999
YIL143C	YAR007C	989
YIL143C	YBR114W	908
YIL143C	YBR154C	915
YIL143C	YDL108W	923
YIL143C	YDL140C	944
YIL143C	YDR311W	994
YIL143C	YDR404C	923
YIL143C	YDR416W	900
YIL143C	YDR460W	995
YIL143C	YEL037C	988
YIL143C	YER148W	956
YIL143C	YER162C	991
YIL143C	YER171W	999
YIL143C	YGL043W	908
YIL143C	YGL070C	922
YIL143C	YGL112C	900
YIL143C	YGR094W	901
YIL143C	YGR258C	999
YIL143C	YIL021W	934
YIL143C	YIL128W	931
YIL143C	YJR035W	951
YIL143C	YJR052W	908
YIL143C	YKL028W	917
YIL143C	YKL058W	947
YIL143C	YLR005W	995

YIL143C	YML010W	902
YIL143C	YML095C	992
YIL143C	YMR201C	993
YIL143C	YMR236W	900
YIL143C	YMR250W	913
YIL143C	YNL312W	987
YIL143C	YOL005C	903
YIL143C	YOL051W	984
YIL143C	YOL135C	907
YIL143C	YOR027W	910
YIL143C	YOR151C	909
YIL143C	YOR168W	923
YIL143C	YOR194C	911
YIL143C	YOR210W	924
YIL143C	YOR224C	919
YIL143C	YOR341W	900
YIL143C	YOR352W	998
YIL143C	YPL022W	993
YIL143C	YPL122C	999
YIL143C	YPR010C	900
YIL143C	YPR025C	934
YIL143C	YPR056W	996
YIL143C	YPR086W	966
YIL143C	YPR110C	901
YIL143C	YPR187W	923
YIL144W	YAL034W-A	995
YIL144W	YDL028C	963
YIL144W	YDL239C	926
YIL144W	YDR016C	983
YIL144W	YDR201W	983
YIL144W	YDR318W	989
YIL144W	YER018C	999
YIL144W	YFL008W	903
YIL144W	YFR031C	902
YIL144W	YGL060W	989
YIL144W	YGL086W	918
YIL144W	YGL093W	942
YIL144W	YGR113W	994
YIL144W	YGR140W	991
YIL144W	YGR179C	937
YIL144W	YGR188C	933
YIL144W	YIR010W	982
YIL144W	YJL030W	924
YIL144W	YJR112W	962

YIL144W	YKL049C	925
YIL144W	YKL052C	982
YIL144W	YKR037C	989
YIL144W	YLR045C	961
YIL144W	YMR117C	999
YIL144W	YMR168C	954
YIL144W	YOL069W	999
YIL144W	YOR195W	902
YIL144W	YPL018W	997
YIL144W	YPL174C	948
YIL144W	YPL209C	928
YIL145C	YBR176W	984
YIL145C	YHR063C	955
YIL147C	YBL056W	976
YIL147C	YDL006W	976
YIL147C	YDL235C	999
YIL147C	YER118C	960
YIL147C	YHR206W	999
YIL147C	YKL198C	998
YIL147C	YLR006C	996
YIL147C	YLR082C	998
YIL147C	YLR113W	960
YIL147C	YOR208W	984
YIL148W	YAL035W	912
YIL148W	YBL027W	906
YIL148W	YBL072C	906
YIL148W	YBL087C	906
YIL148W	YBL092W	915
YIL148W	YBR031W	921
YIL148W	YBR048W	947
YIL148W	YBR082C	911
YIL148W	YBR084C-A	999
YIL148W	YBR118W	916
YIL148W	YBR181C	900
YIL148W	YBR189W	939
YIL148W	YBR191W	984
YIL148W	YCR031C	953
YIL148W	YDL061C	965
YIL148W	YDL075W	981
YIL148W	YDL081C	913
YIL148W	YDL082W	905
YIL148W	YDL130W	923
YIL148W	YDL136W	999
YIL148W	YDL191W	902

YIL148W	YDR012W	929
YIL148W	YDR064W	978
YIL148W	YDR382W	970
YIL148W	YDR385W	903
YIL148W	YDR418W	951
YIL148W	YDR447C	906
YIL148W	YDR450W	906
YIL148W	YDR471W	948
YIL148W	YDR500C	999
YIL148W	YEL054C	906
YIL148W	YER012W	900
YIL148W	YER056C-A	991
YIL148W	YER102W	999
YIL148W	YER117W	999
YIL148W	YER125W	995
YIL148W	YER131W	915
YIL148W	YFL034C-A	904
YIL148W	YFR004W	900
YIL148W	YFR031C-A	901
YIL148W	YFR032C-A	915
YIL148W	YFR050C	900
YIL148W	YGL030W	975
YIL148W	YGL048C	913
YIL148W	YGL076C	906
YIL148W	YGL103W	971
YIL148W	YGL123W	965
YIL148W	YGL135W	905
YIL148W	YGL147C	904
YIL148W	YGR027C	938
YIL148W	YGR034W	908
YIL148W	YGR085C	927
YIL148W	YGR118W	969
YIL148W	YGR148C	934
YIL148W	YGR214W	941
YIL148W	YHL001W	951
YIL148W	YHL015W	952
YIL148W	YHL033C	968
YIL148W	YHR010W	939
YIL148W	YHR021C	905
YIL148W	YHR027C	904
YIL148W	YHR141C	908
YIL148W	YHR200W	940
YIL148W	YHR203C	999
YIL148W	YIL018W	901

YIL148W	YIL052C	994
YIL148W	YIL133C	908
YIL148W	YJL136C	929
YIL148W	YJL177W	956
YIL148W	YJL189W	932
YIL148W	YJL190C	902
YIL148W	YJL191W	953
YIL148W	YJR094W-A	996
YIL148W	YJR123W	966
YIL148W	YJR145C	910
YIL148W	YKL006W	966
YIL148W	YKL156W	976
YIL148W	YKL180W	969
YIL148W	YKL210W	984
YIL148W	YKR057W	908
YIL148W	YKR059W	998
YIL148W	YLL039C	915
YIL148W	YLL045C	908
YIL148W	YLR029C	935
YIL148W	YLR048W	969
YIL148W	YLR061W	996
YIL148W	YLR075W	975
YIL148W	YLR167W	999
YIL148W	YLR185W	999
YIL148W	YLR264W	963
YIL148W	YLR340W	964
YIL148W	YLR344W	996
YIL148W	YLR367W	934
YIL148W	YLR388W	995
YIL148W	YLR406C	907
YIL148W	YLR441C	908
YIL148W	YLR448W	966
YIL148W	YML024W	997
YIL148W	YML026C	906
YIL148W	YML063W	956
YIL148W	YML073C	907
YIL148W	YMR142C	905
YIL148W	YMR194W	980
YIL148W	YMR230W	915
YIL148W	YMR242C	909
YIL148W	YMR260C	908
YIL148W	YMR276W	977
YIL148W	YNL067W	950
YIL148W	YNL069C	908

YIL148W	YNL096C	904
YIL148W	YNL162W	908
YIL148W	YNL178W	982
YIL148W	YNL301C	999
YIL148W	YNL302C	902
YIL148W	YOL038W	912
YIL148W	YOL039W	900
YIL148W	YOL040C	913
YIL148W	YOL120C	905
YIL148W	YOL121C	902
YIL148W	YOL127W	978
YIL148W	YOR063W	942
YIL148W	YOR096W	950
YIL148W	YOR133W	955
YIL148W	YOR167C	912
YIL148W	YOR234C	953
YIL148W	YOR293W	940
YIL148W	YOR312C	958
YIL148W	YOR369C	900
YIL148W	YPL079W	923
YIL148W	YPL081W	910
YIL148W	YPL090C	900
YIL148W	YPL131W	961
YIL148W	YPL143W	968
YIL148W	YPL198W	906
YIL148W	YPL220W	999
YIL148W	YPL249C-A	951
YIL148W	YPR043W	994
YIL148W	YPR080W	900
YIL148W	YPR102C	940
YIL148W	YPR103W	916
YIL148W	YPR132W	999
YIL149C	YDR381W	934
YIL149C	YKR095W	999
YIL149C	YPL169C	934
YIL150C	YBL023C	999
YIL150C	YBL035C	989
YIL150C	YBR060C	998
YIL150C	YBR088C	929
YIL150C	YBR160W	908
YIL150C	YBR202W	999
YIL150C	YDL017W	955
YIL150C	YDL042C	996
YIL150C	YEL032W	989

YIL150C	YGL201C	999
YIL150C	YJL194W	943
YIL150C	YKL045W	997
YIL150C	YLR103C	999
YIL150C	YLR274W	991
YIL150C	YLR442C	995
YIL150C	YML065W	988
YIL150C	YNL102W	999
YIL150C	YNL261W	987
YIL150C	YNL262W	979
YIL150C	YPR019W	993
YIL150C	YPR175W	979
YIL153W	YAL016W	967
YIL153W	YDL047W	999
YIL153W	YDL134C	982
YIL153W	YDR075W	996
YIL153W	YNL201C	998
YIL153W	YNL298W	961
YIL153W	YPL152W	999
YIL155C	YDL022W	989
YIL155C	YDL085W	983
YIL155C	YHL032C	999
YIL155C	YKL106W	902
YIL155C	YMR145C	942
YIL156W	YHL002W	933
YIL156W	YKL129C	935
YIL157C	YFL016C	963
YIL157C	YGR112W	985
YIL157C	YLR203C	997
YIL157C	YML129C	919
YIL157C	YPL172C	963
YIL159W	YCL027W	999
YIL159W	YER149C	999
YIL159W	YKL079W	907
YIL159W	YKL129C	935
YIL159W	YKR055W	967
YIL159W	YLR319C	954
YIL159W	YMR032W	998
YIL159W	YMR232W	998
YIL159W	YNL271C	999
YIL159W	YOR122C	999
YIL159W	YOR181W	955
YIL159W	YPL106C	946
YIL160C	YDR142C	999

YIL160C	YGL205W	941
YIL160C	YGR239C	967
YIL160C	YHR160C	994
YIL160C	YKR009C	918
YIL160C	YML126C	913
YIL160C	YPL028W	913
YIL161W	YNL023C	994
YIL162W	YDL194W	901
YIL162W	YDR477W	945
YIL162W	YGL035C	960
YIL162W	YGR282C	911
YIL162W	YJR090C	917
YIL162W	YLR025W	929
YIL164C	YIL165C	965
YIL165C	YIL164C	965
YIL166C	YPL240C	919
YIL169C	YGR014W	901
YIL169C	YHR211W	922
YIL169C	YIR019C	984
YIL169C	YMR317W	989
YIL169C	YOL155C	991
YIL177C	YBR126C	906
YIR001C	YER165W	908
YIR001C	YGL049C	999
YIR001C	YGR162W	999
YIR001C	YGR250C	999
YIR001C	YNL016W	991
YIR002C	YAR007C	990
YIR002C	YDR097C	984
YIR002C	YER142C	968
YIR002C	YJL092W	973
YIR002C	YKL114C	954
YIR002C	YMR190C	911
YIR002C	YMR201C	954
YIR002C	YPL167C	963
YIR003W	YCR088W	982
YIR003W	YDR388W	919
YIR003W	YER071C	999
YIR003W	YIL034C	999
YIR003W	YJL020C	965
YIR003W	YKL007W	999
YIR003W	YOR276W	962
YIR004W	YDR320C	978
YIR004W	YHR165C	976

YIR005W	YDL030W	977
YIR005W	YGL174W	999
YIR005W	YKL074C	963
YIR005W	YLR016C	999
YIR005W	YML049C	946
YIR005W	YMR240C	946
YIR005W	YMR288W	947
YIR005W	YOL012C	905
YIR005W	YOR319W	947
YIR005W	YPR094W	991
YIR006C	YBL007C	999
YIR006C	YBR234C	966
YIR006C	YDL029W	998
YIR006C	YDL161W	995
YIR006C	YER125W	903
YIR006C	YER133W	982
YIR006C	YFL039C	986
YIR006C	YGL181W	915
YIR006C	YGR241C	968
YIR006C	YHR161C	968
YIR006C	YIL095W	999
YIR006C	YJR065C	964
YIR006C	YNL020C	996
YIR006C	YNL084C	999
YIR006C	YNL243W	995
YIR006C	YOR181W	999
YIR006C	YOR329C	969
YIR008C	YAR007C	934
YIR008C	YBL023C	901
YIR008C	YBL035C	999
YIR008C	YBR087W	918
YIR008C	YBR088C	949
YIR008C	YBR202W	905
YIR008C	YDL102W	964
YIR008C	YEL032W	907
YIR008C	YGL201C	904
YIR008C	YGL207W	981
YIR008C	YHR164C	909
YIR008C	YJR006W	910
YIR008C	YJR043C	920
YIR008C	YJR068W	910
YIR008C	YKL045W	999
YIR008C	YKL113C	920
YIR008C	YLR274W	904

YIR008C	YLR288C	989
YIR008C	YML069W	981
YIR008C	YNL102W	999
YIR008C	YNL262W	998
YIR008C	YNL290W	908
YIR008C	YNL312W	984
YIR008C	YNR052C	989
YIR008C	YOL094C	909
YIR008C	YOR217W	909
YIR009W	YAL024C	927
YIR009W	YBR152W	937
YIR009W	YDL030W	999
YIR009W	YDR184C	927
YIR009W	YER029C	994
YIR009W	YML049C	972
YIR009W	YMR240C	970
YIR009W	YMR288W	972
YIR009W	YNL091W	945
YIR009W	YPL213W	999
YIR009W	YPR182W	949
YIR010W	YAL034W-A	999
YIR010W	YBR211C	906
YIR010W	YDR254W	906
YIR010W	YDR318W	976
YIR010W	YDR383C	902
YIR010W	YER018C	993
YIR010W	YGL093W	941
YIR010W	YGR179C	975
YIR010W	YGR188C	963
YIR010W	YIL144W	982
YIR010W	YJR112W	999
YIR010W	YJR135C	906
YIR010W	YKL089W	995
YIR010W	YLR381W	900
YIR010W	YMR117C	982
YIR010W	YOL069W	982
YIR010W	YPL018W	992
YIR010W	YPL233W	999
YIR011C	YFR004W	928
YIR011C	YGL044C	911
YIR011C	YMR061W	911
YIR012W	YLR075W	999
YIR015W	YAL033W	982
YIR015W	YBL018C	949

YIR015W	YBR167C	952
YIR015W	YBR257W	999
YIR015W	YDR478W	952
YIR015W	YGR030C	995
YIR015W	YHR062C	989
YIR015W	YLR145W	952
YIR015W	YNL221C	981
YIR015W	YNL282W	952
YIR017C	YDR253C	950
YIR017C	YJR060W	997
YIR017C	YNL103W	999
YIR017C	YPL038W	950
YIR018W	YLR220W	926
YIR019C	YAL063C	913
YIR019C	YAR050W	921
YIR019C	YBL113C	900
YIR019C	YBR083W	969
YIR019C	YCR089W	909
YIR019C	YDR477W	934
YIR019C	YGR014W	920
YIR019C	YHR084W	994
YIR019C	YHR211W	956
YIR019C	YIL169C	984
YIR019C	YKR102W	970
YIR019C	YMR317W	979
YIR019C	YNL327W	999
YIR019C	YOL155C	987
YIR021W	Q0115	996
YIR022W	YJR010C-A	999
YIR022W	YLR066W	999
YIR022W	YML055W	999
YIR023W	YBR006W	920
YIR023W	YBR208C	951
YIR023W	YDL170W	954
YIR023W	YDL210W	930
YIR023W	YER040W	961
YIR023W	YGR019W	932
YIR023W	YIR029W	946
YIR023W	YIR032C	949
YIR023W	YKR034W	963
YIR023W	YMR119W	914
YIR023W	YNL314W	995
YIR025W	YBL084C	999
YIR025W	YDL008W	982

YIR025W	YDR118W	999
YIR025W	YDR260C	999
YIR025W	YFR036W	998
YIR025W	YGL240W	999
YIR025W	YGR225W	999
YIR025W	YHL022C	976
YIR025W	YHR166C	999
YIR025W	YKL022C	999
YIR025W	YLR102C	998
YIR025W	YLR127C	999
YIR025W	YNL172W	999
YIR025W	YOR249C	999
YIR025W	YPR007C	914
YIR026C	YGR103W	926
YIR026C	YJR094C	954
YIR026C	YNL307C	955
YIR026C	YOR208W	966
YIR027C	YIR029W	998
YIR027C	YIR032C	965
YIR029W	YBR208C	944
YIR029W	YIR023W	946
YIR029W	YIR027C	998
YIR029W	YIR032C	999
YIR029W	YKR034W	939
YIR029W	YNL314W	948
YIR031C	YAL054C	907
YIR031C	YBL015W	909
YIR031C	YBR160W	906
YIR031C	YCR005C	930
YIR031C	YDL078C	900
YIR031C	YER065C	983
YIR031C	YIR032C	929
YIR031C	YKL029C	907
YIR031C	YKL085W	905
YIR031C	YLR153C	907
YIR031C	YNR001C	900
YIR031C	YPR006C	970
YIR032C	YBR126C	906
YIR032C	YBR208C	988
YIR032C	YER040W	987
YIR032C	YFL021W	904
YIR032C	YIR023W	949
YIR032C	YIR027C	965
YIR032C	YIR029W	999

YIR032C	YIR031C	929
YIR032C	YKR034W	989
YIR032C	YNL314W	952
YIR033W	YBR170C	991
YIR033W	YDL126C	919
YIR033W	YER125W	999
YIR033W	YGL055W	991
YIR033W	YGR048W	935
YIR033W	YKL020C	998
YIR033W	YMR264W	907
YIR034C	YGR267C	909
YIR034C	YNR050C	998
YIR035C	YNL232W	930
YIR037W	YBR216C	944
YIR037W	YBR244W	971
YIR037W	YCL043C	965
YIR037W	YER042W	995
YIR037W	YIR038C	919
YIR037W	YKL026C	970
YIR037W	YLR299W	901
YIR037W	YML007W	992
YIR037W	YOL049W	929
YIR037W	YPL091W	982
YIR037W	YPR035W	974
YIR038C	YIR037W	919
YIR038C	YLL060C	940
YIR038C	YPL091W	932
YJL001W	YBL041W	999
YJL001W	YBR173C	954
YJL001W	YDL097C	915
YJL001W	YDL147W	993
YJL001W	YDR427W	900
YJL001W	YER012W	999
YJL001W	YER021W	978
YJL001W	YER094C	999
YJL001W	YFL007W	999
YJL001W	YFR004W	922
YJL001W	YFR050C	999
YJL001W	YGL011C	999
YJL001W	YGL048C	950
YJL001W	YGR135W	999
YJL001W	YGR253C	999
YJL001W	YHL030W	924
YJL001W	YHR027C	927

YJL001W	YHR200W	914
YJL001W	YIL075C	946
YJL001W	YKL145W	920
YJL001W	YLL039C	970
YJL001W	YML092C	999
YJL001W	YMR314W	999
YJL001W	YOL038W	999
YJL001W	YOR157C	999
YJL001W	YOR362C	999
YJL001W	YPR103W	999
YJL001W	YPR108W	919
YJL001W	YPR180W	999
YJL002C	YBL105C	910
YJL002C	YCL001W	944
YJL002C	YDL232W	999
YJL002C	YEL002C	999
YJL002C	YER087C-B	978
YJL002C	YGL022W	999
YJL002C	YGL100W	962
YJL002C	YGL226C-A	999
YJL002C	YGR227W	984
YJL002C	YLR208W	986
YJL002C	YLR378C	984
YJL002C	YML019W	999
YJL002C	YML130C	984
YJL002C	YMR149W	999
YJL002C	YOR085W	999
YJL002C	YOR103C	999
YJL002C	YPL227C	956
YJL004C	YBR288C	947
YJL004C	YDR320C	915
YJL004C	YGL054C	919
YJL004C	YHL031C	953
YJL004C	YIL109C	990
YJL004C	YLR039C	969
YJL004C	YLR262C	999
YJL004C	YMR123W	919
YJL004C	YNL051W	918
YJL004C	YOL018C	944
YJL004C	YOR070C	988
YJL004C	YPL051W	949
YJL004C	YPR181C	976
YJL005W	YAL038W	940
YJL005W	YAL056W	964

YJL005W	YBR140C	987
YJL005W	YDR013W	962
YJL005W	YER020W	975
YJL005W	YFL039C	982
YJL005W	YGL248W	964
YJL005W	YGR233C	998
YJL005W	YHR205W	932
YJL005W	YIL033C	999
YJL005W	YJL164C	987
YJL005W	YLR310C	989
YJL005W	YNL098C	999
YJL005W	YNL138W	999
YJL005W	YOL081W	916
YJL005W	YOR101W	996
YJL005W	YOR360C	999
YJL005W	YOR371C	971
YJL006C	YKL139W	999
YJL006C	YML112W	999
YJL008C	YDL143W	996
YJL008C	YDR188W	936
YJL008C	YDR212W	932
YJL008C	YFL037W	958
YJL008C	YFL039C	916
YJL008C	YIL142W	940
YJL008C	YJL014W	986
YJL008C	YJL111W	911
YJL008C	YJR064W	920
YJL010C	YBR247C	985
YJL010C	YCL031C	983
YJL010C	YCL059C	911
YJL010C	YCR017C	999
YJL010C	YCR057C	985
YJL010C	YDL014W	983
YJL010C	YDL060W	986
YJL010C	YDL148C	910
YJL010C	YDR299W	986
YJL010C	YDR324C	988
YJL010C	YDR398W	983
YJL010C	YDR449C	985
YJL010C	YEL026W	906
YJL010C	YER082C	983
YJL010C	YGL120C	983
YJL010C	YGL171W	985
YJL010C	YGR090W	983

YJL010C	YGR128C	983
YJL010C	YGR145W	989
YJL010C	YHR196W	983
YJL010C	YJL069C	983
YJL010C	YJL109C	983
YJL010C	YJR002W	985
YJL010C	YKR060W	909
YJL010C	YLL011W	983
YJL010C	YLR175W	983
YJL010C	YLR197W	992
YJL010C	YLR222C	985
YJL010C	YLR409C	985
YJL010C	YMR093W	983
YJL010C	YMR229C	985
YJL010C	YMR290C	983
YJL010C	YNL132W	918
YJL010C	YOR310C	913
YJL010C	YPL012W	983
YJL010C	YPL126W	986
YJL010C	YPL217C	983
YJL010C	YPL266W	983
YJL010C	YPR112C	989
YJL010C	YPR137W	985
YJL010C	YPR144C	914
YJL011C	YBR154C	998
YJL011C	YDL150W	999
YJL011C	YDR005C	941
YJL011C	YDR045C	999
YJL011C	YGR246C	990
YJL011C	YHR143W-A	960
YJL011C	YKL144C	999
YJL011C	YKR025W	999
YJL011C	YNL039W	960
YJL011C	YNL113W	998
YJL011C	YNL151C	999
YJL011C	YNR003C	999
YJL011C	YOR116C	999
YJL011C	YOR207C	999
YJL011C	YOR210W	978
YJL011C	YOR224C	999
YJL011C	YPR110C	999
YJL011C	YPR187W	997
YJL011C	YPR190C	999
YJL012C	YER072W	918

YJL012C	YFR034C	911
YJL013C	YEL003W	915
YJL013C	YEL061C	981
YJL013C	YER016W	993
YJL013C	YFL037W	924
YJL013C	YGL086W	978
YJL013C	YGL116W	999
YJL013C	YGR188C	999
YJL013C	YJL030W	999
YJL013C	YML124C	905
YJL013C	YMR055C	984
YJL013C	YNL172W	926
YJL013C	YOR026W	999
YJL013C	YOR349W	964
YJL013C	YPL018W	959
YJL013C	YPL241C	930
YJL013C	YPR141C	955
YJL014W	YDL143W	999
YJL014W	YDR188W	997
YJL014W	YDR212W	998
YJL014W	YEL003W	982
YJL014W	YIL142W	997
YJL014W	YJL008C	986
YJL014W	YJL111W	984
YJL014W	YJR064W	982
YJL014W	YLR200W	982
YJL014W	YML094W	900
YJL019W	YFR027W	979
YJL019W	YGL075C	968
YJL019W	YJL073W	976
YJL019W	YKL042W	913
YJL019W	YLR233C	981
YJL019W	YMR001C	908
YJL019W	YOL012C	957
YJL019W	YOL104C	998
YJL019W	YOR257W	979
YJL019W	YPL200W	945
YJL020C	YBL007C	998
YJL020C	YDR129C	961
YJL020C	YDR388W	933
YJL020C	YGR028W	935
YJL020C	YHR114W	936
YJL020C	YIR003W	965
YJL020C	YJL146W	925

YJL020C	YKL129C	987
YJL020C	YLR337C	987
YJL020C	YMR109W	999
YJL020C	YOR181W	997
YJL024C	YBR288C	997
YJL024C	YDR320C	903
YJL024C	YFL025C	913
YJL024C	YGR261C	999
YJL024C	YPL195W	999
YJL025W	YBL014C	999
YJL025W	YER148W	993
YJL025W	YKL125W	940
YJL025W	YML043C	999
YJL025W	YMR270C	976
YJL026W	YBR089C-A	911
YJL026W	YCL035C	900
YJL026W	YCR083W	901
YJL026W	YDR513W	902
YJL026W	YER070W	999
YJL026W	YGR180C	999
YJL026W	YGR209C	991
YJL026W	YIL066C	999
YJL026W	YJL115W	938
YJL026W	YJR057W	928
YJL026W	YKL067W	904
YJL026W	YLR043C	989
YJL026W	YOR229W	980
YJL026W	YOR230W	999
YJL026W	YPL091W	990
YJL029C	YDR027C	999
YJL029C	YDR468C	913
YJL029C	YDR484W	999
YJL029C	YKR020W	997
YJL029C	YLR262C	980
YJL029C	YOL018C	912
YJL030W	YAL034W-A	979
YJL030W	YBL079W	917
YJL030W	YBL084C	991
YJL030W	YBR107C	998
YJL030W	YBR111W-A	908
YJL030W	YCL029C	939
YJL030W	YCR065W	984
YJL030W	YDL028C	998
YJL030W	YDR159W	917

YJL030W	YDR214W	900
YJL030W	YDR254W	998
YJL030W	YDR318W	999
YJL030W	YDR334W	906
YJL030W	YDR359C	936
YJL030W	YDR485C	916
YJL030W	YEL003W	931
YJL030W	YEL061C	999
YJL030W	YER007W	964
YJL030W	YER016W	994
YJL030W	YER105C	990
YJL030W	YER107C	915
YJL030W	YFL037W	942
YJL030W	YGL003C	953
YJL030W	YGL060W	991
YJL030W	YGL061C	979
YJL030W	YGL086W	999
YJL030W	YGL116W	999
YJL030W	YGL216W	983
YJL030W	YGR113W	979
YJL030W	YGR140W	911
YJL030W	YGR179C	926
YJL030W	YGR188C	999
YJL030W	YHR191C	967
YJL030W	YIL144W	924
YJL030W	YJL013C	999
YJL030W	YJR053W	992
YJL030W	YJR135C	998
YJL030W	YKL022C	995
YJL030W	YKR037C	983
YJL030W	YKR082W	959
YJL030W	YLR045C	990
YJL030W	YLR381W	998
YJL030W	YML041C	907
YJL030W	YML094W	961
YJL030W	YML124C	952
YJL030W	YMR055C	999
YJL030W	YMR078C	963
YJL030W	YMR094W	998
YJL030W	YMR153W	936
YJL030W	YMR168C	984
YJL030W	YNL102W	976
YJL030W	YNL107W	921
YJL030W	YOL069W	928

YJL030W	YOL072W	917
YJL030W	YOR026W	999
YJL030W	YOR195W	997
YJL030W	YOR349W	995
YJL030W	YPL008W	966
YJL030W	YPL018W	999
YJL030W	YPL209C	924
YJL030W	YPL240C	919
YJL030W	YPL241C	991
YJL030W	YPR046W	999
YJL030W	YPR141C	992
YJL030W	YPR176C	998
YJL031C	YOR370C	920
YJL031C	YPR176C	999
YJL033W	YAL043C	961
YJL033W	YDL014W	999
YJL033W	YDR195W	960
YJL033W	YDR299W	993
YJL033W	YDR365C	988
YJL033W	YER165W	949
YJL033W	YGR145W	981
YJL033W	YGR156W	959
YJL033W	YHR196W	940
YJL033W	YHR203C	972
YJL033W	YJR093C	951
YJL033W	YKR002W	961
YJL033W	YLR115W	961
YJL033W	YLR197W	949
YJL033W	YLR277C	961
YJL033W	YMR061W	967
YJL033W	YMR290C	999
YJL033W	YNL016W	906
YJL033W	YNL317W	977
YJL033W	YOR310C	951
YJL034W	YAL005C	905
YJL034W	YAL058W	933
YJL034W	YBL075C	906
YJL034W	YBR171W	994
YJL034W	YBR283C	920
YJL034W	YDL066W	958
YJL034W	YDL229W	907
YJL034W	YDR057W	991
YJL034W	YDR086C	921
YJL034W	YER103W	905

YJL034W	YFL031W	956
YJL034W	YFR041C	917
YJL034W	YHR079C	999
YJL034W	YJL073W	998
YJL034W	YJR010C-A	923
YJL034W	YKL073W	999
YJL034W	YLL024C	921
YJL034W	YLR207W	995
YJL034W	YLR292C	984
YJL034W	YLR378C	974
YJL034W	YML055W	923
YJL034W	YMR200W	976
YJL034W	YMR214W	999
YJL034W	YNL064C	902
YJL034W	YNL209W	907
YJL034W	YOL031C	999
YJL034W	YOR232W	934
YJL034W	YOR254C	999
YJL034W	YPL094C	919
YJL034W	YPL227C	909
YJL034W	YPR159W	968
YJL035C	YLR316C	984
YJL036W	YAL030W	987
YJL036W	YDL113C	999
YJL036W	YDR425W	999
YJL036W	YGR142W	952
YJL036W	YLR423C	993
YJL036W	YOR132W	966
YJL039C	YDL207W	962
YJL039C	YDR159W	966
YJL039C	YER107C	964
YJL039C	YER110C	915
YJL039C	YFR002W	998
YJL039C	YGL172W	949
YJL039C	YLR335W	957
YJL039C	YMR047C	962
YJL039C	YMR153W	900
YJL039C	YOR257W	961
YJL039C	YPL169C	966
YJL041W	YBL079W	941
YJL041W	YDL116W	991
YJL041W	YDL207W	962
YJL041W	YDR116C	930
YJL041W	YDR159W	999

YJL041W	YDR322W	915
YJL041W	YDR424C	978
YJL041W	YER009W	976
YJL041W	YER105C	942
YJL041W	YER107C	995
YJL041W	YER110C	980
YJL041W	YFR002W	999
YJL041W	YGL092W	984
YJL041W	YGL100W	961
YJL041W	YGL172W	999
YJL041W	YGR119C	999
YJL041W	YIL115C	999
YJL041W	YJL061W	999
YJL041W	YJL063C	926
YJL041W	YJR042W	961
YJL041W	YKL057C	961
YJL041W	YKL186C	989
YJL041W	YKL205W	976
YJL041W	YKR082W	959
YJL041W	YLR208W	953
YJL041W	YLR293C	992
YJL041W	YLR312W-A	920
YJL041W	YLR335W	999
YJL041W	YLR347C	999
YJL041W	YML025C	912
YJL041W	YML103C	942
YJL041W	YMR024W	902
YJL041W	YMR047C	999
YJL041W	YMR129W	941
YJL041W	YMR193W	934
YJL041W	YMR308C	982
YJL041W	YOR098C	997
YJL041W	YOR257W	961
YJL041W	YPL169C	996
YJL042W	YER133W	987
YJL042W	YFL037W	904
YJL044C	YDR456W	979
YJL044C	YLR262C	998
YJL045W	YDR178W	950
YJL045W	YGR244C	957
YJL045W	YKL141W	973
YJL045W	YKL148C	997
YJL045W	YLL041C	999
YJL045W	YLR164W	912

YJL045W	YMR118C	927
YJL045W	YOR142W	950
YJL045W	YPL262W	900
YJL046W	YLR239C	918
YJL046W	YOR196C	938
YJL047C	YDL132W	999
YJL047C	YDR054C	998
YJL047C	YDR139C	910
YJL047C	YDR217C	975
YJL047C	YFR050C	998
YJL047C	YLR320W	997
YJL047C	YNR034W	998
YJL047C	YOL063C	997
YJL047C	YOL133W	997
YJL047C	YPR164W	974
YJL048C	YDL126C	975
YJL048C	YKL130C	900
YJL048C	YKL213C	912
YJL050W	YBR142W	919
YJL050W	YDL031W	918
YJL050W	YDL175C	999
YJL050W	YER165W	931
YJL050W	YHR069C	943
YJL050W	YIL079C	997
YJL050W	YLR432W	940
YJL050W	YNL062C	923
YJL050W	YNL299W	999
YJL050W	YOL115W	999
YJL050W	YOR001W	999
YJL050W	YOR204W	935
YJL050W	YPL190C	997
YJL052W	YCR012W	996
YJL052W	YDR050C	989
YJL052W	YJR009C	999
YJL052W	YKL060C	940
YJL053W	YBL017C	993
YJL053W	YHR012W	999
YJL053W	YJL154C	999
YJL053W	YOR069W	999
YJL053W	YOR132W	999
YJL054W	YBR091C	999
YJL054W	YDL217C	999
YJL054W	YHR005C-A	960
YJL054W	YNR017W	957

YJL054W	YOR297C	999
YJL054W	YPR024W	900
YJL056C	YDR492W	931
YJL056C	YGL255W	997
YJL056C	YHL027W	904
YJL056C	YLR023C	931
YJL056C	YLR130C	981
YJL056C	YOL002C	911
YJL056C	YOL101C	925
YJL058C	YER093C	977
YJL058C	YIL105C	980
YJL058C	YKL203C	993
YJL058C	YMR068W	994
YJL058C	YNL006W	993
YJL058C	YNL047C	980
YJL058C	YOL078W	959
YJL059W	YBR086C	908
YJL059W	YCR021C	978
YJL059W	YEL063C	957
YJL059W	YGR142W	998
YJL060W	YLR314C	959
YJL061W	YBL079W	989
YJL061W	YDL088C	941
YJL061W	YDL116W	949
YJL061W	YDL207W	996
YJL061W	YDR159W	972
YJL061W	YDR192C	939
YJL061W	YDR335W	984
YJL061W	YDR424C	978
YJL061W	YER105C	984
YJL061W	YER107C	999
YJL061W	YFR002W	990
YJL061W	YGL092W	934
YJL061W	YGL172W	988
YJL061W	YGR119C	991
YJL061W	YIL115C	999
YJL061W	YJL041W	999
YJL061W	YJR042W	951
YJL061W	YKL057C	998
YJL061W	YKL068W	986
YJL061W	YKR082W	961
YJL061W	YLR018C	988
YJL061W	YLR347C	947
YJL061W	YLR423C	988

YJL061W	YMR047C	999
YJL061W	YMR129W	957
YJL061W	YOR098C	999
YJL061W	YOR257W	961
YJL061W	YPL169C	966
YJL062W	YBR078W	996
YJL062W	YDR302W	951
YJL062W	YDR331W	927
YJL062W	YEL031W	943
YJL062W	YER118C	921
YJL062W	YFL025C	921
YJL062W	YLL031C	982
YJL062W	YLR242C	923
YJL062W	YNL169C	978
YJL062W	YOR067C	921
YJL063C	YBL038W	999
YJL063C	YBR122C	972
YJL063C	YBR146W	946
YJL063C	YCR046C	992
YJL063C	YCR071C	987
YJL063C	YDR116C	996
YJL063C	YDR237W	998
YJL063C	YDR296W	991
YJL063C	YDR322W	974
YJL063C	YDR462W	972
YJL063C	YEL050C	955
YJL063C	YGL068W	999
YJL063C	YGR220C	999
YJL063C	YHL004W	948
YJL063C	YHR147C	981
YJL063C	YJL041W	926
YJL063C	YKL167C	972
YJL063C	YKL170W	952
YJL063C	YKR006C	972
YJL063C	YKR085C	991
YJL063C	YLR312W-A	972
YJL063C	YLR439W	978
YJL063C	YML025C	997
YJL063C	YMR024W	972
YJL063C	YMR193W	972
YJL063C	YNL005C	992
YJL063C	YNL081C	981
YJL063C	YNL185C	964
YJL063C	YNL252C	974

YJL063C	YNL284C	951
YJL063C	YNR036C	940
YJL063C	YNR037C	936
YJL063C	YOR150W	999
YJL065C	YDR121W	999
YJL065C	YGL133W	999
YJL065C	YOR304W	999
YJL066C	YMR203W	980
YJL068C	YDL168W	997
YJL068C	YOR388C	985
YJL069C	YBL004W	954
YJL069C	YCL059C	952
YJL069C	YCR057C	999
YJL069C	YDL014W	926
YJL069C	YDR299W	931
YJL069C	YDR324C	999
YJL069C	YDR398W	909
YJL069C	YDR449C	999
YJL069C	YGL019W	933
YJL069C	YGL120C	910
YJL069C	YGL171W	911
YJL069C	YGR090W	999
YJL069C	YGR128C	985
YJL069C	YHR148W	996
YJL069C	YHR169W	921
YJL069C	YIL035C	908
YJL069C	YJL010C	983
YJL069C	YJL109C	999
YJL069C	YJR002W	991
YJL069C	YKL099C	953
YJL069C	YKR060W	953
YJL069C	YLL011W	915
YJL069C	YLR129W	999
YJL069C	YLR175W	911
YJL069C	YLR186W	956
YJL069C	YLR197W	928
YJL069C	YLR222C	999
YJL069C	YLR409C	999
YJL069C	YML093W	961
YJL069C	YMR093W	999
YJL069C	YMR229C	941
YJL069C	YMR290C	918
YJL069C	YNL075W	914
YJL069C	YNL132W	928

YJL069C	YOR004W	917
YJL069C	YOR039W	933
YJL069C	YOR059C	928
YJL069C	YOR061W	929
YJL069C	YOR078W	975
YJL069C	YOR145C	962
YJL069C	YPL126W	922
YJL069C	YPL217C	922
YJL070C	YML035C	999
YJL071W	YER069W	992
YJL072C	YDR013W	999
YJL072C	YDR489W	999
YJL072C	YGL113W	947
YJL072C	YJL090C	935
YJL072C	YLR103C	935
YJL072C	YOL146W	999
YJL073W	YJL019W	976
YJL073W	YJL034W	998
YJL073W	YMR214W	998
YJL074C	YDL003W	999
YJL074C	YDR014W	924
YJL074C	YDR180W	989
YJL074C	YFL008W	999
YJL074C	YFR027W	955
YJL074C	YGR098C	928
YJL074C	YIL026C	999
YJL074C	YMR001C	991
YJL074C	YPR007C	988
YJL076W	YAR019C	991
YJL076W	YDL042C	999
YJL076W	YDR110W	999
YJL076W	YDR174W	954
YJL076W	YDR439W	911
YJL076W	YFR028C	999
YJL076W	YGR092W	999
YJL076W	YGR208W	935
YJL076W	YHR152W	927
YJL076W	YIL106W	981
YJL076W	YML064C	997
YJL076W	YMR001C	998
YJL076W	YOR341W	954
YJL076W	YPR010C	954
YJL076W	YPR111W	982
YJL076W	YPR119W	989

YJL080C	YER165W	986
YJL080C	YGR285C	999
YJL080C	YHR005C	975
YJL080C	YJR123W	978
YJL080C	YKL204W	996
YJL080C	YMR116C	997
YJL080C	YOR198C	999
YJL081C	YAL011W	999
YJL081C	YBR009C	920
YJL081C	YBR231C	999
YJL081C	YDL002C	999
YJL081C	YDR190C	999
YJL081C	YDR225W	994
YJL081C	YDR334W	999
YJL081C	YDR359C	999
YJL081C	YDR485C	998
YJL081C	YEL018W	999
YJL081C	YER092W	986
YJL081C	YFL013C	997
YJL081C	YFL024C	999
YJL081C	YFL039C	999
YJL081C	YGL150C	999
YJL081C	YGR002C	999
YJL081C	YHR090C	999
YJL081C	YHR099W	999
YJL081C	YLR052W	988
YJL081C	YLR085C	999
YJL081C	YLR385C	976
YJL081C	YLR399C	984
YJL081C	YML041C	993
YJL081C	YNL059C	999
YJL081C	YNL107W	999
YJL081C	YNL136W	998
YJL081C	YNL215W	991
YJL081C	YOL012C	999
YJL081C	YOR141C	999
YJL081C	YOR244W	999
YJL081C	YPL129W	999
YJL081C	YPL235W	999
YJL081C	YPR023C	999
YJL083W	YIL002C	965
YJL084C	YER059W	952
YJL084C	YER125W	998
YJL084C	YIL050W	987

YJL084C	YOR348C	944
YJL084C	YPL031C	921
YJL085W	YBR102C	999
YJL085W	YDR164C	994
YJL085W	YDR166C	999
YJL085W	YER008C	999
YJL085W	YFL005W	995
YJL085W	YGL233W	998
YJL085W	YIL068C	999
YJL085W	YIL118W	999
YJL085W	YLR166C	999
YJL085W	YPR032W	966
YJL085W	YPR055W	999
YJL087C	YKL108W	901
YJL087C	YLR105C	934
YJL088W	YHR018C	983
YJL088W	YJL130C	926
YJL088W	YJR109C	975
YJL088W	YKL184W	920
YJL088W	YLR438W	996
YJL088W	YMR062C	993
YJL088W	YOL058W	999
YJL088W	YOL140W	997
YJL088W	YOR130C	928
YJL088W	YOR303W	986
YJL088W	YPL111W	987
YJL089W	YDR422C	957
YJL089W	YDR477W	999
YJL089W	YER027C	999
YJL089W	YGL035C	942
YJL089W	YGL115W	907
YJL089W	YMR280C	985
YJL090C	YBR136W	919
YJL090C	YBR278W	945
YJL090C	YDR013W	989
YJL090C	YDR121W	900
YJL090C	YDR489W	990
YJL090C	YGL113W	999
YJL090C	YJL072C	935
YJL090C	YKL108W	999
YJL090C	YLR103C	992
YJL090C	YNL262W	999
YJL090C	YOL146W	957
YJL090C	YPL194W	952

YJL090C	YPR175W	998
YJL091C	YOL020W	909
YJL092W	YBR088C	999
YJL092W	YBR098W	991
YJL092W	YBR136W	975
YJL092W	YCL016C	946
YJL092W	YCL061C	998
YJL092W	YCR066W	999
YJL092W	YDL059C	946
YJL092W	YDL064W	986
YJL092W	YDR004W	997
YJL092W	YDR076W	931
YJL092W	YDR092W	973
YJL092W	YDR363W	956
YJL092W	YDR369C	993
YJL092W	YDR386W	995
YJL092W	YDR419W	957
YJL092W	YDR510W	996
YJL092W	YER095W	999
YJL092W	YGL058W	999
YJL092W	YGL087C	976
YJL092W	YGL133W	962
YJL092W	YGL163C	999
YJL092W	YHR031C	999
YJL092W	YHR154W	947
YJL092W	YHR191C	914
YJL092W	YIR002C	973
YJL092W	YJR043C	999
YJL092W	YKL113C	999
YJL092W	YKR082W	968
YJL092W	YLR032W	999
YJL092W	YLR234W	999
YJL092W	YLR320W	953
YJL092W	YML032C	999
YJL092W	YMR048W	971
YJL092W	YMR078C	955
YJL092W	YMR190C	999
YJL092W	YMR224C	959
YJL092W	YNL218W	942
YJL092W	YNL250W	998
YJL092W	YNL273W	956
YJL092W	YOR144C	998
YJL092W	YOR355W	954
YJL092W	YPL020C	971

YJL092W	YPL022W	986
YJL092W	YPL024W	990
YJL092W	YPL167C	987
YJL092W	YPR135W	929
YJL092W	YPR164W	961
YJL093C	YJL129C	969
YJL093C	YKR050W	969
YJL093C	YLR113W	921
YJL094C	YDR456W	944
YJL094C	YLR138W	957
YJL095W	YBL105C	999
YJL095W	YDR162C	952
YJL095W	YDR293C	913
YJL095W	YGR229C	939
YJL095W	YHR030C	999
YJL095W	YIL122W	909
YJL095W	YLL021W	989
YJL095W	YLR371W	951
YJL095W	YMR307W	924
YJL095W	YNL271C	960
YJL095W	YOR008C	945
YJL095W	YOR231W	999
YJL095W	YOR360C	913
YJL095W	YPL140C	996
YJL098W	YDL047W	999
YJL098W	YFR040W	976
YJL098W	YGL197W	992
YJL098W	YGR161C	995
YJL098W	YKR028W	984
YJL098W	YLR310C	991
YJL098W	YOR048C	966
YJL098W	YOR204W	927
YJL098W	YOR267C	987
YJL099W	YBR023C	998
YJL099W	YDL192W	996
YJL099W	YGR229C	922
YJL099W	YHR142W	932
YJL099W	YKR027W	996
YJL099W	YLR330W	999
YJL099W	YMR237W	999
YJL099W	YOR299W	999
YJL100W	YKL212W	936
YJL100W	YLR305C	991
YJL100W	YNL267W	934

YJL100W	YOR181W	998
YJL101C	YDR111C	907
YJL101C	YLR027C	901
YJL101C	YLR089C	904
YJL101C	YLR299W	928
YJL101C	YLR303W	943
YJL101C	YNL103W	979
YJL101C	YOL049W	997
YJL101C	YOR323C	976
YJL101C	YPL061W	942
YJL101C	YPR035W	936
YJL104W	YIL022W	999
YJL104W	YJL143W	999
YJL104W	YKR065C	940
YJL104W	YLR008C	999
YJL104W	YNL328C	984
YJL104W	YNR017W	999
YJL104W	YPL063W	997
YJL106W	YDR155C	954
YJL106W	YDR207C	967
YJL106W	YDR310C	980
YJL106W	YER020W	976
YJL106W	YHL024W	921
YJL106W	YHR124W	999
YJL106W	YIL126W	985
YJL106W	YJL146W	908
YJL106W	YJR094C	995
YJL106W	YLR079W	996
YJL106W	YNL312W	982
YJL107C	YJL108C	955
YJL108C	YJL107C	955
YJL109C	YBL004W	999
YJL109C	YBR247C	983
YJL109C	YCL059C	997
YJL109C	YCR057C	999
YJL109C	YDL014W	998
YJL109C	YDL148C	997
YJL109C	YDL153C	912
YJL109C	YDR280W	926
YJL109C	YDR299W	931
YJL109C	YDR324C	999
YJL109C	YDR398W	999
YJL109C	YDR449C	999
YJL109C	YEL055C	982

YJL109C	YER082C	999
YJL109C	YGL120C	966
YJL109C	YGL171W	911
YJL109C	YGR090W	999
YJL109C	YGR128C	999
YJL109C	YHR148W	999
YJL109C	YHR169W	917
YJL109C	YHR196W	999
YJL109C	YJL010C	983
YJL109C	YJL069C	999
YJL109C	YJR002W	999
YJL109C	YLL011W	999
YJL109C	YLR129W	998
YJL109C	YLR175W	930
YJL109C	YLR186W	999
YJL109C	YLR197W	999
YJL109C	YLR222C	999
YJL109C	YLR409C	999
YJL109C	YML093W	911
YJL109C	YMR093W	999
YJL109C	YMR128W	997
YJL109C	YMR229C	989
YJL109C	YMR290C	986
YJL109C	YNL075W	951
YJL109C	YNL132W	996
YJL109C	YOL010W	987
YJL109C	YOR004W	917
YJL109C	YOR078W	990
YJL109C	YOR145C	916
YJL109C	YOR310C	986
YJL109C	YPL012W	998
YJL109C	YPL126W	999
YJL109C	YPL217C	999
YJL109C	YPL266W	956
YJL109C	YPR112C	915
YJL109C	YPR137W	999
YJL109C	YPR144C	999
YJL110C	YKR039W	971
YJL110C	YNL021W	977
YJL110C	YNL229C	979
YJL111W	YDL143W	999
YJL111W	YDR188W	999
YJL111W	YDR212W	924
YJL111W	YFL037W	915

YJL111W	YFL039C	922
YJL111W	YGR187C	921
YJL111W	YIL142W	999
YJL111W	YJL008C	911
YJL111W	YJL014W	984
YJL111W	YJR064W	924
YJL111W	YMR028W	994
YJL111W	YOR281C	999
YJL112W	YBR179C	999
YJL112W	YIL065C	999
YJL112W	YLL001W	999
YJL115W	YBL008W	999
YJL115W	YBL088C	914
YJL115W	YBR009C	999
YJL115W	YBR010W	999
YJL115W	YBR087W	995
YJL115W	YBR088C	923
YJL115W	YBR136W	995
YJL115W	YBR195C	983
YJL115W	YBR215W	916
YJL115W	YCL061C	915
YJL115W	YDR181C	998
YJL115W	YDR217C	974
YJL115W	YER110C	907
YJL115W	YER173W	976
YJL115W	YGR180C	975
YJL115W	YGR252W	952
YJL115W	YIL070C	935
YJL115W	YJL026W	938
YJL115W	YJR140C	997
YJL115W	YKR101W	995
YJL115W	YLL002W	999
YJL115W	YLR399C	932
YJL115W	YML032C	975
YJL115W	YML102W	999
YJL115W	YMR127C	985
YJL115W	YMR190C	999
YJL115W	YNL030W	999
YJL115W	YNL031C	999
YJL115W	YNL102W	918
YJL115W	YNR052C	936
YJL115W	YOL012C	978
YJL115W	YOR038C	998
YJL115W	YOR213C	987

YJL115W	YPL153C	999
YJL115W	YPR018W	999
YJL117W	YBR106W	962
YJL117W	YCR037C	998
YJL117W	YDL222C	921
YJL117W	YML123C	994
YJL117W	YMR123W	921
YJL117W	YMR134W	998
YJL121C	YBR117C	975
YJL121C	YGR256W	919
YJL121C	YHR183W	965
YJL121C	YOR095C	995
YJL121C	YPR074C	994
YJL122W	YAL035W	964
YJL122W	YBR267W	971
YJL122W	YDR101C	999
YJL122W	YHR052W	988
YJL122W	YLR009W	957
YJL122W	YMR308C	925
YJL122W	YNL227C	936
YJL122W	YPR016C	931
YJL123C	YPL240C	919
YJL124C	YBL026W	999
YJL124C	YBR055C	974
YJL124C	YCR020C-A	984
YJL124C	YCR077C	999
YJL124C	YDL160C	999
YJL124C	YDL184C	969
YJL124C	YDR037W	931
YJL124C	YDR378C	999
YJL124C	YDR473C	979
YJL124C	YEL015W	955
YJL124C	YER112W	999
YJL124C	YER146W	999
YJL124C	YER165W	944
YJL124C	YER172C	976
YJL124C	YFL017W-A	904
YJL124C	YGL173C	999
YJL124C	YGL213C	908
YJL124C	YGR074W	921
YJL124C	YGR091W	975
YJL124C	YHR165C	981
YJL124C	YJR022W	999
YJL124C	YKL173W	991

YJL124C	YLR147C	998
YJL124C	YLR275W	998
YJL124C	YLR438C-A	999
YJL124C	YMR268C	959
YJL124C	YNL118C	997
YJL124C	YNL145W	976
YJL124C	YNL147W	999
YJL124C	YOL149W	991
YJL124C	YOR159C	905
YJL124C	YOR308C	976
YJL124C	YOR375C	961
YJL124C	YPL157W	950
YJL124C	YPR082C	974
YJL124C	YPR178W	976
YJL124C	YPR182W	989
YJL125C	YDL051W	929
YJL125C	YNL062C	999
YJL127C	YAL021C	999
YJL127C	YDR224C	934
YJL127C	YDR225W	984
YJL127C	YMR179W	957
YJL127C	YMR247C	903
YJL128C	YBL016W	922
YJL128C	YCR073C	923
YJL128C	YDL006W	990
YJL128C	YDL159W	918
YJL128C	YDL235C	936
YJL128C	YDR162C	998
YJL128C	YER118C	999
YJL128C	YGR040W	987
YJL128C	YHR030C	905
YJL128C	YLR113W	997
YJL128C	YLR248W	996
YJL128C	YLR336C	906
YJL128C	YLR362W	999
YJL128C	YNL053W	908
YJL128C	YNR031C	999
YJL128C	YOR127W	914
YJL128C	YOR208W	978
YJL129C	YDR345C	967
YJL129C	YHR094C	967
YJL129C	YJL093C	969
YJL129C	YKR050W	999
YJL129C	YML016C	976

YJL129C	YNL330C	976
YJL129C	YOL004W	996
YJL130C	YDR321W	917
YJL130C	YJL088W	926
YJL130C	YJR109C	971
YJL130C	YKL106W	917
YJL130C	YKL216W	978
YJL130C	YLL018C	914
YJL130C	YLR420W	986
YJL130C	YMR217W	911
YJL130C	YMR300C	916
YJL130C	YNL220W	904
YJL130C	YOL058W	928
YJL130C	YOR303W	993
YJL130C	YPR035W	929
YJL133W	YDL120W	992
YJL133W	YKR071C	916
YJL133W	YLR220W	912
YJL133W	YOR334W	985
YJL134W	YDR294C	999
YJL134W	YDR297W	999
YJL134W	YDR346C	922
YJL134W	YKR053C	969
YJL134W	YLR260W	970
YJL134W	YMR296C	976
YJL134W	YOR061W	904
YJL134W	YOR171C	995
YJL136C	YBL027W	922
YJL136C	YBL072C	929
YJL136C	YBL087C	931
YJL136C	YBL092W	953
YJL136C	YBR031W	953
YJL136C	YBR048W	914
YJL136C	YBR084C-A	940
YJL136C	YBR118W	910
YJL136C	YBR181C	906
YJL136C	YBR189W	970
YJL136C	YBR191W	980
YJL136C	YCR031C	959
YJL136C	YDL061C	960
YJL136C	YDL075W	968
YJL136C	YDL081C	943
YJL136C	YDL082W	937
YJL136C	YDL130W	929

YJL136C	YDL136W	940
YJL136C	YDL191W	930
YJL136C	YDR012W	954
YJL136C	YDR025W	914
YJL136C	YDR064W	985
YJL136C	YDR382W	981
YJL136C	YDR385W	911
YJL136C	YDR418W	974
YJL136C	YDR447C	936
YJL136C	YDR450W	923
YJL136C	YDR471W	943
YJL136C	YDR500C	936
YJL136C	YEL054C	932
YJL136C	YER025W	916
YJL136C	YER056C-A	965
YJL136C	YER074W	922
YJL136C	YER102W	943
YJL136C	YER117W	954
YJL136C	YER131W	935
YJL136C	YER165W	902
YJL136C	YFL034C-A	920
YJL136C	YFR031C-A	912
YJL136C	YFR032C-A	948
YJL136C	YGL030W	981
YJL136C	YGL031C	934
YJL136C	YGL076C	954
YJL136C	YGL103W	967
YJL136C	YGL123W	955
YJL136C	YGL135W	929
YJL136C	YGL147C	933
YJL136C	YGL189C	921
YJL136C	YGR027C	974
YJL136C	YGR034W	931
YJL136C	YGR085C	981
YJL136C	YGR118W	974
YJL136C	YGR148C	979
YJL136C	YGR214W	975
YJL136C	YHL001W	982
YJL136C	YHL015W	967
YJL136C	YHL033C	981
YJL136C	YHR010W	951
YJL136C	YHR021C	937
YJL136C	YHR141C	948
YJL136C	YHR203C	942

YJL136C	YIL018W	912
YJL136C	YIL052C	976
YJL136C	YIL069C	922
YJL136C	YIL133C	932
YJL136C	YIL148W	929
YJL136C	YJL177W	952
YJL136C	YJL189W	943
YJL136C	YJL190C	930
YJL136C	YJL191W	963
YJL136C	YJR007W	900
YJL136C	YJR094W-A	975
YJL136C	YJR123W	979
YJL136C	YJR145C	930
YJL136C	YKL006W	982
YJL136C	YKL156W	969
YJL136C	YKL180W	973
YJL136C	YKR057W	984
YJL136C	YKR059W	916
YJL136C	YKR094C	908
YJL136C	YLL045C	948
YJL136C	YLR029C	941
YJL136C	YLR048W	982
YJL136C	YLR061W	974
YJL136C	YLR075W	973
YJL136C	YLR167W	925
YJL136C	YLR185W	936
YJL136C	YLR264W	961
YJL136C	YLR287C-A	909
YJL136C	YLR325C	930
YJL136C	YLR333C	938
YJL136C	YLR340W	972
YJL136C	YLR344W	974
YJL136C	YLR367W	949
YJL136C	YLR388W	977
YJL136C	YLR406C	927
YJL136C	YLR441C	932
YJL136C	YLR448W	978
YJL136C	YML024W	981
YJL136C	YML026C	923
YJL136C	YML063W	964
YJL136C	YML073C	929
YJL136C	YMR121C	918
YJL136C	YMR142C	942
YJL136C	YMR194W	973

YJL136C	YMR230W	950
YJL136C	YMR242C	934
YJL136C	YMR260C	912
YJL136C	YNL067W	961
YJL136C	YNL069C	931
YJL136C	YNL096C	935
YJL136C	YNL162W	937
YJL136C	YNL178W	988
YJL136C	YNL301C	935
YJL136C	YNL302C	926
YJL136C	YOL039W	929
YJL136C	YOL040C	956
YJL136C	YOL120C	922
YJL136C	YOL121C	926
YJL136C	YOL127W	973
YJL136C	YOR063W	951
YJL136C	YOR096W	968
YJL136C	YOR133W	911
YJL136C	YOR167C	943
YJL136C	YOR182C	909
YJL136C	YOR234C	968
YJL136C	YOR293W	959
YJL136C	YOR312C	934
YJL136C	YOR369C	935
YJL136C	YPL079W	974
YJL136C	YPL081W	935
YJL136C	YPL090C	906
YJL136C	YPL131W	948
YJL136C	YPL143W	965
YJL136C	YPL198W	934
YJL136C	YPL220W	945
YJL136C	YPL237W	912
YJL136C	YPL249C-A	941
YJL136C	YPR043W	946
YJL136C	YPR080W	910
YJL136C	YPR102C	973
YJL136C	YPR132W	953
YJL137C	YEL011W	989
YJL137C	YFR015C	997
YJL137C	YKR058W	994
YJL137C	YLR258W	999
YJL138C	YBL051C	936
YJL138C	YBR101C	925
YJL138C	YDR064W	904

YJL138C	YDR385W	962
YJL138C	YER165W	903
YJL138C	YGL049C	995
YJL138C	YGR162W	999
YJL138C	YHL015W	904
YJL138C	YJR007W	975
YJL138C	YKR059W	999
YJL138C	YMR186W	960
YJL138C	YMR260C	901
YJL138C	YNL178W	906
YJL138C	YOL040C	901
YJL138C	YOL139C	998
YJL140W	YBL026W	984
YJL140W	YBR154C	999
YJL140W	YCR077C	976
YJL140W	YDL005C	910
YJL140W	YDL115C	997
YJL140W	YDL140C	999
YJL140W	YDR404C	999
YJL140W	YDR443C	922
YJL140W	YER022W	981
YJL140W	YER148W	985
YJL140W	YGL025C	923
YJL140W	YGL043W	999
YJL140W	YGL070C	999
YJL140W	YGL127C	946
YJL140W	YGR005C	999
YJL140W	YGR063C	987
YJL140W	YGR104C	983
YJL140W	YGR186W	998
YJL140W	YHL021C	917
YJL140W	YHR041C	912
YJL140W	YHR143W-A	998
YJL140W	YIL021W	999
YJL140W	YLR071C	999
YJL140W	YLR418C	914
YJL140W	YML010W	999
YJL140W	YMR273C	903
YJL140W	YMR277W	960
YJL140W	YNR010W	982
YJL140W	YOL005C	999
YJL140W	YOL145C	912
YJL140W	YOR116C	904
YJL140W	YOR151C	999

YJL140W	YOR207C	901
YJL140W	YOR210W	999
YJL140W	YOR224C	999
YJL140W	YPL129W	999
YJL140W	YPR168W	928
YJL140W	YPR187W	999
YJL141C	YBR195C	904
YJL141C	YDL047W	960
YJL141C	YDR099W	991
YJL141C	YDR223W	930
YJL141C	YER177W	993
YJL141C	YGL073W	972
YJL141C	YKL062W	959
YJL141C	YMR037C	993
YJL141C	YNL098C	983
YJL141C	YPL247C	930
YJL143W	YBL045C	969
YJL143W	YGR033C	999
YJL143W	YIL022W	999
YJL143W	YJL104W	999
YJL143W	YJR045C	992
YJL143W	YLR008C	999
YJL143W	YNR017W	999
YJL143W	YPL063W	999
YJL145W	YMR079W	982
YJL146W	YJL020C	925
YJL146W	YJL106W	908
YJL146W	YPL154C	993
YJL148W	YAL038W	923
YJL148W	YBL039C	922
YJL148W	YBR154C	999
YJL148W	YDL150W	922
YJL148W	YDR045C	921
YJL148W	YDR156W	999
YJL148W	YDR174W	913
YJL148W	YER004W	940
YJL148W	YHR143W-A	961
YJL148W	YJR063W	999
YJL148W	YKL144C	930
YJL148W	YNL113W	999
YJL148W	YNL151C	932
YJL148W	YNL248C	999
YJL148W	YNR003C	921
YJL148W	YOL006C	998

YJL148W	YOR116C	922
YJL148W	YOR207C	922
YJL148W	YOR210W	998
YJL148W	YOR340C	999
YJL148W	YOR341W	999
YJL148W	YPR010C	999
YJL148W	YPR110C	999
YJL148W	YPR187W	999
YJL148W	YPR190C	931
YJL149W	YDL132W	999
YJL149W	YDR328C	999
YJL151C	YER125W	999
YJL152W	YOR281C	998
YJL153C	YDR123C	997
YJL153C	YDR207C	910
YJL153C	YDR287W	933
YJL153C	YDR477W	921
YJL153C	YER120W	986
YJL153C	YGL253W	909
YJL153C	YHL020C	979
YJL153C	YHR046C	957
YJL153C	YLR133W	907
YJL153C	YMR105C	909
YJL153C	YOL004W	915
YJL153C	YOL108C	995
YJL154C	YBL017C	998
YJL154C	YHR012W	999
YJL154C	YJL053W	999
YJL154C	YLR262C	952
YJL154C	YOR069W	999
YJL154C	YOR132W	999
YJL155C	YER003C	919
YJL155C	YFR053C	911
YJL155C	YGL253W	906
YJL155C	YGR240C	967
YJL155C	YLR345W	999
YJL155C	YLR377C	966
YJL155C	YMR205C	986
YJL156C	YDR160W	997
YJL156C	YDR463W	957
YJL156C	YFR029W	999
YJL156C	YKR039W	943
YJL157C	YAL040C	999
YJL157C	YAL041W	999

YJL157C	YBL016W	999
YJL157C	YBR160W	999
YJL157C	YBR200W	999
YJL157C	YCL027W	998
YJL157C	YDR054C	950
YJL157C	YDR103W	985
YJL157C	YHR084W	954
YJL157C	YJR086W	977
YJL157C	YLR229C	999
YJL157C	YLR362W	982
YJL157C	YMR199W	985
YJL157C	YNL271C	999
YJL157C	YOR212W	996
YJL157C	YPL256C	999
YJL157C	YPR120C	978
YJL158C	YJL159W	977
YJL158C	YKL164C	977
YJL159W	YJL158C	977
YJL159W	YKL164C	982
YJL164C	YAL038W	983
YJL164C	YBR274W	920
YJL164C	YDR099W	962
YJL164C	YDR443C	965
YJL164C	YER177W	985
YJL164C	YFL033C	902
YJL164C	YHR205W	978
YJL164C	YIL033C	999
YJL164C	YJL005W	987
YJL164C	YKL038W	928
YJL164C	YKL166C	999
YJL164C	YLR310C	939
YJL164C	YNL093W	921
YJL164C	YNL098C	999
YJL164C	YOR101W	997
YJL164C	YPL203W	999
YJL164C	YPL232W	967
YJL165C	YCR008W	937
YJL166W	Q0105	999
YJL166W	Q0120	999
YJL166W	YBL045C	999
YJL166W	YDR529C	999
YJL166W	YEL024W	999
YJL166W	YFR033C	999
YJL166W	YGR183C	999

YJL166W	YHR001W-A	994
YJL166W	YJR048W	989
YJL166W	YOR065W	999
YJL166W	YPR191W	999
YJL167W	YBR002C	976
YJL167W	YHR190W	926
YJL167W	YMR101C	981
YJL167W	YNR043W	939
YJL167W	YPL069C	987
YJL167W	YPL117C	904
YJL168C	YAL011W	961
YJL168C	YAL013W	960
YJL168C	YBR010W	982
YJL168C	YBR103W	961
YJL168C	YBR112C	971
YJL168C	YBR175W	952
YJL168C	YBR258C	952
YJL168C	YBR279W	986
YJL168C	YCL016C	952
YJL168C	YCR033W	979
YJL168C	YDL033C	952
YJL168C	YDL074C	986
YJL168C	YDL140C	999
YJL168C	YDR155C	952
YJL168C	YDR334W	986
YJL168C	YDR359C	987
YJL168C	YDR440W	954
YJL168C	YDR469W	952
YJL168C	YER164W	986
YJL168C	YGL043W	905
YJL168C	YGL045W	952
YJL168C	YGL127C	965
YJL168C	YGL194C	979
YJL168C	YGL207W	985
YJL168C	YGL244W	987
YJL168C	YGR112W	952
YJL168C	YIL021W	972
YJL168C	YIL112W	952
YJL168C	YKR029C	968
YJL168C	YLR015W	952
YJL168C	YLR085C	989
YJL168C	YLR226W	919
YJL168C	YLR268W	973
YJL168C	YLR386W	952

YJL168C	YLR418C	996
YJL168C	YML041C	979
YJL168C	YML069W	986
YJL168C	YML094W	963
YJL168C	YML128C	952
YJL168C	YNL153C	978
YJL168C	YNL294C	952
YJL168C	YNL297C	956
YJL168C	YNR051C	952
YJL168C	YOL012C	999
YJL168C	YOL068C	952
YJL168C	YOL145C	968
YJL168C	YOR106W	952
YJL168C	YOR123C	952
YJL168C	YOR151C	999
YJL168C	YPL055C	990
YJL168C	YPL138C	966
YJL168C	YPL161C	952
YJL168C	YPR161C	944
YJL170C	YCL067C	922
YJL170C	YKL178C	931
YJL170C	YPL240C	902
YJL171C	YCR044C	913
YJL172W	YLL039C	956
YJL173C	YAR007C	999
YJL173C	YBR136W	996
YJL173C	YCR028C-A	998
YJL173C	YDL156W	992
YJL173C	YDR097C	933
YJL173C	YER104W	988
YJL173C	YLR347C	988
YJL173C	YML032C	999
YJL173C	YMR234W	930
YJL173C	YNL312W	999
YJL174W	YDL049C	925
YJL174W	YGL022W	904
YJL174W	YGR166W	970
YJL174W	YHR206W	983
YJL174W	YNL322C	968
YJL174W	YPR159W	936
YJL176C	YBR289W	999
YJL176C	YDR073W	999
YJL176C	YFL049W	999
YJL176C	YGR275W	997

YJL176C	YHL025W	999
YJL176C	YMR033W	999
YJL176C	YNL207W	960
YJL176C	YNR023W	999
YJL176C	YOR290C	999
YJL176C	YPL016W	999
YJL176C	YPL129W	999
YJL176C	YPR034W	999
YJL177W	YAL035W	929
YJL177W	YBL027W	998
YJL177W	YBL072C	994
YJL177W	YBL087C	970
YJL177W	YBL092W	999
YJL177W	YBR031W	999
YJL177W	YBR048W	999
YJL177W	YBR084C-A	988
YJL177W	YBR118W	901
YJL177W	YBR181C	995
YJL177W	YBR189W	999
YJL177W	YBR191W	999
YJL177W	YCR031C	999
YJL177W	YDL061C	984
YJL177W	YDL075W	999
YJL177W	YDL081C	937
YJL177W	YDL082W	996
YJL177W	YDL083C	964
YJL177W	YDL130W	937
YJL177W	YDL136W	997
YJL177W	YDL191W	916
YJL177W	YDR012W	999
YJL177W	YDR025W	966
YJL177W	YDR064W	999
YJL177W	YDR174W	976
YJL177W	YDR382W	999
YJL177W	YDR385W	901
YJL177W	YDR418W	999
YJL177W	YDR447C	996
YJL177W	YDR450W	995
YJL177W	YDR471W	999
YJL177W	YDR500C	965
YJL177W	YEL054C	926
YJL177W	YER056C-A	985
YJL177W	YER074W	994
YJL177W	YER102W	969

YJL177W	YER117W	986
YJL177W	YER131W	970
YJL177W	YFL034C-A	913
YJL177W	YFR031C-A	998
YJL177W	YFR032C-A	996
YJL177W	YGL014W	950
YJL177W	YGL030W	999
YJL177W	YGL031C	997
YJL177W	YGL076C	998
YJL177W	YGL103W	999
YJL177W	YGL123W	999
YJL177W	YGL135W	996
YJL177W	YGL147C	998
YJL177W	YGL189C	905
YJL177W	YGR027C	999
YJL177W	YGR034W	998
YJL177W	YGR085C	999
YJL177W	YGR118W	999
YJL177W	YGR148C	979
YJL177W	YGR214W	999
YJL177W	YHL001W	999
YJL177W	YHL015W	988
YJL177W	YHL033C	999
YJL177W	YHR010W	973
YJL177W	YHR021C	919
YJL177W	YHR141C	995
YJL177W	YHR203C	999
YJL177W	YIL018W	998
YJL177W	YIL052C	995
YJL177W	YIL069C	995
YJL177W	YIL133C	996
YJL177W	YIL148W	956
YJL177W	YJL136C	952
YJL177W	YJL189W	938
YJL177W	YJL190C	998
YJL177W	YJL191W	996
YJL177W	YJR094W-A	982
YJL177W	YJR123W	999
YJL177W	YJR145C	970
YJL177W	YKL006W	999
YJL177W	YKL156W	977
YJL177W	YKL180W	999
YJL177W	YKR057W	921
YJL177W	YKR094C	905

YJL177W	YLL045C	996
YJL177W	YLR029C	999
YJL177W	YLR048W	999
YJL177W	YLR060W	963
YJL177W	YLR061W	997
YJL177W	YLR075W	999
YJL177W	YLR150W	956
YJL177W	YLR167W	948
YJL177W	YLR185W	969
YJL177W	YLR264W	984
YJL177W	YLR287C-A	994
YJL177W	YLR333C	926
YJL177W	YLR340W	999
YJL177W	YLR344W	992
YJL177W	YLR367W	990
YJL177W	YLR388W	998
YJL177W	YLR406C	919
YJL177W	YLR441C	995
YJL177W	YLR448W	999
YJL177W	YML024W	985
YJL177W	YML026C	918
YJL177W	YML063W	999
YJL177W	YML073C	996
YJL177W	YMR121C	927
YJL177W	YMR142C	996
YJL177W	YMR194W	999
YJL177W	YMR230W	946
YJL177W	YMR242C	996
YJL177W	YMR260C	905
YJL177W	YNL067W	994
YJL177W	YNL069C	996
YJL177W	YNL096C	995
YJL177W	YNL162W	921
YJL177W	YNL178W	999
YJL177W	YNL301C	998
YJL177W	YNL302C	997
YJL177W	YNR037C	941
YJL177W	YOL039W	927
YJL177W	YOL040C	999
YJL177W	YOL120C	914
YJL177W	YOL121C	914
YJL177W	YOL127W	999
YJL177W	YOR063W	999
YJL177W	YOR096W	999

YJL177W	YOR133W	932
YJL177W	YOR167C	939
YJL177W	YOR182C	901
YJL177W	YOR204W	931
YJL177W	YOR234C	999
YJL177W	YOR293W	972
YJL177W	YOR312C	997
YJL177W	YOR369C	912
YJL177W	YPL079W	957
YJL177W	YPL081W	955
YJL177W	YPL090C	995
YJL177W	YPL131W	997
YJL177W	YPL143W	979
YJL177W	YPL198W	995
YJL177W	YPL220W	998
YJL177W	YPL249C-A	997
YJL177W	YPR043W	951
YJL177W	YPR080W	901
YJL177W	YPR102C	993
YJL177W	YPR132W	960
YJL178C	YDL149W	998
YJL178C	YLR431C	991
YJL179W	YEL003W	999
YJL179W	YFL039C	963
YJL179W	YGR078C	999
YJL179W	YLR200W	999
YJL179W	YML094W	999
YJL179W	YML124C	923
YJL179W	YNL153C	999
YJL180C	YBL099W	938
YJL180C	YIL098C	999
YJL180C	YNL315C	966
YJL183W	YBR106W	991
YJL183W	YDR245W	999
YJL183W	YEL036C	999
YJL183W	YGR132C	993
YJL183W	YGR229C	920
YJL183W	YGR231C	995
YJL183W	YJR075W	999
YJL183W	YML115C	921
YJL183W	YPL050C	999
YJL184W	YGR262C	999
YJL184W	YKR038C	999
YJL184W	YKR095W-A	992

YJL184W	YML036W	999
YJL187C	YAL041W	940
YJL187C	YBR133C	999
YJL187C	YBR160W	999
YJL187C	YBR200W	989
YJL187C	YBR274W	921
YJL187C	YDL132W	946
YJL187C	YDL155W	949
YJL187C	YDR054C	973
YJL187C	YDR113C	925
YJL187C	YDR328C	907
YJL187C	YDR507C	999
YJL187C	YGL003C	990
YJL187C	YGL190C	907
YJL187C	YGR108W	954
YJL187C	YHR107C	996
YJL187C	YIL046W	998
YJL187C	YKL048C	990
YJL187C	YKL101W	999
YJL187C	YKR048C	954
YJL187C	YLR079W	992
YJL187C	YLR113W	978
YJL187C	YLR210W	952
YJL187C	YLR229C	972
YJL187C	YMR001C	999
YJL187C	YMR036C	999
YJL187C	YMR199W	941
YJL187C	YMR273C	912
YJL187C	YNL298W	999
YJL187C	YOL133W	940
YJL187C	YPR119W	999
YJL187C	YPR120C	928
YJL189W	YBL027W	927
YJL189W	YBL072C	920
YJL189W	YBL087C	923
YJL189W	YBL092W	919
YJL189W	YBR031W	927
YJL189W	YBR048W	956
YJL189W	YBR084C-A	947
YJL189W	YBR118W	903
YJL189W	YBR181C	963
YJL189W	YBR189W	938
YJL189W	YBR191W	956
YJL189W	YCR031C	936

YJL189W	YDL061C	944
YJL189W	YDL075W	981
YJL189W	YDL081C	914
YJL189W	YDL082W	921
YJL189W	YDL130W	916
YJL189W	YDL136W	936
YJL189W	YDL191W	919
YJL189W	YDR012W	929
YJL189W	YDR064W	981
YJL189W	YDR382W	973
YJL189W	YDR385W	908
YJL189W	YDR418W	931
YJL189W	YDR447C	908
YJL189W	YDR450W	908
YJL189W	YDR471W	931
YJL189W	YDR500C	929
YJL189W	YEL054C	900
YJL189W	YER056C-A	952
YJL189W	YER102W	936
YJL189W	YER117W	947
YJL189W	YER131W	919
YJL189W	YER165W	936
YJL189W	YFL034C-A	925
YJL189W	YFR031C-A	908
YJL189W	YFR032C-A	913
YJL189W	YGL030W	980
YJL189W	YGL031C	914
YJL189W	YGL076C	919
YJL189W	YGL103W	957
YJL189W	YGL123W	936
YJL189W	YGL135W	905
YJL189W	YGL147C	915
YJL189W	YGR027C	934
YJL189W	YGR034W	905
YJL189W	YGR085C	944
YJL189W	YGR118W	980
YJL189W	YGR148C	935
YJL189W	YGR214W	939
YJL189W	YHL001W	958
YJL189W	YHL015W	933
YJL189W	YHL033C	941
YJL189W	YHR010W	924
YJL189W	YHR021C	908
YJL189W	YHR141C	918

YJL189W	YHR203C	931
YJL189W	YIL018W	908
YJL189W	YIL052C	961
YJL189W	YIL133C	917
YJL189W	YIL148W	932
YJL189W	YJL136C	943
YJL189W	YJL177W	938
YJL189W	YJL191W	944
YJL189W	YJR094W-A	954
YJL189W	YJR123W	976
YJL189W	YJR145C	914
YJL189W	YKL006W	959
YJL189W	YKL156W	941
YJL189W	YKL180W	952
YJL189W	YKR057W	909
YJL189W	YKR094C	903
YJL189W	YLL045C	904
YJL189W	YLR029C	919
YJL189W	YLR048W	952
YJL189W	YLR061W	966
YJL189W	YLR075W	950
YJL189W	YLR167W	927
YJL189W	YLR185W	926
YJL189W	YLR264W	943
YJL189W	YLR333C	903
YJL189W	YLR340W	938
YJL189W	YLR344W	950
YJL189W	YLR367W	926
YJL189W	YLR388W	962
YJL189W	YLR406C	962
YJL189W	YLR441C	921
YJL189W	YLR448W	953
YJL189W	YML024W	957
YJL189W	YML026C	908
YJL189W	YML063W	942
YJL189W	YML073C	909
YJL189W	YMR142C	921
YJL189W	YMR194W	974
YJL189W	YMR230W	924
YJL189W	YMR242C	911
YJL189W	YNL067W	944
YJL189W	YNL069C	915
YJL189W	YNL162W	918
YJL189W	YNL178W	958

YJL189W	YNL301C	934
YJL189W	YNL302C	911
YJL189W	YOL039W	905
YJL189W	YOL040C	919
YJL189W	YOL120C	905
YJL189W	YOL121C	912
YJL189W	YOL127W	953
YJL189W	YOR063W	935
YJL189W	YOR096W	948
YJL189W	YOR133W	908
YJL189W	YOR167C	921
YJL189W	YOR234C	943
YJL189W	YOR293W	944
YJL189W	YOR312C	911
YJL189W	YOR369C	900
YJL189W	YPL079W	934
YJL189W	YPL081W	914
YJL189W	YPL131W	935
YJL189W	YPL143W	949
YJL189W	YPL198W	909
YJL189W	YPL220W	924
YJL189W	YPL249C-A	940
YJL189W	YPR043W	921
YJL189W	YPR080W	903
YJL189W	YPR102C	946
YJL189W	YPR132W	940
YJL190C	YAL035W	994
YJL190C	YBL027W	969
YJL190C	YBL072C	996
YJL190C	YBL087C	998
YJL190C	YBL092W	986
YJL190C	YBR031W	998
YJL190C	YBR048W	999
YJL190C	YBR084C-A	969
YJL190C	YBR181C	983
YJL190C	YBR189W	997
YJL190C	YBR191W	928
YJL190C	YCL037C	978
YJL190C	YCR031C	999
YJL190C	YDL014W	946
YJL190C	YDL061C	995
YJL190C	YDL075W	996
YJL190C	YDL081C	955
YJL190C	YDL082W	998

YJL190C	YDL083C	996
YJL190C	YDL130W	902
YJL190C	YDL136W	921
YJL190C	YDL191W	921
YJL190C	YDR012W	965
YJL190C	YDR025W	998
YJL190C	YDR041W	900
YJL190C	YDR064W	999
YJL190C	YDR382W	995
YJL190C	YDR385W	989
YJL190C	YDR418W	996
YJL190C	YDR429C	914
YJL190C	YDR447C	998
YJL190C	YDR450W	991
YJL190C	YDR471W	996
YJL190C	YDR500C	909
YJL190C	YEL054C	920
YJL190C	YER056C-A	900
YJL190C	YER074W	995
YJL190C	YER102W	996
YJL190C	YER117W	972
YJL190C	YER131W	931
YJL190C	YFL034C-A	907
YJL190C	YFR031C-A	999
YJL190C	YFR032C-A	928
YJL190C	YGL030W	997
YJL190C	YGL031C	951
YJL190C	YGL049C	997
YJL190C	YGL076C	998
YJL190C	YGL103W	998
YJL190C	YGL120C	936
YJL190C	YGL123W	999
YJL190C	YGL135W	996
YJL190C	YGL147C	999
YJL190C	YGL189C	914
YJL190C	YGR027C	928
YJL190C	YGR034W	999
YJL190C	YGR085C	999
YJL190C	YGR118W	991
YJL190C	YGR148C	925
YJL190C	YGR162W	997
YJL190C	YGR214W	992
YJL190C	YHL001W	997
YJL190C	YHL015W	999

YJL190C	YHL033C	996
YJL190C	YHL034C	981
YJL190C	YHR010W	903
YJL190C	YHR021C	921
YJL190C	YHR141C	999
YJL190C	YHR203C	998
YJL190C	YIL018W	998
YJL190C	YIL052C	900
YJL190C	YIL069C	995
YJL190C	YIL133C	999
YJL190C	YIL148W	902
YJL190C	YJL136C	930
YJL190C	YJL177W	998
YJL190C	YJL191W	998
YJL190C	YJR002W	931
YJL190C	YJR094W-A	913
YJL190C	YJR123W	999
YJL190C	YJR145C	998
YJL190C	YKL006W	936
YJL190C	YKL156W	921
YJL190C	YKL170W	932
YJL190C	YKL180W	998
YJL190C	YKR057W	923
YJL190C	YKR094C	902
YJL190C	YLL045C	998
YJL190C	YLR029C	995
YJL190C	YLR048W	999
YJL190C	YLR061W	909
YJL190C	YLR075W	995
YJL190C	YLR150W	956
YJL190C	YLR175W	992
YJL190C	YLR185W	910
YJL190C	YLR197W	931
YJL190C	YLR264W	918
YJL190C	YLR287C-A	900
YJL190C	YLR325C	978
YJL190C	YLR333C	928
YJL190C	YLR340W	996
YJL190C	YLR344W	974
YJL190C	YLR367W	912
YJL190C	YLR388W	970
YJL190C	YLR406C	912
YJL190C	YLR441C	998
YJL190C	YLR448W	996

YJL190C	YML024W	997
YJL190C	YML026C	919
YJL190C	YML063W	997
YJL190C	YML073C	997
YJL190C	YMR116C	994
YJL190C	YMR121C	938
YJL190C	YMR125W	939
YJL190C	YMR142C	937
YJL190C	YMR194W	996
YJL190C	YMR230W	998
YJL190C	YMR242C	999
YJL190C	YMR260C	927
YJL190C	YMR309C	915
YJL190C	YNL067W	981
YJL190C	YNL069C	998
YJL190C	YNL081C	904
YJL190C	YNL096C	997
YJL190C	YNL162W	920
YJL190C	YNL178W	999
YJL190C	YNL301C	921
YJL190C	YNL302C	915
YJL190C	YNR037C	956
YJL190C	YOL039W	916
YJL190C	YOL040C	998
YJL190C	YOL120C	921
YJL190C	YOL121C	965
YJL190C	YOL127W	998
YJL190C	YOL139C	997
YJL190C	YOR063W	998
YJL190C	YOR096W	997
YJL190C	YOR133W	901
YJL190C	YOR167C	944
YJL190C	YOR182C	900
YJL190C	YOR204W	931
YJL190C	YOR234C	932
YJL190C	YOR293W	938
YJL190C	YOR312C	996
YJL190C	YOR361C	915
YJL190C	YOR369C	985
YJL190C	YPL079W	936
YJL190C	YPL081W	999
YJL190C	YPL131W	998
YJL190C	YPL143W	925
YJL190C	YPL146C	968

YJL190C	YPL198W	997
YJL190C	YPL220W	912
YJL190C	YPL249C-A	915
YJL190C	YPR043W	913
YJL190C	YPR080W	901
YJL190C	YPR102C	985
YJL190C	YPR132W	921
YJL190C	YPR163C	916
YJL191W	YAL035W	918
YJL191W	YBL027W	916
YJL191W	YBL072C	919
YJL191W	YBL087C	958
YJL191W	YBL092W	943
YJL191W	YBR031W	977
YJL191W	YBR048W	944
YJL191W	YBR079C	921
YJL191W	YBR084C-A	955
YJL191W	YBR118W	907
YJL191W	YBR189W	999
YJL191W	YBR191W	983
YJL191W	YCR031C	999
YJL191W	YDL061C	982
YJL191W	YDL075W	972
YJL191W	YDL081C	921
YJL191W	YDL082W	998
YJL191W	YDL130W	943
YJL191W	YDL136W	948
YJL191W	YDL166C	902
YJL191W	YDL191W	920
YJL191W	YDR012W	984
YJL191W	YDR025W	927
YJL191W	YDR041W	952
YJL191W	YDR064W	999
YJL191W	YDR382W	993
YJL191W	YDR385W	901
YJL191W	YDR418W	982
YJL191W	YDR429C	919
YJL191W	YDR447C	998
YJL191W	YDR450W	979
YJL191W	YDR471W	963
YJL191W	YDR500C	961
YJL191W	YEL054C	931
YJL191W	YER025W	948
YJL191W	YER056C-A	972

YJL191W	YER074W	918
YJL191W	YER102W	954
YJL191W	YER117W	973
YJL191W	YER131W	960
YJL191W	YER165W	936
YJL191W	YFL034C-A	919
YJL191W	YFR031C-A	998
YJL191W	YFR032C-A	937
YJL191W	YGL030W	998
YJL191W	YGL031C	930
YJL191W	YGL076C	963
YJL191W	YGL103W	975
YJL191W	YGL123W	996
YJL191W	YGL135W	925
YJL191W	YGL147C	922
YJL191W	YGL189C	921
YJL191W	YGR027C	980
YJL191W	YGR034W	925
YJL191W	YGR085C	977
YJL191W	YGR118W	973
YJL191W	YGR148C	964
YJL191W	YGR162W	909
YJL191W	YGR214W	995
YJL191W	YHL001W	997
YJL191W	YHL015W	965
YJL191W	YHL033C	987
YJL191W	YHR010W	962
YJL191W	YHR021C	935
YJL191W	YHR141C	928
YJL191W	YHR203C	999
YJL191W	YIL018W	934
YJL191W	YIL052C	980
YJL191W	YIL069C	997
YJL191W	YIL133C	965
YJL191W	YIL148W	953
YJL191W	YJL136C	963
YJL191W	YJL177W	996
YJL191W	YJL189W	944
YJL191W	YJL190C	998
YJL191W	YJR007W	901
YJL191W	YJR094W-A	977
YJL191W	YJR123W	999
YJL191W	YJR145C	930
YJL191W	YKL006W	995

YJL191W	YKL156W	986
YJL191W	YKL170W	964
YJL191W	YKL180W	999
YJL191W	YKR057W	932
YJL191W	YKR059W	930
YJL191W	YKR094C	909
YJL191W	YLL045C	945
YJL191W	YLR029C	971
YJL191W	YLR048W	988
YJL191W	YLR061W	994
YJL191W	YLR075W	964
YJL191W	YLR167W	941
YJL191W	YLR185W	957
YJL191W	YLR264W	977
YJL191W	YLR287C-A	908
YJL191W	YLR325C	914
YJL191W	YLR333C	927
YJL191W	YLR340W	978
YJL191W	YLR344W	971
YJL191W	YLR367W	964
YJL191W	YLR388W	996
YJL191W	YLR406C	925
YJL191W	YLR441C	923
YJL191W	YLR448W	994
YJL191W	YML024W	983
YJL191W	YML026C	979
YJL191W	YML063W	999
YJL191W	YML073C	919
YJL191W	YMR121C	925
YJL191W	YMR142C	998
YJL191W	YMR194W	988
YJL191W	YMR230W	946
YJL191W	YMR242C	927
YJL191W	YMR260C	945
YJL191W	YMR309C	903
YJL191W	YNL067W	977
YJL191W	YNL069C	967
YJL191W	YNL081C	994
YJL191W	YNL096C	931
YJL191W	YNL162W	928
YJL191W	YNL178W	999
YJL191W	YNL301C	956
YJL191W	YNL302C	927
YJL191W	YNR037C	984

YJL191W	YOL039W	939
YJL191W	YOL040C	952
YJL191W	YOL120C	909
YJL191W	YOL121C	927
YJL191W	YOL127W	981
YJL191W	YOR063W	998
YJL191W	YOR096W	999
YJL191W	YOR133W	918
YJL191W	YOR167C	941
YJL191W	YOR182C	908
YJL191W	YOR234C	982
YJL191W	YOR293W	963
YJL191W	YOR312C	936
YJL191W	YOR361C	921
YJL191W	YOR369C	942
YJL191W	YPL079W	957
YJL191W	YPL081W	988
YJL191W	YPL131W	977
YJL191W	YPL143W	982
YJL191W	YPL198W	930
YJL191W	YPL220W	960
YJL191W	YPL237W	923
YJL191W	YPL249C-A	962
YJL191W	YPR041W	905
YJL191W	YPR043W	939
YJL191W	YPR080W	907
YJL191W	YPR102C	999
YJL191W	YPR132W	955
YJL192C	YFL026W	909
YJL192C	YGL008C	909
YJL194W	YAR019C	999
YJL194W	YBL023C	999
YJL194W	YBR060C	999
YJL194W	YBR135W	998
YJL194W	YBR160W	999
YJL194W	YBR202W	999
YJL194W	YCL061C	952
YJL194W	YDL017W	971
YJL194W	YDL042C	976
YJL194W	YDR054C	947
YJL194W	YDR146C	999
YJL194W	YDR446W	982
YJL194W	YEL032W	993
YJL194W	YFL009W	999

YJL194W	YFR028C	999
YJL194W	YGL003C	999
YJL194W	YGL190C	971
YJL194W	YGL201C	990
YJL194W	YGR109C	948
YJL194W	YHR118C	999
YJL194W	YIL150C	943
YJL194W	YJR046W	942
YJL194W	YKL145W	978
YJL194W	YLL004W	999
YJL194W	YLR079W	999
YJL194W	YLR103C	995
YJL194W	YLR274W	999
YJL194W	YML065W	999
YJL194W	YNL102W	956
YJL194W	YNL261W	999
YJL194W	YNL262W	910
YJL194W	YPL153C	959
YJL194W	YPR019W	994
YJL194W	YPR119W	999
YJL194W	YPR120C	996
YJL194W	YPR135W	931
YJL194W	YPR162C	999
YJL196C	YCL045C	923
YJL197W	YHR005C	905
YJL198W	YML123C	987
YJL199C	YPL070W	984
YJL200C	YCR005C	930
YJL200C	YDL066W	904
YJL200C	YIL094C	923
YJL200C	YLR174W	904
YJL200C	YLR304C	915
YJL200C	YNL009W	903
YJL200C	YNL037C	921
YJL200C	YNR001C	926
YJL200C	YOR136W	931
YJL200C	YPR001W	902
YJL201W	YBL007C	970
YJL203W	YAL032C	968
YJL203W	YBR055C	976
YJL203W	YBR152W	931
YJL203W	YDL030W	999
YJL203W	YDL043C	999
YJL203W	YDL098C	974

YJL203W	YDR473C	980
YJL203W	YER029C	993
YJL203W	YER172C	993
YJL203W	YFL017W-A	918
YJL203W	YGR074W	999
YJL203W	YGR091W	974
YJL203W	YHR165C	993
YJL203W	YKL173W	993
YJL203W	YLR117C	974
YJL203W	YLR147C	976
YJL203W	YML049C	998
YJL203W	YMR240C	999
YJL203W	YMR268C	900
YJL203W	YMR288W	998
YJL203W	YOR308C	974
YJL203W	YOR319W	998
YJL203W	YPL151C	974
YJL203W	YPL213W	999
YJL203W	YPR082C	974
YJL203W	YPR178W	993
YJL204C	YAL030W	989
YJL204C	YCR094W	973
YJL204C	YDR328C	999
YJL204C	YER031C	933
YJL207C	YKR017C	998
YJL207C	YOR299W	980
YJL207C	YPR029C	910
YJL208C	YKR079C	999
YJL208C	YMR099C	999
YJL209W	Q0105	927
YJL210W	YDL065C	934
YJL210W	YDR142C	956
YJL210W	YDR244W	995
YJL210W	YDR265W	994
YJL210W	YGL153W	995
YJL210W	YGR077C	993
YJL210W	YLR191W	995
YJL210W	YMR026C	994
YJL210W	YNL214W	993
YJL217W	YER027C	922
YJL218W	YPL070W	975
YJL219W	YBL005W	966
YJL219W	YGL013C	962
YJL225C	YBL113C	909

YJL225C	YHL050C	998
YJL225C	YHR219W	960
YJR002W	YBL004W	999
YJR002W	YBL072C	933
YJR002W	YBR189W	986
YJR002W	YBR247C	999
YJR002W	YCL059C	999
YJR002W	YCR057C	999
YJR002W	YDL014W	999
YJR002W	YDL060W	921
YJR002W	YDL148C	999
YJR002W	YDL153C	996
YJR002W	YDR064W	935
YJR002W	YDR299W	998
YJR002W	YDR324C	999
YJR002W	YDR339C	984
YJR002W	YDR398W	986
YJR002W	YDR449C	999
YJR002W	YEL026W	984
YJR002W	YER082C	999
YJR002W	YER102W	929
YJR002W	YGL120C	918
YJR002W	YGL171W	963
YJR002W	YGR081C	910
YJR002W	YGR090W	999
YJR002W	YGR128C	999
YJR002W	YGR145W	924
YJR002W	YHR148W	999
YJR002W	YHR169W	947
YJR002W	YHR196W	999
YJR002W	YIL069C	925
YJR002W	YJL010C	985
YJR002W	YJL069C	991
YJR002W	YJL109C	999
YJR002W	YJL190C	931
YJR002W	YJR123W	933
YJR002W	YJR145C	931
YJR002W	YKL099C	986
YJR002W	YKR060W	998
YJR002W	YLL011W	999
YJR002W	YLR003C	979
YJR002W	YLR129W	999
YJR002W	YLR175W	921
YJR002W	YLR186W	999

YJR002W	YLR197W	999
YJR002W	YLR222C	999
YJR002W	YLR409C	999
YJR002W	YML093W	989
YJR002W	YMR093W	999
YJR002W	YMR128W	999
YJR002W	YMR229C	994
YJR002W	YMR290C	937
YJR002W	YNL075W	999
YJR002W	YNL132W	996
YJR002W	YOL010W	997
YJR002W	YOR004W	991
YJR002W	YOR078W	999
YJR002W	YOR096W	987
YJR002W	YOR145C	998
YJR002W	YOR310C	999
YJR002W	YPL012W	996
YJR002W	YPL126W	999
YJR002W	YPL217C	999
YJR002W	YPL266W	997
YJR002W	YPR112C	967
YJR002W	YPR137W	999
YJR002W	YPR144C	999
YJR004C	YKL178C	998
YJR004C	YLR040C	962
YJR005W	YBL037W	996
YJR005W	YJR058C	917
YJR005W	YOL062C	998
YJR006W	YAR007C	914
YJR006W	YBR088C	999
YJR006W	YCR066W	984
YJR006W	YDL102W	999
YJR006W	YGL087C	979
YJR006W	YHR164C	927
YJR006W	YIR008C	910
YJR006W	YJR043C	999
YJR006W	YKL113C	960
YJR006W	YNL102W	988
YJR006W	YNL218W	997
YJR006W	YNL312W	900
YJR006W	YOR080W	921
YJR007W	YBL072C	903
YJR007W	YBR079C	999
YJR007W	YBR159W	962

YJR007W	YBR181C	913
YJR007W	YCR031C	901
YJR007W	YDR211W	999
YJR007W	YDR283C	994
YJR007W	YER025W	999
YJR007W	YER102W	903
YJR007W	YER165W	919
YJR007W	YGL049C	984
YJR007W	YGR083C	999
YJR007W	YGR162W	978
YJR007W	YHL015W	907
YJR007W	YHR021C	948
YJR007W	YHR203C	902
YJR007W	YIL133C	900
YJR007W	YJL136C	900
YJR007W	YJL138C	975
YJR007W	YJL191W	901
YJR007W	YJR123W	906
YJR007W	YJR145C	902
YJR007W	YKL156W	924
YJR007W	YKR026C	999
YJR007W	YKR057W	900
YJR007W	YKR059W	968
YJR007W	YLR167W	984
YJR007W	YLR192C	924
YJR007W	YLR291C	999
YJR007W	YMR146C	997
YJR007W	YMR260C	917
YJR007W	YMR309C	997
YJR007W	YNL069C	900
YJR007W	YNL096C	903
YJR007W	YNL302C	904
YJR007W	YOL121C	904
YJR007W	YOL139C	935
YJR007W	YOR096W	903
YJR007W	YOR167C	909
YJR007W	YOR260W	999
YJR007W	YOR361C	961
YJR007W	YOR369C	902
YJR007W	YPL090C	913
YJR007W	YPL237W	999
YJR007W	YPR041W	999
YJR007W	YPR163C	914
YJR009C	YCR012W	992

YJR009C	YDL188C	933
YJR009C	YDR050C	989
YJR009C	YGR192C	969
YJR009C	YJL052W	999
YJR009C	YKL060C	972
YJR009C	YML053C	986
YJR010C-A	YER019C-A	954
YJR010C-A	YER087C-B	948
YJR010C-A	YIR022W	999
YJR010C-A	YJL034W	923
YJR010C-A	YLR066W	998
YJR010C-A	YML055W	997
YJR010C-A	YPL094C	921
YJR010W	YKL001C	999
YJR010W	YLR303W	917
YJR010W	YOL064C	920
YJR010W	YPR167C	945
YJR011C	YGR134W	997
YJR013W	YCL052C	929
YJR013W	YKL165C	931
YJR014W	YER007C-A	999
YJR015W	YHR188C	904
YJR016C	YER086W	938
YJR016C	YHR208W	943
YJR016C	YJR148W	959
YJR016C	YLR355C	996
YJR016C	YMR108W	941
YJR016C	YNL104C	952
YJR016C	YOR108W	934
YJR017C	YDL140C	999
YJR017C	YDR151C	969
YJR017C	YDR155C	981
YJR017C	YER125W	916
YJR017C	YGL043W	951
YJR017C	YGR063C	950
YJR017C	YKL005C	997
YJR017C	YML010W	926
YJR017C	YMR263W	969
YJR017C	YMR277W	981
YJR021C	YER044C-A	969
YJR021C	YHR014W	986
YJR021C	YHR086W	983
YJR021C	YML032C	979
YJR021C	YMR133W	997

YJR021C	YNL210W	986
YJR022W	YBL026W	999
YJR022W	YBR055C	962
YJR022W	YCR020C-A	994
YJR022W	YCR077C	996
YJR022W	YDL051W	953
YJR022W	YDR378C	999
YJR022W	YDR473C	931
YJR022W	YEL015W	930
YJR022W	YER029C	999
YJR022W	YER112W	999
YJR022W	YER146W	999
YJR022W	YER172C	999
YJR022W	YGL173C	999
YJR022W	YGR074W	999
YJR022W	YGR091W	963
YJR022W	YGR158C	949
YJR022W	YHR165C	991
YJR022W	YJL124C	999
YJR022W	YKL173W	998
YJR022W	YLR147C	999
YJR022W	YLR264W	952
YJR022W	YLR275W	999
YJR022W	YLR438C-A	999
YJR022W	YMR268C	999
YJR022W	YNL147W	999
YJR022W	YOL149W	989
YJR022W	YOR308C	998
YJR022W	YOR319W	943
YJR022W	YPR178W	999
YJR024C	YEL038W	985
YJR024C	YNL010W	958
YJR024C	YPR118W	909
YJR025C	YLR231C	985
YJR028W	YBR012W-A	998
YJR031C	YDL137W	998
YJR031C	YDL145C	977
YJR031C	YDL192W	999
YJR031C	YDR170C	912
YJR031C	YEL022W	996
YJR031C	YGL137W	977
YJR031C	YKR030W	989
YJR031C	YNL287W	986
YJR031C	YOR094W	936

YJR032W	YAL005C	999
YJR032W	YBR155W	999
YJR032W	YDR168W	977
YJR032W	YLL024C	942
YJR032W	YLL026W	998
YJR032W	YMR186W	999
YJR032W	YNL330C	976
YJR032W	YOR027W	998
YJR032W	YOR070C	908
YJR032W	YPL240C	999
YJR033C	YBR127C	999
YJR033C	YDL185W	994
YJR033C	YDR202C	999
YJR033C	YDR328C	994
YJR033C	YEL051W	933
YJR033C	YKL080W	915
YJR033C	YOR332W	999
YJR035W	YBR114W	999
YJR035W	YDL140C	929
YJR035W	YDR030C	945
YJR035W	YDR404C	901
YJR035W	YER171W	988
YJR035W	YGL043W	988
YJR035W	YGL070C	998
YJR035W	YGR258C	993
YJR035W	YIL021W	900
YJR035W	YIL143C	951
YJR035W	YJR052W	999
YJR035W	YKL054C	955
YJR035W	YMR201C	951
YJR035W	YOR151C	920
YJR035W	YOR210W	902
YJR035W	YOR224C	900
YJR035W	YPL022W	930
YJR035W	YPR025C	900
YJR035W	YPR187W	901
YJR037W	YDL246C	998
YJR040W	YDL100C	910
YJR040W	YPL240C	919
YJR041C	YKL014C	999
YJR041C	YKR024C	946
YJR041C	YLR221C	983
YJR041C	YLR276C	949
YJR041C	YNR038W	982

YJR041C	YOL144W	999
YJR042W	YDL116W	999
YJR042W	YDL207W	962
YJR042W	YDR159W	966
YJR042W	YDR192C	961
YJR042W	YER105C	959
YJR042W	YFR002W	930
YJR042W	YGL092W	999
YJR042W	YGL100W	999
YJR042W	YGL172W	998
YJR042W	YGR119C	976
YJR042W	YHR004C	918
YJR042W	YHR170W	982
YJR042W	YIL115C	992
YJR042W	YJL041W	961
YJR042W	YJL061W	951
YJR042W	YKL057C	999
YJR042W	YKL068W	999
YJR042W	YKL186C	998
YJR042W	YKR082W	999
YJR042W	YLR208W	999
YJR042W	YLR335W	991
YJR042W	YLR347C	930
YJR042W	YMR047C	999
YJR042W	YMR153W	937
YJR042W	YOR098C	931
YJR042W	YOR160W	906
YJR042W	YOR257W	961
YJR042W	YPL169C	999
YJR043C	YAL024C	919
YJR043C	YBL035C	905
YJR043C	YBR088C	999
YJR043C	YBR094W	924
YJR043C	YBR195C	919
YJR043C	YCL016C	923
YJR043C	YCL061C	959
YJR043C	YCR066W	915
YJR043C	YDL102W	999
YJR043C	YDR076W	963
YJR043C	YDR217C	959
YJR043C	YDR359C	980
YJR043C	YDR363W	917
YJR043C	YDR369C	938
YJR043C	YDR419W	989

YJR043C	YDR440W	906
YJR043C	YER095W	966
YJR043C	YER151C	924
YJR043C	YER173W	975
YJR043C	YGL087C	986
YJR043C	YGL163C	968
YJR043C	YHR164C	984
YJR043C	YIR008C	920
YJR043C	YJL092W	999
YJR043C	YJR006W	999
YJR043C	YKL045W	905
YJR043C	YKL113C	990
YJR043C	YLR032W	910
YJR043C	YLR234W	938
YJR043C	YLR288C	930
YJR043C	YLR370C	917
YJR043C	YML032C	990
YJR043C	YML061C	914
YJR043C	YMR048W	959
YJR043C	YMR078C	930
YJR043C	YMR190C	990
YJR043C	YNL064C	928
YJR043C	YNL102W	999
YJR043C	YNL218W	912
YJR043C	YNL250W	933
YJR043C	YNL273W	974
YJR043C	YOR025W	929
YJR043C	YOR144C	930
YJR043C	YOR368W	950
YJR043C	YPL022W	973
YJR043C	YPL024W	918
YJR043C	YPL194W	964
YJR043C	YPR135W	919
YJR044C	YOL129W	997
YJR045C	YFL016C	999
YJR045C	YIL022W	999
YJR045C	YJL143W	992
YJR045C	YLR008C	967
YJR045C	YLR203C	993
YJR045C	YLR369W	978
YJR045C	YNL064C	909
YJR045C	YNL310C	999
YJR045C	YNR017W	999
YJR045C	YOR232W	999

YJR046W	YBL023C	998
YJR046W	YBR060C	982
YJR046W	YBR202W	983
YJR046W	YEL032W	983
YJR046W	YGL201C	983
YJR046W	YHR118C	997
YJR046W	YJL194W	942
YJR046W	YLR274W	997
YJR046W	YML065W	984
YJR046W	YNL261W	984
YJR046W	YPR019W	983
YJR047C	YBL093C	929
YJR047C	YBR079C	962
YJR047C	YEL034W	993
YJR047C	YHR068W	991
YJR047C	YJR070C	928
YJR047C	YPR065W	946
YJR048W	Q0105	989
YJR048W	YBL045C	990
YJR048W	YDL106C	903
YJR048W	YDR529C	990
YJR048W	YEL024W	994
YJR048W	YEL039C	986
YJR048W	YER086W	909
YJR048W	YFR033C	989
YJR048W	YGR183C	990
YJR048W	YHR001W-A	910
YJR048W	YJL166W	989
YJR048W	YKR066C	999
YJR048W	YOR065W	999
YJR048W	YPR086W	934
YJR048W	YPR191W	991
YJR049C	YEL041W	998
YJR049C	YPL188W	999
YJR050W	YAL032C	968
YJR050W	YBR188C	999
YJR050W	YDL209C	965
YJR050W	YDR416W	999
YJR050W	YGR129W	997
YJR050W	YKR086W	983
YJR050W	YLL036C	999
YJR050W	YLR117C	999
YJR050W	YMR213W	999
YJR050W	YPL151C	994

YJR050W	YPL213W	989
YJR050W	YPR101W	998
YJR052W	YBR114W	999
YJR052W	YDR138W	967
YJR052W	YEL037C	999
YJR052W	YER162C	999
YJR052W	YER171W	958
YJR052W	YGL058W	915
YJR052W	YGR003W	984
YJR052W	YGR258C	970
YJR052W	YIL128W	945
YJR052W	YIL143C	908
YJR052W	YJR035W	999
YJR052W	YLR442C	998
YJR052W	YML095C	969
YJR052W	YMR201C	994
YJR052W	YNL312W	919
YJR052W	YPL022W	971
YJR052W	YPL046C	999
YJR053W	YDL220C	940
YJR053W	YDR328C	972
YJR053W	YER016W	988
YJR053W	YFR028C	939
YJR053W	YGL086W	917
YJR053W	YGR188C	917
YJR053W	YJL030W	992
YJR053W	YKR054C	920
YJR053W	YML064C	999
YJR053W	YMR001C	999
YJR053W	YMR055C	999
YJR053W	YOR026W	949
YJR053W	YOR233W	933
YJR053W	YPL269W	992
YJR056C	YML064C	993
YJR056C	YPL240C	919
YJR057W	YBR252W	941
YJR057W	YER070W	915
YJR057W	YGR180C	911
YJR057W	YIL066C	914
YJR057W	YJL026W	928
YJR057W	YKL024C	984
YJR057W	YKL067W	970
YJR057W	YOR074C	979
YJR058C	YBL037W	999

YJR058C	YGL206C	918
YJR058C	YJR005W	917
YJR058C	YOL062C	917
YJR059W	YGL008C	996
YJR059W	YMR216C	957
YJR060W	YDR253C	957
YJR060W	YDR328C	990
YJR060W	YER016W	965
YJR060W	YGL086W	983
YJR060W	YGR140W	999
YJR060W	YIL046W	912
YJR060W	YIR017C	997
YJR060W	YKL049C	997
YJR060W	YKL089W	997
YJR060W	YLR303W	918
YJR060W	YMR094W	987
YJR060W	YMR168C	999
YJR060W	YNL103W	999
YJR063W	YBL039C	923
YJR063W	YBR154C	999
YJR063W	YDL150W	921
YJR063W	YDR045C	931
YJR063W	YDR156W	999
YJR063W	YFR028C	974
YJR063W	YGL070C	955
YJR063W	YHR143W-A	999
YJR063W	YJL148W	999
YJR063W	YKL144C	921
YJR063W	YNL113W	999
YJR063W	YNL151C	936
YJR063W	YNL248C	999
YJR063W	YNR003C	921
YJR063W	YOR116C	921
YJR063W	YOR207C	921
YJR063W	YOR210W	999
YJR063W	YOR224C	999
YJR063W	YOR340C	999
YJR063W	YOR341W	999
YJR063W	YPR010C	999
YJR063W	YPR110C	999
YJR063W	YPR187W	999
YJR063W	YPR190C	934
YJR064W	YDL029W	935
YJR064W	YDL143W	996

YJR064W	YDR188W	927
YJR064W	YDR212W	924
YJR064W	YGR078C	901
YJR064W	YIL142W	921
YJR064W	YJL008C	920
YJR064W	YJL014W	982
YJR064W	YJL111W	924
YJR065C	YBR109C	903
YJR065C	YBR234C	999
YJR065C	YCR088W	952
YJR065C	YDL029W	999
YJR065C	YIL062C	999
YJR065C	YIR006C	964
YJR065C	YKL013C	999
YJR065C	YLR370C	999
YJR065C	YLR429W	978
YJR065C	YNR035C	999
YJR065C	YOR181W	992
YJR066W	YBR021W	980
YJR066W	YBR077C	970
YJR066W	YCR027C	931
YJR066W	YDL047W	900
YJR066W	YDR173C	901
YJR066W	YDR293C	972
YJR066W	YER040W	999
YJR066W	YER093C	922
YJR066W	YFL021W	961
YJR066W	YGL252C	973
YJR066W	YGR163W	977
YJR066W	YGR218W	924
YJR066W	YHR030C	992
YJR066W	YHR186C	999
YJR066W	YHR205W	994
YJR066W	YKL126W	963
YJR066W	YKL203C	999
YJR066W	YMR028W	968
YJR066W	YMR104C	975
YJR066W	YNL006W	999
YJR066W	YNL135C	983
YJR066W	YNL189W	927
YJR066W	YNL229C	991
YJR066W	YOL078W	904
YJR066W	YPL180W	998
YJR066W	YPL254W	901

YJR067C	YNL260C	992
YJR068W	YBL035C	970
YJR068W	YBR087W	999
YJR068W	YBR088C	999
YJR068W	YER173W	999
YJR068W	YIR008C	910
YJR068W	YMR078C	999
YJR068W	YNL102W	927
YJR068W	YNL290W	999
YJR068W	YOL094C	999
YJR068W	YOR144C	999
YJR068W	YOR217W	999
YJR070C	YEL034W	988
YJR070C	YHR068W	989
YJR070C	YJR047C	928
YJR070C	YLR168C	998
YJR070C	YMR269W	927
YJR072C	YLR243W	950
YJR072C	YOR262W	947
YJR073C	YGR157W	998
YJR073C	YJR074W	902
YJR073C	YPL240C	919
YJR074W	YER009W	969
YJR074W	YGL097W	920
YJR074W	YJR073C	902
YJR074W	YLR293C	999
YJR074W	YOR185C	999
YJR075W	YBR106W	990
YJR075W	YDR245W	999
YJR075W	YEL036C	999
YJR075W	YJL183W	999
YJR075W	YML115C	977
YJR075W	YPL050C	999
YJR075W	YPL221W	921
YJR076C	YBR160W	906
YJR076C	YCL024W	983
YJR076C	YCR002C	999
YJR076C	YDL225W	999
YJR076C	YDR013W	962
YJR076C	YDR218C	980
YJR076C	YDR507C	998
YJR076C	YDR510W	990
YJR076C	YHR107C	999
YJR076C	YJR092W	999

YJR076C	YKL048C	914
YJR076C	YKL101W	987
YJR076C	YLR314C	999
YJR076C	YNL166C	986
YJR076C	YNL233W	925
YJR076C	YPL161C	991
YJR077C	YBR017C	988
YJR077C	YBR069C	948
YJR077C	YEL022W	999
YJR077C	YGR218W	981
YJR077C	YHR020W	968
YJR077C	YJR121W	903
YJR077C	YKL182W	964
YJR077C	YLR342W	960
YJR078W	YOL097C	910
YJR082C	YBL052C	954
YJR082C	YDR359C	996
YJR082C	YEL018W	999
YJR082C	YFL024C	994
YJR082C	YGR002C	996
YJR082C	YHR090C	994
YJR082C	YML041C	919
YJR082C	YNL107W	999
YJR082C	YNL136W	999
YJR082C	YOR244W	999
YJR082C	YPR023C	997
YJR082C	YPR031W	954
YJR083C	YDR388W	980
YJR084W	YDL216C	997
YJR084W	YDR179C	995
YJR084W	YDR363W-A	968
YJR084W	YFL017W-A	922
YJR084W	YIL071C	958
YJR084W	YMR025W	998
YJR084W	YOL117W	995
YJR084W	YPR082C	989
YJR084W	YPR182W	921
YJR086W	YAL041W	907
YJR086W	YBL016W	955
YJR086W	YDL159W	964
YJR086W	YDR103W	999
YJR086W	YFL026W	968
YJR086W	YHL007C	966
YJR086W	YHR005C	999

YJR086W	YHR084W	973
YJR086W	YJL157C	977
YJR086W	YKL178C	945
YJR086W	YLR362W	968
YJR086W	YLR452C	911
YJR086W	YOR212W	999
YJR088C	YDR297W	901
YJR088C	YPL240C	919
YJR089W	YBR156C	999
YJR089W	YGR113W	968
YJR089W	YGR140W	999
YJR089W	YPL209C	998
YJR090C	YBR160W	973
YJR090C	YCL025C	922
YJR090C	YDL132W	999
YJR090C	YDL194W	954
YJR090C	YDR028C	997
YJR090C	YDR054C	999
YJR090C	YDR099W	938
YJR090C	YDR328C	999
YJR090C	YDR345C	902
YJR090C	YER133W	927
YJR090C	YER155C	901
YJR090C	YFL009W	932
YJR090C	YGL253W	967
YJR090C	YGL254W	993
YJR090C	YHR092C	917
YJR090C	YHR094C	993
YJR090C	YIL046W	953
YJR090C	YIL162W	917
YJR090C	YKL038W	999
YJR090C	YML006C	979
YJR090C	YMR032W	944
YJR090C	YMR199W	999
YJR090C	YOL133W	999
YJR090C	YOL136C	907
YJR090C	YPL092W	955
YJR090C	YPL256C	999
YJR091C	YFL037W	982
YJR091C	YLR370C	933
YJR092W	YCR002C	999
YJR092W	YHR107C	935
YJR092W	YIL140W	946
YJR092W	YJR076C	999

YJR092W	YLR314C	935
YJR092W	YLR353W	908
YJR092W	YPL242C	969
YJR092W	YPR122W	954
YJR093C	YAL043C	999
YJR093C	YDR195W	999
YJR093C	YDR301W	999
YJR093C	YER133W	999
YJR093C	YGR156W	999
YJR093C	YIL035C	976
YJR093C	YJL033W	951
YJR093C	YKL018W	999
YJR093C	YKL059C	999
YJR093C	YKR002W	999
YJR093C	YLR115W	999
YJR093C	YLR277C	999
YJR093C	YMR061W	999
YJR093C	YNL222W	999
YJR093C	YNL317W	999
YJR093C	YOR061W	976
YJR093C	YPR107C	999
YJR094C	YDR207C	999
YJR094C	YGL192W	914
YJR094C	YIL126W	997
YJR094C	YIR026C	954
YJR094C	YJL106W	995
YJR094C	YMR139W	999
YJR094C	YNL307C	978
YJR094C	YNL330C	951
YJR094W-A	YAL035W	916
YJR094W-A	YBL027W	992
YJR094W-A	YBL072C	926
YJR094W-A	YBL087C	992
YJR094W-A	YBL092W	999
YJR094W-A	YBR031W	952
YJR094W-A	YBR048W	929
YJR094W-A	YBR084C-A	993
YJR094W-A	YBR118W	909
YJR094W-A	YBR181C	903
YJR094W-A	YBR189W	971
YJR094W-A	YBR191W	999
YJR094W-A	YCR031C	976
YJR094W-A	YDL061C	981
YJR094W-A	YDL075W	999

YJR094W-A	YDL081C	939
YJR094W-A	YDL082W	921
YJR094W-A	YDL130W	937
YJR094W-A	YDL136W	998
YJR094W-A	YDL191W	923
YJR094W-A	YDL192W	944
YJR094W-A	YDR012W	996
YJR094W-A	YDR025W	916
YJR094W-A	YDR064W	998
YJR094W-A	YDR382W	986
YJR094W-A	YDR385W	918
YJR094W-A	YDR418W	997
YJR094W-A	YDR447C	932
YJR094W-A	YDR450W	920
YJR094W-A	YDR471W	956
YJR094W-A	YDR500C	994
YJR094W-A	YEL054C	923
YJR094W-A	YER056C-A	999
YJR094W-A	YER074W	917
YJR094W-A	YER102W	982
YJR094W-A	YER117W	998
YJR094W-A	YER131W	940
YJR094W-A	YFL034C-A	909
YJR094W-A	YFR031C-A	992
YJR094W-A	YFR032C-A	933
YJR094W-A	YGL030W	998
YJR094W-A	YGL031C	991
YJR094W-A	YGL076C	993
YJR094W-A	YGL103W	999
YJR094W-A	YGL123W	975
YJR094W-A	YGL135W	991
YJR094W-A	YGL147C	993
YJR094W-A	YGL189C	921
YJR094W-A	YGR027C	975
YJR094W-A	YGR034W	938
YJR094W-A	YGR085C	972
YJR094W-A	YGR118W	995
YJR094W-A	YGR148C	968
YJR094W-A	YGR214W	972
YJR094W-A	YHL001W	977
YJR094W-A	YHL015W	971
YJR094W-A	YHL033C	998
YJR094W-A	YHR010W	955
YJR094W-A	YHR021C	931

YJR094W-A	YHR141C	992
YJR094W-A	YHR203C	980
YJR094W-A	YIL018W	929
YJR094W-A	YIL052C	999
YJR094W-A	YIL069C	917
YJR094W-A	YIL133C	992
YJR094W-A	YIL148W	996
YJR094W-A	YJL136C	975
YJR094W-A	YJL177W	982
YJR094W-A	YJL189W	954
YJR094W-A	YJL190C	913
YJR094W-A	YJL191W	977
YJR094W-A	YJR123W	998
YJR094W-A	YJR145C	918
YJR094W-A	YKL006W	996
YJR094W-A	YKL156W	986
YJR094W-A	YKL180W	999
YJR094W-A	YKR057W	935
YJR094W-A	YKR094C	902
YJR094W-A	YLL045C	922
YJR094W-A	YLR029C	997
YJR094W-A	YLR048W	985
YJR094W-A	YLR061W	999
YJR094W-A	YLR075W	999
YJR094W-A	YLR167W	993
YJR094W-A	YLR185W	998
YJR094W-A	YLR264W	993
YJR094W-A	YLR287C-A	903
YJR094W-A	YLR325C	927
YJR094W-A	YLR333C	935
YJR094W-A	YLR340W	988
YJR094W-A	YLR344W	999
YJR094W-A	YLR367W	964
YJR094W-A	YLR388W	999
YJR094W-A	YLR406C	923
YJR094W-A	YLR441C	926
YJR094W-A	YLR448W	993
YJR094W-A	YML024W	999
YJR094W-A	YML026C	920
YJR094W-A	YML063W	975
YJR094W-A	YML073C	929
YJR094W-A	YMR121C	949
YJR094W-A	YMR142C	932
YJR094W-A	YMR194W	999

YJR094W-A	YMR230W	955
YJR094W-A	YMR242C	932
YJR094W-A	YNL067W	984
YJR094W-A	YNL069C	936
YJR094W-A	YNL096C	921
YJR094W-A	YNL162W	930
YJR094W-A	YNL178W	998
YJR094W-A	YNL301C	998
YJR094W-A	YNL302C	931
YJR094W-A	YOL039W	924
YJR094W-A	YOL040C	952
YJR094W-A	YOL120C	923
YJR094W-A	YOL121C	931
YJR094W-A	YOL127W	999
YJR094W-A	YOR063W	997
YJR094W-A	YOR096W	980
YJR094W-A	YOR133W	934
YJR094W-A	YOR167C	936
YJR094W-A	YOR182C	903
YJR094W-A	YOR234C	985
YJR094W-A	YOR293W	978
YJR094W-A	YOR312C	944
YJR094W-A	YOR369C	939
YJR094W-A	YPL037C	959
YJR094W-A	YPL079W	967
YJR094W-A	YPL081W	931
YJR094W-A	YPL090C	903
YJR094W-A	YPL131W	998
YJR094W-A	YPL143W	993
YJR094W-A	YPL198W	929
YJR094W-A	YPL220W	993
YJR094W-A	YPL249C-A	969
YJR094W-A	YPR043W	963
YJR094W-A	YPR080W	909
YJR094W-A	YPR102C	997
YJR094W-A	YPR132W	997
YJR097W	YDR320C	978
YJR099W	YDR139C	905
YJR099W	YLR167W	995
YJR101W	Q0140	962
YJR101W	YBL090W	988
YJR101W	YBR146W	988
YJR101W	YBR251W	999
YJR101W	YDR036C	972

YJR101W	YDR041W	999
YJR101W	YDR175C	988
YJR101W	YDR337W	988
YJR101W	YDR347W	997
YJR101W	YGL129C	988
YJR101W	YGR084C	974
YJR101W	YGR165W	988
YJR101W	YGR215W	988
YJR101W	YHL004W	999
YJR101W	YIL093C	988
YJR101W	YJR113C	988
YJR101W	YKL155C	997
YJR101W	YMR158W	974
YJR101W	YMR188C	988
YJR101W	YNL081C	986
YJR101W	YNL137C	988
YJR101W	YNL306W	988
YJR101W	YNR037C	987
YJR101W	YOR158W	988
YJR101W	YOR204W	984
YJR101W	YPL013C	997
YJR101W	YPL118W	988
YJR102C	YHL027W	939
YJR102C	YLR025W	901
YJR102C	YLR417W	999
YJR102C	YMR077C	999
YJR102C	YOR275C	916
YJR102C	YPL002C	999
YJR102C	YPL065W	999
YJR103W	YBL039C	982
YJR103W	YDL140C	926
YJR103W	YKL067W	922
YJR104C	YBR290W	999
YJR104C	YCL017C	991
YJR104C	YDR256C	987
YJR104C	YGL018C	988
YJR104C	YGL167C	999
YJR104C	YGR088W	983
YJR104C	YHR008C	999
YJR104C	YHR053C	993
YJR104C	YKL113C	901
YJR104C	YLL009C	969
YJR104C	YLR369W	987
YJR104C	YMR038C	999

YJR104C	YMR058W	993
YJR104C	YMR177W	903
YJR104C	YOR079C	931
YJR104C	YPL135W	931
YJR104C	YPR074C	993
YJR105W	YDR226W	975
YJR105W	YDR441C	918
YJR105W	YER170W	916
YJR105W	YLR060W	916
YJR105W	YLR209C	942
YJR105W	YLR359W	934
YJR105W	YML022W	940
YJR105W	YML035C	931
YJR105W	YNL141W	916
YJR105W	YPL037C	917
YJR109C	YDL171C	913
YJR109C	YDR394W	967
YJR109C	YER069W	923
YJR109C	YHR074W	901
YJR109C	YJL088W	975
YJR109C	YJL130C	971
YJR109C	YKL104C	921
YJR109C	YKL216W	980
YJR109C	YMR217W	956
YJR109C	YMR300C	969
YJR109C	YOL058W	920
YJR109C	YOR168W	900
YJR109C	YOR303W	999
YJR109C	YPR035W	918
YJR110W	YKL212W	979
YJR110W	YLR240W	944
YJR110W	YNL106C	999
YJR110W	YNL325C	968
YJR110W	YNR006W	999
YJR110W	YOR109W	999
YJR112W	YAL034W-A	999
YJR112W	YBR211C	980
YJR112W	YCL029C	976
YJR112W	YDR318W	976
YJR112W	YER018C	963
YJR112W	YIL144W	962
YJR112W	YIR010W	999
YJR112W	YKL049C	953
YJR112W	YKL089W	980

YJR112W	YLR045C	976
YJR112W	YMR168C	976
YJR112W	YOL069W	991
YJR112W	YOR195W	976
YJR112W	YPL233W	999
YJR113C	Q0140	962
YJR113C	YBL090W	988
YJR113C	YBR146W	998
YJR113C	YBR251W	999
YJR113C	YDL045W-A	986
YJR113C	YDR036C	999
YJR113C	YDR041W	999
YJR113C	YDR175C	988
YJR113C	YDR337W	995
YJR113C	YDR347W	988
YJR113C	YGL123W	902
YJR113C	YGL129C	988
YJR113C	YGR084C	974
YJR113C	YGR165W	988
YJR113C	YGR215W	988
YJR113C	YHL004W	999
YJR113C	YIL093C	988
YJR113C	YJR101W	988
YJR113C	YKL155C	997
YJR113C	YLR069C	905
YJR113C	YMR158W	974
YJR113C	YMR188C	988
YJR113C	YNL081C	998
YJR113C	YNL137C	988
YJR113C	YNL306W	988
YJR113C	YNR036C	999
YJR113C	YNR037C	999
YJR113C	YOR158W	988
YJR113C	YOR187W	916
YJR113C	YPL013C	999
YJR113C	YPL118W	988
YJR117W	YAL058W	913
YJR117W	YMR264W	913
YJR117W	YMR274C	999
YJR117W	YNL098C	918
YJR119C	YBR010W	976
YJR119C	YHR119W	902
YJR119C	YNL031C	976
YJR121W	Q0080	999

YJR121W	Q0085	999
YJR121W	Q0130	999
YJR121W	Q0250	923
YJR121W	YBL099W	999
YJR121W	YBR011C	906
YJR121W	YBR025C	999
YJR121W	YBR039W	999
YJR121W	YDL004W	999
YJR121W	YDL181W	999
YJR121W	YDL185W	980
YJR121W	YDR298C	999
YJR121W	YDR322C-A	998
YJR121W	YDR377W	999
YJR121W	YJR077C	903
YJR121W	YKL016C	999
YJR121W	YLR295C	999
YJR121W	YML081C-A	999
YJR121W	YMR267W	902
YJR121W	YNL315C	993
YJR121W	YOR065W	949
YJR121W	YPL078C	999
YJR121W	YPL271W	999
YJR121W	YPR020W	999
YJR121W	YPR191W	916
YJR123W	YAL035W	994
YJR123W	YBL027W	997
YJR123W	YBL072C	996
YJR123W	YBL087C	965
YJR123W	YBL092W	999
YJR123W	YBR031W	999
YJR123W	YBR048W	999
YJR123W	YBR079C	925
YJR123W	YBR084C-A	976
YJR123W	YBR118W	964
YJR123W	YBR181C	998
YJR123W	YBR189W	999
YJR123W	YBR191W	999
YJR123W	YCL059C	943
YJR123W	YCR031C	999
YJR123W	YDL061C	999
YJR123W	YDL075W	999
YJR123W	YDL081C	933
YJR123W	YDL082W	998
YJR123W	YDL083C	998

YJR123W	YDL130W	942
YJR123W	YDL136W	998
YJR123W	YDL191W	935
YJR123W	YDR012W	999
YJR123W	YDR025W	998
YJR123W	YDR041W	966
YJR123W	YDR064W	999
YJR123W	YDR382W	999
YJR123W	YDR385W	997
YJR123W	YDR418W	999
YJR123W	YDR429C	927
YJR123W	YDR447C	997
YJR123W	YDR450W	999
YJR123W	YDR471W	999
YJR123W	YDR500C	966
YJR123W	YEL054C	925
YJR123W	YER025W	964
YJR123W	YER056C-A	998
YJR123W	YER074W	997
YJR123W	YER102W	998
YJR123W	YER110C	979
YJR123W	YER117W	987
YJR123W	YER131W	977
YJR123W	YER165W	954
YJR123W	YFL034C-A	910
YJR123W	YFR031C-A	998
YJR123W	YFR032C-A	966
YJR123W	YGL030W	999
YJR123W	YGL031C	998
YJR123W	YGL076C	999
YJR123W	YGL103W	999
YJR123W	YGL123W	999
YJR123W	YGL135W	999
YJR123W	YGL147C	998
YJR123W	YGL189C	933
YJR123W	YGR027C	999
YJR123W	YGR034W	996
YJR123W	YGR085C	999
YJR123W	YGR118W	999
YJR123W	YGR148C	999
YJR123W	YGR162W	911
YJR123W	YGR214W	999
YJR123W	YHL001W	999
YJR123W	YHL015W	999

YJR123W	YHL033C	999
YJR123W	YHL034C	982
YJR123W	YHR010W	979
YJR123W	YHR021C	943
YJR123W	YHR141C	996
YJR123W	YHR203C	998
YJR123W	YIL018W	997
YJR123W	YIL052C	999
YJR123W	YIL069C	997
YJR123W	YIL133C	996
YJR123W	YIL148W	966
YJR123W	YJL080C	978
YJR123W	YJL136C	979
YJR123W	YJL177W	999
YJR123W	YJL189W	976
YJR123W	YJL190C	999
YJR123W	YJL191W	999
YJR123W	YJR002W	933
YJR123W	YJR007W	906
YJR123W	YJR094W-A	998
YJR123W	YJR145C	995
YJR123W	YKL006W	999
YJR123W	YKL156W	988
YJR123W	YKL170W	938
YJR123W	YKL180W	999
YJR123W	YKR057W	936
YJR123W	YKR059W	961
YJR123W	YKR094C	908
YJR123W	YLL039C	945
YJR123W	YLL045C	997
YJR123W	YLR029C	999
YJR123W	YLR048W	999
YJR123W	YLR061W	999
YJR123W	YLR075W	999
YJR123W	YLR167W	985
YJR123W	YLR185W	959
YJR123W	YLR197W	943
YJR123W	YLR249W	962
YJR123W	YLR264W	995
YJR123W	YLR287C-A	995
YJR123W	YLR325C	934
YJR123W	YLR333C	965
YJR123W	YLR340W	999
YJR123W	YLR344W	993

YJR123W	YLR367W	988
YJR123W	YLR388W	999
YJR123W	YLR406C	915
YJR123W	YLR441C	997
YJR123W	YLR448W	999
YJR123W	YML024W	999
YJR123W	YML026C	946
YJR123W	YML063W	999
YJR123W	YML073C	997
YJR123W	YMR093W	942
YJR123W	YMR116C	996
YJR123W	YMR121C	937
YJR123W	YMR142C	998
YJR123W	YMR143W	999
YJR123W	YMR146C	915
YJR123W	YMR194W	999
YJR123W	YMR230W	999
YJR123W	YMR242C	998
YJR123W	YMR260C	961
YJR123W	YNL067W	999
YJR123W	YNL069C	996
YJR123W	YNL081C	940
YJR123W	YNL096C	997
YJR123W	YNL132W	938
YJR123W	YNL162W	924
YJR123W	YNL178W	999
YJR123W	YNL209W	978
YJR123W	YNL301C	999
YJR123W	YNL302C	998
YJR123W	YNR037C	969
YJR123W	YOL039W	941
YJR123W	YOL040C	999
YJR123W	YOL120C	949
YJR123W	YOL121C	940
YJR123W	YOL127W	999
YJR123W	YOR063W	999
YJR123W	YOR096W	999
YJR123W	YOR133W	983
YJR123W	YOR167C	958
YJR123W	YOR204W	933
YJR123W	YOR234C	990
YJR123W	YOR293W	986
YJR123W	YOR312C	998
YJR123W	YOR361C	935

YJR123W	YOR369C	970
YJR123W	YPL037C	999
YJR123W	YPL079W	985
YJR123W	YPL081W	997
YJR123W	YPL090C	995
YJR123W	YPL131W	999
YJR123W	YPL143W	997
YJR123W	YPL198W	940
YJR123W	YPL220W	998
YJR123W	YPL237W	929
YJR123W	YPL249C-A	965
YJR123W	YPL266W	978
YJR123W	YPR041W	915
YJR123W	YPR043W	963
YJR123W	YPR080W	966
YJR123W	YPR102C	999
YJR123W	YPR132W	996
YJR123W	YPR163C	915
YJR123W	YPR189W	980
YJR125C	YDR153C	997
YJR125C	YGL206C	998
YJR125C	YGR167W	996
YJR125C	YHR108W	971
YJR125C	YOR111W	943
YJR125C	YPR029C	938
YJR127C	YNL241C	917
YJR130C	YAL012W	942
YJR130C	YER091C	906
YJR130C	YGL184C	941
YJR130C	YGR155W	955
YJR130C	YLR303W	910
YJR130C	YML082W	902
YJR130C	YNL277W	928
YJR131W	YCL001W	955
YJR132W	YER103W	960
YJR132W	YHR088W	923
YJR132W	YLR113W	935
YJR132W	YNL027W	990
YJR132W	YOR326W	902
YJR134C	YLR262C	999
YJR134C	YOR216C	930
YJR135C	YBR107C	999
YJR135C	YBR211C	999
YJR135C	YDR016C	963

YJR135C	YDR254W	999
YJR135C	YDR318W	999
YJR135C	YDR383C	998
YJR135C	YEL061C	939
YJR135C	YER016W	978
YJR135C	YGL060W	981
YJR135C	YGL086W	968
YJR135C	YGR113W	983
YJR135C	YGR140W	981
YJR135C	YGR179C	999
YJR135C	YGR188C	971
YJR135C	YIR010W	906
YJR135C	YJL030W	998
YJR135C	YKR037C	981
YJR135C	YLR315W	999
YJR135C	YLR381W	999
YJR135C	YMR094W	981
YJR135C	YPL018W	999
YJR135C	YPL233W	906
YJR135C	YPR046W	999
YJR135C	YPR141C	922
YJR135W-A	YBR091C	972
YJR135W-A	YEL020W-A	997
YJR135W-A	YGR181W	999
YJR135W-A	YHR005C-A	986
YJR135W-A	YNR017W	999
YJR136C	YKL033W	999
YJR137C	YFR030W	998
YJR137C	YHR042W	916
YJR137C	YKL001C	948
YJR137C	YKR069W	954
YJR137C	YLR303W	937
YJR137C	YPR167C	997
YJR138W	YBR154C	929
YJR138W	YGL190C	901
YJR138W	YGR162W	940
YJR139C	YCR053W	923
YJR139C	YDR158W	997
YJR139C	YER052C	998
YJR139C	YHR025W	988
YJR139C	YHR109W	998
YJR139C	YNL277W	993
YJR140C	YBL008W	998
YJR140C	YBR195C	991

YJR140C	YBR215W	999
YJR140C	YGR274C	923
YJR140C	YJL115W	997
YJR140C	YMR005W	923
YJR140C	YOR038C	999
YJR140C	YPL153C	935
YJR143C	YAL023C	994
YJR143C	YDL095W	989
YJR143C	YER143W	998
YJR143C	YIL140W	981
YJR143C	YOR321W	903
YJR145C	YAL035W	993
YJR145C	YBL027W	971
YJR145C	YBL072C	997
YJR145C	YBL087C	975
YJR145C	YBL092W	984
YJR145C	YBR031W	998
YJR145C	YBR048W	998
YJR145C	YBR079C	915
YJR145C	YBR084C-A	971
YJR145C	YBR118W	922
YJR145C	YBR142W	925
YJR145C	YBR181C	906
YJR145C	YBR189W	996
YJR145C	YBR191W	943
YJR145C	YBR247C	989
YJR145C	YCL011C	936
YJR145C	YCL037C	978
YJR145C	YCL059C	998
YJR145C	YCR031C	930
YJR145C	YCR057C	979
YJR145C	YDL014W	964
YJR145C	YDL060W	982
YJR145C	YDL061C	963
YJR145C	YDL075W	928
YJR145C	YDL081C	943
YJR145C	YDL082W	994
YJR145C	YDL083C	948
YJR145C	YDL130W	917
YJR145C	YDL136W	911
YJR145C	YDL148C	987
YJR145C	YDL191W	911
YJR145C	YDR012W	998
YJR145C	YDR025W	998

YJR145C	YDR064W	996
YJR145C	YDR117C	940
YJR145C	YDR299W	978
YJR145C	YDR382W	941
YJR145C	YDR385W	912
YJR145C	YDR418W	926
YJR145C	YDR447C	995
YJR145C	YDR449C	929
YJR145C	YDR450W	924
YJR145C	YDR471W	994
YJR145C	YDR496C	933
YJR145C	YDR500C	912
YJR145C	YEL054C	998
YJR145C	YER056C-A	913
YJR145C	YER074W	993
YJR145C	YER102W	996
YJR145C	YER117W	975
YJR145C	YER131W	913
YJR145C	YER165W	907
YJR145C	YFL034C-A	927
YJR145C	YFR031C-A	997
YJR145C	YFR032C-A	940
YJR145C	YGL030W	927
YJR145C	YGL076C	995
YJR145C	YGL103W	997
YJR145C	YGL120C	937
YJR145C	YGL123W	976
YJR145C	YGL135W	930
YJR145C	YGL147C	973
YJR145C	YGL173C	925
YJR145C	YGL189C	913
YJR145C	YGR027C	905
YJR145C	YGR034W	979
YJR145C	YGR085C	998
YJR145C	YGR090W	980
YJR145C	YGR118W	925
YJR145C	YGR162W	916
YJR145C	YGR214W	913
YJR145C	YHL001W	924
YJR145C	YHL015W	942
YJR145C	YHL033C	995
YJR145C	YHL034C	996
YJR145C	YHR010W	993
YJR145C	YHR021C	922

YJR145C	YHR141C	946
YJR145C	YHR148W	932
YJR145C	YHR203C	996
YJR145C	YIL018W	998
YJR145C	YIL052C	913
YJR145C	YIL069C	996
YJR145C	YIL133C	995
YJR145C	YIL148W	910
YJR145C	YJL136C	930
YJR145C	YJL177W	970
YJR145C	YJL189W	914
YJR145C	YJL190C	998
YJR145C	YJL191W	930
YJR145C	YJR002W	931
YJR145C	YJR007W	902
YJR145C	YJR094W-A	918
YJR145C	YJR123W	995
YJR145C	YKL006W	924
YJR145C	YKL156W	922
YJR145C	YKL180W	971
YJR145C	YKR057W	930
YJR145C	YKR094C	910
YJR145C	YLL045C	995
YJR145C	YLR029C	994
YJR145C	YLR048W	913
YJR145C	YLR061W	932
YJR145C	YLR075W	903
YJR145C	YLR150W	925
YJR145C	YLR167W	904
YJR145C	YLR175W	992
YJR145C	YLR185W	912
YJR145C	YLR186W	929
YJR145C	YLR197W	931
YJR145C	YLR264W	916
YJR145C	YLR287C-A	906
YJR145C	YLR333C	905
YJR145C	YLR340W	944
YJR145C	YLR344W	979
YJR145C	YLR367W	999
YJR145C	YLR388W	970
YJR145C	YLR406C	929
YJR145C	YLR441C	995
YJR145C	YLR448W	924
YJR145C	YML024W	926

YJR145C	YML026C	924
YJR145C	YML056C	980
YJR145C	YML063W	995
YJR145C	YML073C	995
YJR145C	YMR093W	931
YJR145C	YMR116C	985
YJR145C	YMR121C	914
YJR145C	YMR128W	936
YJR145C	YMR142C	917
YJR145C	YMR194W	925
YJR145C	YMR229C	936
YJR145C	YMR230W	928
YJR145C	YMR242C	995
YJR145C	YMR260C	913
YJR145C	YMR290C	940
YJR145C	YNL067W	974
YJR145C	YNL069C	995
YJR145C	YNL096C	933
YJR145C	YNL132W	970
YJR145C	YNL162W	946
YJR145C	YNL178W	999
YJR145C	YNL301C	916
YJR145C	YNL302C	921
YJR145C	YNL308C	980
YJR145C	YNR037C	939
YJR145C	YOL039W	910
YJR145C	YOL040C	982
YJR145C	YOL120C	998
YJR145C	YOL121C	922
YJR145C	YOL127W	998
YJR145C	YOL139C	916
YJR145C	YOR063W	999
YJR145C	YOR096W	995
YJR145C	YOR133W	912
YJR145C	YOR167C	916
YJR145C	YOR182C	906
YJR145C	YOR204W	931
YJR145C	YOR234C	923
YJR145C	YOR293W	928
YJR145C	YOR312C	997
YJR145C	YOR369C	923
YJR145C	YPL012W	970
YJR145C	YPL079W	943
YJR145C	YPL081W	997

YJR145C	YPL090C	996
YJR145C	YPL131W	998
YJR145C	YPL143W	923
YJR145C	YPL198W	995
YJR145C	YPL217C	929
YJR145C	YPL220W	994
YJR145C	YPL249C-A	926
YJR145C	YPR041W	915
YJR145C	YPR043W	918
YJR145C	YPR080W	922
YJR145C	YPR102C	976
YJR145C	YPR132W	925
YJR145C	YPR144C	984
YJR147W	YGR121C	922
YJR147W	YNL142W	921
YJR148W	YBR176W	905
YJR148W	YCL018W	931
YJR148W	YGR094W	913
YJR148W	YHR208W	999
YJR148W	YJR016C	959
YJR148W	YLL048C	962
YJR148W	YLR044C	927
YJR148W	YMR108W	955
YJR148W	YNL104C	901
YJR151C	YNL327W	999
YJR152W	YER040W	945
YJR159W	YDL246C	998
YJR159W	YHR104W	933
YJR159W	YPL031C	979
YKL001C	YBR118W	923
YKL001C	YJR010W	999
YKL001C	YJR137C	948
YKL001C	YLR303W	925
YKL001C	YNL311C	937
YKL001C	YPL092W	902
YKL001C	YPR080W	923
YKL001C	YPR167C	999
YKL002W	YDR069C	981
YKL002W	YEL068C	944
YKL002W	YGR035C	944
YKL002W	YKL041W	998
YKL002W	YKR035W-A	999
YKL002W	YLR025W	999
YKL002W	YLR108C	944

YKL002W	YMR077C	999
YKL002W	YNL265C	901
YKL002W	YPL084W	945
YKL002W	YPR173C	999
YKL003C	Q0140	962
YKL003C	YBR146W	986
YKL003C	YBR251W	999
YKL003C	YER153C	932
YKL003C	YGL129C	986
YKL003C	YGR084C	970
YKL003C	YGR215W	986
YKL003C	YHL004W	900
YKL003C	YPL013C	986
YKL003C	YPL118W	986
YKL004W	YBR036C	981
YKL004W	YPL057C	984
YKL005C	YJR017C	997
YKL006C-A	YHL031C	999
YKL006C-A	YIL004C	999
YKL006C-A	YKL196C	999
YKL006C-A	YLR026C	999
YKL006C-A	YLR078C	999
YKL006C-A	YLR268W	999
YKL006C-A	YMR197C	933
YKL006C-A	YOR036W	998
YKL006C-A	YOR106W	998
YKL006C-A	YOR327C	998
YKL006W	YAL003W	903
YKL006W	YAL035W	929
YKL006W	YBL027W	918
YKL006W	YBL072C	918
YKL006W	YBL087C	930
YKL006W	YBL092W	974
YKL006W	YBR031W	979
YKL006W	YBR048W	937
YKL006W	YBR084C-A	973
YKL006W	YBR118W	907
YKL006W	YBR181C	904
YKL006W	YBR189W	999
YKL006W	YBR191W	999
YKL006W	YCR031C	997
YKL006W	YDL061C	997
YKL006W	YDL075W	998
YKL006W	YDL081C	937

YKL006W	YDL082W	941
YKL006W	YDL130W	955
YKL006W	YDL136W	973
YKL006W	YDL191W	931
YKL006W	YDR012W	990
YKL006W	YDR025W	920
YKL006W	YDR064W	999
YKL006W	YDR382W	999
YKL006W	YDR385W	908
YKL006W	YDR418W	999
YKL006W	YDR447C	933
YKL006W	YDR450W	920
YKL006W	YDR471W	999
YKL006W	YDR500C	967
YKL006W	YEL054C	927
YKL006W	YER056C-A	999
YKL006W	YER074W	916
YKL006W	YER102W	976
YKL006W	YER117W	980
YKL006W	YER131W	979
YKL006W	YFL034C-A	916
YKL006W	YFR031C-A	910
YKL006W	YFR032C-A	946
YKL006W	YGL030W	999
YKL006W	YGL031C	966
YKL006W	YGL076C	978
YKL006W	YGL103W	999
YKL006W	YGL123W	999
YKL006W	YGL135W	921
YKL006W	YGL147C	939
YKL006W	YGL189C	922
YKL006W	YGR027C	998
YKL006W	YGR034W	924
YKL006W	YGR085C	996
YKL006W	YGR118W	999
YKL006W	YGR148C	999
YKL006W	YGR214W	999
YKL006W	YHL001W	999
YKL006W	YHL015W	996
YKL006W	YHL033C	999
YKL006W	YHR010W	997
YKL006W	YHR021C	927
YKL006W	YHR141C	942
YKL006W	YHR203C	978

YKL006W	YIL052C	999
YKL006W	YIL069C	916
YKL006W	YIL133C	924
YKL006W	YIL148W	966
YKL006W	YJL136C	982
YKL006W	YJL177W	999
YKL006W	YJL189W	959
YKL006W	YJL190C	936
YKL006W	YJL191W	995
YKL006W	YJR094W-A	996
YKL006W	YJR123W	999
YKL006W	YJR145C	924
YKL006W	YKL156W	996
YKL006W	YKL180W	999
YKL006W	YKL181W	942
YKL006W	YKR057W	930
YKL006W	YKR094C	905
YKL006W	YLL045C	958
YKL006W	YLR029C	998
YKL006W	YLR048W	999
YKL006W	YLR060W	917
YKL006W	YLR061W	999
YKL006W	YLR075W	998
YKL006W	YLR167W	964
YKL006W	YLR185W	971
YKL006W	YLR249W	938
YKL006W	YLR264W	999
YKL006W	YLR287C-A	902
YKL006W	YLR325C	924
YKL006W	YLR333C	953
YKL006W	YLR340W	994
YKL006W	YLR344W	998
YKL006W	YLR367W	996
YKL006W	YLR372W	942
YKL006W	YLR388W	999
YKL006W	YLR406C	918
YKL006W	YLR441C	923
YKL006W	YLR448W	999
YKL006W	YML024W	999
YKL006W	YML026C	920
YKL006W	YML063W	999
YKL006W	YML073C	972
YKL006W	YML106W	962
YKL006W	YMR121C	925

YKL006W	YMR142C	949
YKL006W	YMR194W	999
YKL006W	YMR230W	965
YKL006W	YMR242C	938
YKL006W	YMR260C	911
YKL006W	YNL067W	997
YKL006W	YNL069C	915
YKL006W	YNL096C	942
YKL006W	YNL162W	931
YKL006W	YNL178W	999
YKL006W	YNL209W	978
YKL006W	YNL301C	978
YKL006W	YNL302C	925
YKL006W	YOL039W	952
YKL006W	YOL040C	953
YKL006W	YOL120C	922
YKL006W	YOL121C	925
YKL006W	YOL127W	999
YKL006W	YOR063W	999
YKL006W	YOR096W	999
YKL006W	YOR133W	936
YKL006W	YOR167C	967
YKL006W	YOR182C	902
YKL006W	YOR234C	998
YKL006W	YOR293W	994
YKL006W	YOR312C	943
YKL006W	YOR369C	949
YKL006W	YPL037C	999
YKL006W	YPL079W	993
YKL006W	YPL081W	977
YKL006W	YPL090C	904
YKL006W	YPL131W	998
YKL006W	YPL143W	997
YKL006W	YPL198W	934
YKL006W	YPL220W	973
YKL006W	YPL249C-A	967
YKL006W	YPR043W	952
YKL006W	YPR080W	907
YKL006W	YPR102C	999
YKL006W	YPR132W	980
YKL007W	YBR109C	970
YKL007W	YDR129C	955
YKL007W	YER071C	999
YKL007W	YFL039C	975

YKL007W	YGR080W	999
YKL007W	YIL034C	999
YKL007W	YIR003W	999
YKL007W	YLL050C	975
YKL007W	YLR429W	974
YKL007W	YNL138W	978
YKL007W	YOR276W	962
YKL008C	YBR183W	993
YKL008C	YDR294C	998
YKL008C	YDR297W	964
YKL008C	YHL003C	999
YKL008C	YMR298W	999
YKL008C	YPL087W	993
YKL009W	YCR072C	972
YKL009W	YDL031W	985
YKL009W	YDR087C	976
YKL009W	YDR101C	992
YKL009W	YDR496C	966
YKL009W	YER006W	998
YKL009W	YER126C	999
YKL009W	YFL002C	978
YKL009W	YGL111W	988
YKL009W	YGL147C	926
YKL009W	YGR103W	998
YKL009W	YGR245C	986
YKL009W	YHR052W	999
YKL009W	YHR085W	972
YKL009W	YHR088W	983
YKL009W	YHR170W	954
YKL009W	YHR197W	991
YKL009W	YKR081C	999
YKL009W	YLL008W	985
YKL009W	YLL045C	954
YKL009W	YLR002C	980
YKL009W	YLR009W	923
YKL009W	YLR074C	961
YKL009W	YLR175W	949
YKL009W	YLR406C	976
YKL009W	YMR049C	988
YKL009W	YMR290C	989
YKL009W	YNL002C	987
YKL009W	YNL061W	999
YKL009W	YNL110C	996
YKL009W	YNL182C	984

YKL009W	YNR053C	993
YKL009W	YOL077C	999
YKL009W	YOR063W	942
YKL009W	YOR206W	994
YKL009W	YOR272W	997
YKL009W	YPL093W	993
YKL009W	YPL131W	958
YKL009W	YPL198W	947
YKL009W	YPL211W	993
YKL009W	YPL249C-A	934
YKL009W	YPR016C	999
YKL010C	YBR082C	958
YKL010C	YDL190C	909
YKL010C	YDR059C	915
YKL010C	YGL048C	983
YKL010C	YOR259C	908
YKL011C	YDR296W	907
YKL011C	YHR120W	901
YKL012W	YBR119W	999
YKL012W	YDL087C	999
YKL012W	YDR235W	999
YKL012W	YDR240C	999
YKL012W	YER029C	996
YKL012W	YFL017W-A	984
YKL012W	YGR013W	999
YKL012W	YGR074W	997
YKL012W	YHR086W	999
YKL012W	YHR165C	949
YKL012W	YIL061C	999
YKL012W	YLR116W	922
YKL012W	YLR117C	920
YKL012W	YLR147C	997
YKL012W	YLR275W	988
YKL012W	YLR298C	999
YKL012W	YML046W	999
YKL012W	YML049C	974
YKL012W	YMR125W	999
YKL012W	YNL187W	935
YKL012W	YOR159C	997
YKL012W	YPR182W	999
YKL013C	YBR234C	999
YKL013C	YDL029W	999
YKL013C	YIL062C	999
YKL013C	YJR065C	999

YKL013C	YKL129C	949
YKL013C	YLR370C	999
YKL013C	YLR429W	955
YKL013C	YMR109W	993
YKL013C	YNR035C	999
YKL013C	YOR181W	975
YKL014C	YBR142W	980
YKL014C	YDL208W	932
YKL014C	YDR496C	988
YKL014C	YHR066W	986
YKL014C	YHR089C	954
YKL014C	YJR041C	999
YKL014C	YLR221C	986
YKL014C	YMR290C	988
YKL014C	YNL061W	999
YKL014C	YNR038W	912
YKL014C	YOL144W	999
YKL014C	YPL043W	999
YKL015W	YGR057C	981
YKL015W	YHR037W	955
YKL015W	YLR142W	944
YKL015W	YNL229C	950
YKL016C	Q0085	957
YKL016C	YBL099W	999
YKL016C	YBR039W	961
YKL016C	YDL004W	949
YKL016C	YDL181W	937
YKL016C	YDR298C	999
YKL016C	YDR377W	973
YKL016C	YJR121W	999
YKL016C	YLR295C	961
YKL016C	YML081C-A	967
YKL016C	YPL078C	999
YKL016C	YPL271W	928
YKL016C	YPR020W	928
YKL017C	YNL102W	923
YKL017C	YOL108C	938
YKL018W	YAL043C	999
YKL018W	YAR003W	999
YKL018W	YBR175W	997
YKL018W	YBR258C	994
YKL018W	YDR195W	999
YKL018W	YDR301W	999
YKL018W	YDR469W	986

YKL018W	YER133W	999
YKL018W	YGR156W	995
YKL018W	YHR119W	999
YKL018W	YJR093C	999
YKL018W	YKL059C	999
YKL018W	YKR002W	998
YKL018W	YLR015W	999
YKL018W	YLR115W	999
YKL018W	YLR277C	999
YKL018W	YLR448W	978
YKL018W	YMR061W	992
YKL018W	YNL222W	997
YKL018W	YNL317W	999
YKL018W	YOR179C	999
YKL018W	YPL138C	999
YKL018W	YPR107C	999
YKL019W	YDL090C	999
YKL019W	YGL155W	999
YKL020C	YBR170C	998
YKL020C	YDL126C	984
YKL020C	YER125W	999
YKL020C	YGL055W	991
YKL020C	YGR048W	918
YKL020C	YIR033W	998
YKL021C	YAL025C	942
YKL021C	YGL111W	962
YKL021C	YGR103W	989
YKL021C	YHR052W	948
YKL021C	YLR009W	976
YKL021C	YMR049C	979
YKL021C	YMR290C	963
YKL021C	YNL061W	977
YKL021C	YOR272W	957
YKL021C	YPL093W	913
YKL021C	YPR016C	997
YKL022C	YBL084C	999
YKL022C	YBR082C	928
YKL022C	YBR135W	929
YKL022C	YBR160W	999
YKL022C	YDL008W	999
YKL022C	YDL028C	929
YKL022C	YDR113C	998
YKL022C	YDR118W	999
YKL022C	YDR260C	999

YKL022C	YFR036W	999
YKL022C	YGL003C	998
YKL022C	YGL116W	996
YKL022C	YGL190C	907
YKL022C	YGL240W	999
YKL022C	YHR166C	999
YKL022C	YIR025W	999
YKL022C	YJL030W	995
YKL022C	YLL039C	904
YKL022C	YLR102C	999
YKL022C	YLR127C	999
YKL022C	YMR001C	999
YKL022C	YNL172W	999
YKL022C	YOR249C	999
YKL022C	YOR339C	902
YKL022C	YOR360C	973
YKL022C	YPR119W	988
YKL023W	YGL173C	990
YKL023W	YLR398C	993
YKL023W	YPR189W	987
YKL024C	YEL021W	912
YKL024C	YER070W	900
YKL024C	YIL066C	900
YKL024C	YJR057W	984
YKL024C	YKL067W	911
YKL024C	YLR359W	904
YKL024C	YNR012W	945
YKL025C	YDL101C	983
YKL025C	YER165W	939
YKL025C	YGL094C	999
YKL026C	YIR037W	970
YKL026C	YLL060C	938
YKL026C	YOL049W	907
YKL026C	YPL091W	984
YKL028W	YBR154C	919
YKL028W	YDL005C	984
YKL028W	YDL140C	938
YKL028W	YDR167W	902
YKL028W	YDR311W	996
YKL028W	YER007C-A	931
YKL028W	YER148W	905
YKL028W	YGL025C	984
YKL028W	YGL070C	997
YKL028W	YGL112C	904

YKL028W	YGL207W	908
YKL028W	YGR186W	984
YKL028W	YHR143W-A	918
YKL028W	YIL021W	978
YKL028W	YIL143C	917
YKL028W	YKL058W	910
YKL028W	YKR018C	929
YKL028W	YKR062W	999
YKL028W	YOL051W	999
YKL028W	YOR151C	948
YKL028W	YPR086W	925
YKL029C	YAL038W	984
YKL029C	YDL078C	907
YKL029C	YDL143W	975
YKL029C	YDR188W	973
YKL029C	YER086W	967
YKL029C	YFR009W	972
YKL029C	YGL195W	974
YKL029C	YIR031C	907
YKL029C	YKL085W	924
YKL029C	YKL104C	973
YKL029C	YLR106C	972
YKL029C	YMR012W	976
YKL029C	YNL117W	907
YKL029C	YOL126C	901
YKL029C	YOR347C	975
YKL032C	YDR510W	942
YKL032C	YMR201C	901
YKL033W	YGR099W	966
YKL033W	YJR136C	999
YKL033W	YPR085C	973
YKL034W	YBR290W	939
YKL034W	YER125W	950
YKL035W	YBR018C	960
YKL035W	YBR019C	931
YKL035W	YBR126C	932
YKL035W	YDR074W	934
YKL035W	YFR015C	922
YKL035W	YGR032W	900
YKL035W	YKL127W	901
YKL035W	YLR258W	948
YKL035W	YLR342W	900
YKL035W	YMR105C	932
YKL035W	YMR261C	911

YKL035W	YOL045W	982
YKL035W	YPL031C	928
YKL035W	YPR160W	928
YKL037W	YPL240C	919
YKL038W	YBR112C	996
YKL038W	YDL194W	999
YKL038W	YDR277C	976
YKL038W	YGL253W	986
YKL038W	YHR094C	941
YKL038W	YJL164C	928
YKL038W	YJR090C	999
YKL038W	YKL166C	948
YKL038W	YOR047C	999
YKL038W	YPL203W	930
YKL040C	YLR369W	968
YKL040C	YPL135W	976
YKL041W	YCL008C	907
YKL041W	YDR069C	995
YKL041W	YDR486C	999
YKL041W	YKL002W	998
YKL041W	YKR035W-A	995
YKL041W	YLR025W	999
YKL041W	YMR077C	999
YKL041W	YPL002C	996
YKL041W	YPL065W	971
YKL041W	YPL084W	921
YKL041W	YPR173C	981
YKL042W	YBR160W	971
YKL042W	YDL028C	988
YKL042W	YDR356W	999
YKL042W	YJL019W	913
YKL042W	YNL225C	999
YKL042W	YPL124W	988
YKL042W	YPR120C	994
YKL045W	YAR007C	948
YKL045W	YBL023C	901
YKL045W	YBL035C	999
YKL045W	YBR088C	904
YKL045W	YBR202W	906
YKL045W	YDL102W	901
YKL045W	YGL201C	901
YKL045W	YGL207W	981
YKL045W	YHR164C	930
YKL045W	YIL150C	997

YKL045W	YIR008C	999
YKL045W	YJR043C	905
YKL045W	YKL113C	926
YKL045W	YLR274W	907
YKL045W	YML069W	981
YKL045W	YNL102W	999
YKL045W	YNL262W	984
YKL045W	YNR052C	987
YKL046C	YFR032C-A	958
YKL048C	YBR160W	992
YKL048C	YDR422C	946
YKL048C	YDR477W	983
YKL048C	YDR507C	985
YKL048C	YER027C	960
YKL048C	YER129W	999
YKL048C	YGL179C	982
YKL048C	YGL190C	905
YKL048C	YGL208W	928
YKL048C	YGR229C	920
YKL048C	YHR107C	973
YKL048C	YJL187C	990
YKL048C	YJR076C	914
YKL048C	YNL298W	900
YKL049C	YAL034W-A	989
YKL049C	YBR009C	999
YKL049C	YBR010W	968
YKL049C	YDL139C	999
YKL049C	YDR318W	924
YKL049C	YGL060W	901
YKL049C	YGR113W	967
YKL049C	YGR140W	993
YKL049C	YGR179C	995
YKL049C	YIL144W	925
YKL049C	YJR060W	997
YKL049C	YJR112W	953
YKL049C	YKL089W	999
YKL049C	YLR381W	921
YKL049C	YMR094W	939
YKL049C	YMR168C	958
YKL049C	YPL018W	999
YKL051W	YLR305C	936
YKL052C	YBR233W-A	961
YKL052C	YDR016C	999
YKL052C	YDR201W	999

YKL052C	YDR320C-A	998
YKL052C	YGL061C	999
YKL052C	YGR113W	999
YKL052C	YGR140W	978
YKL052C	YIL144W	982
YKL052C	YKL138C-A	999
YKL052C	YKR037C	999
YKL052C	YKR083C	999
YKL054C	YJR035W	955
YKL055C	YER061C	948
YKL057C	YAL009W	918
YKL057C	YAR002W	963
YKL057C	YBL079W	989
YKL057C	YDL088C	947
YKL057C	YDL116W	999
YKL057C	YDL207W	962
YKL057C	YDR159W	973
YKL057C	YDR192C	988
YKL057C	YER105C	959
YKL057C	YER107C	929
YKL057C	YER110C	928
YKL057C	YFR002W	991
YKL057C	YGL086W	931
YKL057C	YGL092W	999
YKL057C	YGL100W	999
YKL057C	YGL172W	993
YKL057C	YGR119C	983
YKL057C	YHR004C	918
YKL057C	YIL063C	984
YKL057C	YIL115C	995
YKL057C	YJL041W	961
YKL057C	YJL061W	998
YKL057C	YJR042W	999
YKL057C	YKL068W	999
YKL057C	YKL186C	958
YKL057C	YKR082W	999
YKL057C	YLR018C	995
YKL057C	YLR208W	999
YKL057C	YLR335W	980
YKL057C	YML103C	993
YKL057C	YML107C	989
YKL057C	YMR047C	998
YKL057C	YMR129W	973
YKL057C	YMR153W	954

YKL057C	YOL072W	967
YKL057C	YOR257W	961
YKL057C	YPL020C	982
YKL057C	YPL169C	994
YKL058W	YBR198C	982
YKL058W	YDL140C	932
YKL058W	YDR167W	939
YKL058W	YDR392W	979
YKL058W	YDR404C	904
YKL058W	YER148W	999
YKL058W	YER171W	907
YKL058W	YGL016W	919
YKL058W	YGL070C	917
YKL058W	YGR274C	984
YKL058W	YIL021W	902
YKL058W	YIL143C	947
YKL058W	YKL028W	910
YKL058W	YML015C	998
YKL058W	YML098W	930
YKL058W	YMR005W	979
YKL058W	YOL051W	959
YKL058W	YOL135C	902
YKL058W	YOR194C	999
YKL058W	YOR210W	904
YKL058W	YOR224C	902
YKL058W	YPR056W	921
YKL058W	YPR086W	998
YKL058W	YPR187W	904
YKL059C	YAL043C	999
YKL059C	YDR195W	999
YKL059C	YDR228C	998
YKL059C	YDR301W	999
YKL059C	YER133W	999
YKL059C	YGR156W	999
YKL059C	YJR093C	999
YKL059C	YKL018W	999
YKL059C	YKR002W	999
YKL059C	YLR115W	999
YKL059C	YLR277C	999
YKL059C	YMR061W	978
YKL059C	YNL222W	994
YKL059C	YNL317W	999
YKL059C	YPR107C	999
YKL060C	YAL038W	944

YKL060C	YBR117C	933
YKL060C	YCR012W	999
YKL060C	YDR050C	999
YKL060C	YDR385W	968
YKL060C	YGR192C	999
YKL060C	YGR240C	949
YKL060C	YHR174W	997
YKL060C	YJL052W	940
YKL060C	YJR009C	972
YKL060C	YKL152C	991
YKL060C	YLR044C	978
YKL060C	YLR377C	985
YKL060C	YMR205C	947
YKL060C	YOL086C	968
YKL060C	YOR133W	970
YKL060C	YPR074C	937
YKL061W	YDR357C	992
YKL061W	YNL086W	995
YKL062W	YDR028C	942
YKL062W	YDR099W	996
YKL062W	YFL033C	997
YKL062W	YGL073W	992
YKL062W	YHR205W	943
YKL062W	YIL033C	916
YKL062W	YIL052C	923
YKL062W	YJL141C	959
YKL062W	YLR113W	954
YKL062W	YLR403W	929
YKL062W	YMR037C	999
YKL062W	YNL098C	993
YKL062W	YPL203W	982
YKL065C	YFR048W	998
YKL065C	YPL019C	998
YKL067W	YBL039C	921
YKL067W	YBR252W	908
YKL067W	YDR226W	951
YKL067W	YDR454C	906
YKL067W	YER070W	913
YKL067W	YER170W	933
YKL067W	YGR209C	910
YKL067W	YGR267C	925
YKL067W	YIL066C	909
YKL067W	YJL026W	904
YKL067W	YJR057W	970

YKL067W	YJR103W	922
YKL067W	YKL024C	911
YKL067W	YKL144C	931
YKL067W	YLR043C	910
YKL067W	YMR203W	965
YKL067W	YOL005C	902
YKL067W	YOR224C	922
YKL067W	YPR010C	995
YKL068W	YAR002W	948
YKL068W	YBL079W	994
YKL068W	YBR017C	982
YKL068W	YBR123C	913
YKL068W	YDL088C	983
YKL068W	YDL116W	999
YKL068W	YDL207W	999
YKL068W	YDR101C	984
YKL068W	YDR159W	969
YKL068W	YDR192C	988
YKL068W	YDR335W	948
YKL068W	YDR395W	913
YKL068W	YDR465C	918
YKL068W	YER105C	956
YKL068W	YER107C	999
YKL068W	YER110C	990
YKL068W	YER165W	913
YKL068W	YFR002W	995
YKL068W	YFR031C	913
YKL068W	YGL092W	999
YKL068W	YGL172W	986
YKL068W	YGR119C	999
YKL068W	YGR218W	913
YKL068W	YJL061W	986
YKL068W	YJR042W	999
YKL068W	YKL057C	999
YKL068W	YKR082W	997
YKL068W	YLL024C	913
YKL068W	YLR335W	993
YKL068W	YLR347C	999
YKL068W	YML103C	993
YKL068W	YMR047C	999
YKL068W	YMR129W	928
YKL068W	YMR308C	990
YKL068W	YNL189W	913
YKL068W	YNL273W	913

YKL068W	YOL123W	913
YKL068W	YOR098C	994
YKL068W	YOR257W	961
YKL068W	YPL125W	969
YKL068W	YPL169C	998
YKL070W	YDL146W	904
YKL072W	YOL004W	973
YKL073W	YFL031W	903
YKL073W	YGL200C	911
YKL073W	YHR079C	977
YKL073W	YJL034W	999
YKL073W	YMR214W	980
YKL073W	YOL031C	996
YKL074C	YBR172C	968
YKL074C	YDL043C	928
YKL074C	YDL084W	997
YKL074C	YDR240C	984
YKL074C	YGL174W	969
YKL074C	YIR005W	963
YKL074C	YLR116W	999
YKL074C	YLR117C	999
YKL074C	YMR240C	904
YKL074C	YMR288W	933
YKL074C	YNL138W-A	919
YKL074C	YPL178W	952
YKL075C	YNL091W	990
YKL078W	YNL186W	996
YKL079W	YFL005W	991
YKL079W	YIL159W	907
YKL079W	YNL272C	956
YKL079W	YOR326W	999
YKL080W	YBR127C	999
YKL080W	YDL185W	999
YKL080W	YEL027W	920
YKL080W	YEL051W	999
YKL080W	YGR020C	909
YKL080W	YHR026W	939
YKL080W	YHR039C-A	954
YKL080W	YJR033C	915
YKL080W	YLR447C	973
YKL080W	YMR054W	999
YKL080W	YOR270C	999
YKL080W	YOR332W	999
YKL080W	YPL234C	924

YKL080W	YPR036W	915
YKL081W	YAL003W	999
YKL081W	YBR025C	991
YKL081W	YBR118W	994
YKL081W	YCR042C	919
YKL081W	YLR249W	996
YKL081W	YPL048W	996
YKL081W	YPR080W	999
YKL082C	YDR309C	940
YKL082C	YHR061C	934
YKL082C	YHR066W	933
YKL082C	YKL099C	916
YKL082C	YKL172W	945
YKL082C	YML109W	951
YKL084W	YGR029W	902
YKL085W	YBR218C	961
YKL085W	YCR005C	992
YKL085W	YGL062W	958
YKL085W	YIR031C	905
YKL085W	YKL029C	924
YKL085W	YKL106W	986
YKL085W	YKR097W	951
YKL085W	YLR027C	932
YKL085W	YLR377C	901
YKL085W	YNL117W	930
YKL085W	YNR001C	997
YKL085W	YOL126C	995
YKL085W	YPL262W	990
YKL085W	YPR001W	901
YKL086W	YDR453C	911
YKL086W	YML028W	956
YKL087C	YOR037W	920
YKL087C	YOR065W	967
YKL088W	YGR090W	956
YKL088W	YIL035C	999
YKL088W	YIL083C	915
YKL088W	YKR072C	999
YKL088W	YML016C	993
YKL088W	YOR039W	999
YKL088W	YOR054C	999
YKL088W	YOR061W	999
YKL089W	YAL034W-A	980
YKL089W	YDR254W	978
YKL089W	YDR318W	998

YKL089W	YGR140W	998
YKL089W	YIR010W	995
YKL089W	YJR060W	997
YKL089W	YJR112W	980
YKL089W	YKL049C	999
YKL089W	YLR381W	964
YKL089W	YMR094W	989
YKL089W	YMR168C	998
YKL089W	YPL233W	981
YKL089W	YPR046W	964
YKL091C	YMR079W	942
YKL092C	YCR038C	968
YKL092C	YGR152C	995
YKL092C	YPL256C	981
YKL093W	YHR205W	903
YKL093W	YKL212W	905
YKL094W	YBR041W	917
YKL095W	YAL032C	980
YKL095W	YDR364C	980
YKL095W	YDR416W	998
YKL095W	YGL120C	980
YKL095W	YGR278W	929
YKL095W	YKL173W	953
YKL095W	YLL036C	998
YKL095W	YLR117C	996
YKL095W	YLR424W	940
YKL095W	YMR213W	948
YKL095W	YNL229C	902
YKL095W	YPL151C	980
YKL095W	YPR101W	997
YKL096W	YKL096W-A	958
YKL096W	YNL012W	908
YKL096W-A	YKL096W	958
YKL099C	YBR247C	958
YKL099C	YCL059C	914
YKL099C	YCR057C	992
YKL099C	YGR128C	912
YKL099C	YIL019W	939
YKL099C	YJL069C	953
YKL099C	YJR002W	986
YKL099C	YKL082C	916
YKL099C	YKR060W	934
YKL099C	YLR003C	909
YKL099C	YLR175W	911

YKL099C	YMR290C	910
YKL099C	YNL075W	913
YKL099C	YOR004W	909
YKL099C	YOR145C	913
YKL099C	YOR310C	908
YKL101W	YBR133C	995
YKL101W	YBR160W	982
YKL101W	YCL024W	919
YKL101W	YDR477W	908
YKL101W	YDR507C	987
YKL101W	YGL003C	999
YKL101W	YGL116W	996
YKL101W	YHR107C	998
YKL101W	YJL187C	999
YKL101W	YJR076C	987
YKL101W	YLR314C	997
YKL101W	YMR001C	976
YKL101W	YMR273C	909
YKL101W	YNL298W	951
YKL103C	YBL078C	911
YKL103C	YDR022C	951
YKL103C	YGL156W	963
YKL103C	YHR113W	974
YKL103C	YOL082W	999
YKL103C	YPR049C	950
YKL104C	YDL171C	924
YKL104C	YDR127W	992
YKL104C	YDR188W	970
YKL104C	YFL017C	988
YKL104C	YFR009W	966
YKL104C	YGL195W	999
YKL104C	YHR020W	972
YKL104C	YJR109C	921
YKL104C	YKL029C	973
YKL104C	YLR180W	975
YKL104C	YMR084W	902
YKL104C	YMR217W	922
YKL104C	YMR300C	912
YKL104C	YOR151C	973
YKL104C	YOR303W	922
YKL104C	YOR370C	906
YKL104C	YPR010C	972
YKL104C	YPR035W	921
YKL106W	YBR218C	903

YKL106W	YDL078C	902
YKL106W	YGL062W	903
YKL106W	YIL155C	902
YKL106W	YJL130C	917
YKL106W	YKL085W	986
YKL106W	YKR097W	905
YKL106W	YPR035W	914
YKL108W	YBR160W	956
YKL108W	YFR028C	906
YKL108W	YJL087C	901
YKL108W	YJL090C	999
YKL108W	YPR120C	996
YKL109W	YBL021C	997
YKL109W	YGL237C	997
YKL109W	YOR290C	967
YKL109W	YOR358W	999
YKL110C	YAL038W	940
YKL110C	YDL047W	941
YKL110C	YDL140C	992
YKL110C	YGR200C	999
YKL110C	YGR252W	930
YKL110C	YHR187W	999
YKL110C	YLR384C	996
YKL110C	YMR312W	924
YKL110C	YPL086C	999
YKL110C	YPL101W	995
YKL112W	YBR114W	902
YKL112W	YNL216W	926
YKL113C	YAR002W	965
YKL113C	YBR088C	999
YKL113C	YBR098W	955
YKL113C	YCL061C	943
YKL113C	YCR014C	928
YKL113C	YDL059C	994
YKL113C	YDL102W	999
YKL113C	YDL164C	940
YKL113C	YDR004W	989
YKL113C	YDR075W	910
YKL113C	YDR076W	988
YKL113C	YDR217C	999
YKL113C	YDR369C	993
YKL113C	YDR386W	957
YKL113C	YER095W	999
YKL113C	YER171W	919

YKL113C	YER173W	999
YKL113C	YGL163C	989
YKL113C	YGL175C	984
YKL113C	YGR258C	948
YKL113C	YHR164C	999
YKL113C	YIR008C	920
YKL113C	YJL092W	999
YKL113C	YJR006W	960
YKL113C	YJR043C	990
YKL113C	YJR104C	901
YKL113C	YKL045W	926
YKL113C	YKR082W	990
YKL113C	YML028W	910
YKL113C	YML032C	999
YKL113C	YML095C	938
YKL113C	YML102W	909
YKL113C	YMR048W	943
YKL113C	YMR078C	949
YKL113C	YMR190C	998
YKL113C	YMR224C	999
YKL113C	YNL102W	942
YKL113C	YNL250W	999
YKL113C	YNL262W	983
YKL113C	YOL090W	929
YKL113C	YOL115W	952
YKL113C	YOR033C	998
YKL113C	YOR144C	999
YKL113C	YOR217W	960
YKL113C	YOR368W	988
YKL113C	YPL020C	993
YKL113C	YPL022W	989
YKL113C	YPL194W	968
YKL113C	YPR135W	927
YKL114C	YAL015C	993
YKL114C	YBL019W	999
YKL114C	YBR098W	900
YKL114C	YBR223C	964
YKL114C	YBR252W	988
YKL114C	YER142C	988
YKL114C	YER162C	921
YKL114C	YGR258C	904
YKL114C	YIR002C	954
YKL114C	YML021C	996
YKL114C	YML032C	989

YKL114C	YML095C	999
YKL114C	YMR156C	997
YKL114C	YMR201C	998
YKL114C	YOL043C	970
YKL114C	YPL022W	999
YKL114C	YPL167C	935
YKL117W	YBR155W	950
YKL117W	YDR168W	966
YKL117W	YGR123C	967
YKL117W	YLR216C	999
YKL117W	YMR186W	999
YKL117W	YOR027W	996
YKL117W	YPL240C	999
YKL119C	YAL042W	920
YKL119C	YHR060W	997
YKL119C	YLR447C	994
YKL119C	YOR270C	993
YKL122C	YDL051W	994
YKL122C	YDL092W	999
YKL122C	YML105C	999
YKL122C	YPL210C	999
YKL122C	YPL243W	999
YKL122C	YPR088C	999
YKL124W	YDL212W	990
YKL125W	YBL014C	993
YKL125W	YJL025W	940
YKL125W	YLR175W	986
YKL125W	YML126C	931
YKL125W	YNL102W	976
YKL125W	YNL248C	983
YKL125W	YOR340C	987
YKL125W	YOR341W	984
YKL125W	YPR010C	984
YKL126W	YBR181C	980
YKL126W	YDR490C	999
YKL126W	YJR066W	963
YKL126W	YKL203C	963
YKL126W	YMR104C	999
YKL126W	YMR186W	971
YKL126W	YOL100W	980
YKL126W	YPL240C	991
YKL127W	YBR196C	913
YKL127W	YDL079C	927
YKL127W	YKL035W	901

YKL127W	YMR105C	984
YKL127W	YMR139W	902
YKL127W	YNL307C	900
YKL127W	YOL128C	927
YKL127W	YPR160W	948
YKL128C	YDR074W	912
YKL128C	YLR028C	910
YKL128C	YMR120C	907
YKL129C	YBR109C	994
YKL129C	YCR094W	900
YKL129C	YDL029W	973
YKL129C	YFL039C	959
YKL129C	YHR114W	936
YKL129C	YIL156W	935
YKL129C	YIL159W	935
YKL129C	YJL020C	987
YKL129C	YKL013C	949
YKL129C	YLR337C	999
YKL129C	YMR109W	999
YKL129C	YNL298W	982
YKL129C	YOR035C	999
YKL129C	YOR181W	999
YKL130C	YAL029C	999
YKL130C	YBR086C	989
YKL130C	YBR130C	999
YKL130C	YDR496C	916
YKL130C	YGL106W	992
YKL130C	YJL048C	900
YKL130C	YKL185W	999
YKL130C	YMR314W	921
YKL130C	YOR035C	906
YKL130C	YOR326W	948
YKL134C	YDL120W	980
YKL135C	YDR123C	982
YKL135C	YDR153C	960
YKL135C	YGL206C	998
YKL135C	YHL019C	999
YKL135C	YHR068W	998
YKL135C	YLR170C	999
YKL135C	YOL108C	999
YKL135C	YPL240C	919
YKL135C	YPL259C	999
YKL135C	YPR029C	999
YKL138C-A	YBR211C	963

YKL138C-A	YBR233W-A	978
YKL138C-A	YDR016C	991
YKL138C-A	YDR201W	996
YKL138C-A	YDR320C-A	991
YKL138C-A	YGL061C	996
YKL138C-A	YGR113W	999
YKL138C-A	YKL052C	999
YKL138C-A	YKR037C	999
YKL138C-A	YKR083C	992
YKL139W	YBR279W	914
YKL139W	YCL011C	979
YKL139W	YDL074C	952
YKL139W	YDL140C	997
YKL139W	YDR477W	900
YKL139W	YFL029C	921
YKL139W	YGL043W	909
YKL139W	YGL058W	935
YKL139W	YGL123W	989
YKL139W	YGL244W	995
YKL139W	YGR063C	901
YKL139W	YJL006C	999
YKL139W	YLR226W	931
YKL139W	YLR418C	994
YKL139W	YML010W	919
YKL139W	YML112W	999
YKL139W	YNL004W	981
YKL139W	YOL145C	978
YKL139W	YOR123C	971
YKL139W	YPL055C	959
YKL139W	YPL086C	915
YKL139W	YPR161C	961
YKL140W	YBR041W	960
YKL140W	YHR072W	946
YKL141W	YBR044C	925
YKL141W	YDR178W	999
YKL141W	YJL045W	973
YKL141W	YKL148C	997
YKL141W	YLL041C	990
YKL141W	YMR118C	903
YKL141W	YPL262W	939
YKL142W	YDR115W	959
YKL142W	YGR086C	999
YKL142W	YMR210W	958
YKL142W	YPL004C	999

YKL143W	YBR077C	957
YKL143W	YBR247C	999
YKL143W	YDL060W	999
YKL143W	YGR163W	952
YKL143W	YGR218W	999
YKL143W	YIL063C	925
YKL143W	YKR007W	955
YKL143W	YML121W	989
YKL143W	YNL178W	999
YKL143W	YNL207W	999
YKL143W	YPL204W	953
YKL143W	YPL239W	976
YKL143W	YPL266W	970
YKL144C	YBL034C	940
YKL144C	YBR154C	999
YKL144C	YDL145C	940
YKL144C	YDL150W	999
YKL144C	YDR005C	941
YKL144C	YDR045C	998
YKL144C	YDR156W	921
YKL144C	YHR143W-A	960
YKL144C	YJL011C	999
YKL144C	YJL148W	930
YKL144C	YJR063W	921
YKL144C	YKL067W	931
YKL144C	YKR025W	999
YKL144C	YNL113W	999
YKL144C	YNL151C	999
YKL144C	YNL201C	940
YKL144C	YNL248C	908
YKL144C	YNR003C	999
YKL144C	YOR116C	999
YKL144C	YOR207C	999
YKL144C	YOR210W	998
YKL144C	YOR224C	999
YKL144C	YOR340C	937
YKL144C	YOR341W	921
YKL144C	YPR010C	921
YKL144C	YPR110C	999
YKL144C	YPR187W	999
YKL144C	YPR190C	999
YKL145W	YBL041W	904
YKL145W	YCR014C	970
YKL145W	YDL007W	999

YKL145W	YDL013W	970
YKL145W	YDL097C	999
YKL145W	YDL147W	999
YKL145W	YDR363W-A	999
YKL145W	YDR394W	999
YKL145W	YDR427W	999
YKL145W	YEL037C	999
YKL145W	YER012W	999
YKL145W	YER021W	999
YKL145W	YER094C	912
YKL145W	YFR004W	999
YKL145W	YFR010W	999
YKL145W	YFR050C	912
YKL145W	YFR052W	999
YKL145W	YGL004C	967
YKL145W	YGL048C	999
YKL145W	YGR218W	973
YKL145W	YGR232W	999
YKL145W	YHL030W	998
YKL145W	YHR027C	999
YKL145W	YHR200W	999
YKL145W	YIL007C	903
YKL145W	YIL075C	999
YKL145W	YJL001W	920
YKL145W	YJL194W	978
YKL145W	YLL039C	988
YKL145W	YLR024C	913
YKL145W	YLR421C	998
YKL145W	YML092C	950
YKL145W	YMR191W	987
YKL145W	YMR276W	993
YKL145W	YMR314W	997
YKL145W	YOL038W	994
YKL145W	YOR056C	908
YKL145W	YOR117W	999
YKL145W	YOR157C	906
YKL145W	YOR259C	999
YKL145W	YOR261C	999
YKL145W	YPR103W	961
YKL145W	YPR108W	999
YKL146W	YCL038C	906
YKL148C	YDR178W	995
YKL148C	YGR244C	959
YKL148C	YJL045W	997

YKL148C	YKL141W	997
YKL148C	YLL041C	999
YKL148C	YLR164W	912
YKL148C	YMR118C	928
YKL148C	YOR142W	951
YKL148C	YPL262W	900
YKL149C	YGR093W	998
YKL151C	YNL200C	919
YKL152C	YCR012W	915
YKL152C	YDR385W	970
YKL152C	YGR192C	999
YKL152C	YGR254W	938
YKL152C	YHR165C	976
YKL152C	YHR174W	942
YKL152C	YKL060C	991
YKL152C	YML028W	957
YKL152C	YMR323W	906
YKL152C	YOR393W	907
YKL154W	YDR292C	999
YKL155C	Q0140	992
YKL155C	YBL090W	988
YKL155C	YBR146W	997
YKL155C	YBR251W	999
YKL155C	YDR036C	999
YKL155C	YDR041W	999
YKL155C	YDR175C	997
YKL155C	YDR337W	997
YKL155C	YDR347W	997
YKL155C	YER050C	918
YKL155C	YGL129C	999
YKL155C	YGR084C	974
YKL155C	YGR165W	988
YKL155C	YGR215W	997
YKL155C	YHL004W	999
YKL155C	YIL093C	997
YKL155C	YJR101W	997
YKL155C	YJR113C	997
YKL155C	YMR158W	974
YKL155C	YMR188C	988
YKL155C	YNL137C	997
YKL155C	YNL306W	988
YKL155C	YNR037C	997
YKL155C	YOR158W	997
YKL155C	YOR204W	984

YKL155C	YPL013C	996
YKL155C	YPL118W	997
YKL155C	YPL132W	933
YKL156W	YAL035W	918
YKL156W	YBL027W	921
YKL156W	YBL072C	931
YKL156W	YBL087C	930
YKL156W	YBL092W	935
YKL156W	YBR031W	942
YKL156W	YBR048W	930
YKL156W	YBR079C	916
YKL156W	YBR084C-A	962
YKL156W	YBR118W	908
YKL156W	YBR181C	903
YKL156W	YBR189W	995
YKL156W	YBR191W	998
YKL156W	YCR031C	975
YKL156W	YDL061C	980
YKL156W	YDL075W	977
YKL156W	YDL081C	926
YKL156W	YDL082W	936
YKL156W	YDL130W	923
YKL156W	YDL136W	981
YKL156W	YDL191W	927
YKL156W	YDR012W	962
YKL156W	YDR025W	918
YKL156W	YDR064W	997
YKL156W	YDR382W	974
YKL156W	YDR385W	912
YKL156W	YDR418W	969
YKL156W	YDR447C	929
YKL156W	YDR450W	918
YKL156W	YDR471W	974
YKL156W	YDR500C	973
YKL156W	YEL054C	930
YKL156W	YER025W	954
YKL156W	YER056C-A	993
YKL156W	YER074W	918
YKL156W	YER102W	964
YKL156W	YER117W	967
YKL156W	YER131W	959
YKL156W	YER165W	925
YKL156W	YFL034C-A	928
YKL156W	YFR031C-A	904

YKL156W	YFR032C-A	951
YKL156W	YGL030W	996
YKL156W	YGL031C	935
YKL156W	YGL076C	956
YKL156W	YGL103W	993
YKL156W	YGL123W	969
YKL156W	YGL135W	920
YKL156W	YGL147C	928
YKL156W	YGL189C	921
YKL156W	YGR027C	980
YKL156W	YGR034W	925
YKL156W	YGR085C	960
YKL156W	YGR118W	992
YKL156W	YGR148C	970
YKL156W	YGR162W	911
YKL156W	YGR214W	977
YKL156W	YHL001W	995
YKL156W	YHL015W	979
YKL156W	YHL033C	974
YKL156W	YHR010W	975
YKL156W	YHR021C	901
YKL156W	YHR141C	943
YKL156W	YHR203C	962
YKL156W	YIL018W	904
YKL156W	YIL052C	997
YKL156W	YIL069C	918
YKL156W	YIL133C	924
YKL156W	YIL148W	976
YKL156W	YJL136C	969
YKL156W	YJL177W	977
YKL156W	YJL189W	941
YKL156W	YJL190C	921
YKL156W	YJL191W	986
YKL156W	YJR007W	924
YKL156W	YJR094W-A	986
YKL156W	YJR123W	988
YKL156W	YJR145C	922
YKL156W	YKL006W	996
YKL156W	YKL180W	994
YKL156W	YKR057W	937
YKL156W	YKR059W	947
YKL156W	YKR094C	905
YKL156W	YLL045C	932
YKL156W	YLR029C	962

YKL156W	YLR048W	991
YKL156W	YLR061W	999
YKL156W	YLR075W	969
YKL156W	YLR167W	954
YKL156W	YLR185W	960
YKL156W	YLR264W	998
YKL156W	YLR287C-A	905
YKL156W	YLR325C	931
YKL156W	YLR333C	933
YKL156W	YLR340W	969
YKL156W	YLR344W	978
YKL156W	YLR367W	968
YKL156W	YLR388W	999
YKL156W	YLR406C	922
YKL156W	YLR441C	928
YKL156W	YLR448W	994
YKL156W	YML024W	996
YKL156W	YML026C	918
YKL156W	YML063W	984
YKL156W	YML073C	929
YKL156W	YMR121C	917
YKL156W	YMR142C	938
YKL156W	YMR194W	996
YKL156W	YMR230W	949
YKL156W	YMR242C	931
YKL156W	YMR260C	926
YKL156W	YNL067W	980
YKL156W	YNL069C	922
YKL156W	YNL096C	921
YKL156W	YNL162W	936
YKL156W	YNL178W	984
YKL156W	YNL301C	964
YKL156W	YNL302C	927
YKL156W	YOL039W	915
YKL156W	YOL040C	955
YKL156W	YOL120C	922
YKL156W	YOL121C	927
YKL156W	YOL127W	995
YKL156W	YOR063W	978
YKL156W	YOR096W	979
YKL156W	YOR133W	925
YKL156W	YOR167C	954
YKL156W	YOR182C	905
YKL156W	YOR234C	999

YKL156W	YOR293W	982
YKL156W	YOR312C	953
YKL156W	YOR361C	915
YKL156W	YOR369C	934
YKL156W	YPL079W	961
YKL156W	YPL081W	947
YKL156W	YPL090C	903
YKL156W	YPL131W	964
YKL156W	YPL143W	991
YKL156W	YPL198W	930
YKL156W	YPL220W	963
YKL156W	YPL237W	932
YKL156W	YPL249C-A	959
YKL156W	YPR043W	955
YKL156W	YPR080W	908
YKL156W	YPR102C	977
YKL156W	YPR132W	974
YKL156W	YPR163C	912
YKL157W	YFL052W	980
YKL157W	YGR148C	980
YKL160W	YGL019W	987
YKL160W	YIL035C	987
YKL160W	YLR418C	968
YKL160W	YOR039W	939
YKL160W	YOR061W	941
YKL160W	YOR123C	903
YKL161C	YHR030C	977
YKL164C	YJL158C	977
YKL164C	YJL159W	982
YKL165C	YJR013W	931
YKL165C	YNL169C	922
YKL166C	YHR214W-A	905
YKL166C	YIL033C	999
YKL166C	YJL164C	999
YKL166C	YKL038W	948
YKL166C	YML001W	927
YKL166C	YNL093W	917
YKL166C	YNL098C	999
YKL166C	YNL227C	900
YKL166C	YPL203W	999
YKL167C	YBL038W	972
YKL167C	YBR122C	972
YKL167C	YCR046C	992
YKL167C	YCR071C	972

YKL167C	YDR116C	972
YKL167C	YDR237W	974
YKL167C	YDR296W	993
YKL167C	YDR322W	974
YKL167C	YDR405W	988
YKL167C	YDR462W	972
YKL167C	YGR220C	998
YKL167C	YJL063C	972
YKL167C	YKR006C	989
YKL167C	YKR085C	972
YKL167C	YLR312W-A	972
YKL167C	YLR439W	974
YKL167C	YML025C	997
YKL167C	YMR024W	972
YKL167C	YMR193W	972
YKL167C	YNL005C	974
YKL167C	YNL252C	974
YKL168C	YDR099W	940
YKL168C	YER177W	928
YKL168C	YFL007W	984
YKL170W	YBL038W	942
YKL170W	YBL087C	935
YKL170W	YBR048W	907
YKL170W	YBR118W	912
YKL170W	YCR031C	965
YKL170W	YDR025W	907
YKL170W	YDR041W	976
YKL170W	YDR450W	948
YKL170W	YEL050C	907
YKL170W	YER117W	935
YKL170W	YFR031C-A	970
YKL170W	YGL123W	982
YKL170W	YGL147C	900
YKL170W	YGR085C	981
YKL170W	YGR220C	962
YKL170W	YHR147C	963
YKL170W	YIL018W	970
YKL170W	YJL063C	952
YKL170W	YJL190C	932
YKL170W	YJL191W	964
YKL170W	YJR123W	938
YKL170W	YLR367W	931
YKL170W	YML026C	948
YKL170W	YNL067W	903

YKL170W	YNL081C	979
YKL170W	YNL178W	965
YKL170W	YNL185C	914
YKL170W	YNR037C	985
YKL170W	YPR080W	912
YKL170W	YPR102C	981
YKL171W	YHR027C	999
YKL172W	YCL054W	977
YKL172W	YDL031W	996
YKL172W	YDL082W	927
YKL172W	YDR060W	990
YKL172W	YER006W	985
YKL172W	YFL002C	976
YKL172W	YFR001W	969
YKL172W	YGL103W	927
YKL172W	YGL111W	986
YKL172W	YGR103W	984
YKL172W	YHR052W	999
YKL172W	YHR066W	996
YKL172W	YHR088W	982
YKL172W	YKL082C	945
YKL172W	YKR081C	999
YKL172W	YLL008W	985
YKL172W	YLL045C	966
YKL172W	YLR002C	980
YKL172W	YLR029C	927
YKL172W	YLR196W	995
YKL172W	YLR276C	993
YKL172W	YMR049C	988
YKL172W	YMR290C	967
YKL172W	YNL002C	992
YKL172W	YNL061W	988
YKL172W	YNL110C	945
YKL172W	YOL077C	999
YKL172W	YOL127W	929
YKL172W	YOR063W	931
YKL172W	YOR206W	989
YKL172W	YOR272W	997
YKL172W	YOR294W	999
YKL172W	YPL093W	990
YKL172W	YPL131W	952
YKL172W	YPL211W	983
YKL173W	YAL032C	998
YKL173W	YBL026W	999

YKL173W	YBL074C	999
YKL173W	YBR055C	999
YKL173W	YBR152W	915
YKL173W	YCR077C	976
YKL173W	YDL030W	978
YKL173W	YDL043C	974
YKL173W	YDL087C	997
YKL173W	YDL098C	994
YKL173W	YDL160C	974
YKL173W	YDL209C	997
YKL173W	YDR243C	990
YKL173W	YDR283C	943
YKL173W	YDR364C	981
YKL173W	YDR378C	993
YKL173W	YDR416W	976
YKL173W	YDR473C	999
YKL173W	YER029C	999
YKL173W	YER112W	992
YKL173W	YER146W	998
YKL173W	YER172C	999
YKL173W	YFL017W-A	996
YKL173W	YGL120C	999
YKL173W	YGL128C	993
YKL173W	YGR013W	989
YKL173W	YGR074W	999
YKL173W	YGR075C	974
YKL173W	YGR091W	999
YKL173W	YGR278W	997
YKL173W	YHR165C	999
YKL173W	YIL061C	908
YKL173W	YJL124C	991
YKL173W	YJL203W	993
YKL173W	YJR022W	998
YKL173W	YKL095W	953
YKL173W	YLL036C	999
YKL173W	YLR117C	999
YKL173W	YLR147C	999
YKL173W	YLR275W	999
YKL173W	YLR424W	994
YKL173W	YLR438C-A	997
YKL173W	YML046W	989
YKL173W	YML049C	998
YKL173W	YMR125W	996
YKL173W	YMR213W	999

YKL173W	YMR240C	997
YKL173W	YMR288W	998
YKL173W	YNL147W	999
YKL173W	YNR011C	911
YKL173W	YOR159C	999
YKL173W	YOR308C	998
YKL173W	YPL151C	991
YKL173W	YPL213W	996
YKL173W	YPR082C	999
YKL173W	YPR101W	991
YKL173W	YPR178W	999
YKL173W	YPR182W	999
YKL175W	YGL255W	949
YKL176C	YER125W	911
YKL176C	YGR057C	992
YKL176C	YKR039W	931
YKL176C	YLR208W	989
YKL178C	YBL016W	910
YKL178C	YDR103W	920
YKL178C	YDR264C	909
YKL178C	YFL026W	995
YKL178C	YHL007C	986
YKL178C	YHR005C	997
YKL178C	YJL170C	931
YKL178C	YJR004C	998
YKL178C	YJR086W	945
YKL178C	YLR040C	982
YKL178C	YLR362W	910
YKL178C	YLR452C	974
YKL178C	YOR212W	989
YKL179C	YHL031C	934
YKL180W	YAL003W	979
YKL180W	YAL035W	923
YKL180W	YBL027W	999
YKL180W	YBL072C	994
YKL180W	YBL087C	997
YKL180W	YBL092W	999
YKL180W	YBR031W	999
YKL180W	YBR048W	999
YKL180W	YBR084C-A	993
YKL180W	YBR118W	911
YKL180W	YBR181C	995
YKL180W	YBR189W	999
YKL180W	YBR191W	999

YKL180W	YCR031C	999
YKL180W	YDL061C	998
YKL180W	YDL075W	999
YKL180W	YDL081C	937
YKL180W	YDL082W	996
YKL180W	YDL083C	964
YKL180W	YDL130W	954
YKL180W	YDL136W	999
YKL180W	YDL191W	916
YKL180W	YDR012W	999
YKL180W	YDR025W	967
YKL180W	YDR064W	999
YKL180W	YDR382W	999
YKL180W	YDR385W	901
YKL180W	YDR418W	999
YKL180W	YDR447C	996
YKL180W	YDR450W	995
YKL180W	YDR471W	999
YKL180W	YDR500C	976
YKL180W	YEL026W	952
YKL180W	YEL054C	926
YKL180W	YER056C-A	999
YKL180W	YER074W	995
YKL180W	YER102W	980
YKL180W	YER117W	993
YKL180W	YER131W	979
YKL180W	YFL034C-A	913
YKL180W	YFR031C-A	999
YKL180W	YFR032C-A	934
YKL180W	YGL030W	999
YKL180W	YGL031C	999
YKL180W	YGL076C	999
YKL180W	YGL103W	999
YKL180W	YGL123W	999
YKL180W	YGL135W	999
YKL180W	YGL147C	999
YKL180W	YGL189C	905
YKL180W	YGR027C	999
YKL180W	YGR034W	998
YKL180W	YGR085C	999
YKL180W	YGR118W	999
YKL180W	YGR148C	999
YKL180W	YGR214W	999
YKL180W	YHL001W	999

YKL180W	YHL015W	999
YKL180W	YHL033C	999
YKL180W	YHR010W	999
YKL180W	YHR021C	919
YKL180W	YHR141C	999
YKL180W	YHR203C	999
YKL180W	YIL018W	998
YKL180W	YIL052C	999
YKL180W	YIL069C	995
YKL180W	YIL133C	999
YKL180W	YIL148W	969
YKL180W	YJL136C	973
YKL180W	YJL177W	999
YKL180W	YJL189W	952
YKL180W	YJL190C	998
YKL180W	YJL191W	999
YKL180W	YJR094W-A	999
YKL180W	YJR123W	999
YKL180W	YJR145C	971
YKL180W	YKL006W	999
YKL180W	YKL156W	994
YKL180W	YKR057W	921
YKL180W	YKR094C	905
YKL180W	YLL045C	997
YKL180W	YLR029C	999
YKL180W	YLR048W	999
YKL180W	YLR060W	913
YKL180W	YLR061W	999
YKL180W	YLR075W	999
YKL180W	YLR167W	964
YKL180W	YLR185W	997
YKL180W	YLR249W	940
YKL180W	YLR264W	998
YKL180W	YLR287C-A	995
YKL180W	YLR333C	930
YKL180W	YLR340W	999
YKL180W	YLR344W	999
YKL180W	YLR367W	992
YKL180W	YLR388W	999
YKL180W	YLR406C	997
YKL180W	YLR441C	995
YKL180W	YLR448W	999
YKL180W	YML024W	999
YKL180W	YML026C	918

YKL180W	YML063W	999
YKL180W	YML073C	996
YKL180W	YML106W	972
YKL180W	YMR121C	928
YKL180W	YMR142C	996
YKL180W	YMR194W	999
YKL180W	YMR230W	950
YKL180W	YMR242C	996
YKL180W	YMR260C	905
YKL180W	YNL067W	999
YKL180W	YNL069C	996
YKL180W	YNL096C	996
YKL180W	YNL132W	944
YKL180W	YNL162W	921
YKL180W	YNL178W	999
YKL180W	YNL209W	998
YKL180W	YNL301C	999
YKL180W	YNL302C	997
YKL180W	YNR037C	942
YKL180W	YOL039W	936
YKL180W	YOL040C	999
YKL180W	YOL120C	914
YKL180W	YOL121C	914
YKL180W	YOL127W	999
YKL180W	YOR063W	999
YKL180W	YOR096W	999
YKL180W	YOR133W	934
YKL180W	YOR167C	955
YKL180W	YOR182C	901
YKL180W	YOR234C	994
YKL180W	YOR293W	979
YKL180W	YOR312C	996
YKL180W	YOR369C	920
YKL180W	YPL037C	999
YKL180W	YPL079W	978
YKL180W	YPL081W	952
YKL180W	YPL090C	903
YKL180W	YPL131W	999
YKL180W	YPL143W	999
YKL180W	YPL198W	995
YKL180W	YPL220W	998
YKL180W	YPL249C-A	997
YKL180W	YPR043W	958
YKL180W	YPR080W	905

YKL180W	YPR102C	999
YKL180W	YPR132W	984
YKL181W	YBL068W	999
YKL181W	YBR276C	998
YKL181W	YER025W	940
YKL181W	YER099C	998
YKL181W	YGL030W	966
YKL181W	YHL011C	999
YKL181W	YKL006W	942
YKL181W	YML106W	994
YKL181W	YOL061W	999
YKL181W	YPR074C	912
YKL182W	YDL112W	960
YKL182W	YJR077C	964
YKL182W	YLR044C	958
YKL182W	YLR195C	976
YKL182W	YNR016C	926
YKL182W	YPL231W	999
YKL183W	YGL162W	998
YKL183W	YGR074W	958
YKL184W	YJL088W	920
YKL184W	YLR146C	996
YKL184W	YOL052C	993
YKL184W	YPL111W	945
YKL184W	YPR069C	977
YKL185W	YAL013W	993
YKL185W	YAL029C	991
YKL185W	YBR095C	998
YKL185W	YBR130C	957
YKL185W	YBR160W	936
YKL185W	YDL076C	993
YKL185W	YDR207C	983
YKL185W	YFR001W	933
YKL185W	YIL084C	993
YKL185W	YKL130C	999
YKL185W	YMR263W	993
YKL185W	YNL097C	993
YKL185W	YNL330C	999
YKL185W	YOL004W	999
YKL185W	YPL139C	993
YKL185W	YPL181W	993
YKL186C	YAR002W	981
YKL186C	YDL116W	996
YKL186C	YDR159W	999

YKL186C	YGL092W	982
YKL186C	YGL100W	992
YKL186C	YIL115C	977
YKL186C	YJL041W	989
YKL186C	YJR042W	998
YKL186C	YKL057C	958
YKL186C	YLR208W	952
YKL186C	YMR047C	999
YKL186C	YOR098C	981
YKL186C	YPL020C	984
YKL186C	YPL169C	999
YKL188C	YDR378C	966
YKL188C	YER146W	946
YKL188C	YGL173C	946
YKL188C	YGL205W	916
YKL188C	YLR438C-A	931
YKL188C	YPL147W	999
YKL189W	YHR102W	995
YKL189W	YNL161W	958
YKL189W	YOR353C	997
YKL190W	YEL027W	957
YKL190W	YGL006W	959
YKL190W	YGR229C	967
YKL190W	YHR030C	990
YKL190W	YLR113W	979
YKL190W	YLR342W	997
YKL190W	YLR433C	999
YKL190W	YML057W	999
YKL190W	YMR211W	911
YKL190W	YNL027W	969
YKL191W	YBL071W-A	999
YKL191W	YDR385W	996
YKL191W	YIL103W	999
YKL191W	YOR133W	986
YKL192C	YER061C	984
YKL193C	YAR014C	932
YKL193C	YER133W	999
YKL193C	YFR003C	996
YKL193C	YLR330W	999
YKL193C	YML016C	996
YKL193C	YMR311C	950
YKL193C	YPL209C	999
YKL195W	YEL020W-A	998
YKL195W	YGR029W	999

YKL195W	YGR181W	996
YKL195W	YLL009C	995
YKL195W	YNL131W	906
YKL196C	YAL014C	975
YKL196C	YBL050W	999
YKL196C	YBR080C	930
YKL196C	YDL058W	930
YKL196C	YDL077C	920
YKL196C	YDR189W	993
YKL196C	YEL013W	992
YKL196C	YER157W	973
YKL196C	YGL095C	920
YKL196C	YGL098W	998
YKL196C	YGL145W	966
YKL196C	YGL212W	997
YKL196C	YGR120C	928
YKL196C	YHL031C	999
YKL196C	YIL004C	999
YKL196C	YKL006C-A	999
YKL196C	YLR026C	999
YKL196C	YLR078C	999
YKL196C	YLR093C	999
YKL196C	YLR268W	999
YKL196C	YLR440C	999
YKL196C	YMR197C	999
YKL196C	YNL258C	966
YKL196C	YOR036W	999
YKL196C	YOR106W	999
YKL196C	YOR327C	999
YKL197C	YAL055W	902
YKL197C	YDR244W	997
YKL197C	YDR265W	902
YKL197C	YGR077C	912
YKL197C	YGR133W	931
YKL197C	YNL329C	999
YKL197C	YOL044W	996
YKL198C	YIL147C	998
YKL201C	YDR285W	999
YKL203C	YBL105C	991
YKL203C	YBR036C	920
YKL203C	YCR027C	991
YKL203C	YDL047W	972
YKL203C	YDL134C	904
YKL203C	YDR188W	984

YKL203C	YDR208W	913
YKL203C	YDR389W	998
YKL203C	YER040W	964
YKL203C	YER093C	999
YKL203C	YFL039C	908
YKL203C	YHR030C	946
YKL203C	YHR186C	995
YKL203C	YIL002C	911
YKL203C	YIL105C	998
YKL203C	YJL058C	993
YKL203C	YJR066W	999
YKL203C	YKL126W	963
YKL203C	YLR342W	973
YKL203C	YLR371W	988
YKL203C	YMR028W	991
YKL203C	YMR068W	999
YKL203C	YMR104C	999
YKL203C	YMR200W	900
YKL203C	YMR307W	926
YKL203C	YNL006W	999
YKL203C	YNL047C	988
YKL203C	YNL090W	985
YKL203C	YNL135C	979
YKL203C	YNL183C	927
YKL203C	YNL229C	913
YKL203C	YOL078W	999
YKL203C	YPL180W	915
YKL203C	YPL268W	976
YKL203C	YPR165W	986
YKL204W	YDR283C	928
YKL204W	YJL080C	996
YKL204W	YOL139C	999
YKL205W	YCR073W-A	954
YKL205W	YER168C	916
YKL205W	YGL105W	999
YKL205W	YGR128C	944
YKL205W	YGR248W	939
YKL205W	YHR163W	946
YKL205W	YJL041W	976
YKL205W	YLR293C	999
YKL205W	YLR335W	912
YKL205W	YNR034W	998
YKL205W	YOR112W	976
YKL205W	YPL212C	981

YKL206C	YBR173C	925
YKL206C	YHR079C	916
YKL206C	YLR199C	998
YKL209C	YFL026W	999
YKL209C	YGR100W	936
YKL209C	YIL015W	958
YKL210W	YBR082C	957
YKL210W	YDR415C	999
YKL210W	YIL148W	984
YKL210W	YOR339C	956
YKL211C	YDR007W	998
YKL211C	YDR354W	999
YKL211C	YER090W	999
YKL211C	YGL026C	995
YKL211C	YGL148W	955
YKL211C	YKL218C	980
YKL212W	YBR164C	937
YKL212W	YFL039C	991
YKL212W	YFR019W	993
YKL212W	YHR004C	901
YKL212W	YHR030C	980
YKL212W	YIL002C	951
YKL212W	YJL100W	936
YKL212W	YJR110W	979
YKL212W	YKL093W	905
YKL212W	YLR240W	988
YKL212W	YLR305C	999
YKL212W	YMR079W	999
YKL212W	YNL106C	998
YKL212W	YNL267W	997
YKL212W	YNL325C	939
YKL212W	YOL065C	999
YKL212W	YOR109W	998
YKL212W	YPL051W	909
YKL212W	YPL240C	919
YKL212W	YPR183W	998
YKL213C	YBL002W	916
YKL213C	YBL058W	999
YKL213C	YBR058C	903
YKL213C	YBR170C	954
YKL213C	YBR273C	993
YKL213C	YDL074C	909
YKL213C	YDL126C	999
YKL213C	YDL190C	995

YKL213C	YDR217C	966
YKL213C	YDR330W	931
YKL213C	YFL044C	930
YKL213C	YGR048W	965
YKL213C	YHL002W	951
YKL213C	YJL048C	912
YKL213C	YLL039C	999
YKL213C	YLR418C	981
YKL213C	YML032C	936
YKL213C	YMR067C	965
YKL213C	YOR341W	987
YKL213C	YPL055C	943
YKL213C	YPR010C	935
YKL214C	YDL084W	994
YKL214C	YDR381W	993
YKL214C	YMR125W	999
YKL214C	YMR308C	919
YKL214C	YPL169C	938
YKL214C	YPL178W	987
YKL216W	YEL021W	994
YKL216W	YJL130C	978
YKL216W	YJR109C	980
YKL216W	YLR014C	988
YKL216W	YLR420W	998
YKL216W	YML106W	976
YKL216W	YMR271C	974
YKL218C	YKL211C	980
YKL220C	YGL071W	995
YKL220C	YLR214W	983
YKL220C	YMR021C	932
YKL220C	YMR058W	929
YKL220C	YOL152W	903
YKR001C	YBR009C	970
YKR001C	YBR049C	956
YKR001C	YBR245C	999
YKR001C	YDL065C	974
YKR001C	YDR303C	981
YKR001C	YDR479C	911
YKR001C	YFR037C	970
YKR001C	YGL133W	968
YKR001C	YGL206C	924
YKR001C	YHR150W	911
YKR001C	YIL068C	977
YKR001C	YIL126W	982

YKR001C	YLL001W	985
YKR001C	YLR033W	970
YKR001C	YLR176C	907
YKR001C	YLR357W	970
YKR001C	YMR004W	985
YKR001C	YMR033W	983
YKR001C	YMR072W	977
YKR001C	YMR091C	988
YKR001C	YMR204C	980
YKR001C	YNL243W	977
YKR001C	YOR036W	903
YKR001C	YOR304W	970
YKR001C	YPL082C	998
YKR001C	YPL154C	901
YKR002W	YAL043C	999
YKR002W	YDR195W	999
YKR002W	YDR228C	993
YKR002W	YDR301W	999
YKR002W	YDR390C	996
YKR002W	YER127W	917
YKR002W	YER133W	999
YKR002W	YGL044C	942
YKR002W	YGR048W	973
YKR002W	YGR156W	999
YKR002W	YHL033C	979
YKR002W	YJL033W	961
YKR002W	YJR093C	999
YKR002W	YKL018W	998
YKR002W	YKL059C	999
YKR002W	YLR115W	999
YKR002W	YLR277C	999
YKR002W	YMR061W	999
YKR002W	YNL222W	999
YKR002W	YNL317W	999
YKR002W	YOL115W	960
YKR002W	YOR001W	998
YKR002W	YOR207C	901
YKR002W	YPL031C	976
YKR002W	YPR107C	999
YKR003W	YPR173C	980
YKR006C	YBL038W	988
YKR006C	YBR122C	972
YKR006C	YCR046C	992
YKR006C	YCR071C	972

YKR006C	YDR116C	972
YKR006C	YDR237W	974
YKR006C	YDR296W	993
YKR006C	YDR322W	974
YKR006C	YDR405W	970
YKR006C	YDR462W	972
YKR006C	YGR220C	998
YKR006C	YJL063C	972
YKR006C	YKL167C	989
YKR006C	YKR085C	972
YKR006C	YLR312W-A	972
YKR006C	YLR439W	974
YKR006C	YML025C	997
YKR006C	YMR024W	972
YKR006C	YMR193W	972
YKR006C	YNL005C	989
YKR006C	YNL252C	974
YKR007W	YBR077C	999
YKR007W	YCR075C	977
YKR007W	YEL013W	951
YKR007W	YGR163W	999
YKR007W	YKL143W	955
YKR007W	YML121W	999
YKR008W	YBR154C	913
YKR008W	YBR245C	999
YKR008W	YCR020W-B	999
YKR008W	YCR052W	999
YKR008W	YDR303C	999
YKR008W	YFR037C	999
YKR008W	YGR056W	999
YKR008W	YGR252W	976
YKR008W	YGR275W	995
YKR008W	YHR056C	996
YKR008W	YIL126W	999
YKR008W	YLR033W	999
YKR008W	YLR321C	999
YKR008W	YLR357W	999
YKR008W	YML127W	999
YKR008W	YMR033W	999
YKR008W	YMR091C	999
YKR008W	YPR034W	999
YKR009C	YGL205W	988
YKR009C	YIL160C	918
YKR010C	YCR086W	997

YKR010C	YDR110W	932
YKR010C	YDR138W	908
YKR010C	YDR439W	943
YKR010C	YFR028C	996
YKR014C	YDR328C	999
YKR014C	YER136W	998
YKR014C	YGR172C	954
YKR014C	YLR026C	910
YKR014C	YMR258C	999
YKR014C	YNL093W	915
YKR014C	YOR089C	989
YKR014C	YOR370C	998
YKR017C	YJL207C	998
YKR018C	YKL028W	929
YKR019C	YIL002C	927
YKR020W	YDR027C	999
YKR020W	YDR468C	999
YKR020W	YDR484W	999
YKR020W	YJL029C	997
YKR020W	YLR262C	913
YKR021W	YER125W	991
YKR022C	YBL010C	932
YKR022C	YGL120C	995
YKR022C	YGL128C	997
YKR022C	YHR165C	997
YKR022C	YLR424W	999
YKR024C	YAL035W	940
YKR024C	YDR060W	966
YKR024C	YJR041C	946
YKR024C	YMR229C	977
YKR024C	YNR038W	910
YKR024C	YOR204W	939
YKR024C	YPR131C	940
YKR025W	YBR154C	997
YKR025W	YDL150W	999
YKR025W	YDR045C	998
YKR025W	YHR143W-A	960
YKR025W	YJL011C	999
YKR025W	YKL144C	999
YKR025W	YNL113W	998
YKR025W	YNL151C	998
YKR025W	YNR003C	999
YKR025W	YOR116C	999
YKR025W	YOR207C	999

YKR025W	YOR210W	997
YKR025W	YOR224C	997
YKR025W	YPR110C	999
YKR025W	YPR187W	997
YKR025W	YPR190C	999
YKR026C	YBR159W	983
YKR026C	YDR211W	999
YKR026C	YDR283C	999
YKR026C	YEL009C	960
YKR026C	YER025W	999
YKR026C	YGL206C	907
YKR026C	YGR083C	999
YKR026C	YJR007W	999
YKR026C	YLR291C	999
YKR026C	YNL265C	982
YKR026C	YOR260W	999
YKR026C	YPL237W	999
YKR027W	YBR023C	903
YKR027W	YJL099W	996
YKR027W	YLR330W	999
YKR027W	YMR237W	999
YKR027W	YOR299W	985
YKR028W	YDL047W	999
YKR028W	YDR293C	976
YKR028W	YFR040W	976
YKR028W	YJL098W	984
YKR028W	YPL049C	903
YKR029C	YAL013W	947
YKR029C	YBR095C	947
YKR029C	YBR103W	999
YKR029C	YCR033W	999
YKR029C	YDR155C	999
YKR029C	YDR207C	914
YKR029C	YDR310C	955
YKR029C	YGL194C	999
YKR029C	YIL084C	922
YKR029C	YIL112W	999
YKR029C	YJL168C	968
YKR029C	YMR263W	956
YKR029C	YMR273C	994
YKR029C	YNL097C	950
YKR029C	YNL330C	951
YKR029C	YOL004W	921
YKR029C	YOL068C	998

YKR029C	YPL139C	941
YKR029C	YPL181W	956
YKR030W	YEL022W	933
YKR030W	YJR031C	989
YKR031C	YBR057C	996
YKR031C	YGL036W	907
YKR031C	YMR079W	999
YKR031C	YNL130C	924
YKR031C	YOR242C	917
YKR031C	YPL145C	982
YKR034W	YBR208C	916
YKR034W	YDL210W	967
YKR034W	YIR023W	963
YKR034W	YIR029W	939
YKR034W	YIR032C	989
YKR034W	YKR039W	931
YKR034W	YNL142W	900
YKR034W	YNL229C	993
YKR034W	YNL314W	951
YKR035W-A	YDR069C	931
YKR035W-A	YDR486C	981
YKR035W-A	YKL002W	999
YKR035W-A	YKL041W	995
YKR035W-A	YLR025W	970
YKR035W-A	YLR181C	994
YKR035W-A	YNL265C	973
YKR035W-A	YPL240C	919
YKR035W-A	YPR173C	999
YKR036C	YIL065C	999
YKR036C	YLL001W	929
YKR037C	YBR233W-A	992
YKR037C	YDR016C	999
YKR037C	YDR201W	999
YKR037C	YDR318W	983
YKR037C	YDR320C-A	996
YKR037C	YER016W	944
YKR037C	YER018C	963
YKR037C	YGL061C	999
YKR037C	YGR113W	999
YKR037C	YIL144W	989
YKR037C	YJL030W	983
YKR037C	YJR135C	981
YKR037C	YKL052C	999
YKR037C	YKL138C-A	999

YKR037C	YKR083C	999
YKR037C	YLR045C	963
YKR037C	YLR381W	983
YKR037C	YPL018W	983
YKR037C	YPL209C	990
YKR038C	YGR262C	999
YKR038C	YJL184W	999
YKR038C	YKR095W-A	993
YKR038C	YML036W	999
YKR039W	YDL210W	923
YKR039W	YDL212W	995
YKR039W	YDR160W	997
YKR039W	YER040W	972
YKR039W	YER125W	949
YKR039W	YFR029W	954
YKR039W	YJL110C	971
YKR039W	YJL156C	943
YKR039W	YKL176C	931
YKR039W	YKR034W	931
YKR039W	YNL183C	952
YKR048C	YAL020C	972
YKR048C	YBL002W	997
YKR048C	YBL003C	999
YKR048C	YBR010W	938
YKR048C	YBR267W	997
YKR048C	YCL024W	999
YKR048C	YDR162C	984
YKR048C	YDR224C	990
YKR048C	YDR225W	999
YKR048C	YDR381W	983
YKR048C	YDR507C	999
YKR048C	YGL241W	999
YKR048C	YGR245C	999
YKR048C	YJL187C	954
YKR048C	YKR079C	966
YKR048C	YLR410W	972
YKR048C	YLR457C	970
YKR048C	YMR139W	999
YKR048C	YNL078W	999
YKR048C	YOL012C	999
YKR048C	YOL070C	999
YKR048C	YOR112W	940
YKR048C	YPR119W	999
YKR050W	YDR345C	967

YKR050W	YHR094C	967
YKR050W	YJL093C	969
YKR050W	YJL129C	999
YKR050W	YOL004W	913
YKR052C	YDL120W	992
YKR052C	YKR071C	915
YKR052C	YLR220W	911
YKR052C	YNR001C	998
YKR052C	YOR334W	985
YKR052C	YPL202C	927
YKR053C	YDR294C	946
YKR053C	YDR297W	945
YKR053C	YJL134W	969
YKR053C	YLR260W	950
YKR053C	YOR171C	945
YKR054C	YDR488C	954
YKR054C	YEL061C	999
YKR054C	YER016W	998
YKR054C	YFL037W	941
YKR054C	YGL216W	995
YKR054C	YJR053W	920
YKR054C	YNL271C	995
YKR054C	YPL155C	909
YKR054C	YPL269W	999
YKR054C	YPR141C	999
YKR055W	YBL085W	988
YKR055W	YBR200W	937
YKR055W	YBR260C	998
YKR055W	YDL135C	987
YKR055W	YER114C	988
YKR055W	YIL118W	979
YKR055W	YIL159W	967
YKR055W	YOR008C	982
YKR055W	YPL161C	970
YKR056W	YLR196W	901
YKR056W	YML032C	998
YKR056W	YMR106C	976
YKR056W	YOR033C	987
YKR057W	YBL027W	922
YKR057W	YBL072C	929
YKR057W	YBL087C	931
YKR057W	YBL092W	944
YKR057W	YBR031W	938
YKR057W	YBR048W	914

YKR057W	YBR084C-A	922
YKR057W	YBR118W	910
YKR057W	YBR181C	906
YKR057W	YBR189W	922
YKR057W	YBR191W	925
YKR057W	YCR031C	932
YKR057W	YDL061C	915
YKR057W	YDL075W	927
YKR057W	YDL081C	943
YKR057W	YDL082W	928
YKR057W	YDL130W	913
YKR057W	YDL136W	930
YKR057W	YDL191W	930
YKR057W	YDR012W	938
YKR057W	YDR025W	914
YKR057W	YDR064W	958
YKR057W	YDR382W	932
YKR057W	YDR385W	911
YKR057W	YDR418W	932
YKR057W	YDR447C	936
YKR057W	YDR450W	923
YKR057W	YDR471W	912
YKR057W	YDR500C	921
YKR057W	YEL054C	932
YKR057W	YER056C-A	909
YKR057W	YER074W	922
YKR057W	YER102W	929
YKR057W	YER117W	931
YKR057W	YER131W	922
YKR057W	YER165W	902
YKR057W	YFL034C-A	920
YKR057W	YFR031C-A	912
YKR057W	YFR032C-A	948
YKR057W	YGL030W	949
YKR057W	YGL031C	904
YKR057W	YGL076C	935
YKR057W	YGL123W	906
YKR057W	YGL135W	929
YKR057W	YGL147C	926
YKR057W	YGL189C	922
YKR057W	YGR027C	931
YKR057W	YGR034W	931
YKR057W	YGR085C	921
YKR057W	YGR118W	933

YKR057W	YGR148C	904
YKR057W	YGR214W	952
YKR057W	YHL001W	930
YKR057W	YHL015W	944
YKR057W	YHL033C	932
YKR057W	YHR010W	912
YKR057W	YHR021C	937
YKR057W	YHR141C	937
YKR057W	YHR203C	930
YKR057W	YIL018W	912
YKR057W	YIL052C	909
YKR057W	YIL069C	922
YKR057W	YIL133C	932
YKR057W	YIL148W	908
YKR057W	YJL136C	984
YKR057W	YJL177W	921
YKR057W	YJL189W	909
YKR057W	YJL190C	923
YKR057W	YJL191W	932
YKR057W	YJR007W	900
YKR057W	YJR094W-A	935
YKR057W	YJR123W	936
YKR057W	YJR145C	930
YKR057W	YKL006W	930
YKR057W	YKL156W	937
YKR057W	YKL180W	921
YKR057W	YKR094C	908
YKR057W	YLL045C	932
YKR057W	YLR029C	918
YKR057W	YLR048W	952
YKR057W	YLR061W	924
YKR057W	YLR075W	922
YKR057W	YLR167W	902
YKR057W	YLR185W	922
YKR057W	YLR264W	916
YKR057W	YLR287C-A	909
YKR057W	YLR325C	930
YKR057W	YLR333C	930
YKR057W	YLR340W	943
YKR057W	YLR344W	931
YKR057W	YLR367W	923
YKR057W	YLR388W	916
YKR057W	YLR406C	927
YKR057W	YLR441C	932

YKR057W	YLR448W	932
YKR057W	YML024W	936
YKR057W	YML026C	923
YKR057W	YML063W	932
YKR057W	YML073C	930
YKR057W	YMR121C	918
YKR057W	YMR142C	926
YKR057W	YMR194W	934
YKR057W	YMR230W	938
YKR057W	YMR242C	934
YKR057W	YMR260C	912
YKR057W	YNL067W	926
YKR057W	YNL069C	931
YKR057W	YNL096C	935
YKR057W	YNL162W	937
YKR057W	YNL178W	951
YKR057W	YNL301C	922
YKR057W	YNL302C	926
YKR057W	YOL039W	913
YKR057W	YOL040C	956
YKR057W	YOL120C	922
YKR057W	YOL121C	926
YKR057W	YOL127W	925
YKR057W	YOR063W	923
YKR057W	YOR096W	935
YKR057W	YOR133W	911
YKR057W	YOR167C	916
YKR057W	YOR182C	909
YKR057W	YOR234C	934
YKR057W	YOR293W	938
YKR057W	YOR312C	934
YKR057W	YOR369C	935
YKR057W	YPL079W	925
YKR057W	YPL081W	921
YKR057W	YPL090C	906
YKR057W	YPL131W	912
YKR057W	YPL143W	934
YKR057W	YPL198W	934
YKR057W	YPL220W	929
YKR057W	YPL249C-A	935
YKR057W	YPR043W	935
YKR057W	YPR080W	910
YKR057W	YPR102C	921
YKR057W	YPR132W	933

YKR058W	YEL011W	989
YKR058W	YFR015C	995
YKR058W	YJL137C	994
YKR058W	YLR258W	997
YKR059W	YBL051C	936
YKR059W	YBR048W	939
YKR059W	YBR079C	910
YKR059W	YBR084C-A	999
YKR059W	YBR101C	925
YKR059W	YBR181C	915
YKR059W	YBR189W	931
YKR059W	YCR031C	945
YKR059W	YDL061C	952
YKR059W	YDL136W	974
YKR059W	YDR064W	967
YKR059W	YDR500C	998
YKR059W	YER025W	949
YKR059W	YER102W	999
YKR059W	YER117W	998
YKR059W	YER131W	909
YKR059W	YER165W	925
YKR059W	YGL049C	997
YKR059W	YGL123W	959
YKR059W	YGR027C	923
YKR059W	YGR118W	964
YKR059W	YGR162W	999
YKR059W	YGR214W	938
YKR059W	YHL015W	942
YKR059W	YHR203C	999
YKR059W	YIL148W	998
YKR059W	YJL136C	916
YKR059W	YJL138C	999
YKR059W	YJL191W	930
YKR059W	YJR007W	968
YKR059W	YJR123W	961
YKR059W	YKL156W	947
YKR059W	YLR048W	970
YKR059W	YLR167W	999
YKR059W	YLR185W	954
YKR059W	YLR264W	947
YKR059W	YLR367W	926
YKR059W	YLR388W	975
YKR059W	YML024W	976
YKR059W	YML063W	948

YKR059W	YMR260C	901
YKR059W	YNL178W	969
YKR059W	YNL301C	998
YKR059W	YOL040C	901
YKR059W	YOL139C	995
YKR059W	YOR096W	943
YKR059W	YOR133W	990
YKR059W	YOR204W	978
YKR059W	YOR293W	918
YKR059W	YPL220W	998
YKR059W	YPL237W	912
YKR059W	YPR132W	999
YKR059W	YPR163C	996
YKR060W	YBL004W	997
YKR060W	YBR247C	963
YKR060W	YCL031C	910
YKR060W	YCL059C	996
YKR060W	YCR057C	999
YKR060W	YDL014W	997
YKR060W	YDL148C	997
YKR060W	YDL153C	910
YKR060W	YDR299W	998
YKR060W	YDR324C	997
YKR060W	YDR449C	998
YKR060W	YER082C	997
YKR060W	YGL171W	920
YKR060W	YGR090W	994
YKR060W	YGR128C	917
YKR060W	YGR145W	997
YKR060W	YHR148W	997
YKR060W	YHR169W	920
YKR060W	YJL010C	909
YKR060W	YJL069C	953
YKR060W	YJR002W	998
YKR060W	YKL099C	934
YKR060W	YLL011W	997
YKR060W	YLR003C	985
YKR060W	YLR129W	997
YKR060W	YLR186W	997
YKR060W	YLR197W	997
YKR060W	YLR222C	918
YKR060W	YLR409C	997
YKR060W	YMR093W	997
YKR060W	YMR128W	997

YKR060W	YMR290C	909
YKR060W	YNL075W	923
YKR060W	YNL132W	997
YKR060W	YOL010W	914
YKR060W	YOR004W	918
YKR060W	YOR096W	925
YKR060W	YOR310C	907
YKR060W	YPL126W	997
YKR060W	YPL217C	923
YKR060W	YPR112C	916
YKR060W	YPR137W	912
YKR060W	YPR144C	999
YKR062W	YBR154C	948
YKR062W	YDL140C	987
YKR062W	YGL127C	915
YKR062W	YGL207W	908
YKR062W	YGR186W	984
YKR062W	YHR041C	978
YKR062W	YHR143W-A	930
YKR062W	YKL028W	999
YKR062W	YNL236W	923
YKR062W	YOL051W	999
YKR062W	YOR151C	949
YKR065C	YIL022W	928
YKR065C	YJL104W	940
YKR065C	YNR017W	999
YKR066C	YDL215C	919
YKR066C	YGR101W	976
YKR066C	YJR048W	999
YKR067W	YFR051C	956
YKR067W	YLR246W	923
YKR067W	YPL240C	902
YKR068C	YBR254C	999
YKR068C	YDR108W	999
YKR068C	YDR246W	999
YKR068C	YDR407C	999
YKR068C	YDR472W	999
YKR068C	YFL038C	999
YKR068C	YGR166W	999
YKR068C	YIL004C	912
YKR068C	YML077W	999
YKR068C	YMR218C	999
YKR068C	YOR115C	999
YKR069W	YBR213W	993

YKR069W	YDL205C	983
YKR069W	YDR047W	905
YKR069W	YJR137C	954
YKR069W	YPR167C	935
YKR071C	YJL133W	916
YKR071C	YKR052C	915
YKR071C	YPR048W	999
YKR072C	YDL047W	998
YKR072C	YDR184C	918
YKR072C	YDR247W	911
YKR072C	YDR436W	958
YKR072C	YIL083C	907
YKR072C	YKL088W	999
YKR072C	YLR138W	987
YKR072C	YML016C	999
YKR072C	YOR054C	992
YKR077W	YBR160W	941
YKR077W	YER111C	942
YKR077W	YLL004W	999
YKR079C	YBR267W	970
YKR079C	YJL208C	999
YKR079C	YKR048C	966
YKR079C	YMR099C	999
YKR080W	YAL044C	900
YKR080W	YBR084W	924
YKR080W	YBR263W	932
YKR080W	YDR408C	941
YKR080W	YFL018C	905
YKR080W	YGL125W	904
YKR080W	YGR204W	936
YKR080W	YLR058C	963
YKR080W	YMR189W	903
YKR080W	YOR074C	918
YKR081C	YBR031W	938
YKR081C	YCL054W	939
YKR081C	YDL031W	999
YKR081C	YDR012W	935
YKR081C	YDR060W	994
YKR081C	YDR087C	999
YKR081C	YDR101C	998
YKR081C	YDR471W	937
YKR081C	YDR496C	996
YKR081C	YER006W	999
YKR081C	YER126C	999

YKR081C	YFL002C	974
YKR081C	YGL076C	938
YKR081C	YGL103W	929
YKR081C	YGL111W	989
YKR081C	YGR085C	990
YKR081C	YGR103W	999
YKR081C	YGR245C	992
YKR081C	YHR052W	999
YKR081C	YHR066W	998
YKR081C	YHR170W	998
YKR081C	YHR197W	999
YKR081C	YKL009W	999
YKR081C	YKL172W	999
YKR081C	YLL008W	999
YKR081C	YLL045C	966
YKR081C	YLR002C	999
YKR081C	YLR009W	983
YKR081C	YLR029C	938
YKR081C	YLR074C	971
YKR081C	YLR196W	966
YKR081C	YLR276C	997
YKR081C	YLR449W	957
YKR081C	YMR049C	998
YKR081C	YMR290C	999
YKR081C	YNL002C	999
YKR081C	YNL061W	999
YKR081C	YNL110C	999
YKR081C	YNR053C	999
YKR081C	YOL077C	999
YKR081C	YOL127W	950
YKR081C	YOR063W	946
YKR081C	YOR096W	941
YKR081C	YOR206W	999
YKR081C	YOR272W	999
YKR081C	YOR294W	999
YKR081C	YPL093W	999
YKR081C	YPL131W	995
YKR081C	YPL198W	947
YKR081C	YPL211W	988
YKR081C	YPR016C	999
YKR081C	YPR102C	977
YKR082W	YAR002W	982
YKR082W	YBL079W	974
YKR082W	YDL088C	941

YKR082W	YDL116W	999
YKR082W	YDL207W	962
YKR082W	YDR076W	973
YKR082W	YDR159W	969
YKR082W	YDR192C	985
YKR082W	YDR424C	954
YKR082W	YER095W	954
YKR082W	YER105C	959
YKR082W	YER116C	940
YKR082W	YFR002W	929
YKR082W	YGL086W	931
YKR082W	YGL092W	984
YKR082W	YGL100W	988
YKR082W	YGL163C	946
YKR082W	YGL172W	998
YKR082W	YGR119C	961
YKR082W	YIL115C	981
YKR082W	YJL030W	959
YKR082W	YJL041W	959
YKR082W	YJL061W	961
YKR082W	YJL092W	968
YKR082W	YJR042W	999
YKR082W	YKL057C	999
YKR082W	YKL068W	997
YKR082W	YKL113C	990
YKR082W	YLR018C	984
YKR082W	YLR208W	995
YKR082W	YLR335W	983
YKR082W	YLR347C	928
YKR082W	YML032C	991
YKR082W	YML103C	992
YKR082W	YML107C	984
YKR082W	YMR047C	992
YKR082W	YMR129W	979
YKR082W	YMR153W	953
YKR082W	YMR224C	954
YKR082W	YNL250W	952
YKR082W	YOL072W	961
YKR082W	YOR098C	930
YKR082W	YOR257W	961
YKR082W	YPL020C	982
YKR082W	YPL169C	966
YKR083C	YBR233W-A	959
YKR083C	YDR016C	999

YKR083C	YDR201W	999
YKR083C	YDR320C-A	991
YKR083C	YGL061C	999
YKR083C	YGR113W	998
YKR083C	YKL052C	999
YKR083C	YKL138C-A	992
YKR083C	YKR037C	999
YKR084C	YNL001W	998
YKR085C	YBL038W	998
YKR085C	YBR122C	972
YKR085C	YCL014W	913
YKR085C	YCR046C	992
YKR085C	YCR071C	972
YKR085C	YDR116C	972
YKR085C	YDR237W	974
YKR085C	YDR296W	992
YKR085C	YDR322W	974
YKR085C	YDR462W	972
YKR085C	YGR220C	997
YKR085C	YJL063C	991
YKR085C	YKL167C	972
YKR085C	YKR006C	972
YKR085C	YLR312W-A	972
YKR085C	YLR439W	974
YKR085C	YML025C	992
YKR085C	YMR024W	972
YKR085C	YMR193W	988
YKR085C	YNL005C	974
YKR085C	YNL252C	974
YKR085C	YOR150W	970
YKR086W	YBR065C	912
YKR086W	YBR188C	928
YKR086W	YDR088C	999
YKR086W	YDR364C	999
YKR086W	YER172C	967
YKR086W	YGR006W	922
YKR086W	YHR165C	998
YKR086W	YJR050W	983
YKR090W	YER155C	914
YKR090W	YLR229C	906
YKR090W	YLR342W	905
YKR090W	YPR165W	987
YKR091W	YBR135W	996
YKR091W	YPL256C	962

YKR092C	YGR204W	955
YKR092C	YGR280C	975
YKR092C	YLR058C	942
YKR092C	YNL112W	956
YKR094C	YBL027W	906
YKR094C	YBL072C	906
YKR094C	YBL087C	906
YKR094C	YBL092W	915
YKR094C	YBR031W	911
YKR094C	YBR084C-A	906
YKR094C	YBR118W	900
YKR094C	YBR181C	900
YKR094C	YBR191W	906
YKR094C	YCR031C	909
YKR094C	YDL075W	907
YKR094C	YDL081C	913
YKR094C	YDL082W	905
YKR094C	YDL122W	968
YKR094C	YDL136W	902
YKR094C	YDL191W	902
YKR094C	YDR012W	911
YKR094C	YDR064W	916
YKR094C	YDR382W	907
YKR094C	YDR385W	903
YKR094C	YDR418W	906
YKR094C	YDR447C	906
YKR094C	YDR450W	906
YKR094C	YDR500C	900
YKR094C	YEL054C	906
YKR094C	YER012W	900
YKR094C	YER102W	906
YKR094C	YER117W	906
YKR094C	YFL034C-A	904
YKR094C	YFR004W	900
YKR094C	YFR031C-A	901
YKR094C	YFR032C-A	915
YKR094C	YFR050C	900
YKR094C	YGL030W	912
YKR094C	YGL076C	906
YKR094C	YGL135W	905
YKR094C	YGL147C	904
YKR094C	YGR034W	908
YKR094C	YGR085C	905
YKR094C	YGR118W	907

YKR094C	YGR214W	905
YKR094C	YHL001W	906
YKR094C	YHL015W	917
YKR094C	YHL033C	908
YKR094C	YHR021C	905
YKR094C	YHR027C	904
YKR094C	YHR141C	908
YKR094C	YHR200W	933
YKR094C	YHR203C	910
YKR094C	YIL018W	901
YKR094C	YIL133C	908
YKR094C	YJL136C	908
YKR094C	YJL177W	905
YKR094C	YJL189W	903
YKR094C	YJL190C	902
YKR094C	YJL191W	909
YKR094C	YJR094W-A	902
YKR094C	YJR123W	908
YKR094C	YJR145C	910
YKR094C	YKL006W	905
YKR094C	YKL156W	905
YKR094C	YKL180W	905
YKR094C	YKR057W	908
YKR094C	YLL039C	915
YKR094C	YLL045C	908
YKR094C	YLR048W	905
YKR094C	YLR061W	906
YKR094C	YLR167W	903
YKR094C	YLR185W	900
YKR094C	YLR264W	903
YKR094C	YLR340W	922
YKR094C	YLR344W	908
YKR094C	YLR367W	902
YKR094C	YLR406C	907
YKR094C	YLR441C	908
YKR094C	YLR448W	908
YKR094C	YML024W	906
YKR094C	YML026C	906
YKR094C	YML063W	908
YKR094C	YML073C	907
YKR094C	YMR142C	905
YKR094C	YMR194W	905
YKR094C	YMR230W	905
YKR094C	YMR242C	909

YKR094C	YMR260C	908
YKR094C	YNL067W	904
YKR094C	YNL069C	908
YKR094C	YNL096C	904
YKR094C	YNL162W	908
YKR094C	YNL178W	919
YKR094C	YNL301C	905
YKR094C	YNL302C	902
YKR094C	YOL039W	900
YKR094C	YOL040C	913
YKR094C	YOL120C	905
YKR094C	YOL121C	902
YKR094C	YOR063W	904
YKR094C	YOR096W	905
YKR094C	YOR133W	903
YKR094C	YOR167C	903
YKR094C	YOR234C	906
YKR094C	YOR293W	905
YKR094C	YOR312C	910
YKR094C	YOR369C	900
YKR094C	YPL079W	906
YKR094C	YPL090C	900
YKR094C	YPL143W	906
YKR094C	YPL198W	906
YKR094C	YPL220W	905
YKR094C	YPL249C-A	906
YKR094C	YPR043W	902
YKR094C	YPR080W	900
YKR094C	YPR102C	905
YKR094C	YPR132W	907
YKR095W	YDR159W	949
YKR095W	YDR381W	934
YKR095W	YGL122C	995
YKR095W	YIL149C	999
YKR095W	YPL169C	934
YKR095W-A	YGR262C	993
YKR095W-A	YJL184W	992
YKR095W-A	YKR038C	993
YKR095W-A	YML036W	992
YKR097W	YAL038W	993
YKR097W	YBR218C	991
YKR097W	YDL078C	951
YKR097W	YGL062W	991
YKR097W	YGR240C	901

YKR097W	YGR254W	960
YKR097W	YHR174W	960
YKR097W	YKL085W	951
YKR097W	YKL106W	905
YKR097W	YLR027C	913
YKR097W	YLR377C	953
YKR097W	YMR205C	900
YKR097W	YOL126C	951
YKR097W	YOR347C	938
YKR099W	YCL030C	974
YKR099W	YDL106C	999
YKR099W	YLR058C	995
YKR101W	YBR009C	940
YKR101W	YBR060C	987
YKR101W	YDL040C	990
YKR101W	YDL042C	999
YKR101W	YDR181C	933
YKR101W	YDR227W	999
YKR101W	YHR013C	941
YKR101W	YHR084W	940
YKR101W	YIL131C	991
YKR101W	YJL115W	995
YKR101W	YLR442C	999
YKR101W	YML065W	999
YKR101W	YML109W	990
YKR101W	YMR127C	999
YKR101W	YNL216W	999
YKR101W	YNL261W	909
YKR101W	YOR213C	963
YKR101W	YPR018W	981
YKR101W	YPR120C	975
YKR102W	YAL063C	904
YKR102W	YAR050W	903
YKR102W	YER109C	981
YKR102W	YHR211W	904
YKR102W	YIR019C	970
YKR102W	YMR317W	929
YKR102W	YNL327W	999
YLL001W	YBR179C	999
YLL001W	YDR150W	938
YLL001W	YDR470C	940
YLL001W	YIL065C	997
YLL001W	YJL112W	999
YLL001W	YKR001C	985

YLL001W	YKR036C	929
YLL001W	YLR368W	927
YLL001W	YOR211C	976
YLL002W	YBR010W	998
YLL002W	YDR217C	966
YLL002W	YJL115W	999
YLL002W	YML032C	987
YLL002W	YNL031C	998
YLL002W	YNL246W	999
YLL002W	YNR010W	957
YLL002W	YOR144C	943
YLL003W	YCL029C	904
YLL003W	YEL061C	976
YLL003W	YOR257W	999
YLL004W	YAL051W	998
YLL004W	YBL023C	983
YLL004W	YBR060C	999
YLL004W	YDR052C	995
YLL004W	YEL056W	999
YLL004W	YHR118C	999
YLL004W	YJL194W	999
YLL004W	YKR077W	999
YLL004W	YML065W	999
YLL004W	YMR036C	999
YLL004W	YNL261W	999
YLL004W	YPL001W	999
YLL004W	YPR018W	998
YLL004W	YPR162C	999
YLL006W	YAL010C	998
YLL006W	YGR132C	908
YLL006W	YGR231C	906
YLL006W	YHR194W	905
YLL006W	YOL009C	993
YLL006W	YOR147W	905
YLL008W	YBR142W	984
YLL008W	YCL054W	981
YLL008W	YDL014W	929
YLL008W	YDL031W	996
YLL008W	YDR060W	978
YLL008W	YDR087C	996
YLL008W	YDR496C	995
YLL008W	YER006W	983
YLL008W	YFL002C	980
YLL008W	YFR001W	992

YLL008W	YGL111W	989
YLL008W	YGR103W	999
YLL008W	YHR052W	997
YLL008W	YHR066W	996
YLL008W	YHR088W	989
YLL008W	YHR197W	989
YLL008W	YKL009W	985
YLL008W	YKL172W	985
YLL008W	YKR081C	999
YLL008W	YLL034C	984
YLL008W	YLL045C	933
YLL008W	YLR002C	989
YLL008W	YLR106C	974
YLL008W	YLR276C	994
YLL008W	YMR049C	992
YLL008W	YMR229C	989
YLL008W	YMR290C	978
YLL008W	YNL002C	986
YLL008W	YNL061W	999
YLL008W	YNL110C	999
YLL008W	YOL077C	994
YLL008W	YOL127W	935
YLL008W	YOR063W	936
YLL008W	YOR206W	984
YLL008W	YOR272W	999
YLL008W	YPL043W	999
YLL008W	YPL093W	978
YLL008W	YPL131W	956
YLL008W	YPL198W	929
YLL008W	YPL211W	987
YLL008W	YPR016C	999
YLL009C	YBR024W	973
YLL009C	YBR037C	998
YLL009C	YJR104C	969
YLL009C	YKL195W	995
YLL009C	YLL013C	948
YLL009C	YPL132W	974
YLL010C	YLR019W	991
YLL010C	YOR043W	999
YLL011W	YBL004W	998
YLL011W	YBL072C	927
YLL011W	YBR247C	999
YLL011W	YCL059C	999
YLL011W	YCR057C	999

YLL011W	YDL014W	999
YLL011W	YDL060W	905
YLL011W	YDL148C	997
YLL011W	YDL153C	910
YLL011W	YDR064W	929
YLL011W	YDR299W	946
YLL011W	YDR324C	997
YLL011W	YDR398W	914
YLL011W	YDR449C	999
YLL011W	YER082C	999
YLL011W	YGL120C	915
YLL011W	YGL171W	932
YLL011W	YGR128C	998
YLL011W	YGR145W	997
YLL011W	YHR148W	998
YLL011W	YHR169W	945
YLL011W	YHR196W	999
YLL011W	YJL010C	983
YLL011W	YJL069C	915
YLL011W	YJL109C	999
YLL011W	YJR002W	999
YLL011W	YKR060W	997
YLL011W	YLR003C	916
YLL011W	YLR129W	998
YLL011W	YLR175W	920
YLL011W	YLR186W	997
YLL011W	YLR197W	998
YLL011W	YLR222C	993
YLL011W	YLR409C	998
YLL011W	YML093W	976
YLL011W	YMR093W	998
YLL011W	YMR229C	972
YLL011W	YMR290C	918
YLL011W	YMR308C	911
YLL011W	YNL075W	948
YLL011W	YNL132W	996
YLL011W	YOL010W	931
YLL011W	YOR004W	996
YLL011W	YOR078W	975
YLL011W	YOR145C	934
YLL011W	YOR310C	961
YLL011W	YPL012W	920
YLL011W	YPL126W	998
YLL011W	YPL217C	998

YLL011W	YPL240C	919
YLL011W	YPR112C	925
YLL011W	YPR137W	999
YLL011W	YPR144C	999
YLL012W	YBR041W	929
YLL012W	YHR072W	988
YLL012W	YLR100W	927
YLL013C	YHR121W	938
YLL013C	YLL009C	948
YLL014W	YLR023C	923
YLL015W	YDR135C	996
YLL018C	YBL076C	922
YLL018C	YDR037W	911
YLL018C	YDR341C	902
YLL018C	YGL105W	930
YLL018C	YGR124W	922
YLL018C	YGR264C	935
YLL018C	YHR020W	918
YLL018C	YJL130C	914
YLL018C	YLR027C	914
YLL018C	YNL220W	908
YLL018C	YOR168W	900
YLL018C	YPL160W	912
YLL018C	YPL240C	914
YLL021W	YBL105C	922
YLL021W	YBR089C-A	915
YLL021W	YCL027W	999
YLL021W	YCR002C	989
YLL021W	YCR009C	926
YLL021W	YDL225W	956
YLL021W	YDR054C	964
YLL021W	YER149C	999
YLL021W	YGR217W	904
YLL021W	YHR030C	999
YLL021W	YHR107C	988
YLL021W	YHR158C	929
YLL021W	YJL095W	989
YLL021W	YLR229C	939
YLL021W	YLR319C	998
YLL021W	YLR330W	976
YLL021W	YLR362W	996
YLL021W	YMR232W	976
YLL021W	YNL271C	995
YLL021W	YNL293W	996

YLL021W	YNL298W	965
YLL021W	YOL112W	996
YLL021W	YOR231W	997
YLL021W	YPL140C	924
YLL021W	YPR119W	925
YLL021W	YPR165W	986
YLL022C	YBR009C	999
YLL022C	YBR010W	997
YLL022C	YBR272C	981
YLL022C	YEL056W	999
YLL022C	YNL030W	999
YLL022C	YNL031C	999
YLL022C	YOL054W	994
YLL022C	YPL001W	999
YLL023C	YLL052C	923
YLL023C	YLR023C	923
YLL023C	YOR165W	919
YLL024C	YAL005C	999
YLL024C	YBL075C	997
YLL024C	YDL229W	971
YLL024C	YDR172W	977
YLL024C	YER103W	997
YLL024C	YER151C	970
YLL024C	YER161C	976
YLL024C	YGL073W	978
YLL024C	YJL034W	921
YLL024C	YJR032W	942
YLL024C	YKL068W	913
YLL024C	YMR186W	966
YLL024C	YNL007C	977
YLL024C	YNL064C	996
YLL024C	YNL209W	906
YLL024C	YOR027W	999
YLL024C	YOR232W	929
YLL024C	YPL106C	995
YLL024C	YPL240C	991
YLL026W	YAL005C	997
YLL026W	YBR101C	913
YLL026W	YBR126C	919
YLL026W	YBR155W	993
YLL026W	YBR169C	918
YLL026W	YCL028W	955
YLL026W	YDL229W	973
YLL026W	YDR172W	999

YLL026W	YER103W	959
YLL026W	YJR032W	998
YLL026W	YLR249W	968
YLL026W	YMR037C	989
YLL026W	YNL007C	936
YLL026W	YNL064C	995
YLL026W	YNL229C	984
YLL026W	YOR027W	998
YLL026W	YPL048W	990
YLL027W	YCL017C	903
YLL027W	YPL252C	942
YLL028W	YIL121W	936
YLL028W	YLL055W	923
YLL029W	YGL220W	974
YLL031C	YDR302W	963
YLL031C	YJL062W	982
YLL031C	YNL169C	906
YLL031C	YOR149C	951
YLL032C	YML119W	992
YLL034C	YDL031W	986
YLL034C	YDR060W	976
YLL034C	YDR087C	974
YLL034C	YDR496C	989
YLL034C	YER006W	983
YLL034C	YFL002C	974
YLL034C	YGL111W	998
YLL034C	YHR052W	987
YLL034C	YHR088W	979
YLL034C	YHR197W	981
YLL034C	YLL008W	984
YLL034C	YLR002C	978
YLL034C	YLR449W	993
YLL034C	YMR049C	979
YLL034C	YMR242C	935
YLL034C	YMR290C	966
YLL034C	YNL002C	980
YLL034C	YNL061W	981
YLL034C	YNL110C	943
YLL034C	YOL077C	988
YLL034C	YOR063W	939
YLL034C	YOR206W	987
YLL034C	YOR272W	994
YLL034C	YOR341W	954
YLL034C	YPL043W	979

YLL034C	YPL093W	982
YLL034C	YPL211W	994
YLL034C	YPR010C	935
YLL036C	YAL032C	999
YLL036C	YBR055C	994
YLL036C	YBR065C	999
YLL036C	YBR188C	999
YLL036C	YCR063W	999
YLL036C	YDL030W	998
YLL036C	YDL209C	999
YLL036C	YDR364C	999
YLL036C	YDR416W	999
YLL036C	YER172C	998
YLL036C	YFL017W-A	961
YLL036C	YGL120C	998
YLL036C	YGL128C	997
YLL036C	YGR129W	998
YLL036C	YGR278W	999
YLL036C	YHR165C	999
YLL036C	YJR050W	999
YLL036C	YKL095W	998
YLL036C	YKL173W	999
YLL036C	YLR117C	999
YLL036C	YLR147C	996
YLL036C	YLR424W	981
YLL036C	YML049C	997
YLL036C	YMR213W	999
YLL036C	YMR240C	996
YLL036C	YMR288W	999
YLL036C	YPL151C	999
YLL036C	YPL213W	999
YLL036C	YPR018W	906
YLL036C	YPR101W	999
YLL036C	YPR178W	934
YLL036C	YPR182W	959
YLL039C	YBL007C	956
YLL039C	YBL041W	902
YLL039C	YBL084C	902
YLL039C	YBR082C	995
YLL039C	YBR088C	999
YLL039C	YDL007W	988
YLL039C	YDL097C	972
YLL039C	YDL126C	996
YLL039C	YDL147W	971

YLL039C	YDL190C	999
YLL039C	YDL234C	956
YLL039C	YDR054C	980
YLL039C	YDR059C	905
YLL039C	YDR069C	993
YLL039C	YDR143C	913
YLL039C	YDR358W	939
YLL039C	YDR427W	970
YLL039C	YDR457W	972
YLL039C	YDR477W	935
YLL039C	YEL037C	999
YLL039C	YEL061C	967
YLL039C	YER012W	900
YLL039C	YER021W	971
YLL039C	YER094C	970
YLL039C	YER125W	999
YLL039C	YER143W	998
YLL039C	YFL010C	911
YLL039C	YFR004W	919
YLL039C	YFR010W	994
YLL039C	YFR050C	905
YLL039C	YFR052W	915
YLL039C	YGL011C	970
YLL039C	YGL206C	961
YLL039C	YGL240W	903
YLL039C	YGR135W	961
YLL039C	YGR232W	903
YLL039C	YHL007C	970
YLL039C	YHL015W	908
YLL039C	YHR027C	993
YLL039C	YHR108W	939
YLL039C	YHR166C	906
YLL039C	YHR200W	961
YLL039C	YIL075C	972
YLL039C	YIL148W	915
YLL039C	YJL001W	970
YLL039C	YJL172W	956
YLL039C	YJR123W	945
YLL039C	YKL022C	904
YLL039C	YKL145W	988
YLL039C	YKL213C	999
YLL039C	YKR094C	915
YLL039C	YLR167W	984
YLL039C	YML097C	999

YLL039C	YMR036C	970
YLL039C	YMR276W	998
YLL039C	YMR296C	956
YLL039C	YNL098C	956
YLL039C	YNL151C	969
YLL039C	YNL178W	906
YLL039C	YNR006W	996
YLL039C	YOL122C	956
YLL039C	YOR117W	970
YLL039C	YOR249C	996
YLL039C	YOR261C	970
YLL039C	YOR339C	903
YLL039C	YPL176C	956
YLL039C	YPL256C	962
YLL039C	YPR103W	970
YLL039C	YPR108W	970
YLL039C	YPR154W	909
YLL039C	YPR165W	956
YLL040C	YBR109C	974
YLL040C	YML092C	940
YLL040C	YNL238W	987
YLL040C	YPR103W	940
YLL041C	YDL120W	903
YLL041C	YDR178W	991
YLL041C	YGR244C	976
YLL041C	YIL125W	908
YLL041C	YJL045W	999
YLL041C	YKL141W	990
YLL041C	YKL148C	999
YLL041C	YMR118C	939
YLL041C	YOR142W	981
YLL041C	YPL262W	981
YLL042C	YBR217W	989
YLL042C	YHR171W	999
YLL042C	YPL149W	978
YLL043W	YDL022W	951
YLL043W	YHR030C	987
YLL043W	YLR113W	998
YLL043W	YOR089C	915
YLL045C	YAL035W	993
YLL045C	YBL027W	995
YLL045C	YBL072C	994
YLL045C	YBL087C	959
YLL045C	YBL092W	971

YLL045C	YBR031W	999
YLL045C	YBR048W	993
YLL045C	YBR084C-A	924
YLL045C	YBR118W	906
YLL045C	YBR142W	968
YLL045C	YBR181C	966
YLL045C	YBR189W	998
YLL045C	YBR191W	948
YLL045C	YCL011C	929
YLL045C	YCR031C	996
YLL045C	YDL014W	962
YLL045C	YDL031W	942
YLL045C	YDL061C	923
YLL045C	YDL075W	996
YLL045C	YDL081C	961
YLL045C	YDL082W	998
YLL045C	YDL083C	949
YLL045C	YDL130W	914
YLL045C	YDL136W	995
YLL045C	YDL191W	925
YLL045C	YDR012W	999
YLL045C	YDR025W	993
YLL045C	YDR060W	941
YLL045C	YDR064W	997
YLL045C	YDR101C	996
YLL045C	YDR382W	998
YLL045C	YDR385W	903
YLL045C	YDR418W	998
YLL045C	YDR447C	996
YLL045C	YDR450W	922
YLL045C	YDR471W	997
YLL045C	YDR496C	945
YLL045C	YDR500C	912
YLL045C	YEL054C	936
YLL045C	YER006W	946
YLL045C	YER056C-A	922
YLL045C	YER074W	994
YLL045C	YER102W	994
YLL045C	YER117W	910
YLL045C	YER131W	957
YLL045C	YFL034C-A	921
YLL045C	YFR001W	981
YLL045C	YFR031C-A	997
YLL045C	YFR032C-A	935

YLL045C	YGL030W	998
YLL045C	YGL031C	956
YLL045C	YGL076C	999
YLL045C	YGL099W	977
YLL045C	YGL103W	995
YLL045C	YGL123W	931
YLL045C	YGL135W	997
YLL045C	YGL147C	999
YLL045C	YGL189C	911
YLL045C	YGL246C	975
YLL045C	YGR027C	967
YLL045C	YGR034W	997
YLL045C	YGR085C	998
YLL045C	YGR103W	991
YLL045C	YGR118W	938
YLL045C	YGR148C	956
YLL045C	YGR214W	967
YLL045C	YHL001W	998
YLL045C	YHL015W	955
YLL045C	YHL033C	999
YLL045C	YHL034C	987
YLL045C	YHR010W	995
YLL045C	YHR021C	923
YLL045C	YHR052W	944
YLL045C	YHR088W	941
YLL045C	YHR141C	967
YLL045C	YHR203C	997
YLL045C	YIL018W	996
YLL045C	YIL052C	934
YLL045C	YIL069C	994
YLL045C	YIL133C	999
YLL045C	YIL148W	908
YLL045C	YJL136C	948
YLL045C	YJL177W	996
YLL045C	YJL189W	904
YLL045C	YJL190C	998
YLL045C	YJL191W	945
YLL045C	YJR094W-A	922
YLL045C	YJR123W	997
YLL045C	YJR145C	995
YLL045C	YKL006W	958
YLL045C	YKL009W	954
YLL045C	YKL156W	932
YLL045C	YKL172W	966

YLL045C	YKL180W	997
YLL045C	YKR057W	932
YLL045C	YKR081C	966
YLL045C	YKR094C	908
YLL045C	YLL008W	933
YLL045C	YLR002C	942
YLL045C	YLR029C	999
YLL045C	YLR048W	950
YLL045C	YLR061W	937
YLL045C	YLR075W	999
YLL045C	YLR167W	902
YLL045C	YLR185W	912
YLL045C	YLR196W	974
YLL045C	YLR264W	933
YLL045C	YLR287C-A	914
YLL045C	YLR325C	939
YLL045C	YLR333C	934
YLL045C	YLR340W	967
YLL045C	YLR344W	936
YLL045C	YLR367W	929
YLL045C	YLR388W	930
YLL045C	YLR406C	929
YLL045C	YLR432W	979
YLL045C	YLR441C	997
YLL045C	YLR448W	996
YLL045C	YML024W	935
YLL045C	YML026C	922
YLL045C	YML063W	998
YLL045C	YML073C	995
YLL045C	YMR121C	953
YLL045C	YMR142C	997
YLL045C	YMR194W	995
YLL045C	YMR230W	938
YLL045C	YMR242C	998
YLL045C	YMR260C	924
YLL045C	YMR290C	949
YLL045C	YNL002C	936
YLL045C	YNL061W	945
YLL045C	YNL067W	972
YLL045C	YNL069C	996
YLL045C	YNL096C	951
YLL045C	YNL112W	976
YLL045C	YNL132W	940
YLL045C	YNL162W	937

YLL045C	YNL178W	998
YLL045C	YNL301C	996
YLL045C	YNL302C	920
YLL045C	YOL039W	940
YLL045C	YOL040C	945
YLL045C	YOL041C	997
YLL045C	YOL077C	949
YLL045C	YOL120C	996
YLL045C	YOL121C	959
YLL045C	YOL127W	996
YLL045C	YOR063W	995
YLL045C	YOR096W	997
YLL045C	YOR133W	903
YLL045C	YOR167C	934
YLL045C	YOR182C	914
YLL045C	YOR206W	933
YLL045C	YOR234C	941
YLL045C	YOR272W	940
YLL045C	YOR293W	942
YLL045C	YOR312C	997
YLL045C	YOR369C	965
YLL045C	YPL012W	935
YLL045C	YPL043W	950
YLL045C	YPL079W	958
YLL045C	YPL081W	928
YLL045C	YPL090C	996
YLL045C	YPL093W	949
YLL045C	YPL131W	998
YLL045C	YPL143W	939
YLL045C	YPL198W	997
YLL045C	YPL211W	954
YLL045C	YPL220W	997
YLL045C	YPL249C-A	997
YLL045C	YPR016C	941
YLL045C	YPR043W	922
YLL045C	YPR080W	906
YLL045C	YPR102C	978
YLL045C	YPR132W	919
YLL048C	YJR148W	962
YLL048C	YNL238W	958
YLL049W	YER016W	953
YLL049W	YMR294W	986
YLL049W	YNR069C	926
YLL049W	YPL174C	984

YLL049W	YPL269W	986
YLL050C	YFL039C	999
YLL050C	YGR080W	944
YLL050C	YIL034C	953
YLL050C	YKL007W	975
YLL050C	YLR429W	918
YLL050C	YMR092C	999
YLL050C	YOR122C	977
YLL051C	YGL071W	948
YLL052C	YLL023C	923
YLL055W	YLL028W	923
YLL055W	YMR161W	923
YLL060C	YIR038C	940
YLL060C	YKL026C	938
YLL060C	YPL091W	935
YLR002C	YDL031W	991
YLR002C	YDR060W	999
YLR002C	YDR496C	981
YLR002C	YER006W	999
YLR002C	YER165W	927
YLR002C	YFL002C	994
YLR002C	YFR001W	992
YLR002C	YGL076C	927
YLR002C	YGL111W	984
YLR002C	YGR103W	996
YLR002C	YHR052W	998
YLR002C	YHR088W	983
YLR002C	YHR197W	991
YLR002C	YKL009W	980
YLR002C	YKL172W	980
YLR002C	YKR081C	999
YLR002C	YLL008W	989
YLR002C	YLL034C	978
YLR002C	YLL045C	942
YLR002C	YLR029C	929
YLR002C	YLR449W	993
YLR002C	YMR049C	986
YLR002C	YMR290C	982
YLR002C	YNL002C	990
YLR002C	YNL061W	990
YLR002C	YNL110C	993
YLR002C	YOL041C	979
YLR002C	YOL077C	990
YLR002C	YOL127W	933

YLR002C	YOR063W	935
YLR002C	YOR204W	931
YLR002C	YOR206W	999
YLR002C	YOR243C	982
YLR002C	YOR272W	999
YLR002C	YOR294W	996
YLR002C	YPL093W	972
YLR002C	YPL131W	952
YLR002C	YPL198W	946
YLR002C	YPL211W	992
YLR002C	YPR016C	999
YLR003C	YBL004W	969
YLR003C	YBR247C	914
YLR003C	YCL031C	975
YLR003C	YCL059C	952
YLR003C	YCR057C	980
YLR003C	YDL014W	942
YLR003C	YDL148C	911
YLR003C	YDL153C	929
YLR003C	YDR299W	943
YLR003C	YDR339C	912
YLR003C	YDR449C	972
YLR003C	YER082C	975
YLR003C	YGL171W	978
YLR003C	YGR090W	991
YLR003C	YGR128C	922
YLR003C	YGR145W	912
YLR003C	YHR148W	976
YLR003C	YHR169W	914
YLR003C	YHR196W	973
YLR003C	YJR002W	979
YLR003C	YKL099C	909
YLR003C	YKR060W	985
YLR003C	YLL011W	916
YLR003C	YLR129W	979
YLR003C	YLR175W	911
YLR003C	YLR197W	921
YLR003C	YLR222C	933
YLR003C	YLR409C	919
YLR003C	YML093W	914
YLR003C	YMR229C	924
YLR003C	YMR290C	910
YLR003C	YNL075W	923
YLR003C	YOR004W	930

YLR003C	YOR078W	973
YLR003C	YOR145C	978
YLR003C	YPL126W	909
YLR003C	YPL217C	936
YLR003C	YPR112C	969
YLR003C	YPR144C	910
YLR005W	YAR007C	977
YLR005W	YBL084C	906
YLR005W	YDL108W	970
YLR005W	YDL140C	984
YLR005W	YDR311W	999
YLR005W	YDR460W	999
YLR005W	YER162C	978
YLR005W	YER171W	999
YLR005W	YGL043W	916
YLR005W	YGR005C	980
YLR005W	YGR258C	997
YLR005W	YIL143C	995
YLR005W	YML095C	984
YLR005W	YMR201C	981
YLR005W	YNL135C	987
YLR005W	YNL312W	978
YLR005W	YOL051W	959
YLR005W	YPL022W	983
YLR005W	YPL122C	997
YLR005W	YPR025C	957
YLR005W	YPR056W	999
YLR005W	YPR070W	924
YLR006C	YCL032W	975
YLR006C	YCR073C	983
YLR006C	YDL013W	929
YLR006C	YDL235C	999
YLR006C	YER118C	998
YLR006C	YHL007C	967
YLR006C	YIL147C	996
YLR006C	YLR362W	999
YLR006C	YMR022W	987
YLR006C	YNR031C	999
YLR007W	YDL105W	904
YLR007W	YDR288W	920
YLR007W	YLR383W	957
YLR007W	YOL034W	990
YLR008C	YGR033C	980
YLR008C	YIL022W	999

YLR008C	YJL104W	999
YLR008C	YJL143W	999
YLR008C	YJR045C	967
YLR008C	YNR017W	999
YLR009W	YDR101C	984
YLR009W	YER126C	996
YLR009W	YGR103W	997
YLR009W	YHR052W	968
YLR009W	YHR170W	915
YLR009W	YJL122W	957
YLR009W	YKL009W	923
YLR009W	YKL021C	976
YLR009W	YKR081C	983
YLR009W	YLR196W	966
YLR009W	YMR290C	905
YLR009W	YNL002C	901
YLR009W	YNL061W	978
YLR009W	YNL110C	936
YLR009W	YNR053C	985
YLR009W	YOL077C	985
YLR009W	YPL093W	999
YLR009W	YPR016C	963
YLR010C	YDL220C	996
YLR010C	YDR082W	999
YLR014C	YEL021W	986
YLR014C	YKL216W	988
YLR014C	YLR420W	903
YLR015W	YAL011W	920
YLR015W	YAR003W	999
YLR015W	YBR175W	999
YLR015W	YBR231C	909
YLR015W	YBR258C	999
YLR015W	YDL140C	970
YLR015W	YDR334W	907
YLR015W	YDR359C	914
YLR015W	YDR469W	999
YLR015W	YDR485C	907
YLR015W	YHR119W	999
YLR015W	YJL168C	952
YLR015W	YKL018W	999
YLR015W	YML041C	909
YLR015W	YOL012C	909
YLR015W	YPL138C	999
YLR016C	YGL174W	981

YLR016C	YIR005W	999
YLR017W	YPR118W	909
YLR018C	YAR002W	949
YLR018C	YBL079W	991
YLR018C	YDL088C	995
YLR018C	YDL116W	958
YLR018C	YDL207W	977
YLR018C	YDR159W	974
YLR018C	YDR192C	934
YLR018C	YER107C	987
YLR018C	YFR002W	940
YLR018C	YGL100W	950
YLR018C	YGR119C	980
YLR018C	YIL115C	989
YLR018C	YJL061W	988
YLR018C	YKL057C	995
YLR018C	YKR082W	984
YLR018C	YLR335W	960
YLR018C	YLR347C	988
YLR018C	YML031W	984
YLR018C	YML103C	986
YLR018C	YMR047C	992
YLR018C	YMR129W	992
YLR018C	YMR153W	974
YLR018C	YOR257W	961
YLR018C	YPL125W	905
YLR018C	YPL169C	968
YLR019W	YDR040C	904
YLR019W	YLL010C	991
YLR021W	YDL020C	969
YLR021W	YHR200W	967
YLR021W	YPL144W	999
YLR022C	YHR087W	903
YLR022C	YNL163C	920
YLR022C	YPR016C	990
YLR023C	YJL056C	931
YLR023C	YLL014W	923
YLR023C	YLL023C	923
YLR023C	YOL101C	957
YLR024C	YDL020C	998
YLR024C	YGL058W	999
YLR024C	YKL145W	913
YLR024C	YMR100W	994
YLR025W	YDR069C	999

YLR025W	YDR486C	999
YLR025W	YGR122W	914
YLR025W	YHL027W	970
YLR025W	YIL162W	929
YLR025W	YJR102C	901
YLR025W	YKL002W	999
YLR025W	YKL041W	999
YLR025W	YKR035W-A	970
YLR025W	YLR181C	997
YLR025W	YMR077C	999
YLR025W	YOR275C	999
YLR025W	YPL002C	980
YLR025W	YPL084W	999
YLR025W	YPR173C	999
YLR026C	YBL040C	901
YLR026C	YBL050W	999
YLR026C	YBL102W	995
YLR026C	YBR080C	944
YLR026C	YDR164C	940
YLR026C	YDR189W	999
YLR026C	YDR468C	999
YLR026C	YEL036C	987
YLR026C	YFL038C	999
YLR026C	YGL223C	964
YLR026C	YGR120C	964
YLR026C	YHL031C	999
YLR026C	YIL004C	999
YLR026C	YIL109C	996
YLR026C	YKL006C-A	999
YLR026C	YKL196C	999
YLR026C	YKR014C	910
YLR026C	YLR078C	999
YLR026C	YLR093C	999
YLR026C	YLR268W	999
YLR026C	YML012W	901
YLR026C	YML067C	906
YLR026C	YMR197C	999
YLR026C	YNL049C	902
YLR026C	YOR327C	999
YLR026C	YPL218W	913
YLR026C	YPR181C	995
YLR027C	YAL012W	901
YLR027C	YBR218C	912
YLR027C	YDL078C	912

YLR027C	YGL062W	912
YLR027C	YGL252C	903
YLR027C	YIL116W	904
YLR027C	YJL101C	901
YLR027C	YKL085W	932
YLR027C	YKR097W	913
YLR027C	YLL018C	914
YLR027C	YOL033W	906
YLR027C	YOL058W	900
YLR027C	YOL126C	904
YLR027C	YPR035W	931
YLR027C	YPR145W	909
YLR028C	YBR084W	909
YLR028C	YBR248C	918
YLR028C	YBR263W	908
YLR028C	YCR073W-A	998
YLR028C	YDR408C	980
YLR028C	YDR441C	901
YLR028C	YER133W	995
YLR028C	YER177W	989
YLR028C	YGL234W	915
YLR028C	YGR204W	959
YLR028C	YHR216W	902
YLR028C	YKL128C	910
YLR028C	YLR058C	907
YLR028C	YLR359W	965
YLR028C	YLR432W	902
YLR028C	YML022W	907
YLR028C	YML056C	902
YLR028C	YMR120C	999
YLR028C	YMR300C	980
YLR028C	YNL220W	942
YLR028C	YNR034W	908
YLR028C	YOR236W	902
YLR029C	YAL035W	924
YLR029C	YBL027W	999
YLR029C	YBL072C	994
YLR029C	YBL087C	992
YLR029C	YBL092W	999
YLR029C	YBR031W	998
YLR029C	YBR048W	997
YLR029C	YBR084C-A	998
YLR029C	YBR118W	936
YLR029C	YBR142W	947

YLR029C	YBR181C	996
YLR029C	YBR189W	999
YLR029C	YBR191W	999
YLR029C	YCR031C	999
YLR029C	YDL014W	950
YLR029C	YDL031W	931
YLR029C	YDL061C	965
YLR029C	YDL075W	999
YLR029C	YDL081C	907
YLR029C	YDL082W	997
YLR029C	YDL083C	972
YLR029C	YDL130W	925
YLR029C	YDL136W	994
YLR029C	YDL191W	929
YLR029C	YDR012W	999
YLR029C	YDR025W	996
YLR029C	YDR060W	940
YLR029C	YDR064W	999
YLR029C	YDR382W	999
YLR029C	YDR418W	997
YLR029C	YDR447C	996
YLR029C	YDR450W	995
YLR029C	YDR471W	998
YLR029C	YDR496C	935
YLR029C	YDR500C	936
YLR029C	YEL054C	928
YLR029C	YER056C-A	969
YLR029C	YER074W	996
YLR029C	YER102W	996
YLR029C	YER117W	946
YLR029C	YER131W	979
YLR029C	YFL034C-A	909
YLR029C	YFR031C-A	999
YLR029C	YFR032C-A	996
YLR029C	YGL030W	999
YLR029C	YGL031C	995
YLR029C	YGL076C	999
YLR029C	YGL103W	999
YLR029C	YGL123W	991
YLR029C	YGL135W	999
YLR029C	YGL147C	999
YLR029C	YGL189C	921
YLR029C	YGR027C	994
YLR029C	YGR034W	997

YLR029C	YGR085C	998
YLR029C	YGR103W	937
YLR029C	YGR118W	981
YLR029C	YGR148C	985
YLR029C	YGR214W	998
YLR029C	YHL001W	992
YLR029C	YHL015W	973
YLR029C	YHL033C	999
YLR029C	YHR010W	978
YLR029C	YHR021C	917
YLR029C	YHR089C	979
YLR029C	YHR141C	992
YLR029C	YHR203C	997
YLR029C	YIL018W	996
YLR029C	YIL052C	992
YLR029C	YIL069C	996
YLR029C	YIL133C	999
YLR029C	YIL148W	935
YLR029C	YJL136C	941
YLR029C	YJL177W	999
YLR029C	YJL189W	919
YLR029C	YJL190C	995
YLR029C	YJL191W	971
YLR029C	YJR094W-A	997
YLR029C	YJR123W	999
YLR029C	YJR145C	994
YLR029C	YKL006W	998
YLR029C	YKL156W	962
YLR029C	YKL172W	927
YLR029C	YKL180W	999
YLR029C	YKR057W	918
YLR029C	YKR081C	938
YLR029C	YLL045C	999
YLR029C	YLR002C	929
YLR029C	YLR048W	994
YLR029C	YLR061W	975
YLR029C	YLR075W	999
YLR029C	YLR167W	929
YLR029C	YLR185W	993
YLR029C	YLR196W	972
YLR029C	YLR264W	963
YLR029C	YLR287C-A	996
YLR029C	YLR325C	916
YLR029C	YLR333C	944

YLR029C	YLR340W	999
YLR029C	YLR344W	996
YLR029C	YLR367W	968
YLR029C	YLR388W	980
YLR029C	YLR406C	910
YLR029C	YLR432W	978
YLR029C	YLR441C	937
YLR029C	YLR448W	999
YLR029C	YML024W	974
YLR029C	YML026C	908
YLR029C	YML063W	999
YLR029C	YML073C	996
YLR029C	YMR121C	920
YLR029C	YMR142C	935
YLR029C	YMR194W	998
YLR029C	YMR229C	944
YLR029C	YMR230W	945
YLR029C	YMR242C	997
YLR029C	YMR260C	906
YLR029C	YMR290C	947
YLR029C	YNL002C	934
YLR029C	YNL067W	992
YLR029C	YNL069C	997
YLR029C	YNL096C	997
YLR029C	YNL132W	938
YLR029C	YNL162W	999
YLR029C	YNL178W	999
YLR029C	YNL301C	999
YLR029C	YNL302C	919
YLR029C	YOL039W	931
YLR029C	YOL040C	997
YLR029C	YOL041C	988
YLR029C	YOL077C	951
YLR029C	YOL120C	998
YLR029C	YOL121C	919
YLR029C	YOL127W	999
YLR029C	YOR063W	999
YLR029C	YOR096W	999
YLR029C	YOR133W	916
YLR029C	YOR167C	940
YLR029C	YOR182C	908
YLR029C	YOR204W	933
YLR029C	YOR206W	931
YLR029C	YOR234C	974

YLR029C	YOR293W	975
YLR029C	YOR294W	985
YLR029C	YOR312C	997
YLR029C	YOR369C	951
YLR029C	YPL079W	957
YLR029C	YPL081W	948
YLR029C	YPL090C	995
YLR029C	YPL093W	945
YLR029C	YPL131W	999
YLR029C	YPL143W	970
YLR029C	YPL198W	997
YLR029C	YPL220W	997
YLR029C	YPL249C-A	929
YLR029C	YPR043W	957
YLR029C	YPR080W	936
YLR029C	YPR102C	999
YLR029C	YPR132W	939
YLR032W	YBR088C	999
YLR032W	YCR066W	999
YLR032W	YDL064W	985
YLR032W	YDL074C	989
YLR032W	YDR076W	919
YLR032W	YDR092W	999
YLR032W	YDR409W	976
YLR032W	YDR419W	976
YLR032W	YER095W	991
YLR032W	YER171W	906
YLR032W	YGL058W	998
YLR032W	YGL087C	999
YLR032W	YGL163C	979
YLR032W	YJL092W	999
YLR032W	YJR043C	910
YLR032W	YML028W	902
YLR032W	YML032C	940
YLR032W	YML095C	902
YLR032W	YNL218W	983
YLR032W	YOR033C	979
YLR032W	YPL022W	992
YLR032W	YPL167C	998
YLR033W	YBR089C-A	978
YLR033W	YBR245C	999
YLR033W	YCR020W-B	999
YLR033W	YCR052W	999
YLR033W	YDR303C	999

YLR033W	YER148W	923
YLR033W	YFR037C	999
YLR033W	YGR056W	999
YLR033W	YGR275W	999
YLR033W	YHR056C	999
YLR033W	YIL126W	999
YLR033W	YKR001C	970
YLR033W	YKR008W	999
YLR033W	YLR182W	916
YLR033W	YLR321C	999
YLR033W	YLR357W	999
YLR033W	YML127W	999
YLR033W	YMR033W	999
YLR033W	YMR091C	999
YLR033W	YPL082C	970
YLR033W	YPR034W	999
YLR034C	YFL041W	944
YLR034C	YMR058W	946
YLR034C	YPL202C	917
YLR035C	YFL003C	920
YLR035C	YMR167W	999
YLR038C	Q0045	983
YLR038C	Q0250	988
YLR038C	Q0275	988
YLR038C	YDL067C	990
YLR038C	YGL187C	981
YLR038C	YGL191W	991
YLR038C	YHR051W	985
YLR038C	YIL111W	978
YLR038C	YLR395C	985
YLR038C	YMR256C	989
YLR038C	YNL052W	981
YLR039C	YBR164C	920
YLR039C	YDR100W	961
YLR039C	YDR137W	999
YLR039C	YEL031W	964
YLR039C	YGR229C	918
YLR039C	YHL031C	959
YLR039C	YJL004C	969
YLR039C	YLR262C	998
YLR039C	YLR309C	955
YLR039C	YML071C	955
YLR039C	YOR070C	905
YLR039C	YOR216C	915

YLR040C	YJR004C	962
YLR040C	YKL178C	982
YLR043C	YBR080C	976
YLR043C	YDR353W	996
YLR043C	YER042W	903
YLR043C	YER070W	989
YLR043C	YGR180C	900
YLR043C	YGR209C	999
YLR043C	YHR106W	976
YLR043C	YIL066C	989
YLR043C	YJL026W	989
YLR043C	YKL067W	910
YLR043C	YML007W	982
YLR043C	YPL091W	998
YLR043C	YPR167C	987
YLR044C	YAL038W	965
YLR044C	YBR196C	979
YLR044C	YCR012W	997
YLR044C	YDR081C	955
YLR044C	YER178W	931
YLR044C	YGL256W	901
YLR044C	YGR087C	969
YLR044C	YHR174W	998
YLR044C	YJR148W	927
YLR044C	YKL060C	978
YLR044C	YKL182W	958
YLR044C	YLR134W	999
YLR044C	YLR249W	975
YLR044C	YMR169C	930
YLR044C	YNL236W	909
YLR044C	YOL086C	997
YLR044C	YOR374W	924
YLR044C	YPL061W	920
YLR045C	YAL034W-A	902
YLR045C	YAL047C	998
YLR045C	YCL029C	999
YLR045C	YEL061C	942
YLR045C	YER016W	999
YLR045C	YFL037W	997
YLR045C	YGL061C	963
YLR045C	YGR113W	996
YLR045C	YGR140W	987
YLR045C	YIL144W	961
YLR045C	YJL030W	990

YLR045C	YJR112W	976
YLR045C	YKR037C	963
YLR045C	YML085C	996
YLR045C	YML124C	979
YLR045C	YMR094W	926
YLR045C	YMR117C	984
YLR045C	YOR195W	928
YLR045C	YPL269W	998
YLR045C	YPR141C	906
YLR047C	YGL071W	942
YLR048W	YAL003W	978
YLR048W	YAL035W	941
YLR048W	YBL027W	920
YLR048W	YBL072C	925
YLR048W	YBL087C	928
YLR048W	YBL092W	952
YLR048W	YBR031W	976
YLR048W	YBR048W	955
YLR048W	YBR079C	945
YLR048W	YBR084C-A	980
YLR048W	YBR118W	921
YLR048W	YBR181C	906
YLR048W	YBR189W	999
YLR048W	YBR191W	999
YLR048W	YCR031C	995
YLR048W	YDL061C	995
YLR048W	YDL075W	999
YLR048W	YDL081C	932
YLR048W	YDL082W	945
YLR048W	YDL130W	948
YLR048W	YDL136W	957
YLR048W	YDL191W	914
YLR048W	YDR012W	983
YLR048W	YDR025W	916
YLR048W	YDR041W	901
YLR048W	YDR064W	999
YLR048W	YDR382W	999
YLR048W	YDR385W	916
YLR048W	YDR418W	999
YLR048W	YDR429C	916
YLR048W	YDR447C	925
YLR048W	YDR450W	943
YLR048W	YDR471W	977
YLR048W	YDR500C	971

YLR048W	YEL054C	910
YLR048W	YER025W	994
YLR048W	YER056C-A	997
YLR048W	YER074W	909
YLR048W	YER102W	984
YLR048W	YER117W	984
YLR048W	YER131W	964
YLR048W	YER165W	974
YLR048W	YFL034C-A	904
YLR048W	YFR031C-A	913
YLR048W	YFR032C-A	933
YLR048W	YGL030W	997
YLR048W	YGL031C	952
YLR048W	YGL076C	979
YLR048W	YGL103W	999
YLR048W	YGL123W	999
YLR048W	YGL135W	909
YLR048W	YGL147C	939
YLR048W	YGL189C	905
YLR048W	YGR027C	982
YLR048W	YGR034W	911
YLR048W	YGR085C	995
YLR048W	YGR118W	999
YLR048W	YGR148C	981
YLR048W	YGR162W	932
YLR048W	YGR214W	999
YLR048W	YHL001W	999
YLR048W	YHL015W	995
YLR048W	YHL033C	999
YLR048W	YHR010W	987
YLR048W	YHR021C	926
YLR048W	YHR141C	917
YLR048W	YHR203C	984
YLR048W	YIL018W	913
YLR048W	YIL052C	999
YLR048W	YIL069C	909
YLR048W	YIL133C	952
YLR048W	YIL148W	969
YLR048W	YJL136C	982
YLR048W	YJL177W	999
YLR048W	YJL189W	952
YLR048W	YJL190C	999
YLR048W	YJL191W	988
YLR048W	YJR094W-A	985

YLR048W	YJR123W	999
YLR048W	YJR145C	913
YLR048W	YKL006W	999
YLR048W	YKL156W	991
YLR048W	YKL180W	999
YLR048W	YKR057W	952
YLR048W	YKR059W	970
YLR048W	YKR094C	905
YLR048W	YLL045C	950
YLR048W	YLR029C	994
YLR048W	YLR060W	998
YLR048W	YLR061W	999
YLR048W	YLR075W	998
YLR048W	YLR167W	954
YLR048W	YLR185W	971
YLR048W	YLR249W	998
YLR048W	YLR264W	998
YLR048W	YLR325C	909
YLR048W	YLR333C	936
YLR048W	YLR340W	998
YLR048W	YLR344W	996
YLR048W	YLR367W	989
YLR048W	YLR388W	999
YLR048W	YLR406C	911
YLR048W	YLR441C	936
YLR048W	YLR448W	999
YLR048W	YML024W	995
YLR048W	YML026C	943
YLR048W	YML063W	997
YLR048W	YML073C	924
YLR048W	YML106W	944
YLR048W	YMR121C	918
YLR048W	YMR142C	956
YLR048W	YMR194W	997
YLR048W	YMR230W	957
YLR048W	YMR242C	927
YLR048W	YMR260C	909
YLR048W	YMR309C	913
YLR048W	YNL067W	995
YLR048W	YNL069C	953
YLR048W	YNL081C	902
YLR048W	YNL096C	911
YLR048W	YNL162W	917
YLR048W	YNL178W	998

YLR048W	YNL209W	970
YLR048W	YNL301C	991
YLR048W	YNL302C	916
YLR048W	YNR037C	902
YLR048W	YOL039W	965
YLR048W	YOL040C	951
YLR048W	YOL120C	927
YLR048W	YOL121C	936
YLR048W	YOL127W	999
YLR048W	YOL139C	913
YLR048W	YOR063W	998
YLR048W	YOR096W	999
YLR048W	YOR133W	956
YLR048W	YOR167C	955
YLR048W	YOR234C	995
YLR048W	YOR293W	978
YLR048W	YOR312C	941
YLR048W	YOR361C	931
YLR048W	YOR369C	926
YLR048W	YPL037C	956
YLR048W	YPL079W	978
YLR048W	YPL081W	970
YLR048W	YPL090C	906
YLR048W	YPL131W	998
YLR048W	YPL143W	987
YLR048W	YPL198W	933
YLR048W	YPL220W	975
YLR048W	YPL237W	921
YLR048W	YPL249C-A	948
YLR048W	YPR043W	960
YLR048W	YPR080W	911
YLR048W	YPR102C	996
YLR048W	YPR132W	974
YLR048W	YPR163C	918
YLR051C	YLR119W	999
YLR052W	YDL002C	999
YLR052W	YDR190C	990
YLR052W	YER092W	998
YLR052W	YFL013C	984
YLR052W	YFL039C	996
YLR052W	YGL150C	999
YLR052W	YJL081C	988
YLR052W	YNL059C	996
YLR052W	YNL215W	987

YLR052W	YOR141C	997
YLR052W	YPL129W	991
YLR052W	YPL235W	989
YLR055C	YAL011W	929
YLR055C	YBR081C	999
YLR055C	YBR111W-A	959
YLR055C	YBR198C	999
YLR055C	YBR231C	931
YLR055C	YCL010C	999
YLR055C	YCL030C	912
YLR055C	YCR093W	982
YLR055C	YDR145W	999
YLR055C	YDR167W	999
YLR055C	YDR176W	999
YLR055C	YDR334W	928
YLR055C	YDR392W	999
YLR055C	YDR448W	999
YLR055C	YDR485C	927
YLR055C	YER148W	999
YLR055C	YER164W	924
YLR055C	YGL066W	999
YLR055C	YGL112C	999
YLR055C	YGR252W	999
YLR055C	YGR274C	931
YLR055C	YHR099W	999
YLR055C	YLR085C	958
YLR055C	YML041C	928
YLR055C	YMR005W	952
YLR055C	YMR223W	999
YLR055C	YMR227C	988
YLR055C	YMR236W	999
YLR055C	YNL107W	932
YLR055C	YOL012C	989
YLR055C	YOL148C	999
YLR055C	YOR202W	953
YLR055C	YPL047W	999
YLR055C	YPL254W	999
YLR056W	YER044C	999
YLR056W	YGL001C	998
YLR056W	YGL013C	976
YLR056W	YGR060W	999
YLR056W	YHR007C	980
YLR056W	YHR072W	998
YLR056W	YHR190W	999

YLR056W	YLR100W	999
YLR056W	YML008C	999
YLR056W	YMR015C	972
YLR056W	YMR202W	971
YLR056W	YNL280C	999
YLR058C	YBR084W	994
YLR058C	YBR263W	985
YLR058C	YCL064C	907
YLR058C	YDL106C	931
YLR058C	YDR019C	981
YLR058C	YDR232W	915
YLR058C	YDR408C	924
YLR058C	YEL046C	999
YLR058C	YFL030W	983
YLR058C	YGL125W	934
YLR058C	YGR155W	920
YLR058C	YGR204W	999
YLR058C	YGR208W	966
YLR058C	YKR080W	963
YLR058C	YKR092C	942
YLR058C	YKR099W	995
YLR058C	YLR028C	907
YLR058C	YMR120C	917
YLR058C	YMR189W	976
YLR058C	YOR074C	940
YLR058C	YOR236W	918
YLR058C	YOR241W	991
YLR058C	YPL023C	900
YLR059C	YGR276C	940
YLR059C	YLR107W	939
YLR059C	YMR302C	927
YLR059C	YPR024W	933
YLR060W	YBR191W	940
YLR060W	YDR064W	979
YLR060W	YER056C-A	944
YLR060W	YFL022C	999
YLR060W	YHL033C	904
YLR060W	YHR203C	953
YLR060W	YJL177W	963
YLR060W	YJR105W	916
YLR060W	YKL006W	917
YLR060W	YKL180W	913
YLR060W	YLR048W	998
YLR060W	YLR061W	977

YLR060W	YLR185W	923
YLR060W	YLR249W	966
YLR060W	YLR344W	910
YLR060W	YLR388W	965
YLR060W	YLR448W	972
YLR060W	YNL301C	942
YLR060W	YPL037C	971
YLR060W	YPL131W	918
YLR061W	YAL003W	942
YLR061W	YAL035W	929
YLR061W	YBL027W	919
YLR061W	YBL072C	915
YLR061W	YBL087C	912
YLR061W	YBL092W	934
YLR061W	YBR031W	964
YLR061W	YBR048W	935
YLR061W	YBR084C-A	998
YLR061W	YBR118W	903
YLR061W	YBR181C	901
YLR061W	YBR189W	999
YLR061W	YBR191W	999
YLR061W	YCR031C	995
YLR061W	YDL061C	998
YLR061W	YDL075W	999
YLR061W	YDL081C	921
YLR061W	YDL082W	924
YLR061W	YDL130W	958
YLR061W	YDL136W	971
YLR061W	YDL191W	913
YLR061W	YDL192W	942
YLR061W	YDR012W	975
YLR061W	YDR064W	999
YLR061W	YDR382W	999
YLR061W	YDR385W	911
YLR061W	YDR418W	995
YLR061W	YDR447C	914
YLR061W	YDR450W	914
YLR061W	YDR471W	991
YLR061W	YDR500C	995
YLR061W	YEL054C	923
YLR061W	YER056C-A	999
YLR061W	YER074W	905
YLR061W	YER102W	994
YLR061W	YER117W	996

YLR061W	YER131W	960
YLR061W	YFL034C-A	905
YLR061W	YFR031C-A	901
YLR061W	YFR032C-A	923
YLR061W	YGL030W	999
YLR061W	YGL031C	939
YLR061W	YGL076C	960
YLR061W	YGL103W	999
YLR061W	YGL123W	999
YLR061W	YGL135W	923
YLR061W	YGL147C	913
YLR061W	YGL189C	915
YLR061W	YGR027C	989
YLR061W	YGR034W	924
YLR061W	YGR085C	981
YLR061W	YGR118W	999
YLR061W	YGR148C	993
YLR061W	YGR214W	995
YLR061W	YHL001W	998
YLR061W	YHL015W	986
YLR061W	YHL033C	998
YLR061W	YHR010W	994
YLR061W	YHR021C	918
YLR061W	YHR141C	931
YLR061W	YHR203C	996
YLR061W	YIL018W	901
YLR061W	YIL052C	999
YLR061W	YIL069C	905
YLR061W	YIL133C	929
YLR061W	YIL148W	996
YLR061W	YJL136C	974
YLR061W	YJL177W	997
YLR061W	YJL189W	966
YLR061W	YJL190C	909
YLR061W	YJL191W	994
YLR061W	YJR094W-A	999
YLR061W	YJR123W	999
YLR061W	YJR145C	932
YLR061W	YKL006W	999
YLR061W	YKL156W	999
YLR061W	YKL180W	999
YLR061W	YKR057W	924
YLR061W	YKR094C	906
YLR061W	YLL045C	937

YLR061W	YLR029C	975
YLR061W	YLR048W	999
YLR061W	YLR060W	977
YLR061W	YLR075W	999
YLR061W	YLR167W	992
YLR061W	YLR185W	997
YLR061W	YLR249W	942
YLR061W	YLR264W	997
YLR061W	YLR287C-A	908
YLR061W	YLR333C	914
YLR061W	YLR340W	994
YLR061W	YLR344W	999
YLR061W	YLR367W	990
YLR061W	YLR388W	999
YLR061W	YLR406C	922
YLR061W	YLR441C	921
YLR061W	YLR448W	999
YLR061W	YML024W	999
YLR061W	YML026C	914
YLR061W	YML063W	994
YLR061W	YML073C	916
YLR061W	YMR121C	911
YLR061W	YMR142C	934
YLR061W	YMR194W	999
YLR061W	YMR230W	951
YLR061W	YMR242C	917
YLR061W	YMR260C	914
YLR061W	YNL067W	980
YLR061W	YNL069C	928
YLR061W	YNL096C	925
YLR061W	YNL162W	931
YLR061W	YNL178W	999
YLR061W	YNL209W	954
YLR061W	YNL301C	994
YLR061W	YNL302C	919
YLR061W	YOL039W	923
YLR061W	YOL040C	912
YLR061W	YOL120C	904
YLR061W	YOL121C	920
YLR061W	YOL127W	999
YLR061W	YOR063W	994
YLR061W	YOR096W	997
YLR061W	YOR133W	943
YLR061W	YOR167C	953

YLR061W	YOR182C	908
YLR061W	YOR234C	995
YLR061W	YOR293W	975
YLR061W	YOR312C	932
YLR061W	YOR369C	922
YLR061W	YPL037C	999
YLR061W	YPL079W	994
YLR061W	YPL081W	961
YLR061W	YPL090C	901
YLR061W	YPL131W	997
YLR061W	YPL143W	997
YLR061W	YPL198W	923
YLR061W	YPL220W	995
YLR061W	YPL249C-A	967
YLR061W	YPR043W	960
YLR061W	YPR080W	903
YLR061W	YPR102C	996
YLR061W	YPR132W	995
YLR064W	YBR162W-A	923
YLR066W	YIR022W	999
YLR066W	YJR010C-A	998
YLR066W	YML055W	999
YLR067C	YDL044C	939
YLR067C	YER153C	910
YLR067C	YGR222W	909
YLR067C	YMR257C	936
YLR069C	YBL038W	918
YLR069C	YGR220C	934
YLR069C	YHR147C	939
YLR069C	YJR113C	905
YLR069C	YNL185C	915
YLR069C	YNR036C	959
YLR070C	YGR194C	973
YLR070C	YHR104W	973
YLR070C	YPL240C	902
YLR071C	YBL093C	999
YLR071C	YBR154C	980
YLR071C	YBR193C	999
YLR071C	YBR253W	999
YLR071C	YBR279W	919
YLR071C	YCR081W	995
YLR071C	YCR084C	935
YLR071C	YDL005C	999
YLR071C	YDL052C	924

YLR071C	YDL108W	987
YLR071C	YDL140C	999
YLR071C	YDR308C	999
YLR071C	YDR404C	980
YLR071C	YDR443C	999
YLR071C	YER022W	999
YLR071C	YGL025C	999
YLR071C	YGL127C	999
YLR071C	YGL151W	998
YLR071C	YGR104C	999
YLR071C	YHR041C	999
YLR071C	YHR058C	999
YLR071C	YIL021W	999
YLR071C	YJL140W	999
YLR071C	YML007W	974
YLR071C	YMR112C	999
YLR071C	YNL236W	999
YLR071C	YNR010W	999
YLR071C	YOL051W	999
YLR071C	YOL135C	999
YLR071C	YOR151C	999
YLR071C	YOR174W	999
YLR071C	YPL042C	999
YLR071C	YPL129W	993
YLR071C	YPR070W	999
YLR071C	YPR168W	999
YLR071C	YPR187W	980
YLR072W	YHR117W	998
YLR074C	YCR072C	999
YLR074C	YDR101C	939
YLR074C	YER006W	978
YLR074C	YER126C	999
YLR074C	YGR103W	960
YLR074C	YGR245C	984
YLR074C	YHR052W	925
YLR074C	YHR085W	922
YLR074C	YHR197W	983
YLR074C	YKL009W	961
YLR074C	YKR081C	971
YLR074C	YLR106C	955
YLR074C	YLR325C	974
YLR074C	YNL002C	959
YLR074C	YNL110C	969
YLR074C	YNL182C	986

YLR074C	YNR053C	998
YLR074C	YOL127W	978
YLR074C	YPL093W	999
YLR074C	YPR016C	973
YLR075W	YAL003W	902
YLR075W	YAL035W	928
YLR075W	YBL027W	999
YLR075W	YBL072C	997
YLR075W	YBL087C	995
YLR075W	YBL092W	996
YLR075W	YBR031W	998
YLR075W	YBR048W	996
YLR075W	YBR084C-A	998
YLR075W	YBR084W	976
YLR075W	YBR118W	919
YLR075W	YBR181C	997
YLR075W	YBR189W	999
YLR075W	YBR191W	999
YLR075W	YCR031C	998
YLR075W	YDL014W	957
YLR075W	YDL061C	980
YLR075W	YDL075W	999
YLR075W	YDL081C	960
YLR075W	YDL082W	996
YLR075W	YDL083C	970
YLR075W	YDL130W	948
YLR075W	YDL136W	999
YLR075W	YDL191W	939
YLR075W	YDL192W	982
YLR075W	YDR012W	997
YLR075W	YDR025W	996
YLR075W	YDR064W	999
YLR075W	YDR077W	974
YLR075W	YDR382W	999
YLR075W	YDR418W	999
YLR075W	YDR447C	996
YLR075W	YDR450W	997
YLR075W	YDR471W	999
YLR075W	YDR500C	989
YLR075W	YEL054C	987
YLR075W	YER056C-A	994
YLR075W	YER074W	996
YLR075W	YER102W	997
YLR075W	YER117W	998

YLR075W	YER131W	948
YLR075W	YFL034C-A	900
YLR075W	YFR031C-A	999
YLR075W	YFR032C-A	993
YLR075W	YFR033C	931
YLR075W	YGL030W	999
YLR075W	YGL031C	999
YLR075W	YGL076C	999
YLR075W	YGL099W	982
YLR075W	YGL103W	999
YLR075W	YGL123W	999
YLR075W	YGL135W	999
YLR075W	YGL147C	994
YLR075W	YGL189C	934
YLR075W	YGR027C	973
YLR075W	YGR034W	997
YLR075W	YGR085C	999
YLR075W	YGR118W	998
YLR075W	YGR148C	990
YLR075W	YGR214W	978
YLR075W	YHL001W	999
YLR075W	YHL015W	988
YLR075W	YHL033C	999
YLR075W	YHR010W	965
YLR075W	YHR021C	927
YLR075W	YHR141C	999
YLR075W	YHR170W	999
YLR075W	YHR203C	999
YLR075W	YIL018W	997
YLR075W	YIL052C	999
YLR075W	YIL069C	931
YLR075W	YIL133C	999
YLR075W	YIL148W	975
YLR075W	YIR012W	999
YLR075W	YJL136C	973
YLR075W	YJL177W	999
YLR075W	YJL189W	950
YLR075W	YJL190C	995
YLR075W	YJL191W	964
YLR075W	YJR094W-A	999
YLR075W	YJR123W	999
YLR075W	YJR145C	903
YLR075W	YKL006W	998
YLR075W	YKL156W	969

YLR075W	YKL180W	999
YLR075W	YKR057W	922
YLR075W	YLL045C	999
YLR075W	YLR029C	999
YLR075W	YLR048W	998
YLR075W	YLR061W	999
YLR075W	YLR150W	989
YLR075W	YLR153C	902
YLR075W	YLR167W	996
YLR075W	YLR180W	987
YLR075W	YLR185W	999
YLR075W	YLR249W	940
YLR075W	YLR264W	974
YLR075W	YLR287C-A	995
YLR075W	YLR325C	937
YLR075W	YLR333C	935
YLR075W	YLR340W	999
YLR075W	YLR344W	999
YLR075W	YLR367W	952
YLR075W	YLR388W	996
YLR075W	YLR406C	902
YLR075W	YLR441C	996
YLR075W	YLR448W	999
YLR075W	YML024W	999
YLR075W	YML026C	907
YLR075W	YML063W	998
YLR075W	YML073C	996
YLR075W	YMR121C	957
YLR075W	YMR142C	996
YLR075W	YMR194W	999
YLR075W	YMR230W	947
YLR075W	YMR242C	996
YLR075W	YMR260C	912
YLR075W	YNL067W	979
YLR075W	YNL069C	997
YLR075W	YNL096C	995
YLR075W	YNL112W	974
YLR075W	YNL132W	944
YLR075W	YNL162W	920
YLR075W	YNL178W	999
YLR075W	YNL301C	999
YLR075W	YNL302C	918
YLR075W	YOL011W	917
YLR075W	YOL039W	916

YLR075W	YOL040C	966
YLR075W	YOL120C	935
YLR075W	YOL121C	918
YLR075W	YOL127W	999
YLR075W	YOR063W	999
YLR075W	YOR096W	999
YLR075W	YOR133W	931
YLR075W	YOR167C	931
YLR075W	YOR234C	999
YLR075W	YOR293W	958
YLR075W	YOR312C	996
YLR075W	YOR369C	953
YLR075W	YPL037C	999
YLR075W	YPL079W	967
YLR075W	YPL081W	947
YLR075W	YPL090C	994
YLR075W	YPL131W	999
YLR075W	YPL143W	974
YLR075W	YPL193W	939
YLR075W	YPL198W	997
YLR075W	YPL220W	999
YLR075W	YPL249C-A	998
YLR075W	YPR043W	976
YLR075W	YPR080W	906
YLR075W	YPR102C	998
YLR075W	YPR132W	997
YLR076C	YDR427W	998
YLR076C	YOR362C	999
YLR078C	YBL050W	962
YLR078C	YBR102C	962
YLR078C	YDL058W	943
YLR078C	YDL145C	903
YLR078C	YDL192W	998
YLR078C	YDR189W	999
YLR078C	YDR238C	911
YLR078C	YER122C	955
YLR078C	YFL038C	991
YLR078C	YGL137W	976
YLR078C	YHL031C	999
YLR078C	YIL004C	999
YLR078C	YKL006C-A	999
YLR078C	YKL196C	999
YLR078C	YLR026C	999
YLR078C	YLR268W	999

YLR078C	YNL287W	988
YLR078C	YOR216C	967
YLR078C	YOR327C	998
YLR079W	YAL040C	998
YLR079W	YAR019C	999
YLR079W	YBR135W	999
YLR079W	YBR160W	999
YLR079W	YDL132W	997
YLR079W	YDL155W	999
YLR079W	YDR054C	999
YLR079W	YDR146C	999
YLR079W	YDR328C	980
YLR079W	YEL037C	999
YLR079W	YFL009W	999
YLR079W	YFR028C	999
YLR079W	YGL003C	999
YLR079W	YGL019W	909
YLR079W	YGR092W	999
YLR079W	YGR108W	915
YLR079W	YGR109C	959
YLR079W	YHR118C	952
YLR079W	YHR200W	999
YLR079W	YIL035C	909
YLR079W	YJL106W	996
YLR079W	YJL187C	992
YLR079W	YJL194W	999
YLR079W	YLR113W	934
YLR079W	YLR210W	999
YLR079W	YML064C	978
YLR079W	YMR001C	996
YLR079W	YMR199W	999
YLR079W	YMR276W	998
YLR079W	YOL133W	999
YLR079W	YOR039W	909
YLR079W	YOR061W	909
YLR079W	YOR080W	944
YLR079W	YPL031C	986
YLR079W	YPL256C	999
YLR079W	YPR119W	999
YLR079W	YPR120C	999
YLR080W	YFL048C	999
YLR081W	YBR018C	995
YLR081W	YBR019C	994
YLR081W	YBR020W	993

YLR081W	YML051W	957
YLR082C	YIL147C	998
YLR085C	YAL011W	999
YLR085C	YBR175W	949
YLR085C	YBR231C	999
YLR085C	YDL074C	978
YLR085C	YDR190C	998
YLR085C	YDR334W	999
YLR085C	YDR392W	916
YLR085C	YDR485C	999
YLR085C	YEL018W	969
YLR085C	YER016W	941
YLR085C	YFL039C	967
YLR085C	YGL058W	959
YLR085C	YGL244W	946
YLR085C	YGR002C	999
YLR085C	YJL081C	999
YLR085C	YJL168C	989
YLR085C	YLR055C	958
YLR085C	YLR385C	978
YLR085C	YLR418C	940
YLR085C	YML041C	999
YLR085C	YNL097C	906
YLR085C	YNL107W	999
YLR085C	YNL136W	901
YLR085C	YOL012C	996
YLR085C	YOR026W	911
YLR085C	YOR123C	943
YLR085C	YOR349W	939
YLR085C	YPL055C	978
YLR085C	YPL235W	998
YLR085C	YPL241C	939
YLR086W	YBL097W	999
YLR086W	YDR325W	998
YLR086W	YFR031C	999
YLR086W	YLR272C	999
YLR087C	YPL240C	919
YLR088W	YDR302W	951
YLR088W	YDR331W	999
YLR088W	YDR434W	997
YLR088W	YHR188C	999
YLR088W	YLR459W	953
YLR089C	YAL062W	913
YLR089C	YBR218C	914

YLR089C	YDR111C	903
YLR089C	YER178W	916
YLR089C	YGL062W	903
YLR089C	YJL101C	904
YLR089C	YPR035W	924
YLR090W	YBL045C	998
YLR090W	YPL240C	919
YLR093C	YBL050W	999
YLR093C	YBR080C	951
YLR093C	YBR288C	921
YLR093C	YDL077C	925
YLR093C	YGL006W	991
YLR093C	YGL212W	999
YLR093C	YIL004C	999
YLR093C	YKL196C	999
YLR093C	YLR026C	999
YLR093C	YLR268W	999
YLR093C	YLR396C	981
YLR093C	YMR197C	999
YLR093C	YOR036W	952
YLR093C	YOR106W	999
YLR095C	YBR245C	971
YLR095C	YFR013W	913
YLR095C	YMR044W	977
YLR097C	YDL132W	979
YLR097C	YDR328C	999
YLR098C	YCL064C	943
YLR098C	YDL076C	957
YLR098C	YDL239C	924
YLR099C	YPL240C	919
YLR100W	YDL132W	983
YLR100W	YER044C	998
YLR100W	YGL001C	999
YLR100W	YGL012W	998
YLR100W	YGR060W	999
YLR100W	YHR007C	999
YLR100W	YHR072W	999
YLR100W	YHR190W	999
YLR100W	YLL012W	927
YLR100W	YLR056W	999
YLR100W	YML008C	999
YLR100W	YMR015C	998
YLR100W	YNL280C	999
YLR102C	YBL084C	999

YLR102C	YBR160W	984
YLR102C	YDL008W	999
YLR102C	YDR113C	955
YLR102C	YDR118W	999
YLR102C	YDR260C	999
YLR102C	YFR036W	999
YLR102C	YGL116W	980
YLR102C	YGL240W	999
YLR102C	YHR166C	999
YLR102C	YIR025W	998
YLR102C	YKL022C	999
YLR102C	YLR127C	999
YLR102C	YMR001C	993
YLR102C	YNL172W	999
YLR102C	YOR249C	999
YLR102C	YPR119W	926
YLR103C	YAR007C	986
YLR103C	YBL023C	999
YLR103C	YBR060C	997
YLR103C	YBR160W	929
YLR103C	YBR202W	999
YLR103C	YCL061C	904
YLR103C	YDL017W	981
YLR103C	YDR013W	942
YLR103C	YDR489W	993
YLR103C	YEL032W	999
YLR103C	YGL113W	998
YLR103C	YGL201C	999
YLR103C	YIL150C	999
YLR103C	YJL072C	935
YLR103C	YJL090C	992
YLR103C	YJL194W	995
YLR103C	YLR274W	999
YLR103C	YML065W	999
YLR103C	YNL102W	995
YLR103C	YNL261W	931
YLR103C	YNL262W	927
YLR103C	YNL273W	990
YLR103C	YNL330C	973
YLR103C	YOL004W	968
YLR103C	YOL006C	923
YLR103C	YPR019W	996
YLR103C	YPR175W	987
YLR105C	YAR008W	999

YLR105C	YIL142W	923
YLR105C	YJL087C	934
YLR105C	YMR059W	998
YLR105C	YPL083C	999
YLR106C	YCR072C	999
YLR106C	YDR101C	977
YLR106C	YER006W	998
YLR106C	YGR103W	992
YLR106C	YGR245C	999
YLR106C	YHR052W	992
YLR106C	YHR085W	998
YLR106C	YHR197W	999
YLR106C	YKL029C	972
YLR106C	YLL008W	974
YLR106C	YLR074C	955
YLR106C	YLR180W	973
YLR106C	YMR012W	975
YLR106C	YNL002C	976
YLR106C	YNL110C	973
YLR106C	YNL182C	997
YLR106C	YNR053C	989
YLR106C	YOR151C	972
YLR106C	YPL093W	987
YLR107W	YLR059C	939
YLR108C	YKL002W	944
YLR109W	YIL008W	929
YLR110C	YOR247W	916
YLR113W	YBR112C	986
YLR113W	YBR182C	998
YLR113W	YCL032W	990
YLR113W	YCR084C	980
YLR113W	YDL006W	998
YLR113W	YDL140C	976
YLR113W	YDL235C	938
YLR113W	YDR103W	936
YLR113W	YDR168W	982
YLR113W	YER075C	999
YLR113W	YER089C	965
YLR113W	YER118C	999
YLR113W	YGL158W	936
YLR113W	YGR014W	905
YLR113W	YGR218W	953
YLR113W	YIL036W	905
YLR113W	YIL147C	960

YLR113W	YJL093C	921
YLR113W	YJL128C	997
YLR113W	YJL187C	978
YLR113W	YJR132W	935
YLR113W	YKL062W	954
YLR113W	YKL190W	979
YLR113W	YLL043W	998
YLR113W	YLR079W	934
YLR113W	YLR138W	949
YLR113W	YLR248W	999
YLR113W	YLR336C	987
YLR113W	YLR362W	900
YLR113W	YMR037C	962
YLR113W	YMR172W	992
YLR113W	YNL167C	999
YLR113W	YNL330C	980
YLR113W	YOR208W	999
YLR113W	YOR212W	906
YLR113W	YPL240C	950
YLR115W	YAL043C	999
YLR115W	YDR195W	999
YLR115W	YDR228C	999
YLR115W	YDR301W	999
YLR115W	YER133W	999
YLR115W	YGR047C	928
YLR115W	YGR156W	999
YLR115W	YJL033W	961
YLR115W	YJR093C	999
YLR115W	YKL018W	999
YLR115W	YKL059C	999
YLR115W	YKR002W	999
YLR115W	YLR277C	999
YLR115W	YMR061W	999
YLR115W	YNL222W	999
YLR115W	YNL317W	999
YLR115W	YPR107C	999
YLR116W	YBR172C	945
YLR116W	YKL012W	922
YLR116W	YKL074C	999
YLR116W	YPL105C	964
YLR116W	YPL257W-B	998
YLR117C	YAL032C	999
YLR117C	YBR065C	999
YLR117C	YBR188C	999

YLR117C	YCR063W	970
YLR117C	YDL030W	976
YLR117C	YDL209C	999
YLR117C	YDR088C	932
YLR117C	YDR364C	999
YLR117C	YDR416W	999
YLR117C	YER013W	999
YLR117C	YER029C	998
YLR117C	YER172C	998
YLR117C	YGL120C	999
YLR117C	YGL128C	998
YLR117C	YGR129W	999
YLR117C	YGR278W	997
YLR117C	YHR165C	999
YLR117C	YJL203W	974
YLR117C	YJR050W	999
YLR117C	YKL012W	920
YLR117C	YKL074C	999
YLR117C	YKL095W	996
YLR117C	YKL173W	999
YLR117C	YLL036C	999
YLR117C	YLR147C	996
YLR117C	YLR424W	999
YLR117C	YML049C	999
YLR117C	YMR125W	981
YLR117C	YMR213W	999
YLR117C	YMR240C	999
YLR117C	YMR288W	999
YLR117C	YPL151C	999
YLR117C	YPL213W	999
YLR117C	YPR101W	999
YLR119W	YCL008C	999
YLR119W	YGR206W	999
YLR119W	YLR051C	999
YLR119W	YLR288C	964
YLR119W	YLR417W	932
YLR119W	YMR077C	992
YLR119W	YPL002C	932
YLR119W	YPL065W	999
YLR119W	YPL240C	919
YLR120C	YDR144C	996
YLR120C	YNL238W	998
YLR127C	YBL084C	999
YLR127C	YBR082C	926

YLR127C	YBR160W	912
YLR127C	YDL008W	999
YLR127C	YDR113C	982
YLR127C	YDR118W	999
YLR127C	YDR260C	999
YLR127C	YFR036W	999
YLR127C	YGL116W	922
YLR127C	YGL240W	999
YLR127C	YHR166C	999
YLR127C	YIR025W	999
YLR127C	YKL022C	999
YLR127C	YLR102C	999
YLR127C	YMR001C	910
YLR127C	YNL172W	999
YLR127C	YOR249C	999
YLR127C	YPR119W	980
YLR128W	YDL132W	999
YLR128W	YLR306W	926
YLR128W	YOL133W	987
YLR129W	YBL004W	997
YLR129W	YBL072C	929
YLR129W	YBR247C	992
YLR129W	YCL059C	975
YLR129W	YCR057C	999
YLR129W	YDL014W	995
YLR129W	YDL060W	914
YLR129W	YDL148C	997
YLR129W	YDL153C	919
YLR129W	YDR064W	931
YLR129W	YDR299W	940
YLR129W	YDR324C	988
YLR129W	YDR398W	926
YLR129W	YDR449C	999
YLR129W	YER082C	999
YLR129W	YGL120C	923
YLR129W	YGL171W	998
YLR129W	YGR090W	999
YLR129W	YGR128C	998
YLR129W	YGR145W	997
YLR129W	YHR148W	998
YLR129W	YHR169W	938
YLR129W	YHR196W	999
YLR129W	YJL069C	999
YLR129W	YJL109C	998

YLR129W	YJR002W	999
YLR129W	YKR060W	997
YLR129W	YLL011W	998
YLR129W	YLR003C	979
YLR129W	YLR175W	939
YLR129W	YLR186W	998
YLR129W	YLR197W	998
YLR129W	YLR222C	999
YLR129W	YLR409C	999
YLR129W	YML093W	946
YLR129W	YMR093W	994
YLR129W	YMR128W	997
YLR129W	YMR229C	965
YLR129W	YMR290C	950
YLR129W	YNL075W	958
YLR129W	YNL132W	996
YLR129W	YNR054C	933
YLR129W	YOL010W	939
YLR129W	YOR004W	947
YLR129W	YOR078W	973
YLR129W	YOR145C	998
YLR129W	YOR310C	981
YLR129W	YPL012W	998
YLR129W	YPL126W	998
YLR129W	YPL217C	998
YLR129W	YPL266W	997
YLR129W	YPR112C	998
YLR129W	YPR137W	997
YLR129W	YPR144C	999
YLR130C	YGL255W	996
YLR130C	YJL056C	981
YLR131C	YDL155W	962
YLR131C	YDR146C	965
YLR131C	YFL034C-B	967
YLR131C	YGR218W	974
YLR131C	YIL131C	939
YLR131C	YNL068C	974
YLR131C	YNL161W	998
YLR131C	YPL256C	962
YLR131C	YPR119W	992
YLR132C	YPR191W	978
YLR133W	YFL031W	922
YLR133W	YGR202C	987
YLR133W	YHR135C	973

YLR133W	YJL153C	907
YLR133W	YMR079W	999
YLR133W	YNL130C	911
YLR134W	YDR081C	937
YLR134W	YGL256W	902
YLR134W	YGR087C	969
YLR134W	YLR044C	999
YLR135W	YBR228W	999
YLR135W	YHR154W	997
YLR135W	YLR234W	961
YLR135W	YML095C	930
YLR135W	YMR190C	999
YLR135W	YPL022W	998
YLR135W	YPL024W	982
YLR138W	YDL047W	987
YLR138W	YDR040C	958
YLR138W	YDR456W	977
YLR138W	YJL094C	957
YLR138W	YKR072C	987
YLR138W	YLR113W	949
YLR139C	YDL044C	999
YLR141W	YBL014C	923
YLR141W	YBL025W	999
YLR141W	YBR010W	968
YLR141W	YMR270C	999
YLR141W	YOR295W	992
YLR142W	YHR037W	999
YLR142W	YKL015W	944
YLR144C	YDR388W	999
YLR144C	YFR024C-A	903
YLR145W	YAL033W	979
YLR145W	YBR167C	952
YLR145W	YBR257W	995
YLR145W	YDR478W	955
YLR145W	YGR030C	980
YLR145W	YHR062C	956
YLR145W	YIR015W	952
YLR145W	YNL221C	976
YLR145W	YNL282W	955
YLR146C	YKL184W	996
YLR146C	YOL052C	966
YLR146C	YPR069C	999
YLR147C	YBL026W	999
YLR147C	YBR055C	999

YLR147C	YBR119W	996
YLR147C	YBR154C	912
YLR147C	YCR077C	997
YLR147C	YDL030W	996
YLR147C	YDL043C	990
YLR147C	YDL087C	999
YLR147C	YDL098C	996
YLR147C	YDL140C	915
YLR147C	YDR235W	988
YLR147C	YDR240C	996
YLR147C	YDR378C	999
YLR147C	YDR473C	999
YLR147C	YEL026W	991
YLR147C	YER029C	999
YLR147C	YER112W	998
YLR147C	YER146W	993
YLR147C	YER172C	999
YLR147C	YFL017W-A	999
YLR147C	YGL070C	950
YLR147C	YGL173C	909
YLR147C	YGR013W	999
YLR147C	YGR074W	999
YLR147C	YGR091W	998
YLR147C	YGR271W	901
YLR147C	YHR086W	999
YLR147C	YHR165C	999
YLR147C	YIL061C	993
YLR147C	YJL124C	998
YLR147C	YJL203W	976
YLR147C	YJR022W	999
YLR147C	YKL012W	997
YLR147C	YKL173W	999
YLR147C	YLL036C	996
YLR147C	YLR117C	996
YLR147C	YLR275W	999
YLR147C	YLR298C	988
YLR147C	YLR438C-A	965
YLR147C	YML046W	989
YLR147C	YML049C	998
YLR147C	YMR125W	999
YLR147C	YMR213W	997
YLR147C	YMR240C	999
YLR147C	YMR288W	997
YLR147C	YNL147W	999

YLR147C	YOL005C	919
YLR147C	YOR151C	901
YLR147C	YOR159C	999
YLR147C	YOR210W	912
YLR147C	YOR224C	942
YLR147C	YOR308C	998
YLR147C	YPL151C	983
YLR147C	YPL178W	983
YLR147C	YPL213W	998
YLR147C	YPR082C	999
YLR147C	YPR178W	996
YLR147C	YPR182W	999
YLR147C	YPR187W	928
YLR148W	YAL002W	999
YLR148W	YDL077C	999
YLR148W	YDR080W	999
YLR148W	YDR323C	988
YLR148W	YDR495C	991
YLR148W	YGL212W	999
YLR148W	YLR396C	999
YLR148W	YMR197C	996
YLR148W	YMR231W	999
YLR148W	YOR106W	999
YLR148W	YPL045W	999
YLR150W	YBL027W	978
YLR150W	YCR031C	956
YLR150W	YDL220C	950
YLR150W	YDR025W	978
YLR150W	YDR364C	956
YLR150W	YDR418W	978
YLR150W	YDR447C	956
YLR150W	YDR471W	956
YLR150W	YER011W	940
YLR150W	YER074W	952
YLR150W	YER102W	973
YLR150W	YER131W	968
YLR150W	YFR031C-A	954
YLR150W	YGL030W	956
YLR150W	YGL178W	970
YLR150W	YGR085C	961
YLR150W	YJL177W	956
YLR150W	YJL190C	956
YLR150W	YJR145C	925
YLR150W	YLR075W	989

YLR150W	YLR448W	960
YLR150W	YML063W	954
YLR150W	YML073C	956
YLR150W	YMR125W	973
YLR150W	YMR242C	960
YLR150W	YNL069C	954
YLR150W	YNL112W	970
YLR150W	YNR052C	982
YLR150W	YOL127W	999
YLR150W	YOR063W	931
YLR150W	YOR096W	964
YLR150W	YOR312C	983
YLR150W	YPL198W	960
YLR150W	YPL249C-A	978
YLR153C	YAL054C	952
YLR153C	YBL015W	905
YLR153C	YER073W	903
YLR153C	YIR031C	907
YLR153C	YLR075W	902
YLR153C	YMR169C	901
YLR153C	YMR170C	939
YLR153C	YNL071W	912
YLR153C	YNL104C	912
YLR153C	YNL117W	932
YLR153C	YNR016C	969
YLR153C	YOR374W	954
YLR153C	YPL028W	964
YLR153C	YPL061W	984
YLR154C	YDR279W	998
YLR154C	YNL072W	997
YLR163C	YHR024C	999
YLR163C	YHR069C	977
YLR163C	YOL021C	938
YLR163C	YOL122C	976
YLR164W	YJL045W	912
YLR164W	YKL148C	912
YLR166C	YBR102C	999
YLR166C	YDR164C	998
YLR166C	YDR166C	999
YLR166C	YDR363W-A	910
YLR166C	YER008C	999
YLR166C	YFL005W	991
YLR166C	YGL233W	999
YLR166C	YGR009C	992

YLR166C	YIL068C	999
YLR166C	YJL085W	999
YLR166C	YLR229C	937
YLR166C	YNL272C	990
YLR166C	YOR326W	984
YLR166C	YPR032W	996
YLR166C	YPR055W	999
YLR167W	YBL027W	901
YLR167W	YBL072C	900
YLR167W	YBL087C	901
YLR167W	YBL092W	905
YLR167W	YBR031W	914
YLR167W	YBR048W	935
YLR167W	YBR082C	911
YLR167W	YBR084C-A	999
YLR167W	YBR118W	917
YLR167W	YBR189W	931
YLR167W	YBR191W	973
YLR167W	YCR031C	933
YLR167W	YDL061C	960
YLR167W	YDL075W	975
YLR167W	YDL081C	905
YLR167W	YDL082W	902
YLR167W	YDL122W	968
YLR167W	YDL130W	923
YLR167W	YDL136W	999
YLR167W	YDL192W	966
YLR167W	YDR012W	923
YLR167W	YDR051C	938
YLR167W	YDR064W	969
YLR167W	YDR382W	968
YLR167W	YDR418W	978
YLR167W	YDR447C	901
YLR167W	YDR450W	900
YLR167W	YDR471W	939
YLR167W	YDR500C	999
YLR167W	YEL054C	901
YLR167W	YER025W	918
YLR167W	YER056C-A	994
YLR167W	YER102W	999
YLR167W	YER117W	999
YLR167W	YER131W	915
YLR167W	YER165W	911
YLR167W	YFR031C-A	959

YLR167W	YFR032C-A	959
YLR167W	YGL030W	971
YLR167W	YGL048C	915
YLR167W	YGL076C	900
YLR167W	YGL103W	970
YLR167W	YGL123W	983
YLR167W	YGL135W	900
YLR167W	YGR027C	938
YLR167W	YGR034W	902
YLR167W	YGR085C	931
YLR167W	YGR118W	972
YLR167W	YGR148C	939
YLR167W	YGR214W	929
YLR167W	YHL001W	940
YLR167W	YHL015W	947
YLR167W	YHL033C	962
YLR167W	YHR010W	932
YLR167W	YHR021C	900
YLR167W	YHR027C	900
YLR167W	YHR141C	902
YLR167W	YHR200W	932
YLR167W	YHR203C	999
YLR167W	YIL052C	994
YLR167W	YIL133C	903
YLR167W	YIL148W	999
YLR167W	YJL136C	925
YLR167W	YJL177W	948
YLR167W	YJL189W	927
YLR167W	YJL191W	941
YLR167W	YJR007W	984
YLR167W	YJR094W-A	993
YLR167W	YJR099W	995
YLR167W	YJR123W	985
YLR167W	YJR145C	904
YLR167W	YKL006W	964
YLR167W	YKL156W	954
YLR167W	YKL180W	964
YLR167W	YKR057W	902
YLR167W	YKR059W	999
YLR167W	YKR094C	903
YLR167W	YLL039C	984
YLR167W	YLL045C	902
YLR167W	YLR029C	929
YLR167W	YLR048W	954

YLR167W	YLR061W	992
YLR167W	YLR075W	996
YLR167W	YLR185W	999
YLR167W	YLR264W	959
YLR167W	YLR340W	971
YLR167W	YLR344W	996
YLR167W	YLR367W	934
YLR167W	YLR388W	993
YLR167W	YLR406C	901
YLR167W	YLR441C	902
YLR167W	YLR448W	955
YLR167W	YML024W	994
YLR167W	YML026C	900
YLR167W	YML063W	941
YLR167W	YML073C	903
YLR167W	YML097C	976
YLR167W	YMR142C	901
YLR167W	YMR194W	977
YLR167W	YMR230W	900
YLR167W	YMR242C	903
YLR167W	YMR260C	901
YLR167W	YMR314W	910
YLR167W	YNL067W	946
YLR167W	YNL069C	902
YLR167W	YNL096C	900
YLR167W	YNL162W	902
YLR167W	YNL178W	982
YLR167W	YNL301C	999
YLR167W	YOL038W	911
YLR167W	YOL040C	904
YLR167W	YOL120C	900
YLR167W	YOL127W	974
YLR167W	YOR063W	948
YLR167W	YOR096W	948
YLR167W	YOR133W	953
YLR167W	YOR167C	910
YLR167W	YOR234C	939
YLR167W	YOR293W	925
YLR167W	YOR312C	947
YLR167W	YPL079W	920
YLR167W	YPL131W	961
YLR167W	YPL143W	954
YLR167W	YPL198W	900
YLR167W	YPL220W	999

YLR167W	YPL249C-A	947
YLR167W	YPR043W	993
YLR167W	YPR102C	938
YLR167W	YPR103W	918
YLR167W	YPR132W	999
YLR168C	YJR070C	998
YLR170C	YGL206C	996
YLR170C	YHL019C	999
YLR170C	YKL135C	999
YLR170C	YPL259C	999
YLR170C	YPR029C	999
YLR172C	YDR385W	967
YLR173W	YIL016W	998
YLR174W	YDL066W	904
YLR174W	YJL200C	904
YLR174W	YLR304C	934
YLR174W	YNL009W	903
YLR174W	YNL037C	985
YLR174W	YOR136W	984
YLR175W	YAL035W	915
YLR175W	YBR247C	995
YLR175W	YCL031C	909
YLR175W	YCL037C	980
YLR175W	YCL059C	999
YLR175W	YCR057C	975
YLR175W	YDL014W	999
YLR175W	YDL153C	909
YLR175W	YDL208W	999
YLR175W	YDR299W	922
YLR175W	YDR324C	915
YLR175W	YDR398W	919
YLR175W	YDR496C	997
YLR175W	YEL026W	980
YLR175W	YGL049C	992
YLR175W	YGL078C	975
YLR175W	YGL120C	978
YLR175W	YGL171W	916
YLR175W	YGL173C	990
YLR175W	YGL244W	923
YLR175W	YGR128C	929
YLR175W	YGR159C	940
YLR175W	YGR283C	946
YLR175W	YHR072W-A	999
YLR175W	YHR089C	999

YLR175W	YHR148W	901
YLR175W	YHR169W	917
YLR175W	YHR196W	996
YLR175W	YHR203C	991
YLR175W	YJL010C	983
YLR175W	YJL069C	911
YLR175W	YJL109C	930
YLR175W	YJL190C	992
YLR175W	YJR002W	921
YLR175W	YJR145C	992
YLR175W	YKL009W	949
YLR175W	YKL099C	911
YLR175W	YKL125W	986
YLR175W	YLL011W	920
YLR175W	YLR003C	911
YLR175W	YLR129W	939
YLR175W	YLR186W	965
YLR175W	YLR197W	999
YLR175W	YLR222C	954
YLR175W	YLR409C	921
YLR175W	YLR410W	925
YLR175W	YML093W	919
YLR175W	YMR093W	920
YLR175W	YMR229C	999
YLR175W	YMR290C	999
YLR175W	YMR310C	990
YLR175W	YNL075W	934
YLR175W	YNL124W	999
YLR175W	YNL307C	993
YLR175W	YNL308C	991
YLR175W	YOL010W	908
YLR175W	YOL077C	938
YLR175W	YOR004W	953
YLR175W	YOR063W	990
YLR175W	YOR145C	950
YLR175W	YOR309C	998
YLR175W	YOR310C	995
YLR175W	YPL012W	999
YLR175W	YPL126W	911
YLR175W	YPL146C	910
YLR175W	YPL217C	914
YLR175W	YPL266W	910
YLR175W	YPR137W	908
YLR176C	YBR009C	911

YLR176C	YBR049C	978
YLR176C	YBR112C	992
YLR176C	YBR245C	984
YLR176C	YCR084C	992
YLR176C	YDR303C	987
YLR176C	YGL133W	963
YLR176C	YIL126W	932
YLR176C	YKR001C	907
YLR176C	YLR357W	979
YLR176C	YPL082C	982
YLR177W	YDR099W	986
YLR177W	YER177W	940
YLR178C	YLR310C	993
YLR178C	YMR297W	999
YLR178C	YOL081W	986
YLR178C	YOR002W	960
YLR180W	YBR236C	983
YLR180W	YDR127W	973
YLR180W	YDR502C	999
YLR180W	YER091C	995
YLR180W	YFR009W	973
YLR180W	YGL195W	976
YLR180W	YGR218W	969
YLR180W	YGR240C	975
YLR180W	YGR264C	907
YLR180W	YHR020W	975
YLR180W	YIL075C	988
YLR180W	YKL104C	975
YLR180W	YLR075W	987
YLR180W	YLR106C	973
YLR180W	YMR012W	974
YLR180W	YOR151C	975
YLR180W	YPL240C	919
YLR181C	YDR486C	999
YLR181C	YKR035W-A	994
YLR181C	YLR025W	997
YLR181C	YNL265C	914
YLR181C	YPR173C	999
YLR182W	YAL040C	996
YLR182W	YBR160W	999
YLR182W	YBR279W	960
YLR182W	YCR081W	925
YLR182W	YDL056W	999
YLR182W	YDR145W	923

YLR182W	YDR308C	924
YLR182W	YDR335W	972
YLR182W	YER111C	999
YLR182W	YER148W	954
YLR182W	YER167W	934
YLR182W	YFR028C	983
YLR182W	YGL207W	958
YLR182W	YGR044C	982
YLR182W	YGR104C	906
YLR182W	YHR030C	999
YLR182W	YHR041C	906
YLR182W	YLR033W	916
YLR182W	YML114C	923
YLR182W	YMR199W	965
YLR182W	YMR227C	923
YLR182W	YMR236W	915
YLR182W	YNL236W	997
YLR182W	YNL261W	999
YLR182W	YNL309W	990
YLR182W	YOL004W	972
YLR182W	YOL116W	974
YLR182W	YOR083W	998
YLR182W	YPL153C	997
YLR182W	YPL256C	999
YLR182W	YPR052C	972
YLR182W	YPR119W	957
YLR182W	YPR120C	965
YLR183C	YDR243C	981
YLR183C	YHR195W	935
YLR185W	YAL035W	914
YLR185W	YBL027W	989
YLR185W	YBL072C	911
YLR185W	YBL087C	990
YLR185W	YBL092W	990
YLR185W	YBR031W	999
YLR185W	YBR048W	967
YLR185W	YBR084C-A	999
YLR185W	YBR118W	915
YLR185W	YBR189W	949
YLR185W	YBR191W	997
YLR185W	YCR031C	955
YLR185W	YDL061C	961
YLR185W	YDL075W	999
YLR185W	YDL081C	963

YLR185W	YDL082W	908
YLR185W	YDL130W	937
YLR185W	YDL136W	999
YLR185W	YDL191W	919
YLR185W	YDR012W	993
YLR185W	YDR025W	900
YLR185W	YDR064W	985
YLR185W	YDR382W	976
YLR185W	YDR418W	996
YLR185W	YDR447C	917
YLR185W	YDR450W	901
YLR185W	YDR471W	946
YLR185W	YDR500C	999
YLR185W	YEL054C	921
YLR185W	YER056C-A	987
YLR185W	YER074W	918
YLR185W	YER102W	999
YLR185W	YER117W	999
YLR185W	YER131W	930
YLR185W	YFL034C-A	911
YLR185W	YFR031C-A	989
YLR185W	YFR032C-A	927
YLR185W	YGL030W	980
YLR185W	YGL031C	989
YLR185W	YGL076C	991
YLR185W	YGL103W	996
YLR185W	YGL123W	973
YLR185W	YGL135W	990
YLR185W	YGL147C	989
YLR185W	YGL189C	918
YLR185W	YGR027C	932
YLR185W	YGR034W	913
YLR185W	YGR085C	921
YLR185W	YGR118W	970
YLR185W	YGR148C	927
YLR185W	YGR214W	937
YLR185W	YHL001W	953
YLR185W	YHL015W	944
YLR185W	YHL033C	995
YLR185W	YHR010W	935
YLR185W	YHR021C	916
YLR185W	YHR026W	944
YLR185W	YHR141C	991
YLR185W	YHR203C	999

YLR185W	YIL052C	978
YLR185W	YIL069C	918
YLR185W	YIL132C	901
YLR185W	YIL133C	990
YLR185W	YIL148W	999
YLR185W	YJL136C	936
YLR185W	YJL177W	969
YLR185W	YJL189W	926
YLR185W	YJL190C	910
YLR185W	YJL191W	957
YLR185W	YJR094W-A	998
YLR185W	YJR123W	959
YLR185W	YJR145C	912
YLR185W	YKL006W	971
YLR185W	YKL156W	960
YLR185W	YKL180W	997
YLR185W	YKR057W	922
YLR185W	YKR059W	954
YLR185W	YKR094C	900
YLR185W	YLL045C	912
YLR185W	YLR029C	993
YLR185W	YLR048W	971
YLR185W	YLR060W	923
YLR185W	YLR061W	997
YLR185W	YLR075W	999
YLR185W	YLR167W	999
YLR185W	YLR264W	949
YLR185W	YLR287C-A	906
YLR185W	YLR325C	917
YLR185W	YLR333C	910
YLR185W	YLR340W	968
YLR185W	YLR344W	998
YLR185W	YLR367W	931
YLR185W	YLR388W	994
YLR185W	YLR406C	910
YLR185W	YLR441C	911
YLR185W	YLR448W	971
YLR185W	YML024W	987
YLR185W	YML026C	901
YLR185W	YML063W	939
YLR185W	YML073C	912
YLR185W	YMR121C	913
YLR185W	YMR142C	906
YLR185W	YMR194W	993

YLR185W	YMR230W	920
YLR185W	YMR242C	909
YLR185W	YNL067W	926
YLR185W	YNL069C	913
YLR185W	YNL096C	920
YLR185W	YNL162W	917
YLR185W	YNL178W	977
YLR185W	YNL301C	999
YLR185W	YNL302C	912
YLR185W	YOL040C	924
YLR185W	YOL121C	912
YLR185W	YOL127W	996
YLR185W	YOR063W	994
YLR185W	YOR096W	958
YLR185W	YOR133W	965
YLR185W	YOR167C	903
YLR185W	YOR182C	906
YLR185W	YOR234C	952
YLR185W	YOR293W	933
YLR185W	YOR312C	926
YLR185W	YOR369C	930
YLR185W	YPL037C	926
YLR185W	YPL079W	928
YLR185W	YPL081W	927
YLR185W	YPL131W	997
YLR185W	YPL143W	955
YLR185W	YPL198W	917
YLR185W	YPL220W	999
YLR185W	YPL249C-A	979
YLR185W	YPR043W	983
YLR185W	YPR102C	991
YLR185W	YPR132W	999
YLR186W	YBL004W	998
YLR186W	YBL072C	931
YLR186W	YBR189W	958
YLR186W	YBR247C	999
YLR186W	YCL059C	999
YLR186W	YCR057C	999
YLR186W	YDL014W	995
YLR186W	YDL060W	903
YLR186W	YDL148C	999
YLR186W	YDL213C	937
YLR186W	YDR064W	943
YLR186W	YDR299W	998

YLR186W	YDR324C	997
YLR186W	YDR339C	902
YLR186W	YDR398W	914
YLR186W	YDR449C	999
YLR186W	YDR502C	918
YLR186W	YEL026W	935
YLR186W	YER082C	997
YLR186W	YGL120C	918
YLR186W	YGL171W	997
YLR186W	YGR090W	999
YLR186W	YGR128C	997
YLR186W	YGR145W	997
YLR186W	YHR148W	998
YLR186W	YHR169W	922
YLR186W	YHR196W	999
YLR186W	YJL069C	956
YLR186W	YJL109C	999
YLR186W	YJR002W	999
YLR186W	YJR145C	929
YLR186W	YKR060W	997
YLR186W	YLL011W	997
YLR186W	YLR129W	998
YLR186W	YLR175W	965
YLR186W	YLR197W	997
YLR186W	YLR222C	998
YLR186W	YLR409C	998
YLR186W	YMR093W	998
YLR186W	YMR128W	997
YLR186W	YMR229C	921
YLR186W	YMR290C	948
YLR186W	YNL075W	948
YLR186W	YNL132W	994
YLR186W	YOL010W	910
YLR186W	YOR004W	923
YLR186W	YOR096W	946
YLR186W	YOR145C	998
YLR186W	YOR310C	998
YLR186W	YPL012W	995
YLR186W	YPL126W	997
YLR186W	YPL217C	998
YLR186W	YPR137W	997
YLR186W	YPR144C	999
YLR188W	YMR301C	942
YLR189C	YGR220C	913

YLR190W	YAL048C	928
YLR190W	YER059W	978
YLR190W	YNL304W	951
YLR190W	YOR326W	916
YLR191W	YDL065C	999
YLR191W	YDR142C	999
YLR191W	YDR244W	999
YLR191W	YDR265W	998
YLR191W	YDR329C	944
YLR191W	YGL153W	999
YLR191W	YGR077C	993
YLR191W	YGR239C	979
YLR191W	YHR160C	980
YLR191W	YJL210W	995
YLR191W	YMR026C	999
YLR191W	YNL214W	999
YLR191W	YNL329C	905
YLR191W	YOL044W	980
YLR192C	YAL035W	970
YLR192C	YBL072C	917
YLR192C	YBR079C	999
YLR192C	YDR091C	999
YLR192C	YDR429C	999
YLR192C	YDR447C	914
YLR192C	YDR450W	918
YLR192C	YER025W	905
YLR192C	YER102W	997
YLR192C	YGR214W	915
YLR192C	YJR007W	924
YLR192C	YMR146C	999
YLR192C	YMR309C	999
YLR192C	YNL178W	915
YLR192C	YNL244C	999
YLR192C	YOR361C	999
YLR192C	YPR041W	999
YLR195C	YER015W	932
YLR195C	YKL182W	976
YLR195C	YOR317W	950
YLR196W	YEL055C	926
YLR196W	YHR170W	978
YLR196W	YKL172W	995
YLR196W	YKR056W	901
YLR196W	YKR081C	966
YLR196W	YLL045C	974

YLR196W	YLR009W	966
YLR196W	YLR029C	972
YLR196W	YLR410W	940
YLR196W	YLR449W	933
YLR196W	YOL041C	999
YLR196W	YOL077C	999
YLR196W	YPL131W	976
YLR196W	YPL207W	946
YLR197W	YBL004W	997
YLR197W	YBL072C	933
YLR197W	YBR189W	941
YLR197W	YBR247C	974
YLR197W	YCL059C	999
YLR197W	YCR057C	999
YLR197W	YDL014W	999
YLR197W	YDL060W	906
YLR197W	YDL148C	997
YLR197W	YDL153C	916
YLR197W	YDR064W	948
YLR197W	YDR299W	931
YLR197W	YDR324C	997
YLR197W	YDR398W	915
YLR197W	YDR449C	997
YLR197W	YDR496C	950
YLR197W	YEL026W	999
YLR197W	YER082C	999
YLR197W	YGL078C	980
YLR197W	YGL120C	999
YLR197W	YGL171W	923
YLR197W	YGR090W	997
YLR197W	YGR128C	998
YLR197W	YGR159C	950
YLR197W	YHR148W	997
YLR197W	YHR169W	911
YLR197W	YHR196W	999
YLR197W	YHR203C	929
YLR197W	YIL035C	906
YLR197W	YIL133C	939
YLR197W	YJL010C	992
YLR197W	YJL033W	949
YLR197W	YJL069C	928
YLR197W	YJL109C	999
YLR197W	YJL190C	931
YLR197W	YJR002W	999

YLR197W	YJR123W	943
YLR197W	YJR145C	931
YLR197W	YKR060W	997
YLR197W	YLL011W	998
YLR197W	YLR003C	921
YLR197W	YLR129W	998
YLR197W	YLR175W	999
YLR197W	YLR186W	997
YLR197W	YLR222C	998
YLR197W	YLR409C	998
YLR197W	YLR448W	939
YLR197W	YML093W	911
YLR197W	YMR093W	997
YLR197W	YMR128W	997
YLR197W	YMR229C	998
YLR197W	YMR290C	997
YLR197W	YNL075W	997
YLR197W	YNL132W	995
YLR197W	YOL010W	915
YLR197W	YOR004W	926
YLR197W	YOR078W	999
YLR197W	YOR096W	942
YLR197W	YOR145C	916
YLR197W	YOR310C	999
YLR197W	YPL012W	995
YLR197W	YPL043W	945
YLR197W	YPL126W	997
YLR197W	YPL217C	998
YLR197W	YPL266W	907
YLR197W	YPR112C	909
YLR197W	YPR137W	999
YLR197W	YPR144C	997
YLR199C	YBR173C	964
YLR199C	YKL206C	998
YLR199C	YPR103W	921
YLR200W	YBL034C	975
YLR200W	YEL003W	999
YLR200W	YFL023W	999
YLR200W	YFL037W	982
YLR200W	YFL039C	967
YLR200W	YGR078C	999
YLR200W	YIL142W	982
YLR200W	YJL014W	982
YLR200W	YJL179W	999

YLR200W	YML085C	982
YLR200W	YML094W	999
YLR200W	YML124C	950
YLR200W	YNL153C	999
YLR200W	YOR265W	915
YLR201C	YDR204W	942
YLR201C	YGL119W	992
YLR201C	YGR255C	951
YLR201C	YML110C	941
YLR201C	YOR125C	952
YLR203C	Q0045	999
YLR203C	YGR112W	999
YLR203C	YIL157C	997
YLR203C	YJR045C	993
YLR203C	YML129C	990
YLR203C	YMR257C	908
YLR203C	YOR232W	907
YLR203C	YPL172C	913
YLR206W	YGL206C	974
YLR207W	YBL061C	999
YLR207W	YBR170C	970
YLR207W	YBR201W	999
YLR207W	YDL126C	976
YLR207W	YDR057W	999
YLR207W	YJL034W	995
YLR207W	YLR378C	952
YLR207W	YML013W	960
YLR207W	YML029W	990
YLR207W	YMR022W	987
YLR207W	YMR264W	954
YLR207W	YMR297W	972
YLR207W	YOL013C	999
YLR208W	YBL079W	920
YLR208W	YDL116W	999
YLR208W	YDL195W	999
YLR208W	YDL212W	986
YLR208W	YDR128W	900
YLR208W	YDR159W	966
YLR208W	YDR299W	904
YLR208W	YER105C	959
YLR208W	YER107C	907
YLR208W	YGL092W	999
YLR208W	YGL100W	999
YLR208W	YGL200C	987

YLR208W	YGR057C	991
YLR208W	YGR172C	974
YLR208W	YHR079C	990
YLR208W	YHR098C	998
YLR208W	YIL109C	996
YLR208W	YIL115C	945
YLR208W	YJL002C	986
YLR208W	YJL041W	953
YLR208W	YJR042W	999
YLR208W	YKL057C	999
YLR208W	YKL176C	989
YLR208W	YKL186C	952
YLR208W	YKR082W	995
YLR208W	YML012W	948
YLR208W	YML130C	973
YLR208W	YMR047C	975
YLR208W	YMR129W	928
YLR208W	YNL006W	972
YLR208W	YNL049C	975
YLR208W	YOR036W	938
YLR208W	YPL085W	999
YLR208W	YPL169C	952
YLR208W	YPL218W	974
YLR208W	YPR181C	999
YLR209C	YDL042C	916
YLR209C	YDL238C	963
YLR209C	YDR400W	994
YLR209C	YDR441C	913
YLR209C	YJR105W	942
YLR209C	YLR245C	979
YLR209C	YML022W	921
YLR209C	YNL129W	992
YLR209C	YNL141W	960
YLR209C	YPR062W	928
YLR210W	YBR135W	907
YLR210W	YBR160W	999
YLR210W	YDL108W	901
YLR210W	YDL155W	960
YLR210W	YGL003C	968
YLR210W	YGR108W	916
YLR210W	YHR129C	910
YLR210W	YJL187C	952
YLR210W	YLR079W	999
YLR210W	YMR036C	951

YLR210W	YPL269W	986
YLR210W	YPR025C	919
YLR210W	YPR119W	967
YLR212C	YAL047C	999
YLR212C	YBR109C	917
YLR212C	YDR356W	999
YLR212C	YEL003W	901
YLR212C	YGR240C	917
YLR212C	YHR172W	999
YLR212C	YMR055C	923
YLR212C	YNL064C	971
YLR212C	YNL126W	999
YLR212C	YNL153C	910
YLR214W	YER145C	937
YLR214W	YGL071W	994
YLR214W	YKL220C	983
YLR214W	YMR021C	998
YLR214W	YMR058W	989
YLR214W	YMR319C	913
YLR214W	YOL152W	906
YLR215C	YER025W	999
YLR215C	YHR115C	984
YLR215C	YNL116W	971
YLR216C	YBR155W	950
YLR216C	YDR214W	971
YLR216C	YGR178C	913
YLR216C	YGR211W	913
YLR216C	YKL117W	999
YLR216C	YMR186W	999
YLR216C	YNL330C	976
YLR216C	YNR032W	917
YLR216C	YOR027W	989
YLR216C	YPL240C	999
YLR220W	YGL167C	986
YLR220W	YIR018W	926
YLR220W	YJL133W	912
YLR220W	YKR052C	911
YLR220W	YMR319C	908
YLR220W	YPL057C	919
YLR221C	YDR060W	914
YLR221C	YJR041C	983
YLR221C	YKL014C	986
YLR221C	YNR038W	997
YLR221C	YOL144W	998

YLR222C	YBL004W	997
YLR222C	YBR247C	975
YLR222C	YCL059C	979
YLR222C	YCR057C	999
YLR222C	YDL014W	994
YLR222C	YDL060W	914
YLR222C	YDL148C	991
YLR222C	YDL153C	933
YLR222C	YDR299W	948
YLR222C	YDR324C	997
YLR222C	YDR398W	923
YLR222C	YDR449C	999
YLR222C	YEL026W	911
YLR222C	YER082C	999
YLR222C	YGL120C	941
YLR222C	YGL171W	951
YLR222C	YGR090W	997
YLR222C	YGR128C	999
YLR222C	YGR145W	915
YLR222C	YHR148W	998
YLR222C	YHR169W	964
YLR222C	YHR170W	909
YLR222C	YHR196W	919
YLR222C	YJL010C	985
YLR222C	YJL069C	999
YLR222C	YJL109C	999
YLR222C	YJR002W	999
YLR222C	YKR060W	918
YLR222C	YLL011W	993
YLR222C	YLR003C	933
YLR222C	YLR129W	999
YLR222C	YLR175W	954
YLR222C	YLR186W	998
YLR222C	YLR197W	998
YLR222C	YLR409C	999
YLR222C	YML093W	938
YLR222C	YMR093W	993
YLR222C	YMR128W	997
YLR222C	YMR229C	949
YLR222C	YMR290C	949
YLR222C	YNL075W	999
YLR222C	YNL132W	998
YLR222C	YOL010W	919
YLR222C	YOR004W	963

YLR222C	YOR078W	992
YLR222C	YOR145C	954
YLR222C	YOR310C	985
YLR222C	YPL012W	924
YLR222C	YPL126W	998
YLR222C	YPL217C	998
YLR222C	YPR112C	923
YLR222C	YPR137W	998
YLR222C	YPR144C	999
YLR223C	YCL031C	995
YLR223C	YGR090W	999
YLR223C	YNL216W	998
YLR223C	YOR039W	978
YLR223C	YPR104C	999
YLR224W	YDR328C	996
YLR226W	YAL021C	905
YLR226W	YBR279W	977
YLR226W	YER068W	984
YLR226W	YJL168C	919
YLR226W	YKL139W	931
YLR226W	YMR125W	930
YLR226W	YPR161C	999
YLR227C	YOL091W	937
YLR228C	YER044C	908
YLR228C	YLR372W	905
YLR228C	YMR070W	980
YLR228C	YMR202W	995
YLR229C	YAL024C	990
YLR229C	YAL041W	999
YLR229C	YAL048C	911
YLR229C	YBL085W	912
YLR229C	YBR200W	999
YLR229C	YCL032W	993
YLR229C	YDL135C	999
YLR229C	YDR122W	968
YLR229C	YDR166C	941
YLR229C	YDR309C	999
YLR229C	YDR379W	971
YLR229C	YDR507C	997
YLR229C	YER008C	998
YLR229C	YER114C	901
YLR229C	YER118C	973
YLR229C	YER136W	910
YLR229C	YER155C	989

YLR229C	YFL005W	941
YLR229C	YFL039C	937
YLR229C	YGL155W	940
YLR229C	YGL233W	991
YLR229C	YGR009C	937
YLR229C	YGR014W	918
YLR229C	YGR070W	964
YLR229C	YGR152C	979
YLR229C	YHL007C	999
YLR229C	YHR061C	999
YLR229C	YHR107C	987
YLR229C	YHR206W	971
YLR229C	YIL118W	996
YLR229C	YIL140W	992
YLR229C	YJL157C	999
YLR229C	YJL187C	972
YLR229C	YKR090W	906
YLR229C	YLL021W	939
YLR229C	YLR166C	937
YLR229C	YLR310C	924
YLR229C	YLR319C	904
YLR229C	YLR337C	958
YLR229C	YLR371W	962
YLR229C	YMR273C	971
YLR229C	YNL098C	960
YLR229C	YNL271C	999
YLR229C	YNL293W	999
YLR229C	YNL298W	999
YLR229C	YOL112W	981
YLR229C	YOL113W	999
YLR229C	YOR127W	999
YLR229C	YOR181W	904
YLR229C	YOR188W	999
YLR229C	YPL115C	999
YLR229C	YPL161C	999
YLR229C	YPL242C	984
YLR229C	YPL256C	909
YLR229C	YPR055W	936
YLR229C	YPR165W	906
YLR231C	YBL098W	989
YLR231C	YJR025C	985
YLR233C	YDL220C	999
YLR233C	YJL019W	981
YLR233C	YLR318W	999

YLR233C	YML032C	976
YLR233C	YMR106C	980
YLR233C	YMR284W	999
YLR234W	YBR098W	997
YLR234W	YBR228W	950
YLR234W	YDL013W	926
YLR234W	YDR076W	978
YLR234W	YDR078C	913
YLR234W	YDR174W	971
YLR234W	YDR386W	996
YLR234W	YER095W	990
YLR234W	YER116C	930
YLR234W	YGL163C	995
YLR234W	YHL006C	982
YLR234W	YHR031C	911
YLR234W	YJL092W	999
YLR234W	YJR043C	938
YLR234W	YLR135W	961
YLR234W	YLR376C	910
YLR234W	YMR190C	999
YLR234W	YMR233W	999
YLR234W	YNL088W	915
YLR234W	YNL218W	928
YLR234W	YOL006C	978
YLR234W	YOR144C	916
YLR234W	YPL022W	983
YLR234W	YPL024W	999
YLR238W	YDR200C	902
YLR238W	YMR052W	915
YLR239C	YJL046W	918
YLR239C	YOR196C	988
YLR240W	YBR097W	999
YLR240W	YBR128C	999
YLR240W	YDR323C	947
YLR240W	YFR019W	943
YLR240W	YHR005C	935
YLR240W	YJR110W	944
YLR240W	YKL212W	988
YLR240W	YLR305C	912
YLR240W	YLR360W	999
YLR240W	YNL106C	919
YLR240W	YNL267W	921
YLR240W	YOR109W	921
YLR240W	YPL120W	999

YLR240W	YPR113W	915
YLR242C	YCR048W	919
YLR242C	YJL062W	923
YLR243W	YJR072C	950
YLR244C	YBL091C	982
YLR245C	YDR400W	934
YLR245C	YHR128W	930
YLR245C	YLR209C	979
YLR245C	YLR291C	958
YLR245C	YNR012W	942
YLR245C	YPL070W	937
YLR246W	YHR175W	923
YLR246W	YKR067W	923
YLR246W	YNL098C	918
YLR246W	YOL110W	953
YLR248W	YDL047W	940
YLR248W	YJL128C	996
YLR248W	YLR113W	999
YLR249W	YAL003W	998
YLR249W	YBR118W	992
YLR249W	YBR191W	944
YLR249W	YBR196C	968
YLR249W	YDL075W	923
YLR249W	YDR064W	954
YLR249W	YDR381W	969
YLR249W	YDR382W	940
YLR249W	YDR385W	972
YLR249W	YER049W	972
YLR249W	YGL123W	948
YLR249W	YGR162W	958
YLR249W	YHL033C	938
YLR249W	YHR064C	987
YLR249W	YJR123W	962
YLR249W	YKL006W	938
YLR249W	YKL081W	996
YLR249W	YKL180W	940
YLR249W	YLL026W	968
YLR249W	YLR044C	975
YLR249W	YLR048W	998
YLR249W	YLR060W	966
YLR249W	YLR061W	942
YLR249W	YLR075W	940
YLR249W	YLR448W	940
YLR249W	YMR186W	986

YLR249W	YNL178W	940
YLR249W	YNL209W	942
YLR249W	YOL086C	970
YLR249W	YOR133W	986
YLR249W	YPL048W	968
YLR249W	YPL131W	998
YLR249W	YPR080W	976
YLR250W	YFL048C	998
YLR254C	YGL216W	955
YLR254C	YOR269W	999
YLR254C	YPL269W	955
YLR256W	YCL037C	976
YLR256W	YGL237C	940
YLR256W	YNL064C	976
YLR256W	YPL240C	999
YLR258W	YBR126C	931
YLR258W	YDR074W	948
YLR258W	YDR477W	961
YLR258W	YEL011W	997
YLR258W	YER054C	976
YLR258W	YER133W	998
YLR258W	YFR015C	999
YLR258W	YGL134W	996
YLR258W	YHL012W	901
YLR258W	YIL045W	983
YLR258W	YJL137C	999
YLR258W	YKL035W	948
YLR258W	YKR058W	997
YLR258W	YLR273C	966
YLR258W	YML100W	915
YLR258W	YOR178C	984
YLR258W	YPL031C	999
YLR258W	YPR160W	961
YLR258W	YPR184W	962
YLR259C	YER086W	982
YLR259C	YMR186W	909
YLR259C	YNL055C	999
YLR259C	YNL085W	999
YLR259C	YOR020C	999
YLR259C	YPL240C	901
YLR260W	YDR294C	966
YLR260W	YDR297W	943
YLR260W	YJL134W	970
YLR260W	YKR053C	950

YLR260W	YOR171C	984
YLR262C	YBR164C	996
YLR262C	YDR027C	978
YLR262C	YDR100W	921
YLR262C	YDR137W	975
YLR262C	YDR189W	999
YLR262C	YDR293C	980
YLR262C	YDR468C	982
YLR262C	YDR484W	995
YLR262C	YER136W	997
YLR262C	YGL198W	910
YLR262C	YGR100W	979
YLR262C	YGR120C	964
YLR262C	YGR172C	993
YLR262C	YHL031C	944
YLR262C	YJL004C	999
YLR262C	YJL029C	980
YLR262C	YJL044C	998
YLR262C	YJL154C	952
YLR262C	YJR134C	999
YLR262C	YKR020W	913
YLR262C	YLR039C	998
YLR262C	YLR309C	999
YLR262C	YMR186W	963
YLR262C	YNL293W	984
YLR262C	YOL012C	983
YLR262C	YOL112W	903
YLR262C	YOR070C	966
YLR262C	YOR370C	912
YLR262C	YPL051W	981
YLR262C	YPL240C	919
YLR263W	YDR285W	999
YLR263W	YER133W	997
YLR263W	YER179W	955
YLR263W	YGL033W	968
YLR263W	YGL175C	939
YLR263W	YGL183C	939
YLR263W	YGL249W	930
YLR263W	YIL072W	999
YLR263W	YOR351C	999
YLR264W	YAL035W	954
YLR264W	YBL027W	909
YLR264W	YBL072C	909
YLR264W	YBL087C	904

YLR264W	YBL092W	938
YLR264W	YBR031W	955
YLR264W	YBR048W	919
YLR264W	YBR079C	917
YLR264W	YBR084C-A	957
YLR264W	YBR118W	902
YLR264W	YBR181C	921
YLR264W	YBR189W	999
YLR264W	YBR191W	999
YLR264W	YCR031C	965
YLR264W	YDL061C	995
YLR264W	YDL075W	980
YLR264W	YDL081C	926
YLR264W	YDL082W	921
YLR264W	YDL130W	925
YLR264W	YDL136W	963
YLR264W	YDL191W	905
YLR264W	YDR012W	972
YLR264W	YDR064W	999
YLR264W	YDR382W	994
YLR264W	YDR385W	905
YLR264W	YDR418W	978
YLR264W	YDR447C	915
YLR264W	YDR450W	912
YLR264W	YDR471W	972
YLR264W	YDR500C	963
YLR264W	YEL054C	910
YLR264W	YER009W	929
YLR264W	YER025W	974
YLR264W	YER056C-A	993
YLR264W	YER074W	900
YLR264W	YER102W	958
YLR264W	YER112W	966
YLR264W	YER117W	966
YLR264W	YER131W	973
YLR264W	YER165W	918
YLR264W	YFL034C-A	917
YLR264W	YFR031C-A	917
YLR264W	YFR032C-A	922
YLR264W	YGL030W	995
YLR264W	YGL031C	952
YLR264W	YGL076C	954
YLR264W	YGL103W	997
YLR264W	YGL123W	995

YLR264W	YGL135W	914
YLR264W	YGL147C	923
YLR264W	YGL189C	902
YLR264W	YGR027C	994
YLR264W	YGR034W	916
YLR264W	YGR085C	979
YLR264W	YGR118W	995
YLR264W	YGR148C	972
YLR264W	YGR162W	912
YLR264W	YGR214W	982
YLR264W	YHL001W	997
YLR264W	YHL015W	995
YLR264W	YHL033C	994
YLR264W	YHR010W	978
YLR264W	YHR021C	913
YLR264W	YHR141C	932
YLR264W	YHR203C	958
YLR264W	YIL018W	917
YLR264W	YIL052C	997
YLR264W	YIL069C	900
YLR264W	YIL133C	918
YLR264W	YIL148W	963
YLR264W	YJL136C	961
YLR264W	YJL177W	984
YLR264W	YJL189W	943
YLR264W	YJL190C	918
YLR264W	YJL191W	977
YLR264W	YJR022W	952
YLR264W	YJR094W-A	993
YLR264W	YJR123W	995
YLR264W	YJR145C	916
YLR264W	YKL006W	999
YLR264W	YKL156W	998
YLR264W	YKL180W	998
YLR264W	YKR057W	916
YLR264W	YKR059W	947
YLR264W	YKR094C	903
YLR264W	YLL045C	933
YLR264W	YLR029C	963
YLR264W	YLR048W	998
YLR264W	YLR061W	997
YLR264W	YLR075W	974
YLR264W	YLR167W	959
YLR264W	YLR185W	949

YLR264W	YLR287C-A	902
YLR264W	YLR333C	925
YLR264W	YLR340W	976
YLR264W	YLR344W	978
YLR264W	YLR367W	996
YLR264W	YLR388W	999
YLR264W	YLR406C	914
YLR264W	YLR441C	909
YLR264W	YLR448W	996
YLR264W	YML024W	997
YLR264W	YML026C	912
YLR264W	YML063W	994
YLR264W	YML073C	913
YLR264W	YML106W	960
YLR264W	YMR121C	903
YLR264W	YMR142C	924
YLR264W	YMR194W	985
YLR264W	YMR230W	959
YLR264W	YMR242C	912
YLR264W	YMR260C	900
YLR264W	YNL067W	995
YLR264W	YNL069C	917
YLR264W	YNL096C	997
YLR264W	YNL162W	918
YLR264W	YNL178W	992
YLR264W	YNL301C	964
YLR264W	YNL302C	910
YLR264W	YOL039W	928
YLR264W	YOL040C	925
YLR264W	YOL120C	908
YLR264W	YOL121C	911
YLR264W	YOL127W	994
YLR264W	YOL149W	939
YLR264W	YOR063W	977
YLR264W	YOR096W	994
YLR264W	YOR133W	921
YLR264W	YOR167C	986
YLR264W	YOR182C	902
YLR264W	YOR234C	996
YLR264W	YOR293W	994
YLR264W	YOR312C	929
YLR264W	YOR361C	933
YLR264W	YOR369C	905
YLR264W	YPL037C	904

YLR264W	YPL079W	966
YLR264W	YPL081W	948
YLR264W	YPL090C	921
YLR264W	YPL131W	962
YLR264W	YPL143W	994
YLR264W	YPL198W	912
YLR264W	YPL220W	960
YLR264W	YPL237W	921
YLR264W	YPL249C-A	938
YLR264W	YPR043W	941
YLR264W	YPR080W	902
YLR264W	YPR102C	994
YLR264W	YPR132W	961
YLR264W	YPR163C	909
YLR265C	YGL090W	999
YLR265C	YLR424W	910
YLR265C	YML032C	907
YLR265C	YOR005C	992
YLR268W	YBL050W	999
YLR268W	YBR080C	981
YLR268W	YDL058W	992
YLR268W	YDL100C	953
YLR268W	YDL192W	993
YLR268W	YDR189W	999
YLR268W	YDR238C	904
YLR268W	YDR498C	999
YLR268W	YEL036C	933
YLR268W	YER122C	952
YLR268W	YER157W	972
YLR268W	YFL038C	999
YLR268W	YGL098W	999
YLR268W	YGL137W	988
YLR268W	YGL145W	999
YLR268W	YGR120C	923
YLR268W	YHL031C	999
YLR268W	YIL004C	999
YLR268W	YIL109C	999
YLR268W	YJL168C	973
YLR268W	YKL006C-A	999
YLR268W	YKL196C	999
YLR268W	YLR026C	999
YLR268W	YLR078C	999
YLR268W	YLR093C	999
YLR268W	YNL258C	997

YLR268W	YNL287W	998
YLR268W	YOR075W	999
YLR268W	YOR216C	979
YLR268W	YOR307C	923
YLR268W	YOR327C	998
YLR268W	YPL218W	977
YLR268W	YPR181C	999
YLR270W	YDR001C	988
YLR270W	YOR173W	999
YLR272C	YBL097W	999
YLR272C	YDR325W	999
YLR272C	YFR031C	999
YLR272C	YLR086W	999
YLR273C	YLR258W	966
YLR274W	YAR007C	987
YLR274W	YBL023C	999
YLR274W	YBR060C	999
YLR274W	YBR160W	909
YLR274W	YBR202W	999
YLR274W	YDL017W	999
YLR274W	YDR013W	901
YLR274W	YDR052C	999
YLR274W	YDR489W	992
YLR274W	YEL032W	999
YLR274W	YGL201C	996
YLR274W	YHR118C	956
YLR274W	YIL150C	991
YLR274W	YIR008C	904
YLR274W	YJL194W	999
YLR274W	YJR046W	997
YLR274W	YKL045W	907
YLR274W	YLR103C	999
YLR274W	YML065W	992
YLR274W	YNL102W	939
YLR274W	YNL261W	999
YLR274W	YNL262W	902
YLR274W	YNL312W	903
YLR274W	YPR019W	998
YLR275W	YBL026W	999
YLR275W	YBL074C	995
YLR275W	YBR055C	999
YLR275W	YBR119W	995
YLR275W	YCR077C	997
YLR275W	YDL030W	992

YLR275W	YDL043C	991
YLR275W	YDL087C	999
YLR275W	YDL098C	976
YLR275W	YDL140C	905
YLR275W	YDR235W	988
YLR275W	YDR240C	996
YLR275W	YDR378C	999
YLR275W	YDR404C	900
YLR275W	YDR473C	997
YLR275W	YEL026W	915
YLR275W	YER029C	999
YLR275W	YER112W	999
YLR275W	YER146W	995
YLR275W	YER172C	999
YLR275W	YFL017W-A	999
YLR275W	YGL128C	992
YLR275W	YGL173C	929
YLR275W	YGR013W	999
YLR275W	YGR074W	999
YLR275W	YGR091W	999
YLR275W	YGR271W	904
YLR275W	YHR086W	999
YLR275W	YHR165C	999
YLR275W	YIL061C	993
YLR275W	YJL124C	998
YLR275W	YJR022W	999
YLR275W	YKL012W	988
YLR275W	YKL173W	999
YLR275W	YLR147C	999
YLR275W	YLR298C	999
YLR275W	YML046W	989
YLR275W	YML049C	997
YLR275W	YMR125W	999
YLR275W	YMR213W	961
YLR275W	YMR240C	998
YLR275W	YMR288W	997
YLR275W	YNL147W	999
YLR275W	YOL005C	909
YLR275W	YOR159C	999
YLR275W	YOR210W	911
YLR275W	YOR224C	921
YLR275W	YOR308C	989
YLR275W	YPL178W	999
YLR275W	YPL213W	999

YLR275W	YPR057W	987
YLR275W	YPR082C	997
YLR275W	YPR178W	990
YLR275W	YPR182W	999
YLR275W	YPR187W	911
YLR276C	YBR142W	984
YLR276C	YDL031W	998
YLR276C	YDR060W	998
YLR276C	YDR496C	991
YLR276C	YER006W	982
YLR276C	YGL111W	986
YLR276C	YGL135W	981
YLR276C	YHR052W	988
YLR276C	YHR066W	993
YLR276C	YJR041C	949
YLR276C	YKL172W	993
YLR276C	YKR081C	997
YLR276C	YLL008W	994
YLR276C	YMR049C	974
YLR276C	YMR290C	993
YLR276C	YMR308C	912
YLR276C	YNL002C	991
YLR276C	YNL061W	982
YLR276C	YNL256W	913
YLR276C	YOL077C	987
YLR276C	YOL127W	936
YLR276C	YOR063W	961
YLR276C	YOR206W	986
YLR276C	YPL012W	982
YLR276C	YPL043W	998
YLR276C	YPL093W	981
YLR276C	YPL211W	979
YLR277C	YAL043C	999
YLR277C	YDR195W	999
YLR277C	YDR228C	999
YLR277C	YDR301W	999
YLR277C	YER133W	999
YLR277C	YGR047C	928
YLR277C	YGR156W	995
YLR277C	YJL033W	961
YLR277C	YJR093C	999
YLR277C	YKL018W	999
YLR277C	YKL059C	999
YLR277C	YKR002W	999

YLR277C	YLR115W	999
YLR277C	YLR448W	976
YLR277C	YMR061W	999
YLR277C	YNL222W	999
YLR277C	YNL317W	999
YLR277C	YOR179C	943
YLR277C	YPR107C	999
YLR284C	YDR244W	914
YLR284C	YOR180C	998
YLR286C	YDR146C	923
YLR286C	YGL028C	910
YLR286C	YHR143W	935
YLR286C	YNL066W	908
YLR286C	YNL161W	924
YLR286C	YNL327W	940
YLR287C	YLR340W	999
YLR287C-A	YBL027W	997
YLR287C-A	YBL072C	992
YLR287C-A	YBL092W	997
YLR287C-A	YBR031W	995
YLR287C-A	YBR048W	996
YLR287C-A	YBR181C	994
YLR287C-A	YBR189W	902
YLR287C-A	YBR191W	997
YLR287C-A	YCR031C	995
YLR287C-A	YDL061C	900
YLR287C-A	YDL075W	995
YLR287C-A	YDL081C	902
YLR287C-A	YDL082W	995
YLR287C-A	YDL083C	952
YLR287C-A	YDL130W	902
YLR287C-A	YDL136W	994
YLR287C-A	YDL191W	905
YLR287C-A	YDR012W	912
YLR287C-A	YDR064W	993
YLR287C-A	YDR382W	995
YLR287C-A	YDR418W	912
YLR287C-A	YDR447C	995
YLR287C-A	YDR450W	901
YLR287C-A	YDR471W	995
YLR287C-A	YDR500C	906
YLR287C-A	YEL054C	912
YLR287C-A	YER056C-A	907
YLR287C-A	YER074W	994

YLR287C-A	YER131W	905
YLR287C-A	YFL034C-A	906
YLR287C-A	YFR031C-A	995
YLR287C-A	YFR032C-A	997
YLR287C-A	YGL014W	978
YLR287C-A	YGL030W	996
YLR287C-A	YGL076C	908
YLR287C-A	YGL103W	995
YLR287C-A	YGL135W	996
YLR287C-A	YGL189C	905
YLR287C-A	YGR027C	997
YLR287C-A	YGR034W	995
YLR287C-A	YGR118W	997
YLR287C-A	YHL001W	994
YLR287C-A	YHL015W	911
YLR287C-A	YHL033C	914
YLR287C-A	YHR010W	910
YLR287C-A	YHR021C	905
YLR287C-A	YHR029C	972
YLR287C-A	YHR141C	997
YLR287C-A	YHR203C	906
YLR287C-A	YIL052C	907
YLR287C-A	YIL069C	901
YLR287C-A	YIL133C	910
YLR287C-A	YJL136C	909
YLR287C-A	YJL177W	994
YLR287C-A	YJL190C	900
YLR287C-A	YJL191W	908
YLR287C-A	YJR094W-A	903
YLR287C-A	YJR123W	995
YLR287C-A	YJR145C	906
YLR287C-A	YKL006W	902
YLR287C-A	YKL156W	905
YLR287C-A	YKL180W	995
YLR287C-A	YKR057W	909
YLR287C-A	YLL045C	914
YLR287C-A	YLR029C	996
YLR287C-A	YLR061W	908
YLR287C-A	YLR075W	995
YLR287C-A	YLR185W	906
YLR287C-A	YLR264W	902
YLR287C-A	YLR340W	902
YLR287C-A	YLR344W	908
YLR287C-A	YLR367W	900

YLR287C-A	YLR388W	900
YLR287C-A	YLR406C	909
YLR287C-A	YLR441C	994
YLR287C-A	YLR448W	995
YLR287C-A	YML024W	902
YLR287C-A	YML026C	901
YLR287C-A	YML063W	995
YLR287C-A	YML073C	995
YLR287C-A	YMR121C	908
YLR287C-A	YMR194W	995
YLR287C-A	YMR230W	906
YLR287C-A	YMR242C	995
YLR287C-A	YNL001W	976
YLR287C-A	YNL069C	995
YLR287C-A	YNL096C	997
YLR287C-A	YNL162W	915
YLR287C-A	YNL178W	995
YLR287C-A	YNL302C	997
YLR287C-A	YOL039W	996
YLR287C-A	YOL040C	997
YLR287C-A	YOL121C	903
YLR287C-A	YOL127W	996
YLR287C-A	YOR063W	993
YLR287C-A	YOR096W	917
YLR287C-A	YOR167C	902
YLR287C-A	YOR182C	901
YLR287C-A	YOR234C	903
YLR287C-A	YOR293W	906
YLR287C-A	YOR312C	903
YLR287C-A	YOR369C	909
YLR287C-A	YPL081W	902
YLR287C-A	YPL143W	903
YLR287C-A	YPL198W	908
YLR287C-A	YPL220W	910
YLR287C-A	YPL249C-A	902
YLR287C-A	YPR043W	903
YLR287C-A	YPR132W	900
YLR288C	YAR007C	964
YLR288C	YBL088C	989
YLR288C	YBR136W	986
YLR288C	YDL220C	948
YLR288C	YDR217C	970
YLR288C	YER095W	972
YLR288C	YER173W	946

YLR288C	YHR031C	975
YLR288C	YHR119W	995
YLR288C	YIR008C	989
YLR288C	YJR043C	930
YLR288C	YLR119W	964
YLR288C	YML028W	900
YLR288C	YMR190C	994
YLR288C	YNL102W	904
YLR288C	YNL312W	954
YLR288C	YOR144C	941
YLR288C	YOR217W	985
YLR288C	YOR368W	999
YLR288C	YPL194W	999
YLR289W	YER154W	923
YLR291C	YBR159W	978
YLR291C	YBR176W	931
YLR291C	YDR211W	999
YLR291C	YDR283C	998
YLR291C	YER025W	999
YLR291C	YGR083C	999
YLR291C	YHL018W	942
YLR291C	YJR007W	999
YLR291C	YKR026C	999
YLR291C	YLR245C	958
YLR291C	YNL265C	985
YLR291C	YOL130W	900
YLR291C	YOR260W	999
YLR291C	YPL070W	996
YLR291C	YPL124W	931
YLR291C	YPL237W	999
YLR292C	YBR171W	999
YLR292C	YDR086C	917
YLR292C	YER087C-B	992
YLR292C	YJL034W	984
YLR292C	YLR301W	933
YLR292C	YLR378C	908
YLR292C	YNL153C	901
YLR292C	YOR254C	999
YLR292C	YPL094C	999
YLR293C	YAR002W	904
YLR293C	YBR017C	995
YLR293C	YDR002W	999
YLR293C	YDR192C	964
YLR293C	YDR335W	976

YLR293C	YER009W	998
YLR293C	YER110C	999
YLR293C	YGL097W	999
YLR293C	YGL164C	903
YLR293C	YGL238W	999
YLR293C	YGL241W	966
YLR293C	YGR128C	900
YLR293C	YGR218W	999
YLR293C	YIL063C	999
YLR293C	YIL115C	972
YLR293C	YJL041W	992
YLR293C	YJR074W	999
YLR293C	YKL205W	999
YLR293C	YLR335W	983
YLR293C	YLR347C	999
YLR293C	YMR235C	996
YLR293C	YMR308C	999
YLR293C	YNL189W	989
YLR293C	YOL021C	987
YLR293C	YOR160W	996
YLR293C	YOR185C	998
YLR293C	YPL012W	907
YLR293C	YPL125W	983
YLR295C	YBL099W	999
YLR295C	YBR039W	917
YLR295C	YDL004W	908
YLR295C	YJR121W	999
YLR295C	YKL016C	961
YLR295C	YPL078C	999
YLR298C	YBR119W	999
YLR298C	YDL087C	999
YLR298C	YDR235W	999
YLR298C	YDR240C	999
YLR298C	YER029C	999
YLR298C	YFL017W-A	992
YLR298C	YGR013W	999
YLR298C	YGR074W	999
YLR298C	YHR086W	999
YLR298C	YIL061C	999
YLR298C	YKL012W	999
YLR298C	YLR147C	988
YLR298C	YLR275W	999
YLR298C	YML046W	999
YLR298C	YMR125W	999

YLR299W	YBR244W	901
YLR299W	YIR037W	901
YLR299W	YJL101C	928
YLR299W	YOL049W	919
YLR299W	YPL091W	945
YLR300W	YGR282C	909
YLR301W	YLR292C	933
YLR303W	YAL012W	940
YLR303W	YER091C	964
YLR303W	YFR055W	908
YLR303W	YGR012W	966
YLR303W	YGR155W	969
YLR303W	YJL101C	943
YLR303W	YJR010W	917
YLR303W	YJR060W	918
YLR303W	YJR130C	910
YLR303W	YJR137C	937
YLR303W	YKL001C	925
YLR303W	YNL103W	985
YLR303W	YNL277W	996
YLR304C	YCR005C	955
YLR304C	YDL066W	909
YLR304C	YDL120W	972
YLR304C	YDR480W	923
YLR304C	YER065C	934
YLR304C	YIL094C	934
YLR304C	YJL200C	915
YLR304C	YLR174W	934
YLR304C	YMR072W	982
YLR304C	YNL009W	908
YLR304C	YNL037C	965
YLR304C	YNR001C	996
YLR304C	YOR136W	994
YLR304C	YPR001W	908
YLR305C	YBL105C	955
YLR305C	YBR036C	915
YLR305C	YDR208W	991
YLR305C	YDR473C	940
YLR305C	YER120W	996
YLR305C	YJL100W	991
YLR305C	YKL051W	936
YLR305C	YKL212W	999
YLR305C	YLR240W	912
YLR305C	YNL106C	943

YLR305C	YNL267W	996
YLR305C	YOR109W	943
YLR305C	YPL268W	951
YLR306W	YDR139C	988
YLR306W	YLR128W	926
YLR309C	YBR164C	988
YLR309C	YDR285W	999
YLR309C	YLR039C	955
YLR309C	YLR262C	999
YLR309C	YOR195W	999
YLR310C	YBR140C	992
YLR310C	YBR225W	999
YLR310C	YDL047W	933
YLR310C	YDL155W	978
YLR310C	YDR096W	971
YLR310C	YDR099W	974
YLR310C	YER177W	975
YLR310C	YGL197W	999
YLR310C	YGR152C	940
YLR310C	YHR205W	980
YLR310C	YJL005W	989
YLR310C	YJL098W	991
YLR310C	YJL164C	939
YLR310C	YLR178C	993
YLR310C	YLR229C	924
YLR310C	YNL098C	999
YLR310C	YOL081W	973
YLR310C	YOR101W	999
YLR310C	YPL256C	977
YLR312W-A	YBL038W	972
YLR312W-A	YCR046C	991
YLR312W-A	YCR071C	988
YLR312W-A	YDR116C	988
YLR312W-A	YDR237W	977
YLR312W-A	YDR296W	991
YLR312W-A	YDR322W	987
YLR312W-A	YGR220C	996
YLR312W-A	YJL041W	920
YLR312W-A	YJL063C	972
YLR312W-A	YKL167C	972
YLR312W-A	YKR006C	972
YLR312W-A	YKR085C	972
YLR312W-A	YLR439W	988
YLR312W-A	YML025C	972

YLR312W-A	YMR024W	988
YLR312W-A	YMR193W	978
YLR312W-A	YNL005C	976
YLR312W-A	YNL252C	972
YLR313C	YLR319C	984
YLR313C	YLR362W	969
YLR314C	YCL024W	983
YLR314C	YCR002C	999
YLR314C	YDL225W	999
YLR314C	YDR409W	989
YLR314C	YDR507C	999
YLR314C	YDR510W	999
YLR314C	YHR107C	999
YLR314C	YJL060W	959
YLR314C	YJR076C	999
YLR314C	YJR092W	935
YLR314C	YKL101W	997
YLR314C	YMR055C	973
YLR314C	YNL233W	949
YLR314C	YOR156C	982
YLR315W	YBR211C	999
YLR315W	YDR254W	997
YLR315W	YDR318W	999
YLR315W	YDR383C	999
YLR315W	YER016W	982
YLR315W	YGR179C	999
YLR315W	YJR135C	999
YLR315W	YPL233W	906
YLR315W	YPR046W	974
YLR316C	YJL035C	984
YLR318W	YBL088C	993
YLR318W	YDL220C	990
YLR318W	YDR082W	999
YLR318W	YDR217C	906
YLR318W	YIL009C-A	999
YLR318W	YLR233C	999
YLR318W	YML061C	991
YLR318W	YMR106C	999
YLR318W	YMR190C	996
YLR318W	YMR284W	999
YLR319C	YDR184C	968
YLR319C	YER149C	994
YLR319C	YFL039C	999
YLR319C	YGL015C	943

YLR319C	YIL159W	954
YLR319C	YLL021W	998
YLR319C	YLR229C	904
YLR319C	YLR313C	984
YLR319C	YLR362W	963
YLR319C	YNL271C	999
YLR319C	YNL298W	960
YLR319C	YPL269W	953
YLR320W	YHR154W	993
YLR320W	YJL047C	997
YLR320W	YJL092W	953
YLR321C	YBR089C-A	916
YLR321C	YBR245C	962
YLR321C	YCR020W-B	998
YLR321C	YCR052W	999
YLR321C	YDR303C	999
YLR321C	YFR037C	999
YLR321C	YGR056W	999
YLR321C	YGR275W	999
YLR321C	YHR056C	996
YLR321C	YIL126W	999
YLR321C	YKR008W	999
YLR321C	YLR033W	999
YLR321C	YLR345W	953
YLR321C	YLR357W	999
YLR321C	YML032C	927
YLR321C	YML127W	999
YLR321C	YMR033W	999
YLR321C	YMR091C	999
YLR321C	YPL070W	987
YLR321C	YPR034W	999
YLR324W	YDL065C	986
YLR324W	YDL089W	957
YLR324W	YDR479C	956
YLR324W	YGR004W	909
YLR324W	YMR204C	985
YLR325C	YBL027W	901
YLR325C	YBL072C	915
YLR325C	YBL087C	961
YLR325C	YBL092W	915
YLR325C	YBR048W	917
YLR325C	YBR084C-A	901
YLR325C	YBR181C	937
YLR325C	YBR189W	927

YLR325C	YBR191W	909
YLR325C	YCR031C	914
YLR325C	YCR072C	970
YLR325C	YDL061C	911
YLR325C	YDL081C	924
YLR325C	YDL082W	939
YLR325C	YDL136W	929
YLR325C	YDL191W	929
YLR325C	YDR025W	917
YLR325C	YDR064W	965
YLR325C	YDR418W	924
YLR325C	YDR447C	965
YLR325C	YDR500C	917
YLR325C	YEL054C	924
YLR325C	YER006W	978
YLR325C	YER074W	929
YLR325C	YER102W	915
YLR325C	YER117W	929
YLR325C	YER126C	970
YLR325C	YER131W	931
YLR325C	YFR031C-A	929
YLR325C	YFR032C-A	949
YLR325C	YGL030W	998
YLR325C	YGL031C	938
YLR325C	YGL076C	941
YLR325C	YGL103W	997
YLR325C	YGL123W	914
YLR325C	YGL147C	958
YLR325C	YGL189C	921
YLR325C	YGR027C	942
YLR325C	YGR034W	950
YLR325C	YGR085C	916
YLR325C	YGR118W	929
YLR325C	YGR148C	932
YLR325C	YGR214W	918
YLR325C	YHL001W	924
YLR325C	YHR021C	931
YLR325C	YHR141C	946
YLR325C	YIL069C	929
YLR325C	YIL133C	953
YLR325C	YJL136C	930
YLR325C	YJL190C	978
YLR325C	YJL191W	914
YLR325C	YJR094W-A	927

YLR325C	YJR123W	934
YLR325C	YKL006W	924
YLR325C	YKL156W	931
YLR325C	YKR057W	930
YLR325C	YLL045C	939
YLR325C	YLR029C	916
YLR325C	YLR048W	909
YLR325C	YLR074C	974
YLR325C	YLR075W	937
YLR325C	YLR185W	917
YLR325C	YLR333C	942
YLR325C	YLR340W	912
YLR325C	YLR367W	911
YLR325C	YLR388W	912
YLR325C	YLR441C	956
YLR325C	YLR448W	916
YLR325C	YML024W	932
YLR325C	YML063W	926
YLR325C	YML073C	927
YLR325C	YMR121C	924
YLR325C	YMR142C	931
YLR325C	YMR194W	930
YLR325C	YMR230W	928
YLR325C	YMR242C	973
YLR325C	YMR260C	917
YLR325C	YNL067W	909
YLR325C	YNL069C	915
YLR325C	YNL096C	927
YLR325C	YNL162W	912
YLR325C	YNL178W	930
YLR325C	YNL301C	904
YLR325C	YNL302C	917
YLR325C	YNR053C	970
YLR325C	YOL040C	969
YLR325C	YOL120C	904
YLR325C	YOL121C	961
YLR325C	YOL127W	998
YLR325C	YOR063W	997
YLR325C	YOR096W	904
YLR325C	YOR167C	921
YLR325C	YOR234C	925
YLR325C	YOR293W	931
YLR325C	YOR312C	914
YLR325C	YOR369C	987

YLR325C	YPL079W	925
YLR325C	YPL081W	919
YLR325C	YPL093W	974
YLR325C	YPL131W	958
YLR325C	YPL143W	926
YLR325C	YPL198W	923
YLR325C	YPL249C-A	998
YLR325C	YPR043W	927
YLR325C	YPR102C	929
YLR325C	YPR132W	929
YLR328W	YDL042C	991
YLR328W	YFR047C	907
YLR328W	YGR010W	999
YLR328W	YLR438W	991
YLR328W	YNL129W	989
YLR328W	YOR209C	957
YLR329W	YER044C-A	940
YLR329W	YHL022C	999
YLR329W	YHR014W	998
YLR329W	YHR157W	999
YLR329W	YML032C	981
YLR330W	YBR023C	968
YLR330W	YCL027W	999
YLR330W	YDL192W	996
YLR330W	YDR351W	926
YLR330W	YER131W	980
YLR330W	YER149C	999
YLR330W	YGL210W	903
YLR330W	YJL099W	999
YLR330W	YKL193C	999
YLR330W	YKR027W	999
YLR330W	YLL021W	976
YLR330W	YMR232W	999
YLR330W	YMR237W	999
YLR330W	YMR307W	922
YLR330W	YOR299W	999
YLR332W	YBR260C	968
YLR332W	YGR023W	993
YLR332W	YGR229C	984
YLR332W	YLR342W	979
YLR332W	YLR371W	981
YLR332W	YNL283C	972
YLR332W	YOR008C	999
YLR332W	YPR165W	999

YLR333C	YBL027W	912
YLR333C	YBL072C	920
YLR333C	YBL087C	935
YLR333C	YBL092W	943
YLR333C	YBR031W	926
YLR333C	YBR048W	912
YLR333C	YBR084C-A	912
YLR333C	YBR118W	907
YLR333C	YBR181C	916
YLR333C	YBR189W	943
YLR333C	YBR191W	937
YLR333C	YCR031C	929
YLR333C	YDL061C	922
YLR333C	YDL075W	919
YLR333C	YDL081C	933
YLR333C	YDL082W	932
YLR333C	YDL136W	916
YLR333C	YDL191W	916
YLR333C	YDR012W	929
YLR333C	YDR025W	912
YLR333C	YDR064W	963
YLR333C	YDR382W	932
YLR333C	YDR385W	907
YLR333C	YDR418W	931
YLR333C	YDR447C	929
YLR333C	YDR450W	914
YLR333C	YDR471W	919
YLR333C	YDR500C	910
YLR333C	YEL054C	916
YLR333C	YER056C-A	914
YLR333C	YER074W	915
YLR333C	YER102W	920
YLR333C	YER117W	923
YLR333C	YER131W	976
YLR333C	YFR031C-A	923
YLR333C	YFR032C-A	934
YLR333C	YGL030W	951
YLR333C	YGL031C	943
YLR333C	YGL076C	945
YLR333C	YGL103W	929
YLR333C	YGL123W	927
YLR333C	YGL135W	911
YLR333C	YGL147C	945
YLR333C	YGL189C	915

YLR333C	YGR027C	964
YLR333C	YGR034W	919
YLR333C	YGR085C	932
YLR333C	YGR118W	944
YLR333C	YGR148C	952
YLR333C	YGR214W	948
YLR333C	YHL001W	952
YLR333C	YHL015W	941
YLR333C	YHL033C	927
YLR333C	YHR010W	919
YLR333C	YHR021C	920
YLR333C	YHR141C	929
YLR333C	YHR203C	905
YLR333C	YIL018W	913
YLR333C	YIL052C	931
YLR333C	YIL069C	915
YLR333C	YIL133C	923
YLR333C	YJL136C	938
YLR333C	YJL177W	926
YLR333C	YJL189W	903
YLR333C	YJL190C	928
YLR333C	YJL191W	927
YLR333C	YJR094W-A	935
YLR333C	YJR123W	965
YLR333C	YJR145C	905
YLR333C	YKL006W	953
YLR333C	YKL156W	933
YLR333C	YKL180W	930
YLR333C	YKR057W	930
YLR333C	YLL045C	934
YLR333C	YLR029C	944
YLR333C	YLR048W	936
YLR333C	YLR061W	914
YLR333C	YLR075W	935
YLR333C	YLR185W	910
YLR333C	YLR264W	925
YLR333C	YLR325C	942
YLR333C	YLR340W	936
YLR333C	YLR344W	925
YLR333C	YLR367W	927
YLR333C	YLR388W	928
YLR333C	YLR406C	905
YLR333C	YLR441C	931
YLR333C	YLR448W	938

YLR333C	YML024W	940
YLR333C	YML026C	914
YLR333C	YML063W	949
YLR333C	YML073C	923
YLR333C	YMR121C	912
YLR333C	YMR142C	928
YLR333C	YMR194W	939
YLR333C	YMR230W	935
YLR333C	YMR242C	942
YLR333C	YMR260C	900
YLR333C	YNL067W	944
YLR333C	YNL069C	908
YLR333C	YNL096C	917
YLR333C	YNL162W	918
YLR333C	YNL178W	958
YLR333C	YNL301C	916
YLR333C	YNL302C	921
YLR333C	YOL039W	921
YLR333C	YOL040C	953
YLR333C	YOL120C	916
YLR333C	YOL121C	931
YLR333C	YOL127W	935
YLR333C	YOR063W	925
YLR333C	YOR096W	928
YLR333C	YOR133W	907
YLR333C	YOR167C	929
YLR333C	YOR234C	934
YLR333C	YOR293W	945
YLR333C	YOR312C	927
YLR333C	YOR369C	953
YLR333C	YPL079W	936
YLR333C	YPL081W	921
YLR333C	YPL131W	921
YLR333C	YPL143W	957
YLR333C	YPL198W	926
YLR333C	YPL220W	911
YLR333C	YPL237W	910
YLR333C	YPL249C-A	929
YLR333C	YPR043W	928
YLR333C	YPR080W	907
YLR333C	YPR102C	960
YLR333C	YPR132W	927
YLR335W	YAR002W	999
YLR335W	YBL079W	998

YLR335W	YDL088C	936
YLR335W	YDL116W	956
YLR335W	YDR002W	999
YLR335W	YDR192C	974
YLR335W	YER110C	999
YLR335W	YFR002W	963
YLR335W	YGL016W	990
YLR335W	YGL097W	998
YLR335W	YGL172W	998
YLR335W	YGL238W	970
YLR335W	YGR119C	999
YLR335W	YIL063C	908
YLR335W	YJL039C	957
YLR335W	YJL041W	999
YLR335W	YJR042W	991
YLR335W	YKL057C	980
YLR335W	YKL068W	993
YLR335W	YKL205W	912
YLR335W	YKR082W	983
YLR335W	YLR018C	960
YLR335W	YLR293C	983
YLR335W	YLR347C	999
YLR335W	YML103C	934
YLR335W	YMR047C	991
YLR335W	YMR153W	949
YLR335W	YNL189W	999
YLR335W	YOR098C	999
YLR335W	YOR112W	977
YLR335W	YOR257W	961
YLR335W	YPL020C	958
YLR335W	YPL153C	938
YLR335W	YPL169C	931
YLR336C	YDL022W	907
YLR336C	YJL128C	906
YLR336C	YLR113W	987
YLR337C	YBL007C	999
YLR337C	YBR260C	983
YLR337C	YDR388W	997
YLR337C	YFL039C	999
YLR337C	YHR114W	984
YLR337C	YIL118W	907
YLR337C	YJL020C	987
YLR337C	YKL129C	999
YLR337C	YLR229C	958

YLR337C	YMR032W	996
YLR337C	YMR109W	999
YLR337C	YOR181W	999
YLR340W	YAL003W	908
YLR340W	YAL035W	934
YLR340W	YBL027W	966
YLR340W	YBL072C	997
YLR340W	YBL087C	971
YLR340W	YBL092W	982
YLR340W	YBR031W	992
YLR340W	YBR048W	997
YLR340W	YBR084C-A	987
YLR340W	YBR084W	974
YLR340W	YBR118W	931
YLR340W	YBR181C	923
YLR340W	YBR189W	998
YLR340W	YBR191W	996
YLR340W	YCR031C	999
YLR340W	YDL061C	978
YLR340W	YDL075W	999
YLR340W	YDL081C	999
YLR340W	YDL082W	997
YLR340W	YDL083C	975
YLR340W	YDL130W	999
YLR340W	YDL136W	952
YLR340W	YDL191W	934
YLR340W	YDR012W	999
YLR340W	YDR025W	927
YLR340W	YDR064W	999
YLR340W	YDR091C	957
YLR340W	YDR382W	999
YLR340W	YDR385W	999
YLR340W	YDR418W	999
YLR340W	YDR447C	947
YLR340W	YDR450W	937
YLR340W	YDR471W	949
YLR340W	YDR500C	972
YLR340W	YEL034W	953
YLR340W	YEL054C	978
YLR340W	YER056C-A	980
YLR340W	YER074W	995
YLR340W	YER102W	979
YLR340W	YER117W	989
YLR340W	YER131W	933

YLR340W	YFL034C-A	920
YLR340W	YFR031C-A	997
YLR340W	YFR032C-A	968
YLR340W	YGL030W	998
YLR340W	YGL031C	937
YLR340W	YGL076C	961
YLR340W	YGL103W	999
YLR340W	YGL123W	999
YLR340W	YGL135W	999
YLR340W	YGL147C	998
YLR340W	YGL189C	919
YLR340W	YGR027C	968
YLR340W	YGR034W	998
YLR340W	YGR085C	999
YLR340W	YGR118W	985
YLR340W	YGR148C	975
YLR340W	YGR214W	984
YLR340W	YHL001W	999
YLR340W	YHL015W	999
YLR340W	YHL033C	997
YLR340W	YHR010W	942
YLR340W	YHR021C	940
YLR340W	YHR141C	939
YLR340W	YHR203C	984
YLR340W	YIL018W	997
YLR340W	YIL052C	995
YLR340W	YIL069C	911
YLR340W	YIL133C	997
YLR340W	YIL148W	964
YLR340W	YJL136C	972
YLR340W	YJL177W	999
YLR340W	YJL189W	938
YLR340W	YJL190C	996
YLR340W	YJL191W	978
YLR340W	YJR094W-A	988
YLR340W	YJR123W	999
YLR340W	YJR145C	944
YLR340W	YKL006W	994
YLR340W	YKL156W	969
YLR340W	YKL180W	999
YLR340W	YKR057W	943
YLR340W	YKR094C	922
YLR340W	YLL045C	967
YLR340W	YLR029C	999

YLR340W	YLR048W	998
YLR340W	YLR061W	994
YLR340W	YLR075W	999
YLR340W	YLR167W	971
YLR340W	YLR185W	968
YLR340W	YLR264W	976
YLR340W	YLR287C	999
YLR340W	YLR287C-A	902
YLR340W	YLR325C	912
YLR340W	YLR333C	936
YLR340W	YLR344W	990
YLR340W	YLR367W	963
YLR340W	YLR388W	996
YLR340W	YLR406C	934
YLR340W	YLR441C	997
YLR340W	YLR448W	999
YLR340W	YML024W	995
YLR340W	YML026C	937
YLR340W	YML063W	998
YLR340W	YML073C	997
YLR340W	YMR121C	960
YLR340W	YMR142C	949
YLR340W	YMR194W	999
YLR340W	YMR230W	951
YLR340W	YMR242C	997
YLR340W	YMR260C	937
YLR340W	YNL067W	981
YLR340W	YNL069C	997
YLR340W	YNL096C	938
YLR340W	YNL132W	940
YLR340W	YNL162W	939
YLR340W	YNL178W	999
YLR340W	YNL301C	998
YLR340W	YNL302C	954
YLR340W	YNR037C	906
YLR340W	YOL039W	999
YLR340W	YOL040C	981
YLR340W	YOL120C	957
YLR340W	YOL121C	954
YLR340W	YOL127W	999
YLR340W	YOR063W	997
YLR340W	YOR096W	981
YLR340W	YOR133W	967
YLR340W	YOR167C	945

YLR340W	YOR182C	902
YLR340W	YOR234C	971
YLR340W	YOR293W	957
YLR340W	YOR312C	952
YLR340W	YOR369C	941
YLR340W	YPL037C	983
YLR340W	YPL079W	969
YLR340W	YPL081W	933
YLR340W	YPL090C	996
YLR340W	YPL131W	999
YLR340W	YPL143W	981
YLR340W	YPL198W	950
YLR340W	YPL220W	999
YLR340W	YPL249C-A	998
YLR340W	YPR043W	971
YLR340W	YPR080W	922
YLR340W	YPR102C	979
YLR340W	YPR132W	984
YLR342W	YAL007C	901
YLR342W	YBL061C	964
YLR342W	YBL105C	986
YLR342W	YBR023C	968
YLR342W	YDR170C	988
YLR342W	YDR245W	981
YLR342W	YEL036C	975
YLR342W	YGL031C	970
YLR342W	YGL103W	974
YLR342W	YGR032W	999
YLR342W	YGR229C	940
YLR342W	YGR231C	972
YLR342W	YHR030C	965
YLR342W	YJR077C	960
YLR342W	YKL035W	900
YLR342W	YKL190W	997
YLR342W	YKL203C	973
YLR342W	YKR090W	905
YLR342W	YLR332W	979
YLR342W	YLR371W	994
YLR342W	YLR433C	998
YLR342W	YML057W	996
YLR342W	YMR306W	969
YLR342W	YNL294C	912
YLR342W	YOR008C	930
YLR342W	YPL089C	972

YLR342W	YPR165W	999
YLR344W	YAL035W	917
YLR344W	YBL027W	999
YLR344W	YBL072C	920
YLR344W	YBL087C	997
YLR344W	YBL092W	998
YLR344W	YBR031W	982
YLR344W	YBR048W	976
YLR344W	YBR084C-A	999
YLR344W	YBR118W	914
YLR344W	YBR181C	997
YLR344W	YBR189W	965
YLR344W	YBR191W	999
YLR344W	YCR031C	965
YLR344W	YDL061C	987
YLR344W	YDL075W	999
YLR344W	YDL081C	941
YLR344W	YDL082W	911
YLR344W	YDL130W	954
YLR344W	YDL136W	999
YLR344W	YDL191W	932
YLR344W	YDR012W	998
YLR344W	YDR025W	972
YLR344W	YDR064W	997
YLR344W	YDR172W	980
YLR344W	YDR382W	994
YLR344W	YDR385W	909
YLR344W	YDR418W	997
YLR344W	YDR447C	997
YLR344W	YDR450W	924
YLR344W	YDR471W	999
YLR344W	YDR500C	986
YLR344W	YEL054C	931
YLR344W	YER056C-A	998
YLR344W	YER074W	905
YLR344W	YER102W	989
YLR344W	YER117W	998
YLR344W	YER131W	930
YLR344W	YFL034C-A	920
YLR344W	YFR031C-A	999
YLR344W	YFR032C-A	938
YLR344W	YGL030W	996
YLR344W	YGL031C	991
YLR344W	YGL076C	993

YLR344W	YGL103W	999
YLR344W	YGL123W	995
YLR344W	YGL135W	992
YLR344W	YGL147C	997
YLR344W	YGL189C	910
YLR344W	YGR027C	963
YLR344W	YGR034W	997
YLR344W	YGR085C	993
YLR344W	YGR118W	994
YLR344W	YGR148C	974
YLR344W	YGR214W	971
YLR344W	YHL001W	978
YLR344W	YHL015W	979
YLR344W	YHL033C	998
YLR344W	YHR010W	959
YLR344W	YHR021C	925
YLR344W	YHR141C	993
YLR344W	YHR203C	995
YLR344W	YIL018W	970
YLR344W	YIL052C	999
YLR344W	YIL069C	905
YLR344W	YIL133C	994
YLR344W	YIL148W	996
YLR344W	YJL136C	974
YLR344W	YJL177W	992
YLR344W	YJL189W	950
YLR344W	YJL190C	974
YLR344W	YJL191W	971
YLR344W	YJR094W-A	999
YLR344W	YJR123W	993
YLR344W	YJR145C	979
YLR344W	YKL006W	998
YLR344W	YKL156W	978
YLR344W	YKL180W	999
YLR344W	YKR057W	931
YLR344W	YKR094C	908
YLR344W	YLL045C	936
YLR344W	YLR029C	996
YLR344W	YLR048W	996
YLR344W	YLR060W	910
YLR344W	YLR061W	999
YLR344W	YLR075W	999
YLR344W	YLR167W	996
YLR344W	YLR185W	998

YLR344W	YLR264W	978
YLR344W	YLR287C-A	908
YLR344W	YLR333C	925
YLR344W	YLR340W	990
YLR344W	YLR367W	987
YLR344W	YLR388W	999
YLR344W	YLR406C	927
YLR344W	YLR441C	915
YLR344W	YLR448W	998
YLR344W	YML024W	999
YLR344W	YML026C	924
YLR344W	YML063W	969
YLR344W	YML073C	970
YLR344W	YMR121C	934
YLR344W	YMR142C	921
YLR344W	YMR194W	999
YLR344W	YMR230W	944
YLR344W	YMR242C	928
YLR344W	YMR260C	917
YLR344W	YNL067W	991
YLR344W	YNL069C	945
YLR344W	YNL096C	933
YLR344W	YNL162W	936
YLR344W	YNL178W	999
YLR344W	YNL301C	997
YLR344W	YNL302C	919
YLR344W	YNR037C	933
YLR344W	YOL039W	922
YLR344W	YOL040C	984
YLR344W	YOL120C	925
YLR344W	YOL121C	920
YLR344W	YOL127W	999
YLR344W	YOR063W	999
YLR344W	YOR096W	981
YLR344W	YOR133W	934
YLR344W	YOR167C	933
YLR344W	YOR182C	908
YLR344W	YOR234C	980
YLR344W	YOR293W	966
YLR344W	YOR312C	938
YLR344W	YOR369C	921
YLR344W	YPL037C	978
YLR344W	YPL079W	967
YLR344W	YPL081W	924

YLR344W	YPL090C	904
YLR344W	YPL131W	999
YLR344W	YPL143W	995
YLR344W	YPL198W	933
YLR344W	YPL220W	987
YLR344W	YPL249C-A	959
YLR344W	YPR043W	971
YLR344W	YPR080W	905
YLR344W	YPR102C	999
YLR344W	YPR132W	998
YLR345W	YAL032C	975
YLR345W	YGR158C	974
YLR345W	YJL155C	999
YLR345W	YLR321C	953
YLR345W	YOR057W	913
YLR347C	YAR002W	999
YLR347C	YBL079W	991
YLR347C	YBR103W	998
YLR347C	YDL088C	997
YLR347C	YDL116W	989
YLR347C	YDL207W	989
YLR347C	YDR083W	940
YLR347C	YDR159W	966
YLR347C	YDR192C	999
YLR347C	YDR225W	992
YLR347C	YDR240C	965
YLR347C	YER009W	967
YLR347C	YER011W	989
YLR347C	YER107C	974
YLR347C	YER110C	999
YLR347C	YFR002W	998
YLR347C	YFR037C	964
YLR347C	YGL092W	999
YLR347C	YGL100W	987
YLR347C	YGL172W	999
YLR347C	YGR103W	973
YLR347C	YGR119C	999
YLR347C	YGR202C	966
YLR347C	YIL115C	999
YLR347C	YJL041W	999
YLR347C	YJL061W	947
YLR347C	YJL173C	988
YLR347C	YJR042W	930
YLR347C	YKL068W	999

YLR347C	YKR082W	928
YLR347C	YLR018C	988
YLR347C	YLR293C	999
YLR347C	YLR335W	999
YLR347C	YML031W	980
YLR347C	YMR047C	999
YLR347C	YMR125W	963
YLR347C	YMR153W	998
YLR347C	YMR308C	999
YLR347C	YNL139C	965
YLR347C	YNL189W	999
YLR347C	YOR001W	995
YLR347C	YOR098C	999
YLR347C	YOR310C	923
YLR347C	YOR361C	923
YLR347C	YPL020C	999
YLR347C	YPL125W	905
YLR347C	YPL169C	994
YLR347C	YPL178W	999
YLR347C	YPR018W	915
YLR347C	YPR093C	976
YLR350W	YHL003C	907
YLR352W	YDL132W	985
YLR352W	YDR328C	998
YLR353W	YBR027C	998
YLR353W	YCL014W	924
YLR353W	YCR038C	954
YLR353W	YGR041W	999
YLR353W	YGR152C	916
YLR353W	YIL140W	928
YLR353W	YJR092W	908
YLR353W	YLR362W	903
YLR353W	YOR299W	903
YLR353W	YOR301W	992
YLR353W	YPR122W	907
YLR354C	YBR117C	959
YLR354C	YGR043C	910
YLR354C	YNL241C	934
YLR354C	YPR074C	994
YLR355C	YCL009C	991
YLR355C	YCL018W	968
YLR355C	YER086W	932
YLR355C	YGL009C	986
YLR355C	YJR016C	996

YLR355C	YMR072W	992
YLR355C	YMR108W	999
YLR355C	YMR302C	911
YLR355C	YNL104C	933
YLR357W	YBR009C	975
YLR357W	YBR049C	983
YLR357W	YBR245C	999
YLR357W	YCR020W-B	999
YLR357W	YCR052W	999
YLR357W	YDL002C	953
YLR357W	YDR224C	989
YLR357W	YDR225W	974
YLR357W	YDR303C	999
YLR357W	YFR037C	999
YLR357W	YGR056W	999
YLR357W	YGR275W	999
YLR357W	YHR056C	999
YLR357W	YIL126W	999
YLR357W	YKR001C	970
YLR357W	YKR008W	999
YLR357W	YLR033W	999
YLR357W	YLR176C	979
YLR357W	YLR321C	999
YLR357W	YML127W	999
YLR357W	YMR033W	999
YLR357W	YMR091C	999
YLR357W	YPL082C	972
YLR357W	YPR034W	999
YLR359W	YAR015W	984
YLR359W	YDR226W	900
YLR359W	YDR441C	913
YLR359W	YGL234W	945
YLR359W	YHR018C	972
YLR359W	YJR105W	934
YLR359W	YKL024C	904
YLR359W	YLR028C	965
YLR359W	YML022W	922
YLR359W	YML035C	935
YLR359W	YMR120C	972
YLR359W	YMR300C	975
YLR359W	YNL220W	996
YLR359W	YOR128C	979
YLR360W	YBR097W	999
YLR360W	YBR128C	983

YLR360W	YLR240W	999
YLR360W	YPL120W	999
YLR361C	YHR079C	925
YLR361C	YMR135C	918
YLR362W	YAL041W	915
YLR362W	YBL016W	999
YLR362W	YBR083W	954
YLR362W	YBR200W	940
YLR362W	YCL032W	999
YLR362W	YDL159W	999
YLR362W	YDR103W	999
YLR362W	YDR168W	999
YLR362W	YER118C	999
YLR362W	YER136W	915
YLR362W	YER149C	901
YLR362W	YFL026W	962
YLR362W	YGR040W	999
YLR362W	YHL007C	999
YLR362W	YHR005C	999
YLR362W	YHR084W	999
YLR362W	YIL049W	912
YLR362W	YJL128C	999
YLR362W	YJL157C	982
YLR362W	YJR086W	968
YLR362W	YKL178C	910
YLR362W	YLL021W	996
YLR362W	YLR006C	999
YLR362W	YLR113W	900
YLR362W	YLR313C	969
YLR362W	YLR319C	963
YLR362W	YLR353W	903
YLR362W	YMR199W	914
YLR362W	YMR232W	998
YLR362W	YNL098C	924
YLR362W	YNL271C	919
YLR362W	YNL298W	982
YLR362W	YOR212W	999
YLR362W	YPL240C	988
YLR362W	YPL256C	909
YLR367W	YAL035W	924
YLR367W	YBL004W	976
YLR367W	YBL027W	969
YLR367W	YBL072C	912
YLR367W	YBL087C	972

YLR367W	YBL092W	984
YLR367W	YBR031W	975
YLR367W	YBR048W	999
YLR367W	YBR079C	911
YLR367W	YBR084C-A	975
YLR367W	YBR189W	999
YLR367W	YBR191W	985
YLR367W	YCR031C	959
YLR367W	YDL061C	995
YLR367W	YDL075W	999
YLR367W	YDL081C	912
YLR367W	YDL082W	931
YLR367W	YDL130W	917
YLR367W	YDL136W	946
YLR367W	YDL191W	921
YLR367W	YDR012W	981
YLR367W	YDR025W	974
YLR367W	YDR041W	900
YLR367W	YDR064W	999
YLR367W	YDR382W	968
YLR367W	YDR385W	901
YLR367W	YDR418W	970
YLR367W	YDR429C	914
YLR367W	YDR447C	998
YLR367W	YDR450W	919
YLR367W	YDR471W	967
YLR367W	YDR500C	931
YLR367W	YEL054C	920
YLR367W	YER025W	952
YLR367W	YER056C-A	970
YLR367W	YER074W	905
YLR367W	YER102W	998
YLR367W	YER117W	983
YLR367W	YER131W	971
YLR367W	YER165W	914
YLR367W	YFL034C-A	907
YLR367W	YFR031C-A	999
YLR367W	YFR032C-A	928
YLR367W	YGL030W	999
YLR367W	YGL031C	944
YLR367W	YGL076C	962
YLR367W	YGL103W	988
YLR367W	YGL123W	994
YLR367W	YGL135W	912

YLR367W	YGL147C	979
YLR367W	YGL189C	914
YLR367W	YGR027C	973
YLR367W	YGR034W	974
YLR367W	YGR085C	999
YLR367W	YGR118W	986
YLR367W	YGR148C	966
YLR367W	YGR214W	978
YLR367W	YHL001W	981
YLR367W	YHL015W	979
YLR367W	YHL033C	971
YLR367W	YHR010W	999
YLR367W	YHR021C	921
YLR367W	YHR141C	928
YLR367W	YHR203C	980
YLR367W	YIL018W	961
YLR367W	YIL052C	979
YLR367W	YIL069C	997
YLR367W	YIL133C	913
YLR367W	YIL148W	934
YLR367W	YJL136C	949
YLR367W	YJL177W	990
YLR367W	YJL189W	926
YLR367W	YJL190C	912
YLR367W	YJL191W	964
YLR367W	YJR094W-A	964
YLR367W	YJR123W	988
YLR367W	YJR145C	999
YLR367W	YKL006W	996
YLR367W	YKL156W	968
YLR367W	YKL170W	931
YLR367W	YKL180W	992
YLR367W	YKR057W	923
YLR367W	YKR059W	926
YLR367W	YKR094C	902
YLR367W	YLL045C	929
YLR367W	YLR029C	968
YLR367W	YLR048W	989
YLR367W	YLR061W	990
YLR367W	YLR075W	952
YLR367W	YLR167W	934
YLR367W	YLR185W	931
YLR367W	YLR264W	996
YLR367W	YLR287C-A	900

YLR367W	YLR325C	911
YLR367W	YLR333C	927
YLR367W	YLR340W	963
YLR367W	YLR344W	987
YLR367W	YLR388W	996
YLR367W	YLR406C	912
YLR367W	YLR441C	915
YLR367W	YLR448W	974
YLR367W	YML024W	972
YLR367W	YML026C	997
YLR367W	YML063W	981
YLR367W	YML073C	920
YLR367W	YMR116C	980
YLR367W	YMR121C	919
YLR367W	YMR142C	932
YLR367W	YMR194W	999
YLR367W	YMR230W	984
YLR367W	YMR242C	922
YLR367W	YMR260C	911
YLR367W	YNL067W	995
YLR367W	YNL069C	912
YLR367W	YNL081C	904
YLR367W	YNL096C	925
YLR367W	YNL162W	920
YLR367W	YNL178W	996
YLR367W	YNL207W	974
YLR367W	YNL301C	959
YLR367W	YNL302C	915
YLR367W	YNR037C	955
YLR367W	YOL039W	916
YLR367W	YOL040C	981
YLR367W	YOL120C	921
YLR367W	YOL121C	915
YLR367W	YOL127W	995
YLR367W	YOL139C	997
YLR367W	YOR056C	976
YLR367W	YOR063W	994
YLR367W	YOR096W	999
YLR367W	YOR133W	911
YLR367W	YOR167C	963
YLR367W	YOR182C	900
YLR367W	YOR234C	974
YLR367W	YOR293W	982
YLR367W	YOR312C	998

YLR367W	YOR361C	913
YLR367W	YOR369C	943
YLR367W	YPL079W	962
YLR367W	YPL081W	950
YLR367W	YPL131W	989
YLR367W	YPL143W	977
YLR367W	YPL198W	930
YLR367W	YPL220W	940
YLR367W	YPL237W	925
YLR367W	YPL249C-A	998
YLR367W	YPR043W	928
YLR367W	YPR102C	995
YLR367W	YPR132W	947
YLR367W	YPR163C	910
YLR368W	YBR179C	989
YLR368W	YDL132W	982
YLR368W	YDR219C	930
YLR368W	YDR328C	975
YLR368W	YLL001W	927
YLR368W	YMR035W	981
YLR369W	YCL017C	972
YLR369W	YDL120W	978
YLR369W	YGL018C	996
YLR369W	YIL022W	969
YLR369W	YJR045C	978
YLR369W	YJR104C	987
YLR369W	YKL040C	968
YLR369W	YOR232W	999
YLR369W	YPL059W	939
YLR369W	YPL135W	989
YLR370C	YBR234C	999
YLR370C	YDL029W	999
YLR370C	YIL062C	999
YLR370C	YJR043C	917
YLR370C	YJR065C	999
YLR370C	YJR091C	933
YLR370C	YKL013C	999
YLR370C	YLR429W	964
YLR370C	YMR109W	908
YLR370C	YNR035C	999
YLR370C	YOR181W	934
YLR371W	YBL105C	947
YLR371W	YBR011C	999
YLR371W	YDL203C	999

YLR371W	YDR208W	932
YLR371W	YDR389W	974
YLR371W	YER155C	994
YLR371W	YGR229C	983
YLR371W	YIL002C	990
YLR371W	YJL095W	951
YLR371W	YKL203C	988
YLR371W	YLR229C	962
YLR371W	YLR332W	981
YLR371W	YLR342W	994
YLR371W	YMR198W	925
YLR371W	YNL090W	983
YLR371W	YNL127W	910
YLR371W	YOL109W	906
YLR371W	YOR008C	997
YLR371W	YPL161C	912
YLR371W	YPR141C	919
YLR371W	YPR165W	999
YLR372W	YCR009C	986
YLR372W	YCR034W	999
YLR372W	YDL015C	990
YLR372W	YDR388W	915
YLR372W	YKL006W	942
YLR372W	YLR228C	905
YLR372W	YML008C	904
YLR372W	YML106W	901
YLR372W	YMR202W	998
YLR373C	YBR098W	927
YLR373C	YDR386W	929
YLR373C	YPL128C	998
YLR376C	YDR078C	999
YLR376C	YHL006C	989
YLR376C	YIL132C	994
YLR376C	YLR234W	910
YLR376C	YMR190C	977
YLR377C	YAL038W	927
YLR377C	YBR117C	924
YLR377C	YBR196C	979
YLR377C	YBR218C	920
YLR377C	YDR028C	942
YLR377C	YFR053C	916
YLR377C	YGL062W	924
YLR377C	YGL253W	951
YLR377C	YGR240C	955

YLR377C	YIL107C	978
YLR377C	YJL155C	966
YLR377C	YKL060C	985
YLR377C	YKL085W	901
YLR377C	YKR097W	953
YLR377C	YMR205C	928
YLR377C	YOL126C	955
YLR377C	YOL136C	971
YLR377C	YPR074C	927
YLR378C	YBR171W	997
YLR378C	YBR283C	986
YLR378C	YDL232W	981
YLR378C	YDR086C	999
YLR378C	YEL002C	981
YLR378C	YER019C-A	991
YLR378C	YER087C-B	999
YLR378C	YGL022W	986
YLR378C	YGL226C-A	996
YLR378C	YJL002C	984
YLR378C	YJL034W	974
YLR378C	YLR207W	952
YLR378C	YLR292C	908
YLR378C	YML019W	998
YLR378C	YMR149W	986
YLR378C	YMR297W	913
YLR378C	YOL013C	993
YLR378C	YOR085W	989
YLR378C	YOR103C	984
YLR378C	YOR254C	999
YLR378C	YPL094C	999
YLR380W	YMR079W	999
YLR381W	YBR107C	999
YLR381W	YBR211C	999
YLR381W	YDR254W	999
YLR381W	YDR318W	999
YLR381W	YEL061C	939
YLR381W	YER016W	949
YLR381W	YGL060W	981
YLR381W	YGL086W	967
YLR381W	YGR140W	981
YLR381W	YGR179C	999
YLR381W	YGR188C	919
YLR381W	YIR010W	900
YLR381W	YJL030W	998

YLR381W	YJR135C	999
YLR381W	YKL049C	921
YLR381W	YKL089W	964
YLR381W	YKR037C	983
YLR381W	YMR094W	981
YLR381W	YMR117C	983
YLR381W	YMR168C	964
YLR381W	YPL008W	922
YLR381W	YPL018W	999
YLR381W	YPL233W	906
YLR381W	YPR046W	999
YLR382C	Q0120	992
YLR382C	YPL160W	929
YLR383W	YEL019C	938
YLR383W	YLR007W	957
YLR383W	YLR435W	998
YLR383W	YML032C	900
YLR384C	YBL071W-A	987
YLR384C	YDL047W	970
YLR384C	YDL074C	942
YLR384C	YDL140C	960
YLR384C	YGR200C	999
YLR384C	YGR229C	918
YLR384C	YGR252W	944
YLR384C	YHR187W	999
YLR384C	YKL110C	996
YLR384C	YMR312W	999
YLR384C	YNL272C	990
YLR384C	YPL086C	999
YLR384C	YPL101W	999
YLR385C	YAL011W	954
YLR385C	YBR231C	999
YLR385C	YDR190C	998
YLR385C	YDR334W	999
YLR385C	YDR485C	994
YLR385C	YFL039C	961
YLR385C	YGR002C	999
YLR385C	YJL081C	976
YLR385C	YLR085C	978
YLR385C	YLR399C	998
YLR385C	YML041C	993
YLR385C	YNL107W	999
YLR385C	YPL235W	954
YLR386W	YBR143C	921

YLR386W	YDR427W	998
YLR386W	YFR019W	990
YLR386W	YFR021W	970
YLR386W	YGR061C	955
YLR386W	YJL168C	952
YLR386W	YNL325C	999
YLR387C	YBR267W	959
YLR388W	YAL003W	954
YLR388W	YAL035W	923
YLR388W	YBL027W	954
YLR388W	YBL072C	902
YLR388W	YBL087C	966
YLR388W	YBL092W	982
YLR388W	YBR031W	976
YLR388W	YBR048W	975
YLR388W	YBR079C	919
YLR388W	YBR084C-A	990
YLR388W	YBR189W	995
YLR388W	YBR191W	999
YLR388W	YCR031C	997
YLR388W	YDL061C	999
YLR388W	YDL075W	999
YLR388W	YDL081C	911
YLR388W	YDL082W	918
YLR388W	YDL130W	957
YLR388W	YDL136W	977
YLR388W	YDL191W	907
YLR388W	YDL192W	940
YLR388W	YDR012W	990
YLR388W	YDR025W	964
YLR388W	YDR064W	999
YLR388W	YDR382W	999
YLR388W	YDR418W	996
YLR388W	YDR447C	911
YLR388W	YDR450W	907
YLR388W	YDR471W	987
YLR388W	YDR500C	997
YLR388W	YEL054C	922
YLR388W	YER025W	961
YLR388W	YER056C-A	999
YLR388W	YER074W	911
YLR388W	YER102W	993
YLR388W	YER117W	998
YLR388W	YER131W	978

YLR388W	YER165W	938
YLR388W	YFL034C-A	912
YLR388W	YFR031C-A	948
YLR388W	YFR032C-A	910
YLR388W	YGL030W	999
YLR388W	YGL031C	953
YLR388W	YGL076C	959
YLR388W	YGL103W	999
YLR388W	YGL123W	999
YLR388W	YGL135W	911
YLR388W	YGL147C	963
YLR388W	YGL189C	912
YLR388W	YGR027C	999
YLR388W	YGR034W	969
YLR388W	YGR085C	997
YLR388W	YGR118W	999
YLR388W	YGR148C	997
YLR388W	YGR162W	910
YLR388W	YGR214W	991
YLR388W	YHL001W	999
YLR388W	YHL015W	991
YLR388W	YHL033C	999
YLR388W	YHR010W	990
YLR388W	YHR021C	913
YLR388W	YHR141C	914
YLR388W	YHR203C	997
YLR388W	YIL018W	948
YLR388W	YIL052C	999
YLR388W	YIL069C	911
YLR388W	YIL133C	913
YLR388W	YIL148W	995
YLR388W	YJL136C	977
YLR388W	YJL177W	998
YLR388W	YJL189W	962
YLR388W	YJL190C	970
YLR388W	YJL191W	996
YLR388W	YJR094W-A	999
YLR388W	YJR123W	999
YLR388W	YJR145C	970
YLR388W	YKL006W	999
YLR388W	YKL156W	999
YLR388W	YKL180W	999
YLR388W	YKR057W	916
YLR388W	YKR059W	975

YLR388W	YLL045C	930
YLR388W	YLR029C	980
YLR388W	YLR048W	999
YLR388W	YLR060W	965
YLR388W	YLR061W	999
YLR388W	YLR075W	996
YLR388W	YLR167W	993
YLR388W	YLR185W	994
YLR388W	YLR264W	999
YLR388W	YLR287C-A	900
YLR388W	YLR325C	912
YLR388W	YLR333C	928
YLR388W	YLR340W	996
YLR388W	YLR344W	999
YLR388W	YLR367W	996
YLR388W	YLR406C	903
YLR388W	YLR441C	917
YLR388W	YLR448W	997
YLR388W	YML024W	999
YLR388W	YML026C	907
YLR388W	YML063W	992
YLR388W	YML073C	908
YLR388W	YMR121C	911
YLR388W	YMR142C	928
YLR388W	YMR146C	900
YLR388W	YMR194W	999
YLR388W	YMR230W	953
YLR388W	YMR242C	911
YLR388W	YMR260C	904
YLR388W	YNL067W	997
YLR388W	YNL069C	913
YLR388W	YNL096C	916
YLR388W	YNL162W	914
YLR388W	YNL178W	999
YLR388W	YNL209W	978
YLR388W	YNL301C	982
YLR388W	YNL302C	912
YLR388W	YNR037C	917
YLR388W	YOL039W	930
YLR388W	YOL040C	978
YLR388W	YOL121C	912
YLR388W	YOL127W	999
YLR388W	YOR063W	998
YLR388W	YOR096W	995

YLR388W	YOR133W	932
YLR388W	YOR167C	971
YLR388W	YOR182C	900
YLR388W	YOR234C	996
YLR388W	YOR293W	981
YLR388W	YOR312C	929
YLR388W	YOR361C	914
YLR388W	YOR369C	925
YLR388W	YPL037C	999
YLR388W	YPL079W	976
YLR388W	YPL081W	944
YLR388W	YPL131W	998
YLR388W	YPL143W	994
YLR388W	YPL198W	920
YLR388W	YPL220W	991
YLR388W	YPL237W	924
YLR388W	YPL249C-A	958
YLR388W	YPR043W	967
YLR388W	YPR102C	999
YLR388W	YPR132W	994
YLR388W	YPR163C	911
YLR389C	YPR122W	999
YLR392C	YER125W	971
YLR393W	Q0085	986
YLR393W	YPL240C	919
YLR394W	YDL154W	934
YLR394W	YDR285W	914
YLR394W	YFL003C	924
YLR395C	Q0045	995
YLR395C	Q0250	978
YLR395C	Q0275	978
YLR395C	YDL067C	978
YLR395C	YGL187C	982
YLR395C	YGL191W	981
YLR395C	YHR001W-A	938
YLR395C	YHR051W	982
YLR395C	YIL111W	978
YLR395C	YLR038C	985
YLR395C	YMR256C	986
YLR395C	YNL052W	981
YLR396C	YAL002W	999
YLR396C	YDL077C	999
YLR396C	YDR080W	999
YLR396C	YDR164C	905

YLR396C	YDR229W	987
YLR396C	YDR323C	946
YLR396C	YDR495C	986
YLR396C	YGL212W	999
YLR396C	YLR093C	981
YLR396C	YLR148W	999
YLR396C	YML001W	990
YLR396C	YMR197C	980
YLR396C	YMR231W	999
YLR396C	YOR036W	935
YLR396C	YOR089C	911
YLR396C	YOR106W	999
YLR396C	YPL045W	999
YLR397C	YDR101C	940
YLR398C	YBR142W	966
YLR398C	YDR060W	984
YLR398C	YDR280W	967
YLR398C	YGL173C	999
YLR398C	YGL213C	999
YLR398C	YGL246C	927
YLR398C	YGR195W	969
YLR398C	YHL033C	974
YLR398C	YKL023W	993
YLR398C	YNL232W	933
YLR398C	YOR048C	969
YLR398C	YOR063W	921
YLR398C	YOR076C	993
YLR398C	YPL240C	919
YLR398C	YPR189W	999
YLR399C	YBR009C	999
YLR399C	YBR010W	991
YLR399C	YBR231C	997
YLR399C	YDL070W	999
YLR399C	YDR190C	970
YLR399C	YDR334W	996
YLR399C	YER148W	930
YLR399C	YGR002C	994
YLR399C	YJL081C	984
YLR399C	YJL115W	932
YLR399C	YLR385C	998
YLR399C	YMR227C	997
YLR399C	YNL030W	972
YLR399C	YNL107W	999
YLR399C	YOL012C	998

YLR399C	YOR244W	975
YLR399C	YPL235W	970
YLR403W	YER103W	913
YLR403W	YKL062W	929
YLR403W	YOR370C	967
YLR404W	YPL240C	902
YLR406C	YBL027W	919
YLR406C	YBL072C	919
YLR406C	YBL087C	915
YLR406C	YBL092W	937
YLR406C	YBR031W	931
YLR406C	YBR048W	900
YLR406C	YBR084C-A	919
YLR406C	YBR118W	905
YLR406C	YBR181C	900
YLR406C	YBR189W	904
YLR406C	YBR191W	921
YLR406C	YCR031C	925
YLR406C	YDL061C	903
YLR406C	YDL075W	908
YLR406C	YDL081C	937
YLR406C	YDL082W	915
YLR406C	YDL130W	907
YLR406C	YDL136W	997
YLR406C	YDL191W	913
YLR406C	YDR012W	931
YLR406C	YDR025W	900
YLR406C	YDR064W	940
YLR406C	YDR382W	938
YLR406C	YDR385W	909
YLR406C	YDR418W	924
YLR406C	YDR447C	923
YLR406C	YDR450W	920
YLR406C	YDR471W	997
YLR406C	YDR500C	910
YLR406C	YEL054C	924
YLR406C	YER006W	974
YLR406C	YER056C-A	910
YLR406C	YER074W	902
YLR406C	YER102W	919
YLR406C	YER117W	915
YLR406C	YER131W	909
YLR406C	YFL034C-A	918
YLR406C	YFR031C-A	912

YLR406C	YFR032C-A	935
YLR406C	YGL030W	930
YLR406C	YGL076C	926
YLR406C	YGL103W	997
YLR406C	YGL135W	925
YLR406C	YGL147C	997
YLR406C	YGL189C	909
YLR406C	YGR027C	906
YLR406C	YGR034W	927
YLR406C	YGR085C	917
YLR406C	YGR118W	921
YLR406C	YGR214W	911
YLR406C	YHL001W	918
YLR406C	YHL015W	932
YLR406C	YHL033C	929
YLR406C	YHR010W	913
YLR406C	YHR021C	922
YLR406C	YHR141C	998
YLR406C	YHR203C	929
YLR406C	YIL018W	912
YLR406C	YIL052C	910
YLR406C	YIL069C	902
YLR406C	YIL133C	928
YLR406C	YIL148W	907
YLR406C	YJL136C	927
YLR406C	YJL177W	919
YLR406C	YJL189W	962
YLR406C	YJL190C	912
YLR406C	YJL191W	925
YLR406C	YJR094W-A	923
YLR406C	YJR123W	915
YLR406C	YJR145C	929
YLR406C	YKL006W	918
YLR406C	YKL009W	976
YLR406C	YKL156W	922
YLR406C	YKL180W	997
YLR406C	YKR057W	927
YLR406C	YKR094C	907
YLR406C	YLL045C	929
YLR406C	YLR029C	910
YLR406C	YLR048W	911
YLR406C	YLR061W	922
YLR406C	YLR075W	902
YLR406C	YLR167W	901

YLR406C	YLR185W	910
YLR406C	YLR264W	914
YLR406C	YLR287C-A	909
YLR406C	YLR333C	905
YLR406C	YLR340W	934
YLR406C	YLR344W	927
YLR406C	YLR367W	912
YLR406C	YLR388W	903
YLR406C	YLR441C	997
YLR406C	YLR448W	921
YLR406C	YML024W	923
YLR406C	YML026C	920
YLR406C	YML063W	921
YLR406C	YML073C	919
YLR406C	YMR121C	910
YLR406C	YMR142C	914
YLR406C	YMR194W	921
YLR406C	YMR230W	923
YLR406C	YMR242C	923
YLR406C	YMR260C	917
YLR406C	YNL067W	916
YLR406C	YNL069C	927
YLR406C	YNL096C	923
YLR406C	YNL162W	934
YLR406C	YNL178W	937
YLR406C	YNL301C	914
YLR406C	YNL302C	916
YLR406C	YOL039W	911
YLR406C	YOL040C	936
YLR406C	YOL120C	914
YLR406C	YOL121C	917
YLR406C	YOL127W	997
YLR406C	YOR063W	997
YLR406C	YOR096W	924
YLR406C	YOR133W	909
YLR406C	YOR167C	913
YLR406C	YOR182C	909
YLR406C	YOR234C	921
YLR406C	YOR293W	924
YLR406C	YOR312C	923
YLR406C	YOR369C	910
YLR406C	YPL079W	921
YLR406C	YPL081W	904
YLR406C	YPL090C	900

YLR406C	YPL131W	906
YLR406C	YPL143W	921
YLR406C	YPL198W	926
YLR406C	YPL220W	925
YLR406C	YPL249C-A	922
YLR406C	YPR043W	923
YLR406C	YPR080W	905
YLR406C	YPR102C	917
YLR406C	YPR132W	921
YLR407W	YOR061W	995
YLR409C	YBL004W	998
YLR409C	YBR247C	978
YLR409C	YCL059C	999
YLR409C	YCR057C	999
YLR409C	YDL014W	997
YLR409C	YDL060W	916
YLR409C	YDL148C	913
YLR409C	YDL153C	923
YLR409C	YDR299W	932
YLR409C	YDR324C	999
YLR409C	YDR398W	914
YLR409C	YDR449C	999
YLR409C	YER082C	999
YLR409C	YGL120C	923
YLR409C	YGL171W	937
YLR409C	YGR090W	999
YLR409C	YGR128C	991
YLR409C	YGR145W	997
YLR409C	YHR148W	997
YLR409C	YHR169W	947
YLR409C	YHR196W	910
YLR409C	YJL010C	985
YLR409C	YJL069C	999
YLR409C	YJL109C	999
YLR409C	YJR002W	999
YLR409C	YKR060W	997
YLR409C	YLL011W	998
YLR409C	YLR003C	919
YLR409C	YLR129W	999
YLR409C	YLR175W	921
YLR409C	YLR186W	998
YLR409C	YLR197W	998
YLR409C	YLR222C	999
YLR409C	YML093W	919

YLR409C	YMR093W	999
YLR409C	YMR229C	937
YLR409C	YMR290C	937
YLR409C	YNL075W	949
YLR409C	YNL132W	998
YLR409C	YOL010W	923
YLR409C	YOR004W	926
YLR409C	YOR027W	940
YLR409C	YOR078W	971
YLR409C	YOR145C	923
YLR409C	YOR310C	957
YLR409C	YPL012W	915
YLR409C	YPL126W	998
YLR409C	YPL217C	998
YLR409C	YPR112C	933
YLR409C	YPR137W	997
YLR409C	YPR144C	998
YLR410W	YDR017C	977
YLR410W	YDR315C	944
YLR410W	YGR017W	994
YLR410W	YGR159C	931
YLR410W	YKR048C	972
YLR410W	YLR175W	925
YLR410W	YLR196W	940
YLR410W	YML069W	964
YLR411W	YBR126C	906
YLR411W	YMR021C	959
YLR411W	YPR124W	997
YLR417W	YCL008C	957
YLR417W	YHL027W	935
YLR417W	YJR102C	999
YLR417W	YLR119W	932
YLR417W	YMR077C	999
YLR417W	YPL002C	999
YLR417W	YPL065W	999
YLR417W	YPL240C	919
YLR418C	YAL011W	939
YLR418C	YAL021C	992
YLR418C	YBR221C	927
YLR418C	YBR231C	942
YLR418C	YBR253W	970
YLR418C	YBR279W	999
YLR418C	YCR084C	961
YLR418C	YDL084W	916

YLR418C	YDL140C	999
YLR418C	YDR138W	966
YLR418C	YDR167W	965
YLR418C	YDR308C	970
YLR418C	YDR334W	963
YLR418C	YDR485C	946
YLR418C	YEL061C	932
YLR418C	YER022W	970
YLR418C	YER111C	969
YLR418C	YER139C	970
YLR418C	YER148W	998
YLR418C	YER161C	980
YLR418C	YGL019W	991
YLR418C	YGL025C	915
YLR418C	YGL043W	908
YLR418C	YGL127C	938
YLR418C	YGL207W	999
YLR418C	YGL244W	999
YLR418C	YGR005C	998
YLR418C	YGR104C	998
YLR418C	YGR186W	946
YLR418C	YHR041C	970
YLR418C	YIL035C	995
YLR418C	YJL140W	914
YLR418C	YJL168C	996
YLR418C	YKL139W	994
YLR418C	YKL160W	968
YLR418C	YKL213C	981
YLR418C	YLR085C	940
YLR418C	YML010W	970
YLR418C	YML041C	948
YLR418C	YML069W	998
YLR418C	YNR010W	912
YLR418C	YOL012C	993
YLR418C	YOL051W	999
YLR418C	YOL145C	999
YLR418C	YOR038C	906
YLR418C	YOR039W	991
YLR418C	YOR061W	995
YLR418C	YOR123C	999
YLR418C	YOR151C	981
YLR418C	YOR308C	913
YLR418C	YPL042C	960
YLR418C	YPL129W	985

YLR418C	YPR070W	983
YLR418C	YPR086W	961
YLR420W	YEL021W	928
YLR420W	YJL130C	986
YLR420W	YKL216W	998
YLR420W	YLR014C	903
YLR421C	YCR014C	970
YLR421C	YDL007W	999
YLR421C	YDL013W	970
YLR421C	YDL097C	999
YLR421C	YDL147W	999
YLR421C	YDR363W-A	999
YLR421C	YDR394W	999
YLR421C	YDR427W	999
YLR421C	YER012W	999
YLR421C	YER021W	992
YLR421C	YFL007W	970
YLR421C	YFR004W	998
YLR421C	YFR010W	992
YLR421C	YFR052W	999
YLR421C	YGL004C	907
YLR421C	YGL048C	999
YLR421C	YGL141W	954
YLR421C	YGR232W	999
YLR421C	YHL030W	999
YLR421C	YHR027C	999
YLR421C	YHR200W	999
YLR421C	YIL075C	999
YLR421C	YKL145W	998
YLR421C	YMR191W	987
YLR421C	YOR117W	999
YLR421C	YOR259C	999
YLR421C	YOR261C	999
YLR421C	YPR108W	997
YLR423C	YDL113C	903
YLR423C	YDR022C	999
YLR423C	YDR320C	980
YLR423C	YER092W	992
YLR423C	YGL180W	999
YLR423C	YGR113W	939
YLR423C	YGR120C	970
YLR423C	YHR197W	906
YLR423C	YJL036W	993
YLR423C	YJL061W	988

YLR423C	YMR025W	984
YLR423C	YPL166W	999
YLR423C	YPR049C	999
YLR423C	YPR185W	999
YLR424W	YBR055C	976
YLR424W	YDL030W	974
YLR424W	YDL209C	992
YLR424W	YDR416W	994
YLR424W	YER029C	990
YLR424W	YER172C	993
YLR424W	YGL090W	974
YLR424W	YGL120C	999
YLR424W	YGL128C	999
YLR424W	YGR091W	974
YLR424W	YHR165C	999
YLR424W	YKL095W	940
YLR424W	YKL173W	994
YLR424W	YKR022C	999
YLR424W	YLL036C	981
YLR424W	YLR117C	999
YLR424W	YLR265C	910
YLR424W	YML049C	976
YLR424W	YMR125W	976
YLR424W	YPL151C	993
YLR424W	YPL213W	974
YLR424W	YPR082C	974
YLR424W	YPR101W	995
YLR425W	YBR011C	999
YLR425W	YBR160W	928
YLR425W	YDR135C	931
YLR425W	YPL066W	999
YLR425W	YPR165W	939
YLR429W	YBR109C	983
YLR429W	YBR143C	904
YLR429W	YBR234C	981
YLR429W	YCR009C	985
YLR429W	YCR088W	994
YLR429W	YDL029W	994
YLR429W	YDR129C	985
YLR429W	YFL039C	998
YLR429W	YGL106W	986
YLR429W	YGR086C	983
YLR429W	YIL062C	979
YLR429W	YJR065C	978

YLR429W	YKL007W	974
YLR429W	YKL013C	955
YLR429W	YLL050C	918
YLR429W	YLR370C	964
YLR429W	YMR109W	970
YLR429W	YNL106C	985
YLR429W	YNR035C	997
YLR429W	YPR171W	966
YLR430W	YDL140C	968
YLR430W	YDL175C	996
YLR430W	YDR349C	952
YLR430W	YER133W	978
YLR430W	YNL251C	999
YLR430W	YPL190C	998
YLR431C	YDL149W	999
YLR431C	YGL180W	946
YLR431C	YJL178C	991
YLR431C	YPR185W	939
YLR432W	YAR073W	980
YLR432W	YCL011C	985
YLR432W	YDR064W	982
YLR432W	YGL076C	979
YLR432W	YGR085C	978
YLR432W	YHR203C	976
YLR432W	YHR216W	999
YLR432W	YJL050W	940
YLR432W	YLL045C	979
YLR432W	YLR028C	902
YLR432W	YLR029C	978
YLR432W	YML056C	999
YLR432W	YML073C	978
YLR432W	YMR120C	910
YLR432W	YMR217W	989
YLR432W	YMR290C	978
YLR432W	YNL178W	980
YLR432W	YNL220W	934
YLR432W	YPL131W	978
YLR432W	YPL198W	978
YLR433C	YBR109C	999
YLR433C	YDL128W	928
YLR433C	YIL105C	976
YLR433C	YKL190W	999
YLR433C	YLR342W	998
YLR433C	YML057W	999

YLR433C	YNL027W	933
YLR433C	YNL047C	936
YLR433C	YNL135C	914
YLR433C	YOR324C	935
YLR435W	YER131W	997
YLR435W	YGL189C	983
YLR435W	YLR383W	998
YLR436C	YMR304W	999
YLR438C-A	YBL026W	999
YLR438C-A	YBR055C	981
YLR438C-A	YCR020C-A	984
YLR438C-A	YCR077C	999
YLR438C-A	YDR334W	949
YLR438C-A	YDR378C	999
YLR438C-A	YDR473C	999
YLR438C-A	YER029C	994
YLR438C-A	YER112W	999
YLR438C-A	YER146W	999
YLR438C-A	YER172C	998
YLR438C-A	YGL173C	999
YLR438C-A	YGR074W	903
YLR438C-A	YGR091W	954
YLR438C-A	YHR165C	935
YLR438C-A	YJL124C	999
YLR438C-A	YJR022W	999
YLR438C-A	YKL173W	997
YLR438C-A	YKL188C	931
YLR438C-A	YLR147C	965
YLR438C-A	YMR268C	999
YLR438C-A	YNL147W	999
YLR438C-A	YOL149W	971
YLR438C-A	YOR159C	989
YLR438C-A	YPR178W	997
YLR438C-A	YPR182W	988
YLR438W	YGR010W	991
YLR438W	YHR037W	947
YLR438W	YJL088W	996
YLR438W	YLR328W	991
YLR438W	YOL058W	943
YLR438W	YOL140W	913
YLR438W	YPL111W	999
YLR438W	YPL240C	902
YLR439W	YBL038W	998
YLR439W	YBR122C	974

YLR439W	YBR282W	970
YLR439W	YCR046C	993
YLR439W	YCR071C	989
YLR439W	YDR116C	988
YLR439W	YDR237W	974
YLR439W	YDR296W	993
YLR439W	YDR322W	974
YLR439W	YDR405W	987
YLR439W	YDR462W	972
YLR439W	YGR220C	999
YLR439W	YHR147C	970
YLR439W	YJL063C	978
YLR439W	YKL167C	974
YLR439W	YKR006C	974
YLR439W	YKR085C	974
YLR439W	YLR312W-A	988
YLR439W	YML025C	993
YLR439W	YMR024W	988
YLR439W	YMR193W	974
YLR439W	YNL005C	974
YLR439W	YNL185C	937
YLR439W	YNL252C	974
YLR439W	YNL284C	998
YLR439W	YOR150W	972
YLR440C	YDR498C	999
YLR440C	YGL098W	999
YLR440C	YGL145W	999
YLR440C	YKL196C	999
YLR440C	YNL258C	999
YLR440C	YOR075W	999
YLR441C	YBL027W	996
YLR441C	YBL072C	997
YLR441C	YBL087C	970
YLR441C	YBL092W	996
YLR441C	YBR031W	997
YLR441C	YBR048W	996
YLR441C	YBR084C-A	925
YLR441C	YBR118W	916
YLR441C	YBR181C	998
YLR441C	YBR189W	997
YLR441C	YBR191W	996
YLR441C	YCR031C	996
YLR441C	YDL061C	916
YLR441C	YDL075W	921

YLR441C	YDL081C	938
YLR441C	YDL082W	997
YLR441C	YDL083C	964
YLR441C	YDL130W	906
YLR441C	YDL136W	995
YLR441C	YDL191W	921
YLR441C	YDR012W	996
YLR441C	YDR025W	996
YLR441C	YDR064W	999
YLR441C	YDR382W	996
YLR441C	YDR385W	919
YLR441C	YDR418W	996
YLR441C	YDR429C	904
YLR441C	YDR447C	998
YLR441C	YDR450W	918
YLR441C	YDR471W	995
YLR441C	YDR500C	911
YLR441C	YEL054C	928
YLR441C	YER025W	901
YLR441C	YER056C-A	905
YLR441C	YER074W	995
YLR441C	YER102W	995
YLR441C	YER110C	978
YLR441C	YER117W	932
YLR441C	YER131W	927
YLR441C	YER165W	907
YLR441C	YFL034C-A	918
YLR441C	YFR031C-A	997
YLR441C	YFR032C-A	927
YLR441C	YGL030W	997
YLR441C	YGL031C	996
YLR441C	YGL076C	997
YLR441C	YGL103W	995
YLR441C	YGL123W	998
YLR441C	YGL135W	996
YLR441C	YGL147C	977
YLR441C	YGL189C	919
YLR441C	YGR027C	928
YLR441C	YGR034W	956
YLR441C	YGR085C	996
YLR441C	YGR118W	998
YLR441C	YGR148C	925
YLR441C	YGR162W	913
YLR441C	YGR214W	943

YLR441C	YHL001W	995
YLR441C	YHL015W	915
YLR441C	YHL033C	995
YLR441C	YHR010W	905
YLR441C	YHR021C	928
YLR441C	YHR141C	997
YLR441C	YHR203C	996
YLR441C	YIL018W	996
YLR441C	YIL052C	905
YLR441C	YIL069C	918
YLR441C	YIL133C	998
YLR441C	YIL148W	908
YLR441C	YJL136C	932
YLR441C	YJL177W	995
YLR441C	YJL189W	921
YLR441C	YJL190C	998
YLR441C	YJL191W	923
YLR441C	YJR094W-A	926
YLR441C	YJR123W	997
YLR441C	YJR145C	995
YLR441C	YKL006W	923
YLR441C	YKL156W	928
YLR441C	YKL180W	995
YLR441C	YKR057W	932
YLR441C	YKR094C	908
YLR441C	YLL045C	997
YLR441C	YLR029C	937
YLR441C	YLR048W	936
YLR441C	YLR061W	921
YLR441C	YLR075W	996
YLR441C	YLR167W	902
YLR441C	YLR185W	911
YLR441C	YLR264W	909
YLR441C	YLR287C-A	994
YLR441C	YLR325C	956
YLR441C	YLR333C	931
YLR441C	YLR340W	997
YLR441C	YLR344W	915
YLR441C	YLR367W	915
YLR441C	YLR388W	917
YLR441C	YLR406C	997
YLR441C	YLR448W	996
YLR441C	YML024W	934
YLR441C	YML026C	918

YLR441C	YML063W	995
YLR441C	YML073C	997
YLR441C	YMR121C	946
YLR441C	YMR142C	938
YLR441C	YMR194W	996
YLR441C	YMR230W	929
YLR441C	YMR242C	999
YLR441C	YMR260C	943
YLR441C	YNL067W	930
YLR441C	YNL069C	996
YLR441C	YNL096C	996
YLR441C	YNL162W	930
YLR441C	YNL178W	997
YLR441C	YNL301C	996
YLR441C	YNL302C	926
YLR441C	YOL039W	907
YLR441C	YOL040C	997
YLR441C	YOL120C	924
YLR441C	YOL121C	955
YLR441C	YOL127W	997
YLR441C	YOL139C	923
YLR441C	YOR063W	996
YLR441C	YOR096W	997
YLR441C	YOR133W	919
YLR441C	YOR167C	909
YLR441C	YOR182C	901
YLR441C	YOR234C	931
YLR441C	YOR293W	930
YLR441C	YOR312C	996
YLR441C	YOR369C	972
YLR441C	YPL079W	940
YLR441C	YPL081W	921
YLR441C	YPL090C	994
YLR441C	YPL131W	958
YLR441C	YPL143W	931
YLR441C	YPL198W	997
YLR441C	YPL220W	995
YLR441C	YPL237W	900
YLR441C	YPL249C-A	932
YLR441C	YPR043W	926
YLR441C	YPR080W	916
YLR441C	YPR102C	939
YLR441C	YPR132W	934
YLR442C	YBR009C	999

YLR442C	YBR010W	999
YLR442C	YBR103W	922
YLR442C	YDL042C	999
YLR442C	YDR143C	985
YLR442C	YDR224C	968
YLR442C	YDR225W	968
YLR442C	YDR227W	999
YLR442C	YDR310C	999
YLR442C	YDR440W	969
YLR442C	YGR252W	911
YLR442C	YHR030C	995
YLR442C	YHR119W	981
YLR442C	YHR154W	975
YLR442C	YHR164C	905
YLR442C	YIL150C	995
YLR442C	YJR052W	998
YLR442C	YKR101W	999
YLR442C	YML032C	984
YLR442C	YML109W	981
YLR442C	YNL030W	999
YLR442C	YNL031C	999
YLR442C	YNL216W	999
YLR447C	YAL026C	984
YLR447C	YBR127C	987
YLR447C	YDL185W	984
YLR447C	YEL027W	946
YLR447C	YEL051W	998
YLR447C	YGR020C	999
YLR447C	YGR105W	981
YLR447C	YHR026W	951
YLR447C	YHR039C-A	991
YLR447C	YHR060W	979
YLR447C	YKL080W	973
YLR447C	YKL119C	994
YLR447C	YMR054W	999
YLR447C	YOR270C	999
YLR447C	YOR332W	950
YLR447C	YPL234C	956
YLR447C	YPR036W	966
YLR448W	YAL003W	964
YLR448W	YAL035W	931
YLR448W	YBL027W	996
YLR448W	YBL072C	995
YLR448W	YBL087C	933

YLR448W	YBL092W	997
YLR448W	YBR031W	999
YLR448W	YBR048W	997
YLR448W	YBR084C-A	977
YLR448W	YBR118W	911
YLR448W	YBR181C	998
YLR448W	YBR189W	999
YLR448W	YBR191W	999
YLR448W	YCR031C	999
YLR448W	YDL061C	979
YLR448W	YDL075W	999
YLR448W	YDL081C	936
YLR448W	YDL082W	997
YLR448W	YDL083C	971
YLR448W	YDL130W	943
YLR448W	YDL136W	998
YLR448W	YDL191W	924
YLR448W	YDR012W	999
YLR448W	YDR025W	918
YLR448W	YDR064W	999
YLR448W	YDR195W	983
YLR448W	YDR382W	999
YLR448W	YDR385W	919
YLR448W	YDR418W	999
YLR448W	YDR447C	996
YLR448W	YDR450W	921
YLR448W	YDR471W	999
YLR448W	YDR500C	971
YLR448W	YEL054C	928
YLR448W	YER056C-A	999
YLR448W	YER074W	996
YLR448W	YER102W	998
YLR448W	YER117W	977
YLR448W	YER131W	959
YLR448W	YFL034C-A	914
YLR448W	YFR031C-A	996
YLR448W	YFR032C-A	997
YLR448W	YGL014W	952
YLR448W	YGL030W	999
YLR448W	YGL031C	939
YLR448W	YGL076C	998
YLR448W	YGL103W	999
YLR448W	YGL123W	995
YLR448W	YGL135W	996

YLR448W	YGL147C	996
YLR448W	YGL189C	917
YLR448W	YGR027C	981
YLR448W	YGR034W	996
YLR448W	YGR085C	999
YLR448W	YGR118W	998
YLR448W	YGR148C	996
YLR448W	YGR156W	978
YLR448W	YGR214W	999
YLR448W	YHL001W	999
YLR448W	YHL015W	982
YLR448W	YHL033C	999
YLR448W	YHR010W	969
YLR448W	YHR021C	932
YLR448W	YHR141C	997
YLR448W	YHR203C	999
YLR448W	YIL018W	996
YLR448W	YIL052C	999
YLR448W	YIL069C	921
YLR448W	YIL133C	996
YLR448W	YIL148W	966
YLR448W	YJL136C	978
YLR448W	YJL177W	999
YLR448W	YJL189W	953
YLR448W	YJL190C	996
YLR448W	YJL191W	994
YLR448W	YJR094W-A	993
YLR448W	YJR123W	999
YLR448W	YJR145C	924
YLR448W	YKL006W	999
YLR448W	YKL018W	978
YLR448W	YKL156W	994
YLR448W	YKL180W	999
YLR448W	YKR057W	932
YLR448W	YKR094C	908
YLR448W	YLL045C	996
YLR448W	YLR029C	999
YLR448W	YLR048W	999
YLR448W	YLR060W	972
YLR448W	YLR061W	999
YLR448W	YLR075W	999
YLR448W	YLR150W	960
YLR448W	YLR167W	955
YLR448W	YLR185W	971

YLR448W	YLR197W	939
YLR448W	YLR249W	940
YLR448W	YLR264W	996
YLR448W	YLR277C	976
YLR448W	YLR287C-A	995
YLR448W	YLR325C	916
YLR448W	YLR333C	938
YLR448W	YLR340W	999
YLR448W	YLR344W	998
YLR448W	YLR367W	974
YLR448W	YLR388W	997
YLR448W	YLR406C	921
YLR448W	YLR441C	996
YLR448W	YML024W	999
YLR448W	YML026C	921
YLR448W	YML063W	999
YLR448W	YML073C	995
YLR448W	YMR061W	980
YLR448W	YMR121C	921
YLR448W	YMR142C	998
YLR448W	YMR194W	999
YLR448W	YMR230W	952
YLR448W	YMR242C	997
YLR448W	YMR260C	916
YLR448W	YMR290C	947
YLR448W	YNL067W	995
YLR448W	YNL069C	996
YLR448W	YNL096C	996
YLR448W	YNL132W	954
YLR448W	YNL162W	948
YLR448W	YNL178W	999
YLR448W	YNL209W	938
YLR448W	YNL301C	998
YLR448W	YNL302C	924
YLR448W	YOL039W	938
YLR448W	YOL040C	997
YLR448W	YOL041C	925
YLR448W	YOL120C	928
YLR448W	YOL121C	925
YLR448W	YOL127W	999
YLR448W	YOR063W	999
YLR448W	YOR096W	999
YLR448W	YOR133W	948
YLR448W	YOR167C	942

YLR448W	YOR182C	904
YLR448W	YOR234C	996
YLR448W	YOR293W	980
YLR448W	YOR312C	997
YLR448W	YOR369C	935
YLR448W	YPL037C	981
YLR448W	YPL079W	974
YLR448W	YPL081W	977
YLR448W	YPL090C	995
YLR448W	YPL131W	999
YLR448W	YPL143W	995
YLR448W	YPL198W	997
YLR448W	YPL220W	998
YLR448W	YPL249C-A	998
YLR448W	YPR043W	960
YLR448W	YPR080W	911
YLR448W	YPR102C	985
YLR448W	YPR132W	975
YLR449W	YBR009C	936
YLR449W	YDL031W	980
YLR449W	YDR496C	991
YLR449W	YER006W	993
YLR449W	YFL002C	992
YLR449W	YFR001W	923
YLR449W	YHR052W	982
YLR449W	YHR088W	980
YLR449W	YKR081C	957
YLR449W	YLL034C	993
YLR449W	YLR002C	993
YLR449W	YLR196W	933
YLR449W	YML074C	963
YLR449W	YMR049C	995
YLR449W	YMR242C	934
YLR449W	YMR290C	981
YLR449W	YNL061W	997
YLR449W	YOL077C	987
YLR449W	YOL127W	927
YLR449W	YOR063W	938
YLR449W	YOR206W	998
YLR449W	YPL093W	997
YLR449W	YPL131W	981
YLR449W	YPL146C	994
YLR449W	YPL198W	978
YLR449W	YPL211W	979

YLR449W	YPR169W	958
YLR450W	YDL126C	972
YLR450W	YER017C	978
YLR450W	YGR157W	919
YLR450W	YHR133C	927
YLR450W	YML075C	999
YLR450W	YML126C	936
YLR450W	YMR022W	976
YLR450W	YMR208W	906
YLR451W	YCL018W	985
YLR451W	YGL009C	924
YLR451W	YNL104C	901
YLR451W	YOR375C	930
YLR452C	YBL016W	998
YLR452C	YDR103W	978
YLR452C	YFL026W	999
YLR452C	YGL178W	999
YLR452C	YGR040W	991
YLR452C	YHR005C	999
YLR452C	YHR158C	952
YLR452C	YJR086W	911
YLR452C	YKL178C	974
YLR452C	YOR212W	998
YLR453C	YBL088C	953
YLR453C	YBR234C	944
YLR453C	YBR275C	993
YLR453C	YDR227W	947
YLR453C	YNL216W	998
YLR453C	YPL002C	905
YLR453C	YPL098C	944
YLR457C	YKR048C	970
YLR459W	YDR331W	992
YLR459W	YDR434W	989
YLR459W	YHR188C	934
YLR459W	YLR088W	953
YML001W	YBL050W	957
YML001W	YBR131W	997
YML001W	YDL077C	998
YML001W	YDR080W	941
YML001W	YDR229W	922
YML001W	YER136W	999
YML001W	YGL124C	921
YML001W	YGL212W	969
YML001W	YKL166C	927

YML001W	YLR396C	990
YML001W	YOR070C	991
YML001W	YOR106W	950
YML001W	YPL203W	923
YML001W	YPL240C	970
YML004C	YDR272W	992
YML004C	YHR104W	991
YML004C	YOR040W	989
YML005W	YGL050W	936
YML006C	YDR040C	928
YML006C	YDR477W	998
YML006C	YJR090C	979
YML007W	YBL093C	974
YML007W	YBR081C	997
YML007W	YBR193C	974
YML007W	YBR216C	998
YML007W	YCR081W	974
YML007W	YDR011W	930
YML007W	YDR359C	992
YML007W	YDR423C	996
YML007W	YDR443C	974
YML007W	YDR448W	985
YML007W	YER110C	976
YML007W	YFL024C	991
YML007W	YGL025C	974
YML007W	YGL112C	985
YML007W	YGL151W	992
YML007W	YGR209C	984
YML007W	YGR218W	987
YML007W	YHR041C	992
YML007W	YHR058C	992
YML007W	YHR206W	997
YML007W	YIR037W	992
YML007W	YLR043C	982
YML007W	YLR071C	974
YML007W	YMR112C	974
YML007W	YMR308C	976
YML007W	YNL236W	992
YML007W	YNR010W	974
YML007W	YOL051W	974
YML007W	YOL135C	974
YML007W	YOR174W	974
YML007W	YOR244W	997
YML007W	YPL042C	974

YML007W	YPL091W	924
YML007W	YPR070W	992
YML007W	YPR168W	974
YML007W	YPR199C	994
YML008C	YER044C	999
YML008C	YGL001C	999
YML008C	YGL012W	999
YML008C	YGR060W	999
YML008C	YHR007C	999
YML008C	YHR072W	999
YML008C	YHR190W	999
YML008C	YLR056W	999
YML008C	YLR100W	999
YML008C	YLR372W	904
YML008C	YMR015C	999
YML008C	YMR202W	999
YML008C	YNL280C	999
YML008C	YOR153W	989
YML009C	YMR193W	970
YML009C	YNL252C	972
YML009C	YNL284C	925
YML010W	YAL021C	944
YML010W	YBL008W	922
YML010W	YBL046W	999
YML010W	YBR009C	906
YML010W	YBR154C	999
YML010W	YBR236C	997
YML010W	YBR279W	998
YML010W	YDL108W	982
YML010W	YDL140C	999
YML010W	YDR075W	999
YML010W	YDR404C	999
YML010W	YER164W	908
YML010W	YGL043W	989
YML010W	YGL070C	998
YML010W	YGL130W	977
YML010W	YGL207W	973
YML010W	YGL244W	995
YML010W	YGR005C	999
YML010W	YGR063C	999
YML010W	YGR116W	999
YML010W	YGR186W	990
YML010W	YHR143W-A	961
YML010W	YIL021W	999

YML010W	YIL143C	902
YML010W	YJL140W	999
YML010W	YJR017C	926
YML010W	YKL139W	919
YML010W	YLR418C	970
YML010W	YML069W	945
YML010W	YNL201C	999
YML010W	YOL005C	999
YML010W	YOL145C	965
YML010W	YOR123C	969
YML010W	YOR151C	999
YML010W	YOR224C	998
YML010W	YOR341W	942
YML010W	YPR010C	942
YML010W	YPR052C	989
YML010W	YPR161C	996
YML010W	YPR187W	999
YML011C	YDR314C	931
YML011C	YEL037C	982
YML011C	YER162C	960
YML012W	YAL007C	999
YML012W	YAR002C-A	999
YML012W	YDL018C	989
YML012W	YGL142C	933
YML012W	YGL200C	999
YML012W	YGR284C	906
YML012W	YLR026C	901
YML012W	YLR208W	948
YML012W	YMR307W	902
YML013W	YBR170C	998
YML013W	YBR201W	995
YML013W	YDL126C	999
YML013W	YDR057W	931
YML013W	YDR411C	996
YML013W	YDR454C	960
YML013W	YGR048W	950
YML013W	YIL030C	999
YML013W	YLR207W	960
YML013W	YMR022W	993
YML013W	YOL013C	997
YML013W	YOR067C	907
YML014W	YNR046W	998
YML015C	YBR081C	988
YML015C	YBR198C	999

YML015C	YCR042C	999
YML015C	YDR145W	999
YML015C	YDR167W	999
YML015C	YDR174W	982
YML015C	YDR448W	996
YML015C	YER148W	999
YML015C	YGL066W	988
YML015C	YGL112C	999
YML015C	YGR274C	999
YML015C	YKL058W	998
YML015C	YML098W	999
YML015C	YML114C	998
YML015C	YMR005W	999
YML015C	YMR227C	999
YML015C	YMR236W	999
YML015C	YOL135C	943
YML015C	YOR174W	974
YML015C	YOR194C	984
YML015C	YPL011C	999
YML015C	YPL129W	991
YML016C	YDR040C	984
YML016C	YDR436W	999
YML016C	YER054C	960
YML016C	YFR003C	998
YML016C	YHR030C	996
YML016C	YJL129C	976
YML016C	YKL088W	993
YML016C	YKL193C	996
YML016C	YKR072C	999
YML016C	YMR311C	951
YML016C	YOR054C	947
YML017W	YNL102W	938
YML017W	YPL119C	925
YML019W	YDL232W	999
YML019W	YEL002C	999
YML019W	YGL022W	999
YML019W	YGL226C-A	999
YML019W	YJL002C	999
YML019W	YLR378C	998
YML019W	YMR149W	999
YML019W	YNR030W	921
YML019W	YOR085W	999
YML019W	YOR103C	999
YML021C	YBL019W	986

YML021C	YBR088C	954
YML021C	YBR252W	998
YML021C	YKL114C	996
YML021C	YPL022W	980
YML022W	YDL238C	920
YML022W	YDR226W	905
YML022W	YDR441C	991
YML022W	YDR454C	933
YML022W	YER170W	903
YML022W	YJR105W	940
YML022W	YLR028C	907
YML022W	YLR209C	921
YML022W	YLR359W	922
YML022W	YML035C	916
YML022W	YMR120C	907
YML022W	YMR217W	918
YML024W	YAL035W	924
YML024W	YBL027W	920
YML024W	YBL072C	927
YML024W	YBL087C	928
YML024W	YBL092W	949
YML024W	YBR031W	955
YML024W	YBR048W	934
YML024W	YBR079C	913
YML024W	YBR084C-A	994
YML024W	YBR118W	920
YML024W	YBR181C	903
YML024W	YBR189W	992
YML024W	YBR191W	999
YML024W	YCR031C	994
YML024W	YDL061C	997
YML024W	YDL075W	999
YML024W	YDL081C	939
YML024W	YDL082W	930
YML024W	YDL130W	942
YML024W	YDL136W	986
YML024W	YDL191W	926
YML024W	YDL192W	978
YML024W	YDR012W	968
YML024W	YDR025W	918
YML024W	YDR064W	999
YML024W	YDR382W	997
YML024W	YDR385W	909
YML024W	YDR418W	996

YML024W	YDR429C	916
YML024W	YDR447C	984
YML024W	YDR450W	919
YML024W	YDR471W	977
YML024W	YDR500C	996
YML024W	YEL054C	930
YML024W	YER025W	952
YML024W	YER056C-A	999
YML024W	YER074W	918
YML024W	YER102W	999
YML024W	YER117W	997
YML024W	YER131W	946
YML024W	YER165W	923
YML024W	YFL034C-A	911
YML024W	YFR031C-A	913
YML024W	YFR032C-A	949
YML024W	YGL030W	999
YML024W	YGL031C	938
YML024W	YGL076C	952
YML024W	YGL103W	999
YML024W	YGL123W	994
YML024W	YGL135W	923
YML024W	YGL147C	924
YML024W	YGL189C	921
YML024W	YGR027C	984
YML024W	YGR034W	923
YML024W	YGR085C	978
YML024W	YGR118W	999
YML024W	YGR148C	997
YML024W	YGR214W	992
YML024W	YHL001W	995
YML024W	YHL015W	984
YML024W	YHL033C	999
YML024W	YHR010W	975
YML024W	YHR021C	929
YML024W	YHR141C	935
YML024W	YHR203C	995
YML024W	YIL018W	913
YML024W	YIL052C	999
YML024W	YIL069C	997
YML024W	YIL133C	925
YML024W	YIL148W	997
YML024W	YJL136C	981
YML024W	YJL177W	985

YML024W	YJL189W	957
YML024W	YJL190C	997
YML024W	YJL191W	983
YML024W	YJR094W-A	999
YML024W	YJR123W	999
YML024W	YJR145C	926
YML024W	YKL006W	999
YML024W	YKL156W	996
YML024W	YKL180W	999
YML024W	YKR057W	936
YML024W	YKR059W	976
YML024W	YKR094C	906
YML024W	YLL045C	935
YML024W	YLR029C	974
YML024W	YLR048W	995
YML024W	YLR061W	999
YML024W	YLR075W	999
YML024W	YLR167W	994
YML024W	YLR185W	987
YML024W	YLR264W	997
YML024W	YLR287C-A	902
YML024W	YLR325C	932
YML024W	YLR333C	940
YML024W	YLR340W	995
YML024W	YLR344W	999
YML024W	YLR367W	972
YML024W	YLR388W	999
YML024W	YLR406C	923
YML024W	YLR441C	934
YML024W	YLR448W	999
YML024W	YML026C	919
YML024W	YML063W	999
YML024W	YML073C	930
YML024W	YMR121C	920
YML024W	YMR142C	957
YML024W	YMR194W	999
YML024W	YMR230W	967
YML024W	YMR242C	930
YML024W	YMR260C	905
YML024W	YMR309C	913
YML024W	YNL067W	995
YML024W	YNL069C	924
YML024W	YNL096C	926
YML024W	YNL162W	935

YML024W	YNL178W	999
YML024W	YNL209W	940
YML024W	YNL301C	991
YML024W	YNL302C	928
YML024W	YOL039W	925
YML024W	YOL040C	956
YML024W	YOL120C	922
YML024W	YOL121C	928
YML024W	YOL127W	999
YML024W	YOR063W	980
YML024W	YOR096W	999
YML024W	YOR133W	937
YML024W	YOR167C	949
YML024W	YOR182C	902
YML024W	YOR204W	970
YML024W	YOR234C	993
YML024W	YOR293W	985
YML024W	YOR312C	946
YML024W	YOR361C	915
YML024W	YOR369C	940
YML024W	YPL037C	999
YML024W	YPL079W	981
YML024W	YPL081W	950
YML024W	YPL090C	903
YML024W	YPL131W	984
YML024W	YPL143W	998
YML024W	YPL198W	933
YML024W	YPL220W	996
YML024W	YPL237W	924
YML024W	YPL249C-A	965
YML024W	YPR043W	974
YML024W	YPR080W	910
YML024W	YPR102C	996
YML024W	YPR132W	999
YML025C	YBL038W	999
YML025C	YBR122C	991
YML025C	YCR046C	992
YML025C	YCR071C	972
YML025C	YDR116C	994
YML025C	YDR237W	998
YML025C	YDR296W	992
YML025C	YDR322W	993
YML025C	YDR405W	992
YML025C	YDR462W	972

YML025C	YGR220C	999
YML025C	YHR147C	902
YML025C	YJL041W	912
YML025C	YJL063C	997
YML025C	YKL167C	997
YML025C	YKR006C	997
YML025C	YKR085C	992
YML025C	YLR312W-A	972
YML025C	YLR439W	993
YML025C	YMR024W	992
YML025C	YMR193W	991
YML025C	YNL005C	992
YML025C	YNL177C	982
YML025C	YNL185C	957
YML025C	YNL252C	992
YML026C	YBL027W	921
YML026C	YBL072C	921
YML026C	YBL087C	927
YML026C	YBL092W	944
YML026C	YBR031W	925
YML026C	YBR048W	921
YML026C	YBR084C-A	921
YML026C	YBR118W	907
YML026C	YBR181C	907
YML026C	YBR189W	969
YML026C	YBR191W	921
YML026C	YCR031C	979
YML026C	YDL061C	906
YML026C	YDL075W	920
YML026C	YDL081C	938
YML026C	YDL082W	920
YML026C	YDL130W	902
YML026C	YDL136W	912
YML026C	YDL191W	912
YML026C	YDR012W	925
YML026C	YDR025W	921
YML026C	YDR041W	912
YML026C	YDR064W	998
YML026C	YDR382W	924
YML026C	YDR385W	912
YML026C	YDR418W	913
YML026C	YDR447C	919
YML026C	YDR450W	901
YML026C	YDR471W	903

YML026C	YDR500C	901
YML026C	YEL054C	913
YML026C	YER056C-A	902
YML026C	YER074W	901
YML026C	YER102W	921
YML026C	YER117W	927
YML026C	YER131W	903
YML026C	YER165W	901
YML026C	YFL034C-A	912
YML026C	YFR031C-A	924
YML026C	YFR032C-A	934
YML026C	YGL030W	912
YML026C	YGL076C	920
YML026C	YGL103W	913
YML026C	YGL123W	956
YML026C	YGL135W	918
YML026C	YGL147C	922
YML026C	YGL189C	903
YML026C	YGR027C	915
YML026C	YGR034W	924
YML026C	YGR085C	934
YML026C	YGR118W	927
YML026C	YGR214W	945
YML026C	YHL001W	921
YML026C	YHL015W	941
YML026C	YHL033C	922
YML026C	YHR010W	903
YML026C	YHR021C	918
YML026C	YHR141C	926
YML026C	YHR203C	924
YML026C	YIL018W	924
YML026C	YIL052C	902
YML026C	YIL069C	997
YML026C	YIL133C	998
YML026C	YIL148W	906
YML026C	YJL136C	923
YML026C	YJL177W	918
YML026C	YJL189W	908
YML026C	YJL190C	919
YML026C	YJL191W	979
YML026C	YJR094W-A	920
YML026C	YJR123W	946
YML026C	YJR145C	924
YML026C	YKL006W	920

YML026C	YKL156W	918
YML026C	YKL170W	948
YML026C	YKL180W	918
YML026C	YKR057W	923
YML026C	YKR094C	906
YML026C	YLL045C	922
YML026C	YLR029C	908
YML026C	YLR048W	943
YML026C	YLR061W	914
YML026C	YLR075W	907
YML026C	YLR167W	900
YML026C	YLR185W	901
YML026C	YLR264W	912
YML026C	YLR287C-A	901
YML026C	YLR333C	914
YML026C	YLR340W	937
YML026C	YLR344W	924
YML026C	YLR367W	997
YML026C	YLR388W	907
YML026C	YLR406C	920
YML026C	YLR441C	918
YML026C	YLR448W	921
YML026C	YML024W	919
YML026C	YML063W	918
YML026C	YML073C	919
YML026C	YMR121C	908
YML026C	YMR142C	919
YML026C	YMR194W	917
YML026C	YMR230W	917
YML026C	YMR242C	925
YML026C	YMR260C	903
YML026C	YNL067W	922
YML026C	YNL069C	962
YML026C	YNL081C	918
YML026C	YNL096C	924
YML026C	YNL162W	926
YML026C	YNL178W	972
YML026C	YNL301C	922
YML026C	YNL302C	914
YML026C	YNR037C	950
YML026C	YOL039W	913
YML026C	YOL040C	999
YML026C	YOL120C	922
YML026C	YOL121C	914

YML026C	YOL127W	909
YML026C	YOR063W	928
YML026C	YOR096W	997
YML026C	YOR133W	912
YML026C	YOR167C	912
YML026C	YOR182C	901
YML026C	YOR234C	920
YML026C	YOR293W	917
YML026C	YOR312C	925
YML026C	YOR369C	914
YML026C	YPL079W	921
YML026C	YPL081W	968
YML026C	YPL090C	907
YML026C	YPL131W	912
YML026C	YPL143W	920
YML026C	YPL198W	920
YML026C	YPL220W	918
YML026C	YPL237W	906
YML026C	YPL249C-A	918
YML026C	YPR043W	920
YML026C	YPR080W	907
YML026C	YPR102C	934
YML026C	YPR132W	927
YML028W	YDR353W	952
YML028W	YDR453C	980
YML028W	YER095W	998
YML028W	YGL058W	977
YML028W	YKL086W	956
YML028W	YKL113C	910
YML028W	YKL152C	957
YML028W	YLR032W	902
YML028W	YLR288C	900
YML028W	YML060W	970
YML028W	YMR190C	973
YML028W	YMR224C	972
YML028W	YOR368W	924
YML029W	YBR201W	999
YML029W	YDR057W	983
YML029W	YLR207W	990
YML029W	YOL013C	994
YML029W	YOR153W	915
YML031W	YDL088C	989
YML031W	YFR002W	990
YML031W	YLR018C	984

YML031W	YLR347C	980
YML031W	YMR047C	962
YML031W	YMR129W	984
YML031W	YMR153W	988
YML031W	YOR257W	961
YML032C	YAL021C	979
YML032C	YAR002W	990
YML032C	YAR007C	999
YML032C	YBR073W	993
YML032C	YBR088C	996
YML032C	YBR136W	994
YML032C	YBR160W	976
YML032C	YBR223C	978
YML032C	YCR028C-A	999
YML032C	YCR066W	997
YML032C	YDL013W	971
YML032C	YDL042C	909
YML032C	YDL059C	999
YML032C	YDL064W	971
YML032C	YDL101C	991
YML032C	YDL102W	997
YML032C	YDL220C	954
YML032C	YDR004W	997
YML032C	YDR075W	932
YML032C	YDR076W	998
YML032C	YDR097C	976
YML032C	YDR217C	994
YML032C	YDR224C	910
YML032C	YDR227W	926
YML032C	YDR369C	996
YML032C	YDR409W	957
YML032C	YDR510W	902
YML032C	YER095W	999
YML032C	YER116C	994
YML032C	YER171W	999
YML032C	YER173W	937
YML032C	YER179W	977
YML032C	YGL058W	999
YML032C	YGL163C	985
YML032C	YGR258C	992
YML032C	YHL022C	911
YML032C	YHR014W	949
YML032C	YHR031C	997
YML032C	YHR134W	970

YML032C	YJL092W	999
YML032C	YJL115W	975
YML032C	YJL173C	999
YML032C	YJR021C	979
YML032C	YJR043C	990
YML032C	YKL113C	999
YML032C	YKL114C	989
YML032C	YKL213C	936
YML032C	YKR056W	998
YML032C	YKR082W	991
YML032C	YLL002W	987
YML032C	YLR032W	940
YML032C	YLR233C	976
YML032C	YLR265C	907
YML032C	YLR321C	927
YML032C	YLR329W	981
YML032C	YLR383W	900
YML032C	YLR442C	984
YML032C	YML095C	998
YML032C	YML102W	968
YML032C	YMR001C	996
YML032C	YMR106C	998
YML032C	YMR137C	922
YML032C	YMR167W	973
YML032C	YMR190C	999
YML032C	YMR224C	982
YML032C	YMR284W	999
YML032C	YNL082W	911
YML032C	YNL250W	995
YML032C	YNL312W	999
YML032C	YOL090W	943
YML032C	YOR005C	991
YML032C	YOR144C	999
YML032C	YPL020C	966
YML032C	YPL022W	999
YML032C	YPL024W	970
YML032C	YPL167C	999
YML032C	YPR119W	922
YML032C	YPR135W	989
YML034W	YDL003W	908
YML034W	YDL089W	998
YML034W	YOL012C	960
YML035C	YDR441C	909
YML035C	YJL070C	999

YML035C	YJR105W	931
YML035C	YLR359W	935
YML035C	YML022W	916
YML035C	YNL220W	918
YML036W	YDL220C	956
YML036W	YGR262C	999
YML036W	YJL184W	999
YML036W	YKR038C	999
YML036W	YKR095W-A	992
YML039W	YMR050C	937
YML041C	YAL011W	997
YML041C	YAR002W	912
YML041C	YAR003W	903
YML041C	YBR175W	952
YML041C	YBR231C	998
YML041C	YDL074C	978
YML041C	YDR190C	999
YML041C	YDR334W	999
YML041C	YDR392W	904
YML041C	YDR485C	998
YML041C	YEL003W	917
YML041C	YEL018W	973
YML041C	YFL039C	999
YML041C	YGL043W	972
YML041C	YGL058W	957
YML041C	YGL127C	987
YML041C	YGL244W	920
YML041C	YGR002C	997
YML041C	YJL030W	907
YML041C	YJL081C	993
YML041C	YJL168C	979
YML041C	YJR082C	919
YML041C	YLR015W	909
YML041C	YLR055C	928
YML041C	YLR085C	999
YML041C	YLR385C	993
YML041C	YLR418C	948
YML041C	YML094W	945
YML041C	YMR263W	908
YML041C	YNL107W	995
YML041C	YNL136W	942
YML041C	YNL153C	913
YML041C	YOL012C	999
YML041C	YOR026W	903

YML041C	YOR123C	938
YML041C	YOR349W	945
YML041C	YPL055C	979
YML041C	YPL235W	999
YML041C	YPL241C	954
YML041C	YPR023C	955
YML042W	YCR005C	920
YML042W	YDR244W	984
YML042W	YNL071W	912
YML043C	YBL014C	999
YML043C	YER148W	993
YML043C	YJL025W	999
YML046W	YBR055C	990
YML046W	YBR119W	999
YML046W	YDL030W	976
YML046W	YDL087C	999
YML046W	YDR235W	999
YML046W	YDR240C	999
YML046W	YER029C	997
YML046W	YER172C	994
YML046W	YGR013W	999
YML046W	YGR074W	999
YML046W	YGR091W	990
YML046W	YHR086W	999
YML046W	YHR165C	998
YML046W	YIL061C	999
YML046W	YKL012W	999
YML046W	YKL173W	989
YML046W	YLR147C	989
YML046W	YLR275W	989
YML046W	YLR298C	999
YML046W	YML049C	976
YML046W	YMR125W	992
YML046W	YMR240C	974
YML046W	YMR288W	976
YML046W	YPL213W	995
YML046W	YPR082C	976
YML046W	YPR178W	976
YML046W	YPR182W	999
YML048W	YHR094C	951
YML048W	YMR031C	923
YML049C	YAL032C	999
YML049C	YBR055C	997
YML049C	YBR152W	945

YML049C	YCR063W	994
YML049C	YDL030W	999
YML049C	YDL043C	993
YML049C	YDL087C	999
YML049C	YDL098C	976
YML049C	YDL209C	993
YML049C	YDR235W	974
YML049C	YDR416W	976
YML049C	YDR473C	978
YML049C	YER029C	999
YML049C	YER172C	999
YML049C	YFL017W-A	987
YML049C	YGL128C	976
YML049C	YGR013W	976
YML049C	YGR074W	997
YML049C	YGR091W	978
YML049C	YGR278W	974
YML049C	YHR165C	999
YML049C	YIL061C	976
YML049C	YIR005W	946
YML049C	YIR009W	972
YML049C	YJL203W	998
YML049C	YKL012W	974
YML049C	YKL173W	998
YML049C	YLL036C	997
YML049C	YLR117C	999
YML049C	YLR147C	998
YML049C	YLR275W	997
YML049C	YLR424W	976
YML049C	YML046W	976
YML049C	YMR125W	999
YML049C	YMR213W	999
YML049C	YMR240C	999
YML049C	YMR288W	999
YML049C	YNL138W-A	998
YML049C	YOR159C	999
YML049C	YOR308C	978
YML049C	YOR319W	999
YML049C	YPL151C	976
YML049C	YPL178W	985
YML049C	YPL213W	999
YML049C	YPR082C	997
YML049C	YPR094W	998
YML049C	YPR101W	976

YML049C	YPR178W	978
YML049C	YPR182W	990
YML051W	YBR018C	972
YML051W	YBR019C	972
YML051W	YBR020W	999
YML051W	YCL045C	912
YML051W	YDR009W	999
YML051W	YLR081W	957
YML051W	YOL148C	900
YML051W	YPL248C	999
YML052W	YBR021W	975
YML052W	YDR388W	906
YML052W	YEL063C	978
YML052W	YGR086C	946
YML052W	YPL004C	950
YML052W	YPL058C	998
YML053C	YJR009C	986
YML054C	YDL174C	984
YML054C	YFL030W	901
YML054C	YIL070C	942
YML054C	YPL063W	972
YML055W	YER019C-A	922
YML055W	YER087C-B	922
YML055W	YIR022W	999
YML055W	YJL034W	923
YML055W	YJR010C-A	997
YML055W	YLR066W	999
YML055W	YOR254C	951
YML055W	YPL094C	924
YML056C	YAR073W	965
YML056C	YBR189W	976
YML056C	YCL037C	972
YML056C	YDR025W	974
YML056C	YER102W	976
YML056C	YGL076C	978
YML056C	YGL135W	979
YML056C	YGL173C	975
YML056C	YHR216W	999
YML056C	YJR145C	980
YML056C	YLR028C	902
YML056C	YLR432W	999
YML056C	YMR120C	901
YML056C	YMR217W	991
YML056C	YNL220W	931

YML056C	YOL139C	976
YML056C	YOR063W	978
YML056C	YPL198W	980
YML057W	YBR109C	999
YML057W	YKL190W	999
YML057W	YLR342W	996
YML057W	YLR433C	999
YML058W	YBR136W	999
YML058W	YDL101C	999
YML058W	YDR388W	901
YML058W	YDR499W	999
YML058W	YER070W	999
YML058W	YHR031C	970
YML058W	YML061C	970
YML058W	YOR330C	976
YML058W	YPL153C	999
YML059C	YBR221C	978
YML060W	YAL015C	962
YML060W	YDR097C	997
YML060W	YDR419W	998
YML060W	YML028W	970
YML060W	YMR201C	915
YML060W	YOL043C	961
YML060W	YOL090W	909
YML061C	YDL220C	998
YML061C	YER095W	963
YML061C	YGL163C	955
YML061C	YHR031C	979
YML061C	YHR164C	944
YML061C	YJR043C	914
YML061C	YLR318W	991
YML061C	YML058W	970
YML061C	YMR106C	980
YML061C	YMR190C	997
YML061C	YMR224C	994
YML061C	YMR284W	999
YML062C	YCL011C	999
YML062C	YDL084W	999
YML062C	YDR138W	999
YML062C	YDR381W	999
YML062C	YHR167W	999
YML062C	YHR193C	913
YML062C	YNL004W	999
YML062C	YNL139C	999

YML062C	YNL253W	999
YML062C	YOR001W	926
YML062C	YPL224C	928
YML063W	YAL035W	918
YML063W	YBL027W	996
YML063W	YBL072C	995
YML063W	YBL087C	931
YML063W	YBL092W	998
YML063W	YBR031W	998
YML063W	YBR048W	996
YML063W	YBR079C	921
YML063W	YBR084C-A	954
YML063W	YBR118W	916
YML063W	YBR181C	995
YML063W	YBR189W	999
YML063W	YBR191W	999
YML063W	YCR031C	998
YML063W	YDL014W	955
YML063W	YDL061C	973
YML063W	YDL075W	998
YML063W	YDL081C	929
YML063W	YDL082W	997
YML063W	YDL083C	966
YML063W	YDL130W	921
YML063W	YDL136W	998
YML063W	YDL191W	920
YML063W	YDR012W	998
YML063W	YDR025W	996
YML063W	YDR064W	999
YML063W	YDR091C	914
YML063W	YDR382W	998
YML063W	YDR385W	918
YML063W	YDR418W	999
YML063W	YDR429C	919
YML063W	YDR447C	996
YML063W	YDR450W	995
YML063W	YDR471W	999
YML063W	YDR500C	944
YML063W	YEL054C	998
YML063W	YER025W	972
YML063W	YER056C-A	993
YML063W	YER074W	995
YML063W	YER102W	998
YML063W	YER117W	959

YML063W	YER131W	986
YML063W	YER165W	929
YML063W	YFL034C-A	918
YML063W	YFR031C-A	996
YML063W	YFR032C-A	927
YML063W	YGL014W	956
YML063W	YGL030W	999
YML063W	YGL031C	998
YML063W	YGL076C	999
YML063W	YGL103W	999
YML063W	YGL123W	987
YML063W	YGL135W	996
YML063W	YGL147C	997
YML063W	YGL189C	918
YML063W	YGR027C	995
YML063W	YGR034W	996
YML063W	YGR085C	999
YML063W	YGR118W	999
YML063W	YGR148C	998
YML063W	YGR162W	911
YML063W	YGR214W	999
YML063W	YHL001W	999
YML063W	YHL015W	995
YML063W	YHL033C	999
YML063W	YHR010W	986
YML063W	YHR021C	928
YML063W	YHR141C	997
YML063W	YHR203C	998
YML063W	YIL018W	995
YML063W	YIL052C	998
YML063W	YIL069C	996
YML063W	YIL133C	996
YML063W	YIL148W	956
YML063W	YJL136C	964
YML063W	YJL177W	999
YML063W	YJL189W	942
YML063W	YJL190C	997
YML063W	YJL191W	999
YML063W	YJR094W-A	975
YML063W	YJR123W	999
YML063W	YJR145C	995
YML063W	YKL006W	999
YML063W	YKL156W	984
YML063W	YKL180W	999

YML063W	YKR057W	932
YML063W	YKR059W	948
YML063W	YKR094C	908
YML063W	YLL045C	998
YML063W	YLR029C	999
YML063W	YLR048W	997
YML063W	YLR061W	994
YML063W	YLR075W	998
YML063W	YLR150W	954
YML063W	YLR167W	941
YML063W	YLR185W	939
YML063W	YLR264W	994
YML063W	YLR287C-A	995
YML063W	YLR325C	926
YML063W	YLR333C	949
YML063W	YLR340W	998
YML063W	YLR344W	969
YML063W	YLR367W	981
YML063W	YLR388W	992
YML063W	YLR406C	921
YML063W	YLR441C	995
YML063W	YLR448W	999
YML063W	YML024W	999
YML063W	YML026C	918
YML063W	YML073C	996
YML063W	YMR121C	936
YML063W	YMR142C	998
YML063W	YMR194W	999
YML063W	YMR230W	963
YML063W	YMR242C	997
YML063W	YMR260C	931
YML063W	YMR290C	949
YML063W	YMR309C	922
YML063W	YNL067W	999
YML063W	YNL069C	996
YML063W	YNL096C	996
YML063W	YNL132W	937
YML063W	YNL162W	930
YML063W	YNL178W	999
YML063W	YNL301C	998
YML063W	YNL302C	926
YML063W	YOL039W	925
YML063W	YOL040C	997
YML063W	YOL120C	998

YML063W	YOL121C	943
YML063W	YOL127W	999
YML063W	YOR063W	999
YML063W	YOR096W	999
YML063W	YOR133W	930
YML063W	YOR167C	959
YML063W	YOR182C	901
YML063W	YOR204W	944
YML063W	YOR234C	986
YML063W	YOR293W	996
YML063W	YOR312C	997
YML063W	YOR361C	928
YML063W	YOR369C	949
YML063W	YPL012W	942
YML063W	YPL079W	979
YML063W	YPL081W	966
YML063W	YPL090C	994
YML063W	YPL131W	978
YML063W	YPL143W	983
YML063W	YPL198W	997
YML063W	YPL220W	997
YML063W	YPL237W	962
YML063W	YPL249C-A	998
YML063W	YPR043W	939
YML063W	YPR080W	916
YML063W	YPR102C	999
YML063W	YPR132W	965
YML063W	YPR163C	923
YML064C	YAL024C	999
YML064C	YAR019C	999
YML064C	YFR028C	999
YML064C	YGR092W	998
YML064C	YIL106W	926
YML064C	YJL076W	997
YML064C	YJR053W	999
YML064C	YJR056C	993
YML064C	YLR079W	978
YML064C	YMR001C	997
YML064C	YMR055C	999
YML064C	YPL242C	976
YML064C	YPR111W	929
YML065W	YBL023C	996
YML065W	YBL035C	933
YML065W	YBR060C	999

YML065W	YBR160W	992
YML065W	YBR202W	990
YML065W	YDL017W	916
YML065W	YEL032W	990
YML065W	YEL056W	999
YML065W	YGL201C	991
YML065W	YHR118C	999
YML065W	YIL150C	988
YML065W	YJL194W	999
YML065W	YJR046W	984
YML065W	YKR101W	999
YML065W	YLL004W	999
YML065W	YLR103C	999
YML065W	YLR274W	992
YML065W	YML109W	988
YML065W	YNL261W	999
YML065W	YPL001W	999
YML065W	YPR019W	995
YML065W	YPR120C	967
YML065W	YPR162C	999
YML065W	YPR175W	954
YML067C	YAL042W	999
YML067C	YBR229C	969
YML067C	YLR026C	906
YML067C	YML130C	934
YML068W	YDR172W	982
YML069W	YBL002W	984
YML069W	YBL008W	925
YML069W	YBL035C	981
YML069W	YBR009C	997
YML069W	YBR010W	917
YML069W	YBR279W	999
YML069W	YDR225W	932
YML069W	YER164W	999
YML069W	YGL019W	999
YML069W	YGL207W	999
YML069W	YGL244W	999
YML069W	YGR063C	959
YML069W	YGR116W	964
YML069W	YIL035C	999
YML069W	YIR008C	981
YML069W	YJL168C	986
YML069W	YKL045W	981
YML069W	YLR410W	964

YML069W	YLR418C	998
YML069W	YML010W	945
YML069W	YNL030W	987
YML069W	YNL102W	999
YML069W	YNL330C	974
YML069W	YOL054W	997
YML069W	YOL145C	999
YML069W	YOR039W	999
YML069W	YOR061W	999
YML069W	YOR123C	999
YML069W	YPR052C	989
YML069W	YPR135W	956
YML070W	YDL022W	934
YML070W	YFL053W	976
YML071C	YBR164C	933
YML071C	YCL001W	909
YML071C	YER157W	999
YML071C	YGL005C	999
YML071C	YGL054C	921
YML071C	YGL223C	999
YML071C	YGR120C	999
YML071C	YLR039C	955
YML071C	YMR292W	905
YML071C	YNL041C	999
YML071C	YNL051W	999
YML071C	YOL018C	911
YML071C	YOR070C	991
YML071C	YPL051W	916
YML071C	YPL240C	919
YML071C	YPR105C	999
YML072C	YGL103W	975
YML072C	YOR086C	902
YML073C	YBL027W	996
YML073C	YBL072C	995
YML073C	YBL087C	955
YML073C	YBL092W	996
YML073C	YBR031W	997
YML073C	YBR048W	996
YML073C	YBR084C-A	924
YML073C	YBR118W	909
YML073C	YBR142W	941
YML073C	YBR181C	996
YML073C	YBR189W	994
YML073C	YBR191W	996

YML073C	YCR031C	996
YML073C	YDL061C	907
YML073C	YDL075W	996
YML073C	YDL081C	942
YML073C	YDL082W	997
YML073C	YDL083C	970
YML073C	YDL130W	902
YML073C	YDL136W	996
YML073C	YDL191W	922
YML073C	YDR012W	997
YML073C	YDR025W	916
YML073C	YDR064W	997
YML073C	YDR382W	996
YML073C	YDR385W	917
YML073C	YDR418W	996
YML073C	YDR447C	997
YML073C	YDR450W	919
YML073C	YDR471W	995
YML073C	YDR496C	935
YML073C	YDR500C	912
YML073C	YEL054C	926
YML073C	YER056C-A	911
YML073C	YER074W	996
YML073C	YER102W	995
YML073C	YER117W	931
YML073C	YER131W	916
YML073C	YFL034C-A	913
YML073C	YFR031C-A	997
YML073C	YFR032C-A	935
YML073C	YGL014W	958
YML073C	YGL030W	997
YML073C	YGL031C	995
YML073C	YGL076C	997
YML073C	YGL103W	995
YML073C	YGL123W	998
YML073C	YGL135W	996
YML073C	YGL147C	997
YML073C	YGL189C	916
YML073C	YGR027C	924
YML073C	YGR034W	996
YML073C	YGR085C	996
YML073C	YGR118W	998
YML073C	YGR148C	906
YML073C	YGR214W	924

YML073C	YHL001W	996
YML073C	YHL015W	998
YML073C	YHL033C	996
YML073C	YHR010W	906
YML073C	YHR021C	967
YML073C	YHR141C	997
YML073C	YHR203C	996
YML073C	YIL018W	996
YML073C	YIL052C	911
YML073C	YIL069C	996
YML073C	YIL133C	997
YML073C	YIL148W	907
YML073C	YJL136C	929
YML073C	YJL177W	996
YML073C	YJL189W	909
YML073C	YJL190C	997
YML073C	YJL191W	919
YML073C	YJR094W-A	929
YML073C	YJR123W	997
YML073C	YJR145C	995
YML073C	YKL006W	972
YML073C	YKL156W	929
YML073C	YKL180W	996
YML073C	YKR057W	930
YML073C	YKR094C	907
YML073C	YLL045C	995
YML073C	YLR029C	996
YML073C	YLR048W	924
YML073C	YLR061W	916
YML073C	YLR075W	996
YML073C	YLR150W	956
YML073C	YLR167W	903
YML073C	YLR185W	912
YML073C	YLR264W	913
YML073C	YLR287C-A	995
YML073C	YLR325C	927
YML073C	YLR333C	923
YML073C	YLR340W	997
YML073C	YLR344W	970
YML073C	YLR367W	920
YML073C	YLR388W	908
YML073C	YLR406C	919
YML073C	YLR432W	978
YML073C	YLR441C	997

YML073C	YLR448W	995
YML073C	YML024W	930
YML073C	YML026C	919
YML073C	YML063W	996
YML073C	YMR121C	920
YML073C	YMR142C	996
YML073C	YMR194W	996
YML073C	YMR230W	929
YML073C	YMR242C	997
YML073C	YMR260C	914
YML073C	YMR290C	940
YML073C	YNL002C	933
YML073C	YNL061W	933
YML073C	YNL067W	926
YML073C	YNL069C	996
YML073C	YNL096C	996
YML073C	YNL112W	976
YML073C	YNL162W	939
YML073C	YNL178W	997
YML073C	YNL301C	996
YML073C	YNL302C	946
YML073C	YOL039W	910
YML073C	YOL040C	998
YML073C	YOL041C	927
YML073C	YOL120C	998
YML073C	YOL121C	934
YML073C	YOL127W	997
YML073C	YOR063W	996
YML073C	YOR096W	995
YML073C	YOR133W	917
YML073C	YOR167C	913
YML073C	YOR182C	903
YML073C	YOR204W	933
YML073C	YOR234C	998
YML073C	YOR293W	929
YML073C	YOR312C	997
YML073C	YOR369C	942
YML073C	YPL079W	927
YML073C	YPL081W	916
YML073C	YPL090C	995
YML073C	YPL093W	937
YML073C	YPL131W	932
YML073C	YPL143W	998
YML073C	YPL198W	997

YML073C	YPL220W	996
YML073C	YPL240C	923
YML073C	YPL249C-A	997
YML073C	YPR043W	929
YML073C	YPR080W	909
YML073C	YPR102C	924
YML073C	YPR132W	928
YML074C	YDR457W	976
YML074C	YLR449W	963
YML075C	YLR450W	999
YML075C	YML126C	942
YML075C	YMR208W	905
YML076C	YPL058C	923
YML077W	YBR254C	999
YML077W	YDR108W	999
YML077W	YDR246W	999
YML077W	YDR407C	999
YML077W	YDR472W	999
YML077W	YFL038C	998
YML077W	YGR166W	999
YML077W	YKR068C	999
YML077W	YMR218C	999
YML077W	YOR115C	999
YML077W	YPR017C	902
YML078W	YDR155C	977
YML078W	YDR304C	962
YML078W	YHR057C	918
YML081C-A	Q0085	957
YML081C-A	YBL099W	999
YML081C-A	YBR039W	934
YML081C-A	YDL004W	925
YML081C-A	YDR377W	984
YML081C-A	YJR121W	999
YML081C-A	YKL016C	967
YML081C-A	YPL078C	999
YML081C-A	YPR020W	957
YML082W	YAL012W	917
YML082W	YER091C	902
YML082W	YGL125W	935
YML082W	YJR130C	902
YML082W	YNL277W	901
YML083C	YDR213W	902
YML085C	YBR107C	911
YML085C	YCL029C	976

YML085C	YDR150W	922
YML085C	YDR212W	988
YML085C	YDR364C	982
YML085C	YEL003W	982
YML085C	YER007W	999
YML085C	YER013W	910
YML085C	YER016W	988
YML085C	YFL037W	999
YML085C	YGR078C	997
YML085C	YGR188C	984
YML085C	YLR045C	996
YML085C	YLR200W	982
YML085C	YML094W	982
YML085C	YML124C	999
YML085C	YMR138W	906
YML085C	YNL148C	981
YML085C	YNL153C	982
YML085C	YNL223W	986
YML085C	YOR026W	988
YML085C	YOR265W	999
YML085C	YOR349W	994
YML088W	YDL132W	999
YML088W	YDR328C	999
YML088W	YER143W	988
YML091C	YBL066C	929
YML091C	YBR173C	928
YML091C	YFR050C	937
YML091C	YMR203W	997
YML091C	YNL118C	998
YML092C	YBL041W	999
YML092C	YBL068W	967
YML092C	YBR173C	929
YML092C	YDL097C	929
YML092C	YDL147W	935
YML092C	YDL188C	942
YML092C	YDR427W	900
YML092C	YER012W	999
YML092C	YER021W	933
YML092C	YER094C	999
YML092C	YFL007W	999
YML092C	YFR004W	928
YML092C	YFR050C	999
YML092C	YFR052W	955
YML092C	YGL011C	999

YML092C	YGL048C	957
YML092C	YGL172W	999
YML092C	YGR135W	999
YML092C	YGR253C	999
YML092C	YHL030W	974
YML092C	YHR027C	908
YML092C	YHR200W	998
YML092C	YIL009C-A	912
YML092C	YJL001W	999
YML092C	YKL145W	950
YML092C	YLL040C	940
YML092C	YMR314W	999
YML092C	YOL038W	999
YML092C	YOR117W	950
YML092C	YOR157C	999
YML092C	YOR259C	900
YML092C	YOR261C	902
YML092C	YOR362C	999
YML092C	YPR103W	999
YML092C	YPR108W	917
YML093W	YBR247C	916
YML093W	YCL059C	915
YML093W	YDL060W	910
YML093W	YDL148C	959
YML093W	YDL153C	916
YML093W	YDR324C	910
YML093W	YDR398W	912
YML093W	YDR449C	911
YML093W	YGL120C	965
YML093W	YGL171W	922
YML093W	YGR128C	920
YML093W	YGR145W	911
YML093W	YHR148W	916
YML093W	YHR169W	916
YML093W	YHR196W	938
YML093W	YJL069C	961
YML093W	YJL109C	911
YML093W	YJR002W	989
YML093W	YLL011W	976
YML093W	YLR003C	914
YML093W	YLR129W	946
YML093W	YLR175W	919
YML093W	YLR197W	911
YML093W	YLR222C	938

YML093W	YLR409C	919
YML093W	YMR093W	914
YML093W	YMR229C	933
YML093W	YMR290C	922
YML093W	YNL075W	920
YML093W	YNL132W	911
YML093W	YOL010W	909
YML093W	YOR004W	938
YML093W	YOR145C	919
YML093W	YOR310C	923
YML093W	YPL012W	911
YML093W	YPL126W	921
YML093W	YPL217C	916
YML093W	YPR112C	920
YML093W	YPR137W	911
YML094W	YAL011W	930
YML094W	YBL034C	976
YML094W	YBR231C	926
YML094W	YDL143W	900
YML094W	YDR188W	902
YML094W	YDR212W	900
YML094W	YDR334W	912
YML094W	YDR485C	963
YML094W	YEL003W	999
YML094W	YER016W	938
YML094W	YFL037W	960
YML094W	YFL039C	970
YML094W	YGL043W	925
YML094W	YGR078C	999
YML094W	YIL142W	900
YML094W	YJL014W	900
YML094W	YJL030W	961
YML094W	YJL168C	963
YML094W	YJL179W	999
YML094W	YLR200W	999
YML094W	YML041C	945
YML094W	YML085C	982
YML094W	YML124C	936
YML094W	YNL153C	999
YML094W	YOR265W	916
YML094W	YPL018W	909
YML094W	YPR046W	922
YML095C	YAL027W	999
YML095C	YAR007C	999

YML095C	YBL019W	992
YML095C	YBR098W	933
YML095C	YBR114W	971
YML095C	YCR066W	952
YML095C	YDL140C	910
YML095C	YDR311W	984
YML095C	YDR386W	938
YML095C	YDR460W	980
YML095C	YEL037C	937
YML095C	YER162C	997
YML095C	YER171W	997
YML095C	YGR063C	937
YML095C	YGR258C	998
YML095C	YIL128W	940
YML095C	YIL143C	992
YML095C	YJR052W	969
YML095C	YKL113C	938
YML095C	YKL114C	999
YML095C	YLR005W	984
YML095C	YLR032W	902
YML095C	YLR135W	930
YML095C	YML032C	998
YML095C	YMR201C	999
YML095C	YMR224C	902
YML095C	YNL312W	989
YML095C	YOL090W	994
YML095C	YPL022W	999
YML097C	YBR102C	945
YML097C	YCR032W	927
YML097C	YLL039C	999
YML097C	YLR167W	976
YML097C	YOR089C	953
YML098W	YBR198C	999
YML098W	YCR042C	999
YML098W	YDR145W	999
YML098W	YDR167W	999
YML098W	YDR443C	909
YML098W	YER068W	918
YML098W	YER148W	999
YML098W	YGL112C	999
YML098W	YGR097W	911
YML098W	YGR274C	999
YML098W	YHR041C	910
YML098W	YKL058W	930

YML098W	YML015C	999
YML098W	YML114C	999
YML098W	YMR005W	999
YML098W	YMR227C	999
YML098W	YMR236W	999
YML098W	YOL051W	959
YML098W	YOR174W	987
YML098W	YOR194C	901
YML098W	YPL011C	999
YML098W	YPL101W	923
YML098W	YPL129W	999
YML098W	YPR070W	906
YML098W	YPR072W	921
YML098W	YPR086W	933
YML099C	YDR173C	963
YML099C	YMR042W	999
YML099C	YMR043W	999
YML100W	YBR126C	999
YML100W	YDR074W	999
YML100W	YLR258W	915
YML100W	YMR261C	999
YML102W	YBL008W	994
YML102W	YBR088C	996
YML102W	YBR195C	999
YML102W	YJL115W	999
YML102W	YKL113C	909
YML102W	YML032C	968
YML102W	YOR038C	907
YML102W	YPL153C	903
YML102W	YPR018W	999
YML103C	YBL079W	988
YML103C	YDL088C	965
YML103C	YDL207W	962
YML103C	YDR159W	972
YML103C	YER105C	998
YML103C	YER110C	935
YML103C	YFR002W	999
YML103C	YGL092W	977
YML103C	YGL172W	943
YML103C	YGR119C	949
YML103C	YJL041W	942
YML103C	YKL057C	993
YML103C	YKL068W	993
YML103C	YKR082W	992

YML103C	YLR018C	986
YML103C	YLR335W	934
YML103C	YMR047C	992
YML103C	YMR129W	999
YML103C	YMR153W	965
YML103C	YOR098C	979
YML103C	YOR257W	961
YML103C	YOR284W	935
YML103C	YPL125W	919
YML103C	YPL169C	966
YML103C	YPL206C	935
YML104C	YHR005C	925
YML105C	YAL015C	954
YML105C	YDL051W	995
YML105C	YDL092W	999
YML105C	YKL122C	999
YML105C	YPL210C	999
YML105C	YPL243W	999
YML105C	YPR088C	999
YML106W	YBL068W	911
YML106W	YEL021W	989
YML106W	YGL030W	946
YML106W	YKL006W	962
YML106W	YKL180W	972
YML106W	YKL181W	994
YML106W	YKL216W	976
YML106W	YLR048W	944
YML106W	YLR264W	960
YML106W	YLR372W	901
YML106W	YMR271C	962
YML106W	YOL061W	936
YML107C	YDL116W	952
YML107C	YKL057C	989
YML107C	YKR082W	984
YML109W	YAL016W	999
YML109W	YBL085W	958
YML109W	YBL105C	992
YML109W	YBR160W	993
YML109W	YDL042C	939
YML109W	YDL134C	999
YML109W	YDL188C	999
YML109W	YDR227W	995
YML109W	YDR293C	941
YML109W	YDR309C	905

YML109W	YGL130W	983
YML109W	YGL190C	999
YML109W	YHR061C	970
YML109W	YHR149C	949
YML109W	YKL082C	951
YML109W	YKR101W	990
YML109W	YLR442C	981
YML109W	YML065W	988
YML109W	YMR273C	999
YML109W	YNL216W	963
YML109W	YNL298W	905
YML109W	YOR362C	945
YML110C	YBR003W	971
YML110C	YDR204W	914
YML110C	YGL115W	916
YML110C	YGL119W	934
YML110C	YGR255C	994
YML110C	YLR201C	941
YML110C	YNR041C	929
YML110C	YOL096C	955
YML110C	YOR125C	988
YML111W	YER125W	998
YML111W	YGL144C	976
YML111W	YMR275C	996
YML111W	YNL183C	925
YML111W	YNL307C	953
YML112W	YJL006C	999
YML112W	YKL139W	999
YML114C	YBR081C	923
YML114C	YBR198C	999
YML114C	YCR042C	999
YML114C	YDR145W	999
YML114C	YDR167W	999
YML114C	YER148W	999
YML114C	YGL112C	999
YML114C	YGR274C	999
YML114C	YLR182W	923
YML114C	YML015C	998
YML114C	YML098W	999
YML114C	YMR005W	999
YML114C	YMR227C	999
YML114C	YMR236W	991
YML114C	YPL011C	999
YML114C	YPL129W	999

YML114C	YPL254W	923
YML115C	YBR023C	902
YML115C	YDR245W	953
YML115C	YEL036C	970
YML115C	YJL183W	921
YML115C	YJR075W	977
YML115C	YPL050C	999
YML115C	YPL221W	915
YML117W	YDR172W	924
YML117W	YER165W	972
YML117W	YGL049C	968
YML117W	YGR229C	931
YML117W	YNL251C	999
YML117W	YPL190C	966
YML119W	YLL032C	992
YML121W	YBR077C	999
YML121W	YGL023C	996
YML121W	YGL097W	998
YML121W	YGR163W	999
YML121W	YIL063C	907
YML121W	YKL143W	989
YML121W	YKR007W	999
YML121W	YMR235C	991
YML121W	YNL113W	989
YML121W	YOL144W	951
YML123C	YBR296C	987
YML123C	YCR037C	998
YML123C	YER148W	903
YML123C	YJL117W	994
YML123C	YJL198W	987
YML123C	YNR013C	987
YML123C	YOL001W	907
YML124C	YBL034C	964
YML124C	YDR150W	901
YML124C	YDR188W	915
YML124C	YEL003W	960
YML124C	YER007W	987
YML124C	YER016W	910
YML124C	YFL037W	931
YML124C	YGL086W	964
YML124C	YGR078C	999
YML124C	YJL013C	905
YML124C	YJL030W	952
YML124C	YJL179W	923

YML124C	YLR045C	979
YML124C	YLR200W	950
YML124C	YML085C	999
YML124C	YML094W	936
YML124C	YMR012W	958
YML124C	YNL148C	981
YML124C	YNL153C	961
YML124C	YOL012C	945
YML124C	YOR265W	990
YML124C	YOR349W	998
YML124C	YPL241C	929
YML124C	YPR141C	981
YML126C	YIL160C	913
YML126C	YKL125W	931
YML126C	YLR450W	936
YML126C	YML075C	942
YML126C	YPL028W	950
YML127W	YBR245C	962
YML127W	YCR020W-B	999
YML127W	YCR052W	999
YML127W	YDR303C	999
YML127W	YFR037C	999
YML127W	YGR056W	999
YML127W	YGR275W	995
YML127W	YHR056C	996
YML127W	YIL126W	999
YML127W	YKR008W	999
YML127W	YLR033W	999
YML127W	YLR321C	999
YML127W	YLR357W	999
YML127W	YMR033W	999
YML127W	YMR091C	999
YML127W	YPR034W	998
YML128C	YJL168C	952
YML129C	Q0045	998
YML129C	YGR112W	988
YML129C	YIL157C	919
YML129C	YLR203C	990
YML130C	YAL042W	977
YML130C	YCL043C	993
YML130C	YJL002C	984
YML130C	YLR208W	973
YML130C	YML067C	934
YML130C	YOL088C	930

YML130C	YPR037C	957
YMR001C	YAL047C	997
YMR001C	YAR019C	999
YMR001C	YBL084C	999
YMR001C	YBR158W	973
YMR001C	YBR160W	999
YMR001C	YDL003W	999
YMR001C	YDL008W	910
YMR001C	YDL134C	967
YMR001C	YDL188C	967
YMR001C	YDR052C	985
YMR001C	YDR113C	960
YMR001C	YFL008W	993
YMR001C	YFR028C	999
YMR001C	YFR036W	912
YMR001C	YGL003C	999
YMR001C	YGL116W	992
YMR001C	YGR092W	999
YMR001C	YGR098C	979
YMR001C	YGR108W	988
YMR001C	YHR023W	903
YMR001C	YHR107C	984
YMR001C	YHR152W	987
YMR001C	YHR166C	989
YMR001C	YIL026C	992
YMR001C	YIL106W	982
YMR001C	YJL019W	908
YMR001C	YJL074C	991
YMR001C	YJL076W	998
YMR001C	YJL187C	999
YMR001C	YJR053W	999
YMR001C	YKL022C	999
YMR001C	YKL101W	976
YMR001C	YLR079W	996
YMR001C	YLR102C	993
YMR001C	YLR127C	910
YMR001C	YML032C	996
YMR001C	YML064C	997
YMR001C	YMR036C	981
YMR001C	YMR055C	999
YMR001C	YNL068C	947
YMR001C	YNL102W	929
YMR001C	YNL172W	910
YMR001C	YNL298W	974

YMR001C	YOR195W	997
YMR001C	YOR372C	954
YMR001C	YOR373W	993
YMR001C	YPL153C	970
YMR001C	YPL209C	984
YMR001C	YPR007C	986
YMR001C	YPR119W	999
YMR004W	YKR001C	985
YMR004W	YOR132W	997
YMR005W	YBR198C	999
YMR005W	YCR042C	999
YMR005W	YDR145W	999
YMR005W	YDR167W	999
YMR005W	YER148W	999
YMR005W	YGL112C	999
YMR005W	YGL127C	960
YMR005W	YGR097W	965
YMR005W	YGR274C	999
YMR005W	YJR140C	923
YMR005W	YKL058W	979
YMR005W	YLR055C	952
YMR005W	YML015C	999
YMR005W	YML098W	999
YMR005W	YML114C	999
YMR005W	YMR227C	999
YMR005W	YMR236W	999
YMR005W	YOL051W	959
YMR005W	YOR174W	969
YMR005W	YPL011C	999
YMR005W	YPL101W	942
YMR005W	YPL129W	991
YMR005W	YPR070W	953
YMR006C	YMR008C	976
YMR008C	YMR006C	976
YMR009W	YEL038W	934
YMR010W	YDR108W	911
YMR010W	YFL025C	911
YMR011W	YBR112C	900
YMR011W	YDL194W	973
YMR011W	YDR277C	909
YMR011W	YPL026C	981
YMR012W	YEL022W	960
YMR012W	YKL029C	976
YMR012W	YLR106C	975

YMR012W	YLR180W	974
YMR012W	YML124C	958
YMR012W	YOR151C	974
YMR013C	YBR002C	994
YMR013C	YBR243C	984
YMR013C	YGR036C	978
YMR013C	YMR101C	986
YMR013C	YMR200W	911
YMR013C	YPL227C	982
YMR013C	YPR183W	991
YMR015C	YEL013W	923
YMR015C	YGL012W	999
YMR015C	YGR060W	999
YMR015C	YGR175C	999
YMR015C	YHR072W	999
YMR015C	YLR056W	972
YMR015C	YLR100W	998
YMR015C	YML008C	999
YMR015C	YNL297C	936
YMR016C	YMR037C	904
YMR016C	YPL203W	987
YMR017W	YAL030W	901
YMR017W	YGR009C	973
YMR017W	YMR183C	980
YMR017W	YOR327C	990
YMR017W	YPL232W	998
YMR019W	YOL004W	904
YMR020W	YMR169C	948
YMR020W	YMR170C	946
YMR020W	YPR069C	915
YMR021C	YKL220C	932
YMR021C	YLR214W	998
YMR021C	YLR411W	959
YMR021C	YMR058W	937
YMR021C	YMR319C	911
YMR021C	YOL152W	987
YMR021C	YPR124W	992
YMR022W	YBR170C	976
YMR022W	YER100W	999
YMR022W	YER125W	979
YMR022W	YFL031W	908
YMR022W	YHR079C	915
YMR022W	YIL030C	997
YMR022W	YLR006C	987

YMR022W	YLR207W	987
YMR022W	YLR450W	976
YMR022W	YML013W	993
YMR022W	YMR264W	993
YMR022W	YNL323W	923
YMR022W	YOL013C	985
YMR023C	YGL236C	997
YMR024W	YBL038W	997
YMR024W	YBR122C	972
YMR024W	YBR282W	970
YMR024W	YCR046C	992
YMR024W	YCR071C	988
YMR024W	YDR116C	988
YMR024W	YDR237W	974
YMR024W	YDR296W	990
YMR024W	YDR322W	976
YMR024W	YDR405W	987
YMR024W	YDR462W	972
YMR024W	YJL041W	902
YMR024W	YJL063C	972
YMR024W	YKL167C	972
YMR024W	YKR006C	972
YMR024W	YKR085C	972
YMR024W	YLR312W-A	988
YMR024W	YLR439W	988
YMR024W	YML025C	992
YMR024W	YMR193W	972
YMR024W	YNL005C	977
YMR024W	YNL185C	916
YMR024W	YNL252C	974
YMR024W	YNL284C	998
YMR024W	YOR150W	978
YMR025W	YDL147W	999
YMR025W	YDL216C	999
YMR025W	YDR179C	999
YMR025W	YIL071C	999
YMR025W	YJR084W	998
YMR025W	YLR423C	984
YMR025W	YNR052C	900
YMR025W	YOL117W	999
YMR026C	YAL055W	913
YMR026C	YDL065C	919
YMR026C	YDR142C	932
YMR026C	YDR244W	999

YMR026C	YDR265W	999
YMR026C	YGL153W	999
YMR026C	YGR077C	992
YMR026C	YJL210W	994
YMR026C	YLR191W	999
YMR026C	YNL214W	993
YMR028W	YAL016W	925
YMR028W	YDL047W	999
YMR028W	YDL134C	999
YMR028W	YDL188C	999
YMR028W	YDR075W	997
YMR028W	YDR188W	927
YMR028W	YHR186C	900
YMR028W	YJL111W	994
YMR028W	YJR066W	968
YMR028W	YKL203C	991
YMR028W	YNR032W	985
YMR028W	YPR040W	999
YMR029C	YFR008W	981
YMR029C	YMR052W	910
YMR031C	YGR130C	995
YMR031C	YML048W	923
YMR032W	YBR260C	926
YMR032W	YDL117W	983
YMR032W	YFL039C	934
YMR032W	YHR023W	951
YMR032W	YIL159W	998
YMR032W	YJR090C	944
YMR032W	YLR337C	996
YMR032W	YNL152W	992
YMR032W	YNL271C	987
YMR033W	YBL006C	960
YMR033W	YBR289W	999
YMR033W	YCR020W-B	998
YMR033W	YCR052W	999
YMR033W	YDR073W	992
YMR033W	YDR303C	999
YMR033W	YFL049W	999
YMR033W	YFR037C	999
YMR033W	YGR056W	999
YMR033W	YGR275W	999
YMR033W	YHL025W	999
YMR033W	YHR056C	997
YMR033W	YIL126W	999

YMR033W	YJL176C	999
YMR033W	YKR001C	983
YMR033W	YKR008W	999
YMR033W	YLR033W	999
YMR033W	YLR321C	999
YMR033W	YLR357W	999
YMR033W	YML127W	999
YMR033W	YMR072W	980
YMR033W	YMR091C	999
YMR033W	YNR023W	999
YMR033W	YOR290C	999
YMR033W	YPL016W	999
YMR033W	YPL129W	999
YMR033W	YPR034W	999
YMR033W	YPR052C	974
YMR035W	YLR368W	981
YMR035W	YMR150C	997
YMR036C	YBR060C	999
YMR036C	YBR160W	999
YMR036C	YBR274W	905
YMR036C	YDL134C	902
YMR036C	YDL155W	948
YMR036C	YFR052W	907
YMR036C	YGL190C	984
YMR036C	YGR108W	948
YMR036C	YHR030C	985
YMR036C	YHR118C	998
YMR036C	YJL187C	999
YMR036C	YLL004W	999
YMR036C	YLL039C	970
YMR036C	YLR210W	951
YMR036C	YMR001C	981
YMR036C	YNL261W	999
YMR036C	YPR119W	983
YMR037C	YDL079C	902
YMR037C	YDR099W	996
YMR037C	YDR335W	956
YMR037C	YDR477W	992
YMR037C	YER133W	930
YMR037C	YFL033C	997
YMR037C	YGL073W	998
YMR037C	YHR205W	944
YMR037C	YJL141C	993
YMR037C	YKL062W	999

YMR037C	YLL026W	989
YMR037C	YLR113W	962
YMR037C	YMR016C	904
YMR037C	YMR139W	949
YMR037C	YNL098C	995
YMR037C	YNL167C	901
YMR037C	YNL307C	901
YMR037C	YOL128C	929
YMR037C	YOR043W	954
YMR037C	YPL042C	990
YMR037C	YPL203W	981
YMR038C	YJR104C	999
YMR039C	YGL044C	926
YMR039C	YMR277W	903
YMR039C	YPR086W	998
YMR042W	YBL093C	962
YMR042W	YDR173C	999
YMR042W	YDR207C	932
YMR042W	YML099C	999
YMR042W	YMR043W	996
YMR043W	YBR202W	979
YMR043W	YCL066W	992
YMR043W	YCL067C	999
YMR043W	YCR084C	973
YMR043W	YCR096C	999
YMR043W	YDR146C	930
YMR043W	YDR173C	999
YMR043W	YDR451C	920
YMR043W	YGL112C	923
YMR043W	YHR084W	999
YMR043W	YIL131C	963
YMR043W	YML099C	999
YMR043W	YMR042W	996
YMR043W	YMR227C	923
YMR043W	YNL068C	999
YMR043W	YOL058W	952
YMR043W	YPR119W	963
YMR044W	YBR245C	972
YMR044W	YLR095C	977
YMR046C	YBL005W-A	998
YMR047C	YAR002W	990
YMR047C	YBL079W	993
YMR047C	YBR017C	928
YMR047C	YDL088C	993

YMR047C	YDL116W	999
YMR047C	YDL207W	995
YMR047C	YDR159W	999
YMR047C	YDR192C	995
YMR047C	YDR335W	953
YMR047C	YER105C	962
YMR047C	YER107C	999
YMR047C	YER110C	985
YMR047C	YFR002W	999
YMR047C	YGL016W	985
YMR047C	YGL092W	999
YMR047C	YGL100W	998
YMR047C	YGL172W	999
YMR047C	YGR119C	995
YMR047C	YGR218W	956
YMR047C	YIL016W	912
YMR047C	YIL063C	962
YMR047C	YIL115C	999
YMR047C	YJL039C	962
YMR047C	YJL041W	999
YMR047C	YJL061W	999
YMR047C	YJR042W	999
YMR047C	YKL057C	998
YMR047C	YKL068W	999
YMR047C	YKL186C	999
YMR047C	YKR082W	992
YMR047C	YLR018C	992
YMR047C	YLR208W	975
YMR047C	YLR335W	991
YMR047C	YLR347C	999
YMR047C	YML031W	962
YMR047C	YML103C	992
YMR047C	YMR080C	920
YMR047C	YMR129W	982
YMR047C	YMR153W	962
YMR047C	YMR308C	999
YMR047C	YNL189W	906
YMR047C	YOR098C	987
YMR047C	YOR112W	988
YMR047C	YOR257W	985
YMR047C	YPL125W	985
YMR047C	YPL169C	999
YMR048W	YCL016C	997
YMR048W	YCL061C	997

YMR048W	YDR075W	905
YMR048W	YDR217C	954
YMR048W	YER016W	949
YMR048W	YHR191C	996
YMR048W	YJL092W	971
YMR048W	YJR043C	959
YMR048W	YKL113C	943
YMR048W	YMR078C	957
YMR048W	YMR190C	970
YMR048W	YNL273W	999
YMR049C	YBR142W	998
YMR049C	YDL031W	981
YMR049C	YDL208W	920
YMR049C	YDR060W	998
YMR049C	YDR087C	995
YMR049C	YDR496C	991
YMR049C	YER006W	999
YMR049C	YER126C	999
YMR049C	YFL002C	995
YMR049C	YGL111W	999
YMR049C	YGR103W	999
YMR049C	YHR052W	999
YMR049C	YHR066W	993
YMR049C	YHR088W	976
YMR049C	YHR197W	983
YMR049C	YKL009W	988
YMR049C	YKL021C	979
YMR049C	YKL172W	988
YMR049C	YKR081C	998
YMR049C	YLL008W	992
YMR049C	YLL034C	979
YMR049C	YLR002C	986
YMR049C	YLR276C	974
YMR049C	YLR449W	995
YMR049C	YMR131C	957
YMR049C	YMR229C	990
YMR049C	YMR290C	999
YMR049C	YNL002C	999
YMR049C	YNL061W	999
YMR049C	YNL110C	999
YMR049C	YOL077C	999
YMR049C	YOL127W	934
YMR049C	YOR063W	965
YMR049C	YOR206W	999

YMR049C	YOR272W	999
YMR049C	YPL043W	999
YMR049C	YPL093W	999
YMR049C	YPL211W	988
YMR049C	YPR016C	999
YMR050C	YML039W	937
YMR052W	YBL016W	919
YMR052W	YDR200C	997
YMR052W	YFR008W	998
YMR052W	YLR238W	915
YMR052W	YMR029C	910
YMR052W	YNL127W	998
YMR053C	YOL004W	976
YMR054W	YBR127C	999
YMR054W	YDL185W	999
YMR054W	YEL027W	901
YMR054W	YEL051W	999
YMR054W	YGR020C	999
YMR054W	YHR039C-A	999
YMR054W	YKL080W	999
YMR054W	YLR447C	999
YMR054W	YOR270C	995
YMR054W	YOR332W	999
YMR054W	YPR036W	999
YMR055C	YAL024C	999
YMR055C	YDL028C	984
YMR055C	YEL061C	996
YMR055C	YER016W	986
YMR055C	YER143W	941
YMR055C	YFR028C	999
YMR055C	YGL086W	994
YMR055C	YGL116W	962
YMR055C	YGR092W	934
YMR055C	YGR188C	995
YMR055C	YHR061C	989
YMR055C	YJL013C	984
YMR055C	YJL030W	999
YMR055C	YJR053W	999
YMR055C	YLR212C	923
YMR055C	YLR314C	973
YMR055C	YML064C	999
YMR055C	YMR001C	999
YMR055C	YNL116W	914
YMR055C	YNL298W	964

YMR055C	YOR026W	965
YMR055C	YOR233W	929
YMR055C	YOR373W	960
YMR055C	YPL269W	999
YMR056C	YBL030C	977
YMR058W	YDR270W	960
YMR058W	YEL065W	954
YMR058W	YER145C	999
YMR058W	YFL041W	999
YMR058W	YGL071W	999
YMR058W	YJR104C	993
YMR058W	YKL220C	929
YMR058W	YLR034C	946
YMR058W	YLR214W	989
YMR058W	YMR021C	937
YMR058W	YMR319C	995
YMR058W	YPL202C	985
YMR058W	YPR124W	956
YMR059W	YAR008W	998
YMR059W	YLR105C	998
YMR059W	YPL083C	998
YMR060C	YAL010C	988
YMR060C	YGR082W	972
YMR060C	YHR083W	999
YMR060C	YNL026W	999
YMR060C	YNL121C	997
YMR061W	YAL043C	999
YMR061W	YDL140C	996
YMR061W	YDR195W	991
YMR061W	YDR228C	999
YMR061W	YDR301W	999
YMR061W	YGL044C	999
YMR061W	YGR156W	995
YMR061W	YIL030C	990
YMR061W	YIR011C	911
YMR061W	YJL033W	967
YMR061W	YJR093C	999
YMR061W	YKL018W	992
YMR061W	YKL059C	978
YMR061W	YKR002W	999
YMR061W	YLR115W	999
YMR061W	YLR277C	999
YMR061W	YLR448W	980
YMR061W	YNL317W	999

YMR061W	YOL123W	976
YMR061W	YOR001W	937
YMR061W	YOR250C	999
YMR062C	YDR300C	912
YMR062C	YER069W	950
YMR062C	YHR018C	963
YMR062C	YJL088W	993
YMR062C	YOL058W	937
YMR062C	YOL140W	985
YMR063W	YPR173C	907
YMR064W	Q0130	903
YMR064W	YOL123W	919
YMR067C	YDL126C	997
YMR067C	YKL213C	965
YMR068W	YER093C	998
YMR068W	YIL105C	999
YMR068W	YJL058C	994
YMR068W	YKL203C	999
YMR068W	YNL006W	998
YMR068W	YNL047C	986
YMR068W	YOL078W	998
YMR069W	YHR005C	918
YMR070W	YLR228C	980
YMR070W	YPL240C	919
YMR070W	YPR065W	959
YMR071C	YDR100W	952
YMR072W	YBL002W	986
YMR072W	YBL003C	989
YMR072W	YBR009C	989
YMR072W	YBR245C	977
YMR072W	YDR148C	909
YMR072W	YDR190C	977
YMR072W	YFL039C	978
YMR072W	YFR037C	978
YMR072W	YKR001C	977
YMR072W	YLR304C	982
YMR072W	YLR355C	992
YMR072W	YMR033W	980
YMR072W	YMR241W	926
YMR072W	YOR058C	954
YMR072W	YPL082C	977
YMR072W	YPR052C	987
YMR075W	YBR095C	986
YMR075W	YDL076C	985

YMR075W	YIL084C	993
YMR075W	YMR263W	986
YMR075W	YNL021W	912
YMR075W	YNL097C	998
YMR075W	YNL330C	999
YMR075W	YOL004W	999
YMR075W	YPL139C	999
YMR075W	YPR023C	999
YMR076C	YDL003W	940
YMR076C	YFL008W	959
YMR076C	YFR027W	991
YMR076C	YIL031W	901
YMR077C	YDR069C	912
YMR077C	YHL027W	946
YMR077C	YJR102C	999
YMR077C	YKL002W	999
YMR077C	YKL041W	999
YMR077C	YLR025W	999
YMR077C	YLR119W	992
YMR077C	YLR417W	999
YMR077C	YOR275C	930
YMR077C	YPL002C	998
YMR077C	YPL065W	999
YMR077C	YPL084W	966
YMR077C	YPR173C	999
YMR078C	YBR087W	999
YMR078C	YBR088C	974
YMR078C	YCL016C	977
YMR078C	YER016W	923
YMR078C	YER173W	999
YMR078C	YFR027W	987
YMR078C	YGR188C	933
YMR078C	YHR191C	999
YMR078C	YJL030W	963
YMR078C	YJL092W	955
YMR078C	YJR043C	930
YMR078C	YJR068W	999
YMR078C	YKL113C	949
YMR078C	YMR048W	957
YMR078C	YNL102W	956
YMR078C	YNL262W	918
YMR078C	YNL290W	999
YMR078C	YOL094C	999
YMR078C	YOR144C	995

YMR078C	YPL008W	979
YMR078C	YPR175W	980
YMR079W	YDL226C	995
YMR079W	YDR164C	932
YMR079W	YGR202C	996
YMR079W	YJL145W	982
YMR079W	YKL091C	942
YMR079W	YKL212W	999
YMR079W	YKR031C	999
YMR079W	YLR133W	999
YMR079W	YLR380W	999
YMR079W	YNL264C	997
YMR079W	YNL267W	988
YMR079W	YNL298W	971
YMR079W	YPL145C	999
YMR080C	YBR143C	995
YMR080C	YDR172W	999
YMR080C	YGL173C	970
YMR080C	YGR072W	999
YMR080C	YHR077C	999
YMR080C	YHR170W	999
YMR080C	YMR047C	920
YMR080C	YNL112W	982
YMR080C	YNL118C	980
YMR080C	YNL244C	943
YMR080C	YOL123W	982
YMR080C	YOL149W	990
YMR080C	YOR076C	908
YMR080C	YOR361C	919
YMR083W	YDR385W	972
YMR083W	YER073W	901
YMR083W	YGL256W	980
YMR083W	YMR169C	900
YMR083W	YMR170C	900
YMR083W	YOL086C	994
YMR083W	YOR374W	901
YMR083W	YPL061W	900
YMR084W	YKL104C	902
YMR084W	YMR085W	952
YMR085W	YMR084W	952
YMR088C	YCL069W	967
YMR089C	YBR185C	980
YMR089C	YER017C	999
YMR089C	YGR132C	974

YMR091C	YBL002W	947
YMR091C	YBL006C	982
YMR091C	YBR245C	999
YMR091C	YCR020W-B	999
YMR091C	YCR052W	999
YMR091C	YDR303C	999
YMR091C	YFR037C	999
YMR091C	YGR056W	999
YMR091C	YGR275W	999
YMR091C	YHR027C	964
YMR091C	YHR056C	999
YMR091C	YIL126W	999
YMR091C	YKR001C	988
YMR091C	YKR008W	999
YMR091C	YLR033W	999
YMR091C	YLR321C	999
YMR091C	YLR357W	999
YMR091C	YML127W	999
YMR091C	YMR033W	999
YMR091C	YPL082C	970
YMR091C	YPL240C	968
YMR091C	YPR034W	999
YMR092C	YFL039C	999
YMR092C	YLL050C	999
YMR093W	YBL004W	997
YMR093W	YBL072C	931
YMR093W	YBR247C	999
YMR093W	YCL059C	997
YMR093W	YCR057C	999
YMR093W	YDL014W	995
YMR093W	YDL060W	954
YMR093W	YDL148C	997
YMR093W	YDR064W	933
YMR093W	YDR299W	926
YMR093W	YDR324C	999
YMR093W	YDR398W	999
YMR093W	YDR449C	999
YMR093W	YEL055C	988
YMR093W	YER082C	999
YMR093W	YGL120C	967
YMR093W	YGL171W	959
YMR093W	YGR090W	997
YMR093W	YGR128C	999
YMR093W	YGR145W	997

YMR093W	YHR148W	998
YMR093W	YHR169W	953
YMR093W	YHR196W	999
YMR093W	YJL010C	983
YMR093W	YJL069C	999
YMR093W	YJL109C	999
YMR093W	YJR002W	999
YMR093W	YJR123W	942
YMR093W	YJR145C	931
YMR093W	YKR060W	997
YMR093W	YLL011W	998
YMR093W	YLR129W	994
YMR093W	YLR175W	920
YMR093W	YLR186W	998
YMR093W	YLR197W	997
YMR093W	YLR222C	993
YMR093W	YLR409C	999
YMR093W	YML093W	914
YMR093W	YMR128W	997
YMR093W	YMR229C	924
YMR093W	YMR290C	953
YMR093W	YNL075W	998
YMR093W	YNL132W	996
YMR093W	YOL010W	931
YMR093W	YOR004W	939
YMR093W	YOR078W	990
YMR093W	YOR096W	933
YMR093W	YOR145C	998
YMR093W	YOR310C	987
YMR093W	YPL012W	999
YMR093W	YPL126W	999
YMR093W	YPL217C	998
YMR093W	YPL266W	999
YMR093W	YPR112C	921
YMR093W	YPR137W	999
YMR093W	YPR144C	999
YMR094W	YDR254W	999
YMR094W	YDR318W	988
YMR094W	YDR328C	999
YMR094W	YGR113W	983
YMR094W	YGR140W	999
YMR094W	YGR188C	935
YMR094W	YJL030W	998
YMR094W	YJR060W	987

YMR094W	YJR135C	981
YMR094W	YKL049C	939
YMR094W	YKL089W	989
YMR094W	YLR045C	926
YMR094W	YLR381W	981
YMR094W	YMR168C	999
YMR094W	YOR057W	999
YMR094W	YOR195W	928
YMR094W	YPL018W	998
YMR094W	YPL240C	958
YMR094W	YPR046W	981
YMR095C	YBR035C	908
YMR095C	YFL059W	999
YMR095C	YFL060C	993
YMR095C	YMR096W	999
YMR095C	YNL333W	999
YMR095C	YNL334C	993
YMR095C	YNR027W	901
YMR095C	YPL240C	919
YMR096W	YBR035C	911
YMR096W	YFL059W	999
YMR096W	YFL060C	997
YMR096W	YGL186C	930
YMR096W	YHR029C	958
YMR096W	YMR095C	999
YMR096W	YNL333W	999
YMR096W	YNL334C	985
YMR096W	YNR027W	900
YMR099C	YJL208C	999
YMR099C	YKR079C	999
YMR100W	YDL020C	983
YMR100W	YGL058W	959
YMR100W	YLR024C	994
YMR101C	YBR002C	997
YMR101C	YJL167W	981
YMR101C	YMR013C	986
YMR104C	YDR490C	987
YMR104C	YER093C	951
YMR104C	YGL049C	979
YMR104C	YGR162W	979
YMR104C	YHR186C	973
YMR104C	YJR066W	975
YMR104C	YKL126W	999
YMR104C	YKL203C	999

YMR104C	YMR109W	904
YMR104C	YOL100W	922
YMR105C	YBR018C	922
YMR105C	YBR126C	911
YMR105C	YBR196C	923
YMR105C	YCL040W	962
YMR105C	YFR053C	964
YMR105C	YJL153C	909
YMR105C	YKL035W	932
YMR105C	YKL127W	984
YMR105C	YMR250W	976
YMR105C	YPR160W	960
YMR106C	YDL220C	999
YMR106C	YDR217C	913
YMR106C	YDR227W	957
YMR106C	YGL163C	909
YMR106C	YKR056W	976
YMR106C	YLR233C	980
YMR106C	YLR318W	999
YMR106C	YML032C	998
YMR106C	YML061C	980
YMR106C	YMR224C	998
YMR106C	YMR284W	999
YMR106C	YOR005C	983
YMR106C	YOR033C	982
YMR108W	YCL009C	999
YMR108W	YCL018W	995
YMR108W	YCL064C	901
YMR108W	YEL020C	916
YMR108W	YER086W	990
YMR108W	YGL009C	968
YMR108W	YHR208W	911
YMR108W	YJR016C	941
YMR108W	YJR148W	955
YMR108W	YLR355C	999
YMR108W	YNL104C	975
YMR109W	YBR109C	999
YMR109W	YBR234C	994
YMR109W	YCR094W	900
YMR109W	YDL019C	913
YMR109W	YDL029W	998
YMR109W	YDR208W	917
YMR109W	YDR388W	995
YMR109W	YFL039C	974

YMR109W	YGL242C	999
YMR109W	YHR114W	999
YMR109W	YIL062C	948
YMR109W	YJL020C	999
YMR109W	YKL013C	993
YMR109W	YKL129C	999
YMR109W	YLR337C	999
YMR109W	YLR370C	908
YMR109W	YLR429W	970
YMR109W	YMR104C	904
YMR109W	YNL271C	909
YMR109W	YNL298W	959
YMR109W	YNR035C	922
YMR109W	YOL100W	900
YMR109W	YOR035C	988
YMR109W	YOR181W	999
YMR111C	YDR510W	942
YMR112C	YBL093C	999
YMR112C	YBR193C	999
YMR112C	YBR253W	999
YMR112C	YCR081W	974
YMR112C	YDL005C	999
YMR112C	YDR308C	998
YMR112C	YDR443C	997
YMR112C	YER022W	999
YMR112C	YGL025C	998
YMR112C	YGL127C	992
YMR112C	YGL151W	997
YMR112C	YGR104C	999
YMR112C	YHR041C	998
YMR112C	YHR058C	998
YMR112C	YLR071C	999
YMR112C	YML007W	974
YMR112C	YNL236W	999
YMR112C	YNR010W	999
YMR112C	YOL051W	998
YMR112C	YOL135C	999
YMR112C	YOR174W	998
YMR112C	YPL042C	989
YMR112C	YPL129W	965
YMR112C	YPR070W	999
YMR112C	YPR168W	998
YMR113W	YBR084W	913
YMR113W	YGR094W	925

YMR113W	YGR204W	915
YMR113W	YNL256W	989
YMR113W	YOR236W	900
YMR116C	YBL072C	988
YMR116C	YBR189W	987
YMR116C	YDL040C	956
YMR116C	YDR064W	993
YMR116C	YDR283C	984
YMR116C	YGR229C	911
YMR116C	YGR285C	946
YMR116C	YHR013C	957
YMR116C	YJL080C	997
YMR116C	YJL190C	994
YMR116C	YJR123W	996
YMR116C	YJR145C	985
YMR116C	YLR367W	980
YMR116C	YOR096W	987
YMR116C	YPR189W	976
YMR117C	YAL034W-A	998
YMR117C	YBR107C	983
YMR117C	YCL029C	984
YMR117C	YDR254W	983
YMR117C	YDR318W	996
YMR117C	YER016W	983
YMR117C	YER018C	999
YMR117C	YER032W	939
YMR117C	YFR046C	993
YMR117C	YGL075C	995
YMR117C	YGL086W	983
YMR117C	YGR113W	983
YMR117C	YIL144W	999
YMR117C	YIR010W	982
YMR117C	YLR045C	984
YMR117C	YLR381W	983
YMR117C	YOL034W	979
YMR117C	YOL069W	999
YMR117C	YPL018W	983
YMR118C	YJL045W	927
YMR118C	YKL141W	903
YMR118C	YKL148C	928
YMR118C	YLL041C	939
YMR119W	YIR023W	914
YMR119W	YPL084W	901
YMR120C	YBR084W	908

YMR120C	YBR248C	919
YMR120C	YBR263W	906
YMR120C	YDR408C	979
YMR120C	YDR441C	902
YMR120C	YGL234W	914
YMR120C	YGR204W	956
YMR120C	YHR216W	901
YMR120C	YKL128C	907
YMR120C	YLR028C	999
YMR120C	YLR058C	917
YMR120C	YLR359W	972
YMR120C	YLR432W	910
YMR120C	YML022W	907
YMR120C	YML056C	901
YMR120C	YMR300C	977
YMR120C	YNL220W	943
YMR120C	YOR236W	902
YMR121C	YBL027W	933
YMR121C	YBL072C	915
YMR121C	YBL087C	942
YMR121C	YBL092W	909
YMR121C	YBR031W	949
YMR121C	YBR048W	914
YMR121C	YBR084C-A	933
YMR121C	YBR118W	936
YMR121C	YBR181C	923
YMR121C	YBR189W	922
YMR121C	YBR191W	917
YMR121C	YCR031C	925
YMR121C	YDL061C	911
YMR121C	YDL075W	910
YMR121C	YDL081C	917
YMR121C	YDL082W	932
YMR121C	YDL130W	907
YMR121C	YDL136W	929
YMR121C	YDL191W	929
YMR121C	YDR012W	949
YMR121C	YDR025W	914
YMR121C	YDR064W	941
YMR121C	YDR382W	935
YMR121C	YDR418W	928
YMR121C	YDR447C	920
YMR121C	YDR450W	908
YMR121C	YDR471W	912

YMR121C	YDR500C	913
YMR121C	YEL054C	928
YMR121C	YER056C-A	908
YMR121C	YER074W	915
YMR121C	YER102W	915
YMR121C	YER117W	927
YMR121C	YER131W	930
YMR121C	YFL034C-A	909
YMR121C	YFR031C-A	940
YMR121C	YFR032C-A	907
YMR121C	YGL030W	943
YMR121C	YGL031C	931
YMR121C	YGL076C	938
YMR121C	YGL103W	905
YMR121C	YGL123W	933
YMR121C	YGL135W	914
YMR121C	YGL147C	951
YMR121C	YGL189C	922
YMR121C	YGR027C	913
YMR121C	YGR034W	942
YMR121C	YGR085C	925
YMR121C	YGR118W	920
YMR121C	YGR148C	919
YMR121C	YGR214W	918
YMR121C	YHL001W	925
YMR121C	YHL015W	915
YMR121C	YHL033C	924
YMR121C	YHR010W	912
YMR121C	YHR021C	917
YMR121C	YHR141C	933
YMR121C	YHR203C	914
YMR121C	YIL018W	926
YMR121C	YIL052C	908
YMR121C	YIL069C	915
YMR121C	YIL133C	940
YMR121C	YJL136C	918
YMR121C	YJL177W	927
YMR121C	YJL190C	938
YMR121C	YJL191W	925
YMR121C	YJR094W-A	949
YMR121C	YJR123W	937
YMR121C	YJR145C	914
YMR121C	YKL006W	925
YMR121C	YKL156W	917

YMR121C	YKL180W	928
YMR121C	YKR057W	918
YMR121C	YLL045C	953
YMR121C	YLR029C	920
YMR121C	YLR048W	918
YMR121C	YLR061W	911
YMR121C	YLR075W	957
YMR121C	YLR185W	913
YMR121C	YLR264W	903
YMR121C	YLR287C-A	908
YMR121C	YLR325C	924
YMR121C	YLR333C	912
YMR121C	YLR340W	960
YMR121C	YLR344W	934
YMR121C	YLR367W	919
YMR121C	YLR388W	911
YMR121C	YLR406C	910
YMR121C	YLR441C	946
YMR121C	YLR448W	921
YMR121C	YML024W	920
YMR121C	YML026C	908
YMR121C	YML063W	936
YMR121C	YML073C	920
YMR121C	YMR142C	923
YMR121C	YMR194W	913
YMR121C	YMR230W	928
YMR121C	YMR242C	944
YMR121C	YMR260C	915
YMR121C	YNL067W	937
YMR121C	YNL069C	940
YMR121C	YNL096C	939
YMR121C	YNL162W	925
YMR121C	YNL178W	943
YMR121C	YNL301C	927
YMR121C	YNL302C	919
YMR121C	YOL039W	910
YMR121C	YOL040C	932
YMR121C	YOL120C	927
YMR121C	YOL121C	932
YMR121C	YOL127W	949
YMR121C	YOR063W	961
YMR121C	YOR096W	931
YMR121C	YOR167C	903
YMR121C	YOR182C	908

YMR121C	YOR234C	921
YMR121C	YOR293W	928
YMR121C	YOR312C	927
YMR121C	YOR369C	961
YMR121C	YPL079W	917
YMR121C	YPL081W	922
YMR121C	YPL131W	965
YMR121C	YPL143W	921
YMR121C	YPL198W	929
YMR121C	YPL220W	914
YMR121C	YPL249C-A	913
YMR121C	YPR043W	949
YMR121C	YPR080W	936
YMR121C	YPR102C	933
YMR121C	YPR132W	920
YMR123W	YBR290W	919
YMR123W	YGL203C	915
YMR123W	YGR105W	940
YMR123W	YIL030C	913
YMR123W	YJL004C	919
YMR123W	YJL117W	921
YMR123W	YMR200W	909
YMR123W	YOR070C	919
YMR123W	YOR270C	933
YMR123W	YPL051W	909
YMR123W	YPL195W	917
YMR124W	YER149C	923
YMR125W	YBR034C	997
YMR125W	YBR119W	999
YMR125W	YCL011C	976
YMR125W	YCL037C	995
YMR125W	YDL030W	994
YMR125W	YDL043C	975
YMR125W	YDL087C	999
YMR125W	YDL209C	982
YMR125W	YDR138W	963
YMR125W	YDR235W	999
YMR125W	YDR240C	999
YMR125W	YDR364C	966
YMR125W	YDR432W	999
YMR125W	YER029C	999
YMR125W	YER165W	992
YMR125W	YER172C	998
YMR125W	YFL017W-A	994

YMR125W	YGL049C	998
YMR125W	YGR013W	999
YMR125W	YGR074W	999
YMR125W	YGR162W	999
YMR125W	YHL034C	979
YMR125W	YHR086W	999
YMR125W	YHR165C	998
YMR125W	YIL061C	999
YMR125W	YIL115C	983
YMR125W	YJL190C	939
YMR125W	YKL012W	999
YMR125W	YKL173W	996
YMR125W	YKL214C	999
YMR125W	YLR117C	981
YMR125W	YLR147C	999
YMR125W	YLR150W	973
YMR125W	YLR226W	930
YMR125W	YLR275W	999
YMR125W	YLR298C	999
YMR125W	YLR347C	963
YMR125W	YLR424W	976
YMR125W	YML046W	992
YMR125W	YML049C	999
YMR125W	YMR240C	999
YMR125W	YNL139C	933
YMR125W	YNL189W	999
YMR125W	YNL251C	999
YMR125W	YOL139C	998
YMR125W	YOR116C	982
YMR125W	YOR159C	966
YMR125W	YPL151C	992
YMR125W	YPL178W	999
YMR125W	YPL190C	998
YMR125W	YPL213W	978
YMR125W	YPR161C	961
YMR125W	YPR182W	993
YMR127C	YBR009C	990
YMR127C	YBR060C	987
YMR127C	YDL042C	906
YMR127C	YDR181C	999
YMR127C	YJL115W	985
YMR127C	YKR101W	999
YMR127C	YNL030W	976
YMR127C	YNL261W	932

YMR127C	YOR213C	999
YMR128W	YBL004W	997
YMR128W	YBL072C	937
YMR128W	YBR247C	999
YMR128W	YCL059C	999
YMR128W	YCR057C	999
YMR128W	YDL014W	994
YMR128W	YDL076C	962
YMR128W	YDL148C	999
YMR128W	YDR064W	931
YMR128W	YDR280W	946
YMR128W	YDR299W	922
YMR128W	YDR324C	999
YMR128W	YDR449C	997
YMR128W	YER082C	997
YMR128W	YER102W	925
YMR128W	YGL120C	983
YMR128W	YGL171W	916
YMR128W	YGR090W	999
YMR128W	YGR128C	997
YMR128W	YHR148W	997
YMR128W	YHR169W	912
YMR128W	YIL084C	922
YMR128W	YJL109C	997
YMR128W	YJR002W	999
YMR128W	YJR145C	936
YMR128W	YKR060W	997
YMR128W	YLR129W	997
YMR128W	YLR186W	997
YMR128W	YLR197W	997
YMR128W	YLR222C	997
YMR128W	YMR093W	997
YMR128W	YMR229C	998
YMR128W	YMR290C	913
YMR128W	YNL075W	997
YMR128W	YNL097C	929
YMR128W	YNL132W	995
YMR128W	YOL010W	999
YMR128W	YOR039W	907
YMR128W	YOR078W	981
YMR128W	YOR096W	931
YMR128W	YOR310C	979
YMR128W	YPL012W	997
YMR128W	YPL126W	997

YMR128W	YPL217C	998
YMR128W	YPR112C	916
YMR128W	YPR137W	999
YMR128W	YPR144C	999
YMR129W	YBL079W	999
YMR129W	YDL088C	997
YMR129W	YDL116W	925
YMR129W	YDR159W	924
YMR129W	YER105C	998
YMR129W	YER107C	954
YMR129W	YER110C	930
YMR129W	YFR002W	999
YMR129W	YGL092W	928
YMR129W	YGL172W	968
YMR129W	YGR119C	970
YMR129W	YIL115C	942
YMR129W	YJL041W	941
YMR129W	YJL061W	957
YMR129W	YKL057C	973
YMR129W	YKL068W	928
YMR129W	YKR082W	979
YMR129W	YLR018C	992
YMR129W	YLR208W	928
YMR129W	YML031W	984
YMR129W	YML103C	999
YMR129W	YMR047C	982
YMR129W	YMR153W	995
YMR129W	YOR098C	988
YMR129W	YOR257W	961
YMR129W	YPL125W	925
YMR129W	YPL169C	915
YMR131C	YGR103W	991
YMR131C	YMR049C	957
YMR131C	YOR001W	934
YMR131C	YOR063W	999
YMR133W	YER044C-A	998
YMR133W	YHR014W	951
YMR133W	YHR157W	947
YMR133W	YJR021C	997
YMR134W	YJL117W	998
YMR135C	YBL049W	921
YMR135C	YBR105C	999
YMR135C	YCL039W	998
YMR135C	YDL176W	916

YMR135C	YDR255C	999
YMR135C	YGL227W	999
YMR135C	YIL017C	999
YMR135C	YIL097W	999
YMR135C	YLR361C	918
YMR137C	YGL058W	948
YMR137C	YML032C	922
YMR137C	YMR224C	983
YMR137C	YOL090W	981
YMR137C	YOR033C	983
YMR137C	YPL132W	945
YMR137C	YPL167C	957
YMR138W	YFL037W	989
YMR138W	YML085C	906
YMR138W	YOR349W	999
YMR138W	YPL241C	998
YMR139W	YDL079C	980
YMR139W	YDR162C	925
YMR139W	YDR207C	995
YMR139W	YJR094C	999
YMR139W	YKL127W	902
YMR139W	YKR048C	999
YMR139W	YMR037C	949
YMR139W	YNL307C	979
YMR140W	YDR477W	988
YMR140W	YER133W	949
YMR140W	YGL087C	995
YMR140W	YGR005C	998
YMR142C	YAL035W	924
YMR142C	YBL027W	921
YMR142C	YBL072C	926
YMR142C	YBL087C	936
YMR142C	YBL092W	942
YMR142C	YBR031W	940
YMR142C	YBR048W	912
YMR142C	YBR084C-A	921
YMR142C	YBR118W	909
YMR142C	YBR181C	919
YMR142C	YBR189W	958
YMR142C	YBR191W	952
YMR142C	YCR031C	997
YMR142C	YDL061C	922
YMR142C	YDL075W	927
YMR142C	YDL081C	929

YMR142C	YDL082W	998
YMR142C	YDL083C	965
YMR142C	YDL136W	995
YMR142C	YDL191W	914
YMR142C	YDR012W	939
YMR142C	YDR025W	912
YMR142C	YDR064W	998
YMR142C	YDR382W	932
YMR142C	YDR385W	914
YMR142C	YDR418W	945
YMR142C	YDR447C	997
YMR142C	YDR450W	998
YMR142C	YDR471W	996
YMR142C	YDR500C	906
YMR142C	YEL054C	918
YMR142C	YER056C-A	923
YMR142C	YER074W	995
YMR142C	YER102W	926
YMR142C	YER117W	928
YMR142C	YER131W	932
YMR142C	YFL034C-A	911
YMR142C	YFR031C-A	997
YMR142C	YFR032C-A	926
YMR142C	YGL030W	997
YMR142C	YGL031C	937
YMR142C	YGL076C	998
YMR142C	YGL103W	996
YMR142C	YGL123W	939
YMR142C	YGL135W	909
YMR142C	YGL147C	952
YMR142C	YGL189C	912
YMR142C	YGR027C	947
YMR142C	YGR034W	995
YMR142C	YGR085C	997
YMR142C	YGR118W	951
YMR142C	YGR148C	952
YMR142C	YGR214W	962
YMR142C	YHL001W	998
YMR142C	YHL015W	926
YMR142C	YHL033C	998
YMR142C	YHR010W	922
YMR142C	YHR021C	927
YMR142C	YHR141C	937
YMR142C	YHR203C	995

YMR142C	YHR216W	970
YMR142C	YIL018W	995
YMR142C	YIL052C	923
YMR142C	YIL069C	910
YMR142C	YIL133C	996
YMR142C	YIL148W	905
YMR142C	YJL136C	942
YMR142C	YJL177W	996
YMR142C	YJL189W	921
YMR142C	YJL190C	937
YMR142C	YJL191W	998
YMR142C	YJR094W-A	932
YMR142C	YJR123W	998
YMR142C	YJR145C	917
YMR142C	YKL006W	949
YMR142C	YKL156W	938
YMR142C	YKL180W	996
YMR142C	YKR057W	926
YMR142C	YKR094C	905
YMR142C	YLL045C	997
YMR142C	YLR029C	935
YMR142C	YLR048W	956
YMR142C	YLR061W	934
YMR142C	YLR075W	996
YMR142C	YLR167W	901
YMR142C	YLR185W	906
YMR142C	YLR264W	924
YMR142C	YLR325C	931
YMR142C	YLR333C	928
YMR142C	YLR340W	949
YMR142C	YLR344W	921
YMR142C	YLR367W	932
YMR142C	YLR388W	928
YMR142C	YLR406C	914
YMR142C	YLR441C	938
YMR142C	YLR448W	998
YMR142C	YML024W	957
YMR142C	YML026C	919
YMR142C	YML063W	998
YMR142C	YML073C	996
YMR142C	YMR121C	923
YMR142C	YMR194W	997
YMR142C	YMR230W	941
YMR142C	YMR242C	997

YMR142C	YMR260C	908
YMR142C	YNL067W	955
YMR142C	YNL069C	995
YMR142C	YNL096C	931
YMR142C	YNL162W	922
YMR142C	YNL178W	998
YMR142C	YNL301C	998
YMR142C	YNL302C	922
YMR142C	YOL039W	927
YMR142C	YOL040C	943
YMR142C	YOL120C	938
YMR142C	YOL121C	936
YMR142C	YOL127W	997
YMR142C	YOR063W	996
YMR142C	YOR096W	997
YMR142C	YOR133W	914
YMR142C	YOR167C	929
YMR142C	YOR204W	962
YMR142C	YOR234C	945
YMR142C	YOR293W	943
YMR142C	YOR312C	929
YMR142C	YOR369C	945
YMR142C	YPL079W	964
YMR142C	YPL081W	942
YMR142C	YPL090C	997
YMR142C	YPL131W	935
YMR142C	YPL143W	998
YMR142C	YPL198W	997
YMR142C	YPL220W	909
YMR142C	YPL249C-A	996
YMR142C	YPR043W	920
YMR142C	YPR080W	911
YMR142C	YPR102C	965
YMR142C	YPR132W	929
YMR143W	YJR123W	999
YMR143W	YMR194W	979
YMR145C	YDL085W	970
YMR145C	YIL155C	942
YMR146C	YAL035W	972
YMR146C	YBL072C	997
YMR146C	YBR079C	999
YMR146C	YBR181C	934
YMR146C	YCR031C	914
YMR146C	YDL061C	900

YMR146C	YDR091C	968
YMR146C	YDR429C	999
YMR146C	YDR450W	915
YMR146C	YER025W	999
YMR146C	YER102W	997
YMR146C	YHL015W	922
YMR146C	YJR007W	997
YMR146C	YJR123W	915
YMR146C	YLR192C	999
YMR146C	YLR388W	900
YMR146C	YMR309C	999
YMR146C	YNL062C	970
YMR146C	YNL096C	913
YMR146C	YNL244C	999
YMR146C	YOR204W	975
YMR146C	YOR361C	999
YMR146C	YPL081W	997
YMR146C	YPL090C	923
YMR146C	YPL105C	999
YMR146C	YPR041W	999
YMR146C	YPR086W	985
YMR149W	YBL105C	910
YMR149W	YDL232W	999
YMR149W	YDR086C	982
YMR149W	YEL002C	999
YMR149W	YGL022W	999
YMR149W	YGL226C-A	999
YMR149W	YJL002C	999
YMR149W	YLR378C	986
YMR149W	YML019W	999
YMR149W	YOR085W	999
YMR149W	YOR103C	999
YMR149W	YPL227C	948
YMR150C	YEL059C-A	990
YMR150C	YMR035W	997
YMR153W	YAR002W	942
YMR153W	YBL079W	999
YMR153W	YDL088C	992
YMR153W	YDL116W	952
YMR153W	YDL207W	974
YMR153W	YDR159W	966
YMR153W	YER105C	995
YMR153W	YFR002W	999
YMR153W	YGL086W	999

YMR153W	YGL100W	944
YMR153W	YGR119C	916
YMR153W	YJL030W	936
YMR153W	YJL039C	900
YMR153W	YJR042W	937
YMR153W	YKL057C	954
YMR153W	YKR082W	953
YMR153W	YLR018C	974
YMR153W	YLR335W	949
YMR153W	YLR347C	998
YMR153W	YML031W	988
YMR153W	YML103C	965
YMR153W	YMR047C	962
YMR153W	YMR129W	995
YMR153W	YMR308C	999
YMR153W	YOR032C	998
YMR153W	YOR257W	961
YMR153W	YPL020C	984
YMR153W	YPL204W	971
YMR154C	YHL027W	991
YMR154C	YOR362C	999
YMR154C	YPL240C	919
YMR156C	YBL019W	948
YMR156C	YKL114C	997
YMR156C	YPL022W	980
YMR157C	YPL240C	919
YMR158W	Q0140	962
YMR158W	YBL090W	974
YMR158W	YBR146W	974
YMR158W	YBR251W	974
YMR158W	YDR041W	974
YMR158W	YDR175C	974
YMR158W	YDR337W	974
YMR158W	YDR347W	974
YMR158W	YGL129C	974
YMR158W	YGR084C	974
YMR158W	YGR165W	997
YMR158W	YGR215W	972
YMR158W	YHL004W	999
YMR158W	YIL093C	974
YMR158W	YJR101W	974
YMR158W	YJR113C	974
YMR158W	YKL155C	974
YMR158W	YMR188C	997

YMR158W	YNL137C	974
YMR158W	YNL306W	974
YMR158W	YNR036C	980
YMR158W	YOR158W	974
YMR158W	YPL013C	999
YMR158W	YPL118W	974
YMR159C	YBL078C	940
YMR159C	YBR217W	999
YMR159C	YHR171W	930
YMR159C	YPL149W	999
YMR161W	YLL055W	923
YMR163C	YOR326W	978
YMR164C	YBR083W	991
YMR164C	YER109C	999
YMR164C	YHR084W	989
YMR164C	YNL142W	987
YMR164C	YOL116W	998
YMR164C	YOR140W	930
YMR165C	YAL009W	914
YMR165C	YHL020C	911
YMR165C	YHR004C	942
YMR165C	YOR311C	928
YMR167W	YBR088C	987
YMR167W	YCR092C	998
YMR167W	YDL102W	979
YMR167W	YDL154W	961
YMR167W	YDR097C	999
YMR167W	YER095W	924
YMR167W	YFL003C	974
YMR167W	YLR035C	999
YMR167W	YML032C	973
YMR167W	YMR190C	999
YMR167W	YNL082W	999
YMR167W	YOL090W	999
YMR167W	YOR033C	999
YMR167W	YPL022W	935
YMR167W	YPL164C	999
YMR168C	YDL064W	972
YMR168C	YDR254W	994
YMR168C	YDR318W	954
YMR168C	YDR328C	999
YMR168C	YGR113W	983
YMR168C	YGR140W	999
YMR168C	YGR179C	987

YMR168C	YGR188C	964
YMR168C	YIL144W	954
YMR168C	YJL030W	984
YMR168C	YJR060W	999
YMR168C	YJR112W	976
YMR168C	YKL049C	958
YMR168C	YKL089W	998
YMR168C	YLR381W	964
YMR168C	YMR094W	999
YMR168C	YOR195W	924
YMR168C	YPL018W	989
YMR168C	YPL209C	913
YMR169C	YBR145W	900
YMR169C	YDL168W	915
YMR169C	YER073W	910
YMR169C	YGL256W	956
YMR169C	YLR044C	930
YMR169C	YLR153C	901
YMR169C	YMR020W	948
YMR169C	YMR083W	900
YMR169C	YMR170C	997
YMR169C	YMR303C	901
YMR169C	YOL086C	900
YMR169C	YOR374W	909
YMR170C	YAL054C	900
YMR170C	YBR145W	900
YMR170C	YDL168W	915
YMR170C	YER073W	909
YMR170C	YGL256W	924
YMR170C	YLR153C	939
YMR170C	YMR020W	946
YMR170C	YMR083W	900
YMR170C	YMR169C	997
YMR170C	YMR303C	901
YMR170C	YOL086C	900
YMR170C	YOR374W	909
YMR172W	YER164W	968
YMR172W	YGL019W	996
YMR172W	YIL035C	997
YMR172W	YLR113W	992
YMR172W	YNL330C	926
YMR172W	YOR039W	999
YMR172W	YOR061W	987
YMR174C	YPL154C	999

YMR176W	YFR006W	907
YMR176W	YGL131C	943
YMR177W	YDR244W	999
YMR177W	YJR104C	903
YMR179W	YJL127C	957
YMR181C	YBR087W	903
YMR183C	YAL030W	999
YMR183C	YDR164C	999
YMR183C	YER008C	993
YMR183C	YGR009C	999
YMR183C	YMR017W	980
YMR183C	YNR049C	934
YMR183C	YOR327C	999
YMR183C	YPL232W	995
YMR186W	YAL005C	959
YMR186W	YAL029C	933
YMR186W	YAL059W	939
YMR186W	YBL051C	919
YMR186W	YBR109C	932
YMR186W	YBR155W	999
YMR186W	YBR196C	972
YMR186W	YCL014W	907
YMR186W	YCR077C	963
YMR186W	YDL229W	925
YMR186W	YDR127W	996
YMR186W	YDR168W	999
YMR186W	YDR214W	999
YMR186W	YDR283C	986
YMR186W	YDR510W	939
YMR186W	YER148W	963
YMR186W	YFL021W	919
YMR186W	YGL073W	999
YMR186W	YGR123C	991
YMR186W	YIL005W	955
YMR186W	YIL021W	963
YMR186W	YJL138C	960
YMR186W	YJR032W	999
YMR186W	YKL117W	999
YMR186W	YKL126W	971
YMR186W	YLL024C	966
YMR186W	YLR216C	999
YMR186W	YLR249W	986
YMR186W	YLR259C	909
YMR186W	YLR262C	963

YMR186W	YNL064C	970
YMR186W	YNL281W	997
YMR186W	YNL307C	919
YMR186W	YNR032W	971
YMR186W	YOL086C	991
YMR186W	YOL090W	939
YMR186W	YOR007C	995
YMR186W	YOR027W	999
YMR186W	YOR057W	996
YMR186W	YOR332W	931
YMR186W	YPL240C	999
YMR187C	YPL048W	935
YMR188C	Q0140	962
YMR188C	YBL090W	988
YMR188C	YBR146W	989
YMR188C	YBR251W	988
YMR188C	YDR036C	999
YMR188C	YDR041W	998
YMR188C	YDR175C	988
YMR188C	YDR337W	988
YMR188C	YDR347W	988
YMR188C	YGL129C	988
YMR188C	YGR084C	988
YMR188C	YGR165W	995
YMR188C	YGR215W	987
YMR188C	YHL004W	999
YMR188C	YIL093C	988
YMR188C	YJR101W	988
YMR188C	YJR113C	988
YMR188C	YKL155C	988
YMR188C	YMR158W	997
YMR188C	YNL137C	988
YMR188C	YNL306W	988
YMR188C	YNR037C	997
YMR188C	YOR158W	988
YMR188C	YPL013C	963
YMR188C	YPL118W	988
YMR189W	YAL044C	999
YMR189W	YBR263W	962
YMR189W	YDR019C	999
YMR189W	YEL046C	900
YMR189W	YFL018C	998
YMR189W	YGL125W	916
YMR189W	YKR080W	903

YMR189W	YLR058C	976
YMR189W	YPL017C	935
YMR189W	YPL023C	907
YMR190C	YBL088C	903
YMR190C	YBR060C	915
YMR190C	YBR094W	946
YMR190C	YBR098W	999
YMR190C	YBR114W	979
YMR190C	YBR228W	996
YMR190C	YCL061C	998
YMR190C	YDL013W	999
YMR190C	YDL154W	945
YMR190C	YDR004W	975
YMR190C	YDR078C	978
YMR190C	YDR110W	975
YMR190C	YDR217C	993
YMR190C	YDR279W	962
YMR190C	YDR363W	977
YMR190C	YDR386W	999
YMR190C	YER095W	999
YMR190C	YER116C	999
YMR190C	YER173W	999
YMR190C	YFL023W	900
YMR190C	YGL163C	945
YMR190C	YGL175C	935
YMR190C	YHL006C	979
YMR190C	YHR031C	999
YMR190C	YHR134W	983
YMR190C	YHR154W	999
YMR190C	YHR164C	999
YMR190C	YIL132C	960
YMR190C	YIR002C	911
YMR190C	YJL092W	999
YMR190C	YJL115W	999
YMR190C	YJR043C	990
YMR190C	YKL113C	998
YMR190C	YLR135W	999
YMR190C	YLR234W	999
YMR190C	YLR288C	994
YMR190C	YLR318W	996
YMR190C	YLR376C	977
YMR190C	YML028W	973
YMR190C	YML032C	999
YMR190C	YML061C	997

YMR190C	YMR048W	970
YMR190C	YMR167W	999
YMR190C	YMR224C	982
YMR190C	YMR284W	996
YMR190C	YNL088W	979
YMR190C	YNL218W	995
YMR190C	YNL250W	997
YMR190C	YOL006C	999
YMR190C	YOL090W	997
YMR190C	YOR033C	997
YMR190C	YOR080W	931
YMR190C	YOR144C	988
YMR190C	YOR368W	994
YMR190C	YPL022W	988
YMR190C	YPL024W	999
YMR190C	YPL153C	998
YMR190C	YPL164C	998
YMR190C	YPR018W	969
YMR190C	YPR164W	962
YMR191W	YDL007W	987
YMR191W	YDL147W	987
YMR191W	YDR363W-A	987
YMR191W	YDR394W	987
YMR191W	YDR427W	987
YMR191W	YER021W	972
YMR191W	YFR004W	987
YMR191W	YFR052W	987
YMR191W	YGL048C	987
YMR191W	YHR027C	972
YMR191W	YHR200W	987
YMR191W	YIL075C	987
YMR191W	YKL145W	987
YMR191W	YLR421C	987
YMR191W	YOR117W	987
YMR191W	YOR259C	987
YMR191W	YOR261C	987
YMR191W	YPR108W	987
YMR192W	YCR009C	995
YMR192W	YDR388W	999
YMR192W	YPL249C	996
YMR193W	YBL038W	972
YMR193W	YBR122C	972
YMR193W	YBR282W	972
YMR193W	YCR046C	992

YMR193W	YCR071C	972
YMR193W	YDR116C	974
YMR193W	YDR237W	974
YMR193W	YDR296W	974
YMR193W	YDR322W	976
YMR193W	YGR220C	997
YMR193W	YHR147C	968
YMR193W	YJL041W	934
YMR193W	YJL063C	972
YMR193W	YKL167C	972
YMR193W	YKR006C	972
YMR193W	YKR085C	988
YMR193W	YLR312W-A	978
YMR193W	YLR439W	974
YMR193W	YML009C	970
YMR193W	YML025C	991
YMR193W	YMR024W	972
YMR193W	YNL005C	978
YMR193W	YNL252C	989
YMR193W	YNL284C	913
YMR194W	YAL035W	938
YMR194W	YBL027W	995
YMR194W	YBL072C	995
YMR194W	YBL087C	929
YMR194W	YBL092W	997
YMR194W	YBR031W	997
YMR194W	YBR048W	997
YMR194W	YBR084C-A	983
YMR194W	YBR118W	908
YMR194W	YBR181C	995
YMR194W	YBR189W	999
YMR194W	YBR191W	999
YMR194W	YCR031C	999
YMR194W	YDL061C	994
YMR194W	YDL075W	999
YMR194W	YDL081C	938
YMR194W	YDL082W	996
YMR194W	YDL083C	969
YMR194W	YDL130W	943
YMR194W	YDL136W	998
YMR194W	YDL191W	926
YMR194W	YDL192W	938
YMR194W	YDR012W	998
YMR194W	YDR025W	913

YMR194W	YDR064W	999
YMR194W	YDR382W	999
YMR194W	YDR385W	911
YMR194W	YDR418W	999
YMR194W	YDR447C	996
YMR194W	YDR450W	996
YMR194W	YDR471W	998
YMR194W	YDR500C	994
YMR194W	YEL054C	924
YMR194W	YER056C-A	999
YMR194W	YER074W	996
YMR194W	YER102W	983
YMR194W	YER117W	982
YMR194W	YER131W	998
YMR194W	YFL034C-A	926
YMR194W	YFR031C-A	995
YMR194W	YFR032C-A	997
YMR194W	YGL014W	950
YMR194W	YGL030W	999
YMR194W	YGL031C	996
YMR194W	YGL076C	997
YMR194W	YGL099W	985
YMR194W	YGL103W	999
YMR194W	YGL123W	995
YMR194W	YGL135W	996
YMR194W	YGL147C	996
YMR194W	YGL189C	920
YMR194W	YGR027C	982
YMR194W	YGR034W	995
YMR194W	YGR085C	998
YMR194W	YGR118W	999
YMR194W	YGR145W	925
YMR194W	YGR148C	994
YMR194W	YGR214W	993
YMR194W	YHL001W	999
YMR194W	YHL015W	981
YMR194W	YHL033C	999
YMR194W	YHR010W	998
YMR194W	YHR021C	930
YMR194W	YHR141C	996
YMR194W	YHR203C	999
YMR194W	YIL018W	996
YMR194W	YIL052C	999
YMR194W	YIL069C	922

YMR194W	YIL133C	996
YMR194W	YIL148W	980
YMR194W	YJL136C	973
YMR194W	YJL177W	999
YMR194W	YJL189W	974
YMR194W	YJL190C	996
YMR194W	YJL191W	988
YMR194W	YJR094W-A	999
YMR194W	YJR123W	999
YMR194W	YJR145C	925
YMR194W	YKL006W	999
YMR194W	YKL156W	996
YMR194W	YKL180W	999
YMR194W	YKR057W	934
YMR194W	YKR094C	905
YMR194W	YLL045C	995
YMR194W	YLR029C	998
YMR194W	YLR048W	997
YMR194W	YLR061W	999
YMR194W	YLR075W	999
YMR194W	YLR167W	977
YMR194W	YLR185W	993
YMR194W	YLR264W	985
YMR194W	YLR287C-A	995
YMR194W	YLR325C	930
YMR194W	YLR333C	939
YMR194W	YLR340W	999
YMR194W	YLR344W	999
YMR194W	YLR367W	999
YMR194W	YLR388W	999
YMR194W	YLR406C	921
YMR194W	YLR441C	996
YMR194W	YLR448W	999
YMR194W	YML024W	999
YMR194W	YML026C	917
YMR194W	YML063W	999
YMR194W	YML073C	996
YMR194W	YMR121C	913
YMR194W	YMR142C	997
YMR194W	YMR143W	979
YMR194W	YMR230W	965
YMR194W	YMR242C	996
YMR194W	YNL067W	980
YMR194W	YNL069C	996

YMR194W	YNL096C	995
YMR194W	YNL132W	945
YMR194W	YNL162W	931
YMR194W	YNL178W	999
YMR194W	YNL301C	998
YMR194W	YNL302C	998
YMR194W	YOL039W	925
YMR194W	YOL040C	997
YMR194W	YOL120C	921
YMR194W	YOL121C	926
YMR194W	YOL127W	999
YMR194W	YOR063W	998
YMR194W	YOR096W	999
YMR194W	YOR133W	937
YMR194W	YOR167C	945
YMR194W	YOR182C	902
YMR194W	YOR234C	995
YMR194W	YOR293W	980
YMR194W	YOR312C	997
YMR194W	YOR369C	936
YMR194W	YPL037C	999
YMR194W	YPL079W	966
YMR194W	YPL081W	950
YMR194W	YPL090C	902
YMR194W	YPL131W	994
YMR194W	YPL143W	995
YMR194W	YPL198W	996
YMR194W	YPL220W	998
YMR194W	YPL249C-A	953
YMR194W	YPR043W	966
YMR194W	YPR080W	908
YMR194W	YPR102C	982
YMR194W	YPR132W	995
YMR195W	YOR297C	940
YMR196W	YHR146W	929
YMR197C	YAL014C	982
YMR197C	YAL030W	962
YMR197C	YBL050W	999
YMR197C	YBR080C	910
YMR197C	YDL077C	938
YMR197C	YDR080W	934
YMR197C	YDR468C	999
YMR197C	YGL095C	980
YMR197C	YGL212W	999

YMR197C	YKL006C-A	933
YMR197C	YKL196C	999
YMR197C	YLR026C	999
YMR197C	YLR093C	999
YMR197C	YLR148W	996
YMR197C	YLR396C	980
YMR197C	YOL018C	999
YMR197C	YOR036W	999
YMR197C	YOR106W	999
YMR197C	YOR327C	999
YMR197C	YOR359W	903
YMR197C	YPL045W	982
YMR198W	YCL061C	915
YMR198W	YDR022C	909
YMR198W	YEL061C	951
YMR198W	YGL216W	924
YMR198W	YLR371W	925
YMR198W	YNL090W	933
YMR198W	YOL149W	960
YMR198W	YPL253C	984
YMR198W	YPR141C	999
YMR199W	YAL040C	997
YMR199W	YBL016W	909
YMR199W	YBR083W	948
YMR199W	YBR110W	918
YMR199W	YBR135W	999
YMR199W	YBR136W	976
YMR199W	YBR160W	999
YMR199W	YDL132W	987
YMR199W	YDR054C	918
YMR199W	YDR328C	976
YMR199W	YER111C	957
YMR199W	YER167W	970
YMR199W	YHR030C	909
YMR199W	YJL157C	985
YMR199W	YJL187C	941
YMR199W	YJR090C	999
YMR199W	YLR079W	999
YMR199W	YLR182W	965
YMR199W	YLR362W	914
YMR199W	YNL298W	976
YMR199W	YOL133W	918
YMR199W	YPL031C	981
YMR199W	YPL256C	999

YMR200W	YBR229C	975
YMR200W	YDR389W	983
YMR200W	YHR101C	969
YMR200W	YJL034W	976
YMR200W	YKL203C	900
YMR200W	YMR013C	911
YMR200W	YMR123W	909
YMR200W	YOR085W	909
YMR201C	YAL015C	905
YMR201C	YAR007C	989
YMR201C	YBL019W	981
YMR201C	YBR114W	996
YMR201C	YDR311W	997
YMR201C	YDR419W	982
YMR201C	YDR460W	981
YMR201C	YEL037C	998
YMR201C	YER162C	999
YMR201C	YER171W	999
YMR201C	YGR258C	998
YMR201C	YIL011W	904
YMR201C	YIL128W	958
YMR201C	YIL143C	993
YMR201C	YIR002C	954
YMR201C	YJR035W	951
YMR201C	YJR052W	994
YMR201C	YKL032C	901
YMR201C	YKL114C	998
YMR201C	YLR005W	981
YMR201C	YML060W	915
YMR201C	YML095C	999
YMR201C	YNL312W	998
YMR201C	YOL043C	926
YMR201C	YOR346W	985
YMR201C	YPL022W	999
YMR201C	YPL167C	998
YMR202W	YCR034W	912
YMR202W	YDR213W	995
YMR202W	YER044C	999
YMR202W	YGL001C	998
YMR202W	YGR060W	999
YMR202W	YGR175C	998
YMR202W	YHR007C	999
YMR202W	YLR056W	971
YMR202W	YLR228C	995

YMR202W	YLR372W	998
YMR202W	YML008C	999
YMR202W	YNL280C	999
YMR203W	YAL010C	944
YMR203W	YGR033C	963
YMR203W	YGR082W	999
YMR203W	YJL066C	980
YMR203W	YKL067W	965
YMR203W	YML091C	997
YMR203W	YNL026W	910
YMR203W	YNL055C	971
YMR203W	YNL070W	989
YMR203W	YNL121C	999
YMR203W	YNL131W	999
YMR203W	YNR017W	989
YMR203W	YOR045W	999
YMR203W	YPR133W-A	999
YMR204C	YKR001C	980
YMR204C	YLR324W	985
YMR204C	YPL112C	984
YMR205C	YAL038W	948
YMR205C	YBR196C	962
YMR205C	YBR218C	910
YMR205C	YFR053C	909
YMR205C	YGL062W	910
YMR205C	YGL253W	906
YMR205C	YGR240C	999
YMR205C	YIL107C	959
YMR205C	YJL155C	986
YMR205C	YKL060C	947
YMR205C	YKR097W	900
YMR205C	YLR377C	928
YMR205C	YOL136C	957
YMR205C	YPR074C	912
YMR207C	YGL115W	914
YMR207C	YNR016C	987
YMR208W	YLR450W	906
YMR208W	YML075C	905
YMR208W	YMR220W	959
YMR209C	YMR243C	998
YMR210W	YKL142W	958
YMR211W	YKL190W	911
YMR213W	YAL032C	999
YMR213W	YBR065C	997

YMR213W	YBR188C	999
YMR213W	YCR063W	999
YMR213W	YDL209C	999
YMR213W	YDR364C	995
YMR213W	YDR416W	999
YMR213W	YER013W	989
YMR213W	YER172C	996
YMR213W	YGL120C	919
YMR213W	YGL128C	999
YMR213W	YGR129W	998
YMR213W	YGR278W	999
YMR213W	YHR165C	999
YMR213W	YJR050W	999
YMR213W	YKL095W	948
YMR213W	YKL173W	999
YMR213W	YLL036C	999
YMR213W	YLR117C	999
YMR213W	YLR147C	997
YMR213W	YLR275W	961
YMR213W	YML049C	999
YMR213W	YMR288W	999
YMR213W	YPL151C	999
YMR213W	YPL213W	953
YMR213W	YPR101W	999
YMR214W	YBR162C	994
YMR214W	YDR320C	924
YMR214W	YFR041C	900
YMR214W	YGL018C	917
YMR214W	YGL027C	928
YMR214W	YJL034W	999
YMR214W	YJL073W	998
YMR214W	YKL073W	980
YMR214W	YOR085W	981
YMR214W	YOR232W	932
YMR214W	YPL240C	931
YMR216C	YDR394W	998
YMR216C	YDR432W	999
YMR216C	YJR059W	957
YMR216C	YNL004W	936
YMR216C	YOR160W	950
YMR217W	YBL039C	932
YMR217W	YDL171C	924
YMR217W	YDR441C	909
YMR217W	YDR454C	919

YMR217W	YHR216W	988
YMR217W	YJL130C	911
YMR217W	YJR109C	956
YMR217W	YKL104C	922
YMR217W	YLR432W	989
YMR217W	YML022W	918
YMR217W	YML056C	991
YMR217W	YMR300C	986
YMR217W	YOR168W	914
YMR217W	YOR303W	982
YMR218C	YBR254C	999
YMR218C	YDR108W	999
YMR218C	YDR246W	999
YMR218C	YDR407C	999
YMR218C	YDR472W	999
YMR218C	YER031C	999
YMR218C	YFL038C	932
YMR218C	YGL210W	986
YMR218C	YGR166W	999
YMR218C	YKR068C	999
YMR218C	YML077W	999
YMR218C	YOR115C	999
YMR219W	YDR096W	905
YMR219W	YFL023W	928
YMR220W	YMR208W	959
YMR220W	YNR043W	969
YMR223W	YBR081C	999
YMR223W	YBR111W-A	999
YMR223W	YBR198C	999
YMR223W	YCL010C	999
YMR223W	YDR145W	999
YMR223W	YDR167W	999
YMR223W	YDR176W	999
YMR223W	YDR392W	999
YMR223W	YDR448W	999
YMR223W	YER164W	980
YMR223W	YGL066W	999
YMR223W	YGL112C	999
YMR223W	YGR240C	902
YMR223W	YGR252W	999
YMR223W	YHR099W	999
YMR223W	YLR055C	999
YMR223W	YMR236W	999
YMR223W	YOL148C	999

YMR223W	YPL047W	999
YMR223W	YPL254W	999
YMR224C	YAR007C	990
YMR224C	YBL088C	999
YMR224C	YBR073W	994
YMR224C	YBR136W	963
YMR224C	YDL059C	970
YMR224C	YDL220C	990
YMR224C	YDR004W	993
YMR224C	YDR076W	964
YMR224C	YDR369C	999
YMR224C	YER095W	973
YMR224C	YGL163C	973
YMR224C	YHR031C	987
YMR224C	YJL092W	959
YMR224C	YKL113C	999
YMR224C	YKR082W	954
YMR224C	YML028W	972
YMR224C	YML032C	982
YMR224C	YML061C	994
YMR224C	YML095C	902
YMR224C	YMR106C	998
YMR224C	YMR137C	983
YMR224C	YMR190C	982
YMR224C	YMR284W	996
YMR224C	YNL250W	999
YMR224C	YOR005C	975
YMR224C	YOR033C	999
YMR224C	YOR144C	995
YMR224C	YPL020C	966
YMR224C	YPL022W	998
YMR225C	YNL284C	925
YMR227C	YAL017W	903
YMR227C	YBR081C	988
YMR227C	YBR198C	999
YMR227C	YCR042C	999
YMR227C	YDL070W	928
YMR227C	YDR145W	999
YMR227C	YDR167W	999
YMR227C	YDR176W	988
YMR227C	YER148W	999
YMR227C	YGL112C	999
YMR227C	YGR252W	997
YMR227C	YGR274C	999

YMR227C	YHR041C	911
YMR227C	YHR099W	923
YMR227C	YLR055C	988
YMR227C	YLR182W	923
YMR227C	YLR399C	997
YMR227C	YML015C	999
YMR227C	YML098W	999
YMR227C	YML114C	999
YMR227C	YMR005W	999
YMR227C	YMR043W	923
YMR227C	YMR236W	999
YMR227C	YOL051W	959
YMR227C	YOR174W	972
YMR227C	YPL011C	999
YMR227C	YPL129W	991
YMR227C	YPL254W	988
YMR228W	YFL036W	999
YMR229C	YBL004W	997
YMR229C	YBL072C	937
YMR229C	YBR048W	925
YMR229C	YBR142W	984
YMR229C	YBR247C	976
YMR229C	YCL059C	999
YMR229C	YCR057C	983
YMR229C	YDL014W	998
YMR229C	YDL148C	999
YMR229C	YDL153C	918
YMR229C	YDR060W	999
YMR229C	YDR280W	959
YMR229C	YDR299W	914
YMR229C	YDR324C	972
YMR229C	YDR398W	917
YMR229C	YDR449C	972
YMR229C	YDR496C	984
YMR229C	YEL026W	919
YMR229C	YER082C	972
YMR229C	YER102W	923
YMR229C	YER165W	940
YMR229C	YGL120C	975
YMR229C	YGL135W	935
YMR229C	YGL171W	995
YMR229C	YGR081C	954
YMR229C	YGR090W	999
YMR229C	YGR103W	986

YMR229C	YGR128C	996
YMR229C	YHR052W	999
YMR229C	YHR148W	911
YMR229C	YHR169W	921
YMR229C	YHR196W	972
YMR229C	YIL035C	933
YMR229C	YJL010C	985
YMR229C	YJL069C	941
YMR229C	YJL109C	989
YMR229C	YJR002W	994
YMR229C	YJR145C	936
YMR229C	YKR024C	977
YMR229C	YLL008W	989
YMR229C	YLL011W	972
YMR229C	YLR003C	924
YMR229C	YLR029C	944
YMR229C	YLR129W	965
YMR229C	YLR175W	999
YMR229C	YLR186W	921
YMR229C	YLR197W	998
YMR229C	YLR222C	949
YMR229C	YLR409C	937
YMR229C	YML093W	933
YMR229C	YMR049C	990
YMR229C	YMR093W	924
YMR229C	YMR128W	998
YMR229C	YMR239C	904
YMR229C	YMR290C	999
YMR229C	YNL061W	996
YMR229C	YNL075W	929
YMR229C	YNL132W	997
YMR229C	YNL175C	956
YMR229C	YOL041C	985
YMR229C	YOL077C	999
YMR229C	YOL080C	943
YMR229C	YOR004W	953
YMR229C	YOR078W	972
YMR229C	YOR145C	931
YMR229C	YOR206W	999
YMR229C	YOR272W	991
YMR229C	YOR310C	989
YMR229C	YPL012W	998
YMR229C	YPL126W	999
YMR229C	YPL217C	936

YMR229C	YPR112C	919
YMR229C	YPR137W	972
YMR229C	YPR144C	975
YMR230W	YBL027W	918
YMR230W	YBL072C	929
YMR230W	YBL087C	925
YMR230W	YBL092W	948
YMR230W	YBR031W	946
YMR230W	YBR048W	918
YMR230W	YBR084C-A	918
YMR230W	YBR118W	909
YMR230W	YBR181C	901
YMR230W	YBR189W	960
YMR230W	YBR191W	961
YMR230W	YCL037C	972
YMR230W	YCR031C	943
YMR230W	YDL061C	979
YMR230W	YDL075W	953
YMR230W	YDL081C	934
YMR230W	YDL082W	938
YMR230W	YDL083C	982
YMR230W	YDL130W	913
YMR230W	YDL136W	937
YMR230W	YDL191W	929
YMR230W	YDR012W	946
YMR230W	YDR025W	918
YMR230W	YDR064W	999
YMR230W	YDR382W	949
YMR230W	YDR385W	910
YMR230W	YDR418W	954
YMR230W	YDR429C	923
YMR230W	YDR447C	932
YMR230W	YDR450W	917
YMR230W	YDR471W	943
YMR230W	YDR500C	928
YMR230W	YEL054C	934
YMR230W	YER025W	913
YMR230W	YER056C-A	944
YMR230W	YER074W	920
YMR230W	YER102W	929
YMR230W	YER117W	933
YMR230W	YER131W	951
YMR230W	YER165W	902
YMR230W	YFL034C-A	917

YMR230W	YFR031C-A	904
YMR230W	YFR032C-A	940
YMR230W	YGL030W	968
YMR230W	YGL031C	952
YMR230W	YGL076C	956
YMR230W	YGL103W	953
YMR230W	YGL123W	938
YMR230W	YGL135W	926
YMR230W	YGL147C	934
YMR230W	YGL189C	925
YMR230W	YGR027C	954
YMR230W	YGR034W	927
YMR230W	YGR085C	961
YMR230W	YGR118W	964
YMR230W	YGR148C	948
YMR230W	YGR162W	916
YMR230W	YGR214W	961
YMR230W	YHL001W	960
YMR230W	YHL015W	998
YMR230W	YHL033C	947
YMR230W	YHR010W	954
YMR230W	YHR021C	931
YMR230W	YHR141C	942
YMR230W	YHR203C	928
YMR230W	YIL018W	904
YMR230W	YIL052C	952
YMR230W	YIL069C	920
YMR230W	YIL133C	928
YMR230W	YIL148W	915
YMR230W	YJL136C	950
YMR230W	YJL177W	946
YMR230W	YJL189W	924
YMR230W	YJL190C	998
YMR230W	YJL191W	946
YMR230W	YJR094W-A	955
YMR230W	YJR123W	999
YMR230W	YJR145C	928
YMR230W	YKL006W	965
YMR230W	YKL156W	949
YMR230W	YKL180W	950
YMR230W	YKR057W	938
YMR230W	YKR094C	905
YMR230W	YLL045C	938
YMR230W	YLR029C	945

YMR230W	YLR048W	957
YMR230W	YLR061W	951
YMR230W	YLR075W	947
YMR230W	YLR167W	900
YMR230W	YLR185W	920
YMR230W	YLR264W	959
YMR230W	YLR287C-A	906
YMR230W	YLR325C	928
YMR230W	YLR333C	935
YMR230W	YLR340W	951
YMR230W	YLR344W	944
YMR230W	YLR367W	984
YMR230W	YLR388W	953
YMR230W	YLR406C	923
YMR230W	YLR441C	929
YMR230W	YLR448W	952
YMR230W	YML024W	967
YMR230W	YML026C	917
YMR230W	YML063W	963
YMR230W	YML073C	929
YMR230W	YMR121C	928
YMR230W	YMR142C	941
YMR230W	YMR194W	965
YMR230W	YMR242C	934
YMR230W	YMR260C	915
YMR230W	YNL067W	959
YMR230W	YNL069C	927
YMR230W	YNL096C	935
YMR230W	YNL162W	931
YMR230W	YNL178W	999
YMR230W	YNL301C	928
YMR230W	YNL302C	927
YMR230W	YOL039W	918
YMR230W	YOL040C	948
YMR230W	YOL120C	920
YMR230W	YOL121C	927
YMR230W	YOL127W	965
YMR230W	YOL139C	930
YMR230W	YOR063W	944
YMR230W	YOR096W	966
YMR230W	YOR133W	910
YMR230W	YOR167C	962
YMR230W	YOR182C	906
YMR230W	YOR234C	967

YMR230W	YOR293W	993
YMR230W	YOR312C	934
YMR230W	YOR361C	912
YMR230W	YOR369C	953
YMR230W	YPL079W	965
YMR230W	YPL081W	939
YMR230W	YPL090C	901
YMR230W	YPL131W	940
YMR230W	YPL143W	962
YMR230W	YPL198W	936
YMR230W	YPL220W	926
YMR230W	YPL237W	910
YMR230W	YPL249C-A	931
YMR230W	YPR043W	931
YMR230W	YPR080W	909
YMR230W	YPR102C	970
YMR230W	YPR132W	939
YMR231W	YAL002W	999
YMR231W	YDL077C	999
YMR231W	YDR080W	999
YMR231W	YDR323C	989
YMR231W	YDR495C	992
YMR231W	YGL212W	992
YMR231W	YLR148W	999
YMR231W	YLR396C	999
YMR231W	YOR106W	905
YMR231W	YPL045W	999
YMR232W	YAL041W	998
YMR232W	YBL016W	998
YMR232W	YBR200W	957
YMR232W	YCL027W	999
YMR232W	YCR009C	999
YMR232W	YDL159W	998
YMR232W	YER149C	998
YMR232W	YIL159W	998
YMR232W	YLL021W	976
YMR232W	YLR330W	999
YMR232W	YLR362W	998
YMR232W	YNL271C	998
YMR233W	YLR234W	999
YMR233W	YOL006C	956
YMR234W	YAR007C	909
YMR234W	YCR028C-A	909
YMR234W	YGR094W	908

YMR234W	YJL173C	930
YMR235C	YDR002W	978
YMR235C	YDR028C	975
YMR235C	YGR218W	997
YMR235C	YIL063C	944
YMR235C	YLR293C	996
YMR235C	YML121W	991
YMR235C	YMR300C	944
YMR235C	YOR098C	976
YMR235C	YOR185C	934
YMR236W	YBR081C	999
YMR236W	YBR111W-A	959
YMR236W	YBR198C	999
YMR236W	YCL010C	999
YMR236W	YCR042C	996
YMR236W	YDR145W	999
YMR236W	YDR167W	999
YMR236W	YDR176W	999
YMR236W	YDR392W	999
YMR236W	YDR448W	999
YMR236W	YEL009C	995
YMR236W	YER022W	978
YMR236W	YER148W	999
YMR236W	YER164W	980
YMR236W	YGL066W	999
YMR236W	YGL112C	999
YMR236W	YGL127C	924
YMR236W	YGR252W	999
YMR236W	YGR274C	999
YMR236W	YHR041C	978
YMR236W	YHR099W	999
YMR236W	YIL143C	900
YMR236W	YLR055C	999
YMR236W	YLR182W	915
YMR236W	YML015C	999
YMR236W	YML098W	999
YMR236W	YML114C	991
YMR236W	YMR005W	999
YMR236W	YMR223W	999
YMR236W	YMR227C	999
YMR236W	YOL051W	959
YMR236W	YOL148C	999
YMR236W	YOR174W	972
YMR236W	YPL011C	999

YMR236W	YPL047W	999
YMR236W	YPL101W	923
YMR236W	YPL129W	991
YMR236W	YPL254W	999
YMR236W	YPR070W	920
YMR236W	YPR086W	943
YMR237W	YBR023C	903
YMR237W	YDR381W	930
YMR237W	YJL099W	999
YMR237W	YKR027W	999
YMR237W	YLR330W	999
YMR237W	YOR299W	981
YMR238W	YHR061C	916
YMR239C	YDL014W	916
YMR239C	YDR028C	927
YMR239C	YMR229C	904
YMR239C	YNL251C	949
YMR239C	YOR001W	940
YMR240C	YBR055C	997
YMR240C	YBR152W	982
YMR240C	YBR237W	948
YMR240C	YCR063W	971
YMR240C	YDL030W	999
YMR240C	YDL043C	999
YMR240C	YDL098C	974
YMR240C	YDR473C	974
YMR240C	YER029C	999
YMR240C	YER172C	999
YMR240C	YFL017W-A	998
YMR240C	YGL174W	974
YMR240C	YGR074W	998
YMR240C	YGR091W	976
YMR240C	YHR165C	999
YMR240C	YIR005W	946
YMR240C	YIR009W	970
YMR240C	YJL203W	999
YMR240C	YKL074C	904
YMR240C	YKL173W	997
YMR240C	YLL036C	996
YMR240C	YLR117C	999
YMR240C	YLR147C	999
YMR240C	YLR275W	998
YMR240C	YML046W	974
YMR240C	YML049C	999

YMR240C	YMR125W	999
YMR240C	YMR288W	999
YMR240C	YNL138W-A	994
YMR240C	YOR308C	976
YMR240C	YOR319W	999
YMR240C	YPL151C	999
YMR240C	YPL213W	999
YMR240C	YPR082C	997
YMR240C	YPR094W	992
YMR240C	YPR178W	976
YMR240C	YPR182W	998
YMR240C	YPR187W	900
YMR241W	YMR072W	926
YMR242C	YAL035W	914
YMR242C	YBL027W	996
YMR242C	YBL072C	995
YMR242C	YBL087C	974
YMR242C	YBL092W	955
YMR242C	YBR031W	997
YMR242C	YBR048W	997
YMR242C	YBR084C-A	929
YMR242C	YBR118W	916
YMR242C	YBR181C	998
YMR242C	YBR189W	995
YMR242C	YBR191W	996
YMR242C	YCR031C	996
YMR242C	YDL061C	911
YMR242C	YDL075W	996
YMR242C	YDL081C	961
YMR242C	YDL082W	998
YMR242C	YDL083C	972
YMR242C	YDL130W	906
YMR242C	YDL136W	996
YMR242C	YDL191W	922
YMR242C	YDR012W	997
YMR242C	YDR025W	919
YMR242C	YDR064W	997
YMR242C	YDR174W	972
YMR242C	YDR195W	981
YMR242C	YDR382W	996
YMR242C	YDR385W	917
YMR242C	YDR418W	997
YMR242C	YDR447C	998
YMR242C	YDR450W	996

YMR242C	YDR471W	996
YMR242C	YDR496C	944
YMR242C	YDR500C	909
YMR242C	YEL054C	934
YMR242C	YER056C-A	903
YMR242C	YER074W	996
YMR242C	YER102W	927
YMR242C	YER117W	932
YMR242C	YER131W	934
YMR242C	YFL034C-A	914
YMR242C	YFR031C-A	998
YMR242C	YFR032C-A	997
YMR242C	YGL014W	958
YMR242C	YGL030W	997
YMR242C	YGL031C	997
YMR242C	YGL076C	998
YMR242C	YGL103W	995
YMR242C	YGL123W	998
YMR242C	YGL135W	996
YMR242C	YGL147C	999
YMR242C	YGL189C	920
YMR242C	YGR027C	944
YMR242C	YGR034W	998
YMR242C	YGR085C	997
YMR242C	YGR118W	960
YMR242C	YGR148C	931
YMR242C	YGR214W	939
YMR242C	YHL001W	996
YMR242C	YHL015W	998
YMR242C	YHL033C	996
YMR242C	YHR010W	905
YMR242C	YHR021C	931
YMR242C	YHR052W	942
YMR242C	YHR141C	998
YMR242C	YHR203C	997
YMR242C	YIL018W	996
YMR242C	YIL052C	903
YMR242C	YIL069C	996
YMR242C	YIL133C	999
YMR242C	YIL148W	909
YMR242C	YJL136C	934
YMR242C	YJL177W	996
YMR242C	YJL189W	911
YMR242C	YJL190C	999

YMR242C	YJL191W	927
YMR242C	YJR094W-A	932
YMR242C	YJR123W	998
YMR242C	YJR145C	995
YMR242C	YKL006W	938
YMR242C	YKL156W	931
YMR242C	YKL180W	996
YMR242C	YKR057W	934
YMR242C	YKR094C	909
YMR242C	YLL034C	935
YMR242C	YLL045C	998
YMR242C	YLR029C	997
YMR242C	YLR048W	927
YMR242C	YLR061W	917
YMR242C	YLR075W	996
YMR242C	YLR150W	960
YMR242C	YLR167W	903
YMR242C	YLR185W	909
YMR242C	YLR264W	912
YMR242C	YLR287C-A	995
YMR242C	YLR325C	973
YMR242C	YLR333C	942
YMR242C	YLR340W	997
YMR242C	YLR344W	928
YMR242C	YLR367W	922
YMR242C	YLR388W	911
YMR242C	YLR406C	923
YMR242C	YLR441C	999
YMR242C	YLR448W	997
YMR242C	YLR449W	934
YMR242C	YML024W	930
YMR242C	YML026C	925
YMR242C	YML063W	997
YMR242C	YML073C	997
YMR242C	YMR121C	944
YMR242C	YMR142C	997
YMR242C	YMR194W	996
YMR242C	YMR230W	934
YMR242C	YMR260C	928
YMR242C	YMR290C	940
YMR242C	YNL002C	935
YMR242C	YNL067W	941
YMR242C	YNL069C	997
YMR242C	YNL096C	998

YMR242C	YNL162W	929
YMR242C	YNL178W	998
YMR242C	YNL301C	997
YMR242C	YNL302C	998
YMR242C	YOL039W	913
YMR242C	YOL040C	998
YMR242C	YOL041C	937
YMR242C	YOL077C	947
YMR242C	YOL120C	944
YMR242C	YOL121C	973
YMR242C	YOL127W	997
YMR242C	YOR063W	994
YMR242C	YOR096W	995
YMR242C	YOR133W	917
YMR242C	YOR167C	926
YMR242C	YOR182C	903
YMR242C	YOR204W	933
YMR242C	YOR206W	941
YMR242C	YOR234C	998
YMR242C	YOR293W	941
YMR242C	YOR312C	995
YMR242C	YOR369C	983
YMR242C	YPL079W	944
YMR242C	YPL081W	926
YMR242C	YPL090C	995
YMR242C	YPL093W	988
YMR242C	YPL131W	995
YMR242C	YPL143W	934
YMR242C	YPL198W	995
YMR242C	YPL220W	996
YMR242C	YPL249C-A	997
YMR242C	YPR043W	932
YMR242C	YPR080W	916
YMR242C	YPR102C	952
YMR242C	YPR132W	933
YMR243C	YMR209C	998
YMR243C	YOR316C	985
YMR246W	YBR041W	974
YMR246W	YGL082W	921
YMR246W	YGL205W	901
YMR246W	YOR317W	999
YMR247C	YBR279W	900
YMR247C	YGL244W	908
YMR247C	YJL127C	903

YMR250W	YAL062W	905
YMR250W	YBR006W	938
YMR250W	YBR109C	972
YMR250W	YDL215C	983
YMR250W	YGR019W	987
YMR250W	YGR124W	906
YMR250W	YIL143C	913
YMR250W	YMR105C	976
YMR250W	YNL220W	901
YMR250W	YOL033W	902
YMR250W	YOL058W	907
YMR250W	YOR374W	910
YMR250W	YOR375C	905
YMR250W	YPR035W	907
YMR250W	YPR145W	905
YMR251W	YBR126C	906
YMR251W-A	YBR161W	932
YMR251W-A	YPL057C	930
YMR255W	YDL207W	999
YMR255W	YDR192C	998
YMR255W	YGL122C	998
YMR255W	YGL172W	934
YMR255W	YIL115C	940
YMR255W	YMR273C	975
YMR255W	YOR046C	999
YMR255W	YPL169C	951
YMR256C	Q0045	997
YMR256C	Q0250	978
YMR256C	Q0275	986
YMR256C	YDL067C	990
YMR256C	YGL187C	989
YMR256C	YGL191W	981
YMR256C	YHR051W	992
YMR256C	YIL111W	978
YMR256C	YLR038C	989
YMR256C	YLR395C	986
YMR256C	YNL052W	978
YMR257C	Q0250	999
YMR257C	YDL044C	910
YMR257C	YGR222W	944
YMR257C	YLR067C	936
YMR257C	YLR203C	908
YMR257C	YNR045W	943
YMR258C	YDR328C	999

YMR258C	YKR014C	999
YMR259C	YBR061C	991
YMR260C	YAL035W	988
YMR260C	YBL027W	905
YMR260C	YBL072C	907
YMR260C	YBL087C	913
YMR260C	YBL092W	907
YMR260C	YBR031W	927
YMR260C	YBR048W	912
YMR260C	YBR079C	911
YMR260C	YBR084C-A	905
YMR260C	YBR181C	932
YMR260C	YBR189W	908
YMR260C	YBR191W	912
YMR260C	YCR031C	945
YMR260C	YDL061C	903
YMR260C	YDL075W	917
YMR260C	YDL081C	905
YMR260C	YDL082W	908
YMR260C	YDL130W	905
YMR260C	YDL136W	907
YMR260C	YDL191W	907
YMR260C	YDR012W	927
YMR260C	YDR025W	912
YMR260C	YDR064W	933
YMR260C	YDR382W	909
YMR260C	YDR418W	922
YMR260C	YDR447C	905
YMR260C	YDR450W	903
YMR260C	YEL054C	922
YMR260C	YER056C-A	902
YMR260C	YER074W	905
YMR260C	YER102W	907
YMR260C	YER117W	913
YMR260C	YER131W	907
YMR260C	YER165W	946
YMR260C	YFL034C-A	912
YMR260C	YFR032C-A	943
YMR260C	YGL030W	942
YMR260C	YGL031C	903
YMR260C	YGL076C	918
YMR260C	YGL123W	930
YMR260C	YGL135W	913
YMR260C	YGL147C	915

YMR260C	YGL189C	907
YMR260C	YGR027C	901
YMR260C	YGR034W	917
YMR260C	YGR085C	912
YMR260C	YGR118W	923
YMR260C	YGR148C	903
YMR260C	YGR162W	980
YMR260C	YGR214W	909
YMR260C	YHL001W	911
YMR260C	YHL015W	922
YMR260C	YHL033C	913
YMR260C	YHR021C	926
YMR260C	YHR141C	913
YMR260C	YHR203C	913
YMR260C	YIL052C	902
YMR260C	YIL069C	905
YMR260C	YIL133C	927
YMR260C	YIL148W	908
YMR260C	YJL136C	912
YMR260C	YJL138C	901
YMR260C	YJL177W	905
YMR260C	YJL190C	927
YMR260C	YJL191W	945
YMR260C	YJR007W	917
YMR260C	YJR123W	961
YMR260C	YJR145C	913
YMR260C	YKL006W	911
YMR260C	YKL156W	926
YMR260C	YKL180W	905
YMR260C	YKR057W	912
YMR260C	YKR059W	901
YMR260C	YKR094C	908
YMR260C	YLL045C	924
YMR260C	YLR029C	906
YMR260C	YLR048W	909
YMR260C	YLR061W	914
YMR260C	YLR075W	912
YMR260C	YLR167W	901
YMR260C	YLR264W	900
YMR260C	YLR325C	917
YMR260C	YLR333C	900
YMR260C	YLR340W	937
YMR260C	YLR344W	917
YMR260C	YLR367W	911

YMR260C	YLR388W	904
YMR260C	YLR406C	917
YMR260C	YLR441C	943
YMR260C	YLR448W	916
YMR260C	YML024W	905
YMR260C	YML026C	903
YMR260C	YML063W	931
YMR260C	YML073C	914
YMR260C	YMR121C	915
YMR260C	YMR142C	908
YMR260C	YMR230W	915
YMR260C	YMR242C	928
YMR260C	YMR309C	904
YMR260C	YNL067W	901
YMR260C	YNL069C	906
YMR260C	YNL096C	919
YMR260C	YNL162W	913
YMR260C	YNL178W	947
YMR260C	YNL244C	913
YMR260C	YNL301C	908
YMR260C	YNL302C	911
YMR260C	YOL040C	909
YMR260C	YOL120C	908
YMR260C	YOL121C	911
YMR260C	YOL127W	908
YMR260C	YOL139C	983
YMR260C	YOR063W	925
YMR260C	YOR096W	910
YMR260C	YOR167C	900
YMR260C	YOR234C	929
YMR260C	YOR293W	915
YMR260C	YOR312C	910
YMR260C	YOR361C	985
YMR260C	YOR369C	930
YMR260C	YPL079W	912
YMR260C	YPL081W	908
YMR260C	YPL090C	918
YMR260C	YPL131W	913
YMR260C	YPL143W	929
YMR260C	YPL198W	918
YMR260C	YPL220W	913
YMR260C	YPL237W	949
YMR260C	YPR041W	965
YMR260C	YPR102C	912

YMR260C	YPR132W	923
YMR260C	YPR163C	980
YMR261C	YBR126C	999
YMR261C	YDR074W	999
YMR261C	YKL035W	911
YMR261C	YML100W	999
YMR263W	YAL013W	999
YMR263W	YBR095C	999
YMR263W	YBR103W	960
YMR263W	YCR033W	913
YMR263W	YDL076C	999
YMR263W	YDR207C	984
YMR263W	YDR334W	914
YMR263W	YGL194C	977
YMR263W	YIL084C	999
YMR263W	YJR017C	969
YMR263W	YKL185W	993
YMR263W	YKR029C	956
YMR263W	YML041C	908
YMR263W	YMR075W	986
YMR263W	YNL097C	999
YMR263W	YNL330C	999
YMR263W	YOL004W	999
YMR263W	YOL012C	984
YMR263W	YPL139C	999
YMR263W	YPL181W	999
YMR264W	YBR170C	996
YMR264W	YDL126C	921
YMR264W	YIL030C	999
YMR264W	YIR033W	907
YMR264W	YJR117W	913
YMR264W	YLR207W	954
YMR264W	YMR022W	993
YMR266W	YPR032W	931
YMR267W	YBL099W	902
YMR267W	YBR011C	975
YMR267W	YBR039W	903
YMR267W	YDL004W	903
YMR267W	YJR121W	902
YMR268C	YBL026W	999
YMR268C	YBR055C	980
YMR268C	YCR077C	998
YMR268C	YDL098C	970
YMR268C	YDR243C	930

YMR268C	YDR378C	999
YMR268C	YER112W	998
YMR268C	YER146W	999
YMR268C	YGL173C	966
YMR268C	YJL124C	959
YMR268C	YJL203W	900
YMR268C	YJR022W	999
YMR268C	YLR438C-A	999
YMR268C	YNL147W	999
YMR268C	YPR082C	970
YMR268C	YPR178W	970
YMR269W	YJR070C	927
YMR270C	YBL025W	999
YMR270C	YER148W	998
YMR270C	YJL025W	976
YMR270C	YLR141W	999
YMR270C	YOR295W	963
YMR271C	YEL021W	965
YMR271C	YKL216W	974
YMR271C	YML106W	962
YMR272C	YBR036C	997
YMR272C	YHL003C	945
YMR272C	YPL057C	919
YMR273C	YAL016W	999
YMR273C	YBR103W	994
YMR273C	YBR160W	977
YMR273C	YCR033W	993
YMR273C	YDL134C	999
YMR273C	YDL188C	999
YMR273C	YDR227W	966
YMR273C	YGL130W	976
YMR273C	YGL190C	999
YMR273C	YGL194C	989
YMR273C	YHR186C	967
YMR273C	YIL033C	963
YMR273C	YIL112W	993
YMR273C	YJL140W	903
YMR273C	YJL187C	912
YMR273C	YKL101W	909
YMR273C	YKR029C	994
YMR273C	YLR229C	971
YMR273C	YML109W	999
YMR273C	YMR255W	975
YMR273C	YNL307C	949

YMR273C	YOL068C	950
YMR273C	YOR046C	969
YMR273C	YPR165W	978
YMR274C	YDR320C	917
YMR274C	YJR117W	999
YMR274C	YNL098C	937
YMR274C	YOR089C	923
YMR275C	YER125W	999
YMR275C	YGL144C	974
YMR275C	YML111W	996
YMR275C	YNL183C	958
YMR275C	YNL307C	946
YMR276W	YBL057C	951
YMR276W	YDL190C	993
YMR276W	YEL037C	999
YMR276W	YHR027C	981
YMR276W	YHR200W	998
YMR276W	YIL148W	977
YMR276W	YKL145W	993
YMR276W	YLL039C	998
YMR276W	YLR079W	998
YMR276W	YNL188W	969
YMR277W	YBR154C	950
YMR277W	YDL108W	952
YMR277W	YDL140C	999
YMR277W	YDR404C	956
YMR277W	YGL070C	950
YMR277W	YGL244W	975
YMR277W	YGR005C	958
YMR277W	YGR186W	999
YMR277W	YHR143W-A	950
YMR277W	YIL021W	965
YMR277W	YJL140W	960
YMR277W	YJR017C	981
YMR277W	YMR039C	903
YMR277W	YNL222W	923
YMR277W	YOL005C	963
YMR277W	YOR151C	955
YMR277W	YOR210W	951
YMR277W	YOR224C	947
YMR277W	YPL240C	919
YMR277W	YPR187W	950
YMR278W	YDL055C	914
YMR278W	YER003C	951

YMR280C	YDR216W	997
YMR280C	YDR422C	929
YMR280C	YDR477W	998
YMR280C	YER027C	928
YMR280C	YGL035C	939
YMR280C	YGL115W	987
YMR280C	YJL089W	985
YMR281W	YPL175W	910
YMR282C	Q0130	936
YMR284W	YBR136W	988
YMR284W	YBR274W	995
YMR284W	YCL061C	968
YMR284W	YDL220C	999
YMR284W	YDR217C	986
YMR284W	YDR227W	998
YMR284W	YER095W	923
YMR284W	YLR233C	999
YMR284W	YLR318W	999
YMR284W	YML032C	999
YMR284W	YML061C	999
YMR284W	YMR106C	999
YMR284W	YMR190C	996
YMR284W	YMR224C	996
YMR284W	YNL250W	971
YMR284W	YOR033C	979
YMR284W	YPL022W	978
YMR284W	YPL167C	970
YMR287C	YPL029W	976
YMR288W	YAL032C	999
YMR288W	YBL026W	913
YMR288W	YBR055C	998
YMR288W	YBR152W	916
YMR288W	YBR154C	904
YMR288W	YBR237W	908
YMR288W	YDL030W	999
YMR288W	YDL043C	994
YMR288W	YDL087C	964
YMR288W	YDL098C	976
YMR288W	YDL209C	976
YMR288W	YDR416W	974
YMR288W	YDR473C	976
YMR288W	YEL026W	901
YMR288W	YER029C	999
YMR288W	YER172C	999

YMR288W	YFL017W-A	990
YMR288W	YGL070C	911
YMR288W	YGL128C	974
YMR288W	YGL174W	974
YMR288W	YGR074W	913
YMR288W	YGR091W	976
YMR288W	YGR278W	993
YMR288W	YHR165C	998
YMR288W	YIR005W	947
YMR288W	YIR009W	972
YMR288W	YJL203W	998
YMR288W	YKL074C	933
YMR288W	YKL173W	998
YMR288W	YLL036C	999
YMR288W	YLR117C	999
YMR288W	YLR147C	997
YMR288W	YLR275W	997
YMR288W	YML046W	976
YMR288W	YML049C	999
YMR288W	YMR213W	999
YMR288W	YMR240C	999
YMR288W	YNL138W-A	994
YMR288W	YNL286W	998
YMR288W	YOR151C	901
YMR288W	YOR159C	999
YMR288W	YOR210W	908
YMR288W	YOR319W	999
YMR288W	YPL151C	979
YMR288W	YPL178W	904
YMR288W	YPL213W	999
YMR288W	YPR082C	998
YMR288W	YPR094W	995
YMR288W	YPR101W	974
YMR288W	YPR178W	978
YMR288W	YPR182W	997
YMR290C	YBL072C	940
YMR290C	YBL087C	972
YMR290C	YBR031W	938
YMR290C	YBR048W	938
YMR290C	YBR142W	999
YMR290C	YBR247C	923
YMR290C	YCL037C	978
YMR290C	YCL054W	978
YMR290C	YCL059C	998

YMR290C	YCR057C	972
YMR290C	YDL014W	997
YMR290C	YDL031W	998
YMR290C	YDL060W	903
YMR290C	YDL082W	947
YMR290C	YDL148C	972
YMR290C	YDL153C	915
YMR290C	YDR012W	933
YMR290C	YDR025W	933
YMR290C	YDR060W	997
YMR290C	YDR087C	992
YMR290C	YDR398W	921
YMR290C	YDR471W	946
YMR290C	YDR496C	983
YMR290C	YEL026W	934
YMR290C	YER006W	999
YMR290C	YER082C	980
YMR290C	YER102W	937
YMR290C	YER126C	999
YMR290C	YFL002C	987
YMR290C	YFR001W	990
YMR290C	YGL076C	951
YMR290C	YGL111W	993
YMR290C	YGL120C	981
YMR290C	YGL135W	971
YMR290C	YGL147C	936
YMR290C	YGL173C	927
YMR290C	YGR054W	974
YMR290C	YGR085C	940
YMR290C	YGR090W	992
YMR290C	YGR103W	999
YMR290C	YGR128C	990
YMR290C	YHR010W	935
YMR290C	YHR052W	997
YMR290C	YHR066W	996
YMR290C	YHR088W	999
YMR290C	YHR148W	914
YMR290C	YHR170W	966
YMR290C	YHR196W	980
YMR290C	YHR197W	992
YMR290C	YHR203C	938
YMR290C	YIL018W	938
YMR290C	YIL035C	910
YMR290C	YIL133C	938

YMR290C	YJL010C	983
YMR290C	YJL033W	999
YMR290C	YJL069C	918
YMR290C	YJL109C	986
YMR290C	YJR002W	937
YMR290C	YJR145C	940
YMR290C	YKL009W	989
YMR290C	YKL014C	988
YMR290C	YKL021C	963
YMR290C	YKL099C	910
YMR290C	YKL172W	967
YMR290C	YKR060W	909
YMR290C	YKR081C	999
YMR290C	YLL008W	978
YMR290C	YLL011W	918
YMR290C	YLL034C	966
YMR290C	YLL045C	949
YMR290C	YLR002C	982
YMR290C	YLR003C	910
YMR290C	YLR009W	905
YMR290C	YLR029C	947
YMR290C	YLR129W	950
YMR290C	YLR175W	999
YMR290C	YLR186W	948
YMR290C	YLR197W	997
YMR290C	YLR222C	949
YMR290C	YLR276C	993
YMR290C	YLR409C	937
YMR290C	YLR432W	978
YMR290C	YLR448W	947
YMR290C	YLR449W	981
YMR290C	YML063W	949
YMR290C	YML073C	940
YMR290C	YML093W	922
YMR290C	YMR049C	999
YMR290C	YMR093W	953
YMR290C	YMR128W	913
YMR290C	YMR229C	999
YMR290C	YMR242C	940
YMR290C	YNL002C	975
YMR290C	YNL061W	999
YMR290C	YNL069C	938
YMR290C	YNL075W	943
YMR290C	YNL110C	999

YMR290C	YNL132W	994
YMR290C	YNL308C	991
YMR290C	YOL041C	988
YMR290C	YOL077C	996
YMR290C	YOL120C	978
YMR290C	YOL127W	949
YMR290C	YOR004W	948
YMR290C	YOR063W	952
YMR290C	YOR096W	949
YMR290C	YOR145C	948
YMR290C	YOR206W	994
YMR290C	YOR272W	999
YMR290C	YOR294W	982
YMR290C	YOR310C	901
YMR290C	YOR312C	937
YMR290C	YPL012W	995
YMR290C	YPL043W	999
YMR290C	YPL093W	998
YMR290C	YPL126W	930
YMR290C	YPL131W	964
YMR290C	YPL198W	966
YMR290C	YPL211W	997
YMR290C	YPL217C	966
YMR290C	YPL220W	969
YMR290C	YPL266W	910
YMR290C	YPR016C	998
YMR290C	YPR112C	916
YMR290C	YPR144C	921
YMR292W	YBL102W	996
YMR292W	YBR080C	923
YMR292W	YGL005C	907
YMR292W	YML071C	905
YMR292W	YNL041C	911
YMR292W	YNL049C	923
YMR293C	YBL080C	999
YMR293C	YHR019C	915
YMR293C	YOR168W	913
YMR294W	YEL061C	972
YMR294W	YER016W	996
YMR294W	YGL216W	939
YMR294W	YGR229C	987
YMR294W	YHR129C	999
YMR294W	YLL049W	986
YMR294W	YOL069W	910

YMR294W	YPL174C	999
YMR294W	YPL269W	999
YMR295C	YDR348C	962
YMR296C	YBR036C	968
YMR296C	YBR058C-A	941
YMR296C	YDR062W	999
YMR296C	YGL008C	919
YMR296C	YJL134W	976
YMR296C	YLL039C	956
YMR297W	YDR057W	995
YMR297W	YEL060C	965
YMR297W	YLR178C	999
YMR297W	YLR207W	972
YMR297W	YLR378C	913
YMR297W	YOL013C	917
YMR297W	YPL154C	991
YMR298W	YHL003C	999
YMR298W	YKL008C	999
YMR299C	YER120W	905
YMR299C	YPL269W	905
YMR300C	YAL012W	988
YMR300C	YAR015W	939
YMR300C	YBL068W	914
YMR300C	YDL171C	907
YMR300C	YDR408C	940
YMR300C	YER099C	914
YMR300C	YGL234W	998
YMR300C	YGR061C	983
YMR300C	YHL011C	914
YMR300C	YJL130C	916
YMR300C	YJR109C	969
YMR300C	YKL104C	912
YMR300C	YLR028C	980
YMR300C	YLR359W	975
YMR300C	YMR120C	977
YMR300C	YMR217W	986
YMR300C	YMR235C	944
YMR300C	YNL141W	903
YMR300C	YOR128C	995
YMR300C	YOR168W	903
YMR300C	YOR303W	956
YMR300C	YPR035W	904
YMR301C	YLR188W	942
YMR302C	YLR059C	927

YMR302C	YLR355C	911
YMR302C	YPR024W	999
YMR303C	YDR216W	996
YMR303C	YER073W	902
YMR303C	YFL042C	904
YMR303C	YGL256W	980
YMR303C	YHL032C	945
YMR303C	YIL053W	927
YMR303C	YMR169C	901
YMR303C	YMR170C	901
YMR303C	YOL086C	998
YMR303C	YOR374W	965
YMR303C	YPL061W	959
YMR304W	YLR436C	999
YMR305C	YGR279C	995
YMR306W	YLR342W	969
YMR306W	YOR190W	912
YMR307W	YBR023C	991
YMR307W	YBR078W	980
YMR307W	YFL025C	911
YMR307W	YGL200C	923
YMR307W	YGR229C	921
YMR307W	YHR026W	912
YMR307W	YHR030C	925
YMR307W	YHR149C	973
YMR307W	YJL095W	924
YMR307W	YKL203C	926
YMR307W	YLR330W	922
YMR307W	YML012W	902
YMR307W	YNL238W	976
YMR307W	YOL030W	936
YMR307W	YOR008C	928
YMR308C	YBL041W	962
YMR308C	YBL079W	920
YMR308C	YBR009C	991
YMR308C	YBR010W	984
YMR308C	YDL088C	916
YMR308C	YDR101C	972
YMR308C	YDR224C	962
YMR308C	YDR225W	992
YMR308C	YDR381W	999
YMR308C	YER178W	924
YMR308C	YFR002W	915
YMR308C	YFR034C	996

YMR308C	YGL071W	973
YMR308C	YGL092W	963
YMR308C	YGL197W	913
YMR308C	YGR081C	996
YMR308C	YGR119C	981
YMR308C	YHR084W	972
YMR308C	YHR152W	933
YMR308C	YIL115C	963
YMR308C	YJL041W	982
YMR308C	YJL122W	925
YMR308C	YKL068W	990
YMR308C	YKL214C	919
YMR308C	YLL011W	911
YMR308C	YLR276C	912
YMR308C	YLR293C	999
YMR308C	YLR347C	999
YMR308C	YML007W	976
YMR308C	YMR047C	999
YMR308C	YMR153W	999
YMR308C	YNL030W	945
YMR308C	YNL031C	915
YMR308C	YNL189W	997
YMR308C	YOL127W	908
YMR308C	YOR098C	966
YMR308C	YOR185C	919
YMR308C	YPL020C	999
YMR308C	YPR093C	975
YMR309C	YAL035W	998
YMR309C	YBL072C	913
YMR309C	YBR048W	913
YMR309C	YBR079C	999
YMR309C	YCR031C	903
YMR309C	YDL014W	977
YMR309C	YDR025W	997
YMR309C	YDR091C	999
YMR309C	YDR429C	999
YMR309C	YER025W	999
YMR309C	YER102W	997
YMR309C	YGL049C	948
YMR309C	YGR162W	919
YMR309C	YGR214W	952
YMR309C	YJL190C	915
YMR309C	YJL191W	903
YMR309C	YJR007W	997

YMR309C	YLR048W	913
YMR309C	YLR192C	999
YMR309C	YML024W	913
YMR309C	YML063W	922
YMR309C	YMR146C	999
YMR309C	YMR260C	904
YMR309C	YNL244C	999
YMR309C	YOR096W	913
YMR309C	YOR204W	978
YMR309C	YOR293W	909
YMR309C	YOR361C	999
YMR309C	YPL001W	935
YMR309C	YPL081W	924
YMR309C	YPL237W	982
YMR309C	YPR041W	999
YMR309C	YPR086W	965
YMR309C	YPR163C	917
YMR310C	YBL032W	950
YMR310C	YDL208W	999
YMR310C	YGR283C	999
YMR310C	YHR089C	983
YMR310C	YLR175W	990
YMR311C	YER059W	988
YMR311C	YER133W	999
YMR311C	YGL134W	963
YMR311C	YIL050W	967
YMR311C	YKL193C	950
YMR311C	YML016C	951
YMR311C	YPL031C	989
YMR311C	YPL209C	998
YMR311C	YPL219W	965
YMR311C	YPL240C	919
YMR312W	YDL074C	942
YMR312W	YGL237C	955
YMR312W	YGR200C	999
YMR312W	YHR187W	999
YMR312W	YKL110C	924
YMR312W	YLR384C	999
YMR312W	YPL086C	999
YMR312W	YPL101W	999
YMR314W	YBL041W	999
YMR314W	YBR173C	929
YMR314W	YDL007W	997
YMR314W	YDL084W	998

YMR314W	YDL097C	932
YMR314W	YDL147W	999
YMR314W	YDR394W	997
YMR314W	YDR427W	998
YMR314W	YER012W	999
YMR314W	YER021W	998
YMR314W	YER094C	999
YMR314W	YFL007W	992
YMR314W	YFR004W	998
YMR314W	YFR010W	985
YMR314W	YFR050C	999
YMR314W	YFR052W	999
YMR314W	YGL011C	999
YMR314W	YGL048C	967
YMR314W	YGR135W	999
YMR314W	YGR253C	999
YMR314W	YHL030W	974
YMR314W	YHR027C	931
YMR314W	YHR200W	999
YMR314W	YIL075C	997
YMR314W	YJL001W	999
YMR314W	YKL130C	921
YMR314W	YKL145W	997
YMR314W	YLR167W	910
YMR314W	YML092C	999
YMR314W	YOL038W	999
YMR314W	YOR117W	997
YMR314W	YOR157C	999
YMR314W	YOR259C	997
YMR314W	YOR261C	999
YMR314W	YOR362C	999
YMR314W	YPR103W	999
YMR314W	YPR108W	997
YMR314W	YPR173C	998
YMR315W	YBR114W	932
YMR316W	YER125W	969
YMR317W	YGR014W	956
YMR317W	YIL169C	989
YMR317W	YIR019C	979
YMR317W	YKR102W	929
YMR317W	YNL327W	999
YMR317W	YOL108C	943
YMR317W	YOL155C	924
YMR319C	YER145C	955

YMR319C	YFL041W	914
YMR319C	YGL071W	958
YMR319C	YLR214W	913
YMR319C	YLR220W	908
YMR319C	YMR021C	911
YMR319C	YMR058W	995
YMR319C	YPR124W	923
YMR323W	YAL038W	929
YMR323W	YDR050C	914
YMR323W	YGR254W	902
YMR323W	YHR174W	901
YMR323W	YKL152C	906
YMR323W	YOR347C	929
YMR323W	YOR393W	901
YMR323W	YPL281C	901
YNL001W	YKR084C	998
YNL001W	YLR287C-A	976
YNL002C	YBR142W	999
YNL002C	YCL054W	980
YNL002C	YDL031W	993
YNL002C	YDL075W	931
YNL002C	YDR060W	998
YNL002C	YDR087C	996
YNL002C	YDR101C	994
YNL002C	YDR496C	987
YNL002C	YER006W	998
YNL002C	YER126C	999
YNL002C	YFL002C	993
YNL002C	YFR001W	990
YNL002C	YGL030W	935
YNL002C	YGL111W	988
YNL002C	YGL135W	932
YNL002C	YGR103W	999
YNL002C	YGR245C	999
YNL002C	YHR052W	999
YNL002C	YHR066W	996
YNL002C	YHR088W	989
YNL002C	YHR197W	994
YNL002C	YIL018W	931
YNL002C	YKL009W	987
YNL002C	YKL172W	992
YNL002C	YKR081C	999
YNL002C	YLL008W	986
YNL002C	YLL034C	980

YNL002C	YLL045C	936
YNL002C	YLR002C	990
YNL002C	YLR009W	901
YNL002C	YLR029C	934
YNL002C	YLR074C	959
YNL002C	YLR106C	976
YNL002C	YLR276C	991
YNL002C	YML073C	933
YNL002C	YMR049C	999
YNL002C	YMR242C	935
YNL002C	YMR290C	975
YNL002C	YNL061W	979
YNL002C	YNL110C	999
YNL002C	YNL182C	988
YNL002C	YNR053C	997
YNL002C	YOL041C	985
YNL002C	YOL077C	993
YNL002C	YOL127W	938
YNL002C	YOR063W	940
YNL002C	YOR206W	993
YNL002C	YOR272W	999
YNL002C	YPL043W	999
YNL002C	YPL093W	998
YNL002C	YPL131W	958
YNL002C	YPL211W	979
YNL002C	YPL249C-A	929
YNL002C	YPR016C	999
YNL004W	YCL011C	910
YNL004W	YDL084W	998
YNL004W	YDR138W	999
YNL004W	YDR381W	999
YNL004W	YHR167W	999
YNL004W	YKL139W	981
YNL004W	YML062C	999
YNL004W	YMR216C	936
YNL004W	YNL139C	999
YNL004W	YNL253W	909
YNL004W	YOR160W	940
YNL005C	YBL038W	999
YNL005C	YBR122C	974
YNL005C	YBR282W	973
YNL005C	YCR046C	992
YNL005C	YCR071C	974
YNL005C	YDR116C	989

YNL005C	YDR237W	994
YNL005C	YDR296W	992
YNL005C	YDR322W	990
YNL005C	YDR405W	986
YNL005C	YDR462W	989
YNL005C	YGR220C	998
YNL005C	YHR147C	988
YNL005C	YJL063C	992
YNL005C	YKL167C	974
YNL005C	YKR006C	989
YNL005C	YKR085C	974
YNL005C	YLR312W-A	976
YNL005C	YLR439W	974
YNL005C	YML025C	992
YNL005C	YMR024W	977
YNL005C	YMR193W	978
YNL005C	YNL185C	978
YNL005C	YNL252C	989
YNL005C	YNL284C	997
YNL005C	YOR150W	992
YNL005C	YOR204W	978
YNL006W	YBL103C	902
YNL006W	YER040W	979
YNL006W	YER093C	999
YNL006W	YGL252C	947
YNL006W	YHR186C	998
YNL006W	YJL058C	993
YNL006W	YJR066W	999
YNL006W	YKL203C	999
YNL006W	YLR208W	972
YNL006W	YMR068W	998
YNL006W	YOL067C	918
YNL006W	YOL078W	999
YNL006W	YPL180W	987
YNL007C	YAL005C	999
YNL007C	YAL041W	994
YNL007C	YBL075C	968
YNL007C	YBR169C	959
YNL007C	YCL028W	999
YNL007C	YDR171W	935
YNL007C	YDR172W	993
YNL007C	YDR320C	978
YNL007C	YER103W	975
YNL007C	YGL018C	900

YNL007C	YGL128C	940
YNL007C	YLL024C	977
YNL007C	YLL026W	936
YNL007C	YNL064C	997
YNL007C	YNL229C	920
YNL007C	YOR027W	983
YNL007C	YPL106C	979
YNL008C	YDR160W	906
YNL008C	YPL084W	907
YNL009W	YDL066W	903
YNL009W	YJL200C	903
YNL009W	YLR174W	903
YNL009W	YLR304C	908
YNL009W	YNL037C	904
YNL009W	YOR136W	902
YNL010W	YJR024C	958
YNL012W	YBR250W	990
YNL012W	YKL096W	908
YNL014W	YOR262W	998
YNL015W	YBR080C	976
YNL015W	YHR138C	942
YNL016W	YDR432W	976
YNL016W	YGL122C	979
YNL016W	YIR001C	991
YNL016W	YJL033W	906
YNL016W	YNL251C	915
YNL020C	YBL007C	979
YNL020C	YCR088W	987
YNL020C	YIL095W	985
YNL020C	YIR006C	996
YNL021W	YCR084C	993
YNL021W	YDR295C	999
YNL021W	YGL194C	994
YNL021W	YJL110C	977
YNL021W	YMR075W	912
YNL021W	YNL097C	939
YNL021W	YNL330C	995
YNL021W	YOL090W	991
YNL021W	YOR323C	927
YNL021W	YPL116W	999
YNL021W	YPR068C	948
YNL021W	YPR179C	999
YNL023C	YIL161W	994
YNL023C	YNL135C	999

YNL025C	YBL093C	998
YNL025C	YBR253W	993
YNL025C	YCR081W	999
YNL025C	YCR084C	990
YNL025C	YDR308C	901
YNL025C	YDR443C	999
YNL025C	YDR477W	984
YNL025C	YGL035C	912
YNL025C	YGL151W	999
YNL025C	YGR097W	934
YNL025C	YGR104C	987
YNL025C	YNL094W	921
YNL025C	YNL236W	999
YNL025C	YOR140W	966
YNL025C	YPL031C	908
YNL025C	YPL042C	999
YNL025C	YPL248C	976
YNL025C	YPL268W	967
YNL025C	YPR070W	917
YNL026W	YAL010C	932
YNL026W	YHR083W	999
YNL026W	YMR060C	999
YNL026W	YMR203W	910
YNL027W	YGL006W	903
YNL027W	YHR206W	973
YNL027W	YJR132W	990
YNL027W	YKL190W	969
YNL027W	YLR433C	933
YNL027W	YPL204W	950
YNL030W	YBL002W	961
YNL030W	YBL003C	986
YNL030W	YBR009C	999
YNL030W	YBR010W	999
YNL030W	YBR245C	994
YNL030W	YDL042C	999
YNL030W	YDR225W	998
YNL030W	YEL056W	999
YNL030W	YER110C	945
YNL030W	YFL024C	956
YNL030W	YFR037C	968
YNL030W	YGL207W	928
YNL030W	YGR252W	999
YNL030W	YJL115W	999
YNL030W	YLL022C	999

YNL030W	YLR399C	972
YNL030W	YLR442C	999
YNL030W	YML069W	987
YNL030W	YMR127C	976
YNL030W	YMR308C	945
YNL030W	YNL031C	999
YNL030W	YNL206C	906
YNL030W	YOL012C	994
YNL030W	YOL054W	995
YNL030W	YOR244W	995
YNL030W	YOR304W	976
YNL030W	YPL001W	999
YNL030W	YPL153C	993
YNL031C	YBR009C	999
YNL031C	YBR010W	998
YNL031C	YBR245C	912
YNL031C	YDL042C	976
YNL031C	YDR225W	994
YNL031C	YDR440W	999
YNL031C	YEL056W	999
YNL031C	YER110C	970
YNL031C	YGL207W	939
YNL031C	YGR252W	999
YNL031C	YJL115W	999
YNL031C	YJR119C	976
YNL031C	YLL002W	998
YNL031C	YLL022C	999
YNL031C	YLR442C	999
YNL031C	YMR308C	915
YNL031C	YNL030W	999
YNL031C	YNL206C	949
YNL031C	YNL261W	998
YNL031C	YOL012C	969
YNL031C	YOL054W	984
YNL031C	YPL001W	999
YNL031C	YPL153C	993
YNL032W	YCR095C	999
YNL032W	YER040W	906
YNL032W	YHL029C	999
YNL032W	YNL056W	999
YNL032W	YNL099C	999
YNL032W	YNL183C	921
YNL032W	YPL240C	919
YNL036W	YPR003C	923

YNL037C	YDL066W	984
YNL037C	YIL094C	923
YNL037C	YJL200C	921
YNL037C	YLR174W	985
YNL037C	YLR304C	965
YNL037C	YNL009W	904
YNL037C	YOR136W	999
YNL038W	YGR216C	952
YNL038W	YOR149C	922
YNL038W	YPL076W	954
YNL038W	YPL175W	950
YNL039W	YDL150W	960
YNL039W	YER148W	999
YNL039W	YGR047C	999
YNL039W	YGR246C	999
YNL039W	YHR143W-A	960
YNL039W	YJL011C	960
YNL039W	YNL113W	961
YNL039W	YNL151C	960
YNL039W	YNR003C	976
YNL039W	YOR116C	960
YNL039W	YOR207C	960
YNL039W	YPL007C	955
YNL039W	YPR110C	960
YNL039W	YPR190C	960
YNL041C	YBR164C	956
YNL041C	YER157W	999
YNL041C	YGL005C	999
YNL041C	YGL223C	999
YNL041C	YGR120C	999
YNL041C	YHL031C	909
YNL041C	YML071C	999
YNL041C	YMR292W	911
YNL041C	YNL051W	999
YNL041C	YOL018C	907
YNL041C	YOR070C	991
YNL041C	YOR089C	915
YNL041C	YPL051W	925
YNL041C	YPL240C	919
YNL041C	YPR105C	999
YNL042W	YDR099W	982
YNL042W	YDR510W	921
YNL044W	YDR108W	903
YNL044W	YDR233C	924

YNL044W	YER031C	955
YNL044W	YGL044C	996
YNL044W	YGR172C	997
YNL044W	YNL263C	981
YNL044W	YPL070W	992
YNL044W	YPL154C	998
YNL047C	YBL105C	968
YNL047C	YER093C	978
YNL047C	YGR058W	903
YNL047C	YIL105C	999
YNL047C	YJL058C	980
YNL047C	YKL203C	988
YNL047C	YLR433C	936
YNL047C	YMR068W	986
YNL047C	YPL059W	991
YNL048W	YBL020W	933
YNL048W	YBL082C	997
YNL048W	YBR110W	970
YNL048W	YGL065C	989
YNL048W	YOR239W	998
YNL049C	YDL195W	969
YNL049C	YDR517W	989
YNL049C	YHR098C	919
YNL049C	YIL109C	999
YNL049C	YLR026C	902
YNL049C	YLR208W	975
YNL049C	YMR292W	923
YNL049C	YPL085W	999
YNL049C	YPL218W	958
YNL049C	YPR181C	999
YNL051W	YBR164C	959
YNL051W	YER157W	999
YNL051W	YGL005C	999
YNL051W	YGL223C	999
YNL051W	YGR120C	999
YNL051W	YIL109C	913
YNL051W	YJL004C	918
YNL051W	YML071C	999
YNL051W	YNL041C	999
YNL051W	YOL018C	905
YNL051W	YOR070C	991
YNL051W	YOR216C	919
YNL051W	YPL051W	915
YNL051W	YPR105C	999

YNL052W	Q0045	995
YNL052W	Q0250	978
YNL052W	Q0275	978
YNL052W	YDL067C	998
YNL052W	YGL187C	997
YNL052W	YGL191W	998
YNL052W	YHR051W	999
YNL052W	YLR038C	981
YNL052W	YLR395C	981
YNL052W	YMR256C	978
YNL053W	YBL016W	999
YNL053W	YER089C	912
YNL053W	YGR040W	968
YNL053W	YHR030C	999
YNL053W	YIL122W	922
YNL053W	YJL128C	908
YNL053W	YOR231W	923
YNL054W	YFR019W	914
YNL054W	YNL325C	996
YNL055C	YIL114C	977
YNL055C	YLR259C	999
YNL055C	YMR203W	971
YNL055C	YNL085W	984
YNL056W	YCR095C	929
YNL056W	YHL029C	923
YNL056W	YNL032W	999
YNL056W	YNL099C	999
YNL058C	YPL240C	919
YNL059C	YDL002C	997
YNL059C	YDR190C	996
YNL059C	YER092W	928
YNL059C	YFL013C	996
YNL059C	YFL039C	991
YNL059C	YGL150C	999
YNL059C	YJL081C	999
YNL059C	YLR052W	996
YNL059C	YNL215W	993
YNL059C	YOR141C	999
YNL059C	YOR189W	954
YNL059C	YPL129W	996
YNL059C	YPL235W	998
YNL061W	YBR142W	998
YNL061W	YCL054W	979
YNL061W	YDL014W	995

YNL061W	YDL031W	999
YNL061W	YDR012W	938
YNL061W	YDR060W	997
YNL061W	YDR087C	999
YNL061W	YDR381W	930
YNL061W	YDR471W	933
YNL061W	YDR496C	999
YNL061W	YER006W	999
YNL061W	YFL002C	999
YNL061W	YFR001W	999
YNL061W	YGL111W	998
YNL061W	YGR085C	955
YNL061W	YGR103W	997
YNL061W	YHR052W	999
YNL061W	YHR066W	996
YNL061W	YHR088W	999
YNL061W	YHR197W	988
YNL061W	YIL018W	931
YNL061W	YKL009W	999
YNL061W	YKL014C	999
YNL061W	YKL021C	977
YNL061W	YKL172W	988
YNL061W	YKR081C	999
YNL061W	YLL008W	999
YNL061W	YLL034C	981
YNL061W	YLL045C	945
YNL061W	YLR002C	990
YNL061W	YLR009W	978
YNL061W	YLR276C	982
YNL061W	YLR449W	997
YNL061W	YML073C	933
YNL061W	YMR049C	999
YNL061W	YMR229C	996
YNL061W	YMR290C	999
YNL061W	YNL002C	979
YNL061W	YNL110C	997
YNL061W	YNL175C	949
YNL061W	YOL041C	998
YNL061W	YOL077C	994
YNL061W	YOL127W	937
YNL061W	YOR063W	961
YNL061W	YOR206W	999
YNL061W	YOR272W	999
YNL061W	YOR294W	987

YNL061W	YPL043W	999
YNL061W	YPL093W	999
YNL061W	YPL131W	964
YNL061W	YPL146C	921
YNL061W	YPL198W	951
YNL061W	YPL211W	999
YNL061W	YPL220W	925
YNL061W	YPR016C	999
YNL062C	YJL050W	923
YNL062C	YJL125C	999
YNL062C	YMR146C	970
YNL062C	YOR361C	995
YNL063W	YGL143C	972
YNL064C	YAL005C	999
YNL064C	YAL040C	908
YNL064C	YBL075C	998
YNL064C	YBR069C	969
YNL064C	YBR072W	963
YNL064C	YBR101C	926
YNL064C	YBR160W	918
YNL064C	YBR169C	905
YNL064C	YCL028W	978
YNL064C	YCL037C	924
YNL064C	YDL229W	985
YNL064C	YDR172W	999
YNL064C	YDR320C	978
YNL064C	YER103W	993
YNL064C	YER171W	934
YNL064C	YGL018C	904
YNL064C	YJL034W	902
YNL064C	YJR043C	928
YNL064C	YJR045C	909
YNL064C	YLL024C	996
YNL064C	YLL026W	995
YNL064C	YLR212C	971
YNL064C	YLR256W	976
YNL064C	YMR186W	970
YNL064C	YNL007C	997
YNL064C	YNL209W	936
YNL064C	YNL229C	990
YNL064C	YOL111C	979
YNL064C	YOR007C	979
YNL064C	YOR027W	998
YNL064C	YOR048C	938

YNL064C	YOR232W	945
YNL064C	YPL106C	999
YNL064C	YPL240C	999
YNL066W	YGL028C	968
YNL066W	YHR143W	981
YNL066W	YLR286C	908
YNL066W	YNL300W	912
YNL066W	YNL327W	911
YNL067W	YAL035W	912
YNL067W	YBL027W	980
YNL067W	YBL072C	922
YNL067W	YBL087C	981
YNL067W	YBL092W	990
YNL067W	YBR031W	987
YNL067W	YBR048W	976
YNL067W	YBR084C-A	986
YNL067W	YBR118W	907
YNL067W	YBR181C	922
YNL067W	YBR189W	997
YNL067W	YBR191W	997
YNL067W	YCR031C	978
YNL067W	YDL061C	986
YNL067W	YDL075W	968
YNL067W	YDL081C	935
YNL067W	YDL082W	943
YNL067W	YDL130W	919
YNL067W	YDL136W	998
YNL067W	YDL191W	923
YNL067W	YDR012W	988
YNL067W	YDR025W	972
YNL067W	YDR064W	997
YNL067W	YDR382W	972
YNL067W	YDR385W	911
YNL067W	YDR418W	994
YNL067W	YDR447C	924
YNL067W	YDR450W	922
YNL067W	YDR471W	993
YNL067W	YDR500C	936
YNL067W	YEL054C	916
YNL067W	YER056C-A	978
YNL067W	YER074W	905
YNL067W	YER102W	956
YNL067W	YER117W	987
YNL067W	YER131W	988

YNL067W	YFL034C-A	911
YNL067W	YFR031C-A	973
YNL067W	YFR032C-A	935
YNL067W	YGL030W	993
YNL067W	YGL031C	970
YNL067W	YGL076C	968
YNL067W	YGL103W	997
YNL067W	YGL123W	994
YNL067W	YGL135W	914
YNL067W	YGL147C	932
YNL067W	YGL189C	908
YNL067W	YGR027C	997
YNL067W	YGR034W	982
YNL067W	YGR085C	997
YNL067W	YGR118W	998
YNL067W	YGR148C	983
YNL067W	YGR214W	997
YNL067W	YHL001W	994
YNL067W	YHL015W	992
YNL067W	YHL033C	997
YNL067W	YHR010W	967
YNL067W	YHR021C	929
YNL067W	YHR141C	940
YNL067W	YHR203C	984
YNL067W	YIL018W	973
YNL067W	YIL052C	995
YNL067W	YIL069C	905
YNL067W	YIL133C	952
YNL067W	YIL148W	950
YNL067W	YJL136C	961
YNL067W	YJL177W	994
YNL067W	YJL189W	944
YNL067W	YJL190C	981
YNL067W	YJL191W	977
YNL067W	YJR094W-A	984
YNL067W	YJR123W	999
YNL067W	YJR145C	974
YNL067W	YKL006W	997
YNL067W	YKL156W	980
YNL067W	YKL170W	903
YNL067W	YKL180W	999
YNL067W	YKR057W	926
YNL067W	YKR094C	904
YNL067W	YLL045C	972

YNL067W	YLR029C	992
YNL067W	YLR048W	995
YNL067W	YLR061W	980
YNL067W	YLR075W	979
YNL067W	YLR167W	946
YNL067W	YLR185W	926
YNL067W	YLR264W	995
YNL067W	YLR325C	909
YNL067W	YLR333C	944
YNL067W	YLR340W	981
YNL067W	YLR344W	991
YNL067W	YLR367W	995
YNL067W	YLR388W	997
YNL067W	YLR406C	916
YNL067W	YLR441C	930
YNL067W	YLR448W	995
YNL067W	YML024W	995
YNL067W	YML026C	922
YNL067W	YML063W	999
YNL067W	YML073C	926
YNL067W	YMR121C	937
YNL067W	YMR142C	955
YNL067W	YMR194W	980
YNL067W	YMR230W	959
YNL067W	YMR242C	941
YNL067W	YMR260C	901
YNL067W	YNL069C	952
YNL067W	YNL096C	916
YNL067W	YNL162W	925
YNL067W	YNL178W	999
YNL067W	YNL301C	967
YNL067W	YNL302C	919
YNL067W	YNR037C	939
YNL067W	YOL039W	930
YNL067W	YOL040C	985
YNL067W	YOL120C	936
YNL067W	YOL121C	928
YNL067W	YOL127W	999
YNL067W	YOR063W	997
YNL067W	YOR096W	992
YNL067W	YOR133W	922
YNL067W	YOR167C	969
YNL067W	YOR234C	991
YNL067W	YOR293W	989

YNL067W	YOR312C	946
YNL067W	YOR369C	933
YNL067W	YPL079W	974
YNL067W	YPL081W	967
YNL067W	YPL090C	910
YNL067W	YPL131W	995
YNL067W	YPL143W	984
YNL067W	YPL198W	933
YNL067W	YPL220W	956
YNL067W	YPL249C-A	943
YNL067W	YPR043W	949
YNL067W	YPR080W	907
YNL067W	YPR102C	999
YNL067W	YPR132W	954
YNL068C	YBR160W	975
YNL068C	YDR146C	978
YNL068C	YIL131C	999
YNL068C	YLR131C	974
YNL068C	YMR001C	947
YNL068C	YMR043W	999
YNL068C	YOR372C	999
YNL068C	YPR119W	999
YNL068C	YPR120C	930
YNL069C	YBL027W	996
YNL069C	YBL072C	920
YNL069C	YBL087C	944
YNL069C	YBL092W	997
YNL069C	YBR031W	997
YNL069C	YBR048W	995
YNL069C	YBR084C-A	997
YNL069C	YBR118W	906
YNL069C	YBR181C	996
YNL069C	YBR189W	997
YNL069C	YBR191W	995
YNL069C	YCR031C	998
YNL069C	YDL014W	938
YNL069C	YDL061C	913
YNL069C	YDL075W	996
YNL069C	YDL081C	949
YNL069C	YDL082W	996
YNL069C	YDL083C	990
YNL069C	YDL130W	914
YNL069C	YDL136W	996
YNL069C	YDL191W	917

YNL069C	YDR012W	997
YNL069C	YDR025W	995
YNL069C	YDR064W	996
YNL069C	YDR382W	996
YNL069C	YDR385W	910
YNL069C	YDR418W	996
YNL069C	YDR447C	997
YNL069C	YDR450W	998
YNL069C	YDR471W	996
YNL069C	YDR500C	913
YNL069C	YEL054C	929
YNL069C	YER056C-A	913
YNL069C	YER074W	995
YNL069C	YER102W	920
YNL069C	YER117W	928
YNL069C	YER131W	911
YNL069C	YFL034C-A	924
YNL069C	YFR031C-A	998
YNL069C	YFR032C-A	923
YNL069C	YGL030W	997
YNL069C	YGL031C	996
YNL069C	YGL076C	997
YNL069C	YGL103W	995
YNL069C	YGL123W	902
YNL069C	YGL135W	996
YNL069C	YGL147C	998
YNL069C	YGL189C	911
YNL069C	YGR027C	908
YNL069C	YGR034W	997
YNL069C	YGR085C	997
YNL069C	YGR118W	931
YNL069C	YGR148C	902
YNL069C	YGR214W	956
YNL069C	YHL001W	995
YNL069C	YHL015W	929
YNL069C	YHL033C	996
YNL069C	YHR010W	996
YNL069C	YHR021C	922
YNL069C	YHR141C	997
YNL069C	YHR203C	997
YNL069C	YIL018W	996
YNL069C	YIL052C	913
YNL069C	YIL069C	995
YNL069C	YIL133C	999

YNL069C	YIL148W	908
YNL069C	YJL136C	931
YNL069C	YJL177W	996
YNL069C	YJL189W	915
YNL069C	YJL190C	998
YNL069C	YJL191W	967
YNL069C	YJR007W	900
YNL069C	YJR094W-A	936
YNL069C	YJR123W	996
YNL069C	YJR145C	995
YNL069C	YKL006W	915
YNL069C	YKL156W	922
YNL069C	YKL180W	996
YNL069C	YKR057W	931
YNL069C	YKR094C	908
YNL069C	YLL045C	996
YNL069C	YLR029C	997
YNL069C	YLR048W	953
YNL069C	YLR061W	928
YNL069C	YLR075W	997
YNL069C	YLR150W	954
YNL069C	YLR167W	902
YNL069C	YLR185W	913
YNL069C	YLR264W	917
YNL069C	YLR287C-A	995
YNL069C	YLR325C	915
YNL069C	YLR333C	908
YNL069C	YLR340W	997
YNL069C	YLR344W	945
YNL069C	YLR367W	912
YNL069C	YLR388W	913
YNL069C	YLR406C	927
YNL069C	YLR441C	996
YNL069C	YLR448W	996
YNL069C	YML024W	924
YNL069C	YML026C	962
YNL069C	YML063W	996
YNL069C	YML073C	996
YNL069C	YMR121C	940
YNL069C	YMR142C	995
YNL069C	YMR194W	996
YNL069C	YMR230W	927
YNL069C	YMR242C	997
YNL069C	YMR260C	906

YNL069C	YMR290C	938
YNL069C	YNL067W	952
YNL069C	YNL096C	997
YNL069C	YNL162W	933
YNL069C	YNL178W	997
YNL069C	YNL301C	995
YNL069C	YNL302C	923
YNL069C	YOL039W	997
YNL069C	YOL040C	997
YNL069C	YOL120C	998
YNL069C	YOL121C	936
YNL069C	YOL127W	997
YNL069C	YOR063W	998
YNL069C	YOR096W	997
YNL069C	YOR133W	910
YNL069C	YOR167C	927
YNL069C	YOR182C	910
YNL069C	YOR204W	931
YNL069C	YOR234C	923
YNL069C	YOR293W	927
YNL069C	YOR312C	996
YNL069C	YOR369C	938
YNL069C	YPL079W	919
YNL069C	YPL081W	958
YNL069C	YPL090C	994
YNL069C	YPL131W	947
YNL069C	YPL143W	998
YNL069C	YPL198W	997
YNL069C	YPL220W	996
YNL069C	YPL237W	905
YNL069C	YPL249C-A	996
YNL069C	YPR043W	936
YNL069C	YPR080W	906
YNL069C	YPR102C	998
YNL069C	YPR132W	931
YNL070W	YGR082W	989
YNL070W	YMR203W	989
YNL070W	YNL121C	989
YNL070W	YNL131W	999
YNL070W	YOR045W	992
YNL070W	YPR133W-A	990
YNL071W	YAL054C	915
YNL071W	YAR035W	905
YNL071W	YBR221C	999

YNL071W	YDR430C	971
YNL071W	YER178W	999
YNL071W	YFL018C	999
YNL071W	YGR193C	987
YNL071W	YLR153C	912
YNL071W	YML042W	912
YNL071W	YNR016C	918
YNL071W	YPL017C	958
YNL071W	YPL028W	916
YNL072W	YDR279W	999
YNL072W	YLR154C	997
YNL074C	YDR309C	921
YNL074C	YHR061C	923
YNL075W	YBR247C	999
YNL075W	YCL059C	934
YNL075W	YCR057C	999
YNL075W	YDL014W	979
YNL075W	YDL060W	912
YNL075W	YDL148C	912
YNL075W	YDL153C	918
YNL075W	YDR299W	945
YNL075W	YDR324C	999
YNL075W	YDR398W	918
YNL075W	YDR449C	997
YNL075W	YGL120C	921
YNL075W	YGL171W	936
YNL075W	YGR081C	910
YNL075W	YGR128C	950
YNL075W	YGR145W	909
YNL075W	YHR148W	999
YNL075W	YHR169W	955
YNL075W	YJL069C	914
YNL075W	YJL109C	951
YNL075W	YJR002W	999
YNL075W	YKL099C	913
YNL075W	YKR060W	923
YNL075W	YLL011W	948
YNL075W	YLR003C	923
YNL075W	YLR129W	958
YNL075W	YLR175W	934
YNL075W	YLR186W	948
YNL075W	YLR197W	997
YNL075W	YLR222C	999
YNL075W	YLR409C	949

YNL075W	YML093W	920
YNL075W	YMR093W	998
YNL075W	YMR128W	997
YNL075W	YMR229C	929
YNL075W	YMR290C	943
YNL075W	YNL132W	921
YNL075W	YOL010W	941
YNL075W	YOR004W	965
YNL075W	YOR078W	972
YNL075W	YOR145C	967
YNL075W	YOR310C	938
YNL075W	YPL012W	923
YNL075W	YPL126W	999
YNL075W	YPL217C	932
YNL075W	YPR112C	923
YNL075W	YPR137W	999
YNL075W	YPR144C	998
YNL076W	YER177W	994
YNL076W	YGL252C	999
YNL076W	YNL229C	921
YNL077W	YER103W	927
YNL078W	YDR507C	917
YNL078W	YDR510W	945
YNL078W	YGR140W	929
YNL078W	YKR048C	999
YNL078W	YOL070C	999
YNL079C	YCL037C	912
YNL079C	YER155C	989
YNL079C	YFL039C	997
YNL079C	YHR030C	923
YNL079C	YIL138C	999
YNL079C	YNL098C	980
YNL079C	YOL076W	998
YNL079C	YOR326W	987
YNL079C	YPR131C	927
YNL081C	Q0140	962
YNL081C	YBL087C	967
YNL081C	YBR048W	904
YNL081C	YBR118W	912
YNL081C	YBR146W	998
YNL081C	YBR251W	934
YNL081C	YCR031C	994
YNL081C	YDR025W	904
YNL081C	YDR041W	995

YNL081C	YDR175C	986
YNL081C	YDR450W	918
YNL081C	YER117W	967
YNL081C	YFR031C-A	950
YNL081C	YGL123W	997
YNL081C	YGR085C	962
YNL081C	YHL004W	904
YNL081C	YIL018W	950
YNL081C	YIL093C	986
YNL081C	YJL063C	981
YNL081C	YJL190C	904
YNL081C	YJL191W	994
YNL081C	YJR101W	986
YNL081C	YJR113C	998
YNL081C	YJR123W	940
YNL081C	YKL170W	979
YNL081C	YLR048W	902
YNL081C	YLR367W	904
YNL081C	YML026C	918
YNL081C	YNL178W	970
YNL081C	YNR036C	999
YNL081C	YNR037C	995
YNL081C	YPL013C	907
YNL081C	YPR080W	912
YNL081C	YPR102C	962
YNL082W	YBR272C	916
YNL082W	YCR092C	999
YNL082W	YDL102W	998
YNL082W	YDL154W	905
YNL082W	YDR097C	998
YNL082W	YFL003C	926
YNL082W	YML032C	911
YNL082W	YMR167W	999
YNL082W	YNL262W	935
YNL082W	YOL090W	999
YNL082W	YOR033C	992
YNL082W	YPL022W	991
YNL082W	YPL164C	910
YNL083W	YBL030C	980
YNL084C	YBL007C	987
YNL084C	YDL029W	908
YNL084C	YDR388W	965
YNL084C	YER125W	995
YNL084C	YIL095W	955

YNL084C	YIR006C	999
YNL084C	YNL243W	984
YNL084C	YOR181W	992
YNL084C	YOR329C	969
YNL085W	YDR171W	954
YNL085W	YER095W	916
YNL085W	YGR178C	982
YNL085W	YGR234W	950
YNL085W	YLR259C	999
YNL085W	YNL055C	984
YNL085W	YNL090W	939
YNL085W	YPR189W	908
YNL086W	YKL061W	995
YNL088W	YCR077C	906
YNL088W	YDR510W	997
YNL088W	YIL031W	954
YNL088W	YIL035C	914
YNL088W	YLR234W	915
YNL088W	YMR190C	979
YNL088W	YOL006C	999
YNL090W	YBR260C	996
YNL090W	YER155C	967
YNL090W	YKL203C	985
YNL090W	YLR371W	983
YNL090W	YMR198W	933
YNL090W	YNL085W	939
YNL090W	YOR122C	968
YNL090W	YPL161C	938
YNL090W	YPR141C	926
YNL091W	YIL038C	927
YNL091W	YIR009W	945
YNL091W	YKL075C	990
YNL091W	YNL164C	958
YNL091W	YNL288W	962
YNL091W	YNR052C	927
YNL092W	YGR241C	911
YNL093W	YIL033C	911
YNL093W	YJL164C	921
YNL093W	YKL166C	917
YNL093W	YKR014C	915
YNL093W	YNL227C	905
YNL093W	YOR089C	904
YNL093W	YOR370C	940
YNL094W	YAL049C	952

YNL094W	YBL007C	924
YNL094W	YCR009C	919
YNL094W	YCR088W	996
YNL094W	YDR388W	999
YNL094W	YFR024C-A	937
YNL094W	YNL025C	921
YNL096C	YAL035W	914
YNL096C	YBL027W	997
YNL096C	YBL072C	994
YNL096C	YBL087C	932
YNL096C	YBL092W	998
YNL096C	YBR031W	997
YNL096C	YBR048W	997
YNL096C	YBR079C	914
YNL096C	YBR084C-A	913
YNL096C	YBR118W	903
YNL096C	YBR181C	996
YNL096C	YBR189W	997
YNL096C	YBR191W	997
YNL096C	YCR031C	996
YNL096C	YDL061C	915
YNL096C	YDL075W	996
YNL096C	YDL081C	935
YNL096C	YDL082W	996
YNL096C	YDL083C	965
YNL096C	YDL130W	920
YNL096C	YDL136W	996
YNL096C	YDL191W	935
YNL096C	YDR012W	942
YNL096C	YDR025W	929
YNL096C	YDR064W	997
YNL096C	YDR382W	996
YNL096C	YDR385W	902
YNL096C	YDR418W	943
YNL096C	YDR429C	901
YNL096C	YDR447C	997
YNL096C	YDR450W	924
YNL096C	YDR471W	996
YNL096C	YDR500C	920
YNL096C	YEL054C	943
YNL096C	YER056C-A	921
YNL096C	YER074W	995
YNL096C	YER102W	916
YNL096C	YER117W	906

YNL096C	YER131W	934
YNL096C	YER165W	903
YNL096C	YFL034C-A	921
YNL096C	YFR031C-A	996
YNL096C	YFR032C-A	998
YNL096C	YGL014W	978
YNL096C	YGL030W	945
YNL096C	YGL031C	927
YNL096C	YGL076C	960
YNL096C	YGL103W	995
YNL096C	YGL123W	912
YNL096C	YGL135W	931
YNL096C	YGL147C	997
YNL096C	YGL189C	926
YNL096C	YGR027C	918
YNL096C	YGR034W	996
YNL096C	YGR085C	996
YNL096C	YGR118W	923
YNL096C	YGR148C	928
YNL096C	YGR162W	914
YNL096C	YGR214W	925
YNL096C	YHL001W	942
YNL096C	YHL015W	945
YNL096C	YHL033C	942
YNL096C	YHR010W	922
YNL096C	YHR021C	921
YNL096C	YHR141C	998
YNL096C	YHR203C	933
YNL096C	YIL052C	921
YNL096C	YIL069C	920
YNL096C	YIL133C	998
YNL096C	YIL148W	904
YNL096C	YJL136C	935
YNL096C	YJL177W	995
YNL096C	YJL190C	997
YNL096C	YJL191W	931
YNL096C	YJR007W	903
YNL096C	YJR094W-A	921
YNL096C	YJR123W	997
YNL096C	YJR145C	933
YNL096C	YKL006W	942
YNL096C	YKL156W	921
YNL096C	YKL180W	996
YNL096C	YKR057W	935

YNL096C	YKR094C	904
YNL096C	YLL045C	951
YNL096C	YLR029C	997
YNL096C	YLR048W	911
YNL096C	YLR061W	925
YNL096C	YLR075W	995
YNL096C	YLR167W	900
YNL096C	YLR185W	920
YNL096C	YLR264W	997
YNL096C	YLR287C-A	997
YNL096C	YLR325C	927
YNL096C	YLR333C	917
YNL096C	YLR340W	938
YNL096C	YLR344W	933
YNL096C	YLR367W	925
YNL096C	YLR388W	916
YNL096C	YLR406C	923
YNL096C	YLR441C	996
YNL096C	YLR448W	996
YNL096C	YML024W	926
YNL096C	YML026C	924
YNL096C	YML063W	996
YNL096C	YML073C	996
YNL096C	YMR121C	939
YNL096C	YMR142C	931
YNL096C	YMR146C	913
YNL096C	YMR194W	995
YNL096C	YMR230W	935
YNL096C	YMR242C	998
YNL096C	YMR260C	919
YNL096C	YNL067W	916
YNL096C	YNL069C	997
YNL096C	YNL162W	936
YNL096C	YNL178W	997
YNL096C	YNL301C	997
YNL096C	YNL302C	921
YNL096C	YOL040C	997
YNL096C	YOL120C	916
YNL096C	YOL121C	938
YNL096C	YOL127W	997
YNL096C	YOL139C	927
YNL096C	YOR063W	996
YNL096C	YOR096W	999
YNL096C	YOR133W	902

YNL096C	YOR167C	912
YNL096C	YOR182C	917
YNL096C	YOR234C	929
YNL096C	YOR293W	935
YNL096C	YOR312C	996
YNL096C	YOR361C	914
YNL096C	YOR369C	965
YNL096C	YPL079W	934
YNL096C	YPL081W	927
YNL096C	YPL090C	994
YNL096C	YPL131W	923
YNL096C	YPL143W	930
YNL096C	YPL146C	968
YNL096C	YPL198W	935
YNL096C	YPL220W	931
YNL096C	YPL249C-A	921
YNL096C	YPR041W	913
YNL096C	YPR043W	921
YNL096C	YPR067W	972
YNL096C	YPR080W	903
YNL096C	YPR102C	934
YNL096C	YPR132W	923
YNL096C	YPR163C	919
YNL097C	YAL013W	999
YNL097C	YBR095C	999
YNL097C	YBR103W	930
YNL097C	YCR033W	924
YNL097C	YDL076C	999
YNL097C	YDR207C	988
YNL097C	YGL194C	932
YNL097C	YIL084C	999
YNL097C	YKL185W	993
YNL097C	YKR029C	950
YNL097C	YLR085C	906
YNL097C	YMR075W	998
YNL097C	YMR128W	929
YNL097C	YMR263W	999
YNL097C	YNL021W	939
YNL097C	YNL330C	999
YNL097C	YOL004W	999
YNL097C	YOL012C	973
YNL097C	YPL139C	999
YNL097C	YPL181W	999
YNL097C	YPR023C	987

YNL098C	YAL024C	996
YNL098C	YAR019C	967
YNL098C	YBL016W	930
YNL098C	YBR083W	987
YNL098C	YBR140C	999
YNL098C	YBR195C	999
YNL098C	YDL035C	991
YNL098C	YDL140C	976
YNL098C	YDR054C	966
YNL098C	YDR410C	970
YNL098C	YDR443C	962
YNL098C	YER020W	999
YNL098C	YFL033C	971
YNL098C	YGL240W	964
YNL098C	YGL248W	995
YNL098C	YGR092W	971
YNL098C	YGR132C	928
YNL098C	YGR152C	995
YNL098C	YGR231C	908
YNL098C	YHL007C	984
YNL098C	YHR084W	997
YNL098C	YHR205W	987
YNL098C	YIL033C	999
YNL098C	YIL049W	902
YNL098C	YIL119C	987
YNL098C	YJL005W	999
YNL098C	YJL141C	983
YNL098C	YJL164C	999
YNL098C	YJR117W	918
YNL098C	YKL062W	993
YNL098C	YKL166C	999
YNL098C	YLL039C	956
YNL098C	YLR229C	960
YNL098C	YLR246W	918
YNL098C	YLR310C	999
YNL098C	YLR362W	924
YNL098C	YMR037C	995
YNL098C	YMR274C	937
YNL098C	YNL079C	980
YNL098C	YNL138W	933
YNL098C	YNL236W	976
YNL098C	YOL081W	999
YNL098C	YOL110W	998
YNL098C	YOR101W	999

YNL098C	YOR360C	998
YNL098C	YPL203W	999
YNL098C	YPL240C	919
YNL099C	YCR095C	999
YNL099C	YHL029C	998
YNL099C	YNL032W	999
YNL099C	YNL056W	999
YNL101W	YCL038C	903
YNL102W	YAL038W	911
YNL102W	YAR007C	955
YNL102W	YBL023C	954
YNL102W	YBL035C	999
YNL102W	YBR060C	946
YNL102W	YBR087W	914
YNL102W	YBR088C	990
YNL102W	YBR160W	964
YNL102W	YBR202W	945
YNL102W	YDL017W	986
YNL102W	YDL102W	998
YNL102W	YDL220C	999
YNL102W	YDR082W	969
YNL102W	YDR505C	925
YNL102W	YEL032W	946
YNL102W	YGL201C	941
YNL102W	YGL207W	999
YNL102W	YHR164C	945
YNL102W	YIL150C	999
YNL102W	YIR008C	999
YNL102W	YJL030W	976
YNL102W	YJL115W	918
YNL102W	YJL194W	956
YNL102W	YJR006W	988
YNL102W	YJR043C	999
YNL102W	YJR068W	927
YNL102W	YKL017C	923
YNL102W	YKL045W	999
YNL102W	YKL113C	942
YNL102W	YKL125W	976
YNL102W	YLR103C	995
YNL102W	YLR274W	939
YNL102W	YLR288C	904
YNL102W	YML017W	938
YNL102W	YML069W	999
YNL102W	YMR001C	929

YNL102W	YMR078C	956
YNL102W	YNL262W	999
YNL102W	YNL290W	915
YNL102W	YNL312W	910
YNL102W	YNR052C	989
YNL102W	YOL090W	945
YNL102W	YOL094C	910
YNL102W	YOR217W	946
YNL102W	YPR019W	940
YNL102W	YPR135W	999
YNL102W	YPR175W	929
YNL103W	YDR253C	992
YNL103W	YIL046W	999
YNL103W	YIR017C	999
YNL103W	YJL101C	979
YNL103W	YJR060W	999
YNL103W	YLR303W	985
YNL103W	YPL038W	998
YNL103W	YPR167C	956
YNL104C	YCL018W	913
YNL104C	YDL131W	905
YNL104C	YDL182W	918
YNL104C	YGL009C	993
YNL104C	YHR208W	902
YNL104C	YJR016C	952
YNL104C	YJR148W	901
YNL104C	YLR153C	912
YNL104C	YLR355C	933
YNL104C	YLR451W	901
YNL104C	YMR108W	975
YNL104C	YOL058W	913
YNL104C	YOR108W	999
YNL106C	YBL007C	987
YNL106C	YCR088W	996
YNL106C	YFR019W	945
YNL106C	YGL206C	989
YNL106C	YIL002C	999
YNL106C	YJR110W	999
YNL106C	YKL212W	998
YNL106C	YLR240W	919
YNL106C	YLR305C	943
YNL106C	YLR429W	985
YNL106C	YNL267W	996
YNL106C	YNL325C	933

YNL106C	YOL065C	928
YNL106C	YOR109W	999
YNL106C	YPR171W	998
YNL107W	YAL011W	999
YNL107W	YBR231C	999
YNL107W	YDR190C	999
YNL107W	YDR334W	999
YNL107W	YDR359C	999
YNL107W	YDR392W	917
YNL107W	YDR485C	999
YNL107W	YEL018W	999
YNL107W	YFL024C	999
YNL107W	YFL039C	999
YNL107W	YGL075C	967
YNL107W	YGR002C	999
YNL107W	YHR090C	999
YNL107W	YHR099W	999
YNL107W	YJL030W	921
YNL107W	YJL081C	999
YNL107W	YJR082C	999
YNL107W	YLR055C	932
YNL107W	YLR085C	999
YNL107W	YLR385C	999
YNL107W	YLR399C	999
YNL107W	YML041C	995
YNL107W	YNL136W	998
YNL107W	YOL012C	998
YNL107W	YOR213C	906
YNL107W	YOR244W	999
YNL107W	YPL129W	909
YNL107W	YPL235W	999
YNL107W	YPR023C	999
YNL110C	YBL004W	920
YNL110C	YCL054W	999
YNL110C	YCL059C	934
YNL110C	YDL031W	999
YNL110C	YDL075W	976
YNL110C	YDL082W	975
YNL110C	YDR060W	981
YNL110C	YDR087C	952
YNL110C	YDR101C	998
YNL110C	YDR471W	975
YNL110C	YDR496C	976
YNL110C	YER006W	999

YNL110C	YER126C	999
YNL110C	YFL002C	997
YNL110C	YGL030W	979
YNL110C	YGL103W	979
YNL110C	YGL111W	999
YNL110C	YGR103W	999
YNL110C	YGR245C	999
YNL110C	YHR052W	999
YNL110C	YHR088W	999
YNL110C	YHR197W	999
YNL110C	YKL009W	996
YNL110C	YKL172W	945
YNL110C	YKR081C	999
YNL110C	YLL008W	999
YNL110C	YLL034C	943
YNL110C	YLR002C	993
YNL110C	YLR009W	936
YNL110C	YLR074C	969
YNL110C	YLR106C	973
YNL110C	YMR049C	999
YNL110C	YMR290C	999
YNL110C	YNL002C	999
YNL110C	YNL061W	997
YNL110C	YNL182C	999
YNL110C	YOL077C	999
YNL110C	YOL127W	977
YNL110C	YOR063W	981
YNL110C	YOR206W	997
YNL110C	YOR272W	999
YNL110C	YPL012W	922
YNL110C	YPL043W	989
YNL110C	YPL093W	999
YNL110C	YPL211W	994
YNL110C	YPR016C	999
YNL111C	YIL043C	902
YNL112W	YBR031W	974
YNL112W	YDR381W	948
YNL112W	YDR471W	976
YNL112W	YGR085C	976
YNL112W	YHR193C	974
YNL112W	YKR092C	956
YNL112W	YLL045C	976
YNL112W	YLR075W	974
YNL112W	YLR150W	970

YNL112W	YML073C	976
YNL112W	YMR080C	982
YNL112W	YOL127W	976
YNL112W	YOR063W	978
YNL113W	YBL039C	910
YNL113W	YBR154C	999
YNL113W	YDL150W	999
YNL113W	YDR045C	998
YNL113W	YDR156W	998
YNL113W	YER148W	915
YNL113W	YFR011C	934
YNL113W	YGL070C	914
YNL113W	YHL018W	915
YNL113W	YHR143W-A	981
YNL113W	YIL021W	999
YNL113W	YJL011C	998
YNL113W	YJL148W	999
YNL113W	YJR063W	999
YNL113W	YKL144C	999
YNL113W	YKR025W	998
YNL113W	YML121W	989
YNL113W	YNL039W	961
YNL113W	YNL151C	998
YNL113W	YNL248C	998
YNL113W	YNR003C	998
YNL113W	YOR116C	999
YNL113W	YOR207C	999
YNL113W	YOR210W	999
YNL113W	YOR224C	998
YNL113W	YOR340C	999
YNL113W	YOR341W	999
YNL113W	YPR010C	999
YNL113W	YPR110C	999
YNL113W	YPR187W	999
YNL113W	YPR190C	998
YNL116W	YHR115C	945
YNL116W	YLR215C	971
YNL116W	YMR055C	914
YNL117W	YAL054C	907
YNL117W	YBL015W	908
YNL117W	YBR218C	978
YNL117W	YCR005C	925
YNL117W	YDL078C	900
YNL117W	YER065C	997

YNL117W	YGL062W	968
YNL117W	YKL029C	907
YNL117W	YKL085W	930
YNL117W	YLR153C	932
YNL117W	YNR001C	923
YNL117W	YOL126C	948
YNL117W	YPL028W	944
YNL117W	YPR006C	971
YNL118C	YCR077C	988
YNL118C	YDL160C	983
YNL118C	YEL015W	999
YNL118C	YER035W	992
YNL118C	YGL173C	997
YNL118C	YGL222C	935
YNL118C	YJL124C	997
YNL118C	YML091C	998
YNL118C	YMR080C	980
YNL118C	YOL149W	999
YNL121C	YBL005W	971
YNL121C	YDR219C	907
YNL121C	YGR082W	999
YNL121C	YHR117W	970
YNL121C	YMR060C	997
YNL121C	YMR203W	999
YNL121C	YNL070W	989
YNL121C	YNL131W	999
YNL121C	YNR017W	939
YNL121C	YOR045W	945
YNL121C	YPR133W-A	943
YNL123W	YOR317W	981
YNL124W	YDL208W	999
YNL124W	YHR072W-A	999
YNL124W	YHR089C	987
YNL124W	YIL104C	953
YNL124W	YLR175W	999
YNL126W	YAL047C	999
YNL126W	YDR356W	999
YNL126W	YHR172W	999
YNL126W	YLR212C	999
YNL127W	YAL016W	965
YNL127W	YDR200C	991
YNL127W	YGL131C	965
YNL127W	YGL251C	943
YNL127W	YLR371W	910

YNL127W	YMR052W	998
YNL129W	YDL042C	919
YNL129W	YDR400W	993
YNL129W	YGR010W	989
YNL129W	YLR209C	992
YNL129W	YLR328W	989
YNL130C	YGR202C	986
YNL130C	YHR123W	973
YNL130C	YKR031C	924
YNL130C	YLR133W	911
YNL131W	YGR033C	999
YNL131W	YGR082W	999
YNL131W	YKL195W	906
YNL131W	YMR203W	999
YNL131W	YNL070W	999
YNL131W	YNL121C	999
YNL131W	YNR017W	984
YNL131W	YOR045W	993
YNL131W	YPR133W-A	991
YNL132W	YBL004W	994
YNL132W	YBL072C	962
YNL132W	YBR031W	945
YNL132W	YBR048W	969
YNL132W	YBR189W	961
YNL132W	YBR247C	999
YNL132W	YCL059C	999
YNL132W	YCR031C	936
YNL132W	YCR057C	999
YNL132W	YDL014W	995
YNL132W	YDL060W	923
YNL132W	YDL148C	999
YNL132W	YDL153C	915
YNL132W	YDR025W	969
YNL132W	YDR064W	949
YNL132W	YDR299W	999
YNL132W	YDR324C	996
YNL132W	YDR447C	945
YNL132W	YDR449C	999
YNL132W	YER082C	999
YNL132W	YER102W	935
YNL132W	YGL030W	944
YNL132W	YGL076C	936
YNL132W	YGL120C	997
YNL132W	YGL135W	944

YNL132W	YGL171W	929
YNL132W	YGR085C	938
YNL132W	YGR090W	999
YNL132W	YGR128C	999
YNL132W	YGR145W	999
YNL132W	YHR148W	995
YNL132W	YHR169W	916
YNL132W	YHR196W	977
YNL132W	YHR203C	935
YNL132W	YIL018W	936
YNL132W	YIL035C	901
YNL132W	YIL069C	964
YNL132W	YIL133C	936
YNL132W	YJL010C	918
YNL132W	YJL069C	928
YNL132W	YJL109C	996
YNL132W	YJR002W	996
YNL132W	YJR123W	938
YNL132W	YJR145C	970
YNL132W	YKL180W	944
YNL132W	YKR060W	997
YNL132W	YLL011W	996
YNL132W	YLL045C	940
YNL132W	YLR029C	938
YNL132W	YLR075W	944
YNL132W	YLR129W	996
YNL132W	YLR186W	994
YNL132W	YLR197W	995
YNL132W	YLR222C	998
YNL132W	YLR340W	940
YNL132W	YLR409C	998
YNL132W	YLR448W	954
YNL132W	YML063W	937
YNL132W	YML093W	911
YNL132W	YMR093W	996
YNL132W	YMR128W	995
YNL132W	YMR194W	945
YNL132W	YMR229C	997
YNL132W	YMR290C	994
YNL132W	YNL075W	921
YNL132W	YNL178W	944
YNL132W	YOL010W	913
YNL132W	YOR004W	913
YNL132W	YOR063W	942

YNL132W	YOR078W	972
YNL132W	YOR096W	948
YNL132W	YOR145C	915
YNL132W	YOR310C	997
YNL132W	YOR312C	933
YNL132W	YPL012W	995
YNL132W	YPL043W	945
YNL132W	YPL081W	980
YNL132W	YPL090C	931
YNL132W	YPL126W	997
YNL132W	YPL198W	945
YNL132W	YPL217C	996
YNL132W	YPR112C	951
YNL132W	YPR137W	999
YNL132W	YPR144C	999
YNL133C	YGR180C	921
YNL135C	YDR174W	974
YNL135C	YDR311W	982
YNL135C	YER052C	998
YNL135C	YIL078W	917
YNL135C	YJR066W	983
YNL135C	YKL203C	979
YNL135C	YLR005W	987
YNL135C	YLR433C	914
YNL135C	YNL023C	999
YNL136W	YBR231C	900
YNL136W	YDR334W	961
YNL136W	YDR359C	999
YNL136W	YDR485C	915
YNL136W	YEL018W	999
YNL136W	YFL024C	998
YNL136W	YFL039C	987
YNL136W	YGR002C	998
YNL136W	YHR090C	999
YNL136W	YHR099W	947
YNL136W	YJL081C	998
YNL136W	YJR082C	999
YNL136W	YLR085C	901
YNL136W	YML041C	942
YNL136W	YNL107W	998
YNL136W	YOL012C	971
YNL136W	YOR244W	998
YNL136W	YPR023C	999
YNL137C	Q0140	962

YNL137C	YBL090W	988
YNL137C	YBR146W	988
YNL137C	YBR251W	999
YNL137C	YDR036C	999
YNL137C	YDR041W	996
YNL137C	YDR175C	988
YNL137C	YDR337W	988
YNL137C	YDR347W	988
YNL137C	YGL129C	988
YNL137C	YGR084C	988
YNL137C	YGR165W	997
YNL137C	YGR215W	988
YNL137C	YHL004W	999
YNL137C	YIL070C	996
YNL137C	YIL093C	988
YNL137C	YJR101W	988
YNL137C	YJR113C	988
YNL137C	YKL155C	997
YNL137C	YMR158W	974
YNL137C	YMR188C	988
YNL137C	YNL306W	988
YNL137C	YOR158W	988
YNL137C	YPL013C	994
YNL137C	YPL118W	988
YNL138W	YCR088W	999
YNL138W	YDL052C	901
YNL138W	YDR388W	984
YNL138W	YFL039C	999
YNL138W	YGR233C	999
YNL138W	YJL005W	999
YNL138W	YKL007W	978
YNL138W	YNL098C	933
YNL138W	YOR122C	999
YNL138W-A	YCR063W	936
YNL138W-A	YKL074C	919
YNL138W-A	YML049C	998
YNL138W-A	YMR240C	994
YNL138W-A	YMR288W	994
YNL138W-A	YOR319W	994
YNL138W-A	YPR094W	945
YNL139C	YCL011C	999
YNL139C	YDL084W	999
YNL139C	YDR138W	999
YNL139C	YDR381W	997

YNL139C	YER011W	938
YNL139C	YHR167W	999
YNL139C	YLR347C	965
YNL139C	YML062C	999
YNL139C	YMR125W	933
YNL139C	YNL004W	999
YNL139C	YNL189W	969
YNL139C	YNL236W	916
YNL139C	YNL253W	999
YNL141W	YBR280C	999
YNL141W	YJR105W	916
YNL141W	YLR209C	960
YNL141W	YMR300C	903
YNL142W	YER040W	935
YNL142W	YER088C	914
YNL142W	YGR121C	999
YNL142W	YJR147W	921
YNL142W	YKR034W	900
YNL142W	YMR164C	987
YNL142W	YNL229C	931
YNL142W	YOL116W	982
YNL142W	YOR032C	906
YNL142W	YPR138C	997
YNL145W	YCR077C	983
YNL145W	YER146W	969
YNL145W	YJL124C	976
YNL147W	YBL026W	999
YNL147W	YBR055C	998
YNL147W	YCR020C-A	984
YNL147W	YCR077C	999
YNL147W	YDL051W	913
YNL147W	YDL098C	999
YNL147W	YDL160C	999
YNL147W	YDR378C	999
YNL147W	YDR473C	974
YNL147W	YER029C	986
YNL147W	YER112W	999
YNL147W	YER146W	999
YNL147W	YER172C	999
YNL147W	YGL173C	999
YNL147W	YGR074W	948
YNL147W	YGR091W	988
YNL147W	YHR165C	992
YNL147W	YJL124C	999

YNL147W	YJR022W	999
YNL147W	YKL173W	999
YNL147W	YLR147C	999
YNL147W	YLR275W	999
YNL147W	YLR438C-A	999
YNL147W	YMR268C	999
YNL147W	YOL149W	984
YNL147W	YOR159C	942
YNL147W	YOR308C	999
YNL147W	YPR082C	999
YNL147W	YPR178W	999
YNL147W	YPR182W	972
YNL148C	YER007W	993
YNL148C	YFL037W	977
YNL148C	YML085C	981
YNL148C	YML124C	981
YNL148C	YOR265W	949
YNL148C	YOR349W	993
YNL149C	YDL055C	980
YNL151C	YBL039C	915
YNL151C	YBR154C	997
YNL151C	YDL150W	999
YNL151C	YDR005C	941
YNL151C	YDR045C	998
YNL151C	YDR156W	921
YNL151C	YHR143W-A	960
YNL151C	YJL011C	999
YNL151C	YJL148W	932
YNL151C	YJR063W	936
YNL151C	YKL144C	999
YNL151C	YKR025W	998
YNL151C	YLL039C	969
YNL151C	YNL039W	960
YNL151C	YNL113W	998
YNL151C	YNL248C	920
YNL151C	YNR003C	999
YNL151C	YOR116C	999
YNL151C	YOR204W	969
YNL151C	YOR207C	999
YNL151C	YOR210W	997
YNL151C	YOR224C	997
YNL151C	YOR340C	921
YNL151C	YOR341W	921
YNL151C	YPR010C	921

YNL151C	YPR110C	999
YNL151C	YPR187W	998
YNL151C	YPR190C	999
YNL152W	YMR032W	992
YNL153C	YBL034C	951
YNL153C	YDL143W	901
YNL153C	YDR188W	901
YNL153C	YDR334W	900
YNL153C	YEL003W	999
YNL153C	YER016W	940
YNL153C	YFL039C	963
YNL153C	YGR078C	999
YNL153C	YHR191C	952
YNL153C	YIL142W	907
YNL153C	YJL168C	978
YNL153C	YJL179W	999
YNL153C	YLR200W	999
YNL153C	YLR212C	910
YNL153C	YLR292C	901
YNL153C	YML041C	913
YNL153C	YML085C	982
YNL153C	YML094W	999
YNL153C	YML124C	961
YNL153C	YOL012C	987
YNL153C	YPL269W	912
YNL153C	YPR046W	907
YNL154C	YDR137W	909
YNL154C	YDR264C	983
YNL154C	YHR135C	999
YNL154C	YOR153W	923
YNL158W	YBR004C	942
YNL159C	YDR160W	906
YNL159C	YPL084W	907
YNL161W	YCL051W	981
YNL161W	YDL155W	962
YNL161W	YDR293C	999
YNL161W	YER033C	914
YNL161W	YFL034C-B	999
YNL161W	YHR102W	976
YNL161W	YIL123W	994
YNL161W	YIL129C	999
YNL161W	YKL189W	958
YNL161W	YLR131C	998
YNL161W	YLR286C	924

YNL161W	YOR247W	902
YNL161W	YOR353C	930
YNL162W	YBL027W	924
YNL162W	YBL072C	924
YNL162W	YBL087C	920
YNL162W	YBL092W	941
YNL162W	YBR031W	939
YNL162W	YBR048W	910
YNL162W	YBR084C-A	924
YNL162W	YBR118W	910
YNL162W	YBR181C	902
YNL162W	YBR189W	914
YNL162W	YBR191W	927
YNL162W	YCR031C	928
YNL162W	YDL061C	913
YNL162W	YDL075W	934
YNL162W	YDL081C	945
YNL162W	YDL082W	923
YNL162W	YDL130W	917
YNL162W	YDL136W	926
YNL162W	YDL191W	926
YNL162W	YDR012W	939
YNL162W	YDR025W	910
YNL162W	YDR064W	948
YNL162W	YDR382W	945
YNL162W	YDR385W	913
YNL162W	YDR418W	930
YNL162W	YDR447C	935
YNL162W	YDR450W	926
YNL162W	YDR471W	920
YNL162W	YDR500C	917
YNL162W	YEL054C	930
YNL162W	YER056C-A	916
YNL162W	YER074W	913
YNL162W	YER102W	924
YNL162W	YER117W	920
YNL162W	YER131W	919
YNL162W	YFL034C-A	926
YNL162W	YFR031C-A	920
YNL162W	YFR032C-A	941
YNL162W	YGL030W	938
YNL162W	YGL031C	904
YNL162W	YGL076C	935
YNL162W	YGL103W	914

YNL162W	YGL135W	932
YNL162W	YGL147C	925
YNL162W	YGL189C	919
YNL162W	YGR027C	919
YNL162W	YGR034W	936
YNL162W	YGR085C	922
YNL162W	YGR118W	929
YNL162W	YGR148C	904
YNL162W	YGR214W	917
YNL162W	YHL001W	931
YNL162W	YHL015W	939
YNL162W	YHL033C	937
YNL162W	YHR010W	920
YNL162W	YHR021C	949
YNL162W	YHR141C	910
YNL162W	YHR203C	946
YNL162W	YIL018W	920
YNL162W	YIL052C	916
YNL162W	YIL069C	913
YNL162W	YIL133C	935
YNL162W	YIL148W	908
YNL162W	YJL136C	937
YNL162W	YJL177W	921
YNL162W	YJL189W	918
YNL162W	YJL190C	920
YNL162W	YJL191W	928
YNL162W	YJR094W-A	930
YNL162W	YJR123W	924
YNL162W	YJR145C	946
YNL162W	YKL006W	931
YNL162W	YKL156W	936
YNL162W	YKL180W	921
YNL162W	YKR057W	937
YNL162W	YKR094C	908
YNL162W	YLL045C	937
YNL162W	YLR029C	999
YNL162W	YLR048W	917
YNL162W	YLR061W	931
YNL162W	YLR075W	920
YNL162W	YLR167W	902
YNL162W	YLR185W	917
YNL162W	YLR264W	918
YNL162W	YLR287C-A	915
YNL162W	YLR325C	912

YNL162W	YLR333C	918
YNL162W	YLR340W	939
YNL162W	YLR344W	936
YNL162W	YLR367W	920
YNL162W	YLR388W	914
YNL162W	YLR406C	934
YNL162W	YLR441C	930
YNL162W	YLR448W	948
YNL162W	YML024W	935
YNL162W	YML026C	926
YNL162W	YML063W	930
YNL162W	YML073C	939
YNL162W	YMR121C	925
YNL162W	YMR142C	922
YNL162W	YMR194W	931
YNL162W	YMR230W	931
YNL162W	YMR242C	929
YNL162W	YMR260C	913
YNL162W	YNL067W	925
YNL162W	YNL069C	933
YNL162W	YNL096C	936
YNL162W	YNL178W	946
YNL162W	YNL301C	923
YNL162W	YNL302C	927
YNL162W	YOL039W	913
YNL162W	YOL040C	948
YNL162W	YOL120C	923
YNL162W	YOL121C	927
YNL162W	YOL127W	921
YNL162W	YOR063W	923
YNL162W	YOR096W	936
YNL162W	YOR133W	913
YNL162W	YOR167C	918
YNL162W	YOR182C	915
YNL162W	YOR234C	928
YNL162W	YOR293W	931
YNL162W	YOR312C	929
YNL162W	YOR369C	932
YNL162W	YPL079W	927
YNL162W	YPL081W	914
YNL162W	YPL090C	902
YNL162W	YPL131W	926
YNL162W	YPL143W	928
YNL162W	YPL198W	935

YNL162W	YPL220W	932
YNL162W	YPL249C-A	931
YNL162W	YPR043W	930
YNL162W	YPR080W	910
YNL162W	YPR102C	922
YNL162W	YPR132W	929
YNL163C	YLR022C	920
YNL163C	YPR016C	995
YNL164C	YNL091W	958
YNL166C	YDL225W	923
YNL166C	YHR107C	988
YNL166C	YJR076C	986
YNL167C	YBR112C	978
YNL167C	YCR084C	996
YNL167C	YGL035C	907
YNL167C	YHR205W	975
YNL167C	YLR113W	999
YNL167C	YMR037C	901
YNL168C	YOR155C	998
YNL169C	YDR294C	928
YNL169C	YGR170W	995
YNL169C	YJL062W	978
YNL169C	YKL165C	922
YNL169C	YLL031C	906
YNL172W	YBL084C	999
YNL172W	YDL008W	999
YNL172W	YDR118W	999
YNL172W	YDR260C	999
YNL172W	YFR036W	999
YNL172W	YGL116W	920
YNL172W	YGL240W	999
YNL172W	YHR166C	999
YNL172W	YIR025W	999
YNL172W	YJL013C	926
YNL172W	YKL022C	999
YNL172W	YLR102C	999
YNL172W	YLR127C	999
YNL172W	YMR001C	910
YNL172W	YOR249C	999
YNL172W	YPL209C	963
YNL175C	YMR229C	956
YNL175C	YNL061W	949
YNL177C	YCR046C	998
YNL177C	YDR296W	979

YNL177C	YML025C	982
YNL178W	YAL003W	925
YNL178W	YAL035W	998
YNL178W	YBL027W	999
YNL178W	YBL072C	997
YNL178W	YBL087C	990
YNL178W	YBL092W	995
YNL178W	YBR031W	999
YNL178W	YBR048W	999
YNL178W	YBR079C	914
YNL178W	YBR084C-A	998
YNL178W	YBR118W	940
YNL178W	YBR181C	997
YNL178W	YBR189W	999
YNL178W	YBR191W	999
YNL178W	YBR247C	988
YNL178W	YCL059C	951
YNL178W	YCR031C	999
YNL178W	YDL061C	999
YNL178W	YDL075W	999
YNL178W	YDL081C	968
YNL178W	YDL082W	998
YNL178W	YDL083C	998
YNL178W	YDL130W	959
YNL178W	YDL136W	977
YNL178W	YDL191W	944
YNL178W	YDL192W	976
YNL178W	YDR012W	999
YNL178W	YDR025W	999
YNL178W	YDR041W	997
YNL178W	YDR064W	999
YNL178W	YDR382W	999
YNL178W	YDR385W	993
YNL178W	YDR418W	999
YNL178W	YDR429C	904
YNL178W	YDR447C	998
YNL178W	YDR450W	999
YNL178W	YDR471W	999
YNL178W	YDR500C	975
YNL178W	YEL054C	953
YNL178W	YER025W	939
YNL178W	YER056C-A	999
YNL178W	YER074W	996
YNL178W	YER102W	999

YNL178W	YER117W	999
YNL178W	YER131W	957
YNL178W	YER165W	946
YNL178W	YFL034C-A	928
YNL178W	YFR031C-A	999
YNL178W	YFR032C-A	971
YNL178W	YGL030W	999
YNL178W	YGL031C	955
YNL178W	YGL076C	998
YNL178W	YGL103W	999
YNL178W	YGL123W	999
YNL178W	YGL135W	997
YNL178W	YGL147C	999
YNL178W	YGL189C	935
YNL178W	YGR027C	996
YNL178W	YGR034W	999
YNL178W	YGR085C	999
YNL178W	YGR118W	999
YNL178W	YGR148C	999
YNL178W	YGR162W	910
YNL178W	YGR214W	999
YNL178W	YHL001W	999
YNL178W	YHL015W	999
YNL178W	YHL033C	999
YNL178W	YHR010W	977
YNL178W	YHR021C	950
YNL178W	YHR141C	946
YNL178W	YHR203C	999
YNL178W	YIL018W	999
YNL178W	YIL052C	999
YNL178W	YIL069C	996
YNL178W	YIL133C	997
YNL178W	YIL148W	982
YNL178W	YJL136C	988
YNL178W	YJL138C	906
YNL178W	YJL177W	999
YNL178W	YJL189W	958
YNL178W	YJL190C	999
YNL178W	YJL191W	999
YNL178W	YJR094W-A	998
YNL178W	YJR123W	999
YNL178W	YJR145C	999
YNL178W	YKL006W	999
YNL178W	YKL143W	999

YNL178W	YKL156W	984
YNL178W	YKL170W	965
YNL178W	YKL180W	999
YNL178W	YKR057W	951
YNL178W	YKR059W	969
YNL178W	YKR094C	919
YNL178W	YLL039C	906
YNL178W	YLL045C	998
YNL178W	YLR029C	999
YNL178W	YLR048W	998
YNL178W	YLR061W	999
YNL178W	YLR075W	999
YNL178W	YLR167W	982
YNL178W	YLR185W	977
YNL178W	YLR192C	915
YNL178W	YLR249W	940
YNL178W	YLR264W	992
YNL178W	YLR287C-A	995
YNL178W	YLR325C	930
YNL178W	YLR333C	958
YNL178W	YLR340W	999
YNL178W	YLR344W	999
YNL178W	YLR367W	996
YNL178W	YLR388W	999
YNL178W	YLR406C	937
YNL178W	YLR432W	980
YNL178W	YLR441C	997
YNL178W	YLR448W	999
YNL178W	YML024W	999
YNL178W	YML026C	972
YNL178W	YML063W	999
YNL178W	YML073C	997
YNL178W	YMR121C	943
YNL178W	YMR142C	998
YNL178W	YMR194W	999
YNL178W	YMR230W	999
YNL178W	YMR242C	998
YNL178W	YMR260C	947
YNL178W	YNL067W	999
YNL178W	YNL069C	997
YNL178W	YNL081C	970
YNL178W	YNL096C	997
YNL178W	YNL132W	944
YNL178W	YNL162W	946

YNL178W	YNL209W	937
YNL178W	YNL301C	999
YNL178W	YNL302C	962
YNL178W	YNR037C	988
YNL178W	YOL039W	946
YNL178W	YOL040C	999
YNL178W	YOL120C	961
YNL178W	YOL121C	963
YNL178W	YOL127W	999
YNL178W	YOR063W	999
YNL178W	YOR096W	999
YNL178W	YOR133W	955
YNL178W	YOR167C	952
YNL178W	YOR182C	905
YNL178W	YOR234C	987
YNL178W	YOR293W	984
YNL178W	YOR312C	998
YNL178W	YOR361C	911
YNL178W	YOR369C	970
YNL178W	YPL037C	999
YNL178W	YPL079W	988
YNL178W	YPL081W	996
YNL178W	YPL090C	996
YNL178W	YPL131W	999
YNL178W	YPL143W	991
YNL178W	YPL198W	998
YNL178W	YPL204W	977
YNL178W	YPL220W	986
YNL178W	YPL237W	928
YNL178W	YPL239W	930
YNL178W	YPL249C-A	971
YNL178W	YPR041W	916
YNL178W	YPR043W	976
YNL178W	YPR080W	934
YNL178W	YPR102C	999
YNL178W	YPR132W	998
YNL178W	YPR163C	909
YNL180C	YDR353W	993
YNL180C	YER155C	954
YNL180C	YFL047W	948
YNL182C	YCR072C	999
YNL182C	YDL031W	981
YNL182C	YDR101C	993
YNL182C	YER006W	993

YNL182C	YGR103W	989
YNL182C	YGR245C	999
YNL182C	YHR052W	996
YNL182C	YHR085W	999
YNL182C	YHR197W	999
YNL182C	YKL009W	984
YNL182C	YLR074C	986
YNL182C	YLR106C	997
YNL182C	YNL002C	988
YNL182C	YNL110C	999
YNL182C	YNL248C	915
YNL182C	YNR053C	994
YNL182C	YOR063W	925
YNL182C	YPL093W	995
YNL182C	YPR016C	998
YNL183C	YBR195C	984
YNL183C	YER040W	952
YNL183C	YER125W	968
YNL183C	YKL203C	927
YNL183C	YKR039W	952
YNL183C	YML111W	925
YNL183C	YMR275C	958
YNL183C	YNL032W	921
YNL183C	YPR040W	905
YNL185C	YBL038W	955
YNL185C	YDR116C	999
YNL185C	YDR237W	993
YNL185C	YDR296W	933
YNL185C	YDR322W	935
YNL185C	YDR405W	931
YNL185C	YEL050C	904
YNL185C	YGL068W	999
YNL185C	YGR220C	970
YNL185C	YHR147C	974
YNL185C	YJL063C	964
YNL185C	YKL170W	914
YNL185C	YLR069C	915
YNL185C	YLR439W	937
YNL185C	YML025C	957
YNL185C	YMR024W	916
YNL185C	YNL005C	978
YNL185C	YNR036C	971
YNL185C	YOR150W	917
YNL186W	YBR251W	932

YNL186W	YDR227W	999
YNL186W	YKL078W	996
YNL187W	YKL012W	935
YNL188W	YAL047C	977
YNL188W	YMR276W	969
YNL188W	YOR257W	999
YNL188W	YPL124W	911
YNL188W	YPL255W	979
YNL189W	YAR002W	999
YNL189W	YCR035C	990
YNL189W	YDL111C	986
YNL189W	YDR002W	915
YNL189W	YDR083W	958
YNL189W	YDR192C	924
YNL189W	YDR280W	993
YNL189W	YER009W	916
YNL189W	YER040W	987
YNL189W	YER110C	982
YNL189W	YFL039C	909
YNL189W	YGL097W	903
YNL189W	YGL238W	999
YNL189W	YGR095C	993
YNL189W	YGR103W	969
YNL189W	YGR104C	923
YNL189W	YGR158C	970
YNL189W	YGR195W	995
YNL189W	YGR202C	960
YNL189W	YGR218W	964
YNL189W	YHR069C	979
YNL189W	YJR066W	927
YNL189W	YKL068W	913
YNL189W	YLR293C	989
YNL189W	YLR335W	999
YNL189W	YLR347C	999
YNL189W	YMR047C	906
YNL189W	YMR125W	999
YNL189W	YMR308C	997
YNL189W	YNL139C	969
YNL189W	YNL232W	993
YNL189W	YOL021C	990
YNL189W	YOL142W	983
YNL189W	YOR001W	999
YNL189W	YOR076C	974
YNL189W	YOR098C	999

YNL189W	YOR310C	965
YNL189W	YPL020C	999
YNL189W	YPL178W	999
YNL189W	YPR119W	994
YNL191W	YBR281C	990
YNL191W	YFR044C	927
YNL192W	YDR231C	997
YNL197C	YAL040C	987
YNL197C	YBR160W	918
YNL197C	YDL224C	993
YNL197C	YNR052C	913
YNL197C	YPL256C	913
YNL199C	YDR099W	938
YNL199C	YPL075W	999
YNL200C	YKL151C	919
YNL201C	YBL002W	926
YNL201C	YBL003C	955
YNL201C	YBL046W	999
YNL201C	YDL101C	919
YNL201C	YDR075W	999
YNL201C	YDR224C	936
YNL201C	YDR225W	936
YNL201C	YDR293C	926
YNL201C	YDR379W	977
YNL201C	YGR063C	969
YNL201C	YHR134W	928
YNL201C	YIL153W	998
YNL201C	YKL144C	940
YNL201C	YML010W	999
YNL201C	YPL153C	992
YNL202W	YOR363C	935
YNL206C	YBR009C	928
YNL206C	YBR010W	953
YNL206C	YNL030W	906
YNL206C	YNL031C	949
YNL206C	YNR052C	986
YNL207W	YBR189W	976
YNL207W	YBR247C	999
YNL207W	YDL060W	999
YNL207W	YDR064W	980
YNL207W	YGR054W	997
YNL207W	YJL176C	960
YNL207W	YKL143W	999
YNL207W	YLR367W	974

YNL207W	YOR056C	999
YNL207W	YOR145C	999
YNL207W	YOR290C	927
YNL207W	YPL012W	949
YNL207W	YPL204W	999
YNL207W	YPL266W	999
YNL209W	YAL005C	979
YNL209W	YBL075C	906
YNL209W	YBR101C	974
YNL209W	YBR155W	907
YNL209W	YBR191W	976
YNL209W	YDL075W	966
YNL209W	YDL229W	999
YNL209W	YDR064W	970
YNL209W	YDR172W	906
YNL209W	YDR382W	966
YNL209W	YER056C-A	976
YNL209W	YER103W	905
YNL209W	YGL030W	942
YNL209W	YGL073W	974
YNL209W	YGL123W	978
YNL209W	YGR214W	909
YNL209W	YHL033C	940
YNL209W	YHR064C	983
YNL209W	YIL052C	978
YNL209W	YJL034W	907
YNL209W	YJR123W	978
YNL209W	YKL006W	978
YNL209W	YKL180W	998
YNL209W	YLL024C	906
YNL209W	YLR048W	970
YNL209W	YLR061W	954
YNL209W	YLR249W	942
YNL209W	YLR388W	978
YNL209W	YLR448W	938
YNL209W	YML024W	940
YNL209W	YNL064C	936
YNL209W	YNL178W	937
YNL209W	YOR063W	972
YNL209W	YOR232W	914
YNL209W	YPL106C	975
YNL210W	YDL043C	967
YNL210W	YDR240C	994
YNL210W	YJR021C	986

YNL212W	YDL143W	962
YNL212W	YIL142W	987
YNL214W	YDL065C	990
YNL214W	YDR142C	999
YNL214W	YDR244W	999
YNL214W	YDR265W	993
YNL214W	YGL153W	999
YNL214W	YGR077C	993
YNL214W	YJL210W	993
YNL214W	YLR191W	999
YNL214W	YMR026C	993
YNL214W	YOL044W	969
YNL215W	YDL002C	994
YNL215W	YDR190C	997
YNL215W	YFL013C	998
YNL215W	YGL150C	981
YNL215W	YJL081C	991
YNL215W	YLR052W	987
YNL215W	YNL059C	993
YNL215W	YOR141C	996
YNL215W	YOR304W	939
YNL215W	YPL235W	995
YNL216W	YBL088C	934
YNL216W	YBR275C	998
YNL216W	YCL030C	923
YNL216W	YDL042C	958
YNL216W	YDR227W	999
YNL216W	YGR274C	959
YNL216W	YKL112W	926
YNL216W	YKR101W	999
YNL216W	YLR223C	998
YNL216W	YLR442C	999
YNL216W	YLR453C	998
YNL216W	YML109W	963
YNL216W	YOL004W	916
YNL216W	YPL075W	999
YNL216W	YPR104C	993
YNL218W	YBR088C	972
YNL218W	YCR066W	993
YNL218W	YDL102W	947
YNL218W	YEL058W	999
YNL218W	YER095W	961
YNL218W	YGL058W	921
YNL218W	YJL092W	942

YNL218W	YJR006W	997
YNL218W	YJR043C	912
YNL218W	YLR032W	983
YNL218W	YLR234W	928
YNL218W	YMR190C	995
YNL219C	YBL082C	984
YNL219C	YEL002C	965
YNL219C	YNR030W	999
YNL219C	YOR002W	967
YNL220W	YHR216W	930
YNL220W	YJL130C	904
YNL220W	YLL018C	908
YNL220W	YLR028C	942
YNL220W	YLR359W	996
YNL220W	YLR432W	934
YNL220W	YML035C	918
YNL220W	YML056C	931
YNL220W	YMR120C	943
YNL220W	YMR250W	901
YNL220W	YOL058W	968
YNL220W	YPL104W	912
YNL221C	YAL033W	999
YNL221C	YBL018C	999
YNL221C	YBR167C	999
YNL221C	YBR257W	999
YNL221C	YDR478W	999
YNL221C	YGR030C	999
YNL221C	YHR062C	999
YNL221C	YIR015W	981
YNL221C	YLR145W	976
YNL221C	YNL282W	999
YNL222W	YAL043C	999
YNL222W	YDL108W	916
YNL222W	YDL140C	980
YNL222W	YDR195W	999
YNL222W	YDR301W	987
YNL222W	YER133W	989
YNL222W	YFL029C	966
YNL222W	YGR156W	995
YNL222W	YJR093C	999
YNL222W	YKL018W	997
YNL222W	YKL059C	994
YNL222W	YKR002W	999
YNL222W	YLR115W	999

YNL222W	YLR277C	999
YNL222W	YMR277W	923
YNL222W	YNL317W	999
YNL222W	YOR151C	997
YNL222W	YOR179C	999
YNL222W	YPL008W	929
YNL222W	YPR086W	999
YNL222W	YPR107C	999
YNL223W	YBL078C	999
YNL223W	YBR217W	942
YNL223W	YFL037W	983
YNL223W	YHR171W	955
YNL223W	YML085C	986
YNL223W	YNR007C	959
YNL223W	YPL149W	941
YNL224C	YGL120C	998
YNL225C	YDL239C	956
YNL225C	YDR356W	993
YNL225C	YKL042W	999
YNL225C	YOL091W	983
YNL225C	YOR129C	981
YNL225C	YOR373W	962
YNL225C	YPL124W	923
YNL227C	YBR267W	920
YNL227C	YDR101C	951
YNL227C	YGL018C	907
YNL227C	YGR285C	908
YNL227C	YJL122W	936
YNL227C	YKL166C	900
YNL227C	YNL093W	905
YNL227C	YPL203W	923
YNL229C	YAL005C	944
YNL229C	YCL028W	904
YNL229C	YDL047W	965
YNL229C	YDR172W	927
YNL229C	YER023W	927
YNL229C	YER040W	999
YNL229C	YFL021W	995
YNL229C	YJL110C	979
YNL229C	YJR066W	991
YNL229C	YKL015W	950
YNL229C	YKL095W	902
YNL229C	YKL203C	913
YNL229C	YKR034W	993

YNL229C	YLL026W	984
YNL229C	YNL007C	920
YNL229C	YNL064C	990
YNL229C	YNL076W	921
YNL229C	YNL142W	931
YNL229C	YPL240C	946
YNL229C	YPR035W	954
YNL230C	YGR185C	998
YNL230C	YPL046C	989
YNL231C	YOR153W	911
YNL232W	YCR035C	999
YNL232W	YDL111C	999
YNL232W	YDR280W	999
YNL232W	YGL213C	907
YNL232W	YGR095C	999
YNL232W	YGR158C	999
YNL232W	YGR195W	999
YNL232W	YHR069C	999
YNL232W	YHR081W	999
YNL232W	YIR035C	930
YNL232W	YLR398C	933
YNL232W	YNL189W	993
YNL232W	YNR024W	998
YNL232W	YOL021C	999
YNL232W	YOL142W	999
YNL232W	YOR001W	999
YNL232W	YOR076C	999
YNL233W	YBL061C	984
YNL233W	YBR023C	982
YNL233W	YCR002C	995
YNL233W	YDL225W	957
YNL233W	YER133W	999
YNL233W	YGR229C	920
YNL233W	YHR107C	991
YNL233W	YJR076C	925
YNL233W	YLR314C	949
YNL233W	YNL271C	974
YNL234W	YBR274W	957
YNL236W	YBL093C	999
YNL236W	YBR193C	999
YNL236W	YBR253W	999
YNL236W	YCR081W	998
YNL236W	YCR084C	971
YNL236W	YDL005C	999

YNL236W	YDL108W	986
YNL236W	YDL140C	986
YNL236W	YDR145W	921
YNL236W	YDR308C	999
YNL236W	YDR397C	973
YNL236W	YDR443C	999
YNL236W	YDR448W	992
YNL236W	YDR477W	971
YNL236W	YER022W	999
YNL236W	YER111C	995
YNL236W	YER159C	982
YNL236W	YER171W	917
YNL236W	YER177W	909
YNL236W	YGL025C	999
YNL236W	YGL043W	973
YNL236W	YGL070C	918
YNL236W	YGL112C	997
YNL236W	YGL127C	999
YNL236W	YGL151W	999
YNL236W	YGR104C	999
YNL236W	YGR252W	997
YNL236W	YGR274C	975
YNL236W	YHR041C	999
YNL236W	YHR058C	998
YNL236W	YIL021W	907
YNL236W	YKR062W	923
YNL236W	YLR044C	909
YNL236W	YLR071C	999
YNL236W	YLR182W	997
YNL236W	YML007W	992
YNL236W	YMR112C	999
YNL236W	YNL025C	999
YNL236W	YNL098C	976
YNL236W	YNL139C	916
YNL236W	YNR010W	999
YNL236W	YOL051W	999
YNL236W	YOL135C	999
YNL236W	YOL145C	908
YNL236W	YOR140W	972
YNL236W	YOR151C	915
YNL236W	YOR174W	998
YNL236W	YPL016W	969
YNL236W	YPL042C	998
YNL236W	YPL129W	975

YNL236W	YPL240C	963
YNL236W	YPR070W	999
YNL236W	YPR168W	999
YNL238W	YBL007C	980
YNL238W	YDR144C	998
YNL238W	YDR170C	988
YNL238W	YGL206C	935
YNL238W	YHR149C	973
YNL238W	YLL040C	987
YNL238W	YLL048C	958
YNL238W	YLR120C	998
YNL238W	YMR307W	976
YNL238W	YOL031C	999
YNL238W	YOR219C	924
YNL240C	YGL091C	943
YNL240C	YHR122W	971
YNL240C	YIL003W	990
YNL241C	YBR196C	998
YNL241C	YCR073W-A	981
YNL241C	YGR248W	994
YNL241C	YGR256W	973
YNL241C	YHR163W	994
YNL241C	YHR183W	989
YNL241C	YJR127C	917
YNL241C	YLR354C	934
YNL241C	YNR034W	972
YNL241C	YPL061W	979
YNL241C	YPR074C	984
YNL242W	YDL149W	992
YNL242W	YFR021W	999
YNL243W	YAL026C	911
YNL243W	YBL007C	984
YNL243W	YBL047C	921
YNL243W	YCR088W	998
YNL243W	YDL226C	918
YNL243W	YDR129C	992
YNL243W	YDR172W	970
YNL243W	YER125W	917
YNL243W	YFL039C	999
YNL243W	YFR024C-A	994
YNL243W	YGL008C	941
YNL243W	YGL206C	979
YNL243W	YGR167W	999
YNL243W	YIL095W	939

YNL243W	YIR006C	995
YNL243W	YKR001C	977
YNL243W	YNL084C	984
YNL243W	YOR181W	970
YNL243W	YOR329C	908
YNL244C	YBR079C	999
YNL244C	YDR047W	959
YNL244C	YDR427W	999
YNL244C	YDR429C	999
YNL244C	YGL048C	999
YNL244C	YGL049C	980
YNL244C	YGR072W	929
YNL244C	YGR135W	999
YNL244C	YHR027C	999
YNL244C	YHR077C	919
YNL244C	YLR192C	999
YNL244C	YMR080C	943
YNL244C	YMR146C	999
YNL244C	YMR260C	913
YNL244C	YMR309C	999
YNL244C	YOR361C	999
YNL244C	YOR362C	999
YNL244C	YPL237W	990
YNL244C	YPR041W	999
YNL246W	YLL002W	999
YNL247W	YAL012W	906
YNL247W	YGR012W	909
YNL248C	YBL039C	962
YNL248C	YBR154C	999
YNL248C	YDL150W	908
YNL248C	YDR045C	919
YNL248C	YDR101C	938
YNL248C	YDR156W	999
YNL248C	YDR174W	996
YNL248C	YDR280W	964
YNL248C	YHR143W-A	954
YNL248C	YJL148W	999
YNL248C	YJR063W	999
YNL248C	YKL125W	983
YNL248C	YKL144C	908
YNL248C	YNL113W	998
YNL248C	YNL151C	920
YNL248C	YNL182C	915
YNL248C	YNR003C	908

YNL248C	YOR116C	908
YNL248C	YOR207C	908
YNL248C	YOR210W	998
YNL248C	YOR224C	998
YNL248C	YOR310C	988
YNL248C	YOR340C	999
YNL248C	YOR341W	999
YNL248C	YPR010C	999
YNL248C	YPR110C	999
YNL248C	YPR187W	999
YNL248C	YPR190C	929
YNL249C	YNR033W	958
YNL250W	YAR002W	917
YNL250W	YAR007C	973
YNL250W	YBL088C	945
YNL250W	YBR073W	968
YNL250W	YBR088C	908
YNL250W	YDL059C	979
YNL250W	YDL102W	973
YNL250W	YDL220C	988
YNL250W	YDR004W	979
YNL250W	YDR076W	972
YNL250W	YDR369C	999
YNL250W	YER095W	999
YNL250W	YER171W	921
YNL250W	YER173W	901
YNL250W	YGL163C	986
YNL250W	YHL022C	997
YNL250W	YHR014W	998
YNL250W	YHR031C	987
YNL250W	YJL092W	998
YNL250W	YJR043C	933
YNL250W	YKL113C	999
YNL250W	YKR082W	952
YNL250W	YML032C	995
YNL250W	YMR190C	997
YNL250W	YMR224C	999
YNL250W	YMR284W	971
YNL250W	YOL090W	916
YNL250W	YOR033C	999
YNL250W	YOR144C	984
YNL250W	YPL020C	966
YNL250W	YPL022W	943
YNL251C	YDL140C	999

YNL251C	YDL175C	997
YNL251C	YGL049C	991
YNL251C	YLR430W	999
YNL251C	YML117W	999
YNL251C	YMR125W	999
YNL251C	YMR239C	949
YNL251C	YNL016W	915
YNL251C	YOL115W	954
YNL251C	YPL178W	984
YNL251C	YPL190C	999
YNL252C	YBL038W	974
YNL252C	YBR122C	974
YNL252C	YBR282W	970
YNL252C	YCR046C	993
YNL252C	YCR071C	974
YNL252C	YDR116C	974
YNL252C	YDR237W	989
YNL252C	YDR296W	992
YNL252C	YDR322W	989
YNL252C	YDR462W	988
YNL252C	YGR220C	993
YNL252C	YHR147C	972
YNL252C	YJL063C	974
YNL252C	YKL167C	974
YNL252C	YKR006C	974
YNL252C	YKR085C	974
YNL252C	YLR312W-A	972
YNL252C	YLR439W	974
YNL252C	YML009C	972
YNL252C	YML025C	992
YNL252C	YMR024W	974
YNL252C	YMR193W	989
YNL252C	YNL005C	989
YNL252C	YNL284C	997
YNL252C	YOR150W	972
YNL253W	YCL011C	998
YNL253W	YDL084W	982
YNL253W	YDR138W	999
YNL253W	YFR050C	999
YNL253W	YHR167W	999
YNL253W	YML062C	999
YNL253W	YNL004W	909
YNL253W	YNL139C	999
YNL256W	YLR276C	913

YNL256W	YMR113W	989
YNL256W	YOR241W	912
YNL257C	YDR477W	976
YNL258C	YDL145C	967
YNL258C	YDR189W	996
YNL258C	YDR498C	999
YNL258C	YFR051C	987
YNL258C	YGL054C	917
YNL258C	YGL098W	999
YNL258C	YGL145W	999
YNL258C	YKL196C	966
YNL258C	YLR268W	997
YNL258C	YLR440C	999
YNL258C	YNL287W	979
YNL258C	YOR075W	999
YNL259C	YDR270W	999
YNL260C	YDR091C	974
YNL260C	YJR067C	992
YNL261W	YBL023C	998
YNL261W	YBR060C	999
YNL261W	YBR160W	986
YNL261W	YBR202W	999
YNL261W	YDL017W	911
YNL261W	YDR217C	998
YNL261W	YDR310C	966
YNL261W	YEL056W	999
YNL261W	YFR028C	998
YNL261W	YGL201C	926
YNL261W	YHR031C	999
YNL261W	YHR118C	999
YNL261W	YIL150C	987
YNL261W	YJL194W	999
YNL261W	YJR046W	984
YNL261W	YKR101W	909
YNL261W	YLL004W	999
YNL261W	YLR103C	931
YNL261W	YLR182W	999
YNL261W	YLR274W	999
YNL261W	YML065W	999
YNL261W	YMR036C	999
YNL261W	YMR127C	932
YNL261W	YNL031C	998
YNL261W	YPL001W	999
YNL261W	YPR018W	998

YNL261W	YPR019W	999
YNL261W	YPR162C	999
YNL262W	YAR007C	989
YNL262W	YBL023C	993
YNL262W	YBR088C	963
YNL262W	YBR160W	913
YNL262W	YBR202W	903
YNL262W	YBR278W	999
YNL262W	YDL017W	918
YNL262W	YDL051W	960
YNL262W	YDL102W	999
YNL262W	YDR097C	908
YNL262W	YDR121W	999
YNL262W	YFL008W	964
YNL262W	YHR031C	974
YNL262W	YIL150C	979
YNL262W	YIR008C	998
YNL262W	YJL090C	999
YNL262W	YJL194W	910
YNL262W	YKL045W	984
YNL262W	YKL113C	983
YNL262W	YLR103C	927
YNL262W	YLR274W	902
YNL262W	YMR078C	918
YNL262W	YNL082W	935
YNL262W	YNL102W	999
YNL262W	YOL090W	986
YNL262W	YOR033C	982
YNL262W	YPL167C	906
YNL262W	YPR019W	904
YNL262W	YPR175W	999
YNL263C	YER031C	988
YNL263C	YER074W-A	934
YNL263C	YFL005W	979
YNL263C	YFL038C	987
YNL263C	YGL198W	941
YNL263C	YGL212W	962
YNL263C	YGR142W	997
YNL263C	YGR172C	999
YNL263C	YHR105W	979
YNL263C	YNL044W	981
YNL264C	YMR079W	997
YNL265C	YBR159W	962
YNL265C	YDR211W	987

YNL265C	YDR300C	912
YNL265C	YDR486C	906
YNL265C	YGR083C	987
YNL265C	YGR095C	913
YNL265C	YGR194C	920
YNL265C	YKL002W	901
YNL265C	YKR026C	982
YNL265C	YKR035W-A	973
YNL265C	YLR181C	914
YNL265C	YLR291C	985
YNL265C	YOL142W	970
YNL265C	YOR260W	987
YNL265C	YPL237W	978
YNL265C	YPR173C	993
YNL267W	YDL192W	979
YNL267W	YDR099W	999
YNL267W	YDR373W	999
YNL267W	YER031C	924
YNL267W	YER177W	995
YNL267W	YFL039C	940
YNL267W	YIL002C	959
YNL267W	YJL100W	934
YNL267W	YKL212W	997
YNL267W	YLR240W	921
YNL267W	YLR305C	996
YNL267W	YMR079W	988
YNL267W	YNL106C	996
YNL267W	YOR109W	995
YNL267W	YPL145C	983
YNL267W	YPL268W	950
YNL270C	YBL037W	962
YNL271C	YBL016W	988
YNL271C	YBL105C	932
YNL271C	YCL027W	999
YNL271C	YDR150W	980
YNL271C	YDR309C	990
YNL271C	YER149C	972
YNL271C	YFL039C	999
YNL271C	YGR229C	972
YNL271C	YHR030C	988
YNL271C	YHR061C	949
YNL271C	YHR084W	925
YNL271C	YHR129C	988
YNL271C	YIL118W	982

YNL271C	YIL159W	999
YNL271C	YJL095W	960
YNL271C	YJL157C	999
YNL271C	YKR054C	995
YNL271C	YLL021W	995
YNL271C	YLR229C	999
YNL271C	YLR319C	999
YNL271C	YLR362W	919
YNL271C	YMR032W	987
YNL271C	YMR109W	909
YNL271C	YMR232W	998
YNL271C	YNL233W	974
YNL271C	YNL289W	932
YNL271C	YNL293W	968
YNL271C	YNL298W	991
YNL271C	YOR058C	968
YNL271C	YOR122C	998
YNL271C	YOR269W	965
YNL271C	YPL115C	977
YNL271C	YPL174C	977
YNL271C	YPL269W	943
YNL271C	YPR165W	999
YNL272C	YDR164C	981
YNL272C	YDR166C	995
YNL272C	YER008C	941
YNL272C	YFL005W	999
YNL272C	YGL210W	940
YNL272C	YGL233W	999
YNL272C	YGR200C	972
YNL272C	YKL079W	956
YNL272C	YLR166C	990
YNL272C	YLR384C	990
YNL272C	YOR326W	996
YNL272C	YPL086C	972
YNL272C	YPR017C	961
YNL272C	YPR032W	904
YNL272C	YPR055W	993
YNL273W	YCL016C	956
YNL273W	YCL061C	999
YNL273W	YDR217C	989
YNL273W	YHR134W	919
YNL273W	YHR191C	992
YNL273W	YJL092W	956
YNL273W	YJR043C	974

YNL273W	YKL068W	913
YNL273W	YLR103C	990
YNL273W	YMR048W	999
YNL273W	YOR144C	948
YNL273W	YOR189W	930
YNL276C	YFL026W	998
YNL277W	YJR130C	928
YNL277W	YJR139C	993
YNL277W	YLR303W	996
YNL277W	YML082W	901
YNL279W	YOL108C	943
YNL280C	YDR458C	998
YNL280C	YER044C	999
YNL280C	YGL001C	998
YNL280C	YGL012W	999
YNL280C	YGR060W	930
YNL280C	YGR175C	999
YNL280C	YHR007C	999
YNL280C	YLR056W	999
YNL280C	YLR100W	999
YNL280C	YML008C	999
YNL280C	YMR202W	999
YNL281W	YDR168W	943
YNL281W	YDR214W	997
YNL281W	YMR186W	997
YNL281W	YPL240C	999
YNL282W	YAL033W	999
YNL282W	YBL018C	999
YNL282W	YBR167C	999
YNL282W	YBR257W	999
YNL282W	YDR478W	952
YNL282W	YGR030C	952
YNL282W	YHR062C	999
YNL282W	YIR015W	952
YNL282W	YLR145W	955
YNL282W	YNL221C	999
YNL283C	YEL034W	981
YNL283C	YLR332W	972
YNL283C	YOL105C	982
YNL283C	YOR008C	994
YNL284C	YBL038W	999
YNL284C	YBR122C	928
YNL284C	YBR282W	934
YNL284C	YCR046C	916

YNL284C	YCR071C	969
YNL284C	YDL145C	917
YNL284C	YDR116C	983
YNL284C	YDR237W	999
YNL284C	YDR238C	917
YNL284C	YDR296W	917
YNL284C	YDR322W	997
YNL284C	YDR462W	999
YNL284C	YEL050C	921
YNL284C	YGR220C	977
YNL284C	YHR147C	994
YNL284C	YJL063C	951
YNL284C	YLR439W	998
YNL284C	YML009C	925
YNL284C	YMR024W	998
YNL284C	YMR193W	913
YNL284C	YMR225C	925
YNL284C	YNL005C	997
YNL284C	YNL252C	997
YNL284C	YOR150W	991
YNL284C	YOR201C	930
YNL286W	YBR237W	990
YNL286W	YDL030W	962
YNL286W	YDL043C	912
YNL286W	YMR288W	998
YNL287W	YBR162C	996
YNL287W	YDL137W	949
YNL287W	YDL145C	999
YNL287W	YDL192W	997
YNL287W	YDL226C	905
YNL287W	YDR170C	924
YNL287W	YDR238C	999
YNL287W	YEL022W	985
YNL287W	YER122C	999
YNL287W	YFL038C	908
YNL287W	YFL048C	976
YNL287W	YFR051C	999
YNL287W	YGL051W	967
YNL287W	YGL137W	999
YNL287W	YGL145W	905
YNL287W	YIL004C	998
YNL287W	YIL076W	999
YNL287W	YJR031C	986
YNL287W	YLR078C	988

YNL287W	YLR268W	998
YNL287W	YNL258C	979
YNL287W	YOR307C	906
YNL287W	YPL010W	999
YNL288W	YAL021C	999
YNL288W	YCR093W	999
YNL288W	YDL165W	999
YNL288W	YER068W	998
YNL288W	YGR134W	999
YNL288W	YGR135W	927
YNL288W	YIL038C	997
YNL288W	YNL091W	962
YNL288W	YNR052C	999
YNL288W	YPR072W	998
YNL289W	YBR160W	930
YNL289W	YER111C	954
YNL289W	YER155C	957
YNL289W	YFR034C	936
YNL289W	YHR030C	969
YNL289W	YNL271C	932
YNL289W	YPL031C	999
YNL290W	YBL035C	998
YNL290W	YBR087W	999
YNL290W	YBR088C	999
YNL290W	YER173W	999
YNL290W	YIR008C	908
YNL290W	YJR068W	999
YNL290W	YMR078C	999
YNL290W	YNL102W	915
YNL290W	YOL094C	999
YNL290W	YOR144C	999
YNL290W	YOR217W	999
YNL291C	YGR217W	993
YNL292W	YGL063W	926
YNL293W	YAL041W	970
YNL293W	YDR099W	957
YNL293W	YER031C	906
YNL293W	YFL005W	997
YNL293W	YHR052W	904
YNL293W	YLL021W	996
YNL293W	YLR229C	999
YNL293W	YLR262C	984
YNL293W	YNL271C	968
YNL293W	YOL112W	999

YNL293W	YOR089C	984
YNL294C	YJL168C	952
YNL294C	YLR342W	912
YNL294C	YPR173C	902
YNL297C	YBR164C	998
YNL297C	YDR141C	999
YNL297C	YIL048W	936
YNL297C	YJL168C	956
YNL297C	YMR015C	936
YNL297C	YOR089C	964
YNL297C	YPL051W	906
YNL298W	YAL041W	992
YNL298W	YBR023C	971
YNL298W	YBR200W	995
YNL298W	YCL016C	930
YNL298W	YCR088W	946
YNL298W	YDL029W	935
YNL298W	YDL097C	919
YNL298W	YDL225W	912
YNL298W	YDR309C	996
YNL298W	YDR379W	921
YNL298W	YDR507C	979
YNL298W	YER111C	951
YNL298W	YER149C	966
YNL298W	YGR135W	963
YNL298W	YHL007C	999
YNL298W	YHR061C	997
YNL298W	YHR107C	998
YNL298W	YHR111W	940
YNL298W	YHR191C	962
YNL298W	YHR200W	988
YNL298W	YIL153W	961
YNL298W	YJL187C	999
YNL298W	YKL048C	900
YNL298W	YKL101W	951
YNL298W	YKL129C	982
YNL298W	YLL021W	965
YNL298W	YLR229C	999
YNL298W	YLR319C	960
YNL298W	YLR362W	982
YNL298W	YML109W	905
YNL298W	YMR001C	974
YNL298W	YMR055C	964
YNL298W	YMR079W	971

YNL298W	YMR109W	959
YNL298W	YMR199W	976
YNL298W	YNL271C	991
YNL298W	YOL113W	902
YNL298W	YOR127W	998
YNL298W	YOR326W	981
YNL298W	YPL115C	992
YNL298W	YPL240C	919
YNL298W	YPL256C	976
YNL299W	YDL175C	964
YNL299W	YIL079C	975
YNL299W	YJL050W	999
YNL299W	YOL115W	999
YNL299W	YOR001W	976
YNL300W	YGL028C	913
YNL300W	YHR143W	912
YNL300W	YNL066W	912
YNL300W	YNL327W	905
YNL301C	YAL035W	917
YNL301C	YBL027W	999
YNL301C	YBL072C	924
YNL301C	YBL087C	991
YNL301C	YBL092W	999
YNL301C	YBR031W	997
YNL301C	YBR048W	975
YNL301C	YBR084C-A	999
YNL301C	YBR118W	926
YNL301C	YBR181C	995
YNL301C	YBR189W	998
YNL301C	YBR191W	998
YNL301C	YCR031C	997
YNL301C	YDL061C	953
YNL301C	YDL075W	999
YNL301C	YDL081C	931
YNL301C	YDL082W	996
YNL301C	YDL083C	965
YNL301C	YDL130W	923
YNL301C	YDL136W	999
YNL301C	YDL191W	923
YNL301C	YDR012W	999
YNL301C	YDR025W	920
YNL301C	YDR060W	936
YNL301C	YDR064W	999
YNL301C	YDR382W	999

YNL301C	YDR385W	928
YNL301C	YDR418W	995
YNL301C	YDR447C	996
YNL301C	YDR450W	922
YNL301C	YDR471W	997
YNL301C	YDR500C	999
YNL301C	YEL054C	913
YNL301C	YER056C-A	987
YNL301C	YER074W	994
YNL301C	YER102W	999
YNL301C	YER117W	999
YNL301C	YER131W	924
YNL301C	YFL034C-A	903
YNL301C	YFR031C-A	999
YNL301C	YFR032C-A	927
YNL301C	YGL014W	978
YNL301C	YGL030W	973
YNL301C	YGL031C	991
YNL301C	YGL076C	999
YNL301C	YGL103W	999
YNL301C	YGL123W	978
YNL301C	YGL135W	999
YNL301C	YGL147C	993
YNL301C	YGL189C	908
YNL301C	YGR027C	941
YNL301C	YGR034W	996
YNL301C	YGR085C	997
YNL301C	YGR118W	975
YNL301C	YGR148C	942
YNL301C	YGR214W	962
YNL301C	YHL001W	964
YNL301C	YHL015W	954
YNL301C	YHL033C	997
YNL301C	YHR010W	955
YNL301C	YHR021C	922
YNL301C	YHR026W	930
YNL301C	YHR141C	999
YNL301C	YHR203C	999
YNL301C	YIL018W	996
YNL301C	YIL052C	977
YNL301C	YIL069C	902
YNL301C	YIL133C	999
YNL301C	YIL148W	999
YNL301C	YJL136C	935

YNL301C	YJL177W	998
YNL301C	YJL189W	934
YNL301C	YJL190C	921
YNL301C	YJL191W	956
YNL301C	YJR094W-A	998
YNL301C	YJR123W	999
YNL301C	YJR145C	916
YNL301C	YKL006W	978
YNL301C	YKL156W	964
YNL301C	YKL180W	999
YNL301C	YKR057W	922
YNL301C	YKR059W	998
YNL301C	YKR094C	905
YNL301C	YLL045C	996
YNL301C	YLR029C	999
YNL301C	YLR048W	991
YNL301C	YLR060W	942
YNL301C	YLR061W	994
YNL301C	YLR075W	999
YNL301C	YLR167W	999
YNL301C	YLR185W	999
YNL301C	YLR264W	964
YNL301C	YLR325C	904
YNL301C	YLR333C	916
YNL301C	YLR340W	998
YNL301C	YLR344W	997
YNL301C	YLR367W	959
YNL301C	YLR388W	982
YNL301C	YLR406C	914
YNL301C	YLR441C	996
YNL301C	YLR448W	998
YNL301C	YML024W	991
YNL301C	YML026C	922
YNL301C	YML063W	998
YNL301C	YML073C	996
YNL301C	YMR121C	927
YNL301C	YMR142C	998
YNL301C	YMR194W	998
YNL301C	YMR230W	928
YNL301C	YMR242C	997
YNL301C	YMR260C	908
YNL301C	YNL067W	967
YNL301C	YNL069C	995
YNL301C	YNL096C	997

YNL301C	YNL162W	923
YNL301C	YNL178W	999
YNL301C	YNL302C	920
YNL301C	YOL039W	912
YNL301C	YOL040C	944
YNL301C	YOL120C	997
YNL301C	YOL121C	921
YNL301C	YOL127W	999
YNL301C	YOR063W	999
YNL301C	YOR096W	998
YNL301C	YOR133W	977
YNL301C	YOR167C	917
YNL301C	YOR204W	937
YNL301C	YOR206W	925
YNL301C	YOR234C	965
YNL301C	YOR293W	939
YNL301C	YOR312C	998
YNL301C	YOR369C	920
YNL301C	YPL079W	940
YNL301C	YPL081W	917
YNL301C	YPL090C	994
YNL301C	YPL131W	999
YNL301C	YPL143W	965
YNL301C	YPL198W	924
YNL301C	YPL220W	999
YNL301C	YPL249C-A	998
YNL301C	YPR043W	996
YNL301C	YPR080W	912
YNL301C	YPR102C	994
YNL301C	YPR132W	999
YNL302C	YBL027W	939
YNL302C	YBL072C	923
YNL302C	YBL087C	922
YNL302C	YBL092W	946
YNL302C	YBR031W	930
YNL302C	YBR048W	915
YNL302C	YBR084C-A	939
YNL302C	YBR118W	907
YNL302C	YBR181C	907
YNL302C	YBR189W	919
YNL302C	YBR191W	919
YNL302C	YCR031C	998
YNL302C	YDL061C	912
YNL302C	YDL075W	916

YNL302C	YDL081C	928
YNL302C	YDL082W	923
YNL302C	YDL130W	904
YNL302C	YDL136W	998
YNL302C	YDL191W	925
YNL302C	YDR012W	930
YNL302C	YDR025W	915
YNL302C	YDR064W	949
YNL302C	YDR382W	926
YNL302C	YDR385W	907
YNL302C	YDR418W	929
YNL302C	YDR447C	998
YNL302C	YDR450W	914
YNL302C	YDR471W	907
YNL302C	YDR500C	911
YNL302C	YEL054C	929
YNL302C	YER056C-A	905
YNL302C	YER074W	931
YNL302C	YER102W	923
YNL302C	YER117W	922
YNL302C	YER131W	921
YNL302C	YER165W	901
YNL302C	YFL034C-A	915
YNL302C	YFR031C-A	997
YNL302C	YFR032C-A	933
YNL302C	YGL014W	972
YNL302C	YGL030W	936
YNL302C	YGL031C	907
YNL302C	YGL076C	928
YNL302C	YGL103W	997
YNL302C	YGL123W	914
YNL302C	YGL135W	922
YNL302C	YGL147C	918
YNL302C	YGL189C	921
YNL302C	YGR027C	922
YNL302C	YGR034W	997
YNL302C	YGR085C	918
YNL302C	YGR118W	932
YNL302C	YGR148C	907
YNL302C	YGR214W	916
YNL302C	YHL001W	925
YNL302C	YHL015W	939
YNL302C	YHL033C	920
YNL302C	YHR010W	907

YNL302C	YHR021C	968
YNL302C	YHR141C	998
YNL302C	YHR203C	921
YNL302C	YIL018W	911
YNL302C	YIL052C	905
YNL302C	YIL069C	931
YNL302C	YIL133C	923
YNL302C	YIL148W	902
YNL302C	YJL136C	926
YNL302C	YJL177W	997
YNL302C	YJL189W	911
YNL302C	YJL190C	915
YNL302C	YJL191W	927
YNL302C	YJR007W	904
YNL302C	YJR094W-A	931
YNL302C	YJR123W	998
YNL302C	YJR145C	921
YNL302C	YKL006W	925
YNL302C	YKL156W	927
YNL302C	YKL180W	997
YNL302C	YKR057W	926
YNL302C	YKR094C	902
YNL302C	YLL045C	920
YNL302C	YLR029C	919
YNL302C	YLR048W	916
YNL302C	YLR061W	919
YNL302C	YLR075W	918
YNL302C	YLR185W	912
YNL302C	YLR264W	910
YNL302C	YLR287C-A	997
YNL302C	YLR325C	917
YNL302C	YLR333C	921
YNL302C	YLR340W	954
YNL302C	YLR344W	919
YNL302C	YLR367W	915
YNL302C	YLR388W	912
YNL302C	YLR406C	916
YNL302C	YLR441C	926
YNL302C	YLR448W	924
YNL302C	YML024W	928
YNL302C	YML026C	914
YNL302C	YML063W	926
YNL302C	YML073C	946
YNL302C	YMR121C	919

YNL302C	YMR142C	922
YNL302C	YMR194W	998
YNL302C	YMR230W	927
YNL302C	YMR242C	998
YNL302C	YMR260C	911
YNL302C	YNL067W	919
YNL302C	YNL069C	923
YNL302C	YNL096C	921
YNL302C	YNL162W	927
YNL302C	YNL178W	962
YNL302C	YNL301C	920
YNL302C	YOL039W	911
YNL302C	YOL040C	954
YNL302C	YOL120C	920
YNL302C	YOL121C	903
YNL302C	YOL127W	920
YNL302C	YOR063W	924
YNL302C	YOR096W	921
YNL302C	YOR133W	907
YNL302C	YOR167C	910
YNL302C	YOR182C	903
YNL302C	YOR234C	933
YNL302C	YOR293W	927
YNL302C	YOR312C	927
YNL302C	YOR369C	941
YNL302C	YPL079W	919
YNL302C	YPL081W	919
YNL302C	YPL090C	907
YNL302C	YPL131W	928
YNL302C	YPL143W	933
YNL302C	YPL198W	928
YNL302C	YPL220W	922
YNL302C	YPL249C-A	927
YNL302C	YPR043W	931
YNL302C	YPR080W	907
YNL302C	YPR102C	918
YNL302C	YPR132W	932
YNL304W	YLR190W	951
YNL304W	YOR326W	995
YNL306W	Q0140	962
YNL306W	YBL090W	988
YNL306W	YBR146W	988
YNL306W	YBR251W	999
YNL306W	YDL045W-A	986

YNL306W	YDR036C	999
YNL306W	YDR041W	999
YNL306W	YDR175C	988
YNL306W	YDR337W	989
YNL306W	YDR347W	988
YNL306W	YGL129C	988
YNL306W	YGR084C	974
YNL306W	YGR165W	988
YNL306W	YGR215W	988
YNL306W	YHL004W	999
YNL306W	YIL093C	988
YNL306W	YJR101W	988
YNL306W	YJR113C	988
YNL306W	YKL155C	988
YNL306W	YMR158W	974
YNL306W	YMR188C	988
YNL306W	YNL137C	988
YNL306W	YNR037C	987
YNL306W	YOR158W	988
YNL306W	YOR204W	984
YNL306W	YPL013C	997
YNL306W	YPL118W	988
YNL307C	YAL038W	984
YNL307C	YDL065C	938
YNL307C	YDR207C	930
YNL307C	YGL144C	968
YNL307C	YGL197W	910
YNL307C	YGR140W	990
YNL307C	YIR026C	955
YNL307C	YJR094C	978
YNL307C	YKL127W	900
YNL307C	YLR175W	993
YNL307C	YML111W	953
YNL307C	YMR037C	901
YNL307C	YMR139W	979
YNL307C	YMR186W	919
YNL307C	YMR273C	949
YNL307C	YMR275C	946
YNL307C	YPL240C	919
YNL308C	YBL072C	980
YNL308C	YBR048W	980
YNL308C	YCL059C	999
YNL308C	YDL014W	998
YNL308C	YDL148C	989

YNL308C	YDR025W	979
YNL308C	YDR064W	981
YNL308C	YDR299W	978
YNL308C	YDR381W	930
YNL308C	YHR089C	990
YNL308C	YJR145C	980
YNL308C	YLR175W	991
YNL308C	YMR290C	991
YNL308C	YPL012W	982
YNL309W	YBR160W	999
YNL309W	YLR182W	990
YNL309W	YOL004W	989
YNL310C	YJR045C	999
YNL311C	YDR328C	924
YNL311C	YIL074C	958
YNL311C	YKL001C	937
YNL312W	YAR007C	999
YNL312W	YBL023C	903
YNL312W	YBL088C	955
YNL312W	YBR088C	903
YNL312W	YBR114W	919
YNL312W	YBR136W	999
YNL312W	YBR160W	910
YNL312W	YCR028C-A	998
YNL312W	YDL059C	925
YNL312W	YDL102W	903
YNL312W	YDL156W	997
YNL312W	YDR097C	985
YNL312W	YDR311W	978
YNL312W	YDR460W	978
YNL312W	YEL032W	901
YNL312W	YEL037C	928
YNL312W	YER104W	966
YNL312W	YER162C	991
YNL312W	YER171W	987
YNL312W	YGR258C	991
YNL312W	YHR164C	998
YNL312W	YIL128W	910
YNL312W	YIL143C	987
YNL312W	YIR008C	984
YNL312W	YJL106W	982
YNL312W	YJL173C	999
YNL312W	YJR006W	900
YNL312W	YJR052W	919

YNL312W	YLR005W	978
YNL312W	YLR274W	903
YNL312W	YLR288C	954
YNL312W	YML032C	999
YNL312W	YML095C	989
YNL312W	YMR201C	998
YNL312W	YNL102W	910
YNL312W	YPL022W	989
YNL312W	YPR019W	902
YNL313C	YAL043C	940
YNL314W	YBR208C	945
YNL314W	YER040W	921
YNL314W	YIR023W	995
YNL314W	YIR029W	948
YNL314W	YIR032C	952
YNL314W	YKR034W	951
YNL315C	YBL099W	963
YNL315C	YJL180C	966
YNL315C	YJR121W	993
YNL316C	YFL022C	920
YNL316C	YIL116W	954
YNL316C	YPR047W	906
YNL317W	YAL043C	999
YNL317W	YDR195W	999
YNL317W	YDR301W	999
YNL317W	YER133W	999
YNL317W	YGR156W	999
YNL317W	YJL033W	977
YNL317W	YJR093C	999
YNL317W	YKL018W	999
YNL317W	YKL059C	999
YNL317W	YKR002W	999
YNL317W	YLR115W	999
YNL317W	YLR277C	999
YNL317W	YMR061W	999
YNL317W	YNL222W	999
YNL317W	YPR107C	999
YNL321W	YDR456W	926
YNL322C	YGL027C	959
YNL322C	YGR229C	922
YNL322C	YJL174W	968
YNL323W	YAL026C	980
YNL323W	YCR094W	995
YNL323W	YDR093W	997

YNL323W	YER166W	997
YNL323W	YMR022W	923
YNL325C	YCR089W	918
YNL325C	YFR019W	991
YNL325C	YFR021W	902
YNL325C	YJR110W	968
YNL325C	YKL212W	939
YNL325C	YLR386W	999
YNL325C	YNL054W	996
YNL325C	YNL106C	933
YNL325C	YOL065C	934
YNL325C	YOR109W	935
YNL325C	YPL240C	919
YNL326C	YEL013W	992
YNL327W	YDR146C	925
YNL327W	YGL028C	907
YNL327W	YGR023W	999
YNL327W	YIR019C	999
YNL327W	YJR151C	999
YNL327W	YKR102W	999
YNL327W	YLR286C	940
YNL327W	YMR317W	999
YNL327W	YNL066W	911
YNL327W	YNL300W	905
YNL327W	YNR044W	999
YNL328C	YFL016C	991
YNL328C	YIL022W	910
YNL328C	YJL104W	984
YNL329C	YAL055W	917
YNL329C	YDR244W	994
YNL329C	YGL153W	959
YNL329C	YGR133W	930
YNL329C	YKL197C	999
YNL329C	YLR191W	905
YNL329C	YOL044W	999
YNL330C	YAL013W	999
YNL330C	YBR010W	903
YNL330C	YBR095C	999
YNL330C	YBR103W	927
YNL330C	YBR112C	996
YNL330C	YBR195C	905
YNL330C	YCR084C	998
YNL330C	YDL042C	994
YNL330C	YDL076C	999

YNL330C	YDR146C	976
YNL330C	YDR207C	999
YNL330C	YGL194C	998
YNL330C	YGR252W	998
YNL330C	YIL084C	999
YNL330C	YJL129C	976
YNL330C	YJR032W	976
YNL330C	YJR094C	951
YNL330C	YKL185W	999
YNL330C	YKR029C	951
YNL330C	YLR103C	973
YNL330C	YLR113W	980
YNL330C	YLR216C	976
YNL330C	YML069W	974
YNL330C	YMR075W	999
YNL330C	YMR172W	926
YNL330C	YMR263W	999
YNL330C	YNL021W	995
YNL330C	YNL097C	999
YNL330C	YOL004W	999
YNL330C	YOR304W	986
YNL330C	YPL116W	948
YNL330C	YPL139C	999
YNL330C	YPL181W	999
YNL330C	YPR023C	999
YNL330C	YPR068C	929
YNL331C	YPL070W	923
YNL333W	YFL059W	999
YNL333W	YFL060C	993
YNL333W	YHR029C	935
YNL333W	YMR095C	999
YNL333W	YMR096W	999
YNL333W	YNL334C	996
YNL334C	YBR035C	908
YNL334C	YFL059W	997
YNL334C	YFL060C	957
YNL334C	YMR095C	993
YNL334C	YMR096W	985
YNL334C	YNL333W	996
YNR001C	YAL038W	901
YNR001C	YBR218C	920
YNR001C	YCR005C	999
YNR001C	YDL078C	913
YNR001C	YGL062W	920

YNR001C	YGL252C	928
YNR001C	YGR254W	917
YNR001C	YHR174W	917
YNR001C	YIR031C	900
YNR001C	YJL200C	926
YNR001C	YKL085W	997
YNR001C	YKR052C	998
YNR001C	YLR304C	996
YNR001C	YNL117W	923
YNR001C	YOL067C	909
YNR001C	YOL126C	925
YNR001C	YOR136W	987
YNR001C	YPR001W	970
YNR003C	YBL034C	946
YNR003C	YBR154C	999
YNR003C	YDL150W	999
YNR003C	YDR005C	997
YNR003C	YDR045C	999
YNR003C	YDR156W	921
YNR003C	YER148W	909
YNR003C	YFR037C	981
YNR003C	YGR047C	984
YNR003C	YGR246C	998
YNR003C	YHR143W-A	960
YNR003C	YJL011C	999
YNR003C	YJL148W	921
YNR003C	YJR063W	921
YNR003C	YKL144C	999
YNR003C	YKR025W	999
YNR003C	YNL039W	976
YNR003C	YNL113W	998
YNR003C	YNL151C	999
YNR003C	YNL248C	908
YNR003C	YOR116C	999
YNR003C	YOR207C	999
YNR003C	YOR210W	997
YNR003C	YOR224C	999
YNR003C	YOR340C	921
YNR003C	YOR341W	921
YNR003C	YPR010C	921
YNR003C	YPR110C	999
YNR003C	YPR187W	999
YNR003C	YPR190C	999
YNR004W	YPL157W	971

YNR006W	YCL008C	998
YNR006W	YDL145C	908
YNR006W	YDR069C	972
YNR006W	YDR153C	905
YNR006W	YGL137W	901
YNR006W	YHL002W	999
YNR006W	YIL076W	905
YNR006W	YJR110W	999
YNR006W	YLL039C	996
YNR007C	YBL078C	999
YNR007C	YBR217W	999
YNR007C	YHR171W	999
YNR007C	YNL223W	959
YNR007C	YOR160W	933
YNR007C	YPL149W	986
YNR008W	YNR019W	959
YNR008W	YOR245C	998
YNR010W	YBL093C	999
YNR010W	YBR193C	999
YNR010W	YBR253W	999
YNR010W	YCR081W	985
YNR010W	YDL005C	999
YNR010W	YDL140C	982
YNR010W	YDR145W	954
YNR010W	YDR167W	995
YNR010W	YDR308C	999
YNR010W	YDR443C	997
YNR010W	YER022W	999
YNR010W	YGL025C	999
YNR010W	YGL127C	999
YNR010W	YGL151W	998
YNR010W	YGR097W	911
YNR010W	YGR104C	999
YNR010W	YHR041C	999
YNR010W	YHR058C	999
YNR010W	YIL021W	982
YNR010W	YJL140W	982
YNR010W	YLL002W	957
YNR010W	YLR071C	999
YNR010W	YLR418C	912
YNR010W	YML007W	974
YNR010W	YMR112C	999
YNR010W	YNL236W	999
YNR010W	YOL051W	999

YNR010W	YOL135C	999
YNR010W	YOR123C	950
YNR010W	YOR151C	982
YNR010W	YOR174W	999
YNR010W	YPL042C	992
YNR010W	YPL129W	991
YNR010W	YPR070W	999
YNR010W	YPR168W	999
YNR011C	YER172C	940
YNR011C	YKL173W	911
YNR011C	YOR148C	976
YNR012W	YDR020C	988
YNR012W	YEL021W	958
YNR012W	YHR128W	992
YNR012W	YKL024C	945
YNR012W	YLR245C	942
YNR013C	YML123C	987
YNR016C	YAL054C	902
YNR016C	YDR138W	930
YNR016C	YDR477W	995
YNR016C	YGL115W	953
YNR016C	YKL182W	926
YNR016C	YLR153C	969
YNR016C	YMR207C	987
YNR016C	YNL071W	918
YNR016C	YPL028W	929
YNR016C	YPL231W	974
YNR017W	YBL045C	969
YNR017W	YBR091C	969
YNR017W	YEL020W-A	967
YNR017W	YEL030W	906
YNR017W	YGR033C	999
YNR017W	YGR181W	999
YNR017W	YHR005C-A	978
YNR017W	YIL022W	999
YNR017W	YJL054W	957
YNR017W	YJL104W	999
YNR017W	YJL143W	999
YNR017W	YJR045C	999
YNR017W	YJR135W-A	999
YNR017W	YKR065C	999
YNR017W	YLR008C	999
YNR017W	YMR203W	989
YNR017W	YNL121C	939

YNR017W	YNL131W	984
YNR017W	YOR232W	966
YNR017W	YPL063W	999
YNR019W	YCR048W	999
YNR019W	YNR008W	959
YNR019W	YOR245C	959
YNR020C	YPL240C	919
YNR022C	YCR046C	915
YNR023W	YBR289W	999
YNR023W	YDR073W	998
YNR023W	YEL009C	988
YNR023W	YFL049W	999
YNR023W	YHL025W	999
YNR023W	YJL176C	999
YNR023W	YMR033W	999
YNR023W	YOR290C	999
YNR023W	YPL016W	999
YNR023W	YPL129W	999
YNR023W	YPR034W	999
YNR024W	YCR035C	960
YNR024W	YDL111C	959
YNR024W	YDR280W	959
YNR024W	YGR095C	959
YNR024W	YGR158C	959
YNR024W	YGR195W	959
YNR024W	YHR069C	998
YNR024W	YNL232W	998
YNR024W	YOL021C	998
YNR024W	YOL142W	998
YNR024W	YOR001W	999
YNR026C	YCL001W	959
YNR026C	YDR480W	925
YNR026C	YGR172C	969
YNR026C	YHR079C	991
YNR026C	YPL204W	977
YNR026C	YPL218W	999
YNR027W	YBR035C	936
YNR027W	YMR095C	901
YNR027W	YMR096W	900
YNR030W	YML019W	921
YNR030W	YNL219C	999
YNR030W	YOR098C	906
YNR031C	YDL006W	973
YNR031C	YDL022W	976

YNR031C	YDL235C	937
YNR031C	YER118C	995
YNR031C	YJL128C	999
YNR031C	YLR006C	999
YNR032C-A	YDL223C	939
YNR032C-A	YOR308C	986
YNR032W	YGR123C	979
YNR032W	YLR216C	917
YNR032W	YMR028W	985
YNR032W	YMR186W	971
YNR033W	YNL249C	958
YNR034W	YCR073W-A	922
YNR034W	YDR248C	914
YNR034W	YGR256W	937
YNR034W	YHR183W	937
YNR034W	YJL047C	998
YNR034W	YKL205W	998
YNR034W	YLR028C	908
YNR034W	YNL241C	972
YNR034W	YOL063C	998
YNR035C	YBR109C	999
YNR035C	YBR234C	999
YNR035C	YDL029W	999
YNR035C	YIL062C	999
YNR035C	YJR065C	999
YNR035C	YKL013C	999
YNR035C	YLR370C	999
YNR035C	YLR429W	997
YNR035C	YMR109W	922
YNR035C	YOR181W	935
YNR036C	Q0140	962
YNR036C	YBL038W	937
YNR036C	YBR146W	988
YNR036C	YBR251W	965
YNR036C	YDR041W	990
YNR036C	YDR116C	912
YNR036C	YDR337W	941
YNR036C	YER050C	999
YNR036C	YGL123W	978
YNR036C	YGR220C	904
YNR036C	YHL004W	986
YNR036C	YHR147C	903
YNR036C	YJL063C	940
YNR036C	YJR113C	999

YNR036C	YLR069C	959
YNR036C	YMR158W	980
YNR036C	YNL081C	999
YNR036C	YNL185C	971
YNR036C	YNR037C	999
YNR036C	YOR187W	963
YNR036C	YPL013C	988
YNR036C	YPR166C	999
YNR037C	Q0140	962
YNR037C	YBL027W	931
YNR037C	YBL038W	934
YNR037C	YBL087C	980
YNR037C	YBL090W	987
YNR037C	YBL092W	944
YNR037C	YBR031W	935
YNR037C	YBR048W	953
YNR037C	YBR084C-A	931
YNR037C	YBR118W	933
YNR037C	YBR146W	999
YNR037C	YBR251W	999
YNR037C	YCR031C	985
YNR037C	YDL061C	910
YNR037C	YDR012W	935
YNR037C	YDR025W	953
YNR037C	YDR041W	999
YNR037C	YDR175C	987
YNR037C	YDR337W	996
YNR037C	YDR347W	987
YNR037C	YDR450W	950
YNR037C	YEL050C	924
YNR037C	YER117W	980
YNR037C	YFR031C-A	982
YNR037C	YGL076C	901
YNR037C	YGL103W	917
YNR037C	YGL123W	996
YNR037C	YGL129C	987
YNR037C	YGL135W	900
YNR037C	YGL147C	938
YNR037C	YGR034W	933
YNR037C	YGR084C	972
YNR037C	YGR085C	982
YNR037C	YGR215W	987
YNR037C	YGR220C	976
YNR037C	YHL004W	999

YNR037C	YHR203C	939
YNR037C	YIL018W	982
YNR037C	YIL093C	987
YNR037C	YJL063C	936
YNR037C	YJL177W	941
YNR037C	YJL190C	956
YNR037C	YJL191W	984
YNR037C	YJR101W	987
YNR037C	YJR113C	999
YNR037C	YJR123W	969
YNR037C	YJR145C	939
YNR037C	YKL155C	997
YNR037C	YKL170W	985
YNR037C	YKL180W	942
YNR037C	YLR048W	902
YNR037C	YLR340W	906
YNR037C	YLR344W	933
YNR037C	YLR367W	955
YNR037C	YLR388W	917
YNR037C	YML026C	950
YNR037C	YMR188C	997
YNR037C	YNL067W	939
YNR037C	YNL081C	995
YNR037C	YNL178W	988
YNR037C	YNL306W	987
YNR037C	YNR036C	999
YNR037C	YOL040C	908
YNR037C	YOL127W	952
YNR037C	YOR063W	956
YNR037C	YOR158W	987
YNR037C	YPL013C	952
YNR037C	YPL118W	987
YNR037C	YPL131W	951
YNR037C	YPL198W	904
YNR037C	YPL220W	900
YNR037C	YPR080W	933
YNR037C	YPR102C	982
YNR038W	YJR041C	982
YNR038W	YKL014C	912
YNR038W	YKR024C	910
YNR038W	YLR221C	997
YNR038W	YOL144W	998
YNR038W	YOR063W	943
YNR038W	YPL193W	945

YNR039C	YDR205W	995
YNR041C	YBR003W	995
YNR041C	YGL119W	904
YNR041C	YGR255C	907
YNR041C	YML110C	929
YNR041C	YOL008W	978
YNR041C	YOL096C	954
YNR043W	YJL167W	939
YNR043W	YMR220W	969
YNR043W	YPL117C	978
YNR044W	YNL327W	999
YNR045W	Q0275	999
YNR045W	YGR222W	960
YNR045W	YMR257C	943
YNR046W	YDR120C	918
YNR046W	YDR140W	999
YNR046W	YML014W	998
YNR046W	YNR050C	969
YNR046W	YOL124C	999
YNR049C	YAL030W	997
YNR049C	YDR164C	999
YNR049C	YFL005W	984
YNR049C	YGL233W	998
YNR049C	YGR009C	999
YNR049C	YIL068C	918
YNR049C	YMR183C	934
YNR049C	YPL232W	990
YNR049C	YPR055W	959
YNR050C	YBR115C	930
YNR050C	YDR516C	925
YNR050C	YGL154C	939
YNR050C	YIR034C	998
YNR050C	YNR046W	969
YNR050C	YOR164C	957
YNR051C	YAL048C	983
YNR051C	YER151C	999
YNR051C	YGR170W	923
YNR051C	YJL168C	952
YNR051C	YPL240C	919
YNR052C	YAL021C	999
YNR052C	YBL035C	987
YNR052C	YBR088C	903
YNR052C	YCR093W	999
YNR052C	YDL101C	911

YNR052C	YDL160C	999
YNR052C	YDL165W	999
YNR052C	YER068W	999
YNR052C	YER165W	986
YNR052C	YGL014W	976
YNR052C	YGL178W	999
YNR052C	YGR092W	985
YNR052C	YGR134W	999
YNR052C	YIL038C	999
YNR052C	YIR008C	989
YNR052C	YJL115W	936
YNR052C	YKL045W	987
YNR052C	YLR150W	982
YNR052C	YMR025W	900
YNR052C	YNL091W	927
YNR052C	YNL102W	989
YNR052C	YNL197C	913
YNR052C	YNL206C	986
YNR052C	YNL288W	999
YNR052C	YOL149W	980
YNR052C	YPR072W	999
YNR053C	YCR072C	993
YNR053C	YDR101C	985
YNR053C	YER006W	996
YNR053C	YER126C	999
YNR053C	YGR103W	998
YNR053C	YGR245C	999
YNR053C	YHR010W	974
YNR053C	YHR085W	990
YNR053C	YHR197W	995
YNR053C	YIL026C	927
YNR053C	YKL009W	993
YNR053C	YKR081C	999
YNR053C	YLR009W	985
YNR053C	YLR074C	998
YNR053C	YLR106C	989
YNR053C	YLR325C	970
YNR053C	YNL002C	997
YNR053C	YNL182C	994
YNR053C	YOL127W	927
YNR053C	YOR063W	987
YNR053C	YPL093W	999
YNR053C	YPL131W	996
YNR053C	YPL249C-A	978

YNR053C	YPR016C	999
YNR054C	YBR048W	970
YNR054C	YCR057C	961
YNR054C	YDL014W	919
YNR054C	YDR299W	970
YNR054C	YDR365C	999
YNR054C	YGR145W	997
YNR054C	YHR169W	965
YNR054C	YLR129W	933
YNR057C	YGR286C	985
YNR057C	YNR058W	998
YNR058W	YGR286C	986
YNR058W	YNR057C	998
YNR060W	YGL071W	954
YNR068C	YNR069C	919
YNR069C	YLL049W	926
YNR069C	YNR068C	919
YNR071C	YPL240C	919
YOL001W	YBR093C	935
YOL001W	YDL106C	983
YOL001W	YDR146C	937
YOL001W	YFL033C	900
YOL001W	YFR034C	999
YOL001W	YGR233C	999
YOL001W	YML123C	907
YOL001W	YPL031C	999
YOL002C	YJL056C	911
YOL003C	YBR023C	918
YOL003C	YGR229C	931
YOL004W	YAL013W	999
YOL004W	YAL034C	902
YOL004W	YBR095C	999
YOL004W	YBR103W	937
YOL004W	YCR033W	916
YOL004W	YCR084C	947
YOL004W	YDL076C	999
YOL004W	YDR146C	999
YOL004W	YDR169C	904
YOL004W	YDR207C	999
YOL004W	YFR013W	954
YOL004W	YGL194C	967
YOL004W	YGR252W	987
YOL004W	YHL020C	992
YOL004W	YHR027C	940

YOL004W	YIL084C	999
YOL004W	YJL129C	996
YOL004W	YJL153C	915
YOL004W	YKL072W	973
YOL004W	YKL185W	999
YOL004W	YKR029C	921
YOL004W	YKR050W	913
YOL004W	YLR103C	968
YOL004W	YLR182W	972
YOL004W	YMR019W	904
YOL004W	YMR053C	976
YOL004W	YMR075W	999
YOL004W	YMR263W	999
YOL004W	YNL097C	999
YOL004W	YNL216W	916
YOL004W	YNL309W	989
YOL004W	YNL330C	999
YOL004W	YPL016W	982
YOL004W	YPL139C	999
YOL004W	YPL181W	999
YOL004W	YPL256C	962
YOL004W	YPR023C	999
YOL005C	YBL026W	917
YOL005C	YBR154C	999
YOL005C	YDL115C	997
YOL005C	YDL140C	999
YOL005C	YDR404C	999
YOL005C	YEL026W	904
YOL005C	YER022W	996
YOL005C	YER029C	910
YOL005C	YER171W	902
YOL005C	YGL043W	999
YOL005C	YGL070C	999
YOL005C	YGL112C	901
YOL005C	YGR005C	999
YOL005C	YGR063C	987
YOL005C	YGR074W	914
YOL005C	YGR104C	983
YOL005C	YGR186W	998
YOL005C	YHR143W-A	998
YOL005C	YIL021W	999
YOL005C	YIL143C	903
YOL005C	YJL140W	999
YOL005C	YKL067W	902

YOL005C	YLR147C	919
YOL005C	YLR275W	909
YOL005C	YML010W	999
YOL005C	YMR277W	963
YOL005C	YOL135C	909
YOL005C	YOR116C	900
YOL005C	YOR151C	999
YOL005C	YOR159C	942
YOL005C	YOR207C	901
YOL005C	YOR210W	999
YOL005C	YOR224C	999
YOL005C	YPL129W	999
YOL005C	YPR086W	902
YOL005C	YPR168W	932
YOL005C	YPR182W	931
YOL005C	YPR187W	999
YOL006C	YBL002W	903
YOL006C	YBR049C	918
YOL006C	YBR223C	993
YOL006C	YDL064W	980
YOL006C	YDR138W	999
YOL006C	YDR156W	959
YOL006C	YGR162W	940
YOL006C	YJL148W	998
YOL006C	YLR103C	923
YOL006C	YLR234W	978
YOL006C	YMR190C	999
YOL006C	YMR233W	956
YOL006C	YNL088W	999
YOL006C	YOL115W	998
YOL008W	YGL119W	970
YOL008W	YNR041C	978
YOL008W	YOR125C	930
YOL009C	YAL010C	990
YOL009C	YGR132C	918
YOL009C	YGR231C	917
YOL009C	YHR194W	913
YOL009C	YLL006W	993
YOL009C	YOR147W	913
YOL010W	YBL004W	999
YOL010W	YBR247C	996
YOL010W	YCL059C	920
YOL010W	YCR057C	972
YOL010W	YDL014W	984

YOL010W	YDL060W	946
YOL010W	YDL148C	999
YOL010W	YDL213C	920
YOL010W	YDR299W	934
YOL010W	YDR324C	916
YOL010W	YDR449C	919
YOL010W	YGL171W	922
YOL010W	YGR128C	914
YOL010W	YHR148W	926
YOL010W	YHR169W	929
YOL010W	YHR196W	971
YOL010W	YHR203C	972
YOL010W	YJL109C	987
YOL010W	YJR002W	997
YOL010W	YKR060W	914
YOL010W	YLL011W	931
YOL010W	YLR129W	939
YOL010W	YLR175W	908
YOL010W	YLR186W	910
YOL010W	YLR197W	915
YOL010W	YLR222C	919
YOL010W	YLR409C	923
YOL010W	YML093W	909
YOL010W	YMR093W	931
YOL010W	YMR128W	999
YOL010W	YNL075W	941
YOL010W	YNL132W	913
YOL010W	YOR004W	919
YOL010W	YOR145C	915
YOL010W	YPL012W	910
YOL010W	YPL126W	918
YOL010W	YPL217C	999
YOL010W	YPR112C	923
YOL010W	YPR137W	993
YOL010W	YPR144C	919
YOL011W	YLR075W	917
YOL012C	YAL011W	995
YOL012C	YAL013W	910
YOL012C	YAR002W	989
YOL012C	YAR003W	976
YOL012C	YBL002W	999
YOL012C	YBL003C	995
YOL012C	YBL052C	953
YOL012C	YBR009C	999

YOL012C	YBR010W	995
YOL012C	YBR095C	913
YOL012C	YBR103W	968
YOL012C	YBR175W	990
YOL012C	YBR231C	971
YOL012C	YBR245C	978
YOL012C	YCL061C	911
YOL012C	YCR065W	900
YOL012C	YDL074C	999
YOL012C	YDR159W	964
YOL012C	YDR190C	997
YOL012C	YDR224C	999
YOL012C	YDR225W	995
YOL012C	YDR289C	904
YOL012C	YDR334W	999
YOL012C	YDR359C	976
YOL012C	YDR363W-A	943
YOL012C	YDR392W	922
YOL012C	YDR469W	958
YOL012C	YDR485C	999
YOL012C	YEL003W	928
YOL012C	YEL018W	991
YOL012C	YER151C	970
YOL012C	YFL039C	997
YOL012C	YGL043W	973
YOL012C	YGL058W	993
YOL012C	YGL127C	942
YOL012C	YGL194C	976
YOL012C	YGL207W	993
YOL012C	YGL241W	995
YOL012C	YGL244W	986
YOL012C	YGR002C	999
YOL012C	YGR078C	974
YOL012C	YGR252W	998
YOL012C	YIL040W	944
YOL012C	YIR005W	905
YOL012C	YJL019W	957
YOL012C	YJL081C	999
YOL012C	YJL115W	978
YOL012C	YJL168C	999
YOL012C	YKR048C	999
YOL012C	YLR015W	909
YOL012C	YLR055C	989
YOL012C	YLR085C	996

YOL012C	YLR262C	983
YOL012C	YLR399C	998
YOL012C	YLR418C	993
YOL012C	YML034W	960
YOL012C	YML041C	999
YOL012C	YML124C	945
YOL012C	YMR263W	984
YOL012C	YNL030W	994
YOL012C	YNL031C	969
YOL012C	YNL097C	973
YOL012C	YNL107W	998
YOL012C	YNL136W	971
YOL012C	YNL153C	987
YOL012C	YOL072W	957
YOL012C	YOR123C	986
YOL012C	YOR244W	969
YOL012C	YOR349W	919
YOL012C	YPL055C	999
YOL012C	YPL127C	959
YOL012C	YPL181W	963
YOL012C	YPL235W	997
YOL012C	YPR023C	916
YOL013C	YBR170C	995
YOL013C	YBR201W	999
YOL013C	YDL126C	999
YOL013C	YDR057W	997
YOL013C	YER125W	901
YOL013C	YIL030C	991
YOL013C	YLR207W	999
YOL013C	YLR378C	993
YOL013C	YML013W	997
YOL013C	YML029W	994
YOL013C	YMR022W	985
YOL013C	YMR297W	917
YOL013C	YOR016C	923
YOL013C	YOR044W	923
YOL016C	YBR109C	999
YOL016C	YFR014C	963
YOL017W	YBR245C	998
YOL017W	YDL042C	978
YOL017W	YDR363W	917
YOL017W	YOL051W	982
YOL018C	YAL030W	989
YOL018C	YBL050W	977

YOL018C	YBR164C	931
YOL018C	YDL100C	954
YOL018C	YDL192W	959
YOL018C	YDR027C	996
YOL018C	YDR164C	921
YOL018C	YDR468C	999
YOL018C	YGL095C	999
YOL018C	YJL004C	944
YOL018C	YJL029C	912
YOL018C	YML071C	911
YOL018C	YMR197C	999
YOL018C	YNL041C	907
YOL018C	YNL051W	905
YOL018C	YOR327C	998
YOL018C	YPL051W	928
YOL020W	YBR058C	903
YOL020W	YDR007W	999
YOL020W	YJL091C	909
YOL021C	YCR035C	999
YOL021C	YDL111C	999
YOL021C	YDR280W	999
YOL021C	YGL097W	943
YOL021C	YGR095C	999
YOL021C	YGR158C	999
YOL021C	YGR195W	999
YOL021C	YHR069C	999
YOL021C	YHR081W	999
YOL021C	YLR163C	938
YOL021C	YLR293C	987
YOL021C	YNL189W	990
YOL021C	YNL232W	999
YOL021C	YNR024W	998
YOL021C	YOL142W	999
YOL021C	YOR001W	999
YOL021C	YOR076C	999
YOL023W	YDR494W	937
YOL030W	YMR307W	936
YOL031C	YHR079C	959
YOL031C	YJL034W	999
YOL031C	YKL073W	996
YOL031C	YNL238W	999
YOL031C	YOR254C	955
YOL033W	YAL062W	914
YOL033W	YGL245W	922

YOL033W	YLR027C	906
YOL033W	YMR250W	902
YOL033W	YOR375C	914
YOL033W	YPR035W	901
YOL034W	YDL105W	987
YOL034W	YDR288W	996
YOL034W	YDR510W	942
YOL034W	YEL019C	996
YOL034W	YLR007W	990
YOL034W	YMR117C	979
YOL038W	YBL041W	999
YOL038W	YBR173C	958
YOL038W	YDL097C	979
YOL038W	YDL147W	981
YOL038W	YDL188C	923
YOL038W	YDR394W	998
YOL038W	YDR427W	973
YOL038W	YER012W	999
YOL038W	YER021W	999
YOL038W	YER094C	999
YOL038W	YFL007W	998
YOL038W	YFR004W	940
YOL038W	YFR050C	999
YOL038W	YFR052W	910
YOL038W	YGL011C	999
YOL038W	YGL048C	932
YOL038W	YGR135W	999
YOL038W	YGR253C	999
YOL038W	YHL030W	997
YOL038W	YHR027C	932
YOL038W	YHR200W	996
YOL038W	YIL075C	938
YOL038W	YIL148W	912
YOL038W	YJL001W	999
YOL038W	YKL145W	994
YOL038W	YLR167W	911
YOL038W	YML092C	999
YOL038W	YMR314W	999
YOL038W	YOR157C	999
YOL038W	YOR259C	914
YOL038W	YOR261C	977
YOL038W	YOR362C	999
YOL038W	YPR103W	999
YOL038W	YPR108W	978

YOL039W	YBL027W	912
YOL039W	YBL072C	911
YOL039W	YBL087C	922
YOL039W	YBL092W	944
YOL039W	YBR031W	954
YOL039W	YBR048W	909
YOL039W	YBR084C-A	912
YOL039W	YBR118W	904
YOL039W	YBR181C	901
YOL039W	YBR189W	946
YOL039W	YBR191W	941
YOL039W	YCR031C	935
YOL039W	YDL061C	923
YOL039W	YDL075W	928
YOL039W	YDL081C	998
YOL039W	YDL082W	947
YOL039W	YDL130W	999
YOL039W	YDL136W	905
YOL039W	YDL191W	905
YOL039W	YDR012W	954
YOL039W	YDR064W	953
YOL039W	YDR382W	998
YOL039W	YDR385W	997
YOL039W	YDR418W	990
YOL039W	YDR447C	913
YOL039W	YDR450W	913
YOL039W	YDR471W	916
YOL039W	YER056C-A	913
YOL039W	YER102W	911
YOL039W	YER117W	911
YOL039W	YER131W	923
YOL039W	YFR031C-A	934
YOL039W	YFR032C-A	913
YOL039W	YGL030W	923
YOL039W	YGL031C	932
YOL039W	YGL076C	945
YOL039W	YGL103W	919
YOL039W	YGL123W	931
YOL039W	YGL135W	908
YOL039W	YGL147C	923
YOL039W	YGR027C	947
YOL039W	YGR034W	910
YOL039W	YGR085C	940
YOL039W	YGR118W	930

YOL039W	YGR148C	929
YOL039W	YGR214W	952
YOL039W	YHL001W	953
YOL039W	YHL015W	936
YOL039W	YHL033C	944
YOL039W	YHR010W	923
YOL039W	YHR021C	904
YOL039W	YHR141C	913
YOL039W	YHR203C	910
YOL039W	YIL018W	913
YOL039W	YIL052C	921
YOL039W	YIL133C	911
YOL039W	YIL148W	900
YOL039W	YJL136C	929
YOL039W	YJL177W	927
YOL039W	YJL189W	905
YOL039W	YJL190C	916
YOL039W	YJL191W	939
YOL039W	YJR094W-A	924
YOL039W	YJR123W	941
YOL039W	YJR145C	910
YOL039W	YKL006W	952
YOL039W	YKL156W	915
YOL039W	YKL180W	936
YOL039W	YKR057W	913
YOL039W	YKR094C	900
YOL039W	YLL045C	940
YOL039W	YLR029C	931
YOL039W	YLR048W	965
YOL039W	YLR061W	923
YOL039W	YLR075W	916
YOL039W	YLR264W	928
YOL039W	YLR287C-A	996
YOL039W	YLR333C	921
YOL039W	YLR340W	999
YOL039W	YLR344W	922
YOL039W	YLR367W	916
YOL039W	YLR388W	930
YOL039W	YLR406C	911
YOL039W	YLR441C	907
YOL039W	YLR448W	938
YOL039W	YML024W	925
YOL039W	YML026C	913
YOL039W	YML063W	925

YOL039W	YML073C	910
YOL039W	YMR121C	910
YOL039W	YMR142C	927
YOL039W	YMR194W	925
YOL039W	YMR230W	918
YOL039W	YMR242C	913
YOL039W	YNL067W	930
YOL039W	YNL069C	997
YOL039W	YNL162W	913
YOL039W	YNL178W	946
YOL039W	YNL301C	912
YOL039W	YNL302C	911
YOL039W	YOL040C	933
YOL039W	YOL120C	912
YOL039W	YOL121C	912
YOL039W	YOL127W	997
YOL039W	YOR063W	934
YOL039W	YOR096W	923
YOL039W	YOR133W	908
YOL039W	YOR167C	925
YOL039W	YOR234C	932
YOL039W	YOR293W	923
YOL039W	YOR312C	913
YOL039W	YOR369C	915
YOL039W	YPL079W	942
YOL039W	YPL081W	916
YOL039W	YPL090C	901
YOL039W	YPL131W	925
YOL039W	YPL143W	926
YOL039W	YPL198W	910
YOL039W	YPL220W	908
YOL039W	YPL249C-A	913
YOL039W	YPR043W	913
YOL039W	YPR080W	904
YOL039W	YPR102C	953
YOL039W	YPR132W	913
YOL040C	YBL027W	999
YOL040C	YBL072C	944
YOL040C	YBL087C	988
YOL040C	YBL092W	999
YOL040C	YBR031W	986
YOL040C	YBR048W	998
YOL040C	YBR084C-A	979
YOL040C	YBR118W	916

YOL040C	YBR181C	997
YOL040C	YBR189W	996
YOL040C	YBR191W	945
YOL040C	YCR031C	994
YOL040C	YDL061C	996
YOL040C	YDL075W	996
YOL040C	YDL081C	976
YOL040C	YDL082W	953
YOL040C	YDL083C	998
YOL040C	YDL130W	901
YOL040C	YDL136W	997
YOL040C	YDL191W	952
YOL040C	YDR012W	986
YOL040C	YDR025W	989
YOL040C	YDR064W	999
YOL040C	YDR382W	939
YOL040C	YDR385W	991
YOL040C	YDR418W	950
YOL040C	YDR429C	900
YOL040C	YDR447C	998
YOL040C	YDR450W	995
YOL040C	YDR471W	995
YOL040C	YDR500C	923
YOL040C	YEL054C	950
YOL040C	YER056C-A	900
YOL040C	YER074W	997
YOL040C	YER102W	944
YOL040C	YER117W	986
YOL040C	YER131W	931
YOL040C	YFL034C-A	910
YOL040C	YFR031C-A	999
YOL040C	YFR032C-A	999
YOL040C	YGL014W	978
YOL040C	YGL030W	964
YOL040C	YGL031C	921
YOL040C	YGL076C	955
YOL040C	YGL103W	997
YOL040C	YGL123W	999
YOL040C	YGL135W	944
YOL040C	YGL147C	999
YOL040C	YGL189C	931
YOL040C	YGR027C	955
YOL040C	YGR034W	999
YOL040C	YGR085C	999

YOL040C	YGR118W	999
YOL040C	YGR148C	922
YOL040C	YGR214W	994
YOL040C	YHL001W	998
YOL040C	YHL015W	999
YOL040C	YHL033C	937
YOL040C	YHR010W	902
YOL040C	YHR021C	959
YOL040C	YHR141C	998
YOL040C	YHR203C	982
YOL040C	YIL018W	999
YOL040C	YIL052C	901
YOL040C	YIL069C	945
YOL040C	YIL133C	950
YOL040C	YIL148W	913
YOL040C	YJL136C	956
YOL040C	YJL138C	901
YOL040C	YJL177W	999
YOL040C	YJL189W	919
YOL040C	YJL190C	998
YOL040C	YJL191W	952
YOL040C	YJR094W-A	952
YOL040C	YJR123W	999
YOL040C	YJR145C	982
YOL040C	YKL006W	953
YOL040C	YKL156W	955
YOL040C	YKL180W	999
YOL040C	YKR057W	956
YOL040C	YKR059W	901
YOL040C	YKR094C	913
YOL040C	YLL045C	945
YOL040C	YLR029C	997
YOL040C	YLR048W	951
YOL040C	YLR061W	912
YOL040C	YLR075W	966
YOL040C	YLR167W	904
YOL040C	YLR185W	924
YOL040C	YLR264W	925
YOL040C	YLR287C-A	997
YOL040C	YLR325C	969
YOL040C	YLR333C	953
YOL040C	YLR340W	981
YOL040C	YLR344W	984
YOL040C	YLR367W	981

YOL040C	YLR388W	978
YOL040C	YLR406C	936
YOL040C	YLR441C	997
YOL040C	YLR448W	997
YOL040C	YML024W	956
YOL040C	YML026C	999
YOL040C	YML063W	997
YOL040C	YML073C	998
YOL040C	YMR121C	932
YOL040C	YMR142C	943
YOL040C	YMR194W	997
YOL040C	YMR230W	948
YOL040C	YMR242C	998
YOL040C	YMR260C	909
YOL040C	YNL067W	985
YOL040C	YNL069C	997
YOL040C	YNL096C	997
YOL040C	YNL162W	948
YOL040C	YNL178W	999
YOL040C	YNL301C	944
YOL040C	YNL302C	954
YOL040C	YNR037C	908
YOL040C	YOL039W	933
YOL040C	YOL120C	944
YOL040C	YOL121C	956
YOL040C	YOL127W	999
YOL040C	YOL139C	902
YOL040C	YOR063W	999
YOL040C	YOR096W	997
YOL040C	YOR133W	919
YOL040C	YOR167C	925
YOL040C	YOR198C	998
YOL040C	YOR234C	955
YOL040C	YOR293W	949
YOL040C	YOR312C	952
YOL040C	YOR369C	971
YOL040C	YPL079W	945
YOL040C	YPL081W	994
YOL040C	YPL090C	995
YOL040C	YPL131W	985
YOL040C	YPL143W	955
YOL040C	YPL198W	957
YOL040C	YPL220W	944
YOL040C	YPL237W	914

YOL040C	YPL249C-A	956
YOL040C	YPR043W	952
YOL040C	YPR080W	916
YOL040C	YPR102C	983
YOL040C	YPR132W	970
YOL041C	YBR142W	998
YOL041C	YDL082W	927
YOL041C	YDR012W	982
YOL041C	YDR060W	998
YOL041C	YGL076C	933
YOL041C	YHR052W	994
YOL041C	YHR203C	925
YOL041C	YIL133C	964
YOL041C	YLL045C	997
YOL041C	YLR002C	979
YOL041C	YLR029C	988
YOL041C	YLR196W	999
YOL041C	YLR448W	925
YOL041C	YML073C	927
YOL041C	YMR229C	985
YOL041C	YMR242C	937
YOL041C	YMR290C	988
YOL041C	YNL002C	985
YOL041C	YNL061W	998
YOL041C	YOL077C	999
YOL041C	YOL127W	929
YOL041C	YOR063W	931
YOL041C	YOR206W	991
YOL041C	YOR272W	979
YOL041C	YOR312C	946
YOL041C	YPL012W	980
YOL041C	YPL043W	999
YOL041C	YPL131W	956
YOL041C	YPL198W	965
YOL043C	YAL015C	986
YOL043C	YKL114C	970
YOL043C	YML060W	961
YOL043C	YMR201C	926
YOL043C	YPL022W	943
YOL044W	YAL055W	981
YOL044W	YDR244W	966
YOL044W	YDR265W	969
YOL044W	YGL153W	969
YOL044W	YKL197C	996

YOL044W	YLR191W	980
YOL044W	YNL214W	969
YOL044W	YNL329C	999
YOL045W	YAL017W	987
YOL045W	YKL035W	982
YOL049W	YBR244W	910
YOL049W	YIR037W	929
YOL049W	YJL101C	997
YOL049W	YKL026C	907
YOL049W	YLR299W	919
YOL049W	YPL091W	914
YOL051W	YAL021C	926
YOL051W	YBL093C	999
YOL051W	YBR193C	999
YOL051W	YBR253W	999
YOL051W	YBR279W	997
YOL051W	YCR042C	984
YOL051W	YCR081W	998
YOL051W	YDL005C	999
YOL051W	YDL108W	913
YOL051W	YDL140C	997
YOL051W	YDR138W	947
YOL051W	YDR145W	977
YOL051W	YDR308C	999
YOL051W	YDR311W	959
YOL051W	YDR443C	999
YOL051W	YEL009C	991
YOL051W	YER022W	999
YOL051W	YER148W	985
YOL051W	YER171W	917
YOL051W	YGL025C	999
YOL051W	YGL070C	965
YOL051W	YGL127C	999
YOL051W	YGL151W	999
YOL051W	YGL244W	970
YOL051W	YGR005C	991
YOL051W	YGR104C	999
YOL051W	YGR186W	917
YOL051W	YGR274C	920
YOL051W	YHR041C	999
YOL051W	YHR058C	999
YOL051W	YHR143W-A	959
YOL051W	YIL021W	964
YOL051W	YIL143C	984

YOL051W	YKL028W	999
YOL051W	YKL058W	959
YOL051W	YKR062W	999
YOL051W	YLR005W	959
YOL051W	YLR071C	999
YOL051W	YLR418C	999
YOL051W	YML007W	974
YOL051W	YML098W	959
YOL051W	YMR005W	959
YOL051W	YMR112C	998
YOL051W	YMR227C	959
YOL051W	YMR236W	959
YOL051W	YNL236W	999
YOL051W	YNR010W	999
YOL051W	YOL017W	982
YOL051W	YOL135C	999
YOL051W	YOL145C	970
YOL051W	YOL148C	910
YOL051W	YOR123C	964
YOL051W	YOR151C	911
YOL051W	YOR174W	999
YOL051W	YOR224C	959
YOL051W	YOR290C	958
YOL051W	YPL016W	983
YOL051W	YPL042C	999
YOL051W	YPL129W	993
YOL051W	YPL248C	999
YOL051W	YPR025C	959
YOL051W	YPR070W	999
YOL051W	YPR168W	999
YOL051W	YPR187W	959
YOL051W	YPR198W	988
YOL052C	YGR101W	908
YOL052C	YKL184W	993
YOL052C	YLR146C	966
YOL052C	YPR069C	963
YOL054W	YBR009C	997
YOL054W	YBR272C	977
YOL054W	YEL056W	994
YOL054W	YGL207W	999
YOL054W	YLL022C	994
YOL054W	YML069W	997
YOL054W	YNL030W	995
YOL054W	YNL031C	984

YOL054W	YPL001W	988
YOL055C	YPL214C	995
YOL055C	YPL258C	969
YOL058W	YGR124W	939
YOL058W	YHR018C	999
YOL058W	YJL088W	999
YOL058W	YJL130C	928
YOL058W	YJR109C	920
YOL058W	YLR027C	900
YOL058W	YLR438W	943
YOL058W	YMR043W	952
YOL058W	YMR062C	937
YOL058W	YMR250W	907
YOL058W	YNL104C	913
YOL058W	YNL220W	968
YOL058W	YOL140W	926
YOL058W	YOR108W	913
YOL058W	YOR303W	908
YOL058W	YPR145W	913
YOL059W	YDL022W	999
YOL061W	YBL068W	994
YOL061W	YBR276C	960
YOL061W	YDL188C	946
YOL061W	YER099C	999
YOL061W	YHL011C	987
YOL061W	YKL181W	999
YOL061W	YML106W	936
YOL061W	YPR074C	920
YOL062C	YBL037W	999
YOL062C	YJR005W	998
YOL062C	YJR058C	917
YOL063C	YJL047C	997
YOL063C	YNR034W	998
YOL064C	YJR010W	920
YOL064C	YPR167C	953
YOL065C	YKL212W	999
YOL065C	YNL106C	928
YOL065C	YNL325C	934
YOL065C	YOR109W	932
YOL066C	YBR153W	965
YOL066C	YOL143C	945
YOL067C	YBL103C	999
YOL067C	YCR005C	944
YOL067C	YGL252C	983

YOL067C	YNL006W	918
YOL067C	YNR001C	909
YOL067C	YOL108C	971
YOL068C	YBR103W	999
YOL068C	YCR033W	998
YOL068C	YDL042C	996
YOL068C	YDR155C	996
YOL068C	YDR310C	999
YOL068C	YGL194C	994
YOL068C	YIL112W	999
YOL068C	YJL168C	952
YOL068C	YKR029C	998
YOL068C	YMR273C	950
YOL068C	YOR279C	988
YOL069W	YAL034W-A	993
YOL069W	YDR201W	962
YOL069W	YDR318W	990
YOL069W	YEL061C	962
YOL069W	YER018C	999
YOL069W	YGL060W	990
YOL069W	YGR140W	978
YOL069W	YGR179C	944
YOL069W	YGR188C	900
YOL069W	YIL144W	999
YOL069W	YIR010W	982
YOL069W	YJL030W	928
YOL069W	YJR112W	991
YOL069W	YMR117C	999
YOL069W	YMR294W	910
YOL069W	YPL018W	940
YOL069W	YPL209C	921
YOL070C	YKR048C	999
YOL070C	YNL078W	999
YOL072W	YAR002W	981
YOL072W	YBR111W-A	999
YOL072W	YDR159W	999
YOL072W	YDR448W	935
YOL072W	YGL086W	915
YOL072W	YGR119C	924
YOL072W	YJL030W	917
YOL072W	YKL057C	967
YOL072W	YKR082W	961
YOL072W	YOL012C	957
YOL072W	YOR098C	921

YOL072W	YOR257W	999
YOL072W	YPL169C	985
YOL076W	YDL014W	940
YOL076W	YIL138C	910
YOL076W	YNL079C	998
YOL076W	YPR131C	999
YOL077C	YBR031W	943
YOL077C	YDL014W	969
YOL077C	YDL031W	987
YOL077C	YDL082W	950
YOL077C	YDR060W	999
YOL077C	YDR087C	998
YOL077C	YDR471W	943
YOL077C	YDR496C	989
YOL077C	YER006W	994
YOL077C	YFL002C	992
YOL077C	YFR001W	987
YOL077C	YGL030W	952
YOL077C	YGL111W	997
YOL077C	YGL135W	940
YOL077C	YGR103W	999
YOL077C	YHR052W	997
YOL077C	YHR088W	997
YOL077C	YHR170W	958
YOL077C	YHR197W	996
YOL077C	YIL133C	949
YOL077C	YKL009W	999
YOL077C	YKL172W	999
YOL077C	YKR081C	999
YOL077C	YLL008W	994
YOL077C	YLL034C	988
YOL077C	YLL045C	949
YOL077C	YLR002C	990
YOL077C	YLR009W	985
YOL077C	YLR029C	951
YOL077C	YLR175W	938
YOL077C	YLR196W	999
YOL077C	YLR276C	987
YOL077C	YLR449W	987
YOL077C	YMR049C	999
YOL077C	YMR229C	999
YOL077C	YMR242C	947
YOL077C	YMR290C	996
YOL077C	YNL002C	993

YOL077C	YNL061W	994
YOL077C	YNL110C	999
YOL077C	YOL041C	999
YOL077C	YOL127W	956
YOL077C	YOR063W	991
YOL077C	YOR206W	998
YOL077C	YOR272W	999
YOL077C	YOR312C	932
YOL077C	YPL012W	941
YOL077C	YPL043W	999
YOL077C	YPL093W	992
YOL077C	YPL131W	966
YOL077C	YPL198W	972
YOL077C	YPL211W	999
YOL077C	YPR016C	927
YOL078W	YER093C	999
YOL078W	YJL058C	959
YOL078W	YJR066W	904
YOL078W	YKL203C	999
YOL078W	YMR068W	998
YOL078W	YNL006W	999
YOL080C	YMR229C	943
YOL081W	YAL056W	977
YOL081W	YBR140C	978
YOL081W	YJL005W	916
YOL081W	YLR178C	986
YOL081W	YLR310C	973
YOL081W	YNL098C	999
YOL081W	YOR101W	999
YOL081W	YOR371C	976
YOL082W	YBL078C	976
YOL082W	YDR311W	952
YOL082W	YER151C	990
YOL082W	YGL156W	979
YOL082W	YKL103C	999
YOL082W	YOR115C	927
YOL082W	YOR302W	976
YOL082W	YPR049C	985
YOL083W	YCR027C	978
YOL086C	YBR196C	972
YOL086C	YCR012W	987
YOL086C	YDR050C	970
YOL086C	YDR385W	968
YOL086C	YER073W	901

YOL086C	YGL256W	979
YOL086C	YKL060C	968
YOL086C	YLR044C	997
YOL086C	YLR249W	970
YOL086C	YMR083W	994
YOL086C	YMR169C	900
YOL086C	YMR170C	900
YOL086C	YMR186W	991
YOL086C	YMR303C	998
YOL086C	YOR133W	970
YOL086C	YOR374W	901
YOL086C	YPL061W	900
YOL087C	YBL067C	998
YOL088C	YCL043C	982
YOL088C	YML130C	930
YOL090W	YAR007C	992
YOL090W	YBR088C	999
YOL090W	YCR092C	999
YOL090W	YDL102W	999
YOL090W	YDR097C	999
YOL090W	YDR295C	988
YOL090W	YER171W	969
YOL090W	YGR258C	950
YOL090W	YKL113C	929
YOL090W	YML032C	943
YOL090W	YML060W	909
YOL090W	YML095C	994
YOL090W	YMR137C	981
YOL090W	YMR167W	999
YOL090W	YMR186W	939
YOL090W	YMR190C	997
YOL090W	YNL021W	991
YOL090W	YNL082W	999
YOL090W	YNL102W	945
YOL090W	YNL250W	916
YOL090W	YNL262W	986
YOL090W	YOR033C	999
YOL090W	YOR217W	919
YOL090W	YOR323C	936
YOL090W	YPL022W	999
YOL090W	YPL164C	948
YOL090W	YPR179C	987
YOL091W	YDL239C	997
YOL091W	YGL170C	991

YOL091W	YLR227C	937
YOL091W	YNL225C	983
YOL091W	YOR177C	991
YOL091W	YOR373W	967
YOL093W	YDR120C	902
YOL094C	YBL035C	996
YOL094C	YBR087W	999
YOL094C	YBR088C	999
YOL094C	YER173W	999
YOL094C	YIR008C	909
YOL094C	YJR068W	999
YOL094C	YMR078C	999
YOL094C	YNL102W	910
YOL094C	YNL290W	999
YOL094C	YOR144C	999
YOL094C	YOR217W	999
YOL096C	YBR003W	926
YOL096C	YDR204W	997
YOL096C	YGL119W	920
YOL096C	YGL134W	910
YOL096C	YGR255C	992
YOL096C	YML110C	955
YOL096C	YNR041C	954
YOL096C	YOR125C	998
YOL096C	YPL219W	915
YOL097C	YJR078W	910
YOL098C	YBL087C	921
YOL100W	YDR490C	999
YOL100W	YGR086C	992
YOL100W	YKL126W	980
YOL100W	YMR104C	922
YOL100W	YMR109W	900
YOL100W	YPL004C	998
YOL101C	YJL056C	925
YOL101C	YLR023C	957
YOL104C	YJL019W	998
YOL104C	YPL200W	935
YOL105C	YBL105C	934
YOL105C	YEL034W	982
YOL105C	YNL283C	982
YOL105C	YOR008C	994
YOL108C	YBL103C	971
YOL108C	YDR123C	999
YOL108C	YFR034C	970

YOL108C	YHL020C	998
YOL108C	YJL153C	995
YOL108C	YKL017C	938
YOL108C	YKL135C	999
YOL108C	YMR317W	943
YOL108C	YNL279W	943
YOL108C	YOL067C	971
YOL108C	YOR344C	977
YOL109W	YLR371W	906
YOL110W	YDR410C	923
YOL110W	YLR246W	953
YOL110W	YNL098C	998
YOL111C	YNL064C	979
YOL111C	YOR007C	999
YOL111C	YOR164C	942
YOL111C	YPL240C	919
YOL112W	YFL005W	931
YOL112W	YHR052W	905
YOL112W	YLL021W	996
YOL112W	YLR229C	981
YOL112W	YLR262C	903
YOL112W	YNL293W	999
YOL113W	YHL007C	986
YOL113W	YLR229C	999
YOL113W	YNL298W	902
YOL115W	YDL175C	999
YOL115W	YDL201W	965
YOL115W	YIL079C	999
YOL115W	YJL050W	999
YOL115W	YKL113C	952
YOL115W	YKR002W	960
YOL115W	YNL251C	954
YOL115W	YNL299W	999
YOL115W	YOL006C	998
YOL115W	YOR001W	999
YOL115W	YPL190C	993
YOL116W	YER109C	989
YOL116W	YLR182W	974
YOL116W	YMR164C	998
YOL116W	YNL142W	982
YOL117W	YDL147W	986
YOL117W	YDL216C	999
YOL117W	YDR179C	999
YOL117W	YIL071C	999

YOL117W	YJR084W	995
YOL117W	YMR025W	999
YOL120C	YBL027W	933
YOL120C	YBL072C	924
YOL120C	YBL087C	923
YOL120C	YBL092W	926
YOL120C	YBR031W	999
YOL120C	YBR048W	920
YOL120C	YBR084C-A	933
YOL120C	YBR118W	911
YOL120C	YBR181C	906
YOL120C	YBR189W	904
YOL120C	YBR191W	929
YOL120C	YCR031C	909
YOL120C	YDL075W	914
YOL120C	YDL081C	931
YOL120C	YDL082W	998
YOL120C	YDL136W	923
YOL120C	YDL191W	923
YOL120C	YDR012W	998
YOL120C	YDR025W	920
YOL120C	YDR064W	949
YOL120C	YDR382W	912
YOL120C	YDR385W	916
YOL120C	YDR418W	913
YOL120C	YDR447C	921
YOL120C	YDR450W	922
YOL120C	YEL054C	913
YOL120C	YER074W	902
YOL120C	YER102W	924
YOL120C	YER117W	923
YOL120C	YER131W	908
YOL120C	YFL034C-A	903
YOL120C	YFR031C-A	998
YOL120C	YFR032C-A	927
YOL120C	YGL030W	925
YOL120C	YGL031C	917
YOL120C	YGL076C	998
YOL120C	YGL103W	903
YOL120C	YGL123W	931
YOL120C	YGL135W	910
YOL120C	YGL147C	935
YOL120C	YGL189C	908
YOL120C	YGR027C	917

YOL120C	YGR034W	925
YOL120C	YGR085C	922
YOL120C	YGR118W	924
YOL120C	YGR148C	917
YOL120C	YGR214W	927
YOL120C	YHL001W	922
YOL120C	YHL015W	927
YOL120C	YHL033C	917
YOL120C	YHR010W	914
YOL120C	YHR021C	922
YOL120C	YHR141C	923
YOL120C	YHR203C	916
YOL120C	YIL018W	998
YOL120C	YIL069C	902
YOL120C	YIL133C	998
YOL120C	YIL148W	905
YOL120C	YJL136C	922
YOL120C	YJL177W	914
YOL120C	YJL189W	905
YOL120C	YJL190C	921
YOL120C	YJL191W	909
YOL120C	YJR094W-A	923
YOL120C	YJR123W	949
YOL120C	YJR145C	998
YOL120C	YKL006W	922
YOL120C	YKL156W	922
YOL120C	YKL180W	914
YOL120C	YKR057W	922
YOL120C	YKR094C	905
YOL120C	YLL045C	996
YOL120C	YLR029C	998
YOL120C	YLR048W	927
YOL120C	YLR061W	904
YOL120C	YLR075W	935
YOL120C	YLR167W	900
YOL120C	YLR264W	908
YOL120C	YLR325C	904
YOL120C	YLR333C	916
YOL120C	YLR340W	957
YOL120C	YLR344W	925
YOL120C	YLR367W	921
YOL120C	YLR406C	914
YOL120C	YLR441C	924
YOL120C	YLR448W	928

YOL120C	YML024W	922
YOL120C	YML026C	922
YOL120C	YML063W	998
YOL120C	YML073C	998
YOL120C	YMR121C	927
YOL120C	YMR142C	938
YOL120C	YMR194W	921
YOL120C	YMR230W	920
YOL120C	YMR242C	944
YOL120C	YMR260C	908
YOL120C	YMR290C	978
YOL120C	YNL067W	936
YOL120C	YNL069C	998
YOL120C	YNL096C	916
YOL120C	YNL162W	923
YOL120C	YNL178W	961
YOL120C	YNL301C	997
YOL120C	YNL302C	920
YOL120C	YOL039W	912
YOL120C	YOL040C	944
YOL120C	YOL121C	921
YOL120C	YOL127W	996
YOL120C	YOR063W	996
YOL120C	YOR096W	916
YOL120C	YOR133W	929
YOL120C	YOR167C	908
YOL120C	YOR206W	976
YOL120C	YOR234C	927
YOL120C	YOR293W	921
YOL120C	YOR312C	935
YOL120C	YOR369C	920
YOL120C	YPL079W	929
YOL120C	YPL081W	904
YOL120C	YPL090C	906
YOL120C	YPL131W	950
YOL120C	YPL143W	997
YOL120C	YPL198W	998
YOL120C	YPL220W	910
YOL120C	YPL249C-A	935
YOL120C	YPR043W	923
YOL120C	YPR080W	911
YOL120C	YPR102C	922
YOL120C	YPR132W	924
YOL121C	YBL027W	939

YOL121C	YBL071W-A	915
YOL121C	YBL072C	923
YOL121C	YBL087C	962
YOL121C	YBL092W	958
YOL121C	YBR031W	930
YOL121C	YBR048W	935
YOL121C	YBR079C	943
YOL121C	YBR084C-A	939
YOL121C	YBR118W	908
YOL121C	YBR181C	956
YOL121C	YBR189W	931
YOL121C	YBR191W	919
YOL121C	YCR031C	927
YOL121C	YDL061C	912
YOL121C	YDL075W	917
YOL121C	YDL081C	944
YOL121C	YDL082W	949
YOL121C	YDL130W	904
YOL121C	YDL136W	925
YOL121C	YDL191W	925
YOL121C	YDR012W	945
YOL121C	YDR025W	915
YOL121C	YDR064W	967
YOL121C	YDR382W	927
YOL121C	YDR385W	907
YOL121C	YDR418W	929
YOL121C	YDR447C	956
YOL121C	YDR450W	914
YOL121C	YDR471W	907
YOL121C	YDR500C	911
YOL121C	YEL054C	929
YOL121C	YER056C-A	905
YOL121C	YER074W	932
YOL121C	YER102W	923
YOL121C	YER117W	923
YOL121C	YER131W	933
YOL121C	YER165W	901
YOL121C	YFL034C-A	915
YOL121C	YFR031C-A	951
YOL121C	YFR032C-A	934
YOL121C	YGL030W	937
YOL121C	YGL031C	928
YOL121C	YGL076C	948
YOL121C	YGL103W	906

YOL121C	YGL123W	914
YOL121C	YGL135W	923
YOL121C	YGL147C	958
YOL121C	YGL189C	921
YOL121C	YGR027C	931
YOL121C	YGR034W	949
YOL121C	YGR085C	928
YOL121C	YGR118W	933
YOL121C	YGR148C	918
YOL121C	YGR214W	927
YOL121C	YHL001W	925
YOL121C	YHL015W	940
YOL121C	YHL033C	920
YOL121C	YHR010W	928
YOL121C	YHR021C	934
YOL121C	YHR141C	949
YOL121C	YHR203C	922
YOL121C	YIL018W	911
YOL121C	YIL052C	905
YOL121C	YIL069C	932
YOL121C	YIL133C	960
YOL121C	YIL148W	902
YOL121C	YJL136C	926
YOL121C	YJL177W	914
YOL121C	YJL189W	912
YOL121C	YJL190C	965
YOL121C	YJL191W	927
YOL121C	YJR007W	904
YOL121C	YJR094W-A	931
YOL121C	YJR123W	940
YOL121C	YJR145C	922
YOL121C	YKL006W	925
YOL121C	YKL156W	927
YOL121C	YKL180W	914
YOL121C	YKR057W	926
YOL121C	YKR094C	902
YOL121C	YLL045C	959
YOL121C	YLR029C	919
YOL121C	YLR048W	936
YOL121C	YLR061W	920
YOL121C	YLR075W	918
YOL121C	YLR185W	912
YOL121C	YLR264W	911
YOL121C	YLR287C-A	903

YOL121C	YLR325C	961
YOL121C	YLR333C	931
YOL121C	YLR340W	954
YOL121C	YLR344W	920
YOL121C	YLR367W	915
YOL121C	YLR388W	912
YOL121C	YLR406C	917
YOL121C	YLR441C	955
YOL121C	YLR448W	925
YOL121C	YML024W	928
YOL121C	YML026C	914
YOL121C	YML063W	943
YOL121C	YML073C	934
YOL121C	YMR121C	932
YOL121C	YMR142C	936
YOL121C	YMR194W	926
YOL121C	YMR230W	927
YOL121C	YMR242C	973
YOL121C	YMR260C	911
YOL121C	YNL067W	928
YOL121C	YNL069C	936
YOL121C	YNL096C	938
YOL121C	YNL162W	927
YOL121C	YNL178W	963
YOL121C	YNL301C	921
YOL121C	YNL302C	903
YOL121C	YOL039W	912
YOL121C	YOL040C	956
YOL121C	YOL120C	921
YOL121C	YOL127W	920
YOL121C	YOL139C	932
YOL121C	YOR063W	941
YOL121C	YOR096W	968
YOL121C	YOR133W	907
YOL121C	YOR167C	923
YOL121C	YOR182C	903
YOL121C	YOR234C	933
YOL121C	YOR293W	927
YOL121C	YOR312C	927
YOL121C	YOR361C	937
YOL121C	YOR369C	972
YOL121C	YPL079W	931
YOL121C	YPL081W	927
YOL121C	YPL090C	907

YOL121C	YPL131W	945
YOL121C	YPL143W	933
YOL121C	YPL198W	928
YOL121C	YPL220W	923
YOL121C	YPL249C-A	927
YOL121C	YPR043W	931
YOL121C	YPR080W	921
YOL121C	YPR102C	933
YOL121C	YPR132W	933
YOL122C	YBL101C	922
YOL122C	YBR290W	957
YOL122C	YDR182W	990
YOL122C	YGL167C	909
YOL122C	YHR050W	978
YOL122C	YLL039C	956
YOL122C	YLR163C	976
YOL123W	YBR017C	999
YOL123W	YBR034C	999
YOL123W	YDR432W	999
YOL123W	YGL122C	998
YOL123W	YGR218W	982
YOL123W	YKL068W	913
YOL123W	YMR061W	976
YOL123W	YMR064W	919
YOL123W	YMR080C	982
YOL124C	YDR120C	984
YOL124C	YNR046W	999
YOL126C	YBR218C	948
YOL126C	YCR005C	927
YOL126C	YDL078C	973
YOL126C	YGL062W	948
YOL126C	YKL029C	901
YOL126C	YKL085W	995
YOL126C	YKR097W	951
YOL126C	YLR027C	904
YOL126C	YLR377C	955
YOL126C	YNL117W	948
YOL126C	YNR001C	925
YOL126C	YPL262W	939
YOL127W	YAL035W	918
YOL127W	YBL027W	999
YOL127W	YBL072C	994
YOL127W	YBL087C	998
YOL127W	YBL092W	999

YOL127W	YBR031W	999
YOL127W	YBR048W	998
YOL127W	YBR084C-A	994
YOL127W	YBR118W	904
YOL127W	YBR181C	996
YOL127W	YBR189W	999
YOL127W	YBR191W	999
YOL127W	YCR031C	999
YOL127W	YDL031W	944
YOL127W	YDL061C	996
YOL127W	YDL075W	999
YOL127W	YDL081C	909
YOL127W	YDL082W	996
YOL127W	YDL083C	961
YOL127W	YDL130W	941
YOL127W	YDL136W	999
YOL127W	YDL191W	925
YOL127W	YDR012W	999
YOL127W	YDR025W	977
YOL127W	YDR064W	999
YOL127W	YDR382W	999
YOL127W	YDR385W	907
YOL127W	YDR418W	999
YOL127W	YDR447C	995
YOL127W	YDR450W	996
YOL127W	YDR471W	999
YOL127W	YDR496C	946
YOL127W	YDR500C	978
YOL127W	YEL054C	933
YOL127W	YER006W	954
YOL127W	YER056C-A	997
YOL127W	YER074W	997
YOL127W	YER102W	998
YOL127W	YER117W	996
YOL127W	YER131W	973
YOL127W	YFL002C	941
YOL127W	YFL034C-A	921
YOL127W	YFR031C-A	999
YOL127W	YFR032C-A	997
YOL127W	YGL014W	966
YOL127W	YGL030W	999
YOL127W	YGL031C	999
YOL127W	YGL076C	999
YOL127W	YGL103W	999

YOL127W	YGL111W	933
YOL127W	YGL123W	998
YOL127W	YGL135W	999
YOL127W	YGL147C	999
YOL127W	YGL189C	926
YOL127W	YGR027C	999
YOL127W	YGR034W	999
YOL127W	YGR085C	999
YOL127W	YGR103W	999
YOL127W	YGR118W	999
YOL127W	YGR148C	997
YOL127W	YGR214W	999
YOL127W	YHL001W	999
YOL127W	YHL015W	999
YOL127W	YHL033C	999
YOL127W	YHR010W	998
YOL127W	YHR021C	924
YOL127W	YHR052W	945
YOL127W	YHR088W	933
YOL127W	YHR141C	999
YOL127W	YHR197W	963
YOL127W	YHR203C	999
YOL127W	YIL018W	999
YOL127W	YIL052C	999
YOL127W	YIL069C	995
YOL127W	YIL133C	999
YOL127W	YIL148W	978
YOL127W	YJL136C	973
YOL127W	YJL177W	999
YOL127W	YJL189W	953
YOL127W	YJL190C	998
YOL127W	YJL191W	981
YOL127W	YJR094W-A	999
YOL127W	YJR123W	999
YOL127W	YJR145C	998
YOL127W	YKL006W	999
YOL127W	YKL156W	995
YOL127W	YKL172W	929
YOL127W	YKL180W	999
YOL127W	YKR057W	925
YOL127W	YKR081C	950
YOL127W	YLL008W	935
YOL127W	YLL045C	996
YOL127W	YLR002C	933

YOL127W	YLR029C	999
YOL127W	YLR048W	999
YOL127W	YLR061W	999
YOL127W	YLR074C	978
YOL127W	YLR075W	999
YOL127W	YLR150W	999
YOL127W	YLR167W	974
YOL127W	YLR185W	996
YOL127W	YLR264W	994
YOL127W	YLR276C	936
YOL127W	YLR287C-A	996
YOL127W	YLR325C	998
YOL127W	YLR333C	935
YOL127W	YLR340W	999
YOL127W	YLR344W	999
YOL127W	YLR367W	995
YOL127W	YLR388W	999
YOL127W	YLR406C	997
YOL127W	YLR441C	997
YOL127W	YLR448W	999
YOL127W	YLR449W	927
YOL127W	YML024W	999
YOL127W	YML026C	909
YOL127W	YML063W	999
YOL127W	YML073C	997
YOL127W	YMR049C	934
YOL127W	YMR121C	949
YOL127W	YMR142C	997
YOL127W	YMR194W	999
YOL127W	YMR230W	965
YOL127W	YMR242C	997
YOL127W	YMR260C	908
YOL127W	YMR290C	949
YOL127W	YMR308C	908
YOL127W	YNL002C	938
YOL127W	YNL061W	937
YOL127W	YNL067W	999
YOL127W	YNL069C	997
YOL127W	YNL096C	997
YOL127W	YNL110C	977
YOL127W	YNL112W	976
YOL127W	YNL162W	921
YOL127W	YNL178W	999
YOL127W	YNL301C	999

YOL127W	YNL302C	920
YOL127W	YNR037C	952
YOL127W	YNR053C	927
YOL127W	YOL039W	997
YOL127W	YOL040C	999
YOL127W	YOL041C	929
YOL127W	YOL077C	956
YOL127W	YOL120C	996
YOL127W	YOL121C	920
YOL127W	YOR063W	999
YOL127W	YOR096W	999
YOL127W	YOR133W	926
YOL127W	YOR167C	963
YOL127W	YOR182C	908
YOL127W	YOR206W	936
YOL127W	YOR234C	996
YOL127W	YOR293W	996
YOL127W	YOR312C	997
YOL127W	YOR369C	949
YOL127W	YPL037C	996
YOL127W	YPL043W	941
YOL127W	YPL079W	977
YOL127W	YPL081W	962
YOL127W	YPL090C	996
YOL127W	YPL093W	964
YOL127W	YPL131W	999
YOL127W	YPL143W	999
YOL127W	YPL198W	996
YOL127W	YPL211W	940
YOL127W	YPL220W	999
YOL127W	YPL249C-A	997
YOL127W	YPR016C	933
YOL127W	YPR043W	967
YOL127W	YPR067W	972
YOL127W	YPR080W	906
YOL127W	YPR102C	999
YOL127W	YPR132W	979
YOL128C	YKL127W	927
YOL128C	YMR037C	929
YOL129W	YDR105C	927
YOL129W	YJR044C	997
YOL130W	YFL050C	977
YOL130W	YLR291C	900
YOL130W	YOR334W	913

YOL133W	YDL030W	962
YOL133W	YDL132W	999
YOL133W	YDR054C	999
YOL133W	YDR328C	999
YOL133W	YFL009W	999
YOL133W	YGR003W	998
YOL133W	YIL001W	931
YOL133W	YIL046W	991
YOL133W	YJL047C	997
YOL133W	YJL187C	940
YOL133W	YJR090C	999
YOL133W	YLR079W	999
YOL133W	YLR128W	987
YOL133W	YMR199W	918
YOL133W	YOR080W	996
YOL133W	YPL256C	997
YOL135C	YBL093C	999
YOL135C	YBR154C	913
YOL135C	YBR193C	999
YOL135C	YBR253W	999
YOL135C	YCR081W	999
YOL135C	YDL005C	999
YOL135C	YDL140C	988
YOL135C	YDR167W	992
YOL135C	YDR308C	999
YOL135C	YDR404C	912
YOL135C	YDR443C	999
YOL135C	YDR448W	997
YOL135C	YER022W	999
YOL135C	YER148W	903
YOL135C	YGL025C	999
YOL135C	YGL112C	975
YOL135C	YGL127C	999
YOL135C	YGL151W	999
YOL135C	YGR104C	999
YOL135C	YHR041C	999
YOL135C	YHR058C	999
YOL135C	YIL021W	978
YOL135C	YIL077C	917
YOL135C	YIL143C	907
YOL135C	YKL058W	902
YOL135C	YLR071C	999
YOL135C	YML007W	974
YOL135C	YML015C	943

YOL135C	YMR112C	999
YOL135C	YNL236W	999
YOL135C	YNR010W	999
YOL135C	YOL005C	909
YOL135C	YOL051W	999
YOL135C	YOR151C	930
YOL135C	YOR174W	999
YOL135C	YOR210W	910
YOL135C	YPL042C	992
YOL135C	YPL129W	991
YOL135C	YPL240C	990
YOL135C	YPR070W	999
YOL135C	YPR168W	999
YOL135C	YPR187W	907
YOL136C	YDR477W	976
YOL136C	YGR240C	976
YOL136C	YJR090C	907
YOL136C	YLR377C	971
YOL136C	YMR205C	957
YOL139C	YBL032W	962
YOL139C	YBL072C	916
YOL139C	YBR079C	918
YOL139C	YBR181C	925
YOL139C	YBR189W	915
YOL139C	YCL011C	977
YOL139C	YCL037C	980
YOL139C	YDL087C	901
YOL139C	YDR447C	916
YOL139C	YER025W	900
YOL139C	YER102W	997
YOL139C	YER165W	999
YOL139C	YGL049C	999
YOL139C	YGL173C	998
YOL139C	YGR162W	999
YOL139C	YHL015W	920
YOL139C	YHL034C	998
YOL139C	YIL133C	925
YOL139C	YJL138C	998
YOL139C	YJL190C	997
YOL139C	YJR007W	935
YOL139C	YJR145C	916
YOL139C	YKL204W	999
YOL139C	YKR059W	995
YOL139C	YLR048W	913

YOL139C	YLR367W	997
YOL139C	YLR441C	923
YOL139C	YML056C	976
YOL139C	YMR125W	998
YOL139C	YMR230W	930
YOL139C	YMR260C	983
YOL139C	YNL096C	927
YOL139C	YOL040C	902
YOL139C	YOL121C	932
YOL139C	YOR204W	995
YOL139C	YOR276W	999
YOL139C	YOR361C	936
YOL139C	YOR369C	920
YOL139C	YPL090C	905
YOL139C	YPL237W	907
YOL139C	YPR041W	911
YOL139C	YPR163C	980
YOL140W	YER069W	954
YOL140W	YJL088W	997
YOL140W	YLR438W	913
YOL140W	YMR062C	985
YOL140W	YOL058W	926
YOL140W	YOR130C	942
YOL142W	YCR035C	999
YOL142W	YDL111C	999
YOL142W	YDR280W	999
YOL142W	YGR095C	999
YOL142W	YGR158C	999
YOL142W	YGR195W	999
YOL142W	YHR069C	999
YOL142W	YHR081W	999
YOL142W	YNL189W	983
YOL142W	YNL232W	999
YOL142W	YNL265C	970
YOL142W	YNR024W	998
YOL142W	YOL021C	999
YOL142W	YOR001W	999
YOL142W	YOR076C	999
YOL143C	YBL033C	906
YOL143C	YBR256C	996
YOL143C	YDR487C	998
YOL143C	YOL066C	945
YOL144W	YJR041C	999
YOL144W	YKL014C	999

YOL144W	YLR221C	998
YOL144W	YML121W	951
YOL144W	YNR038W	998
YOL144W	YPL211W	995
YOL145C	YBR253W	970
YOL145C	YBR279W	999
YOL145C	YDL108W	925
YOL145C	YDL140C	996
YOL145C	YDR167W	965
YOL145C	YDR308C	970
YOL145C	YER022W	970
YOL145C	YER148W	958
YOL145C	YFL029C	966
YOL145C	YGL019W	994
YOL145C	YGL207W	999
YOL145C	YGL244W	999
YOL145C	YGR104C	970
YOL145C	YGR186W	930
YOL145C	YHR041C	970
YOL145C	YIL035C	999
YOL145C	YJL140W	912
YOL145C	YJL168C	968
YOL145C	YKL139W	978
YOL145C	YLR418C	999
YOL145C	YML010W	965
YOL145C	YML069W	999
YOL145C	YNL236W	908
YOL145C	YOL051W	970
YOL145C	YOR039W	999
YOL145C	YOR061W	999
YOL145C	YOR123C	999
YOL145C	YPL129W	909
YOL145C	YPR070W	903
YOL146W	YDR013W	999
YOL146W	YDR489W	999
YOL146W	YGL113W	965
YOL146W	YJL072C	999
YOL146W	YJL090C	957
YOL146W	YPR175W	926
YOL147C	YDL065C	999
YOL147C	YDR244W	951
YOL147C	YGL153W	921
YOL147C	YOR193W	991
YOL147C	YPL112C	995

YOL148C	YBR081C	999
YOL148C	YBR111W-A	959
YOL148C	YBR198C	999
YOL148C	YCL010C	999
YOL148C	YDR145W	999
YOL148C	YDR167W	999
YOL148C	YDR176W	999
YOL148C	YDR392W	999
YOL148C	YDR448W	999
YOL148C	YER148W	999
YOL148C	YER164W	995
YOL148C	YGL066W	999
YOL148C	YGL112C	999
YOL148C	YGR252W	999
YOL148C	YGR274C	931
YOL148C	YHR041C	974
YOL148C	YHR079C	994
YOL148C	YHR099W	999
YOL148C	YLR055C	999
YOL148C	YML051W	900
YOL148C	YMR223W	999
YOL148C	YMR236W	999
YOL148C	YOL051W	910
YOL148C	YOR290C	948
YOL148C	YPL047W	999
YOL148C	YPL254W	999
YOL149W	YBL026W	998
YOL149W	YBR094W	993
YOL149W	YCR077C	997
YOL149W	YDL160C	999
YOL149W	YDR172W	971
YOL149W	YDR206W	976
YOL149W	YDR378C	971
YOL149W	YEL015W	999
YOL149W	YER035W	928
YOL149W	YER112W	997
YOL149W	YER146W	971
YOL149W	YER165W	988
YOL149W	YGL173C	994
YOL149W	YGL222C	929
YOL149W	YGR072W	937
YOL149W	YJL124C	991
YOL149W	YJR022W	989
YOL149W	YLR264W	939

YOL149W	YLR438C-A	971
YOL149W	YMR080C	990
YOL149W	YMR198W	960
YOL149W	YNL118C	999
YOL149W	YNL147W	984
YOL149W	YNR052C	980
YOL149W	YOR167C	983
YOL152W	YGL071W	945
YOL152W	YKL220C	903
YOL152W	YLR214W	906
YOL152W	YMR021C	987
YOL155C	YIL169C	991
YOL155C	YIR019C	987
YOL155C	YMR317W	924
YOL156W	YBL005W	963
YOL156W	YGL013C	959
YOL156W	YPL240C	919
YOR001W	YCR035C	999
YOR001W	YDL084W	998
YOR001W	YDL111C	999
YOR001W	YDL140C	988
YOR001W	YDL201W	969
YOR001W	YDR138W	995
YOR001W	YDR280W	999
YOR001W	YER011W	923
YOR001W	YER165W	972
YOR001W	YGL044C	937
YOR001W	YGR095C	999
YOR001W	YGR158C	999
YOR001W	YGR195W	999
YOR001W	YHR069C	999
YOR001W	YHR081W	999
YOR001W	YJL050W	999
YOR001W	YKR002W	998
YOR001W	YLR347C	995
YOR001W	YML062C	926
YOR001W	YMR061W	937
YOR001W	YMR131C	934
YOR001W	YMR239C	940
YOR001W	YNL189W	999
YOR001W	YNL232W	999
YOR001W	YNL299W	976
YOR001W	YNR024W	999
YOR001W	YOL021C	999

YOR001W	YOL115W	999
YOR001W	YOL142W	999
YOR001W	YOR076C	998
YOR001W	YOR326W	964
YOR001W	YPR110C	941
YOR002W	YDL095W	948
YOR002W	YEL002C	961
YOR002W	YLR178C	960
YOR002W	YNL219C	967
YOR002W	YOR067C	982
YOR002W	YOR085W	941
YOR002W	YPL227C	984
YOR004W	YBR247C	919
YOR004W	YCL059C	941
YOR004W	YDL148C	912
YOR004W	YDL153C	922
YOR004W	YDR398W	920
YOR004W	YDR449C	910
YOR004W	YGL120C	920
YOR004W	YGL171W	947
YOR004W	YGR090W	923
YOR004W	YGR128C	930
YOR004W	YGR145W	910
YOR004W	YHR148W	924
YOR004W	YHR169W	965
YOR004W	YHR196W	915
YOR004W	YJL069C	917
YOR004W	YJL109C	917
YOR004W	YJR002W	991
YOR004W	YKL099C	909
YOR004W	YKR060W	918
YOR004W	YLL011W	996
YOR004W	YLR003C	930
YOR004W	YLR129W	947
YOR004W	YLR175W	953
YOR004W	YLR186W	923
YOR004W	YLR197W	926
YOR004W	YLR222C	963
YOR004W	YLR409C	926
YOR004W	YML093W	938
YOR004W	YMR093W	939
YOR004W	YMR229C	953
YOR004W	YMR290C	948
YOR004W	YNL075W	965

YOR004W	YNL132W	913
YOR004W	YOL010W	919
YOR004W	YOR078W	922
YOR004W	YOR145C	967
YOR004W	YPL012W	924
YOR004W	YPL126W	917
YOR004W	YPL217C	911
YOR004W	YPL266W	914
YOR004W	YPR112C	979
YOR004W	YPR137W	915
YOR004W	YPR144C	915
YOR005C	YCR014C	912
YOR005C	YDR076W	910
YOR005C	YGL090W	999
YOR005C	YGL163C	941
YOR005C	YLR265C	992
YOR005C	YML032C	991
YOR005C	YMR106C	983
YOR005C	YMR224C	975
YOR007C	YBR137W	999
YOR007C	YMR186W	995
YOR007C	YNL064C	979
YOR007C	YOL111C	999
YOR007C	YOR164C	949
YOR008C	YAL023C	982
YOR008C	YBL105C	999
YOR008C	YBR260C	998
YOR008C	YDR356W	969
YOR008C	YEL034W	978
YOR008C	YHR030C	959
YOR008C	YIL002C	936
YOR008C	YIL118W	995
YOR008C	YJL095W	945
YOR008C	YKR055W	982
YOR008C	YLR332W	999
YOR008C	YLR342W	930
YOR008C	YLR371W	997
YOR008C	YMR307W	928
YOR008C	YNL283C	994
YOR008C	YOL105C	994
YOR008C	YPR165W	988
YOR011W	YIL013C	984
YOR014W	YAL016W	999
YOR014W	YBL093C	926

YOR014W	YDL134C	999
YOR014W	YDL188C	999
YOR014W	YDR435C	914
YOR014W	YGL190C	989
YOR014W	YPL152W	987
YOR016C	YGL200C	988
YOR016C	YOL013C	923
YOR016C	YOR044W	923
YOR016C	YOR049C	923
YOR017W	Q0105	931
YOR017W	YER153C	911
YOR017W	YPL029W	932
YOR018W	YER125W	939
YOR018W	YGL115W	983
YOR018W	YGL158W	929
YOR020C	YLR259C	999
YOR023C	YCR082W	970
YOR023C	YDR176W	966
YOR023C	YDR448W	950
YOR023C	YGR252W	965
YOR025W	YDR191W	976
YOR025W	YJR043C	929
YOR026W	YAL011W	902
YOR026W	YCL029C	928
YOR026W	YDL028C	978
YOR026W	YDR334W	903
YOR026W	YDR485C	902
YOR026W	YEL061C	939
YOR026W	YER016W	988
YOR026W	YGL086W	999
YOR026W	YGL116W	999
YOR026W	YGR179C	919
YOR026W	YGR188C	999
YOR026W	YJL013C	999
YOR026W	YJL030W	999
YOR026W	YJR053W	949
YOR026W	YLR085C	911
YOR026W	YML041C	903
YOR026W	YML085C	988
YOR026W	YMR055C	965
YOR027W	YAL005C	999
YOR027W	YBL075C	964
YOR027W	YBR101C	929
YOR027W	YBR112C	928

YOR027W	YDL229W	936
YOR027W	YDR168W	999
YOR027W	YDR214W	951
YOR027W	YER103W	977
YOR027W	YGL073W	960
YOR027W	YIL143C	910
YOR027W	YJR032W	998
YOR027W	YKL117W	996
YOR027W	YLL024C	999
YOR027W	YLL026W	998
YOR027W	YLR216C	989
YOR027W	YLR409C	940
YOR027W	YMR186W	999
YOR027W	YNL007C	983
YOR027W	YNL064C	998
YOR027W	YPL106C	980
YOR027W	YPL240C	999
YOR028C	YGR229C	971
YOR030W	YGR229C	908
YOR030W	YHL027W	933
YOR031W	YHR053C	993
YOR032C	YGR121C	908
YOR032C	YMR153W	998
YOR032C	YNL142W	906
YOR033C	YBR136W	930
YOR033C	YCR092C	942
YOR033C	YDL102W	988
YOR033C	YDL220C	996
YOR033C	YDR097C	955
YOR033C	YDR217C	976
YOR033C	YDR369C	998
YOR033C	YHR164C	987
YOR033C	YKL113C	998
YOR033C	YKR056W	987
YOR033C	YLR032W	979
YOR033C	YMR106C	982
YOR033C	YMR137C	983
YOR033C	YMR167W	999
YOR033C	YMR190C	997
YOR033C	YMR224C	999
YOR033C	YMR284W	979
YOR033C	YNL082W	992
YOR033C	YNL250W	999
YOR033C	YNL262W	982

YOR033C	YOL090W	999
YOR033C	YPL022W	943
YOR033C	YPL167C	984
YOR034C	YHR105W	957
YOR034C	YOR171C	955
YOR035C	YAL029C	998
YOR035C	YBR130C	907
YOR035C	YDL029W	965
YOR035C	YFL039C	941
YOR035C	YKL129C	999
YOR035C	YKL130C	906
YOR035C	YMR109W	988
YOR035C	YOR326W	999
YOR036W	YAL014C	981
YOR036W	YAL030W	998
YOR036W	YDR264C	913
YOR036W	YDR323C	992
YOR036W	YDR468C	998
YOR036W	YGL095C	998
YOR036W	YGL206C	968
YOR036W	YGL212W	999
YOR036W	YHL031C	998
YOR036W	YKL006C-A	998
YOR036W	YKL196C	999
YOR036W	YKR001C	903
YOR036W	YLR093C	952
YOR036W	YLR208W	938
YOR036W	YLR396C	935
YOR036W	YMR197C	999
YOR036W	YOR106W	999
YOR036W	YPL151C	963
YOR036W	YPL240C	945
YOR037W	YKL087C	920
YOR038C	YBL008W	999
YOR038C	YBR010W	909
YOR038C	YBR195C	922
YOR038C	YBR215W	999
YOR038C	YBR289W	923
YOR038C	YDR224C	933
YOR038C	YDR225W	932
YOR038C	YGR270W	927
YOR038C	YJL115W	998
YOR038C	YJR140C	999
YOR038C	YLR418C	906

YOR038C	YML102W	907
YOR039W	YBR279W	972
YOR039W	YCL031C	991
YOR039W	YCL059C	905
YOR039W	YDR299W	922
YOR039W	YER103W	902
YOR039W	YER148W	949
YOR039W	YER164W	999
YOR039W	YGL019W	999
YOR039W	YGL207W	999
YOR039W	YGL244W	968
YOR039W	YGR090W	999
YOR039W	YIL035C	999
YOR039W	YJL069C	933
YOR039W	YKL088W	999
YOR039W	YKL160W	939
YOR039W	YLR079W	909
YOR039W	YLR223C	978
YOR039W	YLR418C	991
YOR039W	YML069W	999
YOR039W	YMR128W	907
YOR039W	YMR172W	999
YOR039W	YOL145C	999
YOR039W	YOR061W	999
YOR040W	YML004C	989
YOR042W	YFR024C-A	997
YOR042W	YGR261C	919
YOR042W	YHR016C	976
YOR042W	YPR119W	962
YOR043W	YHL011C	968
YOR043W	YLL010C	999
YOR043W	YMR037C	954
YOR044W	YOL013C	923
YOR044W	YOR016C	923
YOR045W	YGR082W	958
YOR045W	YMR203W	999
YOR045W	YNL070W	992
YOR045W	YNL121C	945
YOR045W	YNL131W	993
YOR045W	YPR133W-A	998
YOR046C	YDL207W	999
YOR046C	YDR159W	952
YOR046C	YDR172W	906
YOR046C	YDR192C	998

YOR046C	YDR315C	985
YOR046C	YER159C	960
YOR046C	YER165W	920
YOR046C	YGR218W	998
YOR046C	YIL115C	999
YOR046C	YMR255W	999
YOR046C	YMR273C	969
YOR046C	YPL169C	985
YOR047C	YDL138W	952
YOR047C	YDL194W	953
YOR047C	YDR277C	976
YOR047C	YDR477W	999
YOR047C	YER148W	996
YOR047C	YGL115W	999
YOR047C	YKL038W	999
YOR047C	YOR358W	972
YOR048C	YBR069C	960
YOR048C	YDL047W	946
YOR048C	YDR289C	997
YOR048C	YDR432W	963
YOR048C	YGL173C	996
YOR048C	YGL246C	999
YOR048C	YGR218W	938
YOR048C	YHR020W	948
YOR048C	YJL098W	966
YOR048C	YLR398C	969
YOR048C	YNL064C	938
YOR048C	YPL233W	923
YOR049C	YOR016C	923
YOR049C	YOR153W	976
YOR054C	YDL047W	981
YOR054C	YKL088W	999
YOR054C	YKR072C	992
YOR054C	YML016C	947
YOR056C	YBR173C	906
YOR056C	YBR247C	999
YOR056C	YDL060W	999
YOR056C	YFR052W	982
YOR056C	YKL145W	908
YOR056C	YLR367W	976
YOR056C	YNL207W	999
YOR056C	YPR103W	998
YOR057W	YDR328C	999
YOR057W	YLR345W	913

YOR057W	YMR094W	999
YOR057W	YMR186W	996
YOR057W	YPL240C	999
YOR058C	YBL063W	984
YOR058C	YCL029C	974
YOR058C	YDR113C	918
YOR058C	YEL061C	999
YOR058C	YER016W	971
YOR058C	YMR072W	954
YOR058C	YNL271C	968
YOR058C	YPR119W	987
YOR059C	YJL069C	928
YOR061W	YBR279W	985
YOR061W	YCL031C	989
YOR061W	YCL059C	910
YOR061W	YDR043C	941
YOR061W	YDR168W	933
YOR061W	YDR299W	914
YOR061W	YER164W	999
YOR061W	YGL019W	999
YOR061W	YGL207W	999
YOR061W	YGL244W	968
YOR061W	YGR090W	999
YOR061W	YGR116W	934
YOR061W	YHR131C	980
YOR061W	YIL035C	999
YOR061W	YJL069C	929
YOR061W	YJL134W	904
YOR061W	YJR093C	976
YOR061W	YKL088W	999
YOR061W	YKL160W	941
YOR061W	YLR079W	909
YOR061W	YLR407W	995
YOR061W	YLR418C	995
YOR061W	YML069W	999
YOR061W	YMR172W	987
YOR061W	YOL145C	999
YOR061W	YOR039W	999
YOR061W	YOR119C	980
YOR061W	YOR123C	968
YOR061W	YPL240C	958
YOR063W	YAL035W	995
YOR063W	YBL027W	999
YOR063W	YBL072C	994

YOR063W	YBL087C	999
YOR063W	YBL092W	999
YOR063W	YBR031W	999
YOR063W	YBR048W	999
YOR063W	YBR084C-A	991
YOR063W	YBR084W	977
YOR063W	YBR118W	941
YOR063W	YBR142W	940
YOR063W	YBR181C	993
YOR063W	YBR189W	999
YOR063W	YBR191W	999
YOR063W	YCR031C	999
YOR063W	YDL014W	961
YOR063W	YDL031W	937
YOR063W	YDL061C	988
YOR063W	YDL075W	999
YOR063W	YDL081C	914
YOR063W	YDL082W	996
YOR063W	YDL083C	966
YOR063W	YDL130W	932
YOR063W	YDL136W	999
YOR063W	YDL191W	952
YOR063W	YDR012W	999
YOR063W	YDR025W	999
YOR063W	YDR060W	944
YOR063W	YDR064W	999
YOR063W	YDR087C	994
YOR063W	YDR101C	993
YOR063W	YDR382W	999
YOR063W	YDR385W	930
YOR063W	YDR418W	999
YOR063W	YDR447C	995
YOR063W	YDR450W	928
YOR063W	YDR471W	998
YOR063W	YDR496C	948
YOR063W	YDR500C	948
YOR063W	YEL054C	998
YOR063W	YER006W	948
YOR063W	YER056C-A	991
YOR063W	YER074W	994
YOR063W	YER102W	997
YOR063W	YER117W	999
YOR063W	YER126C	953
YOR063W	YER131W	960

YOR063W	YFL002C	942
YOR063W	YFL034C-A	901
YOR063W	YFR001W	982
YOR063W	YFR031C-A	999
YOR063W	YFR032C-A	957
YOR063W	YGL030W	999
YOR063W	YGL031C	999
YOR063W	YGL076C	999
YOR063W	YGL099W	991
YOR063W	YGL103W	999
YOR063W	YGL111W	933
YOR063W	YGL120C	942
YOR063W	YGL123W	999
YOR063W	YGL135W	999
YOR063W	YGL147C	999
YOR063W	YGL189C	916
YOR063W	YGR027C	979
YOR063W	YGR034W	999
YOR063W	YGR054W	976
YOR063W	YGR085C	999
YOR063W	YGR090W	945
YOR063W	YGR103W	999
YOR063W	YGR118W	997
YOR063W	YGR148C	983
YOR063W	YGR214W	997
YOR063W	YGR245C	935
YOR063W	YHL001W	999
YOR063W	YHL015W	997
YOR063W	YHL033C	999
YOR063W	YHR010W	998
YOR063W	YHR021C	934
YOR063W	YHR052W	979
YOR063W	YHR088W	933
YOR063W	YHR141C	999
YOR063W	YHR197W	933
YOR063W	YHR203C	999
YOR063W	YIL018W	999
YOR063W	YIL052C	996
YOR063W	YIL069C	994
YOR063W	YIL133C	999
YOR063W	YIL148W	942
YOR063W	YJL136C	951
YOR063W	YJL177W	999
YOR063W	YJL189W	935

YOR063W	YJL190C	998
YOR063W	YJL191W	998
YOR063W	YJR094W-A	997
YOR063W	YJR123W	999
YOR063W	YJR145C	999
YOR063W	YKL006W	999
YOR063W	YKL009W	942
YOR063W	YKL156W	978
YOR063W	YKL172W	931
YOR063W	YKL180W	999
YOR063W	YKR057W	923
YOR063W	YKR081C	946
YOR063W	YKR094C	904
YOR063W	YLL008W	936
YOR063W	YLL034C	939
YOR063W	YLL045C	995
YOR063W	YLR002C	935
YOR063W	YLR029C	999
YOR063W	YLR048W	998
YOR063W	YLR061W	994
YOR063W	YLR075W	999
YOR063W	YLR150W	931
YOR063W	YLR167W	948
YOR063W	YLR175W	990
YOR063W	YLR185W	994
YOR063W	YLR264W	977
YOR063W	YLR276C	961
YOR063W	YLR287C-A	993
YOR063W	YLR325C	997
YOR063W	YLR333C	925
YOR063W	YLR340W	997
YOR063W	YLR344W	999
YOR063W	YLR367W	994
YOR063W	YLR388W	998
YOR063W	YLR398C	921
YOR063W	YLR406C	997
YOR063W	YLR441C	996
YOR063W	YLR448W	999
YOR063W	YLR449W	938
YOR063W	YML024W	980
YOR063W	YML026C	928
YOR063W	YML056C	978
YOR063W	YML063W	999
YOR063W	YML073C	996

YOR063W	YMR049C	965
YOR063W	YMR121C	961
YOR063W	YMR131C	999
YOR063W	YMR142C	996
YOR063W	YMR194W	998
YOR063W	YMR230W	944
YOR063W	YMR242C	994
YOR063W	YMR260C	925
YOR063W	YMR290C	952
YOR063W	YNL002C	940
YOR063W	YNL061W	961
YOR063W	YNL067W	997
YOR063W	YNL069C	998
YOR063W	YNL096C	996
YOR063W	YNL110C	981
YOR063W	YNL112W	978
YOR063W	YNL132W	942
YOR063W	YNL162W	923
YOR063W	YNL178W	999
YOR063W	YNL182C	925
YOR063W	YNL209W	972
YOR063W	YNL301C	999
YOR063W	YNL302C	924
YOR063W	YNR037C	956
YOR063W	YNR038W	943
YOR063W	YNR053C	987
YOR063W	YOL039W	934
YOR063W	YOL040C	999
YOR063W	YOL041C	931
YOR063W	YOL077C	991
YOR063W	YOL120C	996
YOR063W	YOL121C	941
YOR063W	YOL127W	999
YOR063W	YOR096W	999
YOR063W	YOR133W	942
YOR063W	YOR167C	951
YOR063W	YOR206W	945
YOR063W	YOR234C	976
YOR063W	YOR272W	933
YOR063W	YOR293W	974
YOR063W	YOR312C	996
YOR063W	YOR369C	949
YOR063W	YPL012W	947
YOR063W	YPL037C	965

YOR063W	YPL043W	941
YOR063W	YPL079W	966
YOR063W	YPL081W	952
YOR063W	YPL090C	993
YOR063W	YPL093W	988
YOR063W	YPL131W	999
YOR063W	YPL143W	998
YOR063W	YPL198W	997
YOR063W	YPL211W	936
YOR063W	YPL220W	997
YOR063W	YPL249C-A	995
YOR063W	YPR016C	990
YOR063W	YPR043W	958
YOR063W	YPR080W	941
YOR063W	YPR102C	999
YOR063W	YPR132W	961
YOR063W	YPR189W	996
YOR064C	YBL052C	999
YOR064C	YPL129W	996
YOR065W	Q0105	999
YOR065W	Q0120	999
YOR065W	YBL045C	999
YOR065W	YBL099W	931
YOR065W	YDR375C	917
YOR065W	YDR529C	999
YOR065W	YEL024W	999
YOR065W	YEL039C	953
YOR065W	YER154W	981
YOR065W	YFR033C	999
YOR065W	YGR183C	999
YOR065W	YHR001W-A	999
YOR065W	YJL166W	999
YOR065W	YJR048W	999
YOR065W	YJR121W	949
YOR065W	YKL087C	967
YOR065W	YPR191W	999
YOR067C	YGL027C	938
YOR067C	YGR227W	994
YOR067C	YJL062W	921
YOR067C	YML013W	907
YOR067C	YOR002W	982
YOR069W	YDR172W	916
YOR069W	YHR012W	999
YOR069W	YJL053W	999

YOR069W	YJL154C	999
YOR069W	YOR132W	999
YOR070C	YBR164C	997
YOR070C	YER083C	925
YOR070C	YFL005W	948
YOR070C	YFL038C	999
YOR070C	YGL005C	965
YOR070C	YGL054C	909
YOR070C	YGR261C	911
YOR070C	YHL031C	994
YOR070C	YHR007C	928
YOR070C	YJL004C	988
YOR070C	YJR032W	908
YOR070C	YLR039C	905
YOR070C	YLR262C	966
YOR070C	YML001W	991
YOR070C	YML071C	991
YOR070C	YMR123W	919
YOR070C	YNL041C	991
YOR070C	YNL051W	991
YOR070C	YOR089C	943
YOR070C	YPL051W	986
YOR073W	YER016W	965
YOR073W	YGR188C	993
YOR074C	YBR084W	939
YOR074C	YBR252W	956
YOR074C	YBR263W	940
YOR074C	YDR019C	926
YOR074C	YGL125W	957
YOR074C	YGR204W	938
YOR074C	YHR144C	981
YOR074C	YJR057W	979
YOR074C	YKR080W	918
YOR074C	YLR058C	940
YOR074C	YOR236W	999
YOR075W	YDL126C	903
YOR075W	YDR189W	999
YOR075W	YDR498C	999
YOR075W	YGL098W	999
YOR075W	YGL145W	999
YOR075W	YLR268W	999
YOR075W	YLR440C	999
YOR075W	YNL258C	999
YOR076C	YCR035C	999

YOR076C	YDL111C	999
YOR076C	YDR280W	999
YOR076C	YGL213C	993
YOR076C	YGR095C	999
YOR076C	YGR158C	999
YOR076C	YGR195W	999
YOR076C	YHR069C	999
YOR076C	YHR081W	992
YOR076C	YLR398C	993
YOR076C	YMR080C	908
YOR076C	YNL189W	974
YOR076C	YNL232W	999
YOR076C	YOL021C	999
YOR076C	YOL142W	999
YOR076C	YOR001W	998
YOR076C	YPR189W	958
YOR078W	YBL004W	980
YOR078W	YBR247C	981
YOR078W	YCL059C	970
YOR078W	YCR057C	983
YOR078W	YDL014W	932
YOR078W	YDL148C	967
YOR078W	YDR299W	944
YOR078W	YDR324C	990
YOR078W	YDR449C	972
YOR078W	YER082C	991
YOR078W	YGL011C	967
YOR078W	YGL171W	952
YOR078W	YGR090W	982
YOR078W	YGR128C	990
YOR078W	YHR148W	992
YOR078W	YHR169W	915
YOR078W	YHR196W	971
YOR078W	YJL069C	975
YOR078W	YJL109C	990
YOR078W	YJR002W	999
YOR078W	YLL011W	975
YOR078W	YLR003C	973
YOR078W	YLR129W	973
YOR078W	YLR197W	999
YOR078W	YLR222C	992
YOR078W	YLR409C	971
YOR078W	YMR093W	990
YOR078W	YMR128W	981

YOR078W	YMR229C	972
YOR078W	YNL075W	972
YOR078W	YNL132W	972
YOR078W	YOR004W	922
YOR078W	YOR145C	975
YOR078W	YOR310C	985
YOR078W	YPL012W	972
YOR078W	YPL126W	990
YOR078W	YPL217C	979
YOR078W	YPL266W	970
YOR078W	YPR112C	936
YOR078W	YPR137W	999
YOR079C	YJR104C	931
YOR080W	YDL132W	999
YOR080W	YDR054C	979
YOR080W	YDR328C	998
YOR080W	YDR381W	913
YOR080W	YFL009W	975
YOR080W	YJR006W	921
YOR080W	YLR079W	944
YOR080W	YMR190C	931
YOR080W	YOL133W	996
YOR083W	YAL040C	997
YOR083W	YBR160W	996
YOR083W	YDL056W	939
YOR083W	YER111C	998
YOR083W	YER167W	980
YOR083W	YLR182W	998
YOR085W	YCL001W	907
YOR085W	YDL232W	999
YOR085W	YEL002C	999
YOR085W	YER087C-B	979
YOR085W	YGL022W	999
YOR085W	YGL027C	990
YOR085W	YGL226C-A	999
YOR085W	YGR227W	989
YOR085W	YJL002C	999
YOR085W	YLR378C	989
YOR085W	YML019W	999
YOR085W	YMR149W	999
YOR085W	YMR200W	909
YOR085W	YMR214W	981
YOR085W	YOR002W	941
YOR085W	YOR103C	999

YOR085W	YPL227C	984
YOR086C	YML072C	902
YOR089C	YAL002W	984
YOR089C	YCR009C	963
YOR089C	YDR080W	919
YOR089C	YDR323C	991
YOR089C	YDR388W	970
YOR089C	YER136W	999
YOR089C	YGL095C	957
YOR089C	YGR172C	990
YOR089C	YIL044C	915
YOR089C	YKR014C	989
YOR089C	YLL043W	915
YOR089C	YLR396C	911
YOR089C	YML097C	953
YOR089C	YMR274C	923
YOR089C	YNL041C	915
YOR089C	YNL093W	904
YOR089C	YNL293W	984
YOR089C	YNL297C	964
YOR089C	YOR070C	943
YOR089C	YOR370C	974
YOR090C	YER178W	923
YOR090C	YIL042C	912
YOR091W	YAL036C	995
YOR094W	YBL060W	990
YOR094W	YCL034W	987
YOR094W	YDR170C	966
YOR094W	YEL022W	989
YOR094W	YJR031C	936
YOR094W	YOR122C	964
YOR094W	YOR181W	906
YOR095C	YBL068W	911
YOR095C	YBR117C	934
YOR095C	YCR036W	961
YOR095C	YDR248C	922
YOR095C	YER099C	911
YOR095C	YGR256W	936
YOR095C	YHL011C	914
YOR095C	YHR183W	931
YOR095C	YJL121C	995
YOR095C	YPR074C	963
YOR096W	YAL035W	919
YOR096W	YBL004W	935

YOR096W	YBL027W	996
YOR096W	YBL071W-A	901
YOR096W	YBL072C	996
YOR096W	YBL087C	906
YOR096W	YBL092W	996
YOR096W	YBR031W	998
YOR096W	YBR048W	997
YOR096W	YBR079C	994
YOR096W	YBR084C-A	954
YOR096W	YBR118W	903
YOR096W	YBR181C	993
YOR096W	YBR189W	999
YOR096W	YBR191W	999
YOR096W	YBR247C	931
YOR096W	YCL037C	925
YOR096W	YCL059C	953
YOR096W	YCR031C	999
YOR096W	YDL014W	970
YOR096W	YDL060W	935
YOR096W	YDL061C	976
YOR096W	YDL075W	999
YOR096W	YDL081C	927
YOR096W	YDL082W	995
YOR096W	YDL083C	955
YOR096W	YDL130W	943
YOR096W	YDL136W	997
YOR096W	YDL148C	982
YOR096W	YDL191W	935
YOR096W	YDR012W	999
YOR096W	YDR025W	997
YOR096W	YDR064W	999
YOR096W	YDR091C	985
YOR096W	YDR299W	941
YOR096W	YDR382W	999
YOR096W	YDR385W	902
YOR096W	YDR418W	999
YOR096W	YDR429C	916
YOR096W	YDR447C	995
YOR096W	YDR449C	933
YOR096W	YDR450W	924
YOR096W	YDR471W	999
YOR096W	YDR500C	961
YOR096W	YEL054C	943
YOR096W	YER025W	953

YOR096W	YER056C-A	997
YOR096W	YER074W	997
YOR096W	YER082C	929
YOR096W	YER102W	997
YOR096W	YER117W	957
YOR096W	YER131W	971
YOR096W	YER165W	935
YOR096W	YFL034C-A	921
YOR096W	YFR031C-A	993
YOR096W	YFR032C-A	936
YOR096W	YGL030W	999
YOR096W	YGL031C	953
YOR096W	YGL076C	998
YOR096W	YGL103W	999
YOR096W	YGL120C	939
YOR096W	YGL123W	998
YOR096W	YGL135W	995
YOR096W	YGL147C	997
YOR096W	YGL189C	926
YOR096W	YGR027C	981
YOR096W	YGR034W	995
YOR096W	YGR085C	999
YOR096W	YGR090W	981
YOR096W	YGR118W	997
YOR096W	YGR145W	929
YOR096W	YGR148C	996
YOR096W	YGR162W	912
YOR096W	YGR214W	994
YOR096W	YHL001W	999
YOR096W	YHL015W	999
YOR096W	YHL033C	999
YOR096W	YHL034C	939
YOR096W	YHR010W	999
YOR096W	YHR021C	921
YOR096W	YHR141C	936
YOR096W	YHR148W	938
YOR096W	YHR203C	998
YOR096W	YIL018W	996
YOR096W	YIL052C	999
YOR096W	YIL069C	996
YOR096W	YIL133C	997
YOR096W	YIL148W	950
YOR096W	YJL136C	968
YOR096W	YJL177W	999

YOR096W	YJL189W	948
YOR096W	YJL190C	997
YOR096W	YJL191W	999
YOR096W	YJR002W	987
YOR096W	YJR007W	903
YOR096W	YJR094W-A	980
YOR096W	YJR123W	999
YOR096W	YJR145C	995
YOR096W	YKL006W	999
YOR096W	YKL156W	979
YOR096W	YKL180W	999
YOR096W	YKR057W	935
YOR096W	YKR059W	943
YOR096W	YKR060W	925
YOR096W	YKR081C	941
YOR096W	YKR094C	905
YOR096W	YLL045C	997
YOR096W	YLR029C	999
YOR096W	YLR048W	999
YOR096W	YLR061W	997
YOR096W	YLR075W	999
YOR096W	YLR150W	964
YOR096W	YLR167W	948
YOR096W	YLR185W	958
YOR096W	YLR186W	946
YOR096W	YLR197W	942
YOR096W	YLR264W	994
YOR096W	YLR287C-A	917
YOR096W	YLR325C	904
YOR096W	YLR333C	928
YOR096W	YLR340W	981
YOR096W	YLR344W	981
YOR096W	YLR367W	999
YOR096W	YLR388W	995
YOR096W	YLR406C	924
YOR096W	YLR441C	997
YOR096W	YLR448W	999
YOR096W	YML024W	999
YOR096W	YML026C	997
YOR096W	YML063W	999
YOR096W	YML073C	995
YOR096W	YMR093W	933
YOR096W	YMR116C	987
YOR096W	YMR121C	931

YOR096W	YMR128W	931
YOR096W	YMR142C	997
YOR096W	YMR194W	999
YOR096W	YMR230W	966
YOR096W	YMR242C	995
YOR096W	YMR260C	910
YOR096W	YMR290C	949
YOR096W	YMR309C	913
YOR096W	YNL067W	992
YOR096W	YNL069C	997
YOR096W	YNL096C	999
YOR096W	YNL132W	948
YOR096W	YNL162W	936
YOR096W	YNL178W	999
YOR096W	YNL301C	998
YOR096W	YNL302C	921
YOR096W	YOL039W	923
YOR096W	YOL040C	997
YOR096W	YOL120C	916
YOR096W	YOL121C	968
YOR096W	YOL127W	999
YOR096W	YOR063W	999
YOR096W	YOR133W	918
YOR096W	YOR167C	959
YOR096W	YOR182C	917
YOR096W	YOR234C	994
YOR096W	YOR293W	988
YOR096W	YOR310C	993
YOR096W	YOR312C	997
YOR096W	YOR361C	919
YOR096W	YOR369C	951
YOR096W	YPL012W	936
YOR096W	YPL037C	953
YOR096W	YPL079W	971
YOR096W	YPL081W	999
YOR096W	YPL090C	996
YOR096W	YPL126W	933
YOR096W	YPL131W	999
YOR096W	YPL143W	982
YOR096W	YPL198W	995
YOR096W	YPL217C	942
YOR096W	YPL220W	998
YOR096W	YPL237W	931
YOR096W	YPL249C-A	995

YOR096W	YPR041W	915
YOR096W	YPR043W	940
YOR096W	YPR080W	903
YOR096W	YPR102C	995
YOR096W	YPR132W	964
YOR096W	YPR144C	981
YOR096W	YPR163C	916
YOR098C	YAR002W	999
YOR098C	YBL079W	998
YOR098C	YBR111W-A	936
YOR098C	YDL029W	927
YOR098C	YDL116W	928
YOR098C	YDL207W	991
YOR098C	YDR159W	993
YOR098C	YDR192C	963
YOR098C	YER107C	926
YOR098C	YER110C	997
YOR098C	YFR002W	926
YOR098C	YGL016W	990
YOR098C	YGL092W	931
YOR098C	YGL172W	948
YOR098C	YGR119C	981
YOR098C	YIL115C	912
YOR098C	YJL041W	997
YOR098C	YJL061W	999
YOR098C	YJR042W	931
YOR098C	YKL068W	994
YOR098C	YKL186C	981
YOR098C	YKR082W	930
YOR098C	YLR335W	999
YOR098C	YLR347C	999
YOR098C	YML103C	979
YOR098C	YMR047C	987
YOR098C	YMR129W	988
YOR098C	YMR235C	976
YOR098C	YMR308C	966
YOR098C	YNL189W	999
YOR098C	YNR030W	906
YOR098C	YOL072W	921
YOR098C	YOR160W	963
YOR098C	YOR257W	961
YOR098C	YPL020C	958
YOR098C	YPL153C	938
YOR098C	YPL169C	995

YOR100C	YAR035W	951
YOR101W	YAR019C	967
YOR101W	YBR140C	998
YOR101W	YBR159W	962
YOR101W	YIL033C	998
YOR101W	YJL005W	996
YOR101W	YJL164C	997
YOR101W	YLR310C	999
YOR101W	YNL098C	999
YOR101W	YOL081W	999
YOR101W	YOR335C	935
YOR101W	YOR360C	929
YOR103C	YDL232W	999
YOR103C	YEL002C	999
YOR103C	YER087C-B	979
YOR103C	YGL022W	999
YOR103C	YGL226C-A	999
YOR103C	YJL002C	999
YOR103C	YLR378C	984
YOR103C	YML019W	999
YOR103C	YMR149W	999
YOR103C	YOR085W	999
YOR106W	YAL030W	978
YOR106W	YBL050W	999
YOR106W	YBR080C	988
YOR106W	YDL077C	944
YOR106W	YDR080W	997
YOR106W	YEL013W	904
YOR106W	YGL124C	907
YOR106W	YGL212W	999
YOR106W	YJL168C	952
YOR106W	YKL006C-A	998
YOR106W	YKL196C	999
YOR106W	YLR093C	999
YOR106W	YLR148W	999
YOR106W	YLR396C	999
YOR106W	YML001W	950
YOR106W	YMR197C	999
YOR106W	YMR231W	905
YOR106W	YOR036W	999
YOR106W	YPL045W	998
YOR107W	YER020W	955
YOR107W	YHR005C	970
YOR108W	YGL009C	939

YOR108W	YJR016C	934
YOR108W	YNL104C	999
YOR108W	YOL058W	913
YOR109W	YFR019W	956
YOR109W	YGL206C	992
YOR109W	YHR108W	974
YOR109W	YIL002C	997
YOR109W	YJR110W	999
YOR109W	YKL212W	998
YOR109W	YLR240W	921
YOR109W	YLR305C	943
YOR109W	YNL106C	999
YOR109W	YNL267W	995
YOR109W	YNL325C	935
YOR109W	YOL065C	932
YOR109W	YPL268W	914
YOR109W	YPR029C	974
YOR110W	YAL001C	999
YOR110W	YBR123C	999
YOR110W	YDR362C	999
YOR110W	YGR047C	999
YOR110W	YPL007C	999
YOR111W	YJR125C	943
YOR112W	YKL205W	976
YOR112W	YKR048C	940
YOR112W	YLR335W	977
YOR112W	YMR047C	988
YOR115C	YAL026C	910
YOR115C	YBR254C	999
YOR115C	YDR108W	999
YOR115C	YDR246W	999
YOR115C	YDR407C	999
YOR115C	YDR472W	999
YOR115C	YGR166W	999
YOR115C	YKR068C	999
YOR115C	YML077W	999
YOR115C	YMR218C	999
YOR115C	YOL082W	927
YOR116C	YBR154C	999
YOR116C	YDL140C	980
YOR116C	YDL150W	999
YOR116C	YDR005C	999
YOR116C	YDR045C	999
YOR116C	YDR156W	921

YOR116C	YDR224C	978
YOR116C	YDR404C	932
YOR116C	YGL070C	999
YOR116C	YGR246C	901
YOR116C	YHR143W-A	961
YOR116C	YIL021W	941
YOR116C	YJL011C	999
YOR116C	YJL140W	904
YOR116C	YJL148W	922
YOR116C	YJR063W	921
YOR116C	YKL144C	999
YOR116C	YKR025W	999
YOR116C	YMR125W	982
YOR116C	YNL039W	960
YOR116C	YNL113W	999
YOR116C	YNL151C	999
YOR116C	YNL248C	908
YOR116C	YNR003C	999
YOR116C	YOL005C	900
YOR116C	YOR151C	980
YOR116C	YOR207C	999
YOR116C	YOR210W	999
YOR116C	YOR224C	999
YOR116C	YOR304W	968
YOR116C	YOR340C	921
YOR116C	YOR341W	938
YOR116C	YPL082C	970
YOR116C	YPL178W	982
YOR116C	YPR010C	946
YOR116C	YPR086W	937
YOR116C	YPR110C	999
YOR116C	YPR187W	999
YOR116C	YPR190C	999
YOR117W	YBL041W	909
YOR117W	YBR272C	994
YOR117W	YDL007W	999
YOR117W	YDL097C	999
YOR117W	YDL147W	999
YOR117W	YDR363W-A	999
YOR117W	YDR394W	999
YOR117W	YDR427W	999
YOR117W	YEL037C	992
YOR117W	YER012W	999
YOR117W	YER021W	998

YOR117W	YER094C	913
YOR117W	YFL007W	942
YOR117W	YFR004W	999
YOR117W	YFR010W	988
YOR117W	YFR050C	908
YOR117W	YFR052W	999
YOR117W	YGL004C	949
YOR117W	YGL048C	999
YOR117W	YGL141W	954
YOR117W	YGR092W	911
YOR117W	YGR232W	999
YOR117W	YHL030W	997
YOR117W	YHR027C	999
YOR117W	YHR200W	999
YOR117W	YIL007C	974
YOR117W	YIL075C	998
YOR117W	YKL145W	999
YOR117W	YLL039C	970
YOR117W	YLR421C	999
YOR117W	YML092C	950
YOR117W	YMR191W	987
YOR117W	YMR314W	997
YOR117W	YOR157C	906
YOR117W	YOR259C	999
YOR117W	YOR261C	999
YOR117W	YPR103W	907
YOR117W	YPR108W	998
YOR119C	YOR061W	980
YOR120W	YHR104W	976
YOR122C	YER008C	991
YOR122C	YFL039C	999
YOR122C	YIL159W	999
YOR122C	YLL050C	977
YOR122C	YNL090W	968
YOR122C	YNL138W	999
YOR122C	YNL271C	998
YOR122C	YOR094W	964
YOR122C	YOR363C	932
YOR123C	YAL011W	908
YOR123C	YBR231C	910
YOR123C	YBR253W	964
YOR123C	YBR279W	999
YOR123C	YDR308C	964
YOR123C	YDR311W	911

YOR123C	YDR334W	913
YOR123C	YDR443C	916
YOR123C	YDR485C	909
YOR123C	YER022W	964
YOR123C	YER148W	952
YOR123C	YER171W	916
YOR123C	YGL019W	966
YOR123C	YGL207W	999
YOR123C	YGL244W	999
YOR123C	YGR104C	964
YOR123C	YGR186W	932
YOR123C	YHR041C	964
YOR123C	YIL035C	968
YOR123C	YJL168C	952
YOR123C	YKL139W	971
YOR123C	YKL160W	903
YOR123C	YLR085C	943
YOR123C	YLR418C	999
YOR123C	YML010W	969
YOR123C	YML041C	938
YOR123C	YML069W	999
YOR123C	YNR010W	950
YOR123C	YOL012C	986
YOR123C	YOL051W	964
YOR123C	YOL145C	999
YOR123C	YOR061W	968
YOR123C	YOR174W	912
YOR123C	YPL083C	998
YOR123C	YPR070W	942
YOR124C	YBL101C	930
YOR124C	YER125W	999
YOR124C	YGR268C	950
YOR124C	YOR138C	999
YOR124C	YPR030W	916
YOR125C	YDR204W	986
YOR125C	YGL119W	912
YOR125C	YLR201C	952
YOR125C	YML110C	988
YOR125C	YOL008W	930
YOR125C	YOL096C	998
YOR127W	YDR309C	905
YOR127W	YDR379W	999
YOR127W	YGR152C	970
YOR127W	YJL128C	914

YOR127W	YLR229C	999
YOR127W	YNL298W	998
YOR127W	YOR212W	970
YOR127W	YPL115C	911
YOR127W	YPL256C	962
YOR127W	YPR165W	994
YOR128C	YAR015W	998
YOR128C	YCR066W	906
YOR128C	YDR399W	960
YOR128C	YDR408C	996
YOR128C	YGL234W	999
YOR128C	YGR061C	931
YOR128C	YLR359W	979
YOR128C	YMR300C	995
YOR129C	YNL225C	981
YOR130C	YJL088W	928
YOR130C	YOL140W	942
YOR130C	YPL111W	941
YOR132W	YBL017C	906
YOR132W	YHR012W	999
YOR132W	YJL036W	966
YOR132W	YJL053W	999
YOR132W	YJL154C	999
YOR132W	YMR004W	997
YOR132W	YOR069W	999
YOR133W	YBL027W	914
YOR133W	YBL072C	912
YOR133W	YBL087C	927
YOR133W	YBL092W	914
YOR133W	YBR031W	937
YOR133W	YBR048W	931
YOR133W	YBR084C-A	970
YOR133W	YBR118W	951
YOR133W	YBR181C	906
YOR133W	YBR189W	914
YOR133W	YBR191W	935
YOR133W	YCR031C	925
YOR133W	YDL061C	921
YOR133W	YDL075W	940
YOR133W	YDL081C	916
YOR133W	YDL082W	914
YOR133W	YDL130W	911
YOR133W	YDL136W	939
YOR133W	YDR012W	941

YOR133W	YDR064W	954
YOR133W	YDR382W	944
YOR133W	YDR385W	999
YOR133W	YDR418W	920
YOR133W	YDR447C	909
YOR133W	YDR450W	912
YOR133W	YDR471W	913
YOR133W	YDR500C	960
YOR133W	YEL054C	901
YOR133W	YER056C-A	932
YOR133W	YER086W	960
YOR133W	YER102W	990
YOR133W	YER117W	971
YOR133W	YFL034C-A	909
YOR133W	YFR031C-A	913
YOR133W	YFR032C-A	923
YOR133W	YGL030W	965
YOR133W	YGL076C	913
YOR133W	YGL103W	925
YOR133W	YGL123W	944
YOR133W	YGL135W	906
YOR133W	YGL147C	910
YOR133W	YGR027C	907
YOR133W	YGR034W	909
YOR133W	YGR085C	916
YOR133W	YGR118W	964
YOR133W	YGR148C	909
YOR133W	YGR187C	953
YOR133W	YGR214W	932
YOR133W	YHL001W	925
YOR133W	YHL015W	972
YOR133W	YHL033C	935
YOR133W	YHR010W	913
YOR133W	YHR021C	912
YOR133W	YHR141C	913
YOR133W	YHR203C	994
YOR133W	YIL018W	913
YOR133W	YIL052C	930
YOR133W	YIL103W	982
YOR133W	YIL133C	911
YOR133W	YIL148W	955
YOR133W	YJL136C	911
YOR133W	YJL177W	932
YOR133W	YJL189W	908

YOR133W	YJL190C	901
YOR133W	YJL191W	918
YOR133W	YJR094W-A	934
YOR133W	YJR123W	983
YOR133W	YJR145C	912
YOR133W	YKL006W	936
YOR133W	YKL060C	970
YOR133W	YKL156W	925
YOR133W	YKL180W	934
YOR133W	YKL191W	986
YOR133W	YKR057W	911
YOR133W	YKR059W	990
YOR133W	YKR094C	903
YOR133W	YLL045C	903
YOR133W	YLR029C	916
YOR133W	YLR048W	956
YOR133W	YLR061W	943
YOR133W	YLR075W	931
YOR133W	YLR167W	953
YOR133W	YLR185W	965
YOR133W	YLR249W	986
YOR133W	YLR264W	921
YOR133W	YLR333C	907
YOR133W	YLR340W	967
YOR133W	YLR344W	934
YOR133W	YLR367W	911
YOR133W	YLR388W	932
YOR133W	YLR406C	909
YOR133W	YLR441C	919
YOR133W	YLR448W	948
YOR133W	YML024W	937
YOR133W	YML026C	912
YOR133W	YML063W	930
YOR133W	YML073C	917
YOR133W	YMR142C	914
YOR133W	YMR194W	937
YOR133W	YMR230W	910
YOR133W	YMR242C	917
YOR133W	YNL067W	922
YOR133W	YNL069C	910
YOR133W	YNL096C	902
YOR133W	YNL162W	913
YOR133W	YNL178W	955
YOR133W	YNL301C	977

YOR133W	YNL302C	907
YOR133W	YOL039W	908
YOR133W	YOL040C	919
YOR133W	YOL086C	970
YOR133W	YOL120C	929
YOR133W	YOL121C	907
YOR133W	YOL127W	926
YOR133W	YOR063W	942
YOR133W	YOR096W	918
YOR133W	YOR151C	981
YOR133W	YOR167C	905
YOR133W	YOR234C	923
YOR133W	YOR293W	910
YOR133W	YOR312C	927
YOR133W	YOR369C	901
YOR133W	YPL079W	913
YOR133W	YPL090C	906
YOR133W	YPL131W	958
YOR133W	YPL143W	927
YOR133W	YPL198W	913
YOR133W	YPL220W	975
YOR133W	YPL249C-A	925
YOR133W	YPR043W	949
YOR133W	YPR080W	949
YOR133W	YPR102C	916
YOR133W	YPR132W	999
YOR134W	YPR165W	990
YOR136W	YBL022C	927
YOR136W	YDL066W	984
YOR136W	YGL195W	975
YOR136W	YIL094C	998
YOR136W	YIL125W	980
YOR136W	YJL200C	931
YOR136W	YLR174W	984
YOR136W	YLR304C	994
YOR136W	YNL009W	902
YOR136W	YNL037C	999
YOR136W	YNR001C	987
YOR136W	YPL235W	969
YOR138C	YER125W	999
YOR138C	YGR268C	990
YOR138C	YOR124C	999
YOR140W	YBL093C	958
YOR140W	YBR112C	936

YOR140W	YCR081W	979
YOR140W	YDR443C	943
YOR140W	YGL181W	963
YOR140W	YMR164C	930
YOR140W	YNL025C	966
YOR140W	YNL236W	972
YOR140W	YPL203W	995
YOR141C	YDL002C	999
YOR141C	YDR190C	997
YOR141C	YER092W	998
YOR141C	YFL013C	998
YOR141C	YFL039C	998
YOR141C	YGL150C	999
YOR141C	YJL081C	999
YOR141C	YLR052W	997
YOR141C	YNL059C	999
YOR141C	YNL215W	996
YOR141C	YPL129W	996
YOR141C	YPL235W	999
YOR142W	YDR148C	993
YOR142W	YGR244C	999
YOR142W	YIL125W	966
YOR142W	YJL045W	950
YOR142W	YKL148C	951
YOR142W	YLL041C	981
YOR144C	YBR087W	999
YOR144C	YBR088C	959
YOR144C	YBR094W	959
YOR144C	YBR098W	965
YOR144C	YCL061C	954
YOR144C	YDR004W	984
YOR144C	YDR076W	982
YOR144C	YDR386W	957
YOR144C	YER095W	984
YOR144C	YER173W	999
YOR144C	YGL163C	985
YOR144C	YHR164C	969
YOR144C	YJL092W	998
YOR144C	YJR043C	930
YOR144C	YJR068W	999
YOR144C	YKL113C	999
YOR144C	YLL002W	943
YOR144C	YLR234W	916
YOR144C	YLR288C	941

YOR144C	YML032C	999
YOR144C	YMR078C	995
YOR144C	YMR190C	988
YOR144C	YMR224C	995
YOR144C	YNL250W	984
YOR144C	YNL273W	948
YOR144C	YNL290W	999
YOR144C	YOL094C	999
YOR144C	YOR217W	962
YOR144C	YOR368W	923
YOR144C	YPL008W	962
YOR144C	YPL153C	999
YOR144C	YPL194W	964
YOR144C	YPR135W	990
YOR145C	YBL004W	997
YOR145C	YBR189W	981
YOR145C	YBR247C	981
YOR145C	YCR057C	998
YOR145C	YDL014W	913
YOR145C	YDL060W	999
YOR145C	YDL153C	911
YOR145C	YDR064W	980
YOR145C	YDR299W	942
YOR145C	YDR324C	997
YOR145C	YDR339C	904
YOR145C	YDR398W	917
YOR145C	YDR449C	997
YOR145C	YEL026W	943
YOR145C	YGL120C	922
YOR145C	YGL171W	916
YOR145C	YGR090W	990
YOR145C	YGR128C	937
YOR145C	YHR148W	998
YOR145C	YHR169W	955
YOR145C	YHR196W	970
YOR145C	YJL069C	962
YOR145C	YJL109C	916
YOR145C	YJR002W	998
YOR145C	YKL099C	913
YOR145C	YLL011W	934
YOR145C	YLR003C	978
YOR145C	YLR129W	998
YOR145C	YLR175W	950
YOR145C	YLR186W	998

YOR145C	YLR197W	916
YOR145C	YLR222C	954
YOR145C	YLR409C	923
YOR145C	YML093W	919
YOR145C	YMR093W	998
YOR145C	YMR229C	931
YOR145C	YMR290C	948
YOR145C	YNL075W	967
YOR145C	YNL132W	915
YOR145C	YNL207W	999
YOR145C	YOL010W	915
YOR145C	YOR004W	967
YOR145C	YOR078W	975
YOR145C	YPL012W	997
YOR145C	YPL126W	999
YOR145C	YPL217C	962
YOR145C	YPL266W	999
YOR145C	YPR112C	910
YOR145C	YPR144C	911
YOR147W	YAL010C	905
YOR147W	YGL219C	917
YOR147W	YHR194W	975
YOR147W	YLL006W	905
YOR147W	YOL009C	913
YOR148C	YNR011C	976
YOR149C	YDR302W	947
YOR149C	YLL031C	951
YOR149C	YNL038W	922
YOR150W	YBL038W	999
YOR150W	YBR122C	973
YOR150W	YBR146W	975
YOR150W	YCR046C	991
YOR150W	YCR071C	970
YOR150W	YDR116C	993
YOR150W	YDR237W	996
YOR150W	YDR296W	992
YOR150W	YDR322W	972
YOR150W	YDR462W	970
YOR150W	YGR220C	985
YOR150W	YHL004W	962
YOR150W	YHR147C	952
YOR150W	YJL063C	999
YOR150W	YKR085C	970
YOR150W	YLR439W	972

YOR150W	YMR024W	978
YOR150W	YNL005C	992
YOR150W	YNL185C	917
YOR150W	YNL252C	972
YOR150W	YNL284C	991
YOR151C	YBL039C	977
YOR151C	YBR154C	999
YOR151C	YBR236C	983
YOR151C	YBR279W	927
YOR151C	YDL005C	923
YOR151C	YDL108W	986
YOR151C	YDL115C	997
YOR151C	YDL140C	999
YOR151C	YDR030C	935
YOR151C	YDR138W	973
YOR151C	YDR167W	977
YOR151C	YDR171W	969
YOR151C	YDR289C	917
YOR151C	YDR404C	999
YOR151C	YER022W	981
YOR151C	YER029C	900
YOR151C	YER148W	933
YOR151C	YER171W	908
YOR151C	YFL017W-A	902
YOR151C	YGL025C	922
YOR151C	YGL043W	999
YOR151C	YGL070C	999
YOR151C	YGL127C	983
YOR151C	YGL244W	979
YOR151C	YGR005C	999
YOR151C	YGR063C	998
YOR151C	YGR074W	901
YOR151C	YGR094W	915
YOR151C	YGR104C	986
YOR151C	YGR186W	999
YOR151C	YHR058C	915
YOR151C	YHR143W-A	999
YOR151C	YHR165C	903
YOR151C	YIL021W	999
YOR151C	YIL143C	909
YOR151C	YJL140W	999
YOR151C	YJL168C	999
YOR151C	YJR035W	920
YOR151C	YKL028W	948

YOR151C	YKL104C	973
YOR151C	YKR062W	949
YOR151C	YLR071C	999
YOR151C	YLR106C	972
YOR151C	YLR147C	901
YOR151C	YLR180W	975
YOR151C	YLR418C	981
YOR151C	YML010W	999
YOR151C	YMR012W	974
YOR151C	YMR277W	955
YOR151C	YMR288W	901
YOR151C	YNL222W	997
YOR151C	YNL236W	915
YOR151C	YNR010W	982
YOR151C	YOL005C	999
YOR151C	YOL051W	911
YOR151C	YOL135C	930
YOR151C	YOR116C	980
YOR151C	YOR133W	981
YOR151C	YOR207C	931
YOR151C	YOR210W	999
YOR151C	YOR224C	999
YOR151C	YPL129W	999
YOR151C	YPR010C	995
YOR151C	YPR070W	915
YOR151C	YPR086W	999
YOR151C	YPR093C	989
YOR151C	YPR110C	972
YOR151C	YPR168W	942
YOR151C	YPR182W	904
YOR151C	YPR187W	999
YOR153W	YAL053W	998
YOR153W	YBL005W	999
YOR153W	YDR011W	999
YOR153W	YDR406W	984
YOR153W	YGL008C	952
YOR153W	YGL013C	999
YOR153W	YGR266W	949
YOR153W	YGR281W	999
YOR153W	YIL013C	907
YOR153W	YML008C	989
YOR153W	YML029W	915
YOR153W	YNL154C	923
YOR153W	YNL231C	911

YOR153W	YOR049C	976
YOR153W	YOR328W	903
YOR153W	YOR380W	910
YOR153W	YPL058C	924
YOR154W	YER140W	999
YOR155C	YNL168C	998
YOR156C	YAL032C	916
YOR156C	YDL013W	997
YOR156C	YDL064W	997
YOR156C	YDR096W	900
YOR156C	YDR409W	999
YOR156C	YDR446W	983
YOR156C	YDR510W	999
YOR156C	YEL019C	989
YOR156C	YER016W	974
YOR156C	YER116C	996
YOR156C	YHR156C	904
YOR156C	YLR314C	982
YOR156C	YPL020C	922
YOR156C	YPL269W	972
YOR156C	YPR034W	983
YOR157C	YBL041W	999
YOR157C	YBR173C	992
YOR157C	YDL007W	905
YOR157C	YDL097C	941
YOR157C	YDL147W	942
YOR157C	YDR394W	903
YOR157C	YDR427W	928
YOR157C	YER012W	999
YOR157C	YER021W	939
YOR157C	YER094C	999
YOR157C	YER144C	999
YOR157C	YFR004W	938
YOR157C	YFR050C	999
YOR157C	YFR052W	910
YOR157C	YGL011C	999
YOR157C	YGL048C	953
YOR157C	YGR135W	999
YOR157C	YGR253C	999
YOR157C	YHR027C	926
YOR157C	YHR200W	981
YOR157C	YIL075C	909
YOR157C	YJL001W	999
YOR157C	YKL145W	906

YOR157C	YML092C	999
YOR157C	YMR314W	999
YOR157C	YOL038W	999
YOR157C	YOR117W	906
YOR157C	YOR259C	921
YOR157C	YOR362C	999
YOR157C	YPR103W	999
YOR157C	YPR108W	927
YOR158W	Q0140	962
YOR158W	YBL090W	988
YOR158W	YBR146W	988
YOR158W	YBR251W	988
YOR158W	YDR041W	988
YOR158W	YDR175C	988
YOR158W	YDR337W	988
YOR158W	YDR347W	998
YOR158W	YER050C	986
YOR158W	YER153C	998
YOR158W	YGL129C	988
YOR158W	YGR061C	929
YOR158W	YGR084C	988
YOR158W	YGR165W	988
YOR158W	YGR215W	988
YOR158W	YHL004W	999
YOR158W	YIL093C	988
YOR158W	YJR101W	988
YOR158W	YJR113C	988
YOR158W	YKL155C	997
YOR158W	YMR158W	974
YOR158W	YMR188C	988
YOR158W	YNL137C	988
YOR158W	YNL306W	988
YOR158W	YNR037C	987
YOR158W	YPL013C	996
YOR158W	YPL118W	989
YOR159C	YBL026W	998
YOR159C	YBR055C	999
YOR159C	YBR119W	987
YOR159C	YDL030W	977
YOR159C	YDR473C	987
YOR159C	YEL026W	909
YOR159C	YER029C	999
YOR159C	YER172C	999
YOR159C	YFL017W-A	999

YOR159C	YGR074W	999
YOR159C	YHR165C	999
YOR159C	YJL124C	905
YOR159C	YKL012W	997
YOR159C	YKL173W	999
YOR159C	YLR147C	999
YOR159C	YLR275W	999
YOR159C	YLR438C-A	989
YOR159C	YML049C	999
YOR159C	YMR125W	966
YOR159C	YMR288W	999
YOR159C	YNL147W	942
YOR159C	YOL005C	942
YOR159C	YOR224C	929
YOR159C	YPL213W	999
YOR159C	YPR178W	999
YOR159C	YPR182W	999
YOR159C	YPR187W	915
YOR160W	YBR017C	950
YOR160W	YDR432W	999
YOR160W	YJR042W	906
YOR160W	YLR293C	996
YOR160W	YMR216C	950
YOR160W	YNL004W	940
YOR160W	YNR007C	933
YOR160W	YOR098C	963
YOR162C	YDR011W	954
YOR164C	YNR050C	957
YOR164C	YOL111C	942
YOR164C	YOR007C	949
YOR165W	YGL126W	923
YOR165W	YLL023C	919
YOR165W	YPR028W	995
YOR167C	YAL035W	945
YOR167C	YBL027W	909
YOR167C	YBL072C	909
YOR167C	YBL087C	925
YOR167C	YBL092W	946
YOR167C	YBR031W	934
YOR167C	YBR084C-A	909
YOR167C	YBR118W	902
YOR167C	YBR181C	933
YOR167C	YBR189W	981
YOR167C	YBR191W	966

YOR167C	YCR031C	939
YOR167C	YDL061C	967
YOR167C	YDL075W	941
YOR167C	YDL081C	937
YOR167C	YDL082W	923
YOR167C	YDL130W	901
YOR167C	YDL136W	916
YOR167C	YDL191W	905
YOR167C	YDR012W	940
YOR167C	YDR064W	983
YOR167C	YDR382W	957
YOR167C	YDR385W	905
YOR167C	YDR418W	948
YOR167C	YDR447C	914
YOR167C	YDR450W	912
YOR167C	YDR471W	940
YOR167C	YDR500C	912
YOR167C	YEL015W	959
YOR167C	YEL054C	910
YOR167C	YER025W	944
YOR167C	YER056C-A	941
YOR167C	YER074W	900
YOR167C	YER102W	909
YOR167C	YER117W	916
YOR167C	YER131W	963
YOR167C	YFL034C-A	907
YOR167C	YFR031C-A	929
YOR167C	YFR032C-A	922
YOR167C	YGL030W	961
YOR167C	YGL031C	963
YOR167C	YGL076C	954
YOR167C	YGL103W	960
YOR167C	YGL123W	976
YOR167C	YGL135W	914
YOR167C	YGL147C	936
YOR167C	YGL189C	902
YOR167C	YGR027C	970
YOR167C	YGR034W	929
YOR167C	YGR085C	955
YOR167C	YGR118W	968
YOR167C	YGR148C	961
YOR167C	YGR214W	954
YOR167C	YHL001W	970
YOR167C	YHL015W	965

YOR167C	YHL033C	945
YOR167C	YHR010W	963
YOR167C	YHR021C	912
YOR167C	YHR141C	943
YOR167C	YHR203C	916
YOR167C	YIL018W	916
YOR167C	YIL052C	954
YOR167C	YIL069C	900
YOR167C	YIL133C	930
YOR167C	YIL148W	912
YOR167C	YJL136C	943
YOR167C	YJL177W	939
YOR167C	YJL189W	921
YOR167C	YJL190C	944
YOR167C	YJL191W	941
YOR167C	YJR007W	909
YOR167C	YJR094W-A	936
YOR167C	YJR123W	958
YOR167C	YJR145C	916
YOR167C	YKL006W	967
YOR167C	YKL156W	954
YOR167C	YKL180W	955
YOR167C	YKR057W	916
YOR167C	YKR094C	903
YOR167C	YLL045C	934
YOR167C	YLR029C	940
YOR167C	YLR048W	955
YOR167C	YLR061W	953
YOR167C	YLR075W	931
YOR167C	YLR167W	910
YOR167C	YLR185W	903
YOR167C	YLR264W	986
YOR167C	YLR287C-A	902
YOR167C	YLR325C	921
YOR167C	YLR333C	929
YOR167C	YLR340W	945
YOR167C	YLR344W	933
YOR167C	YLR367W	963
YOR167C	YLR388W	971
YOR167C	YLR406C	913
YOR167C	YLR441C	909
YOR167C	YLR448W	942
YOR167C	YML024W	949
YOR167C	YML026C	912

YOR167C	YML063W	959
YOR167C	YML073C	913
YOR167C	YMR121C	903
YOR167C	YMR142C	929
YOR167C	YMR194W	945
YOR167C	YMR230W	962
YOR167C	YMR242C	926
YOR167C	YMR260C	900
YOR167C	YNL067W	969
YOR167C	YNL069C	927
YOR167C	YNL096C	912
YOR167C	YNL162W	918
YOR167C	YNL178W	952
YOR167C	YNL301C	917
YOR167C	YNL302C	910
YOR167C	YOL039W	925
YOR167C	YOL040C	925
YOR167C	YOL120C	908
YOR167C	YOL121C	923
YOR167C	YOL127W	963
YOR167C	YOL149W	983
YOR167C	YOR063W	951
YOR167C	YOR096W	959
YOR167C	YOR133W	905
YOR167C	YOR182C	902
YOR167C	YOR234C	970
YOR167C	YOR293W	976
YOR167C	YOR312C	920
YOR167C	YOR361C	914
YOR167C	YOR369C	938
YOR167C	YPL079W	959
YOR167C	YPL081W	927
YOR167C	YPL090C	920
YOR167C	YPL131W	921
YOR167C	YPL143W	954
YOR167C	YPL198W	912
YOR167C	YPL220W	925
YOR167C	YPL237W	919
YOR167C	YPL249C-A	912
YOR167C	YPR043W	913
YOR167C	YPR080W	902
YOR167C	YPR102C	986
YOR167C	YPR132W	923
YOR167C	YPR163C	914

YOR168W	YBL076C	924
YOR168W	YBL080C	916
YOR168W	YDR037W	944
YOR168W	YDR341C	904
YOR168W	YGL105W	914
YOR168W	YGR264C	948
YOR168W	YHR020W	927
YOR168W	YIL143C	923
YOR168W	YJR109C	900
YOR168W	YLL018C	900
YOR168W	YMR217W	914
YOR168W	YMR293C	913
YOR168W	YMR300C	903
YOR168W	YOR352W	925
YOR168W	YPL040C	901
YOR168W	YPL160W	936
YOR168W	YPR035W	915
YOR171C	YDR062W	903
YOR171C	YDR264C	902
YOR171C	YDR294C	999
YOR171C	YDR297W	997
YOR171C	YJL134W	995
YOR171C	YKR053C	945
YOR171C	YLR260W	984
YOR171C	YOR034C	955
YOR173W	YLR270W	999
YOR174W	YBL093C	999
YOR174W	YBR193C	999
YOR174W	YBR198C	943
YOR174W	YBR253W	999
YOR174W	YCR081W	974
YOR174W	YDL005C	999
YOR174W	YDL108W	997
YOR174W	YDL140C	975
YOR174W	YDR145W	969
YOR174W	YDR167W	991
YOR174W	YDR308C	999
YOR174W	YDR443C	997
YOR174W	YER022W	999
YOR174W	YER148W	944
YOR174W	YGL025C	999
YOR174W	YGL127C	999
YOR174W	YGL151W	997
YOR174W	YGR104C	999

YOR174W	YGR274C	943
YOR174W	YHR041C	998
YOR174W	YHR058C	999
YOR174W	YIL021W	975
YOR174W	YLR071C	999
YOR174W	YML007W	974
YOR174W	YML015C	974
YOR174W	YML098W	987
YOR174W	YMR005W	969
YOR174W	YMR112C	998
YOR174W	YMR227C	972
YOR174W	YMR236W	972
YOR174W	YNL236W	998
YOR174W	YNR010W	999
YOR174W	YOL051W	999
YOR174W	YOL135C	999
YOR174W	YOR123C	912
YOR174W	YPL011C	974
YOR174W	YPL042C	998
YOR174W	YPL129W	991
YOR174W	YPR070W	999
YOR174W	YPR168W	999
YOR175C	YDL052C	999
YOR176W	YDL120W	990
YOR176W	YDR047W	961
YOR177C	YGL170C	939
YOR177C	YOL091W	991
YOR178C	YBR045C	906
YOR178C	YDR028C	942
YOR178C	YDR477W	900
YOR178C	YER133W	999
YOR178C	YGL073W	909
YOR178C	YGL134W	903
YOR178C	YLR258W	984
YOR178C	YPL219W	903
YOR179C	YAL043C	999
YOR179C	YDR195W	999
YOR179C	YER133W	995
YOR179C	YGR156W	999
YOR179C	YKL018W	999
YOR179C	YLR277C	943
YOR179C	YNL222W	999
YOR180C	YLR284C	998
YOR181W	YBL007C	999

YOR181W	YBR200W	994
YOR181W	YBR234C	997
YOR181W	YBR260C	993
YOR181W	YCL034W	995
YOR181W	YCR009C	981
YOR181W	YCR088W	987
YOR181W	YDL029W	999
YOR181W	YDR129C	919
YOR181W	YDR388W	999
YOR181W	YER118C	939
YOR181W	YFL039C	999
YOR181W	YFR024C-A	999
YOR181W	YGL206C	928
YOR181W	YGR136W	981
YOR181W	YGR167W	903
YOR181W	YHR016C	997
YOR181W	YHR114W	999
YOR181W	YIL062C	971
YOR181W	YIL159W	955
YOR181W	YIR006C	999
YOR181W	YJL020C	997
YOR181W	YJL100W	998
YOR181W	YJR065C	992
YOR181W	YKL013C	975
YOR181W	YKL129C	999
YOR181W	YLR229C	904
YOR181W	YLR337C	999
YOR181W	YLR370C	934
YOR181W	YMR109W	999
YOR181W	YNL084C	992
YOR181W	YNL243W	970
YOR181W	YNR035C	935
YOR181W	YOR094W	906
YOR181W	YPR154W	972
YOR181W	YPR159W	976
YOR182C	YBL092W	905
YOR182C	YBR031W	912
YOR182C	YBR189W	902
YOR182C	YCR031C	908
YOR182C	YDL061C	900
YOR182C	YDL075W	909
YOR182C	YDL081C	902
YOR182C	YDL130W	902
YOR182C	YDL136W	905

YOR182C	YDL191W	905
YOR182C	YDR012W	912
YOR182C	YDR064W	903
YOR182C	YDR382W	914
YOR182C	YDR418W	912
YOR182C	YDR447C	902
YOR182C	YDR450W	901
YOR182C	YDR471W	910
YOR182C	YDR500C	906
YOR182C	YEL054C	912
YOR182C	YER056C-A	907
YOR182C	YER074W	901
YOR182C	YER131W	905
YOR182C	YFL034C-A	906
YOR182C	YFR032C-A	905
YOR182C	YGL030W	911
YOR182C	YGL076C	908
YOR182C	YGL135W	910
YOR182C	YGL189C	905
YOR182C	YGR034W	908
YOR182C	YGR118W	900
YOR182C	YHL001W	903
YOR182C	YHL015W	911
YOR182C	YHL033C	914
YOR182C	YHR010W	910
YOR182C	YHR021C	905
YOR182C	YHR141C	915
YOR182C	YHR203C	906
YOR182C	YIL052C	907
YOR182C	YIL069C	901
YOR182C	YIL133C	910
YOR182C	YJL136C	909
YOR182C	YJL177W	901
YOR182C	YJL190C	900
YOR182C	YJL191W	908
YOR182C	YJR094W-A	903
YOR182C	YJR145C	906
YOR182C	YKL006W	902
YOR182C	YKL156W	905
YOR182C	YKL180W	901
YOR182C	YKR057W	909
YOR182C	YLL045C	914
YOR182C	YLR029C	908
YOR182C	YLR061W	908

YOR182C	YLR185W	906
YOR182C	YLR264W	902
YOR182C	YLR287C-A	901
YOR182C	YLR340W	902
YOR182C	YLR344W	908
YOR182C	YLR367W	900
YOR182C	YLR388W	900
YOR182C	YLR406C	909
YOR182C	YLR441C	901
YOR182C	YLR448W	904
YOR182C	YML024W	902
YOR182C	YML026C	901
YOR182C	YML063W	901
YOR182C	YML073C	903
YOR182C	YMR121C	908
YOR182C	YMR194W	902
YOR182C	YMR230W	906
YOR182C	YMR242C	903
YOR182C	YNL069C	910
YOR182C	YNL096C	917
YOR182C	YNL162W	915
YOR182C	YNL178W	905
YOR182C	YNL302C	903
YOR182C	YOL121C	903
YOR182C	YOL127W	908
YOR182C	YOR096W	917
YOR182C	YOR167C	902
YOR182C	YOR234C	903
YOR182C	YOR293W	906
YOR182C	YOR312C	903
YOR182C	YOR369C	909
YOR182C	YPL081W	902
YOR182C	YPL143W	903
YOR182C	YPL198W	908
YOR182C	YPL220W	910
YOR182C	YPL249C-A	902
YOR182C	YPR043W	903
YOR182C	YPR132W	900
YOR184W	YCR053W	933
YOR184W	YEL046C	901
YOR184W	YER081W	986
YOR184W	YGR208W	989
YOR184W	YIL074C	986
YOR185C	YER009W	986

YOR185C	YGL016W	903
YOR185C	YGL097W	999
YOR185C	YJR074W	999
YOR185C	YLR293C	998
YOR185C	YMR235C	934
YOR185C	YMR308C	919
YOR187W	YJR113C	916
YOR187W	YNR036C	963
YOR188W	YAL041W	995
YOR188W	YBR200W	998
YOR188W	YDR309C	910
YOR188W	YER155C	994
YOR188W	YHR061C	912
YOR188W	YLR229C	999
YOR189W	YBL088C	914
YOR189W	YBR136W	905
YOR189W	YGL150C	996
YOR189W	YNL059C	954
YOR189W	YNL273W	930
YOR190W	YMR306W	912
YOR191W	YDR510W	949
YOR193W	YOL147C	991
YOR193W	YPL112C	984
YOR194C	YBR198C	900
YOR194C	YDL140C	906
YOR194C	YDR167W	904
YOR194C	YDR174W	905
YOR194C	YER148W	999
YOR194C	YGL016W	920
YOR194C	YGR274C	984
YOR194C	YIL143C	911
YOR194C	YKL058W	999
YOR194C	YML015C	984
YOR194C	YML098W	901
YOR194C	YPR056W	901
YOR194C	YPR086W	998
YOR195W	YDL003W	937
YOR195W	YDR318W	941
YOR195W	YEL061C	998
YOR195W	YER016W	994
YOR195W	YFR028C	947
YOR195W	YGL086W	993
YOR195W	YGR063C	917
YOR195W	YGR098C	991

YOR195W	YGR113W	984
YOR195W	YGR140W	954
YOR195W	YGR179C	941
YOR195W	YGR188C	927
YOR195W	YHR014W	910
YOR195W	YHR152W	938
YOR195W	YHR191C	953
YOR195W	YIL144W	902
YOR195W	YJL030W	997
YOR195W	YJR112W	976
YOR195W	YLR045C	928
YOR195W	YLR309C	999
YOR195W	YMR001C	997
YOR195W	YMR094W	928
YOR195W	YMR168C	924
YOR195W	YPL018W	955
YOR195W	YPR141C	920
YOR196C	YJL046W	938
YOR196C	YLR239C	988
YOR197W	YBL106C	920
YOR197W	YER112W	917
YOR197W	YGR166W	998
YOR198C	YGR085C	982
YOR198C	YHL015W	990
YOR198C	YJL080C	999
YOR198C	YOL040C	998
YOR198C	YPR052C	939
YOR201C	YNL284C	930
YOR202W	YBR248C	996
YOR202W	YCL030C	999
YOR202W	YEL009C	970
YOR202W	YER055C	973
YOR202W	YIL116W	997
YOR202W	YLR055C	953
YOR204W	YBR048W	931
YOR204W	YBR146W	986
YOR204W	YBR189W	931
YOR204W	YCL011C	978
YOR204W	YCR031C	929
YOR204W	YDL083C	931
YOR204W	YDL160C	916
YOR204W	YDR025W	962
YOR204W	YDR041W	987
YOR204W	YDR117C	927

YOR204W	YDR429C	998
YOR204W	YDR432W	963
YOR204W	YDR450W	976
YOR204W	YER102W	929
YOR204W	YFR031C-A	941
YOR204W	YGL030W	931
YOR204W	YGL129C	982
YOR204W	YGL135W	929
YOR204W	YGL173C	923
YOR204W	YGR054W	909
YOR204W	YGR085C	933
YOR204W	YGR159C	923
YOR204W	YGR162W	998
YOR204W	YHL004W	985
YOR204W	YHR089C	941
YOR204W	YIL018W	942
YOR204W	YIL069C	960
YOR204W	YIL133C	931
YOR204W	YJL050W	935
YOR204W	YJL098W	927
YOR204W	YJL177W	931
YOR204W	YJL190C	931
YOR204W	YJR101W	984
YOR204W	YJR123W	933
YOR204W	YJR145C	931
YOR204W	YKL155C	984
YOR204W	YKR024C	939
YOR204W	YKR059W	978
YOR204W	YLR002C	931
YOR204W	YLR029C	933
YOR204W	YML024W	970
YOR204W	YML063W	944
YOR204W	YML073C	933
YOR204W	YMR142C	962
YOR204W	YMR146C	975
YOR204W	YMR242C	933
YOR204W	YMR309C	978
YOR204W	YNL005C	978
YOR204W	YNL069C	931
YOR204W	YNL151C	969
YOR204W	YNL301C	937
YOR204W	YNL306W	984
YOR204W	YOL139C	995
YOR204W	YOR310C	923

YOR204W	YOR361C	976
YOR204W	YPL081W	974
YOR204W	YPL090C	964
YOR204W	YPL118W	991
YOR204W	YPL249C-A	927
YOR204W	YPR041W	977
YOR205C	YBR251W	906
YOR206W	YDL014W	997
YOR206W	YDL031W	988
YOR206W	YDR060W	999
YOR206W	YDR087C	995
YOR206W	YDR496C	983
YOR206W	YER006W	999
YOR206W	YER165W	933
YOR206W	YFL002C	994
YOR206W	YFR001W	932
YOR206W	YGL076C	940
YOR206W	YGL111W	994
YOR206W	YGR090W	905
YOR206W	YGR103W	998
YOR206W	YHR052W	999
YOR206W	YHR066W	980
YOR206W	YHR088W	995
YOR206W	YHR197W	980
YOR206W	YIL133C	941
YOR206W	YKL009W	994
YOR206W	YKL172W	989
YOR206W	YKR081C	999
YOR206W	YLL008W	984
YOR206W	YLL034C	987
YOR206W	YLL045C	933
YOR206W	YLR002C	999
YOR206W	YLR029C	931
YOR206W	YLR276C	986
YOR206W	YLR449W	998
YOR206W	YMR049C	999
YOR206W	YMR229C	999
YOR206W	YMR242C	941
YOR206W	YMR290C	994
YOR206W	YNL002C	993
YOR206W	YNL061W	999
YOR206W	YNL110C	997
YOR206W	YNL301C	925
YOR206W	YOL041C	991

YOR206W	YOL077C	998
YOR206W	YOL120C	976
YOR206W	YOL127W	936
YOR206W	YOR063W	945
YOR206W	YOR272W	998
YOR206W	YPL043W	997
YOR206W	YPL093W	990
YOR206W	YPL131W	960
YOR206W	YPL211W	991
YOR206W	YPR016C	998
YOR207C	YBR154C	999
YOR207C	YBR245C	970
YOR207C	YDL140C	985
YOR207C	YDL150W	999
YOR207C	YDR005C	998
YOR207C	YDR045C	999
YOR207C	YDR156W	921
YOR207C	YDR167W	954
YOR207C	YDR404C	927
YOR207C	YEL026W	914
YOR207C	YER148W	904
YOR207C	YGL070C	913
YOR207C	YGR094W	911
YOR207C	YHR143W-A	992
YOR207C	YIL021W	938
YOR207C	YJL011C	999
YOR207C	YJL140W	901
YOR207C	YJL148W	922
YOR207C	YJR063W	921
YOR207C	YKL144C	999
YOR207C	YKR002W	901
YOR207C	YKR025W	999
YOR207C	YNL039W	960
YOR207C	YNL113W	999
YOR207C	YNL151C	999
YOR207C	YNL248C	908
YOR207C	YNR003C	999
YOR207C	YOL005C	901
YOR207C	YOR116C	999
YOR207C	YOR151C	931
YOR207C	YOR210W	999
YOR207C	YOR224C	999
YOR207C	YOR340C	921
YOR207C	YOR341W	924

YOR207C	YPR010C	933
YOR207C	YPR086W	918
YOR207C	YPR110C	999
YOR207C	YPR187W	999
YOR207C	YPR190C	999
YOR208W	YBL056W	939
YOR208W	YDL006W	998
YOR208W	YDR162C	909
YOR208W	YER075C	999
YOR208W	YER089C	979
YOR208W	YHR030C	980
YOR208W	YIL147C	984
YOR208W	YIR026C	966
YOR208W	YJL128C	978
YOR208W	YLR113W	999
YOR209C	YFR047C	990
YOR209C	YGL037C	987
YOR209C	YGR010W	960
YOR209C	YLR328W	957
YOR210W	YBL026W	917
YOR210W	YBR154C	999
YOR210W	YBR198C	901
YOR210W	YDL115C	996
YOR210W	YDL140C	999
YOR210W	YDL150W	998
YOR210W	YDR045C	901
YOR210W	YDR156W	998
YOR210W	YDR167W	912
YOR210W	YDR404C	999
YOR210W	YEL026W	957
YOR210W	YER022W	981
YOR210W	YER029C	929
YOR210W	YER148W	928
YOR210W	YER171W	927
YOR210W	YFL017W-A	971
YOR210W	YGL043W	995
YOR210W	YGL070C	999
YOR210W	YGL112C	901
YOR210W	YGL127C	907
YOR210W	YGL166W	900
YOR210W	YGR005C	998
YOR210W	YGR074W	950
YOR210W	YGR186W	998
YOR210W	YHR058C	901

YOR210W	YHR143W-A	999
YOR210W	YIL021W	999
YOR210W	YIL143C	924
YOR210W	YJL011C	978
YOR210W	YJL140W	999
YOR210W	YJL148W	998
YOR210W	YJR035W	902
YOR210W	YJR063W	999
YOR210W	YKL058W	904
YOR210W	YKL144C	998
YOR210W	YKR025W	997
YOR210W	YLR147C	912
YOR210W	YLR275W	911
YOR210W	YMR277W	951
YOR210W	YMR288W	908
YOR210W	YNL113W	999
YOR210W	YNL151C	997
YOR210W	YNL248C	998
YOR210W	YNR003C	997
YOR210W	YOL005C	999
YOR210W	YOL135C	910
YOR210W	YOR116C	999
YOR210W	YOR151C	999
YOR210W	YOR207C	999
YOR210W	YOR224C	999
YOR210W	YOR340C	999
YOR210W	YOR341W	999
YOR210W	YPL129W	984
YOR210W	YPL178W	915
YOR210W	YPR010C	999
YOR210W	YPR025C	900
YOR210W	YPR086W	927
YOR210W	YPR110C	999
YOR210W	YPR168W	905
YOR210W	YPR182W	944
YOR210W	YPR187W	999
YOR210W	YPR190C	998
YOR211C	YBR179C	999
YOR211C	YDR470C	999
YOR211C	YGR101W	996
YOR211C	YLL001W	976
YOR212W	YAL041W	999
YOR212W	YBL016W	999
YOR212W	YBR083W	943

YOR212W	YBR200W	926
YOR212W	YCL032W	999
YOR212W	YCR093W	916
YOR212W	YDL159W	999
YOR212W	YDL165W	900
YOR212W	YDR103W	999
YOR212W	YDR183W	950
YOR212W	YDR264C	999
YOR212W	YFL026W	999
YOR212W	YGR040W	985
YOR212W	YHL007C	999
YOR212W	YHR005C	999
YOR212W	YHR066W	976
YOR212W	YHR084W	983
YOR212W	YIL015W	919
YOR212W	YIL047C	978
YOR212W	YJL157C	996
YOR212W	YJR086W	999
YOR212W	YKL178C	989
YOR212W	YLR113W	906
YOR212W	YLR362W	999
YOR212W	YLR452C	998
YOR212W	YOR127W	970
YOR212W	YOR281C	950
YOR212W	YPL256C	909
YOR213C	YDR181C	999
YOR213C	YDR227W	906
YOR213C	YJL115W	987
YOR213C	YKR101W	963
YOR213C	YMR127C	999
YOR213C	YNL107W	906
YOR216C	YDL058W	999
YOR216C	YDL192W	983
YOR216C	YDR285W	956
YOR216C	YER005W	923
YOR216C	YER157W	997
YOR216C	YGL054C	943
YOR216C	YGR120C	983
YOR216C	YJR134C	930
YOR216C	YLR039C	915
YOR216C	YLR078C	967
YOR216C	YLR268W	979
YOR216C	YNL051W	919
YOR216C	YPL240C	919

YOR217W	YBL035C	998
YOR217W	YBR087W	999
YOR217W	YBR088C	999
YOR217W	YDL102W	911
YOR217W	YDR217C	967
YOR217W	YER173W	999
YOR217W	YIR008C	909
YOR217W	YJR068W	999
YOR217W	YKL113C	960
YOR217W	YLR288C	985
YOR217W	YNL102W	946
YOR217W	YNL290W	999
YOR217W	YOL090W	919
YOR217W	YOL094C	999
YOR217W	YOR144C	962
YOR219C	YNL238W	924
YOR219C	YOR357C	902
YOR224C	YBL026W	933
YOR224C	YBR154C	999
YOR224C	YDL115C	997
YOR224C	YDL140C	999
YOR224C	YDL150W	999
YOR224C	YDR045C	997
YOR224C	YDR167W	908
YOR224C	YDR404C	999
YOR224C	YEL026W	931
YOR224C	YER022W	981
YOR224C	YER029C	939
YOR224C	YER148W	910
YOR224C	YER171W	921
YOR224C	YFL017W-A	935
YOR224C	YGL043W	995
YOR224C	YGL070C	999
YOR224C	YGL112C	900
YOR224C	YGL127C	905
YOR224C	YGR005C	999
YOR224C	YGR074W	941
YOR224C	YGR089W	911
YOR224C	YGR104C	983
YOR224C	YGR186W	998
YOR224C	YHR058C	907
YOR224C	YHR143W-A	999
YOR224C	YIL021W	999
YOR224C	YIL143C	919

YOR224C	YJL011C	999
YOR224C	YJL140W	999
YOR224C	YJR035W	900
YOR224C	YJR063W	999
YOR224C	YKL058W	902
YOR224C	YKL067W	922
YOR224C	YKL144C	999
YOR224C	YKR025W	997
YOR224C	YLR147C	942
YOR224C	YLR275W	921
YOR224C	YML010W	998
YOR224C	YMR277W	947
YOR224C	YNL113W	998
YOR224C	YNL151C	997
YOR224C	YNL248C	998
YOR224C	YNR003C	999
YOR224C	YOL005C	999
YOR224C	YOL051W	959
YOR224C	YOR116C	999
YOR224C	YOR151C	999
YOR224C	YOR159C	929
YOR224C	YOR207C	999
YOR224C	YOR210W	999
YOR224C	YOR340C	999
YOR224C	YOR341W	999
YOR224C	YPL129W	999
YOR224C	YPL178W	900
YOR224C	YPR010C	998
YOR224C	YPR086W	921
YOR224C	YPR110C	999
YOR224C	YPR168W	935
YOR224C	YPR182W	953
YOR224C	YPR187W	999
YOR224C	YPR190C	999
YOR226C	YCL017C	989
YOR226C	YPL088W	904
YOR227W	YER133W	957
YOR229W	YGL016W	905
YOR229W	YGR180C	995
YOR229W	YJL026W	980
YOR229W	YOR230W	998
YOR229W	YOR283W	901
YOR230W	YGL016W	901
YOR230W	YGR180C	999

YOR230W	YJL026W	999
YOR230W	YOR229W	998
YOR231W	YBL105C	997
YOR231W	YDR162C	909
YOR231W	YHR030C	999
YOR231W	YIL113W	932
YOR231W	YJL095W	999
YOR231W	YLL021W	997
YOR231W	YNL053W	923
YOR231W	YPL089C	949
YOR231W	YPL140C	996
YOR231W	YPR165W	928
YOR232W	YAL005C	924
YOR232W	YBL075C	927
YOR232W	YBR227C	900
YOR232W	YDL229W	917
YOR232W	YEL030W	999
YOR232W	YER103W	931
YOR232W	YFL016C	995
YOR232W	YIL022W	999
YOR232W	YJL034W	934
YOR232W	YJR045C	999
YOR232W	YLL024C	929
YOR232W	YLR203C	907
YOR232W	YLR369W	999
YOR232W	YMR214W	932
YOR232W	YNL064C	945
YOR232W	YNL209W	914
YOR232W	YNR017W	966
YOR233W	YJR053W	933
YOR233W	YMR055C	929
YOR234C	YAL035W	915
YOR234C	YBL027W	953
YOR234C	YBL072C	925
YOR234C	YBL087C	929
YOR234C	YBL092W	948
YOR234C	YBR031W	955
YOR234C	YBR048W	936
YOR234C	YBR084C-A	969
YOR234C	YBR118W	912
YOR234C	YBR181C	904
YOR234C	YBR189W	995
YOR234C	YBR191W	998
YOR234C	YCR031C	999

YOR234C	YDL061C	970
YOR234C	YDL075W	999
YOR234C	YDL081C	926
YOR234C	YDL082W	998
YOR234C	YDL130W	925
YOR234C	YDL136W	964
YOR234C	YDL191W	928
YOR234C	YDR012W	969
YOR234C	YDR025W	924
YOR234C	YDR064W	998
YOR234C	YDR382W	999
YOR234C	YDR385W	913
YOR234C	YDR418W	979
YOR234C	YDR447C	998
YOR234C	YDR450W	920
YOR234C	YDR471W	987
YOR234C	YDR500C	954
YOR234C	YEL054C	931
YOR234C	YER056C-A	993
YOR234C	YER074W	998
YOR234C	YER102W	951
YOR234C	YER117W	960
YOR234C	YER131W	971
YOR234C	YFL034C-A	925
YOR234C	YFR031C-A	907
YOR234C	YFR032C-A	948
YOR234C	YGL030W	997
YOR234C	YGL031C	952
YOR234C	YGL076C	966
YOR234C	YGL103W	995
YOR234C	YGL123W	977
YOR234C	YGL135W	918
YOR234C	YGL147C	938
YOR234C	YGL189C	920
YOR234C	YGR027C	980
YOR234C	YGR034W	998
YOR234C	YGR085C	999
YOR234C	YGR118W	995
YOR234C	YGR148C	977
YOR234C	YGR214W	995
YOR234C	YHL001W	997
YOR234C	YHL015W	979
YOR234C	YHL033C	979
YOR234C	YHR010W	974

YOR234C	YHR021C	932
YOR234C	YHR141C	938
YOR234C	YHR203C	957
YOR234C	YIL018W	907
YOR234C	YIL052C	993
YOR234C	YIL069C	923
YOR234C	YIL133C	924
YOR234C	YIL148W	953
YOR234C	YJL136C	968
YOR234C	YJL177W	999
YOR234C	YJL189W	943
YOR234C	YJL190C	932
YOR234C	YJL191W	982
YOR234C	YJR094W-A	985
YOR234C	YJR123W	990
YOR234C	YJR145C	923
YOR234C	YKL006W	998
YOR234C	YKL156W	999
YOR234C	YKL180W	994
YOR234C	YKR057W	934
YOR234C	YKR094C	906
YOR234C	YLL045C	941
YOR234C	YLR029C	974
YOR234C	YLR048W	995
YOR234C	YLR061W	995
YOR234C	YLR075W	999
YOR234C	YLR167W	939
YOR234C	YLR185W	952
YOR234C	YLR264W	996
YOR234C	YLR287C-A	903
YOR234C	YLR325C	925
YOR234C	YLR333C	934
YOR234C	YLR340W	971
YOR234C	YLR344W	980
YOR234C	YLR367W	974
YOR234C	YLR388W	996
YOR234C	YLR406C	921
YOR234C	YLR441C	931
YOR234C	YLR448W	996
YOR234C	YML024W	993
YOR234C	YML026C	920
YOR234C	YML063W	986
YOR234C	YML073C	998
YOR234C	YMR121C	921

YOR234C	YMR142C	945
YOR234C	YMR194W	995
YOR234C	YMR230W	967
YOR234C	YMR242C	998
YOR234C	YMR260C	929
YOR234C	YNL067W	991
YOR234C	YNL069C	923
YOR234C	YNL096C	929
YOR234C	YNL162W	928
YOR234C	YNL178W	987
YOR234C	YNL301C	965
YOR234C	YNL302C	933
YOR234C	YOL039W	932
YOR234C	YOL040C	955
YOR234C	YOL120C	927
YOR234C	YOL121C	933
YOR234C	YOL127W	996
YOR234C	YOR063W	976
YOR234C	YOR096W	994
YOR234C	YOR133W	923
YOR234C	YOR167C	970
YOR234C	YOR182C	903
YOR234C	YOR293W	995
YOR234C	YOR312C	946
YOR234C	YOR369C	938
YOR234C	YPL079W	973
YOR234C	YPL081W	958
YOR234C	YPL090C	904
YOR234C	YPL131W	969
YOR234C	YPL143W	991
YOR234C	YPL198W	931
YOR234C	YPL220W	949
YOR234C	YPL249C-A	954
YOR234C	YPR043W	947
YOR234C	YPR080W	912
YOR234C	YPR102C	993
YOR234C	YPR132W	962
YOR236W	YBR084W	909
YOR236W	YBR263W	926
YOR236W	YGR204W	909
YOR236W	YLR028C	902
YOR236W	YLR058C	918
YOR236W	YMR113W	900
YOR236W	YMR120C	902

YOR236W	YOR074C	999
YOR236W	YOR241W	927
YOR239W	YNL048W	998
YOR241W	YBR263W	912
YOR241W	YEL046C	920
YOR241W	YER091C	918
YOR241W	YGL125W	931
YOR241W	YLR058C	991
YOR241W	YNL256W	912
YOR241W	YOR236W	927
YOR242C	YKR031C	917
YOR242C	YPL232W	918
YOR243C	YBR251W	952
YOR243C	YDR165W	906
YOR243C	YGR162W	902
YOR243C	YHL004W	915
YOR243C	YLR002C	982
YOR243C	YOR356W	906
YOR244W	YBL003C	966
YOR244W	YBR009C	999
YOR244W	YDL042C	908
YOR244W	YDR225W	926
YOR244W	YDR334W	927
YOR244W	YDR359C	999
YOR244W	YEL018W	999
YOR244W	YER177W	939
YOR244W	YFL024C	999
YOR244W	YFL039C	999
YOR244W	YGR002C	999
YOR244W	YGR252W	958
YOR244W	YHR090C	999
YOR244W	YHR099W	999
YOR244W	YJL081C	999
YOR244W	YJR082C	999
YOR244W	YLR399C	975
YOR244W	YML007W	997
YOR244W	YNL030W	995
YOR244W	YNL107W	999
YOR244W	YNL136W	998
YOR244W	YOL012C	969
YOR244W	YPR023C	999
YOR245C	YNR008W	998
YOR245C	YNR019W	959
YOR247W	YLR110C	916

YOR247W	YNL161W	902
YOR247W	YOR248W	998
YOR248W	YOR247W	998
YOR249C	YBL084C	999
YOR249C	YDL008W	999
YOR249C	YDR118W	999
YOR249C	YDR260C	999
YOR249C	YFR036W	999
YOR249C	YGL240W	999
YOR249C	YHR166C	999
YOR249C	YIR025W	999
YOR249C	YKL022C	999
YOR249C	YLL039C	996
YOR249C	YLR102C	999
YOR249C	YLR127C	999
YOR249C	YNL172W	999
YOR250C	YBL075C	932
YOR250C	YDR228C	999
YOR250C	YDR301W	953
YOR250C	YGL044C	999
YOR250C	YGR047C	916
YOR250C	YHR216W	990
YOR250C	YMR061W	999
YOR251C	YOR346W	998
YOR253W	YDL040C	990
YOR253W	YGL144C	918
YOR253W	YHR013C	999
YOR254C	YBR171W	999
YOR254C	YDL072C	921
YOR254C	YDR086C	979
YOR254C	YER087C-B	997
YOR254C	YFR041C	992
YOR254C	YGR285C	918
YOR254C	YJL034W	999
YOR254C	YLR292C	999
YOR254C	YLR378C	999
YOR254C	YML055W	951
YOR254C	YOL031C	955
YOR254C	YOR291W	921
YOR254C	YPL094C	999
YOR257W	YAR002W	961
YOR257W	YBL079W	961
YOR257W	YBR111W-A	999
YOR257W	YDL088C	961

YOR257W	YDL116W	961
YOR257W	YDL207W	961
YOR257W	YDR159W	997
YOR257W	YDR192C	961
YOR257W	YDR356W	969
YOR257W	YEL037C	900
YOR257W	YER105C	961
YOR257W	YER107C	961
YOR257W	YFR002W	961
YOR257W	YGL086W	932
YOR257W	YGL092W	961
YOR257W	YGL100W	961
YOR257W	YGL172W	961
YOR257W	YGR119C	961
YOR257W	YHR102W	988
YOR257W	YIL115C	961
YOR257W	YJL019W	979
YOR257W	YJL039C	961
YOR257W	YJL041W	961
YOR257W	YJL061W	961
YOR257W	YJR042W	961
YOR257W	YKL057C	961
YOR257W	YKL068W	961
YOR257W	YKR082W	961
YOR257W	YLL003W	999
YOR257W	YLR018C	961
YOR257W	YLR335W	961
YOR257W	YML031W	961
YOR257W	YML103C	961
YOR257W	YMR047C	985
YOR257W	YMR129W	961
YOR257W	YMR153W	961
YOR257W	YNL188W	999
YOR257W	YOL072W	999
YOR257W	YOR098C	961
YOR259C	YBL041W	920
YOR259C	YCR014C	970
YOR259C	YDL007W	999
YOR259C	YDL013W	970
YOR259C	YDL097C	999
YOR259C	YDL147W	999
YOR259C	YDR108W	970
YOR259C	YDR363W-A	999
YOR259C	YDR394W	999

YOR259C	YDR427W	999
YOR259C	YEL037C	998
YOR259C	YER012W	999
YOR259C	YER021W	999
YOR259C	YER094C	913
YOR259C	YFL007W	973
YOR259C	YFR004W	999
YOR259C	YFR010W	999
YOR259C	YFR050C	910
YOR259C	YFR052W	999
YOR259C	YGL048C	999
YOR259C	YGL141W	954
YOR259C	YGR232W	998
YOR259C	YGR253C	913
YOR259C	YHL030W	998
YOR259C	YHR027C	999
YOR259C	YHR200W	999
YOR259C	YIL007C	911
YOR259C	YIL075C	998
YOR259C	YKL010C	908
YOR259C	YKL145W	999
YOR259C	YLR421C	999
YOR259C	YML092C	900
YOR259C	YMR191W	987
YOR259C	YMR314W	997
YOR259C	YOL038W	914
YOR259C	YOR117W	999
YOR259C	YOR157C	921
YOR259C	YOR261C	999
YOR259C	YOR362C	919
YOR259C	YPL248C	996
YOR259C	YPR103W	923
YOR259C	YPR108W	999
YOR260W	YDR211W	999
YOR260W	YEL009C	990
YOR260W	YER025W	999
YOR260W	YGR083C	999
YOR260W	YJR007W	999
YOR260W	YKR026C	999
YOR260W	YLR291C	999
YOR260W	YNL265C	987
YOR260W	YPL237W	999
YOR261C	YBL041W	910
YOR261C	YBR272C	988

YOR261C	YDL007W	999
YOR261C	YDL097C	999
YOR261C	YDL147W	999
YOR261C	YDR363W-A	999
YOR261C	YDR394W	999
YOR261C	YDR427W	999
YOR261C	YEL037C	981
YOR261C	YER012W	999
YOR261C	YER021W	999
YOR261C	YER094C	928
YOR261C	YFL007W	973
YOR261C	YFR004W	999
YOR261C	YFR010W	999
YOR261C	YFR050C	992
YOR261C	YFR052W	999
YOR261C	YGL048C	999
YOR261C	YGL141W	954
YOR261C	YGR135W	990
YOR261C	YGR232W	999
YOR261C	YGR253C	906
YOR261C	YHL030W	999
YOR261C	YHR027C	999
YOR261C	YHR200W	999
YOR261C	YIL075C	999
YOR261C	YKL145W	999
YOR261C	YLL039C	970
YOR261C	YLR421C	999
YOR261C	YML092C	902
YOR261C	YMR191W	987
YOR261C	YMR314W	999
YOR261C	YOL038W	977
YOR261C	YOR117W	999
YOR261C	YOR259C	999
YOR261C	YPR103W	906
YOR261C	YPR108W	999
YOR262W	YJR072C	947
YOR262W	YNL014W	998
YOR264W	YCR086W	984
YOR265W	YEL003W	913
YOR265W	YER007W	999
YOR265W	YFL037W	999
YOR265W	YGR078C	997
YOR265W	YLR200W	915
YOR265W	YML085C	999

YOR265W	YML094W	916
YOR265W	YML124C	990
YOR265W	YNL148C	949
YOR265W	YOR349W	999
YOR266W	YGR062C	978
YOR267C	YDL047W	920
YOR267C	YJL098W	987
YOR269W	YCL029C	947
YOR269W	YEL061C	909
YOR269W	YER016W	990
YOR269W	YGL216W	931
YOR269W	YLR254C	999
YOR269W	YNL271C	965
YOR269W	YPL269W	995
YOR270C	YBR127C	999
YOR270C	YDL185W	999
YOR270C	YEL027W	999
YOR270C	YEL051W	999
YOR270C	YGR020C	999
YOR270C	YGR105W	984
YOR270C	YHR026W	959
YOR270C	YHR039C-A	999
YOR270C	YHR060W	992
YOR270C	YKL080W	999
YOR270C	YKL119C	993
YOR270C	YLR447C	999
YOR270C	YMR054W	995
YOR270C	YMR123W	933
YOR270C	YOR332W	999
YOR270C	YPL037C	938
YOR270C	YPL234C	967
YOR270C	YPR036W	999
YOR270C	YPR149W	985
YOR272W	YBR142W	999
YOR272W	YDL031W	992
YOR272W	YDL082W	940
YOR272W	YDR060W	999
YOR272W	YDR087C	998
YOR272W	YDR496C	993
YOR272W	YER006W	998
YOR272W	YFL002C	993
YOR272W	YGL111W	999
YOR272W	YGR103W	999
YOR272W	YHR052W	999

YOR272W	YHR088W	997
YOR272W	YHR197W	992
YOR272W	YKL009W	997
YOR272W	YKL021C	957
YOR272W	YKL172W	997
YOR272W	YKR081C	999
YOR272W	YLL008W	999
YOR272W	YLL034C	994
YOR272W	YLL045C	940
YOR272W	YLR002C	999
YOR272W	YMR049C	999
YOR272W	YMR229C	991
YOR272W	YMR290C	999
YOR272W	YNL002C	999
YOR272W	YNL061W	999
YOR272W	YNL110C	999
YOR272W	YOL041C	979
YOR272W	YOL077C	999
YOR272W	YOR063W	933
YOR272W	YOR206W	998
YOR272W	YPL043W	999
YOR272W	YPL093W	998
YOR272W	YPL131W	961
YOR272W	YPL198W	937
YOR272W	YPL211W	999
YOR272W	YPR016C	985
YOR275C	YHL027W	995
YOR275C	YJR102C	916
YOR275C	YLR025W	999
YOR275C	YMR077C	930
YOR275C	YPL065W	908
YOR275C	YPR173C	999
YOR276W	YDL160C	985
YOR276W	YGL049C	938
YOR276W	YGR162W	999
YOR276W	YHR084W	981
YOR276W	YIR003W	962
YOR276W	YKL007W	962
YOR276W	YOL139C	999
YOR278W	YDL205C	995
YOR278W	YDR047W	904
YOR278W	YGL040C	961
YOR278W	YGR060W	975
YOR279C	YDR310C	999

YOR279C	YOL068C	988
YOR281C	YDL143W	999
YOR281C	YDR188W	995
YOR281C	YGR187C	913
YOR281C	YIL142W	999
YOR281C	YJL111W	999
YOR281C	YJL152W	998
YOR281C	YOR212W	950
YOR283W	YOR229W	901
YOR284W	YCR088W	991
YOR284W	YML103C	935
YOR288C	YCL043C	976
YOR290C	YBR009C	997
YOR290C	YBR089C-A	938
YOR290C	YBR112C	945
YOR290C	YBR289W	999
YOR290C	YDR073W	999
YOR290C	YDR146C	996
YOR290C	YDR448W	976
YOR290C	YEL009C	999
YOR290C	YFL049W	999
YOR290C	YFR034C	975
YOR290C	YFR037C	976
YOR290C	YGL043W	912
YOR290C	YGR116W	990
YOR290C	YGR252W	999
YOR290C	YGR275W	990
YOR290C	YHL025W	999
YOR290C	YJL176C	999
YOR290C	YKL109W	967
YOR290C	YMR033W	999
YOR290C	YNL207W	927
YOR290C	YNR023W	999
YOR290C	YOL051W	958
YOR290C	YOL148C	948
YOR290C	YPL016W	999
YOR290C	YPL129W	993
YOR290C	YPL240C	926
YOR290C	YPR034W	999
YOR290C	YPR052C	988
YOR291W	YOR254C	921
YOR293W	YAL035W	912
YOR293W	YBL027W	918
YOR293W	YBL072C	929

YOR293W	YBL087C	925
YOR293W	YBL092W	956
YOR293W	YBR031W	951
YOR293W	YBR048W	926
YOR293W	YBR079C	914
YOR293W	YBR084C-A	933
YOR293W	YBR118W	909
YOR293W	YBR181C	911
YOR293W	YBR189W	985
YOR293W	YBR191W	985
YOR293W	YCR031C	966
YOR293W	YDL061C	968
YOR293W	YDL075W	966
YOR293W	YDL081C	935
YOR293W	YDL082W	941
YOR293W	YDL130W	924
YOR293W	YDL136W	957
YOR293W	YDL191W	929
YOR293W	YDR012W	959
YOR293W	YDR025W	918
YOR293W	YDR064W	987
YOR293W	YDR382W	967
YOR293W	YDR385W	910
YOR293W	YDR418W	989
YOR293W	YDR429C	901
YOR293W	YDR447C	932
YOR293W	YDR450W	917
YOR293W	YDR471W	990
YOR293W	YDR500C	936
YOR293W	YEL054C	970
YOR293W	YER025W	936
YOR293W	YER056C-A	970
YOR293W	YER074W	920
YOR293W	YER102W	944
YOR293W	YER117W	942
YOR293W	YER131W	975
YOR293W	YER165W	917
YOR293W	YFL034C-A	926
YOR293W	YFR031C-A	905
YOR293W	YFR032C-A	941
YOR293W	YGL030W	983
YOR293W	YGL031C	970
YOR293W	YGL076C	960
YOR293W	YGL103W	994

YOR293W	YGL123W	963
YOR293W	YGL135W	926
YOR293W	YGL147C	936
YOR293W	YGL189C	925
YOR293W	YGR027C	979
YOR293W	YGR034W	936
YOR293W	YGR085C	972
YOR293W	YGR118W	984
YOR293W	YGR148C	980
YOR293W	YGR162W	910
YOR293W	YGR214W	991
YOR293W	YHL001W	976
YOR293W	YHL015W	979
YOR293W	YHL033C	971
YOR293W	YHR010W	980
YOR293W	YHR021C	931
YOR293W	YHR141C	946
YOR293W	YHR203C	943
YOR293W	YIL018W	905
YOR293W	YIL052C	991
YOR293W	YIL069C	920
YOR293W	YIL133C	928
YOR293W	YIL148W	940
YOR293W	YJL136C	959
YOR293W	YJL177W	972
YOR293W	YJL189W	944
YOR293W	YJL190C	938
YOR293W	YJL191W	963
YOR293W	YJR094W-A	978
YOR293W	YJR123W	986
YOR293W	YJR145C	928
YOR293W	YKL006W	994
YOR293W	YKL156W	982
YOR293W	YKL180W	979
YOR293W	YKR057W	938
YOR293W	YKR059W	918
YOR293W	YKR094C	905
YOR293W	YLL045C	942
YOR293W	YLR029C	975
YOR293W	YLR048W	978
YOR293W	YLR061W	975
YOR293W	YLR075W	958
YOR293W	YLR167W	925
YOR293W	YLR185W	933

YOR293W	YLR264W	994
YOR293W	YLR287C-A	906
YOR293W	YLR325C	931
YOR293W	YLR333C	945
YOR293W	YLR340W	957
YOR293W	YLR344W	966
YOR293W	YLR367W	982
YOR293W	YLR388W	981
YOR293W	YLR406C	924
YOR293W	YLR441C	930
YOR293W	YLR448W	980
YOR293W	YML024W	985
YOR293W	YML026C	917
YOR293W	YML063W	996
YOR293W	YML073C	929
YOR293W	YMR121C	928
YOR293W	YMR142C	943
YOR293W	YMR194W	980
YOR293W	YMR230W	993
YOR293W	YMR242C	941
YOR293W	YMR260C	915
YOR293W	YMR309C	909
YOR293W	YNL067W	989
YOR293W	YNL069C	927
YOR293W	YNL096C	935
YOR293W	YNL162W	931
YOR293W	YNL178W	984
YOR293W	YNL301C	939
YOR293W	YNL302C	927
YOR293W	YOL039W	923
YOR293W	YOL040C	949
YOR293W	YOL120C	921
YOR293W	YOL121C	927
YOR293W	YOL127W	996
YOR293W	YOR063W	974
YOR293W	YOR096W	988
YOR293W	YOR133W	910
YOR293W	YOR167C	976
YOR293W	YOR182C	906
YOR293W	YOR234C	995
YOR293W	YOR312C	945
YOR293W	YOR361C	940
YOR293W	YOR369C	952
YOR293W	YPL079W	974

YOR293W	YPL081W	963
YOR293W	YPL090C	902
YOR293W	YPL131W	952
YOR293W	YPL143W	983
YOR293W	YPL198W	936
YOR293W	YPL220W	941
YOR293W	YPL237W	929
YOR293W	YPL249C-A	940
YOR293W	YPR043W	940
YOR293W	YPR080W	909
YOR293W	YPR102C	998
YOR293W	YPR132W	947
YOR293W	YPR163C	915
YOR294W	YDR381W	930
YOR294W	YER002W	998
YOR294W	YGL029W	925
YOR294W	YGR085C	996
YOR294W	YHL033C	984
YOR294W	YKL172W	999
YOR294W	YKR081C	999
YOR294W	YLR002C	996
YOR294W	YLR029C	985
YOR294W	YMR290C	982
YOR294W	YNL061W	987
YOR294W	YPL131W	999
YOR294W	YPR102C	996
YOR295W	YBL025W	953
YOR295W	YLR141W	992
YOR295W	YMR270C	963
YOR297C	YBR091C	992
YOR297C	YDL217C	999
YOR297C	YDR188W	987
YOR297C	YEL020W-A	932
YOR297C	YGR178C	924
YOR297C	YHR005C-A	998
YOR297C	YJL054W	999
YOR297C	YMR195W	940
YOR297C	YPR040W	924
YOR298C-A	YEL009C	910
YOR298C-A	YER148W	978
YOR298W	YOR386W	998
YOR299W	YGR041W	910
YOR299W	YJL099W	999
YOR299W	YJL207C	980

YOR299W	YKR027W	985
YOR299W	YLR330W	999
YOR299W	YLR353W	903
YOR299W	YMR237W	981
YOR301W	YGR041W	991
YOR301W	YHR005C	944
YOR301W	YLR353W	992
YOR301W	YPR122W	915
YOR302W	YOL082W	976
YOR303W	YDL171C	925
YOR303W	YJL088W	986
YOR303W	YJL130C	993
YOR303W	YJR109C	999
YOR303W	YKL104C	922
YOR303W	YMR217W	982
YOR303W	YMR300C	956
YOR303W	YOL058W	908
YOR304W	YBR009C	982
YOR304W	YBR245C	999
YOR304W	YDL002C	990
YOR304W	YDR121W	999
YOR304W	YDR190C	970
YOR304W	YDR207C	935
YOR304W	YDR224C	975
YOR304W	YER092W	942
YOR304W	YER164W	994
YOR304W	YFR013W	912
YOR304W	YFR037C	972
YOR304W	YGL133W	999
YOR304W	YIL126W	976
YOR304W	YJL065C	999
YOR304W	YKR001C	970
YOR304W	YNL030W	976
YOR304W	YNL215W	939
YOR304W	YNL330C	986
YOR304W	YOR116C	968
YOR304W	YPL082C	974
YOR307C	YFL038C	985
YOR307C	YLR268W	923
YOR307C	YNL287W	906
YOR308C	YBL026W	996
YOR308C	YBL074C	990
YOR308C	YBR055C	998
YOR308C	YBR152W	999

YOR308C	YCR077C	977
YOR308C	YDL030W	976
YOR308C	YDL098C	989
YOR308C	YDL160C	972
YOR308C	YDR283C	978
YOR308C	YDR378C	997
YOR308C	YDR473C	997
YOR308C	YER029C	989
YOR308C	YER112W	993
YOR308C	YER172C	999
YOR308C	YFL017W-A	939
YOR308C	YGR074W	989
YOR308C	YGR075C	972
YOR308C	YGR091W	999
YOR308C	YHR165C	999
YOR308C	YJL124C	976
YOR308C	YJL203W	974
YOR308C	YJR022W	998
YOR308C	YKL173W	998
YOR308C	YLR147C	998
YOR308C	YLR275W	989
YOR308C	YLR418C	913
YOR308C	YML049C	978
YOR308C	YMR240C	976
YOR308C	YNL147W	999
YOR308C	YNR032C-A	986
YOR308C	YPL213W	977
YOR308C	YPR082C	989
YOR308C	YPR178W	999
YOR309C	YLR175W	998
YOR310C	YCL054W	927
YOR310C	YCR057C	994
YOR310C	YCR060W	921
YOR310C	YDL014W	999
YOR310C	YDL148C	999
YOR310C	YDL153C	923
YOR310C	YDR064W	993
YOR310C	YDR324C	984
YOR310C	YDR398W	922
YOR310C	YDR432W	940
YOR310C	YDR449C	987
YOR310C	YEL026W	999
YOR310C	YER011W	923
YOR310C	YER082C	908

YOR310C	YER165W	939
YOR310C	YGL120C	999
YOR310C	YGR128C	987
YOR310C	YGR159C	948
YOR310C	YHR034C	976
YOR310C	YHR052W	969
YOR310C	YHR148W	974
YOR310C	YHR196W	985
YOR310C	YHR203C	981
YOR310C	YJL010C	913
YOR310C	YJL033W	951
YOR310C	YJL109C	986
YOR310C	YJR002W	999
YOR310C	YKL099C	908
YOR310C	YKR060W	907
YOR310C	YLL011W	961
YOR310C	YLR129W	981
YOR310C	YLR175W	995
YOR310C	YLR186W	998
YOR310C	YLR197W	999
YOR310C	YLR222C	985
YOR310C	YLR347C	923
YOR310C	YLR409C	957
YOR310C	YML093W	923
YOR310C	YMR093W	987
YOR310C	YMR128W	979
YOR310C	YMR229C	989
YOR310C	YMR290C	901
YOR310C	YNL075W	938
YOR310C	YNL132W	997
YOR310C	YNL189W	965
YOR310C	YNL248C	988
YOR310C	YOR078W	985
YOR310C	YOR096W	993
YOR310C	YOR204W	923
YOR310C	YPL126W	999
YOR310C	YPL235W	976
YOR310C	YPR137W	999
YOR311C	YMR165C	928
YOR312C	YBL027W	996
YOR312C	YBL072C	997
YOR312C	YBL087C	932
YOR312C	YBL092W	940
YOR312C	YBR031W	997

YOR312C	YBR048W	997
YOR312C	YBR084C-A	946
YOR312C	YBR118W	916
YOR312C	YBR181C	995
YOR312C	YBR189W	997
YOR312C	YBR191W	946
YOR312C	YCR031C	997
YOR312C	YDL061C	922
YOR312C	YDL075W	996
YOR312C	YDL081C	939
YOR312C	YDL082W	996
YOR312C	YDL130W	906
YOR312C	YDL136W	998
YOR312C	YDL191W	922
YOR312C	YDR012W	944
YOR312C	YDR025W	996
YOR312C	YDR064W	998
YOR312C	YDR101C	940
YOR312C	YDR382W	996
YOR312C	YDR385W	917
YOR312C	YDR418W	996
YOR312C	YDR447C	996
YOR312C	YDR450W	925
YOR312C	YDR471W	996
YOR312C	YDR500C	944
YOR312C	YEL054C	933
YOR312C	YER056C-A	923
YOR312C	YER074W	995
YOR312C	YER102W	998
YOR312C	YER117W	957
YOR312C	YER131W	930
YOR312C	YFL034C-A	914
YOR312C	YFR031C-A	996
YOR312C	YFR032C-A	939
YOR312C	YGL030W	948
YOR312C	YGL031C	910
YOR312C	YGL076C	997
YOR312C	YGL103W	996
YOR312C	YGL123W	945
YOR312C	YGL135W	996
YOR312C	YGL147C	929
YOR312C	YGL189C	920
YOR312C	YGR027C	941
YOR312C	YGR034W	996

YOR312C	YGR085C	996
YOR312C	YGR090W	976
YOR312C	YGR118W	945
YOR312C	YGR148C	921
YOR312C	YGR214W	939
YOR312C	YHL001W	996
YOR312C	YHL015W	949
YOR312C	YHL033C	996
YOR312C	YHL034C	995
YOR312C	YHR010W	998
YOR312C	YHR021C	931
YOR312C	YHR141C	929
YOR312C	YHR203C	997
YOR312C	YIL018W	996
YOR312C	YIL052C	928
YOR312C	YIL069C	995
YOR312C	YIL133C	996
YOR312C	YIL148W	958
YOR312C	YJL136C	934
YOR312C	YJL177W	997
YOR312C	YJL189W	911
YOR312C	YJL190C	996
YOR312C	YJL191W	936
YOR312C	YJR094W-A	944
YOR312C	YJR123W	998
YOR312C	YJR145C	997
YOR312C	YKL006W	943
YOR312C	YKL156W	953
YOR312C	YKL180W	996
YOR312C	YKR057W	934
YOR312C	YKR094C	910
YOR312C	YLL045C	997
YOR312C	YLR029C	997
YOR312C	YLR048W	941
YOR312C	YLR061W	932
YOR312C	YLR075W	996
YOR312C	YLR150W	983
YOR312C	YLR167W	947
YOR312C	YLR185W	926
YOR312C	YLR264W	929
YOR312C	YLR287C-A	903
YOR312C	YLR325C	914
YOR312C	YLR333C	927
YOR312C	YLR340W	952

YOR312C	YLR344W	938
YOR312C	YLR367W	998
YOR312C	YLR388W	929
YOR312C	YLR406C	923
YOR312C	YLR441C	996
YOR312C	YLR448W	997
YOR312C	YML024W	946
YOR312C	YML026C	925
YOR312C	YML063W	997
YOR312C	YML073C	997
YOR312C	YMR121C	927
YOR312C	YMR142C	929
YOR312C	YMR194W	997
YOR312C	YMR230W	934
YOR312C	YMR242C	995
YOR312C	YMR260C	910
YOR312C	YMR290C	937
YOR312C	YNL067W	946
YOR312C	YNL069C	996
YOR312C	YNL096C	996
YOR312C	YNL132W	933
YOR312C	YNL162W	929
YOR312C	YNL178W	998
YOR312C	YNL301C	998
YOR312C	YNL302C	927
YOR312C	YOL039W	913
YOR312C	YOL040C	952
YOR312C	YOL041C	946
YOR312C	YOL077C	932
YOR312C	YOL120C	935
YOR312C	YOL121C	927
YOR312C	YOL127W	997
YOR312C	YOR063W	996
YOR312C	YOR096W	997
YOR312C	YOR133W	927
YOR312C	YOR167C	920
YOR312C	YOR182C	903
YOR312C	YOR234C	946
YOR312C	YOR293W	945
YOR312C	YOR369C	941
YOR312C	YPL079W	931
YOR312C	YPL081W	915
YOR312C	YPL090C	997
YOR312C	YPL131W	934

YOR312C	YPL143W	941
YOR312C	YPL198W	997
YOR312C	YPL220W	998
YOR312C	YPL249C-A	930
YOR312C	YPR043W	944
YOR312C	YPR080W	916
YOR312C	YPR102C	935
YOR312C	YPR132W	955
YOR316C	YIL023C	904
YOR316C	YMR243C	985
YOR317W	YBR041W	974
YOR317W	YGL205W	926
YOR317W	YLR195C	950
YOR317W	YMR246W	999
YOR317W	YNL123W	981
YOR319W	YBR055C	920
YOR319W	YDL030W	999
YOR319W	YDL043C	992
YOR319W	YER029C	973
YOR319W	YER172C	968
YOR319W	YHR165C	921
YOR319W	YIR005W	947
YOR319W	YJL203W	998
YOR319W	YJR022W	943
YOR319W	YML049C	999
YOR319W	YMR240C	999
YOR319W	YMR288W	999
YOR319W	YNL138W-A	994
YOR319W	YPL178W	915
YOR319W	YPL213W	935
YOR319W	YPR094W	949
YOR321W	YAL023C	927
YOR321W	YDL095W	979
YOR321W	YJR143C	903
YOR322C	YER125W	979
YOR323C	YCR079W	999
YOR323C	YDR300C	999
YOR323C	YER023W	985
YOR323C	YHR033W	983
YOR323C	YJL101C	976
YOR323C	YNL021W	927
YOR323C	YOL090W	936
YOR324C	YLR433C	935
YOR326W	YAL029C	999

YOR326W	YBR109C	999
YOR326W	YCL063W	999
YOR326W	YDL111C	966
YOR326W	YDR103W	971
YOR326W	YDR166C	987
YOR326W	YDR388W	976
YOR326W	YER155C	932
YOR326W	YFL005W	999
YOR326W	YFL039C	999
YOR326W	YGL106W	999
YOR326W	YGL233W	984
YOR326W	YGR009C	990
YOR326W	YGR195W	965
YOR326W	YHR023W	999
YOR326W	YHR030C	915
YOR326W	YIL070C	924
YOR326W	YIL118W	985
YOR326W	YJR132W	902
YOR326W	YKL079W	999
YOR326W	YKL130C	948
YOR326W	YLR166C	984
YOR326W	YLR190W	916
YOR326W	YMR163C	978
YOR326W	YNL079C	987
YOR326W	YNL272C	996
YOR326W	YNL298W	981
YOR326W	YNL304W	995
YOR326W	YOR001W	964
YOR326W	YOR035C	999
YOR326W	YPL269W	999
YOR326W	YPR032W	984
YOR326W	YPR055W	987
YOR327C	YAL030W	999
YOR327C	YDR164C	999
YOR327C	YDR189W	998
YOR327C	YDR468C	999
YOR327C	YER143W	995
YOR327C	YGR009C	999
YOR327C	YIL004C	998
YOR327C	YKL006C-A	998
YOR327C	YKL196C	999
YOR327C	YLR026C	999
YOR327C	YLR078C	998
YOR327C	YLR268W	998

YOR327C	YMR017W	990
YOR327C	YMR183C	999
YOR327C	YMR197C	999
YOR327C	YOL018C	998
YOR327C	YPL232W	999
YOR327C	YPL240C	919
YOR328W	YBL005W	925
YOR328W	YDR011W	909
YOR328W	YDR406W	903
YOR328W	YGL013C	982
YOR328W	YGR281W	922
YOR328W	YIL013C	910
YOR328W	YOR153W	903
YOR329C	YCR088W	904
YOR329C	YER133W	999
YOR329C	YGL206C	985
YOR329C	YIL095W	991
YOR329C	YIR006C	969
YOR329C	YNL084C	969
YOR329C	YNL243W	908
YOR329C	YPL209C	931
YOR330C	YER070W	976
YOR330C	YML058W	976
YOR332W	YBR011C	900
YOR332W	YBR127C	996
YOR332W	YDL185W	999
YOR332W	YDR202C	960
YOR332W	YEL027W	946
YOR332W	YEL051W	999
YOR332W	YGR020C	999
YOR332W	YHR026W	957
YOR332W	YHR039C-A	999
YOR332W	YJR033C	999
YOR332W	YKL080W	999
YOR332W	YLR447C	950
YOR332W	YMR054W	999
YOR332W	YMR186W	931
YOR332W	YOR270C	999
YOR332W	YPL234C	953
YOR332W	YPL240C	970
YOR332W	YPR036W	999
YOR333C	YER063W	998
YOR334W	YJL133W	985
YOR334W	YKR052C	985

YOR334W	YOL130W	913
YOR335C	YOR101W	935
YOR336W	YCL004W	942
YOR336W	YPR159W	951
YOR339C	YBR082C	906
YOR339C	YDR059C	904
YOR339C	YKL022C	902
YOR339C	YKL210W	956
YOR339C	YLL039C	903
YOR340C	YBR154C	999
YOR340C	YDL150W	921
YOR340C	YDR045C	921
YOR340C	YDR156W	999
YOR340C	YFR028C	942
YOR340C	YHR143W-A	999
YOR340C	YJL148W	999
YOR340C	YJR063W	999
YOR340C	YKL125W	987
YOR340C	YKL144C	937
YOR340C	YNL113W	999
YOR340C	YNL151C	921
YOR340C	YNL248C	999
YOR340C	YNR003C	921
YOR340C	YOR116C	921
YOR340C	YOR207C	921
YOR340C	YOR210W	999
YOR340C	YOR224C	999
YOR340C	YOR341W	999
YOR340C	YPR010C	999
YOR340C	YPR110C	999
YOR340C	YPR187W	999
YOR340C	YPR190C	921
YOR341W	YBR154C	999
YOR341W	YDL150W	921
YOR341W	YDR045C	929
YOR341W	YDR156W	999
YOR341W	YER171W	900
YOR341W	YFR028C	905
YOR341W	YHR143W-A	960
YOR341W	YIL143C	900
YOR341W	YJL076W	954
YOR341W	YJL148W	999
YOR341W	YJR063W	999
YOR341W	YKL125W	984

YOR341W	YKL144C	921
YOR341W	YKL213C	987
YOR341W	YLL034C	954
YOR341W	YML010W	942
YOR341W	YNL113W	999
YOR341W	YNL151C	921
YOR341W	YNL248C	999
YOR341W	YNR003C	921
YOR341W	YOR116C	938
YOR341W	YOR207C	924
YOR341W	YOR210W	999
YOR341W	YOR224C	999
YOR341W	YOR340C	999
YOR341W	YPR010C	999
YOR341W	YPR110C	999
YOR341W	YPR187W	999
YOR341W	YPR190C	921
YOR344C	YOL108C	977
YOR346W	YCR066W	928
YOR346W	YDR419W	999
YOR346W	YGL058W	925
YOR346W	YIL139C	999
YOR346W	YMR201C	985
YOR346W	YOR251C	998
YOR346W	YPL167C	999
YOR347C	YAL038W	921
YOR347C	YBR218C	915
YOR347C	YBR221C	909
YOR347C	YDR050C	930
YOR347C	YDR226W	905
YOR347C	YDR454C	900
YOR347C	YER170W	905
YOR347C	YER178W	905
YOR347C	YGL062W	915
YOR347C	YGR254W	931
YOR347C	YHR174W	930
YOR347C	YKL029C	975
YOR347C	YKR097W	938
YOR347C	YMR323W	929
YOR347C	YOR393W	929
YOR348C	YDL210W	950
YOR348C	YER040W	904
YOR348C	YJL084C	944
YOR349W	YAL011W	920

YOR349W	YBR231C	908
YOR349W	YCL029C	965
YOR349W	YDR318W	926
YOR349W	YDR334W	951
YOR349W	YDR485C	947
YOR349W	YEL061C	987
YOR349W	YER007W	999
YOR349W	YFL037W	999
YOR349W	YGL086W	946
YOR349W	YJL013C	964
YOR349W	YJL030W	995
YOR349W	YLR085C	939
YOR349W	YML041C	945
YOR349W	YML085C	994
YOR349W	YML124C	998
YOR349W	YMR138W	999
YOR349W	YNL148C	993
YOR349W	YOL012C	919
YOR349W	YOR265W	999
YOR349W	YPL241C	987
YOR351C	YDR285W	985
YOR351C	YER133W	920
YOR351C	YER179W	962
YOR351C	YGL033W	959
YOR351C	YGL249W	933
YOR351C	YHR014W	998
YOR351C	YIL072W	999
YOR351C	YLR263W	999
YOR352W	YIL143C	998
YOR352W	YOR168W	925
YOR353C	YHR102W	999
YOR353C	YKL189W	997
YOR353C	YNL161W	930
YOR355W	YJL092W	954
YOR356W	YGR207C	997
YOR356W	YOR243C	906
YOR356W	YPR004C	998
YOR357C	YER145C	924
YOR357C	YOR219C	902
YOR358W	YBL021C	999
YOR358W	YGL237C	999
YOR358W	YKL109W	999
YOR358W	YOR047C	972
YOR359W	YMR197C	903

YOR360C	YDL055C	909
YOR360C	YDR477W	995
YOR360C	YGL248W	999
YOR360C	YJL005W	999
YOR360C	YJL095W	913
YOR360C	YKL022C	973
YOR360C	YNL098C	998
YOR360C	YOR101W	929
YOR361C	YAL035W	999
YOR361C	YBL072C	997
YOR361C	YBR079C	999
YOR361C	YBR181C	908
YOR361C	YBR189W	934
YOR361C	YCR031C	920
YOR361C	YDL061C	914
YOR361C	YDR025W	997
YOR361C	YDR064W	932
YOR361C	YDR429C	999
YOR361C	YER025W	976
YOR361C	YER131W	915
YOR361C	YER165W	928
YOR361C	YGL123W	916
YOR361C	YGR027C	914
YOR361C	YGR118W	914
YOR361C	YGR162W	942
YOR361C	YGR214W	955
YOR361C	YHL015W	911
YOR361C	YIL071C	915
YOR361C	YJL190C	915
YOR361C	YJL191W	921
YOR361C	YJR007W	961
YOR361C	YJR123W	935
YOR361C	YKL156W	915
YOR361C	YLR048W	931
YOR361C	YLR192C	999
YOR361C	YLR264W	933
YOR361C	YLR347C	923
YOR361C	YLR367W	913
YOR361C	YLR388W	914
YOR361C	YML024W	915
YOR361C	YML063W	928
YOR361C	YMR080C	919
YOR361C	YMR146C	999
YOR361C	YMR230W	912

YOR361C	YMR260C	985
YOR361C	YMR309C	999
YOR361C	YNL062C	995
YOR361C	YNL096C	914
YOR361C	YNL178W	911
YOR361C	YNL244C	999
YOR361C	YOL121C	937
YOR361C	YOL139C	936
YOR361C	YOR096W	919
YOR361C	YOR167C	914
YOR361C	YOR204W	976
YOR361C	YOR293W	940
YOR361C	YPL001W	957
YOR361C	YPL081W	923
YOR361C	YPL090C	908
YOR361C	YPL237W	998
YOR361C	YPR041W	999
YOR361C	YPR086W	955
YOR361C	YPR163C	916
YOR362C	YBL041W	999
YOR362C	YBR173C	917
YOR362C	YDL097C	928
YOR362C	YDL147W	931
YOR362C	YDR394W	909
YOR362C	YDR427W	918
YOR362C	YER012W	999
YOR362C	YER021W	959
YOR362C	YER094C	999
YOR362C	YFL007W	991
YOR362C	YFL017C	943
YOR362C	YFR004W	956
YOR362C	YFR050C	999
YOR362C	YFR052W	903
YOR362C	YGL011C	999
YOR362C	YGL048C	972
YOR362C	YGR135W	999
YOR362C	YGR253C	999
YOR362C	YHR027C	999
YOR362C	YHR200W	965
YOR362C	YIL007C	902
YOR362C	YJL001W	999
YOR362C	YLR076C	999
YOR362C	YML092C	999
YOR362C	YML109W	945

YOR362C	YMR154C	999
YOR362C	YMR314W	999
YOR362C	YNL244C	999
YOR362C	YOL038W	999
YOR362C	YOR157C	999
YOR362C	YOR259C	919
YOR362C	YPR103W	999
YOR362C	YPR108W	917
YOR363C	YAL051W	999
YOR363C	YDR216W	993
YOR363C	YNL202W	935
YOR363C	YOR122C	932
YOR367W	YDR129C	998
YOR367W	YFL039C	998
YOR368W	YAR007C	919
YOR368W	YCL061C	949
YOR368W	YDR217C	976
YOR368W	YER095W	916
YOR368W	YER179W	996
YOR368W	YJR043C	950
YOR368W	YKL113C	988
YOR368W	YLR288C	999
YOR368W	YML028W	924
YOR368W	YMR190C	994
YOR368W	YOR144C	923
YOR368W	YPL022W	976
YOR368W	YPL153C	991
YOR368W	YPL194W	999
YOR369C	YBL027W	915
YOR369C	YBL072C	923
YOR369C	YBL087C	980
YOR369C	YBL092W	938
YOR369C	YBR031W	947
YOR369C	YBR048W	932
YOR369C	YBR084C-A	915
YOR369C	YBR118W	911
YOR369C	YBR181C	953
YOR369C	YBR189W	950
YOR369C	YBR191W	928
YOR369C	YCR031C	942
YOR369C	YDL061C	925
YOR369C	YDL075W	910
YOR369C	YDL081C	968
YOR369C	YDL082W	959

YOR369C	YDL130W	920
YOR369C	YDL136W	937
YOR369C	YDL191W	937
YOR369C	YDR012W	940
YOR369C	YDR025W	932
YOR369C	YDR064W	975
YOR369C	YDR382W	935
YOR369C	YDR385W	901
YOR369C	YDR418W	952
YOR369C	YDR447C	971
YOR369C	YDR450W	914
YOR369C	YDR471W	915
YOR369C	YDR500C	929
YOR369C	YEL054C	946
YOR369C	YER025W	926
YOR369C	YER056C-A	912
YOR369C	YER074W	937
YOR369C	YER102W	923
YOR369C	YER117W	934
YOR369C	YER131W	952
YOR369C	YFL034C-A	918
YOR369C	YFR031C-A	967
YOR369C	YFR032C-A	936
YOR369C	YGL030W	964
YOR369C	YGL031C	956
YOR369C	YGL076C	966
YOR369C	YGL103W	909
YOR369C	YGL123W	952
YOR369C	YGL135W	925
YOR369C	YGL147C	979
YOR369C	YGL189C	942
YOR369C	YGR027C	948
YOR369C	YGR034W	959
YOR369C	YGR085C	925
YOR369C	YGR118W	940
YOR369C	YGR148C	940
YOR369C	YGR214W	937
YOR369C	YHL001W	949
YOR369C	YHL015W	917
YOR369C	YHL033C	933
YOR369C	YHR010W	925
YOR369C	YHR021C	934
YOR369C	YHR141C	954
YOR369C	YHR203C	923

YOR369C	YIL052C	912
YOR369C	YIL069C	937
YOR369C	YIL133C	974
YOR369C	YIL148W	900
YOR369C	YJL136C	935
YOR369C	YJL177W	912
YOR369C	YJL189W	900
YOR369C	YJL190C	985
YOR369C	YJL191W	942
YOR369C	YJR007W	902
YOR369C	YJR094W-A	939
YOR369C	YJR123W	970
YOR369C	YJR145C	923
YOR369C	YKL006W	949
YOR369C	YKL156W	934
YOR369C	YKL180W	920
YOR369C	YKR057W	935
YOR369C	YKR094C	900
YOR369C	YLL045C	965
YOR369C	YLR029C	951
YOR369C	YLR048W	926
YOR369C	YLR061W	922
YOR369C	YLR075W	953
YOR369C	YLR185W	930
YOR369C	YLR264W	905
YOR369C	YLR287C-A	909
YOR369C	YLR325C	987
YOR369C	YLR333C	953
YOR369C	YLR340W	941
YOR369C	YLR344W	921
YOR369C	YLR367W	943
YOR369C	YLR388W	925
YOR369C	YLR406C	910
YOR369C	YLR441C	972
YOR369C	YLR448W	935
YOR369C	YML024W	940
YOR369C	YML026C	914
YOR369C	YML063W	949
YOR369C	YML073C	942
YOR369C	YMR121C	961
YOR369C	YMR142C	945
YOR369C	YMR194W	936
YOR369C	YMR230W	953
YOR369C	YMR242C	983

YOR369C	YMR260C	930
YOR369C	YNL067W	933
YOR369C	YNL069C	938
YOR369C	YNL096C	965
YOR369C	YNL162W	932
YOR369C	YNL178W	970
YOR369C	YNL301C	920
YOR369C	YNL302C	941
YOR369C	YOL039W	915
YOR369C	YOL040C	971
YOR369C	YOL120C	920
YOR369C	YOL121C	972
YOR369C	YOL127W	949
YOR369C	YOL139C	920
YOR369C	YOR063W	949
YOR369C	YOR096W	951
YOR369C	YOR133W	901
YOR369C	YOR167C	938
YOR369C	YOR182C	909
YOR369C	YOR234C	938
YOR369C	YOR293W	952
YOR369C	YOR312C	941
YOR369C	YPL079W	951
YOR369C	YPL081W	945
YOR369C	YPL131W	939
YOR369C	YPL143W	939
YOR369C	YPL198W	945
YOR369C	YPL220W	925
YOR369C	YPL237W	907
YOR369C	YPL249C-A	936
YOR369C	YPR043W	939
YOR369C	YPR080W	911
YOR369C	YPR102C	950
YOR369C	YPR132W	940
YOR369C	YPR163C	914
YOR370C	YCL051W	918
YOR370C	YFL005W	929
YOR370C	YFL038C	999
YOR370C	YJL031C	920
YOR370C	YKL104C	906
YOR370C	YKR014C	998
YOR370C	YLR262C	912
YOR370C	YLR403W	967
YOR370C	YNL093W	940

YOR370C	YOR089C	974
YOR370C	YPR176C	996
YOR371C	YAL056W	971
YOR371C	YBR140C	963
YOR371C	YDL035C	905
YOR371C	YER020W	967
YOR371C	YGL121C	919
YOR371C	YJL005W	971
YOR371C	YOL081W	976
YOR372C	YBR160W	999
YOR372C	YBR245C	903
YOR372C	YIL131C	950
YOR372C	YMR001C	954
YOR372C	YNL068C	999
YOR372C	YPR119W	995
YOR373W	YAL047C	962
YOR373W	YDL239C	956
YOR373W	YDR356W	968
YOR373W	YMR001C	993
YOR373W	YMR055C	960
YOR373W	YNL225C	962
YOR373W	YOL091W	967
YOR374W	YAL054C	901
YOR374W	YBR145W	901
YOR374W	YDL080C	935
YOR374W	YDL168W	916
YOR374W	YER073W	904
YOR374W	YGL256W	966
YOR374W	YGR019W	900
YOR374W	YLR044C	924
YOR374W	YLR153C	954
YOR374W	YMR083W	901
YOR374W	YMR169C	909
YOR374W	YMR170C	909
YOR374W	YMR250W	910
YOR374W	YMR303C	965
YOR374W	YOL086C	901
YOR374W	YPL061W	970
YOR375C	YAL062W	999
YOR375C	YDL171C	991
YOR375C	YDL215C	986
YOR375C	YEL009C	928
YOR375C	YER040W	932
YOR375C	YFR055W	906

YOR375C	YGR124W	903
YOR375C	YHR037W	924
YOR375C	YJL124C	961
YOR375C	YLR451W	930
YOR375C	YMR250W	905
YOR375C	YOL033W	914
YOR375C	YPR035W	996
YOR375C	YPR145W	903
YOR380W	YOR153W	910
YOR381W	YGL071W	963
YOR384W	YGL071W	945
YOR386W	YOR298W	998
YOR387C	YGL258W	999
YOR388C	YBL039C	916
YOR388C	YJL068C	985
YOR393W	YAL038W	929
YOR393W	YDR050C	914
YOR393W	YGR254W	902
YOR393W	YHR174W	902
YOR393W	YKL152C	907
YOR393W	YMR323W	901
YOR393W	YOR347C	929
YOR393W	YPL281C	901
YPL001W	YBL023C	999
YPL001W	YBR009C	999
YPL001W	YBR060C	999
YPL001W	YBR079C	955
YPL001W	YBR272C	983
YPL001W	YEL056W	999
YPL001W	YHR118C	999
YPL001W	YLL004W	999
YPL001W	YLL022C	999
YPL001W	YML065W	999
YPL001W	YMR309C	935
YPL001W	YNL030W	999
YPL001W	YNL031C	999
YPL001W	YNL261W	999
YPL001W	YOL054W	988
YPL001W	YOR361C	957
YPL001W	YPR023C	940
YPL001W	YPR162C	999
YPL002C	YHL027W	910
YPL002C	YJR102C	999
YPL002C	YKL041W	996

YPL002C	YLR025W	980
YPL002C	YLR119W	932
YPL002C	YLR417W	999
YPL002C	YLR453C	905
YPL002C	YMR077C	998
YPL002C	YPL065W	998
YPL003W	YDL132W	946
YPL003W	YDR054C	901
YPL003W	YDR328C	901
YPL003W	YFL009W	905
YPL003W	YPR066W	999
YPL004C	YCR009C	968
YPL004C	YCR088W	968
YPL004C	YDR490C	999
YPL004C	YGL173C	988
YPL004C	YGR086C	999
YPL004C	YKL142W	999
YPL004C	YML052W	950
YPL004C	YOL100W	998
YPL007C	YAL001C	999
YPL007C	YBR123C	999
YPL007C	YDR362C	999
YPL007C	YER148W	939
YPL007C	YGR047C	999
YPL007C	YNL039W	955
YPL007C	YOR110W	999
YPL008W	YAL043C	929
YPL008W	YCL061C	902
YPL008W	YFR027W	977
YPL008W	YGL086W	965
YPL008W	YGR188C	927
YPL008W	YHR191C	999
YPL008W	YJL030W	966
YPL008W	YLR381W	922
YPL008W	YMR078C	979
YPL008W	YNL222W	929
YPL008W	YOR144C	962
YPL008W	YPR046W	922
YPL010W	YBR162C	962
YPL010W	YDL137W	905
YPL010W	YDL145C	999
YPL010W	YDL192W	942
YPL010W	YDR238C	999
YPL010W	YFR051C	999

YPL010W	YGL137W	999
YPL010W	YIL076W	999
YPL010W	YNL287W	999
YPL011C	YBR081C	988
YPL011C	YBR198C	999
YPL011C	YCR042C	999
YPL011C	YDR145W	999
YPL011C	YDR167W	999
YPL011C	YDR176W	988
YPL011C	YDR448W	994
YPL011C	YER148W	999
YPL011C	YGL066W	988
YPL011C	YGL112C	999
YPL011C	YGR274C	999
YPL011C	YML015C	999
YPL011C	YML098W	999
YPL011C	YML114C	999
YPL011C	YMR005W	999
YPL011C	YMR227C	999
YPL011C	YMR236W	999
YPL011C	YOR174W	974
YPL011C	YPL129W	998
YPL011C	YPL254W	988
YPL012W	YBL004W	994
YPL012W	YBL072C	942
YPL012W	YBR031W	941
YPL012W	YBR048W	931
YPL012W	YBR189W	931
YPL012W	YBR247C	999
YPL012W	YCL059C	967
YPL012W	YCR057C	972
YPL012W	YDL014W	995
YPL012W	YDL060W	999
YPL012W	YDL075W	929
YPL012W	YDL148C	998
YPL012W	YDR060W	997
YPL012W	YDR064W	936
YPL012W	YDR299W	922
YPL012W	YDR496C	939
YPL012W	YEL026W	909
YPL012W	YER082C	913
YPL012W	YER102W	929
YPL012W	YGL103W	943
YPL012W	YGL120C	964

YPL012W	YGL135W	940
YPL012W	YGL171W	916
YPL012W	YGR081C	999
YPL012W	YGR090W	994
YPL012W	YGR128C	998
YPL012W	YHR052W	951
YPL012W	YHR089C	969
YPL012W	YHR148W	912
YPL012W	YHR169W	918
YPL012W	YHR196W	972
YPL012W	YHR203C	931
YPL012W	YJL010C	983
YPL012W	YJL109C	998
YPL012W	YJR002W	996
YPL012W	YJR145C	970
YPL012W	YLL011W	920
YPL012W	YLL045C	935
YPL012W	YLR129W	998
YPL012W	YLR175W	999
YPL012W	YLR186W	995
YPL012W	YLR197W	995
YPL012W	YLR222C	924
YPL012W	YLR276C	982
YPL012W	YLR293C	907
YPL012W	YLR409C	915
YPL012W	YML063W	942
YPL012W	YML093W	911
YPL012W	YMR093W	999
YPL012W	YMR128W	997
YPL012W	YMR229C	998
YPL012W	YMR290C	995
YPL012W	YNL075W	923
YPL012W	YNL110C	922
YPL012W	YNL132W	995
YPL012W	YNL207W	949
YPL012W	YNL308C	982
YPL012W	YOL010W	910
YPL012W	YOL041C	980
YPL012W	YOL077C	941
YPL012W	YOR004W	924
YPL012W	YOR063W	947
YPL012W	YOR078W	972
YPL012W	YOR096W	936
YPL012W	YOR145C	997

YPL012W	YPL043W	999
YPL012W	YPL126W	996
YPL012W	YPL131W	940
YPL012W	YPL217C	998
YPL012W	YPL266W	999
YPL012W	YPR112C	915
YPL012W	YPR144C	999
YPL013C	Q0140	962
YPL013C	YBL090W	996
YPL013C	YBR146W	999
YPL013C	YBR251W	999
YPL013C	YDR041W	998
YPL013C	YDR175C	997
YPL013C	YDR337W	999
YPL013C	YDR347W	996
YPL013C	YGL129C	997
YPL013C	YGR084C	993
YPL013C	YGR165W	998
YPL013C	YHL004W	999
YPL013C	YIL093C	997
YPL013C	YJR101W	997
YPL013C	YJR113C	999
YPL013C	YKL003C	986
YPL013C	YKL155C	996
YPL013C	YMR158W	999
YPL013C	YMR188C	963
YPL013C	YNL081C	907
YPL013C	YNL137C	994
YPL013C	YNL306W	997
YPL013C	YNR036C	988
YPL013C	YNR037C	952
YPL013C	YOR158W	996
YPL013C	YPL118W	997
YPL014W	YBR135W	901
YPL014W	YPL256C	962
YPL016W	YBR289W	999
YPL016W	YDR073W	992
YPL016W	YDR176W	974
YPL016W	YDR448W	972
YPL016W	YEL009C	996
YPL016W	YER161C	991
YPL016W	YFL049W	999
YPL016W	YGR252W	970
YPL016W	YHL025W	999

YPL016W	YJL176C	999
YPL016W	YMR033W	999
YPL016W	YNL236W	969
YPL016W	YNR023W	999
YPL016W	YOL004W	982
YPL016W	YOL051W	983
YPL016W	YOR290C	999
YPL016W	YPL129W	970
YPL016W	YPL240C	959
YPL016W	YPR034W	999
YPL017C	YBR221C	962
YPL017C	YDR019C	921
YPL017C	YDR148C	972
YPL017C	YER178W	958
YPL017C	YIL125W	963
YPL017C	YMR189W	935
YPL017C	YNL071W	958
YPL018W	YAL034W-A	995
YPL018W	YBR107C	999
YPL018W	YBR211C	999
YPL018W	YDR254W	999
YPL018W	YDR318W	999
YPL018W	YDR383C	995
YPL018W	YEL061C	989
YPL018W	YER016W	933
YPL018W	YGL060W	991
YPL018W	YGL086W	967
YPL018W	YGR113W	999
YPL018W	YGR140W	999
YPL018W	YGR179C	999
YPL018W	YGR188C	991
YPL018W	YIL144W	997
YPL018W	YIR010W	992
YPL018W	YJL013C	959
YPL018W	YJL030W	999
YPL018W	YJR135C	999
YPL018W	YKL049C	999
YPL018W	YKR037C	983
YPL018W	YLR381W	999
YPL018W	YML094W	909
YPL018W	YMR094W	998
YPL018W	YMR117C	983
YPL018W	YMR168C	989
YPL018W	YOL069W	940

YPL018W	YOR195W	955
YPL018W	YPL233W	965
YPL018W	YPR046W	996
YPL019C	YKL065C	998
YPL020C	YAR002W	999
YPL020C	YAR027W	924
YPL020C	YDL013W	994
YPL020C	YDL116W	925
YPL020C	YDL207W	988
YPL020C	YDR076W	966
YPL020C	YDR100W	906
YPL020C	YDR192C	989
YPL020C	YDR510W	999
YPL020C	YER095W	969
YPL020C	YER110C	927
YPL020C	YER116C	973
YPL020C	YGL163C	966
YPL020C	YIL031W	997
YPL020C	YJL092W	971
YPL020C	YKL057C	982
YPL020C	YKL113C	993
YPL020C	YKL186C	984
YPL020C	YKR082W	982
YPL020C	YLR335W	958
YPL020C	YLR347C	999
YPL020C	YML032C	966
YPL020C	YMR153W	984
YPL020C	YMR224C	966
YPL020C	YMR308C	999
YPL020C	YNL189W	999
YPL020C	YNL250W	966
YPL020C	YOR098C	958
YPL020C	YOR156C	922
YPL020C	YPL169C	918
YPL022W	YAL015C	933
YPL022W	YAL027W	999
YPL022W	YAR007C	999
YPL022W	YBL019W	999
YPL022W	YBR098W	923
YPL022W	YBR114W	971
YPL022W	YBR223C	999
YPL022W	YCR066W	999
YPL022W	YCR092C	968
YPL022W	YDL059C	988

YPL022W	YDL140C	909
YPL022W	YDR097C	924
YPL022W	YDR311W	983
YPL022W	YDR386W	944
YPL022W	YDR460W	980
YPL022W	YEL037C	944
YPL022W	YER095W	999
YPL022W	YER162C	997
YPL022W	YER171W	997
YPL022W	YER173W	911
YPL022W	YGL058W	993
YPL022W	YGL163C	945
YPL022W	YGR258C	997
YPL022W	YIL128W	932
YPL022W	YIL143C	993
YPL022W	YJL092W	986
YPL022W	YJR035W	930
YPL022W	YJR043C	973
YPL022W	YJR052W	971
YPL022W	YKL113C	989
YPL022W	YKL114C	999
YPL022W	YLR005W	983
YPL022W	YLR032W	992
YPL022W	YLR135W	998
YPL022W	YLR234W	983
YPL022W	YML021C	980
YPL022W	YML032C	999
YPL022W	YML095C	999
YPL022W	YMR156C	980
YPL022W	YMR167W	935
YPL022W	YMR190C	988
YPL022W	YMR201C	999
YPL022W	YMR224C	998
YPL022W	YMR284W	978
YPL022W	YNL082W	991
YPL022W	YNL250W	943
YPL022W	YNL312W	989
YPL022W	YOL043C	943
YPL022W	YOL090W	999
YPL022W	YOR033C	943
YPL022W	YOR368W	976
YPL022W	YPL167C	999
YPL022W	YPR025C	902
YPL022W	YPR056W	900

YPL023C	YAL044C	913
YPL023C	YBR263W	900
YPL023C	YDR019C	913
YPL023C	YGL125W	999
YPL023C	YLR058C	900
YPL023C	YMR189W	907
YPL024W	YBR098W	998
YPL024W	YBR228W	978
YPL024W	YDL013W	979
YPL024W	YDR386W	998
YPL024W	YER095W	996
YPL024W	YER116C	905
YPL024W	YHL006C	908
YPL024W	YHR031C	955
YPL024W	YJL092W	990
YPL024W	YJR043C	918
YPL024W	YLR135W	982
YPL024W	YLR234W	999
YPL024W	YML032C	970
YPL024W	YMR190C	999
YPL026C	YDL147W	999
YPL026C	YDL194W	985
YPL026C	YMR011W	981
YPL028W	YAL054C	915
YPL028W	YGL205W	905
YPL028W	YIL160C	913
YPL028W	YLR153C	964
YPL028W	YML126C	950
YPL028W	YNL071W	916
YPL028W	YNL117W	944
YPL028W	YNR016C	929
YPL029W	Q0140	943
YPL029W	YDR194C	904
YPL029W	YMR287C	976
YPL029W	YOR017W	932
YPL031C	YBR093C	970
YPL031C	YDL127W	999
YPL031C	YDL179W	999
YPL031C	YDL225W	953
YPL031C	YDL246C	970
YPL031C	YDR146C	996
YPL031C	YDR388W	997
YPL031C	YDR477W	997
YPL031C	YEL009C	984

YPL031C	YER059W	999
YPL031C	YER133W	905
YPL031C	YFL033C	987
YPL031C	YFR034C	999
YPL031C	YGL134W	999
YPL031C	YGL215W	997
YPL031C	YGR233C	999
YPL031C	YHR071W	999
YPL031C	YIL050W	999
YPL031C	YJL084C	921
YPL031C	YJR159W	979
YPL031C	YKL035W	928
YPL031C	YKR002W	976
YPL031C	YLR079W	986
YPL031C	YLR258W	999
YPL031C	YMR199W	981
YPL031C	YMR311C	989
YPL031C	YNL025C	908
YPL031C	YNL289W	999
YPL031C	YOL001W	999
YPL031C	YPL219W	999
YPL031C	YPL254W	903
YPL031C	YPL256C	909
YPL037C	YAL003W	988
YPL037C	YBR031W	924
YPL037C	YBR084C-A	940
YPL037C	YBR191W	999
YPL037C	YDL061C	968
YPL037C	YDL075W	999
YPL037C	YDL192W	999
YPL037C	YDR064W	999
YPL037C	YDR233C	938
YPL037C	YDR252W	999
YPL037C	YDR382W	999
YPL037C	YDR418W	970
YPL037C	YDR500C	963
YPL037C	YEL027W	964
YPL037C	YER056C-A	999
YPL037C	YER068W	918
YPL037C	YER102W	946
YPL037C	YER117W	971
YPL037C	YGL030W	999
YPL037C	YGL103W	998
YPL037C	YGL123W	999

YPL037C	YGR118W	980
YPL037C	YGR148C	939
YPL037C	YHL001W	950
YPL037C	YHL033C	999
YPL037C	YHR193C	999
YPL037C	YHR203C	935
YPL037C	YIL052C	999
YPL037C	YJR094W-A	959
YPL037C	YJR105W	917
YPL037C	YJR123W	999
YPL037C	YKL006W	999
YPL037C	YKL180W	999
YPL037C	YLR048W	956
YPL037C	YLR060W	971
YPL037C	YLR061W	999
YPL037C	YLR075W	999
YPL037C	YLR185W	926
YPL037C	YLR264W	904
YPL037C	YLR340W	983
YPL037C	YLR344W	978
YPL037C	YLR388W	999
YPL037C	YLR448W	981
YPL037C	YML024W	999
YPL037C	YMR194W	999
YPL037C	YNL178W	999
YPL037C	YOL127W	996
YPL037C	YOR063W	965
YPL037C	YOR096W	953
YPL037C	YOR270C	938
YPL037C	YPL131W	977
YPL037C	YPL220W	933
YPL037C	YPR132W	965
YPL038W	YDR253C	996
YPL038W	YIR017C	950
YPL038W	YNL103W	998
YPL040C	YBL076C	913
YPL040C	YDR341C	902
YPL040C	YGR264C	950
YPL040C	YHR206W	914
YPL040C	YOR168W	901
YPL040C	YPL160W	901
YPL042C	YBL093C	999
YPL042C	YBR193C	989
YPL042C	YBR253W	999

YPL042C	YCR081W	999
YPL042C	YCR084C	994
YPL042C	YDL005C	997
YPL042C	YDL140C	998
YPL042C	YDR308C	999
YPL042C	YDR335W	994
YPL042C	YDR443C	999
YPL042C	YDR477W	995
YPL042C	YEL009C	995
YPL042C	YER022W	999
YPL042C	YER068W	912
YPL042C	YGL025C	995
YPL042C	YGL127C	999
YPL042C	YGL151W	995
YPL042C	YGR104C	999
YPL042C	YHR041C	991
YPL042C	YHR058C	995
YPL042C	YLR071C	999
YPL042C	YLR418C	960
YPL042C	YML007W	974
YPL042C	YMR037C	990
YPL042C	YMR112C	989
YPL042C	YNL025C	999
YPL042C	YNL236W	998
YPL042C	YNR010W	992
YPL042C	YOL051W	999
YPL042C	YOL135C	992
YPL042C	YOR174W	998
YPL042C	YPL248C	999
YPL042C	YPR070W	999
YPL042C	YPR168W	996
YPL043W	YBL004W	998
YPL043W	YBR142W	999
YPL043W	YDL014W	984
YPL043W	YDL031W	994
YPL043W	YDR060W	999
YPL043W	YDR496C	999
YPL043W	YER006W	997
YPL043W	YGL111W	985
YPL043W	YGR103W	999
YPL043W	YHR052W	999
YPL043W	YHR066W	996
YPL043W	YHR088W	985
YPL043W	YHR089C	973

YPL043W	YKL014C	999
YPL043W	YLL008W	999
YPL043W	YLL034C	979
YPL043W	YLL045C	950
YPL043W	YLR197W	945
YPL043W	YLR276C	998
YPL043W	YMR049C	999
YPL043W	YMR290C	999
YPL043W	YNL002C	999
YPL043W	YNL061W	999
YPL043W	YNL110C	989
YPL043W	YNL132W	945
YPL043W	YOL041C	999
YPL043W	YOL077C	999
YPL043W	YOL127W	941
YPL043W	YOR063W	941
YPL043W	YOR206W	997
YPL043W	YOR272W	999
YPL043W	YPL012W	999
YPL043W	YPL093W	997
YPL043W	YPL198W	936
YPL043W	YPL211W	983
YPL045W	YAL002W	999
YPL045W	YDL077C	999
YPL045W	YDR080W	999
YPL045W	YDR495C	987
YPL045W	YGL212W	986
YPL045W	YLR148W	999
YPL045W	YLR396C	999
YPL045W	YMR197C	982
YPL045W	YMR231W	999
YPL045W	YOR106W	998
YPL046C	YBR114W	998
YPL046C	YER059W	924
YPL046C	YGL115W	949
YPL046C	YGR003W	945
YPL046C	YJR052W	999
YPL046C	YNL230C	989
YPL047W	YBR081C	999
YPL047W	YBR111W-A	998
YPL047W	YBR198C	999
YPL047W	YCL010C	999
YPL047W	YDR145W	999
YPL047W	YDR167W	999

YPL047W	YDR176W	999
YPL047W	YDR392W	999
YPL047W	YDR448W	999
YPL047W	YGL066W	999
YPL047W	YGL112C	999
YPL047W	YGR252W	999
YPL047W	YHR099W	999
YPL047W	YLR055C	999
YPL047W	YMR223W	999
YPL047W	YMR236W	999
YPL047W	YOL148C	999
YPL047W	YPL254W	999
YPL048W	YAL003W	996
YPL048W	YAL026C	976
YPL048W	YER042W	923
YPL048W	YKL081W	996
YPL048W	YLL026W	990
YPL048W	YLR249W	968
YPL048W	YMR187C	935
YPL048W	YPR080W	997
YPL049C	YBL016W	999
YPL049C	YBL017C	929
YPL049C	YBR083W	937
YPL049C	YDR480W	999
YPL049C	YGR040W	999
YPL049C	YGR178C	913
YPL049C	YHR084W	999
YPL049C	YKR028W	903
YPL050C	YBR106W	990
YPL050C	YDR245W	999
YPL050C	YEL036C	999
YPL050C	YGR132C	993
YPL050C	YGR231C	994
YPL050C	YJL183W	999
YPL050C	YJR075W	999
YPL050C	YML115C	999
YPL051W	YDR320C	949
YPL051W	YGL005C	929
YPL051W	YGL054C	923
YPL051W	YHL031C	945
YPL051W	YJL004C	949
YPL051W	YKL212W	909
YPL051W	YLR262C	981
YPL051W	YML071C	916

YPL051W	YMR123W	909
YPL051W	YNL041C	925
YPL051W	YNL051W	915
YPL051W	YNL297C	906
YPL051W	YOL018C	928
YPL051W	YOR070C	986
YPL055C	YAL011W	968
YPL055C	YBR231C	964
YPL055C	YDL074C	999
YPL055C	YDR334W	976
YPL055C	YDR359C	924
YPL055C	YDR485C	968
YPL055C	YER111C	937
YPL055C	YJL168C	990
YPL055C	YKL139W	959
YPL055C	YKL213C	943
YPL055C	YLR085C	978
YPL055C	YML041C	979
YPL055C	YOL012C	999
YPL057C	YBR036C	999
YPL057C	YBR161W	977
YPL057C	YCR009C	980
YPL057C	YDR270W	993
YPL057C	YKL004W	984
YPL057C	YLR220W	919
YPL057C	YMR251W-A	930
YPL057C	YMR272C	919
YPL058C	YDR011W	910
YPL058C	YFR014C	909
YPL058C	YGR224W	921
YPL058C	YML052W	998
YPL058C	YML076C	923
YPL058C	YOR153W	924
YPL059W	YIL105C	987
YPL059W	YLR369W	939
YPL059W	YNL047C	991
YPL059W	YPR067W	935
YPL061W	YAL054C	900
YPL061W	YBR145W	900
YPL061W	YDL022W	950
YPL061W	YDL168W	915
YPL061W	YGL256W	924
YPL061W	YJL101C	942
YPL061W	YLR044C	920

YPL061W	YLR153C	984
YPL061W	YMR083W	900
YPL061W	YMR303C	959
YPL061W	YNL241C	979
YPL061W	YOL086C	900
YPL061W	YOR374W	970
YPL063W	YGR033C	999
YPL063W	YJL104W	997
YPL063W	YJL143W	999
YPL063W	YML054C	972
YPL063W	YNR017W	999
YPL065W	YCL008C	999
YPL065W	YGR206W	999
YPL065W	YHL027W	933
YPL065W	YJR102C	999
YPL065W	YKL041W	971
YPL065W	YLR119W	999
YPL065W	YLR417W	999
YPL065W	YMR077C	999
YPL065W	YOR275C	908
YPL065W	YPL002C	998
YPL066W	YBR011C	999
YPL066W	YLR425W	999
YPL069C	YCR032W	926
YPL069C	YEL031W	908
YPL069C	YHR190W	923
YPL069C	YJL167W	987
YPL070W	YEL066W	984
YPL070W	YER023W	923
YPL070W	YHL018W	959
YPL070W	YJL199C	984
YPL070W	YJL218W	975
YPL070W	YLR245C	937
YPL070W	YLR291C	996
YPL070W	YLR321C	987
YPL070W	YNL044W	992
YPL070W	YNL331C	923
YPL070W	YPL154C	984
YPL074W	YER133W	965
YPL075W	YNL199C	999
YPL075W	YNL216W	999
YPL076W	YDR437W	986
YPL076W	YGR216C	991
YPL076W	YNL038W	954

YPL076W	YPL175W	994
YPL078C	Q0080	999
YPL078C	Q0085	999
YPL078C	Q0130	999
YPL078C	YBL099W	999
YPL078C	YBR039W	999
YPL078C	YDL004W	999
YPL078C	YDL181W	999
YPL078C	YDR298C	999
YPL078C	YDR322C-A	998
YPL078C	YDR377W	999
YPL078C	YJR121W	999
YPL078C	YKL016C	999
YPL078C	YLR295C	999
YPL078C	YML081C-A	999
YPL078C	YPL271W	999
YPL078C	YPR020W	999
YPL079W	YBL027W	923
YPL079W	YBL072C	920
YPL079W	YBL087C	936
YPL079W	YBL092W	965
YPL079W	YBR031W	954
YPL079W	YBR048W	925
YPL079W	YBR084C-A	939
YPL079W	YBR118W	907
YPL079W	YBR181C	919
YPL079W	YBR189W	980
YPL079W	YBR191W	996
YPL079W	YCR031C	957
YPL079W	YDL061C	958
YPL079W	YDL075W	970
YPL079W	YDL081C	933
YPL079W	YDL082W	951
YPL079W	YDL130W	915
YPL079W	YDL136W	924
YPL079W	YDL191W	912
YPL079W	YDR012W	958
YPL079W	YDR025W	916
YPL079W	YDR064W	991
YPL079W	YDR382W	972
YPL079W	YDR385W	913
YPL079W	YDR418W	974
YPL079W	YDR447C	932
YPL079W	YDR450W	921

YPL079W	YDR471W	961
YPL079W	YDR500C	923
YPL079W	YEL054C	919
YPL079W	YER056C-A	964
YPL079W	YER074W	906
YPL079W	YER102W	935
YPL079W	YER117W	940
YPL079W	YER131W	953
YPL079W	YFL034C-A	913
YPL079W	YFR031C-A	929
YPL079W	YFR032C-A	928
YPL079W	YGL030W	976
YPL079W	YGL031C	963
YPL079W	YGL076C	971
YPL079W	YGL103W	974
YPL079W	YGL123W	975
YPL079W	YGL135W	916
YPL079W	YGL147C	950
YPL079W	YGL189C	910
YPL079W	YGR027C	972
YPL079W	YGR034W	933
YPL079W	YGR085C	988
YPL079W	YGR118W	980
YPL079W	YGR148C	994
YPL079W	YGR214W	980
YPL079W	YHL001W	978
YPL079W	YHL015W	962
YPL079W	YHL033C	977
YPL079W	YHR010W	962
YPL079W	YHR021C	926
YPL079W	YHR141C	950
YPL079W	YHR203C	956
YPL079W	YIL018W	914
YPL079W	YIL052C	977
YPL079W	YIL069C	906
YPL079W	YIL133C	933
YPL079W	YIL148W	923
YPL079W	YJL136C	974
YPL079W	YJL177W	957
YPL079W	YJL189W	934
YPL079W	YJL190C	936
YPL079W	YJL191W	957
YPL079W	YJR094W-A	967
YPL079W	YJR123W	985

YPL079W	YJR145C	943
YPL079W	YKL006W	993
YPL079W	YKL156W	961
YPL079W	YKL180W	978
YPL079W	YKR057W	925
YPL079W	YKR094C	906
YPL079W	YLL045C	958
YPL079W	YLR029C	957
YPL079W	YLR048W	978
YPL079W	YLR061W	994
YPL079W	YLR075W	967
YPL079W	YLR167W	920
YPL079W	YLR185W	928
YPL079W	YLR264W	966
YPL079W	YLR325C	925
YPL079W	YLR333C	936
YPL079W	YLR340W	969
YPL079W	YLR344W	967
YPL079W	YLR367W	962
YPL079W	YLR388W	976
YPL079W	YLR406C	921
YPL079W	YLR441C	940
YPL079W	YLR448W	974
YPL079W	YML024W	981
YPL079W	YML026C	921
YPL079W	YML063W	979
YPL079W	YML073C	927
YPL079W	YMR121C	917
YPL079W	YMR142C	964
YPL079W	YMR194W	966
YPL079W	YMR230W	965
YPL079W	YMR242C	944
YPL079W	YMR260C	912
YPL079W	YNL067W	974
YPL079W	YNL069C	919
YPL079W	YNL096C	934
YPL079W	YNL162W	927
YPL079W	YNL178W	988
YPL079W	YNL301C	940
YPL079W	YNL302C	919
YPL079W	YOL039W	942
YPL079W	YOL040C	945
YPL079W	YOL120C	929
YPL079W	YOL121C	931

YPL079W	YOL127W	977
YPL079W	YOR063W	966
YPL079W	YOR096W	971
YPL079W	YOR133W	913
YPL079W	YOR167C	959
YPL079W	YOR234C	973
YPL079W	YOR293W	974
YPL079W	YOR312C	931
YPL079W	YOR369C	951
YPL079W	YPL081W	952
YPL079W	YPL090C	902
YPL079W	YPL131W	959
YPL079W	YPL143W	973
YPL079W	YPL198W	926
YPL079W	YPL220W	934
YPL079W	YPL249C-A	928
YPL079W	YPR043W	936
YPL079W	YPR080W	907
YPL079W	YPR102C	999
YPL079W	YPR132W	944
YPL081W	YAL035W	912
YPL081W	YBL027W	902
YPL081W	YBL072C	917
YPL081W	YBL087C	916
YPL081W	YBL092W	929
YPL081W	YBR031W	940
YPL081W	YBR048W	999
YPL081W	YBR079C	997
YPL081W	YBR084C-A	912
YPL081W	YBR118W	901
YPL081W	YBR189W	999
YPL081W	YBR191W	952
YPL081W	YCL011C	972
YPL081W	YCR031C	998
YPL081W	YDL061C	993
YPL081W	YDL075W	925
YPL081W	YDL081C	905
YPL081W	YDL082W	945
YPL081W	YDL083C	999
YPL081W	YDL130W	905
YPL081W	YDL136W	928
YPL081W	YDL191W	920
YPL081W	YDR012W	939
YPL081W	YDR025W	997

YPL081W	YDR064W	999
YPL081W	YDR382W	937
YPL081W	YDR385W	989
YPL081W	YDR418W	968
YPL081W	YDR447C	919
YPL081W	YDR450W	996
YPL081W	YDR471W	955
YPL081W	YDR500C	924
YPL081W	YEL054C	923
YPL081W	YER025W	920
YPL081W	YER056C-A	933
YPL081W	YER074W	917
YPL081W	YER102W	998
YPL081W	YER117W	916
YPL081W	YER131W	951
YPL081W	YFL034C-A	906
YPL081W	YFR031C-A	912
YPL081W	YFR032C-A	918
YPL081W	YGL030W	964
YPL081W	YGL031C	936
YPL081W	YGL076C	952
YPL081W	YGL103W	960
YPL081W	YGL123W	999
YPL081W	YGL135W	908
YPL081W	YGL147C	923
YPL081W	YGL189C	917
YPL081W	YGR027C	947
YPL081W	YGR034W	907
YPL081W	YGR085C	934
YPL081W	YGR118W	995
YPL081W	YGR148C	951
YPL081W	YGR214W	998
YPL081W	YHL001W	957
YPL081W	YHL015W	992
YPL081W	YHL033C	953
YPL081W	YHR010W	937
YPL081W	YHR021C	917
YPL081W	YHR141C	923
YPL081W	YHR203C	921
YPL081W	YIL018W	901
YPL081W	YIL052C	939
YPL081W	YIL069C	997
YPL081W	YIL133C	962
YPL081W	YIL148W	910

YPL081W	YJL136C	935
YPL081W	YJL177W	955
YPL081W	YJL189W	914
YPL081W	YJL190C	999
YPL081W	YJL191W	988
YPL081W	YJR094W-A	931
YPL081W	YJR123W	997
YPL081W	YJR145C	997
YPL081W	YKL006W	977
YPL081W	YKL156W	947
YPL081W	YKL180W	952
YPL081W	YKR057W	921
YPL081W	YLL045C	928
YPL081W	YLR029C	948
YPL081W	YLR048W	970
YPL081W	YLR061W	961
YPL081W	YLR075W	947
YPL081W	YLR185W	927
YPL081W	YLR264W	948
YPL081W	YLR287C-A	902
YPL081W	YLR325C	919
YPL081W	YLR333C	921
YPL081W	YLR340W	933
YPL081W	YLR344W	924
YPL081W	YLR367W	950
YPL081W	YLR388W	944
YPL081W	YLR406C	904
YPL081W	YLR441C	921
YPL081W	YLR448W	977
YPL081W	YML024W	950
YPL081W	YML026C	968
YPL081W	YML063W	966
YPL081W	YML073C	916
YPL081W	YMR121C	922
YPL081W	YMR142C	942
YPL081W	YMR146C	997
YPL081W	YMR194W	950
YPL081W	YMR230W	939
YPL081W	YMR242C	926
YPL081W	YMR260C	908
YPL081W	YMR309C	924
YPL081W	YNL067W	967
YPL081W	YNL069C	958
YPL081W	YNL096C	927

YPL081W	YNL132W	980
YPL081W	YNL162W	914
YPL081W	YNL178W	996
YPL081W	YNL301C	917
YPL081W	YNL302C	919
YPL081W	YOL039W	916
YPL081W	YOL040C	994
YPL081W	YOL120C	904
YPL081W	YOL121C	927
YPL081W	YOL127W	962
YPL081W	YOR063W	952
YPL081W	YOR096W	999
YPL081W	YOR167C	927
YPL081W	YOR182C	902
YPL081W	YOR204W	974
YPL081W	YOR234C	958
YPL081W	YOR293W	963
YPL081W	YOR312C	915
YPL081W	YOR361C	923
YPL081W	YOR369C	945
YPL081W	YPL079W	952
YPL081W	YPL131W	951
YPL081W	YPL143W	948
YPL081W	YPL198W	921
YPL081W	YPL220W	917
YPL081W	YPL237W	925
YPL081W	YPL249C-A	926
YPL081W	YPR043W	914
YPL081W	YPR080W	901
YPL081W	YPR102C	954
YPL081W	YPR132W	931
YPL081W	YPR163C	913
YPL082C	YBL003C	983
YPL082C	YBR009C	975
YPL082C	YBR049C	973
YPL082C	YBR089C-A	969
YPL082C	YBR245C	999
YPL082C	YDL013W	904
YPL082C	YDR224C	985
YPL082C	YDR303C	991
YPL082C	YDR392W	999
YPL082C	YDR510W	935
YPL082C	YER116C	906
YPL082C	YER148W	999

YPL082C	YER159C	960
YPL082C	YFR013W	988
YPL082C	YFR037C	972
YPL082C	YGL133W	968
YPL082C	YHR099W	972
YPL082C	YIL126W	991
YPL082C	YKR001C	998
YPL082C	YLR033W	970
YPL082C	YLR176C	982
YPL082C	YLR357W	972
YPL082C	YMR072W	977
YPL082C	YMR091C	970
YPL082C	YOR116C	970
YPL082C	YOR304W	974
YPL082C	YPR086W	981
YPL083C	YAR008W	999
YPL083C	YLR105C	999
YPL083C	YMR059W	998
YPL083C	YOR123C	998
YPL084W	YDR069C	999
YPL084W	YER125W	987
YPL084W	YHL027W	928
YPL084W	YKL002W	945
YPL084W	YKL041W	921
YPL084W	YLR025W	999
YPL084W	YMR077C	966
YPL084W	YMR119W	901
YPL084W	YNL008C	907
YPL084W	YNL159C	907
YPL084W	YPR173C	999
YPL085W	YCR067C	989
YPL085W	YDL195W	999
YPL085W	YDR299W	921
YPL085W	YHR079C	957
YPL085W	YIL109C	999
YPL085W	YLR208W	999
YPL085W	YNL049C	999
YPL085W	YPL218W	999
YPL085W	YPR181C	999
YPL086C	YAL020C	987
YPL086C	YBL071W-A	989
YPL086C	YBR009C	920
YPL086C	YDL074C	952
YPL086C	YGR200C	999

YPL086C	YGR252W	996
YPL086C	YHR187W	999
YPL086C	YKL110C	999
YPL086C	YKL139W	915
YPL086C	YLR384C	999
YPL086C	YMR312W	999
YPL086C	YNL272C	972
YPL086C	YPL101W	999
YPL087W	YHL003C	997
YPL087W	YKL008C	993
YPL088W	YOR226C	904
YPL089C	YBL016W	911
YPL089C	YBR182C	986
YPL089C	YER111C	939
YPL089C	YGR040W	924
YPL089C	YGR229C	993
YPL089C	YHR030C	999
YPL089C	YLR342W	972
YPL089C	YOR231W	949
YPL090C	YAL035W	940
YPL090C	YBL027W	994
YPL090C	YBL072C	995
YPL090C	YBL087C	901
YPL090C	YBL092W	912
YPL090C	YBR031W	995
YPL090C	YBR048W	995
YPL090C	YBR079C	906
YPL090C	YBR084C-A	902
YPL090C	YBR181C	994
YPL090C	YBR189W	995
YPL090C	YBR191W	902
YPL090C	YCR031C	994
YPL090C	YDL075W	995
YPL090C	YDL081C	910
YPL090C	YDL082W	995
YPL090C	YDL136W	995
YPL090C	YDR012W	997
YPL090C	YDR025W	994
YPL090C	YDR064W	996
YPL090C	YDR382W	901
YPL090C	YDR385W	906
YPL090C	YDR447C	995
YPL090C	YDR450W	907
YPL090C	YDR471W	995

YPL090C	YER025W	961
YPL090C	YER074W	994
YPL090C	YER102W	995
YPL090C	YER117W	901
YPL090C	YFL034C-A	900
YPL090C	YFR031C-A	995
YPL090C	YFR032C-A	917
YPL090C	YGL030W	909
YPL090C	YGL076C	907
YPL090C	YGL103W	995
YPL090C	YGL135W	902
YPL090C	YGL147C	909
YPL090C	YGR027C	900
YPL090C	YGR034W	995
YPL090C	YGR085C	997
YPL090C	YGR118W	904
YPL090C	YGR214W	906
YPL090C	YHL001W	994
YPL090C	YHL015W	913
YPL090C	YHL033C	994
YPL090C	YHR021C	903
YPL090C	YHR141C	902
YPL090C	YHR203C	995
YPL090C	YIL018W	994
YPL090C	YIL069C	994
YPL090C	YIL148W	900
YPL090C	YJL136C	906
YPL090C	YJL177W	995
YPL090C	YJR007W	913
YPL090C	YJR094W-A	903
YPL090C	YJR123W	995
YPL090C	YJR145C	996
YPL090C	YKL006W	904
YPL090C	YKL156W	903
YPL090C	YKL180W	903
YPL090C	YKR057W	906
YPL090C	YKR094C	900
YPL090C	YLL045C	996
YPL090C	YLR029C	995
YPL090C	YLR048W	906
YPL090C	YLR061W	901
YPL090C	YLR075W	994
YPL090C	YLR264W	921
YPL090C	YLR340W	996

YPL090C	YLR344W	904
YPL090C	YLR406C	900
YPL090C	YLR441C	994
YPL090C	YLR448W	995
YPL090C	YML024W	903
YPL090C	YML026C	907
YPL090C	YML063W	994
YPL090C	YML073C	995
YPL090C	YMR142C	997
YPL090C	YMR146C	923
YPL090C	YMR194W	902
YPL090C	YMR230W	901
YPL090C	YMR242C	995
YPL090C	YMR260C	918
YPL090C	YNL067W	910
YPL090C	YNL069C	994
YPL090C	YNL096C	994
YPL090C	YNL132W	931
YPL090C	YNL162W	902
YPL090C	YNL178W	996
YPL090C	YNL301C	994
YPL090C	YNL302C	907
YPL090C	YOL039W	901
YPL090C	YOL040C	995
YPL090C	YOL120C	906
YPL090C	YOL121C	907
YPL090C	YOL127W	996
YPL090C	YOL139C	905
YPL090C	YOR063W	993
YPL090C	YOR096W	996
YPL090C	YOR133W	906
YPL090C	YOR167C	920
YPL090C	YOR204W	964
YPL090C	YOR234C	904
YPL090C	YOR293W	902
YPL090C	YOR312C	997
YPL090C	YOR361C	908
YPL090C	YPL079W	902
YPL090C	YPL143W	903
YPL090C	YPL198W	995
YPL090C	YPL220W	902
YPL090C	YPL249C-A	902
YPL090C	YPR041W	922
YPL090C	YPR043W	903

YPL090C	YPR102C	945
YPL090C	YPR132W	904
YPL091W	YBR244W	979
YPL091W	YCL035C	927
YPL091W	YCR083W	987
YPL091W	YDR353W	994
YPL091W	YDR513W	930
YPL091W	YER070W	988
YPL091W	YGR209C	999
YPL091W	YHR106W	990
YPL091W	YHR206W	909
YPL091W	YIL066C	988
YPL091W	YIR037W	982
YPL091W	YIR038C	932
YPL091W	YJL026W	990
YPL091W	YKL026C	984
YPL091W	YLL060C	935
YPL091W	YLR043C	998
YPL091W	YLR299W	945
YPL091W	YML007W	924
YPL091W	YOL049W	914
YPL091W	YPR167C	906
YPL092W	YGL254W	991
YPL092W	YJR090C	955
YPL092W	YKL001C	902
YPL093W	YBL087C	972
YPL093W	YBR142W	918
YPL093W	YCL054W	978
YPL093W	YCR072C	990
YPL093W	YDL031W	979
YPL093W	YDL082W	987
YPL093W	YDR060W	952
YPL093W	YDR087C	936
YPL093W	YDR101C	999
YPL093W	YDR471W	936
YPL093W	YDR496C	990
YPL093W	YER006W	999
YPL093W	YER126C	999
YPL093W	YFL002C	989
YPL093W	YFR001W	984
YPL093W	YFR031C-A	953
YPL093W	YGL030W	935
YPL093W	YGL076C	943
YPL093W	YGL103W	936

YPL093W	YGL111W	999
YPL093W	YGL135W	933
YPL093W	YGL147C	931
YPL093W	YGR085C	937
YPL093W	YGR103W	999
YPL093W	YGR245C	999
YPL093W	YHR010W	982
YPL093W	YHR052W	998
YPL093W	YHR066W	997
YPL093W	YHR085W	998
YPL093W	YHR088W	989
YPL093W	YHR170W	926
YPL093W	YHR197W	999
YPL093W	YKL009W	993
YPL093W	YKL021C	913
YPL093W	YKL172W	990
YPL093W	YKR081C	999
YPL093W	YLL008W	978
YPL093W	YLL034C	982
YPL093W	YLL045C	949
YPL093W	YLR002C	972
YPL093W	YLR009W	999
YPL093W	YLR029C	945
YPL093W	YLR074C	999
YPL093W	YLR106C	987
YPL093W	YLR276C	981
YPL093W	YLR325C	974
YPL093W	YLR449W	997
YPL093W	YML073C	937
YPL093W	YMR049C	999
YPL093W	YMR242C	988
YPL093W	YMR290C	998
YPL093W	YNL002C	998
YPL093W	YNL061W	999
YPL093W	YNL110C	999
YPL093W	YNL182C	995
YPL093W	YNR053C	999
YPL093W	YOL077C	992
YPL093W	YOL127W	964
YPL093W	YOR063W	988
YPL093W	YOR206W	990
YPL093W	YOR272W	998
YPL093W	YPL043W	997
YPL093W	YPL131W	993

YPL093W	YPL198W	935
YPL093W	YPL211W	993
YPL093W	YPL249C-A	931
YPL093W	YPR016C	999
YPL093W	YPR102C	977
YPL094C	YBR171W	999
YPL094C	YDR086C	946
YPL094C	YEL036C	963
YPL094C	YER019C-A	908
YPL094C	YER087C-B	979
YPL094C	YJL034W	919
YPL094C	YJR010C-A	921
YPL094C	YLR292C	999
YPL094C	YLR378C	999
YPL094C	YML055W	924
YPL094C	YOR254C	999
YPL095C	YGL161C	998
YPL096W	YEL037C	999
YPL097W	YGR185C	908
YPL098C	YLR453C	944
YPL100W	YBL103C	956
YPL101W	YDL074C	942
YPL101W	YER148W	923
YPL101W	YGR200C	999
YPL101W	YHR187W	999
YPL101W	YKL110C	995
YPL101W	YLR384C	999
YPL101W	YML098W	923
YPL101W	YMR005W	942
YPL101W	YMR236W	923
YPL101W	YMR312W	999
YPL101W	YPL086C	999
YPL104W	YNL220W	912
YPL105C	YLR116W	964
YPL105C	YMR146C	999
YPL106C	YAL005C	999
YPL106C	YBR101C	998
YPL106C	YBR140C	970
YPL106C	YBR155W	966
YPL106C	YBR169C	999
YPL106C	YDL229W	998
YPL106C	YDR168W	914
YPL106C	YDR172W	980
YPL106C	YGL073W	971

YPL106C	YIL033C	975
YPL106C	YIL159W	946
YPL106C	YLL024C	995
YPL106C	YNL007C	979
YPL106C	YNL064C	999
YPL106C	YNL209W	975
YPL106C	YOR027W	980
YPL106C	YPL240C	990
YPL111W	YFL012W	998
YPL111W	YHR018C	970
YPL111W	YJL088W	987
YPL111W	YKL184W	945
YPL111W	YLR438W	999
YPL111W	YOR130C	941
YPL111W	YPL160W	928
YPL112C	YDL065C	998
YPL112C	YDR329C	998
YPL112C	YDR479C	931
YPL112C	YHR150W	931
YPL112C	YMR204C	984
YPL112C	YOL147C	995
YPL112C	YOR193W	984
YPL115C	YAL041W	992
YPL115C	YDL135C	938
YPL115C	YER149C	908
YPL115C	YER155C	983
YPL115C	YHR107C	949
YPL115C	YLR229C	999
YPL115C	YNL271C	977
YPL115C	YNL298W	992
YPL115C	YOR127W	911
YPL115C	YPL256C	994
YPL115C	YPR165W	969
YPL116W	YNL021W	999
YPL116W	YNL330C	948
YPL116W	YPR068C	949
YPL117C	YJL167W	904
YPL117C	YNR043W	978
YPL118W	Q0140	962
YPL118W	Q0275	912
YPL118W	YBL090W	995
YPL118W	YBR146W	988
YPL118W	YBR251W	999
YPL118W	YDR036C	999

YPL118W	YDR041W	999
YPL118W	YDR175C	988
YPL118W	YDR337W	988
YPL118W	YDR347W	988
YPL118W	YER050C	986
YPL118W	YGL129C	988
YPL118W	YGR084C	974
YPL118W	YGR165W	988
YPL118W	YGR215W	988
YPL118W	YHL004W	999
YPL118W	YIL093C	988
YPL118W	YJR101W	988
YPL118W	YJR113C	988
YPL118W	YKL003C	986
YPL118W	YKL155C	997
YPL118W	YMR158W	974
YPL118W	YMR188C	988
YPL118W	YNL137C	988
YPL118W	YNL306W	988
YPL118W	YNR037C	987
YPL118W	YOR158W	989
YPL118W	YOR204W	991
YPL118W	YPL013C	997
YPL119C	YML017W	925
YPL120W	YBR097W	999
YPL120W	YBR128C	999
YPL120W	YGR218W	908
YPL120W	YLR240W	999
YPL120W	YLR360W	999
YPL121C	YER179W	942
YPL121C	YHR079C-A	953
YPL122C	YDL108W	962
YPL122C	YDR079C-A	999
YPL122C	YDR311W	999
YPL122C	YDR460W	995
YPL122C	YER148W	980
YPL122C	YER171W	999
YPL122C	YIL128W	906
YPL122C	YIL143C	999
YPL122C	YLR005W	997
YPL122C	YPR056W	999
YPL122C	YPR070W	916
YPL124W	YBR109C	972
YPL124W	YDR356W	998

YPL124W	YKL042W	988
YPL124W	YLR291C	931
YPL124W	YNL188W	911
YPL124W	YNL225C	923
YPL124W	YPL255W	983
YPL125W	YDL088C	915
YPL125W	YDR159W	921
YPL125W	YGL100W	905
YPL125W	YGL172W	961
YPL125W	YGR119C	965
YPL125W	YHR088W	984
YPL125W	YKL068W	969
YPL125W	YLR018C	905
YPL125W	YLR293C	983
YPL125W	YLR347C	905
YPL125W	YML103C	919
YPL125W	YMR047C	985
YPL125W	YMR129W	925
YPL125W	YPL169C	922
YPL126W	YBL004W	997
YPL126W	YBL072C	931
YPL126W	YBR247C	968
YPL126W	YCL059C	995
YPL126W	YCR057C	999
YPL126W	YDL014W	995
YPL126W	YDL060W	915
YPL126W	YDL148C	999
YPL126W	YDL153C	913
YPL126W	YDR064W	933
YPL126W	YDR299W	998
YPL126W	YDR324C	999
YPL126W	YDR398W	999
YPL126W	YDR449C	999
YPL126W	YEL055C	987
YPL126W	YER082C	999
YPL126W	YGL171W	935
YPL126W	YGR090W	999
YPL126W	YGR128C	999
YPL126W	YGR145W	997
YPL126W	YHR148W	999
YPL126W	YHR169W	918
YPL126W	YHR196W	999
YPL126W	YJL010C	986
YPL126W	YJL069C	922

YPL126W	YJL109C	999
YPL126W	YJR002W	999
YPL126W	YKR060W	997
YPL126W	YLL011W	998
YPL126W	YLR003C	909
YPL126W	YLR129W	998
YPL126W	YLR175W	911
YPL126W	YLR186W	997
YPL126W	YLR197W	997
YPL126W	YLR222C	998
YPL126W	YLR409C	998
YPL126W	YML093W	921
YPL126W	YMR093W	999
YPL126W	YMR128W	997
YPL126W	YMR229C	999
YPL126W	YMR290C	930
YPL126W	YNL075W	999
YPL126W	YNL132W	997
YPL126W	YOL010W	918
YPL126W	YOR004W	917
YPL126W	YOR078W	990
YPL126W	YOR096W	933
YPL126W	YOR145C	999
YPL126W	YOR310C	999
YPL126W	YPL012W	996
YPL126W	YPL217C	998
YPL126W	YPL266W	999
YPL126W	YPR112C	947
YPL126W	YPR137W	999
YPL126W	YPR144C	999
YPL127C	YBR009C	984
YPL127C	YOL012C	959
YPL127C	YPL153C	988
YPL127C	YPR052C	910
YPL128C	YGR071C	987
YPL128C	YLR373C	998
YPL129W	YBL052C	999
YPL129W	YBL093C	965
YPL129W	YBR081C	923
YPL129W	YBR154C	999
YPL129W	YBR193C	965
YPL129W	YBR198C	997
YPL129W	YBR253W	991
YPL129W	YBR279W	984

YPL129W	YBR289W	999
YPL129W	YCR042C	999
YPL129W	YDL002C	991
YPL129W	YDL005C	965
YPL129W	YDL140C	999
YPL129W	YDR073W	977
YPL129W	YDR145W	994
YPL129W	YDR167W	999
YPL129W	YDR190C	991
YPL129W	YDR308C	994
YPL129W	YDR364C	962
YPL129W	YDR404C	999
YPL129W	YDR443C	916
YPL129W	YER022W	998
YPL129W	YER092W	997
YPL129W	YER148W	999
YPL129W	YFL013C	990
YPL129W	YFL039C	999
YPL129W	YGL025C	991
YPL129W	YGL043W	985
YPL129W	YGL070C	999
YPL129W	YGL112C	995
YPL129W	YGL127C	934
YPL129W	YGL150C	999
YPL129W	YGL244W	908
YPL129W	YGR005C	999
YPL129W	YGR104C	977
YPL129W	YGR186W	999
YPL129W	YGR274C	999
YPL129W	YHL025W	999
YPL129W	YHR041C	965
YPL129W	YHR058C	965
YPL129W	YHR143W-A	965
YPL129W	YIL021W	999
YPL129W	YIL126W	993
YPL129W	YJL081C	999
YPL129W	YJL140W	999
YPL129W	YJL176C	999
YPL129W	YLR052W	991
YPL129W	YLR071C	993
YPL129W	YLR418C	985
YPL129W	YML015C	991
YPL129W	YML098W	999
YPL129W	YML114C	999

YPL129W	YMR005W	991
YPL129W	YMR033W	999
YPL129W	YMR112C	965
YPL129W	YMR227C	991
YPL129W	YMR236W	991
YPL129W	YNL059C	996
YPL129W	YNL107W	909
YPL129W	YNL236W	975
YPL129W	YNR010W	991
YPL129W	YNR023W	999
YPL129W	YOL005C	999
YPL129W	YOL051W	993
YPL129W	YOL135C	991
YPL129W	YOL145C	909
YPL129W	YOR064C	996
YPL129W	YOR141C	996
YPL129W	YOR151C	999
YPL129W	YOR174W	991
YPL129W	YOR210W	984
YPL129W	YOR224C	999
YPL129W	YOR290C	993
YPL129W	YPL011C	998
YPL129W	YPL016W	970
YPL129W	YPL235W	999
YPL129W	YPR034W	998
YPL129W	YPR056W	984
YPL129W	YPR070W	992
YPL129W	YPR168W	991
YPL129W	YPR187W	999
YPL131W	YAL035W	923
YPL131W	YBL027W	999
YPL131W	YBL072C	994
YPL131W	YBL087C	998
YPL131W	YBL092W	998
YPL131W	YBR031W	996
YPL131W	YBR048W	998
YPL131W	YBR084C-A	997
YPL131W	YBR118W	912
YPL131W	YBR142W	981
YPL131W	YBR189W	998
YPL131W	YBR191W	999
YPL131W	YCL037C	978
YPL131W	YCR031C	980
YPL131W	YDL014W	960

YPL131W	YDL031W	956
YPL131W	YDL061C	992
YPL131W	YDL075W	999
YPL131W	YDL081C	905
YPL131W	YDL082W	931
YPL131W	YDL083C	951
YPL131W	YDL130W	955
YPL131W	YDL136W	996
YPL131W	YDL191W	940
YPL131W	YDR012W	999
YPL131W	YDR025W	998
YPL131W	YDR060W	959
YPL131W	YDR064W	999
YPL131W	YDR382W	999
YPL131W	YDR385W	919
YPL131W	YDR418W	999
YPL131W	YDR447C	914
YPL131W	YDR450W	912
YPL131W	YDR471W	963
YPL131W	YDR496C	967
YPL131W	YDR500C	970
YPL131W	YEL054C	928
YPL131W	YER006W	967
YPL131W	YER056C-A	991
YPL131W	YER074W	931
YPL131W	YER102W	998
YPL131W	YER117W	995
YPL131W	YER131W	935
YPL131W	YFL002C	959
YPL131W	YFL034C-A	906
YPL131W	YFR031C-A	998
YPL131W	YFR032C-A	904
YPL131W	YGL030W	998
YPL131W	YGL031C	994
YPL131W	YGL076C	996
YPL131W	YGL103W	999
YPL131W	YGL111W	964
YPL131W	YGL120C	935
YPL131W	YGL123W	999
YPL131W	YGL135W	999
YPL131W	YGL147C	999
YPL131W	YGL189C	921
YPL131W	YGR027C	960
YPL131W	YGR034W	988

YPL131W	YGR085C	999
YPL131W	YGR103W	999
YPL131W	YGR118W	982
YPL131W	YGR148C	979
YPL131W	YGR214W	984
YPL131W	YHL001W	978
YPL131W	YHL015W	994
YPL131W	YHL033C	999
YPL131W	YHL034C	981
YPL131W	YHR010W	956
YPL131W	YHR021C	922
YPL131W	YHR052W	990
YPL131W	YHR088W	960
YPL131W	YHR141C	992
YPL131W	YHR203C	994
YPL131W	YIL018W	987
YPL131W	YIL052C	997
YPL131W	YIL069C	931
YPL131W	YIL133C	999
YPL131W	YIL148W	961
YPL131W	YJL136C	948
YPL131W	YJL177W	997
YPL131W	YJL189W	935
YPL131W	YJL190C	998
YPL131W	YJL191W	977
YPL131W	YJR094W-A	998
YPL131W	YJR123W	999
YPL131W	YJR145C	998
YPL131W	YKL006W	998
YPL131W	YKL009W	958
YPL131W	YKL156W	964
YPL131W	YKL172W	952
YPL131W	YKL180W	999
YPL131W	YKR057W	912
YPL131W	YKR081C	995
YPL131W	YLL008W	956
YPL131W	YLL045C	998
YPL131W	YLR002C	952
YPL131W	YLR029C	999
YPL131W	YLR048W	998
YPL131W	YLR060W	918
YPL131W	YLR061W	997
YPL131W	YLR075W	999
YPL131W	YLR167W	961

YPL131W	YLR185W	997
YPL131W	YLR196W	976
YPL131W	YLR249W	998
YPL131W	YLR264W	962
YPL131W	YLR325C	958
YPL131W	YLR333C	921
YPL131W	YLR340W	999
YPL131W	YLR344W	999
YPL131W	YLR367W	989
YPL131W	YLR388W	998
YPL131W	YLR406C	906
YPL131W	YLR432W	978
YPL131W	YLR441C	958
YPL131W	YLR448W	999
YPL131W	YLR449W	981
YPL131W	YML024W	984
YPL131W	YML026C	912
YPL131W	YML063W	978
YPL131W	YML073C	932
YPL131W	YMR121C	965
YPL131W	YMR142C	935
YPL131W	YMR194W	994
YPL131W	YMR230W	940
YPL131W	YMR242C	995
YPL131W	YMR260C	913
YPL131W	YMR290C	964
YPL131W	YNL002C	958
YPL131W	YNL061W	964
YPL131W	YNL067W	995
YPL131W	YNL069C	947
YPL131W	YNL096C	923
YPL131W	YNL162W	926
YPL131W	YNL178W	999
YPL131W	YNL301C	999
YPL131W	YNL302C	928
YPL131W	YNR037C	951
YPL131W	YNR053C	996
YPL131W	YOL039W	925
YPL131W	YOL040C	985
YPL131W	YOL041C	956
YPL131W	YOL077C	966
YPL131W	YOL120C	950
YPL131W	YOL121C	945
YPL131W	YOL127W	999

YPL131W	YOR063W	999
YPL131W	YOR096W	999
YPL131W	YOR133W	958
YPL131W	YOR167C	921
YPL131W	YOR206W	960
YPL131W	YOR234C	969
YPL131W	YOR272W	961
YPL131W	YOR293W	952
YPL131W	YOR294W	999
YPL131W	YOR312C	934
YPL131W	YOR369C	939
YPL131W	YPL012W	940
YPL131W	YPL037C	977
YPL131W	YPL079W	959
YPL131W	YPL081W	951
YPL131W	YPL093W	993
YPL131W	YPL143W	974
YPL131W	YPL198W	998
YPL131W	YPL211W	963
YPL131W	YPL220W	967
YPL131W	YPL249C-A	996
YPL131W	YPR016C	997
YPL131W	YPR043W	968
YPL131W	YPR080W	912
YPL131W	YPR102C	999
YPL131W	YPR132W	976
YPL132W	Q0045	982
YPL132W	Q0250	982
YPL132W	Q0275	979
YPL132W	YBR037C	932
YPL132W	YER141W	978
YPL132W	YKL155C	933
YPL132W	YLL009C	974
YPL132W	YMR137C	945
YPL132W	YPL172C	995
YPL134C	YIL098C	929
YPL135W	YCL017C	999
YPL135W	YDL120W	999
YPL135W	YGL018C	999
YPL135W	YJR104C	931
YPL135W	YKL040C	976
YPL135W	YLR369W	989
YPL137C	YER122C	923
YPL137C	YER133W	998

YPL137C	YPL170W	923
YPL138C	YAR003W	999
YPL138C	YBR175W	999
YPL138C	YBR258C	999
YPL138C	YDR469W	999
YPL138C	YHR119W	999
YPL138C	YJL168C	966
YPL138C	YKL018W	999
YPL138C	YLR015W	999
YPL139C	YAL013W	999
YPL139C	YBR095C	999
YPL139C	YBR103W	948
YPL139C	YDL076C	999
YPL139C	YDR207C	993
YPL139C	YIL084C	999
YPL139C	YKL185W	993
YPL139C	YKR029C	941
YPL139C	YMR075W	999
YPL139C	YMR263W	999
YPL139C	YNL097C	999
YPL139C	YNL330C	999
YPL139C	YOL004W	999
YPL139C	YPL181W	999
YPL139C	YPR023C	999
YPL140C	YHR030C	999
YPL140C	YIL094C	911
YPL140C	YJL095W	996
YPL140C	YLL021W	924
YPL140C	YOR231W	996
YPL141C	YPL240C	919
YPL143W	YAL035W	923
YPL143W	YBL027W	953
YPL143W	YBL072C	925
YPL143W	YBL087C	930
YPL143W	YBL092W	948
YPL143W	YBR031W	958
YPL143W	YBR048W	936
YPL143W	YBR084C-A	977
YPL143W	YBR118W	912
YPL143W	YBR181C	903
YPL143W	YBR189W	985
YPL143W	YBR191W	998
YPL143W	YCR031C	999
YPL143W	YDL061C	978

YPL143W	YDL075W	981
YPL143W	YDL081C	926
YPL143W	YDL082W	929
YPL143W	YDL130W	928
YPL143W	YDL136W	999
YPL143W	YDL191W	929
YPL143W	YDR012W	969
YPL143W	YDR025W	924
YPL143W	YDR064W	999
YPL143W	YDR382W	982
YPL143W	YDR385W	913
YPL143W	YDR418W	983
YPL143W	YDR447C	930
YPL143W	YDR450W	997
YPL143W	YDR471W	973
YPL143W	YDR500C	967
YPL143W	YEL054C	931
YPL143W	YER056C-A	980
YPL143W	YER074W	924
YPL143W	YER102W	965
YPL143W	YER117W	972
YPL143W	YER131W	963
YPL143W	YFL034C-A	914
YPL143W	YFR031C-A	998
YPL143W	YFR032C-A	948
YPL143W	YGL030W	997
YPL143W	YGL031C	937
YPL143W	YGL076C	952
YPL143W	YGL103W	994
YPL143W	YGL123W	982
YPL143W	YGL135W	998
YPL143W	YGL147C	928
YPL143W	YGL189C	921
YPL143W	YGR027C	978
YPL143W	YGR034W	923
YPL143W	YGR085C	999
YPL143W	YGR118W	997
YPL143W	YGR148C	974
YPL143W	YGR214W	994
YPL143W	YHL001W	986
YPL143W	YHL015W	981
YPL143W	YHL033C	982
YPL143W	YHR010W	967
YPL143W	YHR021C	932

YPL143W	YHR141C	928
YPL143W	YHR203C	964
YPL143W	YIL018W	997
YPL143W	YIL052C	996
YPL143W	YIL069C	924
YPL143W	YIL133C	998
YPL143W	YIL148W	968
YPL143W	YJL136C	965
YPL143W	YJL177W	979
YPL143W	YJL189W	949
YPL143W	YJL190C	925
YPL143W	YJL191W	982
YPL143W	YJR094W-A	993
YPL143W	YJR123W	997
YPL143W	YJR145C	923
YPL143W	YKL006W	997
YPL143W	YKL156W	991
YPL143W	YKL180W	999
YPL143W	YKR057W	934
YPL143W	YKR094C	906
YPL143W	YLL045C	939
YPL143W	YLR029C	970
YPL143W	YLR048W	987
YPL143W	YLR061W	997
YPL143W	YLR075W	974
YPL143W	YLR167W	954
YPL143W	YLR185W	955
YPL143W	YLR264W	994
YPL143W	YLR287C-A	903
YPL143W	YLR325C	926
YPL143W	YLR333C	957
YPL143W	YLR340W	981
YPL143W	YLR344W	995
YPL143W	YLR367W	977
YPL143W	YLR388W	994
YPL143W	YLR406C	921
YPL143W	YLR441C	931
YPL143W	YLR448W	995
YPL143W	YML024W	998
YPL143W	YML026C	920
YPL143W	YML063W	983
YPL143W	YML073C	998
YPL143W	YMR121C	921
YPL143W	YMR142C	998

YPL143W	YMR194W	995
YPL143W	YMR230W	962
YPL143W	YMR242C	934
YPL143W	YMR260C	929
YPL143W	YNL067W	984
YPL143W	YNL069C	998
YPL143W	YNL096C	930
YPL143W	YNL162W	928
YPL143W	YNL178W	991
YPL143W	YNL301C	965
YPL143W	YNL302C	933
YPL143W	YOL039W	926
YPL143W	YOL040C	955
YPL143W	YOL120C	997
YPL143W	YOL121C	933
YPL143W	YOL127W	999
YPL143W	YOR063W	998
YPL143W	YOR096W	982
YPL143W	YOR133W	927
YPL143W	YOR167C	954
YPL143W	YOR182C	903
YPL143W	YOR234C	991
YPL143W	YOR293W	983
YPL143W	YOR312C	941
YPL143W	YOR369C	939
YPL143W	YPL079W	973
YPL143W	YPL081W	948
YPL143W	YPL090C	903
YPL143W	YPL131W	974
YPL143W	YPL198W	931
YPL143W	YPL220W	958
YPL143W	YPL249C-A	999
YPL143W	YPR043W	951
YPL143W	YPR080W	912
YPL143W	YPR102C	983
YPL143W	YPR132W	973
YPL144W	YDL020C	970
YPL144W	YGR135W	967
YPL144W	YGR253C	980
YPL144W	YHR200W	967
YPL144W	YLR021W	999
YPL145C	YKR031C	982
YPL145C	YMR079W	999
YPL145C	YNL267W	983

YPL145C	YPL147W	940
YPL146C	YER006W	934
YPL146C	YHR034C	945
YPL146C	YJL190C	968
YPL146C	YLR175W	910
YPL146C	YLR449W	994
YPL146C	YNL061W	921
YPL146C	YNL096C	968
YPL146C	YPL211W	945
YPL147W	YER015W	935
YPL147W	YGL205W	920
YPL147W	YKL188C	999
YPL147W	YPL145C	940
YPL149W	YBL078C	980
YPL149W	YBR217W	999
YPL149W	YHR171W	992
YPL149W	YLL042C	978
YPL149W	YMR159C	999
YPL149W	YNL223W	941
YPL149W	YNR007C	986
YPL151C	YAL032C	999
YPL151C	YBR065C	999
YPL151C	YBR188C	968
YPL151C	YCR063W	990
YPL151C	YDL030W	976
YPL151C	YDL209C	999
YPL151C	YDR364C	970
YPL151C	YDR416W	999
YPL151C	YER029C	993
YPL151C	YER172C	996
YPL151C	YGL120C	999
YPL151C	YGL128C	990
YPL151C	YGR278W	997
YPL151C	YHR165C	992
YPL151C	YJL203W	974
YPL151C	YJR050W	994
YPL151C	YKL095W	980
YPL151C	YKL173W	991
YPL151C	YLL036C	999
YPL151C	YLR117C	999
YPL151C	YLR147C	983
YPL151C	YLR424W	993
YPL151C	YML049C	976
YPL151C	YMR125W	992

YPL151C	YMR213W	999
YPL151C	YMR240C	999
YPL151C	YMR288W	979
YPL151C	YOR036W	963
YPL151C	YPL213W	998
YPL151C	YPR101W	995
YPL152W	YAL016W	999
YPL152W	YDL047W	986
YPL152W	YDL134C	999
YPL152W	YDL188C	999
YPL152W	YGL190C	976
YPL152W	YIL153W	999
YPL152W	YOR014W	987
YPL153C	YAR002W	939
YPL153C	YBL088C	999
YPL153C	YBR009C	912
YPL153C	YBR087W	996
YPL153C	YBR136W	999
YPL153C	YCL061C	996
YPL153C	YDL017W	997
YPL153C	YDL101C	999
YPL153C	YDL220C	997
YPL153C	YDR052C	999
YPL153C	YDR071C	921
YPL153C	YDR075W	917
YPL153C	YDR076W	992
YPL153C	YDR113C	998
YPL153C	YDR217C	999
YPL153C	YDR369C	946
YPL153C	YDR499W	973
YPL153C	YER089C	999
YPL153C	YER095W	969
YPL153C	YER111C	985
YPL153C	YER173W	986
YPL153C	YHR031C	933
YPL153C	YJL115W	999
YPL153C	YJL194W	959
YPL153C	YJR140C	935
YPL153C	YLR182W	997
YPL153C	YLR335W	938
YPL153C	YML058W	999
YPL153C	YML102W	903
YPL153C	YMR001C	970
YPL153C	YMR190C	998

YPL153C	YNL030W	993
YPL153C	YNL031C	993
YPL153C	YNL201C	992
YPL153C	YOR098C	938
YPL153C	YOR144C	999
YPL153C	YOR368W	991
YPL153C	YPL127C	988
YPL153C	YPL194W	958
YPL154C	YAL048C	900
YPL154C	YBR114W	921
YPL154C	YDR069C	997
YPL154C	YDR233C	992
YPL154C	YEL060C	983
YPL154C	YHR146W	994
YPL154C	YJL146W	993
YPL154C	YKR001C	901
YPL154C	YMR174C	999
YPL154C	YMR297W	991
YPL154C	YNL044W	998
YPL154C	YPL070W	984
YPL155C	YCL029C	994
YPL155C	YEL061C	992
YPL155C	YER016W	961
YPL155C	YGL216W	984
YPL155C	YKR054C	909
YPL155C	YPL269W	999
YPL156C	YPL240C	902
YPL157W	YER029C	984
YPL157W	YJL124C	950
YPL157W	YNR004W	971
YPL160W	Q0120	976
YPL160W	YBL076C	975
YPL160W	YDR037W	912
YPL160W	YDR341C	915
YPL160W	YGR264C	954
YPL160W	YHR020W	978
YPL160W	YLL018C	912
YPL160W	YLR382C	929
YPL160W	YOR168W	936
YPL160W	YPL040C	901
YPL160W	YPL111W	928
YPL161C	YAL041W	966
YPL161C	YDR218C	954
YPL161C	YGR152C	900

YPL161C	YHR061C	905
YPL161C	YHR107C	965
YPL161C	YJL168C	952
YPL161C	YJR076C	991
YPL161C	YKR055W	970
YPL161C	YLR229C	999
YPL161C	YLR371W	912
YPL161C	YNL090W	938
YPL161C	YPL240C	968
YPL161C	YPR165W	999
YPL164C	YDL154W	950
YPL164C	YDR097C	991
YPL164C	YFL003C	966
YPL164C	YMR167W	999
YPL164C	YMR190C	998
YPL164C	YNL082W	910
YPL164C	YOL090W	948
YPL166W	YDR022C	999
YPL166W	YGL180W	964
YPL166W	YLR423C	999
YPL166W	YPR049C	996
YPL167C	YBR088C	902
YPL167C	YBR272C	924
YPL167C	YCR066W	998
YPL167C	YDL102W	971
YPL167C	YDR076W	905
YPL167C	YDR092W	996
YPL167C	YDR419W	999
YPL167C	YER095W	964
YPL167C	YER142C	997
YPL167C	YER171W	984
YPL167C	YER173W	920
YPL167C	YGL058W	998
YPL167C	YGL087C	999
YPL167C	YIL139C	999
YPL167C	YIR002C	963
YPL167C	YJL092W	987
YPL167C	YKL114C	935
YPL167C	YLR032W	998
YPL167C	YML032C	999
YPL167C	YMR137C	957
YPL167C	YMR201C	998
YPL167C	YMR284W	970
YPL167C	YNL262W	906

YPL167C	YOR033C	984
YPL167C	YOR346W	999
YPL167C	YPL022W	999
YPL169C	YAR002W	998
YPL169C	YBL079W	968
YPL169C	YBR111W-A	971
YPL169C	YDL088C	905
YPL169C	YDL116W	999
YPL169C	YDL207W	967
YPL169C	YDR101C	985
YPL169C	YDR138W	998
YPL169C	YDR159W	999
YPL169C	YDR192C	954
YPL169C	YDR381W	999
YPL169C	YDR432W	962
YPL169C	YER105C	966
YPL169C	YER107C	997
YPL169C	YER110C	922
YPL169C	YER165W	941
YPL169C	YFR002W	972
YPL169C	YGL092W	992
YPL169C	YGL100W	999
YPL169C	YGR119C	994
YPL169C	YHR170W	919
YPL169C	YIL115C	997
YPL169C	YIL149C	934
YPL169C	YJL039C	966
YPL169C	YJL041W	996
YPL169C	YJL061W	966
YPL169C	YJR042W	999
YPL169C	YKL057C	994
YPL169C	YKL068W	998
YPL169C	YKL186C	999
YPL169C	YKL214C	938
YPL169C	YKR082W	966
YPL169C	YKR095W	934
YPL169C	YLR018C	968
YPL169C	YLR208W	952
YPL169C	YLR335W	931
YPL169C	YLR347C	994
YPL169C	YML103C	966
YPL169C	YMR047C	999
YPL169C	YMR129W	915
YPL169C	YMR255W	951

YPL169C	YOL072W	985
YPL169C	YOR046C	985
YPL169C	YOR098C	995
YPL169C	YPL020C	918
YPL169C	YPL125W	922
YPL170W	YHR007C	949
YPL170W	YPL137C	923
YPL171C	YHR179W	999
YPL172C	Q0045	991
YPL172C	Q0070	932
YPL172C	Q0250	976
YPL172C	Q0275	966
YPL172C	YAL039C	931
YPL172C	YER141W	995
YPL172C	YHR051W	905
YPL172C	YIL157C	963
YPL172C	YLR203C	913
YPL172C	YPL132W	995
YPL174C	YDR106W	976
YPL174C	YDR488C	957
YPL174C	YEL061C	974
YPL174C	YHR129C	999
YPL174C	YIL144W	948
YPL174C	YLL049W	984
YPL174C	YMR294W	999
YPL174C	YNL271C	977
YPL174C	YPL269W	982
YPL175W	YGR216C	991
YPL175W	YMR281W	910
YPL175W	YNL038W	950
YPL175W	YPL076W	994
YPL176C	YBR290W	943
YPL176C	YER125W	968
YPL176C	YLL039C	956
YPL177C	YGR184C	998
YPL177C	YPR194C	902
YPL178W	YBL026W	911
YPL178W	YBR119W	999
YPL178W	YDL087C	999
YPL178W	YDR235W	970
YPL178W	YDR240C	999
YPL178W	YDR364C	956
YPL178W	YDR404C	901
YPL178W	YDR432W	999

YPL178W	YER029C	961
YPL178W	YFL017W-A	905
YPL178W	YGL049C	914
YPL178W	YGL070C	907
YPL178W	YGR013W	999
YPL178W	YGR074W	906
YPL178W	YHL022C	976
YPL178W	YHR086W	999
YPL178W	YHR165C	915
YPL178W	YIL021W	983
YPL178W	YIL061C	999
YPL178W	YKL074C	952
YPL178W	YKL214C	987
YPL178W	YLR147C	983
YPL178W	YLR275W	999
YPL178W	YLR347C	999
YPL178W	YML049C	985
YPL178W	YMR125W	999
YPL178W	YMR288W	904
YPL178W	YNL189W	999
YPL178W	YNL251C	984
YPL178W	YOR116C	982
YPL178W	YOR210W	915
YPL178W	YOR224C	900
YPL178W	YOR319W	915
YPL178W	YPL190C	997
YPL178W	YPR025C	906
YPL178W	YPR082C	913
YPL178W	YPR182W	911
YPL178W	YPR187W	914
YPL180W	YHR186C	990
YPL180W	YJR066W	998
YPL180W	YKL203C	915
YPL180W	YNL006W	987
YPL181W	YAL013W	999
YPL181W	YBR095C	999
YPL181W	YBR103W	974
YPL181W	YBR112C	936
YPL181W	YCR033W	955
YPL181W	YDL076C	999
YPL181W	YDR207C	998
YPL181W	YGL194C	975
YPL181W	YIL084C	999
YPL181W	YKL185W	993

YPL181W	YKR029C	956
YPL181W	YMR263W	999
YPL181W	YNL097C	999
YPL181W	YNL330C	999
YPL181W	YOL004W	999
YPL181W	YOL012C	963
YPL181W	YPL139C	999
YPL183C	YBR061C	976
YPL183W-A	YGR220C	926
YPL186C	YAL008W	913
YPL187W	YFL026W	995
YPL187W	YGL089C	906
YPL188W	YEL041W	998
YPL188W	YJR049C	999
YPL190C	YDL175C	981
YPL190C	YER165W	973
YPL190C	YGL049C	970
YPL190C	YJL050W	997
YPL190C	YLR430W	998
YPL190C	YML117W	966
YPL190C	YMR125W	998
YPL190C	YNL251C	999
YPL190C	YOL115W	993
YPL190C	YPL178W	997
YPL193W	YFR032C-A	924
YPL193W	YHR034C	912
YPL193W	YLR075W	939
YPL193W	YNR038W	945
YPL194W	YBL088C	999
YPL194W	YBR136W	997
YPL194W	YBR160W	916
YPL194W	YDL220C	956
YPL194W	YER173W	999
YPL194W	YJL090C	952
YPL194W	YJR043C	964
YPL194W	YKL113C	968
YPL194W	YLR288C	999
YPL194W	YOR144C	964
YPL194W	YOR368W	999
YPL194W	YPL153C	958
YPL195W	YBR288C	999
YPL195W	YDR080W	976
YPL195W	YDR320C	911
YPL195W	YGR261C	999

YPL195W	YHR004C	905
YPL195W	YJL024C	999
YPL195W	YMR123W	917
YPL198W	YAL035W	998
YPL198W	YBL027W	930
YPL198W	YBL072C	995
YPL198W	YBL087C	934
YPL198W	YBL092W	955
YPL198W	YBR031W	997
YPL198W	YBR048W	994
YPL198W	YBR084C-A	930
YPL198W	YBR118W	913
YPL198W	YBR142W	964
YPL198W	YBR181C	907
YPL198W	YBR189W	931
YPL198W	YBR191W	926
YPL198W	YCR031C	930
YPL198W	YDL014W	941
YPL198W	YDL031W	939
YPL198W	YDL040C	972
YPL198W	YDL061C	912
YPL198W	YDL075W	994
YPL198W	YDL081C	938
YPL198W	YDL082W	997
YPL198W	YDL130W	913
YPL198W	YDL136W	926
YPL198W	YDL191W	926
YPL198W	YDR012W	997
YPL198W	YDR025W	996
YPL198W	YDR064W	997
YPL198W	YDR382W	940
YPL198W	YDR385W	913
YPL198W	YDR418W	943
YPL198W	YDR447C	995
YPL198W	YDR450W	920
YPL198W	YDR471W	996
YPL198W	YDR496C	969
YPL198W	YDR500C	916
YPL198W	YEL054C	937
YPL198W	YER006W	942
YPL198W	YER056C-A	910
YPL198W	YER074W	996
YPL198W	YER102W	995
YPL198W	YER117W	934

YPL198W	YER131W	923
YPL198W	YFL034C-A	920
YPL198W	YFR031C-A	997
YPL198W	YFR032C-A	938
YPL198W	YGL030W	945
YPL198W	YGL031C	908
YPL198W	YGL076C	999
YPL198W	YGL103W	993
YPL198W	YGL123W	951
YPL198W	YGL135W	997
YPL198W	YGL147C	997
YPL198W	YGL189C	923
YPL198W	YGR027C	927
YPL198W	YGR034W	934
YPL198W	YGR085C	997
YPL198W	YGR118W	932
YPL198W	YGR148C	908
YPL198W	YGR214W	925
YPL198W	YHL001W	997
YPL198W	YHL015W	940
YPL198W	YHL033C	997
YPL198W	YHR010W	997
YPL198W	YHR021C	930
YPL198W	YHR088W	927
YPL198W	YHR141C	935
YPL198W	YHR203C	997
YPL198W	YIL018W	996
YPL198W	YIL052C	910
YPL198W	YIL069C	996
YPL198W	YIL133C	997
YPL198W	YIL148W	906
YPL198W	YJL136C	934
YPL198W	YJL177W	995
YPL198W	YJL189W	909
YPL198W	YJL190C	997
YPL198W	YJL191W	930
YPL198W	YJR094W-A	929
YPL198W	YJR123W	940
YPL198W	YJR145C	995
YPL198W	YKL006W	934
YPL198W	YKL009W	947
YPL198W	YKL156W	930
YPL198W	YKL180W	995
YPL198W	YKR057W	934

YPL198W	YKR081C	947
YPL198W	YKR094C	906
YPL198W	YLL008W	929
YPL198W	YLL045C	997
YPL198W	YLR002C	946
YPL198W	YLR029C	997
YPL198W	YLR048W	933
YPL198W	YLR061W	923
YPL198W	YLR075W	997
YPL198W	YLR150W	960
YPL198W	YLR167W	900
YPL198W	YLR185W	917
YPL198W	YLR264W	912
YPL198W	YLR287C-A	908
YPL198W	YLR325C	923
YPL198W	YLR333C	926
YPL198W	YLR340W	950
YPL198W	YLR344W	933
YPL198W	YLR367W	930
YPL198W	YLR388W	920
YPL198W	YLR406C	926
YPL198W	YLR432W	978
YPL198W	YLR441C	997
YPL198W	YLR448W	997
YPL198W	YLR449W	978
YPL198W	YML024W	933
YPL198W	YML026C	920
YPL198W	YML056C	980
YPL198W	YML063W	997
YPL198W	YML073C	997
YPL198W	YMR121C	929
YPL198W	YMR142C	997
YPL198W	YMR194W	996
YPL198W	YMR230W	936
YPL198W	YMR242C	995
YPL198W	YMR260C	918
YPL198W	YMR290C	966
YPL198W	YNL061W	951
YPL198W	YNL067W	933
YPL198W	YNL069C	997
YPL198W	YNL096C	935
YPL198W	YNL132W	945
YPL198W	YNL162W	935
YPL198W	YNL178W	998

YPL198W	YNL301C	924
YPL198W	YNL302C	928
YPL198W	YNR037C	904
YPL198W	YOL039W	910
YPL198W	YOL040C	957
YPL198W	YOL041C	965
YPL198W	YOL077C	972
YPL198W	YOL120C	998
YPL198W	YOL121C	928
YPL198W	YOL127W	996
YPL198W	YOR063W	997
YPL198W	YOR096W	995
YPL198W	YOR133W	913
YPL198W	YOR167C	912
YPL198W	YOR182C	908
YPL198W	YOR234C	931
YPL198W	YOR272W	937
YPL198W	YOR293W	936
YPL198W	YOR312C	997
YPL198W	YOR369C	945
YPL198W	YPL043W	936
YPL198W	YPL079W	926
YPL198W	YPL081W	921
YPL198W	YPL090C	995
YPL198W	YPL093W	935
YPL198W	YPL131W	998
YPL198W	YPL143W	931
YPL198W	YPL211W	947
YPL198W	YPL220W	996
YPL198W	YPL249C-A	932
YPL198W	YPR043W	929
YPL198W	YPR080W	913
YPL198W	YPR102C	927
YPL198W	YPR132W	932
YPL199C	YPL240C	902
YPL200W	YJL019W	945
YPL200W	YOL104C	935
YPL201C	YER062C	992
YPL201C	YIL053W	934
YPL202C	YGL071W	999
YPL202C	YKR052C	927
YPL202C	YLR034C	917
YPL202C	YMR058W	985
YPL203W	YBR274W	923

YPL203W	YER109C	922
YPL203W	YGL116W	903
YPL203W	YHR214W-A	901
YPL203W	YIL033C	999
YPL203W	YJL164C	999
YPL203W	YKL038W	930
YPL203W	YKL062W	982
YPL203W	YKL166C	999
YPL203W	YML001W	923
YPL203W	YMR016C	987
YPL203W	YMR037C	981
YPL203W	YNL098C	999
YPL203W	YNL227C	923
YPL203W	YOR140W	995
YPL204W	YBR247C	999
YPL204W	YDL060W	997
YPL204W	YER106W	987
YPL204W	YHR185C	977
YPL204W	YKL143W	953
YPL204W	YMR153W	971
YPL204W	YNL027W	950
YPL204W	YNL178W	977
YPL204W	YNL207W	999
YPL204W	YNR026C	977
YPL204W	YPL266W	991
YPL204W	YPR181C	900
YPL206C	YML103C	935
YPL207W	YLR196W	946
YPL208W	YBL087C	999
YPL208W	YDR361C	999
YPL209C	YAR014C	952
YPL209C	YBR010W	976
YPL209C	YBR156C	999
YPL209C	YDL028C	984
YPL209C	YDR201W	929
YPL209C	YEL061C	997
YPL209C	YER133W	999
YPL209C	YFR003C	995
YPL209C	YGL061C	983
YPL209C	YGL116W	938
YPL209C	YGL240W	984
YPL209C	YGR113W	996
YPL209C	YGR140W	999
YPL209C	YGR179C	973

YPL209C	YGR188C	914
YPL209C	YIL144W	928
YPL209C	YJL030W	924
YPL209C	YJR089W	998
YPL209C	YKL193C	999
YPL209C	YKR037C	990
YPL209C	YMR001C	984
YPL209C	YMR168C	913
YPL209C	YMR311C	998
YPL209C	YNL172W	963
YPL209C	YOL069W	921
YPL209C	YOR329C	931
YPL210C	YDL051W	999
YPL210C	YDL092W	999
YPL210C	YER019C-A	923
YPL210C	YKL122C	999
YPL210C	YML105C	999
YPL210C	YPL243W	999
YPL210C	YPR088C	999
YPL211W	YBR142W	980
YPL211W	YCL054W	975
YPL211W	YCR035C	941
YPL211W	YDL031W	989
YPL211W	YDR060W	999
YPL211W	YDR087C	995
YPL211W	YDR101C	987
YPL211W	YDR471W	938
YPL211W	YDR496C	993
YPL211W	YER006W	998
YPL211W	YFL002C	993
YPL211W	YGL111W	995
YPL211W	YGL135W	938
YPL211W	YGR014W	900
YPL211W	YGR103W	999
YPL211W	YHR052W	998
YPL211W	YHR066W	994
YPL211W	YHR088W	981
YPL211W	YHR197W	993
YPL211W	YKL009W	993
YPL211W	YKL172W	983
YPL211W	YKR081C	988
YPL211W	YLL008W	987
YPL211W	YLL034C	994
YPL211W	YLL045C	954

YPL211W	YLR002C	992
YPL211W	YLR276C	979
YPL211W	YLR449W	979
YPL211W	YMR049C	988
YPL211W	YMR290C	997
YPL211W	YNL002C	979
YPL211W	YNL061W	999
YPL211W	YNL110C	994
YPL211W	YOL077C	999
YPL211W	YOL127W	940
YPL211W	YOL144W	995
YPL211W	YOR063W	936
YPL211W	YOR206W	991
YPL211W	YOR272W	999
YPL211W	YPL043W	983
YPL211W	YPL093W	993
YPL211W	YPL131W	963
YPL211W	YPL146C	945
YPL211W	YPL198W	947
YPL211W	YPR016C	978
YPL212C	YKL205W	981
YPL213W	YAL032C	999
YPL213W	YBR055C	978
YPL213W	YBR152W	928
YPL213W	YDL030W	999
YPL213W	YDL043C	998
YPL213W	YDL098C	974
YPL213W	YDL209C	998
YPL213W	YDR416W	998
YPL213W	YDR482C	906
YPL213W	YER029C	999
YPL213W	YER172C	998
YPL213W	YFL017W-A	985
YPL213W	YGR074W	999
YPL213W	YGR091W	976
YPL213W	YHR086W	948
YPL213W	YHR165C	999
YPL213W	YIR009W	999
YPL213W	YJL203W	999
YPL213W	YJR050W	989
YPL213W	YKL173W	996
YPL213W	YLL036C	999
YPL213W	YLR117C	999
YPL213W	YLR147C	998

YPL213W	YLR275W	999
YPL213W	YLR424W	974
YPL213W	YML046W	995
YPL213W	YML049C	999
YPL213W	YMR125W	978
YPL213W	YMR213W	953
YPL213W	YMR240C	999
YPL213W	YMR288W	999
YPL213W	YOR159C	999
YPL213W	YOR308C	977
YPL213W	YOR319W	935
YPL213W	YPL151C	998
YPL213W	YPR082C	976
YPL213W	YPR101W	998
YPL213W	YPR178W	983
YPL213W	YPR182W	962
YPL214C	YOL055C	995
YPL214C	YPL258C	994
YPL215W	YGR174C	994
YPL217C	YBL004W	998
YPL217C	YBR048W	939
YPL217C	YBR247C	999
YPL217C	YCL031C	922
YPL217C	YCL059C	999
YPL217C	YCR057C	999
YPL217C	YDL014W	995
YPL217C	YDL148C	999
YPL217C	YDR064W	942
YPL217C	YDR299W	998
YPL217C	YDR324C	998
YPL217C	YDR449C	998
YPL217C	YEL026W	962
YPL217C	YER082C	999
YPL217C	YGL019W	915
YPL217C	YGL120C	966
YPL217C	YGL171W	938
YPL217C	YGR081C	920
YPL217C	YGR090W	998
YPL217C	YGR128C	998
YPL217C	YGR145W	998
YPL217C	YHR148W	998
YPL217C	YHR169W	930
YPL217C	YHR196W	999
YPL217C	YJL010C	983

YPL217C	YJL069C	922
YPL217C	YJL109C	999
YPL217C	YJR002W	999
YPL217C	YJR145C	929
YPL217C	YKR060W	923
YPL217C	YLL011W	998
YPL217C	YLR003C	936
YPL217C	YLR129W	998
YPL217C	YLR175W	914
YPL217C	YLR186W	998
YPL217C	YLR197W	998
YPL217C	YLR222C	998
YPL217C	YLR409C	998
YPL217C	YML093W	916
YPL217C	YMR093W	998
YPL217C	YMR128W	998
YPL217C	YMR229C	936
YPL217C	YMR290C	966
YPL217C	YNL075W	932
YPL217C	YNL132W	996
YPL217C	YOL010W	999
YPL217C	YOR004W	911
YPL217C	YOR078W	979
YPL217C	YOR096W	942
YPL217C	YOR145C	962
YPL217C	YPL012W	998
YPL217C	YPL126W	998
YPL217C	YPL266W	923
YPL217C	YPR112C	998
YPL217C	YPR137W	999
YPL217C	YPR144C	999
YPL218W	YCR067C	998
YPL218W	YDL195W	997
YPL218W	YGL200C	974
YPL218W	YHR098C	955
YPL218W	YIL004C	999
YPL218W	YIL109C	999
YPL218W	YLR026C	913
YPL218W	YLR208W	974
YPL218W	YLR268W	977
YPL218W	YNL049C	958
YPL218W	YNR026C	999
YPL218W	YPL085W	999
YPL218W	YPR181C	999

YPL219W	YBR160W	949
YPL219W	YDL179W	981
YPL219W	YER059W	975
YPL219W	YGL215W	961
YPL219W	YMR311C	965
YPL219W	YOL096C	915
YPL219W	YOR178C	903
YPL219W	YPL031C	999
YPL219W	YPL256C	940
YPL220W	YAL035W	914
YPL220W	YBL027W	914
YPL220W	YBL072C	916
YPL220W	YBL087C	911
YPL220W	YBL092W	937
YPL220W	YBR031W	997
YPL220W	YBR048W	956
YPL220W	YBR084C-A	999
YPL220W	YBR118W	926
YPL220W	YBR181C	994
YPL220W	YBR189W	943
YPL220W	YBR191W	990
YPL220W	YCR031C	964
YPL220W	YDL061C	966
YPL220W	YDL075W	999
YPL220W	YDL081C	938
YPL220W	YDL082W	995
YPL220W	YDL130W	935
YPL220W	YDL136W	999
YPL220W	YDL191W	918
YPL220W	YDR012W	998
YPL220W	YDR025W	904
YPL220W	YDR064W	998
YPL220W	YDR382W	999
YPL220W	YDR385W	906
YPL220W	YDR418W	999
YPL220W	YDR447C	996
YPL220W	YDR450W	918
YPL220W	YDR471W	997
YPL220W	YDR496C	939
YPL220W	YDR500C	999
YPL220W	YEL054C	961
YPL220W	YER056C-A	993
YPL220W	YER074W	906
YPL220W	YER102W	999

YPL220W	YER117W	999
YPL220W	YER131W	924
YPL220W	YFL034C-A	920
YPL220W	YFR031C-A	995
YPL220W	YFR032C-A	928
YPL220W	YGL030W	999
YPL220W	YGL076C	996
YPL220W	YGL103W	998
YPL220W	YGL123W	972
YPL220W	YGL135W	999
YPL220W	YGL147C	914
YPL220W	YGL189C	914
YPL220W	YGR027C	941
YPL220W	YGR034W	996
YPL220W	YGR085C	997
YPL220W	YGR118W	977
YPL220W	YGR148C	939
YPL220W	YGR214W	945
YPL220W	YHL001W	997
YPL220W	YHL015W	966
YPL220W	YHL033C	999
YPL220W	YHR010W	944
YPL220W	YHR021C	920
YPL220W	YHR026W	966
YPL220W	YHR141C	932
YPL220W	YHR203C	999
YPL220W	YIL018W	995
YPL220W	YIL052C	994
YPL220W	YIL069C	906
YPL220W	YIL133C	932
YPL220W	YIL148W	999
YPL220W	YJL136C	945
YPL220W	YJL177W	998
YPL220W	YJL189W	924
YPL220W	YJL190C	912
YPL220W	YJL191W	960
YPL220W	YJR094W-A	993
YPL220W	YJR123W	998
YPL220W	YJR145C	994
YPL220W	YKL006W	973
YPL220W	YKL156W	963
YPL220W	YKL180W	998
YPL220W	YKR057W	929
YPL220W	YKR059W	998

YPL220W	YKR094C	905
YPL220W	YLL045C	997
YPL220W	YLR029C	997
YPL220W	YLR048W	975
YPL220W	YLR061W	995
YPL220W	YLR075W	999
YPL220W	YLR167W	999
YPL220W	YLR185W	999
YPL220W	YLR264W	960
YPL220W	YLR287C-A	910
YPL220W	YLR333C	911
YPL220W	YLR340W	999
YPL220W	YLR344W	987
YPL220W	YLR367W	940
YPL220W	YLR388W	991
YPL220W	YLR406C	925
YPL220W	YLR441C	995
YPL220W	YLR448W	998
YPL220W	YML024W	996
YPL220W	YML026C	918
YPL220W	YML063W	997
YPL220W	YML073C	996
YPL220W	YMR121C	914
YPL220W	YMR142C	909
YPL220W	YMR194W	998
YPL220W	YMR230W	926
YPL220W	YMR242C	996
YPL220W	YMR260C	913
YPL220W	YMR290C	969
YPL220W	YNL061W	925
YPL220W	YNL067W	956
YPL220W	YNL069C	996
YPL220W	YNL096C	931
YPL220W	YNL162W	932
YPL220W	YNL178W	986
YPL220W	YNL301C	999
YPL220W	YNL302C	922
YPL220W	YNR037C	900
YPL220W	YOL039W	908
YPL220W	YOL040C	944
YPL220W	YOL120C	910
YPL220W	YOL121C	923
YPL220W	YOL127W	999
YPL220W	YOR063W	997

YPL220W	YOR096W	998
YPL220W	YOR133W	975
YPL220W	YOR167C	925
YPL220W	YOR182C	910
YPL220W	YOR234C	949
YPL220W	YOR293W	941
YPL220W	YOR312C	998
YPL220W	YOR369C	925
YPL220W	YPL037C	933
YPL220W	YPL079W	934
YPL220W	YPL081W	917
YPL220W	YPL090C	902
YPL220W	YPL131W	967
YPL220W	YPL143W	958
YPL220W	YPL198W	996
YPL220W	YPL249C-A	948
YPL220W	YPR043W	996
YPL220W	YPR080W	957
YPL220W	YPR102C	998
YPL220W	YPR132W	999
YPL221W	YBR199W	926
YPL221W	YJR075W	921
YPL221W	YML115C	915
YPL224C	YML062C	928
YPL227C	YAL058W	923
YPL227C	YBR243C	974
YPL227C	YCL001W	905
YPL227C	YEL002C	991
YPL227C	YFL025C	903
YPL227C	YGL226C-A	938
YPL227C	YGR036C	956
YPL227C	YJL002C	956
YPL227C	YJL034W	909
YPL227C	YMR013C	982
YPL227C	YMR149W	948
YPL227C	YOR002W	984
YPL227C	YOR085W	984
YPL227C	YPR183W	939
YPL228W	YCR084C	974
YPL228W	YDL140C	976
YPL228W	YGL130W	999
YPL231W	YKL182W	999
YPL231W	YNR016C	974
YPL232W	YAL030W	999

YPL232W	YBL050W	984
YPL232W	YDR164C	999
YPL232W	YGL233W	910
YPL232W	YGR009C	999
YPL232W	YJL164C	967
YPL232W	YMR017W	998
YPL232W	YMR183C	995
YPL232W	YNR049C	990
YPL232W	YOR242C	918
YPL232W	YOR327C	999
YPL232W	YPR032W	975
YPL233W	YAL034W-A	999
YPL233W	YBR069C	993
YPL233W	YBR107C	906
YPL233W	YBR211C	977
YPL233W	YDR254W	906
YPL233W	YDR318W	906
YPL233W	YDR383C	901
YPL233W	YGL060W	981
YPL233W	YGR140W	906
YPL233W	YGR179C	991
YPL233W	YIR010W	999
YPL233W	YJR112W	999
YPL233W	YJR135C	906
YPL233W	YKL089W	981
YPL233W	YLR315W	906
YPL233W	YLR381W	906
YPL233W	YOR048C	923
YPL233W	YPL018W	965
YPL233W	YPR046W	906
YPL234C	YBR011C	961
YPL234C	YBR127C	977
YPL234C	YDL185W	994
YPL234C	YEL027W	994
YPL234C	YEL051W	940
YPL234C	YGR020C	920
YPL234C	YGR105W	976
YPL234C	YHR026W	993
YPL234C	YHR039C-A	918
YPL234C	YKL080W	924
YPL234C	YLR447C	956
YPL234C	YOR270C	967
YPL234C	YOR332W	953
YPL234C	YPR036W	969

YPL235W	YAL011W	999
YPL235W	YBR231C	999
YPL235W	YCR060W	999
YPL235W	YDL002C	998
YPL235W	YDR190C	999
YPL235W	YDR334W	999
YPL235W	YDR485C	999
YPL235W	YER092W	991
YPL235W	YER148W	916
YPL235W	YFL013C	991
YPL235W	YFL039C	999
YPL235W	YGL150C	999
YPL235W	YGR002C	999
YPL235W	YGR099W	959
YPL235W	YGR163W	972
YPL235W	YHR034C	999
YPL235W	YHR099W	963
YPL235W	YJL081C	999
YPL235W	YLR052W	989
YPL235W	YLR085C	998
YPL235W	YLR385C	954
YPL235W	YLR399C	970
YPL235W	YML041C	999
YPL235W	YNL059C	998
YPL235W	YNL107W	999
YPL235W	YNL215W	995
YPL235W	YOL012C	997
YPL235W	YOR136W	969
YPL235W	YOR141C	999
YPL235W	YOR310C	976
YPL235W	YPL129W	999
YPL237W	YBR079C	999
YPL237W	YBR143C	972
YPL237W	YBR159W	962
YPL237W	YBR189W	951
YPL237W	YCR031C	925
YPL237W	YDL061C	921
YPL237W	YDR047W	911
YPL237W	YDR064W	927
YPL237W	YDR211W	999
YPL237W	YDR447C	900
YPL237W	YDR450W	906
YPL237W	YER025W	999
YPL237W	YER102W	910

YPL237W	YER131W	948
YPL237W	YGL123W	920
YPL237W	YGR027C	947
YPL237W	YGR083C	999
YPL237W	YGR118W	943
YPL237W	YGR159C	931
YPL237W	YGR214W	937
YPL237W	YHL015W	922
YPL237W	YHR203C	910
YPL237W	YIL133C	905
YPL237W	YJL136C	912
YPL237W	YJL191W	923
YPL237W	YJR007W	999
YPL237W	YJR123W	929
YPL237W	YKL156W	932
YPL237W	YKR026C	999
YPL237W	YKR059W	912
YPL237W	YLR048W	921
YPL237W	YLR264W	921
YPL237W	YLR291C	999
YPL237W	YLR333C	910
YPL237W	YLR367W	925
YPL237W	YLR388W	924
YPL237W	YLR441C	900
YPL237W	YML024W	924
YPL237W	YML026C	906
YPL237W	YML063W	962
YPL237W	YMR230W	910
YPL237W	YMR260C	949
YPL237W	YMR309C	982
YPL237W	YNL069C	905
YPL237W	YNL178W	928
YPL237W	YNL244C	990
YPL237W	YNL265C	978
YPL237W	YOL040C	914
YPL237W	YOL139C	907
YPL237W	YOR096W	931
YPL237W	YOR167C	919
YPL237W	YOR260W	999
YPL237W	YOR293W	929
YPL237W	YOR361C	998
YPL237W	YOR369C	907
YPL237W	YPL081W	925
YPL237W	YPR041W	999

YPL237W	YPR132W	902
YPL237W	YPR163C	916
YPL239W	YKL143W	976
YPL239W	YNL178W	930
YPL240C	YAL005C	991
YPL240C	YAL023C	919
YPL240C	YAL024C	919
YPL240C	YAL049C	902
YPL240C	YAR015W	923
YPL240C	YBL006C	919
YPL240C	YBL051C	919
YPL240C	YBR047W	919
YPL240C	YBR155W	999
YPL240C	YBR280C	919
YPL240C	YBR290W	970
YPL240C	YCL063W	919
YPL240C	YCR060W	996
YPL240C	YCR077C	919
YPL240C	YDL077C	919
YPL240C	YDL192W	919
YPL240C	YDL197C	919
YPL240C	YDL198C	919
YPL240C	YDL199C	970
YPL240C	YDL229W	924
YPL240C	YDL239C	919
YPL240C	YDR163W	919
YPL240C	YDR168W	999
YPL240C	YDR214W	999
YPL240C	YDR276C	919
YPL240C	YDR277C	919
YPL240C	YDR283C	994
YPL240C	YDR310C	919
YPL240C	YDR328C	962
YPL240C	YDR392W	919
YPL240C	YDR443C	919
YPL240C	YDR490C	919
YPL240C	YEL003W	919
YPL240C	YEL048C	919
YPL240C	YEL053C	919
YPL240C	YEL063C	902
YPL240C	YEL064C	919
YPL240C	YER103W	929
YPL240C	YER148W	919
YPL240C	YER151C	919

YPL240C	YFL011W	970
YPL240C	YFL021W	919
YPL240C	YFL039C	902
YPL240C	YFL054C	919
YPL240C	YFR010W	934
YPL240C	YFR036W	919
YPL240C	YGL073W	969
YPL240C	YGL212W	919
YPL240C	YGR063C	919
YPL240C	YGR123C	994
YPL240C	YGR180C	919
YPL240C	YGR229C	944
YPL240C	YGR289C	902
YPL240C	YHL036W	902
YPL240C	YHR027C	939
YPL240C	YHR081W	919
YPL240C	YHR135C	902
YPL240C	YHR156C	919
YPL240C	YHR176W	919
YPL240C	YIL006W	919
YPL240C	YIL021W	919
YPL240C	YIL041W	902
YPL240C	YIL052C	920
YPL240C	YIL053W	919
YPL240C	YIL065C	919
YPL240C	YIL076W	919
YPL240C	YIL166C	919
YPL240C	YJL030W	919
YPL240C	YJL123C	919
YPL240C	YJL170C	902
YPL240C	YJR032W	999
YPL240C	YJR040W	919
YPL240C	YJR056C	919
YPL240C	YJR073C	919
YPL240C	YJR088C	919
YPL240C	YKL037W	919
YPL240C	YKL117W	999
YPL240C	YKL126W	991
YPL240C	YKL135C	919
YPL240C	YKL212W	919
YPL240C	YKR035W-A	919
YPL240C	YKR067W	902
YPL240C	YLL011W	919
YPL240C	YLL018C	914

YPL240C	YLL024C	991
YPL240C	YLR070C	902
YPL240C	YLR087C	919
YPL240C	YLR090W	919
YPL240C	YLR099C	919
YPL240C	YLR113W	950
YPL240C	YLR119W	919
YPL240C	YLR180W	919
YPL240C	YLR216C	999
YPL240C	YLR256W	999
YPL240C	YLR259C	901
YPL240C	YLR262C	919
YPL240C	YLR362W	988
YPL240C	YLR393W	919
YPL240C	YLR398C	919
YPL240C	YLR404W	902
YPL240C	YLR417W	919
YPL240C	YLR438W	902
YPL240C	YML001W	970
YPL240C	YML071C	919
YPL240C	YML073C	923
YPL240C	YMR070W	919
YPL240C	YMR091C	968
YPL240C	YMR094W	958
YPL240C	YMR095C	919
YPL240C	YMR154C	919
YPL240C	YMR157C	919
YPL240C	YMR186W	999
YPL240C	YMR214W	931
YPL240C	YMR277W	919
YPL240C	YMR311C	919
YPL240C	YNL032W	919
YPL240C	YNL041C	919
YPL240C	YNL058C	919
YPL240C	YNL064C	999
YPL240C	YNL098C	919
YPL240C	YNL229C	946
YPL240C	YNL236W	963
YPL240C	YNL281W	999
YPL240C	YNL298W	919
YPL240C	YNL307C	919
YPL240C	YNL325C	919
YPL240C	YNR020C	919
YPL240C	YNR051C	919

YPL240C	YNR071C	919
YPL240C	YOL111C	919
YPL240C	YOL135C	990
YPL240C	YOL156W	919
YPL240C	YOR027W	999
YPL240C	YOR036W	945
YPL240C	YOR057W	999
YPL240C	YOR061W	958
YPL240C	YOR216C	919
YPL240C	YOR290C	926
YPL240C	YOR327C	919
YPL240C	YOR332W	970
YPL240C	YPL016W	959
YPL240C	YPL106C	990
YPL240C	YPL141C	919
YPL240C	YPL156C	902
YPL240C	YPL161C	968
YPL240C	YPL199C	902
YPL240C	YPL257W	902
YPL240C	YPR046W	919
YPL241C	YAL011W	918
YPL241C	YBR231C	909
YPL241C	YCL029C	965
YPL241C	YDR334W	931
YPL241C	YDR485C	952
YPL241C	YEL061C	976
YPL241C	YFL037W	903
YPL241C	YGL086W	946
YPL241C	YJL013C	930
YPL241C	YJL030W	991
YPL241C	YLR085C	939
YPL241C	YML041C	954
YPL241C	YML124C	929
YPL241C	YMR138W	998
YPL241C	YOR349W	987
YPL242C	YAR019C	929
YPL242C	YBR109C	995
YPL242C	YCR002C	939
YPL242C	YDL117W	951
YPL242C	YDR085C	905
YPL242C	YFL039C	989
YPL242C	YGL106W	999
YPL242C	YGR092W	910
YPL242C	YHR023W	997

YPL242C	YHR107C	961
YPL242C	YJR092W	969
YPL242C	YLR229C	984
YPL242C	YML064C	976
YPL242C	YPR111W	907
YPL242C	YPR188C	913
YPL243W	YDL051W	999
YPL243W	YDL092W	999
YPL243W	YKL122C	999
YPL243W	YML105C	999
YPL243W	YPL210C	999
YPL243W	YPR088C	999
YPL246C	YGL212W	941
YPL246C	YHR105W	955
YPL247C	YJL141C	930
YPL248C	YBR018C	953
YPL248C	YBR019C	934
YPL248C	YBR020W	994
YPL248C	YDL140C	967
YPL248C	YDR009W	983
YPL248C	YDR145W	992
YPL248C	YDR167W	992
YPL248C	YDR308C	920
YPL248C	YDR392W	950
YPL248C	YDR448W	986
YPL248C	YDR477W	937
YPL248C	YER022W	997
YPL248C	YER148W	999
YPL248C	YGL048C	999
YPL248C	YGL112C	999
YPL248C	YGR252W	910
YPL248C	YHR099W	986
YPL248C	YML051W	999
YPL248C	YNL025C	976
YPL248C	YOL051W	999
YPL248C	YOR259C	996
YPL248C	YPL042C	999
YPL248C	YPR086W	998
YPL249C	YCR009C	995
YPL249C	YDR388W	999
YPL249C	YFL038C	932
YPL249C	YMR192W	996
YPL249C-A	YBL027W	996
YPL249C-A	YBL072C	927

YPL249C-A	YBL087C	930
YPL249C-A	YBL092W	938
YPL249C-A	YBR031W	997
YPL249C-A	YBR048W	914
YPL249C-A	YBR084C-A	950
YPL249C-A	YBR118W	909
YPL249C-A	YBR142W	982
YPL249C-A	YBR181C	902
YPL249C-A	YBR189W	942
YPL249C-A	YBR191W	960
YPL249C-A	YCR031C	997
YPL249C-A	YDL061C	939
YPL249C-A	YDL075W	997
YPL249C-A	YDL081C	975
YPL249C-A	YDL082W	997
YPL249C-A	YDL083C	972
YPL249C-A	YDL130W	928
YPL249C-A	YDL136W	997
YPL249C-A	YDL191W	927
YPL249C-A	YDR012W	941
YPL249C-A	YDR025W	914
YPL249C-A	YDR064W	998
YPL249C-A	YDR101C	983
YPL249C-A	YDR382W	997
YPL249C-A	YDR385W	912
YPL249C-A	YDR418W	997
YPL249C-A	YDR447C	997
YPL249C-A	YDR450W	918
YPL249C-A	YDR471W	997
YPL249C-A	YDR500C	956
YPL249C-A	YEL054C	997
YPL249C-A	YER006W	929
YPL249C-A	YER056C-A	957
YPL249C-A	YER074W	996
YPL249C-A	YER102W	952
YPL249C-A	YER117W	955
YPL249C-A	YER131W	932
YPL249C-A	YFL034C-A	919
YPL249C-A	YFR031C-A	996
YPL249C-A	YFR032C-A	950
YPL249C-A	YGL030W	998
YPL249C-A	YGL031C	995
YPL249C-A	YGL076C	997
YPL249C-A	YGL103W	997

YPL249C-A	YGL123W	933
YPL249C-A	YGL135W	996
YPL249C-A	YGL147C	996
YPL249C-A	YGL189C	920
YPL249C-A	YGR027C	945
YPL249C-A	YGR034W	996
YPL249C-A	YGR085C	997
YPL249C-A	YGR103W	925
YPL249C-A	YGR118W	954
YPL249C-A	YGR148C	921
YPL249C-A	YGR214W	945
YPL249C-A	YHL001W	997
YPL249C-A	YHL015W	955
YPL249C-A	YHL033C	952
YPL249C-A	YHR010W	998
YPL249C-A	YHR021C	931
YPL249C-A	YHR141C	931
YPL249C-A	YHR203C	998
YPL249C-A	YIL018W	996
YPL249C-A	YIL052C	954
YPL249C-A	YIL069C	923
YPL249C-A	YIL133C	996
YPL249C-A	YIL148W	951
YPL249C-A	YJL136C	941
YPL249C-A	YJL177W	997
YPL249C-A	YJL189W	940
YPL249C-A	YJL190C	915
YPL249C-A	YJL191W	962
YPL249C-A	YJR094W-A	969
YPL249C-A	YJR123W	965
YPL249C-A	YJR145C	926
YPL249C-A	YKL006W	967
YPL249C-A	YKL009W	934
YPL249C-A	YKL156W	959
YPL249C-A	YKL180W	997
YPL249C-A	YKR057W	935
YPL249C-A	YKR094C	906
YPL249C-A	YLL045C	997
YPL249C-A	YLR029C	929
YPL249C-A	YLR048W	948
YPL249C-A	YLR061W	967
YPL249C-A	YLR075W	998
YPL249C-A	YLR150W	978
YPL249C-A	YLR167W	947

YPL249C-A	YLR185W	979
YPL249C-A	YLR264W	938
YPL249C-A	YLR287C-A	902
YPL249C-A	YLR325C	998
YPL249C-A	YLR333C	929
YPL249C-A	YLR340W	998
YPL249C-A	YLR344W	959
YPL249C-A	YLR367W	998
YPL249C-A	YLR388W	958
YPL249C-A	YLR406C	922
YPL249C-A	YLR441C	932
YPL249C-A	YLR448W	998
YPL249C-A	YML024W	965
YPL249C-A	YML026C	918
YPL249C-A	YML063W	998
YPL249C-A	YML073C	997
YPL249C-A	YMR121C	913
YPL249C-A	YMR142C	996
YPL249C-A	YMR194W	953
YPL249C-A	YMR230W	931
YPL249C-A	YMR242C	997
YPL249C-A	YNL002C	929
YPL249C-A	YNL067W	943
YPL249C-A	YNL069C	996
YPL249C-A	YNL096C	921
YPL249C-A	YNL162W	931
YPL249C-A	YNL178W	971
YPL249C-A	YNL301C	998
YPL249C-A	YNL302C	927
YPL249C-A	YNR053C	978
YPL249C-A	YOL039W	913
YPL249C-A	YOL040C	956
YPL249C-A	YOL120C	935
YPL249C-A	YOL121C	927
YPL249C-A	YOL127W	997
YPL249C-A	YOR063W	995
YPL249C-A	YOR096W	995
YPL249C-A	YOR133W	925
YPL249C-A	YOR167C	912
YPL249C-A	YOR182C	902
YPL249C-A	YOR204W	927
YPL249C-A	YOR234C	954
YPL249C-A	YOR293W	940
YPL249C-A	YOR312C	930

YPL249C-A	YOR369C	936
YPL249C-A	YPL079W	928
YPL249C-A	YPL081W	926
YPL249C-A	YPL090C	902
YPL249C-A	YPL093W	931
YPL249C-A	YPL131W	996
YPL249C-A	YPL143W	999
YPL249C-A	YPL198W	932
YPL249C-A	YPL220W	948
YPL249C-A	YPR016C	925
YPL249C-A	YPR043W	946
YPL249C-A	YPR080W	909
YPL249C-A	YPR102C	931
YPL249C-A	YPR132W	957
YPL252C	YCL017C	989
YPL252C	YDR376W	949
YPL252C	YER141W	982
YPL252C	YGL018C	932
YPL252C	YLL027W	942
YPL253C	YHR191C	923
YPL253C	YMR198W	984
YPL253C	YPR141C	973
YPL254W	YBR081C	999
YPL254W	YBR111W-A	966
YPL254W	YBR198C	999
YPL254W	YCL010C	999
YPL254W	YDR145W	999
YPL254W	YDR167W	999
YPL254W	YDR176W	999
YPL254W	YDR392W	999
YPL254W	YDR448W	999
YPL254W	YER040W	961
YPL254W	YER148W	994
YPL254W	YER164W	980
YPL254W	YGL066W	999
YPL254W	YGL112C	999
YPL254W	YGR252W	999
YPL254W	YHR099W	999
YPL254W	YJR066W	901
YPL254W	YLR055C	999
YPL254W	YML114C	923
YPL254W	YMR223W	999
YPL254W	YMR227C	988
YPL254W	YMR236W	999

YPL254W	YOL148C	999
YPL254W	YPL011C	988
YPL254W	YPL031C	903
YPL254W	YPL047W	999
YPL255W	YGL075C	994
YPL255W	YNL188W	979
YPL255W	YPL124W	983
YPL256C	YAL040C	999
YPL256C	YBL016W	909
YPL256C	YBR110W	917
YPL256C	YBR135W	999
YPL256C	YBR136W	967
YPL256C	YBR160W	999
YPL256C	YCL014W	962
YPL256C	YDL126C	994
YPL256C	YDL132W	999
YPL256C	YDL179W	946
YPL256C	YDR054C	938
YPL256C	YDR099W	962
YPL256C	YDR309C	933
YPL256C	YDR328C	999
YPL256C	YER059W	942
YPL256C	YER111C	999
YPL256C	YER167W	970
YPL256C	YFL009W	981
YPL256C	YGL003C	995
YPL256C	YGL207W	910
YPL256C	YGL215W	946
YPL256C	YGR108W	983
YPL256C	YGR109C	978
YPL256C	YHL007C	996
YPL256C	YHR061C	933
YPL256C	YJL157C	999
YPL256C	YJR090C	999
YPL256C	YKL092C	981
YPL256C	YKR091W	962
YPL256C	YLL039C	962
YPL256C	YLR079W	999
YPL256C	YLR131C	962
YPL256C	YLR182W	999
YPL256C	YLR229C	909
YPL256C	YLR310C	977
YPL256C	YLR362W	909
YPL256C	YMR199W	999

YPL256C	YNL197C	913
YPL256C	YNL298W	976
YPL256C	YOL004W	962
YPL256C	YOL133W	997
YPL256C	YOR127W	962
YPL256C	YOR212W	909
YPL256C	YPL014W	962
YPL256C	YPL031C	909
YPL256C	YPL115C	994
YPL256C	YPL219W	940
YPL256C	YPL267W	962
YPL257W	YPL240C	902
YPL257W-B	YBR012W-B	998
YPL257W-B	YLR116W	998
YPL257W-B	YPR137C-B	998
YPL258C	YOL055C	969
YPL258C	YPL214C	994
YPL259C	YGL206C	988
YPL259C	YHL019C	985
YPL259C	YKL135C	999
YPL259C	YLR170C	999
YPL259C	YPR029C	999
YPL262W	YDL078C	937
YPL262W	YDR178W	948
YPL262W	YJL045W	900
YPL262W	YKL085W	990
YPL262W	YKL141W	939
YPL262W	YKL148C	900
YPL262W	YLL041C	981
YPL262W	YOL126C	939
YPL263C	YDR432W	940
YPL263C	YER165W	940
YPL266W	YBL004W	997
YPL266W	YBR189W	978
YPL266W	YBR247C	999
YPL266W	YCL059C	906
YPL266W	YCR057C	999
YPL266W	YDL014W	937
YPL266W	YDL060W	999
YPL266W	YDR064W	944
YPL266W	YDR299W	922
YPL266W	YDR324C	999
YPL266W	YDR449C	997
YPL266W	YEL026W	917

YPL266W	YER082C	997
YPL266W	YGR090W	993
YPL266W	YGR128C	974
YPL266W	YHR196W	989
YPL266W	YJL010C	983
YPL266W	YJL109C	956
YPL266W	YJR002W	997
YPL266W	YJR123W	978
YPL266W	YKL143W	970
YPL266W	YLR129W	997
YPL266W	YLR175W	910
YPL266W	YLR197W	907
YPL266W	YMR093W	999
YPL266W	YMR290C	910
YPL266W	YNL207W	999
YPL266W	YOR004W	914
YPL266W	YOR078W	970
YPL266W	YOR145C	999
YPL266W	YPL012W	999
YPL266W	YPL126W	999
YPL266W	YPL204W	991
YPL266W	YPL217C	923
YPL266W	YPR137W	990
YPL266W	YPR144C	997
YPL267W	YBR160W	961
YPL267W	YDL155W	962
YPL267W	YDR099W	977
YPL267W	YER177W	997
YPL267W	YGL003C	999
YPL267W	YGL116W	934
YPL267W	YPL256C	962
YPL268W	YDR173C	984
YPL268W	YDR208W	941
YPL268W	YKL203C	976
YPL268W	YLR305C	951
YPL268W	YNL025C	967
YPL268W	YNL267W	950
YPL268W	YOR109W	914
YPL269W	YBR160W	997
YPL269W	YCL029C	999
YPL269W	YDL064W	966
YPL269W	YDL101C	919
YPL269W	YDR150W	999
YPL269W	YDR488C	982

YPL269W	YER016W	999
YPL269W	YHR129C	998
YPL269W	YHR134W	975
YPL269W	YJR053W	992
YPL269W	YKR054C	999
YPL269W	YLL049W	986
YPL269W	YLR045C	998
YPL269W	YLR210W	986
YPL269W	YLR254C	955
YPL269W	YLR319C	953
YPL269W	YMR055C	999
YPL269W	YMR294W	999
YPL269W	YMR299C	905
YPL269W	YNL153C	912
YPL269W	YNL271C	943
YPL269W	YOR156C	972
YPL269W	YOR269W	995
YPL269W	YOR326W	999
YPL269W	YPL155C	999
YPL269W	YPL174C	982
YPL269W	YPR120C	990
YPL271W	YBL099W	999
YPL271W	YBR039W	997
YPL271W	YDL004W	997
YPL271W	YDL130W-A	911
YPL271W	YDR298C	912
YPL271W	YJR121W	999
YPL271W	YKL016C	928
YPL271W	YPL078C	999
YPL281C	YGR254W	902
YPL281C	YHR174W	902
YPL281C	YMR323W	901
YPL281C	YOR393W	901
YPR001W	YBR218C	902
YPR001W	YGL062W	902
YPR001W	YJL200C	902
YPR001W	YKL085W	901
YPR001W	YLR304C	908
YPR001W	YNR001C	970
YPR003C	YNL036W	923
YPR004C	YGR207C	999
YPR004C	YOR356W	998
YPR005C	YDR040C	914
YPR006C	YIR031C	970

YPR006C	YNL117W	971
YPR007C	YDL003W	959
YPR007C	YFL008W	984
YPR007C	YHL022C	901
YPR007C	YIR025W	914
YPR007C	YJL074C	988
YPR007C	YMR001C	986
YPR010C	YAL038W	912
YPR010C	YBL039C	915
YPR010C	YBR154C	999
YPR010C	YDL150W	921
YPR010C	YDR045C	928
YPR010C	YDR156W	998
YPR010C	YDR211W	972
YPR010C	YGL245W	975
YPR010C	YGR094W	916
YPR010C	YHR143W-A	999
YPR010C	YIL143C	900
YPR010C	YJL076W	954
YPR010C	YJL148W	999
YPR010C	YJR063W	999
YPR010C	YKL067W	995
YPR010C	YKL104C	972
YPR010C	YKL125W	984
YPR010C	YKL144C	921
YPR010C	YKL213C	935
YPR010C	YLL034C	935
YPR010C	YML010W	942
YPR010C	YNL113W	999
YPR010C	YNL151C	921
YPR010C	YNL248C	999
YPR010C	YNR003C	921
YPR010C	YOR116C	946
YPR010C	YOR151C	995
YPR010C	YOR207C	933
YPR010C	YOR210W	999
YPR010C	YOR224C	998
YPR010C	YOR340C	999
YPR010C	YOR341W	999
YPR010C	YPR110C	999
YPR010C	YPR187W	998
YPR010C	YPR190C	921
YPR016C	YBR267W	949
YPR016C	YCR072C	995

YPR016C	YDL031W	999
YPR016C	YDR087C	999
YPR016C	YDR101C	998
YPR016C	YDR496C	998
YPR016C	YER002W	958
YPR016C	YER006W	999
YPR016C	YER126C	999
YPR016C	YFL002C	976
YPR016C	YFR001W	999
YPR016C	YGL030W	955
YPR016C	YGL099W	998
YPR016C	YGL111W	998
YPR016C	YGL147C	964
YPR016C	YGR103W	999
YPR016C	YGR245C	999
YPR016C	YHR052W	998
YPR016C	YHR066W	982
YPR016C	YHR088W	999
YPR016C	YHR197W	999
YPR016C	YIL018W	927
YPR016C	YJL122W	931
YPR016C	YKL009W	999
YPR016C	YKL021C	997
YPR016C	YKR081C	999
YPR016C	YLL008W	999
YPR016C	YLL045C	941
YPR016C	YLR002C	999
YPR016C	YLR009W	963
YPR016C	YLR022C	990
YPR016C	YLR074C	973
YPR016C	YMR049C	999
YPR016C	YMR290C	998
YPR016C	YNL002C	999
YPR016C	YNL061W	999
YPR016C	YNL110C	999
YPR016C	YNL163C	995
YPR016C	YNL182C	998
YPR016C	YNR053C	999
YPR016C	YOL077C	927
YPR016C	YOL127W	933
YPR016C	YOR063W	990
YPR016C	YOR206W	998
YPR016C	YOR272W	985
YPR016C	YPL093W	999

YPR016C	YPL131W	997
YPR016C	YPL211W	978
YPR016C	YPL249C-A	925
YPR017C	YFL005W	999
YPR017C	YFL038C	999
YPR017C	YGL233W	960
YPR017C	YML077W	902
YPR017C	YNL272C	961
YPR017C	YPR055W	961
YPR018W	YBL008W	999
YPR018W	YBR009C	987
YPR018W	YBR010W	985
YPR018W	YBR088C	976
YPR018W	YBR195C	999
YPR018W	YDL101C	974
YPR018W	YHR118C	998
YPR018W	YJL115W	999
YPR018W	YKR101W	981
YPR018W	YLL004W	998
YPR018W	YLL036C	906
YPR018W	YLR347C	915
YPR018W	YML102W	999
YPR018W	YMR190C	969
YPR018W	YNL261W	998
YPR019W	YAR007C	939
YPR019W	YBL023C	999
YPR019W	YBR060C	999
YPR019W	YBR202W	999
YPR019W	YDL017W	999
YPR019W	YDR052C	983
YPR019W	YDR143C	929
YPR019W	YDR489W	980
YPR019W	YEL032W	999
YPR019W	YGL201C	999
YPR019W	YIL150C	993
YPR019W	YJL194W	994
YPR019W	YJR046W	983
YPR019W	YLR103C	996
YPR019W	YLR274W	998
YPR019W	YML065W	995
YPR019W	YNL102W	940
YPR019W	YNL261W	999
YPR019W	YNL262W	904
YPR019W	YNL312W	902

YPR019W	YPR162C	905
YPR019W	YPR175W	904
YPR020W	YBL099W	999
YPR020W	YJR121W	999
YPR020W	YKL016C	928
YPR020W	YML081C-A	957
YPR020W	YPL078C	999
YPR023C	YBR095C	997
YPR023C	YDR359C	999
YPR023C	YEL018W	999
YPR023C	YEL056W	916
YPR023C	YFL024C	999
YPR023C	YFL039C	969
YPR023C	YGR002C	998
YPR023C	YHR090C	997
YPR023C	YHR099W	996
YPR023C	YIL084C	993
YPR023C	YJL081C	999
YPR023C	YJR082C	997
YPR023C	YML041C	955
YPR023C	YMR075W	999
YPR023C	YNL097C	987
YPR023C	YNL107W	999
YPR023C	YNL136W	999
YPR023C	YNL330C	999
YPR023C	YOL004W	999
YPR023C	YOL012C	916
YPR023C	YOR244W	999
YPR023C	YPL001W	940
YPR023C	YPL139C	999
YPR023C	YPR161C	904
YPR024W	YBL030C	905
YPR024W	YBL099W	983
YPR024W	YBR039W	984
YPR024W	YCL044C	991
YPR024W	YDL181W	903
YPR024W	YER154W	903
YPR024W	YJL054W	900
YPR024W	YLR059C	933
YPR024W	YMR302C	999
YPR025C	YBR160W	907
YPR025C	YDL108W	999
YPR025C	YDL140C	937
YPR025C	YDL155W	920

YPR025C	YDR311W	993
YPR025C	YDR460W	999
YPR025C	YDR508C	998
YPR025C	YEL037C	900
YPR025C	YER068W	979
YPR025C	YER171W	996
YPR025C	YGR108W	900
YPR025C	YIL143C	934
YPR025C	YJR035W	900
YPR025C	YLR005W	957
YPR025C	YLR210W	919
YPR025C	YOL051W	959
YPR025C	YOR210W	900
YPR025C	YPL022W	902
YPR025C	YPL178W	906
YPR025C	YPR056W	993
YPR025C	YPR086W	906
YPR025C	YPR119W	901
YPR026W	YBR001C	986
YPR026W	YDR001C	983
YPR026W	YDR074W	924
YPR028W	YGR004W	998
YPR028W	YGR172C	985
YPR028W	YOR165W	995
YPR029C	YGL206C	988
YPR029C	YHL019C	999
YPR029C	YJL207C	910
YPR029C	YJR125C	938
YPR029C	YKL135C	999
YPR029C	YLR170C	999
YPR029C	YOR109W	974
YPR029C	YPL259C	999
YPR030W	YER125W	985
YPR030W	YOR124C	916
YPR031W	YBL052C	999
YPR031W	YJR082C	954
YPR032W	YBL106C	972
YPR032W	YBR102C	978
YPR032W	YDR166C	966
YPR032W	YEL037C	905
YPR032W	YER008C	998
YPR032W	YFL005W	988
YPR032W	YGL233W	999
YPR032W	YGR009C	999

YPR032W	YHR023W	981
YPR032W	YIL118W	998
YPR032W	YJL085W	966
YPR032W	YLR166C	996
YPR032W	YMR266W	931
YPR032W	YNL272C	904
YPR032W	YOR326W	984
YPR032W	YPL232W	975
YPR032W	YPR055W	998
YPR032W	YPR110C	923
YPR033C	YCL030C	960
YPR034W	YBR245C	957
YPR034W	YBR289W	999
YPR034W	YCR020W-B	998
YPR034W	YCR052W	999
YPR034W	YDR073W	998
YPR034W	YDR303C	999
YPR034W	YER148W	923
YPR034W	YFL049W	999
YPR034W	YFR037C	999
YPR034W	YGR056W	999
YPR034W	YGR275W	999
YPR034W	YHL025W	999
YPR034W	YHR056C	996
YPR034W	YIL126W	999
YPR034W	YJL176C	999
YPR034W	YKR008W	999
YPR034W	YLR033W	999
YPR034W	YLR321C	999
YPR034W	YLR357W	999
YPR034W	YML127W	998
YPR034W	YMR033W	999
YPR034W	YMR091C	999
YPR034W	YNR023W	999
YPR034W	YOR156C	983
YPR034W	YOR290C	999
YPR034W	YPL016W	999
YPR034W	YPL129W	998
YPR034W	YPR052C	956
YPR035W	YAL062W	989
YPR035W	YBR126C	935
YPR035W	YDL171C	986
YPR035W	YDL215C	904
YPR035W	YDR111C	930

YPR035W	YDR171W	984
YPR035W	YER040W	977
YPR035W	YGR124W	919
YPR035W	YHR037W	953
YPR035W	YIR037W	974
YPR035W	YJL101C	936
YPR035W	YJL130C	929
YPR035W	YJR109C	918
YPR035W	YKL104C	921
YPR035W	YKL106W	914
YPR035W	YLR027C	931
YPR035W	YLR089C	924
YPR035W	YMR250W	907
YPR035W	YMR300C	904
YPR035W	YNL229C	954
YPR035W	YOL033W	901
YPR035W	YOR168W	915
YPR035W	YOR375C	996
YPR035W	YPR145W	916
YPR036W	YBR127C	999
YPR036W	YDL185W	999
YPR036W	YEL027W	970
YPR036W	YEL051W	999
YPR036W	YER005W	915
YPR036W	YER036C	921
YPR036W	YGR020C	999
YPR036W	YHR026W	924
YPR036W	YHR039C-A	999
YPR036W	YKL080W	915
YPR036W	YLR447C	966
YPR036W	YMR054W	999
YPR036W	YOR270C	999
YPR036W	YOR332W	999
YPR036W	YPL234C	969
YPR037C	YML130C	957
YPR040W	YDL188C	941
YPR040W	YDR075W	936
YPR040W	YFR040W	986
YPR040W	YMR028W	999
YPR040W	YNL183C	905
YPR040W	YOR297C	924
YPR041W	YAL035W	997
YPR041W	YBR048W	915
YPR041W	YBR079C	999

YPR041W	YBR181C	919
YPR041W	YBR189W	914
YPR041W	YCR031C	905
YPR041W	YDR025W	919
YPR041W	YDR091C	997
YPR041W	YDR429C	999
YPR041W	YER025W	999
YPR041W	YGL049C	977
YPR041W	YGR162W	977
YPR041W	YJL191W	905
YPR041W	YJR007W	999
YPR041W	YJR123W	915
YPR041W	YJR145C	915
YPR041W	YLR192C	999
YPR041W	YMR146C	999
YPR041W	YMR260C	965
YPR041W	YMR309C	999
YPR041W	YNL096C	913
YPR041W	YNL178W	916
YPR041W	YNL244C	999
YPR041W	YOL139C	911
YPR041W	YOR096W	915
YPR041W	YOR204W	977
YPR041W	YOR361C	999
YPR041W	YPL090C	922
YPR041W	YPL237W	999
YPR041W	YPR086W	976
YPR041W	YPR163C	919
YPR043W	YBL027W	932
YPR043W	YBL072C	926
YPR043W	YBL087C	933
YPR043W	YBL092W	939
YPR043W	YBR031W	939
YPR043W	YBR048W	943
YPR043W	YBR084C-A	997
YPR043W	YBR118W	919
YPR043W	YBR181C	903
YPR043W	YBR189W	933
YPR043W	YBR191W	966
YPR043W	YCR031C	941
YPR043W	YDL061C	941
YPR043W	YDL075W	968
YPR043W	YDL081C	939
YPR043W	YDL082W	921

YPR043W	YDL130W	920
YPR043W	YDL136W	983
YPR043W	YDL191W	923
YPR043W	YDR012W	948
YPR043W	YDR025W	916
YPR043W	YDR064W	974
YPR043W	YDR382W	963
YPR043W	YDR385W	918
YPR043W	YDR418W	942
YPR043W	YDR447C	932
YPR043W	YDR450W	920
YPR043W	YDR471W	921
YPR043W	YDR500C	996
YPR043W	YEL054C	923
YPR043W	YER056C-A	961
YPR043W	YER074W	917
YPR043W	YER102W	993
YPR043W	YER117W	999
YPR043W	YER131W	921
YPR043W	YFL034C-A	909
YPR043W	YFR031C-A	929
YPR043W	YFR032C-A	933
YPR043W	YGL030W	965
YPR043W	YGL031C	907
YPR043W	YGL076C	929
YPR043W	YGL103W	946
YPR043W	YGL123W	947
YPR043W	YGL135W	922
YPR043W	YGL147C	939
YPR043W	YGL189C	921
YPR043W	YGR027C	940
YPR043W	YGR034W	938
YPR043W	YGR085C	937
YPR043W	YGR118W	960
YPR043W	YGR148C	922
YPR043W	YGR214W	937
YPR043W	YHL001W	943
YPR043W	YHL015W	941
YPR043W	YHL033C	948
YPR043W	YHR010W	922
YPR043W	YHR021C	931
YPR043W	YHR141C	930
YPR043W	YHR203C	996
YPR043W	YIL018W	999

YPR043W	YIL052C	960
YPR043W	YIL069C	917
YPR043W	YIL133C	934
YPR043W	YIL148W	994
YPR043W	YJL136C	946
YPR043W	YJL177W	951
YPR043W	YJL189W	921
YPR043W	YJL190C	913
YPR043W	YJL191W	939
YPR043W	YJR094W-A	963
YPR043W	YJR123W	963
YPR043W	YJR145C	918
YPR043W	YKL006W	952
YPR043W	YKL156W	955
YPR043W	YKL180W	958
YPR043W	YKR057W	935
YPR043W	YKR094C	902
YPR043W	YLL045C	922
YPR043W	YLR029C	957
YPR043W	YLR048W	960
YPR043W	YLR061W	960
YPR043W	YLR075W	976
YPR043W	YLR167W	993
YPR043W	YLR185W	983
YPR043W	YLR264W	941
YPR043W	YLR287C-A	903
YPR043W	YLR325C	927
YPR043W	YLR333C	928
YPR043W	YLR340W	971
YPR043W	YLR344W	971
YPR043W	YLR367W	928
YPR043W	YLR388W	967
YPR043W	YLR406C	923
YPR043W	YLR441C	926
YPR043W	YLR448W	960
YPR043W	YML024W	974
YPR043W	YML026C	920
YPR043W	YML063W	939
YPR043W	YML073C	929
YPR043W	YMR121C	949
YPR043W	YMR142C	920
YPR043W	YMR194W	966
YPR043W	YMR230W	931
YPR043W	YMR242C	932

YPR043W	YNL067W	949
YPR043W	YNL069C	936
YPR043W	YNL096C	921
YPR043W	YNL162W	930
YPR043W	YNL178W	976
YPR043W	YNL301C	996
YPR043W	YNL302C	931
YPR043W	YOL039W	913
YPR043W	YOL040C	952
YPR043W	YOL120C	923
YPR043W	YOL121C	931
YPR043W	YOL127W	967
YPR043W	YOR063W	958
YPR043W	YOR096W	940
YPR043W	YOR133W	949
YPR043W	YOR167C	913
YPR043W	YOR182C	903
YPR043W	YOR234C	947
YPR043W	YOR293W	940
YPR043W	YOR312C	944
YPR043W	YOR369C	939
YPR043W	YPL079W	936
YPR043W	YPL081W	914
YPR043W	YPL090C	903
YPR043W	YPL131W	968
YPR043W	YPL143W	951
YPR043W	YPL198W	929
YPR043W	YPL220W	996
YPR043W	YPL249C-A	946
YPR043W	YPR080W	909
YPR043W	YPR102C	938
YPR043W	YPR132W	998
YPR046W	YAL034W-A	995
YPR046W	YBR107C	999
YPR046W	YBR211C	998
YPR046W	YDR254W	992
YPR046W	YDR318W	999
YPR046W	YDR320C-A	963
YPR046W	YDR383C	930
YPR046W	YEL061C	966
YPR046W	YER016W	951
YPR046W	YER018C	963
YPR046W	YGL060W	981
YPR046W	YGL086W	944

YPR046W	YGR113W	983
YPR046W	YGR140W	981
YPR046W	YGR179C	998
YPR046W	YGR188C	990
YPR046W	YJL030W	999
YPR046W	YJR135C	999
YPR046W	YKL089W	964
YPR046W	YLR315W	974
YPR046W	YLR381W	999
YPR046W	YML094W	922
YPR046W	YMR094W	981
YPR046W	YNL153C	907
YPR046W	YPL008W	922
YPR046W	YPL018W	996
YPR046W	YPL233W	906
YPR046W	YPL240C	919
YPR047W	YNL316C	906
YPR048W	YKR071C	999
YPR049C	YDL113C	984
YPR049C	YDL149W	999
YPR049C	YDR228C	905
YPR049C	YDR320C	935
YPR049C	YGL180W	999
YPR049C	YKL103C	950
YPR049C	YLR423C	999
YPR049C	YOL082W	985
YPR049C	YPL166W	996
YPR049C	YPR185W	998
YPR051W	YCR020C-A	999
YPR051W	YEL053C	999
YPR051W	YPR131C	946
YPR052C	YBR009C	956
YPR052C	YBR010W	980
YPR052C	YBR089C-A	999
YPR052C	YBR181C	968
YPR052C	YDR224C	919
YPR052C	YER148W	967
YPR052C	YGL071W	947
YPR052C	YGL207W	999
YPR052C	YGR047C	956
YPR052C	YHR203C	968
YPR052C	YIL130W	998
YPR052C	YLR182W	972
YPR052C	YML010W	989

YPR052C	YML069W	989
YPR052C	YMR033W	974
YPR052C	YMR072W	987
YPR052C	YOR198C	939
YPR052C	YOR290C	988
YPR052C	YPL127C	910
YPR052C	YPR034W	956
YPR054W	YFL029C	986
YPR054W	YGR032W	953
YPR055W	YBR102C	999
YPR055W	YBR200W	974
YPR055W	YDR164C	991
YPR055W	YDR166C	999
YPR055W	YDR363W-A	906
YPR055W	YER008C	999
YPR055W	YFL005W	978
YPR055W	YGL233W	999
YPR055W	YGR009C	994
YPR055W	YIL068C	999
YPR055W	YJL085W	999
YPR055W	YLR166C	999
YPR055W	YLR229C	936
YPR055W	YNL272C	993
YPR055W	YNR049C	959
YPR055W	YOR326W	987
YPR055W	YPR017C	961
YPR055W	YPR032W	998
YPR056W	YBL084C	912
YPR056W	YBR253W	984
YPR056W	YCR042C	984
YPR056W	YDL108W	982
YPR056W	YDL140C	986
YPR056W	YDR037W	934
YPR056W	YDR079C-A	996
YPR056W	YDR167W	907
YPR056W	YDR311W	999
YPR056W	YDR460W	999
YPR056W	YER022W	984
YPR056W	YER148W	986
YPR056W	YER171W	996
YPR056W	YGL070C	900
YPR056W	YGR005C	984
YPR056W	YGR104C	984
YPR056W	YGR186W	984

YPR056W	YHR041C	984
YPR056W	YIL143C	996
YPR056W	YKL058W	921
YPR056W	YLR005W	999
YPR056W	YOR194C	901
YPR056W	YPL022W	900
YPR056W	YPL122C	999
YPR056W	YPL129W	984
YPR056W	YPR025C	993
YPR056W	YPR086W	988
YPR057W	YBR119W	974
YPR057W	YGR013W	970
YPR057W	YGR074W	986
YPR057W	YIL061C	968
YPR057W	YLR275W	987
YPR060C	YBR166C	950
YPR062W	YDR400W	929
YPR062W	YHR128W	996
YPR062W	YLR209C	928
YPR065W	YBR112C	959
YPR065W	YCR084C	959
YPR065W	YDR044W	945
YPR065W	YJR047C	946
YPR065W	YMR070W	959
YPR066W	YDR139C	947
YPR066W	YPL003W	999
YPR067W	YBR181C	970
YPR067W	YDL075W	970
YPR067W	YNL096C	972
YPR067W	YOL127W	972
YPR067W	YPL059W	935
YPR068C	YBR112C	914
YPR068C	YCR084C	908
YPR068C	YGL194C	931
YPR068C	YNL021W	948
YPR068C	YNL330C	929
YPR068C	YPL116W	949
YPR069C	YKL184W	977
YPR069C	YLR146C	999
YPR069C	YMR020W	915
YPR069C	YOL052C	963
YPR070W	YBL093C	999
YPR070W	YBR193C	999
YPR070W	YBR198C	912

YPR070W	YBR253W	999
YPR070W	YCR081W	997
YPR070W	YDL005C	999
YPR070W	YDR145W	909
YPR070W	YDR167W	994
YPR070W	YDR308C	999
YPR070W	YDR443C	999
YPR070W	YDR448W	994
YPR070W	YER022W	999
YPR070W	YER171W	907
YPR070W	YGL025C	999
YPR070W	YGL112C	998
YPR070W	YGL127C	999
YPR070W	YGL151W	999
YPR070W	YGR104C	999
YPR070W	YGR274C	912
YPR070W	YHR041C	999
YPR070W	YHR058C	999
YPR070W	YLR005W	924
YPR070W	YLR071C	999
YPR070W	YLR418C	983
YPR070W	YML007W	992
YPR070W	YML098W	906
YPR070W	YMR005W	953
YPR070W	YMR112C	999
YPR070W	YMR236W	920
YPR070W	YNL025C	917
YPR070W	YNL236W	999
YPR070W	YNR010W	999
YPR070W	YOL051W	999
YPR070W	YOL135C	999
YPR070W	YOL145C	903
YPR070W	YOR123C	942
YPR070W	YOR151C	915
YPR070W	YOR174W	999
YPR070W	YPL042C	999
YPR070W	YPL122C	916
YPR070W	YPL129W	992
YPR070W	YPR168W	999
YPR072W	YAL021C	999
YPR072W	YCR093W	999
YPR072W	YDL165W	999
YPR072W	YDR145W	983
YPR072W	YER068W	999

YPR072W	YGL112C	983
YPR072W	YGR134W	934
YPR072W	YIL038C	999
YPR072W	YML098W	921
YPR072W	YNL288W	998
YPR072W	YNR052C	999
YPR073C	YBR256C	926
YPR074C	YBL068W	900
YPR074C	YBR117C	999
YPR074C	YBR196C	942
YPR074C	YCR036W	935
YPR074C	YDR050C	906
YPR074C	YER099C	900
YPR074C	YGR043C	958
YPR074C	YGR240C	909
YPR074C	YHL011C	901
YPR074C	YJL121C	994
YPR074C	YJR104C	993
YPR074C	YKL060C	937
YPR074C	YKL181W	912
YPR074C	YLR354C	994
YPR074C	YLR377C	927
YPR074C	YMR205C	912
YPR074C	YNL241C	984
YPR074C	YOL061W	920
YPR074C	YOR095C	963
YPR075C	YCL032W	928
YPR079W	YBL017C	969
YPR080W	YAL003W	999
YPR080W	YBL027W	908
YPR080W	YBL072C	909
YPR080W	YBL087C	925
YPR080W	YBL092W	911
YPR080W	YBR031W	920
YPR080W	YBR048W	900
YPR080W	YBR084C-A	908
YPR080W	YBR118W	986
YPR080W	YBR189W	901
YPR080W	YBR191W	907
YPR080W	YCR031C	907
YPR080W	YDL075W	905
YPR080W	YDL081C	914
YPR080W	YDL082W	910
YPR080W	YDL136W	905

YPR080W	YDR012W	920
YPR080W	YDR025W	900
YPR080W	YDR041W	924
YPR080W	YDR064W	929
YPR080W	YDR382W	913
YPR080W	YDR385W	947
YPR080W	YDR418W	907
YPR080W	YDR447C	910
YPR080W	YDR450W	907
YPR080W	YEL054C	907
YPR080W	YER074W	909
YPR080W	YER102W	909
YPR080W	YER117W	917
YPR080W	YER131W	904
YPR080W	YFL034C-A	902
YPR080W	YFR031C-A	905
YPR080W	YFR032C-A	906
YPR080W	YGL030W	940
YPR080W	YGL031C	904
YPR080W	YGL076C	914
YPR080W	YGL123W	925
YPR080W	YGL135W	906
YPR080W	YGL147C	907
YPR080W	YGL189C	904
YPR080W	YGR027C	923
YPR080W	YGR034W	905
YPR080W	YGR085C	907
YPR080W	YGR118W	932
YPR080W	YGR214W	912
YPR080W	YHL001W	907
YPR080W	YHL015W	978
YPR080W	YHL033C	906
YPR080W	YHL034C	956
YPR080W	YHR021C	908
YPR080W	YHR141C	910
YPR080W	YHR203C	922
YPR080W	YIL018W	905
YPR080W	YIL069C	909
YPR080W	YIL133C	907
YPR080W	YIL148W	900
YPR080W	YJL136C	910
YPR080W	YJL177W	901
YPR080W	YJL189W	903
YPR080W	YJL190C	901

YPR080W	YJL191W	907
YPR080W	YJR094W-A	909
YPR080W	YJR123W	966
YPR080W	YJR145C	922
YPR080W	YKL001C	923
YPR080W	YKL006W	907
YPR080W	YKL081W	999
YPR080W	YKL156W	908
YPR080W	YKL170W	912
YPR080W	YKL180W	905
YPR080W	YKR057W	910
YPR080W	YKR094C	900
YPR080W	YLL045C	906
YPR080W	YLR029C	936
YPR080W	YLR048W	911
YPR080W	YLR061W	903
YPR080W	YLR075W	906
YPR080W	YLR249W	976
YPR080W	YLR264W	902
YPR080W	YLR333C	907
YPR080W	YLR340W	922
YPR080W	YLR344W	905
YPR080W	YLR406C	905
YPR080W	YLR441C	916
YPR080W	YLR448W	911
YPR080W	YML024W	910
YPR080W	YML026C	907
YPR080W	YML063W	916
YPR080W	YML073C	909
YPR080W	YMR121C	936
YPR080W	YMR142C	911
YPR080W	YMR194W	908
YPR080W	YMR230W	909
YPR080W	YMR242C	916
YPR080W	YNL067W	907
YPR080W	YNL069C	906
YPR080W	YNL081C	912
YPR080W	YNL096C	903
YPR080W	YNL162W	910
YPR080W	YNL178W	934
YPR080W	YNL301C	912
YPR080W	YNL302C	907
YPR080W	YNR037C	933
YPR080W	YOL039W	904

YPR080W	YOL040C	916
YPR080W	YOL120C	911
YPR080W	YOL121C	921
YPR080W	YOL127W	906
YPR080W	YOR063W	941
YPR080W	YOR096W	903
YPR080W	YOR133W	949
YPR080W	YOR167C	902
YPR080W	YOR234C	912
YPR080W	YOR293W	909
YPR080W	YOR312C	916
YPR080W	YOR369C	911
YPR080W	YPL048W	997
YPR080W	YPL079W	907
YPR080W	YPL081W	901
YPR080W	YPL131W	912
YPR080W	YPL143W	912
YPR080W	YPL198W	913
YPR080W	YPL220W	957
YPR080W	YPL249C-A	909
YPR080W	YPR043W	909
YPR080W	YPR102C	907
YPR080W	YPR132W	932
YPR081C	YBR121C	989
YPR082C	YBL026W	998
YPR082C	YBR055C	999
YPR082C	YCR077C	974
YPR082C	YDL030W	976
YPR082C	YDL098C	989
YPR082C	YDL160C	972
YPR082C	YDR378C	997
YPR082C	YDR473C	994
YPR082C	YEL026W	949
YPR082C	YER029C	999
YPR082C	YER112W	993
YPR082C	YER172C	999
YPR082C	YFL017W-A	985
YPR082C	YGR074W	997
YPR082C	YGR091W	999
YPR082C	YGR271W	903
YPR082C	YHR165C	998
YPR082C	YJL124C	974
YPR082C	YJL203W	974
YPR082C	YJR084W	989

YPR082C	YKL173W	999
YPR082C	YLR147C	999
YPR082C	YLR275W	997
YPR082C	YLR424W	974
YPR082C	YML046W	976
YPR082C	YML049C	997
YPR082C	YMR240C	997
YPR082C	YMR268C	970
YPR082C	YMR288W	998
YPR082C	YNL147W	999
YPR082C	YOR308C	989
YPR082C	YPL178W	913
YPR082C	YPL213W	976
YPR082C	YPR101W	974
YPR082C	YPR178W	978
YPR082C	YPR182W	991
YPR085C	YKL033W	973
YPR086W	YBR079C	998
YPR086W	YBR154C	980
YPR086W	YBR198C	904
YPR086W	YDL140C	999
YPR086W	YDL171C	904
YPR086W	YDR123C	978
YPR086W	YDR145W	978
YPR086W	YDR167W	994
YPR086W	YDR216W	987
YPR086W	YDR404C	939
YPR086W	YDR429C	974
YPR086W	YER148W	999
YPR086W	YER171W	922
YPR086W	YFR034C	967
YPR086W	YGL070C	994
YPR086W	YGL112C	938
YPR086W	YGL241W	972
YPR086W	YGR005C	984
YPR086W	YGR186W	998
YPR086W	YGR274C	991
YPR086W	YIL021W	939
YPR086W	YIL143C	966
YPR086W	YJR048W	934
YPR086W	YKL028W	925
YPR086W	YKL058W	998
YPR086W	YLR418C	961
YPR086W	YML098W	933

YPR086W	YMR039C	998
YPR086W	YMR146C	985
YPR086W	YMR236W	943
YPR086W	YMR309C	965
YPR086W	YNL222W	999
YPR086W	YOL005C	902
YPR086W	YOR116C	937
YPR086W	YOR151C	999
YPR086W	YOR194C	998
YPR086W	YOR207C	918
YPR086W	YOR210W	927
YPR086W	YOR224C	921
YPR086W	YOR361C	955
YPR086W	YPL082C	981
YPR086W	YPL248C	998
YPR086W	YPR025C	906
YPR086W	YPR041W	976
YPR086W	YPR056W	988
YPR086W	YPR187W	944
YPR088C	YDL051W	999
YPR088C	YDL092W	999
YPR088C	YKL122C	999
YPR088C	YML105C	999
YPR088C	YPL210C	999
YPR088C	YPL243W	999
YPR093C	YBR017C	977
YPR093C	YDL140C	998
YPR093C	YER110C	977
YPR093C	YGL241W	980
YPR093C	YLR347C	976
YPR093C	YMR308C	975
YPR093C	YOR151C	989
YPR094W	YCR063W	929
YPR094W	YDR381W	908
YPR094W	YIR005W	991
YPR094W	YML049C	998
YPR094W	YMR240C	992
YPR094W	YMR288W	995
YPR094W	YNL138W-A	945
YPR094W	YOR319W	949
YPR095C	YDL137W	905
YPR100W	YBL038W	933
YPR100W	YCR046C	921
YPR101W	YAL032C	989

YPR101W	YBR065C	988
YPR101W	YBR188C	998
YPR101W	YCR063W	988
YPR101W	YDL030W	974
YPR101W	YDL209C	995
YPR101W	YDR364C	988
YPR101W	YDR416W	999
YPR101W	YER029C	995
YPR101W	YER172C	997
YPR101W	YGL120C	974
YPR101W	YGL128C	972
YPR101W	YGR129W	990
YPR101W	YHR165C	991
YPR101W	YJR050W	998
YPR101W	YKL095W	997
YPR101W	YKL173W	991
YPR101W	YLL036C	999
YPR101W	YLR117C	999
YPR101W	YLR424W	995
YPR101W	YML049C	976
YPR101W	YMR213W	999
YPR101W	YMR288W	974
YPR101W	YPL151C	995
YPR101W	YPL213W	998
YPR101W	YPR082C	974
YPR102C	YAL035W	916
YPR102C	YBL027W	997
YPR102C	YBL072C	917
YPR102C	YBL087C	998
YPR102C	YBL092W	999
YPR102C	YBR031W	988
YPR102C	YBR048W	974
YPR102C	YBR084C-A	982
YPR102C	YBR118W	907
YPR102C	YBR181C	961
YPR102C	YBR189W	999
YPR102C	YBR191W	999
YPR102C	YCL059C	943
YPR102C	YCR031C	988
YPR102C	YDL061C	990
YPR102C	YDL075W	998
YPR102C	YDL081C	939
YPR102C	YDL082W	958
YPR102C	YDL130W	916

YPR102C	YDL136W	993
YPR102C	YDL191W	911
YPR102C	YDR012W	999
YPR102C	YDR025W	999
YPR102C	YDR041W	966
YPR102C	YDR064W	998
YPR102C	YDR382W	993
YPR102C	YDR385W	916
YPR102C	YDR418W	999
YPR102C	YDR447C	933
YPR102C	YDR450W	934
YPR102C	YDR471W	993
YPR102C	YDR500C	927
YPR102C	YEL054C	996
YPR102C	YER056C-A	985
YPR102C	YER074W	905
YPR102C	YER102W	948
YPR102C	YER117W	990
YPR102C	YER131W	993
YPR102C	YFL034C-A	913
YPR102C	YFR031C-A	998
YPR102C	YFR032C-A	936
YPR102C	YGL030W	992
YPR102C	YGL031C	999
YPR102C	YGL076C	997
YPR102C	YGL103W	999
YPR102C	YGL123W	998
YPR102C	YGL135W	991
YPR102C	YGL147C	999
YPR102C	YGL189C	901
YPR102C	YGR027C	999
YPR102C	YGR034W	984
YPR102C	YGR085C	999
YPR102C	YGR118W	999
YPR102C	YGR148C	999
YPR102C	YGR214W	997
YPR102C	YHL001W	995
YPR102C	YHL015W	989
YPR102C	YHL033C	999
YPR102C	YHR010W	995
YPR102C	YHR021C	920
YPR102C	YHR141C	995
YPR102C	YHR147C	914
YPR102C	YHR203C	983

YPR102C	YIL018W	983
YPR102C	YIL052C	999
YPR102C	YIL069C	905
YPR102C	YIL133C	994
YPR102C	YIL148W	940
YPR102C	YJL136C	973
YPR102C	YJL177W	993
YPR102C	YJL189W	946
YPR102C	YJL190C	985
YPR102C	YJL191W	999
YPR102C	YJR094W-A	997
YPR102C	YJR123W	999
YPR102C	YJR145C	976
YPR102C	YKL006W	999
YPR102C	YKL156W	977
YPR102C	YKL170W	981
YPR102C	YKL180W	999
YPR102C	YKR057W	921
YPR102C	YKR081C	977
YPR102C	YKR094C	905
YPR102C	YLL045C	978
YPR102C	YLR029C	999
YPR102C	YLR048W	996
YPR102C	YLR061W	996
YPR102C	YLR075W	998
YPR102C	YLR167W	938
YPR102C	YLR185W	991
YPR102C	YLR264W	994
YPR102C	YLR325C	929
YPR102C	YLR333C	960
YPR102C	YLR340W	979
YPR102C	YLR344W	999
YPR102C	YLR367W	995
YPR102C	YLR388W	999
YPR102C	YLR406C	917
YPR102C	YLR441C	939
YPR102C	YLR448W	985
YPR102C	YML024W	996
YPR102C	YML026C	934
YPR102C	YML063W	999
YPR102C	YML073C	924
YPR102C	YMR121C	933
YPR102C	YMR142C	965
YPR102C	YMR194W	982

YPR102C	YMR230W	970
YPR102C	YMR242C	952
YPR102C	YMR260C	912
YPR102C	YNL067W	999
YPR102C	YNL069C	998
YPR102C	YNL081C	962
YPR102C	YNL096C	934
YPR102C	YNL162W	922
YPR102C	YNL178W	999
YPR102C	YNL301C	994
YPR102C	YNL302C	918
YPR102C	YNR037C	982
YPR102C	YOL039W	953
YPR102C	YOL040C	983
YPR102C	YOL120C	922
YPR102C	YOL121C	933
YPR102C	YOL127W	999
YPR102C	YOR063W	999
YPR102C	YOR096W	995
YPR102C	YOR133W	916
YPR102C	YOR167C	986
YPR102C	YOR234C	993
YPR102C	YOR293W	998
YPR102C	YOR294W	996
YPR102C	YOR312C	935
YPR102C	YOR369C	950
YPR102C	YPL079W	999
YPR102C	YPL081W	954
YPR102C	YPL090C	945
YPR102C	YPL093W	977
YPR102C	YPL131W	999
YPR102C	YPL143W	983
YPR102C	YPL198W	927
YPR102C	YPL220W	998
YPR102C	YPL249C-A	931
YPR102C	YPR043W	938
YPR102C	YPR080W	907
YPR102C	YPR132W	950
YPR103W	YBL041W	999
YPR103W	YBR082C	999
YPR103W	YBR173C	999
YPR103W	YDL007W	908
YPR103W	YDL020C	999
YPR103W	YDL097C	924

YPR103W	YDL147W	998
YPR103W	YDR059C	999
YPR103W	YDR069C	999
YPR103W	YDR177W	999
YPR103W	YDR394W	918
YPR103W	YDR427W	926
YPR103W	YER012W	999
YPR103W	YER021W	922
YPR103W	YER094C	999
YPR103W	YFL007W	998
YPR103W	YFR004W	960
YPR103W	YFR050C	999
YPR103W	YFR052W	927
YPR103W	YGL011C	999
YPR103W	YGL048C	999
YPR103W	YGL058W	999
YPR103W	YGR135W	999
YPR103W	YGR184C	998
YPR103W	YGR253C	999
YPR103W	YHL030W	999
YPR103W	YHR027C	935
YPR103W	YHR052W	998
YPR103W	YHR200W	999
YPR103W	YIL075C	940
YPR103W	YIL148W	916
YPR103W	YJL001W	999
YPR103W	YKL145W	961
YPR103W	YLL039C	970
YPR103W	YLL040C	940
YPR103W	YLR167W	918
YPR103W	YLR199C	921
YPR103W	YML092C	999
YPR103W	YMR314W	999
YPR103W	YOL038W	999
YPR103W	YOR056C	998
YPR103W	YOR117W	907
YPR103W	YOR157C	999
YPR103W	YOR259C	923
YPR103W	YOR261C	906
YPR103W	YOR362C	999
YPR103W	YPR108W	944
YPR104C	YDR223W	911
YPR104C	YHR205W	908
YPR104C	YLR223C	999

YPR104C	YNL216W	993
YPR105C	YDR189W	979
YPR105C	YER157W	999
YPR105C	YGL005C	999
YPR105C	YGL223C	999
YPR105C	YGR120C	999
YPR105C	YML071C	999
YPR105C	YNL041C	999
YPR105C	YNL051W	999
YPR106W	YBL105C	928
YPR106W	YDR152W	938
YPR107C	YAL043C	999
YPR107C	YDR195W	999
YPR107C	YDR301W	999
YPR107C	YER133W	999
YPR107C	YGR156W	999
YPR107C	YJR093C	999
YPR107C	YKL018W	999
YPR107C	YKL059C	999
YPR107C	YKR002W	999
YPR107C	YLR115W	999
YPR107C	YLR277C	999
YPR107C	YNL222W	999
YPR107C	YNL317W	999
YPR108W	YBL041W	931
YPR108W	YDL007W	999
YPR108W	YDL097C	999
YPR108W	YDL147W	999
YPR108W	YDR363W-A	999
YPR108W	YDR394W	999
YPR108W	YDR427W	999
YPR108W	YER012W	999
YPR108W	YER021W	999
YPR108W	YER094C	942
YPR108W	YFR004W	999
YPR108W	YFR010W	999
YPR108W	YFR050C	947
YPR108W	YFR052W	999
YPR108W	YGL011C	901
YPR108W	YGL048C	999
YPR108W	YGR135W	913
YPR108W	YGR232W	998
YPR108W	YGR253C	918
YPR108W	YHL030W	992

YPR108W	YHR027C	999
YPR108W	YHR200W	999
YPR108W	YIL075C	999
YPR108W	YJL001W	919
YPR108W	YKL145W	999
YPR108W	YLL039C	970
YPR108W	YLR421C	997
YPR108W	YML092C	917
YPR108W	YMR191W	987
YPR108W	YMR314W	997
YPR108W	YOL038W	978
YPR108W	YOR117W	998
YPR108W	YOR157C	927
YPR108W	YOR259C	999
YPR108W	YOR261C	999
YPR108W	YOR362C	917
YPR108W	YPR103W	944
YPR110C	YBL039C	970
YPR110C	YBR154C	999
YPR110C	YDL150W	999
YPR110C	YDL160C	918
YPR110C	YDR005C	997
YPR110C	YDR045C	965
YPR110C	YDR156W	998
YPR110C	YER148W	918
YPR110C	YER171W	901
YPR110C	YFR028C	903
YPR110C	YGL156W	928
YPR110C	YGR094W	912
YPR110C	YHR143W-A	958
YPR110C	YIL143C	901
YPR110C	YJL011C	999
YPR110C	YJL148W	999
YPR110C	YJR063W	999
YPR110C	YKL144C	999
YPR110C	YKR025W	999
YPR110C	YNL039W	960
YPR110C	YNL113W	999
YPR110C	YNL151C	999
YPR110C	YNL248C	999
YPR110C	YNR003C	999
YPR110C	YOR001W	941
YPR110C	YOR116C	999
YPR110C	YOR151C	972

YPR110C	YOR207C	999
YPR110C	YOR210W	999
YPR110C	YOR224C	999
YPR110C	YOR340C	999
YPR110C	YOR341W	999
YPR110C	YPR010C	999
YPR110C	YPR032W	923
YPR110C	YPR187W	999
YPR110C	YPR190C	999
YPR111W	YAR019C	974
YPR111W	YGR092W	999
YPR111W	YIL106W	999
YPR111W	YJL076W	982
YPR111W	YML064C	929
YPR111W	YPL242C	907
YPR112C	YBR247C	943
YPR112C	YCL059C	925
YPR112C	YCR057C	967
YPR112C	YDL014W	935
YPR112C	YDL060W	931
YPR112C	YDL148C	920
YPR112C	YDL153C	917
YPR112C	YDR299W	940
YPR112C	YDR324C	933
YPR112C	YDR339C	912
YPR112C	YDR449C	930
YPR112C	YER082C	987
YPR112C	YGL171W	936
YPR112C	YGR090W	924
YPR112C	YGR128C	924
YPR112C	YGR145W	950
YPR112C	YHR148W	921
YPR112C	YHR169W	924
YPR112C	YHR196W	932
YPR112C	YJL010C	989
YPR112C	YJL109C	915
YPR112C	YJR002W	967
YPR112C	YKR060W	916
YPR112C	YLL011W	925
YPR112C	YLR003C	969
YPR112C	YLR129W	998
YPR112C	YLR197W	909
YPR112C	YLR222C	923
YPR112C	YLR409C	933

YPR112C	YML093W	920
YPR112C	YMR093W	921
YPR112C	YMR128W	916
YPR112C	YMR229C	919
YPR112C	YMR290C	916
YPR112C	YNL075W	923
YPR112C	YNL132W	951
YPR112C	YOL010W	923
YPR112C	YOR004W	979
YPR112C	YOR078W	936
YPR112C	YOR145C	910
YPR112C	YPL012W	915
YPR112C	YPL126W	947
YPR112C	YPL217C	998
YPR112C	YPR137W	925
YPR112C	YPR144C	928
YPR113W	YBR029C	963
YPR113W	YCL004W	922
YPR113W	YER026C	905
YPR113W	YLR240W	915
YPR118W	YJR024C	909
YPR118W	YLR017W	909
YPR119W	YAL024C	987
YPR119W	YAR019C	999
YPR119W	YBL084C	983
YPR119W	YBR135W	999
YPR119W	YBR160W	999
YPR119W	YBR274W	911
YPR119W	YCL014W	997
YPR119W	YDL056W	960
YPR119W	YDL108W	901
YPR119W	YDL155W	996
YPR119W	YDR113C	998
YPR119W	YDR118W	968
YPR119W	YDR146C	999
YPR119W	YDR369C	904
YPR119W	YDR460W	918
YPR119W	YDR478W	956
YPR119W	YDR507C	912
YPR119W	YEL032W	923
YPR119W	YEL061C	979
YPR119W	YER111C	999
YPR119W	YFL029C	934
YPR119W	YFR028C	988

YPR119W	YFR036W	916
YPR119W	YGL003C	999
YPR119W	YGL116W	998
YPR119W	YGR092W	998
YPR119W	YGR108W	999
YPR119W	YHR158C	984
YPR119W	YHR166C	999
YPR119W	YHR191C	978
YPR119W	YIL101C	936
YPR119W	YIL131C	997
YPR119W	YJL076W	989
YPR119W	YJL187C	999
YPR119W	YJL194W	999
YPR119W	YKL022C	988
YPR119W	YKR048C	999
YPR119W	YLL021W	925
YPR119W	YLR079W	999
YPR119W	YLR102C	926
YPR119W	YLR127C	980
YPR119W	YLR131C	992
YPR119W	YLR182W	957
YPR119W	YLR210W	967
YPR119W	YML032C	922
YPR119W	YMR001C	999
YPR119W	YMR036C	983
YPR119W	YMR043W	963
YPR119W	YNL068C	999
YPR119W	YNL189W	994
YPR119W	YOR042W	962
YPR119W	YOR058C	987
YPR119W	YOR372C	995
YPR119W	YPR025C	901
YPR119W	YPR120C	945
YPR120C	YBR135W	997
YPR120C	YBR136W	984
YPR120C	YBR160W	999
YPR120C	YDL017W	909
YPR120C	YDL101C	941
YPR120C	YDR052C	908
YPR120C	YDR113C	968
YPR120C	YER012W	929
YPR120C	YER111C	996
YPR120C	YFL009W	939
YPR120C	YGL003C	999

YPR120C	YGL116W	987
YPR120C	YGR109C	999
YPR120C	YHR031C	933
YPR120C	YHR118C	998
YPR120C	YIL131C	932
YPR120C	YJL157C	978
YPR120C	YJL187C	928
YPR120C	YJL194W	996
YPR120C	YKL042W	994
YPR120C	YKL108W	996
YPR120C	YKR101W	975
YPR120C	YLR079W	999
YPR120C	YLR182W	965
YPR120C	YML065W	967
YPR120C	YNL068C	930
YPR120C	YPL269W	990
YPR120C	YPR119W	945
YPR122W	YDR461W	906
YPR122W	YJR092W	954
YPR122W	YLR353W	907
YPR122W	YLR389C	999
YPR122W	YOR301W	915
YPR124W	YHR175W	957
YPR124W	YLR411W	997
YPR124W	YMR021C	992
YPR124W	YMR058W	956
YPR124W	YMR319C	923
YPR129W	YDL160C	907
YPR129W	YEL015W	958
YPR129W	YGL014W	903
YPR131C	YDR394W	950
YPR131C	YHR087W	925
YPR131C	YKR024C	940
YPR131C	YNL079C	927
YPR131C	YOL076W	999
YPR131C	YPR051W	946
YPR132W	YAL035W	923
YPR132W	YBL027W	922
YPR132W	YBL072C	928
YPR132W	YBL087C	930
YPR132W	YBL092W	935
YPR132W	YBR031W	941
YPR132W	YBR048W	959
YPR132W	YBR079C	909

YPR132W	YBR084C-A	999
YPR132W	YBR118W	944
YPR132W	YBR181C	904
YPR132W	YBR189W	950
YPR132W	YBR191W	994
YPR132W	YCR031C	954
YPR132W	YDL061C	964
YPR132W	YDL075W	996
YPR132W	YDL081C	939
YPR132W	YDL082W	930
YPR132W	YDL130W	928
YPR132W	YDL136W	999
YPR132W	YDL191W	922
YPR132W	YDL192W	980
YPR132W	YDR012W	945
YPR132W	YDR025W	922
YPR132W	YDR051C	998
YPR132W	YDR064W	988
YPR132W	YDR382W	976
YPR132W	YDR385W	955
YPR132W	YDR418W	964
YPR132W	YDR447C	933
YPR132W	YDR450W	927
YPR132W	YDR471W	950
YPR132W	YDR500C	999
YPR132W	YEL054C	927
YPR132W	YER025W	932
YPR132W	YER056C-A	994
YPR132W	YER074W	922
YPR132W	YER102W	999
YPR132W	YER117W	999
YPR132W	YER131W	929
YPR132W	YER165W	912
YPR132W	YFL034C-A	913
YPR132W	YFR031C-A	916
YPR132W	YFR032C-A	935
YPR132W	YGL030W	995
YPR132W	YGL031C	910
YPR132W	YGL076C	933
YPR132W	YGL103W	973
YPR132W	YGL123W	980
YPR132W	YGL135W	921
YPR132W	YGL147C	923
YPR132W	YGL189C	921

YPR132W	YGR027C	947
YPR132W	YGR034W	924
YPR132W	YGR085C	939
YPR132W	YGR118W	985
YPR132W	YGR148C	947
YPR132W	YGR214W	949
YPR132W	YHL001W	963
YPR132W	YHL015W	976
YPR132W	YHL033C	974
YPR132W	YHR010W	941
YPR132W	YHR021C	932
YPR132W	YHR026W	906
YPR132W	YHR141C	929
YPR132W	YHR203C	999
YPR132W	YIL018W	916
YPR132W	YIL052C	994
YPR132W	YIL069C	922
YPR132W	YIL133C	933
YPR132W	YIL148W	999
YPR132W	YJL136C	953
YPR132W	YJL177W	960
YPR132W	YJL189W	940
YPR132W	YJL190C	921
YPR132W	YJL191W	955
YPR132W	YJR094W-A	997
YPR132W	YJR123W	996
YPR132W	YJR145C	925
YPR132W	YKL006W	980
YPR132W	YKL156W	974
YPR132W	YKL180W	984
YPR132W	YKR057W	933
YPR132W	YKR059W	999
YPR132W	YKR094C	907
YPR132W	YLL045C	919
YPR132W	YLR029C	939
YPR132W	YLR048W	974
YPR132W	YLR061W	995
YPR132W	YLR075W	997
YPR132W	YLR167W	999
YPR132W	YLR185W	999
YPR132W	YLR264W	961
YPR132W	YLR287C-A	900
YPR132W	YLR325C	929
YPR132W	YLR333C	927

YPR132W	YLR340W	984
YPR132W	YLR344W	998
YPR132W	YLR367W	947
YPR132W	YLR388W	994
YPR132W	YLR406C	921
YPR132W	YLR441C	934
YPR132W	YLR448W	975
YPR132W	YML024W	999
YPR132W	YML026C	927
YPR132W	YML063W	965
YPR132W	YML073C	928
YPR132W	YMR121C	920
YPR132W	YMR142C	929
YPR132W	YMR194W	995
YPR132W	YMR230W	939
YPR132W	YMR242C	933
YPR132W	YMR260C	923
YPR132W	YNL067W	954
YPR132W	YNL069C	931
YPR132W	YNL096C	923
YPR132W	YNL162W	929
YPR132W	YNL178W	998
YPR132W	YNL301C	999
YPR132W	YNL302C	932
YPR132W	YOL039W	913
YPR132W	YOL040C	970
YPR132W	YOL120C	924
YPR132W	YOL121C	933
YPR132W	YOL127W	979
YPR132W	YOR063W	961
YPR132W	YOR096W	964
YPR132W	YOR133W	999
YPR132W	YOR167C	923
YPR132W	YOR182C	900
YPR132W	YOR234C	962
YPR132W	YOR293W	947
YPR132W	YOR312C	955
YPR132W	YOR369C	940
YPR132W	YPL037C	965
YPR132W	YPL079W	944
YPR132W	YPL081W	931
YPR132W	YPL090C	904
YPR132W	YPL131W	976
YPR132W	YPL143W	973

YPR132W	YPL198W	932
YPR132W	YPL220W	999
YPR132W	YPL237W	902
YPR132W	YPL249C-A	957
YPR132W	YPR043W	998
YPR132W	YPR080W	932
YPR132W	YPR102C	950
YPR133C	YDL140C	953
YPR133C	YGR063C	962
YPR133C	YGR116W	999
YPR133W-A	YGR082W	991
YPR133W-A	YMR203W	999
YPR133W-A	YNL070W	990
YPR133W-A	YNL121C	943
YPR133W-A	YNL131W	991
YPR133W-A	YOR045W	998
YPR135W	YBR136W	908
YPR135W	YCL061C	912
YPR135W	YDR013W	906
YPR135W	YDR489W	930
YPR135W	YGL207W	908
YPR135W	YHR164C	988
YPR135W	YHR191C	992
YPR135W	YJL092W	929
YPR135W	YJL194W	931
YPR135W	YJR043C	919
YPR135W	YKL113C	927
YPR135W	YML032C	989
YPR135W	YML069W	956
YPR135W	YNL102W	999
YPR135W	YOR144C	990
YPR137C-B	YPL257W-B	998
YPR137W	YBL004W	999
YPR137W	YBR247C	971
YPR137W	YCL059C	999
YPR137W	YCR057C	999
YPR137W	YDL014W	999
YPR137W	YDL060W	914
YPR137W	YDL148C	991
YPR137W	YDL153C	909
YPR137W	YDR299W	951
YPR137W	YDR324C	999
YPR137W	YDR449C	999
YPR137W	YEL026W	949

YPR137W	YER082C	999
YPR137W	YGL171W	912
YPR137W	YGR090W	983
YPR137W	YGR128C	912
YPR137W	YGR145W	923
YPR137W	YHR148W	997
YPR137W	YHR169W	914
YPR137W	YHR196W	999
YPR137W	YJL010C	985
YPR137W	YJL109C	999
YPR137W	YJR002W	999
YPR137W	YKR060W	912
YPR137W	YLL011W	999
YPR137W	YLR129W	997
YPR137W	YLR175W	908
YPR137W	YLR186W	997
YPR137W	YLR197W	999
YPR137W	YLR222C	998
YPR137W	YLR409C	997
YPR137W	YML093W	911
YPR137W	YMR093W	999
YPR137W	YMR128W	999
YPR137W	YMR229C	972
YPR137W	YNL075W	999
YPR137W	YNL132W	999
YPR137W	YOL010W	993
YPR137W	YOR004W	915
YPR137W	YOR078W	999
YPR137W	YOR310C	999
YPR137W	YPL126W	999
YPR137W	YPL217C	999
YPR137W	YPL266W	990
YPR137W	YPR112C	925
YPR137W	YPR144C	999
YPR138C	YGR121C	998
YPR138C	YNL142W	997
YPR140W	YGL173C	958
YPR141C	YBL063W	984
YPR141C	YCL055W	905
YPR141C	YEL061C	999
YPR141C	YER016W	933
YPR141C	YFL037W	920
YPR141C	YGL086W	991
YPR141C	YGL216W	998

YPR141C	YHR191C	946
YPR141C	YJL013C	955
YPR141C	YJL030W	992
YPR141C	YJR135C	922
YPR141C	YKR054C	999
YPR141C	YLR045C	906
YPR141C	YLR371W	919
YPR141C	YML124C	981
YPR141C	YMR198W	999
YPR141C	YNL090W	926
YPR141C	YOR195W	920
YPR141C	YPL253C	973
YPR143W	YDR312W	906
YPR143W	YIL035C	920
YPR144C	YBL004W	999
YPR144C	YBL072C	980
YPR144C	YBR247C	999
YPR144C	YCL059C	999
YPR144C	YCR057C	999
YPR144C	YDL014W	998
YPR144C	YDL060W	914
YPR144C	YDL148C	999
YPR144C	YDL153C	909
YPR144C	YDR064W	981
YPR144C	YDR299W	998
YPR144C	YDR324C	999
YPR144C	YDR398W	911
YPR144C	YDR449C	999
YPR144C	YER082C	997
YPR144C	YGL120C	909
YPR144C	YGL171W	921
YPR144C	YGR090W	999
YPR144C	YGR128C	999
YPR144C	YGR145W	999
YPR144C	YHR148W	998
YPR144C	YHR169W	924
YPR144C	YHR196W	999
YPR144C	YJL010C	914
YPR144C	YJL109C	999
YPR144C	YJR002W	999
YPR144C	YJR145C	984
YPR144C	YKR060W	999
YPR144C	YLL011W	999
YPR144C	YLR003C	910

YPR144C	YLR129W	999
YPR144C	YLR186W	999
YPR144C	YLR197W	997
YPR144C	YLR222C	999
YPR144C	YLR409C	998
YPR144C	YMR093W	999
YPR144C	YMR128W	999
YPR144C	YMR229C	975
YPR144C	YMR290C	921
YPR144C	YNL075W	998
YPR144C	YNL132W	999
YPR144C	YOL010W	919
YPR144C	YOR004W	915
YPR144C	YOR096W	981
YPR144C	YOR145C	911
YPR144C	YPL012W	999
YPR144C	YPL126W	999
YPR144C	YPL217C	999
YPR144C	YPL266W	997
YPR144C	YPR112C	928
YPR144C	YPR137W	999
YPR145W	YAL062W	903
YPR145W	YCR024C	905
YPR145W	YGR124W	999
YPR145W	YHR019C	930
YPR145W	YLR027C	909
YPR145W	YMR250W	905
YPR145W	YOL058W	913
YPR145W	YOR375C	903
YPR145W	YPR035W	916
YPR149W	YEL013W	998
YPR149W	YOR270C	985
YPR152C	YBR194W	991
YPR154W	YHR114W	999
YPR154W	YLL039C	909
YPR154W	YOR181W	972
YPR159W	YBL105C	913
YPR159W	YFR042W	998
YPR159W	YGL027C	931
YPR159W	YGR143W	996
YPR159W	YGR166W	990
YPR159W	YJL034W	968
YPR159W	YJL174W	936
YPR159W	YOR181W	976

YPR159W	YOR336W	951
YPR160W	YEL011W	991
YPR160W	YFR015C	949
YPR160W	YHL012W	903
YPR160W	YIL099W	985
YPR160W	YKL035W	928
YPR160W	YKL127W	948
YPR160W	YLR258W	961
YPR160W	YMR105C	960
YPR160W	YPR184W	991
YPR161C	YDL140C	976
YPR161C	YGR063C	975
YPR161C	YJL168C	944
YPR161C	YKL139W	961
YPR161C	YLR226W	999
YPR161C	YML010W	996
YPR161C	YMR125W	961
YPR161C	YPR023C	904
YPR162C	YBL023C	922
YPR162C	YBR060C	999
YPR162C	YBR202W	901
YPR162C	YEL032W	903
YPR162C	YEL056W	999
YPR162C	YGL201C	904
YPR162C	YHR118C	999
YPR162C	YJL194W	999
YPR162C	YLL004W	999
YPR162C	YML065W	999
YPR162C	YNL261W	999
YPR162C	YPL001W	999
YPR162C	YPR019W	905
YPR162C	YPR175W	954
YPR163C	YBR079C	915
YPR163C	YBR181C	912
YPR163C	YBR189W	923
YPR163C	YCR031C	910
YPR163C	YDR064W	911
YPR163C	YDR429C	986
YPR163C	YER025W	917
YPR163C	YER131W	915
YPR163C	YER165W	919
YPR163C	YGL123W	914
YPR163C	YGR027C	913
YPR163C	YGR162W	987

YPR163C	YGR214W	920
YPR163C	YIL133C	914
YPR163C	YJL190C	916
YPR163C	YJR007W	914
YPR163C	YJR123W	915
YPR163C	YKL156W	912
YPR163C	YKR059W	996
YPR163C	YLR048W	918
YPR163C	YLR264W	909
YPR163C	YLR367W	910
YPR163C	YLR388W	911
YPR163C	YML063W	923
YPR163C	YMR260C	980
YPR163C	YMR309C	917
YPR163C	YNL096C	919
YPR163C	YNL178W	909
YPR163C	YOL139C	980
YPR163C	YOR096W	916
YPR163C	YOR167C	914
YPR163C	YOR293W	915
YPR163C	YOR361C	916
YPR163C	YOR369C	914
YPR163C	YPL081W	913
YPR163C	YPL237W	916
YPR163C	YPR041W	919
YPR164W	YDL059C	913
YPR164W	YJL047C	974
YPR164W	YJL092W	961
YPR164W	YMR190C	962
YPR165W	YAL041W	947
YPR165W	YAL048C	903
YPR165W	YBL105C	999
YPR165W	YBR260C	950
YPR165W	YDL135C	999
YPR165W	YDL240W	995
YPR165W	YDR389W	996
YPR165W	YER008C	976
YPR165W	YER136W	936
YPR165W	YER155C	997
YPR165W	YGL155W	983
YPR165W	YGR023W	913
YPR165W	YGR032W	999
YPR165W	YGR070W	995
YPR165W	YHR030C	990

YPR165W	YHR206W	981
YPR165W	YKL203C	986
YPR165W	YKR090W	987
YPR165W	YLL021W	986
YPR165W	YLL039C	956
YPR165W	YLR229C	906
YPR165W	YLR332W	999
YPR165W	YLR342W	999
YPR165W	YLR371W	999
YPR165W	YLR425W	939
YPR165W	YMR273C	978
YPR165W	YNL271C	999
YPR165W	YOR008C	988
YPR165W	YOR127W	994
YPR165W	YOR134W	990
YPR165W	YOR231W	928
YPR165W	YPL115C	969
YPR165W	YPL161C	999
YPR166C	Q0140	968
YPR166C	YNR036C	999
YPR167C	YCR083W	909
YPR167C	YFR030W	970
YPR167C	YGR209C	989
YPR167C	YHR042W	909
YPR167C	YJR010W	945
YPR167C	YJR137C	997
YPR167C	YKL001C	999
YPR167C	YKR069W	935
YPR167C	YLR043C	987
YPR167C	YNL103W	956
YPR167C	YOL064C	953
YPR167C	YPL091W	906
YPR168W	YBL093C	999
YPR168W	YBR154C	938
YPR168W	YBR193C	999
YPR168W	YBR253W	999
YPR168W	YCR081W	983
YPR168W	YDL005C	999
YPR168W	YDL140C	950
YPR168W	YDR167W	991
YPR168W	YDR308C	999
YPR168W	YDR404C	938
YPR168W	YDR443C	998
YPR168W	YER022W	999

YPR168W	YER148W	902
YPR168W	YGL025C	999
YPR168W	YGL070C	904
YPR168W	YGL127C	999
YPR168W	YGL151W	999
YPR168W	YGR104C	999
YPR168W	YHR041C	998
YPR168W	YHR058C	999
YPR168W	YHR143W-A	931
YPR168W	YIL021W	936
YPR168W	YJL140W	928
YPR168W	YLR071C	999
YPR168W	YML007W	974
YPR168W	YMR112C	998
YPR168W	YNL236W	999
YPR168W	YNR010W	999
YPR168W	YOL005C	932
YPR168W	YOL051W	999
YPR168W	YOL135C	999
YPR168W	YOR151C	942
YPR168W	YOR174W	999
YPR168W	YOR210W	905
YPR168W	YOR224C	935
YPR168W	YPL042C	996
YPR168W	YPL129W	991
YPR168W	YPR070W	999
YPR168W	YPR187W	939
YPR169W	YLR449W	958
YPR171W	YBL007C	993
YPR171W	YBR109C	968
YPR171W	YDR388W	995
YPR171W	YFL039C	972
YPR171W	YFR024C-A	977
YPR171W	YGL106W	984
YPR171W	YLR429W	966
YPR171W	YNL106C	998
YPR173C	YDR069C	988
YPR173C	YER128W	908
YPR173C	YGL045W	907
YPR173C	YHR001W	978
YPR173C	YKL002W	999
YPR173C	YKL041W	981
YPR173C	YKR003W	980
YPR173C	YKR035W-A	999

YPR173C	YLR025W	999
YPR173C	YLR181C	999
YPR173C	YMR063W	907
YPR173C	YMR077C	999
YPR173C	YMR314W	998
YPR173C	YNL265C	993
YPR173C	YNL294C	902
YPR173C	YOR275C	999
YPR173C	YPL084W	999
YPR174C	YER110C	964
YPR175W	YAR007C	984
YPR175W	YBL002W	983
YPR175W	YBL023C	960
YPR175W	YBR160W	994
YPR175W	YBR278W	999
YPR175W	YDR013W	975
YPR175W	YDR121W	999
YPR175W	YIL150C	979
YPR175W	YJL090C	998
YPR175W	YLR103C	987
YPR175W	YML065W	954
YPR175W	YMR078C	980
YPR175W	YNL102W	929
YPR175W	YNL262W	999
YPR175W	YOL146W	926
YPR175W	YPR019W	904
YPR175W	YPR162C	954
YPR176C	YFL038C	999
YPR176C	YJL030W	998
YPR176C	YJL031C	999
YPR176C	YOR370C	996
YPR178W	YBL026W	997
YPR178W	YBL074C	977
YPR178W	YBR055C	999
YPR178W	YBR152W	999
YPR178W	YCR077C	976
YPR178W	YDL030W	978
YPR178W	YDL043C	987
YPR178W	YDL098C	999
YPR178W	YDL160C	972
YPR178W	YDR283C	977
YPR178W	YDR378C	996
YPR178W	YDR473C	999
YPR178W	YER029C	996

YPR178W	YER112W	993
YPR178W	YER146W	999
YPR178W	YER172C	999
YPR178W	YGL173C	976
YPR178W	YGR013W	976
YPR178W	YGR074W	978
YPR178W	YGR075C	972
YPR178W	YGR091W	999
YPR178W	YGR241C	998
YPR178W	YHR165C	999
YPR178W	YJL124C	976
YPR178W	YJL203W	993
YPR178W	YJR022W	999
YPR178W	YKL173W	999
YPR178W	YLL036C	934
YPR178W	YLR147C	996
YPR178W	YLR275W	990
YPR178W	YLR438C-A	997
YPR178W	YML046W	976
YPR178W	YML049C	978
YPR178W	YMR240C	976
YPR178W	YMR268C	970
YPR178W	YMR288W	978
YPR178W	YNL147W	999
YPR178W	YOR159C	999
YPR178W	YOR308C	999
YPR178W	YPL213W	983
YPR178W	YPR082C	978
YPR178W	YPR182W	998
YPR179C	YDR295C	999
YPR179C	YNL021W	999
YPR179C	YOL090W	987
YPR180W	YDR390C	999
YPR180W	YDR510W	998
YPR180W	YJL001W	999
YPR181C	YCR067C	983
YPR181C	YDL195W	999
YPR181C	YDL212W	977
YPR181C	YDR170C	978
YPR181C	YDR299W	903
YPR181C	YDR517W	999
YPR181C	YER151C	902
YPR181C	YGL200C	980
YPR181C	YGR172C	969

YPR181C	YHR079C	993
YPR181C	YHR098C	999
YPR181C	YIL004C	999
YPR181C	YIL109C	999
YPR181C	YJL004C	976
YPR181C	YLR026C	995
YPR181C	YLR208W	999
YPR181C	YLR268W	999
YPR181C	YNL049C	999
YPR181C	YPL085W	999
YPR181C	YPL204W	900
YPR181C	YPL218W	999
YPR182W	YBL026W	997
YPR182W	YBR055C	999
YPR182W	YBR065C	915
YPR182W	YBR119W	995
YPR182W	YBR154C	961
YPR182W	YDL030W	975
YPR182W	YDL087C	999
YPR182W	YDR235W	999
YPR182W	YDR240C	986
YPR182W	YDR378C	900
YPR182W	YEL026W	939
YPR182W	YER029C	999
YPR182W	YER146W	974
YPR182W	YER172C	999
YPR182W	YFL017W-A	998
YPR182W	YGL070C	957
YPR182W	YGL173C	906
YPR182W	YGR013W	999
YPR182W	YGR074W	999
YPR182W	YGR091W	928
YPR182W	YHR165C	999
YPR182W	YIL061C	993
YPR182W	YIR009W	949
YPR182W	YJL124C	989
YPR182W	YJR084W	921
YPR182W	YKL012W	999
YPR182W	YKL173W	999
YPR182W	YLL036C	959
YPR182W	YLR147C	999
YPR182W	YLR275W	999
YPR182W	YLR438C-A	988
YPR182W	YML046W	999

YPR182W	YML049C	990
YPR182W	YMR125W	993
YPR182W	YMR240C	998
YPR182W	YMR288W	997
YPR182W	YNL147W	972
YPR182W	YOL005C	931
YPR182W	YOR151C	904
YPR182W	YOR159C	999
YPR182W	YOR210W	944
YPR182W	YOR224C	953
YPR182W	YPL178W	911
YPR182W	YPL213W	962
YPR182W	YPR082C	991
YPR182W	YPR178W	998
YPR182W	YPR187W	929
YPR183W	YBL082C	909
YPR183W	YBR002C	936
YPR183W	YBR110W	979
YPR183W	YBR243C	968
YPR183W	YDL055C	997
YPR183W	YDL095W	918
YPR183W	YGR036C	952
YPR183W	YKL212W	998
YPR183W	YMR013C	991
YPR183W	YPL227C	939
YPR184W	YBR072W	980
YPR184W	YEL011W	926
YPR184W	YIL099W	985
YPR184W	YLR258W	962
YPR184W	YPR160W	991
YPR185W	YDL149W	935
YPR185W	YDR477W	903
YPR185W	YEL013W	999
YPR185W	YGL180W	999
YPR185W	YLR423C	999
YPR185W	YLR431C	939
YPR185W	YPR049C	998
YPR186C	YPR187W	928
YPR187W	YAL038W	914
YPR187W	YBL026W	919
YPR187W	YBR154C	999
YPR187W	YBR198C	900
YPR187W	YBR279W	929
YPR187W	YDL115C	996

YPR187W	YDL140C	999
YPR187W	YDL150W	999
YPR187W	YDR045C	902
YPR187W	YDR156W	998
YPR187W	YDR167W	918
YPR187W	YDR404C	999
YPR187W	YEL026W	936
YPR187W	YER022W	981
YPR187W	YER029C	928
YPR187W	YER094C	922
YPR187W	YER148W	934
YPR187W	YER171W	925
YPR187W	YFL017W-A	905
YPR187W	YGL043W	998
YPR187W	YGL070C	999
YPR187W	YGL112C	901
YPR187W	YGL127C	906
YPR187W	YGR005C	999
YPR187W	YGR063C	987
YPR187W	YGR074W	938
YPR187W	YGR094W	911
YPR187W	YGR104C	983
YPR187W	YGR186W	999
YPR187W	YHR058C	901
YPR187W	YHR143W-A	999
YPR187W	YIL021W	999
YPR187W	YIL143C	923
YPR187W	YJL011C	997
YPR187W	YJL140W	999
YPR187W	YJL148W	999
YPR187W	YJR035W	901
YPR187W	YJR063W	999
YPR187W	YKL058W	904
YPR187W	YKL144C	999
YPR187W	YKR025W	997
YPR187W	YLR071C	980
YPR187W	YLR147C	928
YPR187W	YLR275W	911
YPR187W	YML010W	999
YPR187W	YMR240C	900
YPR187W	YMR277W	950
YPR187W	YNL113W	999
YPR187W	YNL151C	998
YPR187W	YNL248C	999

YPR187W	YNR003C	999
YPR187W	YOL005C	999
YPR187W	YOL051W	959
YPR187W	YOL135C	907
YPR187W	YOR116C	999
YPR187W	YOR151C	999
YPR187W	YOR159C	915
YPR187W	YOR207C	999
YPR187W	YOR210W	999
YPR187W	YOR224C	999
YPR187W	YOR340C	999
YPR187W	YOR341W	999
YPR187W	YPL129W	999
YPR187W	YPL178W	914
YPR187W	YPR010C	998
YPR187W	YPR086W	944
YPR187W	YPR110C	999
YPR187W	YPR168W	939
YPR187W	YPR182W	929
YPR187W	YPR186C	928
YPR187W	YPR190C	999
YPR188C	YGL106W	926
YPR188C	YHR023W	999
YPR188C	YPL242C	913
YPR189W	YDL111C	981
YPR189W	YDR064W	981
YPR189W	YDR280W	997
YPR189W	YGL173C	992
YPR189W	YGL213C	999
YPR189W	YGR158C	978
YPR189W	YGR285C	997
YPR189W	YHR010W	975
YPR189W	YHR141C	909
YPR189W	YJR123W	980
YPR189W	YKL023W	987
YPR189W	YLR398C	999
YPR189W	YMR116C	976
YPR189W	YNL085W	908
YPR189W	YOR063W	996
YPR189W	YOR076C	958
YPR190C	YBL039C	918
YPR190C	YBR154C	999
YPR190C	YDL150W	999
YPR190C	YDR005C	999

YPR190C	YDR045C	998
YPR190C	YDR156W	921
YPR190C	YHR143W-A	960
YPR190C	YJL011C	999
YPR190C	YJL148W	931
YPR190C	YJR063W	934
YPR190C	YKL144C	999
YPR190C	YKR025W	999
YPR190C	YNL039W	960
YPR190C	YNL113W	998
YPR190C	YNL151C	999
YPR190C	YNL248C	929
YPR190C	YNR003C	999
YPR190C	YOR116C	999
YPR190C	YOR207C	999
YPR190C	YOR210W	998
YPR190C	YOR224C	999
YPR190C	YOR340C	921
YPR190C	YOR341W	921
YPR190C	YPR010C	921
YPR190C	YPR110C	999
YPR190C	YPR187W	999
YPR191W	Q0105	999
YPR191W	YBL045C	999
YPR191W	YDL189W	929
YPR191W	YDR529C	999
YPR191W	YEL024W	999
YPR191W	YFR033C	999
YPR191W	YGR183C	999
YPR191W	YHR001W-A	991
YPR191W	YJL166W	999
YPR191W	YJR048W	991
YPR191W	YJR121W	916
YPR191W	YLR132C	978
YPR191W	YOR065W	999
YPR194C	YPL177C	902
YPR198W	YOL051W	988
YPR199C	YML007W	994
YPR199C	YPR200C	936
YPR199C	YPR201W	941
YPR200C	YPR199C	936
YPR200C	YPR201W	958
YPR201W	YDR135C	998
YPR201W	YPR199C	941

YPR201W	YPR200C	958
